# Overexpression of ELF3 in the PTEN-deficient lung epithelium promotes lung cancer development by inhibiting ferroptosis

**DOI:** 10.1038/s41419-024-07274-5

**Published:** 2024-12-18

**Authors:** Zengzhuang Yuan, Xinyan Han, Manyu Xiao, Taoyu Zhu, Yaping Xu, Qian Tang, Chen Lian, Zijin Wang, Junming Li, Boyu Wang, Changhui Li, Xiaochen Xiang, Ruobai Jin, Yufei Liu, Xinyu Yu, Kehang Zhang, Songsong Li, Madhumita Ray, Rong Li, Artiom Gruzdev, Shiqun Shao, Fangwei Shao, Hua Wang, Wang Lian, Yong Tang, Di Chen, Ying Lei, Xuru Jin, Qinglin Li, Weiwen Long, Huaqiong Huang, Francesco J. DeMayo, Jian Liu

**Affiliations:** 1 Department of Respiratory and Critical Care Medicine, the Second Affiliated Hospital, and Centre for Infection Immunity and Cancer (IIC) of Zhejiang University-University of Edinburgh Institute (ZJU-UoE Institute), Zhejiang University School of Medicine, Zhejiang University, Hangzhou, China; 2 Edinburgh Medical School: Biomedical Sciences, College of Medicine and Veterinary Medicine, The University of Edinburgh, Edinburgh, UK; 3 Reproductive & Developmental Biology Laboratory, National Institute of Environmental Health Sciences (NIEHS), Research Triangle Park, NC USA; 4 Department of Obstetrics, Gynecology and Women’ Health, University of Missouri, Columbia, MO USA; 5 Gene Editing and Mouse Model Core, National Institute of Environmental Health Sciences (NIEHS), Research Triangle Park, NC USA; 6 Zhejiang Key Laboratory of Smart Biomaterials and Center for Bionanoengineering, College of Chemical and Biological Engineering, Zhejiang University, Hangzhou, China; 7 Zhejiang University-University of Illinois Urbana-Champaign Institute, Zhejiang University, Haining, China; 8 Biomedical and Heath Translational Research Center of Zhejiang Province, Haining, Zhejiang China; 9 National Key Laboratory of Biobased Transportation Fuel Technology, ZJU-UIUC Institute, Zhejiang University, Hangzhou, China; 10 Department of Materials Science and Engineering, University of Illinois at Urbana-Champaign, Urbana, IL USA; 11 Department of Thoracic Surgery, the Second Affiliated Hospital, Zhejiang University School of Medicine, Zhejiang University, Hangzhou, China; 12 Department of Thoracic Surgery, Shenzhen Nanshan People’s Hospital, Shenzhen, China; 13 Center for Regeneration and Cell Therapy of Zhejiang University-University of Edinburgh Institute (ZJU-UoE Institute), Zhejiang University School of Medicine, Zhejiang University, Hangzhou, Zhejiang China; 14 Department of Respiratory and Critical Care Medicine, The Quzhou Affiliated Hospital of Wenzhou Medical Hospital, Quzhou People’s Hospital, Wenzhou, China; 15 Department of Respiratory and Critical Care Medicine, The First Affiliated Hospital of Wenzhou Medical University, Wenzhou, China; 16 Key Laboratory of Head & Neck Cancer Translational Research of Zhejiang Province, Zhejiang Cancer Hospital, Hangzhou, Zhejiang China; 17 Department of Biochemistry and Molecular Biology, Boonshoft School of Medicine, Wright State University, Dayton, OH USA; 18 Key Laboratory of Respiratory Disease of Zhejiang Province, Department of Respiratory and Critical Care Medicine, Second Affiliated Hospital of Zhejiang University School of Medicine, Hangzhou, Zhejiang China; 19 Zhejiang Key Laboratory of Medical Imaging Artificial Intelligence, Haining, Zhejiang China; 20 Dr. Li Dak Sum & Yip Yio Chin Center for Stem Cell and Regenerative Medicine, Zhejiang University, Hangzhou, China

**Keywords:** Non-small-cell lung cancer, Cancer genomics

## Abstract

Ferroptosis has been shown to play a crucial role in preventing cancer development, but the underlying mechanisms of dysregulated genes and genetic alternations driving cancer development by regulating ferroptosis remain unclear. Here, we showed that the synergistic role of ELF3 overexpression and PTEN deficiency in driving lung cancer development was highly dependent on the regulation of ferroptosis. Human *ELF3* (h*ELF3*) overexpression in murine lung epithelial cells only caused hyperplasia with increased proliferation and ferroptosis. h*ELF3* overexpression and *Pten* genetic disruption significantly induced lung tumor development with increased proliferation and inhibited ferroptosis. Mechanistically, we found it was due to the induction of SLC7A11, a typical ferroptosis inhibitor, and ELF3 directly and positively regulated SLC7A11 in the PTEN-deficient background. Erastin-mediated inhibition of SLC7A11 induced ferroptosis in cells with ELF3 overexpression and PTEN deficiency and thus inhibited cell colony formation and tumor development. Clinically, human lung tumors showed a negative correlation between *ELF3* and *PTEN* expression and a positive correlation between *ELF3* and *SLC7A11* in a subset of human lung tumors with *PTEN*-low expression. *ELF3* and *SLC7A11* expression levels were negatively associated with lung cancer patients’ survival rates. In summary, ferroptosis induction can effectively attenuate lung tumor development induced by *ELF3* overexpression and *PTEN* downregulation or loss-of-function mutations.

## Introduction

Lung cancer is the leading cause of cancer-associated death worldwide [1]. Benefiting from identifying its driver genes or mutations, such as *EGFR*, *ALK*, and *KRAS*, some targeted therapies were developed and effectively inhibit the development of lung tumors harboring these mutations [2]. The lung tumor driver roles of these genetic mutations (*e.g*., *EGFR*^L858R^, *KRAS*^G12D^) have been explored in these genetic mouse models [3]. However, only some lung cancer patients have these validated drivers. Therefore, identifying more dysregulated genes and genetic alternations in driving lung cancer development is urgently needed.

ELF3 is a transcription factor [4]. Its functions in cancer development are context-dependent [4]. For example, it plays a tumor suppressor in breast cancer and biliary tract carcinoma, and acts as an oncogene in gastrointestinal neuroendocrine carcinoma, pancreatic carcinoma, cervical carcinoma, hepatic carcinoma, bladder carcinoma, thyroid carcinoma, and nasopharyngeal carcinoma. Moreover, it had dual roles in some cancer types, including colorectal carcinoma, prostate carcinoma, ovarian carcinoma, and lung cancer [4]. Previously, we revealed that ELF3 was gradually induced during the development of lung tumors induced by the deletion of *Pten* and *Smad4* in mouse lung epithelium [5]. We showed that its knockdown reduced the proliferation of human bronchial epithelial cells [5]. And then, ELF3 overexpression in lung cancer cells was found to promote cell growth and metastasis through PI3K/AKT and ERK signaling pathways [6]. Furthermore, both cellular and xenograft studies showed *ELF3* as an oncogene in lung cancer [7]. In contrast, ELF3 was also reported to inhibit the progression of complete epithelial-mesenchymal transition (EMT) [8]. So far, the in vivo role of ELF3 in spontaneous lung tumor development has not been investigated.

Ferroptosis was found to be a new type of programmed cell death [9]. It is characterized by intracellular iron accumulation, the accumulation of lipid peroxidation products malondialdehyde (MDA) on cell membranes, and glutathione depletion [10]. Moreover, the reduction of glutathione (GSH) and glutathione peroxidase 4 (GPX4) triggered ferroptosis [10]. Ferroptosis can be inhibited by SLC7A11, an antiporter of cystine/glutamate, which imports cystine for glutathione biosynthesis and antioxidant defense [11]. Moreover, ferroptosis is a crucial inhibitor of cancer development with links to tumor suppressors (*e.g.*, *TP53*, *BAP1*, *AMER1*) and the oncogenic mutations (*e.g*., RAS family, *RBMS1*, *OTUB1*) [12]. Moreover, cancer cell death caused by ferroptosis can trigger and affect the immune response of tumor microenvironments (TME) [13]. Meanwhile, the different types of immune cells show different responses to ferroptosis, with either inhibition or promotion effects [14]. Clinically, targeting ferroptosis has been applied in treating cancer [14]. For example, some tumor suppressors (*e.g*., *RB1*) were lost or mutated, making cancer cells more sensitive to ferroptosis [15, 16]. Moreover, ferroptosis induction by GPX4 inhibitor (*e.g*., N6F11) sensitizes cancer cells to immune therapy (*e.g.*, PD-1 antibody), chemotherapy or radiation therapy [17].

In this study, using genetically modified mice, we specifically overexpressed h*ELF3* in mouse lung epithelium by generating genetic mouse models and investigated the in vivo role of ELF3 in lung cancer development. h*ELF3* overexpression promoted cell proliferation and ferroptosis, leading to hyperplasia in the lung epithelium. By integrating the clinical analyses and genetic mouse models, we found that *ELF3* overexpression in the PTEN-deficient background promoted cell proliferation and inhibited ferroptosis by inducing SLC7A11 expression, thus promoting lung cancer development. Targeting SLC7A11 to induce ferroptosis may effectively treat human lung tumors with *ELF3* overexpression and *PTEN* downregulation or loss-of-function mutations.

## Results

### Overexpression of *ELF3* in human and murine lung epithelium promotes cell proliferation and hyperplasia

**Fig. 1:**
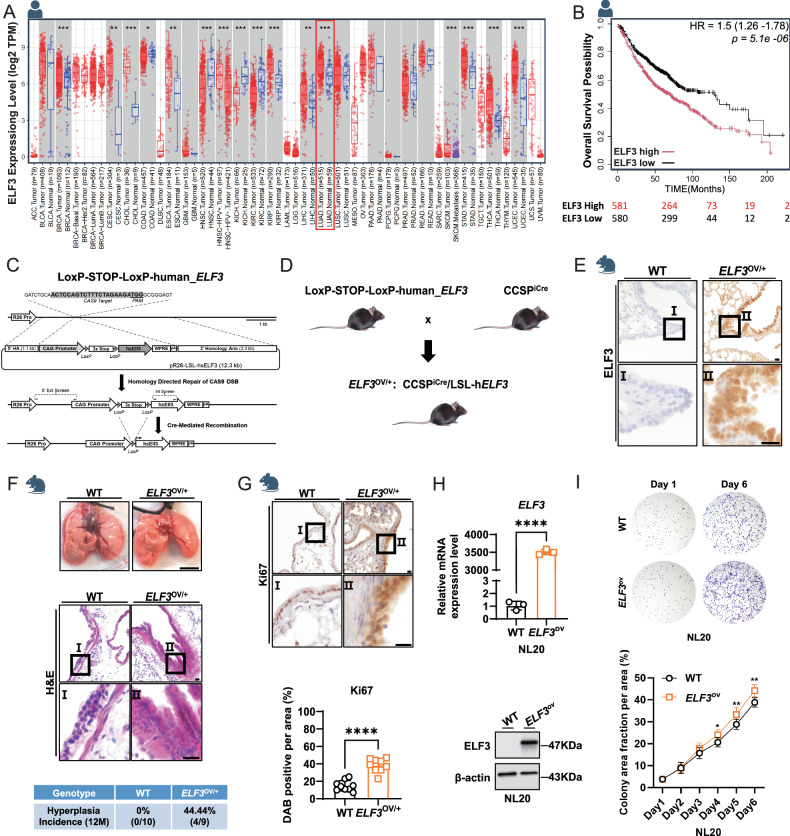
Overexpression of *ELF3* in human and murine lung epithelium promotes cell proliferation and hyperplasia. **A** *ELF3* expression levels across various cancer types and corresponding adjust normal tissues in TCGA (PMID: 32442275). **B** The correlation between *ELF3* expression levels and overall survival in lung cancer patients (PMID: 37783508). The numbers below the graph are the numbers of patients. **C**, **D** Strategy for constructing a mouse model (*ELF3*^OV/+^) with overexpression of human *ELF3* in mouse lung epithelial cells. **E** Comparative analysis of hELF3 expression levels in lung tissues of WT and *ELF3*^OV/+^ mice through IHC staining. Scale bar, 10 μm. **F** Statistical analysis of lung tissue phenotypes in WT (*n* = 10) and *ELF3*^OV/+^ (*n* = 9) mice based on appearance and H&E staining. The scale bar of lung tissues represents 0.5 cm, of H&E represents 10 μm. **G** Comparative analysis of Ki67 expression levels in lung tissues of WT (*n* = 8) and *ELF3*^OV/+^ (*n* = 9) through IHC staining, followed by quantitative analysis of Ki67-positive staining using ImageJ. Statistical comparisons were performed using the Unpaired t-test, * *p* < 0.05, **p < 0.01, ***p < 0.001. Error bars represent the SEM. Scale bar, 10 μm. **H** RT-qPCR and WB detected expression levels of ELF3 in NL20 WT and ELF3ov cells. The 2^−ΔΔCt^ method was used to determine the relative expression levels of *ELF3* mRNA. *n* = 3 (three independent experiments). Statistical comparisons were performed using the Unpaired t-test, *p < 0.05, **p < 0.01, ***p < 0.001. Error bars represent the SEM. **I** Detection of differences in cell proliferation capacity after overexpression of ELF3 in NL20 cells using cell colony formation assay. *n* = 3 (three independent experiments). Statistical comparisons were performed using the 2-way ANOVA, *p < 0.05, **p < 0.01, ***p < 0.001. Error bars represent the SEM.

As we reported before, ELF3 expression is significantly increased in lung cancer patients compared to the normal control group, as well as in other types of cancer (*e.g*., breast cancer, cervical squamous cell carcinoma, and endocervical adenocarcinoma, stomach adenocarcinoma) (Fig. 1A). In addition, its mRNA expression was negatively correlated with the survival of lung cancer patients (Fig. 1B). To investigate its role in lung cancer development, we knocked in LoxP-STOP-LoxP-h*ELF3* into mouse Rosa26 gene locus using CRISPR/Cas9 (Fig. 1C). By crossing with CCSP^iCre^ mouse that expresses strong Cre activity in lung bronchial epithelium, we generated the mice (*ELF3*^OV/+^: CCSP^iCre^ ELF3^OV/+^) that highly overexpress h*ELF3* in lung epithelium (Fig. 1D, E). Unexpectedly, we only observed lung hyperplasia in 44.44% of 12-month-old *ELF3*^OV/+^ mice and no tumor development was observed (Fig. 1F). Consistently, the expression of Ki67, a molecular marker for cell proliferation, was observed to be increased after the overexpression of *ELF3* in mouse lung epithelium (Fig. 1G). Meanwhile, we observed a similar effect in human bronchial epithelial cells NL20 in which we overexpressed ELF3 (Fig. 1H, I). Taken together, overexpression of *ELF3* in human and murine lung epithelium induces proliferation and hyperplasia.

### Overexpression of *ELF3* in human or murine lung epithelium induces ferroptosis

**Fig. 2:**
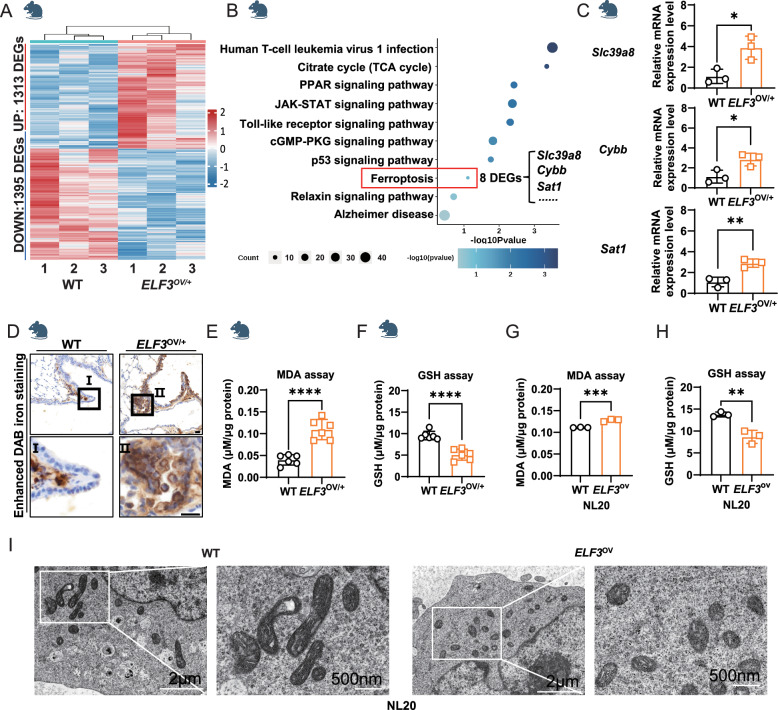
Overexpression of ELF3 in human and murine lung epithelium induces ferroptosis. **A** Transcriptomic analysis of lung tissues from WT (*n* = 3) and *ELF3*^OV/+^ (*n* = 3). DEGs represent the differentially expressed genes from *ELF3*^OV/+^ vs WT. **B** Conducting pathway enrichment analysis of DEGs from *ELF3*^OV/+^ vs WT by KEGG. **C** Gene expression levels of *Slc39a8*, *Cybb*, and *Sat1* in mouse lung tissue samples were detected by RT-qPCR. The 2^−ΔΔCt^ method was used to determine the relative expression levels of *Slc39a8*, *Cybb* and *Sat1* mRNA. *n* = 3 (three independent experiments). Statistical comparisons were performed using the Unpaired t-test, *p < 0.05, **p < 0.01, ***p < 0.001. Error bars represent the SEM. **D** Comparing the expression levels of iron ions in lung tissues between WT and *ELF3*^OV/+^ mice using Prussian blue staining. Scale bar, 10 μm. Detecting the content of MDA (**E**) and GSH (**F**) in lung tissues of WT (*n* = 6) and *ELF3*^OV/+^ (*n* = 6) mice, μM/μg protein represents the level of MDA/GSH. Statistical comparisons were performed using the Unpaired t-test, *p < 0.05, **p < 0.01, ***p < 0.001. Error bars represent the SEM. Detecting the content of MDA (**G**) and GSH (**H**) in NL20 cells of WT and *ELF3*^ov/+^, μM/μg protein represents the level of MDA/GSH. *n* = 3 (three independent experiments). Statistical comparisons were performed using the Unpaired t-test, *p < 0.05, **p < 0.01, ***p < 0.001. Error bars represent the SEM. (**I**) The TEM captures mitochondria in NL20 WT and *ELF3*^ov/+^ cell lines. The scale bar above is 2 μm, and the below one is 500 nm.

To investigate the reason that ELF3 overexpression failed to induce the formation of lung tumors even though it has been reported to be a potential oncogene in human lung cancer cells and xenograft models [6, 18], we conducted the transcriptome analysis of mouse lungs with ELF3 overexpression using RNA-Seq. We identified 2,708 differentially expressed genes (DEGs) between 12-month-old *ELF3*^OV/+^ and control mouse lungs (Fig. 2A and Supplementary Table 1). Some typical cancer-related pathways were identified in the KEGG analysis of these DEGs, including the p53 signaling pathway, TNF signaling pathway, cell cycle, and ferroptosis (Fig. 2B). We are interested in the enriched ferroptosis pathway since it has been reported to be a vital regulator and target for different cancer types [10]. Therefore, we validated the upregulation of 3 ferroptosis-promoting genes among 8 ferroptosis-related DEGs, which are potentially responsible for the initiation of ferroptosis in ELF3-overexpressing cells (Fig. 2C). Then we detected the expression of several ferroptosis markers, such as Perls-Diaminobenzidine (DAB), an indicator of iron deposition [19], Propanondialdehyde (MDA), a typical product of ferroptosis-related lipid peroxidation [20], and GSH as an important ferroptosis suppressor and non-enzymatic antioxidant [21]. Notably, we observed the induction of ferroptosis after overexpression of *ELF3*, evidenced by the increased DAB staining (Fig. 2D), the MDA expression level (Fig. 2E), and the decreased GSH level (Fig. 2F). Consistently, we observed the similar effect on the expression changes of MDA and GSH in human bronchial epithelial cells after the *ELF3* overexpression (Fig. 2G, H). In addition, the mitochondria of cells in the *ELF3*^*OV/+*^ group were significantly smaller and spherical than those of other groups, with reduced membrane density and fewer mitochondrial cristae (Fig. 2I). These typical morphological changes of ferroptosis indicated that cells with high ELF3 expression were undergoing ferroptosis (Fig. 2I). In sum, *ELF3* overexpression in human and murine lung epithelium causes ferroptosis.

### Overexpression of *ELF3* under the PTEN-deficient human and murine lung epithelium promotes lung cancer development

**Fig. 3:**
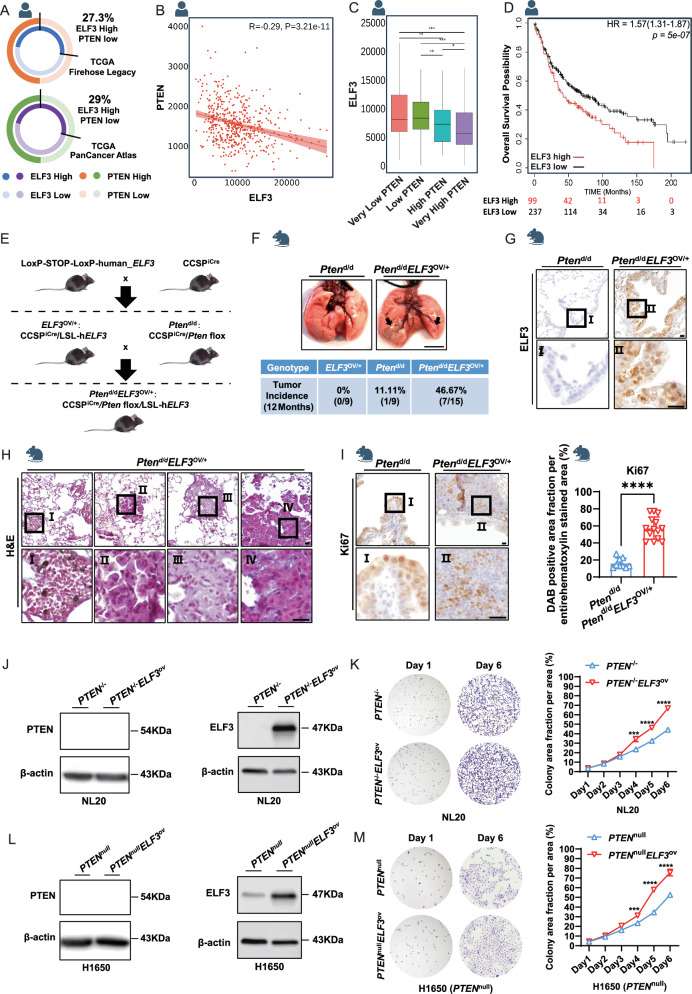
Overexpression of *ELF3* under the PTEN-deficient Human and Murine Lung Epithelium Promotes Lung Cancer Development. **A** The percentage of lung cancer patients with ELF3 overexpression and PTEN low or deficient in two databases (*n* = 586 and 566, respectively). **B** The correlation between *ELF3* and *PTEN* at the mRNA level based on lung cancer patients’ data (PMID: 29625048). **C** The *ELF3* expression in different groups of lung cancer patients with diverse *PTEN* expression levels (PMID: 29625048). **D** The correlation between *ELF3* levels and overall survival in lung cancer patients with low *PTEN* expression (PMID: 29625048). **E** Strategy for constructing a mouse model (*Pten*^d/d^*ELF3*^OV/+^) with deletion of *Pten* and overexpression of h*ELF3* in lung epithelial cells. **F** Lung cancer incidence in 12-month-old *Pten*^d/d^ (*n* = 9) and *Pten*^d/d^*ELF3*^OV/+^ (*n* = 15) mice based on appearance and H&E staining. Scale bar, 0.5 cm. **G** Comparative analysis of hELF3 expression levels in lung tissues of *Pten*^d/d^ and *Pten*^d/d^*ELF3*^OV/+^ mice through IHC staining. Scale bar, 10 μm. **H** Analysis of the morphological characteristics of lung tumors in *Pten*^d/d^*ELF3*^OV/+^ mice through H&E staining. Scale bar, 10 μm. **I** Comparative analysis of Ki67 expression levels in lung tissues of *Pten*^d/d^ (*n* = 9) and *Pten*^d/d^*ELF3*^OV/+^ (*n* = 14) mice through IHC staining, followed by quantitative analysis of Ki67-positive staining using ImageJ. Statistical comparisons were performed using the Unpaired t-test, *p < 0.05, **p < 0.01, ***p < 0.001. Error bars represent the SEM. Scale bar, 10 μm. **J** Expression levels of PTEN and ELF3 in NL20 *PTEN*^−/−^ and *PTEN*^−/−^*ELF3*^ov^ cells were detected by WB. *n* = 3 (three independent experiments). **K** Detection of differences in cell proliferation capacity of NL20 *PTEN*^−/−^ and *PTEN*^−/−^*ELF3*^ov^ cells using cell colony formation assay. *n* = 3 (three independent experiments). Statistical comparisons were performed using the 2-way ANOVA, *p < 0.05, **p < 0.01, ***p < 0.001. Error bars represent the SEM. **L** Expression levels of PTEN and ELF3 in H1650 *PTEN*^null^ and *PTEN*^null^*ELF3*^ov^ cells were detected by WB. *n* = 3 (three independent experiments). **M** Detection of differences in cell proliferation capacity of H1650 *PTEN*^null^ and *PTEN*^null^*ELF3*^ov^ cells using cell colony formation assay. *n* = 3 (three independent experiments). Statistical comparisons were performed using the 2-way ANOVA, *p < 0.05, **p < 0.01, ***p < 0.001. Error bars represent the SEM.

Although single overexpression of h*ELF3* in murine lung epithelium did not induce lung tumor development (Fig. 1), the elevated expression of *ELF3* in human lung tumors (Fig. 1A) suggests its potential role in lung cancer development. Given that we have found that *Elf3* was induced during murine lung tumor development by deletion of *Pten* and *Smad4* in lung epithelium and that *PTEN* was one of the top mutated genes in human lung tumors [5], we hypothesized that ELF3 overexpression in combination with PTEN genetic disruption might promote lung tumor development. Therefore, we counted the proportion of lung cancer patients with low expression of PTEN and high expression of ELF3 as a percentage of all lung adenocarcinoma patients recorded in two databases, which were 27.3 and 29%, respectively (Fig. 3A). Moreover, we examined the expression levels of *ELF3* and *PTEN* in human lung tumors and found they were negatively correlated (Fig. 3B). Moreover, we found that *ELF3* expression was increased in human lung tumors with lower *PTEN* expression (Fig. 3C). Additionally, *ELF3* mRNA expression was still negatively correlated with the survival of lung cancer patients with low expression of *PTEN* (Fig. 3D). These results suggested that there might be a synergistic role of ELF3 overexpression and PTEN deficiency in promoting lung tumor development.

To investigate the role of ELF3 overexpression in PTEN deficient setting in lung tumor development, we generated mice (*ELF3*^OV/+^*Pten*^d/d^: CCSP^iCre^ *ELF3*^OV/+^ *Pten*^f/f^) with both *ELF3* overexpression and *Pten* deletion by crossing *ELF3*^OV/+^ with *Pten* floxed mice (Fig. 3E). Of important note, we observed that ELF3 overexpression increased the rate of lung tumor development from 11.11% to 46.67% in 12-month-old mice under PTEN deficient background (Fig. 3F–H). Moreover, we also observed the increased Ki67 (Fig. 3I), similar to that of ELF3 overexpression under wild-type PTEN background (Fig. 1G). Moreover, overexpression of *ELF3* in human bronchial epithelial cells under the *PTEN*-knockout background significantly promotes the formation of colonies (Fig. 3J, K). The effect caused by *ELF3* overexpression in a *PTEN*-deficient background was more substantial than that of a wild-type PTEN-positive background (Fig. 1H, I). These differential effects were also seen in human lung cancer cells with *PTEN*-null background and *ELF3* overexpression (Fig. 3L, M). Taken together, *ELF3* overexpression and *PTEN* deficiency in human and murine lung epithelium synergistically facilitate lung cancer development.

### Overexpression of *ELF3* in PTEN-deficient human and murine lung epithelium inhibits ferroptosis

**Fig. 4:**
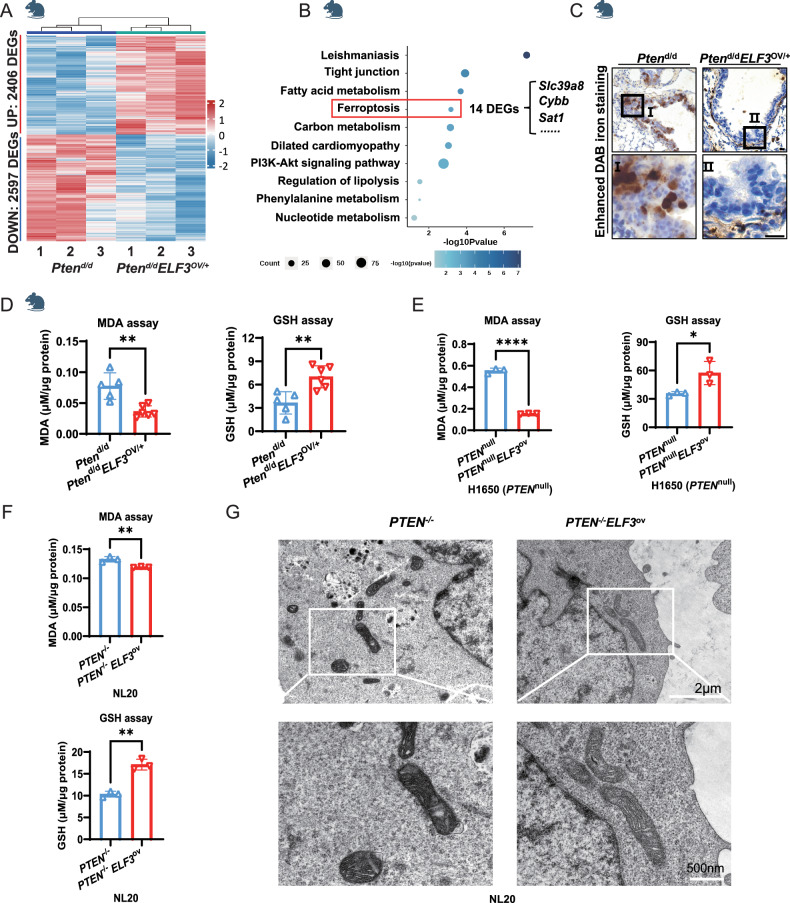
Overexpression of *ELF3* in PTEN-deficient human and murine lung epithelium inhibits ferroptosis. **A** Transcriptomic analysis of lung tissues from *Pten*^d/d^ (*n* = 3) and *Pten*^d/d^*ELF3*^OV/+^ (*n* = 3). DEGs represent the differentially expressed genes from *Pten*^d/d^*ELF3*^OV/+^ vs *Pten*^d/d^. **B** Conducting pathway enrichment analysis of DEGs from *Pten*^d/d^*ELF3*^OV/+^ vs *Pten*^d/d^ by KEGG. **C** Comparing the expression levels of iron ions in lung tissues between *Pten*^d/d^ and *Pten*^d/d^*ELF3*^OV/+^ mice using Prussian blue staining. Scale bar, 10 μm. **D** Detecting the content of MDA (left) and GSH (right) in lung tissues of *Pten*^d/d^ (*n* = 5) and *Pten*^d/d^*ELF3*^OV/+^ (*n* = 6) mice, μM/μg protein represents the level of MDA/GSH. Statistical comparisons were performed using the Unpaired t-test, *p < 0.05, **p < 0.01, ***p < 0.001. Error bars represent the SEM. **E** Detecting the content of MDA (left) and GSH (right) in H1650 *PTEN*^null^ and *PTEN*^null^*ELF3*^ov^ cells, μM/μg protein represents the level of MDA/GSH. *n* = 3 (three independent experiments). Statistical comparisons were performed using the Unpaired t-test, *p < 0.05, ** *p* < 0.01, ***p < 0.001. Error bars represent the SEM. **F** Detecting the content of MDA (upper) and GSH (lower) in NL20 *PTEN*^−/−^ and *PTEN*^−/−^*ELF3*^ov^ cells, μM/μg protein represents the level of MDA/GSH. *n* = 3 (three independent experiments). Statistical comparisons were performed using the Unpaired t-test, *p < 0.05, **p < 0.01, ***p < 0.001. Error bars represent the SEM. **G** The TEM captures of mitochondria in NL20 *PTEN*^−/−^ and *PTEN*^−/−^*ELF3*^ov^ cell lines. The scale bar above is 2 μm, and the below one is 500 nm.

To decipher the mechanisms underlying the role of *ELF3* overexpression in promoting lung cancer development under the *PTEN*-deficient human and murine lung epithelium, we performed RNA-Seq analysis to identify the DEGs between 12-month-old *ELF3*^OV/+^*Pten*^d/d^ and *Pten*^d/d^ mouse lungs. There are 5003 DEGs identified (Fig. 4A and Supplementary Table 2). Interestingly, ferroptosis-related genes were enriched again (Fig. 4B). Indeed, in contrast to ferroptosis induction in *ELF3*^OV/+^ in comparison with wild-type control mouse lungs (Fig. 2), ferroptosis was inhibited by overexpression of *ELF3* under *PTEN* deficiency background both in mice (Fig. 4C, D) and in human lung epithelial cells H1650 (Fig. 4E) and human lung cancer cells NL20 (Fig. 4F), as demonstrated by ferroptosis characteristics, such as the decreased DAB staining and MDA expression or the increased GSH expression (Fig. 4). In addition, the mitochondrial morphology of NL20 cells in the *PTEN*^*−/−*^*ELF3*^*OV/+*^ group was as normal as that of *PTEN*^*−/−*^ and wild-type cells, indicating the repression of ferroptosis in *PTEN*^*−/−*^*ELF3*^*OV/+*^ cells (Fig. 4G). Moreover, we detected the expression of these three ferroptosis-promoting genes, *Slc39a8*, *Sat1*, and *Cybb*. We found that these three genes were still upregulated in *ELF3*^*OV/+*^*Pten*^*d/d*^ compared to that of *Pten*^*d/d*^, similar to the increase observed in the comparison between *ELF3*^*OV/+*^ and WT (Fig. S1D). Therefore, the inhibited ferroptosis of *ELF3*^*OV/+*^*Pten*^*d/d*^ compared to that of *Pten*^d/d^ (Fig. 4) was considered to be caused by other ferroptosis-related genes specifically dysregulated in *ELF3*^*OV/+*^*Pten*^*d/d*^ mouse group compared to other three groups (WT, *ELF3*^*OV/+*^, *ELF3*^*OV/+*^*Pten*^*d/d*^).

### Overexpression of *ELF3* in PTEN-deficient human and murine lung epithelium induces the expression of ferroptosis inhibitor SLC7A11

**Fig. 5:**
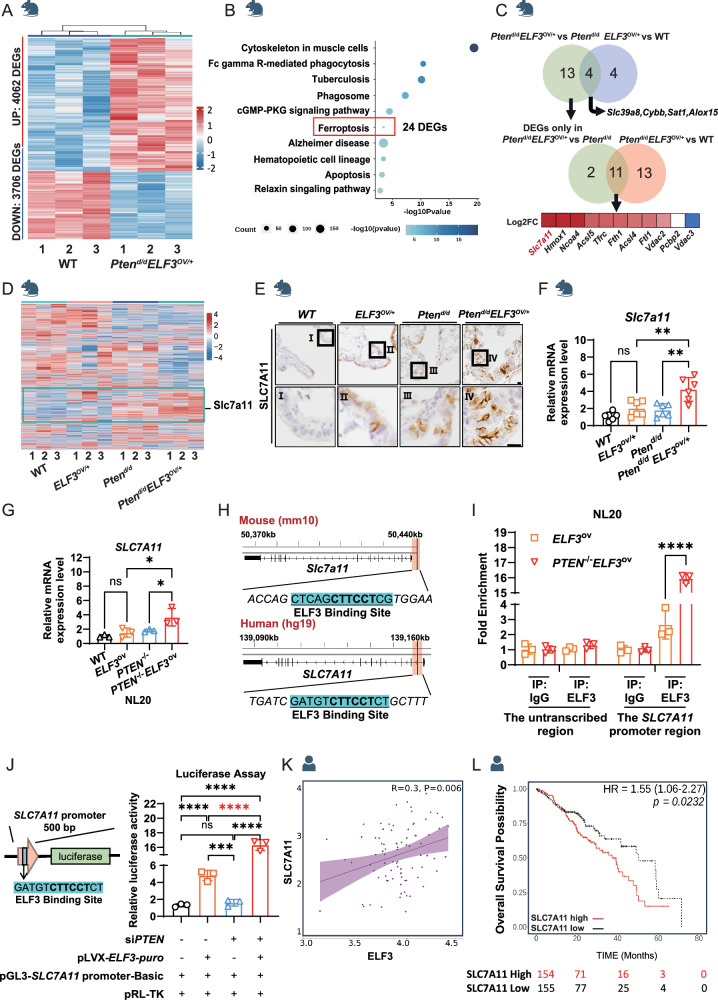
Overexpression of *ELF3* in PTEN-deficient Human and Murine Lung Epithelium Induces the Expression of Ferroptosis Inhibitor SLC7A11. **A** Transcriptomic analysis of lung tissues from WT (*n* = 3) and *Pten*^d/d^*ELF3*^OV/+^ (*n* = 3). DEGs represent the differentially expressed genes from *Pten*^d/d^*ELF3*^OV/+^ vs WT. **B** Conducting pathway enrichment analysis of DEGs from *Pten*^d/d^*ELF3*^OV/+^ vs WT by KEGG. **C** Venn diagrams indicate the strategy to select DEGs related to tumorigenesis and inhibition of ferroptosis from three groups, *Pten*^d/d^*ELF3*^OV/+^ vs WT, *Pten*^d/d^*ELF3*^OV/+^ vs WT and *Pten*^d/d^*ELF3*^OV/+^ vs WT. **D** Joint analysis of 4 groups of transcriptomic data of mouse models, showing the expression profiling of all genes in 4 groups (12 samples), Wild-type, *ELF3*^OV/+^, *Pten*^d/d^ and *Pten*^d/d^*ELF3*^OV/+^. **E**, **F** Comparative analysis of SLC7A11 expression levels in lung tissues of WT, *ELF3*^OV/+^, *Pten*^d/d^ and *Pten*^d/d^*ELF3*^OV/+^ mice through IHC staining and RT-qPCR. The 2^−ΔΔCt^ method was used to determine the relative expression levels of *Slc7a11* mRNA. *n* = 3 (three independent experiments). Statistical comparisons were performed using the one-way ANOVA, *p < 0.05, **p < 0.01, ***p < 0.001. **G** Expression levels of SLC7A11 in NL20 *PTEN*^−/−^ and *PTEN*^−/−^*ELF3*^ov^ cells were detected by RT-qPCR and WB. The 2^−ΔΔCt^ method was used to determine the relative expression levels of *SLC7A11* mRNA. *n* = 3 (three independent experiments). Statistical comparisons were performed using the Unpaired t-test, *p < 0.05, **p < 0.01, ***p < 0.001. Error bars represent the SEM. **H** ELF3 motif is detected at the promoter region of *SLC7A11*. **I**, **J** Left: Verification of the binding frequency of ELF3 at the SLC7A11 promoter region through ChIP-qPCR. Right: Detecting the regulatory relationship between ELF3 and SLC7A11 through dual-luciferase reporter gene assay. **K** The correlation between *ELF3* and *SLC7A11* expression in lung cancer patients with low *PTEN* expression (PMID: 29625048). **L** The correlation between *SLC7A11* expression levels and overall survival in lung cancer patients with low *PTEN* expression and high expression of *ELF3* (PMID: 29625048).

We did a comprehensive analysis to explore the underlying mechanisms by which *ELF3* overexpression inhibits ferroptosis under the *PTEN*-deficient human and murine lung epithelium. First, we identified the 7768 DEGs related to lung cancer development by comparing the transcriptome profiles between *ELF3*^OV/+^*Pten*^d/d^ and wild-type control mouse lungs (Fig. 5A and Supplementary Table 3). Second, KEGG analysis was applied to these DEGs. As expected, genes related to ferroptosis were enriched (Fig. 5B). Third, we overlapped the DEGs of ferroptosis pathways from these comparisons (Fig. 5C). We aimed to identify the DEGs specifically affected by ELF3 overexpression in PTEN-deficient background (*ELF3*^OV/+^*Pten*^d/d^ vs *Pten*^d/d^). And these DEGs were expected to be related to ferroptosis and cancer development (*ELF3*^OV/+^*Pten*^d/d^ vs WT). Here, overlapped DEGs were identified, and *Slc7a11*, a well-known ferroptosis inhibitor in an iron-dependent manner [22], was the top upregulated one among these genes (Fig. 5C). It was also a representative gene from the group of DEGs in the *ELF3*^*OV/+*^*Pten*^*d/d*^ group compared to the other three groups (Fig. 5D). Indeed, we confirmed the induced expression of SLC7A11 in mice (Fig. 5E, F), human bronchial epithelial cells NL20 (Figs. 5G and S1F), and lung cancer cells H1650 Fig. S1E, G) with the background of *ELF3* overexpression and PTEN deficiency. Since ELF3 is a transcription factor and mRNA expression of *Slc7a11*/*SLC7A11* was induced by *ELF3* overexpression, we employed the motif analysis of the SLC7A11 promoter. Importantly, we identified the conserved ELF3 motifs in human and murine SLC7A11 promoters (Fig. 5H).

To examine if ELF3 can directly regulate the SLC7A11 mRNA expression, we first conducted the ELF3 Chip-qPCR assay on the SLC7A11 promoter, and we found that the ELF3 binding ability was significantly increased in *ELF3* overexpression and PTEN deficient background compared to *ELF3* overexpression background (Figs. 5I and S1H). We then cloned its promoter containing the ELF3 motif sequence into the luciferase reporter plasmid (Fig. 5J left). ELF3 overexpression significantly induced the luciferase signals under the regulation of the *SLC7A11* promoter compared to the controls (Fig. 5J right and Fig. S1I). Consistently, we observed the positive correlation between *ELF3* and *SLC7A11* expression only in human lung cancer tumors with the *PTEN* low expression, not in the whole patient population (Figs. 5K and S1J). Moreover, the expression level of *SLC7A11* was negatively correlated to the survival of lung cancer patients with low expression levels of *PTEN* in lung tumors (Fig. 5L). Altogether, *ELF3* overexpression in PTEN-deficient human and murine lung epithelium induces the expression of SLC7A11, thus inhibiting ferroptosis.

### Erastin, Targeting SLC7A11, attenuate the development of lung cancer with the ELF3 overexpression and the PTEN-deficient background by inducing ferroptosis

**Fig. 6:**
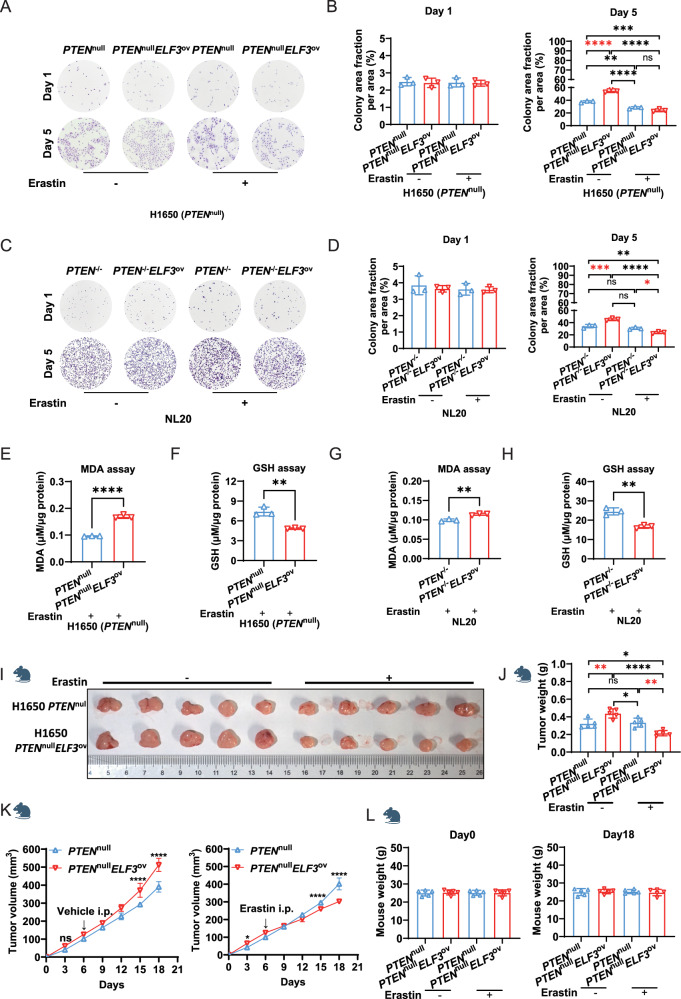
Erastin, targeting SLC7A11, significantly induces ferroptosis of lung cancer cells with ELF3 overexpression and PTEN-deficient background. **A** Cell proliferation capacity of H1650 *PTEN*^null^ and *PTEN*^null^*ELF3*^ov^ cells after erastin treatment measured using cell colony formation assay. *n* = 3 (three independent experiments). **B** Quantitative analysis of cell colony formation of H1650 *PTEN*^null^ and *PTEN*^null^*ELF3*^ov^ cells treated with erastin on the first and fifth days using ImageJ. Statistical comparisons were performed using the 2-way ANOVA, *p < 0.05, **p < 0.01, ***p < 0.001. Error bars represent the SEM. **C** Cell proliferation capacity of NL20 *PTEN*^−/−^ and *PTEN*^−/−^*ELF3*^ov^ cells after erastin treatment using cell colony formation assay. *n* = 3 (three independent experiments). **D** Quantitative analysis of cell colony formation of NL20 *PTEN*^−/−^ and *PTEN*^−/−^*ELF3*^ov^ cells treated with erastin on the first and fifth days using ImageJ. Statistical comparisons were performed using the 2-way ANOVA, *p < 0.05, **p < 0.01, ***p < 0.001. Error bars represent the SEM. MDA (**E**) and GSH (**F**) assays of H1650 *PTEN*^null^ and *PTEN*^null^*ELF3*^ov^ cells after five days of erastin treatment. μM/μg protein represents the level of MDA/GSH. *n* = 3 (three independent experiments). Statistical comparisons were performed using the Unpaired t-test, *p < 0.05, **p < 0.01, ***p < 0.001. Error bars represent the SEM. MDA (**G**) and GSH (**H**) assays in NL20 *PTEN*^−/−^ and *PTEN*^−/−^*ELF3*^ov^ cells after five days of erastin treatment. μM/μg protein represents the level of MDA/GSH. *n* = 3 (three independent experiments). Statistical comparisons were performed using the Unpaired t-test, *p < 0.05, **p < 0.01, ***p < 0.001. Error bars represent the SEM. **I**–**L** The volume of the xenograft tumors was measured and quantified. Each group consists of five nude mice (*n* = 5). Statistical comparisons were performed using the 1-way & 2-way ANOVA, *p < 0.05, **p < 0.01, ***p < 0.001. Error bars represent the SEM.

To investigate if inhibiting SLC7A11 can prevent cell growth induced by *ELF3* overexpression under a PTEN-deficient background, we treated cells with erastin, an inhibitor of SLC7A11 [23]. Consistent with the previous results, cell colonies were significantly increased by *ELF3* overexpression under PTEN-deficient background either in lung cancer cells H1650 (Fig. 6A, B) or in lung epithelial cells NL20 (Fig. 6C, D). Of note, these effects were abolished by the treatment of erastin (Fig. 6A–D). As expected, erastin treatment significantly induced ferroptosis in these cells, evidenced by the increased MDA concentration and the decreased GSH expression (Fig. 6E–H). We then conducted similar experiments in vivo using xenograft models and observed that erastin specifically and significantly attenuated the development of lung cancer with the ELF3 overexpression and the PTEN-deficient background (Fig. 6I–L). Thus, these results suggest that SLC7A11 inhibition by applying the available clinical drug might be an effective therapy for treating lung cancer patients with both ELF3 overexpression and low levels of PTEN expression or PTEN mutation.

## Discussion

**Fig. 7:**
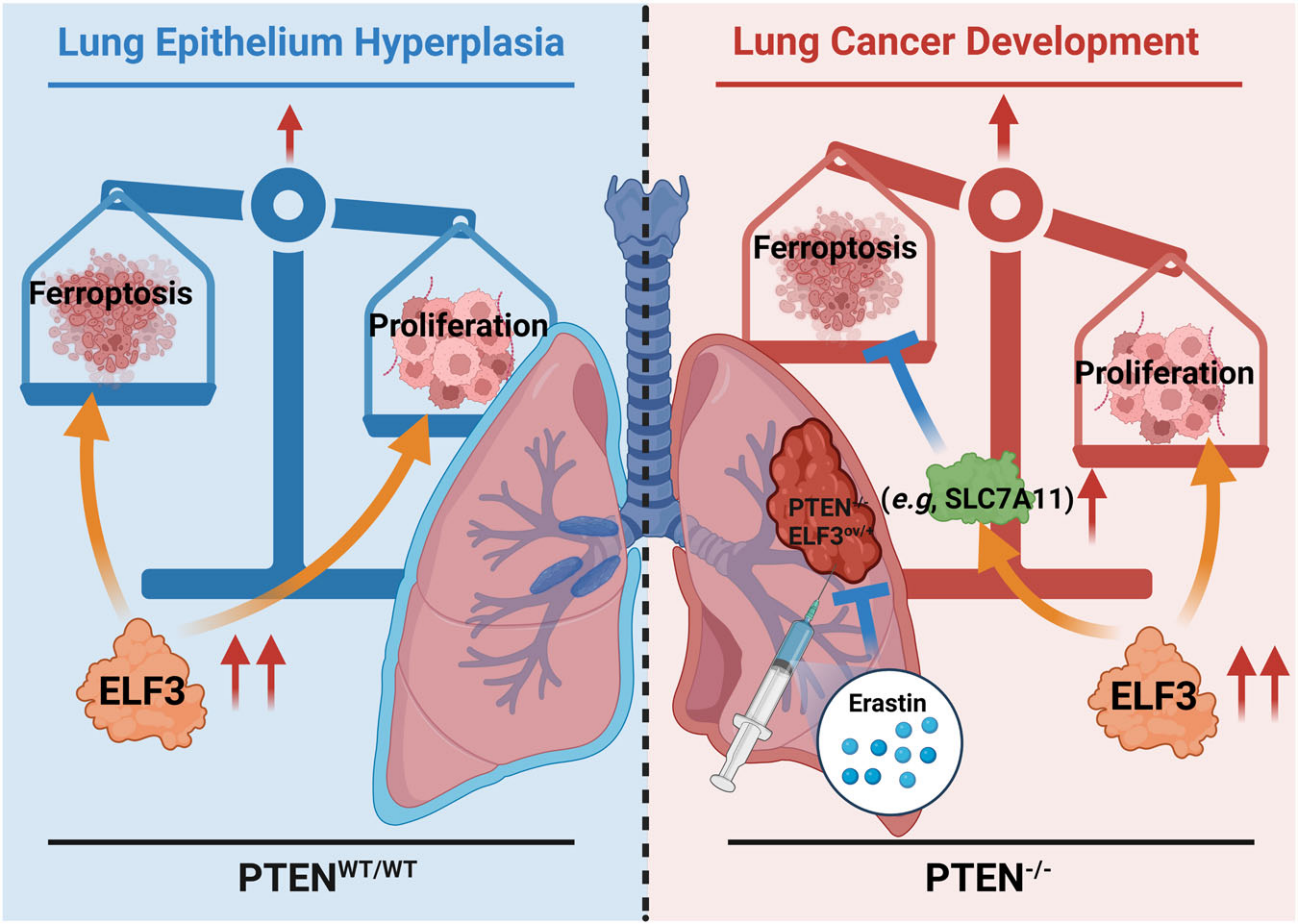
The working model of this study. ELF3 overexpression promoted proliferation and ferroptosis at the same time; thus, the induced ferroptosis likely formed a barrier to constrain these hyperplastic lung epithelia from being transformed into lung tumors (Fig. 7 left); *ELF3* overexpression in the *Pten* deletion mouse lung epithelium significantly promoted lung tumor developments by inhibiting ferroptosis while remaining the proliferation-promoting effect (Fig. 7 right). Mechanistically, this effect is largely dependent on the induced expression of SLC7A11, a typical inhibitor of ferroptosis, in mouse lungs and human lung epithelial cells and cancer cells (Fig. 7 right). Importantly, targeting SLC7A11 using its inhibitor, erastin, showed obvious inhibition of the colony formation of lung cancer cells and cancer development with ELF3 overexpression and PTEN deficiency by inducing ferroptosis.

This study revealed that overexpression of *ELF3* in the PTEN-deficient lung epithelium promoted lung cancer development by inhibiting ferroptosis. Although ELF3 expression is highly elevated in human lung tumors, we found overexpression of *ELF3* in mouse lung epithelium only caused hyperplasia. Molecular analyses revealed that ELF3 overexpression promoted proliferation and ferroptosis at the same time; thus, the induced ferroptosis likely formed a barrier to constrain these hyperplastic lung epithelia from being transformed into lung tumors (Fig. 7 left). Given the co-occurrence of ELF3 overexpression and *PTEN* mutation or deficiency in human lung tumors, we investigated the effect of ELF3 overexpression in promoting lung tumor development under the *Pten* deficient background. Of note, we found that *ELF3* overexpression in the *Pten* deletion mouse lung epithelium significantly promoted lung tumor development by inhibiting ferroptosis while maintaining the proliferation-promoting effect (Fig. 7 right). Mechanistically, we found that this effect is largely dependent on the induced expression of SLC7A11, a typical inhibitor of ferroptosis, in mouse lungs human lung epithelial cells and cancer cells (Fig. 7 right). Importantly, targeting SLC7A11 using its inhibitor, erastin, showed obvious inhibition on the colony formation of lung cancer cells and cancer development with ELF3 overexpression and PTEN deficiency by inducing ferroptosis (Fig. 7).

Our study suggests that inhibition of SLC7A11 using erastin can potentially be an effective treatment strategy to prevent the development of human lung tumors with both *ELF3* overexpression and the downregulated or mutated *PTEN*. Of note, PRLX93936, an analog of erastin, synergizes with Cisplatin to induce ferroptosis in non-small cell lung cancer cells [24], and PRLX93936 has been taken for the clinical trial in treating patients with multiple myeloma (NCT01695590).

As in previous reports, identification of the driver genetic alternations was the foundation of precision medicine in cancer treatment. Although we observed the negative correlation between *ELF3* and *PTEN* expression in a large number of human lung tumors (Fig. 3A, B), it remained to be determined if these alternations drive lung tumor development. In fact, *Pten* deletion alone has been reported to be insufficient inducing lung tumors [5]. Similarly, our current study showed that ELF3 overexpression did not cause lung tumorigenesis. However, we identified the synergistic role of *ELF3* overexpression and PTEN deficiency in driving lung tumor development (Fig. 3G). Moreover, we found that this oncogenic event was mainly owing to the induction of SLC7A11 and its repression of ferroptosis (Fig. 7).

Ferroptosis acts as a barrier to prevent hyperplasia from developing into cancer. Either *ELF3* overexpression or *Pten* deletion alone induced ferroptosis in the hyperplastic lung epithelium (Figs. 2C and 4C), indicating the induction of ferroptosis unlikely to be a direct effect of these genetic alternations. To attenuate ferroptosis, a “two-hit” oncogenic scenario, such as *ELF3* overexpression and *Pten* deletion, is required. Therefore, our findings showed the interplays between the synergistic effects of genetic mutations in cells and the pathologic steps (*e.g*., ferroptosis) of tumor development. Moreover, targeting the synergistic downstream molecule of these interplays, such as SLC7A11, can effectively restore the barrier by inducing ferroptosis.

Mouse-derived Elf3 was found to be up-regulated with mouse lung cancer development in our previous article published in 2015 [5], suggesting its pro-carcinogenic role. Our current Fig. 1 also shows that high expression of human-derived ELF3 in mouse lung epithelial cells promotes cell proliferation. This is consistent with the effect of high expression of ELF3 in human bronchial epithelial cells NL20 and human lung adenocarcinoma H1650 cells, i.e., ELF3 positively regulates cell growth. Combined with the conserved DNA and protein sequences of murine and human ELF3 (Figs. S2 and S3), we concluded that there are similarities in the functionality of human and murine ELF3, such as the regulation of cell growth.

Tumorigenesis is a slow and chronic process with the gradual accumulation of genetic, epigenetic, and metabolic alterations. These alterations occur over an extended period and are involved in a complex network of signaling pathways and metabolic processes. As with other metabolic pathways, the various mechanisms driving tumor initiation and progression must be carefully investigated and cannot be overlooked. Therefore, we planned to conduct relevant follow-up studies to continue exploring the relevant mechanisms of how ELF3 regulates tumor occurrence and development. For instance, we found that the overexpression of ELF3 resulted in the alteration in the p53 pathway, as TP53 is one of the most frequently altered genes in human cancers and has been a focus of oncological research. Gradually increased evidence indicates that p53 regulated multiple cell processes, including cell metabolism, ferroptosis, and tumor environment [25]. Besides, several metabolism pathways were enriched in ELF3 overexpression tissues, such as nucleotide metabolism, lipolysis, and carbon metabolism. Due to the rapid proliferation, cancer cells require a larger support of de novo synthesis of nucleotides, lipids, and amino acids. In this case, overexpressed ELF3 as a critical upstream regulator might promote cell proliferation by enhancing the cell metabolism in these pathways [26].

Further studies are warranted on the underlying mechanisms of the synergistic role of ELF3 overexpression or PTEN deletion in promoting lung tumor development. For example, an increase in ELF3’s transcriptional activity or the remodeled microenvironment in the PTEN-deficient background and the subsequent induction of SLC7A11 might be caused by the loss of the phosphatase activity of PTEN. Examination of the protein modification of ELF3 or the remodeled microenvironment between the wild type and the PTEN-deficient background will be promising research directions.

In summary, our findings revealed that overexpression of ELF3 in the PTEN-deficient lung epithelium promoted lung cancer development by inhibiting ferroptosis partially through the directly induced expression of SLC7A11. With the approval of several drugs (*e.g*., sorafenib, sulfasalazine, and artesunate) by the U.S. Food and Drug Administration (FDA) for treating different cancer types by inducing ferroptosis, the reactivation of ferroptosis could potentially revolutionize the treatment of human lung tumors with ELF3 overexpression and PTEN downregulation or loss-of-mutations.

## Materials and methods

### Generation of Rosa26-LSL-hsElf3 mouse line

The cre-inducible Elf3 expression allele, Rosa26-LSL-hsElf3, was generated via CRISPR/Cas9-assisted ES targeting in the G4 ES cell line (B6129F1). The Cas9 double-stranded break in the Rosa26 endogenous locus was generated by targeting the ACTCCAGTCTTTCTAGAAGANGG sequence. CAS9 nuclease, Cas9 sgRNA, and puromycin resistance were delivered via the pSpCas9(BB)-2A-Puro (PX459) V2.0 plasmid, a gift from Feng Zhang [27]. The repair template includes a 1.1 kb 5′ homology arm, CMV-enhancer, chicken beta-actin promoter (CAG promoter), Loxp-flanked SV40 stop cassette, human ELF3 ORF, WHP Post-transcriptional Regulatory Element (WPRE), BGH polyA signal, and 3.3 kb 3′ homology arm. The repair template was based on a modified version of Ai9, a gift from Hongkui Zeng [28]. The human *ELF3* ORF was amplified from piRES-puro-ELF3, a gift from Ronny Drapkin (Addgene #25728). The Cas9 delivery plasmid and repair template were co-transfected in a molar ratio of 1:5 with Lipofectamine 2000, with clonal selection and expansion following previously published protocols [29]. Targeted clones were identified by a 5′ external PCR screen, a human *ELF3* ORF-specific PCR screen, and an endogenous locus-specific PCR screen (to verify zygosity). Correctly targeted heterozygous ES clones were microinjected into albino C57BL/6J blastocysts, and non-surgically transferred to pseudo-pregnant SWISS/Webster females. Germline chimeras were bred to C57BL/6J mice for at least two generations.

### Mouse models

All animal experiments were conducted following protocols approved by the Experimental Animal Center of the Zhejiang University-University of Edinburgh Institute. By inducing homologous recombination using CRISPR/Cas9, the human *ELF3* gene was knocked into the mouse *Rosa26* locus, and the *ELF3*^OV/+^(CCSP^iCre^/LoxP-STOP-LoxP-h*ELF3*) mouse model was obtained by crossing with CCSP^iCre^ mice [5]. Similarly, crossing CCSP^iCre^ mice with *Pten*^f/f^ mice (PMID: 11857804) generated *Pten*^*d/d*^ (CCSP^iCre^/*Pten*^f/f^), *Pten*^d/d^*ELF3*^OV/+^(CCSP^iCre^/ *Pten*^f/f^/LoxP-STOP-LoxP-h*ELF3*) mice. The lung tissues were collected from mice at 12 months of age for freezing and fixation.

### Cell lines

The HEK-293T, NL20 and H1650 cell lines were obtained from ATCC. HEK-293T cells were cultured in DMED (Sigma) with 10% FBS and 1% penicillin/streptomycin (Beyotime, C0222). NL20 cells were cultured in DMEM/F-12 (Sigma) with 5 mg/L insulin (Solarbio I8830), 10 μg/L epidermal growth factor (Solarbio P00033), 1 mg/L transferrin (Solarbio P00033), 500 μg/L hydrocortisone (Solarbio IH0100) and 4% FBS. H1650 cells were cultured in RPMI-1640 (Sigma) with 10% FBS and 1% penicillin-streptomycin (Beyotime C0222). At 80–90% confluence, cells were detached with 0.25% trypsin-EDTA (Gibco 25200072). For cell colony formation assays, 1000 cells per well were seeded into six-well plates, with the medium changed every two days, for CCK-8 assays (Vazyme A311-01), 4000 cells per well were seeded into 96-well plates, and after cells adhered, the medium was replaced with erastin (MCE HY-15763)-containing medium, followed by detection after 24 h of culture. *ELF3*^ov^, *PTEN*^−/−^ and *PTEN*^−/−^*ELF3*^ov^ cells were generated by CRISPR/Cas9-mediated genetic editing techniques. All cell lines were routinely PCR-tested for *Mycoplasma*.

### Plasmids

psPAX2, pMD2.G, lentiCRISPR-sg*PTEN*-*puro*, pGL3-Basic, pRL-TK plasmids were obtained from Addgene, and pLVX-*puro* plasmid was purchased from MiaoLing Biology. The *ELF3* open reading frame (ORF) and *SLC7A11* promoter containing the ELF3 motif were amplified from human cDNA by PCR. After digestion with EcoRI and XbaI, the *ELF3* ORF was cloned into the pLVX-*puro* plasmid to generate the pLVX-*ELF3*-*puro* plasmid. After digestion with KpnI and XmaI, the *SLC7A11* promoter was cloned into the pGL3-Basic plasmid to obtain the pGL3-*SLC7A11* promoter-Basic plasmid. Primer sequences and concentrations used were ELF3 ORF, 10 μM, F:CGGAATTCATGGCTGCAACCTGTGAGAT/R:GCTCTAGATCAGTTCCGACTCTGGAGAA, *SLC7A11* promoter, 10 μM, F:GGGGTACCCCAAAATCTCTTTAAAGTGTGTGCTTTG/R:TCCCCCCGGGGGGAAGTAGGGACACACGG. All plasmids were sequenced and subjected to endotoxin-free extraction.

### Histologic analysis

Mice lung tissues were fixed in 4% paraformaldehyde (PFA) and embedded in paraffin. Sections (5 μm) were deparaffinized, rehydrated, and stained with hematoxylin (Solarbio G1140) and eosin (Solarbio G1100) for H&E, or performed enhanced iron staining for Prussian Blue Staining (Solarbio G1428). For IHC, sections (5 μm) were deparaffinized, rehydrated, then subjected to antigen retrieval at 100 °C using a microwave for 25 minutes. Antigen retrieval was performed using Antigen Unmasking Solution (Vector Laboratories H-3300-250) /1 × TE Buffer. Endogenous peroxidase activity was blocked using an IHC Kit (PV-6000) from ZSGB Bio. 5% BSA was used for room temperature blocking for 1 h. The primary antibodies and concentration used were ELF3 (Sigma HPA003316, 1:1000), Ki67 (Abcam ab15580, 1:2000), Slc7a11 (Abcam ab307601, 1:500). All primary antibodies were incubated overnight. After the incubation with the secondary antibody from the ZSGB IHC Kit, Vectastain ABC Kit (Vector Laboratories PK-6100), ZSGB DAB, and hematoxylin staining were performed. Slides were imaged using a Nikon Upright Fluorescence Microscope.

### RNA extraction and RT-qPCR

Total RNA was extracted from cell lines or frozen mice lung samples using RNAiso Plus (Takara 9109). The tissue was homogenized into a uniform paste before extraction. cDNA was synthesized using HiScript III All-in-one RT SuperMix (Vazyme R333-01). RT-qPCR was conducted using ChamQ Universal SYBR qPCR Master Mix (Vazyme Q711-02) and the 2^−ΔΔCt^ method was used to determine relative mRNA expressions compared with controls. Primer sequences used were *ELF3* (10 μM), F:CAACTATGGGGCCAAAAGAA/R:TTCCGACTCTGGAGAACCTC, *SLC7A11* (10 μM), F:GCGTGGGCATGTCTCTGAC/R:GCTGGTAATGGACCAAAGACTTC, *Slc7a11* (10 μM), F:GTGGAACTGCTCGTAATACGC/R:CGTGCTATTTAGGACCATCACC.

### Western blots

Cells were lysed using RIPA (Beyotime P0013B) with PMSF (Beyotime ST506). Protein determination was performed using the Enhanced BCA Protein Assay Kit (Beyotime P0010). Proteins were separated by SDS-PAGE gel and transferred onto nitrocellulose membranes. After being blocked in 5% skim milk for 1 h, membranes were probed with various primary antibodies overnight at 4 °C, including ELF3 (Abcam ab133621, 1:2000), PTEN (CST 9559S, 1:500), SLC7A11 (Abcam ab307601, 1:1000), β-actin (Fude FD0060, 1:5000), followed by incubation with horseradish peroxidase-linked secondary antibodies for 1 h at room temperature, and visualized using ECL (Biosharp BL520A).

### MDA and GSH assays

The concentration of Malondialdehyde (MDA) and GSH were measured using Lipid Peroxidation MDA Assay Kit (Beyotime S0131M) and Total Glutathione Assay Kit (Beyotime S0052). Cells or tissue lysates were prepared. For the MDA assay, lysates were reacted with MDA assay solution at 100 °C for 15 minutes, then centrifuged at 1000 × *g* for 10 min. The absorbance of the supernatant at 535 nm was measured on a Tecan Spark Microplate Reader. For GSH assay, lysates were reacted with GSH assay solution for 25 min at 25 °C. The absorbance at 535 nm/412 nm was measured on the Tecan Spark Microplate Reader. The levels of MDA and GSH were calculated based on the concentration of proteins from an equal amount of cells or tissues.

### Transmission electron microscopy

Pre-prepared NL20 WT, *ELF3*^*ov*^*, PTEN*^*−/−*^ and *PTEN*^*−/−*^*ELF3*^*ov*^ cells were digested with trypsin. After that, the digestion was terminated with culture medium, and the samples were centrifuged at 1000 rpm for 6 min to discard the supernatant. A 2.5% glutaraldehyde (pH 7.2–7.4) fixative was added to suspend the cell pellet. The samples were fixed at room temperature in the dark for 30 minutes before being stored at 4 °C. All TEM images of the samples were supported by Biossci.

### Luciferase assays

A 24-well plate with HEK-293T cells was prepared and transfected with siRNA (Tsingke) and plasmids separately as follows: siControl + pGL3-*SLC7A11* promoter-Basic + pLVX-puro + pRL-TK, siControl + pGL3-*SLC7A11* promoter-Basic + pLVX-*ELF3*-puro + pRL-TK, si*PTEN* + pGL3-*SLC7A11* promoter-Basic + pLVX-puro + pRL-TK, si*PTEN* + pGL3-*SLC7A11* promoter-Basic + pLVX-*ELF3*-puro + pRL-TK, and an untransfected blank control group. Renilla luciferase gene (pRL-TK) was used as an internal reference. Cell extracts were prepared 48 h after transfection and luciferase activity was measured with the Dual-Luciferase Reporter Gene Assay Kit (YEASEN 11402ES60) on Tecan Spark Microplate Reader.

### RNA-seq analysis

Raw reads were filtered by trim galore and were aligned to human genome build 37 (hg19) using the Hisat2 (v2.2.1) software. Paired reads were assigned to genes by Subreads/featureCounts (v2.0.1) based on the GTF file from genecode (v19). Differentially expressed genes were identified and normalized by the Deseq2 (v 1.30.1) package. Genes were considered as differential expression genes (DEGs) if the fold change between tumor and normal tissue is significant enough (|Log2FC| ≥ 0.20), and the *P* value is smaller than 0.05. The gene expression heatmap was done with the R package ComplexHeatmap (v2.14.0) to confirm the similar expression patterns between replicates. Pathway enrichment was performed using the Gene Ontology and KEGG pathway enrichment through the R package clusterProfiler (v3.18.1).

### Analysis based on patient data from public database

The expression level of ELF3 between various cancer types and corresponding normal tissues was collected from the database TIMER2.0 (http://timer.comp-genomics.org/timer/). The survival curve was performed and the *P* value was calculated by Kaplan-Meier Plotter (http://kmplot.com/analysis/) and R packages, survival (v3.5.7) and survminer (v0.4.9), based on published data of patients from TCGA database. Spearman correlation between different gene signatures and the *P* value was calculated using R package stats (v4.0.5). Boxplots were generated by R package reshape2 (v1.4.4) and ggplot2 (v3.4.3) to depict the expression level of genes between groups.

### ChIP-qPCR

ChIP–qPCR assay in NL20 *ELF3*^*ov*^ and *PTEN*^*−/−*^*ELF3*^*ov*^ cell lines was performed to verify the relationship between ELF3 and *SLC7A11* promoter. Briefly, cross-linked cell lysates were sonicated and subjected to immunoprecipitation. The immunoprecipitated DNA was purified with Phase Lock GelTM (PLG) Heavy (TIANGEN WM5-2302830). Two percent of each sample volume was separated as the input. ChIP for ELF3 was performed using an ELF3 antibody (Abcam ab133621, 1:100). The purified DNA fragments were analyzed by qPCR. Primer sequences were as follows: *SLC7A11* promoter region (10 μM), F:CATACACAGGTGTTTCTGAGTAGTA/R:ACTTTCAACTTTGGTGTCTCTTGGT, untranscribed region (10 μM), F:AGGCAGTGGTTACTGCAAGC/R:CAAACAAAGGATCTGTCTTCTGAGC.

### Xenograft

All female nude mice (4–6 weeks old) were purchased from GemPharmatechand (Jiangsu, China), subcutaneously injected with H1650 *PTEN*^null^/*PTEN*^null^*ELF3*^ov^ cells (1 × 10^6^ cells/point). Nude mice were monitored, xenograft tumor volumes were measured with a sliding caliper, and tumor volumes were calculated using the formula (*L* × *W*^2^)/2. When xenograft tumors reached approximately 100 mm^3^, they were randomly divided into two groups for intraperitoneal injections of vehicle/erastin, administered every three days. After two weeks, the mice were euthanized, and the xenograft tumors were collected. The volume of the xenograft tumors was measured every three days, and the nude mice and xenograft tumors were weighed at the end point of the treatment.

## Supplementary information

Supplementary Figures and Tables

Original WB

## Data Availability

The RNA-Seq raw data was deposited to CNCB NGDC (CRA015996).

## References

[1] Leiter A, Veluswamy RR, Wisnivesky JP. The global burden of lung cancer: current status and future trends. Nat Rev Clin Oncol. 2023;20:624–39. 37479810 10.1038/s41571-023-00798-3

[2] Brea E, Rotow J. Targeted therapy for non-small cell lung cancer first line and beyond. Hematol Oncol Clin N. 2023;37:575–94. 10.1016/j.hoc.2023.02.009 37024384

[3] McFadden DG, Politi K, Bhutkar A, Chen FK, Song XL, Pirun M, et al. Mutational landscape of EGFR-, MYC-, and Kras-driven genetically engineered mouse models of lung adenocarcinoma. Proc Natl Acad Sci USA. 2016;113:E6409–E17. 27702896 10.1073/pnas.1613601113 PMC5081629

[4] Zhao YC, Li YQ, Zhang RF, Wang F, Wang TJ, Jiao Y. The role of erastin in ferroptosis and its prospects in cancer therapy. Oncotargets Ther. 2020;13:5429–41. 10.2147/OTT.S254995 PMC7295539 32606760

[5] Liu J, Cho SN, Akkanti B, Jin NL, Mao JQ, Long WW, et al. ErbB2 pathway activation upon loss promotes lung tumor growth and metastasis. Cell Rep. 2015;10:1599–613. 25753424 10.1016/j.celrep.2015.02.014 PMC7405934

[6] Wang H, Yu ZQ, Huo SF, Chen Z, Ou ZL, Mai JJ, et al. Overexpression of ELF3 facilitates cell growth and metastasis through PI3K/Akt and ERK signaling pathways in non-small cell lung cancer. Int J Biochem Cell Biol. 2018;94:98–106. 29208568 10.1016/j.biocel.2017.12.002

[7] Enfield KSS, Marshall EA, Anderson C, Ng KW, Rahmati S, Xu Z, et al. Epithelial tumor suppressor ELF3 is a lineage-specific amplified oncogene in lung adenocarcinoma. Nat Commun. 2019;10:5438. 31780666 10.1038/s41467-019-13295-y PMC6882813

[8] Subbalakshmi AR, Sahoo S, Manjunatha P, Goyal S, Kasiviswanathan VA, Mahesh Y, et al. The ELF3 transcription factor is associated with an epithelial phenotype and represses epithelial-mesenchymal transition. J Biol Eng. 2023;17:17. 36864480 10.1186/s13036-023-00333-z PMC9983220

[9] Li J, Cao F, Yin HL, Huang ZJ, Lin ZT, Mao N, et al. Ferroptosis: past, present and future. Cell Death Dis. 2020;11:88. 32015325 10.1038/s41419-020-2298-2 PMC6997353

[10] Mao XD, Liu KS, Shen SQ, Meng LJ, Chen SZ. Ferroptosis, a new form of cell death: mechanisms, biology and role in gynecological malignant tumor. Am J Cancer Res. 2023;13:2751–62. 37559994 PMC10408495

[11] Koppula P, Zhuang L, Gan BY. Cystine transporter SLC7A11/xCT in cancer: ferroptosis, nutrient dependency, and cancer therapy. Protein Cell. 2021;12:599–620. 33000412 10.1007/s13238-020-00789-5 PMC8310547

[12] Zhou Q, Meng Y, Li DS, Yao L, Le JY, Liu YH, et al. Ferroptosis in cancer: From molecular mechanisms to therapeutic strategies. Signal Transduct Target Ther. 2024;9:55. 38453898 10.1038/s41392-024-01769-5 PMC10920854

[13] Kim R, Taylor D, Vonderheide RH, Gabrilovich DI. Ferroptosis of immune cells in the tumor microenvironment. Trends Pharmacol Sci. 2023;44:542–52. 37380530 10.1016/j.tips.2023.06.005

[14] Dang Q, Sun ZQ, Wang Y, Wang LB, Liu ZQ, Han XW. Ferroptosis: a double-edged sword mediating immune tolerance of cancer. Cell Death Dis. 2022;13:925. 36335094 10.1038/s41419-022-05384-6 PMC9637147

[15] Jiang XJ, Stockwell BR, Conrad M. Ferroptosis: mechanisms, biology and role in disease. Nat Rev Mol Cell Bio. 2021;22:266–82. 33495651 10.1038/s41580-020-00324-8 PMC8142022

[16] Dyson NJ. : a prototype tumor suppressor and an enigma. Gene Dev. 2016;30:1492–502. 27401552 10.1101/gad.282145.116 PMC4949322

[17] Ma TY, Du JT, Zhang YF, Wang YY, Wang BX, Zhang TH. GPX4-independent ferroptosis-a new strategy in disease’s therapy. Cell Death Discov. 2022;8:434. 36309489 10.1038/s41420-022-01212-0 PMC9617873

[18] Zheng LB, Xu M, Xu JJ, Wu K, Fang Q, Liang YL, et al. ELF3 promotes epithelial-mesenchymal transition by protecting ZEB1 from miR-141-3p-mediated silencing in hepatocellular carcinoma. Cell Death Dis. 2018;9:387. 29523781 10.1038/s41419-018-0399-y PMC5845010

[19] Heidari M, Johnstone DM, Bassett B, Graham RM, Chua ACG, House MJ, et al. Brain iron accumulation affects myelin-related molecular systems implicated in a rare neurogenetic disease family with neuropsychiatric features. Mol Psychiatr. 2016;21:1599–607. 10.1038/mp.2015.192 PMC5078858 26728570

[20] Liao Z, Wen E, Feng Y, GSH-responsive degradable nanodrug for glucose metabolism intervention and induction of ferroptosis to enhance magnetothermal anti-tumor therapy. J Nanobiotechnol. 2024;22:147. 10.1186/s12951-024-02425-4 PMC11321096 38570829

[21] Yu Y, Yan Y, Niu FL, Wang YJ, Chen XY, Su GD, et al. Ferroptosis: a cell death connecting oxidative stress, inflammation and cardiovascular diseases. Cell Death Discov. 2021;7:193. 34312370 10.1038/s41420-021-00579-w PMC8313570

[22] Yan YL, Teng HQ, Hang QL, Kondiparthi L, Lei G, Horbath A. et al. SLC7A11 expression level dictates differential responses to oxidative stress in cancer cells. Nat Commun. 2023;14:3673. 37339981 10.1038/s41467-023-39401-9 PMC10281978

[23] Xu XT, Zhang XY, Wei CQ, Zheng DX, Lu X, Yang YY, et al. Targeting SLC7A11 specifically suppresses the progression of colorectal cancer stem cells via inducing ferroptosis. Eur J Pharm Sci. 2020;152:105450. 32621966 10.1016/j.ejps.2020.105450

[24] Liang ZG, Zhao WJ, Li XJ, Wang LF, Meng LF, Yu R. Cisplatin synergizes with PRLX93936 to induce ferroptosis in non-small cell lung cancer cells. Biochem Biophys Res Co. 2021;569:79–85. 10.1016/j.bbrc.2021.06.088 34237431

[25] Wang HL, Guo M, Wei HD, Chen YH. Targeting p53 pathways: mechanisms, structures, and advances in therapy. Signal Transduct Target Ther. 2023;8:92. 36859359 10.1038/s41392-023-01347-1 PMC9977964

[26] Martínez-Reyes I, Chandel NS. Cancer metabolism: looking forward. Nat Rev Cancer. 2021;21:669–80. 34272515 10.1038/s41568-021-00378-6

[27] Ran FA, Hsu PD, Wright J, Agarwala V, Scott DA, Zhang F. Genome engineering using the CRISPR-Cas9 system. Nat Protoc. 2013;8:2281–308. 24157548 10.1038/nprot.2013.143 PMC3969860

[28] Madisen L, Zwingman TA, Sunkin SM, Oh SW, Zariwala HA, Gu H, et al. A robust and high-throughput Cre reporting and characterization system for the whole mouse brain. Nat Neurosci. 2010;13:133–U311. 20023653 10.1038/nn.2467 PMC2840225

[29] Gruzdev A, Scott GJ, Hagler TB, Ray MK. CRISPR/Cas9-assisted genome editing in murine embryonic stem cells. Methods Mol Biol. 2019;1960:1–21. 30798517 10.1007/978-1-4939-9167-9_1

