# Correction: Overexpression of ELF3 in the PTEN-deficient lung epithelium promotes lung cancer development by inhibiting ferroptosis

**DOI:** 10.1038/s41419-025-07397-3

**Published:** 2025-02-10

**Authors:** Zengzhuang Yuan, Xinyan Han, Manyu Xiao, Taoyu Zhu, Yaping Xu, Qian Tang, Chen Lian, Zijin Wang, Junming Li, Boyu Wang, Changhui Li, Xiaochen Xiang, Ruobai Jin, Yufei Liu, Xinyu Yu, Kehang Zhang, Songsong Li, Madhumita Ray, Rong Li, Artiom Gruzdev, Shiqun Shao, Fangwei Shao, Hua Wang, Lian Wang, Yong Tang, Di Chen, Ying Lei, Xuru Jin, Qinglin Li, Weiwen Long, Huaqiong Huang, Francesco J. DeMayo, Jian Liu

**Affiliations:** 1 Department of Respiratory and Critical Care Medicine, the Second Affiliated Hospital, and Centre for Infection Immunity and Cancer (IIC) of Zhejiang University-University of Edinburgh Institute (ZJU-UoE Institute), Zhejiang University School of Medicine, Zhejiang University, Hangzhou, China; 2 Edinburgh Medical School: Biomedical Sciences, College of Medicine and Veterinary Medicine, The University of Edinburgh, Edinburgh, UK; 3 Reproductive & Developmental Biology Laboratory, National Institute of Environmental Health Sciences (NIEHS), Research Triangle Park, NC USA; 4 Department of Obstetrics, Gynecology and Women’ Health, University of Missouri, Columbia, MO USA; 5 Gene Editing and Mouse Model Core, National Institute of Environmental Health Sciences (NIEHS), Research Triangle Park, NC USA; 6 Zhejiang Key Laboratory of Smart Biomaterials and Center for Bionanoengineering, College of Chemical and Biological Engineering, Zhejiang University, Hangzhou, China; 7 Zhejiang University-University of Illinois Urbana-Champaign Institute, Zhejiang University, Haining, China; 8 Biomedical and Heath Translational Research Center of Zhejiang Province, Haining, Zhejiang China; 9 National Key Laboratory of Biobased Transportation Fuel Technology, ZJU-UIUC Institute, Zhejiang University, Hangzhou, China; 10 Department of Materials Science and Engineering, University of Illinois at Urbana-Champaign, Urbana, IL USA; 11 Department of Thoracic Surgery, the Second Affiliated Hospital, Zhejiang University School of Medicine, Zhejiang University, Hangzhou, China; 12 Department of Thoracic Surgery, Shenzhen Nanshan People’s Hospital, Shenzhen, China; 13 Center for Regeneration and Cell Therapy of Zhejiang University-University of Edinburgh Institute (ZJU-UoE Institute), Zhejiang University School of Medicine, Zhejiang University, Hangzhou, Zhejiang China; 14 Department of Respiratory and Critical Care Medicine, The Quzhou Affiliated Hospital of Wenzhou Medical Hospital, Quzhou People’s Hospital, Wenzhou, China; 15 Department of Respiratory and Critical Care Medicine, The First Affiliated Hospital of Wenzhou Medical University, Wenzhou, China; 16 Key Laboratory of Head & Neck Cancer Translational Research of Zhejiang Province, Zhejiang Cancer Hospital, Hangzhou, Zhejiang China; 17 Department of Biochemistry and Molecular Biology, Boonshoft School of Medicine, Wright State University, Dayton, OH USA; 18 Key Laboratory of Respiratory Disease of Zhejiang Province, Department of Respiratory and Critical Care Medicine, Second Affiliated Hospital of Zhejiang University School of Medicine, Hangzhou, Zhejiang China; 19 Zhejiang Key Laboratory of Medical Imaging Artificial Intelligence, Haining, Zhejiang China; 20 Dr. Li Dak Sum & Yip Yio Chin Center for Stem Cell and Regenerative Medicine, Zhejiang University, Hangzhou, China

**Keywords:** Non-small-cell lung cancer, Cancer genomics

Correction to: *Cell Death and Disease* 10.1038/s41419-024-07274-5, published online 18 December 2024

In this article the gene name “SCL7A11” has been misspelled. It should read “SLC7A11”.

The original article has been corrected.

