# Supplementary material for: Overexpression of ELF3 in the PTEN-deficient lung epithelium promotes lung cancer development by inhibiting ferroptosis
Source: Cell Death Dis. 2024 Dec 18;15(12):897. doi: 10.1038/s41419-024-07274-5 (PMC11655876; doi:10.1038/s41419-024-07274-5)
Supplement: Supplementary file 1 — Supplementary Figures and Tables [file 41419_2024_7274_MOESM1_ESM.pdf]

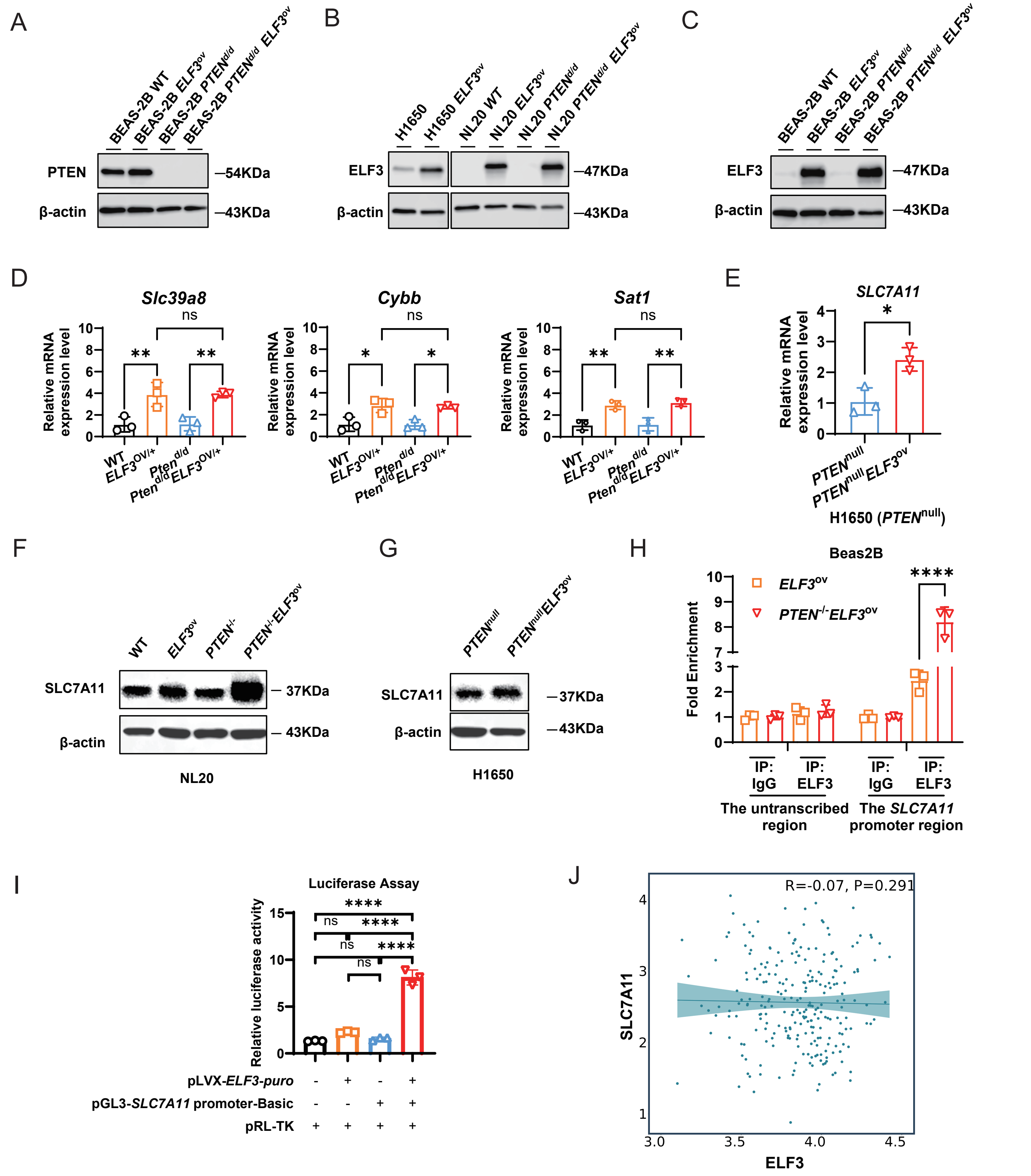

Supplementary Fig. S1. (A) PTEN expression in BEAS-2B cell lines detected by WB. (B) ELF3 expression in NL20 and H1650 cell lines detected by WB. (C) ELF3 expression in BEAS-2B cell lines detected by WB. (D) Gene expression levels of *Slc39a8, Cybb and Sat1* in mouse lung tissue samples detected by RT-qPCR. (E) Gene expression levels of ELF3 in H1650 were detected by RT-qPCR. (F & G) slc7a11 expression in NL20 and H1650 cell lines detected by WB. (H) ELF3 binding strength on the SLC7A11 promoter region detected by ChIP-qPCR. (I) Luciferase assay detecting the expression of luciferase induced by ELF3 to the SLC7A11 promoter region. (J) The relationship between the expression levels of ELF3 and SLC7A11 in lung cancer patients.

```
Score = 17110.0
Length of alignment = 372
Sequence NP_031947.1/1-371 (Sequence length = 371)
Sequence  CAG33387.1/1-371 (Sequence length = 371)

NP_031947.1/1-371 MAATCEISNVFSNYFNAMYSSEDPTLAPAPPT-TFGTEDLVLTLNNQQMTLEGP
                  |||||||||.|||||.|||||||.|||. ||. |||..||||||.| ||.|||
 CAG33387.1/1-371 MAATCEISNIFSNYFSAMYSSEDSTLASVPPAATFGADDLVLTLSNPQMSLEGT

NP_031947.1/1-371 EKASWTSERPQFWSKTQVLEWISYQVEEKNKYDASSIDFSRCDMDGATLCSCALE
                  ||||| .|.||||||||||.|||||||||||||||.||||||||||||||.||||
 CAG33387.1/1-371 EKASWLGEQPQFWSKTQVLDWISYQVEEKNKYDASAIDFSRCDMDGATLCNCALE

NP_031947.1/1-371 ELRLVFGPLGDQLHAQLRDLTSNSSDELSWIIELLEEDGMSFQESLGDSGPFDQ
                  ||||||||||||||||||||||.|||||||||||||||||.|||.| |.|||||
 CAG33387.1/1-371 ELRLVFGPLGDQLHAQLRDLTSSSSDELSWIIELLEEDGMAFQEAL-DPGPFDQ

NP_031947.1/1-371 GSPFAQELLDDGRQASPYYCSTYGPGAPSPGSSDVSTARTATPQSSHASDSGGS
                  ||||||||||||.|||||  .. |.||||||||||||| |....|||.||||||
 CAG33387.1/1-371 GSPFAQELLDDGQQASPYHPGSCGAGAPSPGSSDVSTAGTGASRSSHSSDSGGS

NP_031947.1/1-371 DVDLDLTESKVFPRDGFPDYKKGEPKHGKRKRGRPRKLSKEYWDCLEGKKSKHA
                  ||||| |..|.|| ||| | |||.|| |||||||||||||||||||||||||||
 CAG33387.1/1-371 DVDLDPTDGKLFPSDGFRDCKKGDPKLGKRKRGRPRKLSKEYWDCLEGKKSKHA

NP_031947.1/1-371 PRGTHLWEFIRDILIHPELNEGLMKWENRHEGVFKFLRSEAVAQLWGQKKKNSN
                  ||||||||||||||||||||||||||||||||||||||||||||||||||||||
 CAG33387.1/1-371 PRGTHLWEFIRDILIHPELNEGLMKWENRHEGVFKFLRSEAVAQLWGQKKKNSN

NP_031947.1/1-371 MTYEKLSRAMRYYYKREILERVDGRRLVYKFGKNSSGWKEEEVGESRN
                  ||||||||||||||||||||||||||||||||||||||||||| .|||
 CAG33387.1/1-371 MTYEKLSRAMRYYYKREILERVDGRRLVYKFGKNSSGWKEEEVLQSRN

Percentage ID = 87.10
```

Supplementary Fig. S2. Alignment result of human and mouse ELF3 amino acid sequence.

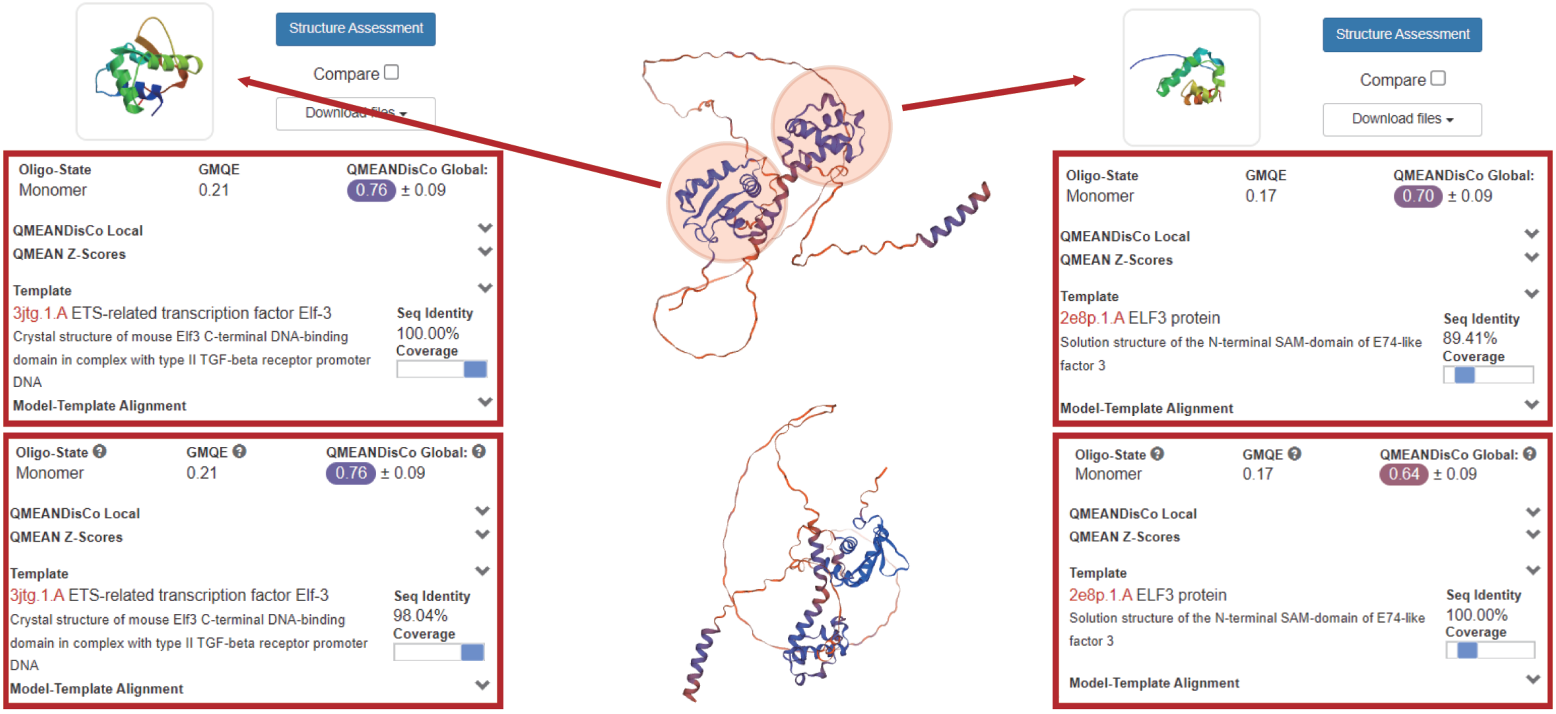

Supplementary Fig. S3. Structural and functional comparasion between the hELF3 (human ELF3) and mELF3 (mouse ELF3)

Supplementary Table 1 DEGs between the ELF3^{OV} vs WT groups

gene_id	baseMean	log2FoldC	lfcSE	stat	pvalue	padj	WT1	WT2	WT3	ELF3OV1	ELF3OV2	ELF3OV3	change
Gm6123	103.642	-4.5	0.47359	-9.502	2.06E-21	2.62E-18	152.769	293.721	149.043	6.13035	8.91451	11.2735	Down
Rgs20	78.37	-1.3966	0.58363	-2.3929	0.01672	0.21274	72.935	122.631	145.147	32.6952	15.848	80.9644	Down
St18	19.0981	1.21827	0.60129	2.0261	0.04275	0.37291	18.7266	8.90062	6.81897	32.6952	20.8005	26.6465	Up
Tcf24	152.408	-0.491	0.21919	-2.2402	0.02508	0.27353	178.395	154.277	201.647	122.607	139.661	117.86	Down
Slco5a1	126.151	-0.7139	0.30682	-2.3266	0.01999	0.23859	184.309	169.112	116.897	90.9336	120.841	74.8152	Down
Eya1	354.613	-0.7729	0.33819	-2.2854	0.02229	0.25495	295.683	599.309	447.13	221.714	202.062	361.778	Down
Il17a	2.88035	4.99332	2.08748	2.39204	0.01676	0.21309	0	0	0	1.02173	3.962	12.2984	Up
Mcm3	1217.81	0.42573	0.19172	2.22053	0.02638	0.28094	1191.6	829.736	1096.88	1583.67	1336.19	1268.78	Up
Gm28438	545.982	-3.9229	0.23801	-16.482	4.96E-61	1.53E-57	879.163	1187.74	1006.29	55.1732	60.4206	87.1136	Down
Gm5525	171.431	0.42848	0.1881	2.27792	0.02273	0.25741	144.884	156.255	137.354	184.932	197.11	208.048	Up
4931428L	160.275	-1.0211	0.27583	-3.7019	0.00021	0.00987	188.251	260.096	195.802	96.0422	86.1736	135.282	Down
Rab23	1744.59	-0.3022	0.14641	-2.064	0.03902	0.3548	1764.24	2159.88	1855.73	1499.89	1462.97	1724.85	Down
Bag2	863.956	-0.5591	0.21343	-2.6196	0.0088	0.13938	918.587	1222.35	946.863	714.186	554.68	827.067	Down
Bend6	148.208	0.57068	0.22988	2.48251	0.01305	0.17905	130.1	119.664	108.129	154.281	208.996	168.078	Up
Gm3052	91.9737	0.9803	0.28438	3.44715	0.00057	0.02084	57.1653	55.3817	73.0604	142.02	124.803	99.412	Up
Ccdc115	1428.41	0.34988	0.13883	2.52023	0.01173	0.16717	1162.03	1245.1	1360.87	1599	1455.05	1748.42	Up
Plekhb2	7888.88	0.31861	0.153	2.08243	0.0373	0.34397	7994.27	6484.6	6585.18	8267.8	9876.28	8125.14	Up
Lonrf2	33.1172	-1.2161	0.61963	-1.9626	0.04969	0.39986	26.6114	83.0725	29.2242	28.6083	16.8385	14.3481	Down
Chst10	100.769	0.51856	0.26367	1.96666	0.04922	0.39755	93.6328	67.2491	87.6725	129.759	123.813	102.487	Up
Npas2	399.893	-2.0461	0.47854	-4.2758	1.90E-05	0.0014	225.704	987.969	717.94	136.911	123.813	207.023	Down
Il1rl1	204.274	0.61727	0.20022	3.08297	0.00205	0.05157	167.553	160.211	155.862	281.996	210.977	249.042	Up
Slc9a4	503.809	1.32039	0.47988	2.75148	0.00593	0.10748	458.308	250.206	155.862	819.424	1017.24	321.808	Up
Fhl2	793.325	-2.3668	0.4579	-5.1687	2.36E-07	3.91E-05	700.768	2435.8	850.423	178.802	303.093	291.062	Down
Ecrg4	51.0656	-0.8062	0.4015	-2.0079	0.04466	0.37936	55.1941	92.9621	46.7587	39.8473	32.6865	38.9449	Down
Nabp1	2227.26	0.59778	0.30198	1.97953	0.04776	0.39169	1823.38	1912.64	1581.03	2439.88	3941.2	1665.41	Up
Gm28055	84.4054	-0.6424	0.27958	-2.2977	0.02158	0.25004	92.6472	109.774	106.181	75.6077	51.506	70.7157	Down
Gm8420	8.07115	-2.3132	0.85437	-2.7075	0.00678	0.1171	10.8417	15.8233	13.6379	2.04345	1.981	4.09946	Down
Stat1	3443.7	0.50849	0.21782	2.33447	0.01957	0.236	2882.91	3067.75	2578.54	3804.91	5227.86	3100.22	Up
Mstn	4.94248	-3.7638	1.58731	-2.3712	0.01773	0.22084	5.91365	17.8012	3.89655	2.04345	0	0	Down
Osgepl1	748.097	-0.4669	0.17574	-2.6565	0.00789	0.12886	811.156	1025.55	767.621	679.447	632.93	571.875	Down
Rftn2	1449.59	-0.3259	0.16616	-1.9611	0.04987	0.39986	1461.66	1747.49	1628.76	1405.89	1381.75	1072.01	Down
Casp8	3100.2	0.33294	0.147	2.26491	0.02352	0.26285	2986.39	2609.86	2636.02	3650.62	3742.11	2976.21	Up
Cd28	341.842	0.69067	0.28275	2.44264	0.01458	0.19345	297.654	259.107	227.948	458.755	531.899	275.689	Up
Ctla4	202.26	1.14537	0.3914	2.92638	0.00343	0.07439	133.057	148.344	96.4397	411.755	275.359	148.606	Up
Icos	280.389	0.83574	0.26465	3.15792	0.00159	0.04285	215.848	170.101	218.207	387.234	437.801	253.142	Up
Ndufs1	1032.02	-0.7745	0.20403	-3.796	0.00015	0.0073	1189.63	1646.62	1071.55	724.403	825.087	734.829	Down
Gm28982	46.9733	-0.8805	0.35374	-2.4892	0.0128	0.17714	60.1221	48.4589	74.0345	36.7821	31.696	30.746	Down
Fzd5	1380.62	0.31255	0.13103	2.38532	0.01706	0.216	1271.44	1093.79	1329.7	1550.98	1562.02	1475.81	Up
Akr1cl	27.5278	-2.6099	0.71234	-3.6639	0.00025	0.01111	30.5539	87.0283	24.3535	10.2173	8.91451	4.09946	Down
Rpe	2158.71	0.27187	0.1329	2.04578	0.04078	0.36329	2140.74	1821.66	1905.42	2359.16	2551.53	2173.74	Up
Kansl1l	2014.24	0.35592	0.13334	2.6692	0.0076	0.12601	1816.48	1640.68	1844.04	2214.08	2502.01	2068.18	Up
Myl1	95.2191	-2.2386	0.8604	-2.6018	0.00927	0.14356	66.0358	339.213	66.2414	16.3476	75.2781	8.19893	Down
Erbb4	206.043	-1.0934	0.54454	-2.008	0.04464	0.37936	173.467	453.932	214.31	137.933	47.544	209.073	Down
Vwc2l	3.55695	3.30045	1.55459	2.12304	0.03375	0.32407	0	0.98896	0.97414	8.1738	1.981	9.22379	Up
Bard1	115.307	0.77	0.30348	2.53724	0.01117	0.16237	95.604	91.9731	68.1897	181.867	146.594	107.611	Up
Gm8885	3.7679	-5.3389	1.67829	-3.1811	0.00147	0.04083	5.91365	8.90062	7.79311	0	0	0	Down
C530043A	87.1147	-2.5656	0.88035	-2.9143	0.00357	0.07578	54.2085	347.124	45.7845	20.4345	5.94301	49.1936	Down
Arpc2	18296.3	0.21115	0.10462	2.01817	0.04357	0.37434	17722.2	16357.4	16800	19229.9	21002.6	18665.9	Up
Stk36	1473.88	-0.5625	0.26552	-2.1185	0.03414	0.32547	1647.94	1754.41	1870.35	1157.61	785.467	1627.49	Down
Ihh	89.9193	-1.0242	0.28482	-3.5961	0.00032	0.0136	136.014	112.741	113	45.9776	71.3161	60.4671	Down
Resp18	40.8273	-1.8185	0.71701	-2.5362	0.01121	0.1626	76.8775	61.3154	52.6035	15.3259	2.9715	35.8703	Down
Des	8305.96	-0.8628	0.25133	-3.4329	0.0006	0.02174	7773.5	14829.4	9551.43	5692.03	5449.74	6539.67	Down
Speg	553.159	-0.9498	0.26985	-3.5196	0.00043	0.01691	658.387	983.024	545.518	349.43	454.64	327.957	Down
Obsl1	784.729	-0.7138	0.1922	-3.7141	0.0002	0.00953	933.371	1146.2	845.552	629.383	653.731	500.135	Down
Inha	369.188	-0.72	0.21348	-3.3727	0.00074	0.02531	476.049	535.026	367.25	260.54	257.53	318.733	Down
Slc4a3	1333.54	-0.8705	0.23161	-3.7586	0.00017	0.00835	1388.72	2276.58	1506.99	825.554	950.881	1052.54	Down
Gm29069	2.03203	4.48997	2.20757	2.0339	0.04196	NA	0	0	0	1.02173	2.9715	8.19893	Up
Epha4	891.206	-0.4982	0.23806	-2.0927	0.03637	0.33907	1114.72	1274.77	741.319	738.707	831.03	646.69	Down
Scg2	17.4638	-3.2211	0.79446	-4.0544	5.03E-05	0.00317	34.4963	46.481	13.6379	4.0869	1.981	4.09946	Down
Wdfy1	3465.56	-0.6212	0.20071	-3.095	0.00197	0.05007	4334.71	3940.01	4326.15	2982.42	2018.64	3191.43	Down
Ccl20	130.181	2.43438	0.49825	4.88581	1.03E-06	0.00013	34.4963	51.4258	36.0431	281.996	296.16	80.9644	Up
Sphkap	50.1984	-1.3388	0.60875	-2.1992	0.02786	0.2884	42.3812	133.509	39.9397	24.5214	18.8195	42.0195	Down
Gpr55	24.9165	2.02286	0.92053	2.1975	0.02798	0.28884	19.7122	5.93375	3.89655	23.4997	85.1831	11.2735	Up
Gm18180	13.5396	-1.598	0.76487	-2.0892	0.03669	0.3402	21.6834	29.6687	9.74139	3.06518	9.90501	7.17406	Down
Gm16341	64.4066	-0.787	0.34649	-2.2715	0.02312	0.26041	57.1653	93.951	93.5173	41.8907	44.5725	55.3428	Down
B3gnt7	650.945	1.02901	0.49764	2.06776	0.03866	0.35259	572.639	378.771	333.155	550.71	1678.9	391.499	Up
Efhd1	313.737	1.05772	0.41743	2.53392	0.01128	0.16348	100.532	248.228	262.043	538.449	254.559	478.612	Up
Neu2	161.828	0.78368	0.34565	2.26726	0.02337	0.2617	159.669	84.0614	113	190.041	150.556	273.639	Up
Spp2	342.377	-1.0481	0.37961	-2.7611	0.00576	0.10589	395.229	492.501	496.811	201.28	115.889	352.554	Down
Asb18	26.0011	-4.4169	1.32463	-3.3345	0.00085	0.02818	19.7122	116.697	12.6638	0	6.93351	0	Down
Rab17	296.977	-0.8538	0.21091	-4.048	5.17E-05	0.00325	321.308	369.87	455.897	191.063	208.005	235.719	Down
Lrrfip1	8630.2	0.25268	0.09263	2.72779	0.00638	0.11295	7704.5	7686.18	8238.29	9259.9	9217.6	9674.73	Up
Ramp1	1090.16	0.52494	0.20933	2.50769	0.01215	0.17108	801.3	1008.74	871.854	1176.01	1064.79	1618.26	Up
Klhl30	112.828	-2.3972	0.58042	-4.1301	3.63E-05	0.00244	122.215	366.903	79.8794	24.5214	46.5535	36.8952	Down
Ndufa10	4315.17	-0.3991	0.2034	-1.962	0.04976	0.39986	4364.28	6335.27	4025.14	3640.41	3563.82	3962.13	Down
Gpc1	1284.83	-1.1487	0.29652	-3.874	0.00011	0.0057	1567.12	2548.54	1197.22	802.054	934.042	660.014	Down
Ankmy1	210.071	-1.2381	0.36023	-3.437	0.00059	0.02146	294.697	328.334	262.043	113.412	71.3161	190.625	Down
Gpr35	344.525	0.47857	0.23589	2.02881	0.04248	0.37204	280.898	284.82	298.086	337.169	526.946	339.231	Up
Kif1a	948.154	-0.6654	0.21984	-3.0267	0.00247	0.05873	1289.18	1178.84	1020.9	707.034	568.547	924.429	Down
D2hgdh	376.891	1.37562	0.16954	8.11362	4.91E-16	4.24E-13	241.474	187.902	199.698	518.015	554.68	559.577	Up
Gal3st2b	252.419	-0.8944	0.2627	-3.4046	0.00066	0.02334	299.625	337.235	347.767	159.389	132.727	237.769	Down
Gal3st2c	6.1017	-3.4671	1.52061	-2.2801	0.0226	0.25693	9.85609	22.746	0.97414	2.04345	0.9905	0	Down

Pdcd1	63.9419	1.93055	0.68585	2.81484	0.00488	0.09435	41.3956	24.724	13.6379	91.9553	183.243	28.6962	Up
Slco4c1	3111.18	0.25737	0.12534	2.05335	0.04004	0.36041	2725.21	2962.92	2815.26	3148.96	3764.89	3249.85	Up
Gin1	822.645	0.25055	0.12703	1.97244	0.04856	0.39474	708.653	815.89	729.63	874.597	878.574	928.528	Up
Pam	21748.1	-1.063	0.30058	-3.5366	0.00041	0.01618	21843.1	44609.9	21797.3	14061	16081.8	12095.5	Down
Cdh20	14.0096	-2.1859	0.80914	-2.7015	0.0069	0.11831	12.8129	39.5583	16.5604	2.04345	6.93351	6.1492	Down
Rnf152	180.202	-0.5751	0.23941	-2.4024	0.01629	0.20977	170.51	267.019	209.44	143.042	143.623	147.581	Down
Tnfrsf11a	374.578	0.58527	0.21383	2.73713	0.0062	0.11034	279.913	358.003	261.069	420.951	530.908	396.623	Up
A530053N	11.2114	-2.8665	0.92246	-3.1075	0.00189	0.04868	15.7697	34.6135	8.76725	4.0869	1.981	2.04973	Down
D830032E	8.42603	-2.6034	1.16852	-2.2279	0.02589	0.27824	10.8417	26.7019	5.84483	2.04345	0	5.12433	Down
Sctr	7.55986	-3.0067	1.17199	-2.5655	0.0103	0.15409	7.88487	22.746	9.74139	0	3.962	1.02487	Down
Dbi	3511.59	-0.4055	0.17537	-2.3121	0.02077	0.24509	3547.21	4735.13	3723.16	2757.64	2853.63	3452.77	Down
3110009E	352.725	-0.3727	0.14058	-2.6512	0.00802	0.13049	391.287	395.583	407.19	320.822	299.131	302.335	Down
Steap3	2421.36	-0.5759	0.17669	-3.2594	0.00112	0.03367	3382.61	2568.32	2744.15	2188.54	1975.06	1669.51	Down
Actr3	15872.5	0.26398	0.12964	2.0363	0.04172	0.36924	14141.5	14706.8	14425	17332.5	19346.5	15282.8	Up
Zranb3	305.488	0.78959	0.35593	2.21841	0.02653	0.28164	271.042	223.505	177.293	309.583	595.291	256.216	Up
Lct	6.72918	2.28281	0.98048	2.32826	0.0199	0.23828	3.94243	0.98896	1.94828	16.3476	7.92401	9.22379	Up
Mcm6	975.228	1.76964	0.21534	8.21778	2.07E-16	1.87E-13	554.898	359.981	412.061	1728.76	1481.79	1313.88	Up
Cxcr4	2232.03	0.70443	0.20694	3.404	0.00066	0.02336	1961.36	1554.64	1577.13	2905.79	3267.66	2125.57	Up
Rab7b	438.928	1.00163	0.36454	2.74769	0.006	0.10819	410.013	237.35	229.897	544.58	846.878	364.852	Up
6030442K	40.3273	0.97365	0.46177	2.10853	0.03498	0.3306	33.5107	25.7129	22.4052	37.8038	42.5915	79.9395	Up
Cntn2	29.1209	-3.3151	1.33658	-2.4803	0.01313	0.17964	3.94243	128.565	26.3017	0	13.867	2.04973	Down
Lrrn2	197.526	-1.067	0.31311	-3.4078	0.00065	0.02324	216.834	386.683	198.724	127.716	117.87	137.332	Down
Lax1	538.095	0.94331	0.35032	2.69275	0.00709	0.1204	396.215	339.213	369.199	703.969	1047.95	372.026	Up
Fmod	485.331	-0.8525	0.27806	-3.0657	0.00217	0.05353	855.508	563.706	454.923	361.691	391.248	284.913	Down
Btg2	17483.3	-0.7475	0.27075	-2.7607	0.00577	0.10589	15223.7	28772.7	21744.7	12563.1	10366.6	16228.8	Down
Chil1	37031.4	1.10168	0.32679	3.37125	0.00075	0.02537	32487.6	20394.3	17743	45246.1	72406.6	33910.8	Up
Lgr6	551.55	-2.1709	0.49007	-4.4299	9.43E-06	0.00081	555.883	1703.97	448.104	137.933	202.062	261.341	Down
Ptpn7	425.317	0.97923	0.3362	2.91268	0.00358	0.07594	302.582	264.052	292.242	575.231	807.258	310.534	Up
Tnnt2	6292.29	-4.0657	1.20715	-3.368	0.00076	0.02559	9068.59	19551.7	7006	95.0205	1798.75	233.669	Down
Inava	155.699	-0.7174	0.34884	-2.0564	0.03974	0.35945	118.273	253.173	209.44	91.9553	117.87	143.481	Down
9230116N	62.4016	-0.7514	0.2911	-2.5811	0.00985	0.15019	79.8343	78.1277	76.9569	38.8256	52.4965	48.1687	Down
Kif14	72.8277	0.64955	0.30779	2.11039	0.03482	0.32956	47.3092	72.1939	50.6552	81.738	101.031	84.039	Up
Gm33994	3.51491	3.28973	1.6217	2.02857	0.0425	0.37204	0	0	1.94828	7.15208	8.91451	3.0746	Up
Ptprc	6718.4	0.66161	0.25926	2.5519	0.01071	0.15782	5858.46	5242.47	4512.21	7721.18	11249.1	5726.95	Up
Aspm	135.256	1.32785	0.42783	3.10371	0.00191	0.04907	94.6184	80.1056	56.5	251.344	236.73	92.2379	Up
Rgs1	448.592	0.92178	0.40889	2.25434	0.02417	0.26772	451.409	341.191	137.354	646.752	685.427	429.419	Up
Ptgs2	1456.7	-0.8116	0.23333	-3.4783	0.0005	0.01912	1857.87	1840.45	1869.37	1043.18	748.819	1380.49	Down
Hmcn1	5692.48	-0.2879	0.1467	-1.9623	0.04972	0.39986	5683.02	6115.72	6976.78	5758.44	4830.67	4790.22	Down
Ivns1abp	10555.9	0.22158	0.09227	2.40126	0.01634	0.21027	9403.69	10048.8	9788.14	11448.4	11706.7	10939.4	Up
Arpc5	14348.3	0.27063	0.11175	2.42179	0.01544	0.20055	13803.5	12783.3	12432.9	15563.9	16809.8	14696.6	Up
Ncf2	1738.9	0.70676	0.35027	2.01778	0.04361	0.3745	1672.58	1313.34	978.035	1835.02	3309.26	1325.15	Up
Lamc2	7759.92	0.39796	0.20034	1.98638	0.04699	0.38829	8003.14	5389.82	6696.23	10245.9	8659.95	7564.53	Up
Shcbp1l	57.9874	1.20838	0.53413	2.26235	0.02368	0.26383	29.5683	36.5914	38.9655	54.1514	145.604	43.0444	Up
Rgs16	14.5762	1.60542	0.59554	2.69576	0.00702	0.1197	9.85609	5.93375	5.84483	23.4997	20.8005	21.5222	Up
A830008E	39.9322	0.84764	0.40146	2.11136	0.03474	0.32919	37.4531	26.7019	21.431	62.3252	45.563	46.119	Up
Gm9530	124.573	-0.5499	0.27301	-2.0142	0.04399	0.37695	113.345	166.145	164.629	83.7815	111.927	107.611	Down
BC034090	623.468	-0.516	0.17295	-2.9836	0.00285	0.06469	642.617	729.851	828.992	495.537	576.471	467.339	Down
Gm5532	146.675	-2.5559	0.57765	-4.4246	9.66E-06	0.00082	139.956	487.556	124.69	36.7821	63.3921	27.6714	Down
Tdrd5	138.012	1.27128	0.44871	2.83321	0.00461	0.09147	70.9638	78.1277	93.5173	147.128	333.799	104.536	Up
Tor3a	4553.73	0.25667	0.12344	2.07933	0.03759	0.34585	4291.34	4067.58	4090.41	4932.89	5464.59	4475.59	Up
Ralgps2	1552.39	-0.3374	0.10588	-3.1865	0.00144	0.04023	1775.08	1764.3	1659.93	1422.24	1374.82	1317.98	Down
Gm28694	180.145	-0.7278	0.369	-1.9725	0.04856	0.39474	191.208	223.505	259.121	152.237	70.3256	184.476	Down
Pappa2	147.854	0.8046	0.38397	2.09549	0.03613	0.33781	118.273	75.1608	129.56	225.801	232.768	105.561	Up
Fmo6	119.426	-1.3522	0.60912	-2.2198	0.02643	0.28101	80.8199	362.948	71.1121	67.4339	59.4301	74.8152	Down
Mroh9	55.0677	-0.8508	0.4139	-2.0556	0.03982	0.35955	84.7624	73.1829	54.5518	37.8038	24.7625	55.3428	Down
Prrx1	937.056	-0.5905	0.1586	-3.7232	0.0002	0.0093	1128.52	1288.61	961.475	755.055	722.075	766.6	Down
Gpr161	172.598	-0.5517	0.23326	-2.3651	0.01802	0.22383	221.762	216.582	177.293	113.412	144.613	161.929	Down
Cd247	472.124	0.47104	0.158	2.98134	0.00287	0.06496	398.186	382.727	406.216	625.296	515.06	505.259	Up
Gm26665	1.86642	4.36696	2.17618	2.00671	0.04478	NA	0	0	0	2.04345	1.981	7.17406	Up
Dusp27	56.429	-3.8288	0.63692	-6.0115	1.84E-09	6.02E-07	59.1365	195.814	61.3707	6.13035	7.92401	8.19893	Down
Mael	2.12075	-4.5102	2.16611	-2.0822	0.03733	NA	3.94243	0.98896	7.79311	0	0	0	Down
Fam78b	894.918	0.60242	0.23812	2.52984	0.01141	0.16459	554.898	663.591	913.742	1107.55	1239.12	890.608	Up
Rxrg	124.154	-3.0216	0.56215	-5.375	7.66E-08	1.50E-05	131.086	412.396	119.819	16.3476	38.6295	26.6465	Down
Nuf2	149.428	1.47504	0.38543	3.82697	0.00013	0.00662	100.532	87.0283	49.6811	262.583	262.483	134.257	Up
Rgs4	815.499	-0.4596	0.19456	-2.3624	0.01816	0.22436	1055.59	952.367	825.095	748.925	761.695	549.328	Down
Ccdc190	20.6804	-1.2086	0.57555	-2.1	0.03573	0.33519	18.7266	44.5031	23.3793	11.239	11.886	14.3481	Down
Sh2d1b1	395.812	1.18138	0.46229	2.5555	0.0106	0.15699	343.977	258.118	124.69	487.363	869.66	291.062	Up
Olfml2b	793.008	-0.4976	0.23927	-2.0798	0.03755	0.34561	815.098	1131.37	838.733	649.817	504.165	818.868	Down
Dusp12	79.3718	-0.6245	0.2605	-2.3975	0.01651	0.21132	97.5753	86.0394	105.207	64.3687	57.449	65.5914	Down
Fcgr2b	1796.02	1.22284	0.35896	3.40661	0.00066	0.02324	1147.25	1190.71	894.259	2154.82	4007.57	1381.52	Up
Fcgr4	741.029	1.81934	0.88509	2.05555	0.03983	0.35955	453.38	347.124	181.19	546.623	2538.65	379.2	Up
Fcgr3	2187.19	1.09208	0.41532	2.6295	0.00855	0.13608	1811.55	1492.34	886.466	2432.73	4922.79	1577.27	Up
Fcer1g	2153.27	0.98854	0.31457	3.14252	0.00167	0.04444	1525.72	1638.7	1165.07	2372.45	4317.59	1900.1	Up
Arhgap30	1839.13	0.71909	0.32547	2.20939	0.02715	0.28514	1587.82	1363.77	1218.65	2182.41	3380.58	1301.58	Up
Cd244a	378.048	0.71515	0.34546	2.07015	0.03844	0.35169	272.028	345.146	241.586	363.734	726.037	319.758	Up
Slamf7	433.599	1.37724	0.41008	3.3585	0.00078	0.02628	249.359	220.538	253.276	525.167	1050.92	302.335	Up
Cd48	1504.05	0.77148	0.284	2.71649	0.0066	0.11532	1269.46	1111.59	952.708	1879.97	2596.1	1214.47	Up
Slamf1	167.913	0.90903	0.3978	2.28516	0.0223	0.25497	84.7624	158.233	107.155	197.193	325.875	134.257	Up
Cd84	1763.1	1.08982	0.38708	2.81545	0.00487	0.09431	1422.23	1057.2	902.052	2034.26	3910.5	1252.39	Up
Slamf6	548.795	0.70895	0.29258	2.42311	0.01539	0.19995	499.704	394.594	355.561	844.967	771.6	426.344	Up
Slamf9	525.219	1.05055	0.25246	4.16118	3.17E-05	0.00221	441.553	331.301	253.276	671.274	837.964	615.944	Up
4933439K	209.052	-0.6804	0.30553	-2.2269	0.02596	0.27859	223.733	267.019	281.526	123.629	126.784	231.62	Down
Slamf8	225.761	1.41936	0.43113	3.2922	0.00099	0.03131	103.489	144.388	120.793	295.279	535.861	154.755	Up

Ackr1	51.7166	1.27988	0.57258	2.23528	0.0254	0.27517	40.41	28.6798	21.431	124.65	64.3826	30.746	Up
Ifi206	392.054	0.61063	0.24576	2.48469	0.01297	0.17832	357.776	353.058	220.155	492.472	521.994	406.872	Up
Ifi208	1200.38	1.53818	0.34172	4.50128	6.75E-06	0.00065	413.956	434.153	996.544	1912.67	1849.27	1595.72	Up
Spta1	75.0243	-1.6389	0.66499	-2.4646	0.01372	0.18549	77.8631	189.88	73.0604	10.2173	67.3541	31.7708	Down
Rgs7	47.8627	-1.9956	0.60276	-3.3107	0.00093	0.02986	43.3668	143.399	42.8621	14.3042	23.772	19.4725	Down
Efcab2	773.037	-0.7952	0.28718	-2.7692	0.00562	0.10469	788.487	1247.08	906.923	520.058	409.077	766.6	Down
Kif28	6.4285	-3.0793	1.40049	-2.1987	0.0279	0.28853	6.89926	19.7792	7.79311	0	0	4.09946	Down
Coq8a	1404.24	-1.564	0.3866	-4.0455	5.22E-05	0.00326	1607.53	3576.07	1112.47	653.904	748.819	726.63	Down
Gm37267	8.65595	1.93152	0.94871	2.03594	0.04176	0.36941	3.94243	1.97792	4.87069	5.10863	24.7625	11.2735	Up
Cenpf	164.554	1.5318	0.3577	4.28232	1.85E-05	0.00137	95.604	87.0283	71.1121	321.843	273.378	138.357	Up
Vash2	30.3259	-1.3084	0.52271	-2.5032	0.01231	0.17216	42.3812	40.5473	46.7587	9.19553	28.7245	14.3481	Down
Gm8407	5.58153	-3.9619	1.52572	-2.5967	0.00941	0.14527	1.97122	18.7902	10.7155	1.02173	0.9905	0	Down
Nsl1	87.0938	1.04925	0.37504	2.79773	0.00515	0.09792	79.8343	54.3927	36.0431	144.063	112.917	95.3125	Up
Dtl	116.406	1.29054	0.36434	3.5421	0.0004	0.01596	100.532	55.3817	46.7587	202.302	137.68	155.78	Up
Lpgat1	6571.98	-0.3636	0.11812	-3.078	0.00208	0.05192	7398.96	7984.85	6803.38	5672.62	5486.38	6085.65	Down
1700034H	147.238	-1.0184	0.25672	-3.9669	7.28E-05	0.00417	159.669	204.714	226.974	100.129	80.2306	111.71	Down
Gm32460	6.91443	2.89462	1.14033	2.5384	0.01114	0.16205	3.94243	0	0.97414	16.3476	7.92401	12.2984	Up
Traf5	1033.89	0.44584	0.16717	2.66701	0.00765	0.12664	1043.76	812.924	769.569	1139.22	1249.02	1188.84	Up
Rcor3	888.569	-0.2541	0.11363	-2.2365	0.02532	0.27488	926.472	963.245	1010.18	793.881	830.04	807.594	Down
Olah	46.6532	-1.361	0.61087	-2.2279	0.02589	0.27824	98.5609	38.5694	64.2931	24.5214	9.90501	44.0692	Down
Cdnf	138.594	-0.9691	0.3536	-2.7407	0.00613	0.10967	168.539	263.063	118.845	105.238	100.041	75.8401	Down
Frmd4a	4625.84	0.37813	0.12937	2.9228	0.00347	0.07476	4193.77	3807.49	4068	5574.53	5496.29	4614.97	Up
Phyh	4498.44	-0.6977	0.25649	-2.7201	0.00653	0.11449	4465.79	7878.04	4352.45	3400.3	3136.92	3757.16	Down
Mcm10	107.57	0.63024	0.30966	2.03529	0.04182	0.36968	79.8343	71.205	102.285	152.237	146.594	93.2628	Up
Ccdc3	210.088	-0.8736	0.37216	-2.3474	0.0189	0.23012	232.604	414.373	168.526	113.412	160.461	171.153	Down
Gm13391	52.4731	1.46889	0.62802	2.33893	0.01934	0.234	29.5683	29.6687	24.3535	43.9342	154.518	32.7957	Up
Gm38386	3.01523	5.06094	1.84447	2.74384	0.00607	0.10892	0	0	0	4.0869	9.90501	4.09946	Up
Gata3	365.514	0.63379	0.24081	2.63194	0.00849	0.13561	273.014	331.301	255.224	526.189	479.402	327.957	Up
Itih5	635.205	-0.3342	0.16089	-2.0773	0.03777	0.34709	737.235	783.255	604.94	585.449	571.519	528.831	Down
Prkcq	1043.89	0.51395	0.17592	2.92146	0.00348	0.07481	802.286	951.378	826.069	1407.94	1255.96	1019.74	Up
Il2ra	168.965	0.70611	0.26	2.71583	0.00661	0.11532	128.129	130.542	126.638	241.127	237.72	149.63	Up
E030013I1	295.685	-0.6427	0.16358	-3.9288	8.54E-05	0.00475	337.078	394.594	349.716	236.019	217.91	238.794	Down
Vim	44675.6	0.34791	0.16426	2.11803	0.03417	0.32567	41604.5	35795.3	40544.6	55964	53195.8	40949.5	Up
Mrc1	3878.41	0.86454	0.41772	2.06968	0.03848	0.35169	4010.44	2788.86	1450.49	4933.91	7325.74	2760.99	Up
Slc39a12	44.4596	0.9636	0.43077	2.23695	0.02529	0.27483	30.5539	32.6356	27.2759	56.1949	83.2021	36.8952	Up
Pip4k2a	2319.15	0.4547	0.19614	2.31818	0.02044	0.24239	2223.53	1849.35	1797.29	2664.66	3232.99	2147.09	Up
Gm13375	263.454	-0.5606	0.18645	-3.0066	0.00264	0.06128	290.755	361.959	289.319	199.236	212.958	226.495	Down
Il1rn	497.282	1.41471	0.3794	3.72881	0.00019	0.00913	392.272	250.206	171.448	773.446	961.776	434.543	Up
Tor4a	2514.23	0.34311	0.12654	2.71142	0.0067	0.11621	2160.45	2063.96	2425.61	2679.99	3020.04	2735.37	Up
Grin1	38.6983	-1.0677	0.39348	-2.7136	0.00666	0.11573	40.41	55.3817	61.3707	29.63	20.8005	24.5968	Down
Entpd2	188.791	-0.5493	0.24625	-2.2308	0.02569	0.27716	197.122	287.787	188.009	155.302	143.623	160.904	Down
Fut7	45.7934	0.98371	0.47807	2.05769	0.03962	0.35889	40.41	16.8123	35.069	58.2383	82.2116	42.0195	Up
Ptgds	38.1563	-2.2297	1.03803	-2.148	0.03171	0.31185	16.7553	165.156	6.81897	10.2173	21.791	8.19893	Down
A230005N	2.45837	-4.7227	1.91977	-2.4601	0.01389	0.18691	2.95683	6.92271	4.87069	0	0	0	Down
Slc2a6	236.138	0.69166	0.34114	2.02747	0.04261	0.37265	196.136	188.891	156.836	271.779	426.906	176.277	Up
Gm10134	367.904	0.46953	0.16033	2.92851	0.00341	0.07409	310.467	296.687	318.543	461.82	442.754	377.151	Up
Gfi1b	20.6883	1.50053	0.54786	2.73889	0.00616	0.11002	11.8273	9.88958	10.7155	28.6083	42.5915	20.4973	Up
Miga2	1520.28	-0.3595	0.16622	-2.163	0.03054	0.30531	1443.92	2018.46	1663.83	1315.98	1285.67	1393.82	Down
Gm14488	24.4855	-1.1958	0.52005	-2.2994	0.02148	0.24997	23.6546	43.5142	35.069	13.2824	10.8955	20.4973	Down
Usp20	1498.31	-0.3248	0.1422	-2.2841	0.02237	0.25524	1662.72	1721.78	1614.15	1314.96	1171.76	1504.5	Down
Hmcn2	271.649	-0.6941	0.32208	-2.1551	0.03116	0.30876	265.129	441.075	301.009	200.258	142.632	279.788	Down
Ass1	1711.15	0.32097	0.14576	2.20207	0.02766	0.28707	1355.21	1602.11	1607.33	1824.8	1762.1	2115.32	Up
Plpp7	245.446	-0.8775	0.35925	-2.4425	0.01458	0.19345	238.517	455.91	259.121	169.606	116.879	232.645	Down
Lcn2	11568.1	1.56267	0.36743	4.25293	2.11E-05	0.00153	8668.43	5106.98	3778.68	16348.6	24624.8	10881	Up
Slc25a25	2061.54	-0.306	0.13121	-2.3321	0.0197	0.23657	2234.37	2342.84	2260.98	1676.65	2072.13	1782.24	Down
Ak1	1745.24	-1.0877	0.36481	-2.9815	0.00287	0.06496	2024.44	3426.74	1669.67	1014.57	699.294	1636.71	Down
Eng	32649.9	0.37755	0.18436	2.04785	0.04058	0.36253	25350.8	26657.4	33197.7	34871.5	43907.9	31914.3	Up
Lmx1b	43.1105	-3.0237	0.53781	-5.6222	1.89E-08	4.57E-06	85.748	93.951	50.6552	14.3042	9.90501	4.09946	Down
C130021I2	122.377	-2.8558	0.4302	-6.6384	3.17E-11	1.34E-08	189.237	325.367	130.535	21.4562	28.7245	38.9449	Down
C230014C	77.9873	0.75624	0.29354	2.57629	0.00999	0.15156	56.1797	66.2602	51.6293	83.7815	118.86	91.2131	Up
Phf19	226.019	0.69196	0.30433	2.2737	0.02298	0.25959	244.431	153.289	120.793	322.865	258.521	256.216	Up
Traf1	4648.67	0.28093	0.09924	2.83073	0.00464	0.09178	4025.23	4141.76	4425.51	5049.37	5274.42	4975.72	Up
Hc	22673.3	0.60276	0.12783	4.71521	2.41E-06	0.00027	16559.2	20077.8	17376.7	26018.2	29131.6	26876.1	Up
Glo1-ps	36.2982	-2.1796	1.10537	-1.9718	0.04863	0.39488	154.741	19.7792	3.89655	4.0869	16.8385	18.4476	Down
Lhx6	301.325	1.04459	0.25222	4.14166	3.45E-05	0.00236	190.222	173.068	226.974	514.95	393.229	309.509	Up
Ptgs1	4777.01	0.50059	0.24034	2.08285	0.03727	0.34397	4307.11	4106.15	3456.24	5630.73	7236.6	3925.24	Up
Nek6	1804.06	0.48691	0.19101	2.54918	0.0108	0.15841	1565.15	1465.64	1476.79	1885.08	2653.55	1778.14	Up
Kynu	308.194	1.47565	0.57234	2.5783	0.00993	0.15089	270.057	120.653	98.388	378.038	821.125	160.904	Up
Gm13479	5.25937	-2.396	1.12509	-2.1296	0.03321	0.32161	9.85609	7.91166	8.76725	0	2.9715	2.04973	Down
Lypd6b	540.81	-0.3756	0.15495	-2.4241	0.01535	0.19982	564.754	603.264	664.362	523.123	449.687	439.667	Down
Nmi	1724.05	0.24557	0.09926	2.47392	0.01336	0.18196	1572.05	1546.73	1614.15	1895.3	1803.7	1912.4	Up
Gm14033	5.58808	-5.9071	1.67226	-3.5324	0.00041	0.01635	10.8417	18.7902	3.89655	0	0	0	Down
Cytip	5636.78	0.52434	0.22728	2.30702	0.02105	0.24709	5446.47	4551.19	3873.17	6436.87	8386.57	5126.38	Up
Ly75	1485.55	0.73866	0.32073	2.30307	0.02127	0.24897	1444.9	1075	820.225	1555.07	2700.11	1317.98	Up
Ifih1	2453.04	0.33273	0.15236	2.18378	0.02898	0.29496	2354.62	2159.88	1999.91	2704.51	3104.23	2395.11	Up
Slc38a11	118.713	-2.8167	0.65667	-4.2894	1.79E-05	0.00134	72.935	422.285	128.586	21.4562	47.544	19.4725	Down
Scn7a	19375.2	-0.4261	0.16523	-2.5788	0.00992	0.15089	20555.9	21301.2	24789.9	18400.3	13711.5	17492.4	Down
Xirp2	283.085	-4.2761	1.88096	-2.2734	0.023	0.25968	314.409	1059.17	241.586	0	79.2401	4.09946	Down
Stk39	1200.65	-0.6953	0.28984	-2.399	0.01644	0.21099	1246.8	2144.06	1062.79	864.38	1089.55	796.321	Down
Spc25	359.193	0.53956	0.22501	2.39798	0.01649	0.21116	258.229	296.687	323.414	537.428	370.447	368.952	Up
Lrp2	3609.72	0.56699	0.28514	1.9885	0.04676	0.38731	3977.92	2564.37	2185.97	4152.29	5528.98	3248.82	Up
Klhl41	329.715	-2.5636	0.58865	-4.355	1.33E-05	0.00105	271.042	1094.78	326.336	68.4556	165.414	52.2682	Down
Klhl23	885.647	-0.3823	0.15996	-2.3899	0.01686	0.21411	875.221	1128.4	1003.36	791.837	818.154	696.909	Down

Sp5	168.441	0.86184	0.24194	3.56215	0.00037	0.01496	144.884	97.9068	115.922	191.063	215.929	244.943 Up
Metap1d	855.669	-0.4167	0.12851	-3.2424	0.00119	0.03511	940.271	960.278	1034.54	792.859	710.189	695.884 Down
Platr26	8.71177	-5.5574	1.4846	-3.7434	0.00018	0.00879	12.8129	29.6687	8.76725	1.02173	0	0 Down
Ttn	4969.14	-3.8747	1.05022	-3.6894	0.00022	0.01026	5428.73	18412.4	4070.93	274.844	1434.25	193.7 Down
Gm37004	33.7552	1.96738	0.67188	2.92816	0.00341	0.07409	19.7122	6.92271	14.6121	71.5208	71.3161	18.4476 Up
Frzb	24.9243	-2.8535	1.0966	-2.6021	0.00927	0.14353	9.85609	113.73	7.79311	2.04345	7.92401	8.19893 Down
Gm4735	611.005	-2.687	0.21425	-12.541	4.44E-36	8.71E-33	1304.95	840.614	1027.72	155.302	169.376	168.078 Down
Gm13710	296.179	1.29179	0.27776	4.65077	3.31E-06	0.00035	129.115	230.427	155.862	425.038	492.279	344.355 Up
Prg2	57.0657	2.01545	0.65474	3.07824	0.00208	0.05192	30.5539	23.735	13.6379	63.347	177.3	33.8206 Up
Gm13712	7.71308	-3.3815	1.02379	-3.3029	0.00096	0.03049	15.7697	9.88958	16.5604	2.04345	0.9905	1.02487 Down
Olfr1033	4634.29	-0.7323	0.35888	-2.0405	0.0413	0.36628	3501.87	7187.75	6667.98	3245	5039.67	2163.49 Down
Ptpmt1	494.452	-0.2655	0.12986	-2.0442	0.04093	0.3642	547.013	511.291	561.104	443.429	444.735	459.14 Down
Rapsn	81.0579	-0.8921	0.37728	-2.3646	0.01805	0.22385	97.5753	147.355	71.1121	50.0645	49.525	70.7157 Down
Spi1	1575.38	0.8913	0.36844	2.41909	0.01556	0.20168	1520.79	1025.55	764.699	1831.95	3080.46	1228.81 Up
Mybpc3	1318.09	-4.4532	1.32051	-3.3723	0.00075	0.02531	1112.75	5511.46	939.07	26.5649	297.15	21.5222 Down
Pacsin3	1323.98	-0.4643	0.21502	-2.1594	0.03082	0.30715	1507	1808.8	1289.76	1090.18	897.394	1350.77 Down
Gm13780	15.7089	-1.6894	0.71786	-2.3534	0.0186	0.22746	17.741	41.5362	12.6638	9.19553	5.94301	7.17406 Down
Lrrc4c	131.633	-0.764	0.242	-3.1569	0.00159	0.04292	154.741	154.277	188.009	116.477	88.1546	88.1385 Down
Gm10800	170.588	2.90861	1.08508	2.68055	0.00735	0.1232	35.4819	69.2271	15.5862	712.143	75.2781	115.81 Up
Cd44	9006.82	0.66258	0.23412	2.83008	0.00465	0.09178	7598.06	7102.7	6221.82	11719.2	13767	7632.18 Up
Gm13881	11.2144	-2.3281	0.81005	-2.874	0.00405	0.08338	17.741	23.735	14.6121	2.04345	1.981	7.17406 Down
Cd59a	893.381	-0.8036	0.33026	-2.4332	0.01497	0.19658	1239.9	1327.18	840.682	824.532	365.495	762.5 Down
Depdc7	117.568	0.62022	0.24105	2.57294	0.01008	0.15228	87.7192	86.0394	104.233	165.52	131.737	130.158 Up
Gm13886	33.7811	-0.8634	0.39014	-2.2131	0.02689	0.28339	38.4387	52.4148	39.9397	26.5649	22.7815	22.547 Down
Hadhb-ps	1682.09	-0.6552	0.25613	-2.5582	0.01052	0.1567	1758.33	2851.17	1563.49	1127.98	1422.36	1369.22 Down
Kif18a	290.447	0.51802	0.18376	2.81905	0.00482	0.09354	241.474	246.251	228.923	360.669	377.381	287.987 Up
Lgr4	460.18	-0.5531	0.23547	-2.3489	0.01883	0.22961	521.387	680.403	440.311	349.43	446.716	322.833 Down
Aven	803.042	0.27957	0.14056	1.989	0.0467	0.38699	707.667	691.282	777.363	965.53	805.277	871.136 Up
Scg5	63.2963	-1.124	0.29311	-3.8349	0.00013	0.00644	84.7624	79.1166	96.4397	43.9342	38.6295	36.8952 Down
Arhgap11	330.427	0.98431	0.31862	3.08927	0.00201	0.05092	237.532	230.427	197.75	519.036	547.747	250.067 Up
A530058N	18.9132	-4.7608	1.23058	-3.8687	0.00011	0.0058	6.89926	83.0725	19.4828	2.04345	1.981	0 Down
Rasgrp1	679.274	0.46143	0.22699	2.03285	0.04207	0.37095	623.89	576.563	514.345	803.076	975.643	582.124 Up
Eif2ak4	2033.97	0.40443	0.12935	3.12671	0.00177	0.0465	1880.54	1629.8	1741.76	2530.81	2167.22	2253.68 Up
Bmf	2018.34	0.67624	0.13109	5.15864	2.49E-07	4.06E-05	1689.33	1429.04	1543.04	2340.77	2691.19	2416.63 Up
Bub1b	293.339	0.80882	0.34408	2.35069	0.01874	0.22882	264.143	228.449	147.095	343.3	525.956	251.092 Up
Pak6	1266.13	1.38744	0.24258	5.71941	1.07E-08	2.78E-06	675.142	678.425	747.164	2453.16	1327.27	1715.63 Up
Knstrn	212.743	1.588	0.27125	5.85439	4.79E-09	1.34E-06	127.144	101.863	89.6207	405.625	301.112	251.092 Up
Ivd	6565.96	-0.4236	0.16329	-2.5945	0.00947	0.14581	6920.94	8440.76	7207.65	4906.33	5410.12	6509.95 Down
Knl1	84.2226	1.48944	0.37201	4.00373	6.24E-05	0.00372	52.2373	41.5362	38.9655	149.172	147.585	75.8401 Up
Rad51	147.532	1.18297	0.38896	3.04136	0.00236	0.05665	113.345	98.8958	58.4483	290.17	193.148	131.183 Up
Gchfr	170.394	1.24247	0.42516	2.92235	0.00347	0.0748	96.5897	103.841	103.259	368.843	107.965	241.868 Up
Dnajc17	474.437	-0.3383	0.16489	-2.0519	0.04018	0.36086	524.344	544.916	520.19	359.647	420.963	476.563 Down
Gm14207	181.262	0.97733	0.31674	3.08556	0.00203	0.05138	106.446	116.697	143.198	293.235	277.34	150.655 Up
Dll4	3397.55	0.62315	0.16471	3.78324	0.00015	0.00761	2389.12	2433.83	3201.99	4204.4	4339.38	3816.6 Up
Chac1	214.086	0.91678	0.2356	3.89126	9.97E-05	0.00537	131.086	129.554	184.112	240.105	292.198	307.46 Up
Exd1	300.888	1.06807	0.24013	4.44785	8.67E-06	0.00075	170.51	220.538	191.905	505.754	317.951	398.673 Up
Nusap1	376.623	0.77236	0.28775	2.68412	0.00727	0.1224	321.308	246.251	266.914	603.84	517.041	304.385 Up
Gm28042	7.91284	-2.0773	0.90426	-2.2972	0.0216	0.25016	16.7553	13.8454	7.79311	1.02173	3.962	4.09946 Down
Pla2g4e	34.2074	-2.9202	1.04028	-2.8071	0.005	0.09613	34.4963	113.73	33.1207	4.0869	19.81	0 Down
Pla2g4f	531.039	1.23892	0.2096	5.91098	3.40E-09	1.05E-06	378.474	318.445	251.328	848.032	672.55	717.406 Up
Lrrc57	1543.63	-0.216	0.09891	-2.1843	0.02894	0.29481	1701.16	1612.99	1662.85	1400.79	1447.12	1436.86 Down
Haus2	1304.78	-0.3431	0.13133	-2.6127	0.00898	0.14076	1463.63	1465.64	1448.54	1060.55	1290.62	1099.68 Down
AV039307	30.432	1.32693	0.44208	3.00155	0.00269	0.06203	19.7122	10.8785	21.431	40.869	43.582	46.119 Up
Map1a	578.991	-0.3868	0.16618	-2.3278	0.01992	0.23828	739.207	583.485	645.854	460.798	553.69	490.911 Down
Pdia3	33354.7	0.23262	0.09363	2.48452	0.01297	0.17832	30922.5	30145.4	30946.4	37282.8	36908	33923.1 Up
Hypk	37.7357	0.80215	0.37225	2.15491	0.03117	0.30876	27.597	22.746	32.1466	53.1297	41.601	49.1936 Up
Mfap1b	646.781	-0.423	0.15113	-2.799	0.00513	0.0977	726.394	793.144	703.328	484.298	589.348	584.174 Down
Frmd5	927.269	-1.6548	0.7443	-2.2233	0.02619	0.28002	477.035	2861.06	884.518	452.624	359.552	528.831 Down
Casc4	4120.41	-0.8171	0.25959	-3.1474	0.00165	0.04397	5456.33	4905.23	5409.39	1861.58	3429.11	3660.82 Down
Eif3j1	2113.49	-0.4884	0.19536	-2.5	0.01242	0.17268	2481.76	2311.2	2610.69	1327.22	2031.52	1918.55 Down
Gatm	666.655	2.80789	0.38447	7.30331	2.81E-13	1.60E-10	202.05	177.023	120.793	1514.2	1427.31	558.552 Up
Bloc1s6	1895.74	0.39358	0.12985	3.03104	0.00244	0.05803	1618.37	1723.75	1574.21	2239.62	2300.93	1917.52 Up
Slc24a5	346.519	-0.6801	0.21784	-3.1222	0.00179	0.04698	366.646	374.815	538.699	258.497	285.264	255.192 Down
Fam227b	85.6282	0.59763	0.30475	1.96108	0.04987	0.39986	71.9494	61.3154	71.1121	132.824	80.2306	96.3374 Up
Atp8b4	463.644	0.82718	0.28324	2.92047	0.0035	0.07481	313.424	372.837	316.595	484.298	847.869	446.842 Up
Blvra	1332.26	0.29044	0.13457	2.15822	0.03091	0.30734	1284.25	1176.86	1134.87	1412.02	1620.46	1365.12 Up
Ncaph	573.938	0.29652	0.14651	2.02382	0.04299	0.37324	487.876	492.501	565	674.339	584.396	639.516 Up
Dusp2	394.014	0.7456	0.31062	2.4004	0.01638	0.21052	269.071	423.274	190.931	550.71	516.051	414.046 Up
Gpat2	50.3124	-2.0777	0.51826	-4.009	6.10E-05	0.00367	43.3668	129.554	71.1121	19.4128	14.8575	23.5719 Down
Mall	623.073	-0.5631	0.25349	-2.2214	0.02632	0.2807	652.473	897.974	678.975	391.321	455.63	662.063 Down
Bub1	225.82	0.57815	0.19655	2.94152	0.00327	0.07204	163.611	194.825	185.086	300.387	277.34	233.669 Up
Morrbid	579.031	0.42918	0.2052	2.09154	0.03648	0.33955	474.078	538.982	467.587	785.707	693.351	514.483 Up
Vinac1	64.0422	-0.7461	0.29368	-2.5406	0.01107	0.16147	71.9494	85.0504	83.7759	41.8907	55.468	46.119 Down
Polr1b	1175.12	-0.3027	0.14163	-2.1376	0.03255	0.31668	1252.71	1252.02	1389.12	934.879	1061.82	1160.15 Down
Ckap2l	160.343	0.98555	0.34469	2.85926	0.00425	0.08595	106.446	141.421	75.0087	270.757	221.872	146.556 Up
Sirpa	5737.86	0.77708	0.27487	2.82707	0.0047	0.09231	5130.09	3982.53	3574.11	7001.88	9838.64	4899.88 Up
Tgm3	25.5742	1.58601	0.53533	2.96268	0.00305	0.06868	18.7266	9.88958	9.74139	42.9125	46.5535	25.6216 Up
Cpxm1	725.558	-0.4691	0.22398	-2.0945	0.03622	0.3383	738.221	962.256	827.044	738.707	627.978	459.14 Down
Pced1a	1144.26	0.40044	0.16291	2.45797	0.01397	0.18776	991.522	917.753	1050.12	1493.76	1303.5	1108.9 Up
Siglec1	451.165	1.20451	0.58335	2.0648	0.03894	0.35477	401.143	270.975	147.095	542.536	1163.84	181.401 Up
Cdc25b	2078.61	0.33645	0.1206	2.78978	0.00527	0.09983	1906.17	1731.67	1874.24	2331.58	2489.13	2138.9 Up
Gm14051	275.075	-1.4654	0.50129	-2.9233	0.00346	0.07476	320.323	646.779	244.509	119.542	72.3066	246.993 Down
Chgb	40.2148	-1.2899	0.42493	-3.0355	0.0024	0.0575	52.2373	73.1829	45.7845	26.5649	15.848	27.6714 Down

Tmx4	2991.11	-0.4424	0.16935	-2.6122	0.00899	0.14086	3307.7	4122.97	2907.8	2379.6	2577.28	2651.33	Down
Pak7	9.57146	-1.9043	0.88759	-2.1455	0.03192	0.31285	17.741	21.7571	5.84483	2.04345	5.94301	4.09946	Down
Snap25	39.5178	-2.369	0.5987	-3.9569	7.59E-05	0.0043	44.3524	90.9841	63.319	4.0869	13.867	20.4973	Down
Snx5	7400.69	0.27337	0.13678	1.99855	0.04566	0.38232	7254.08	6769.42	6081.55	7976.61	8937.29	7385.18	Up
Slc24a3	1451.99	-0.6139	0.21022	-2.9204	0.0035	0.07481	1438	2260.76	1570.31	1122.88	1071.72	1248.29	Down
Ralgapa2	4590.32	0.40473	0.12166	3.3268	0.00088	0.02874	4290.35	3861.88	3699.78	5360.99	5485.39	4843.52	Up
Nkx2-2	3.61263	-5.2778	1.79177	-2.9456	0.00322	0.07154	10.8417	7.91166	2.92242	0	0	0	Down
Zfp120	948.81	0.26126	0.13231	1.97453	0.04832	0.39416	808.199	948.411	832.888	1028.88	1088.56	985.921	Up
Cst7	130.336	0.90614	0.34813	2.60289	0.00924	0.14331	105.46	72.1939	94.4914	161.433	236.73	111.71	Up
Pygb	6112.73	-0.5426	0.2373	-2.2865	0.02222	0.2547	6333.52	9568.17	5844.83	4419.98	4522.63	5987.27	Down
Gins1	151.928	0.5772	0.26133	2.20867	0.0272	0.28553	149.813	112.741	103.259	217.628	172.347	155.78	Up
Rspo4	66.5238	-1.5127	0.45111	-3.3532	0.0008	0.02658	122.215	57.3596	115.922	32.6952	22.7815	48.1687	Down
Angpt4	80.5727	-1.4129	0.45881	-3.0795	0.00207	0.05192	74.9063	184.935	91.569	57.2166	29.715	45.0941	Down
Rem1	351.885	-0.5918	0.21691	-2.7283	0.00637	0.11287	497.732	319.433	452	277.909	279.321	284.913	Down
Tpx2	275.131	1.56825	0.3222	4.86725	1.13E-06	0.00014	153.755	167.134	95.4656	486.341	474.45	273.639	Up
Foxs1	123.049	-1.0682	0.40401	-2.6441	0.00819	0.13205	115.316	182.957	201.647	41.8907	97.0691	99.412	Down
Hck	2786.7	0.47393	0.17876	2.65125	0.00802	0.13049	2760.69	2150.98	2087.58	3292	3634.15	2794.81	Up
Bpifa5	5.41895	-5.8629	1.93348	-3.0323	0.00243	0.05797	0.98561	23.735	7.79311	0	0	0	Down
Bpifb1	8172.79	-5.1742	1.04209	-4.9652	6.86E-07	9.82E-05	5650.49	34874.6	7190.12	452.624	744.857	124.009	Down
Bpifb5	344.758	-0.628	0.25366	-2.4756	0.0133	0.18144	341.021	524.148	390.63	297.322	208.996	306.435	Down
Myh7b	24.6776	-1.3484	0.65696	-2.0525	0.04012	0.36086	22.669	53.4037	30.1983	17.3693	4.9525	19.4725	Down
Cnbd2	1399.91	2.15323	0.39964	5.38788	7.13E-08	1.44E-05	481.963	512.28	547.466	2852.66	3103.24	901.882	Up
Myl9	5164.47	-0.8378	0.35467	-2.3623	0.01816	0.22436	3594.52	9133.03	7142.38	3018.18	3006.17	5092.56	Down
Gm14276	47.2229	0.7369	0.35911	2.05202	0.04017	0.36086	36.4675	43.5142	26.3017	65.3904	60.4206	51.2433	Up
Rbl1	1197.39	0.39078	0.14776	2.64469	0.00818	0.13205	1170.9	991.925	945.889	1373.2	1452.07	1250.34	Up
Adig	121.718	-1.4633	0.46534	-3.1447	0.00166	0.04428	111.374	315.478	109.104	72.5425	63.3921	58.4174	Down
Fam83d	85.7811	1.14287	0.40528	2.81994	0.0048	0.09345	50.266	74.1719	36.0431	130.781	147.585	75.8401	Up
Ptprt	121.097	-0.9697	0.37878	-2.56	0.01047	0.1561	112.359	218.56	150.017	67.4339	62.4016	115.81	Down
Gtsf1l	56.3143	-2.7876	0.83719	-3.3297	0.00087	0.02858	41.3956	224.493	29.2242	10.2173	6.93351	25.6216	Down
Jph2	1327.21	-1.7705	0.37342	-4.7413	2.12E-06	0.00025	1277.35	3476.19	1404.71	531.297	625.997	647.715	Down
Slpi	2101.45	0.90173	0.45005	2.0036	0.04511	0.38075	2560.61	976.102	859.19	3916.27	2679.3	1617.24	Up
Rbpjl	3210.88	0.27054	0.12341	2.19221	0.02836	0.29075	2621.72	3031.16	3079.25	3569.91	3653.96	3309.29	Up
Wfdc13	256.574	-1.2289	0.56453	-2.1769	0.02949	0.29871	100.532	684.359	294.19	179.824	155.509	125.034	Down
Ube2c	306.884	1.26837	0.32214	3.93729	8.24E-05	0.00461	174.453	223.505	142.224	587.492	433.839	279.788	Up
Cdh22	142.104	-1.0054	0.36922	-2.7229	0.00647	0.11397	151.784	287.787	129.56	78.6729	93.1071	111.71	Down
Zfp663	108.205	-0.9829	0.35924	-2.736	0.00622	0.11063	194.165	142.41	94.4914	72.5425	56.4585	89.1633	Down
Ocstamp	14.9548	1.83131	0.91198	2.00806	0.04464	0.37936	3.94243	11.8675	3.89655	11.239	48.5345	10.2487	Up
Fam210b	2185.01	-0.2832	0.09572	-2.9588	0.00309	0.06932	2388.13	2395.26	2412.94	1945.37	1917.61	2050.76	Down
Aurka	230.501	0.5744	0.21351	2.69027	0.00714	0.12092	204.021	195.814	155.862	302.431	291.207	233.669	Up
Ctcflos	13.5075	-4.2526	1.26578	-3.3596	0.00078	0.02626	14.7841	57.3596	4.87069	0	1.981	2.04973	Down
Pck1	639.813	-5.906	1.35491	-4.359	1.31E-05	0.00103	362.704	3175.54	237.69	4.0869	46.5535	12.2984	Down
Zbp1	811.699	1.21248	0.4635	2.61594	0.0089	0.14016	541.099	563.706	363.354	901.162	2032.51	468.364	Up
Ctsz	9079.13	0.54811	0.226	2.4252	0.0153	0.1994	9043.95	6903.92	6177.01	10237.7	13448	8664.22	Up
Zfp972	431.003	-0.5513	0.16444	-3.3524	0.0008	0.02661	563.768	531.07	442.259	364.756	337.761	346.405	Down
Tcfl5	8.59011	-1.8522	0.87261	-2.1226	0.03378	0.32409	16.7553	13.8454	9.74139	2.04345	1.981	7.17406	Down
Eef1a2	974.71	-4.2278	1.85593	-2.278	0.02273	0.25741	1066.43	3486.08	999.466	2.04345	292.198	2.04973	Down
Zfp512b	304.271	-0.4588	0.20636	-2.2231	0.02621	0.28004	345.949	307.566	403.293	224.78	300.122	243.918	Down
Pkia	2706.96	-0.5231	0.20357	-2.5697	0.01018	0.15318	2569.48	4079.45	2928.26	2208.97	2083.02	2372.56	Down
Hey1	5517.36	0.40436	0.11201	3.61009	0.00031	0.01306	4393.84	4888.42	4965.18	6275.44	5965.79	6615.51	Up
Fabp5	5151.94	0.55824	0.21796	2.56125	0.01043	0.15565	4270.64	4508.66	3723.16	4788.83	7978.48	5641.89	Up
Fabp4	2762.89	-2.4228	0.96888	-2.5006	0.0124	0.17252	2667.06	9410.93	1893.73	518.015	1773	314.634	Down
Ralyl	59.107	-1.4638	0.66968	-2.1858	0.02883	0.29409	52.2373	166.145	41.888	15.3259	25.753	53.293	Down
Car3	4486.77	-6.6375	0.99243	-6.6881	2.26E-11	9.97E-09	5953.08	16604.6	4095.28	32.6952	199.091	35.8703	Down
A930001A	434.486	0.98893	0.40611	2.43514	0.01489	0.19589	198.107	205.703	469.535	560.927	378.371	794.271	Up
Sirpb1a	200.653	1.10127	0.40956	2.6889	0.00717	0.12123	157.697	136.476	88.6466	257.475	415.02	148.606	Up
Sirpb1b	272.448	0.84793	0.4154	2.04123	0.04123	0.36578	263.158	212.626	108.129	293.235	526.946	230.595	Up
Dnajc5b	23.5106	-1.4048	0.66188	-2.1225	0.0338	0.32409	23.6546	51.4258	27.2759	10.2173	5.94301	22.547	Down
Trim55	230.577	-2.8597	0.64578	-4.4283	9.50E-06	0.00081	227.676	746.663	241.586	17.3693	43.582	106.586	Down
Cpa3	430.566	-0.9891	0.44977	-2.1992	0.02787	0.2884	207.963	847.537	662.414	255.431	258.521	351.529	Down
Nlgn1	241.013	-4.0959	1.17258	-3.4931	0.00048	0.01831	25.6258	724.906	615.656	13.2824	29.715	36.8952	Down
Ect2	331.323	0.85746	0.22884	3.74701	0.00018	0.00868	239.503	252.184	215.285	546.623	368.466	365.877	Up
Pld1	1901.92	0.35352	0.16045	2.20334	0.02757	0.28707	1720.87	1741.56	1547.91	2101.69	2475.26	1824.26	Up
Usp13	470.892	-0.9507	0.29504	-3.2221	0.00127	0.03687	649.516	826.769	385.759	320.822	357.571	284.913	Down
Ccna2	298.708	0.94441	0.35535	2.65768	0.00787	0.12853	230.632	222.516	159.759	503.711	465.535	210.098	Up
Rpl31-ps1	11.0548	-2.1642	1.06872	-2.025	0.04287	0.37291	34.4963	17.8012	1.94828	3.06518	5.94301	3.0746	Down
Gm36823	4.27105	-2.8953	1.21592	-2.3812	0.01726	0.21705	6.89926	6.92271	8.76725	1.02173	0.9905	1.02487	Down
Platr4	7.90368	-2.7591	1.36679	-2.0187	0.04352	0.37421	1.97122	24.724	14.6121	0	0.9905	5.12433	Down
Pcdh18	2050.08	-0.5094	0.14521	-3.508	0.00045	0.01757	2130.89	2346.8	2747.07	1757.37	1599.66	1718.7	Down
Gm6209	19.9833	1.81212	0.74767	2.42368	0.01536	0.19982	10.8417	11.8675	3.89655	19.4128	56.4585	17.4227	Up
Ndufc1	789.311	-0.4811	0.23262	-2.0682	0.03862	0.35249	826.926	1066.1	866.009	620.187	510.108	846.539	Down
Gm10727	24.8967	-1.6491	0.82617	-1.9961	0.04592	0.38348	43.3668	52.4148	17.5345	2.04345	23.772	10.2487	Down
Gm6394	9.86592	-2.1104	0.94399	-2.2356	0.02538	0.27515	20.6978	5.93375	21.431	1.02173	3.962	6.1492	Down
Sucnr1	12.0069	-4.5001	1.26304	-3.5629	0.00037	0.01496	12.8129	42.5252	13.6379	3.06518	0	0	Down
Rap2b	505.056	0.42223	0.19151	2.20472	0.02747	0.28689	492.804	430.197	372.121	586.47	653.731	495.01	Up
Veph1	96.6317	0.89669	0.26318	3.40712	0.00066	0.02324	67.0214	80.1056	55.5259	117.498	137.68	121.959	Up
Shox2	40.1275	-4.86	1.00409	-4.8402	1.30E-06	0.00016	55.1941	151.311	26.3017	0	6.93351	1.02487	Down
Smc4	3606.23	0.3213	0.12077	2.66038	0.00781	0.12796	3322.49	3036.1	3260.44	4002.1	4349.29	3666.97	Up
B3galnt1	669.846	-0.419	0.21237	-1.9728	0.04852	0.39474	850.58	736.774	712.095	529.254	724.056	466.314	Down
Bche	732.001	-0.6023	0.25902	-2.3255	0.02005	0.23903	674.156	1180.82	792.949	670.252	597.272	476.563	Down
Etfdh	2991.68	-0.4891	0.24302	-2.0127	0.04415	0.37732	3033.7	4820.18	2628.23	2482.79	2621.86	2363.34	Down
Rxfp1	165.184	-3.8907	1.50101	-2.5921	0.00954	0.14663	133.057	689.304	106.181	3.06518	57.449	2.04973	Down
Gucy1b1	7855.67	-0.2728	0.12275	-2.2225	0.02625	0.28036	7818.83	8970.84	8999.09	7451.44	7333.67	6560.17	Down
Rbm46	182.058	-1.2497	0.25385	-4.923	8.52E-07	0.00012	279.913	201.747	287.371	111.368	123.813	88.1385	Down

Rbm46os	211.161	-0.7219	0.2853	-2.5302	0.0114	0.16459	262.172	232.405	294.19	201.28	171.357	105.561	Down
Sfrp2	125.293	1.3978	0.67701	2.06466	0.03895	0.35477	98.5609	76.1498	32.1466	69.4773	387.286	88.1385	Up
Rps3a1	40910.1	1.06822	0.18797	5.68294	1.32E-08	3.40E-06	27014.5	24497.5	27749.3	54685.8	43752.4	67761.1	Up
Cd1d2	88.6717	-2.7716	0.37783	-7.3355	2.21E-13	1.29E-10	205.007	121.642	137.354	28.6083	15.848	23.5719	Down
Gm10705	124.734	0.53574	0.21905	2.4458	0.01445	0.19242	95.604	96.9179	113	144.063	160.461	138.357	Up
Fcrl5	123.967	1.39134	0.49781	2.79492	0.00519	0.0986	68.9926	61.3154	75.0087	173.693	297.15	67.6411	Up
Sh2d2a	303.12	0.88721	0.3572	2.48383	0.013	0.17855	200.079	183.946	254.25	405.625	560.623	214.197	Up
Iqgap3	180.048	1.50465	0.40656	3.7009	0.00021	0.00987	120.244	101.863	59.4225	390.299	249.606	158.854	Up
Glmp	4567.67	0.21163	0.10216	2.0716	0.0383	0.35064	4516.06	4099.23	4084.56	4848.09	5054.53	4803.55	Up
Gba	2681.88	0.29306	0.13387	2.18917	0.02858	0.29242	2569.48	2385.37	2276.56	2952.79	3256.77	2650.3	Up
Muc1	16940.9	0.32422	0.15833	2.04779	0.04058	0.36253	15729.3	15952.9	13453.8	16039	21376	19094.3	Up
Cks1b	467.027	0.49159	0.21661	2.26944	0.02324	0.26127	369.603	463.821	331.207	637.557	451.668	548.303	Up
Kcnn3	603.437	1.16692	0.42619	2.73805	0.00618	0.11021	530.257	324.378	261.069	823.511	1281.71	399.698	Up
Tpm3	15292.7	1.77613	0.11662	15.2298	2.24E-52	6.06E-49	7460.07	6757.55	6517.96	24510.2	24581.3	21929.1	Up
Npr1	4774.01	0.41331	0.15877	2.60324	0.00923	0.14326	3866.54	3666.07	4751.85	5180.15	6078.7	5100.76	Up
Gm43595	488.154	0.47002	0.23187	2.02708	0.04265	0.37277	413.956	297.676	516.293	598.731	545.766	556.502	Up
S100a4	158.623	0.57976	0.26379	2.19784	0.02796	0.28884	97.5753	161.2	122.741	215.584	176.309	178.327	Up
Lor	35.8487	-1.4814	0.4648	-3.1871	0.00144	0.04019	68.9926	48.4589	40.9138	26.5649	16.8385	13.3233	Down
S100a10	6908.07	0.42148	0.16555	2.54596	0.0109	0.15966	6203.42	5162.36	6352.36	8858.36	6673	8198.93	Up
Celf3	78.5815	-0.6918	0.30634	-2.2584	0.02392	0.2656	107.431	107.796	75.9828	72.5425	55.468	52.2682	Down
Tmod4	154.066	-0.8067	0.25444	-3.1704	0.00152	0.04188	165.582	245.262	177.293	126.694	104.003	105.561	Down
Tnfaip8l2	477.2	0.83886	0.26343	3.1843	0.00145	0.04043	386.359	357.014	283.474	569.101	812.211	455.04	Up
Gm4349	6.835	2.07983	1.04536	1.98959	0.04664	0.38675	1.97122	1.97792	3.89655	12.2607	17.829	3.0746	Up
Ctsk	2833.14	1.05335	0.39567	2.66219	0.00776	0.12767	2297.45	1940.34	1289.76	2803.61	6429.34	2238.31	Up
Ctss	9932.47	1.5862	0.41567	3.81598	0.00014	0.00686	7325.04	4481.96	3082.17	15311.6	22039.6	7354.44	Up
Gm20633	81.8158	-1.1054	0.25339	-4.3625	1.29E-05	0.00102	114.331	110.763	110.078	48.0211	56.4585	51.2433	Down
Gm20627	179.569	-1.3847	0.22696	-6.1013	1.05E-09	3.55E-07	239.503	255.151	284.448	78.6729	108.955	110.686	Down
Gm42743	127.755	-9.4587	1.21016	-7.816	5.45E-15	4.06E-12	301.596	209.659	254.25	1.02173	0	0	Down
BC107364	4.56189	5.65694	1.60389	3.52701	0.00042	0.01654	0	0	0	8.1738	7.92401	11.2735	Up
Gm10685	4.77695	5.72305	2.28357	2.50618	0.0122	0.17151	0	0	0	11.239	0	17.4227	Up
Hjv	342.222	-4.1642	1.37669	-3.0248	0.00249	0.05898	347.92	1258.94	338.026	2.04345	94.0976	12.2984	Down
Itga10	271.072	0.81648	0.20603	3.96293	7.40E-05	0.00422	179.381	230.427	179.241	396.429	312.998	327.957	Up
Ankrd35	68.7787	0.716	0.30458	2.35079	0.01873	0.22882	49.2804	48.4589	58.4483	83.7815	103.012	69.6909	Up
Rnf115	3414.98	0.23486	0.09935	2.36387	0.01809	0.2242	3207.17	3159.72	3046.13	3697.62	3863.94	3515.29	Up
Prkab2	759.873	-0.3711	0.18509	-2.005	0.04496	0.38034	824.955	896.985	849.449	595.666	815.182	576.999	Down
Pde4dip	4025.47	-0.9083	0.34517	-2.6313	0.0085	0.13575	3654.64	8372.52	3729.98	2420.47	3537.08	2438.16	Down
Phgdh	722.641	0.68762	0.31217	2.20274	0.02761	0.28707	660.358	595.353	405.242	807.163	1257.94	609.795	Up
Gm22341	2.03618	4.49249	2.06097	2.1798	0.02927	NA	0	0	0	4.0869	1.981	6.1492	Up
Casq2	1222.14	-4.0091	1.31389	-3.0513	0.00228	0.05522	1210.33	4611.51	1082.27	16.3476	372.428	39.9698	Down
Gm16160	36.5418	-1.5031	0.73243	-2.0523	0.04014	0.36086	40.41	54.3927	67.2156	18.3911	2.9715	35.8703	Down
Gm43241	5.60679	-2.4867	1.16791	-2.1292	0.03324	0.32163	14.7841	6.92271	6.81897	3.06518	0	2.04973	Down
A230001M	35.9794	-1.9738	0.65433	-3.0164	0.00256	0.06003	35.4819	75.1608	61.3707	11.239	4.9525	27.6714	Down
Ngf	25.9092	-1.9762	0.59636	-3.3139	0.00092	0.02966	44.3524	43.5142	36.0431	6.13035	5.94301	19.4725	Down
Sycp1	40.1784	1.75461	0.61692	2.84413	0.00445	0.08938	14.7841	25.7129	14.6121	114.433	35.658	35.8703	Up
Ptpn22	842.715	1.02385	0.30791	3.32515	0.00088	0.02887	637.689	494.479	534.802	1133.09	1580.84	675.387	Up
Gm5546	27.9538	-1.2899	0.48419	-2.6641	0.00772	0.1274	47.3092	39.5583	32.1466	23.4997	11.886	13.3233	Down
Kcnd3	165.493	-0.6948	0.29923	-2.322	0.02023	0.24073	164.597	264.052	185.086	108.303	107.965	162.954	Down
Gm5547	98.5715	2.58265	0.36662	7.04444	1.86E-12	8.94E-10	29.5683	35.6025	19.4828	181.867	206.024	118.884	Up
Chia1	6330.8	1.87129	0.25123	7.44862	9.43E-14	5.82E-11	3078.06	1839.46	3236.09	11173.6	8066.64	10591	Up
Chil3	36728	3.5821	1.11871	3.20199	0.00136	0.03893	11609.5	4075.5	1297.55	50903.4	139441	13041.4	Up
Chil4	32.8654	3.62478	1.29158	2.80646	0.00501	0.09615	10.8417	3.95583	0	33.7169	145.604	3.0746	Up
Gm6522	46.9232	3.2611	1.12648	2.89496	0.00379	0.07951	24.6402	0.98896	0.97414	49.0428	180.271	25.6216	Up
Cd53	3490.48	0.83172	0.28376	2.93104	0.00338	0.07369	2946.97	2460.53	2126.54	4615.13	5985.6	2808.13	Up
Gm27008	47.8829	1.33407	0.46681	2.85784	0.00427	0.08616	35.4819	23.735	22.4052	76.6294	91.1261	37.92	Up
Kcna2	672.778	-0.7828	0.13847	-5.653	1.58E-08	3.96E-06	785.53	864.349	903.026	500.645	523.975	459.14	Down
Csf1	2522.87	0.51927	0.2317	2.24113	0.02502	0.27341	2214.66	2007.58	1998.93	2756.62	3958.04	2201.41	Up
Gstm5	482.479	-0.6356	0.22218	-2.8607	0.00423	0.08572	528.286	687.326	545.518	345.343	316.96	471.438	Down
Gstm7	400.612	-0.7394	0.26985	-2.74	0.00614	0.10974	389.315	685.348	428.621	253.388	315.97	331.032	Down
Gm12494	15.2917	-3.0094	0.84264	-3.5713	0.00036	0.01465	27.597	28.6798	25.3276	0	2.9715	7.17406	Down
Gm12499	2.12872	-4.5152	1.97781	-2.2829	0.02244	NA	2.95683	4.94479	4.87069	0	0	0	Down
Sypl2	45.1611	-2.4679	0.51702	-4.7733	1.81E-06	0.00022	59.1365	122.631	47.7328	12.2607	14.8575	14.3481	Down
Mybphl	1298	-4.2672	1.9147	-2.2287	0.02584	0.27824	1374.92	4723.26	1305.35	2.04345	380.352	2.04973	Down
Psrc1	148.385	0.72519	0.35395	2.04887	0.04048	0.36238	154.741	107.796	73.0604	236.019	178.29	140.407	Up
Fam102b	2642.32	1.04347	0.19476	5.35765	8.43E-08	1.63E-05	1946.58	1648.59	1583.95	3632.23	4233.4	2809.16	Up
Amy1	912.152	-0.7414	0.25357	-2.9239	0.00346	0.07473	903.803	1398.39	1122.21	707.034	504.165	837.315	Down
S1pr1	21867.2	0.3324	0.1541	2.15697	0.03101	0.30787	18015.9	18993.9	21067.7	24065.7	28021.3	21038.4	Up
Extl2	992.686	-0.3302	0.15581	-2.1195	0.03405	0.32522	1145.28	1221.36	950.759	880.727	817.163	940.827	Down
Gpr88	95.0589	-0.8707	0.26281	-3.3131	0.00092	0.02969	107.431	141.421	119.819	66.4121	59.4301	75.8401	Down
Lrrc39	219.085	-1.56	0.48228	-3.2347	0.00122	0.03573	183.323	617.11	181.19	110.346	113.908	108.636	Down
Slc35a3	111.157	-0.5652	0.27708	-2.0397	0.04138	0.36683	165.582	102.852	129.56	85.8249	98.0596	85.0639	Down
Agl	1244.2	-0.503	0.24863	-2.0232	0.04305	0.37324	1349.3	1948.25	1079.35	905.249	1179.69	1003.34	Down
Frrs1	480.627	1.26214	0.24355	5.18231	2.19E-07	3.76E-05	296.668	299.654	252.302	677.404	862.726	495.01	Up
Palmd	4870.36	-0.5848	0.11255	-5.196	2.04E-07	3.55E-05	5858.46	6101.87	5572.07	3760.97	3710.42	4218.35	Down
4930455H	18.0763	-3.2766	0.70974	-4.6166	3.90E-06	0.0004	40.41	26.7019	31.1724	2.04345	1.981	6.1492	Down
1700061I1	10.8269	-5.8768	1.36733	-4.298	1.72E-05	0.0013	27.597	19.7792	16.5604	0	0	1.02487	Down
Plppr5	15.8378	-1.7894	0.63684	-2.8098	0.00496	0.09559	16.7553	27.6908	29.2242	9.19553	3.962	8.19893	Down
Dpyd	513.643	-0.368	0.14511	-2.536	0.01121	0.1626	589.394	615.132	531.88	422.994	440.773	481.687	Down
Myoz2	1729.41	-3.7771	1.55434	-2.43	0.0151	0.19735	1994.87	5722.11	1954.12	10.2173	670.569	24.5968	Down
Synpo2	1594.25	-0.9187	0.29462	-3.1182	0.00182	0.04752	1440.96	3116.21	1698.9	1048.29	1055.87	1205.24	Down
Prss12	598.767	0.60178	0.19541	3.07953	0.00207	0.05192	459.294	488.545	479.276	603.84	893.432	668.213	Up
Ank2	227.648	-0.9761	0.45451	-2.1476	0.03175	0.31185	206.978	531.07	167.552	158.367	189.186	112.735	Down
Alpk1	882.206	-1.0035	0.19139	-5.2434	1.58E-07	2.81E-05	955.055	1311.36	1265.41	529.254	671.56	560.602	Down
Fam241a	1488.76	0.38573	0.13406	2.87735	0.00401	0.08279	1274.39	1315.31	1282.94	1625.57	1535.28	1899.08	Up

Pitx2	40.7385	-4.8863	1.00077	-4.8826	1.05E-06	0.00013	86.7336	113.73	36.0431	0	7.92401	0	Down
Elovl6	547.225	-0.8886	0.37025	-2.3999	0.0164	0.21065	475.063	1159.06	497.785	311.626	459.592	380.225	Down
Egf	115.45	-1.6918	0.457	-3.7019	0.00021	0.00987	113.345	304.599	111.052	52.108	62.4016	49.1936	Down
6330410L	11.4977	-5.0618	1.61387	-3.1364	0.00171	0.04532	1.97122	49.4479	15.5862	0	1.981	0	Down
Cyp2u1	212.223	-0.6443	0.29948	-2.1514	0.03144	0.31047	212.891	361.959	201.647	172.672	138.67	185.501	Down
Sgms2	991.903	-0.4871	0.23314	-2.0891	0.0367	0.3402	955.055	1364.76	1153.38	758.12	668.588	1051.51	Down
Gm29865	20.7351	-1.9687	0.6045	-3.2567	0.00113	0.0339	27.597	24.724	46.7587	5.10863	7.92401	12.2984	Down
Dkk2	59.0999	-1.665	0.46623	-3.5711	0.00036	0.01465	49.2804	140.432	79.8794	27.5866	28.7245	28.6962	Down
Cenpe	130.002	1.20519	0.29791	4.0455	5.22E-05	0.00326	72.935	91.9731	71.1121	187.997	225.834	130.158	Up
Manba	2031.44	0.48967	0.15474	3.16448	0.00155	0.04258	1935.74	1715.84	1418.35	2419.45	2326.69	2372.56	Up
Slc39a8	13456.4	0.40054	0.16954	2.36249	0.01815	0.22436	14008.5	10046.8	10745.7	15406.6	16366	14164.7	Up
Gm43357	1.85392	4.35832	2.05246	2.12347	0.03371	NA	0	0	0	4.0869	3.962	3.0746	Up
Lamtor3	3618.48	0.24047	0.10131	2.37355	0.01762	0.22001	3502.85	3202.25	3247.78	3995.97	3996.67	3765.36	Up
Dapp1	1146.84	0.87734	0.24219	3.62254	0.00029	0.01265	1093.04	707.105	625.397	1371.16	1668.99	1415.34	Up
1110002E	81.62	-3.563	0.65814	-5.4138	6.17E-08	1.26E-05	115.316	272.952	63.319	7.15208	22.7815	8.19893	Down
Adh6b	58.7656	-1.5373	0.49429	-3.1101	0.00187	0.04831	44.3524	105.819	112.026	41.8907	19.81	28.6962	Down
Rap1gds1	3794.17	0.34092	0.16664	2.04586	0.04077	0.36329	3650.69	3183.46	3209.79	4697.89	4562.25	3460.97	Up
Unc5c	752.42	-0.3336	0.16467	-2.026	0.04276	0.37291	876.206	799.078	841.656	726.447	552.699	718.431	Down
Gbp5	1257.59	1.35182	0.44315	3.05048	0.00228	0.05531	882.12	585.463	656.569	1458	3184.46	778.898	Up
Gbp7	3385.64	0.74363	0.32291	2.30293	0.02128	0.24897	2535.97	2545.58	2514.25	3693.54	6541.27	2483.25	Up
Gbp3	2152.7	0.94635	0.31587	2.99602	0.00274	0.0628	1541.49	1600.13	1271.25	2495.05	4269.06	1739.2	Up
Gbp2b	36.7087	0.90449	0.40357	2.24125	0.02501	0.27341	28.5827	26.7019	21.431	56.1949	52.4965	34.8454	Up
Gbp2	5404.28	0.98372	0.29871	3.29318	0.00099	0.03127	3909.91	3479.15	3501.05	6387.83	10656.8	4490.96	Up
Gm42705	9.2793	2.39929	0.95911	2.50158	0.01236	0.17237	1.97122	5.93375	0.97414	23.4997	7.92401	15.373	Up
Ddah1	2040.76	0.4498	0.15863	2.8355	0.00458	0.09104	1590.77	1757.38	1827.48	2406.16	2669.4	1993.36	Up
Bcl10	2872.16	0.25263	0.10298	2.45321	0.01416	0.18932	2786.32	2567.34	2510.36	3195.96	3125.03	3047.95	Up
Adgrl4	17501.5	0.4661	0.19705	2.3654	0.01801	0.22379	16812.5	12923.7	14359.8	20321.1	24406.9	16184.7	Up
Ifi44l	86.6534	-6.6671	0.80725	-8.2591	1.47E-16	1.38E-13	96.5897	282.842	135.405	2.04345	0.9905	2.04973	Down
Ptgfr	1070.45	-0.7463	0.2864	-2.6058	0.00917	0.14264	1252.71	1876.05	895.233	806.141	918.194	674.362	Down
Gm43618	60.2858	-0.9839	0.42645	-2.3072	0.02104	0.24709	68.007	118.675	53.5776	50.0645	39.62	31.7708	Down
Dnajb4	6265.58	-0.2421	0.12202	-1.984	0.04726	0.38984	6254.67	7344.99	6770.26	5807.49	5343.75	6072.33	Down
Nexn	2422.24	-1.1119	0.29909	-3.7176	0.0002	0.00944	2328.01	5016	2592.18	1407.94	1684.84	1504.5	Down
Miga1	2265.91	-0.2759	0.09939	-2.776	0.0055	0.10317	2377.29	2467.45	2600.95	2075.12	2045.38	2029.23	Down
Zzz3	3307.02	0.25209	0.12413	2.03088	0.04227	0.37149	3374.72	2769.08	2912.67	3590.34	3582.64	3612.65	Up
Ak5	90.5944	-0.9115	0.34046	-2.6773	0.00742	0.12358	148.827	105.819	100.336	46.9994	84.1926	57.3925	Down
Acadm	6396.8	-0.7221	0.23946	-3.0154	0.00257	0.06011	6386.74	10927	6581.28	4634.55	4484	5367.22	Down
Cryz	809.907	-0.4382	0.12147	-3.6073	0.00031	0.0131	925.487	976.102	894.259	725.425	658.683	679.486	Down
Tnni3k	198.409	-2.9552	0.52779	-5.5992	2.15E-08	5.00E-06	220.776	644.801	188.983	30.6518	68.3446	36.8952	Down
4930570G	37.5964	1.91853	0.42069	4.56045	5.10E-06	0.00052	19.7122	12.8565	14.6121	73.5642	50.5155	54.3179	Up
Negr1	298.527	0.87885	0.29397	2.98956	0.00279	0.06378	238.517	255.151	137.354	371.908	469.497	318.733	Up
Gm20752	39.8952	3.47996	0.77336	4.49978	6.80E-06	0.00065	11.8273	5.93375	1.94828	58.2383	132.727	28.6962	Up
Depdc1a	54.5863	1.58807	0.48313	3.287	0.00101	0.03169	27.597	40.5473	13.6379	103.194	86.1736	56.3676	Up
Penk	176.989	-1.9255	0.28392	-6.7818	1.19E-11	5.34E-09	212.891	357.014	270.811	66.4121	87.1641	67.6411	Down
4930430E	81.0716	2.24464	0.71671	3.13185	0.00174	0.04597	41.3956	35.6025	7.79311	84.8032	261.492	55.3428	Up
Tox	144.239	1.13692	0.29282	3.8827	0.0001	0.00554	107.431	92.9621	70.138	254.41	170.366	170.128	Up
Car8	7894.52	0.3438	0.13287	2.58755	0.00967	0.14804	7603.97	7100.72	6170.19	8488.49	8509.39	9494.36	Up
Asph	4232.77	-0.5209	0.19788	-2.6321	0.00848	0.13561	4114.92	6113.74	4737.24	3306.3	3115.13	4009.28	Down
Gm11814	11.3283	4.02375	1.04317	3.85723	0.00011	0.006	0	2.96687	0.97414	29.63	12.8765	21.5222	Up
Gm12918	439.489	0.36969	0.1825	2.02574	0.04279	0.37291	373.546	425.252	351.664	432.19	481.383	572.9	Up
Pdp1	1678.81	-0.3511	0.11111	-3.1603	0.00158	0.04279	1799.72	1995.72	1850.86	1448.81	1432.26	1545.5	Down
Fut9	48.0531	-2.645	0.86453	-3.0595	0.00222	0.05418	27.597	182.957	37.9914	8.1738	4.9525	26.6465	Down
Rragd	1362.4	-0.5415	0.26406	-2.0506	0.04031	0.36148	1673.56	2012.53	1159.22	913.422	1002.39	1413.29	Down
Cnr1	140.549	-0.7484	0.37451	-1.9984	0.04567	0.38232	109.403	222.516	196.776	102.173	139.661	72.7655	Down
Tmem215	22.6759	-1.0364	0.47701	-2.1728	0.0298	0.30054	36.4675	28.6798	26.3017	13.2824	12.8765	18.4476	Down
Aqp7	37.719	-4.0218	0.90425	-4.4477	8.68E-06	0.00075	28.5827	164.167	20.4569	3.06518	5.94301	4.09946	Down
Aqp3	114.156	-1.821	0.74915	-2.4308	0.01506	0.19718	52.2373	418.329	63.319	43.9342	73.2971	33.8206	Down
Myorg	1009.09	-0.2725	0.11856	-2.2986	0.02153	0.25004	1054.6	1158.07	1099.8	886.858	968.71	886.509	Down
Dnaic1	2102.92	-0.754	0.38107	-1.9786	0.04786	0.3921	1800.71	3148.84	2971.12	1367.07	857.774	2471.98	Down
Cntfr	513.959	-0.75	0.2834	-2.6465	0.00813	0.13167	558.84	882.151	492.914	468.972	373.419	307.46	Down
Atp8b5	172.405	-1.1626	0.47512	-2.4469	0.01441	0.19196	159.669	392.616	162.681	136.911	55.468	127.083	Down
Cd72	464.14	0.79633	0.26194	3.04019	0.00236	0.05679	411.984	307.566	298.086	715.208	635.902	416.096	Up
Arhgef39	212.811	1.15121	0.2285	5.03809	4.70E-07	7.15E-05	148.827	144.388	103.259	324.909	295.169	260.316	Up
Tpm2	3017.71	-0.555	0.24296	-2.2841	0.02237	0.25524	2645.37	4386.03	3741.67	2488.92	1928.51	2915.74	Down
Tmem8b	809.089	-0.2888	0.12847	-2.2482	0.02457	0.27053	898.875	812.924	957.578	737.686	728.018	719.456	Down
Olfr157	2.02473	4.48527	1.99077	2.25304	0.02426	NA	0	0	0	4.0869	3.962	4.09946	Up
Gm12411	1182.58	0.27515	0.12171	2.26067	0.02378	0.2647	1104.87	1064.12	1041.35	1322.11	1186.62	1376.39	Up
Aldh1b1	425.469	-2.9152	0.57956	-5.0299	4.91E-07	7.41E-05	559.826	1317.29	376.992	68.4556	187.205	43.0444	Down
Igfbpl1	17.4158	-3.2289	0.81244	-3.9743	7.06E-05	0.00409	39.4243	32.6356	22.4052	4.0869	5.94301	0	Down
Stra6l	180.59	2.35651	1.08782	2.16627	0.03029	0.30382	90.676	41.5362	44.8104	109.325	742.876	54.3179	Up
Trim14	1551.11	0.45725	0.18259	2.50418	0.01227	0.17177	1278.33	1296.52	1347.23	1942.3	2046.37	1395.87	Up
Anks6	287.648	-0.3838	0.18319	-2.0949	0.03618	0.33807	317.366	346.135	313.673	220.693	289.226	238.794	Down
Tgfbr1	3431.72	0.43348	0.20078	2.15898	0.03085	0.30729	3152.96	2666.23	2940.92	3885.62	4879.21	3065.37	Up
Erp44	5437.51	0.24789	0.09134	2.71404	0.00665	0.11573	5186.27	4910.18	4818.09	5962.79	5946.97	5800.74	Up
Cavin4	74.0102	-1.6739	0.6315	-2.6507	0.00803	0.13053	52.2373	226.471	59.4225	21.4562	46.5535	37.92	Down
Acnat1	23.3846	-2.9309	0.95688	-3.063	0.00219	0.0539	6.89926	80.1056	37.0173	3.06518	2.9715	10.2487	Down
Grin3a	128.162	-0.5198	0.26033	-1.9967	0.04585	0.38322	116.302	170.101	166.578	97.0639	100.041	118.884	Down
Smc2os	9.08075	2.01806	1.01935	1.97976	0.04773	0.39169	0	3.95583	6.81897	16.3476	8.91451	18.4476	Up
Smc2	695.664	0.41992	0.20601	2.03838	0.04151	0.36785	640.646	533.048	611.759	869.488	910.27	608.77	Up
Abca1	4377.01	0.36184	0.12453	2.90558	0.00367	0.07734	3941.45	3763.97	3787.45	5500.97	4746.48	4521.71	Up
4930522C	25.661	1.17378	0.57944	2.0257	0.0428	0.37291	13.7985	24.724	8.76725	46.9994	22.7815	36.8952	Up
Rps15a-ps	103.685	-8.2593	1.08885	-7.5853	3.32E-14	2.39E-11	273.999	213.615	132.483	0	0.9905	1.02487	Down
Gm12505	4.60699	-2.555	1.29206	-1.9775	0.04799	0.39249	8.87048	10.8785	3.89655	0	2.9715	1.02487	Down
Orm1	54.8132	-1.4803	0.43579	-3.3969	0.00068	0.02381	90.676	98.8958	52.6035	19.4128	39.62	27.6714	Down

Orm3	72.4135	-0.5673	0.26039	-2.1787	0.02935	0.29757	82.7911	85.0504	91.569	55.1732	59.4301	60.4671 Down
Tmem268	1237.59	0.45711	0.18941	2.4134	0.0158	0.204	1139.36	1044.34	945.889	1330.29	1752.2	1213.44 Up
Tnfsf8	205.736	1.40229	0.32496	4.31522	1.59E-05	0.00121	91.6616	113.73	133.457	316.735	390.257	188.575 Up
Tnc	513.601	0.62036	0.30551	2.03055	0.0423	0.37149	340.035	432.175	442.259	883.792	399.172	584.174 Up
Tle1	4066.49	0.39152	0.15783	2.48069	0.01311	0.17956	3642.81	3507.83	3403.64	5162.78	4896.05	3785.85 Up
C630043F	465.131	0.41829	0.1794	2.33165	0.01972	0.23657	441.553	387.672	365.302	610.992	521.003	464.264 Up
Frmd3	21.6589	-1.3615	0.59055	-2.3054	0.02114	0.24787	26.6114	47.47	19.4828	8.1738	13.867	14.3481 Down
Gm5860	25.0595	-1.6448	0.60315	-2.7271	0.00639	0.11296	24.6402	42.5252	46.7587	5.10863	12.8765	18.4476 Down
Gm12551	12.2708	4.14806	0.93155	4.45284	8.47E-06	0.00075	1.97122	0.98896	0.97414	20.4345	27.734	21.5222 Up
Dennd4c	2035.04	0.40538	0.18932	2.14121	0.03226	0.31492	2031.34	1709.91	1511.86	2350.99	2676.33	1929.82 Up
Slc24a2	102.87	-1.806	0.49651	-3.6373	0.00028	0.01204	135.028	268.997	75.9828	34.7387	59.4301	43.0444 Down
Mrpl48-ps	258.035	-0.8681	0.36817	-2.3577	0.01839	0.22573	245.417	372.837	381.862	260.54	101.031	186.526 Down
Klhl9	4637.31	0.43624	0.11167	3.90644	9.37E-05	0.00511	3791.64	4235.71	3797.19	5601.1	5347.71	5050.54 Up
Gm13274	21.9339	2.11787	0.72232	2.93203	0.00337	0.07357	6.89926	15.8233	1.94828	28.6083	46.5535	31.7708 Up
Gm13284	6.07255	4.13056	1.36871	3.01784	0.00255	0.05989	1.97122	0	0	12.2607	9.90501	12.2984 Up
Cdkn2a	77.6221	0.89261	0.2771	3.22129	0.00128	0.03689	54.2085	44.5031	64.2931	99.1074	98.0596	105.561 Up
Dmrta1	19.4626	1.91024	0.58673	3.25573	0.00113	0.03397	8.87048	3.95583	11.6897	35.7604	25.753	30.746 Up
Gm12666	20.2945	-5.8946	1.31739	-4.4744	7.66E-06	0.0007	10.8417	54.3927	54.5518	0	1.981	0 Down
Tusc1	264.021	-0.8622	0.2307	-3.7371	0.00019	0.00897	396.215	277.897	347.767	164.498	174.328	223.421 Down
Eqtn	29.4242	1.25142	0.59755	2.09426	0.03624	0.33832	13.7985	30.6577	7.79311	48.0211	46.5535	29.7211 Up
Jun	14892.4	0.27672	0.12606	2.19516	0.02815	0.28977	12616.8	14782	13006.7	17533.8	15100.2	16314.8 Up
Junos	717.985	1.17471	0.35569	3.30262	0.00096	0.03049	307.51	711.061	303.931	847.01	930.08	1208.32 Up
Gm830	101.964	1.83275	0.53005	3.4577	0.00054	0.02037	38.4387	80.1056	15.5862	192.084	128.765	156.804 Up
Fggy	480.367	1.2558	0.41918	2.99587	0.00274	0.0628	483.934	229.438	137.354	678.426	554.68	798.371 Up
Cachd1	1645.42	0.35077	0.12527	2.80006	0.00511	0.09747	1536.56	1296.52	1506.02	1892.24	1865.11	1776.09 Up
Raver2	3385.4	-0.3561	0.13871	-2.5673	0.01025	0.15358	3690.12	3509.81	4203.41	3153.04	3091.35	2664.65 Down
B020004J(	5.65547	2.56523	1.20537	2.12816	0.03332	0.32227	1.97122	0.98896	1.94828	3.06518	19.81	6.1492 Up
Insl5	59.8408	-2.1988	0.56872	-3.8663	0.00011	0.00585	87.7192	98.8958	108.129	30.6518	4.9525	28.6962 Down
Plpp3	13865.6	-0.3036	0.14924	-2.0341	0.04194	0.37014	13691.1	15597.8	16668.5	12006.3	14062.1	11168 Down
Pcsk9	13.0452	-1.9334	0.81187	-2.3814	0.01724	0.21705	14.7841	31.6467	15.5862	2.04345	3.962	10.2487 Down
Dio1	135.281	-1.3223	0.43887	-3.0129	0.00259	0.06041	214.863	232.405	132.483	60.2818	44.5725	127.083 Down
Shisal2a	234.887	-0.9358	0.46048	-2.0321	0.04214	0.37113	240.489	332.29	352.638	128.737	72.3066	282.863 Down
Txndc12	2919.42	0.32546	0.09906	3.28531	0.00102	0.03178	2694.65	2514.92	2564.91	3208.22	3151.77	3382.06 Up
Ttc39a	2393.89	-0.7384	0.2921	-2.5278	0.01148	0.16493	2607.92	3478.17	2894.17	1564.26	1206.43	2612.38 Down
Skint8	8.1437	3.50787	1.41376	2.48123	0.01309	0.17941	0	3.95583	0	16.3476	3.962	24.5968 Up
Skint3	101.722	-1.1338	0.46372	-2.4449	0.01449	0.19279	133.057	188.891	97.4139	36.7821	104.993	49.1936 Down
Mknk1	2922.66	0.28948	0.14295	2.02508	0.04286	0.37291	2771.53	2518.88	2600.95	3360.45	3525.19	2758.94 Up
Ptch2	179.321	-0.5574	0.25962	-2.147	0.03179	0.3119	173.467	229.438	237.69	179.824	138.67	116.835 Down
Kif2c	84.2648	1.41769	0.50815	2.78988	0.00527	0.09983	50.266	50.4369	37.0173	187.997	132.727	47.1438 Up
Cdc20	406.002	1.03424	0.19465	5.31346	1.08E-07	2.00E-05	271.042	270.975	257.173	664.121	499.212	473.488 Up
Tie1	16489.7	0.2808	0.14005	2.00501	0.04496	0.38034	14487.5	13574.4	16608.1	18778.3	19264.3	16225.7 Up
Olfr1343-	3.37714	5.22322	1.74307	2.99657	0.00273	0.0628	0	0	0	9.19553	5.94301	5.12433 Up
Gm12877	37.1984	1.90186	0.42741	4.44969	8.60E-06	0.00075	11.8273	12.8565	22.4052	64.3687	58.4396	53.293 Up
Ppt1	5828.03	0.62789	0.17605	3.56652	0.00036	0.01481	5046.32	4479.98	4212.18	7084.64	8334.07	5810.99 Up
Cap1	15974.4	0.36477	0.14537	2.5092	0.0121	0.17068	15213.9	13554.7	13128.5	17733.1	20364.7	15851.6 Up
Ndufs5	1616.42	-0.4996	0.22544	-2.2161	0.02669	0.28205	1616.4	2075.82	1988.22	1294.53	1020.22	1703.33 Down
Rhbdl2	46.3279	-1.909	0.55812	-3.4204	0.00063	0.02243	44.3524	121.642	53.5776	16.3476	28.7245	13.3233 Down
Cdca8	169.379	1.28839	0.36863	3.49504	0.00047	0.01821	110.388	114.719	70.138	326.952	250.597	143.481 Up
Grik3	105.134	-0.7282	0.31885	-2.2838	0.02239	0.25524	173.467	115.708	104.233	82.7598	92.1166	62.5168 Down
Lsm10	775.512	0.34247	0.14779	2.31734	0.02048	0.2424	676.128	647.768	727.682	821.467	802.306	977.722 Up
Clspn	87.3204	1.33743	0.45867	2.91589	0.00355	0.07546	68.9926	45.4921	34.0948	155.302	156.499	63.5417 Up
Gjb3	131.246	0.6423	0.23185	2.77028	0.0056	0.10454	110.388	90.9841	106.181	150.194	185.224	144.506 Up
Rnf19b	2997.63	0.3832	0.16114	2.378	0.01741	0.21817	2867.14	2381.41	2557.11	3282.8	3930.31	2966.99 Up
Hpca	9.57338	-1.7518	0.84417	-2.0751	0.03797	0.34837	8.87048	23.735	11.6897	2.04345	4.9525	6.1492 Down
Fndc5	987.653	-3.0527	0.75816	-4.0265	5.66E-05	0.00347	1207.37	3286.31	794.897	88.8901	261.492	286.962 Down
Marcksl1	881.934	0.43784	0.13865	3.15783	0.00159	0.04285	823.969	734.796	688.716	972.683	1087.57	983.871 Up
Lck	985.676	0.72026	0.23838	3.02145	0.00252	0.05937	823.969	607.22	802.69	1341.53	1450.09	888.559 Up
Fam167b	43.8394	-2.0705	0.58641	-3.5309	0.00041	0.0164	129.115	36.5914	46.7587	17.3693	17.829	15.373 Down
E330017L1	31.9392	-2.9348	0.71954	-4.0787	4.53E-05	0.00291	32.5251	105.819	31.1724	5.10863	11.886	5.12433 Down
Tinagl1	10282	0.43453	0.18362	2.36642	0.01796	0.2233	8900.05	7712.88	9622.54	12126.9	13813.5	9515.88 Up
Fabp3	2288.39	-3.2313	0.48923	-6.605	3.98E-11	1.62E-08	2759.7	7643.66	2005.75	319.8	675.522	325.907 Down
Nkain1	119.012	-0.8457	0.33404	-2.5318	0.01135	0.16434	175.438	186.913	96.4397	100.129	77.2591	77.8898 Down
Laptm5	6799.33	0.71094	0.33536	2.11995	0.03401	0.32506	6812.53	5189.06	3469.88	7321.68	12288.2	5714.65 Up
Fgr	1035.28	0.93718	0.32663	2.86923	0.00411	0.08423	839.739	828.747	462.716	1195.42	1917.61	967.473 Up
Tent5b	1154.94	0.35268	0.14436	2.44303	0.01456	0.19345	1061.5	1015.66	966.345	1374.22	1389.67	1122.23 Up
Nr0b2	8.90106	-5.5892	2.02606	-2.7587	0.0058	0.10597	8.87048	43.5142	0	1.02173	0	0 Down
Sfn	1166.53	-0.781	0.23766	-3.2861	0.00102	0.03174	1034.89	1782.1	1607.33	788.772	804.287	981.822 Down
Cd52	1926.79	0.54059	0.17451	3.09771	0.00195	0.04978	1784.94	1476.51	1448.54	2309.1	2615.91	1925.72 Up
Sh3bgrl3	3778.85	0.31145	0.13544	2.29947	0.02148	0.24997	3722.64	3230.93	3164	4599.81	3878.8	4076.92 Up
Cep85	9608.01	2.35828	0.84852	2.77928	0.00545	0.10239	4794	3865.84	748.138	16037	25056.7	7146.39 Up
Trim63	170.344	-4.1761	1.88933	-2.2104	0.02708	0.28467	149.813	678.425	140.276	2.04345	51.506	0 Down
Stmn1	4547.2	0.32306	0.15465	2.08894	0.03671	0.3402	4057.75	3685.85	4376.8	5656.27	4316.6	5189.92 Up
Selenon	4057.45	0.29967	0.11262	2.66094	0.00779	0.12787	3708.85	3320.92	3882.92	4447.57	4593.94	4390.53 Up
Runx3	236.104	0.7472	0.32017	2.3338	0.01961	0.236	214.863	158.233	155.862	273.822	415.02	198.824 Up
Grhl3	68.9203	-0.884	0.4468	-1.9786	0.04786	0.3921	56.1797	102.852	109.104	44.9559	29.715	70.7157 Down
Il22ra1	48.8168	-0.9653	0.39552	-2.4406	0.01466	0.19369	62.0933	71.205	60.3966	20.4345	33.677	45.0941 Down
Cnr2	580.78	0.60315	0.23144	2.60609	0.00916	0.14264	531.243	375.804	476.354	712.143	843.907	545.229 Up
Ephb2	209.454	-1.1187	0.26306	-4.2527	2.11E-05	0.00153	220.776	365.914	273.733	130.781	115.889	149.63 Down
Rap1gap	3776.12	0.67188	0.31598	2.1263	0.03348	0.32299	3377.68	2721.61	2637.97	3687.41	7155.38	3076.65 Up
Pla2g2d	269.418	1.25902	0.57913	2.17396	0.02971	0.30007	205.007	171.09	100.336	245.214	763.676	131.183 Up
Pla2g2e	12.5519	-1.6518	0.74728	-2.2104	0.02708	0.28467	17.741	29.6687	9.74139	4.0869	7.92401	6.1492 Down
Pax7	5.92099	-5.9906	1.7689	-3.3866	0.00071	0.02449	13.7985	19.7792	1.94828	0	0	0 Down
Padi1	21.7248	-2.2918	0.77613	-2.9529	0.00315	0.07037	17.741	62.3044	28.25	4.0869	13.867	4.09946 Down

Crocc	718.955	-0.5411	0.16531	-3.273	0.00106	0.0325	811.156	761.498	983.88	563.992	553.69	639.516	Down
Fam131c	97.5255	-0.8529	0.38716	-2.203	0.0276	0.28707	117.287	138.454	120.793	78.6729	35.658	94.2877	Down
Clcnka	39.8616	2.29407	1.03477	2.21698	0.02662	0.28184	5.91365	34.6135	0	53.1297	89.1451	56.3676	Up
Hspb7	2018.17	-2.8101	0.73171	-3.8405	0.00012	0.00633	2375.32	6368.89	1853.79	373.951	872.631	264.415	Down
Tmem82	111.963	-0.6784	0.29752	-2.28	0.02261	0.25693	145.87	113.73	153.914	81.738	110.936	65.5914	Down
Slc25a34	142.029	-1.3052	0.38257	-3.4116	0.00065	0.02309	207.963	282.842	115.922	62.3252	98.0596	85.0639	Down
Gm29367	32.5685	-1.2146	0.49944	-2.4318	0.01502	0.19718	31.5395	50.4369	54.5518	14.3042	14.8575	29.7211	Down
Lrrc38	2.46355	-4.7255	1.97301	-2.3951	0.01662	0.21222	6.89926	5.93375	1.94828	0	0	0	Down
Tnfrsf1b	2583.73	0.58156	0.27913	2.08351	0.0372	0.34382	2262.96	2124.28	1822.61	2866.96	4380.99	2044.61	Up
Zfp986	2.85562	3.93452	1.7035	2.30967	0.02091	0.24604	0	0.98896	0	4.0869	6.93351	5.12433	Up
Zfp987	5.03944	3.84331	1.43616	2.6761	0.00745	0.12383	0	1.97792	0	8.1738	11.886	8.19893	Up
Zfp981	35.6024	2.05742	0.62788	3.27675	0.00105	0.03233	13.7985	22.746	4.87069	54.1514	83.2021	34.8454	Up
Zfp979	259.026	-0.5438	0.20871	-2.6053	0.00918	0.14264	285.827	324.378	311.724	210.475	250.597	171.153	Down
Mfn2	4172.69	-0.6182	0.19008	-3.2526	0.00114	0.0342	4454.95	6327.35	4377.78	3182.67	3604.43	3088.95	Down
Plod1	3949.11	0.51943	0.19234	2.70065	0.00692	0.11842	2893.75	4052.75	2790.91	4904.28	4922.79	4130.21	Up
Nppa	1041.22	-9.785	1.93112	-5.067	4.04E-07	6.19E-05	300.611	5873.42	66.2414	0	2.9715	4.09946	Down
Fbxo44	454.582	-0.511	0.21964	-2.3266	0.01998	0.23859	668.243	411.407	523.112	361.691	368.466	394.573	Down
Fbxo2	165.193	-0.6493	0.28628	-2.2681	0.02333	0.2617	254.287	146.366	204.569	112.39	120.841	152.705	Down
Gm13206	8.48302	2.68237	1.0779	2.48851	0.01283	0.17723	0.98561	1.97792	3.89655	8.1738	29.715	6.1492	Up
Masp2	123.421	-1.7573	0.26529	-6.6239	3.50E-11	1.45E-08	170.51	168.123	232.819	62.3252	54.4775	52.2682	Down
Gm572	5.61675	-2.4887	1.24723	-1.9953	0.04601	0.38389	5.91365	17.8012	4.87069	3.06518	0	2.04973	Down
Cort	32.584	-1.9823	0.55347	-3.5815	0.00034	0.01424	59.1365	28.6798	68.1897	8.1738	12.8765	18.4476	Down
Pik3cd	1799.03	0.50954	0.17965	2.83637	0.00456	0.09101	1531.64	1486.4	1435.88	1989.3	2588.18	1762.77	Up
Tmem201	1314.54	-0.3173	0.13713	-2.3136	0.02069	0.2444	1304.95	1557.61	1512.84	1174.98	1088.56	1248.29	Down
Slc25a33	737.059	0.41163	0.18397	2.23746	0.02526	0.27483	612.063	706.116	579.612	816.359	716.132	992.07	Up
Eno1	6394.4	0.50051	0.13699	3.65355	0.00026	0.01147	5529.26	5811.12	4548.25	7577.12	7262.35	7638.33	Up
Rnf207	136.176	-3.2099	0.54601	-5.8789	4.13E-09	1.21E-06	173.467	449.976	113.974	18.3911	36.6485	24.5968	Down
Ajap1	24.2081	1.23989	0.51116	2.42562	0.01528	0.19929	7.88487	16.8123	18.5086	30.6518	39.62	31.7708	Up
Wrap73	916.596	0.24745	0.12111	2.04313	0.04104	0.36471	810.17	816.879	887.44	947.139	989.51	1048.44	Up
Gm13110	166.11	-0.9837	0.39948	-2.4624	0.0138	0.18629	189.237	203.725	268.862	104.216	58.4396	172.177	Down
Mxra8	20060.1	-0.4332	0.13905	-3.1152	0.00184	0.04766	21691.3	25040.4	22415.9	17073	15130.9	19009.2	Down
Acap3	1792.43	0.37639	0.16408	2.29398	0.02179	0.2515	1606.54	1487.39	1585.9	2270.27	2151.37	1653.11	Up
Tnfrsf4	200.517	0.59815	0.22651	2.64073	0.00827	0.13297	167.553	150.322	160.733	289.148	242.673	192.675	Up
Gm37090	492.71	-0.677	0.19149	-3.5352	0.00041	0.01622	524.344	622.055	672.156	411.755	314.979	410.971	Down
9430015G	1073.82	0.35915	0.16834	2.13349	0.03288	0.31902	894.933	895.996	1031.61	1358.89	1251.99	1009.49	Up
Isg15	568.442	0.74078	0.16779	4.41493	1.01E-05	0.00084	489.848	385.694	401.345	777.533	699.294	656.939	Up
Perm1	500.161	-1.5841	0.43775	-3.6187	0.0003	0.01276	479.991	1330.15	440.311	231.932	324.884	193.7	Down
Samd11	39.0312	3.15612	0.91448	3.45127	0.00056	0.02067	7.88487	10.8785	4.87069	76.6294	127.775	6.1492	Up
Dbf4	501.918	0.47652	0.1367	3.48585	0.00049	0.01872	444.51	424.263	390.63	572.166	611.139	568.801	Up
Abcb4	60.6765	-1.3818	0.55025	-2.5113	0.01203	0.17	55.1941	160.211	47.7328	28.6083	42.5915	29.7211	Down
Tmem243	4987.06	0.3347	0.15476	2.1627	0.03056	0.30543	5004.92	4166.48	4062.16	5692.03	6116.34	4880.41	Up
Cacna2d1	2495.19	-0.34	0.12204	-2.7858	0.00534	0.10063	2765.62	3016.32	2581.47	2240.64	2064.2	2302.87	Down
Gm28710	5.9148	-2.9483	1.19089	-2.4757	0.0133	0.18144	12.8129	4.94479	13.6379	2.04345	0	2.04973	Down
Ptpn12	7441.46	0.29058	0.12807	2.26889	0.02327	0.26151	6773.1	6246.26	7064.45	8269.85	8928.37	7366.74	Up
Gsap	5272.82	0.52502	0.15531	3.38056	0.00072	0.02491	4939.87	3894.52	4137.17	6543.13	6643.29	5478.93	Up
AI506816	1547.02	0.40141	0.15982	2.51165	0.01202	0.16994	1287.2	1392.45	1319.96	1911.65	1918.6	1452.23	Up
Kcnh2	892.894	-1.2543	0.2269	-5.5277	3.24E-08	7.30E-06	1105.85	1616.95	1052.07	479.189	486.336	616.969	Down
Asb10	68.1598	-4.43	0.85118	-5.2046	1.94E-07	3.44E-05	64.0646	290.754	36.0431	2.04345	9.90501	6.1492	Down
Smarcd3	680.128	-0.3856	0.12612	-3.0571	0.00223	0.05441	790.458	804.023	716.966	574.21	587.367	607.745	Down
Gm7361	18.7929	-3.1957	1.15911	-2.757	0.00583	0.10621	11.8273	80.1056	9.74139	6.13035	4.9525	0	Down
Cnpy1	21.3157	-1.3845	0.57077	-2.4256	0.01528	0.19929	19.7122	45.4921	27.2759	14.3042	11.886	9.22379	Down
Hadha	7262.08	-0.3509	0.15866	-2.2118	0.02698	0.28409	7595.1	9666.08	7161.87	5966.88	6595.75	6586.81	Down
Hadhb	6844.31	-0.5402	0.24092	-2.2422	0.02495	0.27329	6843.08	11247.4	6242.28	5349.75	5548.79	5834.56	Down
Gm9899	249.271	-1.0993	0.26504	-4.1477	3.36E-05	0.00231	279.913	410.418	329.259	163.476	118.86	193.7	Down
Kcnk3	1751.6	-0.7062	0.26134	-2.7021	0.00689	0.11825	1627.24	3079.62	1808.98	1351.74	1420.38	1221.64	Down
Trim54	165.116	-4.3034	1.68418	-2.5552	0.01061	0.15699	149.813	663.591	129.56	6.13035	41.601	0	Down
Slc5a1	459.84	0.87457	0.29214	2.99364	0.00276	0.0632	347.92	359.981	265.94	458.755	848.859	477.587	Up
Spon2	856.517	-1.187	0.53376	-2.2239	0.02615	0.27986	322.294	1411.24	1837.23	400.516	399.172	768.649	Down
Gm20465	6.23302	6.10776	1.49798	4.07732	4.56E-05	0.00292	0	0	0	13.2824	13.867	10.2487	Up
Gm9903	21.5119	-1.3373	0.61266	-2.1828	0.02905	0.29524	19.7122	42.5252	30.1983	17.3693	5.94301	13.3233	Down
Tacc3	347.446	0.50426	0.21797	2.31339	0.0207	0.24442	322.294	263.063	276.655	495.537	399.172	327.957	Up
Nat8l	209.451	-1.1166	0.33101	-3.3732	0.00074	0.02531	261.186	371.848	226.974	102.173	106.974	187.55	Down
Dok7	197.76	-1.2789	0.37362	-3.423	0.00062	0.02229	175.438	426.241	238.664	107.281	143.623	95.3125	Down
Trmt44	413.137	0.3481	0.15193	2.29118	0.02195	0.25271	355.805	349.102	385.759	451.603	507.136	429.419	Up
Htra3	1187.46	-0.7611	0.24744	-3.076	0.0021	0.05218	1231.03	1965.06	1284.89	960.422	988.52	694.859	Down
Ablim2	493.561	-0.6154	0.18944	-3.2486	0.00116	0.03459	520.401	727.873	543.569	390.299	404.124	375.101	Down
Ccdc96	695.06	-0.6881	0.26277	-2.6186	0.00883	0.13958	739.207	1015.66	818.276	450.581	418.982	727.655	Down
Ppp2r2c	821.498	-1.1637	0.32509	-3.5795	0.00034	0.01432	890.005	1138.29	1379.38	524.145	296.16	701.008	Down
Wfs1	1569.49	-0.336	0.11483	-2.9262	0.00343	0.07439	1779.02	1860.23	1615.12	1406.92	1394.63	1361.02	Down
Cytl1	1523.68	-0.321	0.1555	-2.064	0.03902	0.3548	1571.06	1925.5	1581.03	1278.18	1514.48	1271.86	Down
Otop1	33.9354	-8.5098	2.52099	-3.3756	0.00074	0.02521	15.7697	183.946	3.89655	0	0	0	Down
Zfp518b	447.408	-0.3187	0.14513	-2.1956	0.02812	0.28971	477.035	498.435	514.345	436.277	385.305	373.051	Down
Gm40293	4.93491	-5.7279	2.39119	-2.3954	0.0166	0.21213	0	25.7129	3.89655	0	0	0	Down
C1qtnf7	2372.76	-0.3461	0.10012	-3.4564	0.00055	0.02042	2795.19	2539.64	2633.1	2110.88	2072.13	2085.6	Down
Bst1	1763.34	1.27349	0.43063	2.9573	0.0031	0.06959	1670.61	856.438	568.897	2441.92	3608.39	1433.79	Up
Ncapg	99.459	1.532	0.47777	3.20659	0.00134	0.03837	48.2948	55.3817	49.6811	184.932	201.072	57.3925	Up
Ppargc1a	446.629	-1.1765	0.34079	-3.4524	0.00056	0.02063	390.301	876.217	591.302	213.541	246.635	361.778	Down
Slc34a2	97785.1	0.55312	0.20061	2.75713	0.00583	0.10621	99910.2	66746.8	71142.3	113551	131733	103627	Up
Smim20	1249.17	-0.2809	0.14181	-1.981	0.04759	0.39078	1348.31	1507.17	1255.66	1141.27	1028.14	1214.47	Down
Gm43268	297.521	-0.3441	0.16078	-2.1402	0.03234	0.3155	350.877	311.522	336.078	284.04	253.568	249.042	Down
Gm15975	60.1061	-2.0886	0.4353	-4.7981	1.60E-06	0.00019	92.6472	109.774	89.6207	10.2173	30.7055	27.6714	Down
Pcdh7	677.475	-0.9945	0.30227	-3.2901	0.001	0.03144	705.696	1357.84	642.931	472.037	447.706	438.643	Down
Dthd1	19.0222	-1.4636	0.67141	-2.1798	0.02927	0.29717	20.6978	48.4589	14.6121	12.2607	9.90501	8.19893	Down

Tlr1	335.957	0.57342	0.1916	2.99288	0.00276	0.06326	309.481	263.063	237.69	430.146	426.906	348.454	Up
Gm43552	128.209	-0.4738	0.2341	-2.0238	0.04299	0.37324	169.525	132.52	145.147	122.607	100.041	99.412	Down
Rhoh	423.718	0.53165	0.23256	2.28608	0.02225	0.25476	390.301	292.732	356.535	593.622	529.918	379.2	Up
Yipf7	111.438	-4.2323	1.49469	-2.8315	0.00463	0.09178	119.259	410.418	105.207	1.02173	31.696	1.02487	Down
Corin	457.971	-2.5256	0.53055	-4.7603	1.93E-06	0.00023	483.934	1474.54	382.836	122.607	206.024	77.8898	Down
Tec	768.403	0.33223	0.14541	2.28487	0.02232	0.25503	763.847	655.679	621.5	811.25	901.356	856.788	Up
Sgcb	2684.11	-0.4278	0.1487	-2.8772	0.00401	0.08279	2892.76	3526.62	2818.18	2215.1	2153.35	2498.62	Down
Stap1	496.291	0.70607	0.30837	2.2897	0.02204	0.25315	420.855	373.826	337.052	566.036	888.479	391.499	Up
Gnrhr	2.02993	4.48852	2.00728	2.23612	0.02534	NA	0	0	0	5.10863	2.9715	4.09946	Up
Tmprss11a	8.0797	-1.9244	0.87276	-2.2049	0.02746	0.28689	7.88487	19.7792	10.7155	4.0869	3.962	2.04973	Down
Ugt2b34	6.93407	-2.8543	1.42257	-2.0064	0.04481	0.38008	1.97122	32.6356	1.94828	2.04345	1.981	1.02487	Down
Sult1d1	3330.61	-1.7182	0.8416	-2.0416	0.0412	0.36565	3129.31	8265.71	3930.65	1580.61	362.523	2714.87	Down
Jchain	10679.2	3.25584	0.73137	4.45173	8.52E-06	0.00075	2403.9	2579.2	1089.09	19905.3	32128.9	5968.82	Up
Cxcl5	155.723	-2.3083	0.75588	-3.0538	0.00226	0.05483	27.597	372.837	376.992	36.7821	82.2116	37.92	Down
Cxcl3	61.7787	3.42846	0.83848	4.08891	4.33E-05	0.00281	5.91365	17.8012	7.79311	263.605	37.639	37.92	Up
Cxcl2	78.2997	1.50975	0.68557	2.20218	0.02765	0.28707	76.8775	27.6908	17.5345	220.693	61.4111	65.5914	Up
Areg	605.524	-1.1543	0.51677	-2.2336	0.02551	0.27596	1126.55	575.574	804.638	452.624	105.984	567.776	Down
Cxcl9	635.637	4.77882	1.15698	4.13044	3.62E-05	0.00244	65.0502	54.3927	14.6121	1108.57	2457.43	113.76	Up
Art3	2422.49	-1.1739	0.36811	-3.1889	0.00143	0.04	2327.02	5571.79	2172.33	1328.24	1847.28	1288.26	Down
Cxcl13	738.401	1.96334	0.76923	2.55234	0.0107	0.15773	208.949	504.369	190.931	1570.39	1615.51	340.255	Up
Gm7993	474.362	-5.0049	0.37969	-13.182	1.12E-39	2.42E-36	487.876	1214.44	1057.91	23.4997	31.696	30.746	Down
Prdm8	51.7921	-0.8436	0.35394	-2.3836	0.01714	0.21666	56.1797	79.1166	64.2931	41.8907	41.601	27.6714	Down
Prkg2	1337.11	-0.3119	0.1346	-2.3174	0.02048	0.2424	1450.82	1433	1559.6	1220.96	1297.56	1060.74	Down
Cds1	4478.08	-0.5167	0.22487	-2.2979	0.02157	0.25004	4786.12	5929.79	5098.64	3161.22	3006.17	4886.56	Down
Mapk10	565.747	-3.699	0.92946	-3.9797	6.90E-05	0.00401	585.452	2114.39	452	27.5866	152.537	62.5168	Down
Zfp951	300.883	0.7037	0.17508	4.01925	5.84E-05	0.00357	222.748	210.648	253.276	382.125	335.78	400.723	Up
Gbp8	3130.02	0.48664	0.17087	2.84807	0.0044	0.08844	2327.02	2938.19	2556.14	3568.89	4236.37	3153.51	Up
Gbp11	44.501	1.08144	0.53785	2.01068	0.04436	0.37818	33.5107	39.5583	12.6638	46.9994	88.1546	46.119	Up
Btbd8	74.8366	-0.6463	0.28757	-2.2476	0.0246	0.27077	77.8631	85.0504	111.052	60.2818	59.4301	55.3428	Down
Crlf2	1086.78	0.5351	0.13068	4.09486	4.22E-05	0.00276	846.638	925.665	890.363	1252.64	1412.45	1192.94	Up
Tpst2	4328.13	0.29097	0.11783	2.46948	0.01353	0.18354	3694.06	3798.59	4186.85	4623.31	5126.83	4539.13	Up
Myo18b	257.747	-3.0807	1.09719	-2.8079	0.00499	0.09607	264.143	820.835	298.086	18.3911	132.727	12.2984	Down
Selplg	2595.21	0.66269	0.24656	2.6877	0.00719	0.12138	2363.49	1964.07	1700.85	3067.22	4150.2	2325.42	Up
Acacb	1398.12	-0.6831	0.24692	-2.7666	0.00566	0.1051	1692.29	2235.05	1242.03	923.64	1240.11	1055.61	Down
Myo1h	633.001	-0.6944	0.34853	-1.9924	0.04632	0.38491	616.005	927.643	803.664	392.343	305.074	753.276	Down
Pla2g1b	472.866	0.46389	0.21041	2.20471	0.02747	0.28689	441.553	311.522	439.336	494.515	521.003	629.268	Up
C330018A	31.6204	-1.0467	0.47423	-2.2072	0.0273	0.2859	30.5539	54.3927	42.8621	17.3693	15.848	28.6962	Down
Hspb8	5572.33	-0.4232	0.17968	-2.3554	0.0185	0.22687	5786.51	7922.54	5442.51	5002.37	4671.2	4608.82	Down
Gm7478	9.51354	-3.7271	1.16746	-3.1925	0.00141	0.03981	22.669	16.8123	13.6379	0	3.962	0	Down
Gm15728	253.004	-0.8519	0.35857	-2.3757	0.01751	0.21913	342.992	359.981	273.733	186.976	96.0786	258.266	Down
Nos1	18.4499	-3.023	0.8794	-3.4376	0.00059	0.02146	12.8129	68.2381	17.5345	4.0869	4.9525	3.0746	Down
Tesc	125.962	-0.8131	0.30206	-2.692	0.0071	0.12048	109.403	204.714	167.552	86.8467	97.0691	90.1882	Down
1700021F	79.614	-0.5927	0.27585	-2.1488	0.03165	0.31154	93.6328	111.752	81.8276	68.4556	57.449	64.5665	Down
Tbx5	1998.33	-0.5383	0.25004	-2.1527	0.03135	0.30994	2034.3	2918.42	2147.98	1609.22	2082.03	1198.07	Down
Sds	61.861	-1.9185	0.57247	-3.3513	0.0008	0.02668	56.1797	175.046	62.3449	26.5649	35.658	15.373	Down
Dtx1	663.841	-0.631	0.19168	-3.2921	0.00099	0.03131	868.321	846.548	705.276	533.341	425.915	603.646	Down
Arpc3	9033.49	0.23445	0.10095	2.32246	0.02021	0.24068	7877.97	8136.16	8889.01	9784.04	9591.02	9922.75	Up
Atp2a2	37414.6	-1.5263	0.37767	-4.0414	5.31E-05	0.0033	38952.2	91560.7	36124	15922.6	26142.3	15786	Down
Rhof	579.961	0.51696	0.22917	2.25578	0.02408	0.26726	533.214	397.561	500.707	765.272	771.6	511.408	Up
Kntc1	98.1736	1.19551	0.4166	2.86969	0.00411	0.08418	62.0933	68.2381	48.7069	206.389	127.775	75.8401	Up
Hcar1	53.1844	-1.0385	0.45893	-2.2628	0.02365	0.26379	48.2948	111.752	54.5518	40.869	26.7435	36.8952	Down
Gm16001	32.9521	-1.0347	0.42465	-2.4366	0.01483	0.19554	49.2804	51.4258	32.1466	25.5431	18.8195	20.4973	Down
2810006K	534.527	0.29954	0.14745	2.03144	0.04221	0.37137	492.804	522.17	422.776	600.775	582.414	586.223	Up
Scarb1	4724.03	0.44949	0.17595	2.55457	0.01063	0.15715	4575.2	3678.92	3728.03	5621.53	6206.48	4534.01	Up
Adgrd1	535.973	-1.0658	0.33822	-3.1512	0.00163	0.04351	619.948	958.3	598.121	328.996	495.25	215.222	Down
Nipsnap2	6132.37	-0.6032	0.25345	-2.3798	0.01732	0.21748	6716.92	9462.35	6008.49	4299.42	3960.02	6346.99	Down
Phkg1	287.126	-2.022	0.44418	-4.5522	5.31E-06	0.00053	308.496	806.001	267.888	87.8684	106.974	145.531	Down
Galnt17	130.91	-0.5617	0.25144	-2.2341	0.02548	0.27576	181.352	160.211	126.638	107.281	92.1166	117.86	Down
Eln	6847.89	-0.7167	0.35609	-2.0126	0.04415	0.37732	4178	11845.7	9520.26	5061.63	6339.21	4142.51	Down
Mlxipl	341.458	-0.925	0.34492	-2.6816	0.00733	0.12303	324.265	696.226	321.466	204.345	228.806	273.639	Down
Srrm3	8.04281	-3.4401	1.21554	-2.8301	0.00465	0.09178	3.94243	18.7902	21.431	2.04345	0	2.04973	Down
Vgf	6.74747	-3.1678	1.06068	-2.9866	0.00282	0.06413	13.7985	14.8344	7.79311	2.04345	0.9905	1.02487	Down
Irs3	180.332	-0.5229	0.23533	-2.2218	0.02629	0.28068	182.338	237.35	218.207	139.976	125.794	178.327	Down
Pilra	537.45	1.11863	0.36613	3.05529	0.00225	0.05468	457.322	352.069	207.492	715.208	1022.2	470.413	Up
Azgp1	790.297	0.83517	0.27801	3.0041	0.00266	0.06165	540.114	642.823	520.19	945.096	686.417	1407.14	Up
Gm454	3.60689	-5.2757	1.69305	-3.1161	0.00183	0.04756	7.88487	7.91166	5.84483	0	0	0	Down
Pvrig	9.06701	2.56346	0.87844	2.91818	0.00352	0.07506	4.92804	1.97792	0.97414	12.2607	16.8385	17.4227	Up
6330403L	584.352	-0.3694	0.13537	-2.7289	0.00636	0.11277	603.193	687.326	685.794	515.971	524.965	488.861	Down
Elfn1	896.567	0.39556	0.16602	2.38258	0.01719	0.21691	842.695	713.039	767.621	1035.01	1151.95	869.086	Up
Amz1	736.614	0.91113	0.24906	3.65832	0.00025	0.0113	636.703	491.512	406.216	961.444	1177.71	746.102	Up
Card11	880.065	0.84838	0.38579	2.19908	0.02787	0.2884	938.3	483.601	463.69	1148.42	1598.67	647.715	Up
Fscn1	758.422	1.09578	0.2314	4.73533	2.19E-06	0.00025	493.79	461.843	494.862	1240.37	1136.1	723.555	Up
0610040B	61.4789	-0.6138	0.29671	-2.0685	0.03859	0.35239	81.8055	66.2602	75.0087	42.9125	47.544	55.3428	Down
Nptx2	37.5765	-2.1487	0.47149	-4.5572	5.18E-06	0.00052	54.2085	79.1166	50.6552	8.1738	14.8575	18.4476	Down
Arpc1b	9385.6	0.39789	0.18212	2.18481	0.0289	0.29455	8801.49	7833.54	7663.55	10313.3	12991.4	8710.33	Up
Flt3	307.158	0.90091	0.34602	2.6036	0.00922	0.14322	273.014	171.09	198.724	348.408	589.348	262.366	Up
Mtus2	842.772	-1.0375	0.33617	-3.0863	0.00203	0.0513	952.098	1763.31	684.819	491.45	570.528	594.422	Down
Gm15407	2.46466	-4.7261	1.98464	-2.3813	0.01725	0.21705	4.92804	7.91166	1.94828	0	0	0	Down
2310047D	16.5244	1.1716	0.57012	2.05503	0.03988	0.35961	10.8417	11.8675	7.79311	16.3476	28.7245	23.5719	Up
Alox5ap	810.347	0.69385	0.27137	2.55682	0.01056	0.15671	733.293	658.646	465.638	932.835	1317.37	754.301	Up
Gm15997	133.265	-0.7986	0.37357	-2.1378	0.03253	0.31668	113.345	253.173	141.25	77.6511	118.86	95.3125	Down
Wdr95	829.6	-0.7156	0.28524	-2.5089	0.01211	0.17074	727.379	1505.19	861.138	644.709	587.367	651.815	Down
N4bp2l1	1329.54	0.35027	0.17234	2.03244	0.04211	0.37101	1179.77	1165.98	1161.17	1486.61	1763.09	1220.62	Up

Kl	98.1777	-0.6889	0.32152	-2.1427	0.03214	0.31414	79.8343	147.355	136.379	74.586	81.2211	69.6909	Down
Gm20559	2847.23	0.34358	0.1491	2.30437	0.0212	0.24829	2798.14	2131.2	2599.98	3018.18	3181.49	3354.39	Up
Peg10	170.729	-0.5742	0.21766	-2.6383	0.00833	0.13379	183.323	187.902	241.586	131.803	148.575	131.183	Down
Pdk4	2032.65	-1.0572	0.4374	-2.4171	0.01565	0.20256	1679.48	4298.01	2260	947.139	2180.09	831.166	Down
Dync1i1	2068.68	-0.8225	0.35132	-2.3412	0.01922	0.23297	2132.86	3226.97	2568.8	1463.11	795.372	2224.98	Down
Tac1	3.78766	-5.3461	2.41584	-2.2129	0.0269	0.28339	5.91365	16.8123	0	0	0	0	Down
C1galt1	4466.02	0.48542	0.20333	2.38737	0.01697	0.21493	3457.52	2932.26	4775.23	5227.15	5390.31	5013.64	Up
Col28a1	59.1653	0.83135	0.34242	2.42787	0.01519	0.19829	43.3668	40.5473	43.8362	84.8032	89.1451	53.293	Up
Nxph1	49.8137	1.68584	0.84224	2.00161	0.04533	0.38132	47.3092	13.8454	9.74139	49.0428	159.471	19.4725	Up
Thsd7a	270.798	-0.7829	0.26524	-2.9515	0.00316	0.07062	320.323	457.888	249.379	205.367	197.11	194.725	Down
Ppp1r3a	744.681	-0.8574	0.32813	-2.6129	0.00898	0.14076	943.228	1318.28	617.604	466.928	714.151	407.897	Down
Tfec	366.022	1.27356	0.41596	3.06174	0.0022	0.054	322.294	185.924	134.431	578.297	693.351	281.838	Up
Met	3631.47	0.52621	0.20282	2.5945	0.00947	0.14581	3004.14	2368.55	3556.58	3600.56	4450.32	4808.67	Up
Capza2	11687.5	0.24327	0.11067	2.1982	0.02793	0.28877	10565.7	10510.6	11037	12751.1	13634.2	11626.1	Up
St7	1234.21	0.32324	0.13098	2.46795	0.01359	0.18422	1046.72	1055.22	1187.47	1379.33	1266.85	1469.66	Up
Wnt2	837.185	-0.3869	0.19509	-1.9833	0.04733	0.38999	906.76	786.222	1153.38	657.991	800.325	718.431	Down
Cttnbp2	296.286	-0.8135	0.19895	-4.0887	4.34E-05	0.00281	385.373	416.351	331.207	241.127	181.262	222.396	Down
Ankrd7	6.04298	3.04819	1.34694	2.26305	0.02363	0.26374	0	0	3.89655	7.15208	11.886	13.3233	Up
Kcnd2	94.6837	-2.9432	0.62527	-4.7071	2.51E-06	0.00028	110.388	314.489	77.9311	16.3476	36.6485	12.2984	Down
Ptprz1	1039.24	-0.7488	0.37543	-1.9945	0.0461	0.38407	884.091	1706.94	1318.01	714.186	439.782	1172.45	Down
Aass	683.969	0.56193	0.16042	3.50286	0.00046	0.01781	490.833	538.982	627.345	841.902	743.866	860.887	Up
Asb15	54.333	-1.9073	0.66204	-2.8809	0.00397	0.0823	50.266	172.079	35.069	24.5214	29.715	14.3481	Down
Lmod2	603.804	-3.6619	1.04967	-3.4886	0.00049	0.01856	831.854	1937.37	588.38	24.5214	210.977	29.7211	Down
Grm8	58.057	-1.0956	0.5463	-2.0056	0.0449	0.38025	68.9926	111.752	56.5	42.9125	13.867	54.3179	Down
Gm42547	1097.14	0.27917	0.13759	2.02905	0.04245	0.37204	903.803	1052.25	1017.97	1246.5	1269.82	1092.51	Up
Lep	176.535	-7.4385	0.84174	-8.837	9.83E-19	1.12E-15	216.834	683.37	152.94	4.0869	1.981	0	Down
Flnc	689.72	-2.0624	0.40167	-5.1346	2.83E-07	4.46E-05	602.207	1912.64	824.121	250.323	304.084	244.943	Down
Irf5	1304.03	0.62229	0.21122	2.94622	0.00322	0.07154	1159.08	993.903	928.354	1427.35	2007.75	1307.73	Up
Cpa1	16.1836	-1.2757	0.63519	-2.0083	0.04461	0.37936	15.7697	21.7571	31.1724	6.13035	7.92401	14.3481	Down
Mest	477.376	-0.5136	0.1568	-3.2753	0.00106	0.03241	504.632	589.419	590.328	356.582	404.124	419.17	Down
Gm43748	77.7842	-0.9171	0.44214	-2.0742	0.03806	0.34886	76.8775	146.366	81.8276	49.0428	33.677	78.9147	Down
9330158H	103.842	-1.9987	0.51409	-3.8879	0.0001	0.00543	82.7911	297.676	117.871	35.7604	35.658	53.293	Down
Chrm2	1364.8	-0.9795	0.41717	-2.3479	0.01888	0.23007	1544.45	2934.24	954.656	745.86	1334.2	675.387	Down
Ptn	67.6345	-1.491	0.50538	-2.9503	0.00317	0.07075	63.079	162.189	74.0345	24.5214	29.715	52.2682	Down
Tbxas1	661.55	0.97	0.31996	3.03163	0.00243	0.05803	576.581	410.418	354.586	831.684	1227.23	568.801	Up
Rab19	541.169	0.4392	0.15894	2.76332	0.00572	0.10587	522.373	434.153	421.802	651.861	640.854	575.975	Up
Trbv1	28.0799	1.40626	0.43676	3.21976	0.00128	0.03704	15.7697	11.8675	18.5086	41.8907	44.5725	35.8703	Up
Trbv19	23.296	1.35941	0.64876	2.0954	0.03614	0.33781	9.85609	7.91166	21.431	28.6083	52.4965	19.4725	Up
Trbc1	629.051	0.71578	0.17955	3.98654	6.70E-05	0.00394	474.078	430.197	524.087	882.771	807.258	655.914	Up
Trbc2	607.508	0.69218	0.20296	3.41047	0.00065	0.02315	527.301	402.506	463.69	900.14	720.094	631.317	Up
Trpv6	249.931	0.80955	0.21071	3.84196	0.00012	0.00631	220.776	173.068	150.991	304.474	339.742	310.534	Up
Clcn1	40.0064	-2.4969	0.76745	-3.2535	0.00114	0.03414	73.9207	97.9068	32.1466	2.04345	23.772	10.2487	Down
Ezh2	895.317	0.52597	0.18248	2.88238	0.00395	0.08203	808.199	682.381	711.121	1235.27	1063.8	871.136	Up
Pdia4	6896.82	0.34811	0.13461	2.58617	0.0097	0.14849	5848.6	5905.07	6452.69	7635.35	8650.04	6889.15	Up
Sspo	316.247	-0.9408	0.31329	-3.0031	0.00267	0.0618	253.301	566.673	427.647	208.432	244.654	196.774	Down
Atp6v0e2	2078.86	-0.3404	0.16842	-2.0211	0.04327	0.37347	2247.19	2469.43	2252.21	1626.59	1664.04	2213.71	Down
Gimap9	815.536	0.40897	0.17013	2.40389	0.01622	0.20901	609.106	687.326	805.613	1036.03	869.66	885.484	Up
Aoc1	1350.76	-1.3873	0.40905	-3.3914	0.0007	0.02422	1748.47	2717.66	1396.91	665.143	380.352	1196.02	Down
Cycs	3702.9	-0.5706	0.22588	-2.526	0.01154	0.1652	3440.76	5952.54	3883.89	2980.37	2888.3	3071.52	Down
Snx10	1848.94	0.57565	0.22863	2.51783	0.01181	0.16798	1709.05	1601.12	1144.61	2009.73	2752.6	1876.53	Up
Hoxa1	80.8329	-0.8346	0.34333	-2.4308	0.01507	0.19718	74.9063	135.487	100.336	50.0645	53.487	70.7157	Down
Crhr2	68.6761	-3.7054	0.74307	-4.9865	6.15E-07	9.03E-05	82.7911	253.173	46.7587	3.06518	10.8955	15.373	Down
Adcyap1r1	322.795	-0.8674	0.28144	-3.0821	0.00206	0.05162	515.473	460.854	274.707	265.649	209.986	210.098	Down
Pde1c	168.829	-1.0727	0.37918	-2.8291	0.00467	0.09198	133.057	341.191	212.362	86.8467	126.784	112.735	Down
Herc6	1116.99	0.60191	0.25301	2.37897	0.01736	0.21785	925.487	819.846	916.664	1300.66	1806.67	932.628	Up
Gm5570	4.0578	5.48798	1.78302	3.0779	0.00208	0.05192	0	0	0	3.06518	6.93351	14.3481	Up
Tnip3	143.974	0.91123	0.32197	2.83021	0.00465	0.09178	87.7192	120.653	91.569	198.215	241.682	124.009	Up
Ndnf	8173.38	0.61491	0.22927	2.68206	0.00732	0.12303	7189.03	6089.01	6094.21	12801.2	7161.32	9705.48	Up
Gm31520	32.8496	-1.705	0.83234	-2.0484	0.04052	0.36247	29.5683	98.8958	22.4052	18.3911	24.7625	3.0746	Down
Mad2l1	612.694	0.47655	0.19236	2.47732	0.01324	0.18081	592.351	452.943	491.94	830.663	693.351	614.92	Up
4930597C	1.96469	-4.3995	2.07598	-2.1192	0.03407	NA	5.91365	1.97792	3.89655	0	0	0	Down
Igkv9-124	41.7947	2.17842	0.98992	2.20062	0.02776	0.28797	13.7985	28.6798	2.92242	27.5866	163.433	14.3481	Up
Igkv1-122	52.5752	5.98259	1.28842	4.64334	3.43E-06	0.00036	1.97122	0.98896	1.94828	272.801	34.6675	3.0746	Up
Igkv17-12	109.756	2.42634	0.74864	3.24098	0.00119	0.03519	31.5395	44.5031	27.2759	112.39	411.058	31.7708	Up
Igkv1-117	581.089	3.41326	0.78755	4.33402	1.46E-05	0.00113	146.856	89.9952	62.3449	828.619	2053.31	305.41	Up
Igkv14-11	142.668	2.63719	0.81293	3.24404	0.00118	0.03499	21.6834	27.6908	69.1638	79.6946	596.282	61.492	Up
Igkv1-110	660.689	3.65286	0.45222	8.07755	6.61E-16	5.49E-13	137.985	69.2271	84.7501	877.662	2135.52	658.989	Up
Igkv2-109	81.0798	1.99815	0.65072	3.07066	0.00214	0.05296	67.0214	12.8565	17.5345	146.107	175.319	67.6411	Up
Igkv2-107	5.5827	-2.1866	1.08629	-2.0129	0.04412	0.37732	11.8273	4.94479	10.7155	1.02173	3.962	1.02487	Down
Igkv16-10	53.817	1.75389	0.73813	2.37613	0.0175	0.21902	29.5683	30.6577	13.6379	127.716	106.974	14.3481	Up
Igkv10-96	302.745	3.36741	0.98103	3.43254	0.0006	0.02174	98.5609	35.6025	26.3017	1223.01	192.157	240.843	Up
Igkv10-94	58.7339	2.3421	0.74125	3.15964	0.00158	0.04279	11.8273	30.6577	15.5862	114.433	161.452	18.4476	Up
Igkv19-93	130.554	2.53922	0.32353	7.84855	4.21E-15	3.25E-12	39.4243	38.5694	37.0173	191.063	305.074	172.177	Up
Igkv4-91	241.939	3.50231	1.07417	3.26049	0.00111	0.03359	39.4243	23.735	54.5518	363.734	926.118	44.0692	Up
Igkv4-86	37.2074	3.07743	1.0989	2.80046	0.0051	0.09743	7.88487	11.8675	3.89655	15.3259	5.94301	178.327	Up
Igkv13-84	61.5804	5.05289	1.14622	4.40831	1.04E-05	0.00086	4.92804	1.97792	3.89655	292.213	63.3921	3.0746	Up
Igkv4-80	48.1983	4.98999	1.02067	4.88893	1.01E-06	0.00013	3.94243	0	4.87069	27.5866	216.92	35.8703	Up
Igkv4-74	80.0535	5.73072	1.68236	3.40635	0.00066	0.02324	4.92804	3.95583	0	8.1738	383.324	79.9395	Up
Igkv4-72	100.841	2.43595	0.26896	9.05684	1.34E-19	1.61E-16	35.4819	31.6467	27.2759	159.389	185.224	166.028	Up
Igkv4-70	241.617	4.34434	1.61782	2.6853	0.00725	0.12207	14.7841	42.5252	10.7155	84.8032	1287.65	9.22379	Up
Igkv4-68	82.4161	2.53389	0.7754	3.26783	0.00108	0.03301	23.6546	30.6577	18.5086	264.627	140.651	16.3979	Up
Igkv4-57-	154.162	2.53876	1.27052	1.99821	0.04569	0.38232	14.7841	14.8344	106.181	175.737	574.49	38.9449	Up
Igkv4-57	158.347	2.8397	0.62051	4.57637	4.73E-06	0.00048	21.6834	15.8233	78.9052	127.716	359.552	346.405	Up

Igkv4-53	116.287	2.03655	0.83094	2.45091	0.01425	0.1903	13.7985	78.1277	44.8104	425.038	99.0501	36.8952 Up
Igkv4-51	4.48928	3.67554	1.709	2.1507	0.0315	0.31075	0	0	1.94828	5.10863	17.829	2.04973 Up
Igkv4-50	34.9203	2.19756	1.06263	2.06803	0.03864	0.3525	1.97122	31.6467	3.89655	107.281	54.4775	10.2487 Up
Igkv5-48	92.796	1.64764	0.62052	2.65525	0.00792	0.12926	82.7911	20.7681	31.1724	127.716	230.787	63.5417 Up
Igkv5-45	33.216	4.42765	1.09876	4.02967	5.59E-05	0.00344	5.91365	0.98896	1.94828	132.824	52.4965	5.12433 Up
Igkv12-44	293.121	4.68518	0.95407	4.91076	9.07E-07	0.00012	25.6258	15.8233	24.3535	771.403	851.831	69.6909 Up
Igkv5-43	261.943	4.28316	1.29586	3.30527	0.00095	0.03031	30.5539	29.6687	16.5604	1059.53	419.972	15.373 Up
Igkv12-41	48.6502	1.86838	0.69718	2.6799	0.00736	0.1232	22.669	9.88958	30.1983	85.8249	122.822	20.4973 Up
Igkv8-27	49.0756	3.06352	0.74398	4.11772	3.83E-05	0.00254	21.6834	2.96687	6.81897	55.1732	154.518	53.293 Up
Igkv6-25	61.0179	2.72235	0.584	4.66158	3.14E-06	0.00034	23.6546	10.8785	13.6379	54.1514	77.2591	186.526 Up
Igkv8-24	65.107	2.46767	0.73066	3.37733	0.00073	0.02509	16.7553	12.8565	30.1983	60.2818	236.73	33.8206 Up
Igkv6-23	80.301	2.17461	0.59462	3.65715	0.00026	0.01133	37.4531	15.8233	34.0948	154.281	197.11	43.0444 Up
Igkv8-21	131.622	4.49996	1.42755	3.15222	0.00162	0.04341	18.7266	5.93375	8.76725	69.4773	674.531	12.2984 Up
Igkv8-19	60.1439	2.80957	0.74097	3.79176	0.00015	0.00739	9.85609	5.93375	29.2242	191.063	36.6485	88.1385 Up
Igkv6-17	283.327	3.68027	1.11535	3.29965	0.00097	0.0307	68.007	30.6577	24.3535	468.972	1060.83	47.1438 Up
Igkv6-15	218.157	2.66581	0.69997	3.80849	0.00014	0.00701	79.8343	63.2933	35.069	813.293	243.663	73.7903 Up
Igkv6-14	32.3878	3.31882	0.80532	4.12112	3.77E-05	0.00252	5.91365	6.92271	4.87069	95.0205	70.3256	11.2735 Up
Igkv3-12	106.483	2.91531	0.89028	3.27461	0.00106	0.03241	33.5107	24.724	16.5604	51.0863	485.345	27.6714 Up
Igkv3-10	238.287	4.78101	1.47513	3.24108	0.00119	0.03519	18.7266	18.7902	12.6638	67.4339	1291.61	20.4973 Up
Igkv3-7	57.3393	2.91723	0.72867	4.00351	6.24E-05	0.00372	14.7841	5.93375	19.4828	208.432	56.4585	38.9449 Up
Igkv3-5	195.928	4.81627	1.10156	4.37224	1.23E-05	0.00099	25.6258	3.95583	10.7155	190.041	847.869	97.3623 Up
Igkv3-4	136.955	2.79765	1.30572	2.14262	0.03214	0.31414	54.2085	27.6908	21.431	90.9336	613.12	14.3481 Up
Igkv3-1	29.1482	2.74814	0.93187	2.94908	0.00319	0.07096	5.91365	12.8565	3.89655	110.346	33.677	8.19893 Up
Gm30211	788.32	1.10608	0.46905	2.35812	0.01837	0.22573	694.854	448.987	356.535	1136.16	1695.74	397.648 Up
Smyd1	805.262	-2.0148	0.42815	-4.7057	2.53E-06	0.00028	953.084	2252.85	667.285	281.996	415.02	261.341 Down
Cd8b1	364.26	0.88124	0.20034	4.39877	1.09E-05	0.00089	255.273	216.582	297.112	540.493	447.706	428.394 Up
Cd8a	454.155	0.87651	0.16514	5.30765	1.11E-07	2.05E-05	314.409	294.71	351.664	659.013	551.709	553.428 Up
Mat2a	25011.1	-0.4815	0.15237	-3.16	0.00158	0.04279	24107	32833.4	30499.3	20341.5	21370.1	20915.5 Down
Capg	2781.42	0.71431	0.24344	2.93431	0.00334	0.07329	2697.61	1844.41	1777.8	3449.34	4239.34	2680.02 Up
Tgoln1	9409.15	0.37239	0.17634	2.1118	0.0347	0.32919	8584.65	8147.04	7872.99	10089.5	12976.6	8784.13 Up
Suclg1	5840.05	-0.3495	0.17823	-1.9609	0.04989	0.39986	5824.95	7833.54	5973.42	4889.98	4660.31	5858.13 Down
Reg1	6.90288	-5.2152	1.63081	-3.1979	0.00138	0.03942	4.92804	28.6798	6.81897	0	0.9905	0 Down
Gm20383	20.0437	-3.4351	0.77589	-4.4273	9.54E-06	0.00081	27.597	40.5473	41.888	6.13035	0	4.09946 Down
Eva1a	485.292	-0.9128	0.25529	-3.5756	0.00035	0.01451	518.43	638.867	744.242	350.452	243.663	416.096 Down
Tacr1	194.498	-2.6916	0.56079	-4.7997	1.59E-06	0.00019	366.646	398.55	245.483	42.9125	99.0501	14.3481 Down
Dok1	1157.91	0.40659	0.18723	2.17161	0.02988	0.30087	1162.03	841.603	983.88	1283.29	1515.47	1161.17 Up
Lbx2	87.1344	0.68477	0.28142	2.43325	0.01496	0.19658	79.8343	54.3927	66.2414	110.346	93.1071	118.884 Up
Actg2	1469.14	-1.2486	0.37161	-3.3598	0.00078	0.02626	1136.41	2853.14	2214.22	819.424	585.386	1206.27 Down
Stambp	2223.48	-0.3084	0.13437	-2.2954	0.02171	0.25084	2280.7	2359.65	2740.25	2059.8	1836.39	2064.08 Down
Gm7443	4.19648	2.91221	1.47323	1.97676	0.04807	0.3926	2.95683	0	0	5.10863	8.91451	8.19893 Up
Dysf	1762.85	-0.4477	0.18771	-2.385	0.01708	0.21608	1594.71	2247.9	2260	1562.22	1576.88	1335.4 Down
Cyp26b1	2438.16	-0.9921	0.45589	-2.1763	0.02954	0.29903	3012.02	2881.82	3841.03	3063.13	1107.38	723.555 Down
Spr-ps1	29.0957	-3.1902	0.59386	-5.3719	7.79E-08	1.52E-05	32.5251	71.205	53.5776	6.13035	3.962	7.17406 Down
Nat8f7	125.861	-0.5289	0.26442	-2	0.0455	0.38155	168.539	115.708	161.707	112.39	87.1641	109.661 Down
Nat8f3	160.006	0.60619	0.30275	2.00228	0.04525	0.3813	175.438	106.807	98.388	228.866	176.309	174.227 Up
Alms1-ps2	50.0651	1.14677	0.52292	2.19299	0.02831	0.29049	54.2085	18.7902	20.4569	58.2383	56.4585	92.2379 Up
Nat8f6	41.8784	-1.5865	0.3855	-4.1154	3.87E-05	0.00255	58.1509	51.4258	78.9052	21.4562	19.81	21.5222 Down
Dusp11	10259.6	0.20967	0.09541	2.19765	0.02797	0.28884	10053.2	9091.49	9401.41	10987.6	11212.5	10811.3 Up
Tgfa	1954.74	0.28603	0.11339	2.52261	0.01165	0.16637	1677.51	1764.3	1843.07	2047.54	2293.01	2103.02 Up
Pcyox1	4896.11	0.41453	0.20695	2.00306	0.04517	0.38075	5168.53	3784.74	3639.38	5551.03	6573.95	4659.04 Up
Gm28719	65.5512	-1.7772	0.45829	-3.8779	0.00011	0.00562	56.1797	125.598	122.741	19.4128	38.6295	30.746 Down
Gm44386	29.8143	-3.3217	0.73243	-4.5352	5.76E-06	0.00057	39.4243	92.9621	30.1983	9.19553	1.981	5.12433 Down
Anxa4	12295.6	0.24144	0.12267	1.96828	0.04904	0.3969	12520.2	10295.1	10992.2	13776.9	12821	13368.4 Up
Aak1	3661.39	0.24338	0.09758	2.49408	0.01263	0.17504	3318.54	3341.69	3399.74	3914.23	4188.83	3805.33 Up
Gkn3	115.58	1.82673	0.37244	4.90479	9.35E-07	0.00013	37.4531	75.1608	39.9397	138.955	229.796	172.177 Up
Arhgap25	1200.15	0.57391	0.19776	2.90203	0.00371	0.07796	1142.32	835.67	915.69	1549.96	1583.81	1173.47 Up
Prokr1	32.2491	1.48248	0.64522	2.29764	0.02158	0.25004	9.85609	15.8233	25.3276	28.6083	85.1831	28.6962 Up
1810020C	442.015	-0.5551	0.27028	-2.0537	0.04	0.36025	482.948	589.419	505.578	310.605	265.454	498.085 Down
Kbtbd12	50.036	-2.1744	0.78823	-2.7586	0.00581	0.10597	50.266	177.023	18.5086	10.2173	25.753	18.4476 Down
Mcm2	869.22	0.40209	0.17882	2.2486	0.02454	0.27035	904.789	677.436	664.362	1059.53	919.185	990.02 Up
4933427D	117.21	-0.7185	0.35474	-2.0254	0.04282	0.37291	164.597	142.41	130.535	67.4339	133.718	64.5665 Down
Slc41a3	690.664	-0.7822	0.28522	-2.7424	0.0061	0.10923	651.487	1246.09	722.811	506.776	586.377	430.444 Down
Fbln2	864.352	-1.1008	0.20278	-5.4288	5.67E-08	1.17E-05	1145.28	1445.86	945.889	605.883	534.87	508.333 Down
Lmod3	97.7199	-3.9279	0.71153	-5.5203	3.38E-08	7.53E-06	116.302	355.036	78.9052	9.19553	22.7815	4.09946 Down
Cav3	138.878	-1.8788	0.43148	-4.3543	1.34E-05	0.00105	155.726	368.881	130.535	61.3035	59.4301	57.3925 Down
Mtmr14	2081.22	0.20788	0.1054	1.97237	0.04857	0.39474	1971.22	1885.94	1937.56	2144.6	2373.24	2174.77 Up
Cidec	978.753	-1.6267	0.47154	-3.4497	0.00056	0.02074	1178.79	2648.43	608.837	523.123	585.386	327.957 Down
Cand2	284.568	-1.4134	0.3203	-4.4126	1.02E-05	0.00085	320.323	616.121	304.905	152.237	141.642	172.177 Down
Gm4640	23.769	-1.0515	0.4866	-2.1608	0.03071	0.30647	30.5539	27.6908	37.9914	14.3042	20.8005	11.2735 Down
Ret	1592.87	0.23799	0.11972	1.98783	0.04683	0.38762	1510.94	1343.01	1531.35	1657.24	1700.69	1814.01 Up
Tmem121	163.77	0.54391	0.24485	2.22145	0.02632	0.2807	154.741	98.8958	146.121	201.28	197.11	184.476 Up
Bid	628.787	0.30736	0.14555	2.11166	0.03472	0.32919	530.257	565.684	590.328	649.817	774.572	662.063 Up
Slc6a13	34.2213	-4.1246	0.75338	-5.4747	4.38E-08	9.56E-06	49.2804	114.719	30.1983	2.04345	3.962	5.12433 Down
Gm6637	18.5521	1.22113	0.51868	2.35431	0.01856	0.22731	8.87048	12.8565	11.6897	29.63	26.7435	21.5222 Up
Gdf3	20.8774	5.97068	1.29353	4.6158	3.92E-06	0.0004	1.97122	0	0	27.5866	77.2591	18.4476 Up
Dppa3	2.70693	4.90373	1.84076	2.66398	0.00772	0.1274	0	0	0	6.13035	3.962	6.1492 Up
Clec4a3	779.55	1.34611	0.34847	3.8629	0.00011	0.00591	470.135	517.225	333.155	972.683	1705.64	678.461 Up
Clec4b1	86.1751	0.91386	0.43553	2.09829	0.03588	0.336	67.0214	83.0725	29.2242	134.868	119.851	83.0141 Up
Clec4a2	369.795	1.09224	0.2705	4.0379	5.39E-05	0.00334	306.524	235.372	166.578	546.623	552.699	410.971 Up
Clec4n	560.758	1.60323	0.79109	2.0266	0.0427	0.37291	528.286	194.825	110.078	1139.22	1052.9	339.231 Up
Clec4e	356.858	1.42451	0.56308	2.52986	0.01141	0.16459	282.87	212.626	85.7242	421.973	935.033	202.923 Up
Clstn3	241	-1.0503	0.31847	-3.298	0.00097	0.03083	270.057	438.108	266.914	138.955	120.841	211.122 Down
C1rl	434.365	-0.4647	0.23506	-1.9769	0.04805	0.3926	623.89	514.258	373.095	369.865	399.172	325.907 Down

Ptpn6	2446.77	0.51644	0.21714	2.37839	0.01739	0.21806	2419.67	1880.01	1740.79	2894.55	3479.63	2265.98	Up
Eno2	353.397	-0.4579	0.22969	-1.9934	0.04622	0.38473	330.179	492.501	404.268	309.583	245.644	338.206	Down
Cdca3	222.071	0.9479	0.41284	2.29605	0.02167	0.25055	112.359	237.35	105.207	421.973	251.587	203.948	Up
Cd4	435.142	1.00865	0.37885	2.66241	0.00776	0.12767	350.877	239.328	276.655	617.122	840.935	285.938	Up
Cd9	25836.3	0.26207	0.09841	2.66297	0.00775	0.12767	24629.4	22399.9	23459.2	29053.8	28599.7	26876.1	Up
Vwf	30169	0.62737	0.21491	2.91923	0.00351	0.07495	21600.6	27165.7	22366.2	35934.1	46023.6	27923.5	Up
Gm29009	37.2759	-1.8272	0.79504	-2.2983	0.02155	0.25004	27.597	119.664	27.2759	11.239	30.7055	7.17406	Down
Kcna5	43.367	-1.672	0.72882	-2.2941	0.02178	0.2515	29.5683	124.609	43.8362	17.3693	7.92401	36.8952	Down
Rad51ap1	95.1361	1.38272	0.41762	3.31095	0.00093	0.02986	59.1365	48.4589	50.6552	169.606	175.319	67.6411	Up
Cracr2a	2935.33	0.2839	0.13792	2.05851	0.03954	0.35848	2995.26	2562.39	2384.69	3077.44	3183.47	3408.7	Up
Tspan11	3872.49	0.63387	0.21156	2.99621	0.00273	0.0628	2700.57	3000.5	3404.61	4146.16	6059.88	3923.19	Up
9330102E	71.2265	-1.3236	0.51406	-2.5747	0.01003	0.15202	120.244	135.487	49.6811	44.9559	55.468	21.5222	Down
A2ml1	1967.46	-1.5133	0.75367	-2.0079	0.04465	0.37936	1494.18	6146.37	1101.75	1106.53	990.501	965.424	Down
BC035044	179.59	0.68662	0.26033	2.6375	0.00835	0.134	111.374	171.09	130.535	209.454	264.464	190.625	Up
Clec2g	248.851	1.28587	0.5483	2.34521	0.01902	0.23116	55.1941	138.454	240.612	321.843	557.652	179.352	Up
BC064078	698.431	-1.1528	0.42531	-2.7106	0.00672	0.11632	1467.57	1024.56	398.423	426.059	331.818	542.154	Down
Clec12a	830.448	0.63678	0.18527	3.43706	0.00059	0.02146	637.689	752.597	560.13	911.379	1168.79	952.1	Up
Clec1b	877.626	0.49431	0.1947	2.53885	0.01112	0.16205	606.149	850.504	729.63	979.835	1190.58	909.056	Up
Clec7a	2818.48	1.43756	0.68786	2.0899	0.03663	0.3402	2651.29	1254	654.621	4085.88	6098.51	2166.57	Up
Olr1	628.916	1.7297	0.50412	3.43113	0.0006	0.02181	487.876	234.383	151.966	1146.38	1348.07	404.822	Up
Klrd1	564.941	0.6728	0.23303	2.88713	0.00389	0.08104	353.834	533.048	419.854	651.861	846.878	584.174	Up
Klrk1	421.551	0.92565	0.33187	2.78919	0.00528	0.09992	205.992	329.323	337.052	490.428	791.41	375.101	Up
Klrc2	178.799	0.69799	0.34578	2.0186	0.04353	0.37421	93.6328	189.88	125.664	198.215	292.198	173.202	Up
Klrc1	127.45	1.12248	0.31509	3.56238	0.00037	0.01496	57.1653	91.9731	91.569	180.845	211.967	131.183	Up
Klra6	8.03598	2.36159	1.13908	2.07325	0.03815	0.34953	0	3.95583	3.89655	4.0869	17.829	18.4476	Up
Klra2	377.758	1.36733	0.33203	4.1181	3.82E-05	0.00254	163.611	262.074	207.492	582.383	724.056	326.932	Up
Magohb	524.046	-0.6341	0.28883	-2.1953	0.02814	0.28977	801.3	668.536	442.259	496.559	302.103	433.518	Down
Apold1	139.948	-0.9827	0.3987	-2.4647	0.01371	0.18549	101.518	266.03	189.957	105.238	69.3351	107.611	Down
Gprc5a	38965.7	0.52632	0.19061	2.76119	0.00576	0.10589	28272.2	27889.6	39645.5	51534.8	41072.1	45380	Up
Gm6728	43.9194	-2.3299	0.9055	-2.5731	0.01008	0.15228	174.453	34.6135	10.7155	19.4128	7.92401	16.3979	Down
Plbd1	3041.44	0.39949	0.20263	1.97156	0.04866	0.39489	2774.49	2858.09	2236.62	2955.85	4327.5	3096.12	Up
Gucy2c	105.105	0.8836	0.39667	2.22755	0.02591	0.27838	50.266	57.3596	113.974	146.107	102.022	160.904	Up
BC049715	72.2088	-1.8477	0.54538	-3.3879	0.0007	0.02445	163.611	136.476	38.9655	26.5649	29.715	37.92	Down
Art4	1182.2	0.6692	0.2464	2.71588	0.00661	0.11532	646.559	871.272	1220.6	1557.11	1454.06	1343.6	Up
Arhgdib	192.397	0.43127	0.18973	2.27311	0.02302	0.25972	148.827	185.924	156.836	214.562	225.834	222.396	Up
Ptpro	369.904	0.60214	0.2868	2.09949	0.03577	0.33545	286.812	358.992	235.742	343.3	607.177	387.399	Up
Pik3c2g	194.637	0.93742	0.44086	2.12632	0.03348	0.32299	82.7911	149.333	168.526	219.671	407.096	140.407	Up
Slco1a4	7.91836	-1.8801	0.83204	-2.2597	0.02384	0.26511	8.87048	14.8344	13.6379	4.0869	1.981	4.09946	Down
Gys2	24.3716	-4.2942	0.96214	-4.4632	8.08E-06	0.00073	25.6258	98.8958	14.6121	3.06518	1.981	2.04973	Down
Abcc9	924.877	-0.4694	0.22073	-2.1265	0.03346	0.32299	925.487	1343.99	952.708	745.86	920.175	661.038	Down
Gm15707	7.74674	-2.0332	0.91544	-2.221	0.02635	0.28075	12.8129	11.8675	12.6638	6.13035	1.981	1.02487	Down
Lmntd1	404.974	-0.632	0.19993	-3.1612	0.00157	0.04279	423.812	589.419	463.69	343.3	329.837	279.788	Down
Tuba3b	5.10404	-3.193	1.47762	-2.1609	0.0307	0.30647	2.95683	20.7681	3.89655	1.02173	1.981	0	Down
Gm15762	22.6034	-2.6943	1.22616	-2.1973	0.028	0.28884	2.95683	36.5914	77.9311	10.2173	7.92401	0	Down
Far2	96.3928	0.54894	0.25708	2.13533	0.03273	0.31785	70.9638	74.1719	89.6207	127.716	99.0501	116.835	Up
3300002P	2.03618	4.49249	2.06097	2.1798	0.02927	NA	0	0	0	4.0869	1.981	6.1492	Up
Pirb	1347.74	0.72453	0.34848	2.07909	0.03761	0.34589	1240.88	1104.67	703.328	1596.96	2447.53	993.095	Up
Gm15448	34.4077	1.12464	0.43938	2.55957	0.01048	0.15619	29.5683	19.7792	15.5862	36.7821	53.487	51.2433	Up
Pira2	16.9352	1.80376	0.89378	2.01812	0.04358	0.37434	11.8273	6.92271	3.89655	13.2824	56.4585	9.22379	Up
Lair1	542.317	1.12801	0.42131	2.67736	0.00742	0.12358	486.891	313.5	221.129	648.796	1176.72	406.872	Up
Lilra5	617.035	1.22715	0.4757	2.57968	0.00989	0.15061	534.2	338.224	235.742	698.86	1500.61	394.573	Up
Gm15931	120.955	1.45593	0.64937	2.24205	0.02496	0.27329	107.431	50.4369	36.0431	127.716	345.685	58.4174	Up
Rdh13	1104.66	-0.468	0.15999	-2.9251	0.00344	0.07458	1139.36	1384.54	1322.88	823.511	910.27	1047.41	Down
Gm15494	146.367	2.51712	0.38624	6.517	7.17E-11	2.87E-08	40.41	38.5694	51.6293	177.78	384.314	185.501	Up
Tnnt1	155.857	-2.1657	0.36696	-5.9019	3.59E-09	1.08E-06	152.769	333.279	278.604	56.1949	43.582	70.7157	Down
Tnni3	5257.57	-3.2666	0.45123	-7.2393	4.51E-13	2.50E-10	5762.85	16382.1	6431.26	555.819	1481.79	931.603	Down
Ube2s	2856.53	0.24599	0.12171	2.02117	0.04326	0.37347	2619.75	2697.88	2523.02	3160.2	2794.2	3344.14	Up
Shisa7	181.034	1.00025	0.26318	3.80068	0.00014	0.0072	146.856	121.642	93.5173	258.497	195.129	270.565	Up
Isoc2b	372.418	1.22756	0.36588	3.35504	0.00079	0.02644	329.193	229.438	110.078	497.58	493.269	574.95	Up
Isoc2a	1618.52	0.25347	0.12074	2.09925	0.03579	0.3355	1588.8	1485.42	1356	1705.26	1835.4	1740.22	Up
Sbk2	273.702	-3.4443	0.92316	-3.731	0.00019	0.00911	382.416	939.51	182.164	25.5431	93.1071	19.4725	Down
Sbk3	946.711	-4.0583	0.81155	-5.0007	5.71E-07	8.45E-05	1432.09	3045	881.595	72.5425	207.015	42.0195	Down
Zfp579	2582.15	-0.2302	0.10829	-2.1257	0.03353	0.32299	2864.18	2597	2901.96	2384.71	2454.46	2290.58	Down
Zim1	9.86878	-3.1395	1.09561	-2.8656	0.00416	0.08496	9.85609	32.6356	10.7155	2.04345	3.962	0	Down
Peg3	714.448	-0.4316	0.20326	-2.1233	0.03373	0.32401	651.487	924.676	885.492	535.384	698.303	591.348	Down
Zfp264	42.6957	-6.9703	1.13314	-6.1513	7.69E-10	2.63E-07	91.6616	50.4369	112.026	0	0	2.04973	Down
Zfp773	158.553	-0.5896	0.30064	-1.961	0.04988	0.39986	220.776	203.725	147.095	142.02	149.566	88.1385	Down
Gm45844	229.721	0.53908	0.182	2.962	0.00306	0.06875	187.266	165.156	209.44	262.583	282.293	271.589	Up
Zscan18	522.148	-0.3318	0.12814	-2.5893	0.00962	0.14749	573.624	600.298	571.819	485.32	444.735	457.09	Down
Lig1	808.774	0.33438	0.15981	2.09235	0.03641	0.33907	743.149	680.403	722.811	1047.27	863.717	795.296	Up
Sult2a2	28.5918	2.79464	0.74828	3.73475	0.00019	0.00902	8.87048	3.95583	8.76725	70.4991	13.867	65.5914	Up
Sult2a1	254.335	5.1641	1.78948	2.88581	0.0039	0.0813	37.4531	3.95583	0	724.403	66.3636	693.834	Up
Dhx34	1122.19	-0.2231	0.10442	-2.1362	0.03266	0.31743	1170.9	1210.48	1244.95	1031.94	1047.95	1026.92	Down
C5ar2	122.234	0.97122	0.33806	2.8729	0.00407	0.08354	94.6184	81.0946	72.0863	144.063	228.806	112.735	Up
Dact3	833.22	-0.3636	0.15356	-2.3681	0.01788	0.22242	1067.41	905.886	839.707	709.077	697.313	779.923	Down
Ptgir	333.842	0.77038	0.37726	2.04204	0.04115	0.36537	308.496	217.571	214.31	388.256	643.826	230.595	Up
Dmpk	3114.48	-0.9442	0.21419	-4.4084	1.04E-05	0.00086	3576.77	5403.67	3315.97	2035.28	2170.19	2185.01	Down
Gpr4	956.048	0.25733	0.11291	2.27903	0.02267	0.2572	849.595	863.36	900.104	1056.46	1074.69	992.07	Up
Ppp1r13l	651.566	-0.5612	0.22515	-2.4926	0.01268	0.17568	792.429	807.979	729.63	504.732	397.191	677.436	Down
Ckm	1376.76	-4.7558	1.48857	-3.1949	0.0014	0.0396	1275.38	5330.48	1359.9	13.2824	275.359	6.1492	Down
Bcam	32450.7	0.2852	0.14018	2.03454	0.0419	0.36989	32964.7	25655.6	29140.4	38010.2	33712.7	35220.5	Up
Gm26600	18.6593	-1.1148	0.54382	-2.05	0.04037	0.36179	17.741	27.6908	31.1724	12.2607	13.867	9.22379	Down
Cblc	706.428	0.35519	0.13095	2.71243	0.00668	0.11604	606.149	642.823	610.785	724.403	819.144	835.266	Up

Gm16251	11.7772	-3.7026	1.04369	-3.5475	0.00039	0.0157	11.8273	15.8233	37.9914	0	2.9715	2.04973 Down
Nlrp4e	56.6817	-1.2308	0.60219	-2.0438	0.04097	0.36426	29.5683	91.9731	116.897	25.5431	21.791	54.3179 Down
Zfp61	747.989	-0.4428	0.12143	-3.6468	0.00027	0.0117	905.774	815.89	864.061	638.578	650.759	612.87 Down
Lypd5	22.2149	-3.0711	0.70654	-4.3466	1.38E-05	0.00108	43.3668	49.4479	26.3017	1.02173	4.9525	8.19893 Down
Cadm4	526.076	-0.4301	0.21879	-1.9659	0.04931	0.39793	538.142	743.696	529.931	530.275	423.934	390.474 Down
Zfp575	9.21456	-2.5474	0.90052	-2.8288	0.00467	0.09198	9.85609	23.735	13.6379	1.02173	3.962	3.0746 Down
Tgfb1	3780.54	0.44694	0.19419	2.30159	0.02136	0.24952	3447.66	3022.26	3128.93	4484.35	5246.68	3353.36 Up
Axl	5879.6	0.52512	0.25098	2.0923	0.03641	0.33907	5480.97	4763.81	4218.99	6932.41	9109.64	4771.78 Up
Cyp2a21-	38.905	-1.4396	0.57444	-2.5061	0.01221	0.17151	60.1221	88.0173	22.4052	23.4997	16.8385	22.547 Down
Pld3	5724.09	0.50894	0.2475	2.05635	0.03975	0.35945	5752.01	4490.86	3931.62	6386.81	8749.09	5034.14 Up
9530053A	583.414	-0.707	0.33339	-2.1208	0.03394	0.32502	686.969	849.515	634.164	380.082	274.369	675.387 Down
Dll3	6.22382	-5.0624	1.54235	-3.2823	0.00103	0.03195	4.92804	16.8123	14.6121	0	0.9905	0 Down
Acp7	40.9589	-2.0885	0.72257	-2.8904	0.00385	0.08043	34.4963	117.686	46.7587	7.15208	8.91451	30.746 Down
Rinl	1147.03	0.48705	0.19572	2.48857	0.01283	0.17723	786.516	1003.79	1075.45	1299.63	1568.95	1147.85 Up
Lgals4	1059.92	-0.4637	0.16412	-2.8253	0.00472	0.09241	1144.29	1334.1	1207.93	794.902	835.983	1042.29 Down
Ryr1	133.034	0.66545	0.27675	2.40452	0.01619	0.20878	111.374	103.841	93.5173	164.498	204.043	120.934 Up
C230062I1	456.647	0.33991	0.17328	1.96161	0.04981	0.39986	417.898	345.146	446.155	553.775	466.526	510.383 Up
Zfp382	60.889	0.64474	0.29655	2.17416	0.02969	0.30007	51.2517	43.5142	47.7328	85.8249	68.3446	68.666 Up
Cox7a1	189.065	-3.0299	0.52881	-5.7297	1.01E-08	2.68E-06	209.935	589.419	211.388	23.4997	67.3541	32.7957 Down
Tyrobp	1827.75	0.82674	0.31988	2.58455	0.00975	0.14899	1774.1	1276.74	903.026	2048.56	3260.73	1703.33 Up
Hspb6	1327.26	-1.6736	0.38725	-4.3217	1.55E-05	0.00119	1175.83	3503.88	1383.28	624.274	669.579	606.721 Down
2200002J2	1.79962	-4.273	2.11239	-2.0228	0.04309	NA	1.97122	3.95583	4.87069	0	0	0 Down
D7Ertd128	18.2419	3.04888	0.75194	4.0547	5.02E-05	0.00317	6.89926	2.96687	1.94828	29.63	48.5345	19.4725 Up
Mag	121.686	0.84122	0.26314	3.19682	0.00139	0.03943	91.6616	95.9289	74.0345	145.085	190.176	133.233 Up
Fxyd5	4660.07	0.54721	0.10785	5.0736	3.90E-07	6.02E-05	3878.37	3674.97	3806.93	5693.05	5806.32	5100.76 Up
Fxyd1	2529.53	-0.5082	0.14361	-3.5387	0.0004	0.01608	2566.53	3339.71	3005.22	2094.54	2008.74	2162.47 Down
Fxyd3	174.473	-0.7589	0.21362	-3.5525	0.00038	0.01549	219.791	249.217	188.983	121.585	124.803	142.456 Down
Kctd15	944.179	-0.5142	0.12367	-4.1578	3.21E-05	0.00222	1182.73	1132.36	1017	776.511	776.553	779.923 Down
Cebpa	7108.57	0.45774	0.18199	2.51523	0.0119	0.16878	7314.2	5290.93	5365.56	7788.61	7642.7	9249.41 Up
Rpl17-ps9	77.3818	0.57043	0.28363	2.01118	0.04431	0.37788	53.2229	76.1498	57.4742	101.151	88.1546	88.1385 Up
Plekhf1	521.853	-0.3806	0.17169	-2.217	0.02662	0.28184	544.056	683.37	543.569	414.82	491.288	454.016 Down
Zfp619	256.877	-0.4821	0.18899	-2.551	0.01074	0.15802	270.057	318.445	309.776	223.758	234.749	184.476 Down
Gm37494	3059.97	-0.5211	0.14009	-3.7195	0.0002	0.00939	3433.86	3386.19	3999.81	2332.6	2421.77	2785.59 Down
4930435C	1.85287	4.35756	2.20802	1.97351	0.04844	NA	0	0	0	6.13035	3.962	1.02487 Up
Vstm2b	62.0662	-1.3527	0.44873	-3.0145	0.00257	0.06015	81.8055	107.796	77.9311	36.7821	16.8385	51.2433 Down
Zfp936	4.3967	4.58731	1.69556	2.70549	0.00682	0.11755	0	0	0.97414	3.06518	5.94301	16.3979 Up
Siglecf	488.725	1.05681	0.40892	2.58436	0.00976	0.14899	509.56	272.952	169.5	727.468	813.201	439.667 Up
Nkg7	554.176	1.02482	0.23201	4.41712	1.00E-05	0.00084	320.323	456.899	318.543	753.012	872.631	603.646 Up
Etfbl	118.043	-0.8443	0.42055	-2.0076	0.04469	0.37936	146.856	80.1056	227.948	80.7163	104.993	67.6411 Down
Siglece	287.39	0.81934	0.37022	2.2131	0.02689	0.28339	261.186	210.648	151.966	352.495	534.87	213.172 Up
Klk8	621.474	0.61935	0.24037	2.5767	0.00997	0.15148	515.473	593.375	361.405	807.163	614.111	837.315 Up
Klk1b11	3.53898	4.2612	1.65942	2.56788	0.01023	0.15345	0	0	0.97414	10.2173	5.94301	4.09946 Up
Klk1b22	450.898	12.2843	1.22123	10.0589	8.39E-24	1.17E-20	0	0	0	1189.29	936.023	580.074 Up
Klk1	61.6705	-1.0949	0.50041	-2.188	0.02867	0.29286	128.129	81.0946	42.8621	37.8038	53.487	26.6465 Down
Shank1	80.6875	-1.2322	0.40253	-3.061	0.00221	0.05407	99.5465	91.9731	148.069	64.3687	52.4965	27.6714 Down
5430431A	121.223	-1.7899	0.70187	-2.5502	0.01076	0.15803	89.6904	398.55	75.9828	20.4345	82.2116	60.4671 Down
Atf5	3640.62	-0.4172	0.12046	-3.4638	0.00053	0.01997	4291.34	4332.63	3866.36	3364.54	3085.41	2903.44 Down
Il4i1	85.2213	-1.2126	0.36719	-3.3024	0.00096	0.03049	112.359	83.0725	161.707	48.0211	41.601	64.5665 Down
Pnkp	1692.8	0.2851	0.1259	2.26455	0.02354	0.26285	1523.75	1663.43	1391.07	1862.61	1777.95	1938.02 Up
Cpt1c	466.388	-0.6484	0.22266	-2.9119	0.00359	0.07604	598.264	430.197	679.949	325.93	400.162	363.827 Down
Gm15545	354.884	-1.2916	0.38857	-3.3241	0.00089	0.02894	523.358	235.372	753.009	193.106	202.062	222.396 Down
Rps11	76.1125	-1.1634	0.50355	-2.3104	0.02087	0.24598	115.316	44.5031	155.862	62.3252	36.6485	42.0195 Down
Rpl13a	759.647	0.34937	0.16481	2.11985	0.03402	0.32506	675.142	607.22	721.837	900.14	726.037	927.504 Up
Slc17a7	406.423	-5.6495	1.85519	-3.0452	0.00232	0.05622	390.301	1673.32	327.311	2.04345	45.563	0 Down
Hrc	789.778	-4.5927	1.57983	-2.9071	0.00365	0.07708	951.112	2832.38	766.647	2.04345	178.29	8.19893 Down
Snrnp70	9908.68	-0.3098	0.11385	-2.7214	0.0065	0.11421	10509.5	10537.3	11859.2	8564.1	9433.53	8548.41 Down
Fut1	14.8108	2.21927	0.84176	2.63646	0.00838	0.13431	3.94243	5.93375	5.84483	18.3911	46.5535	8.19893 Up
Kcnj14	26.6252	-3.1445	0.60683	-5.1819	2.20E-07	3.76E-05	42.3812	51.4258	49.6811	10.2173	2.9715	3.0746 Down
Kcnj11	215.794	-2.8764	1.37045	-2.0989	0.03583	0.33565	168.539	835.67	135.405	7.15208	135.699	12.2984 Down
Abcc8	220.493	-2.2775	1.03794	-2.1943	0.02822	0.28987	190.222	756.553	150.017	19.4128	155.509	51.2433 Down
Saa3	221.392	2.35556	0.45856	5.13682	2.79E-07	4.44E-05	97.5753	47.47	72.0863	469.994	482.374	158.854 Up
Mrgprb1	2.63364	-4.8217	2.20803	-2.1837	0.02898	0.29496	1.97122	12.8565	0.97414	0	0	0 Down
Csrp3	2416.88	-4.1041	1.46857	-2.7946	0.0052	0.0986	2327.02	8675.14	2702.26	18.3911	750.8	27.6714 Down
E2f8	96.6562	0.91963	0.27944	3.29102	0.001	0.03138	68.9926	70.216	61.3707	151.215	129.756	98.3871 Up
Nell1	22.7865	-1.8168	0.83642	-2.1721	0.02985	0.30087	14.7841	76.1498	15.5862	10.2173	14.8575	5.12433 Down
Svip	1362.23	-0.3757	0.14626	-2.5686	0.01021	0.15335	1716.93	1553.65	1345.29	1115.72	1194.54	1247.26 Down
Tubgcp5	1652.91	0.27945	0.13784	2.02738	0.04262	0.37265	1528.68	1606.07	1345.29	1954.56	1821.53	1661.31 Up
Gm34121	65.0844	-0.9738	0.38396	-2.5363	0.0112	0.1626	79.8343	107.796	71.1121	30.6518	39.62	61.492 Down
A230057D	953.639	-0.844	0.36876	-2.2888	0.02209	0.25362	1581.9	1252.02	840.682	757.098	359.552	930.578 Down
A330076H	265.681	-0.8679	0.37276	-2.3284	0.01989	0.23828	474.078	286.798	268.862	185.954	111.927	266.465 Down
Gm32633	260.952	-1.0537	0.51808	-2.0339	0.04196	0.37014	427.754	302.621	326.336	45.9776	212.958	250.067 Down
Gm45212	3.94828	-2.7664	1.30958	-2.1124	0.03465	0.32919	6.89926	7.91166	5.84483	2.04345	0.9905	0 Down
Cers3	72.2583	0.89764	0.35196	2.55041	0.01076	0.15803	41.3956	60.3264	49.6811	83.7815	72.3066	126.059 Up
Fam169b	292.78	0.72873	0.21835	3.33738	0.00085	0.02793	254.287	240.317	166.578	345.343	385.305	364.852 Up
Fanci	35.8301	1.00012	0.43733	2.28686	0.0222	0.25464	25.6258	16.8123	29.2242	49.0428	59.4301	34.8454 Up
Ticrr	29.3537	1.49055	0.53532	2.78439	0.00536	0.10097	15.7697	19.7792	10.7155	37.8038	64.3826	27.6714 Up
Plin1	546.07	-6.5244	0.93699	-6.9631	3.33E-12	1.56E-09	532.229	2270.65	438.362	12.2607	18.8195	4.09946 Down
Anpep	4973.87	0.34782	0.17571	1.97953	0.04776	0.39169	4675.73	4452.29	4003.71	4986.02	6793.85	4931.65 Up
Iqgap1	24677.4	0.29152	0.13487	2.16151	0.03066	0.3062	25126.1	20304.3	21147.6	27325	28726.5	25435.1 Up
Alpk3	205.881	-1.9803	0.51547	-3.8416	0.00012	0.00631	224.719	589.419	171.448	78.6729	121.832	49.1936 Down
2900076A	568.619	-0.4473	0.21388	-2.0914	0.03649	0.33955	559.826	834.681	573.768	504.732	504.165	434.543 Down
Gm37829	87.817	-1.5706	0.51955	-3.0229	0.0025	0.05921	87.7192	234.383	72.0863	32.6952	41.601	58.4174 Down
Fsd2	577.822	-1.202	0.47813	-2.5139	0.01194	0.1691	443.524	1451.79	521.164	284.04	241.682	524.731 Down

Adamtsl3	720.855	-0.5925	0.17639	-3.3592	0.00078	0.02626	823.969	960.278	816.328	645.73	601.234	477.587	Down
Folh1	58.9685	-1.084	0.45997	-2.3567	0.01844	0.22623	81.8055	118.675	39.9397	39.8473	36.6485	36.8952	Down
Ctsc	16144.9	0.58072	0.22963	2.52893	0.01144	0.16462	17349.7	9515.75	11950.7	19897.1	18897.8	19258.3	Up
Rps13-ps2	171.281	3.65306	0.27369	13.3475	1.22E-40	2.94E-37	24.6402	23.735	27.2759	314.691	269.416	367.927	Up
Prss23	4680.69	0.48274	0.19277	2.5042	0.01227	0.17177	3881.33	4367.24	3465.98	5994.46	6133.18	4241.92	Up
Me3	488.128	-0.9667	0.3758	-2.5723	0.0101	0.15245	479.006	1052.25	406.216	359.647	374.409	257.241	Down
Sytl2	1577.28	-0.5055	0.22654	-2.2315	0.02565	0.27683	1741.57	2077.8	1732.99	1203.59	1004.37	1703.33	Down
Prcp	2889.72	0.45855	0.14378	3.1893	0.00143	0.04	2645.37	2295.37	2362.29	3458.54	3657.92	2918.82	Up
C230038L	9.56923	-1.6074	0.75814	-2.1201	0.03399	0.32506	8.87048	16.8123	17.5345	4.0869	3.962	6.1492	Down
Kctd21	1059.99	0.40379	0.15614	2.58602	0.00971	0.14849	968.853	774.354	994.595	1118.79	1252.98	1250.34	Up
Thrsp	1049.18	-3.385	0.74223	-4.5606	5.10E-06	0.00052	1175.83	3549.37	1019.92	315.713	131.737	102.487	Down
Pak1	1610.57	0.21067	0.10742	1.96112	0.04986	0.39986	1496.15	1476.51	1506.99	1638.85	1834.41	1710.5	Up
Myo7a	1490.23	0.97081	0.29664	3.27263	0.00107	0.0325	1336.49	918.742	765.673	1646	2723.88	1550.62	Up
Lrrc32	4169.88	0.36042	0.15513	2.32336	0.02016	0.24026	3241.67	3392.13	4321.28	4455.74	4804.92	4803.55	Up
Uvrag	3149.59	0.23886	0.11809	2.02276	0.0431	0.37332	3171.69	2707.77	2788.96	3446.28	3506.37	3276.5	Up
Dgat2	1783.27	-1.1868	0.40159	-2.9553	0.00312	0.06999	2264.93	4054.73	1114.41	904.227	1180.68	1180.65	Down
Serpinh1	13429.1	0.44225	0.14988	2.95076	0.00317	0.07072	10808.2	9941.01	13411	15547.6	15368.6	15498	Up
Gm10605	82.3505	-0.8454	0.38905	-2.1729	0.02979	0.30054	130.1	88.0173	99.3621	71.5208	72.3066	32.7957	Down
Chrdl2	31.1459	-2.3065	0.55471	-4.158	3.21E-05	0.00222	41.3956	69.2271	44.8104	17.3693	7.92401	6.1492	Down
Gm39059	6.90542	-2.0489	1.02421	-2.0005	0.04545	0.38155	9.85609	9.88958	13.6379	4.0869	3.962	0	Down
Fchsd2	449.585	0.54518	0.17111	3.18605	0.00144	0.04024	419.869	330.312	346.793	515.971	583.405	501.159	Up
Art2a	44.0761	1.56196	0.62112	2.51477	0.01191	0.16878	27.597	25.7129	13.6379	41.8907	122.822	32.7957	Up
Inppl1	4024.2	-0.2575	0.10569	-2.4362	0.01484	0.19554	4491.42	4585.8	4069.95	3582.17	3635.14	3780.73	Down
Folr2	121.818	-1.1213	0.28145	-3.984	6.78E-05	0.00397	147.841	207.681	145.147	78.6729	62.4016	89.1633	Down
Il18bp	616.414	0.52789	0.23304	2.26522	0.0235	0.2628	516.459	507.335	490.966	689.665	950.881	543.179	Up
Art5	45.4571	-1.5146	0.55075	-2.7501	0.00596	0.10785	66.0358	106.807	29.2242	22.478	28.7245	19.4725	Down
Pgap2	2434.07	0.43272	0.17953	2.4102	0.01594	0.20568	2300.41	1998.68	1916.13	2384.71	2634.73	3369.76	Up
Rhog	4422.99	0.3967	0.1215	3.26499	0.00109	0.0332	3728.56	3623.54	4104.05	4954.35	5442.8	4684.66	Up
Trim34a	610.265	2.03868	0.95999	2.12364	0.0337	0.32401	557.855	72.1939	86.6983	648.796	1466.93	829.116	Up
Trim5	441.377	2.40153	1.02991	2.3318	0.01971	0.23657	345.949	33.6246	41.888	599.753	987.529	639.516	Up
Trim30a	2365.37	0.62981	0.21847	2.8828	0.00394	0.082	2106.25	1835.51	1629.73	2668.75	3674.76	2277.25	Up
Gm5900	129.372	-3.883	0.54353	-7.144	9.07E-13	4.89E-10	165.582	284.82	276.655	4.0869	29.715	15.373	Down
Dchs1	655.807	0.44934	0.18757	2.39564	0.01659	0.21213	648.531	447.009	567.923	816.359	746.838	708.182	Up
Gm4070	259.664	0.82398	0.40105	2.05455	0.03992	0.35982	186.28	206.692	169.5	243.171	558.642	193.7	Up
Gvin1	125.746	1.96887	0.6009	3.27655	0.00105	0.03233	72.935	49.4479	31.1724	82.7598	396.2	121.959	Up
Gm4759	50.3309	-1.8883	0.93712	-2.015	0.04391	0.3764	64.0646	83.0725	90.5949	57.2166	3.962	3.0746	Down
Gm8995	1941.59	1.13256	0.29516	3.83707	0.00012	0.0064	1553.32	1136.31	959.526	2162.99	3788.67	2048.71	Up
Olfml1	770.309	-0.5301	0.18165	-2.9181	0.00352	0.07506	851.566	977.091	902.052	764.251	574.49	552.403	Down
Cyb5r2	35.0552	-2.0465	0.85645	-2.3895	0.01687	0.21417	35.4819	95.9289	37.9914	8.1738	30.7055	2.04973	Down
Lyve1	18494.8	0.64099	0.27464	2.33394	0.0196	0.236	14804.8	12197.8	16354.8	28618.5	25027	13965.8	Up
2310014F(	418.811	-0.5752	0.16468	-3.4926	0.00048	0.01831	478.02	507.335	518.242	384.169	326.865	298.236	Down
Arntl	1044.29	-1.2301	0.47178	-2.6073	0.00913	0.14228	439.581	1977.92	1975.55	529.254	713.161	630.293	Down
Rras2	5002.09	-0.4674	0.16849	-2.7743	0.00553	0.10361	4759.5	6162.2	6494.58	4318.83	4501.83	3775.61	Down
Calca	177.274	-1.5546	0.23873	-6.512	7.42E-11	2.91E-08	305.539	258.118	229.897	103.194	93.1071	73.7903	Down
Sox6	550.441	-0.6469	0.18724	-3.455	0.00055	0.02049	642.617	728.862	643.906	388.256	378.371	520.632	Down
Plekha7	952.443	-0.9835	0.16358	-6.0123	1.83E-09	6.02E-07	1268.48	1418.17	1108.57	578.297	631.94	709.207	Down
Rps13	9965.86	0.66386	0.20602	3.22237	0.00127	0.03687	7590.17	6771.4	8776.01	12802.2	9317.64	14537.7	Up
Gm44777	55.3814	0.7686	0.33048	2.32572	0.02003	0.23902	37.4531	48.4589	37.0173	79.6946	73.2971	56.3676	Up
Nucb2	2625.46	0.28359	0.12675	2.23736	0.02526	0.27483	2652.27	2257.79	2194.73	2794.42	2938.82	2914.72	Up
Xylt1	283.104	0.64892	0.24575	2.64061	0.00828	0.13297	221.762	223.505	216.259	382.125	411.058	243.918	Up
Arl6ip1	13877.3	0.79238	0.13534	5.85497	4.77E-09	1.34E-06	10200.1	8923.37	11354.6	17457.2	18505.5	16823.2	Up
Syt17	411.561	-1.6518	0.19543	-8.4522	2.86E-17	2.81E-14	680.07	650.734	542.595	182.889	234.749	178.327	Down
Gm44652	36.4105	-0.7942	0.39787	-1.9962	0.04592	0.38348	33.5107	55.3817	49.6811	25.5431	28.7245	25.6216	Down
Coq7	1412.27	-0.8904	0.23938	-3.7195	0.0002	0.00939	1693.28	2210.32	1600.51	934.879	760.705	1273.91	Down
Tmc7	420.469	-0.6981	0.32552	-2.1444	0.032	0.31324	404.1	682.381	474.405	262.583	461.573	237.769	Down
Tmc5	658.468	-1.0935	0.44812	-2.4401	0.01468	0.19381	852.552	1395.42	442.259	337.169	649.769	273.639	Down
Iqck	826.515	-0.9097	0.3395	-2.6796	0.00737	0.1232	1080.23	1123.46	1032.59	562.971	304.084	855.763	Down
Gprc5b	1928.02	-0.9004	0.27879	-3.2299	0.00124	0.03619	3560.02	2149.99	1822.61	1401.81	1099.46	1534.22	Down
Gpr139	1.68363	4.2195	2.11605	1.99405	0.04615	NA	0	0	0	3.06518	3.962	3.0746	Up
Acsm5	55.1725	-2.0332	0.50722	-4.0086	6.11E-05	0.00367	103.489	118.675	43.8362	24.5214	13.867	26.6465	Down
Gm39078	3.10636	-5.0606	2.01079	-2.5167	0.01185	0.1683	9.85609	0.98896	7.79311	0	0	0	Down
Acsm1	567.403	-0.8188	0.29576	-2.7686	0.00563	0.10469	660.358	870.283	641.957	516.993	251.587	463.239	Down
Rexo5	516.347	0.39741	0.16681	2.38248	0.0172	0.21691	478.02	449.976	409.138	604.861	648.778	507.309	Up
Abca15	10.6477	-6.8376	1.36599	-5.0056	5.57E-07	8.29E-05	20.6978	21.7571	21.431	0	0	0	Down
Cog7	3385.58	-0.259	0.12966	-1.9974	0.04578	0.38275	3387.54	4114.07	3564.37	3226.61	3107.2	2913.69	Down
Ubfd1	2768.41	0.26462	0.09549	2.77125	0.00558	0.10441	2455.15	2515.91	2574.65	2904.77	3085.41	3074.6	Up
Ndufab1	2176.4	-0.4147	0.18176	-2.2818	0.0225	0.25626	2105.26	2933.25	2422.68	1908.58	1621.45	2067.15	Down
Dctn5	737.005	0.71355	0.16394	4.35256	1.35E-05	0.00105	483.934	574.585	616.63	910.357	1005.36	831.166	Up
Ern2	22.4053	-3.4765	1.061	-3.2766	0.00105	0.03233	7.88487	100.874	14.6121	2.04345	5.94301	3.0746	Down
Kdm8	333.548	0.90388	0.18542	4.87485	1.09E-06	0.00014	261.186	220.538	215.285	486.341	381.343	436.593	Up
Il21r	384.452	0.77026	0.27262	2.82536	0.00472	0.09241	320.323	289.765	242.561	554.797	577.462	321.808	Up
1700123J1	12.7091	-2.5437	1.09868	-2.3152	0.0206	0.24365	15.7697	40.5473	8.76725	0	2.9715	8.19893	Down
Lat	885.189	0.69824	0.15646	4.46275	8.09E-06	0.00073	592.351	701.171	731.578	1109.59	985.548	1190.89	Up
Il27	50.4262	0.8075	0.39874	2.02513	0.04285	0.37291	30.5539	39.5583	39.9397	60.2818	88.1546	44.0692	Up
Nupr1	6718.82	-0.4424	0.21172	-2.0897	0.03665	0.3402	6471.51	8985.67	7765.83	5474.4	4588	7027.51	Down
Coro1a	6080.08	0.4907	0.18245	2.68952	0.00716	0.1211	5556.86	5150.49	4460.58	7102.01	8397.47	5813.04	Up
Gdpd3	1012.44	-4.023	0.16466	-24.431	#######	#######	2071.75	1748.48	1902.49	123.629	125.794	102.487	Down
Gm9967	12.7034	-1.5639	0.66244	-2.3609	0.01823	0.22473	15.7697	16.8123	24.3535	5.10863	4.9525	9.22379	Down
Sez6l2	105.706	-0.5347	0.23437	-2.2816	0.02251	0.25629	118.273	114.719	142.224	82.7598	89.1451	87.1136	Down
Kif22	149.078	0.8894	0.21565	4.12432	3.72E-05	0.00249	107.431	106.807	99.3621	218.649	194.138	168.078	Up
Qprt	59.6155	0.57903	0.29527	1.96105	0.04987	0.39986	48.2948	44.5031	50.6552	69.4773	81.2211	63.5417	Up
Spn	1203.53	0.70392	0.31998	2.19986	0.02782	0.28824	944.213	946.433	856.268	1146.38	2329.66	998.219	Up
Gm45184	25.7274	-1.8519	0.51624	-3.5872	0.00033	0.01399	32.5251	42.5252	45.7845	16.3476	6.93351	10.2487	Down

Stx1b	296.199	-1.1037	0.2998	-3.6815	0.00023	0.0105	346.934	507.335	358.483	163.476	140.651	260.316	Down
Pycard	357.673	0.71558	0.15988	4.4756	7.62E-06	0.0007	287.798	283.831	240.612	460.798	418.982	454.016	Up
Trim72	165.189	-3.4173	1.11707	-3.0592	0.00222	0.05418	161.64	616.121	128.586	8.1738	66.3636	10.2487	Down
Itgax	3106.33	1.51159	0.44278	3.41385	0.00064	0.02294	2525.13	1290.59	1023.82	4172.73	7320.79	2304.92	Up
9130023H	354.435	0.80412	0.1807	4.44992	8.59E-06	0.00075	244.431	263.063	266.914	383.147	456.621	512.433	Up
Rgs10	987.208	0.9871	0.15812	6.24277	4.30E-10	1.55E-07	733.293	601.287	651.699	1186.22	1476.84	1273.91	Up
Gm32816	3.69363	2.70425	1.25296	2.1583	0.0309	0.30734	0.98561	0.98896	0.97414	6.13035	6.93351	6.1492	Up
Gm40457	26.9769	-6.312	1.36858	-4.6121	3.99E-06	0.00041	27.597	116.697	15.5862	0	1.981	0	Down
Bag3	4736.34	-0.2461	0.11671	-2.1091	0.03494	0.33048	4793.02	5615.3	5009.99	4172.73	4524.61	4302.39	Down
Plpp4	14.1112	3.09557	0.99313	3.117	0.00183	0.04755	5.91365	2.96687	0	37.8038	27.734	10.2487	Up
Gm39094	1221.26	2.81358	0.17	16.5507	1.58E-61	5.69E-58	320.323	247.24	344.845	2006.67	2140.47	2268.03	Up
Fgfr2	12958.4	0.4091	0.16756	2.4415	0.01463	0.19354	11884.5	10762.8	10752.5	17806.6	12616	13927.9	Up
Mki67	760.728	1.47244	0.33495	4.39603	1.10E-05	0.0009	373.546	508.324	327.311	1466.18	1264.87	624.143	Up
Gm9347	24.3029	-5.1316	0.94259	-5.4442	5.20E-08	1.11E-05	69.9782	46.481	25.3276	0	1.981	2.04973	Down
Ebf3	14.546	-2.4865	1.11103	-2.238	0.02522	0.27466	9.85609	58.3485	5.84483	9.19553	1.981	2.04973	Down
Tcerg1l	44.2695	-1.1942	0.44868	-2.6616	0.00778	0.1277	45.338	81.0946	58.4483	16.3476	34.6675	29.7211	Down
Bnip3	658.672	-0.2341	0.11828	-1.9791	0.04781	0.39195	718.509	719.961	697.483	588.514	597.272	630.293	Down
Dpysl4	21.5338	-5.9812	1.31959	-4.5326	5.83E-06	0.00057	25.6258	84.0614	17.5345	0	1.981	0	Down
Stk32c	378.869	-1.1864	0.48722	-2.4351	0.01489	0.19589	379.459	562.717	637.087	429.125	165.414	99.412	Down
Lrrc27	479.072	-0.5372	0.16983	-3.1633	0.00156	0.04258	492.804	650.734	558.181	389.277	371.438	411.996	Down
Nkx6-2	54.8955	-1.697	0.46762	-3.6291	0.00028	0.01236	81.8055	97.9068	72.0863	11.239	37.639	28.6962	Down
Adgra1	14.2197	-2.0822	0.95711	-2.1755	0.02959	0.29945	15.7697	41.5362	11.6897	2.04345	1.981	12.2984	Down
Adam8	2789.01	-0.9154	0.46365	-1.9744	0.04833	0.39416	2468.95	6300.65	2166.48	1715.48	3000.23	1082.26	Down
Msx3	2.63005	-4.8197	1.9947	-2.4163	0.01568	0.20282	3.94243	9.88958	1.94828	0	0	0	Down
5830411N	97.5687	1.01823	0.46486	2.19043	0.02849	0.29175	81.8055	45.4921	66.2414	97.0639	216.92	77.8898	Up
Cox8b	106.759	-3.124	0.62825	-4.9725	6.61E-07	9.58E-05	108.417	362.948	103.259	10.2173	18.8195	36.8952	Down
Nlrp6	21.1998	1.15771	0.58904	1.96543	0.04937	0.39822	8.87048	21.7571	8.76725	28.6083	36.6485	22.547	Up
Ifitm3	16253.2	0.28086	0.10476	2.68086	0.00734	0.1232	13949.3	14197.5	15881.4	18106	17391.2	17993.6	Up
Gm45299	5.59128	-3.9539	1.42383	-2.777	0.00549	0.10304	8.87048	14.8344	7.79311	0	0	2.04973	Down
Gm45889	5.41996	-3.2979	1.47196	-2.2405	0.02506	0.27344	4.92804	17.8012	6.81897	0	2.9715	0	Down
Lsp1	3717.59	0.45019	0.19128	2.35357	0.01859	0.22746	3471.31	3224	2731.48	4254.46	5139.71	3484.54	Up
Tnfrsf26	244.88	0.95384	0.36556	2.60925	0.00907	0.14179	205.007	140.432	154.888	274.844	494.26	199.849	Up
Retn	180.679	-4.242	0.60079	-7.0606	1.66E-12	8.13E-10	151.784	600.298	277.629	6.13035	27.734	20.4973	Down
Mcemp1	967.102	1.15793	0.4321	2.67975	0.00737	0.1232	967.868	512.28	315.621	1297.59	1904.73	804.52	Up
Cd209d	60.8483	-1.7038	0.43581	-3.9095	9.25E-05	0.00507	70.9638	128.565	79.8794	29.63	38.6295	17.4227	Down
Cd209b	136.928	-1.2421	0.32799	-3.7872	0.00015	0.00751	133.057	271.963	172.423	82.7598	75.2781	86.0887	Down
Cd209f	294.277	-1.9866	0.37455	-5.3038	1.13E-07	2.08E-05	420.855	662.602	326.336	128.737	70.3256	156.804	Down
Cd209g	108.347	-1.9462	0.41628	-4.6753	2.94E-06	0.00032	153.755	258.118	104.233	43.9342	33.677	56.3676	Down
Shcbp1	76.3913	1.38442	0.44961	3.07915	0.00208	0.05192	37.4531	58.3485	31.1724	147.128	124.803	59.4422	Up
Gm5605	3.18084	5.1381	1.85083	2.77611	0.0055	0.10317	0	0	0	3.06518	10.8955	5.12433	Up
Myo16	66.7779	-1.1588	0.53846	-2.1521	0.03139	0.3102	38.4387	101.863	136.379	38.8256	26.7435	58.4174	Down
Rab20	741.253	0.32095	0.15863	2.02332	0.04304	0.37324	723.437	610.187	643.906	762.207	935.033	772.749	Up
F7	1248.09	1.46073	0.45693	3.19684	0.00139	0.03943	961.954	596.342	437.388	1889.17	2857.59	746.102	Up
F10	515.531	1.54799	0.46502	3.32886	0.00087	0.02862	303.567	299.654	185.086	787.75	1231.19	285.938	Up
Adprhl1	213.012	-3.8891	1.24244	-3.1302	0.00175	0.04609	148.827	865.338	183.138	4.0869	64.3826	12.2984	Down
Cfap97d2	443.826	-0.7837	0.30651	-2.5567	0.01057	0.15671	510.545	715.017	458.819	341.256	210.977	426.344	Down
Myom2	1046.01	-3.6423	1.19644	-3.0443	0.00233	0.05634	1428.15	3636.4	746.19	23.4997	381.343	60.4671	Down
Gm20796	42.8599	-1.5054	0.52122	-2.8883	0.00387	0.08082	58.1509	97.9068	34.0948	27.5866	15.848	23.5719	Down
Ckap2	194.03	1.75051	0.35143	4.98116	6.32E-07	9.22E-05	100.532	106.807	59.4225	398.473	300.122	198.824	Up
Slc20a2	2097.56	-0.5928	0.22001	-2.6944	0.00705	0.12008	2212.69	3326.85	2028.16	1531.57	1757.15	1728.95	Down
Ank1	89.0859	-2.4866	0.57797	-4.3022	1.69E-05	0.00128	93.6328	293.721	66.2414	23.4997	28.7245	28.6962	Down
Ido1	183.178	0.98661	0.4749	2.0775	0.03775	0.34709	94.6184	152.3	121.767	161.433	438.792	130.158	Up
Htra4	174.664	-0.725	0.26046	-2.7835	0.00538	0.10115	182.338	272.952	197.75	125.672	155.509	113.76	Down
Adrb3	1083.68	-0.6968	0.23882	-2.9178	0.00352	0.07506	1166.96	1493.33	1360.87	778.555	618.073	1084.31	Down
Gm26795	2.95588	-4.9884	1.97345	-2.5277	0.01148	0.16493	1.97122	11.8675	3.89655	0	0	0	Down
Nrg1	877.519	-0.532	0.23376	-2.2759	0.02285	0.25839	938.3	1025.55	1148.51	769.359	507.136	876.26	Down
Ppp1r3b	588.4	-0.5675	0.26789	-2.1185	0.03414	0.32547	725.408	869.294	513.371	356.582	564.585	501.159	Down
Gm19410	14.644	-3.3146	1.17237	-2.8273	0.00469	0.09231	7.88487	58.3485	13.6379	0	5.94301	2.04973	Down
Zdhhc2	440.253	0.44822	0.17787	2.52	0.01174	0.16717	427.754	375.804	313.673	534.362	475.44	514.483	Up
Gm45660	6.23471	-6.0652	1.52854	-3.968	7.25E-05	0.00416	15.7697	13.8454	7.79311	0	0	0	Down
Asah1	13316.2	0.25682	0.12713	2.0202	0.04336	0.37382	13293.9	11569.8	11538.7	15170.6	15150.7	13173.6	Up
F11	94.5395	-0.8855	0.41458	-2.136	0.03268	0.31749	99.5465	98.8958	169.5	88.8901	37.639	72.7655	Down
Sorbs2	1401.77	-1.491	0.44059	-3.3841	0.00071	0.02463	1135.42	3753.1	1315.09	592.601	641.845	972.598	Down
Snx25	8398.19	0.32216	0.14613	2.20456	0.02749	0.28689	8666.46	6576.57	7150.18	9596.04	9498.9	8900.96	Up
Slc25a4	14295.8	-0.9784	0.27642	-3.5395	0.0004	0.01606	15089.7	27961.8	13845.4	9228.22	9303.77	10346	Down
Enpp6	93.987	-1.132	0.57071	-1.9835	0.04731	0.38996	61.1077	248.228	77.9311	38.8256	74.2876	63.5417	Down
9330121K	3.38614	3.22206	1.56069	2.06452	0.03897	0.35477	0	0.98896	0.97414	8.1738	1.981	8.19893	Up
Hand2os1	95.532	-2.7291	0.62166	-4.39	1.13E-05	0.00092	146.856	237.35	113.974	10.2173	52.4965	12.2984	Down
5033428I2	3.61424	-3.2706	1.6174	-2.0222	0.04316	0.37341	2.95683	7.91166	8.76725	0	0	2.04973	Down
Hmgb2	2442.76	0.39053	0.12603	3.09884	0.00194	0.04965	2180.17	2136.15	2026.21	2924.18	2921.98	2467.88	Up
Galntl6	110.383	-1.7844	0.71333	-2.5016	0.01236	0.17237	69.9782	395.583	47.7328	56.1949	42.5915	50.2184	Down
BC030500	28.8643	-1.7217	0.85823	-2.0061	0.04484	0.38016	16.7553	81.0946	35.069	2.04345	20.8005	17.4227	Down
Palld	5927.38	-0.5591	0.22897	-2.4416	0.01462	0.19354	5059.13	8455.59	7670.37	5195.47	5172.4	4011.33	Down
Tll1	244.456	0.74659	0.28844	2.5884	0.00964	0.14778	242.46	154.277	150.991	389.277	269.416	260.316	Up
Cpe	2538.25	-0.9334	0.34577	-2.6996	0.00694	0.11869	2793.22	5249.39	1953.15	1679.72	2073.12	1480.93	Down
Psd3	3344.28	-0.2764	0.13718	-2.0151	0.04389	0.3764	3292.92	4064.62	3633.54	3278.72	2916.03	2879.87	Down
Csgalnact1	1608.13	0.32879	0.13312	2.46982	0.01352	0.18348	1293.12	1440.91	1543.04	1741.02	1932.47	1698.2	Up
Cilp2	145.684	-3.6267	0.64009	-5.6658	1.46E-08	3.72E-06	148.827	544.916	114.948	11.239	33.677	20.4973	Down
Ncan	49.2002	-1.9079	0.60078	-3.1757	0.00149	0.04128	84.7624	78.1277	70.138	11.239	8.91451	42.0195	Down
Slc25a42	955.134	-0.3663	0.12143	-3.0165	0.00256	0.06003	1157.1	1047.31	1022.85	854.162	845.888	803.495	Down
Ifi30	4501.4	0.77096	0.19578	3.93782	8.22E-05	0.00461	4025.23	3079.62	2874.68	5774.79	6497.69	4756.4	Up
Kcnn1	122.708	-0.7914	0.3681	-2.1499	0.03156	0.31095	220.776	154.277	91.569	88.8901	78.2496	102.487	Down
Plvap	13931.7	0.52368	0.21055	2.48712	0.01288	0.17759	12363.5	9204.23	12724.2	18116.2	18443.1	12739.1	Up

Bst2	4514.29	0.36914	0.0937	3.93943	8.17E-05	0.00459	3861.61	3851.99	4105.99	4953.32	5093.16	5219.64	Up
Fam129c	280.021	-0.5029	0.21211	-2.3708	0.01775	0.22094	309.481	282.842	392.578	256.453	203.053	235.719	Down
Gm5373	21.4134	2.3059	0.63747	3.61725	0.0003	0.01279	9.85609	4.94479	6.81897	40.869	47.544	18.4476	Up
Olfr373	7.72431	-3.3783	1.04333	-3.238	0.0012	0.03542	14.7841	12.8565	14.6121	2.04345	0	2.04973	Down
Rab8a	4415.26	0.22659	0.10207	2.22004	0.02642	0.28101	4349.49	3892.54	3965.72	4656	4838.6	4789.2	Up
Fam32a	6084.92	0.24581	0.11416	2.1531	0.03131	0.30974	5738.21	5211.81	5753.26	6314.26	6361.99	7129.99	Up
Tmem38a	1843.82	-1.3171	0.2849	-4.623	3.78E-06	0.0004	2199.88	3823.31	1871.32	988.008	959.795	1220.62	Down
Nwd1	646.296	-1.0162	0.344	-2.954	0.00314	0.07019	764.832	1340.04	489.992	430.146	440.773	411.996	Down
Mcm5	699.908	0.77346	0.21416	3.61165	0.0003	0.01301	587.423	528.104	434.466	1088.14	814.192	747.127	Up
4933431K	107.074	-0.8416	0.40672	-2.0692	0.03853	0.35193	107.431	199.77	105.207	85.8249	97.0691	47.1438	Down
Rnf150	1464.75	-0.2051	0.10135	-2.0242	0.04294	0.3732	1573.03	1582.33	1550.83	1401.81	1374.82	1305.68	Down
Gm10644	15.0775	1.67317	0.66124	2.53034	0.0114	0.16459	3.94243	6.92271	10.7155	16.3476	21.791	30.746	Up
Asf1b	196.383	0.86841	0.29073	2.98699	0.00282	0.06412	168.539	138.454	110.078	271.779	308.046	181.401	Up
Il27ra	591.774	0.44325	0.20641	2.14744	0.03176	0.31185	531.243	481.623	491.94	746.881	784.477	514.483	Up
C330011M	99.2823	-0.6697	0.24331	-2.7524	0.00592	0.10726	137.985	116.697	111.052	79.6946	70.3256	79.9395	Down
Mir23a	16.8624	-1.2238	0.61148	-2.0014	0.04534	0.38132	18.7266	33.6246	18.5086	6.13035	11.886	12.2984	Down
Mylk3	987.069	-3.6055	0.99952	-3.6073	0.00031	0.0131	1108.81	3503.88	860.164	49.0428	330.827	69.6909	Down
Gm45424	31.7486	-1.1558	0.58079	-1.99	0.04659	0.3865	35.4819	31.6467	64.2931	20.4345	8.91451	29.7211	Down
Rps13-ps1	2169.01	-5.1992	0.22774	-22.83	#######	#######	4096.19	4189.23	4383.62	96.0422	90.1356	158.854	Down
Adcy7	4504.11	0.44044	0.15944	2.76235	0.00574	0.10587	4271.63	3583	3611.13	5224.08	5875.65	4459.19	Up
Snx20	565.987	0.78921	0.28748	2.74531	0.00605	0.1087	480.977	401.517	362.38	703.969	975.643	471.438	Up
Gm36243	40.3861	-1.2367	0.48093	-2.5714	0.01013	0.15262	54.2085	60.3264	55.5259	20.4345	12.8765	38.9449	Down
Slc6a2	9783.16	1.1394	0.28279	4.02915	5.60E-05	0.00344	7451.2	5358.17	5517.52	14741.5	17259.5	8371.1	Up
Mt3	23.7249	-3.2832	1.06373	-3.0865	0.00203	0.0513	40.41	65.2712	23.3793	0	0.9905	12.2984	Down
Mt2	185.696	-0.8781	0.23084	-3.804	0.00014	0.00712	246.402	238.339	236.716	152.237	99.0501	141.431	Down
Mt1	2171.69	-0.6156	0.23452	-2.625	0.00867	0.1375	2632.56	2867.98	2383.72	1779.85	1199.5	2166.57	Down
Herpud1	17677.8	0.3039	0.12887	2.35816	0.01837	0.22573	17395	15850	14223.4	18563.7	19127.6	20907.3	Up
Nlrc5	1733.34	0.77675	0.34388	2.25879	0.0239	0.26545	1292.13	1395.42	1145.59	1940.26	3414.26	1212.42	Up
Cpne2	940.538	0.43295	0.16553	2.61544	0.00891	0.14026	782.573	840.614	778.337	1054.42	1263.88	923.404	Up
Ccl22	475.109	1.21947	0.44151	2.76206	0.00574	0.10587	323.28	148.344	384.785	753.012	912.251	328.982	Up
Cx3cl1	2447.54	0.33384	0.10402	3.20941	0.00133	0.03804	2266.9	2031.32	2198.63	2674.88	2784.3	2729.22	Up
Ccl17	344.414	1.28849	0.39231	3.28433	0.00102	0.03181	213.877	104.83	281.526	533.341	612.13	320.783	Up
Coq9	2660.16	-0.5308	0.25479	-2.0833	0.03722	0.34382	2599.05	4395.92	2437.29	1987.26	2088.97	2452.5	Down
Gm10286	10.8451	-1.8611	0.87514	-2.1267	0.03345	0.32299	17.741	13.8454	19.4828	1.02173	9.90501	3.0746	Down
Tepp	82.7224	-0.8771	0.37777	-2.3218	0.02024	0.24074	137.985	82.0835	101.31	36.7821	64.3826	73.7903	Down
Prss54	18.9828	-1.4588	0.62284	-2.3422	0.01917	0.2326	23.6546	28.6798	31.1724	4.0869	9.90501	16.3979	Down
Ndrg4	693.231	-2.5619	0.52258	-4.9024	9.47E-07	0.00013	587.423	2432.84	536.75	189.019	197.11	216.247	Down
Cdh11	5249.83	-0.3203	0.14957	-2.1415	0.03223	0.3148	4896.5	6270.98	6323.13	4923.69	4423.58	4661.09	Down
Cdh16	443.867	0.78021	0.37267	2.09359	0.0363	0.33858	332.15	196.803	451.026	693.752	340.732	648.74	Up
Rrad	385.834	-0.9339	0.33975	-2.7487	0.00598	0.1081	422.826	771.387	325.362	240.105	240.692	314.634	Down
Gm33023	28.4277	-1.3214	0.52229	-2.53	0.01141	0.16459	25.6258	45.4921	50.6552	18.3911	9.90501	20.4973	Down
Ces2b	116.627	0.62499	0.28529	2.19071	0.02847	0.29168	111.374	79.1166	84.7501	117.498	130.746	176.277	Up
Nol3	776.655	-0.3813	0.19288	-1.9769	0.04805	0.3926	955.055	879.184	801.716	720.316	530.908	772.749	Down
Agrp	139.934	-0.6374	0.3019	-2.1114	0.03473	0.32919	194.165	146.366	170.474	85.8249	92.1166	150.655	Down
Pla2g15	2146.52	0.58311	0.28723	2.03013	0.04234	0.37156	2183.12	1593.21	1379.38	2491.99	3471.71	1759.69	Up
Cdh3	55.602	-0.9221	0.34258	-2.6918	0.00711	0.12048	78.8487	83.0725	56.5	31.6735	44.5725	38.9449	Down
Has3	209.413	-0.6006	0.18165	-3.3063	0.00095	0.03029	252.316	272.952	231.845	172.672	154.518	172.177	Down
Ddx19b	1232.98	-0.2956	0.1267	-2.3332	0.01964	0.23623	1305.93	1369.71	1400.81	1069.75	1033.09	1218.57	Down
Clec18a	5.7785	-1.9588	0.98413	-1.9904	0.04654	0.38628	7.88487	13.8454	5.84483	3.06518	1.981	2.04973	Down
Cenpn	140.594	0.72081	0.25653	2.80987	0.00496	0.09559	100.532	119.664	98.388	207.41	181.262	136.307	Up
Pkd1l2	42.1988	-1.1348	0.52697	-2.1534	0.03129	0.30967	37.4531	71.205	65.2673	17.3693	17.829	44.0692	Down
Hsd17b2	43.3367	-1.3301	0.45333	-2.934	0.00335	0.07329	55.1941	84.0614	46.7587	17.3693	21.791	34.8454	Down
Gm32352	24.9663	-1.6288	0.7704	-2.1142	0.0345	0.32847	11.8273	67.2491	34.0948	9.19553	6.93351	20.4973	Down
Wfdc1	1977.29	-0.4663	0.17633	-2.6446	0.00818	0.13205	1799.72	2565.36	2517.17	1633.74	1668.99	1678.73	Down
Pgbd5	18.6598	-1.7694	0.69603	-2.5421	0.01102	0.16088	12.8129	44.5031	29.2242	10.2173	4.9525	10.2487	Down
Agt	56.6492	-1.727	0.50702	-3.4062	0.00066	0.02324	86.7336	127.576	46.7587	15.3259	30.7055	32.7957	Down
Trim67	21.3277	-1.8834	0.94007	-2.0034	0.04513	0.38075	10.8417	80.1056	9.74139	5.10863	10.8955	11.2735	Down
Gm45805	141.334	-0.8753	0.43262	-2.0233	0.04305	0.37324	147.841	230.427	170.474	85.8249	51.506	161.929	Down
Gm10718	3.89955	3.44778	1.70149	2.02633	0.04273	0.37291	0.98561	0	0.97414	16.3476	0.9905	4.09946	Up
Olfr369-p	3.76869	-5.3391	1.67232	-3.1927	0.00141	0.03981	8.87048	6.92271	6.81897	0	0	0	Down
Casp1	986.015	0.59716	0.23688	2.52095	0.0117	0.16705	894.933	805.012	654.621	1127.98	1521.41	912.131	Up
Casp4	1501.47	0.26049	0.1031	2.52666	0.01152	0.16512	1369.01	1392.45	1337.49	1650.09	1692.77	1567.02	Up
Mmp13	76.0718	1.45691	0.39962	3.64577	0.00027	0.0117	55.1941	31.6467	35.069	87.8684	157.49	89.1633	Up
Mmp12	665.392	2.50772	0.42458	5.90634	3.50E-09	1.06E-06	257.244	183.946	155.862	1142.29	1762.1	490.911	Up
Gm10709	490.465	0.4693	0.17011	2.75884	0.0058	0.10597	475.063	409.429	349.716	586.47	564.585	557.527	Up
Piwil4	422.868	0.49988	0.18849	2.65204	0.008	0.13039	385.373	352.069	313.673	482.254	576.471	427.369	Up
Izumo1r	83.5386	1.0884	0.48001	2.26747	0.02336	0.2617	82.7911	40.5473	37.0173	132.824	147.585	60.4671	Up
Panx1	505.415	0.3204	0.1418	2.25948	0.02385	0.26511	483.934	434.153	430.569	543.558	602.225	538.055	Up
Hephl1	5.60267	-4.9045	2.18488	-2.2448	0.02478	0.27237	0	29.6687	2.92242	0	0	1.02487	Down
Fat3	1154.22	-0.4522	0.19715	-2.2938	0.0218	0.2515	1055.59	1524.97	1420.29	1045.23	837.964	1041.26	Down
Olfm2	192.29	0.99665	0.22377	4.45398	8.43E-06	0.00075	103.489	138.454	143.198	233.975	246.635	287.987	Up
Col5a3	219.585	-0.7223	0.34361	-2.1021	0.03554	0.33412	160.654	400.528	259.121	164.498	158.48	174.227	Down
Icam1	20560.8	0.41756	0.18956	2.20277	0.02761	0.28707	20133	15114.2	17570.5	25847.6	25879.8	18819.6	Up
Pde4a	1199.8	-0.2526	0.12286	-2.0556	0.03982	0.35955	1420.26	1208.51	1284.89	1067.7	1103.42	1114.03	Down
S1pr5	222.07	0.71991	0.36394	1.97809	0.04792	0.39227	145.87	198.781	158.785	180.845	430.868	217.272	Up
Gm16853	34.9552	-0.9069	0.39984	-2.2682	0.02332	0.2617	50.266	52.4148	34.0948	25.5431	21.791	25.6216	Down
Spc24	191.25	0.83987	0.25026	3.35605	0.00079	0.02639	146.856	163.178	101.31	275.866	232.768	227.52	Up
Cnn1	2179.85	-1.2629	0.39189	-3.2227	0.00127	0.03687	1640.05	4588.77	3003.27	1191.33	848.859	1806.84	Down
Bmper	1345.59	-0.5002	0.2196	-2.2776	0.02275	0.25751	1174.85	1830.56	1724.23	1170.9	1232.18	940.827	Down
Tbx20	358.774	-4.5076	1.23546	-3.6485	0.00026	0.01165	348.905	1340.04	373.095	4.0869	76.2686	10.2487	Down
Glb1l3	232.265	0.40114	0.20012	2.00451	0.04502	0.38062	207.963	192.847	199.698	314.691	242.673	235.719	Up
Jam3	860.31	-1.0212	0.2235	-4.5691	4.90E-06	0.0005	1059.53	1369.71	1028.69	607.927	430.868	665.138	Down
Gm31013	7.24998	-4.3483	1.56818	-2.7728	0.00556	0.104	7.88487	29.6687	3.89655	0	0	2.04973	Down

Tmem45b	220.844	-5.8176	1.0323	-5.6355	1.75E-08	4.28E-06	226.69	901.93	173.397	2.04345	14.8575	6.1492	Down
Barx2	486.231	-0.8959	0.44013	-2.0356	0.04179	0.36956	560.811	727.873	608.837	272.801	149.566	597.497	Down
Arhgap32	1861.43	-0.3827	0.15322	-2.4974	0.01251	0.17385	1895.33	2245.92	2179.15	1656.22	1402.55	1789.42	Down
Kcnj5	241.505	-4.2081	0.56315	-7.4725	7.87E-14	5.15E-11	304.553	819.846	250.354	17.3693	43.582	13.3233	Down
Gm37192	38.5015	-1.5504	0.43475	-3.5662	0.00036	0.01481	48.2948	77.1387	46.7587	24.5214	15.848	18.4476	Down
Gm33838	9.45334	2.43288	1.06443	2.28562	0.02228	0.25494	1.97122	4.94479	1.94828	34.7387	5.94301	7.17406	Up
Cdon	801.188	-0.4318	0.16328	-2.6443	0.00819	0.13205	955.055	1012.69	792.949	624.274	748.819	673.337	Down
Chek1	169.34	0.72912	0.29667	2.45771	0.01398	0.18778	125.172	129.554	127.612	264.627	232.768	136.307	Up
Siae	1195.64	0.24875	0.12301	2.02212	0.04316	0.37341	1156.12	1083.9	1038.43	1219.94	1284.68	1390.74	Up
Hspa8	91176.7	0.39495	0.19316	2.04473	0.04088	0.36391	63905.9	72905	99510.2	103005	110019	97714.8	Up
Crtam	46.9845	1.42053	0.50267	2.82599	0.00471	0.09241	39.4243	15.8233	21.431	89.9118	73.2971	42.0195	Up
Ubash3b	636.276	0.73592	0.24402	3.01585	0.00256	0.06009	466.193	508.324	457.845	825.554	1007.34	552.403	Up
Trim29	514.99	1.2227	0.45083	2.71212	0.00669	0.11605	382.416	321.411	223.078	669.23	1194.54	299.261	Up
Thy1	1246.64	0.46696	0.1445	3.23157	0.00123	0.03607	957.026	1025.55	1157.28	1560.17	1466.93	1312.85	Up
Cxcr5	473.206	-0.4606	0.21865	-2.1064	0.03517	0.33182	703.725	490.523	450.052	415.842	378.371	400.723	Down
Tmem25	197.815	-0.5492	0.25371	-2.1645	0.03043	0.30453	217.82	265.041	222.104	147.128	127.775	207.023	Down
Cd3g	613.831	0.81437	0.25706	3.16805	0.00153	0.04216	467.179	323.389	544.543	871.532	870.65	605.696	Up
Cd3d	407.082	0.7299	0.18197	4.01113	6.04E-05	0.00366	284.841	304.599	329.259	593.622	484.355	445.817	Up
Cd3e	143.734	0.7663	0.32156	2.38309	0.01717	0.21681	113.345	94.94	111.052	210.475	220.882	111.71	Up
Scn4b	220.21	-3.563	0.991	-3.5953	0.00032	0.01361	254.287	814.901	149.043	19.4128	71.3161	12.2984	Down
Fxyd6	1320.14	-0.5582	0.1729	-3.2285	0.00124	0.03632	1825.35	1598.16	1293.66	1064.64	1000.41	1138.63	Down
Gm31557	1.7966	-4.2709	2.13689	-1.9986	0.04565	NA	2.95683	1.97792	5.84483	0	0	0	Down
Ankk1	95.153	-1.3366	0.49416	-2.7048	0.00684	0.11768	105.46	162.189	141.25	46.9994	22.7815	92.2379	Down
Il18	875.882	0.75807	0.31049	2.44152	0.01463	0.19354	914.645	568.651	469.535	1048.29	1433.25	820.918	Up
Dlat	3744.72	-0.4447	0.20186	-2.203	0.0276	0.28707	3972.99	5482.78	3496.18	3042.7	2995.27	3478.39	Down
1810046K	182.292	-0.7253	0.27093	-2.6773	0.00742	0.12358	183.323	293.721	204.569	130.781	161.452	119.909	Down
Sln	2597.51	-4.762	1.86216	-2.5572	0.01055	0.15671	2995.26	9192.37	2843.51	6.13035	545.766	2.04973	Down
Idh3a	5647.98	-0.5287	0.23651	-2.2356	0.02538	0.27515	5716.53	8940.18	5357.76	4180.9	4302.74	5389.77	Down
Acsbg1	136.024	1.01285	0.41122	2.46306	0.01378	0.18605	88.7048	104.83	76.9569	189.019	266.445	90.1882	Up
Crabp1	12.5397	-1.8627	0.65577	-2.8405	0.0045	0.09015	22.669	18.7902	17.5345	5.10863	3.962	7.17406	Down
Tmem266	36.6028	-1.1236	0.44756	-2.5106	0.01205	0.17012	49.2804	57.3596	43.8362	22.478	13.867	32.7957	Down
Etfa	6639.59	-0.4858	0.22448	-2.1639	0.03047	0.30479	7037.25	9593.88	6609.53	5395.73	4419.61	6781.54	Down
Cspg4	979.743	-0.272	0.12936	-2.1022	0.03553	0.33412	978.709	1110.6	1126.1	840.88	887.489	934.678	Down
Ppcdc	2005.53	-0.2378	0.11637	-2.0438	0.04097	0.36426	2073.72	2235.05	2202.53	1863.63	1697.72	1960.57	Down
Islr	759.836	-0.4306	0.14639	-2.9417	0.00326	0.07204	820.026	968.19	828.992	703.969	625.997	611.845	Down
Loxl1	3370.73	-0.4118	0.18592	-2.215	0.02676	0.28244	2991.32	4324.71	4229.71	3046.79	3007.16	2624.68	Down
Hcn4	27.0881	-1.4975	0.51984	-2.8808	0.00397	0.0823	35.4819	51.4258	33.1207	20.4345	13.867	8.19893	Down
Myo9a	147.51	1.03838	0.31314	3.31605	0.00091	0.02954	115.316	72.1939	102.285	257.475	189.186	148.606	Up
Gm20275	30.3961	1.03205	0.49052	2.104	0.03538	0.33334	26.6114	9.88958	23.3793	44.9559	39.62	37.92	Up
Kif23	451.063	0.62259	0.25271	2.46367	0.01375	0.18584	392.272	413.384	260.095	574.21	633.921	432.493	Up
Paqr5	113.624	-0.899	0.38102	-2.3594	0.01831	0.22539	160.654	165.156	117.871	89.9118	42.5915	105.561	Down
Smad6	11107.3	0.39686	0.16796	2.36283	0.01814	0.22436	8412.17	9990.45	10364.8	11239	14892.2	11745	Up
Lctl	69.7636	-6.0864	0.96403	-6.3134	2.73E-10	1.03E-07	112.359	268.008	32.1466	4.0869	1.981	0	Down
Igdcc4	184.316	-1.5501	0.39499	-3.9243	8.70E-05	0.00482	243.445	418.329	162.681	77.6511	122.822	80.9644	Down
Rasl12	3675.88	-0.4226	0.18115	-2.3329	0.01965	0.23629	3594.52	4336.58	4700.22	2625.83	3184.46	3613.68	Down
Pif1	36.3614	0.96879	0.45664	2.12157	0.03387	0.32468	23.6546	31.6467	18.5086	55.1732	58.4396	30.746	Up
Rbpms2	485.353	-0.6444	0.26259	-2.454	0.01413	0.18925	464.222	817.868	493.888	337.169	396.2	402.772	Down
Fbxl22	298.639	-1.4888	0.48282	-3.0835	0.00205	0.05156	218.805	818.857	283.474	117.498	188.195	165.003	Down
Usp3	2626.36	0.26382	0.13065	2.01925	0.04346	0.37418	2596.09	2266.69	2297.99	2902.72	3087.39	2607.26	Up
Tpm1	29893.2	-1.0795	0.2754	-3.9196	8.87E-05	0.00489	30169.5	59929.9	31648.8	18533.1	18667	20411.2	Down
Ccnb2	270.914	0.98123	0.26308	3.72973	0.00019	0.00913	176.424	192.847	177.293	449.559	381.343	248.018	Up
Gm6018	11.0816	1.93879	0.76378	2.53842	0.01114	0.16205	2.95683	4.94479	5.84483	11.239	14.8575	26.6465	Up
Gm27188	11.3926	-2.3553	0.92728	-2.54	0.01108	0.16162	8.87048	37.5804	10.7155	5.10863	1.981	4.09946	Down
4930509E	33.4632	-0.9352	0.43876	-2.1315	0.03305	0.32034	33.5107	59.3375	38.9655	23.4997	18.8195	26.6465	Down
Rab27a	4191.96	0.43474	0.19953	2.1788	0.02935	0.29757	3052.43	3825.29	3817.65	4816.41	5817.21	3822.75	Up
Wdr72	332.647	-1.7551	0.41956	-4.1831	2.88E-05	0.00202	313.424	701.171	525.061	107.281	104.003	244.943	Down
Scg3	56.5099	-0.8013	0.38902	-2.0597	0.03943	0.35808	84.7624	73.1829	57.4742	28.6083	37.639	57.3925	Down
Mlip	269.399	-3.7577	1.85145	-2.0296	0.0424	0.37174	274.985	1028.52	201.647	10.2173	101.031	0	Down
Klhl31	250.634	-3.5293	1.08726	-3.246	0.00117	0.03481	159.669	959.289	264.966	8.1738	29.715	81.9893	Down
Cgas	204.849	0.55239	0.23264	2.37444	0.01758	0.21964	168.539	171.09	158.785	217.628	307.055	205.998	Up
Gm8116	390.059	-0.4368	0.15581	-2.8036	0.00505	0.09677	448.452	408.44	489.018	350.452	313.989	330.007	Down
Gm10635	10.218	-2.3336	0.82401	-2.832	0.00463	0.09174	9.85609	24.724	16.5604	4.0869	1.981	4.09946	Down
Col12a1	2107.46	0.50994	0.20814	2.44993	0.01429	0.19071	2047.11	1330.15	1839.17	2859.81	2225.66	2342.84	Up
4930429F	372.213	-0.5195	0.26346	-1.9717	0.04864	0.39488	354.819	540.96	419.854	315.713	374.409	227.52	Down
Filip1	631.461	-0.6116	0.18651	-3.2791	0.00104	0.03224	666.271	898.963	724.759	453.646	479.402	565.726	Down
Ttk	78.3608	0.97652	0.41389	2.35939	0.0183	0.22539	53.2229	68.2381	37.0173	116.477	133.718	61.492	Up
Snap91	47.2076	-3.4226	0.66362	-5.1574	2.50E-07	4.06E-05	58.1509	151.311	49.6811	4.0869	13.867	6.1492	Down
Bcl2a1a	281.569	1.16505	0.32285	3.60867	0.00031	0.01308	213.877	155.266	151.966	331.039	562.604	274.664	Up
Bcl2a1b	30.7236	0.98355	0.42591	2.3093	0.02093	0.24614	26.6114	15.8233	19.4828	34.7387	42.5915	45.0941	Up
Ctsh	23623	0.44303	0.14308	3.09635	0.00196	0.04995	23392.4	18505.4	18174.5	27801.1	26592	27272.7	Up
Plscr5	55.811	-0.8302	0.3021	-2.7482	0.00599	0.1081	67.0214	77.1387	70.138	38.8256	36.6485	45.0941	Down
1700057G	31.4755	-2.9046	0.78402	-3.7047	0.00021	0.00983	22.669	114.719	29.2242	10.2173	7.92401	4.09946	Down
Paqr9	139.709	-3.3037	0.70617	-4.6783	2.89E-06	0.00032	219.791	445.031	96.4397	10.2173	54.4775	12.2984	Down
Trpc1	1068.52	-0.2434	0.12022	-2.0247	0.04289	0.37291	1152.18	1165.98	1157.28	1023.77	1018.23	893.683	Down
Atp1b3	11652.5	0.2426	0.07977	3.04126	0.00236	0.05665	10610.1	10701.5	10713.6	12767.5	12468.4	12654	Up
Rasa2	1772.48	-0.2417	0.11209	-2.156	0.03108	0.30832	1856.89	1912.64	1992.11	1592.87	1542.21	1738.17	Down
Mras	1199.99	0.25944	0.12731	2.03786	0.04156	0.36801	1085.16	1059.17	1132.92	1268.98	1438.21	1215.49	Up
Ppp2r3a	2139.15	-0.6705	0.18359	-3.6524	0.00026	0.0115	2393.06	3202.25	2287.28	1495.81	1634.33	1822.21	Down
Ky	499.91	-1.9065	0.85153	-2.2389	0.02516	0.27427	438.596	1264.88	664.362	128.737	68.3446	434.543	Down
Gm5627	4.6095	-3.6538	1.54969	-2.3577	0.01839	0.22573	4.92804	14.8344	5.84483	0	0	2.04973	Down
Nphp3	274.308	-0.6166	0.24844	-2.4818	0.01307	0.17925	314.409	313.5	368.224	156.324	223.853	269.54	Down
Mapkapk3	2821.45	0.31924	0.15501	2.05941	0.03945	0.35814	2479.79	2677.11	2374.95	2875.14	3677.73	2844	Up
6430571L	24.7624	-1.8982	0.57214	-3.3176	0.00091	0.02944	45.338	45.4921	26.3017	6.13035	8.91451	16.3979	Down

Cacna2d2	36.6793	-2.4807	0.88017	-2.8184	0.00483	0.09356	24.6402	149.333	12.6638	12.2607	9.90501	11.2735	Down
Slc38a3	99.4233	-3.9661	0.89041	-4.4543	8.42E-06	0.00075	155.726	310.533	94.4914	2.04345	31.696	2.04973	Down
4833445I0	36.678	0.82021	0.38245	2.14465	0.03198	0.31321	23.6546	26.7019	29.2242	36.7821	53.487	50.2184	Up
Uqcrc1	7703.16	-0.4118	0.20783	-1.9815	0.04753	0.39072	7488.66	11484.8	7412.22	6464.46	6276.8	7092.07	Down
Pfkfb4	3405.19	0.47007	0.16411	2.86441	0.00418	0.08511	3066.23	2848.2	2651.61	3759.95	4681.11	3424.08	Up
Shisa5	16256.2	0.3261	0.11655	2.79803	0.00514	0.09791	15062.1	13675.3	14542.9	18155	19532.7	16569	Up
Fbxw26	25.8343	-3.029	1.22268	-2.4774	0.01324	0.18081	32.5251	91.9731	13.6379	1.02173	15.848	0	Down
Mir6236	378.372	2.02124	0.97796	2.06679	0.03875	0.35327	137	194.825	116.897	1450.85	157.49	213.172	Up
Ngp	157.813	1.35575	0.44396	3.05377	0.00226	0.05483	103.489	124.609	37.9914	178.802	301.112	200.874	Up
Lrrc2	336.808	-2.3687	0.59392	-3.9882	6.66E-05	0.00393	295.683	1139.28	258.147	92.977	175.319	59.4422	Down
Susd5	34.1831	-1.9298	0.62164	-3.1044	0.00191	0.04907	26.6114	92.9621	42.8621	12.2607	9.90501	20.4973	Down
Osbpl10	803.217	-0.3054	0.1322	-2.3101	0.02088	0.24603	908.731	890.062	865.035	645.73	764.667	745.077	Down
Eomes	101.278	0.67391	0.31528	2.13753	0.03255	0.31668	75.8919	98.8958	59.4225	112.39	155.509	105.561	Up
Itga9	1961.12	0.78279	0.27201	2.87781	0.004	0.08279	1516.85	1427.07	1381.33	3607.71	1838.37	1995.41	Up
Scn5a	1916.86	-1.5714	0.42713	-3.679	0.00023	0.01058	2232.4	3918.25	2454.83	746.881	482.374	1666.43	Down
Scn10a	45.3797	-3.725	0.66422	-5.6081	2.05E-08	4.86E-06	68.007	136.476	48.7069	2.04345	10.8955	6.1492	Down
Ccr8	31.4926	1.6266	0.60425	2.69196	0.0071	0.12048	22.669	13.8454	9.74139	37.8038	78.2496	26.6465	Up
Gm47050	14.285	1.65859	0.8321	1.99327	0.04623	0.38473	8.87048	4.94479	6.81897	9.19553	43.582	12.2984	Up
Gm39458	11.4162	3.40793	1.52723	2.23145	0.02565	0.27683	4.92804	0	0.97414	4.0869	56.4585	2.04973	Up
Zfp651	1424.24	-0.3325	0.14814	-2.2442	0.02482	0.27245	1731.71	1668.37	1362.82	1212.79	1228.22	1341.55	Down
Klhl40	11.1792	-3.9648	1.20788	-3.2825	0.00103	0.03195	5.91365	42.5252	14.6121	2.04345	1.981	0	Down
Hhatl	663.426	-1.0794	0.47353	-2.2795	0.02264	0.25704	541.099	1243.12	917.639	373.951	183.243	721.506	Down
Zkscan7	117.909	-0.562	0.25339	-2.2182	0.02654	0.28168	167.553	125.598	128.586	88.8901	87.1641	109.661	Down
Clec3b	2865.91	-0.6836	0.21107	-3.2387	0.0012	0.03538	2955.84	3854.96	3786.48	2780.11	1877.99	1940.07	Down
Slc6a20a	349.427	1.1127	0.23802	4.67484	2.94E-06	0.00032	247.388	240.317	175.345	428.103	590.339	415.071	Up
Xcr1	237.646	1.01444	0.36729	2.76198	0.00575	0.10587	184.309	165.156	122.741	223.758	496.241	233.669	Up
Ccr1	594.087	0.73791	0.34354	2.14799	0.03171	0.31185	660.358	376.793	299.061	778.555	928.099	521.657	Up
Ccr2	1970.44	0.70788	0.23354	3.03111	0.00244	0.05803	1417.31	1811.77	1260.54	2173.21	3142.86	2016.94	Up
Ccr5	491.02	1.3004	0.37351	3.48156	0.0005	0.01892	277.942	264.052	308.802	666.165	1078.66	350.504	Up
Plekhg1	1705.46	0.41255	0.20316	2.03064	0.04229	0.37149	1543.46	1362.78	1483.61	2099.65	2292.02	1451.21	Up
Syne1	7380.88	-0.2781	0.11236	-2.4747	0.01333	0.18166	7392.07	8181.65	8696.13	6472.63	6758.19	6784.61	Down
Gm21321	9.96508	-6.7424	1.45637	-4.6296	3.66E-06	0.00039	13.7985	13.8454	32.1466	0	0	0	Down
Ulbp1	326.323	0.77131	0.2916	2.64513	0.00817	0.13205	273.999	217.571	231.845	383.147	569.538	281.838	Up
Zc3h12d	267.423	0.59544	0.2751	2.16442	0.03043	0.30453	258.229	190.869	189.957	391.321	347.666	226.495	Up
Gm48727	29.3623	0.95528	0.42872	2.22822	0.02587	0.27824	16.7553	22.746	20.4569	42.9125	43.582	29.7211	Up
Samd5	1093.84	0.32736	0.14345	2.28209	0.02248	0.25623	983.638	862.371	1064.73	1309.85	1192.56	1149.9	Up
Rab32	1105.81	0.95764	0.36002	2.65992	0.00782	0.12796	917.602	788.2	549.414	1300.66	2236.55	842.44	Up
Grm1	36.5888	-4.2213	1.06052	-3.9805	6.88E-05	0.00401	15.7697	175.046	17.5345	7.15208	1.981	2.04973	Down
B230208H	179.02	-0.5131	0.25064	-2.0472	0.04063	0.36257	179.381	211.637	240.612	114.433	174.328	153.73	Down
Stx11	1406.7	0.25275	0.12723	1.98655	0.04697	0.38829	1230.04	1303.45	1318.01	1456.98	1691.78	1439.94	Up
Adat2	202.027	-1.2108	0.23274	-5.2022	1.97E-07	3.46E-05	274.985	231.416	339.974	122.607	109.946	133.233	Down
Gm47712	15.4911	-2.3615	0.75743	-3.1178	0.00182	0.04752	25.6258	38.5694	13.6379	6.13035	6.93351	2.04973	Down
Cited2	3919.2	0.25611	0.10212	2.508	0.01214	0.17104	3439.77	3577.06	3699.78	4167.62	4118.5	4512.48	Up
Txlnb	791.032	-3.2364	0.91765	-3.5269	0.00042	0.01654	897.89	2934.24	458.819	92.977	281.302	80.9644	Down
Gm48483	4.26708	-5.5182	1.85728	-2.9711	0.00297	0.06689	1.97122	16.8123	6.81897	0	0	0	Down
Tnfaip3	1406.54	0.50657	0.24727	2.04868	0.04049	0.3624	1219.2	1133.35	1133.9	1607.17	2201.88	1143.75	Up
Ahi1	897.162	-0.3625	0.16446	-2.2043	0.0275	0.28689	938.3	969.179	1120.26	756.077	698.303	900.857	Down
1700021A	5.71434	4.03639	1.4316	2.81949	0.00481	0.09349	0	1.97792	0	13.2824	12.8765	6.1492	Up
Raet1e	9.72761	2.48282	0.85455	2.9054	0.00367	0.07734	4.92804	2.96687	0.97414	11.239	19.81	18.4476	Up
Vnn1	1198.03	0.82989	0.35398	2.34446	0.01905	0.23143	1406.46	575.574	605.914	1376.26	1396.61	1827.34	Up
Enpp3	89.711	-0.593	0.28214	-2.102	0.03555	0.33412	113.345	126.587	83.7759	77.6511	71.3161	65.5914	Down
Gm36172	8.89533	1.81711	0.92009	1.97493	0.04828	0.394	7.88487	0.98896	2.92242	10.2173	11.886	19.4725	Up
Tmem200	407.746	-0.5178	0.21638	-2.3929	0.01672	0.21274	403.114	501.402	535.776	336.148	272.388	397.648	Down
Samd3	125.691	0.62271	0.31554	1.97344	0.04844	0.39463	75.8919	128.565	92.5432	124.65	194.138	138.357	Up
Themis	68.3323	1.12816	0.35118	3.21245	0.00132	0.03769	37.4531	42.5252	48.7069	121.585	93.1071	66.6163	Up
Soga3	1.96198	-4.3977	2.07594	-2.1184	0.03414	NA	1.97122	3.95583	5.84483	0	0	0	Down
Nkain2	50.8712	1.20051	0.4509	2.66248	0.00776	0.12767	38.4387	36.5914	17.5345	50.0645	69.3351	93.2628	Up
Trdn	932.449	-3.5586	1.12483	-3.1637	0.00156	0.04258	1019.12	3303.12	834.837	32.6952	351.628	53.293	Down
Gm48018	37.4765	-1.7829	0.53654	-3.3229	0.00089	0.02901	44.3524	86.0394	43.8362	9.19553	16.8385	24.5968	Down
Calhm6	61.8129	1.61087	0.52599	3.06256	0.00219	0.05391	20.6978	45.4921	25.3276	62.3252	154.518	62.5168	Up
Frk	598.058	0.44216	0.19217	2.30087	0.0214	0.24952	552.926	538.982	429.595	613.035	811.22	642.591	Up
Amd2	114.657	-1.0732	0.26963	-3.9804	6.88E-05	0.00401	136.014	133.509	196.776	73.5642	74.2876	73.7903	Down
Lama4	4621.46	-0.3384	0.16266	-2.0801	0.03751	0.34545	4485.51	5288.95	5708.45	4222.79	4506.78	3516.31	Down
Mfsd4b5	147.649	-0.9606	0.48163	-1.9945	0.0461	0.38407	196.136	214.604	174.371	103.194	35.658	161.929	Down
Ddo	2268.4	-0.8075	0.33441	-2.4147	0.01575	0.20351	2542.87	2985.66	3132.83	1578.57	895.413	2475.05	Down
Cdc40	2284.87	0.28836	0.14436	1.99747	0.04577	0.38275	2317.17	1891.88	1962.89	2523.66	2718.92	2294.67	Up
Wasf1	178.827	-0.6187	0.21515	-2.8758	0.00403	0.08303	180.366	252.184	217.233	142.02	137.68	143.481	Down
Qrsl1	784.143	-0.371	0.14241	-2.6054	0.00918	0.14264	800.314	982.035	870.88	683.534	652.74	715.356	Down
Speer5-ps	3.42701	-5.2026	1.83577	-2.834	0.0046	0.09132	3.94243	3.95583	12.6638	0	0	0	Down
Bves	223.691	-2.6339	0.5986	-4.4001	1.08E-05	0.00089	242.46	700.182	213.336	20.4345	97.0691	68.666	Down
Ascc3	1860.88	0.27813	0.12254	2.26977	0.02322	0.26122	1571.06	1719.8	1755.4	2082.28	2161.27	1875.5	Up
Ros1	129.535	2.05999	0.33279	6.19016	6.01E-10	2.13E-07	52.2373	42.5252	55.5259	230.91	135.699	260.316	Up
Hsf2	1305.15	-0.3044	0.12035	-2.5289	0.01144	0.16462	1405.48	1421.13	1500.17	1190.31	1072.71	1241.11	Down
Smpdl3a	10153.2	0.26788	0.13381	2.00187	0.0453	0.38132	10271	9171.6	8197.38	10408.3	11636.4	11234.6	Up
Sowahc	2670.54	0.32772	0.15515	2.11224	0.03467	0.32919	2638.47	2262.74	2204.48	2785.22	3411.28	2721.02	Up
P4ha1	7587.85	0.31687	0.15696	2.01881	0.04351	0.37421	6575.98	6071.21	7626.53	8731.66	9220.57	7301.14	Up
Oit3	48.9903	-0.8189	0.34144	-2.3984	0.01647	0.21109	54.2085	58.3485	75.0087	32.6952	32.6865	40.9946	Down
Pald1	3834.01	0.3289	0.16668	1.9732	0.04847	0.39468	3737.43	2971.82	3487.42	4553.83	4657.33	3596.25	Up
D830039M	16.3206	-2.3427	0.94943	-2.4675	0.01361	0.18435	7.88487	58.3485	15.5862	6.13035	7.92401	2.04973	Down
Lrrc20	918.342	-0.3458	0.11839	-2.921	0.00349	0.07481	995.465	1075	1013.1	811.25	766.648	848.589	Down
Col13a1	3189.2	-0.3752	0.15433	-2.4311	0.01506	0.19718	3776.85	3549.37	3478.65	2770.92	3204.27	2355.14	Down
Pbld2	316.094	0.87057	0.30075	2.89464	0.0038	0.07951	259.215	217.571	193.854	334.104	585.386	306.435	Up
Mypn	286.806	-3.4568	0.58341	-5.9253	3.12E-09	9.76E-07	245.417	989.947	341.923	28.6083	84.1926	30.746	Down

Zfp365	1414.74	0.4899	0.19869	2.46562	0.01368	0.18518	1284.25	958.3	1287.81	1973.97	1457.03	1527.05	Up
Cdk1	344.082	1.37643	0.3535	3.89378	9.87E-05	0.00534	175.438	276.908	121.767	639.6	498.222	352.554	Up
A330049N	22.2359	4.70443	1.10372	4.26235	2.02E-05	0.00148	0	4.94479	0	32.6952	75.2781	20.4973	Up
Pcdh15	23.2172	1.65894	0.68245	2.43087	0.01506	0.19718	16.7553	12.8565	3.89655	33.7169	19.81	52.2682	Up
Specc1l	8660.76	-0.2602	0.0928	-2.8034	0.00506	0.09677	9062.67	9772.88	9483.24	7833.57	8135.97	7676.25	Down
Ggt5	775.149	0.50203	0.1845	2.72096	0.00651	0.11428	754.976	518.214	651.699	848.032	962.767	915.205	Up
Derl3	192.765	1.52306	0.43795	3.47774	0.00051	0.01912	124.187	66.2602	108.129	271.779	442.754	143.481	Up
Itgb2	5440.66	1.17516	0.42658	2.75486	0.00587	0.10674	4518.03	3131.04	2370.08	6543.13	12599.2	3482.49	Up
Gm18119	3.95678	-2.7686	1.32524	-2.0891	0.0367	0.3402	5.91365	11.8675	2.92242	1.02173	0.9905	1.02487	Down
Gatd3a	1920.7	-0.5242	0.19693	-2.662	0.00777	0.12767	2116.1	2852.16	1829.43	1483.55	1615.51	1627.49	Down
Gm47015	253.604	1.93344	0.25902	7.46448	8.36E-14	5.31E-11	99.5465	128.565	87.6725	316.735	486.336	402.772	Up
Prtn3	58.0118	-0.785	0.36631	-2.1431	0.0321	0.31414	92.6472	61.3154	66.2414	54.1514	31.696	42.0195	Down
Cfd	1150.85	-5.1203	0.94151	-5.4384	5.38E-08	1.14E-05	1494.18	3414.87	1803.13	13.2824	139.661	39.9698	Down
Cirbp	3479.08	-0.4292	0.14904	-2.8797	0.00398	0.08243	3730.53	4070.55	4177.11	2958.92	2560.44	3376.93	Down
Apc2	171.838	-0.5992	0.2268	-2.642	0.00824	0.13272	163.611	232.405	225.026	133.846	134.708	141.431	Down
Mob3a	2477.78	0.31377	0.13933	2.252	0.02432	0.26894	2241.27	2112.41	2274.61	2676.92	3116.12	2445.33	Up
Lingo3	7.05124	-4.3272	1.49722	-2.8901	0.00385	0.08043	3.94243	22.746	13.6379	0	1.981	0	Down
Tmprss9	14.3872	-1.4299	0.62943	-2.2718	0.0231	0.26032	21.6834	21.7571	19.4828	11.239	3.962	8.19893	Down
Gng7	234.839	-0.4195	0.19604	-2.1401	0.03235	0.3155	277.942	285.809	242.561	218.649	213.948	170.128	Down
Atcayos	19.2935	-2.6153	0.91468	-2.8592	0.00425	0.08595	10.8417	66.2602	22.4052	2.04345	3.962	10.2487	Down
Gna15	326.47	0.70113	0.33249	2.10874	0.03497	0.3306	279.913	230.427	235.742	343.3	610.149	259.291	Up
Nuak1	1491.57	-0.3472	0.15728	-2.2073	0.02729	0.2859	1465.6	1761.33	1783.65	1349.7	1438.21	1150.92	Down
Ascl1	96.8496	-1.4991	0.55899	-2.6818	0.00732	0.12303	110.388	211.637	107.155	43.9342	18.8195	89.1633	Down
Parpbp	39.8747	1.13861	0.44326	2.56869	0.01021	0.15335	22.669	30.6577	21.431	62.3252	68.3446	33.8206	Up
Dram1	20079.4	0.35607	0.16243	2.19215	0.02837	0.29075	20738.2	14633.6	17470.2	21614.6	22438.8	23581.1	Up
Slc5a8	73.0976	-7.1746	1.76357	-4.0682	4.74E-05	0.00302	12.8129	389.649	33.1207	1.02173	1.981	0	Down
Ano4	26.1542	-3.3373	0.84965	-3.9278	8.57E-05	0.00476	31.5395	80.1056	31.1724	0	6.93351	7.17406	Down
Gas2l3	131.681	1.15534	0.35141	3.28775	0.00101	0.03165	88.7048	85.0504	71.1121	185.954	249.606	109.661	Up
Gm20757	3.11082	-5.0625	1.89296	-2.6744	0.00749	0.12437	1.97122	8.90062	7.79311	0	0	0	Down
Gm29684	41.143	1.26003	0.5434	2.31879	0.02041	0.24213	17.741	29.6687	25.3276	62.3252	86.1736	25.6216	Up
Nudt4	14111.9	-0.6403	0.25334	-2.5274	0.01149	0.16493	13634.9	24229.5	13714.9	10730.2	10113	12249.2	Down
Dcn	9443.73	-0.9611	0.23925	-4.0173	5.89E-05	0.00359	13496.9	15018.3	8919.21	5986.29	7757.6	5484.06	Down
Lum	803.469	-1.8539	0.38213	-4.8515	1.23E-06	0.00015	947.17	2018.46	810.483	456.711	244.654	343.33	Down
Mgat4c	15.5889	1.41037	0.63622	2.21679	0.02664	0.28184	8.87048	8.90062	7.79311	14.3042	18.8195	34.8454	Up
Nts	34.4219	-1.9857	0.71936	-2.7604	0.00577	0.10589	12.8129	71.205	80.8535	16.3476	8.91451	16.3979	Down
Rpl6l	1550.01	0.30783	0.14502	2.12272	0.03378	0.32409	1417.31	1508.16	1230.34	1643.96	1613.53	1886.78	Up
Nap1l1	16571.5	-0.2045	0.09673	-2.1136	0.03455	0.32867	17651.3	17613.3	17967	15347.3	16312.6	14537.7	Down
Gm5176	51.3768	-0.9129	0.37386	-2.4419	0.01461	0.19354	74.9063	55.3817	71.1121	29.63	48.5345	28.6962	Down
Caps2	172.095	-0.6947	0.3483	-1.9947	0.04608	0.38407	191.208	248.228	198.724	115.455	83.2021	195.749	Down
Lyz2	360085	0.80726	0.26714	3.02189	0.00251	0.05935	372732	199073	213870	487610	504413	382814	Up
Gm48903	28.8374	-2.2306	0.60465	-3.689	0.00023	0.01026	22.669	74.1719	45.7845	12.2607	8.91451	9.22379	Down
Ppm1h	1589.92	0.28543	0.10828	2.6359	0.00839	0.13443	1390.69	1502.23	1406.66	1716.5	1832.43	1691.03	Up
Slc26a10	298.454	-0.9566	0.27334	-3.4998	0.00047	0.01799	261.186	458.877	461.742	181.867	215.929	211.122	Down
Arhgap9	1195.27	0.64968	0.19839	3.27472	0.00106	0.03241	1011.23	974.124	806.587	1380.35	1784.88	1214.47	Up
Gpr182	3752.97	0.6313	0.21013	3.00432	0.00266	0.06165	3266.31	2247.9	3319.86	5360.99	4347.31	3975.45	Up
Atp5b	37164.3	-0.451	0.19993	-2.2556	0.0241	0.26729	38011	55480.5	35286.2	30381	31066.1	32760.9	Down
Gls2	112.784	0.54318	0.27103	2.0041	0.04506	0.38066	98.5609	103.841	73.0604	110.346	152.537	138.357	Up
Stat2	3399.91	0.46882	0.2366	1.98151	0.04753	0.39072	2763.65	3002.48	2790.91	3791.62	5244.7	2806.08	Up
Cs	10290.7	-0.3884	0.18314	-2.1206	0.03395	0.32502	10509.5	14645.5	9847.57	8882.88	8790.7	9068.01	Down
Coq10a	2232.23	-0.3821	0.18661	-2.0475	0.04061	0.36253	2256.06	3157.74	2164.54	1863.63	1904.73	2046.66	Down
Ikzf4	221.768	-0.6616	0.23616	-2.8014	0.00509	0.09724	205.007	284.82	325.362	172.672	164.423	178.327	Down
Tespa1	224.914	0.69608	0.27726	2.51059	0.01205	0.17012	198.107	158.233	158.785	283.018	355.59	195.749	Up
Sfi1	3778.56	-0.3678	0.11584	-3.175	0.0015	0.04132	3838.95	4487.89	4445.97	3261.35	3362.75	3274.45	Down
Inpp5j	59.1891	-2.6252	0.58222	-4.5089	6.52E-06	0.00063	75.8919	183.946	45.7845	17.3693	18.8195	13.3233	Down
Osbp2	103.5	-1.924	0.42695	-4.5063	6.60E-06	0.00063	120.244	267.019	104.233	43.9342	44.5725	40.9946	Down
Mtfp1	464.861	-0.544	0.25392	-2.1426	0.03215	0.31414	493.79	748.641	412.061	360.669	377.381	396.623	Down
Tbc1d10a	2898.89	0.23948	0.1167	2.05199	0.04017	0.36086	2856.29	2554.48	2565.88	3076.42	3354.83	2985.43	Up
Lif	293.868	-0.66	0.22065	-2.9911	0.00278	0.06352	280.898	396.572	402.319	205.367	222.863	255.192	Down
Ascc2	5552.4	0.40813	0.17384	2.34769	0.01889	0.23008	4524.93	4781.61	5009.99	5918.86	5356.63	7722.36	Up
Zmat5	726.426	0.43748	0.20543	2.12952	0.03321	0.32161	646.559	563.706	640.983	842.923	649.769	1014.62	Up
Nf2	5307.97	-0.2542	0.10799	-2.3539	0.01858	0.22743	5550.95	5836.83	5935.43	5072.87	4980.24	4471.49	Down
Nipsnap1	1036.11	-0.7958	0.17892	-4.4476	8.68E-06	0.00075	1383.79	1265.87	1294.63	815.337	600.244	856.788	Down
Dbnl	5230.6	0.28055	0.12191	2.3012	0.02138	0.24952	4994.08	4519.54	4657.36	5232.26	6242.14	5738.22	Up
Pgam2	1127.28	-2.5373	0.51599	-4.9173	8.77E-07	0.00012	1060.51	3662.11	1047.2	272.801	516.051	204.973	Down
Myl7	7501.85	-4.2139	1.32908	-3.1705	0.00152	0.04188	9151.38	24001	9557.27	34.7387	2076.09	190.625	Down
Camk2b	4800.94	3.42945	1.31911	2.59981	0.00933	0.14418	1473.49	907.864	65.2673	7236.88	17807.2	1314.9	Up
Ogdh	15889.5	-0.3723	0.14883	-2.5016	0.01236	0.17237	16558.2	21083.6	16143.4	13310	14379.1	13862.3	Down
Myo1g	2074.32	0.76115	0.28931	2.63088	0.00852	0.13583	1615.41	1680.24	1322.88	2465.42	3696.55	1665.41	Up
Igfbp3	7161.18	-0.518	0.23022	-2.25	0.02445	0.26955	7980.47	6410.43	10908.4	6895.62	5234.8	5537.35	Down
Sun3	10.8836	-1.8521	0.84567	-2.1901	0.02851	0.29183	31.5395	7.91166	11.6897	5.10863	4.9525	4.09946	Down
Upp1	1255.76	0.50426	0.20856	2.41781	0.01561	0.20227	894.933	875.228	1345.29	1560.17	1460	1398.94	Up
Spata48	53.6201	-0.8024	0.36441	-2.202	0.02766	0.28707	53.2229	85.0504	66.2414	42.9125	44.5725	29.7211	Down
Ikzf1	1732.48	0.50119	0.22323	2.24521	0.02475	0.27233	1470.53	1457.72	1375.48	2039.36	2585.21	1466.58	Up
Fignl1	124.858	0.97253	0.40733	2.38755	0.01696	0.21493	108.417	89.0062	55.5259	187.997	214.939	93.2628	Up
Ddc	186.645	-0.9044	0.33299	-2.7159	0.00661	0.11532	184.309	325.367	220.155	157.346	87.1641	145.531	Down
Vstm2a	131.15	1.02713	0.52057	1.97307	0.04849	0.39468	140.942	82.0835	36.0431	175.737	249.606	102.487	Up
Plek	3730.08	0.93153	0.3026	3.07845	0.00208	0.05192	2900.65	2721.61	2075.89	4376.05	7141.51	3164.79	Up
Etaa1os	16.3343	-1.1606	0.56671	-2.048	0.04056	0.36253	21.6834	17.8012	28.25	9.19553	12.8765	8.19893	Down
8430419K	36.7156	-1.0131	0.40442	-2.5051	0.01224	0.17173	43.3668	42.5252	61.3707	23.4997	19.81	29.7211	Down
Slc1a4	3550.4	-0.7515	0.3197	-2.3506	0.01874	0.22882	3468.36	4508.66	5386.99	2384.71	1656.12	3897.56	Down
Mdh1	12859.5	-0.699	0.26606	-2.6271	0.00861	0.13683	12254.1	22903.3	12587.8	9639.98	8861.02	10910.7	Down
Otx1	114.635	-3.9898	0.63642	-6.2691	3.63E-10	1.33E-07	105.46	385.694	155.862	20.4345	3.962	16.3979	Down
Xpo1	8509.93	0.21532	0.09358	2.30089	0.0214	0.24952	7611.86	7701.02	8315.25	9273.18	9076.95	9081.34	Up

1700093K	11.3742	-1.8049	0.70967	-2.5433	0.01098	0.16054	23.6546	11.8675	17.5345	4.0869	4.9525	6.1492 Down
Acyp2	1020.01	-0.3917	0.17092	-2.2917	0.02192	0.2525	1033.9	1346.96	1092.01	984.943	799.334	862.937 Down
Il9r	179.186	1.29163	0.47132	2.74047	0.00614	0.10967	166.568	73.1829	72.0863	302.431	334.789	126.059 Up
Hba-a2	3310.89	-2.1366	1.00834	-2.1189	0.03409	0.32547	8528.47	3544.43	4111.84	161.433	774.572	2744.59 Down
Dock2	1843.07	0.6532	0.30041	2.17437	0.02968	0.30007	1703.13	1386.52	1208.91	2271.3	3111.16	1377.42 Up
Hmmr	120.768	1.69231	0.46493	3.63994	0.00027	0.01195	61.1077	70.216	39.9397	256.453	215.929	80.9644 Up
Adra1b	153.714	-2.0472	0.39627	-5.1661	2.39E-07	3.94E-05	231.618	365.914	145.147	56.1949	76.2686	47.1438 Down
Il12b	53.441	1.76955	0.64412	2.74724	0.00601	0.10824	30.5539	19.7792	22.4052	81.738	143.623	22.547 Up
Ebf1	1041.02	-0.4124	0.19132	-2.1554	0.03113	0.30867	1079.24	1375.64	1111.49	1070.77	828.059	780.948 Down
Nipal4	16.9521	-2.1049	0.78021	-2.6979	0.00698	0.11922	11.8273	38.5694	32.1466	9.19553	7.92401	2.04973 Down
Cyfip2	2728.95	-0.5119	0.20695	-2.4736	0.01338	0.18201	3066.23	3842.1	2715.9	1930.04	2082.03	2737.42 Down
Itk	492.148	0.68956	0.26377	2.61425	0.00894	0.14065	405.085	282.842	442.259	648.796	726.037	447.866 Up
Havcr2	531.431	1.16095	0.41905	2.77043	0.0056	0.10454	475.063	252.184	258.147	585.449	1196.53	421.22 Up
Timd2	8.71539	4.06499	1.17569	3.45754	0.00055	0.02037	0	1.97792	0.97414	17.3693	23.772	8.19893 Up
Irgm1	3312.91	0.72916	0.26935	2.7071	0.00679	0.11716	2692.68	2454.59	2332.09	3780.38	5831.08	2786.61 Up
Gfpt2	1118.26	-0.5788	0.29178	-1.9838	0.04728	0.38984	1153.16	1494.32	1371.59	742.794	1315.39	632.342 Down
Gm26542	122.66	-0.7749	0.38204	-2.0282	0.04254	0.3722	123.201	146.366	194.828	115.455	49.525	106.586 Down
Cdkn2aipr	2826.39	0.23337	0.10233	2.28059	0.02257	0.25683	2682.83	2515.91	2596.08	3225.59	2987.35	2950.59 Up
Olfr1372-	306.051	1.1936	0.32316	3.69347	0.00022	0.01012	282.87	135.487	140.276	481.233	410.067	386.374 Up
Fstl4	153.676	-1.5257	0.27403	-5.5679	2.58E-08	5.92E-06	179.381	288.776	216.259	68.4556	86.1736	83.0141 Down
Irf1	6834.36	0.39605	0.15167	2.61124	0.00902	0.14115	5722.44	5405.64	6578.36	7151.06	8866.96	7281.67 Up
Pdlim4	271.386	-1.1623	0.32584	-3.567	0.00036	0.01481	323.28	552.828	249.379	146.107	175.319	181.401 Down
Csf2	43.6335	0.85093	0.38598	2.20463	0.02748	0.28689	25.6258	33.6246	34.0948	52.108	43.582	72.7655 Up
Acsl6	36.8709	-2.1866	0.94207	-2.321	0.02028	0.24108	21.6834	123.62	36.0431	18.3911	0.9905	20.4973 Down
Tnip1	2442.18	0.34202	0.15245	2.24357	0.02486	0.27251	2489.65	1952.2	2020.36	2722.9	2944.76	2523.22 Up
Gm2a	6190.27	0.39621	0.17657	2.2439	0.02484	0.27245	5914.64	5455.09	4667.1	6720.91	8334.07	6049.78 Up
Gria1	1059.84	-0.6074	0.20643	-2.9423	0.00326	0.07204	967.868	1332.13	1539.14	812.272	909.28	798.371 Down
Gm12250	328.149	1.23195	0.53532	2.30136	0.02137	0.24952	320.323	179.99	87.6725	369.865	782.496	228.545 Up
Irgm2	4276.96	0.90543	0.39198	2.30985	0.0209	0.24604	2867.14	3165.65	2899.04	4021.51	9745.54	2962.89 Up
Olfr317	9.70334	-2.6351	0.95641	-2.7552	0.00586	0.1067	5.91365	27.6908	16.5604	2.04345	3.962	2.04973 Down
Olfr316	6.39979	-5.1034	1.47826	-3.4523	0.00056	0.02063	15.7697	13.8454	7.79311	0	0.9905	0 Down
Olfr314	7.10254	-1.8746	0.92703	-2.0222	0.04316	0.37341	8.87048	16.8123	7.79311	5.10863	1.981	2.04973 Down
Obscn	1411.38	-3.6517	1.07825	-3.3867	0.00071	0.02449	1629.21	4808.31	1406.66	72.5425	497.231	54.3179 Down
Aldh3a1	2102.06	-1.3506	0.37313	-3.6196	0.0003	0.01275	2555.68	4394.93	2109.01	1215.85	673.541	1663.36 Down
Slc47a1	351.296	-1.3046	0.38854	-3.3576	0.00079	0.02632	397.2	751.608	351.664	180.845	292.198	134.257 Down
Grap	5443.26	0.34003	0.16307	2.08525	0.03705	0.34277	4669.81	4428.55	5315.87	6645.3	6541.27	5058.74 Up
Akap10	1591.1	0.24631	0.12509	1.96913	0.04894	0.3964	1530.65	1394.43	1441.73	1887.13	1689.79	1602.89 Up
Trpv2	1180.85	0.70913	0.26379	2.68828	0.00718	0.12136	1069.39	827.758	791.975	1389.55	1965.15	1041.26 Up
Tvp23bos	37.8252	1.28916	0.57297	2.24995	0.02445	0.26955	39.4243	11.8675	14.6121	38.8256	51.506	70.7157 Up
Hs3st3a1	243.872	0.82356	0.3256	2.52937	0.01143	0.16462	224.719	176.035	127.612	311.626	410.067	213.172 Up
Myocd	109.167	-1.4446	0.38934	-3.7104	0.00021	0.00963	93.6328	236.361	149.043	52.108	63.3921	60.4671 Down
Gm12295	90.7549	-2.779	0.69714	-3.9862	6.71E-05	0.00394	119.259	295.698	60.3966	9.19553	43.582	16.3979 Down
Myh8	6.74274	-6.1781	1.75364	-3.523	0.00043	0.01675	4.92804	30.6577	4.87069	0	0	0 Down
Dhrs7c	355.139	-4.0033	1.59742	-2.5061	0.01221	0.17151	408.042	1246.09	351.664	1.02173	117.87	6.1492 Down
Pik3r5	1182.91	0.9766	0.35273	2.76866	0.00563	0.10469	924.501	780.288	686.768	1434.5	2420.78	850.639 Up
Ccdc42	160.278	-0.7796	0.3394	-2.297	0.02162	0.25019	131.086	272.952	203.595	112.39	95.0881	146.556 Down
Myh10	9269.38	-0.3414	0.15296	-2.2318	0.02563	0.27683	10320.3	10534.4	10228.5	8308.67	9399.85	6824.58 Down
Aurkb	165.415	1.49576	0.38726	3.86247	0.00011	0.00591	86.7336	111.752	61.3707	293.235	303.093	136.307 Up
Vamp2	7942.18	-0.2231	0.08699	-2.5647	0.01033	0.15431	8705.88	8491.19	8468.19	7586.31	7204.9	7196.61 Down
Atp1b2	533.376	-0.5146	0.16131	-3.1901	0.00142	0.03994	616.991	659.635	605.914	378.038	446.716	492.96 Down
Sat2	158.68	-0.5644	0.222	-2.5424	0.01101	0.16084	163.611	196.803	207.492	130.781	110.936	142.456 Down
Ybx2	157.798	-0.9378	0.35606	-2.6337	0.00845	0.13521	116.302	271.963	233.793	120.564	111.927	92.2379 Down
Slc2a4	722.835	-3.0658	0.54624	-5.6126	1.99E-08	4.78E-06	802.286	2510.96	561.104	77.6511	187.205	197.799 Down
Acadvl	2262.42	-0.4964	0.1901	-2.611	0.00903	0.14115	2506.4	3280.37	2156.74	1742.04	1901.76	1987.21 Down
Clec10a	268.189	-0.7972	0.35932	-2.2187	0.02651	0.28156	327.222	495.468	198.724	233.975	202.062	151.68 Down
Alox15	110.632	-3.0866	1.23374	-2.5018	0.01236	0.17237	81.8055	436.131	75.9828	14.3042	52.4965	3.0746 Down
Arrb2	2108.85	0.43413	0.19342	2.24454	0.0248	0.27238	2122.02	1586.29	1673.57	2407.18	2835.8	2028.21 Up
Cxcl16	4085.32	0.42558	0.14949	2.84697	0.00441	0.08866	3523.55	3312.02	3625.74	4964.56	5152.59	3933.44 Up
Zmynd15	580.277	0.87173	0.31718	2.74837	0.00599	0.1081	413.956	331.301	485.121	709.077	1069.74	472.463 Up
Pld2	2374.48	0.24812	0.11884	2.08779	0.03682	0.34093	2274.78	2096.59	2141.16	2548.18	2796.18	2389.99 Up
Pfn1	16966.6	0.21868	0.09522	2.29661	0.02164	0.25032	16030.9	15081.6	15936.9	19165.5	17601.2	17983.3 Up
Eno3	2745.91	-1.6663	0.36936	-4.5115	6.44E-06	0.00062	2958.8	6807	2762.66	1057.49	1731.4	1158.1 Down
Inca1	131.089	-0.577	0.22356	-2.5809	0.00985	0.15019	174.453	151.311	145.147	91.9553	110.936	112.735 Down
Scimp	239.97	1.09918	0.34183	3.21557	0.0013	0.03739	236.546	101.863	119.819	316.735	362.523	302.335 Up
Mis12	1405.9	0.37034	0.11974	3.09298	0.00198	0.05034	1306.92	1130.38	1242.03	1594.91	1641.26	1519.88 Up
Pimreg	107.881	2.04809	0.48575	4.21631	2.48E-05	0.00178	30.5539	68.2381	27.2759	253.388	167.395	100.437 Up
Pitpnm3	2834.33	-0.3872	0.17976	-2.1539	0.03125	0.30941	2670.01	3379.27	3587.75	2495.05	2107.79	2766.11 Down
Xaf1	3174.54	0.69312	0.20446	3.39002	0.0007	0.0243	2889.8	2386.36	2002.83	3939.77	4592.95	3235.5 Up
Itgae	420.111	0.77657	0.37302	2.08183	0.03736	0.34417	460.279	215.593	253.276	458.755	759.714	373.051 Up
Aspa	852.031	-0.4727	0.17831	-2.651	0.00802	0.13049	882.12	1202.57	886.466	749.946	699.294	691.784 Down
Cluh	3319.97	-0.3423	0.16236	-2.1082	0.03501	0.3306	3111.57	4386.03	3638.41	2841.42	2977.45	2964.94 Down
Serpinf1	2073.8	-0.4808	0.20576	-2.3367	0.01945	0.23509	2093.43	3063.79	2091.48	1703.22	1944.35	1546.52 Down
Slc6a4	558.072	0.5592	0.26336	2.1233	0.03373	0.32401	361.718	549.861	442.259	509.841	864.707	620.044 Up
Efcab5	190.178	-0.6575	0.27626	-2.3802	0.0173	0.21738	211.906	281.853	204.569	114.433	137.68	190.625 Down
Coro6	907.632	-1.3344	0.37376	-3.5702	0.00036	0.01467	880.149	2050.11	969.268	528.232	669.579	348.454 Down
Spag5	145.174	0.75935	0.27589	2.75242	0.00592	0.10726	115.316	110.763	97.4139	232.953	178.29	136.307 Up
Lyrm9	486.744	-0.5037	0.16228	-3.1039	0.00191	0.04907	560.811	618.099	533.828	372.93	456.621	378.176 Down
Lgals9	6930.22	0.36421	0.15546	2.34286	0.01914	0.23229	5926.47	5859.58	6394.25	7917.35	8950.17	6533.52 Up
Rhbdl3	382.475	-0.682	0.3025	-2.2544	0.02417	0.26772	443.524	585.463	384.785	331.039	363.514	186.526 Down
Spaca3	7.25608	-2.127	0.8877	-2.3961	0.01657	0.212	14.7841	11.8675	8.76725	4.0869	1.981	2.04973 Down
Ccl7	119.817	1.21025	0.33696	3.59165	0.00033	0.01378	71.9494	54.3927	90.5949	192.084	196.119	113.76 Up
Ccl12	130.782	1.1907	0.31174	3.81949	0.00013	0.00678	101.518	78.1277	59.4225	226.823	175.319	143.481 Up
Ccl1	3.56226	4.2692	1.80776	2.3616	0.0182	0.22454	0	0.98896	0	14.3042	1.981	4.09946 Up

Slfn9	230.658	1.20982	0.37172	3.25466	0.00114	0.03405	203.035	90.9841	123.716	398.473	355.59	212.147	Up
Gm11427	162.534	0.80774	0.26616	3.03475	0.00241	0.05757	124.187	99.8848	130.535	191.063	262.483	167.053	Up
Rasl10b	223.509	-1.3559	0.50445	-2.6878	0.00719	0.12138	262.172	572.607	129.56	143.042	147.585	86.0887	Down
Gas2l2	515.327	-0.6983	0.3343	-2.0888	0.03672	0.3402	499.704	826.769	586.431	365.778	249.606	563.676	Down
Ccl5	839.856	0.73178	0.28152	2.59938	0.00934	0.14426	660.358	677.436	556.233	1004.36	1453.06	687.685	Up
Ccl9	1710.8	0.85925	0.36373	2.36236	0.01816	0.22436	937.314	1711.89	998.492	2573.73	2857.59	1185.77	Up
Ccl3	172.389	1.03355	0.37897	2.72722	0.00639	0.11296	171.496	93.951	74.0345	287.105	240.692	167.053	Up
Wfdc17	171.66	1.18822	0.50956	2.33184	0.01971	0.23657	62.0933	180.979	71.1121	240.105	350.637	125.034	Up
Dhrs11	748.829	-0.5722	0.20066	-2.8516	0.00435	0.08771	771.732	1131.37	783.207	592.601	631.94	582.124	Down
Brip1	112.16	0.8682	0.40432	2.1473	0.03177	0.31185	83.7767	101.863	52.6035	163.476	187.205	84.039	Up
Prr11	146.185	1.24256	0.39307	3.16116	0.00157	0.04279	119.259	71.205	70.138	243.171	257.53	115.81	Up
Mtmr4	3073.06	0.33701	0.1667	2.02174	0.0432	0.37341	2525.13	2630.63	2991.58	3366.59	4004.59	2919.84	Up
Mir142hg	281.266	0.37547	0.19122	1.9636	0.04958	0.39948	234.575	284.82	215.285	334.104	330.827	287.987	Up
Tspoap1	885.8	-0.6853	0.32451	-2.1117	0.03471	0.32919	826.926	1361.8	1088.11	632.448	430.868	974.647	Down
Olfr464	38.4408	-1.8929	0.86934	-2.1774	0.02945	0.29843	113.345	9.88958	58.4483	19.4128	4.9525	24.5968	Down
Dynll2	6045.58	-0.2698	0.10079	-2.6774	0.00742	0.12358	6521.77	6587.45	6718.63	5437.62	5171.4	5836.61	Down
Cuedc1	2535.72	-0.2242	0.10761	-2.0837	0.03719	0.34382	2709.44	2685.02	2802.6	2250.86	2251.41	2515.02	Down
Akap1	2079.69	-0.239	0.10922	-2.1878	0.02868	0.29288	2245.22	2281.53	2227.85	1773.72	1920.58	2029.23	Down
Tmem100	39372.1	0.31759	0.16116	1.97059	0.04877	0.39549	30013.8	34138.8	41015.1	47277.3	43667.2	40120.4	Up
B230206L	164.701	-0.8756	0.32072	-2.7302	0.00633	0.1124	195.151	203.725	240.612	132.824	69.3351	146.556	Down
Cacna1g	33.6486	-1.3974	0.5889	-2.3728	0.01765	0.22022	23.6546	62.3044	60.3966	24.5214	21.791	9.22379	Down
Sgca	162.391	-3.1175	0.60462	-5.1561	2.52E-07	4.06E-05	183.323	568.651	121.767	30.6518	51.506	18.4476	Down
Pdk2	2571	-0.422	0.17993	-2.3451	0.01902	0.23116	2779.42	3592.88	2460.67	2157.88	2082.03	2353.09	Down
Atp5g1	3252.23	-0.8351	0.26483	-3.1532	0.00161	0.04332	3255.47	5926.83	3321.81	2302.97	2064.2	2642.1	Down
Hoxb13	3.78352	-3.3449	1.68493	-1.9852	0.04713	0.38902	1.97122	11.8675	6.81897	2.04345	0	0	Down
Skap1	615.756	0.60589	0.19255	3.14667	0.00165	0.04403	463.236	479.645	522.138	815.337	831.03	583.149	Up
Ppp1r1b	153.71	-0.9699	0.40512	-2.3942	0.01666	0.21248	206.978	283.831	119.819	67.4339	139.661	104.536	Down
Tcap	2552.26	-3.1564	0.98935	-3.1904	0.00142	0.03994	5205	6478.66	2085.63	107.281	1148.98	287.987	Down
Thra	3482.16	-0.2602	0.11908	-2.185	0.02889	0.29455	3430.9	3981.55	3973.51	3086.63	3320.16	3100.22	Down
Nr1d1	10981.6	0.46886	0.20808	2.2533	0.02424	0.26823	12085.5	7506.19	8046.38	13241.6	12610.1	12399.9	Up
Cdc6	161.691	0.7681	0.34666	2.21574	0.02671	0.28205	165.582	95.9289	97.4139	266.67	201.072	143.481	Up
Top2a	736.386	1.51896	0.3345	4.54093	5.60E-06	0.00056	358.762	455.91	328.285	1394.66	1297.56	583.149	Up
Krt222	100.633	-1.3103	0.47013	-2.7871	0.00532	0.10049	92.6472	238.339	99.3621	58.2383	76.2686	38.9449	Down
A830036E	13.8823	1.8945	0.94712	2.00027	0.04547	0.38155	9.85609	0	7.79311	14.3042	26.7435	24.5968	Up
Krt34	3.19959	5.14556	2.44631	2.1034	0.03543	0.33353	0	0	0	0	7.92401	11.2735	Up
Krt15	87.186	-4.4074	1.93594	-2.2766	0.02281	0.25801	5.91365	439.097	54.5518	6.13035	0	17.4227	Down
Krt9	56.7644	-0.8874	0.30344	-2.9245	0.00345	0.07465	67.0214	72.1939	81.8276	38.8256	36.6485	44.0692	Down
Kcnh4	63.718	1.62925	0.66744	2.44104	0.01465	0.19355	52.2373	17.8012	23.3793	83.7815	173.338	31.7708	Up
Aoc3	510.477	-2.239	0.45054	-4.9697	6.71E-07	9.66E-05	512.517	1523	491.94	149.172	151.547	234.694	Down
Brca1	124.955	0.9498	0.29424	3.22797	0.00125	0.03633	96.5897	90.9841	68.1897	207.41	159.471	127.083	Up
Sost	15.8065	-3.0684	1.05032	-2.9214	0.00349	0.07481	10.8417	56.3706	17.5345	6.13035	3.962	0	Down
Nags	42.0256	-1.5206	0.5921	-2.5681	0.01022	0.15345	94.6184	49.4479	42.8621	29.63	7.92401	27.6714	Down
2810433D	163.994	0.66005	0.31889	2.06984	0.03847	0.35169	172.482	89.9952	118.845	238.062	155.509	209.073	Up
Kif18b	115.209	1.11591	0.36389	3.06665	0.00216	0.05343	63.079	85.0504	70.138	207.41	173.338	92.2379	Up
Myl4	10468.9	-4.239	1.2356	-3.4307	0.0006	0.02181	11188.6	36765.5	11700.4	270.757	2619.87	268.515	Down
Ace	30777.1	0.34504	0.12661	2.72519	0.00643	0.11347	27610.8	27062.8	26669	35489.6	37642	30188.4	Up
Taco1	382.691	-0.3291	0.16748	-1.9652	0.04939	0.39825	420.855	479.645	377.966	360.669	322.903	334.106	Down
Limd2	5724.74	0.26597	0.10699	2.48603	0.01292	0.17801	5556.86	5083.24	4955.44	6143.63	6046.02	6563.24	Up
Scn4a	106.793	-1.3882	0.35385	-3.923	8.75E-05	0.00483	129.115	221.527	113	68.4556	58.4396	50.2184	Down
Prr29	886.378	-0.7303	0.26679	-2.7374	0.00619	0.11033	884.091	1408.28	1025.77	537.428	584.396	878.31	Down
Milr1	230.607	0.83122	0.37458	2.21908	0.02648	0.28143	182.338	193.836	121.767	266.67	442.754	176.277	Up
Gm11677	3.11847	-5.0656	1.79732	-2.8184	0.00483	0.09356	5.91365	8.90062	3.89655	0	0	0	Down
BC006965	3.46507	-3.2038	1.59406	-2.0098	0.04445	0.37865	4.92804	11.8675	1.94828	1.02173	0	1.02487	Down
Sox9	34.4577	-2.712	0.67693	-4.0063	6.17E-05	0.0037	37.4531	108.785	33.1207	14.3042	6.93351	6.1492	Down
2610035D	291.364	1.12205	0.32079	3.49774	0.00047	0.01809	138.971	215.593	195.802	484.298	466.526	246.993	Up
Kif19a	1486.06	-0.6666	0.27083	-2.4613	0.01385	0.18672	1643.01	2056.04	1770.98	1023.77	804.287	1618.26	Down
Cd300a	713.767	0.79504	0.40187	1.97837	0.04789	0.39216	559.826	568.651	437.388	566.036	1579.85	570.85	Up
Cd300ld	402.641	1.11623	0.3433	3.2515	0.00115	0.03429	298.639	285.809	178.267	439.342	817.163	396.623	Up
Cd300c2	1014.32	1.33472	0.35925	3.71525	0.0002	0.00951	730.336	589.419	408.164	1168.85	2252.4	936.727	Up
Cd300e	301.94	1.04212	0.5004	2.08255	0.03729	0.34397	189.237	241.306	161.707	247.258	781.505	190.625	Up
Rab37	117.671	0.79227	0.30774	2.57449	0.01004	0.15202	88.7048	86.0394	83.7759	125.672	206.024	115.81	Up
Tmem104	1180.08	0.23827	0.10892	2.18752	0.0287	0.29295	1078.26	1106.64	1063.76	1227.09	1343.12	1261.61	Up
Tsen54	1221.14	-0.4033	0.14371	-2.8067	0.00501	0.09615	1306.92	1389.49	1475.82	918.531	1098.47	1137.6	Down
Myo15b	639.531	-1.2848	0.28338	-4.5337	5.80E-06	0.00057	820.026	969.179	931.276	264.627	312.998	539.079	Down
Ubald2	1551.12	0.27839	0.10642	2.61607	0.00889	0.14016	1364.08	1433	1408.6	1717.52	1611.54	1771.99	Up
Mxra7	4153.06	0.25814	0.1084	2.38131	0.01725	0.21705	3672.38	3894.52	3780.63	4802.11	4579.09	4189.65	Up
Gm11734	211.964	-0.9331	0.38383	-2.431	0.01506	0.19718	188.251	356.025	290.293	135.889	88.1546	213.172	Down
Birc5	217.538	1.11494	0.34606	3.22182	0.00127	0.03687	163.611	149.333	99.3621	417.886	280.312	194.725	Up
Cep131	700.137	-0.3115	0.15325	-2.0327	0.04208	0.37095	750.048	777.321	798.794	593.622	562.604	718.431	Down
Gcgr	95.6692	-2.3605	0.44742	-5.2757	1.32E-07	2.40E-05	194.165	116.697	169.5	27.5866	15.848	50.2184	Down
Myadml2	87.6348	-2.8901	0.61184	-4.7237	2.32E-06	0.00026	90.676	297.676	75.0087	20.4345	29.715	12.2984	Down
Metrnl	938.932	0.43647	0.21645	2.01649	0.04375	0.37549	937.314	869.294	587.406	972.683	1184.64	1082.26	Up
Gm12586	26.7928	-8.1688	3.90839	-2.0901	0.03661	0.3402	85.748	0	75.0087	0	0	0	Down
Pomc	13.8868	-1.3687	0.66879	-2.0465	0.04071	0.36296	16.7553	30.6577	12.6638	6.13035	8.91451	8.19893	Down
Fam228b	69.803	-0.7429	0.34264	-2.1681	0.03015	0.30293	66.0358	88.0173	108.129	39.8473	60.4206	56.3676	Down
Apob	228.495	2.17815	0.50632	4.30189	1.69E-05	0.00128	80.8199	115.708	51.6293	187.997	675.522	259.291	Up
Osr1	511.648	-0.5955	0.16664	-3.5736	0.00035	0.01458	516.459	661.613	669.233	410.734	409.077	402.772	Down
Gen1	83.7062	0.96353	0.40786	2.36241	0.01816	0.22436	50.266	77.1387	42.8621	154.281	106.974	70.7157	Up
Gm4294	212.271	-0.5476	0.21899	-2.5006	0.0124	0.17252	277.942	260.096	218.207	146.107	169.376	201.899	Down
Greb1	63.303	-1.1442	0.37329	-3.0652	0.00218	0.05356	98.5609	84.0614	78.9052	23.4997	44.5725	50.2184	Down
E2f6	2270.96	-0.2542	0.12586	-2.0195	0.04343	0.37418	2352.65	2684.03	2374.95	2047.54	2230.61	1935.97	Down
Gm36495	3.7783	-4.3143	1.60986	-2.6799	0.00736	0.1232	5.91365	9.88958	5.84483	1.02173	0	0	Down
Atp6v1c2	5220.31	0.4709	0.21458	2.19456	0.0282	0.28986	5070.96	3790.68	4265.75	5510.16	4976.28	7708.02	Up

Odc1	7181.52	0.38887	0.16871	2.30492	0.02117	0.24807	6638.07	6133.52	5886.72	7595.51	7005.81	9829.49	Up
Gm36862	6.72788	-4.255	1.45131	-2.9318	0.00337	0.07357	5.91365	21.7571	10.7155	0	1.981	0	Down
Gm46313	3.27286	-5.1358	1.74357	-2.9456	0.00322	0.07154	6.89926	4.94479	7.79311	0	0	0	Down
Gm46338	1.96883	-4.4022	2.04839	-2.1491	0.03163	NA	2.95683	5.93375	2.92242	0	0	0	Down
Gm5784	26.3312	2.30494	0.78545	2.93453	0.00334	0.07329	19.7122	4.94479	1.94828	54.1514	48.5345	28.6962	Up
Gm9259	4.90686	-5.7202	1.61456	-3.5429	0.0004	0.01595	5.91365	9.88958	13.6379	0	0	0	Down
Gm49692	135.869	-2.4062	0.27975	-8.6014	7.88E-18	8.10E-15	179.381	258.118	248.405	38.8256	50.5155	39.9698	Down
Gm48693	43.8747	-0.9813	0.48676	-2.0161	0.04379	0.37573	53.2229	53.4037	68.1897	23.4997	48.5345	16.3979	Down
1700030C	225.159	-1.0832	0.41008	-2.6415	0.00825	0.13283	142.913	421.296	353.612	148.15	175.319	109.661	Down
Cpsf3	3859.86	0.45183	0.13378	3.37739	0.00073	0.02509	3630	3136.98	3013.98	4394.44	4171	4812.77	Up
Iah1	2002.26	1.23934	0.2652	4.67316	2.97E-06	0.00032	1720.87	899.952	953.682	2810.77	2725.86	2902.42	Up
Adam17	2865.54	0.25903	0.12942	2.00152	0.04534	0.38132	2607.92	2580.19	2638.94	3163.26	3443.97	2758.94	Up
Gm49586	4.08737	-4.4355	1.60842	-2.7577	0.00582	0.10618	6.89926	4.94479	11.6897	0	0.9905	0	Down
Ywhaq	14413.8	0.28712	0.13083	2.19463	0.02819	0.28986	12911.5	13088.9	12952.1	16039	13852.2	17639	Up
Gm4036	13.1488	-1.9504	0.90077	-2.1653	0.03037	0.30443	10.8417	12.8565	38.9655	10.2173	3.962	2.04973	Down
Gm20336	4.76834	-3.709	1.51153	-2.4538	0.01414	0.18925	4.92804	13.8454	7.79311	2.04345	0	0	Down
Gm9292	10.5075	-4.3091	1.11089	-3.879	0.0001	0.00561	14.7841	29.6687	15.5862	0	1.981	1.02487	Down
Gm6969	293.038	-2.4506	0.6207	-3.9482	7.88E-05	0.00445	795.386	605.242	85.7242	100.129	101.031	70.7157	Down
Rrm2	340.678	1.359	0.33318	4.07883	4.53E-05	0.00291	189.237	218.56	165.604	633.47	564.585	272.614	Up
Mboat2	253.705	-0.9714	0.34206	-2.8397	0.00452	0.0902	237.532	522.17	248.405	171.65	172.347	170.128	Down
Kidins220	6079.95	0.24031	0.12079	1.98943	0.04665	0.38675	6206.38	5343.34	5174.62	6597.28	6740.36	6417.71	Up
Id2	4130.46	0.64801	0.12314	5.26235	1.42E-07	2.56E-05	3528.48	3222.03	2903.91	4845.02	5175.37	5107.93	Up
Gm17746	4.09293	-5.4583	1.93883	-2.8153	0.00487	0.09431	0.98561	12.8565	10.7155	0	0	0	Down
Gm36723	168.529	1.26483	0.37485	3.37419	0.00074	0.02526	142.913	89.9952	64.2931	228.866	314.979	170.128	Up
Rnf144a	7866.12	0.32012	0.14752	2.16997	0.03001	0.30184	7288.58	6111.76	7590.49	9633.85	7918.06	8653.97	Up
Cmpk2	1540.14	0.27328	0.12589	2.17087	0.02994	0.30129	1302.97	1536.84	1344.31	1634.76	1656.12	1765.84	Up
Sox11	849.369	0.88221	0.26923	3.27675	0.00105	0.03233	800.314	416.351	575.716	1248.55	903.337	1151.95	Up
Gm31333	7.0227	2.12403	0.89537	2.37222	0.01768	0.22046	2.95683	2.96687	1.94828	10.2173	15.848	8.19893	Up
Slc26a4	611.761	1.55967	0.41078	3.79686	0.00015	0.00729	388.33	316.467	225.026	843.945	1454.06	442.742	Up
Pik3cg	709.954	0.94388	0.37934	2.48823	0.01284	0.17726	599.25	492.501	365.302	741.773	1509.52	551.378	Up
Twist1	18.5264	-2.4403	1.10755	-2.2033	0.02757	0.28707	11.8273	78.1277	3.89655	4.0869	2.9715	10.2487	Down
Agr2	156.225	-2.7631	1.09642	-2.5201	0.01173	0.16717	142.913	582.496	91.569	16.3476	14.8575	89.1633	Down
Lsmem1	48.8043	-1.8542	0.60386	-3.0706	0.00214	0.05296	38.4387	134.498	56.5	14.3042	32.6865	16.3979	Down
Nrcam	555.87	-1.0118	0.51158	-1.9778	0.04796	0.39243	200.079	1191.69	837.759	409.712	381.343	314.634	Down
Gm48508	2.35932	3.63952	1.79167	2.03135	0.04222	0.37137	0	0.98896	0	6.13035	3.962	3.0746	Up
Nova1	843.601	-0.6777	0.16198	-4.184	2.86E-05	0.00202	868.321	1157.08	1089.09	654.926	625.006	667.188	Down
Coch	115.171	-1.7748	0.44669	-3.9732	7.09E-05	0.00409	143.899	279.875	111.052	36.7821	73.2971	46.119	Down
Gm35188	306.515	-0.4303	0.17629	-2.441	0.01465	0.19355	330.179	374.815	350.69	227.845	263.473	292.087	Down
Akap6	531.24	-1.7996	0.42042	-4.2804	1.87E-05	0.00138	551.941	1451.79	472.457	194.128	278.331	238.794	Down
Baz1a	2036.75	0.47157	0.19162	2.4609	0.01386	0.18672	1896.31	1613.98	1610.25	2372.45	2841.75	1885.75	Up
Gm49376	143.172	0.61809	0.22691	2.72399	0.00645	0.1138	104.475	104.83	129.56	194.128	173.338	152.705	Up
Gm38103	41.8265	1.18245	0.40194	2.94181	0.00326	0.07204	33.5107	26.7019	16.5604	65.3904	54.4775	54.3179	Up
Pax9	66.8968	-4.6666	1.51724	-3.0757	0.0021	0.05218	5.91365	348.113	32.1466	9.19553	3.962	2.04973	Down
Gm2568	58.349	-1.2866	0.58363	-2.2044	0.02749	0.28689	58.1509	140.432	49.6811	34.7387	15.848	51.2433	Down
Gm22973	7.03853	-4.3076	1.39682	-3.0839	0.00204	0.05154	9.85609	8.90062	21.431	2.04345	0	0	Down
Gm527	234.065	-0.4573	0.21643	-2.1129	0.03461	0.32907	301.596	230.427	280.552	223.758	203.053	165.003	Down
Mdga2	33.0239	-1.7405	0.67574	-2.5757	0.01	0.15172	25.6258	80.1056	46.7587	6.13035	12.8765	26.6465	Down
Rn7s1	6.64121	6.1988	2.97244	2.08543	0.03703	0.34277	0	0	0	39.8473	0	0	Up
Lrr1	19.4666	1.83794	0.72724	2.5273	0.01149	0.16493	4.92804	8.90062	11.6897	54.1514	22.7815	14.3481	Up
Rn7s2	5.9417	3.47051	1.7695	1.9613	0.04984	0.39986	0.98561	0.98896	0.97414	30.6518	0	2.04973	Up
L2hgdh	755.278	-0.303	0.15152	-1.9996	0.04555	0.38182	814.113	951.378	737.423	679.447	662.645	686.66	Down
Atl1	344.344	-0.4856	0.16906	-2.8725	0.00407	0.08354	373.546	440.086	391.604	267.692	273.378	319.758	Down
Gm48695	3.94439	-2.7635	1.30903	-2.1111	0.03476	0.32926	6.89926	5.93375	7.79311	0	0.9905	2.04973	Down
Abhd12b	238.019	-1.801	0.59868	-3.0083	0.00263	0.06102	333.136	329.323	447.13	65.3904	29.715	223.421	Down
Gm24474	67.2713	-1.4527	0.53854	-2.6976	0.00698	0.11924	76.8775	169.112	49.6811	39.8473	45.563	22.547	Down
Gm40437	142.616	-0.5749	0.20758	-2.7695	0.00561	0.10469	170.51	170.101	171.448	103.194	129.756	110.686	Down
L3hypdh	385.672	-0.4038	0.20474	-1.9722	0.04859	0.39479	469.15	481.623	367.25	314.691	390.257	291.062	Down
Six1	864.074	-0.7838	0.39522	-1.9831	0.04735	0.39	974.767	1290.59	1014.08	538.449	312.998	1053.56	Down
Trmt5	723.117	-0.4532	0.17285	-2.6219	0.00874	0.13855	762.861	952.367	791.975	610.992	534.87	685.635	Down
D830013C	44.9282	-2.2213	0.75421	-2.9452	0.00323	0.07157	56.1797	142.41	23.3793	6.13035	15.848	25.6216	Down
Prkch	5908.67	0.34592	0.14575	2.3734	0.01763	0.22001	5274.98	4779.63	5556.49	6783.23	7328.72	5729	Up
Rhoj	4568.68	0.61522	0.17546	3.50627	0.00045	0.01765	4115.9	3021.27	3690.04	5676.71	6129.22	4778.95	Up
Esr2	217.02	0.48217	0.22551	2.13815	0.0325	0.31662	149.813	183.946	209.44	294.257	224.844	239.819	Up
Sptb	498.904	-0.6688	0.33731	-1.9826	0.04741	0.39017	464.222	963.245	410.112	350.452	417.991	387.399	Down
Gm4864	81.384	-0.9189	0.36012	-2.5515	0.01072	0.15787	145.87	73.1829	100.336	45.9776	60.4206	62.5168	Down
Arg2	563.284	0.65468	0.25943	2.52358	0.01162	0.16614	534.2	419.318	359.457	692.73	866.688	507.309	Up
Rad51b	627.086	-0.6228	0.31049	-2.0058	0.04488	0.38019	616.991	988.958	675.078	486.341	324.884	670.262	Down
Srsf5	29477.1	-0.2374	0.10956	-2.1664	0.03028	0.30382	30695.8	30287.8	34705.6	27251.5	26002.6	27919.4	Down
Smoc1	353.903	-1.8673	0.52608	-3.5495	0.00039	0.01561	356.79	1079.94	229.897	111.368	146.594	198.824	Down
Pnma1	157.446	-0.5379	0.24767	-2.172	0.02986	0.30087	158.683	212.626	188.009	123.629	106.974	154.755	Down
Npc2	62517.4	0.46288	0.18322	2.52643	0.01152	0.16512	63623	49224.4	44872.7	65360.8	82335.4	69687.8	Up
Fos	10203.1	-1.274	0.36085	-3.5306	0.00041	0.0164	11715.9	19388.5	12205	8187.09	3035.89	6686.22	Down
Batf	382.183	0.75338	0.17581	4.28507	1.83E-05	0.00136	259.215	287.787	306.854	479.189	538.832	421.22	Up
Flvcr2	1024.66	0.92292	0.2902	3.18026	0.00147	0.04089	826.926	682.381	613.707	1522.37	1688.8	813.743	Up
Gm805	13.0147	-1.4232	0.69018	-2.0621	0.0392	0.35628	16.7553	12.8565	27.2759	4.0869	8.91451	8.19893	Down
Esrrb	31.8976	-3.1049	1.07244	-2.8951	0.00379	0.07951	44.3524	99.8848	27.2759	2.04345	17.829	0	Down
Ism2	36.5822	-0.858	0.38819	-2.2103	0.02708	0.28467	40.41	44.5031	56.5	25.5431	21.791	30.746	Down
Dio2	93.8558	-2.2365	0.74519	-3.0013	0.00269	0.06203	47.3092	349.102	68.1897	26.5649	52.4965	19.4725	Down
Tshr	65.9874	-2.6312	0.64556	-4.0759	4.58E-05	0.00293	79.8343	190.869	70.138	28.6083	4.9525	21.5222	Down
Gm19951	1157.41	2.46384	0.73768	3.33999	0.00084	0.02771	342.992	443.053	279.578	3951.01	1239.12	688.71	Up
Gm18795	13.0158	-3.1281	1.275	-2.4534	0.01415	0.18932	9.85609	55.3817	4.87069	2.04345	5.94301	0	Down
Chga	16.3315	-1.6751	0.64231	-2.6079	0.00911	0.14214	22.669	18.7902	33.1207	8.1738	3.962	11.2735	Down
Asb2	717.342	-1.8464	0.49747	-3.7115	0.00021	0.00961	518.43	2134.17	715.018	222.736	431.858	281.838	Down

Ifi27l2a	1607.25	-0.7621	0.34016	-2.2405	0.02506	0.27344	1577.96	3172.58	1316.06	949.183	1419.39	1208.32 Down
Gm47267	7.0896	-2.0852	0.8758	-2.3809	0.01727	0.2171	10.8417	12.8565	10.7155	2.04345	1.981	4.09946 Down
Ppp4r4	1326.01	-0.5539	0.18879	-2.9342	0.00334	0.07329	1324.66	1587.28	1820.66	1082.01	1225.25	916.23 Down
Serpina3g	679.53	1.14183	0.4671	2.44453	0.0145	0.19287	487.876	486.567	297.112	854.162	1591.73	359.728 Up
Gm47648	58.5549	-0.9245	0.33689	-2.7443	0.00606	0.10885	71.9494	91.9731	66.2414	32.6952	49.525	38.9449 Down
Bdkrb1	147.263	-1.1456	0.23655	-4.843	1.28E-06	0.00016	180.366	233.394	194.828	98.0856	100.041	76.8649 Down
Bcl11b	297.219	0.65465	0.2296	2.85118	0.00436	0.08774	233.589	218.56	240.612	444.451	371.438	274.664 Up
Cyp46a1	91.0858	-0.5149	0.25127	-2.0491	0.04045	0.36231	112.359	106.807	102.285	83.7815	63.3921	77.8898 Down
Evl	299.539	0.67465	0.32173	2.09693	0.036	0.33698	296.668	205.703	189.957	367.821	496.241	240.843 Up
Ankrd9	413.234	-0.9089	0.19259	-4.719	2.37E-06	0.00027	555.883	583.485	478.302	275.866	246.635	339.231 Down
Gm48630	4.11344	-3.5011	1.72077	-2.0346	0.04189	0.36989	4.92804	15.8233	1.94828	0	1.981	0 Down
Lbhd2	4.8504	3.78362	1.63127	2.31943	0.02037	0.24185	0	1.97792	0	10.2173	14.8575	2.04973 Up
Ckb	4102.98	-0.7663	0.2745	-2.7917	0.00524	0.09941	3427.95	6880.18	5195.08	2873.09	2566.39	3675.17 Down
A530016L	60.6746	-6.4871	1.10585	-5.8662	4.46E-09	1.28E-06	55.1941	259.107	45.7845	0	3.962	0 Down
Tmem179	29.799	-5.4347	1.2137	-4.4778	7.54E-06	0.0007	17.741	142.41	14.6121	0	1.981	2.04973 Down
Igha	15455.3	3.39526	0.46092	7.36629	1.75E-13	1.05E-10	1273.41	4298.01	2477.23	34487.3	36634.7	13561 Up
Ighe	9.07334	4.74046	1.49207	3.1771	0.00149	0.04114	0.98561	0	0.97414	29.63	20.8005	2.04973 Up
Ighg2c	7911.55	6.90647	1.29732	5.32363	1.02E-07	1.93E-05	97.5753	239.328	55.5259	13510.3	32871.8	694.859 Up
Ighg2b	4169.45	5.03884	1.27246	3.95991	7.50E-05	0.00426	87.7192	266.03	384.785	6280.55	17577.4	420.195 Up
Ighg1	1352.19	5.92124	1.25664	4.71194	2.45E-06	0.00028	16.7553	65.2712	49.6811	3044.74	4817.8	118.884 Up
Ighg3	928.227	5.07528	1.03237	4.91616	8.83E-07	0.00012	114.331	21.7571	24.3535	1758.39	3191.39	459.14 Up
Ighm	28936.1	2.20886	1.04548	2.11276	0.03462	0.32907	17229.4	7714.86	5931.53	26059.1	107646	9036.24 Up
Ighv2-2	23.3048	2.37923	0.87654	2.71433	0.00664	0.11573	7.88487	2.96687	11.6897	36.7821	72.3066	8.19893 Up
Ighv5-4	17.9291	2.25239	1.14534	1.96657	0.04923	0.39755	9.85609	2.96687	5.84483	23.4997	64.3826	1.02487 Up
Ighv5-6	21.3884	3.30334	0.7851	4.20754	2.58E-05	0.00184	4.92804	4.94479	1.94828	34.7387	65.3731	16.3979 Up
Ighv5-9	35.5809	5.13519	2.02906	2.53082	0.01138	0.16459	2.95683	1.97792	0.97414	18.3911	189.186	0 Up
Ighv2-5	18.7247	3.38593	1.22676	2.76005	0.00578	0.10589	3.94243	0.98896	4.87069	1.02173	27.734	73.7903 Up
Ighv5-12	5.58854	4.01235	1.8683	2.14759	0.03175	0.31185	0	0	1.94828	27.5866	2.9715	1.02487 Up
Ighv2-9-1	26.2247	3.6213	1.05871	3.42048	0.00063	0.02243	3.94243	6.92271	0.97414	57.2166	84.1926	4.09946 Up
Ighv5-17	88.0201	3.31211	0.93695	3.535	0.00041	0.01622	11.8273	30.6577	5.84483	91.9553	371.438	16.3979 Up
Ighv3-1	10.031	4.88885	1.7051	2.86719	0.00414	0.08461	0	0.98896	0.97414	52.108	0.9905	5.12433 Up
Ighv14-2	156.143	5.46953	1.26692	4.31721	1.58E-05	0.00121	16.7553	1.97792	1.94828	588.514	274.369	53.293 Up
Ighv14-3	15.7769	2.67556	0.82712	3.23481	0.00122	0.03573	2.95683	8.90062	0.97414	16.3476	32.6865	32.7957 Up
Ighv9-3	64.0904	2.49376	0.68573	3.63664	0.00028	0.01205	25.6258	14.8344	17.5345	88.8901	209.986	27.6714 Up
Ighv14-4	16.5703	3.03528	1.11679	2.71787	0.00657	0.11517	4.92804	2.96687	2.92242	10.2173	74.2876	4.09946 Up
Ighv3-6	385.676	4.9637	1.16376	4.26524	2.00E-05	0.00146	28.5827	27.6908	15.5862	584.427	1608.57	49.1936 Up
Ighv13-2	30.865	4.93011	2.45882	2.00507	0.04496	0.38034	2.95683	0	2.92242	13.2824	0	166.028 Up
Ighv6-3	44.4408	2.49082	0.60152	4.14085	3.46E-05	0.00236	21.6834	5.93375	12.6638	68.4556	115.889	42.0195 Up
Ighv6-6	54.9118	1.64577	0.72189	2.2798	0.02262	0.25695	24.6402	40.5473	14.6121	169.606	55.468	24.5968 Up
Ighv1-4	16.8049	4.00617	0.9026	4.43849	9.06E-06	0.00078	2.95683	2.96687	0	40.869	37.639	16.3979 Up
Ighv1-7	17.9849	3.31629	0.9993	3.31861	0.0009	0.02938	1.97122	5.93375	1.94828	5.10863	38.6295	54.3179 Up
Ighv1-9	129.207	4.87291	0.6844	7.12001	1.08E-12	5.42E-10	15.7697	5.93375	3.89655	84.8032	451.668	213.172 Up
Ighv1-18	77.3868	3.64513	0.99451	3.66527	0.00025	0.01109	15.7697	5.93375	12.6638	92.977	330.827	6.1492 Up
Ighv1-19	44.3769	4.19746	0.6314	6.64787	2.97E-11	1.28E-08	2.95683	6.92271	3.89655	71.5208	130.746	50.2184 Up
Ighv1-22	20.0235	2.44455	0.71302	3.42842	0.00061	0.02196	6.89926	3.95583	7.79311	30.6518	55.468	15.373 Up
Ighv1-34	11.2944	4.02155	1.37343	2.9281	0.00341	0.07409	1.97122	0.98896	0.97414	13.2824	49.525	1.02487 Up
Ighv1-36	2.35332	4.70276	2.09898	2.2405	0.02506	0.27344	0	0	0	7.15208	5.94301	1.02487 Up
Ighv1-39	68.774	3.29809	1.49742	2.20252	0.02763	0.28707	2.95683	2.96687	32.1466	193.106	175.319	6.1492 Up
Ighv1-50	24.1204	3.14244	0.99768	3.14977	0.00163	0.04367	1.97122	5.93375	6.81897	34.7387	90.1356	5.12433 Up
Ighv1-52	71.3586	3.18664	0.90896	3.50581	0.00046	0.01765	23.6546	14.8344	3.89655	197.193	177.3	11.2735 Up
Ighv1-53	381.159	4.43477	1.27325	3.48303	0.0005	0.01885	19.7122	33.6246	47.7328	165.52	1939.4	80.9644 Up
Ighv1-55	156.417	3.30277	1.18554	2.78588	0.00534	0.10063	37.4531	12.8565	36.0431	283.018	551.709	17.4227 Up
Ighv8-8	71.5612	3.56384	0.66548	5.35527	8.54E-08	1.63E-05	21.6834	7.91166	3.89655	69.4773	104.003	222.396 Up
Ighv1-59	32.863	2.94719	1.1538	2.55434	0.01064	0.15715	17.741	1.97792	2.92242	34.7387	135.699	4.09946 Up
Ighv1-61	8.04534	3.49545	1.24289	2.81235	0.00492	0.095	1.97122	0.98896	0.97414	22.478	19.81	2.04973 Up
Ighv1-62-	17.0738	3.59168	1.4046	2.55708	0.01056	0.15671	1.97122	1.97792	3.89655	16.3476	78.2496	0 Up
Ighv1-64	217.37	4.02235	0.36378	11.0572	2.02E-28	3.36E-25	17.741	23.735	34.0948	562.971	368.466	297.211 Up
Ighv1-69	45.9783	2.16898	0.82998	2.6133	0.00897	0.14073	23.6546	16.8123	9.74139	15.3259	170.366	39.9698 Up
Ighv8-12	35.1862	1.72032	0.60573	2.84007	0.00451	0.09019	27.597	9.88958	11.6897	45.9776	84.1926	31.7708 Up
Ighv1-72	54.9999	3.76621	0.9786	3.84855	0.00012	0.00618	4.92804	8.90062	8.76725	69.4773	231.777	6.1492 Up
Ighv1-74	28.5739	3.30896	0.94031	3.519	0.00043	0.01692	3.94243	4.94479	6.81897	123.629	19.81	12.2984 Up
Ighv1-75	25.8452	3.0459	1.13789	2.67678	0.00743	0.12367	9.85609	5.93375	0.97414	8.1738	118.86	11.2735 Up
Ighv1-76	69.9876	2.50752	0.6132	4.08924	4.33E-05	0.00281	19.7122	13.8454	29.2242	223.758	84.1926	49.1936 Up
Ighv1-78	27.4119	2.80355	0.92312	3.03704	0.00239	0.05726	10.8417	0.98896	8.76725	91.9553	39.62	12.2984 Up
Ighv1-80	108.931	3.96308	0.85119	4.65594	3.23E-06	0.00035	26.6114	7.91166	4.87069	331.039	257.53	25.6216 Up
Ighv1-81	405.569	6.55497	1.54912	4.23143	2.32E-05	0.00167	7.88487	12.8565	4.87069	204.345	2186.04	17.4227 Up
Ighv1-82	163.382	3.74733	1.2088	3.10003	0.00194	0.04951	43.3668	14.8344	9.74139	466.928	424.925	20.4973 Up
Gm9260	86.5572	-4.06	0.53802	-7.5462	4.48E-14	3.12E-11	77.8631	196.803	215.285	6.13035	8.91451	14.3481 Down
Vipr2	1424.68	-0.5811	0.23236	-2.5007	0.01239	0.17252	1582.89	1727.71	1812.87	1056.46	1515.47	852.688 Down
Ncapg2	357.754	0.56773	0.27728	2.04754	0.0406	0.36253	309.481	266.03	289.319	477.146	530.908	273.639 Up
Ptprn2	219.217	-0.7666	0.31465	-2.4363	0.01484	0.19554	208.949	378.771	240.612	150.194	129.756	207.023 Down
Gm5441	200.548	-0.8668	0.4271	-2.0296	0.0424	0.37174	172.482	435.142	169.5	128.737	111.927	185.501 Down
Cdca7l	359.393	0.50121	0.24934	2.01016	0.04441	0.37851	350.877	283.831	258.147	414.82	528.927	319.758 Up
Calml3	534.552	-2.6594	1.18517	-2.2439	0.02484	0.27245	199.093	2283.5	286.397	89.9118	60.4206	287.987 Down
Gm46401	18.6656	1.61747	0.6647	2.4334	0.01496	0.19658	4.92804	11.8675	10.7155	32.6952	13.867	37.92 Up
Idi2	3.2282	5.15736	1.90264	2.71064	0.00672	0.11632	0	0	0	11.239	1.981	6.1492 Up
Gm24187	9.63565	2.28222	1.15841	1.97013	0.04882	0.39577	3.94243	4.94479	0.97414	36.7821	2.9715	8.19893 Up
Ryr2	1970.08	-3.4194	0.47906	-7.1378	9.49E-13	5.00E-10	2513.3	6508.33	1788.52	183.911	432.849	393.548 Down
Mtr	1856	-0.4268	0.18306	-2.3313	0.01974	0.23669	1911.1	2624.69	1849.89	1586.74	1688.8	1474.78 Down
Actn2	2324.15	-3.8356	1.23905	-3.0956	0.00196	0.05002	2322.09	8280.55	2429.5	41.8907	745.847	125.034 Down
Gpr137b-	2336.19	0.4288	0.20666	2.0749	0.038	0.34842	1471.51	2254.82	2248.31	2487.9	2959.62	2594.96 Up
Gm2423	199.65	-0.4907	0.2164	-2.2677	0.02335	0.2617	248.373	232.405	219.181	166.541	196.119	135.282 Down
Gm48496	22.1556	-3.9431	0.6856	-5.7513	8.86E-09	2.40E-06	40.41	39.5583	44.8104	3.06518	0.9905	4.09946 Down

Amph	1404.09	-0.556	0.21139	-2.6302	0.00853	0.13593	1252.71	1771.22	1990.17	1045.23	1274.77	1090.46 Down
Sfrp4	112.849	-0.8855	0.41655	-2.1257	0.03353	0.32299	71.9494	201.747	165.604	64.3687	82.2116	91.2131 Down
Gpx6	5.79978	6.00473	2.00424	2.99601	0.00274	0.0628	0	0	0	2.04345	30.7055	2.04973 Up
AK157302	51.6006	-2.0946	0.38958	-5.3765	7.59E-08	1.50E-05	93.6328	90.9841	66.2414	14.3042	18.8195	25.6216 Down
Hist1h4n	9.38315	1.76941	0.83104	2.12914	0.03324	0.32163	0.98561	5.93375	5.84483	17.3693	13.867	12.2984 Up
Prss16	47.1411	1.0202	0.39927	2.55519	0.01061	0.15699	44.3524	22.746	26.3017	57.2166	58.4396	73.7903 Up
Vmn1r203	12.3742	-1.9586	0.76857	-2.5484	0.01082	0.15868	14.7841	28.6798	15.5862	2.04345	4.9525	8.19893 Down
Mrs2	442.321	-0.3818	0.18396	-2.0756	0.03793	0.3483	482.948	569.64	449.078	336.148	433.839	382.275 Down
Gm40841	45.5665	-1.2832	0.4735	-2.7101	0.00673	0.11638	51.2517	89.9952	52.6035	24.5214	38.6295	16.3979 Down
Uqcrfs1	5510.3	-0.4682	0.23877	-1.9608	0.0499	0.39986	5747.08	8625.69	4817.12	4353.57	4300.75	5217.59 Down
1700018A	88.1143	-0.6323	0.31728	-1.993	0.04626	0.38485	83.7767	124.609	113	82.7598	73.2971	51.2433 Down
Foxc1	499.186	-0.7815	0.23136	-3.378	0.00073	0.02509	523.358	820.835	549.414	391.321	385.305	324.882 Down
Mylk4	444.608	-4.0019	1.239	-3.23	0.00124	0.03619	342.992	1854.3	313.673	30.6518	118.86	7.17406 Down
Serpinb6b	8276.15	0.29481	0.13092	2.25178	0.02434	0.26895	8099.73	6744.69	7456.06	9606.26	9437.49	8312.69 Up
Gm16984	8.87028	1.81072	0.80361	2.25322	0.02425	0.26823	5.91365	3.95583	1.94828	10.2173	16.8385	14.3481 Up
Ly86	723.012	0.47104	0.21272	2.21438	0.0268	0.28275	613.049	656.668	548.44	898.097	990.501	631.317 Up
Dsp	4595.69	-0.4923	0.17666	-2.7866	0.00533	0.10055	4848.21	6410.43	4858.03	3634.28	3473.69	4349.53 Down
Tfap2a	37.4505	-1.6468	0.66642	-2.4712	0.01347	0.18314	23.6546	103.841	42.8621	11.239	27.734	15.373 Down
Gm28707	11.3889	1.63052	0.6966	2.34068	0.01925	0.23313	3.94243	3.95583	8.76725	16.3476	15.848	19.4725 Up
Cd83	1131.48	0.57425	0.24849	2.31094	0.02084	0.24574	1161.05	800.067	766.647	1281.24	1700.69	1079.18 Up
Gm34084	50.2284	1.13204	0.41858	2.70444	0.00684	0.11768	44.3524	28.6798	21.431	54.1514	87.1641	65.5914 Up
Rbm24	911.947	-1.8133	0.41795	-4.3386	1.43E-05	0.00111	1070.37	2202.41	986.802	331.039	245.644	635.417 Down
Cap2	822.764	-1.2098	0.32339	-3.741	0.00018	0.00885	788.487	1796.94	861.138	477.146	493.269	519.607 Down
Id4	703.603	-0.9598	0.34779	-2.7597	0.00579	0.10589	709.638	1273.78	804.638	475.102	289.226	669.237 Down
Wnk2	236.419	-2.4397	0.4585	-5.3211	1.03E-07	1.94E-05	314.409	630.955	252.302	78.6729	37.639	104.536 Down
Hist1h2al	72.3814	-8.6376	1.21268	-7.1228	1.06E-12	5.42E-10	148.827	131.531	152.94	0	0.9905	0 Down
Cks2	276.93	0.57364	0.19005	3.01834	0.00254	0.05985	252.316	229.438	186.06	348.408	333.799	311.559 Up
Nfil3	586.059	-0.8699	0.41781	-2.0821	0.03733	0.34405	293.711	1079.94	899.13	370.886	457.611	415.071 Down
Hrh2	72.9798	0.91908	0.46553	1.97428	0.04835	0.39416	76.8775	39.5583	35.069	105.238	125.794	55.3428 Up
Unc5a	260.396	1.68906	0.62188	2.71604	0.00661	0.11532	20.6978	222.516	126.638	416.864	447.706	327.957 Up
Hk3	307.172	0.88749	0.44241	2.00601	0.04486	0.38016	243.445	262.074	141.25	308.561	674.531	213.172 Up
Fgfr4	3010.97	-0.6409	0.18332	-3.4959	0.00047	0.01818	4389.9	2896.66	3720.24	2454.18	2360.36	2244.46 Down
Mxd3	66.9498	2.03805	0.44899	4.53915	5.65E-06	0.00056	33.5107	22.746	22.4052	160.411	98.0596	64.5665 Up
F12	7.59368	-1.8095	0.9088	-1.9911	0.04647	0.38596	7.88487	19.7792	7.79311	2.04345	3.962	4.09946 Down
Tifab	490.829	0.88907	0.29224	3.04232	0.00235	0.05658	360.733	377.782	294.19	582.383	897.394	432.493 Up
Tgfbi	3542.66	0.54058	0.19542	2.76629	0.00567	0.1051	3116.49	3028.19	2515.23	4636.59	4715.77	3243.7 Up
Gkap1	1645.73	0.20632	0.10363	1.9909	0.04649	0.38601	1593.73	1527.94	1463.16	1713.43	1790.83	1785.32 Up
Zfp808	329.009	-0.4862	0.21137	-2.3004	0.02143	0.24972	440.567	402.506	308.802	292.213	292.198	237.769 Down
Fbp2	84.8634	-1.087	0.48099	-2.2599	0.02383	0.2651	113.345	127.576	105.207	77.6511	19.81	65.5914 Down
Prxl2c	3930.35	0.49192	0.10013	4.91297	8.97E-07	0.00012	3335.3	3076.65	3388.05	4727.52	4493.9	4560.65 Up
Zfp369	1514.18	-0.2275	0.10756	-2.1153	0.03441	0.32776	1658.78	1640.68	1600.51	1482.52	1357.98	1344.62 Down
4933433G	276.602	0.49487	0.18315	2.70193	0.00689	0.11825	228.661	225.482	234.767	349.43	348.656	272.614 Up
Rsl1	536.567	0.7807	0.15238	5.12342	3.00E-07	4.66E-05	375.517	421.296	387.707	740.751	687.408	606.721 Up
Gm49064	118.511	-1.0545	0.25666	-4.1084	3.98E-05	0.00262	177.41	165.156	137.354	72.5425	66.3636	92.2379 Down
Zfp457	124.295	-0.6706	0.28238	-2.3749	0.01755	0.21948	138.971	170.101	149.043	100.129	118.86	68.666 Down
Gm28044	42.3673	0.71326	0.34997	2.03807	0.04154	0.36798	32.5251	30.6577	33.1207	62.3252	51.506	44.0692 Up
Zfp429	376.853	0.39403	0.1457	2.70432	0.00684	0.11768	337.078	325.367	314.647	459.776	406.105	418.145 Up
Zfp874a	989.734	0.5264	0.11766	4.47379	7.68E-06	0.0007	801.3	815.89	816.328	1191.33	1228.22	1085.33 Up
Gm26965	142.36	0.63692	0.27811	2.29015	0.02201	0.25312	141.928	98.8958	93.5173	197.193	183.243	139.382 Up
Zfp738	370.903	0.58344	0.15762	3.7015	0.00021	0.00987	278.927	327.345	284.448	433.212	424.925	476.563 Up
Gm10037	79.8653	0.67238	0.24691	2.72317	0.00647	0.11397	60.1221	61.3154	63.319	96.0422	101.031	97.3623 Up
BC048507	3.11656	-5.0649	1.78636	-2.8353	0.00458	0.09104	4.92804	8.90062	4.87069	0	0	0 Down
Ube2ql1	43.4636	-1.4443	0.42997	-3.3591	0.00078	0.02626	49.2804	72.1939	69.1638	29.63	13.867	26.6465 Down
Slc6a19	25.704	2.47115	0.81973	3.0146	0.00257	0.06015	0	11.8675	11.6897	42.9125	40.6105	47.1438 Up
Trip13	87.295	0.73703	0.32075	2.29787	0.02157	0.25004	51.2517	66.2602	78.9052	121.585	124.803	80.9644 Up
Gm47469	109.716	-1.3543	0.39831	-3.4002	0.00067	0.02361	150.798	128.565	193.854	88.8901	63.3921	32.7957 Down
Pcsk1	140.622	-1.3685	0.30525	-4.4832	7.35E-06	0.00069	223.733	165.156	219.181	81.738	53.487	100.437 Down
Gm4149	148.899	0.61481	0.29862	2.0588	0.03951	0.35838	121.23	115.708	115.922	196.171	117.87	226.495 Up
Rhobtb3	541.486	-0.5319	0.1576	-3.375	0.00074	0.02522	629.804	716.995	573.768	476.124	426.906	425.319 Down
Ckmt2	1868.72	-4.1961	1.25965	-3.3312	0.00086	0.02847	1964.32	7026.55	1641.42	41.8907	500.203	37.92 Down
Cmya5	1045.22	-2.9082	0.48493	-5.9971	2.01E-09	6.47E-07	1192.59	3372.35	969.268	152.237	363.514	221.371 Down
Arsb	2231.68	0.3283	0.12511	2.62421	0.00869	0.13771	2120.04	1947.26	1869.37	2323.4	2702.09	2427.91 Up
Zbed3	819.801	0.25937	0.11935	2.17319	0.02977	0.30052	777.645	723.917	737.423	904.227	930.08	845.514 Up
F2rl1	932.24	0.50532	0.1807	2.79648	0.00517	0.09821	807.214	680.403	824.121	1146.38	900.365	1234.96 Up
Sv2c	52.2146	-1.1541	0.32895	-3.5084	0.00045	0.01757	82.7911	59.3375	74.0345	30.6518	33.677	32.7957 Down
Naip2	773.016	0.73577	0.28665	2.56681	0.01026	0.15371	673.171	548.872	518.242	897.075	1353.02	647.715 Up
Naip6	244.637	0.57822	0.27612	2.09405	0.03626	0.33835	212.891	192.847	183.138	255.431	402.143	221.371 Up
Cenph	68.4995	0.64659	0.28737	2.24998	0.02445	0.26955	46.3236	60.3264	53.5776	87.8684	90.1356	72.7655 Up
Ccnb1	198.728	1.87281	0.37256	5.02691	4.98E-07	7.48E-05	85.748	99.8848	70.138	418.907	350.637	167.053 Up
Rnf180	284.739	0.95567	0.33378	2.86315	0.00419	0.08529	245.417	179.99	155.862	394.386	502.184	230.595 Up
Gm10739	2.5533	4.81878	2.0771	2.31996	0.02034	0.24164	0	0	0	6.13035	0.9905	8.19893 Up
Elovl7	575.374	0.36608	0.14774	2.47793	0.01321	0.18073	524.344	532.059	452	604.861	694.341	644.641 Up
Depdc1b	55.3914	1.09137	0.41667	2.61926	0.00881	0.13941	27.597	40.5473	37.9914	110.346	57.449	58.4174 Up
Il31ra	3406.54	1.96538	0.99891	1.96753	0.04912	0.39723	1382.81	1885.94	898.156	13049.5	1574.9	1647.98 Up
Ccno	258.817	0.62853	0.24423	2.57357	0.01007	0.15222	186.28	253.173	170.474	305.496	265.454	372.026 Up
Mcidas	55.8556	1.10819	0.30791	3.59908	0.00032	0.01347	40.41	34.6135	31.1724	78.6729	70.3256	79.9395 Up
Gzmk	75.0732	1.57325	0.72514	2.16958	0.03004	0.30199	79.8343	23.735	9.74139	176.758	104.003	56.3676 Up
Hspb3	11.5139	-5.0634	1.59266	-3.1792	0.00148	0.04099	3.94243	54.3927	8.76725	0	1.981	0 Down
Fst	340.543	-0.983	0.31393	-3.1311	0.00174	0.04603	445.495	495.468	415.957	240.105	312.998	133.233 Down
Gm6416	13.6234	-7.1929	1.45139	-4.9559	7.20E-07	0.0001	13.7985	48.4589	19.4828	0	0	0 Down
Gm21188	204.08	0.90968	0.46382	1.96129	0.04985	0.39986	116.302	217.571	91.569	198.215	435.82	165.003 Up
BC147527	155.011	0.73907	0.34141	2.16475	0.03041	0.30453	141.928	135.487	71.1121	197.193	235.739	148.606 Up
Gm10340	6.22437	-5.0612	1.75917	-2.877	0.00401	0.08279	0.98561	17.8012	17.5345	0	0	1.02487 Down

Gm8108	12.1595	-5.1275	1.26698	-4.047	5.19E-05	0.00326	24.6402	32.6356	13.6379	2.04345	0	0	Down
Gm2974	12.6526	-3.5202	0.91249	-3.8578	0.00011	0.006	13.7985	32.6356	23.3793	4.0869	0.9905	1.02487	Down
Gm3173	9.5026	-3.7069	1.16029	-3.1948	0.0014	0.0396	6.89926	17.8012	28.25	0	0.9905	3.0746	Down
Gm3239	3.44159	-5.2081	2.42223	-2.1501	0.03154	0.3109	0	12.8565	7.79311	0	0	0	Down
Gm3252	16.0432	-5.5424	1.18453	-4.679	2.88E-06	0.00032	20.6978	32.6356	40.9138	1.02173	0.9905	0	Down
Gm26650	2.47065	-3.6643	1.82161	-2.0116	0.04427	0.37773	4.92804	6.92271	1.94828	0	0	1.02487	Down
Gm3194	4.91812	-4.7121	1.60417	-2.9374	0.00331	0.07278	3.94243	14.8344	9.74139	0	0.9905	0	Down
Gm3453	3.28194	-5.1394	1.80927	-2.8406	0.0045	0.09015	3.94243	10.8785	4.87069	0	0	0	Down
Gm3411	16.1376	-2.9368	0.78683	-3.7324	0.00019	0.00908	18.7266	42.5252	24.3535	6.13035	0.9905	4.09946	Down
Gm26630	3.93575	-5.4016	1.70531	-3.1675	0.00154	0.04219	4.92804	11.8675	6.81897	0	0	0	Down
Gm3667	13.3484	-2.2113	0.73891	-2.9927	0.00277	0.06326	15.7697	25.7129	24.3535	8.1738	1.981	4.09946	Down
Gm10406	17.9366	-3.2708	0.97077	-3.3693	0.00075	0.02551	15.7697	60.3264	21.431	6.13035	3.962	0	Down
Gm3696	9.05137	-2.3141	0.86813	-2.6656	0.00768	0.12697	7.88487	21.7571	15.5862	2.04345	3.962	3.0746	Down
Gm3739	44.8163	-2.0647	0.59562	-3.4665	0.00053	0.0198	32.5251	87.0283	97.4139	20.4345	7.92401	23.5719	Down
Pxk	4758.13	0.36336	0.12881	2.82088	0.00479	0.09326	4457.91	4004.29	4024.17	5318.08	5889.52	4854.79	Up
Oit1	193.845	-2.5564	0.65578	-3.8983	9.69E-05	0.00526	96.5897	735.785	161.707	67.4339	56.4585	45.0941	Down
4930452B	424.865	-1.0203	0.23866	-4.2752	1.91E-05	0.0014	473.092	716.006	518.242	299.366	226.825	315.659	Down
4930455B	7.53412	-6.3386	1.43845	-4.4065	1.05E-05	0.00086	14.7841	14.8344	15.5862	0	0	0	Down
Ptprg	7307.08	0.39022	0.18545	2.1042	0.03536	0.33334	5842.69	6732.83	6399.12	9336.53	9143.31	6387.99	Up
Gm49355	247.103	-0.4911	0.19049	-2.5784	0.00993	0.15089	279.913	254.162	332.181	217.628	205.034	193.7	Down
Gm5087	13.8726	4.33101	1.31023	3.30554	0.00095	0.03031	1.97122	1.97792	0	31.6735	45.563	2.04973	Up
Sntn	2760.85	-0.8106	0.40896	-1.9821	0.04747	0.39055	3073.13	4111.1	3365.65	1841.15	862.726	3311.34	Down
Lrrc3b	80.7526	-3.3012	0.63791	-5.1749	2.28E-07	3.88E-05	92.6472	288.776	58.4483	12.2607	11.886	20.4973	Down
Gm21738	24.0413	2.83732	1.04743	2.70884	0.00675	0.11674	6.89926	5.93375	4.87069	108.303	5.94301	12.2984	Up
Nid2	521.249	-0.6548	0.30836	-2.1235	0.03371	0.32401	557.855	772.376	582.535	421.973	545.766	246.993	Down
Anxa7	11183.3	0.22111	0.08649	2.55642	0.01058	0.15675	10545	9978.59	10460.3	12242.3	11802.8	12070.9	Up
Synpo2l	288.508	-3.4528	1.01366	-3.4063	0.00066	0.02324	310.467	1016.65	259.121	15.3259	107.965	21.5222	Down
Plau	524.866	0.53795	0.1778	3.02563	0.00248	0.05888	367.632	465.799	451.026	609.97	699.294	555.477	Up
Zfp503	3278.31	0.28705	0.14136	2.03065	0.04229	0.37149	3079.04	2801.72	2978.92	4123.68	3448.92	3237.55	Up
Kcnma1	122.312	-0.7256	0.36842	-1.9696	0.04888	0.39608	106.446	206.692	144.173	65.3904	87.1641	124.009	Down
Gm34059	357.523	-1.4459	0.40839	-3.5406	0.0004	0.01603	361.718	741.719	465.638	219.671	96.0786	260.316	Down
Asb14	994.77	-0.7166	0.3038	-2.3588	0.01834	0.22562	988.566	1574.42	1147.54	672.295	521.994	1063.81	Down
Cacna2d3	156.32	-0.9074	0.46279	-1.9608	0.0499	0.39986	148.827	343.168	119.819	127.716	130.746	67.6411	Down
Chdh	196.249	-0.5709	0.2646	-2.1574	0.03098	0.30783	176.424	284.82	242.561	145.085	188.195	140.407	Down
Cacna1d	1505.79	-0.3985	0.18809	-2.1188	0.03411	0.32547	1410.41	1754.41	1972.63	1258.77	1491.69	1146.82	Down
Prkcd	6941.85	0.33108	0.15591	2.12353	0.03371	0.32401	6206.38	5723.1	6516.99	7542.38	9002.66	6659.58	Up
Itih4	5038.02	1.08189	0.34273	3.15666	0.0016	0.04292	3330.37	4042.86	2325.27	6644.28	9941.66	3943.68	Up
Itih1	136.345	0.74109	0.30971	2.39285	0.01672	0.21274	106.446	71.205	128.586	147.128	210.977	153.73	Up
Colq	1564.09	-0.628	0.2181	-2.8796	0.00398	0.08243	1684.41	2138.13	1875.22	1579.59	1120.26	986.946	Down
Ogdhl	92.894	-1.764	0.60553	-2.9132	0.00358	0.0759	52.2373	284.82	93.5173	37.8038	34.6675	54.3179	Down
Tmem273	253.486	0.80583	0.30654	2.62884	0.00857	0.13624	195.151	186.913	171.448	314.691	446.716	205.998	Up
Ptpn20	124.292	-0.9226	0.44432	-2.0763	0.03786	0.34778	147.841	215.593	124.69	92.977	39.62	125.034	Down
Sncg	574.922	-1.2424	0.31071	-3.9986	6.37E-05	0.00379	796.372	1073.02	555.259	298.344	268.426	458.115	Down
Ldb3	1925.98	-3.4514	0.80315	-4.2973	1.73E-05	0.0013	2106.25	6628.99	1852.81	161.433	595.291	211.122	Down
Cdhr1	199.667	-3.6048	1.06492	-3.385	0.00071	0.02459	211.906	774.354	120.793	15.3259	65.3731	10.2487	Down
2610528A	11.0257	1.56463	0.69941	2.23705	0.02528	0.27483	6.89926	5.93375	3.89655	12.2607	21.791	15.373	Up
Tspan14	4704.08	0.27675	0.10247	2.70075	0.00692	0.11842	4125.76	4119.01	4518.05	5135.19	5348.7	4977.77	Up
Sftpa1	183393	0.53062	0.16702	3.17707	0.00149	0.04114	181746	129541	138839	206646	229324	214261	Up
Mbl1	176.931	0.56951	0.22646	2.51488	0.01191	0.16878	114.331	148.344	164.629	222.736	190.176	221.371	Up
Sftpd	47624.7	0.78154	0.20245	3.8604	0.00011	0.00595	43441.7	29033.8	32619	55696.3	70526.6	54430.6	Up
Gm8113	152.639	0.71232	0.27484	2.59174	0.00955	0.14667	138.971	106.807	101.31	241.127	157.49	170.128	Up
4930503E	42.4468	1.88965	0.70149	2.69377	0.00706	0.12013	36.4675	7.91166	9.74139	66.4121	32.6865	101.462	Up
Gm5930	336.079	2.06861	0.57323	3.6087	0.00031	0.01308	252.316	95.9289	39.9397	447.516	380.352	800.42	Up
Gm8229	382.746	2.25989	0.59142	3.8211	0.00013	0.00675	259.215	71.205	66.2414	426.059	354.599	1119.15	Up
AC160336	1436.42	0.9905	0.35779	2.7684	0.00563	0.10469	1460.67	623.044	801.716	1545.87	1518.44	2668.75	Up
Gm15218	8.08512	-1.7445	0.86155	-2.0248	0.04289	0.37291	5.91365	17.8012	13.6379	4.0869	2.9715	4.09946	Down
Cdkn3	74.9974	1.09464	0.3505	3.12309	0.00179	0.0469	37.4531	57.3596	48.7069	127.716	105.984	72.7655	Up
Gm34934	241.091	-0.3907	0.18601	-2.1006	0.03568	0.33513	244.431	287.787	288.345	232.953	192.157	200.874	Down
Lgals3	8697.52	0.38708	0.19155	2.02076	0.0433	0.3735	9557.45	6519.21	6536.47	9992.47	10383.4	9196.12	Up
Dlgap5	105.529	0.83661	0.3946	2.12015	0.03399	0.32506	85.748	87.0283	54.5518	167.563	162.442	75.8401	Up
Tlr11	74.9553	1.71089	0.52507	3.25843	0.00112	0.03374	42.3812	36.5914	26.3017	98.0856	195.129	51.2433	Up
Rnase6	686.292	0.65445	0.30315	2.15882	0.03086	0.30729	693.869	455.91	450.052	700.904	1183.65	633.367	Up
Gm7247	11.8482	1.70457	0.75267	2.2647	0.02353	0.26285	7.88487	2.96687	5.84483	18.3911	25.753	10.2487	Up
Ndrg2	6020.56	-0.726	0.23729	-3.0595	0.00222	0.05418	6894.33	9553.34	6064.99	4828.67	5289.27	3492.74	Down
Rab2b	143.48	-0.4265	0.21525	-1.9814	0.04755	0.39072	186.28	164.167	143.198	117.498	127.775	121.959	Down
Rps19-ps2	17.3038	-1.2183	0.6082	-2.0031	0.04517	0.38075	23.6546	19.7792	29.2242	7.15208	16.8385	7.17406	Down
Trac	634.213	0.8253	0.28258	2.92054	0.00349	0.07481	512.517	401.517	458.819	930.792	998.425	503.209	Up
Gm43305	415.855	6.38234	0.5572	11.4543	2.24E-30	4.03E-27	7.88487	18.7902	2.92242	629.383	1341.14	495.01	Up
Haus4	532.192	0.28638	0.14552	1.96798	0.04907	0.39703	503.646	443.053	491.94	623.252	596.282	534.98	Up
Ajuba	2411.67	-0.28	0.12605	-2.2212	0.02634	0.28071	2607.92	2480.31	2846.43	2218.17	1995.86	2321.32	Down
Gm49130	1.80648	-4.2779	2.13678	-2.002	0.04528	NA	2.95683	5.93375	1.94828	0	0	0	Down
Myh6	16812.7	-3.9003	1.87937	-2.0753	0.03796	0.34837	21352.2	55484.5	17707.9	21.4562	6254.02	56.3676	Down
Mhrt	46.6484	-5.0886	1.03465	-4.9182	8.74E-07	0.00012	50.266	168.123	53.5776	0	7.92401	0	Down
Myh7	326.486	-1.4501	0.39597	-3.6621	0.00025	0.01116	318.352	800.067	315.621	154.281	159.471	211.122	Down
Pck2	2556.06	0.21892	0.09934	2.20372	0.02754	0.28707	2422.63	2325.04	2339.88	2610.51	2820.95	2817.36	Up
Fitm1	59.2238	-3.0127	0.6766	-4.4528	8.48E-06	0.00075	95.604	162.189	58.4483	3.06518	24.7625	11.2735	Down
Psme1	7313.35	0.26503	0.09864	2.68694	0.00721	0.12156	6730.72	6372.85	6826.76	7876.48	7670.44	8402.88	Up
Psme2	4102.62	0.23489	0.08693	2.70223	0.00689	0.11825	3767.98	3720.46	3819.6	4396.48	4373.06	4538.11	Up
Irf9	3851.3	0.42751	0.1854	2.30591	0.02112	0.24769	3284.05	3380.26	3190.3	4316.79	5418.04	3518.36	Up
Cbln3	7.25663	-1.7223	0.8402	-2.0499	0.04038	0.36179	10.8417	10.8785	11.6897	5.10863	2.9715	2.04973	Down
Gm5801	45.1418	2.64325	0.72127	3.66471	0.00025	0.01109	13.7985	13.8454	9.74139	103.194	114.898	15.373	Up
Mcpt4	39.3132	-1.991	0.90483	-2.2004	0.02778	0.28801	14.7841	150.322	23.3793	6.13035	21.791	19.4725	Down
Gja3	52.2997	-5.9464	1.10484	-5.3822	7.36E-08	1.47E-05	57.1653	218.56	33.1207	0	4.9525	0	Down

Ska3	98.5413	1.104	0.24599	4.48803	7.19E-06	0.00068	55.1941	70.216	62.3449	133.846	144.613	125.034	Up
3110083C	90.5505	-0.6797	0.31133	-2.1831	0.02903	0.29524	113.345	128.565	92.5432	57.2166	60.4206	91.2131	Down
Phf11a	113.146	0.89035	0.38118	2.33575	0.0195	0.23543	78.8487	87.0283	72.0863	136.911	217.91	86.0887	Up
Phf11b	797.929	0.54621	0.27288	2.00165	0.04532	0.38132	570.667	775.343	600.069	932.835	1259.92	648.74	Up
Shisa2	418.984	-0.7161	0.32164	-2.2264	0.02599	0.27879	660.358	464.81	437.388	308.561	201.072	441.717	Down
Atp8a2	126.566	-0.6915	0.33079	-2.0906	0.03657	0.34009	116.302	199.77	152.94	68.4556	102.022	119.909	Down
Tnfrsf19	2400.59	-0.2972	0.14536	-2.0449	0.04087	0.36391	2304.35	2888.75	2748.04	1988.28	2316.78	2157.34	Down
Sgcg	35.637	-2.3837	0.67135	-3.5506	0.00038	0.01558	36.4675	115.708	27.2759	9.19553	12.8765	12.2984	Down
Dleu2	1720.01	-0.5212	0.13389	-3.8931	9.90E-05	0.00534	1849.99	2176.7	2055.43	1542.81	1366.89	1328.23	Down
Gata4	202.997	-2.8525	0.68769	-4.1479	3.36E-05	0.00231	264.143	662.602	143.198	22.478	103.012	22.547	Down
Gm49390	7.3979	-6.3119	1.65661	-3.8101	0.00014	0.00698	7.88487	30.6577	5.84483	0	0	0	Down
Gm5463	5.58975	-5.9075	1.71262	-3.4494	0.00056	0.02074	7.88487	21.7571	3.89655	0	0	0	Down
Nuggc	14.6741	2.31289	0.80479	2.87391	0.00405	0.08338	5.91365	5.93375	2.92242	20.4345	42.5915	10.2487	Up
Scara5	798.307	-1.2219	0.30763	-3.9719	7.13E-05	0.00411	999.407	1605.08	748.138	490.428	566.566	380.225	Down
Pbk	139.557	1.99281	0.38554	5.16889	2.35E-07	3.91E-05	53.2229	63.2933	51.6293	332.061	208.005	129.133	Up
Esco2	97.1735	1.25389	0.47924	2.61643	0.00889	0.14016	55.1941	78.1277	38.9655	181.867	167.395	61.492	Up
Scara3	473.462	-1.7293	0.27414	-6.308	2.83E-10	1.05E-07	510.545	996.87	675.078	240.105	206.024	212.147	Down
Ephx2	502.13	-2.1022	0.4287	-4.9037	9.41E-07	0.00013	556.869	1426.08	460.768	164.498	243.663	160.904	Down
Ptk2b	1888.29	0.6547	0.26507	2.46994	0.01351	0.18348	1827.32	1380.59	1193.32	2260.06	3007.16	1661.31	Up
Adra1a	1602.09	-0.8458	0.20589	-4.1082	3.99E-05	0.00262	1662.72	2300.32	2213.24	1041.14	1388.68	1006.42	Down
Cdca2	127.062	1.25452	0.31893	3.93352	8.37E-05	0.00467	78.8487	79.1166	67.2156	236.019	181.262	119.909	Up
Dock5	1849.89	0.40336	0.20547	1.96315	0.04963	0.39975	1785.92	1529.92	1463.16	2191.6	2522.81	1605.96	Up
Entpd4b	63.9472	1.63924	0.76641	2.13884	0.03245	0.31621	15.7697	34.6135	42.8621	14.3042	164.423	111.71	Up
Phyhip	47.9315	-1.9748	0.5883	-3.3567	0.00079	0.02637	81.8055	93.951	53.5776	6.13035	33.677	18.4476	Down
Hr	595.973	0.69294	0.34852	1.98826	0.04678	0.38737	281.884	550.85	533.828	1041.14	508.127	660.014	Up
Gm41206	2.38144	4.71832	2.10942	2.23678	0.0253	0.27483	0	0	0	8.1738	0.9905	5.12433	Up
Cysltr2	221.222	0.73303	0.2567	2.85552	0.0043	0.08671	154.741	200.758	143.198	255.431	345.685	227.52	Up
Sucla2	5151.97	-0.3404	0.16337	-2.0834	0.03721	0.34382	5200.07	6891.06	5179.49	4659.07	4218.54	4763.58	Down
Lcp1	11686.9	0.7114	0.26859	2.64861	0.00808	0.13094	11013.2	8182.64	7391.76	13046.4	19827.8	10659.6	Up
Epsti1	1101.3	0.67578	0.24468	2.76195	0.00575	0.10587	837.767	1014.67	691.638	1259.79	1745.26	1058.69	Up
Tnfsf11	35.6594	1.46139	0.67664	2.15978	0.03079	0.30698	9.85609	26.7019	20.4569	61.3035	79.2401	16.3979	Up
Kbtbd6	17.2439	1.60504	0.77866	2.06129	0.03928	0.35682	8.87048	11.8675	4.87069	42.9125	26.7435	8.19893	Up
Rps3a2	4915.03	-4.4495	0.22891	-19.438	3.69E-84	2.66E-80	9293.3	8127.26	10778.8	352.495	368.466	569.825	Down
Acod1	66.7588	2.67888	0.83949	3.19107	0.00142	0.03994	23.6546	17.8012	12.6638	73.5642	258.521	14.3481	Up
Gm34907	293.618	0.68428	0.30754	2.22503	0.02608	0.27935	309.481	199.77	166.578	429.125	389.267	267.49	Up
Scel	3687.93	0.66973	0.32547	2.05771	0.03962	0.35889	4164.2	1683.21	2693.49	5666.49	4045.21	3875.02	Up
Tpm3-rs7	8503.87	-3.0526	0.17674	-17.271	7.72E-67	4.17E-63	14782.2	14539.7	16213.6	1661.33	2254.38	1572.14	Down
Dct	6.2396	-3.0347	1.2332	-2.4609	0.01386	0.18672	3.94243	11.8675	17.5345	2.04345	0	2.04973	Down
Sox21	107.972	-4.3531	0.63942	-6.8078	9.91E-12	4.55E-09	98.5609	382.727	136.379	10.2173	15.848	4.09946	Down
Slc15a1	164.795	0.53342	0.26867	1.9854	0.0471	0.38896	175.438	104.83	123.716	212.519	170.366	201.899	Up
Fgf14	119.845	-1.3165	0.34138	-3.8564	0.00012	0.006	125.172	202.736	185.086	60.2818	51.506	94.2877	Down
5430437J:	154.773	1.19418	0.34735	3.43801	0.00059	0.02146	123.201	97.9068	61.3707	233.975	260.502	151.68	Up
Il7r	1627.48	0.81151	0.24483	3.31463	0.00092	0.02962	1179.77	1066.1	1298.53	2261.08	2560.44	1398.94	Up
Adamts12	147.793	-0.7519	0.37345	-2.0135	0.04406	0.377	101.518	254.162	200.673	130.781	95.0881	104.536	Down
Cdh12	6.70283	1.82639	0.8739	2.08992	0.03663	0.3402	2.95683	2.96687	2.92242	7.15208	10.8955	13.3233	Up
Gm6533	12.2966	-2.651	0.99712	-2.6586	0.00785	0.12826	19.7122	5.93375	37.9914	1.02173	2.9715	6.1492	Down
Basp1	977.233	-1.4975	0.42454	-3.5275	0.00042	0.01654	722.451	2425.91	1181.63	388.256	698.303	446.842	Down
Otulinl	2125.61	0.47946	0.23889	2.00706	0.04474	0.37966	1794.79	1881	1651.16	2576.79	3153.75	1696.15	Up
Osr2	22.3231	-1.1035	0.54314	-2.0318	0.04218	0.37132	17.741	39.5583	34.0948	17.3693	12.8765	12.2984	Down
Pabpc1	22182.5	0.30364	0.13927	2.18027	0.02924	0.29698	22375.3	19478.5	17716.7	23868.5	26484	23172.2	Up
1100001I1	22.2799	-1.4669	0.54053	-2.7137	0.00665	0.11573	30.5539	23.735	43.8362	13.2824	7.92401	14.3481	Down
Lrp12	859.903	0.79924	0.38892	2.05506	0.03987	0.35961	881.134	546.894	454.923	894.01	1723.47	658.989	Up
AU02279:	147.356	1.74995	0.57212	3.0587	0.00222	0.05418	96.5897	57.3596	48.7069	214.562	396.2	70.7157	Up
Gm33251	3.69202	2.7045	1.35729	1.99258	0.04631	0.38491	1.97122	0	0.97414	8.1738	6.93351	4.09946	Up
Abra	149.957	-1.9655	0.51982	-3.7811	0.00016	0.00766	149.813	430.197	136.379	60.2818	86.1736	36.8952	Down
Angpt1	5339.52	-0.5007	0.1269	-3.9457	7.96E-05	0.00449	6107.82	6572.62	6090.31	4677.46	4681.11	3907.81	Down
Sybu	329.415	-0.4907	0.19493	-2.5172	0.01183	0.16816	369.603	447.998	337.052	272.801	244.654	304.385	Down
Rpl15-ps6	371.616	-3.8047	0.22189	-17.147	6.64E-66	2.87E-62	676.128	772.376	632.216	42.9125	44.5725	61.492	Down
Ccn3	805.202	-1.1474	0.35135	-3.2658	0.00109	0.03316	710.624	1520.03	1097.85	462.842	335.78	704.083	Down
Tbc1d31	554.316	0.31696	0.1452	2.18296	0.02904	0.29524	527.301	452.943	500.707	666.165	585.386	593.397	Up
Atad2	1193.92	0.51787	0.22187	2.3341	0.01959	0.236	1203.43	886.106	856.268	1561.2	1563.01	1093.53	Up
Myc	603.216	0.3664	0.14365	2.55063	0.01075	0.15803	547.998	493.49	539.673	654.926	748.819	634.392	Up
Pvt1	224.376	0.61516	0.26185	2.34926	0.01881	0.2295	204.021	161.2	166.578	314.691	306.065	193.7	Up
Fam49b	3827.77	0.32335	0.15717	2.0573	0.03966	0.35901	3452.59	3545.41	3203.94	4372.98	4842.56	3549.11	Up
Gm7859	17.6797	-6.5935	1.36994	-4.813	1.49E-06	0.00018	15.7697	42.5252	46.7587	0	0	1.02487	Down
Khdrbs3	1307.36	-0.3613	0.15527	-2.327	0.01996	0.23859	1301	1702	1407.63	1161.7	1071.72	1200.12	Down
Lypd2	1753.81	-1.3733	0.43881	-3.1297	0.00175	0.04609	1531.64	3993.41	2067.12	1002.31	485.345	1443.01	Down
Lynx1	1968.84	-0.7156	0.22244	-3.2171	0.00129	0.03729	2314.21	2947.1	2080.76	1271.03	1319.35	1880.63	Down
Gm17189	272.445	-0.7846	0.26709	-2.9377	0.00331	0.07278	272.028	425.252	337.052	150.194	229.796	220.346	Down
Ly6m	142.873	-1.3448	0.47973	-2.8032	0.00506	0.09677	157.697	289.765	167.552	58.2383	43.582	140.407	Down
Ly6e	54350.1	0.39475	0.08847	4.46206	8.12E-06	0.00073	47805	46591.8	46484.9	61388.3	64417.2	59413.5	Up
Ly6i	670.401	3.2229	0.54334	5.9316	3.00E-09	9.53E-07	121.23	132.52	135.405	816.359	2435.64	381.25	Up
Ly6a	39649.9	0.36531	0.14204	2.57182	0.01012	0.15256	34423.4	35551.1	33995.5	43090.2	51634.8	39204.2	Up
Ly6c2	1288.09	1.21599	0.39426	3.0842	0.00204	0.05154	880.149	850.504	595.199	1559.15	2945.75	897.782	Up
Gm46519	3.83062	4.38187	2.13001	2.05721	0.03967	0.35901	0	0	0.97414	7.15208	14.8575	0	Up
Mb	5407.08	-5.3189	1.88341	-2.8241	0.00474	0.09258	5136.01	22329.7	4183.93	3.06518	779.524	10.2487	Down
Apol6	212.141	-1.5337	0.55877	-2.7448	0.00606	0.10879	183.323	588.43	174.371	135.889	141.642	49.1936	Down
Apol9a	113.282	1.16287	0.46052	2.5251	0.01157	0.16553	83.7767	95.9289	30.1983	133.846	212.958	122.984	Up
Apol7c	160.35	1.64799	0.33821	4.87271	1.10E-06	0.00014	73.9207	67.2491	91.569	269.735	311.017	148.606	Up
Apol9b	284.769	0.92467	0.32533	2.84226	0.00448	0.08982	218.805	238.339	132.483	287.105	511.098	320.783	Up
Pvalb	5.60444	4.00424	1.61767	2.47532	0.01331	0.18148	0	1.97792	0	17.3693	1.981	12.2984	Up
Ncf4	736.095	0.68831	0.31451	2.18848	0.02864	0.29271	616.005	579.529	495.837	817.38	1343.12	564.701	Up
Csf2rb2	604.649	1.10188	0.41595	2.64907	0.00807	0.13085	538.142	344.157	270.811	885.836	1199.5	389.449	Up

Csf2rb	1984.64	1.10888	0.3505	3.16371	0.00156	0.04258	1635.12	1224.33	912.768	2615.62	3956.06	1563.95	Up
Il2rb	844.694	0.86052	0.29535	2.91355	0.00357	0.07588	552.926	665.569	581.561	1049.31	1537.26	681.536	Up
Gm49490	28.8536	-1.9611	0.68274	-2.8724	0.00407	0.08354	30.5539	68.2381	38.9655	19.4128	12.8765	3.0746	Down
Sstr3	35.4403	-2.764	0.69733	-3.9637	7.38E-05	0.00422	35.4819	121.642	28.25	8.1738	10.8955	8.19893	Down
Rac2	3117.95	0.64569	0.2487	2.59622	0.00943	0.14539	2931.2	2319.11	2044.72	3682.3	4937.65	2792.76	Up
Cyth4	1815.54	0.65056	0.27873	2.33401	0.01959	0.236	1643.01	1300.48	1295.6	2059.8	3105.22	1489.13	Up
Grap2	789.64	0.68807	0.23246	2.95995	0.00308	0.06914	647.545	585.463	581.561	1039.09	1197.52	686.66	Up
Mrtfa	1983.59	0.34935	0.14922	2.34115	0.01922	0.23297	1807.61	1572.44	1853.79	2252.9	2461.39	1953.39	Up
Csdc2	326.403	-2.3931	0.53301	-4.4897	7.13E-06	0.00068	397.2	926.654	321.466	54.1514	187.205	71.7406	Down
Cenpm	47.9336	1.10066	0.41207	2.67104	0.00756	0.12551	21.6834	42.5252	27.2759	75.6077	71.3161	49.1936	Up
Sult4a1	46.3097	-1.4068	0.4716	-2.983	0.00285	0.06475	85.748	69.2271	46.7587	17.3693	19.81	38.9449	Down
Pnpla3	61.5865	-3.3408	0.74572	-4.4799	7.47E-06	0.00069	57.1653	235.372	43.8362	7.15208	18.8195	7.17406	Down
Gm49513	71.4137	-1.6691	0.6526	-2.5576	0.01054	0.15671	57.1653	195.814	73.0604	32.6952	57.449	12.2984	Down
Parvg	735.394	0.79354	0.30323	2.61696	0.00887	0.14005	656.415	479.645	478.302	943.053	1271.8	583.149	Up
Shisal1	162.815	-0.4834	0.24261	-1.9927	0.04629	0.38491	200.079	217.571	151.966	135.889	153.528	117.86	Down
Fam118a	921.06	-0.3205	0.16262	-1.9712	0.0487	0.3951	925.487	985.002	1158.25	842.923	723.066	891.633	Down
Gm19277	9.38754	-5.6682	1.97923	-2.8639	0.00419	0.08518	13.7985	41.5362	0	0	0.9905	0	Down
Wnt7b	416.843	-0.6307	0.25076	-2.5153	0.01189	0.16878	530.257	623.044	366.276	358.626	273.378	349.479	Down
Lncppara	442.254	-0.4843	0.22076	-2.1936	0.02826	0.29024	377.488	580.518	589.354	355.56	372.428	378.176	Down
Ppara	197.193	-0.8058	0.34812	-2.3148	0.02062	0.24375	155.726	303.61	293.216	163.476	99.0501	168.078	Down
Gtse1	108.539	0.83465	0.30659	2.72239	0.00648	0.11407	65.0502	93.951	75.0087	156.324	160.461	100.437	Up
Mlc1	3471.94	0.54998	0.22909	2.40069	0.01636	0.21047	3800.51	2161.86	2491.85	4030.71	4335.42	4011.33	Up
Mov10l1	46.2177	-3.9257	0.70082	-5.6016	2.12E-08	4.99E-06	69.9782	150.322	39.9397	4.0869	9.90501	3.0746	Down
Mapk8ip2	7.56146	-2.9957	1.01876	-2.9406	0.00328	0.07219	13.7985	14.8344	11.6897	3.06518	1.981	0	Down
Lrrk2	7919.04	0.41416	0.20162	2.05417	0.03996	0.36	7992.3	6353.07	6025.05	8887.99	10933.1	7322.67	Up
Gm26760	80.6355	-1.3424	0.52226	-2.5705	0.01016	0.15295	89.6904	202.736	54.5518	59.2601	38.6295	38.9449	Down
Pdzrn4	32.2225	-1.3911	0.69143	-2.012	0.04422	0.37762	31.5395	88.0173	20.4569	11.239	27.734	14.3481	Down
D030018L	1230.76	-0.8634	0.38521	-2.2414	0.025	0.27341	1111.77	1846.38	1807.03	637.557	539.823	1441.99	Down
Pfkm	2170.43	-1.2932	0.31274	-4.1352	3.55E-05	0.00241	2534	4747.99	1966.79	1213.81	1273.78	1286.21	Down
Rnd1	691.123	0.38066	0.14369	2.64914	0.00807	0.13085	606.149	577.552	617.604	755.055	715.142	875.235	Up
Prph	98.9619	-1.9039	0.51895	-3.6688	0.00024	0.01097	204.021	162.189	102.285	30.6518	19.81	74.8152	Down
Troap	64.1299	1.52085	0.59547	2.55404	0.01065	0.15717	13.7985	60.3264	25.3276	136.911	94.0976	54.3179	Up
Faim2	1298.54	-0.3656	0.14371	-2.5442	0.01095	0.16037	1537.55	1423.11	1426.14	1154.55	1260.91	988.996	Down
Aqp2	34.6583	-1.9592	0.49084	-3.9915	6.57E-05	0.00388	64.0646	67.2491	34.0948	18.3911	12.8765	11.2735	Down
Racgap1	512.345	0.86083	0.24805	3.47036	0.00052	0.01958	328.208	438.108	325.362	753.012	751.79	477.587	Up
Tmprss12	23.6695	-4.24	1.04528	-4.0563	4.99E-05	0.00317	32.5251	67.2491	35.069	0	0	7.17406	Down
Slc11a2	4392.01	-0.6889	0.16456	-4.1865	2.83E-05	0.00201	5126.15	5975.28	5161.96	3025.33	3073.52	3989.8	Down
Pou6f1	1985.57	-0.2383	0.11314	-2.1067	0.03514	0.3317	2114.13	2058.02	2275.59	1775.76	1937.42	1752.52	Down
Gm49492	266.947	-0.447	0.1871	-2.3891	0.01689	0.21427	341.021	287.787	295.164	195.15	240.692	241.868	Down
Bin2	1282.2	0.68311	0.29877	2.28638	0.02223	0.2547	1123.59	1016.65	812.432	1413.05	2282.11	1045.36	Up
Nr4a1	7882.54	-1.4236	0.39379	-3.615	0.0003	0.01287	6894.33	17884.3	9673.2	3930.58	2700.11	6212.74	Down
Krt88	9.7471	-1.3834	0.70348	-1.9665	0.04924	0.39755	17.741	11.8675	12.6638	5.10863	4.9525	6.1492	Down
Krt5	23.7291	-6.1316	1.79951	-3.4074	0.00066	0.02324	6.89926	132.52	0.97414	0	1.981	0	Down
Espl1	226.285	0.93695	0.23335	4.0153	5.94E-05	0.0036	143.899	164.167	157.81	347.387	317.951	226.495	Up
Nckap1l	1987.47	0.80902	0.32545	2.48583	0.01292	0.17801	1837.17	1319.27	1176.76	2155.84	3784.7	1651.06	Up
Ppp1r1a	223.938	-1.5008	0.36787	-4.0798	4.51E-05	0.00291	273.999	512.28	206.517	119.542	98.0596	133.233	Down
Srl	1666.53	-3.9895	1.02116	-3.9068	9.35E-05	0.00511	1712	6129.56	1565.44	68.4556	442.754	80.9644	Down
Cdip1	3301.82	-0.2159	0.10282	-2.1	0.03573	0.33519	3527.49	3556.29	3561.45	2976.29	2918.02	3271.37	Down
Sec14l5	718.36	-0.9093	0.27588	-3.2961	0.00098	0.031	809.185	1072.03	931.276	500.645	329.837	667.188	Down
Rbfox1	107.037	-1.7315	0.45292	-3.8229	0.00013	0.00672	119.259	278.886	95.4656	50.0645	54.4775	44.0692	Down
Abat	450.665	-0.9928	0.37233	-2.6665	0.00766	0.12672	405.085	951.378	443.233	216.606	324.884	362.803	Down
Rpl39l	3.51023	3.28783	1.60968	2.04254	0.0411	0.36508	0	0	1.94828	5.10863	9.90501	4.09946	Up
Ciita	65.6025	0.97722	0.45973	2.12564	0.03353	0.32299	41.3956	41.5362	49.6811	102.173	118.86	39.9698	Up
Rmi2	48.866	1.32635	0.40451	3.2789	0.00104	0.03224	37.4531	23.735	22.4052	89.9118	65.3731	54.3179	Up
Tnfrsf17	23.0368	2.20867	0.71647	3.08271	0.00205	0.05157	9.85609	8.90062	5.84483	41.8907	59.4301	12.2984	Up
Gm15738	14.0079	-1.9648	0.69411	-2.8307	0.00465	0.09178	23.6546	28.6798	14.6121	4.0869	8.91451	4.09946	Down
2310015D	13.9952	-3.6807	1.07528	-3.423	0.00062	0.02229	6.89926	57.3596	13.6379	2.04345	1.981	2.04973	Down
Myh11	4979.66	-0.6593	0.25918	-2.5437	0.01097	0.16049	4202.64	7839.47	6252.02	3409.5	3455.86	4718.48	Down
Gm33696	24.6396	-1.6508	0.72719	-2.2701	0.0232	0.26121	47.3092	31.6467	33.1207	13.2824	1.981	20.4973	Down
Sdf2l1	995.425	0.71004	0.19472	3.64639	0.00027	0.0117	693.869	770.398	801.716	1220.96	1497.64	987.971	Up
Prodh	2198	-0.2421	0.11252	-2.1517	0.03142	0.31043	2294.5	2417.01	2434.37	1960.69	1907.7	2173.74	Down
Rtn4r	42.8723	-0.9174	0.45551	-2.014	0.04401	0.37695	38.4387	78.1277	51.6293	38.8256	29.715	20.4973	Down
4933432I0	27.7785	-1.1182	0.50014	-2.2359	0.02536	0.27515	33.5107	49.4479	31.1724	10.2173	20.8005	21.5222	Down
2010309G	93.8096	1.17201	0.44844	2.61352	0.00896	0.14073	56.1797	63.2933	53.5776	149.172	183.243	57.3925	Up
Iglc2	323.346	0.90273	0.34849	2.5904	0.00959	0.14714	245.417	201.747	228.923	597.709	439.782	226.495	Up
Iglv3	53.4957	1.07622	0.49377	2.17962	0.02929	0.29719	48.2948	29.6687	25.3276	81.738	99.0501	36.8952	Up
Iglv2	113.856	1.73543	0.82811	2.09565	0.03611	0.33781	21.6834	45.4921	90.5949	254.41	254.559	16.3979	Up
Lamp3	27905.6	0.58317	0.20379	2.86156	0.00422	0.08564	27795.2	18248.3	20980	33851.8	37883.7	28674.7	Up
Klhl6	1009.6	0.49715	0.19847	2.50492	0.01225	0.17173	892.962	922.698	696.509	1265.92	1322.32	957.225	Up
Cyp2ab1	119.922	1.03519	0.44312	2.33613	0.01948	0.23533	104.475	67.2491	64.2931	131.803	260.502	91.2131	Up
Thpo	68.3776	-0.706	0.32685	-2.16	0.03077	0.30693	81.8055	71.205	101.31	65.3904	48.5345	42.0195	Down
Liph	2695.9	-0.3089	0.14831	-2.0825	0.03729	0.34397	2968.65	3001.49	2979.89	2202.84	2194.95	2827.6	Down
Adipoq	618.469	-6.2415	0.83182	-7.5035	6.22E-14	4.19E-11	651.487	2462.51	548.44	7.15208	22.7815	18.4476	Down
Rtp4	1911.2	0.40211	0.1703	2.36123	0.01821	0.22464	1633.15	1851.33	1455.36	2137.45	2499.03	1890.88	Up
Cldn1	607.982	0.63629	0.30104	2.11363	0.03455	0.32867	555.883	487.556	384.785	692.73	1037.05	489.886	Up
Uts2b	15.981	-4.4951	1.21531	-3.6987	0.00022	0.00994	13.7985	69.2271	8.76725	2.04345	0	2.04973	Down
Fgf12	424.108	-3.1961	0.54857	-5.8261	5.67E-09	1.57E-06	410.013	1456.74	427.647	63.347	136.689	50.2184	Down
Plaat1	43.257	-4.6305	0.94651	-4.8921	9.97E-07	0.00013	33.5107	177.023	38.9655	0	5.94301	4.09946	Down
Atp13a3	15462.7	0.26952	0.12091	2.22911	0.02581	0.27823	15093.6	13718.8	13255.1	15594.6	16869.2	18244.7	Up
Gm46565	290.032	-0.7307	0.30517	-2.3943	0.01665	0.21248	339.049	423.274	323.414	210.475	141.642	302.335	Down
Gm41442	481.22	-0.6429	0.23866	-2.6939	0.00706	0.12013	542.085	483.601	734.5	374.973	445.725	306.435	Down
Gm49767	221.037	-0.5688	0.17314	-3.285	0.00102	0.03178	255.273	272.952	263.992	182.889	189.186	161.929	Down
Apod	438.212	-0.8692	0.39391	-2.2067	0.02734	0.28614	400.157	880.173	418.88	204.345	439.782	285.938	Down

Bdh1	340.158	-0.865	0.39172	-2.2082	0.02723	0.28557	284.841	721.939	310.75	232.953	190.176	300.286	Down
Nrros	2171.22	0.41887	0.16273	2.57401	0.01005	0.15213	1860.83	1858.25	1855.73	2374.49	2959.62	2118.4	Up
Smco1	28.1095	-3.9756	0.7688	-5.1712	2.33E-07	3.91E-05	37.4531	89.9952	31.1724	2.04345	5.94301	2.04973	Down
Tm4sf19	5.3561	3.31122	1.40617	2.35478	0.01853	0.22715	0	0.98896	1.94828	2.04345	14.8575	12.2984	Up
Gm20056	16.1722	1.63964	0.65673	2.49668	0.01254	0.1741	6.89926	8.90062	7.79311	22.478	37.639	13.3233	Up
Kalrn	1504.25	0.27974	0.14236	1.96508	0.04941	0.39825	1440.96	1346.96	1288.79	1740	1773.99	1434.81	Up
Cd86	537.999	0.90316	0.33969	2.65875	0.00784	0.12826	490.833	308.555	325.362	616.1	1032.1	455.04	Up
Eaf2	164.063	1.24499	0.44049	2.82639	0.00471	0.09241	101.518	109.774	80.8535	195.15	383.324	113.76	Up
Fbxo40	241.101	-3.5479	1.13828	-3.1169	0.00183	0.04755	202.05	953.356	177.293	24.5214	82.2116	7.17406	Down
Gm4600	5.03317	2.36385	1.04262	2.26722	0.02338	0.2617	0.98561	1.97792	1.94828	8.1738	8.91451	8.19893	Up
Popdc2	1402.4	-1.4511	0.31418	-4.6188	3.86E-06	0.0004	1465.6	3195.32	1500.17	772.424	769.619	711.257	Down
Tigit	76.2996	1.2598	0.63766	1.97567	0.04819	0.39347	62.0933	47.47	25.3276	131.803	163.433	27.6714	Up
Drd3	17.562	-2.4821	0.95168	-2.6081	0.0091	0.14214	26.6114	31.6467	31.1724	3.06518	12.8765	0	Down
Sidt1	551.319	0.39953	0.14767	2.70552	0.00682	0.11755	497.732	476.678	452	562.971	633.921	684.61	Up
Cd200r1	377.899	1.20297	0.38088	3.15837	0.00159	0.04285	347.92	179.99	158.785	573.188	677.503	330.007	Up
Cd200r4	463.935	1.20672	0.50883	2.37154	0.01771	0.22074	497.732	187.902	155.862	544.58	1044.98	352.554	Up
Cd200r3	79.3038	-0.8027	0.40122	-2.0007	0.04543	0.38155	70.9638	109.774	121.767	57.2166	80.2306	35.8703	Down
Cd96	198.065	0.96284	0.22975	4.19085	2.78E-05	0.00197	138.971	135.487	128.586	237.04	324.884	223.421	Up
6430553K	2.13254	-4.5174	1.978	-2.2838	0.02238	NA	4.92804	4.94479	2.92242	0	0	0	Down
Morc1	49.8206	-1.8582	0.57666	-3.2223	0.00127	0.03687	36.4675	137.465	60.3966	22.478	26.7435	15.373	Down
Trat1	116.447	0.8749	0.3493	2.50473	0.01225	0.17173	90.676	67.2491	88.6466	173.693	187.205	91.2131	Up
Cip2a	232.044	0.65936	0.27206	2.42356	0.01537	0.19982	196.136	182.957	160.733	326.952	332.808	192.675	Up
G730013B	70.452	1.03695	0.45127	2.29784	0.02157	0.25004	27.597	57.3596	53.5776	97.0639	130.746	56.3676	Up
Impg2	60.6889	0.90377	0.35616	2.53756	0.01116	0.16233	41.3956	44.5031	40.9138	108.303	62.4016	66.6163	Up
Gpr15	20.5066	1.14598	0.51669	2.21793	0.02656	0.28171	11.8273	11.8675	14.6121	27.5866	36.6485	20.4973	Up
Pou1f1	19.3311	-1.5549	0.77811	-1.9983	0.04568	0.38232	15.7697	40.5473	30.1983	3.06518	6.93351	19.4725	Down
Vgll3	1906.26	-0.6079	0.17012	-3.5734	0.00035	0.01458	2031.34	2363.61	2511.33	1485.59	1748.23	1297.48	Down
Samsn1	792.855	0.87604	0.33758	2.59502	0.00946	0.14579	709.638	582.496	385.759	1006.4	1431.27	641.566	Up
Gm30790	3.78383	-5.3445	1.94429	-2.7488	0.00598	0.1081	3.94243	16.8123	1.94828	0	0	0	Down
Adamts1	5822.14	-0.4854	0.15557	-3.12	0.00181	0.04728	7311.25	7198.63	5867.24	4468.01	4608.8	5478.93	Down
Gm49708	515.976	-2.0707	0.90666	-2.2839	0.02238	0.25524	778.631	1477.5	244.509	107.281	381.343	106.586	Down
Mrap	156.236	-3.2985	0.66977	-4.9248	8.44E-07	0.00012	110.388	602.275	138.328	20.4345	47.544	18.4476	Down
Gm7831	8.24149	-2.5781	0.90898	-2.8363	0.00456	0.09101	15.7697	18.7902	7.79311	3.06518	1.981	2.04973	Down
Ifnar2	1646.52	0.31613	0.12551	2.51869	0.01178	0.16768	1500.1	1533.87	1366.72	1783.93	1982.98	1711.53	Up
Ifngr2	3827.4	0.24041	0.10772	2.23182	0.02563	0.27683	3505.81	3605.74	3416.3	4057.27	4462.21	3917.04	Up
D430001F	59.6783	-0.7804	0.35202	-2.2168	0.02663	0.28184	73.9207	91.9731	60.3966	36.7821	38.6295	56.3676	Down
Kcne2	1580.88	0.427	0.20333	2.09998	0.03573	0.33519	1449.83	1095.77	1500.17	1778.82	1509.52	2151.19	Up
1810053B	5.27968	-2.0752	1.04415	-1.9875	0.04687	0.3878	5.91365	13.8454	5.84483	2.04345	1.981	2.04973	Down
2410003I1	3.61598	-5.2791	1.84684	-2.8584	0.00426	0.08609	4.92804	13.8454	2.92242	0	0	0	Down
Lca5l	460.117	-0.6874	0.29893	-2.2995	0.02148	0.24997	484.919	612.165	605.914	314.691	236.73	506.284	Down
Sh3bgr	305.487	-1.8966	0.39139	-4.8457	1.26E-06	0.00016	318.352	806.001	320.492	138.955	115.889	133.233	Down
Fndc1	886.488	-0.7831	0.19019	-4.1175	3.83E-05	0.00254	1014.19	1110.6	1239.1	776.511	533.88	644.641	Down
Agpat4	3180.32	0.32467	0.13981	2.32229	0.02022	0.24068	2740.98	2737.44	2993.53	3803.88	3753.01	3053.08	Up
Slc22a3	324.528	-0.8936	0.29664	-3.0124	0.00259	0.06044	386.359	517.225	362.38	197.193	316.96	167.053	Down
Slc22a1	93.5844	-2.0589	0.4761	-4.3246	1.53E-05	0.00118	115.316	222.516	114.948	24.5214	24.7625	59.4422	Down
Airn	1419.39	-0.4137	0.13753	-3.0082	0.00263	0.06102	1618.37	1448.82	1797.29	1231.18	1152.94	1267.76	Down
Mrgprh	5.9249	-5.9915	1.84461	-3.2481	0.00116	0.0346	6.89926	26.7019	1.94828	0	0	0	Down
Gpr31c	8.37272	2.42545	1.06584	2.27562	0.02287	0.25842	3.94243	3.95583	0	17.3693	18.8195	6.1492	Up
Smoc2	628.012	-0.5615	0.25322	-2.2174	0.0266	0.28184	699.782	998.848	547.466	497.58	456.621	567.776	Down
Gm35576	9.05319	-4.6838	1.48215	-3.1601	0.00158	0.04279	7.88487	36.5914	7.79311	0	0	2.04973	Down
Has1	128.111	-1.0378	0.28938	-3.5862	0.00034	0.01402	222.748	129.554	164.629	83.7815	92.1166	75.8401	Down
Fpr1	535.746	1.38014	0.56239	2.45405	0.01413	0.18925	548.984	217.571	125.664	935.9	1071.72	314.634	Up
Vmn2r96	31.726	1.40692	0.48432	2.90496	0.00367	0.07734	17.741	21.7571	12.6638	49.0428	59.4301	29.7211	Up
Vmn2r97	137.463	2.96778	0.37222	7.97321	1.55E-15	1.24E-12	25.6258	45.4921	22.4052	275.866	285.264	170.128	Up
4930549P	8.94535	5.64005	1.4803	3.81006	0.00014	0.00698	0.98561	0	0	29.63	14.8575	8.19893	Up
Vmn2r10(	10.7298	6.89196	1.43501	4.80274	1.57E-06	0.00019	0	0	0	14.3042	33.677	16.3979	Up
Amdhd2	1134.92	0.32851	0.1055	3.11371	0.00185	0.04778	1031.93	1017.64	969.268	1240.37	1287.65	1262.63	Up
Ntn3	150.265	0.62932	0.20517	3.06737	0.00216	0.05336	127.144	124.609	102.285	187.997	182.252	177.302	Up
Ccnf	153.324	0.96531	0.30188	3.19765	0.00139	0.03942	101.518	108.785	101.31	250.323	226.825	131.183	Up
Abca17	280.69	-0.8172	0.29798	-2.7426	0.0061	0.10923	426.769	242.295	405.242	199.236	158.48	252.117	Down
Rpl3l	56.313	-2.5834	0.60826	-4.2471	2.17E-05	0.00156	73.9207	158.233	57.4742	16.3476	25.753	6.1492	Down
Gm50022	4.11715	-3.4777	1.75037	-1.9868	0.04694	0.38824	16.7553	3.95583	1.94828	2.04345	0	0	Down
Tpsb2	21.9117	-3.4456	1.15707	-2.9779	0.0029	0.06557	4.92804	102.852	12.6638	2.04345	6.93351	2.04973	Down
Cacna1h	313.422	-1.6394	0.44453	-3.688	0.00023	0.01027	269.071	731.829	422.776	93.9987	234.749	128.108	Down
Sox8	46.9877	1.54946	0.47767	3.24381	0.00118	0.03499	16.7553	33.6246	21.431	88.8901	80.2306	40.9946	Up
Mcrip2	468.009	-0.7403	0.29267	-2.5297	0.01142	0.16459	454.366	830.725	471.483	299.366	328.846	423.27	Down
A930017K	6.46943	3.14642	1.4725	2.13679	0.03262	0.31712	1.97122	1.97792	0	2.04345	28.7245	4.09946	Up
Pdia2	524.684	-0.7765	0.32099	-2.4192	0.01555	0.20168	552.926	701.171	733.526	319.8	257.53	583.149	Down
Dusp1	27643.8	-0.7704	0.24654	-3.1248	0.00178	0.04668	26500.1	45487.1	32575.2	23083.8	15917.3	22299	Down
Nkx2-5	55.8721	-5.0342	1.92616	-2.6136	0.00896	0.14073	45.338	222.516	57.4742	0	9.90501	0	Down
9630028I(	1259.87	-0.8232	0.41522	-1.9826	0.04741	0.39017	1809.58	1766.28	1253.72	767.316	420.963	1541.4	Down
Hmga1	1155.54	0.44038	0.14192	3.10307	0.00192	0.04912	911.688	1025.55	1004.34	1196.44	1317.37	1477.86	Up
Scube3	196.729	-0.7123	0.31407	-2.268	0.02333	0.2617	159.669	290.754	282.5	150.194	116.879	180.376	Down
Def6	563.437	0.47265	0.17699	2.6704	0.00758	0.12566	436.625	477.667	501.681	587.492	775.562	601.596	Up
Fkbp5	3919.4	0.96648	0.47604	2.03026	0.04233	0.37156	2477.82	3346.63	2136.29	4921.65	8921.44	1712.55	Up
Slc26a8	104.324	-0.8333	0.25912	-3.2157	0.0013	0.03739	121.23	132.52	147.095	71.5208	63.3921	90.1882	Down
Cpne5	130.57	1.16237	0.33498	3.46999	0.00052	0.01958	92.6472	80.1056	69.1638	258.497	143.623	139.382	Up
Pi16	1256.95	-1.6304	0.31655	-5.1504	2.60E-07	4.16E-05	1423.22	2910.5	1366.72	693.752	630.949	516.532	Down
Btbd9	1333.73	0.20303	0.1013	2.00417	0.04505	0.38066	1216.24	1254	1249.82	1411	1398.59	1472.73	Up
Abcg1	3900.48	1.0411	0.30386	3.42624	0.00061	0.0221	3843.87	1873.09	1936.59	5779.9	5714.2	4255.24	Up
Tff2	659.87	-3.793	0.7939	-4.7777	1.77E-06	0.00021	565.739	2414.05	713.069	79.6946	143.623	43.0444	Down
Ubash3a	138.224	1.58416	0.3799	4.16993	3.05E-05	0.00213	80.8199	65.2712	61.3707	227.845	281.302	112.735	Up
Ndufv3	2283.56	-0.29	0.14416	-2.0115	0.04427	0.37773	2250.14	2728.54	2558.09	1951.5	1924.54	2288.53	Down

Sik1	6499.75	-0.299	0.1391	-2.1495	0.03159	0.31109	7385.17	7776.18	6351.38	5319.1	5863.77	6302.93	Down
Rasal3	1057.72	0.77992	0.20913	3.72933	0.00019	0.00913	807.214	726.884	801.716	1479.46	1546.17	984.896	Up
Pglyrp2	138.997	0.92494	0.30152	3.06757	0.00216	0.05336	100.532	76.1498	111.052	198.215	218.901	129.133	Up
Cyp4f40	10.5689	-1.6726	0.81365	-2.0557	0.03981	0.35955	13.7985	26.7019	7.79311	4.0869	6.93351	4.09946	Down
Gm26693	130.654	0.48656	0.2185	2.22687	0.02596	0.27859	114.331	118.675	93.5173	144.063	155.509	157.829	Up
Gm50050	31.0569	0.80603	0.40148	2.00763	0.04468	0.37936	21.6834	20.7681	25.3276	46.9994	34.6675	36.8952	Up
Zfp870	579.444	0.28611	0.1333	2.14642	0.03184	0.31225	485.905	537.993	542.595	672.295	625.006	612.87	Up
Zfp472	773.003	0.3016	0.13119	2.29899	0.02151	0.25004	650.502	699.193	727.682	854.162	795.372	911.106	Up
Myo1f	2054.59	0.7615	0.34121	2.23176	0.02563	0.27683	1792.82	1554.64	1226.44	2260.06	3978.84	1514.75	Up
Kifc1	147.539	1.25188	0.33948	3.68764	0.00023	0.01027	103.489	99.8848	58.4483	268.714	204.043	150.655	Up
Tapbp	14259.2	0.22969	0.1136	2.02195	0.04318	0.37341	11985	13702	13692.5	15112.3	16228.4	14834.9	Up
H2-K2	1547.24	0.46467	0.18886	2.4604	0.01388	0.18685	1043.76	1346.96	1509.91	1678.69	2031.52	1672.58	Up
H2-K1	73124.9	0.62233	0.22047	2.82276	0.00476	0.09288	50283.8	62223.3	60272.9	80666.2	115845	69458.2	Up
H2-DMa	3378.61	0.58581	0.19472	3.00841	0.00263	0.06102	3048.49	2515.91	2541.53	3944.88	4950.52	3270.35	Up
H2-DMb1	3214.04	0.71051	0.18559	3.8284	0.00013	0.0066	2265.91	2461.52	2587.31	3741.56	4924.77	3303.14	Up
Psmb9	2385.56	0.39579	0.19783	2.00072	0.04542	0.38155	2318.15	1927.48	1935.61	2350.99	3400.39	2380.76	Up
Tap1	4110.19	0.76936	0.30624	2.51225	0.012	0.16977	3248.57	3178.51	2691.54	4267.75	7880.42	3394.36	Up
Psmb8	712.856	0.62087	0.27867	2.228	0.02588	0.27824	680.07	526.126	479.276	805.12	1175.72	610.82	Up
H2-Ab1	23362	0.86388	0.19732	4.37798	1.20E-05	0.00097	16635.1	16613.5	16459	29670.9	37712.3	23081	Up
H2-Aa	34901.6	0.87646	0.18542	4.72694	2.28E-06	0.00026	22604	24889.1	26350.4	43061.6	55618.6	36885.9	Up
H2-Eb1	19288.6	1.02997	0.26281	3.91901	8.89E-05	0.00489	8975.94	14471.4	14597.5	24554.1	33163.9	19968.5	Up
H2-Eb2	240.322	0.91105	0.28979	3.14382	0.00167	0.04435	185.294	178.012	137.354	364.756	368.466	208.048	Up
Btnl4	32.2505	6.01272	2.51565	2.39013	0.01684	0.21408	0	1.97792	0.97414	12.2607	178.29	0	Up
Vwa7	138.755	-0.5957	0.2899	-2.055	0.03988	0.35961	180.366	142.41	178.267	132.824	122.822	75.8401	Down
Clic1	11773.7	0.24611	0.08131	3.02688	0.00247	0.05873	10550	10825.1	10940.6	12674.5	12857.7	12794.4	Up
Aif1	307.18	0.7049	0.15599	4.51888	6.22E-06	0.0006	253.301	220.538	226.974	367.821	394.219	380.225	Up
Ltb	1012.87	0.54187	0.19635	2.75977	0.00578	0.10589	1019.12	720.95	734.5	1352.76	1144.03	1105.83	Up
Tnf	131.144	1.55478	0.41761	3.72302	0.0002	0.0093	82.7911	77.1387	39.9397	221.714	253.568	111.71	Up
Lta	39.4914	1.49863	0.39696	3.77526	0.00016	0.00783	26.6114	17.8012	17.5345	66.4121	60.4206	48.1687	Up
Gm18733	7.22669	-5.2821	2.12781	-2.4824	0.01305	0.17905	38.4387	0	3.89655	0	0	1.02487	Down
Gm11131	306.441	0.63654	0.21371	2.97851	0.0029	0.0655	238.517	194.825	286.397	362.713	417.991	338.206	Up
H2-Q5	2109.68	0.78686	0.37408	2.10349	0.03542	0.33353	1215.26	1721.78	1707.66	2209.99	4354.24	1449.16	Up
H2-Q7	8072.4	0.87193	0.3361	2.59425	0.00948	0.14581	5180.36	6016.82	5916.92	8249.41	16744.4	6326.5	Up
Tcf19	420.42	0.65788	0.27145	2.42357	0.01537	0.19982	403.114	305.588	269.836	678.426	397.191	468.364	Up
Tubb5	16007	0.49154	0.14157	3.47198	0.00052	0.0195	12386.1	12831.7	14700.7	20754.3	18638.3	16730.9	Up
H2-T22	4887.49	0.70931	0.26666	2.66	0.00781	0.12796	2708.45	4232.74	4187.82	5196.5	8122.11	4877.34	Up
Gm20495	2.87138	3.94289	1.81868	2.16799	0.03016	0.30293	0	0	0.97414	2.04345	3.962	10.2487	Up
H2-M5	175.198	-1.0528	0.28437	-3.7024	0.00021	0.00987	158.683	275.919	274.707	108.303	120.841	112.735	Down
Ubd	61.3413	3.05872	1.01652	3.00901	0.00262	0.06102	3.94243	29.6687	5.84483	77.6511	240.692	10.2487	Up
H2-M3	1402.41	0.42829	0.20673	2.07169	0.03829	0.35064	1280.31	1127.41	1179.68	1467.2	2058.26	1301.58	Up
H2-M2	177.228	2.98875	0.51971	5.7508	8.88E-09	2.40E-06	41.3956	42.5252	35.069	393.364	453.649	97.3623	Up
Olfr111	4.48848	5.63457	2.35396	2.39365	0.01668	0.21266	0	0	0	6.13035	20.8005	0	Up
Mep1a	13.1967	-1.9513	0.73801	-2.644	0.00819	0.13205	13.7985	24.724	24.3535	8.1738	1.981	6.1492	Down
Rcan2	2187.13	-0.7199	0.27413	-2.6262	0.00863	0.13709	2335.89	3856.94	1972.63	1721.61	1854.22	1381.52	Down
Nfkbie	624.298	0.75514	0.32286	2.33887	0.01934	0.234	546.027	467.777	379.914	769.359	1113.32	469.389	Up
Srf	2412.87	-0.359	0.13715	-2.6179	0.00885	0.13977	2405.87	2987.64	2741.23	1966.82	2172.17	2203.46	Down
Pgc	90.8444	0.94007	0.32818	2.86446	0.00418	0.08511	50.266	69.2271	67.2156	151.215	85.1831	121.959	Up
Treml4	385.074	1.04322	0.472	2.21023	0.02709	0.28467	239.503	331.301	184.112	297.322	954.843	303.36	Up
Trem2	320.893	1.28215	0.51398	2.49452	0.01261	0.17493	281.884	168.123	111.052	323.887	822.116	218.296	Up
Apobec2	195.005	-2.6821	0.48359	-5.5463	2.92E-08	6.63E-06	226.69	617.11	168.526	48.0211	58.4396	51.2433	Down
Unc5cl	265.551	-1.2236	0.29332	-4.1717	3.02E-05	0.00212	253.301	462.832	399.397	186.976	125.794	165.003	Down
Gm49896	6.40892	-4.1627	1.52277	-2.7336	0.00626	0.11134	1.97122	22.746	11.6897	1.02173	0	1.02487	Down
Rftn1	2366.56	0.57057	0.24711	2.30894	0.02095	0.24625	1683.42	1913.63	2116.8	2885.35	3654.95	1945.2	Up
Tbc1d5	1829.76	0.21673	0.10216	2.12139	0.03389	0.32468	1673.56	1723.75	1680.39	2071.04	1923.55	1906.25	Up
Sgo1	87.9932	1.14737	0.42992	2.66881	0.00761	0.12606	62.0933	55.3817	46.7587	168.585	135.699	59.4422	Up
Shd	35.3876	-1.2284	0.62435	-1.9674	0.04913	0.39723	44.3524	83.0725	21.431	21.4562	29.715	12.2984	Down
Plin4	1257.1	-2.4908	0.457	-5.4504	5.03E-08	1.09E-05	1376.9	3966.71	1059.86	286.083	461.573	391.499	Down
Plin5	496.679	-0.9338	0.37479	-2.4915	0.01272	0.1761	541.099	942.477	472.457	275.866	235.739	512.433	Down
Tnfaip8l1	125.782	0.71355	0.26167	2.72695	0.00639	0.11296	97.5753	83.0725	105.207	192.084	146.594	130.158	Up
Uhrf1	326.097	0.72292	0.23275	3.10605	0.0019	0.04886	245.417	251.195	241.586	509.841	403.134	305.41	Up
Ptprs	3548.48	-0.2282	0.09291	-2.4562	0.01404	0.18835	3874.43	3713.54	3897.53	3364.54	3173.56	3267.27	Down
Ndufa11	1227.05	-0.4039	0.20057	-2.0137	0.04405	0.377	1205.4	1616.95	1370.61	1040.12	869.66	1259.56	Down
Nrtn	165.789	-0.7542	0.37809	-1.9948	0.04606	0.38407	264.143	257.129	103.259	117.498	130.746	121.959	Down
Prr22	40.722	-0.9033	0.39494	-2.2872	0.02219	0.25456	41.3956	59.3375	58.4483	36.7821	23.772	24.5968	Down
Tubb4a	1576.23	-0.4839	0.21505	-2.2502	0.02444	0.26955	1360.14	2045.17	2109.01	1498.87	1244.07	1200.12	Down
Tnfsf14	136.833	0.89357	0.39013	2.29047	0.02199	0.25304	126.158	94.94	66.2414	203.323	226.825	103.511	Up
Vav1	942.966	0.71329	0.31385	2.27269	0.02304	0.25987	871.278	719.961	552.337	1127.98	1644.23	742.003	Up
Adgre1	1128.27	0.8678	0.43098	2.01356	0.04406	0.377	1166.96	792.155	437.388	1278.18	2279.14	815.793	Up
Gm20742	3.61271	-5.2779	1.91631	-2.7542	0.00588	0.10686	1.97122	14.8344	4.87069	0	0	0	Down
Gm36201	11.5483	-1.6063	0.80552	-1.9942	0.04613	0.38422	9.85609	29.6687	12.6638	4.0869	8.91451	4.09946	Down
Lama1	229.456	1.31378	0.29727	4.41954	9.89E-06	0.00083	152.769	99.8848	142.224	406.647	228.806	346.405	Up
Tgif1	1372.66	0.27787	0.10488	2.64957	0.00806	0.13085	1224.13	1290.59	1207.93	1462.09	1500.61	1550.62	Up
Gm26510	61.3513	0.68096	0.33264	2.04711	0.04065	0.36257	36.4675	45.4921	59.4225	86.8467	74.2876	65.5914	Up
Myom1	1904.14	-1.9082	0.43738	-4.3628	1.28E-05	0.00102	1774.1	5307.74	1939.51	519.036	1071.72	812.719	Down
Ndc80	134.755	1.09515	0.35979	3.04384	0.00234	0.05636	94.6184	100.874	62.3449	231.932	206.024	112.735	Up
Lbh	10664.6	-0.3631	0.08792	-4.1297	3.63E-05	0.00244	12352.6	12150.3	11495.8	9385.57	9335.47	9267.86	Down
Lclat1	3420.47	-0.3083	0.1424	-2.1649	0.0304	0.30453	3662.52	4170.44	3520.54	2972.2	2783.31	3413.83	Down
Capn13	103.263	-2.7123	0.48086	-5.6405	1.70E-08	4.21E-06	89.6904	243.284	204.569	39.8473	24.7625	17.4227	Down
Nlrc4	182.896	0.68381	0.33864	2.01932	0.04345	0.37418	163.611	152.3	105.207	218.649	310.027	147.581	Up
Sos1	243.586	-0.3456	0.17201	-2.0089	0.04455	0.37919	266.114	286.798	264.966	233.975	214.939	194.725	Down
Slc8a1	1550.09	-0.5799	0.28165	-2.0589	0.03951	0.35838	1319.73	2583.16	1669.67	1276.13	1465.94	985.921	Down
4933433H	3.66906	3.35879	1.69311	1.98379	0.04728	0.38984	0	0	1.94828	6.13035	11.886	2.04973	Up
Zfp36l2	7225.84	0.38539	0.11184	3.446	0.00057	0.0209	6636.1	6252.19	5911.07	8231.02	8666.88	7657.8	Up

Lrpprc	2819.14	-0.3378	0.16855	-2.004	0.04507	0.38066	3107.62	3743.21	2592.18	2480.75	2452.48	2538.59	Down
Gm30794	9.24926	-3.655	1.7192	-2.126	0.03351	0.32299	6.89926	44.5031	0	2.04345	0	2.04973	Down
Gm30938	4.61498	-4.6149	2.07329	-2.2259	0.02602	0.27903	10.8417	15.8233	0	0	0	1.02487	Down
Lhcgr	24.9088	-1.7389	0.55328	-3.1428	0.00167	0.04444	21.6834	50.4369	42.8621	10.2173	9.90501	14.3481	Down
Gm20939	151.798	0.41607	0.20812	1.99921	0.04559	0.382	140.942	124.609	124.69	159.389	195.129	166.028	Up
Greb1l	285.797	-2.5324	0.25184	-10.056	8.67E-24	1.17E-20	464.222	464.81	532.854	87.8684	58.4396	106.586	Down
Esco1	2055.63	0.36127	0.0995	3.63087	0.00028	0.0123	1786.91	1738.59	1873.27	2292.75	2374.23	2268.03	Up
Rbbp8	2458.05	1.04882	0.44046	2.38119	0.01726	0.21705	1912.08	1342.02	1551.8	2369.38	6047.01	1526.03	Up
Gm25289	2.2996	-4.6262	2.18043	-2.1217	0.03386	NA	0.98561	9.88958	2.92242	0	0	0	Down
Gm2629	28.311	1.1398	0.51157	2.22802	0.02588	0.27824	17.741	15.8233	19.4828	23.4997	57.449	35.8703	Up
Gm10036	1272.64	0.89567	0.33451	2.67753	0.00742	0.12358	687.955	584.474	1396.91	1571.41	1427.31	1967.74	Up
Cdh2	335.304	-1.4326	0.34623	-4.1376	3.51E-05	0.00239	357.776	762.487	347.767	186.976	202.062	154.755	Down
Dsc2	240.242	-2.4301	0.54996	-4.4186	9.93E-06	0.00083	289.769	690.293	235.742	38.8256	50.5155	136.307	Down
Fhod3	237.869	-2.2604	0.4624	-4.8884	1.02E-06	0.00013	297.654	628.977	254.25	61.3035	131.737	53.293	Down
Gm33228	145.572	-0.8562	0.42254	-2.0264	0.04273	0.37291	181.352	228.449	152.94	93.9987	164.423	52.2682	Down
Wdr33	4524.56	-0.3494	0.10522	-3.3208	0.0009	0.02919	4765.42	5016	5427.9	3936.71	3922.38	4078.97	Down
Kif20a	140.681	1.11502	0.42362	2.63214	0.00848	0.13561	134.043	72.1939	60.3966	261.562	200.081	115.81	Up
Cdc25c	26.1369	1.8313	0.56123	3.26301	0.0011	0.03339	13.7985	9.88958	10.7155	59.2601	40.6105	22.547	Up
Egr1	7448.43	-1.1089	0.38899	-2.8508	0.00436	0.08777	7756.74	12596.4	10180.7	6284.63	2034.49	5837.64	Down
Gm50163	55.5098	-1.063	0.48575	-2.1884	0.02864	0.29271	50.266	101.863	73.0604	36.7821	18.8195	52.2682	Down
Sting1	1671.45	0.60317	0.23509	2.56573	0.0103	0.15408	1323.67	1374.65	1282.94	1837.06	2711	1499.38	Up
E230025N	94.0487	-1.1146	0.39705	-2.8072	0.005	0.09613	109.403	192.847	83.7759	49.0428	56.4585	72.7655	Down
Arhgap26	224.843	0.50216	0.23098	2.17407	0.0297	0.30007	205.992	176.035	176.319	305.496	282.293	202.923	Up
Gm3650	167.185	5.8828	0.53406	11.0151	3.23E-28	4.98E-25	3.94243	7.91166	4.87069	512.906	207.015	266.465	Up
Eif3j2	521.574	3.14957	0.36535	8.6206	6.66E-18	7.19E-15	140.942	110.763	65.2673	1376.26	698.303	737.903	Up
Gm41715	14.4937	-4.3454	1.30555	-3.3284	0.00087	0.02862	13.7985	58.3485	10.7155	0	0	4.09946	Down
Ticam2	206.064	1.04664	0.42071	2.4878	0.01285	0.17736	156.712	142.41	104.233	277.909	423.934	131.183	Up
Snx24	1054.33	0.30258	0.14221	2.12772	0.03336	0.32248	1009.26	957.311	866.009	1250.59	1199.5	1043.31	Up
Ppic	5961.6	0.2482	0.12404	2.00093	0.0454	0.38155	5767.78	5146.54	5435.69	6878.26	5817.21	6724.14	Up
Lmnb1	1200.49	0.3656	0.11195	3.26575	0.00109	0.03316	1106.84	1039.39	1001.41	1361.96	1388.68	1304.65	Up
Adamts19	52.2979	-3.7206	0.68493	-5.432	5.57E-08	1.16E-05	80.8199	163.178	47.7328	3.06518	13.867	5.12433	Down
Chsy3	265.274	-0.9049	0.2793	-3.2397	0.0012	0.0353	349.891	451.954	235.742	169.606	203.053	181.401	Down
Gm4951	368.062	2.16234	0.5697	3.79555	0.00015	0.0073	216.834	90.9841	95.4656	459.776	1132.14	213.172	Up
Gm4841	34.0157	1.93533	0.86326	2.24188	0.02497	0.27329	19.7122	13.8454	8.76725	32.6952	119.851	9.22379	Up
Gm50237	473.565	2.62466	0.89158	2.94383	0.00324	0.0718	207.963	30.6577	157.81	601.796	1463.96	379.2	Up
Iigp1	2121.27	1.72681	0.50898	3.39272	0.00069	0.02414	1260.59	875.228	817.302	2803.61	5973.71	997.194	Up
Cd74	69967.8	0.77686	0.27692	2.80534	0.00503	0.0964	62773.4	46417.7	45525.4	86147.8	120385	58556.7	Up
Slc6a7	21.6524	-1.7075	0.62161	-2.7468	0.00602	0.10828	22.669	52.4148	24.3535	9.19553	6.93351	14.3481	Down
Pdgfrb	5811.52	-0.4065	0.14387	-2.8254	0.00472	0.09241	5682.03	6715.03	7477.49	4936.98	5211.02	4846.59	Down
Pcyox1l	615.741	0.37941	0.12238	3.10039	0.00193	0.04951	514.488	532.059	559.156	709.077	703.256	676.411	Up
1500015A	469.462	-0.5471	0.14644	-3.736	0.00019	0.00899	527.301	604.253	540.647	398.473	354.599	391.499	Down
Adrb2	6091.32	0.40264	0.15767	2.55364	0.01066	0.15725	5364.67	5279.06	5096.69	7883.63	7228.68	5695.18	Up
Alpk2	50.8121	-1.9578	0.69369	-2.8223	0.00477	0.09292	63.079	142.41	37.0173	24.5214	31.696	6.1492	Down
Malt1	2047.56	0.58642	0.2898	2.02357	0.04301	0.37324	1924.89	1537.83	1448.54	2220.21	3520.24	1633.64	Up
Gnal	86.1216	-0.6358	0.25937	-2.4511	0.01424	0.1903	99.5465	100.874	113.974	75.6077	69.3351	57.3925	Down
B430212C	17.1799	-1.3343	0.66084	-2.0191	0.04347	0.37418	15.7697	39.5583	18.5086	10.2173	12.8765	6.1492	Down
Cidea	524.979	-1.9323	0.41721	-4.6315	3.63E-06	0.00038	439.581	1424.1	632.216	196.171	186.214	271.589	Down
Ska1	42.4251	1.636	0.52331	3.12628	0.00177	0.04651	17.741	27.6908	16.5604	76.6294	85.1831	30.746	Up
Acaa2	8949.08	-0.5585	0.2144	-2.6052	0.00918	0.14264	11028	12974.1	7978.19	6642.24	6728.47	8343.43	Down
Lipg	2566.82	-0.3963	0.18937	-2.0925	0.0364	0.33907	2545.83	3150.82	3054.9	2059.8	2711	1878.58	Down
Gm7276	4.42473	-5.5706	1.63698	-3.403	0.00067	0.0234	5.91365	11.8675	8.76725	0	0	0	Down
St8sia5	10.9957	-3.9513	1.20722	-3.273	0.00106	0.0325	11.8273	33.6246	16.5604	0	3.962	0	Down
Slc14a1	265.216	-1.1142	0.25179	-4.4251	9.64E-06	0.00082	320.323	421.296	346.793	139.976	144.613	218.296	Down
Slc14a2	271.246	-2.0043	0.49593	-4.0415	5.31E-05	0.0033	221.762	625.022	455.897	90.9336	53.487	180.376	Down
Kcng2	109.571	-3.0664	0.7634	-4.0168	5.90E-05	0.00359	83.7767	388.661	114.948	6.13035	48.5345	15.373	Down
D330025C	11.8226	-6.9884	1.4723	-4.7465	2.07E-06	0.00024	13.7985	42.5252	14.6121	0	0	0	Down
Gm31621	4.60756	-3.0182	1.21478	-2.4846	0.01297	0.17832	7.88487	10.8785	5.84483	1.02173	0.9905	1.02487	Down
Cd226	626.213	0.76281	0.26124	2.91992	0.0035	0.07486	383.402	568.651	441.285	680.469	1044.98	638.491	Up
Tcirg1	5507.21	0.36021	0.12704	2.83536	0.00458	0.09104	4905.37	4708.43	4856.08	6012.85	6948.36	5612.17	Up
Unc93b1	5530.97	0.32574	0.16325	1.99534	0.04601	0.38389	5155.72	5083.24	4488.83	5702.25	7316.83	5438.96	Up
Doc2g	395.549	-2.2614	0.51875	-4.3594	1.30E-05	0.00103	306.524	1314.33	342.897	120.564	148.575	140.407	Down
Ndufv1	5638.23	-0.425	0.17875	-2.3774	0.01744	0.21842	5617.97	7969.02	5801	4894.06	4469.14	5078.21	Down
Carns1	347.488	-0.7433	0.28986	-2.5645	0.01033	0.15432	415.927	583.485	305.88	296.3	277.34	205.998	Down
Slc29a2	133.995	-0.8354	0.28511	-2.9302	0.00339	0.07381	158.683	193.836	162.681	128.737	84.1926	75.8401	Down
Cd248	334.224	-0.9473	0.22251	-4.2572	2.07E-05	0.00151	348.905	548.872	422.776	225.801	240.692	218.296	Down
Ctsw	417.135	0.6791	0.22356	3.03762	0.00238	0.05722	276.956	358.992	326.336	419.929	637.883	482.712	Up
Neat1	104716	-0.4157	0.13912	-2.9878	0.00281	0.06408	102309	127671	129115	94545.4	88156.6	86500.7	Down
Slc25a45	1405.07	0.32409	0.14675	2.20839	0.02722	0.28557	1247.78	1192.68	1303.4	1427.35	1797.76	1461.46	Up
Cdca5	84.1981	1.74723	0.38938	4.48721	7.22E-06	0.00068	28.5827	44.5031	42.8621	169.606	138.67	80.9644	Up
Batf2	375.758	0.8247	0.27714	2.97572	0.00292	0.06596	327.222	216.582	269.836	397.451	645.807	397.648	Up
Gm14963	61.9283	-0.8131	0.34429	-2.3616	0.0182	0.22454	60.1221	86.0394	90.5949	38.8256	39.62	56.3676	Down
Pygm	2734.34	-1.7145	0.34886	-4.9148	8.89E-07	0.00012	2770.55	6849.52	2954.56	1238.33	1471.88	1121.2	Down
Nrxn2	624.986	-0.5169	0.17795	-2.9048	0.00367	0.07734	685.984	705.127	816.328	475.102	606.187	461.19	Down
Ccdc88b	655.535	0.66858	0.31776	2.10408	0.03537	0.33334	617.977	450.965	450.052	685.578	1190.58	538.055	Up
Vegfb	1854.98	-0.4489	0.22702	-1.9774	0.048	0.39249	2043.17	2790.84	1589.79	1553.02	1460.99	1692.05	Down
Zbtb3	151.982	0.54932	0.24686	2.22518	0.02607	0.27935	118.273	103.841	148.069	185.954	203.053	152.705	Up
Gm50321	131.289	-1.85	0.6698	-2.762	0.00574	0.10587	194.165	313.5	109.104	18.3911	121.832	30.746	Down
Incenp	531.186	0.51553	0.23898	2.15716	0.03099	0.30787	521.387	424.263	366.276	652.883	748.819	473.488	Up
Cd5	321.395	1.03988	0.32957	3.15531	0.0016	0.04306	286.812	153.289	190.931	489.406	521.994	285.938	Up
A430093F	70.112	0.9349	0.47193	1.98102	0.04759	0.39078	52.2373	46.481	45.7845	113.412	123.813	38.9449	Up
Cd6	288.855	0.8583	0.3745	2.29185	0.02191	0.2525	246.402	169.112	200.673	394.386	530.908	191.65	Up
Slc15a3	767.404	1.06454	0.43324	2.45718	0.014	0.18794	759.904	383.716	345.819	910.357	1650.17	554.452	Up
AW11201	2906.31	0.55857	0.1644	3.3976	0.00068	0.02379	2418.68	2355.7	2277.54	3884.6	3703.48	2797.88	Up

Ms4a8a	1151.09	0.98764	0.4387	2.25128	0.02437	0.26917	1043.76	741.719	529.931	1574.48	2379.18	637.467 Up
Ms4a14	68.9713	0.94425	0.46728	2.02072	0.04331	0.3735	41.3956	54.3927	45.7845	62.3252	152.537	57.3925 Up
Ms4a7	290.333	1.11995	0.35782	3.12991	0.00175	0.04609	228.661	185.924	134.431	349.43	588.358	255.192 Up
Ms4a4b	1249.76	0.75289	0.19565	3.84814	0.00012	0.00618	978.709	848.526	965.371	1792.11	1709.6	1204.22 Up
Ms4a6d	684.874	1.37332	0.44743	3.06937	0.00215	0.05312	451.409	421.296	271.785	823.511	1704.65	436.593 Up
Oosp1	19.6778	2.41215	0.75034	3.21474	0.00131	0.03744	7.88487	5.93375	4.87069	29.63	57.449	12.2984 Up
Gm6365	61.6031	-1.1791	0.30903	-3.8154	0.00014	0.00686	71.9494	89.0062	95.4656	35.7604	42.5915	34.8454 Down
Mpeg1	9686.57	1.26057	0.41699	3.02302	0.0025	0.05921	8208.15	5195.99	3710.49	11110.2	22472.5	7422.08 Up
Lpxn	611.079	0.84285	0.30538	2.76	0.00578	0.10589	555.883	372.837	383.811	786.729	1057.85	509.358 Up
Ostf1	8198.64	0.24271	0.10993	2.20781	0.02726	0.28574	7512.31	7765.3	7254.41	8796.03	9590.03	8273.74 Up
Carnmt1	962.397	0.29271	0.14267	2.05168	0.0402	0.36086	963.925	830.725	800.742	982.9	1128.18	1067.91 Up
C730002L	30.5937	-3.0912	0.56892	-5.4335	5.53E-08	1.16E-05	75.8919	45.4921	42.8621	6.13035	3.962	9.22379 Down
Tmc1	9.32708	1.75818	0.83606	2.10293	0.03547	0.33377	3.94243	4.94479	3.89655	10.2173	24.7625	8.19893 Up
1110059E	2704.1	-0.2793	0.11998	-2.3277	0.01993	0.23828	2824.75	2942.15	3127.96	2494.03	2233.58	2602.13 Down
C330002G	391.808	-0.8543	0.27435	-3.1137	0.00185	0.04778	482.948	511.291	519.216	296.3	178.29	362.803 Down
Ptar1	4043.91	-0.2209	0.09746	-2.2668	0.02341	0.26189	4254.87	4198.13	4605.73	3686.39	3783.71	3734.61 Down
Fam189a2	11004.4	-0.4293	0.19908	-2.1565	0.03104	0.30805	9243.04	14034.3	14612.1	9288.51	9357.26	9491.28 Down
Pgm5	1359.13	-0.4208	0.18282	-2.302	0.02134	0.24947	1370.98	1918.58	1378.41	1210.74	1185.63	1090.46 Down
Kank1	2095.34	-0.378	0.17226	-2.1945	0.0282	0.28986	2426.57	2705.79	1972.63	1612.28	1942.37	1912.4 Down
Dmrt3	22.2151	-4.9997	1.2506	-3.9979	6.39E-05	0.00379	7.88487	103.841	17.5345	0	1.981	2.04973 Down
Gm35438.	11.5613	-1.9583	0.81631	-2.3989	0.01644	0.21099	20.6978	24.724	9.74139	2.04345	3.962	8.19893 Down
Vldlr	3037.26	-0.4401	0.20578	-2.1388	0.03245	0.31621	3091.85	4333.61	3065.61	2657.51	2929.9	2145.04 Down
Gm50114	24.9419	-4.3452	1.00662	-4.3166	1.58E-05	0.00121	17.741	82.0835	42.8621	1.02173	5.94301	0 Down
Kcnv2	22.9225	-2.6235	1.00864	-2.601	0.0093	0.14379	28.5827	74.1719	15.5862	12.2607	6.93351	0 Down
Gm5518	154.071	-0.8354	0.26807	-3.1163	0.00183	0.04756	200.079	178.012	214.31	80.7163	111.927	139.382 Down
Cd274	1755.95	0.67018	0.28339	2.3649	0.01804	0.22383	1480.38	1510.14	1075.45	1832.98	3064.61	1572.14 Up
Pdcd1lg2	70.2427	1.5199	0.60246	2.52282	0.01164	0.16637	32.5251	31.6467	44.8104	113.412	170.366	28.6962 Up
Mlana	199.886	-1.856	0.51531	-3.6016	0.00032	0.01336	207.963	596.342	135.405	92.977	71.3161	95.3125 Down
Gm36860	3.44565	-5.2096	1.74414	-2.9869	0.00282	0.06412	5.91365	9.88958	4.87069	0	0	0 Down
Acta2	12959.7	-0.8999	0.38651	-2.3281	0.01991	0.23828	8498.9	22171.5	19955.2	7781.46	6250.06	13100.9 Down
Ch25h	609.927	1.10697	0.32111	3.44728	0.00057	0.02084	487.876	348.113	324.388	923.64	1083.61	491.936 Up
Ifit3	2532.21	0.56466	0.25699	2.19718	0.02801	0.28884	2789.27	1763.31	1576.16	3394.17	3227.05	2443.28 Up
Ifit1	1215.38	0.60814	0.27173	2.23805	0.02522	0.27466	907.746	960.278	1020.9	1616.37	1878.98	908.031 Up
Kif20b	196.225	0.73733	0.3321	2.22023	0.0264	0.28101	154.741	153.289	133.457	298.344	298.141	139.382 Up
Ankrd1	5072.85	-2.6725	0.93194	-2.8676	0.00414	0.08457	2903.6	20085.7	3320.84	1424.29	1415.43	1287.23 Down
Gm32027	5.42597	-3.9093	1.43919	-2.7163	0.0066	0.11532	7.88487	14.8344	7.79311	2.04345	0	0 Down
Hectd2os	51.9303	-1.5079	0.66872	-2.2548	0.02414	0.26765	23.6546	153.289	53.5776	22.478	24.7625	33.8206 Down
Ppp1r3c	1179.5	-0.8158	0.36342	-2.2449	0.02478	0.27237	885.077	2434.81	1193.32	694.773	865.698	1003.34 Down
Kif11	323.021	1.05212	0.3173	3.3158	0.00091	0.02954	233.589	233.394	163.655	521.08	521.994	264.415 Up
Myof	7995.99	0.27521	0.13739	2.00315	0.04516	0.38075	7448.25	7044.35	7214.47	8773.56	9871.33	7623.98 Up
Cep55	136.642	1.75055	0.36975	4.73442	2.20E-06	0.00025	78.8487	57.3596	51.6293	304.474	189.186	138.357 Up
Ffar4	1329.91	-0.4677	0.20925	-2.2351	0.02541	0.27517	1534.59	1320.26	1775.85	998.226	959.795	1390.74 Down
Hells	264.705	0.64096	0.26382	2.4295	0.01512	0.19752	223.733	221.527	175.345	394.386	344.694	228.545 Up
Gm5827	3.94768	-3.4237	1.62223	-2.1105	0.03482	0.32956	15.7697	3.95583	1.94828	1.02173	0.9905	0 Down
Entpd1	8118.8	0.48906	0.21185	2.30846	0.02097	0.24642	6665.67	6417.35	7184.27	9702.3	11802.8	6940.39 Up
Blnk	828.541	0.42171	0.19032	2.21576	0.02671	0.28205	826.926	748.641	549.414	921.596	975.643	949.026 Up
Pik3ap1	2310.13	0.80166	0.31201	2.56935	0.01019	0.15322	2320.12	1460.69	1272.22	2804.64	3973.89	2029.23 Up
Gm6937	23.21	-1.9747	0.52624	-3.7524	0.00018	0.00854	31.5395	41.5362	37.9914	7.15208	13.867	7.17406 Down
Sfrp5	66.2987	-3.601	0.58369	-6.1695	6.85E-10	2.39E-07	87.7192	211.637	68.1897	6.13035	13.867	10.2487 Down
Golga7b	4.27636	-3.5615	1.68935	-2.1082	0.03501	0.3306	3.94243	16.8123	2.92242	0	1.981	0 Down
Got1	2301.79	-1.1936	0.30588	-3.9022	9.53E-05	0.00519	2292.53	4887.43	2429.5	1217.9	1536.27	1447.11 Down
Kcnip2	164.492	-0.9873	0.36617	-2.6963	0.00701	0.11961	215.848	318.445	121.767	104.216	112.917	113.76 Down
Col17a1	328.462	-0.7245	0.21655	-3.3457	0.00082	0.02719	409.028	429.208	389.655	239.084	308.046	195.749 Down
Rbm20	404.597	-2.1534	0.41985	-5.129	2.91E-07	4.56E-05	527.301	1070.05	384.785	140.998	205.034	99.412 Down
Adra2a	151.126	-1.4913	0.41229	-3.6171	0.0003	0.01279	156.712	302.621	209.44	71.5208	46.5535	119.909 Down
Gm50186	151.279	-1.3598	0.46357	-2.9335	0.00335	0.07334	226.69	177.023	249.379	62.3252	41.601	150.655 Down
Nrap	404.327	-3.5499	1.01866	-3.4849	0.00049	0.01875	387.344	1487.39	360.431	24.5214	140.651	25.6216 Down
Casp7	1911.23	0.26724	0.13174	2.0286	0.0425	0.37204	1902.22	1670.35	1631.68	2156.86	2195.94	1910.35 Up
Gm32441	54.6902	-0.8947	0.41201	-2.1716	0.02988	0.30087	64.0646	79.1166	70.138	30.6518	25.753	58.4174 Down
1700019N	3.43877	-4.1724	1.6654	-2.5054	0.01223	0.17173	5.91365	3.95583	9.74139	1.02173	0	0 Down
E330013P	8.55923	2.07598	0.8656	2.39831	0.01647	0.21109	5.91365	2.96687	0.97414	12.2607	13.867	15.373 Up
Csf2ra	2383.63	0.55121	0.24789	2.22365	0.02617	0.27994	2146.66	1929.46	1725.2	2849.59	3698.53	1952.37 Up
Cacna1f	21.0603	1.53398	0.53623	2.86069	0.00423	0.08572	6.89926	14.8344	10.7155	30.6518	37.639	25.6216 Up
Gata1	61.8674	1.20774	0.42758	2.82456	0.00473	0.09253	30.5539	53.4037	28.25	65.3904	120.841	72.7655 Up
Was	561.109	0.71629	0.31282	2.28976	0.02204	0.25315	526.315	419.318	328.285	648.796	977.624	466.314 Up
Xk	295.184	-0.407	0.17558	-2.3179	0.02045	0.2424	322.294	369.87	317.569	260.54	275.359	225.47 Down
Cybb	5447.99	1.45302	0.45544	3.19037	0.00142	0.03994	4011.43	2893.69	1840.15	7086.69	13469.8	3386.16 Up
Otc	62.2013	1.02793	0.41981	2.44852	0.01434	0.19133	20.6978	51.4258	50.6552	88.8901	70.3256	91.2131 Up
Tspan7	39107.6	0.25444	0.12588	2.0213	0.04325	0.37347	34406.6	32984.7	39612.4	44260.1	43718.7	39663.3 Up
AA414768	142.749	0.85518	0.29499	2.89906	0.00374	0.07863	141.928	87.0283	75.9828	184.932	185.224	181.401 Up
Ndp	18.202	-1.1793	0.53209	-2.2163	0.02667	0.28205	27.597	27.6908	20.4569	14.3042	8.91451	10.2487 Down
Zcchc12	51.9638	-1.1746	0.57255	-2.0515	0.04022	0.36086	47.3092	76.1498	92.5432	24.5214	13.867	57.3925 Down
Gm14569	107.897	-0.753	0.31166	-2.4162	0.01568	0.20282	183.323	122.631	100.336	81.738	74.2876	85.0639 Down
Sh2d1a	104.032	0.99706	0.40816	2.44283	0.01457	0.19345	60.1221	77.1387	71.1121	177.78	169.376	68.666 Up
Prr32	14.1378	-7.2463	1.51234	-4.7915	1.66E-06	0.0002	17.741	56.3706	10.7155	0	0	0 Down
Gm29242	39.7489	6.32234	1.10425	5.72545	1.03E-08	2.72E-06	0.98561	0	1.94828	64.3687	146.594	24.5968 Up
Sash3	1318.17	0.831	0.28752	2.89027	0.00385	0.08043	1126.55	862.371	857.242	1493.76	2407.91	1161.17 Up
Aifm1	2120.18	-0.3549	0.15686	-2.2623	0.02368	0.26383	2400.94	2713.7	2024.26	1780.87	1799.74	2001.56 Down
Rab33a	36.1086	-1.6891	0.67684	-2.4955	0.01258	0.17455	25.6258	97.9068	41.888	13.2824	28.7245	9.22379 Down
Arhgap36	16.047	-1.6432	0.72644	-2.262	0.0237	0.2639	29.5683	32.6356	10.7155	12.2607	4.9525	6.1492 Down
Igsf1	148.471	-3.8515	1.39226	-2.7663	0.00567	0.1051	211.906	567.662	53.5776	3.06518	50.5155	4.09946 Down
Rap2c	3298.79	0.27003	0.13163	2.05147	0.04022	0.36086	2948.94	2990.61	3033.47	3724.19	3947.15	3148.39 Up
Fhl1	53080.1	-0.3597	0.10898	-3.3001	0.00097	0.0307	55623.8	59483.9	63879.1	48535	46682.3	44276.3 Down

Fmr1nb	9.86999	4.8616	1.75804	2.76535	0.00569	0.10531	1.97122	0	0	2.04345	3.962	51.2433	Up
Hmgb3	606.196	-0.4146	0.13122	-3.1594	0.00158	0.04279	680.07	729.851	668.259	485.32	542.794	530.881	Down
Prrg3	5713.27	0.39098	0.17162	2.2782	0.02271	0.25741	5976.73	4278.23	4576.5	6736.24	6779.98	5931.92	Up
Cnga2	169.711	-1.2009	0.33179	-3.6195	0.0003	0.01275	304.553	175.046	229.897	103.194	71.3161	134.257	Down
Xlr4a	356.192	-1.4056	0.60655	-2.3173	0.02049	0.2424	108.417	679.414	763.725	139.976	301.112	144.506	Down
Srpk3	277.361	-0.6482	0.20395	-3.1784	0.00148	0.04105	318.352	395.583	301.983	247.258	199.091	201.899	Down
Pdzd4	314.987	-0.3728	0.15639	-2.3836	0.01715	0.21666	381.431	335.257	349.716	257.475	286.255	279.788	Down
Gm5640	9.23231	-2.3526	1.09006	-2.1582	0.03091	0.30734	14.7841	25.7129	5.84483	0	4.9525	4.09946	Down
Mpp1	4234.25	0.30985	0.15697	1.97387	0.0484	0.39439	4107.03	3780.79	3456.24	4457.79	5445.77	4157.88	Up
5430427C	182.633	0.75782	0.3496	2.1677	0.03018	0.303	171.496	133.509	102.285	233.975	310.027	144.506	Up
Arhgef9	310.589	-0.562	0.21366	-2.6301	0.00854	0.13593	331.165	432.175	347.767	233.975	299.131	219.321	Down
Asb12	9.69681	-3.1251	1.3318	-2.3465	0.01895	0.23054	12.8129	30.6577	8.76725	0	5.94301	0	Down
Las1l	2312.2	-0.3219	0.11062	-2.9104	0.00361	0.07634	2583.28	2470.42	2653.55	1988.28	1970.11	2207.56	Down
Tmem28	338.506	0.37384	0.18581	2.01198	0.04422	0.37762	335.107	275.919	273.733	338.191	428.887	379.2	Up
Kif4	141.068	0.70634	0.27355	2.58211	0.00982	0.14987	126.158	107.796	87.6725	218.649	156.499	149.63	Up
Gdpd2	841.718	-1.4686	0.22634	-6.4886	8.67E-11	3.34E-08	950.127	1421.13	1338.47	434.233	373.419	532.93	Down
Tex11	499.993	0.84272	0.2883	2.92308	0.00347	0.07476	526.315	270.975	276.655	664.121	612.13	649.765	Up
Itgb1bp2	397.247	-2.4482	0.41633	-5.8804	4.09E-09	1.21E-06	415.927	1164	434.466	136.911	131.737	100.437	Down
Cxcr3	285.296	0.8804	0.38789	2.26971	0.02322	0.26122	238.517	178.012	186.06	453.646	483.364	172.177	Up
Gm4779	2.77376	-4.8975	1.98231	-2.4706	0.01349	0.18332	1.97122	3.95583	10.7155	0	0	0	Down
Ercc6l	102.875	1.19675	0.33984	3.52152	0.00043	0.01682	70.9638	38.5694	77.9311	159.389	152.537	117.86	Up
Gm9109	1.96445	-4.3993	2.0663	-2.1291	0.03325	NA	1.97122	4.94479	4.87069	0	0	0	Down
Cox7b	3878.13	-0.4519	0.19322	-2.3385	0.01936	0.2341	4034.1	5340.37	4067.03	3287.91	2725.86	3813.53	Down
Tlr13	644.153	1.10191	0.44029	2.50271	0.01232	0.17227	522.373	443.053	263.017	710.099	1487.73	438.643	Up
Rtl3	9.05137	-2.5175	1.0406	-2.4193	0.01555	0.20168	7.88487	24.724	13.6379	0	3.962	4.09946	Down
Lpar4	80.2613	-0.7357	0.32294	-2.2781	0.02272	0.25741	90.676	110.763	99.3621	59.2601	42.5915	78.9147	Down
P2ry10b	245.708	0.73657	0.30307	2.43037	0.01508	0.19729	214.863	202.736	135.405	356.582	355.59	209.073	Up
Brwd3	788.34	0.26965	0.12694	2.12423	0.03365	0.32398	735.264	707.105	702.354	887.879	908.289	789.147	Up
Gm6377	216.591	0.70348	0.29454	2.38838	0.01692	0.21459	161.64	176.035	156.836	229.888	378.371	196.774	Up
Pcdh19	316.997	-0.4813	0.19664	-2.4477	0.01438	0.19165	312.438	383.716	412.061	278.931	285.264	229.57	Down
Nox1	86.98	0.86698	0.31854	2.7217	0.00649	0.11421	81.8055	48.4589	54.5518	130.781	109.946	96.3374	Up
Armcx4	3242.22	0.70997	0.23514	3.01933	0.00253	0.05972	2532.03	2020.44	2827.92	5157.67	3058.67	3856.57	Up
Armcx6	155.738	-0.6321	0.19648	-3.2171	0.00129	0.03729	199.093	171.09	197.75	123.629	118.86	124.009	Down
Tcp11x2	2.3549	4.70375	1.91361	2.45804	0.01397	0.18776	0	0	0	4.0869	5.94301	4.09946	Up
Arxes2	862.343	-0.43	0.2005	-2.1448	0.03197	0.31321	880.149	1239.16	850.423	719.295	679.484	805.545	Down
Arxes1	36.3148	-1.1674	0.58774	-1.9863	0.047	0.38829	39.4243	82.0835	29.2242	23.4997	11.886	31.7708	Down
Serpina7	117.23	-0.8238	0.26933	-3.0587	0.00222	0.05418	142.913	177.023	129.56	98.0856	88.1546	67.6411	Down
Cldn2	106.583	-2.0994	0.38166	-5.5008	3.78E-08	8.33E-06	149.813	233.394	135.405	34.7387	57.449	28.6962	Down
Pak3	61.2476	0.72146	0.34953	2.06408	0.03901	0.3548	48.2948	56.3706	34.0948	91.9553	75.2781	61.492	Up
Capn6	96.2437	1.06405	0.28378	3.74962	0.00018	0.00861	76.8775	58.3485	51.6293	148.15	130.746	111.71	Up
Sat1	14873	0.33106	0.12945	2.55751	0.01054	0.15671	12043.2	14374.5	13104.1	15683.5	15925.3	18107.3	Up
Smpx	1189.69	-4.9484	2.22461	-2.2244	0.02612	0.27969	1134.44	4565.03	1214.75	2.04345	221.872	0	Down
Klhl34	15.0397	-1.3619	0.62107	-2.1929	0.02832	0.29049	19.7122	30.6577	14.6121	7.15208	9.90501	8.19893	Down
Pdha1	3611.13	-0.4798	0.22303	-2.151	0.03147	0.31064	3923.71	5425.42	3269.21	2809.74	3476.66	2762.01	Down
Ctps2	2480.51	0.2248	0.10236	2.19605	0.02809	0.28953	2415.73	2228.12	2219.09	2611.53	2647.61	2760.99	Up
Rnf138rt1	54.6158	-1.2335	0.5109	-2.4144	0.01576	0.20358	76.8775	66.2602	86.6983	37.8038	11.886	48.1687	Down
Vegfd	3561.78	-0.5239	0.24366	-2.1502	0.03154	0.3109	4785.13	4557.12	3262.39	3034.52	3590.57	2140.94	Down
Asb11	153.511	-2.0639	0.63265	-3.2624	0.0011	0.03342	128.129	492.501	122.741	42.9125	103.012	31.7708	Down
Tlr8	185.184	1.00139	0.49501	2.02294	0.04308	0.37331	181.352	119.664	69.1638	271.779	361.533	107.611	Up
Tlr7	602.939	1.00081	0.43648	2.2929	0.02185	0.25197	556.869	445.031	203.595	794.902	1181.67	435.568	Up
Mid1-ps1	123.163	5.26933	1.43478	3.67257	0.00024	0.01083	2.95683	8.90062	6.81897	4.0869	300.122	416.096	Up

Supplementary Table 3 DEGs between the $Pten^{d/d}ELF3^{OV}$ vs WT groups

gene_id	baseMean	log2FoldC	lfcSE	stat	pvalue	padj	WT1	WT2	WT3	PtenddOv	PtenddOv	PtenddOv	change
Gm37381	28.7775	-0.9615	0.48215	-1.9943	0.04612	0.12806	28.0575	39.2886	46.7893	18.6609	13.7283	26.1404	Down
Rp1	1352.22	-0.5294	0.25304	-2.0923	0.03642	0.10657	1361.27	1852.31	1579.38	1184.97	690.641	1444.76	Down
Rgs20	66.5851	-2.3142	0.41551	-5.5695	2.55E-08	4.27E-07	71.5951	118.824	142.278	16.5875	20.0645	30.162	Down
Atp6v1h	5930.97	0.28272	0.08529	3.31495	0.00092	0.00518	5465.42	5397.87	5191.7	6853.73	6459.71	6217.4	Up
Npbwr1	150.774	2.07574	0.44029	4.71448	2.42E-06	2.75E-05	27.09	71.8694	74.4809	300.648	130.947	299.609	Up
Alkal1	852.143	2.9984	0.15859	18.9071	9.97E-80	7.17E-76	205.11	210.817	152.781	1622.46	1489	1432.7	Up
Adhfe1	464.938	-0.6252	0.16615	-3.7626	0.00017	0.0012	599.851	619.993	472.667	346.263	352.712	398.139	Down
Vcpip1	3150.63	0.20151	0.09864	2.04281	0.04107	0.11698	2915.08	2915.02	2963	3367.25	3107.88	3635.53	Up
1700034P	29.6901	-1.0491	0.45338	-2.3139	0.02067	0.06842	40.6351	41.2051	38.1953	22.8077	23.2326	12.0648	Down
Snhg6	291.975	-0.6476	0.18243	-3.5501	0.00039	0.00247	330.886	339.224	399.141	263.326	210.149	209.123	Down
Ppp1r42	219.669	-0.4982	0.25111	-1.9839	0.04726	0.13061	233.168	295.144	243.495	207.343	126.723	212.14	Down
Arfgef1	7732.01	0.24629	0.07331	3.35947	0.00078	0.00453	7075.34	7065.24	7080.46	8292.69	8234.89	8643.43	Up
Prex2	10533.5	-0.5712	0.09251	-6.175	6.61E-10	1.50E-08	13009	11933.2	12833.6	8159.99	9070.2	8195.02	Down
A830018L	355.107	-1.1961	0.25932	-4.6127	3.98E-06	4.30E-05	392.806	597.953	492.72	204.233	158.404	284.528	Down
Slco5a1	126.865	-0.6062	0.26164	-2.3168	0.02052	0.06809	180.923	163.862	114.586	103.672	106.659	91.4915	Down
Prdm14	1.91735	-4.3359	2.12192	-2.0434	0.04102	NA	1.935	5.74955	3.81953	0	0	0	Down
Lactb2	3390.34	0.27746	0.09577	2.89719	0.00377	0.01708	3174.37	2966.77	3054.67	3552.83	3997.06	3596.32	Up
Xkr9	11.9692	6.10204	1.37332	4.44326	8.86E-06	8.82E-05	0.9675	0	0	18.6609	19.0085	33.1782	Up
Sbspon	100.389	-0.8306	0.34378	-2.416	0.01569	0.05502	79.3351	177.278	128.909	67.3865	76.0338	73.3943	Down
Ly96	395.405	0.50939	0.18772	2.71351	0.00666	0.02734	361.846	306.643	310.337	465.485	548.077	380.042	Up
Gdap1	6.78673	-2.7708	1.10198	-2.5143	0.01193	0.0441	7.74001	13.4156	14.3232	2.07343	3.16808	0	Down
Gm28153	102.571	-1.2403	0.27829	-4.4567	8.32E-06	8.35E-05	169.313	120.741	142.278	52.8725	73.9218	56.3024	Down
Il17f	14.8518	2.26053	1.02607	2.2031	0.02759	0.08609	7.74001	5.74955	1.90977	40.4319	2.11205	31.1674	Up
Mcm3	1170.68	0.38274	0.16225	2.35887	0.01833	0.06238	1169.71	803.979	1075.2	1278.27	1392.9	1304	Up
Paqr8	3241.66	1.44135	0.20953	6.87901	6.03E-12	1.93E-10	2020.14	1391.39	1822.87	4188.33	6152.4	3874.81	Up
Gm28064	11.6907	3.48533	1.06516	3.27212	0.00107	0.00589	0	1.91652	3.81953	8.29373	38.0169	18.0972	Up
Tmem14a	542.148	-0.3821	0.16269	-2.3486	0.01885	0.06376	579.533	665.99	594.892	540.129	475.211	397.133	Down
Gsta3	789.332	-1.2411	0.27172	-4.5677	4.93E-06	5.22E-05	1532.52	973.591	822.154	505.917	334.76	567.046	Down
Gm4956	5006.48	-0.8126	0.14508	-5.6014	2.13E-08	3.60E-07	7368.49	6018.82	5754.12	3697.97	3162.8	4036.68	Down
Kcnq5	623.961	-0.7014	0.18269	-3.839	0.00012	0.00091	697.569	775.231	845.071	445.788	583.982	396.128	Down
Ogfrl1	2359.02	1.00848	0.15855	6.36059	2.01E-10	5.09E-09	1943.71	1398.1	1357.84	3110.15	3234.61	3109.7	Up
Smap1	758.279	0.37516	0.13391	2.80147	0.00509	0.02203	736.269	604.661	639.772	812.785	917.686	838.504	Up
Gm10925	3010.32	-0.6242	0.24925	-2.5043	0.01227	0.0452	2877.35	5424.7	2652.66	2311.88	2381.34	2413.97	Down
Gm29216	1095.66	-0.574	0.26631	-2.1555	0.03112	0.09425	1114.56	1917.48	900.455	860.474	763.506	1017.47	Down
Gm9898	22.8572	-1.22	0.52788	-2.3112	0.02082	0.06879	31.9275	22.0399	42.0148	14.514	10.5603	16.0864	Down
Prim2	518.552	0.48114	0.15439	3.11646	0.00183	0.00925	484.718	367.013	446.885	629.287	609.327	574.084	Up
Bend6	175.146	1.00378	0.25187	3.98533	6.74E-05	0.00054	127.71	115.949	105.992	178.315	295.687	227.221	Up
Dst	8879.76	-0.4411	0.08993	-4.9049	9.35E-07	1.16E-05	10032	10504.4	10143.7	6993.69	7945.53	7659.14	Down
Ptpn18	856.377	0.885	0.14045	6.30132	2.95E-10	7.20E-09	556.313	692.821	555.742	1173.56	1116.22	1043.61	Up
4930568A	6.93659	2.38073	1.18485	2.00931	0.0445	0.12451	0	4.79129	1.90977	17.6242	4.2241	13.0702	Up
Plekhb2	9657.13	0.85974	0.11777	7.29997	2.88E-13	1.13E-11	7847.4	6283.3	6455.01	12881.2	12507.6	11968.3	Up
Hs6st1	1386.71	0.7382	0.14086	5.24083	1.60E-07	2.29E-06	1125.2	962.092	1031.27	1493.91	1759.34	1948.47	Up
Uggt1	5033.73	0.47276	0.09681	4.88359	1.04E-06	1.28E-05	4586.92	4028.52	4033.43	5723.71	6045.75	5784.07	Up
Neurl3	1922.85	0.43653	0.12482	3.49728	0.00047	0.00294	1819.87	1449.85	1632.85	2098.31	2177.52	2358.67	Up
Sema4c	1699.49	-0.3671	0.11596	-3.1656	0.00155	0.00804	1750.21	1880.1	2113.16	1489.76	1548.13	1415.6	Down
Cox5b	2232.94	-0.3383	0.13453	-2.5148	0.01191	0.04407	2223.32	2865.19	2391.98	2070.32	2038.13	1808.72	Down
Zap70	597.093	-0.808	0.21237	-3.8046	0.00014	0.00103	841.726	722.527	716.162	484.146	306.247	511.749	Down
Tmem131	4590.36	0.27658	0.08741	3.16411	0.00156	0.00807	4289.9	4097.51	4067.8	4781.33	5017.18	5288.41	Up
Vwa3b	1101.6	-0.5047	0.24869	-2.0294	0.04242	0.11998	1192.93	1390.43	1293.87	1005.61	555.469	1171.29	Down
Mgat4a	1715.18	0.75147	0.09911	7.58228	3.40E-14	1.55E-12	1185.19	1330.06	1319.65	2122.16	2133.17	2200.82	Up
2010300C	3321.53	0.51042	0.1349	3.78355	0.00015	0.00111	2966.36	2754.99	2498.93	3975.81	3366.61	4366.46	Up
Mitd1	1324.63	0.2404	0.11224	2.14185	0.03221	0.09671	1208.41	1176.74	1258.54	1529.16	1301.02	1473.92	Up
Aff3	1852.7	-0.4565	0.11846	-3.8539	0.00012	0.00087	2203	1951.97	2275.49	1495.98	1481.6	1708.18	Down
Lonrf2	31.5328	-1.328	0.60735	-2.1865	0.02878	0.0891	26.1225	80.4937	28.6465	14.514	25.3446	14.0756	Down
Pdcl3	3939.93	-0.3992	0.11488	-3.4751	0.00051	0.00316	4034.48	4546.94	4863.22	3542.46	3517.62	3134.84	Down
Npas2	441.543	-1.296	0.4204	-3.0828	0.00205	0.01017	221.558	957.3	703.749	224.967	225.989	315.696	Down
Cnot11	1975.75	0.21684	0.0969	2.23785	0.02523	0.08028	1882.76	1901.19	1698.74	2175.03	2102.55	2094.25	Up
Rnf149	2442.93	1.7212	0.15684	10.9743	5.08E-28	8.98E-26	1322.57	1145.12	943.424	3297.79	4046.69	3901.96	Up
Creg2	120.031	1.2933	0.4336	2.9827	0.00286	0.01349	93.8476	88.1598	26.7367	167.948	139.395	204.096	Up
Il1r1	2703.48	-0.3313	0.12187	-2.7183	0.00656	0.02704	2683.85	3195.79	3157.8	2181.25	2565.09	2437.09	Down
Il1rl2	963.615	0.40357	0.18408	2.19235	0.02835	0.08808	655.966	1027.25	805.921	1017.02	1076.09	1199.44	Up
Il18r1	8883.91	0.40544	0.1274	3.18243	0.00146	0.00767	6461.94	8223.78	8245.41	9640.42	10687	10045	Up
Il18rap	743.7	0.8437	0.16496	5.1146	3.14E-07	4.31E-06	495.361	596.995	504.178	983.843	1084.54	797.283	Up
Slc9a4	765.427	2.14879	0.33959	6.32769	2.49E-10	6.17E-09	449.888	242.439	152.781	1250.28	1525.96	971.217	Up
Slc9a2	353.065	-0.6312	0.18305	-3.4483	0.00056	0.00343	457.628	473.38	356.171	264.363	310.471	256.377	Down
Tmem182	474.485	-6.0373	1.45275	-4.1558	3.24E-05	0.00028	590.176	1811.11	402.961	14.514	0	28.1512	Down
Mrps9	2158.73	-0.3206	0.10136	-3.1629	0.00156	0.0081	2552.27	2365.94	2274.53	1853.65	2034.96	1871.05	Down
Fhl2	892.142	-1.3997	0.67882	-2.0619	0.03922	0.11276	687.894	2360.19	833.613	543.239	250.278	677.64	Down
Nck2	2201.33	-0.2999	0.0882	-3.4001	0.00067	0.004	2379.09	2400.44	2508.48	1954.21	1917.74	2048	Down
Ecrg4	135.354	1.71049	0.34232	4.99679	5.83E-07	7.61E-06	54.1801	90.0763	45.8344	256.069	146.788	219.177	Up
Uxs1	1786.08	0.32314	0.09177	3.52108	0.00043	0.00272	1579.93	1585.92	1594.65	1936.59	2093.04	1926.35	Up
Tpp2	3893.19	0.28252	0.10012	2.82169	0.00478	0.02088	3484.94	3566.64	3488.19	3891.83	4332.87	4594.68	Up
Ercc5	1887.45	-0.312	0.10962	-2.8461	0.00443	0.01956	2099.48	1896.39	2276.44	1686.74	1648.46	1717.22	Down
Mettl21e	117.944	-0.7625	0.26759	-2.8497	0.00438	0.01937	128.678	168.654	148.007	74.6435	77.0899	110.594	Down
Gulp1	1411.23	-0.5544	0.10567	-5.2463	1.55E-07	2.23E-06	1677.65	1648.2	1711.15	1168.38	1213.37	1048.63	Down
Col3a1	11687.2	0.47634	0.20019	2.37944	0.01734	0.0596	6847.01	11237.5	11240.9	15442.9	13211.9	12143.2	Up
Col5a2	3072.16	0.40582	0.13692	2.96403	0.00304	0.01424	2253.31	2801.95	2873.24	3734.25	3625.34	3144.89	Up
Dnah7b	797.16	-0.6922	0.24139	-2.8674	0.00414	0.01851	901.711	1058.88	994.033	668.682	386.505	773.153	Down
Dnah7c	631.812	-0.9946	0.20958	-4.7457	2.08E-06	2.39E-05	782.709	797.271	944.379	431.274	309.415	525.825	Down
Tmeff2	617.075	-1.1298	0.2176	-5.1922	2.08E-07	2.93E-06	641.453	892.139	1007.4	359.74	469.931	331.782	Down
9330175M	746.156	-0.9655	0.14087	-6.8534	7.21E-12	2.30E-10	858.174	1017.67	1084.75	488.293	535.405	492.646	Down
Nabp1	3288.27	1.48504	0.18354	8.09115	5.91E-16	3.44E-14	1789.88	1853.27	1549.77	5102.72	5815.53	3618.44	Up

Stat1	3180.06	0.36795	0.13094	2.81008	0.00495	0.02156	2829.94	2972.52	2527.58	3246.99	4089.99	3413.34 Up
Nab1	5225.42	0.3063	0.10044	3.04953	0.00229	0.01117	4777.52	4451.11	4789.69	5277.92	6134.45	5921.81 Up
Gm553	117.771	-1.0173	0.26438	-3.848	0.00012	0.00089	134.483	162.904	175.698	78.7904	61.2495	93.5023 Down
Mstn	4.83087	-3.6888	1.59257	-2.3163	0.02054	0.06814	5.80501	17.2487	3.81953	0	2.11205	0 Down
Osgepl1	679.229	-0.73	0.15364	-4.7513	2.02E-06	2.33E-05	796.254	993.714	752.448	500.734	538.573	493.652 Down
Dnah7a	1053.67	-0.4977	0.23658	-2.1039	0.03539	0.10413	1222.92	1450.8	1027.45	913.347	594.542	1112.98 Down
Hecw2	2641.44	-0.7858	0.15983	-4.9165	8.81E-07	1.10E-05	2802.85	3324.2	3903.56	1688.81	1971.6	2157.59 Down
Ccdc150	441.833	-0.7215	0.22609	-3.1913	0.00142	0.00747	463.433	551.957	634.997	354.557	242.886	403.166 Down
Pgap1	637.534	0.51335	0.16698	3.07431	0.00211	0.01041	640.486	457.089	478.396	739.178	732.882	777.175 Up
Ankrd44	5299.74	-0.6047	0.12519	-4.8304	1.36E-06	1.63E-05	5728.58	6439.5	7015.52	3807.86	4187.14	4619.82 Down
Hspe1	2247.38	-0.3389	0.14129	-2.3985	0.01646	0.05721	2291.04	2348.69	2890.43	2173.99	2009.62	1770.51 Down
Rftn2	1155.38	-1.0979	0.13613	-8.0652	7.31E-16	4.14E-14	1434.8	1693.24	1596.56	702.893	832.148	672.613 Down
Gm10561	101.077	-1.0049	0.3216	-3.1246	0.00178	0.00905	89.9776	156.196	158.511	62.203	80.2579	59.3187 Down
Plcl1	964.501	-0.5032	0.10898	-4.6174	3.89E-06	4.22E-05	1193.9	1080.92	1118.17	830.409	801.523	762.094 Down
Maip1	857.873	-0.262	0.11678	-2.2436	0.02486	0.07931	912.354	982.215	911.913	767.17	841.652	731.932 Down
Aox1	4786.63	-0.6436	0.19066	-3.3759	0.00074	0.0043	7640.36	4670.55	5200.29	3794.38	3384.56	4029.65 Down
Aox3	7612.15	-1.2998	0.20957	-6.2022	5.57E-10	1.28E-08	12753.6	10192	9534.51	5103.75	3052.97	5036.05 Down
Clk1	16064.1	-0.5414	0.09031	-5.9954	2.03E-09	4.21E-08	18300.3	18844.2	19986.7	12939.3	12416.7	13897.7 Down
Orc2	107.756	0.71249	0.26149	2.72477	0.00643	0.02664	101.588	68.9946	74.4809	147.214	123.555	130.702 Up
Flacc1	123.078	-0.5393	0.2498	-2.1589	0.03086	0.09369	115.133	159.071	163.285	102.635	89.7622	108.583 Down
Casp8	3482.41	0.67571	0.12655	5.33935	9.33E-08	1.39E-06	2931.53	2528.84	2583.91	4163.45	4824.98	3861.74 Up
Trak2	5450.66	-0.2449	0.11637	-2.1048	0.03531	0.10397	5218.7	6071.53	6446.41	4722.24	5285.41	4959.64 Down
Tmem237	1376.46	-0.427	0.11459	-3.7268	0.00019	0.00136	1577.99	1488.18	1670.09	1080.26	1177.47	1264.79 Down
Als2	409.382	0.60298	0.19368	3.11327	0.00185	0.00934	393.773	256.813	324.66	460.302	487.884	532.862 Up
Gm26813	93.1747	0.82505	0.35688	2.31186	0.02079	0.06871	64.8226	71.8694	64.932	126.479	161.572	69.3727 Up
Bmpr2	33889.6	-0.2551	0.11608	-2.1974	0.028	0.08716	37423.9	34112.1	39096.7	28660	34744.3	29300.4 Down
Ica1l	187.762	-0.5645	0.22354	-2.5253	0.01156	0.04306	220.59	257.772	193.841	127.516	148.9	177.956 Down
Carf	653.974	-0.3247	0.12894	-2.518	0.0118	0.04378	653.063	764.69	763.906	565.01	605.103	572.073 Down
Eif4a-ps4	2407.7	0.40191	0.1261	3.18717	0.00144	0.00756	2381.99	1880.1	1961.33	2722.42	2869.22	2631.13 Up
Cd28	212.6	-0.5907	0.23094	-2.5578	0.01053	0.03991	292.185	251.064	223.443	199.049	129.891	179.967 Down
Gm11581	7.08355	-3.2055	1.19103	-2.6914	0.00712	0.02892	4.83751	16.2904	17.1879	2.07343	2.11205	0 Down
Pard3b	1683.85	-0.3904	0.11125	-3.5089	0.00045	0.00283	2032.72	1859.02	1839.1	1331.14	1547.08	1494.03 Down
Nrp2	4051.59	0.63112	0.13089	4.82181	1.42E-06	1.69E-05	3551.7	2936.1	3049.9	4455.8	5528.29	4787.72 Up
Ndufs1	1115.1	-0.4065	0.18358	-2.2144	0.0268	0.08415	1167.77	1595.5	1050.37	893.649	915.574	1067.74 Down
Zdbf2	19.6191	-2.6399	0.97663	-2.7031	0.00687	0.0281	12.5775	34.4973	54.4283	4.14686	0	12.0648 Down
Gm39653	6.62711	-2.7451	1.04285	-2.6323	0.00848	0.03335	13.545	14.3739	6.68418	1.03672	2.11205	2.0108 Down
Dytn	12.7578	-2.5314	0.80517	-3.144	0.00167	0.00855	21.285	26.8312	17.1879	1.03672	3.16808	7.03781 Down
Mdh1b	600.231	-1.0494	0.26243	-3.999	6.36E-05	0.00051	698.536	974.549	755.312	431.274	246.054	495.663 Down
2810408I1	12.6777	1.76796	0.7167	2.46681	0.01363	0.04922	2.9025	9.58259	4.77441	22.8077	16.8964	19.1026 Up
Fzd5	1520.31	0.60961	0.12258	4.973	6.59E-07	8.49E-06	1248.08	1059.83	1303.42	1883.71	1894.51	1732.31 Up
Plekhm3	1822.36	0.26174	0.12341	2.12092	0.03393	0.10073	1877.92	1562.92	1531.63	1941.77	2103.6	1916.29 Up
Akr1cl	30.107	-1.7039	0.62337	-2.7333	0.00627	0.02609	29.9925	84.3268	23.8721	15.5507	15.8404	11.0594 Down
Idh1	9154.53	0.62711	0.13981	4.48552	7.27E-06	7.43E-05	8108.63	7625.82	5852.48	11230.7	11382.9	10726.6 Up
Pth2r	26.1467	-1.6937	0.5192	-3.2621	0.00111	0.00607	29.025	38.3303	52.5186	13.4773	8.4482	15.081 Down
Map2	1723.3	0.65178	0.14797	4.40491	1.06E-05	0.0001	1452.22	1331.98	1237.53	1808.03	2051.86	2458.21 Up
Rpe	2291.42	0.48301	0.11006	4.38884	1.14E-05	0.00011	2101.41	1765.11	1867.75	2553.43	2673.86	2786.97 Up
Acadl	7919.24	-0.393	0.1666	-2.3591	0.01832	0.06236	8849.74	10973	7151.12	7360.68	6922.25	6258.62 Down
Myl1	83.0461	-3.5215	0.71119	-4.9516	7.36E-07	9.41E-06	64.8226	328.683	64.932	12.4406	5.28013	22.1188 Down
Erbb4	145.1	-4.0231	0.55934	-7.1925	6.36E-13	2.37E-11	170.28	439.841	210.074	31.1015	4.2241	15.081 Down
Gm28497	15.6193	2.83483	0.7678	3.69213	0.00022	0.00152	3.87001	5.74955	1.90977	36.2851	13.7283	32.1728 Up
Abca12	8.5099	-2.8877	1.35932	-2.1244	0.03364	0.10007	4.83751	23.9565	16.233	0	0	6.03241 Down
Fn1	28798.5	0.89219	0.15619	5.71214	1.12E-08	2.00E-07	21761	20967.7	17772.3	33880.9	45439.7	32969.1 Up
Apol7d	10.8217	1.46219	0.73041	2.00189	0.0453	0.1262	4.83751	8.62433	3.81953	14.514	20.0645	13.0702 Up
4933417E	11.4988	1.58141	0.73525	2.15084	0.03149	0.09515	3.87001	9.58259	3.81953	14.514	21.1205	16.0864 Up
Mreg	447.452	1.08124	0.20139	5.36884	7.92E-08	1.20E-06	218.655	339.224	303.653	557.753	684.304	581.122 Up
Tmem169	7.68875	2.32004	0.92646	2.50421	0.01227	0.0452	3.87001	1.91652	1.90977	12.4406	17.9524	8.04321 Up
Xrcc5	2206.36	0.41541	0.12079	3.43895	0.00058	0.00354	1853.73	2049.72	1769.4	2600.08	2255.67	2709.56 Up
Rpl37a	5574.55	-0.5166	0.14778	-3.4957	0.00047	0.00296	6557.72	5592.4	7535.94	4933.73	4826.04	4001.5 Down
Igfbp2	9512.84	-1.7587	0.1813	-9.7004	3.00E-22	3.19E-20	14962.4	16124.6	12970.2	4854.94	4950.65	3214.27 Down
Tns1	52954.7	-0.6323	0.1242	-5.0913	3.56E-07	4.83E-06	62643.8	62077.9	68410.7	44071.8	45183.1	35340.8 Down
Rufy4	316.839	2.24617	0.33916	6.62284	3.52E-11	1.00E-09	161.573	114.991	54.4283	472.742	616.719	480.582 Up
Cxcr1	372.24	3.82193	0.78857	4.84667	1.26E-06	1.51E-05	101.588	27.7895	18.1428	529.762	1044.41	511.749 Up
Arpc2	19603.7	0.44992	0.11506	3.91043	9.21E-05	0.00071	17396.6	15849.6	16467.9	22365.1	25366.8	20176.4 Up
Pnkd	1873.61	-0.2619	0.10711	-2.4451	0.01448	0.0516	2009.5	2056.42	2063.5	1856.76	1698.09	1557.37 Down
Catip	2190.57	-0.7607	0.20338	-3.7403	0.00018	0.0013	2465.19	3047.26	2752.93	1918.96	1136.28	1822.79 Down
Slc11a1	703.377	0.58432	0.20752	2.81571	0.00487	0.02123	669.511	596.037	423.013	716.371	976.823	838.504 Up
Vil1	144.517	1.53501	0.32227	4.76312	1.91E-06	2.21E-05	43.5376	96.7841	82.1199	226.004	176.356	242.302 Up
Plcd4	314.836	1.49694	0.23053	6.4936	8.38E-11	2.24E-09	144.158	159.071	190.977	351.447	477.323	566.041 Up
Rnf25	2013.64	-0.2286	0.1039	-2.2002	0.02779	0.08663	2287.17	2011.38	2220.1	1920	1753	1890.15 Down
Stk36	1501.86	-0.416	0.15257	-2.7267	0.0064	0.02654	1617.66	1699.95	1833.38	1277.23	1070.81	1512.12 Down
Ttll4	2764.03	-0.3069	0.14425	-2.1278	0.03336	0.09936	2948.94	3020.43	3201.72	2548.25	2027.57	2837.24 Down
Cyp27a1	1901.09	-0.6386	0.09273	-6.8871	5.69E-12	1.83E-10	2232.03	2434.94	2278.35	1525.01	1477.38	1458.84 Down
Ihh	86.1942	-1.11	0.29888	-3.7138	0.0002	0.00142	133.515	109.241	110.766	63.2397	39.0729	61.3295 Down
Slc23a3	117.236	1.04948	0.44392	2.3641	0.01807	0.06165	127.71	60.3703	41.06	153.434	212.261	108.583 Up
Cnppd1	4409.17	0.21488	0.10843	1.98182	0.0475	0.13114	4199.92	3841.66	4202.44	4937.88	5019.29	4253.85 Up
Atg9a	750.443	0.2793	0.13488	2.07075	0.03838	0.11098	735.301	585.496	713.298	826.263	827.924	814.375 Up
Dnajb2	5272.31	-0.2262	0.11493	-1.9683	0.04903	0.13455	5273.85	5511.9	6268.81	5044.66	4434.25	5100.4 Down
Ptprn	472.262	1.01646	0.26552	3.82822	0.00013	0.00095	346.366	285.561	305.563	503.844	477.323	914.915 Up
Resp18	34.162	-3.3282	0.53993	-6.1642	7.08E-10	1.60E-08	75.4651	59.412	51.5637	9.33044	3.16808	6.03241 Down
Dnpep	3141.21	0.28069	0.12806	2.19191	0.02839	0.08814	3178.24	2804.82	2526.62	3630.58	3585.21	3121.77 Up
Des	7121.43	-1.4643	0.25954	-5.642	1.68E-08	2.91E-07	7630.68	14369.1	9362.63	3537.27	4988.66	2840.26 Down
Speg	519.248	-1.1198	0.25383	-4.4119	1.02E-05	0.0001	646.291	952.509	534.734	341.08	386.505	254.366 Down
Obsl1	711.296	-1.016	0.15123	-6.7183	1.84E-11	5.55E-10	916.224	1110.62	828.838	472.742	467.819	471.533 Down

Inha	355.462	-0.7732	0.25068	-3.0845	0.00204	0.01012	467.303	518.418	359.991	260.216	189.029	337.815	Down
Stk11ip	2032.9	-0.2992	0.0977	-3.0627	0.00219	0.01076	2194.29	2336.23	2198.14	1885.79	1880.78	1702.14	Down
Slc4a3	1229.6	-1.1142	0.19938	-5.5882	2.29E-08	3.86E-07	1363.21	2205.91	1477.2	716.371	843.764	771.142	Down
Sgpp2	808.527	1.94761	0.29514	6.59886	4.14E-11	1.17E-09	525.353	223.274	250.179	1162.16	1336.93	1353.27	Up
Farsb	3326.75	0.41723	0.08591	4.85668	1.19E-06	1.44E-05	2718.68	2881.48	2946.77	3779.87	3915.74	3717.97	Up
BC035947	63.4095	1.72399	0.3366	5.12173	3.03E-07	4.16E-06	38.7001	23.9565	25.7818	108.855	87.6501	95.5131	Up
Mogat1	245.044	2.59846	0.44041	5.90008	3.63E-09	7.25E-08	57.0826	119.782	31.5111	493.477	257.67	510.744	Up
5730419F	8.74466	-2.6517	1.13624	-2.3337	0.01961	0.06578	29.025	5.74955	10.5037	0	3.16808	4.0216	Down
Scg2	16.7292	-3.4962	0.94426	-3.7026	0.00021	0.00147	33.8626	45.0382	13.3684	2.07343	0	6.03241	Down
Ap1s3	808.915	0.77025	0.15638	4.92552	8.41E-07	1.06E-05	552.443	607.536	634.042	1045.01	852.212	1162.24	Up
Mrpl44	1192.85	-0.3596	0.10832	-3.3197	0.0009	0.00511	1304.19	1415.35	1302.46	1057.45	1100.38	977.25	Down
Fam124b	83.7782	-1.39	0.56411	-2.4641	0.01374	0.04954	34.8301	137.031	191.931	35.2483	64.4175	39.2106	Down
Dock10	3373.53	0.58619	0.17357	3.37729	0.00073	0.00428	3125.03	2426.31	2540.94	3324.75	4902.07	3922.07	Up
A030005L	59.8089	-0.7269	0.29665	-2.4502	0.01428	0.05108	75.4651	68.0364	80.2102	46.6522	42.241	46.2484	Down
A030014E	3.85859	-3.3431	1.58753	-2.1058	0.03522	0.10381	2.9025	3.83303	14.3232	1.03672	1.05603	0	Down
Slc19a3	332.895	-0.5769	0.17044	-3.3849	0.00071	0.00419	343.463	428.342	423.968	288.207	243.942	269.447	Down
Ccl20	97.3145	1.96582	0.52671	3.73227	0.00019	0.00134	33.8626	49.8294	35.3307	236.371	47.5211	180.972	Up
Daw1	1046.53	-0.819	0.26484	-3.0923	0.00199	0.00992	1154.23	1431.64	1421.82	943.411	439.307	888.774	Down
Sphkap	40.8462	-2.5859	0.6274	-4.1217	3.76E-05	0.00032	41.6026	129.365	39.1502	7.25701	11.6163	16.0864	Down
Pid1	2031.85	-0.7375	0.12591	-5.8573	4.70E-09	9.18E-08	2540.66	2598.8	2480.79	1701.25	1556.58	1313.05	Down
Dner	42.1837	-1.4164	0.5352	-2.6465	0.00813	0.03226	37.7326	91.0346	55.3832	34.2116	11.6163	23.1242	Down
Trip12	9935.74	0.23953	0.07472	3.20584	0.00135	0.00716	9323.81	8930.97	9083.8	10487.4	10746.1	11042.3	Up
AC147806	58.2809	1.09652	0.3204	3.42234	0.00062	0.00373	44.5051	29.706	37.2404	80.8638	73.9218	83.4483	Up
Sp100	5185.98	0.23426	0.08658	2.7058	0.00681	0.02793	4984.57	4686.84	4626.41	5502.89	5440.64	5874.56	Up
A630001G	993.163	0.47263	0.13911	3.39765	0.00068	0.00403	910.419	882.556	702.794	1143.5	1200.7	1119.01	Up
Itm2c	12100.2	0.23788	0.09631	2.46997	0.01351	0.04888	11463	10823.5	11027.9	13959.4	13364	11963.3	Up
2810459M	135.354	1.73238	0.47935	3.61406	0.0003	0.00199	121.905	34.4973	31.5111	212.527	219.653	192.032	Up
Psmd1	4963.68	0.30515	0.07512	4.06209	4.86E-05	0.0004	4387.62	4464.53	4469.81	5385.74	5619.11	5455.31	Up
Gm16341	63.0226	-0.7776	0.3666	-2.1212	0.0339	0.10067	56.1151	91.0346	91.6688	60.1295	36.9609	42.2268	Down
B3gnt7	1645.39	2.77871	0.22615	12.2871	1.06E-34	3.23E-32	562.118	367.013	326.57	2999.22	3319.09	2298.35	Up
Efhd1	329.23	1.21024	0.32737	3.69681	0.00022	0.0015	98.6851	240.523	256.863	393.952	483.66	501.695	Up
Ngef	819.064	-1.0705	0.18555	-5.7697	7.94E-09	1.47E-07	1383.53	984.132	961.567	595.075	498.444	491.641	Down
Sag	88.6758	-0.9605	0.2612	-3.6774	0.00024	0.0016	119.003	121.699	110.766	49.7624	65.4736	65.3511	Down
Dgkd	5604.32	0.51183	0.11114	4.60547	4.12E-06	4.44E-05	5113.25	4463.57	4284.56	6666.08	6944.42	6154.06	Up
Spp2	364.488	-0.6954	0.30592	-2.273	0.02302	0.07461	387.968	477.213	486.99	298.574	153.124	383.058	Down
Gm29538	290.865	-1.266	0.2392	-5.2925	1.21E-07	1.76E-06	323.145	437.924	471.712	183.499	128.835	200.075	Down
Arl4c	4453.23	0.3224	0.13883	2.32223	0.02022	0.06732	3938.7	3366.36	4568.16	4798.96	4702.48	5344.71	Up
Asb18	24.8184	-5.1414	1.17322	-4.3823	1.17E-05	0.00011	19.35	113.075	12.4135	0	1.05603	3.0162	Down
Iqca	2006.69	-0.8748	0.27029	-3.2367	0.00121	0.00654	2211.71	2863.28	2716.64	1428.59	834.26	1985.67	Down
Ackr3	2866.65	-0.3903	0.14269	-2.7349	0.00624	0.026	3275.96	3617.43	2862.74	2820.9	2202.87	2420	Down
Cops8	3898.6	-0.2163	0.09686	-2.2327	0.02557	0.08106	4036.42	4154.05	4380.05	3536.24	3874.56	3410.32	Down
Mlph	3724.72	2.06718	0.1563	13.2257	6.23E-40	2.99E-37	1280.97	1712.41	1312.01	5693.64	6856.77	5492.5	Up
Prlh	16.188	1.38601	0.5765	2.40417	0.01621	0.05651	7.74001	9.58259	9.54883	29.028	21.1205	20.108	Up
Rab17	289.75	-0.8593	0.26867	-3.1982	0.00138	0.00733	315.405	358.389	446.885	286.134	165.796	165.891	Down
Lrrfip1	9754.86	0.61847	0.10605	5.83194	5.48E-09	1.05E-07	7562.96	7447.59	8075.44	11157.1	13120.1	11166	Up
Ramp1	1021.36	0.42263	0.17326	2.43926	0.01472	0.05226	786.579	977.424	854.62	1178.75	949.367	1381.42	Up
Ube2f	2910.24	0.21665	0.07821	2.76994	0.00561	0.02385	2707.07	2727.2	2642.16	3131.92	3114.22	3138.86	Up
Espnl	77.9129	-1.0264	0.39145	-2.6219	0.00874	0.03419	79.3351	105.408	128.909	52.8725	29.5687	71.3835	Down
Klhl30	121.615	-1.6548	0.51655	-3.2036	0.00136	0.00721	119.97	355.514	78.3004	52.8725	70.7537	52.2808	Down
Hes6	2786.39	0.8048	0.18423	4.36855	1.25E-05	0.00012	2170.11	2226.03	1690.14	4433	3036.07	3162.99	Up
Gm28382	47.4909	1.0456	0.52988	1.97328	0.04846	0.13324	15.48	51.746	25.7818	66.3498	40.129	85.4591	Up
Twist2	38.4381	-1.4175	0.54576	-2.5973	0.0094	0.0363	30.96	91.0346	45.8344	13.4773	22.1765	27.1458	Down
Ndufa10	4266.7	-0.3553	0.17566	-2.0226	0.04312	0.12153	4284.1	6138.6	3945.58	3956.11	3580.98	3694.85	Down
Gpc1	1171.33	-1.4885	0.26394	-5.6395	1.71E-08	2.94E-07	1538.33	2469.43	1173.55	643.801	701.201	501.695	Down
Ankmy1	227.701	-0.7832	0.2901	-2.6998	0.00694	0.02834	289.283	318.142	256.863	179.352	101.378	221.188	Down
Dusp28	563.218	-0.2993	0.15185	-1.9713	0.04869	0.13369	655.966	637.242	571.02	573.304	497.388	444.387	Down
Gpr35	362.303	0.656	0.184	3.56513	0.00036	0.00235	275.738	275.978	292.194	360.777	522.733	446.398	Up
Pask	81.9398	0.85868	0.31226	2.74992	0.00596	0.02503	66.7576	45.0382	63.0223	99.5247	86.5941	130.702	Up
Hdlbp	17744.8	0.28901	0.07044	4.10293	4.08E-05	0.00034	15747.1	16324.9	15848.2	19526.5	19201.7	19820.5	Up
Bok	1511.14	-0.6357	0.12497	-5.0866	3.64E-07	4.94E-06	1769.56	1894.48	1852.47	1279.31	1024.34	1246.7	Down
Dtymk	1682.84	-0.2786	0.09818	-2.8374	0.00455	0.02003	1875.02	1744.03	1915.5	1557.15	1450.98	1554.35	Down
Pdcd1	48.291	1.4432	0.5622	2.56706	0.01026	0.03904	40.6351	23.9565	13.3684	47.6889	46.4651	117.632	Up
Gm15427	2415.71	-0.4043	0.11672	-3.4644	0.00053	0.00326	2984.74	2505.85	2765.34	2135.63	2194.42	1908.25	Down
St8sia4	1462.79	0.50707	0.21503	2.3581	0.01837	0.06247	1559.61	1067.5	997.853	1563.37	2121.55	1466.88	Up
Slco4c1	5321.93	1.5082	0.10391	14.5144	9.82E-48	7.85E-45	2675.14	2870.94	2759.61	7099.43	8172.58	8353.88	Up
Panct2	913.912	0.90366	0.14493	6.2352	4.51E-10	1.06E-08	591.143	644.908	674.147	1107.21	1086.65	1379.41	Up
Pam	20794.1	-1.1514	0.2539	-4.5348	5.77E-06	6.00E-05	21441.8	43225.1	21366.5	12472.7	12214	14044.4	Down
Cntnap5b	19.399	-1.4624	0.68208	-2.1441	0.03203	0.09635	19.35	45.0382	21.0074	17.6242	6.33615	7.03781	Down
Cdh20	14.8099	-1.6294	0.72054	-2.2613	0.02374	0.07644	12.5775	38.3303	16.233	8.29373	7.39218	6.03241	Down
Rnf152	166.934	-0.7701	0.23784	-3.2379	0.0012	0.00651	167.378	258.73	205.3	125.443	114.051	130.702	Down
Pign	166.084	0.77958	0.1893	4.11834	3.82E-05	0.00032	125.775	117.866	123.18	193.866	219.653	216.161	Up
Relch	3106.74	0.39365	0.10514	3.74404	0.00018	0.00128	2699.33	2502.97	2854.14	3293.65	3758.39	3531.97	Up
Tnfrsf11a	644.82	1.76921	0.15292	11.5698	5.86E-31	1.25E-28	274.77	346.89	255.909	1003.54	988.44	999.368	Up
Zcchc2	1812	0.26897	0.1057	2.54459	0.01094	0.04117	1713.45	1726.78	1490.57	2023.67	1939.92	1977.62	Up
Gm20753	112.939	-0.6078	0.25362	-2.3964	0.01656	0.05746	151.898	107.325	149.917	83.974	95.0423	89.4807	Down
Phlpp1	3139.81	-0.3287	0.08027	-4.0948	4.23E-05	0.00035	3407.54	3507.23	3573.17	2756.63	2753.06	2841.26	Down
Bcl2	1505.55	-0.3862	0.14372	-2.6869	0.00721	0.02922	1423.19	1836.02	1858.2	1215.03	1390.79	1310.04	Down
Vps4b	6405.92	0.20166	0.08555	2.35722	0.01841	0.0626	6008.18	5759.13	6109.34	6630.83	7240.11	6687.93	Up
Serpinb5	85.4586	6.23388	0.73852	8.441	3.15E-17	2.16E-15	2.9025	3.83303	0	133.736	89.7622	282.518	Up
Serpinb12	68.4191	3.89251	0.68346	5.69531	1.23E-08	2.19E-07	3.87001	18.2069	3.81953	140.993	48.5772	195.048	Up
Serpinb3b	4.75116	2.30502	1.13311	2.03425	0.04193	0.11898	0.9675	2.87478	0.95488	9.33044	6.33615	8.04321	Up
Serpinb11	18.8679	4.22275	0.79941	5.28233	1.28E-07	1.86E-06	0	2.87478	2.86465	34.2116	39.0729	34.1836	Up
Cdh19	36.6995	-1.9798	0.58476	-3.3856	0.00071	0.00418	39.6676	102.534	33.4209	17.6242	16.8964	10.054	Down

Tsn	3900.32	0.25879	0.08945	2.89299	0.00382	0.01727	3377.55	3629.88	3646.7	4187.3	4457.48	4103.04	Up
Clasp1	2726.38	-0.388	0.14669	-2.6453	0.00816	0.03235	2704.17	3677.8	2890.43	2229.98	2555.58	2300.36	Down
Tfcp2l1	2484.72	0.47732	0.14277	3.3432	0.00083	0.00475	1764.72	2043.97	2423.49	2801.21	2776.29	3098.65	Up
Gm29455	40.4036	-1.6912	0.55463	-3.0492	0.00229	0.01118	46.4401	85.285	53.4734	10.3672	12.6723	34.1836	Down
Inhbb	2111.34	0.64948	0.10253	6.3346	2.38E-10	5.93E-09	1711.51	1540.88	1679.64	2437.32	2564.03	2734.69	Up
Ralb	3941.34	0.415	0.12628	3.28625	0.00102	0.00565	3728.75	2944.73	3461.45	4457.88	4856.66	4198.55	Up
Gm27184	71.7929	-0.7741	0.28468	-2.7192	0.00654	0.02699	100.62	81.452	89.759	60.1295	47.5211	51.2754	Down
Epb41l5	6916.08	0.73128	0.11451	6.38595	1.70E-10	4.38E-09	5819.52	4637.97	5142.04	8557.05	8992.06	8347.84	Up
Tmem177	472.236	-0.4139	0.1344	-3.0795	0.00207	0.01026	499.231	553.873	565.291	409.503	423.466	382.052	Down
Cfap221	670.737	-0.8258	0.22199	-3.7199	0.0002	0.00139	832.051	909.387	831.703	471.706	342.152	637.424	Down
Sctr	6.72758	-5.1474	1.52326	-3.3792	0.00073	0.00426	7.74001	22.0399	9.54883	1.03672	0	0	Down
Tmem37	1843.79	0.67298	0.19875	3.38607	0.00071	0.00418	1708.61	1409.6	1145.86	2509.89	2532.35	1756.44	Up
Dbi	4781.28	0.53384	0.14302	3.73259	0.00019	0.00133	3482.04	4588.14	3649.56	5513.25	6252.73	5201.94	Up
3110009E	343.114	-0.387	0.14091	-2.7465	0.00602	0.02525	384.098	383.303	399.141	316.198	290.407	285.534	Down
C1ql2	3.72102	-3.2766	1.65609	-1.9785	0.04787	0.13199	11.61	2.87478	5.7293	0	2.11205	0	Down
Marco	4048.22	2.89507	0.88101	3.28607	0.00102	0.00565	2055.94	605.619	216.758	4554.29	8266.57	8590.15	Up
Actr3	17868.9	0.61943	0.09947	6.22729	4.75E-10	1.10E-08	13881.7	14250.3	14139.9	20742.6	23936.9	20261.8	Up
Slc35f5	3931.67	0.27077	0.1086	2.49322	0.01266	0.04632	3353.36	3775.54	3562.67	4118.87	4056.19	4723.37	Up
Gpr39	391.83	1.10966	0.1636	6.7827	1.18E-11	3.66E-10	280.575	218.483	245.405	500.734	583.982	521.803	Up
Lypd1	55.768	1.76739	0.44187	3.99975	6.34E-05	0.00051	40.6351	13.4156	21.9623	80.8638	79.2019	98.5293	Up
Mgat5	1438.78	0.61284	0.14818	4.13586	3.54E-05	0.0003	1179.38	1210.28	1023.63	1555.07	1627.34	2036.94	Up
Tmem163	2027.82	1.02351	0.23508	4.35378	1.34E-05	0.00013	1763.76	1175.78	1072.33	2533.73	2141.62	3479.69	Up
Zranb3	407.537	1.44626	0.22109	6.54142	6.09E-11	1.67E-09	266.063	216.566	173.789	552.57	725.489	510.744	Up
Ubxn4	7278.46	0.22813	0.07784	2.93087	0.00338	0.0156	6553.85	6620.61	6938.18	7696.58	7788.19	8073.37	Up
Mcm6	603.268	0.84004	0.19468	4.31492	1.60E-05	0.00015	544.703	348.806	403.915	771.317	703.313	847.553	Up
Cd55b	127.817	-1.2147	0.24928	-4.8727	1.10E-06	1.34E-05	206.078	154.28	175.698	86.0474	63.3615	81.4375	Down
Gm16083	91.2374	-0.6748	0.33489	-2.0151	0.04389	0.12326	87.0751	103.492	146.097	73.6068	50.6892	86.4645	Down
C4bp	67.0433	3.02062	0.64446	4.68708	2.77E-06	3.11E-05	8.70751	27.7895	7.63906	140.993	41.185	175.945	Up
Gm29427	60.2742	1.18803	0.3822	3.10837	0.00188	0.00948	25.155	49.8294	35.3307	98.488	63.3615	89.4807	Up
AA98686C	7904.11	0.57372	0.12623	4.54518	5.49E-06	5.75E-05	6832.5	5672.89	6553.36	10146.3	8319.37	9900.18	Up
Gm28856	83.0714	1.39688	0.31549	4.42764	9.53E-06	9.38E-05	34.8301	56.5373	45.8344	109.892	104.547	146.789	Up
Gm15848	885.448	1.91327	0.30137	6.34846	2.17E-10	5.44E-09	417.961	434.091	262.593	1601.73	804.691	1791.62	Up
Pigr	32476.3	3.15373	0.29768	10.5943	3.17E-26	4.75E-24	4642.07	8719.2	6322.28	64815.5	33308.1	77050.9	Up
Ctse	842.984	0.53247	0.14773	3.60424	0.00031	0.00206	781.741	696.654	589.163	1074.04	944.087	972.223	Up
Rab7b	429.042	1.00071	0.25535	3.91896	8.89E-05	0.00069	402.481	229.982	225.352	577.451	647.344	491.641	Up
Slc26a9	5854.55	1.19511	0.20046	5.96172	2.50E-09	5.11E-08	3513	3143.09	4021.97	7798.18	10541.2	6109.82	Up
Pm20d1	520.184	0.53942	0.17973	3.00134	0.00269	0.0128	416.026	474.338	381.953	565.01	542.797	740.98	Up
Slc41a1	3023.54	0.30059	0.13143	2.28712	0.02219	0.07239	2995.38	2823.03	2310.82	3193.08	3235.66	3583.25	Up
Elk4	57.9415	1.11704	0.43825	2.54887	0.01081	0.04079	57.0826	33.5391	19.0977	83.974	86.5941	67.3619	Up
Mfsd4a	1892.09	3.08916	0.21281	14.5159	9.61E-48	7.85E-45	482.783	445.59	265.457	3733.21	3167.02	3258.5	Up
Cdk18	1851.56	0.44083	0.12771	3.45196	0.00056	0.00339	1660.23	1457.51	1594.65	2266.26	2275.73	1854.96	Up
Lemd1	71.3633	3.11944	0.70522	4.42338	9.72E-06	9.55E-05	15.48	21.0817	7.63906	161.728	22.1765	200.075	Up
Klhdc8a	798.991	0.57965	0.21995	2.63542	0.0084	0.03309	468.271	858.6	594.892	1018.05	947.255	906.872	Up
Nuak2	1098.32	0.60548	0.12453	4.86215	1.16E-06	1.41E-05	836.889	910.346	866.079	1389.2	1416.13	1171.29	Up
Tmcc2	8983.38	-0.8951	0.14558	-6.1486	7.82E-10	1.75E-08	10047.5	11413.8	13590.8	6027.47	7042.63	5778.04	Down
Lrrn2	175.271	-1.5378	0.34735	-4.4272	9.55E-06	9.39E-05	212.85	374.679	194.796	102.635	58.0814	108.583	Down
Pik3c2b	1266.12	0.50849	0.22855	2.22481	0.02609	0.08238	781.741	1198.78	1155.41	1271.01	1251.39	1938.41	Up
Ppp1r15b	6946.14	0.30218	0.07716	3.91618	9.00E-05	0.00069	6281.02	5996.78	6386.26	7524.48	7699.48	7788.84	Up
Plekha6	4296.87	0.40483	0.1377	2.93984	0.00328	0.0152	3461.72	4290.12	3342.09	4671.44	4593.71	5422.13	Up
Sox13	5160.67	-0.4117	0.13728	-2.999	0.00271	0.01288	5086.16	5894.25	6695.64	4265.05	4896.79	4126.17	Down
Optc	20.953	-1.9419	0.68157	-2.8492	0.00438	0.0194	19.35	39.2886	41.06	4.14686	15.8404	6.03241	Down
Gm15851	72.8607	-1.6036	0.34951	-4.5881	4.47E-06	4.78E-05	74.4976	130.323	124.135	32.1382	34.8488	41.2214	Down
Chil1	102484	2.98294	0.21133	14.1151	3.07E-45	2.07E-42	31890.8	19761.2	17392.2	192966	174811	178082	Up
Mybph	13.4589	1.68396	0.72283	2.32967	0.01982	0.06634	3.87001	6.70781	8.59395	24.8812	10.5603	26.1404	Up
Ppfia4	517.519	0.6938	0.15107	4.59274	4.37E-06	4.69E-05	419.896	432.175	334.209	651.058	649.456	618.322	Up
Tmem183	4327.7	0.25868	0.09432	2.74261	0.0061	0.02549	3867.1	3747.75	4207.21	4644.49	4941.14	4558.49	Up
Cyb5r1	3078.3	0.39278	0.13657	2.876	0.00403	0.01808	2891.86	2464.64	2628.79	3446.04	3998.11	3040.33	Up
Gm15454	22.2204	-1.2117	0.53058	-2.2837	0.02239	0.07286	29.9925	33.5391	29.6014	7.25701	15.8404	17.0918	Down
Syt2	92.2809	1.353	0.64769	2.08896	0.03671	0.10724	67.7251	46.9547	41.06	108.855	262.95	26.1404	Up
Lgr6	501.47	-2.8219	0.63128	-4.4702	7.82E-06	7.89E-05	545.671	1651.08	439.246	103.672	161.572	107.578	Down
Ptpn7	369.764	0.71673	0.14881	4.81628	1.46E-06	1.74E-05	297.023	255.855	286.465	430.237	459.371	489.63	Up
Gm4204	1985.58	-0.4057	0.10281	-3.9461	7.94E-05	0.00062	2361.67	2192.5	2234.43	1690.88	1836.43	1597.58	Down
Rnpep	3222.84	0.80486	0.11927	6.74794	1.50E-11	4.57E-10	2486.48	2420.56	2132.25	4180.04	4427.91	3689.82	Up
Lmod1	398.436	-1.37	0.22783	-6.0131	1.82E-09	3.80E-08	465.368	697.612	560.516	222.894	269.286	174.94	Down
Tnnt2	5902.36	-5.6321	0.70513	-7.9873	1.38E-15	7.51E-14	8901.98	18944.8	6867.52	244.665	79.2019	376.02	Down
Kif21b	1262.2	0.46199	0.15191	3.04131	0.00236	0.01144	1065.22	1157.58	962.522	1283.45	1713.93	1390.47	Up
Mroh3	9.3706	2.10997	0.87496	2.4115	0.01589	0.05559	3.87001	4.79129	1.90977	8.29373	24.2886	13.0702	Up
Inava	259.768	0.80732	0.2795	2.88844	0.00387	0.01748	116.1	245.314	205.3	302.721	366.441	322.734	Up
Camsap2	338.858	0.61707	0.25877	2.38459	0.0171	0.05899	267.03	220.399	315.111	339.006	331.592	560.008	Up
9230116N	59.9608	-0.8178	0.30233	-2.705	0.00683	0.02798	78.3676	75.7024	75.4357	49.7624	43.297	37.1998	Down
Ptprc	6430.82	0.61312	0.13956	4.39318	1.12E-05	0.00011	5750.83	5079.73	4423.02	7011.31	8711.15	7608.87	Up
Atp6v1g3	35.8647	1.83593	0.46139	3.97911	6.92E-05	0.00055	17.415	13.4156	16.233	49.7624	78.1459	40.216	Up
Dennd1b	1618.29	0.37262	0.1182	3.15256	0.00162	0.00835	1345.79	1573.46	1312.01	1766.56	1928.3	1783.58	Up
Aspm	99.145	0.70866	0.28479	2.48838	0.01283	0.04687	92.8801	77.619	55.3832	125.443	109.827	133.718	Up
4930590L	3.53495	-4.1828	1.82398	-2.2932	0.02183	0.07146	3.87001	14.3739	1.90977	0	1.05603	0	Down
Ro60	5019.16	0.25202	0.09462	2.66358	0.00773	0.03093	4645.94	4473.15	4626.41	5129.67	5902.13	5337.67	Up
Rgs1	648.908	1.71651	0.37295	4.60248	4.17E-06	4.50E-05	443.116	330.599	134.638	1309.37	958.871	716.851	Up
Rgs18	308.136	-0.5771	0.17595	-3.28	0.00104	0.00575	337.658	428.342	340.893	228.077	253.446	260.399	Down
Pla2g4a	2120.95	0.51045	0.1407	3.62797	0.00029	0.0019	1910.82	1859.98	1478.16	2567.95	2252.5	2656.27	Up
Ptgs2	2232.2	0.54808	0.20055	2.73285	0.00628	0.02613	1823.74	1783.32	1832.42	2427.99	3525.01	2000.75	Up
Ptgs2os2	636.532	0.48833	0.24896	1.96145	0.04983	0.13646	673.381	356.472	559.561	695.636	899.734	634.408	Up
Rbm3-ps	227.749	0.41898	0.17806	2.35305	0.01862	0.06316	193.5	209.859	181.428	235.334	260.838	285.534	Up
Hmcn1	4942.35	-0.6976	0.12021	-5.8027	6.53E-09	1.23E-07	5578.61	5925.87	6838.87	3473	3809.08	4028.64	Down

Fam129a	4176.22	0.36066	0.10812	3.33586	0.00085	0.00485	3622.33	3902.99	3445.22	4688.03	5080.54	4318.2 Up
Tsen15	945.03	-0.3582	0.11336	-3.1596	0.00158	0.00818	1013.94	1037.79	1133.45	878.098	802.579	804.321 Down
Colgalt2	1228.79	-0.5666	0.1679	-3.3745	0.00074	0.00432	1344.83	1453.68	1602.29	976.586	1188.03	807.337 Down
Arpc5	15430.3	0.51447	0.11781	4.36701	1.26E-05	0.00012	13549.9	12386.5	12187.2	18241	20195.4	16022.1 Up
Ncf2	2110.22	1.18207	0.20652	5.72366	1.04E-08	1.87E-07	1641.85	1272.57	958.702	2898.66	3374	2515.51 Up
Lamc2	11561.7	1.34008	0.15863	8.44761	2.97E-17	2.06E-15	7856.11	5222.51	6563.86	15882.5	17840.5	16005 Up
Shcbp1l	49.1493	0.90404	0.33891	2.66748	0.00764	0.03064	29.025	35.4556	38.1953	57.0194	72.8658	62.3349 Up
Dhx9	4904.44	0.3538	0.1279	2.76635	0.00567	0.02407	4461.15	3719	4738.13	5164.92	5379.39	5964.04 Up
Npl	395.386	-0.3891	0.17037	-2.2837	0.02239	0.07286	444.083	493.503	407.735	350.41	292.519	384.063 Down
Rgs16	13.9593	1.56754	0.66862	2.34445	0.01906	0.06426	9.67501	5.74955	5.7293	18.6609	14.7844	29.1566 Up
Rnasel	1706.65	0.75694	0.12902	5.86682	4.44E-09	8.75E-08	1275.17	1394.27	1137.27	2063.06	2363.38	2006.78 Up
Rgsl1	43.5261	-1.5327	0.37385	-4.0999	4.13E-05	0.00035	75.4651	64.2033	54.4283	19.6976	23.2326	24.1296 Down
Teddm2	48.5927	-1.2793	0.59441	-2.1521	0.03139	0.09491	74.4976	79.5355	52.5186	41.4686	6.33615	37.1998 Down
Glul	21397.5	-0.6594	0.11303	-5.8336	5.42E-09	1.04E-07	25285.7	27683.1	25643.4	18402.7	14771.7	16598.2 Down
Gm28286	88.7535	1.88168	0.53874	3.49275	0.00048	0.00299	70.6276	24.9147	18.1428	87.0841	209.093	122.659 Up
Mr1	2979.95	0.2619	0.12319	2.12606	0.0335	0.09973	2725.45	2921.73	2483.65	3370.36	2878.73	3499.8 Up
Stx6	3447.06	0.28528	0.08036	3.55021	0.00038	0.00247	3151.15	3078.89	3091.91	3861.77	3846.04	3652.62 Up
BC034090	569.761	-0.7627	0.18029	-4.2304	2.33E-05	0.00021	630.811	707.195	812.605	448.898	475.211	343.847 Down
Xpr1	2755.45	0.28897	0.09	3.21064	0.00132	0.00707	2538.72	2458.89	2443.55	2877.92	3192.36	3021.23 Up
Gm5532	139.42	-2.809	0.53108	-5.2893	1.23E-07	1.79E-06	137.385	472.422	122.225	48.7256	30.6247	25.135 Down
Cep350	624.751	0.43774	0.2165	2.02189	0.04319	0.12169	440.213	649.699	502.268	733.995	559.693	862.634 Up
Tor1aip2	8279.95	0.41221	0.09605	4.29155	1.77E-05	0.00016	7772.91	6833.34	6709.01	9280.68	9526.4	9557.34 Up
Tdrd5	897.479	4.44062	0.34867	12.736	3.73E-37	1.44E-34	69.6601	75.7024	91.6688	1535.38	2727.71	884.753 Up
Nphs2	39.99	5.61323	0.75883	7.39724	1.39E-13	5.79E-12	1.935	1.91652	0.95488	69.46	100.322	65.3511 Up
Axdnd1	478.963	3.65756	0.35035	10.4396	1.63E-25	2.32E-23	40.6351	98.7006	71.6162	770.28	1287.29	605.251 Up
Soat1	16844.7	1.55581	0.13536	11.4935	1.42E-30	2.92E-28	9724.36	7637.32	8290.29	25107.2	27916	22393.3 Up
Abl2	2173.45	0.25013	0.10241	2.44251	0.01459	0.05189	2031.75	1895.44	2029.13	2338.83	2538.68	2206.85 Up
Fam20b	4762.6	0.44173	0.07865	5.61625	1.95E-08	3.33E-07	4072.21	4121.47	3923.61	5510.14	5606.44	5341.69 Up
Ralgps2	1528.29	-0.3123	0.09259	-3.3728	0.00074	0.00434	1742.47	1709.53	1627.12	1396.46	1356.99	1337.18 Down
Gm28694	176.418	-0.717	0.33343	-2.1504	0.03152	0.09521	187.695	216.566	253.999	155.507	71.8097	172.929 Down
Rasal2	2273.83	-0.347	0.10884	-3.1887	0.00143	0.00753	2561.94	2405.23	2670.81	1819.44	2119.44	2066.1 Down
2810025M	1632.28	-0.5657	0.1652	-3.4244	0.00062	0.0037	2006.6	1701.87	2136.07	1406.82	1481.6	1060.7 Down
Sec16b	389.797	-0.6734	0.168	-4.0083	6.12E-05	0.00049	398.611	505.961	532.825	297.537	306.247	297.599 Down
Pappa2	203.385	1.51713	0.26982	5.62278	1.88E-08	3.21E-07	116.1	72.8277	126.999	266.436	367.497	270.453 Up
Tnr	93.3156	-1.5978	0.3653	-4.374	1.22E-05	0.00012	84.1726	172.487	164.24	39.3952	43.297	56.3024 Down
4930523C	1214.81	-0.3646	0.11432	-3.1892	0.00143	0.00752	1447.38	1231.36	1423.73	1039.83	1090.87	1055.67 Down
Tnn	760.738	-1.4852	0.13512	-10.992	4.17E-28	7.44E-26	1183.25	1065.58	1114.35	449.935	362.217	389.09 Down
Rabgap1l	11840.9	0.32426	0.10406	3.11616	0.00183	0.00926	9902.38	11082.3	10562.9	12664.5	12397.7	14435.5 Up
Gas5	2949.76	-0.3329	0.13531	-2.4602	0.01389	0.04997	3127.93	2961.98	3775.61	2629.11	2831.2	2372.75 Down
Ankrd45	1129.98	-0.6222	0.27046	-2.3007	0.02141	0.07031	1298.39	1491.05	1320.6	1022.2	498.444	1149.17 Down
Prdx6	89339.4	-0.3464	0.15909	-2.1771	0.02947	0.09069	120165	84654.5	95218.1	89842.8	72129.7	74026.7 Down
Suco	2852.45	0.36774	0.10943	3.36046	0.00078	0.00452	2329.74	2407.15	2735.74	3062.46	3172.3	3407.3 Up
Vamp4	3175.57	0.50551	0.12356	4.09122	4.29E-05	0.00036	2912.18	2366.9	2595.37	3533.13	4084.71	3561.13 Up
Myocos	4.80646	4.75522	1.5547	3.05862	0.00222	0.01089	0.9675	0	0	7.25701	10.5603	10.054 Up
Fmo1	17381.4	-0.7355	0.15219	-4.8328	1.35E-06	1.61E-05	23883.7	16957.3	24314.2	13247.2	13228.8	12657 Down
Fmo2	52746.4	-0.7039	0.17145	-4.1057	4.03E-05	0.00034	81555.5	57445.7	57094.4	43475.7	34484.5	42422.9 Down
Fmo3	6410.36	-1.5405	0.56295	-2.7364	0.00621	0.02591	9097.42	10754.5	8770.6	3935.37	1185.92	4718.35 Down
Mroh9	42.086	-2.2059	0.8092	-2.726	0.00641	0.02658	83.2051	70.9111	53.4734	22.8077	0	22.1188 Down
Prrx1	927.292	-0.5428	0.19419	-2.795	0.00519	0.02237	1107.79	1248.61	942.469	679.049	950.423	635.413 Down
Kifap3	2859.46	-0.3564	0.08871	-4.0172	5.89E-05	0.00047	3185.98	3282.99	3163.53	2370.97	2613.66	2539.64 Down
Scyl3	205.433	0.47969	0.20023	2.39572	0.01659	0.05754	191.565	148.53	174.744	210.453	242.886	264.42 Up
F5	309.074	0.65031	0.28262	2.30098	0.02139	0.07029	191.565	341.14	189.067	363.887	307.303	461.479 Up
Atp1b1	37190.9	1.10089	0.10662	10.3254	5.41E-25	7.16E-23	22652.1	26395.2	21907.9	52631	50953.2	48606.1 Up
Gm26685	8.68441	2.75879	0.98011	2.81477	0.00488	0.02127	0	3.83303	2.86465	18.6609	12.6723	14.0756 Up
Gpr161	162.559	-0.6853	0.20702	-3.3102	0.00093	0.00526	217.688	209.859	173.789	133.736	125.667	114.616 Down
Mpzl1	2674.36	0.26427	0.12767	2.06988	0.03846	0.1112	2157.53	2454.1	2678.45	2955.68	3151.18	2649.23 Up
Rcsd1	4063.75	-0.4739	0.07861	-6.0277	1.66E-09	3.52E-08	4687.54	4653.3	4834.57	3443.97	3455.31	3307.77 Down
Creg1	9639.14	0.42426	0.20874	2.03251	0.0421	0.11935	11376.8	6867.84	6451.19	11263.9	11357.6	10517.5 Up
Dusp27	52.3397	-5.6436	0.84318	-6.6932	2.18E-11	6.50E-10	58.0501	189.735	60.1576	2.07343	0	4.0216 Down
Gpa33	15.7677	2.36889	0.73043	3.24313	0.00118	0.00641	3.87001	3.83303	7.63906	39.3952	13.7283	26.1404 Up
Ildr2	1190.19	2.90541	0.29158	9.96446	2.18E-23	2.52E-21	428.603	229.024	183.338	1773.82	2579.87	1946.46 Up
Fam78b	581.182	-0.5697	0.20493	-2.7801	0.00543	0.02323	544.703	642.992	895.68	436.457	483.66	483.598 Down
Tmco1	6118.34	-0.2788	0.07967	-3.4996	0.00047	0.00292	6784.12	6808.43	6530.44	5380.56	5753.23	5453.29 Down
Rxrg	117.693	-3.4202	0.57056	-5.9944	2.04E-09	4.24E-08	128.678	399.594	117.451	16.5875	33.7928	10.054 Down
Pbx1	6310.2	-0.2802	0.07779	-3.6022	0.00032	0.00208	6929.25	6706.85	7127.25	5681.2	5585.32	5831.33 Down
Nuf2	113.9	0.96368	0.34288	2.81053	0.00495	0.02153	98.6851	84.3268	48.699	168.985	108.771	173.934 Up
Rgs5	553.26	-1.7297	0.78419	-2.2057	0.0274	0.08562	400.546	1791.94	358.081	231.188	186.916	350.885 Down
Ccdc190	20.0897	-1.225	0.61566	-1.9897	0.04662	0.12919	18.3825	43.1216	22.9172	7.25701	14.7844	14.0756 Down
Hsd17b7	1307.59	1.16598	0.16056	7.2621	3.81E-13	1.47E-11	762.391	892.139	763.906	1678.44	2171.19	1577.47 Up
3110045C	44.8343	0.80493	0.34955	2.30275	0.02129	0.07003	32.895	35.4556	29.6014	62.203	47.5211	61.3295 Up
Ddr2	4960.79	-0.3337	0.12635	-2.641	0.00826	0.03269	5601.83	5551.19	5442.83	3750.84	4980.22	4437.84 Down
Uap1	4461.77	0.2817	0.08443	3.33647	0.00085	0.00485	3915.48	4023.73	4143.24	5032.22	4992.89	4663.05 Up
Uhmk1	8253.37	0.26849	0.08672	3.09621	0.00196	0.0098	7667.45	7168.73	7626.65	8508.33	9279.29	9269.8 Up
Sh2d1b1	638.185	2.13535	0.32919	6.48673	8.77E-11	2.34E-09	337.658	250.106	122.225	909.2	1343.26	866.656 Up
Gm7694	941.956	0.43673	0.13153	3.32025	0.0009	0.0051	714.016	846.142	841.252	995.247	1089.82	1165.26 Up
Olfml2b	696.718	-0.895	0.16531	-5.4143	6.15E-08	9.53E-07	800.124	1096.25	822.154	479.999	472.043	509.738 Down
Atf6	5148.62	0.25624	0.08407	3.04812	0.0023	0.01121	4912.97	4602.52	4562.43	5465.57	5542.02	5806.19 Up
Dusp12	77.4295	-0.6299	0.28033	-2.247	0.02464	0.07873	95.7826	83.3685	103.127	54.9459	55.9693	71.3835 Down
Fcrlb	39.5419	3.42542	0.56551	6.05719	1.39E-09	2.99E-08	12.5775	3.83303	3.81953	94.3411	63.3615	59.3187 Up
Fcgr2b	2568.95	1.9573	0.16253	12.0426	2.12E-33	5.72E-31	1126.17	1153.74	876.582	3682.41	3768.95	4805.82 Up
Gm10522	36.8812	2.68373	0.61386	4.37189	1.23E-05	0.00012	16.4475	5.74955	7.63906	65.3131	30.6247	95.5131 Up
Fcgr4	816.58	2.03878	0.32419	6.2889	3.20E-10	7.74E-09	445.051	336.349	177.608	1165.27	1788.91	986.298 Up
Fcgr3	3154.37	1.85753	0.22869	8.12257	4.56E-16	2.69E-14	1778.27	1446.01	868.943	4908.85	5197.76	4726.39 Up

Cfap126	2545.74	-0.436	0.21063	-2.0697	0.03848	0.11122	2612.25	3237.96	2932.45	2414.51	1476.32	2600.97 Down
Pcp4l1	8540.15	-0.7346	0.20878	-3.5184	0.00043	0.00274	8359.21	13247	10399.6	6783.23	4886.23	7565.64 Down
Fcer1g	2705.58	1.50582	0.17502	8.60378	7.71E-18	5.76E-16	1497.69	1587.83	1142.04	3777.79	4759.51	3468.63 Up
Ndufs2	8323.13	-0.3147	0.15441	-2.0381	0.04154	0.11807	8115.4	11392.7	8173.8	7698.65	7549.53	7008.65 Down
B4galt3	2140.29	0.41478	0.09939	4.1734	3.00E-05	0.00026	1726.99	1795.78	1981.38	2409.33	2427.8	2500.43 Up
Nit1	2382.93	0.213	0.10493	2.02987	0.04237	0.11987	2150.76	2322.82	2148.49	2774.25	2389.79	2511.49 Up
Nectin4	1085.74	0.35499	0.10042	3.53517	0.00041	0.00259	965.566	956.342	936.74	1195.33	1179.58	1280.88 Up
Arhgap30	2041.82	1.00475	0.15244	6.59113	4.36E-11	1.22E-09	1558.64	1321.44	1194.56	2510.93	3143.79	2521.55 Up
Tstd1	271.53	-0.7085	0.17337	-4.0865	4.38E-05	0.00036	297.023	359.347	354.262	224.967	198.533	195.048 Down
F11r	13912.9	0.30639	0.10001	3.06357	0.00219	0.01074	13446.3	11369.7	12507.1	15566.3	15869.9	14718.1 Up
Itln1	316.577	6.02117	1.87288	3.21492	0.0013	0.00698	5.80501	22.9982	0	481.036	24.2886	1365.33 Up
Cd244a	422.354	1.01646	0.17235	5.89777	3.68E-09	7.35E-08	267.03	334.432	236.811	555.68	588.206	551.965 Up
Slamf7	352.774	0.99665	0.16126	6.18054	6.39E-10	1.45E-08	244.778	213.692	248.27	470.669	423.466	515.771 Up
Cd48	1502.75	0.82228	0.15961	5.15169	2.58E-07	3.59E-06	1246.14	1077.08	933.875	1906.52	2184.92	1667.96 Up
Slamf1	88.8506	-0.8339	0.33038	-2.5242	0.0116	0.04318	83.2051	153.321	105.037	66.3498	52.8013	72.3889 Down
Cd84	2525.92	1.84241	0.20421	9.02193	1.85E-19	1.55E-17	1396.1	1024.38	884.222	3633.69	4823.92	3393.23 Up
Gm10521	37.2193	1.8294	0.49781	3.67489	0.00024	0.00161	20.3175	19.1652	9.54883	45.6155	83.426	45.243 Up
Ncstn	7158.4	0.54398	0.08873	6.13057	8.76E-10	1.95E-08	6154.28	5780.22	5539.28	8754.03	8593.93	8128.67 Up
Igsf8	2254.27	0.73027	0.20113	3.63088	0.00028	0.00188	2268.79	1410.56	1407.5	2741.08	3084.65	2613.04 Up
Kcnj10	82.2299	1.82865	0.318	5.75056	8.90E-09	1.63E-07	26.1225	41.2051	41.06	124.406	108.771	151.816 Up
Slamf9	618.809	1.43464	0.21965	6.53149	6.51E-11	1.78E-09	433.441	321.017	248.27	796.198	894.453	1019.48 Up
Igsf9	363.482	0.5339	0.15057	3.54591	0.00039	0.0025	292.185	310.476	288.375	393.952	421.354	474.549 Up
Tagln2	27453.6	0.40779	0.14296	2.8524	0.00434	0.01924	25556.6	20708	24532.9	31112.9	35921.8	26889.4 Up
Vsig8	23.8084	0.98319	0.48002	2.04824	0.04054	0.11572	13.545	20.1234	14.3232	36.2851	26.4006	32.1728 Up
Slamf8	158.918	0.72213	0.22215	3.25061	0.00115	0.00629	101.588	139.906	118.405	186.609	184.804	222.194 Up
Aim2	845.831	1.13691	0.22548	5.0422	4.60E-07	6.11E-06	593.078	585.496	407.735	1138.31	1462.6	887.769 Up
Ifi214	79.3395	-0.7045	0.33994	-2.0725	0.03822	0.11056	128.678	82.4102	84.0297	55.9827	47.5211	77.4159 Down
Ifi213	1219.58	-0.4411	0.13879	-3.178	0.00148	0.00777	1375.79	1365.52	1472.43	868.768	1132.06	1102.92 Down
Ifi209	993.283	-0.2567	0.12814	-2.0033	0.04514	0.12593	1124.24	1169.08	951.063	874.988	890.229	950.104 Down
Ifi207	830.582	1.07989	0.20214	5.34242	9.17E-08	1.37E-06	561.151	652.574	386.728	1233.69	1044.41	1104.94 Up
Ifi204	495.616	0.66852	0.28877	2.31504	0.02061	0.06831	424.733	508.835	214.849	685.269	563.918	576.095 Up
Ifi203-ps	390.979	0.55356	0.23192	2.3868	0.017	0.05872	416.026	274.062	260.683	438.531	407.626	548.949 Up
Ifi211	238.672	0.65326	0.28467	2.29483	0.02174	0.07122	147.06	257.772	151.826	332.786	223.877	318.712 Up
Spta1	69.1578	-2.0029	0.48246	-4.1515	3.30E-05	0.00028	76.4326	183.986	71.6162	21.771	38.0169	23.1242 Down
Fmn2	318.786	-1.0249	0.23784	-4.3092	1.64E-05	0.00015	565.988	380.429	336.119	229.114	186.916	214.15 Down
Rgs7	42.6179	-2.7986	0.66039	-4.2378	2.26E-05	0.0002	42.5701	138.948	42.0148	16.5875	10.5603	5.027 Down
Kmo	316.373	0.4757	0.23721	2.00542	0.04492	0.12549	355.073	220.399	218.668	332.786	360.105	411.209 Up
Opn3	171.054	1.09789	0.26297	4.17496	2.98E-05	0.00026	136.418	113.075	77.3455	266.436	224.933	208.118 Up
Wdr64	2.23099	4.65934	2.03473	2.2899	0.02203	NA	0	0	0	2.07343	5.28013	6.03241 Up
Adss	6852.34	0.29334	0.0773	3.7947	0.00015	0.00107	6212.33	6101.23	6160.9	7583.58	7259.12	7796.88 Up
Catspere2	1601.26	1.07891	0.09717	11.1038	1.20E-28	2.20E-26	1012.01	1068.46	1006.45	2111.79	2121.55	2287.29 Up
Desi2	1757.41	0.20078	0.10057	1.99635	0.0459	0.12757	1629.27	1742.11	1534.5	1932.44	1892.4	1813.74 Up
B230369F	67.6242	-1.1257	0.34895	-3.2261	0.00126	0.00676	84.1726	91.9928	102.172	48.7256	26.4006	52.2808 Down
Gm38300	134.591	-0.6792	0.23664	-2.8701	0.0041	0.01836	149.963	154.28	192.886	117.149	88.7061	104.562 Down
Efcab2	702.562	-1.0949	0.21827	-5.0164	5.27E-07	6.90E-06	774.001	1208.36	888.996	455.118	359.049	529.846 Down
Kif26b	936.738	-0.7063	0.18304	-3.8584	0.00011	0.00085	903.646	1367.44	1213.66	739.178	629.391	767.121 Down
Smyd3	1173.96	-0.7497	0.17792	-4.2137	2.51E-05	0.00022	1268.39	1752.66	1396.04	884.319	733.938	1008.42 Down
Cnst	3136.09	-0.2564	0.09308	-2.7549	0.00587	0.02475	3338.85	3662.46	3240.87	2824.01	2904.07	2846.29 Down
Kif28	6.11448	-3.4357	1.39853	-2.4566	0.01402	0.05035	6.77251	19.1652	7.63906	3.11015	0	0 Down
Coq8a	1306.72	-1.8454	0.51069	-3.6137	0.0003	0.002	1577.99	3465.06	1090.48	557.753	562.862	586.149 Down
Psen2	1876.62	0.37885	0.12298	3.08064	0.00207	0.01023	1670.88	1650.12	1573.65	2110.75	2379.23	1875.07 Up
Gm5069	241.267	-0.5757	0.16304	-3.5313	0.00041	0.00263	311.535	278.853	275.961	196.976	193.253	191.026 Down
Stum	75.2367	-0.9236	0.32273	-2.8617	0.00421	0.01877	78.3676	130.323	86.8943	55.9827	48.5772	51.2754 Down
Parp1	2993.74	0.53777	0.13424	4.00592	6.18E-05	0.0005	2784.47	2323.78	2218.19	3239.74	3912.57	3483.71 Up
Acbd3	219.577	0.55785	0.25233	2.21078	0.02705	0.08472	199.305	142.781	190.977	238.445	210.149	335.804 Up
Wdr26	8573.59	0.23529	0.08594	2.73776	0.00619	0.02583	7782.58	7869.22	7976.14	8667.98	9725.99	9419.6 Up
A430110L	61.2809	-0.7807	0.29206	-2.6731	0.00751	0.03023	79.3351	79.5355	73.526	41.4686	48.5772	45.243 Down
Gm8146	3.25005	5.20259	1.83744	2.83144	0.00463	0.02035	0	0	0	3.11015	6.33615	10.054 Up
Lbr	3509.95	-0.3586	0.08767	-4.09	4.31E-05	0.00036	4110.91	3734.33	3986.64	3015.81	3054.03	3157.96 Down
Enah	4326.1	-0.8529	0.18409	-4.6331	3.60E-06	3.94E-05	4470.82	6281.39	5954.65	3438.79	2427.8	3383.17 Down
Degs1	8320	0.3187	0.12848	2.48059	0.01312	0.04767	8464.67	6822.8	6926.72	9541.93	9822.09	8341.81 Up
Capn8	82.2595	0.99073	0.34234	2.89402	0.0038	0.01723	67.7251	45.0382	52.5186	132.7	73.9218	121.654 Up
Susd4	1280.88	0.58163	0.13994	4.15616	3.24E-05	0.00028	958.794	1061.75	1058.01	1504.27	1337.98	1764.48 Up
Tlr5	486.808	0.57697	0.19581	2.94662	0.00321	0.01494	476.011	346.89	349.487	547.386	516.396	684.678 Up
Taf1a	984.096	-0.4421	0.14244	-3.1037	0.00191	0.0096	1006.2	1150.87	1244.21	846.997	742.386	913.909 Down
Gm37986	29.3789	-1.3956	0.43167	-3.2331	0.00122	0.00661	43.5376	42.1634	42.0148	19.6976	14.7844	14.0756 Down
1700112H	2.08874	4.56221	2.1536	2.11841	0.03414	NA	0	0	0	2.07343	8.4482	2.0108 Up
Gm37800	27.1511	3.71027	0.81718	4.54032	5.62E-06	5.86E-05	9.67501	0	1.90977	52.8725	61.2495	37.1998 Up
Mtarc1	60.0763	-1.1671	0.42661	-2.7358	0.00622	0.02596	63.8551	101.575	84.0297	49.7624	19.0085	42.2268 Down
C130074G	3305.14	-0.3759	0.17304	-2.1724	0.02982	0.09146	3710.37	3058.76	4430.66	2686.13	3376.11	2568.8 Down
Rab3gap2	2360.54	0.43117	0.11089	3.88833	0.0001	0.00077	1980.48	1877.23	2173.31	2772.18	2857.6	2502.44 Up
Tgfb2	1760.78	0.85259	0.11892	7.16947	7.53E-13	2.75E-11	1402.88	1162.37	1200.29	2166.74	2234.55	2397.88 Up
Spata17	1021.19	-0.5484	0.21732	-2.5236	0.01162	0.04324	1047.8	1295.57	1295.78	908.163	573.422	1006.41 Down
Esrrg	391.697	-1.0726	0.32735	-3.2765	0.00105	0.00581	430.538	654.491	507.998	247.775	139.395	369.988 Down
Kcnk2	3124.65	-0.5016	0.16078	-3.1201	0.00181	0.00915	4061.57	3247.54	3678.21	3113.26	2440.47	2206.85 Down
Cenpf	125.999	1.03636	0.31367	3.30399	0.00095	0.00536	93.8476	84.3268	69.7065	154.471	120.387	233.253 Up
Gm29678	14.3801	3.35446	0.88484	3.79106	0.00015	0.00108	1.935	5.74955	0	32.1382	25.3446	21.1134 Up
Gm30725	10.2311	2.60727	0.84495	3.0857	0.00203	0.0101	4.83751	0.95826	2.86465	14.514	21.1205	17.0918 Up
Nsl1	78.6763	0.87807	0.38148	2.30177	0.02135	0.07016	78.3676	52.7042	35.3307	116.112	73.9218	115.621 Up
Batf3	132.707	0.60498	0.22199	2.72533	0.00642	0.02662	103.523	112.116	100.263	175.205	136.227	168.907 Up
Atf3	1394.32	1.03269	0.30821	3.35061	0.00081	0.00465	774.001	1295.57	677.012	1712.65	2678.08	1228.6 Up
Gm37168	39.5051	2.04715	0.67468	3.03427	0.00241	0.01167	24.1875	5.74955	16.233	41.4686	117.219	32.1728 Up
Dtl	121.472	1.42172	0.35617	3.99171	6.56E-05	0.00052	98.6851	53.6625	45.8344	151.361	152.068	227.221 Up

1700034H	134.136	-1.3443	0.37261	-3.6078	0.00031	0.00204	156.735	198.36	222.488	63.2397	44.3531	119.643	Down
Slc30a1	3436.8	-0.414	0.11714	-3.534	0.00041	0.00261	3956.11	3620.3	4203.39	2691.31	2908.29	3241.41	Down
Gm26879	30.0789	-1.0158	0.50542	-2.0098	0.04445	0.12441	22.2525	47.9129	50.6088	20.7343	15.8404	23.1242	Down
Sertad4	163.746	-0.8869	0.28937	-3.0651	0.00218	0.01069	200.273	284.603	152.781	135.81	102.434	106.572	Down
Irf6	195.605	0.57932	0.24276	2.38638	0.01702	0.05878	178.988	121.699	169.969	215.637	203.813	283.523	Up
Lamb3	4853.23	0.57215	0.16926	3.38034	0.00072	0.00425	4943.93	3168.96	3597.04	5898.91	5740.55	5770	Up
A330023F	836.343	-0.547	0.26408	-2.0713	0.03834	0.11086	736.269	1065.58	1177.37	636.544	477.323	924.969	Down
Meig1	626.995	-0.8003	0.19897	-4.0222	5.76E-05	0.00047	805.929	782.897	801.147	496.587	329.48	545.933	Down
Cdnf	127.217	-1.2445	0.34098	-3.6498	0.00026	0.00176	165.443	254.897	116.496	72.5701	64.4175	89.4807	Down
Bend7	3788.19	0.56556	0.15227	3.71412	0.0002	0.00142	3530.41	2431.1	3203.63	4409.15	4733.11	4421.75	Up
Sephs1	2823.43	0.28678	0.08273	3.46658	0.00053	0.00324	2594.84	2512.55	2523.76	3205.53	3085.71	3018.21	Up
Phyh	4470.13	-0.6279	0.21959	-2.8595	0.00424	0.01888	4383.75	7633.49	4266.42	3468.85	3362.38	3705.91	Down
Mcm10	122.664	0.97958	0.23939	4.09198	4.28E-05	0.00036	78.3676	68.9946	100.263	160.691	165.796	161.87	Up
Optn	1893.06	0.84236	0.14718	5.7233	1.04E-08	1.88E-07	1245.17	1555.25	1266.17	2301.51	2777.35	2212.89	Up
Ccdc3	195.032	-1.084	0.35694	-3.037	0.00239	0.01159	228.33	401.51	165.195	137.883	146.788	90.4861	Down
Gm13391	52.8836	1.5291	0.3309	4.62107	3.82E-06	4.15E-05	29.025	28.7478	23.8721	73.6068	87.6501	74.3997	Up
Gm38386	3.64625	5.36634	1.79655	2.98703	0.00282	0.01334	0	0	0	5.18358	12.6723	4.0216	Up
Gm13217	33.9171	0.89884	0.45181	1.98942	0.04665	0.12925	27.09	22.0399	21.9623	44.5788	28.5127	59.3187	Up
Itih5	475.763	-1.4138	0.17729	-7.9741	1.54E-15	8.32E-14	723.691	758.941	592.982	299.611	225.989	253.361	Down
Prkcq	701.596	-0.5754	0.13141	-4.3785	1.20E-05	0.00011	787.546	921.845	809.741	531.835	553.357	605.251	Down
8030442B	225.754	0.42049	0.20531	2.04802	0.04056	0.11575	206.078	188.777	184.292	241.555	313.64	220.183	Up
Il2ra	152.403	0.51581	0.21244	2.42807	0.01518	0.05359	125.775	126.49	124.135	199.049	149.956	189.015	Up
Itga8	10550.2	-0.7306	0.10472	-6.9762	3.03E-12	1.01E-10	13873	12195.8	13428.5	7550.4	8642.51	7610.88	Down
E030013I1	227.835	-1.7651	0.24022	-7.348	2.01E-13	8.11E-12	330.886	382.345	342.803	120.259	119.331	71.3835	Down
Vim	45601.8	0.45807	0.13739	3.33403	0.00086	0.00488	40840.2	34684.2	39743.2	53418.9	60124.8	44799.7	Up
Mrc1	8054.8	2.32063	0.30004	7.73453	1.04E-14	5.00E-13	3936.76	2702.29	1421.82	12955.8	16072.7	11239.4	Up
Slc39a12	78.4237	2.11291	0.28289	7.4691	8.07E-14	3.50E-12	29.9925	31.6225	26.7367	129.589	130.947	121.654	Up
Cacnb2	623.126	-0.9552	0.17382	-5.4955	3.90E-08	6.28E-07	685.959	982.215	798.282	419.87	458.315	394.117	Down
Arl5b	2098.96	-0.2652	0.11883	-2.2319	0.02562	0.08116	2361.67	2052.59	2459.78	1758.27	1995.89	1965.56	Down
Plxdc2	8911.04	-0.2549	0.08009	-3.1823	0.00146	0.00767	9501.83	10099.1	9487.72	7885.26	8111.33	8381.02	Down
Nebl	14933.1	-0.3808	0.09539	-3.9919	6.56E-05	0.00052	17532.1	17252.5	15893.1	12213.5	12828.6	13878.6	Down
Dnajc1	2993.62	0.2595	0.10156	2.55512	0.01062	0.04018	2956.68	2528.84	2689.91	3147.47	3295.86	3342.96	Up
Commd3	3626.22	0.21067	0.0855	2.46408	0.01374	0.04954	3285.63	3313.66	3486.28	3850.36	4055.14	3766.23	Up
Spag6	51.3876	-0.7801	0.34586	-2.2556	0.02409	0.07734	59.9851	70.9111	63.9772	47.6889	29.5687	36.1944	Down
Pip4k2a	2176.09	0.35172	0.13219	2.66061	0.0078	0.03113	2182.68	1791.94	1761.76	2306.69	2674.91	2338.56	Up
Armc3	790.707	-0.8877	0.23764	-3.7357	0.00019	0.00132	893.004	1160.45	1026.5	636.544	361.161	666.581	Down
Msrb2	1087.41	-0.3453	0.12548	-2.7514	0.00593	0.02496	1204.54	1293.65	1152.54	1067.82	884.949	920.947	Down
Otud1	2651.11	-0.5132	0.09331	-5.4996	3.81E-08	6.16E-07	3243.06	3123.92	2985.92	2238.27	2250.39	2065.09	Down
Gm13375	260.443	-0.517	0.18015	-2.8697	0.00411	0.01837	285.413	350.723	283.6	217.71	229.158	196.053	Down
Enkur	2274.74	-0.4415	0.21379	-2.0653	0.0389	0.11206	2221.38	2896.82	2742.42	2139.78	1330.59	2317.45	Down
Thnsl1	904.349	0.42894	0.12093	3.54711	0.00039	0.00249	718.854	810.687	783.004	973.476	1123.61	1016.46	Up
Gpr158	25.874	-2.6091	0.92937	-2.8074	0.00499	0.02169	19.35	102.534	11.4586	9.33044	10.5603	2.0108	Down
Apbb1ip	1468.5	1.25982	0.26799	4.70106	2.59E-06	2.92E-05	1179.38	828.894	587.253	1864.02	2706.59	1644.84	Up
Mastl	107.443	0.67238	0.24071	2.79329	0.00522	0.02247	73.5301	77.619	97.3981	129.589	127.779	138.745	Up
Hnmt	1691.94	0.24801	0.12262	2.02265	0.04311	0.12153	1667.97	1443.14	1529.72	1896.15	1641.06	1973.6	Up
Il1rn	692.975	2.07953	0.27606	7.5328	4.97E-14	2.21E-12	385.066	242.439	168.059	1001.47	1303.14	1057.68	Up
Psd4	1364.88	0.5238	0.14541	3.6023	0.00032	0.00208	1274.2	978.382	1106.71	1546.78	1794.19	1489	Up
Cacna1b	122.117	0.63561	0.27678	2.29646	0.02165	0.07097	75.4651	120.741	90.7139	176.242	127.779	141.762	Up
Arrdc1	5586.74	0.27966	0.1162	2.4067	0.0161	0.05618	4915.87	4830.58	5394.13	6471.18	6516.73	5391.96	Up
Zmynd19	1077.47	0.32792	0.11719	2.79813	0.00514	0.02221	1025.55	858.6	982.574	1206.74	1164.8	1226.59	Up
Pnpla7	2045.31	1.04979	0.19379	5.41713	6.06E-08	9.40E-07	1598.31	1370.31	1028.41	2649.85	3267.34	2357.67	Up
AL732309	761.766	-1.3429	0.21224	-6.3271	2.50E-10	6.19E-09	845.596	1174.83	1257.58	370.108	535.405	387.079	Down
Tor4a	2615.35	0.50054	0.11451	4.3712	1.24E-05	0.00012	2120.76	1999.89	2377.66	2924.58	3304.3	2964.93	Up
Rnf208	180.779	-1.0038	0.2481	-4.0461	5.21E-05	0.00042	236.07	252.022	235.856	123.369	85.5381	151.816	Down
Tprn	656.648	0.34896	0.14483	2.40949	0.01597	0.05586	531.158	665.99	535.689	777.537	709.649	719.867	Up
Lrrc26	889.348	-1.1812	0.20817	-5.6741	1.39E-08	2.45E-07	1040.06	1584.96	1078.06	636.544	463.595	532.862	Down
Man1b1	4672.56	0.42112	0.11109	3.79082	0.00015	0.00108	4059.64	3541.72	4384.82	5200.17	5425.86	5423.13	Up
Dpp7	1951.66	0.84926	0.10353	8.20329	2.34E-16	1.42E-14	1463.83	1409.6	1306.28	2547.21	2627.39	2355.65	Up
Uap1l1	3148.69	0.39394	0.13387	2.94262	0.00325	0.01508	2901.54	2518.3	2744.33	3768.46	3924.19	3035.31	Up
Sapcd2	72.345	-1.3468	0.38878	-3.464	0.00053	0.00327	61.9201	134.156	115.541	39.3952	33.7928	49.2646	Down
Npdc1	6393.54	0.44299	0.1029	4.30496	1.67E-05	0.00015	5162.59	5463.99	5631.9	7648.89	7786.08	6667.82	Up
Fut7	46.1519	1.04599	0.44955	2.32674	0.01998	0.06676	39.6676	16.2904	34.3758	63.2397	77.0899	46.2484	Up
Abca2	3816.92	0.2673	0.10987	2.43283	0.01498	0.05301	3672.64	3459.31	3260.93	3938.48	4595.82	3974.35	Up
Ptgds	34.6681	-2.8826	0.99511	-2.8968	0.00377	0.0171	16.4475	160.029	6.68418	10.3672	8.4482	6.03241	Down
Kcnt1	9.26353	1.64989	0.78074	2.11325	0.03458	0.1023	3.87001	5.74955	3.81953	10.3672	12.6723	19.1026	Up
Gm13542	3.53197	-3.2312	1.60166	-2.0174	0.04365	0.12273	4.83751	5.74955	8.59395	0	0	2.0108	Down
Ubac1	1785.73	-0.2561	0.10445	-2.452	0.01421	0.05089	1841.16	2123.5	1866.8	1611.06	1621	1650.87	Down
Tmem250	3566.14	-0.2528	0.08054	-3.1388	0.0017	0.00868	3838.08	3895.32	3899.74	3344.45	3287.41	3131.82	Down
Ccdc187	3763.94	-0.7732	0.24624	-3.1403	0.00169	0.00864	3809.05	5378.71	5059.92	2955.68	1769.9	3610.39	Down
Gpsm1	1830.66	-0.6031	0.12582	-4.7931	1.64E-06	1.93E-05	2052.07	2487.64	2083.55	1518.79	1502.72	1339.19	Down
Sec16a	5186.82	0.27637	0.08569	3.2252	0.00126	0.00678	4759.14	4545.98	4769.64	5766.21	5376.23	5903.71	Up
Egfl7	1486.09	-0.3554	0.17662	-2.012	0.04422	0.1239	1584.77	1405.77	2013.85	1292.78	1495.33	1124.04	Down
Dipk1b	369.705	1.29466	0.22131	5.85006	4.91E-09	9.57E-08	198.338	276.937	167.105	459.265	557.581	559.003	Up
Med22	2499.57	0.39468	0.12186	3.23886	0.0012	0.0065	2363.61	1923.23	2192.41	2874.81	3021.29	2622.09	Up
Surf4	11809.4	0.53008	0.09439	5.61593	1.96E-08	3.34E-07	10179.1	9594.09	9218.44	14544.1	14359.8	12960.6	Up
Mymk	7.84904	1.96182	0.92122	2.12959	0.03321	0.09905	3.87001	3.83303	1.90977	12.4406	19.0085	6.03241	Up
Adamtsl2	3097.73	-0.281	0.11783	-2.3852	0.01707	0.05892	3591.37	3335.7	3268.56	3124.66	2512.28	2753.79	Down
Dbh	7.2524	-3.2493	1.43936	-2.2574	0.02398	0.07707	9.67501	25.873	3.81953	4.14686	0	0	Down
Vav2	2732.08	0.84209	0.12117	6.94937	3.67E-12	1.20E-10	1894.37	1947.18	2028.17	3168.2	3985.44	3369.1	Up
Brd3os	387.017	-0.6006	0.13968	-4.2996	1.71E-05	0.00016	497.296	442.715	459.299	322.419	296.743	303.631	Down
Col5a1	1455.31	-0.6067	0.19724	-3.0759	0.0021	0.01037	1400.94	1707.62	2161.85	1216.07	1307.36	938.039	Down
Ppp1r26	716.922	-0.305	0.15061	-2.0251	0.04286	0.12094	814.636	665.99	896.635	666.608	627.279	630.386	Down
1700007K	3108.1	-0.7283	0.26917	-2.7057	0.00682	0.02793	3080.52	3967.19	4581.53	2684.06	1363.33	2971.96	Down

Gbgt1	91.1337	1.3664	0.29677	4.60418	4.14E-06	4.47E-05	66.7576	47.9129	38.1953	135.81	120.387	137.74	Up
Gm10134	418.216	0.82693	0.14967	5.5249	3.30E-08	5.41E-07	304.763	287.478	312.247	555.68	576.59	472.538	Up
Ralgds	4331.46	0.51119	0.1195	4.27765	1.89E-05	0.00017	3854.53	3143.09	3718.31	5053.99	5451.2	4767.61	Up
Spaca9	1285.5	-0.519	0.20122	-2.5794	0.0099	0.03788	1235.5	1568.67	1738.84	1165.27	796.243	1208.49	Down
Ak8	964.292	-0.5666	0.20286	-2.7929	0.00522	0.02249	943.314	1407.68	1102.89	776.5	629.391	925.974	Down
Ddx31	689.736	0.32992	0.11205	2.94429	0.00324	0.01502	591.143	617.119	625.448	754.729	747.666	802.31	Up
Cfap77	921.801	-0.4647	0.2287	-2.032	0.04216	0.11944	1011.04	1061.75	1134.4	870.841	502.668	950.104	Down
Rapgef1	4089.78	-0.297	0.14065	-2.1116	0.03472	0.10262	3871.94	4331.33	5324.43	3490.62	3857.66	3662.68	Down
Slc27a4	2212.54	0.39294	0.12674	3.10031	0.00193	0.00969	2143.98	1663.54	1931.73	2640.52	2478.49	2416.98	Up
Odf2	3684.07	-0.2879	0.11762	-2.4473	0.01439	0.05135	3918.38	3808.12	4424.93	3329.93	3009.67	3613.41	Down
Gle1	2671.15	-0.2418	0.10244	-2.3606	0.01825	0.06214	3108.58	2755.95	2818.81	2567.95	2473.21	2302.37	Down
Wdr34	1564.99	-0.4981	0.17345	-2.8715	0.00409	0.0183	1706.67	1928.02	1862.98	1448.29	983.16	1460.85	Down
Zdhhc12	811.261	0.52238	0.141	3.70493	0.00021	0.00146	630.811	678.447	688.471	1042.94	1008.5	818.396	Up
Zer1	2124.99	-0.2081	0.10038	-2.0728	0.03819	0.11051	2372.31	2212.62	2248.75	2073.43	2017.01	1825.81	Down
Tbc1d13	2231.63	0.40236	0.12371	3.25248	0.00114	0.00626	2019.18	1905.02	1842.92	2430.06	2888.23	2304.38	Up
Endog	2857.91	-0.9319	0.18067	-5.158	2.50E-07	3.48E-06	3084.39	4056.31	4109.82	2350.23	1587.21	1959.53	Down
Kyat1	1350.53	-0.7326	0.09968	-7.3496	1.99E-13	8.03E-12	1675.71	1640.54	1742.66	1040.86	957.815	1045.62	Down
Dolpp1	1493.97	0.41002	0.10036	4.08531	4.40E-05	0.00036	1193.9	1296.52	1358.8	1702.29	1698.09	1714.21	Up
Ptpa	6063.45	0.21373	0.09228	2.31598	0.02056	0.06817	5696.65	5498.49	5650.04	6745.91	6793.41	5996.21	Up
Gm14487	19.5954	-1.291	0.63143	-2.0445	0.0409	0.11656	17.415	41.2051	24.827	6.2203	15.8404	12.0648	Down
9330198N	52.7499	-0.9023	0.39753	-2.2697	0.02322	0.07518	46.4401	82.4102	77.3455	46.6522	27.4567	36.1944	Down
Ptges	2630.35	1.35127	0.08857	15.2566	1.49E-52	1.89E-49	1478.34	1529.38	1436.14	3792.31	3909.41	3636.53	Up
Tor1b	3385.69	0.24759	0.07826	3.16371	0.00156	0.00808	3066.01	3138.3	3083.32	3583.93	3724.6	3717.97	Up
BC005624	3672.17	-0.2814	0.09652	-2.9154	0.00355	0.01627	4054.8	3952.82	4080.21	3300.9	3074.09	3570.18	Down
Gpr107	3615.07	0.45966	0.07804	5.88994	3.86E-09	7.67E-08	3088.26	3080.8	2963	4169.67	4144.9	4243.8	Up
Ncs1	712.544	-0.5425	0.23698	-2.2893	0.02206	0.0721	714.984	839.435	980.665	585.744	403.402	751.034	Down
Hmcn2	240.207	-1.0991	0.29638	-3.7085	0.00021	0.00144	260.258	427.383	295.059	176.242	100.322	181.978	Down
Ass1	1206.37	-0.6813	0.13332	-5.1099	3.22E-07	4.41E-06	1330.31	1552.38	1575.56	864.621	881.781	1033.55	Down
Nup214	2311.56	0.80165	0.1153	6.95268	3.58E-12	1.18E-10	1827.61	1621.37	1607.07	2747.3	3217.71	2848.3	Up
Fam78a	432.758	-0.556	0.21164	-2.6271	0.00861	0.03377	429.571	664.073	451.66	331.749	390.729	328.766	Down
Plpp7	205.105	-1.6281	0.30523	-5.3341	9.60E-08	1.42E-06	234.135	441.757	253.999	82.9373	97.1543	120.648	Down
Prrc2b	4172.67	0.61866	0.15833	3.90749	9.33E-05	0.00071	3942.57	2777.99	3153.98	4497.27	5095.32	5568.92	Up
Pomt1	1050.97	-0.3237	0.12811	-2.5266	0.01152	0.04294	1071.02	1147.99	1286.23	956.889	855.381	988.309	Down
Swi5	4142.79	-0.2169	0.10664	-2.0342	0.04193	0.11898	4140.91	4543.1	4677.02	4100.21	3518.68	3876.83	Down
Dnm1	908.746	0.41131	0.14379	2.86041	0.00423	0.01884	886.231	730.193	723.801	940.301	1138.4	1033.55	Up
1110008P	359.622	0.7634	0.13475	5.66522	1.47E-08	2.57E-07	270.9	265.438	263.548	459.265	456.203	442.376	Up
Lcn2	47950.4	3.97859	0.47374	8.39824	4.53E-17	3.03E-15	8509.18	4948.45	3703.99	93763.7	55581.8	121195	Up
St6galnac4	1956.49	0.40972	0.11918	3.4377	0.00059	0.00355	1853.73	1523.63	1664.36	2094.17	2230.33	2372.75	Up
St6galnac[illegible]	1540.63	-0.2934	0.13342	-2.1992	0.02786	0.08682	1887.6	1684.62	1518.26	1273.09	1358.05	1522.18	Down
Ak1	1733.83	-1.0056	0.24346	-4.1303	3.62E-05	0.00031	1987.25	3320.37	1636.67	1145.57	1063.42	1249.71	Down
Eng	21275.4	-0.9071	0.16298	-5.5658	2.61E-08	4.34E-07	24885.1	25829.9	32541.5	13303.1	17762.3	13330.6	Down
Fpgs	1197.67	-0.3665	0.12145	-3.0174	0.00255	0.01225	1216.15	1438.35	1392.22	1040.86	1115.16	983.282	Down
Sh2d3c	4759.95	-0.7123	0.11444	-6.2246	4.83E-10	1.12E-08	5991.74	5298.21	6445.46	3828.59	3379.28	3616.43	Down
Ttc16	1973.41	-1.0084	0.21074	-4.7849	1.71E-06	2.00E-05	2300.72	2819.2	2789.21	1545.74	897.622	1487.99	Down
Cfap157	944.238	-0.441	0.1637	-2.6939	0.00706	0.02875	975.241	1173.87	1113.39	868.768	648.4	885.758	Down
Garnl3	1142.15	0.45598	0.19367	2.35443	0.01855	0.06298	1207.44	927.594	754.357	1316.63	1481.6	1165.26	Up
Ralgps1	920.127	0.30221	0.11052	2.73435	0.00625	0.02603	832.051	780.981	859.395	1039.83	958.871	1049.64	Up
C130021I2	158.383	-0.9691	0.35612	-2.7214	0.0065	0.02686	185.76	315.267	127.954	131.663	97.1543	92.4969	Down
Mvb12b	3358.88	0.50637	0.09218	5.49345	3.94E-08	6.35E-07	2978.94	2702.29	2645.03	4006.91	3924.19	3895.93	Up
C230014C	140.174	1.98129	0.23223	8.53156	1.44E-17	1.04E-15	55.1476	64.2033	50.6088	198.013	241.83	231.242	Up
Hspa5	38785	0.4019	0.12788	3.14289	0.00167	0.00858	28894.4	32498.4	38859	45098.2	44074.3	43285.5	Up
Cutal	686.164	0.99117	0.17254	5.7446	9.21E-09	1.69E-07	565.021	387.136	425.878	869.805	903.958	965.185	Up
Traf1	3272.07	-0.7471	0.12386	-6.0324	1.62E-09	3.43E-08	3951.28	4013.19	4338.03	2768.03	2399.29	2162.62	Down
Hc	25888.9	0.95986	0.20033	4.79135	1.66E-06	1.95E-05	16255	19454.6	17033.2	26789.8	29900.3	45900.6	Up
AI182371	350.277	0.45463	0.22263	2.04208	0.04114	0.11711	242.843	351.681	292.194	384.622	332.648	497.673	Up
Cntrl	3631.54	0.40454	0.0814	4.96989	6.70E-07	8.62E-06	3040.86	3159.38	3176.9	4022.46	4122.72	4266.92	Up
Gsn	120638	-0.8748	0.13932	-6.2792	3.40E-10	8.18E-09	164725	151102	152568	95443.2	90654.5	69333.4	Down
Ttll11	350.82	-0.5966	0.14792	-4.0331	5.50E-05	0.00045	387.968	433.133	445.93	264.363	281.959	291.566	Down
Ndufa8	3332.29	-0.3067	0.12907	-2.3761	0.0175	0.06001	3289.5	4239.34	3526.38	3057.28	3096.27	2784.96	Down
Morn5	276.398	-0.6859	0.23348	-2.9377	0.00331	0.01529	266.063	390.011	366.675	243.628	165.796	226.215	Down
Gm13431	57.6991	0.69061	0.30852	2.23847	0.02519	0.08019	36.7651	45.9964	49.6539	68.4232	74.9778	70.3781	Up
Ptgs1	7118.24	1.42416	0.1153	12.3519	4.76E-35	1.49E-32	4227.98	3978.69	3387.92	10046.8	10902.4	10165.6	Up
Lhx2	4.48333	4.65063	1.79267	2.59424	0.00948	0.03654	0.9675	0	0	19.6976	4.2241	2.0108	Up
Nek6	3173.51	1.73263	0.13544	12.7922	1.81E-37	7.24E-35	1536.39	1420.14	1447.6	4795.85	5645.51	4195.54	Up
Nr5a1os	2.04333	4.5338	2.10754	2.15123	0.03146	NA	0	0	0	3.11015	2.11205	7.03781	Up
Nr6a1	1128.28	0.50445	0.11604	4.34719	1.38E-05	0.00013	851.401	947.718	999.762	1293.82	1397.12	1279.88	Up
Olfml2a	1579.14	-0.9647	0.1991	-4.8454	1.26E-06	1.52E-05	1934.04	2102.42	2228.7	1082.33	775.123	1352.26	Down
Kynu	554.113	2.57281	0.34906	7.37066	1.70E-13	6.98E-12	265.095	116.908	96.4432	850.107	1203.87	792.256	Up
Lypd6b	687.705	0.38396	0.13662	2.81033	0.00495	0.02154	554.378	584.538	651.23	858.401	728.658	749.024	Up
Nmi	1722.78	0.30483	0.10918	2.79209	0.00524	0.02253	1543.16	1498.72	1582.24	1938.66	2047.63	1726.27	Up
Tnfaip6	194.23	0.97777	0.36842	2.65393	0.00796	0.03167	95.7826	163.862	132.729	180.389	407.626	184.994	Up
Neb	216.139	-0.3938	0.20015	-1.9677	0.0491	0.13472	220.59	277.895	237.766	203.196	198.533	158.853	Down
Cacnb4	52.2698	-1.3123	0.38859	-3.3771	0.00073	0.00428	53.2126	95.8259	74.4809	30.0648	35.9049	24.1296	Down
Stam2	2889.03	0.25437	0.09491	2.68015	0.00736	0.02972	2752.54	2705.16	2447.36	3044.83	3165.96	3218.29	Up
Fmnl2	2966.42	-0.4764	0.12276	-3.8809	0.0001	0.00079	3240.16	3901.07	3214.14	2354.38	2672.8	2415.98	Down
Prpf40a	4590.16	0.30211	0.08885	3.40036	0.00067	0.004	4238.62	4059.18	4036.29	4812.43	5024.57	5369.85	Up
Gm13502	36.5655	1.45451	0.68843	2.11281	0.03462	0.10237	23.22	32.5808	2.86465	41.4686	54.9133	64.3457	Up
Rprm	703.122	1.78737	0.24987	7.15331	8.47E-13	3.06E-11	397.643	257.772	292.194	1046.05	1428.8	796.277	Up
Galnt13	623.998	1.53982	0.26775	5.75101	8.87E-09	1.63E-07	409.253	213.692	335.164	976.586	1123.61	685.683	Up
Gm14033	5.79684	-3.9717	1.51905	-2.6146	0.00893	0.03477	10.6425	18.2069	3.81953	0	2.11205	0	Down
Kcnj3	670.921	-5.742	1.65129	-3.4773	0.00051	0.00314	803.994	2563.34	584.388	13.4773	0	60.3241	Down
Cytip	6081.63	0.75907	0.14588	5.20355	1.96E-07	2.77E-06	5346.41	4409.91	3796.61	8201.46	7533.68	7201.69	Up
Acvr1c	27.7479	-2.7411	0.66133	-4.1449	3.40E-05	0.00029	26.1225	86.2433	32.466	6.2203	7.39218	8.04321	Down

Upp2	917.01	-0.3896	0.11927	-3.2668	0.00109	0.00598	1014.91	1102.96	1002.63	793.088	732.882	855.596	Down
Cd302	4132.04	0.95421	0.13255	7.19893	6.07E-13	2.26E-11	3170.5	2845.07	2424.45	5249.93	5172.41	5929.85	Up
Ly75	2566.26	1.89431	0.22267	8.50724	1.78E-17	1.26E-15	1418.36	1041.63	804.011	3487.51	4962.26	3683.79	Up
Pla2r1	1600.61	-0.5497	0.13044	-4.2144	2.50E-05	0.00022	1997.89	1631.91	2075.92	1241.99	1356.99	1298.98	Down
Itgb6	4366.49	1.31245	0.16389	8.00799	1.17E-15	6.42E-14	2072.39	2849.86	2598.24	5338.05	7188.36	6152.05	Up
Tank	2875.79	0.38245	0.08462	4.51954	6.20E-06	6.42E-05	2428.43	2539.39	2522.8	3212.78	3170.19	3381.16	Up
Psmd14	3967.01	0.2919	0.0782	3.73287	0.00019	0.00133	3530.41	3633.72	3536.89	4383.23	4466.99	4250.83	Up
Ifih1	2657	0.58962	0.10655	5.53347	3.14E-08	5.17E-07	2311.36	2092.84	1960.37	3032.39	3248.33	3296.71	Up
Gca	1399.91	0.70643	0.10825	6.52587	6.76E-11	1.84E-09	1156.16	1008.09	1027.45	1676.37	1728.71	1802.68	Up
Fign	625.221	-1.3024	0.20239	-6.4352	1.23E-10	3.22E-09	835.921	1090.5	742.899	365.961	297.799	418.247	Down
Grb14	5089.38	0.3874	0.12124	3.19544	0.0014	0.00738	4214.44	4905.33	4110.77	5267.55	5736.33	6301.85	Up
Scn3a	780.509	-1.0957	0.24202	-4.5274	5.97E-06	6.20E-05	737.236	1318.56	1134.4	402.246	602.99	487.619	Down
Galnt3	2710.23	1.30181	0.10038	12.9691	1.83E-38	7.75E-36	1640.88	1562.92	1488.66	3813.04	4115.33	3640.56	Up
Xirp2	267.896	-5.4607	1.00641	-5.4259	5.77E-08	9.01E-07	308.633	1026.3	236.811	11.4039	2.11205	22.1188	Down
B3galt1	133.529	-0.636	0.2784	-2.2845	0.02234	0.07275	130.613	198.36	158.511	78.7904	118.275	116.627	Down
Stk39	1972.97	0.78709	0.24629	3.19583	0.00139	0.00738	1223.89	2077.5	1041.78	2312.91	2892.45	2289.3	Up
Gm13597	323.224	0.7524	0.16899	4.45241	8.49E-06	8.51E-05	265.095	229.024	228.217	386.695	458.315	371.998	Up
Cers6	1712.07	0.38266	0.11458	3.33967	0.00084	0.0048	1598.31	1320.48	1540.23	1896.15	1996.94	1920.32	Up
Nostrin	5065.38	-0.4107	0.1604	-2.5602	0.01046	0.03969	4795.9	5528.19	7020.3	3910.49	4578.93	4558.49	Down
Lrp2	9674.22	2.53681	0.21236	11.9457	6.83E-33	1.80E-30	3904.84	2484.76	2142.76	14888.3	17631.4	16993.3	Up
Klhl41	307.636	-3.0479	0.71265	-4.2768	1.90E-05	0.00017	266.063	1060.79	319.886	44.5788	55.9693	98.5293	Down
Ccdc173	559.455	-0.5093	0.24961	-2.0402	0.04133	0.11753	602.753	731.151	637.862	471.706	300.967	612.289	Down
Myo3b	100.444	0.83605	0.3507	2.38394	0.01713	0.05904	107.393	57.4955	51.5637	134.773	106.659	144.778	Up
Rpl9-ps7	198.028	-0.7421	0.25345	-2.9279	0.00341	0.01573	226.395	206.984	310.337	138.92	123.555	181.978	Down
Sp5	345.176	2.29421	0.26812	8.55667	1.16E-17	8.47E-16	142.223	94.8676	113.631	647.947	684.304	388.085	Up
Gad1	64.8371	-1.1089	0.37969	-2.9206	0.00349	0.01605	60.9526	116.908	87.8492	39.3952	51.7452	32.1728	Down
Gorasp2	7150.98	0.23068	0.08903	2.59099	0.00957	0.03684	6463.88	6424.17	6853.19	7895.63	8032.13	7236.88	Up
Slc25a12	2726.9	-0.3433	0.16323	-2.1034	0.03543	0.10422	2769.96	3809.08	2570.54	2335.72	2452.09	2424.02	Down
Metap1d	811.524	-0.5186	0.11193	-4.633	3.60E-06	3.94E-05	922.996	930.469	1014.09	653.131	654.736	693.727	Down
Platr26	9.69172	-2.5986	1.08651	-2.3917	0.01677	0.05811	12.5775	28.7478	8.59395	6.2203	0	2.0108	Down
Pdk1	2118.22	-0.4375	0.12529	-3.492	0.00048	0.00299	2452.62	2707.08	2151.35	1717.84	1755.11	1925.34	Down
Rapgef4	1667.92	-0.6372	0.11552	-5.5158	3.47E-08	5.64E-07	2032.72	2160.87	1897.35	1301.08	1415.07	1200.45	Down
Map3k20	4001.97	-0.5607	0.12756	-4.3952	1.11E-05	0.00011	4327.63	5338.46	4643.6	2999.22	3584.15	3118.75	Down
Ola1	2407.01	0.38146	0.10592	3.60136	0.00032	0.00208	2114.96	2256.7	1900.22	2706.87	2810.08	2653.25	Up
Scrn3	2058.45	-0.375	0.08359	-4.4865	7.24E-06	7.39E-05	2293.95	2337.19	2342.33	1832.91	1767.79	1776.54	Down
Gpr155	3267.4	-1.2706	0.1527	-8.321	8.72E-17	5.60E-15	5116.15	4829.62	3914.06	2177.1	1663.24	1904.23	Down
Atp5g3	8946.01	-0.3376	0.17217	-1.9606	0.04993	0.13668	8741.38	12662.4	8559.57	8490.7	8078.59	7143.37	Down
Gm13660	2.58057	4.86898	1.94428	2.50426	0.01227	0.0452	0	0	0	8.29373	3.16808	4.0216	Up
Gm13655	33.3563	1.13875	0.51773	2.1995	0.02784	0.08677	30.96	10.5408	21.0074	59.0928	44.3531	34.1836	Up
Nfe2l2	17265.8	0.51023	0.1116	4.57189	4.83E-06	5.13E-05	15002.1	12596.3	15133.9	19673.8	19573.4	21615.1	Up
Gm13669	189.915	0.77309	0.18695	4.13533	3.54E-05	0.0003	144.158	144.697	131.774	265.399	231.27	222.194	Up
Agps	5494	0.26772	0.09461	2.8298	0.00466	0.02044	5142.27	4781.71	5033.19	5550.58	6173.52	6282.75	Up
Pde11a	25.1726	-1.7798	0.54126	-3.2882	0.00101	0.00562	24.1875	43.1216	49.6539	14.514	9.50423	10.054	Down
Osbpl6	6513.08	-0.4678	0.14324	-3.2658	0.00109	0.006	8024.46	7490.71	7164.49	6339.52	4514.51	5544.79	Down
Fkbp7	1314.47	-0.583	0.13136	-4.4382	9.07E-06	9.00E-05	1605.08	1568.67	1555.5	1151.79	1110.94	894.807	Down
Ttn	4616.22	-5.6602	1.10638	-5.1159	3.12E-07	4.28E-06	5329	17840.9	3990.46	116.112	34.8488	386.074	Down
Ccdc141	4490.79	0.51876	0.10359	5.00784	5.50E-07	7.20E-06	3821.63	3872.32	3382.2	5237.49	5011.9	5619.19	Up
Zfp385b	418.547	1.44193	0.21466	6.71727	1.85E-11	5.57E-10	282.51	206.026	187.157	509.027	701.201	625.359	Up
Gm14461	2.08752	4.56149	2.16346	2.10843	0.03499	NA	0	0	0	5.18358	6.33615	1.0054	Up
Pde1a	258.004	-0.7645	0.17594	-4.3456	1.39E-05	0.00013	294.12	358.389	321.796	196.976	203.813	172.929	Down
Gm4735	898.944	-0.4378	0.19321	-2.2662	0.02344	0.07572	1280.97	814.52	1007.4	699.783	869.109	721.878	Down
Gm13680	263.425	-0.4021	0.17145	-2.3452	0.01902	0.06419	314.438	265.438	319.886	238.445	209.093	233.253	Down
Itgav	3782.04	0.63591	0.09935	6.40073	1.55E-10	3.99E-09	3147.28	2729.12	3008.84	4443.36	4810.2	4553.46	Up
Fam171b	89.83	-0.9727	0.3112	-3.1257	0.00177	0.00901	118.035	121.699	117.451	46.6522	50.6892	84.4537	Down
Gm13711	14.6315	2.46781	1.16943	2.11028	0.03483	0.10285	3.87001	5.74955	3.81953	63.2397	1.05603	10.054	Up
Tfpi	2624.58	-0.5306	0.11964	-4.4351	9.20E-06	9.11E-05	2966.36	3416.19	2922.9	2000.86	2355.99	2085.2	Down
Med19	2046.85	0.21861	0.1032	2.11827	0.03415	0.10127	1897.27	1809.19	1969.92	2282.85	2027.57	2294.32	Up
Zdhhc5	4446.34	0.3459	0.09277	3.72837	0.00019	0.00135	4051.9	3845.49	3850.09	4726.39	5335.04	4869.16	Up
Smtnl1	59.64	1.55267	0.59249	2.62058	0.00878	0.0343	8.70751	35.4556	46.7893	132.7	51.7452	82.4429	Up
Slc43a1	263.22	-1.5598	0.27484	-5.675	1.39E-08	2.44E-07	491.491	340.182	347.577	182.462	114.051	103.556	Down
Slc43a3	20710.5	-0.5594	0.11301	-4.9497	7.43E-07	9.49E-06	27392.9	23029.8	23604.7	16891.2	17893.3	15451	Down
Tnks1bp1	8334.61	0.26833	0.10345	2.59384	0.00949	0.03656	7610.37	6820.89	8254.01	8904.35	9125.12	9292.92	Up
Olfr1033	3844.16	-1.4672	0.24785	-5.9196	3.23E-09	6.50E-08	3437.53	6964.62	6536.17	2265.22	2153.24	1708.18	Down
Agbl2	1735.8	-0.5497	0.24936	-2.2043	0.0275	0.08587	1900.17	2227.95	2059.68	1519.83	852.212	1854.96	Down
Mtch2	6608.77	0.2424	0.09364	2.58866	0.00963	0.03703	6116.54	6388.71	5659.59	7141.94	6853.6	7492.25	Up
Ndufs3	4086.01	-0.2594	0.13014	-1.9931	0.04625	0.1283	3947.41	5203.34	4206.26	3820.3	3698.2	3640.56	Down
Spi1	2159.74	1.58702	0.26261	6.04324	1.51E-09	3.23E-08	1492.85	993.714	749.583	3111.18	4122.72	2488.37	Up
Mybpc3	1259.32	-5.1804	0.88231	-5.8714	4.32E-09	8.53E-08	1092.31	5340.38	920.507	50.7991	44.3531	107.578	Down
Acp2	1347.85	0.84731	0.11999	7.06167	1.65E-12	5.72E-11	1031.36	986.048	871.808	1600.69	1801.58	1795.65	Up
1110051M	2517.48	-0.4573	0.10238	-4.4663	7.96E-06	8.01E-05	2978.94	2971.56	2789.21	2171.92	1944.14	2249.08	Down
Arhgap1	8674.63	0.26593	0.08458	3.14397	0.00167	0.00855	8183.13	7565.45	7883.51	9691.22	9708.04	9016.43	Up
Creb3l1	1327.15	0.4092	0.19671	2.08026	0.0375	0.10896	837.856	1238.07	1344.48	1600.69	1635.78	1306.02	Up
Cry2	4222.79	-0.3835	0.13271	-2.8898	0.00386	0.01743	5429.62	4313.12	4599.67	3980.99	3285.29	3728.03	Down
Slc35c1	1896.32	0.74281	0.11405	6.51281	7.38E-11	1.99E-09	1551.87	1347.31	1356.89	2514.04	2203.92	2403.91	Up
Chst1	988.305	-0.6024	0.1852	-3.2528	0.00114	0.00625	1215.18	1122.12	1237.53	719.481	1001.11	634.408	Down
Tspan18	4881.02	-0.6149	0.14294	-4.302	1.69E-05	0.00016	5525.4	5897.12	6294.59	3581.85	4597.93	3389.21	Down
Hsd17b12	6208.65	0.59473	0.09509	6.25452	3.99E-10	9.43E-09	4935.22	5116.14	4788.74	7266.34	8076.48	7068.97	Up
Lrrc4c	134.353	-0.6006	0.2209	-2.7187	0.00655	0.02701	151.898	149.488	184.292	105.745	116.163	98.5293	Down
Prr5l	1372.61	0.51933	0.18328	2.83347	0.0046	0.02024	1263.56	1105.83	1015.04	1561.29	1998	1291.94	Up
Slc1a2	178.016	-0.7276	0.21069	-3.4535	0.00055	0.00338	193.5	220.399	252.089	145.14	117.219	139.751	Down
Cd44	13731.7	1.59976	0.1541	10.381	3.03E-25	4.11E-23	7458.47	6882.21	6098.84	19085.9	24927.5	17937.4	Up
Ehf	2770.17	0.68469	0.31714	2.15896	0.03085	0.09368	2148.82	2345.82	1880.16	3401.46	1676.97	5167.76	Up
Abtb2	442.224	-0.3246	0.15463	-2.0989	0.03582	0.10515	441.181	486.795	547.148	379.438	429.802	368.982	Down

Caprin1	12783.4	0.37275	0.08046	4.63264	3.61E-06	3.94E-05	11462	10871.4	11090	13731.3	14853	14692.9	Up
D430041D	67.3839	0.77609	0.3724	2.08403	0.03716	0.10823	67.7251	50.7877	30.5563	72.5701	98.2104	84.4537	Up
Ccdc73	422.005	-0.4785	0.16206	-2.9525	0.00315	0.01469	432.473	476.255	565.291	329.676	365.385	362.95	Down
Wt1os	9.53504	2.13717	0.97661	2.18835	0.02864	0.08875	6.77251	3.83303	0	16.5875	17.9524	12.0648	Up
Rcn1	2719.09	-0.5773	0.12673	-4.5557	5.22E-06	5.49E-05	3400.77	2934.19	3432.8	2175.03	2417.24	1954.5	Down
Immp1l	2307.49	-0.2665	0.09061	-2.9414	0.00327	0.01514	2450.68	2623.71	2485.56	2060.99	2188.08	2035.94	Down
Gm14015	18.4256	-1.4366	0.65952	-2.1783	0.02938	0.09049	34.8301	28.7478	17.1879	4.14686	10.5603	15.081	Down
Mpped2	216.021	-0.4447	0.18806	-2.3647	0.01805	0.0616	258.323	222.316	266.412	191.792	196.421	160.864	Down
Hadhb-ps	1673.61	-0.5828	0.21773	-2.6766	0.00744	0.02994	1726.02	2762.66	1532.59	1319.74	1428.8	1271.83	Down
Mettl15	353.243	-0.441	0.14651	-3.0098	0.00261	0.01251	402.481	439.841	378.134	312.051	289.351	297.599	Down
Bbox1	781.249	-1.0424	0.33897	-3.0752	0.0021	0.01039	872.686	1313.77	969.206	521.468	250.278	760.083	Down
Fibin	2948.54	-1.0403	0.1303	-7.984	1.42E-15	7.70E-14	4132.2	3576.22	4194.8	2052.7	2060.31	1675	Down
Slc5a12	143.453	-0.9288	0.30059	-3.0898	0.002	0.00998	178.988	163.862	221.533	65.3131	100.322	130.702	Down
Olfr1318	6.48728	3.18466	1.29313	2.46276	0.01379	0.0497	3.87001	0	0	10.3672	11.6163	13.0702	Up
Lpcat4	1908.75	1.09939	0.12386	8.87639	6.91E-19	5.56E-17	1138.75	1306.11	1199.33	2918.36	2447.87	2442.12	Up
Nutm1	1.88621	4.41731	2.12293	2.08076	0.03746	NA	0	0	0	5.18358	2.11205	4.0216	Up
Gm21985	41.1358	-1.045	0.37949	-2.7537	0.00589	0.02482	64.8226	56.5373	44.8795	31.1015	25.3446	24.1296	Down
Ryr3	131.921	-0.8744	0.37661	-2.3216	0.02025	0.06738	111.263	251.064	149.917	81.9006	69.6977	127.686	Down
Fmn1	401.215	0.49519	0.20729	2.38887	0.0169	0.05846	354.106	399.594	245.405	439.568	491.052	477.565	Up
Grem1	36.5024	-1.4411	0.68816	-2.0941	0.03625	0.10619	18.3825	96.7841	44.8795	33.1749	13.7283	12.0648	Down
Scg5	57.5458	-1.4854	0.32145	-4.6208	3.82E-06	4.16E-05	83.2051	76.6607	94.5334	34.2116	28.5127	28.1512	Down
Arhgap11	490.723	1.81893	0.15053	12.0833	1.29E-33	3.58E-31	233.168	223.274	193.841	694.6	794.131	805.326	Up
A530058N	19.2459	-3.5367	1.14651	-3.0847	0.00204	0.01012	6.77251	80.4937	19.0977	2.07343	0	7.03781	Down
Meis2	1196.7	-0.4579	0.15184	-3.0159	0.00256	0.0123	1603.15	1209.32	1342.57	1060.56	1070.81	893.801	Down
G630016G	40.1471	-1.3443	0.41088	-3.2719	0.00107	0.00589	55.1476	68.9946	48.699	15.5507	25.3446	27.1458	Down
Rasgrp1	624.327	0.30595	0.14056	2.17669	0.0295	0.09076	612.428	558.665	504.178	643.801	675.856	751.034	Up
Gm13986	59.1673	-0.8512	0.34079	-2.4976	0.0125	0.04587	71.5951	77.619	79.2553	49.7624	28.5127	48.2592	Down
Gpr176	22.7346	2.09278	0.59452	3.5201	0.00043	0.00273	6.77251	6.70781	12.4135	21.771	47.5211	41.2214	Up
Eif2ak4	1927.5	0.32575	0.10492	3.10482	0.0019	0.00957	1845.99	1579.21	1707.33	2071.36	2153.24	2207.86	Up
Bmf	2097	0.81717	0.10904	7.49423	6.67E-14	2.93E-12	1658.3	1384.68	1512.53	2601.12	2773.12	2652.25	Up
Bub1b	396.429	1.48912	0.22901	6.50245	7.90E-11	2.12E-09	259.29	221.358	144.187	529.762	595.598	628.376	Up
Ankrd63	374.204	-1.5275	0.24116	-6.334	2.39E-10	5.94E-09	405.383	561.54	699.929	179.352	228.101	170.918	Down
Plcb2	1338.74	1.18481	0.24338	4.86821	1.13E-06	1.37E-05	1028.45	743.609	681.786	1645.27	2520.73	1412.59	Up
Phgr1	221.144	2.88055	0.30538	9.43282	3.99E-21	3.83E-19	73.5301	40.2469	44.8795	364.924	502.668	300.615	Up
Knstrn	145.589	0.85287	0.23891	3.56985	0.00036	0.00231	124.808	98.7006	87.8492	211.49	163.684	187.005	Up
Ivd	6081.36	-0.6088	0.1513	-4.024	5.72E-05	0.00046	6793.8	8178.74	7065.18	4897.45	3978.05	5574.95	Down
Bahd1	2223.7	0.27575	0.10084	2.73446	0.00625	0.02602	2037.56	1852.31	2145.62	2369.93	2480.6	2456.19	Up
Rad51	124.919	0.87644	0.34867	2.51369	0.01195	0.04416	111.263	95.8259	57.293	179.352	107.715	198.064	Up
Rmdn3	384.861	0.56426	0.15561	3.62605	0.00029	0.00191	346.366	286.519	298.878	444.751	432.97	499.684	Up
Gchfr	83.5023	-0.5376	0.24826	-2.1655	0.03035	0.09253	94.8151	100.617	101.218	66.3498	68.6416	69.3727	Down
Zfyve19	1413.83	0.2051	0.09675	2.11982	0.03402	0.10097	1309.03	1331.98	1299.6	1585.14	1434.08	1523.18	Up
Ppp1r14d	487.549	2.23194	0.19306	11.5609	6.50E-31	1.38E-28	136.418	207.942	169.014	900.906	731.826	779.186	Up
Spint1	5603.7	0.86801	0.15196	5.71222	1.12E-08	2.00E-07	4870.4	3511.06	3519.7	7610.53	7146.12	6964.41	Up
Rhov	148.955	-0.5999	0.29963	-2.0021	0.04527	0.12615	180.923	160.987	196.706	119.222	76.0338	159.859	Down
Vps18	1652.75	0.61492	0.13755	4.47038	7.81E-06	7.89E-05	1349.66	1283.11	1284.32	1947.99	2321.14	1730.29	Up
Chac1	230.53	1.12562	0.46102	2.44158	0.01462	0.052	128.678	125.532	180.473	599.222	155.236	194.042	Up
Chp1	13218	0.6394	0.13708	4.66455	3.09E-06	3.43E-05	11552	10274.4	9181.2	15454.3	18357.9	14487.8	Up
Itpka	1058.61	-0.8369	0.20153	-4.1526	3.29E-05	0.00028	1244.21	1252.44	1575.56	816.932	559.693	902.85	Down
Mapkbp1	662.778	0.74155	0.11284	6.57165	4.98E-11	1.38E-09	504.068	476.255	507.998	805.528	834.26	848.558	Up
Ehd4	26351.4	-0.438	0.12661	-3.4596	0.00054	0.00331	29590.1	33576.4	27796.6	21209.1	25316.1	20619.8	Down
Pla2g4e	32.1844	-3.4149	0.70258	-4.8605	1.17E-06	1.42E-05	33.8626	110.2	32.466	6.2203	6.33615	4.0216	Down
Pla2g4f	1203.04	2.76376	0.21101	13.0975	3.40E-39	1.56E-36	371.521	308.559	246.32	1820.47	2649.57	1821.79	Up
Vps39	3169.94	0.30008	0.10657	2.81564	0.00487	0.02123	3072.78	2737.74	2713.78	3292.61	3754.17	3448.52	Up
Tmem87a	4129.94	0.38753	0.08585	4.51386	6.37E-06	6.57E-05	3522.67	3514.89	3698.26	4497.27	4640.18	4906.36	Up
Ganc	468.118	0.30845	0.13754	2.24262	0.02492	0.07948	421.831	392.886	440.201	498.66	492.108	563.024	Up
Snap23	5046.33	0.37775	0.09204	4.10421	4.06E-05	0.00034	4373.11	4242.21	4552.88	5322.5	5976.05	5811.22	Up
Stard9	3012.72	-0.3165	0.12582	-2.5154	0.01189	0.04402	3327.24	2913.11	3785.16	2574.17	2773.12	2703.52	Down
Tmem62	1937.64	0.36585	0.10227	3.5773	0.00035	0.00226	1831.48	1610.83	1637.62	2248.64	2101.49	2195.8	Up
Tubgcp4	450.817	0.45851	0.22416	2.0455	0.04081	0.11632	455.693	270.229	413.464	452.008	535.405	578.105	Up
Map1a	570.752	-0.3587	0.14653	-2.4478	0.01437	0.05131	725.626	565.373	633.087	519.395	477.323	503.706	Down
Ppip5k1	2229.34	0.4059	0.10402	3.90204	9.54E-05	0.00073	1946.61	2026.72	1779.9	2595.94	2640.06	2386.82	Up
Ckmt1	7532.11	2.53335	0.20437	12.396	2.75E-35	8.71E-33	2248.47	2534.59	1873.48	13220.2	16000.9	9315.04	Up
Strc	49.9789	2.38902	0.50333	4.74638	2.07E-06	2.38E-05	23.22	18.2069	6.68418	91.231	55.9693	104.562	Up
Pdia3	34601.2	0.38885	0.06909	5.62842	1.82E-08	3.12E-07	30354.4	29209.6	30334.7	39537.2	39310.5	38860.8	Up
Frmd5	735.56	-3.7475	0.75972	-4.9327	8.11E-07	1.03E-05	468.271	2772.24	867.034	91.231	92.9302	121.654	Down
Spg11	1256.86	0.40257	0.12449	3.2338	0.00122	0.0066	1222.92	1024.38	1000.72	1380.91	1417.19	1495.03	Up
Patl2	767.591	0.61735	0.16154	3.82162	0.00013	0.00097	654.998	622.868	539.509	831.446	1095.1	861.629	Up
Duox1	6.94697	2.64571	1.09947	2.40636	0.01611	0.05622	0	1.91652	3.81953	8.29373	10.5603	17.0918	Up
Shf	121.336	-0.53	0.23377	-2.2673	0.02337	0.07555	133.515	138.948	157.556	98.488	114.051	85.4591	Down
Gatm	348.265	1.71391	0.23074	7.42776	1.10E-13	4.64E-12	198.338	171.528	118.405	494.513	479.436	627.37	Up
AA467197	145.595	2.17508	0.31086	6.99693	2.62E-12	8.76E-11	47.4076	60.3703	50.6088	297.537	155.236	262.41	Up
Cep152	397.658	0.32073	0.15342	2.09055	0.03657	0.10692	357.008	341.14	362.855	388.768	443.531	492.646	Up
Secisbp2l	10723.9	0.76804	0.10026	7.66083	1.85E-14	8.61E-13	7820.31	7509.87	8474.59	12552.6	13600.6	14385.3	Up
Galk2	2947.48	0.3284	0.10412	3.15394	0.00161	0.00831	2678.04	2792.37	2370.02	3399.39	3197.64	3247.44	Up
Dtwd1	514.781	-0.4102	0.12646	-3.2434	0.00118	0.00641	546.638	595.079	620.674	446.825	435.082	444.387	Down
Atp8b4	417.737	0.6408	0.16654	3.84782	0.00012	0.00089	307.665	361.264	310.337	464.449	587.15	475.555	Up
Slc27a2	768.552	1.86491	0.30573	6.09988	1.06E-09	2.34E-08	244.778	515.543	232.991	1196.37	956.759	1464.87	Up
Hdc	12824.2	1.0939	0.13036	8.39135	4.81E-17	3.20E-15	7270.77	8995.17	8281.7	15345.5	19080.3	17971.5	Up
Gabpb1	2962.39	0.46387	0.12482	3.71641	0.0002	0.00141	2442.94	2421.52	2605.88	3399.39	3890.4	3014.19	Up
Usp50	79.8759	1.24675	0.33938	3.67366	0.00024	0.00162	41.6026	52.7042	47.7441	153.434	100.322	83.4483	Up
Sppl2a	8512.35	0.33597	0.09109	3.68856	0.00023	0.00154	7502.01	7602.82	7471.96	8824.53	10173.7	9499.03	Up
Blvra	1557.41	0.73078	0.14928	4.89545	9.81E-07	1.21E-05	1260.65	1140.33	1112.44	2034.04	2194.42	1602.61	Up
1810024B	66.4805	-0.9836	0.29185	-3.3704	0.00075	0.00437	87.0751	84.3268	93.5785	45.6155	38.0169	50.27	Down

Tmem127	5369.47	0.3178	0.09778	3.25011	0.00115	0.00629	5154.85	4511.48	4675.11	5645.95	6118.61	6110.83	Up
Kcnip3	206.037	-1.0949	0.18943	-5.7799	7.48E-09	1.39E-07	267.998	316.225	257.818	138.92	123.555	131.708	Down
Prom2	204.852	-1.9324	0.35826	-5.3939	6.90E-08	1.06E-06	295.088	311.434	367.63	89.1576	40.129	125.675	Down
Mal	3555.29	1.35054	0.11317	11.9342	7.85E-33	2.04E-30	2244.6	1942.39	1821.92	4958.61	5297.02	5067.22	Up
Mall	894.868	0.55299	0.18973	2.91467	0.00356	0.01629	640.486	870.099	665.553	1075.07	1250.33	867.661	Up
Mtln	746.798	-0.2835	0.13079	-2.1675	0.0302	0.09222	777.871	904.596	777.275	724.664	658.96	637.424	Down
Acoxl	1600.3	-1.7258	0.32819	-5.2583	1.45E-07	2.10E-06	2371.35	2437.81	2563.86	684.232	362.217	1182.35	Down
Bcl2l11	2199	-0.5271	0.12905	-4.0847	4.41E-05	0.00037	2501.96	2341.98	2944.86	1799.74	1931.47	1673.99	Down
Zc3h8	573.34	-0.3972	0.16492	-2.4082	0.01603	0.05597	632.746	615.202	707.568	477.926	423.466	583.133	Down
Vinac1	62.5588	-0.7457	0.29631	-2.5164	0.01185	0.04393	70.6276	82.4102	82.1199	42.5053	45.4091	52.2808	Down
Ttl	2201.1	-0.4085	0.10779	-3.7897	0.00015	0.00109	2435.2	2348.69	2748.15	1937.62	1800.52	1936.4	Down
Polr1b	1154.4	-0.2852	0.10851	-2.6284	0.00858	0.03368	1229.69	1213.16	1361.66	1009.76	1023.29	1088.85	Down
AI847159	51.3497	1.40562	0.37774	3.72117	0.0002	0.00138	22.2525	29.706	32.466	93.3044	55.9693	74.3997	Up
Gm4430	32.3194	-1.0137	0.42517	-2.3842	0.01712	0.05902	42.5701	44.0799	42.9697	19.6976	28.5127	16.0864	Down
Ckap2l	180.932	1.29073	0.28845	4.47465	7.65E-06	7.77E-05	104.49	137.031	73.526	250.885	206.981	312.68	Up
Gm14023	12.7428	1.67435	0.76939	2.17619	0.02954	0.0908	4.83751	3.83303	9.54883	23.8845	25.3446	9.04861	Up
Il1a	15.1317	1.24779	0.62726	1.98929	0.04667	0.12927	12.5775	4.79129	9.54883	20.7343	19.0085	24.1296	Up
Il1b	2486.33	2.12184	0.5058	4.19501	2.73E-05	0.00024	1314.83	1043.54	428.742	3287.43	5387.84	3455.56	Up
Spcs2-ps	70.2384	0.64714	0.27892	2.32016	0.02033	0.06757	58.0501	51.746	54.4283	96.4146	82.37	78.4213	Up
Sirpa	8871.08	1.71943	0.18236	9.42888	4.15E-21	3.96E-19	5035.85	3858.91	3503.47	12988	16373.7	11466.6	Up
Pdyn	14.5189	3.01582	0.97094	3.10609	0.0019	0.00954	0	7.66607	1.90977	40.4319	19.0085	18.0972	Up
Ebf4	257.499	-0.4964	0.2359	-2.1041	0.03537	0.1041	362.813	218.483	322.75	202.16	239.718	199.069	Down
Cpxm1	958.592	0.41224	0.15955	2.58381	0.00977	0.03747	724.659	932.386	810.696	1220.21	933.526	1130.07	Up
Pced1a	882.785	-0.2661	0.11369	-2.3407	0.01925	0.06479	973.306	889.264	1029.36	795.161	796.243	813.369	Down
4930473A	239.156	-0.6043	0.21517	-2.8085	0.00498	0.02164	312.503	281.728	271.187	217.71	208.037	143.772	Down
Mrps26	1038.53	-0.33	0.14286	-2.31	0.02089	0.06897	1256.78	1075.17	1138.22	953.779	1018.01	789.24	Down
Lzts3	780.948	0.84156	0.1575	5.34311	9.14E-08	1.36E-06	556.313	573.039	549.058	1075.07	814.196	1118.01	Up
4930402H	4499.09	-0.2503	0.10466	-2.3916	0.01677	0.05811	4494.04	5148.72	5022.68	4098.14	3853.44	4377.52	Down
Gfra4	67.0106	-0.8215	0.40737	-2.0165	0.04374	0.12295	118.035	91.9928	46.7893	46.6522	43.297	55.297	Down
Adam33	781.735	0.46184	0.12398	3.72504	0.0002	0.00137	725.626	639.159	608.26	873.951	933.526	909.888	Up
Siglec1	992.39	2.68673	0.31404	8.5554	1.17E-17	8.53E-16	393.773	262.563	144.187	1574.77	2156.4	1422.64	Up
Hspa12b	4483.67	-0.708	0.16827	-4.2078	2.58E-05	0.00023	4298.61	5626.89	6761.53	3265.65	3439.47	3509.85	Down
1700037H	1701.32	-0.3847	0.1744	-2.2059	0.02739	0.0856	1468.67	2043.01	2268.8	1394.38	1462.6	1570.44	Down
Spef1	2709.26	-0.2666	0.11809	-2.2571	0.024	0.07712	2825.1	2843.15	3208.41	2581.42	2195.48	2601.98	Down
Ap5s1	1183.14	0.64227	0.15281	4.20296	2.63E-05	0.00023	887.199	1038.75	846.026	1487.69	1613.61	1225.58	Up
Adra1d	45.9828	-1.0102	0.48371	-2.0885	0.03675	0.10735	56.1151	54.6207	73.526	34.2116	44.3531	13.0702	Down
5330413P	286.054	-0.9867	0.2303	-4.2842	1.83E-05	0.00017	396.676	384.262	359.991	186.609	140.451	248.334	Down
Rassf2	4531.88	0.89642	0.13095	6.84574	7.61E-12	2.42E-10	3208.23	2981.14	3313.44	5349.45	5468.1	6870.91	Up
Gm14051	286.036	-1.1405	0.46335	-2.4615	0.01383	0.04983	314.438	626.701	239.676	151.361	83.426	300.615	Down
Gpcpd1	5986.25	0.64413	0.16215	3.97248	7.11E-05	0.00056	4772.68	5385.41	3856.77	6973.99	8461.93	6466.74	Up
Shld1	765.754	-0.7597	0.13644	-5.5682	2.57E-08	4.30E-07	872.686	1059.83	955.838	547.386	619.887	538.895	Down
Chgb	35.396	-1.8812	0.45433	-4.1406	3.46E-05	0.0003	51.2776	70.9111	44.8795	18.6609	10.5603	16.0864	Down
Bmp2	611.25	1.41544	0.22404	6.3179	2.65E-10	6.53E-09	375.391	291.311	333.254	757.839	1183.8	725.899	Up
Plcb4	3681.31	-0.3704	0.10619	-3.4886	0.00049	0.00303	4101.24	4510.52	3842.45	3045.87	3195.53	3392.22	Down
Ankef1	221.009	-1.7192	0.294	-5.8476	4.99E-09	9.67E-08	258.323	476.255	282.645	91.231	92.9302	124.67	Down
Snap25	38.7235	-2.3331	0.56125	-4.1569	3.23E-05	0.00028	43.5376	88.1598	62.0674	10.3672	22.1765	6.03241	Down
Slx4ip	539.794	0.55925	0.1488	3.75848	0.00017	0.00122	391.838	505.961	411.555	655.204	636.783	637.424	Up
Btbd3	4604.54	-0.3264	0.11538	-2.8288	0.00467	0.02048	4577.25	5236.88	5555.51	4321.03	3794.3	4142.25	Down
Rps19-ps	90.6718	-0.5536	0.27564	-2.0083	0.04462	0.1248	100.62	100.617	122.225	76.717	85.5381	58.3133	Down
Macrod2	395.259	-0.527	0.14077	-3.7434	0.00018	0.00129	485.686	436.008	478.396	319.308	307.303	344.852	Down
Bfsp1	237.545	1.60021	0.24171	6.62031	3.58E-11	1.02E-09	120.938	148.53	84.0297	333.823	398.122	339.825	Up
Dstn	39842.8	0.29636	0.0838	3.53642	0.00041	0.00258	35137.7	34684.2	37472.5	43830.3	45908.6	42023.7	Up
Rrbp1	13570.7	0.57693	0.08357	6.90382	5.06E-12	1.64E-10	10399.7	11182.9	11095.7	16464.1	16785.5	15496.2	Up
Snx5	7806.55	0.46958	0.11572	4.05791	4.95E-05	0.00041	7120.81	6559.28	5961.33	8994.55	9868.56	8334.77	Up
Mgme1	687.896	-0.3052	0.12153	-2.5114	0.01202	0.04442	821.409	741.692	718.072	641.727	608.271	596.203	Down
Sec23b	5885.07	0.38705	0.10151	3.81303	0.00014	0.001	4875.24	5285.75	5140.13	7029.97	6052.08	6927.21	Up
Dtd1	1003.2	-0.3306	0.11074	-2.9855	0.00283	0.0134	1114.56	1102	1136.31	820.042	951.479	894.807	Down
Rin2	10124.4	-0.3483	0.13496	-2.5811	0.00985	0.03772	9353.8	11925.5	12742.9	8620.29	9133.56	8970.19	Down
Ralgapa2	5951.05	1.05894	0.10124	10.4592	1.33E-25	1.90E-23	4211.53	3742	3626.65	7765	8456.65	7904.46	Up
Gm14114	5.69416	5.00695	1.52385	3.28572	0.00102	0.00566	0	0.95826	0	12.4406	13.7283	7.03781	Up
Kiz	3598.05	0.6923	0.13452	5.14662	2.65E-07	3.68E-06	2963.46	2857.53	2432.09	4414.34	3942.14	4978.75	Up
Xrn2	5377.1	0.23881	0.09687	2.46544	0.01368	0.04939	5305.78	4593.89	4899.5	5666.69	6068.98	5727.77	Up
Pax1	3.84074	-5.3379	2.32476	-2.2961	0.02167	0.07102	4.83751	18.2069	0	0	0	0	Down
9030622C	591.93	-0.4929	0.21443	-2.2988	0.02152	0.0706	682.089	587.413	806.876	566.047	362.217	546.938	Down
Foxa2	3339.24	0.25195	0.12749	1.97628	0.04812	0.13245	3348.52	2815.36	2981.14	3892.87	3815.42	3182.09	Up
Thbd	35471.6	-0.6854	0.08614	-7.9574	1.76E-15	9.40E-14	41857	43321	46051.1	27965.4	25831.4	27803.4	Down
Cd93	18287.1	-0.3422	0.13653	-2.5063	0.0122	0.04499	17465.3	20749.2	23122.5	15837.9	17745.5	14802.5	Down
Gzf1	2555.5	0.53858	0.08949	6.01809	1.76E-09	3.70E-08	2084.97	2057.38	2109.34	3020.99	3182.86	2877.46	Up
Cst8	255.254	-1.278	0.31437	-4.0651	4.80E-05	0.00039	422.798	281.728	380.043	125.443	100.322	221.188	Down
Cst9	22.1855	-1.1645	0.55731	-2.0895	0.03667	0.10714	26.1225	26.8312	39.1502	14.514	7.39218	19.1026	Down
Cstdc2	409.292	-0.554	0.19523	-2.8379	0.00454	0.02001	440.213	442.715	577.704	297.537	394.953	302.626	Down
Syndig1	7.42401	-2.6303	0.98521	-2.6698	0.00759	0.03047	6.77251	18.2069	13.3684	2.07343	2.11205	2.0108	Down
Zfp120	949.039	0.32396	0.11478	2.82235	0.00477	0.02086	793.351	918.97	816.425	1024.28	1084.54	1056.68	Up
Gm14121	349.875	0.37676	0.17037	2.2115	0.027	0.08459	311.535	345.931	255.909	413.65	379.113	393.112	Up
Cst7	72.2935	-0.6651	0.32323	-2.0577	0.03962	0.11365	103.523	69.9529	92.6236	49.7624	47.5211	70.3781	Down
Apmap	4148.89	0.23123	0.09848	2.34796	0.01888	0.06382	4053.83	3772.66	3624.74	4477.58	4747.89	4216.65	Up
Entpd6	1101.47	0.42665	0.09998	4.26722	1.98E-05	0.00018	942.346	968.799	908.094	1267.9	1303.14	1218.55	Up
Abhd12	4886.66	0.72561	0.17602	4.12238	3.75E-05	0.00032	4365.37	3657.67	3026.02	5998.44	7141.9	5130.56	Up
Gins1	155.175	0.68228	0.22953	2.97252	0.00295	0.01388	147.06	109.241	101.218	179.352	190.085	204.096	Up
Ninl	590.293	0.80635	0.16825	4.7927	1.65E-06	1.94E-05	511.808	367.971	408.69	774.427	795.187	683.673	Up
Gm14150	137.898	-0.5408	0.25956	-2.0834	0.03722	0.10836	130.613	206.026	153.736	117.149	98.2104	121.654	Down
Nsfl1c	3942.66	-0.204	0.09775	-2.0866	0.03692	0.10777	3973.53	4204.84	4484.13	3563.19	3887.23	3543.03	Down
Fkbp1a	21578.6	-0.3277	0.13639	-2.4024	0.01629	0.05671	20877.7	23904.7	27273.4	19162.7	21074	17179.3	Down

Sdcbp2	435.974	0.43093	0.21589	1.99609	0.04592	0.12762	297.99	456.131	359.991	485.183	421.354	595.197	Up
Tmem74b	10.6932	-2.3805	0.84138	-2.8292	0.00467	0.02046	20.3175	22.0399	11.4586	6.2203	2.11205	2.0108	Down
Rspo4	67.2798	-1.3384	0.4864	-2.7516	0.00593	0.02494	119.97	55.579	113.631	40.4319	55.9693	18.0972	Down
Angpt4	67.9397	-2.3949	0.44733	-5.3538	8.61E-08	1.29E-06	73.5301	179.194	89.759	24.8812	22.1765	18.0972	Down
Slc52a3	2126.5	0.75966	0.15753	4.82236	1.42E-06	1.69E-05	1759.89	1449.85	1527.81	2875.85	2976.94	2168.65	Up
Tcf15	31.8242	-1.5485	0.51792	-2.9898	0.00279	0.01323	56.1151	60.3703	25.7818	18.6609	17.9524	12.0648	Down
Tbc1d20	6734.59	0.42718	0.10054	4.24873	2.15E-05	0.00019	5947.23	5469.74	5817.15	8041.8	8102.88	7028.76	Up
Defb20	12.2049	-1.7715	0.71184	-2.4887	0.01282	0.04684	15.48	17.2487	23.8721	6.2203	7.39218	3.0162	Down
H13	8218.87	0.324	0.09719	3.33384	0.00086	0.00488	7350.11	7197.48	7351.64	9524.31	9617.22	8272.44	Up
Id1	6813.21	-0.5788	0.15153	-3.8199	0.00013	0.00098	7652.94	8969.3	7863.46	6281.46	5679.3	4432.81	Down
Cox4i2	2252.78	-0.6796	0.16676	-4.0755	4.59E-05	0.00038	2449.71	2676.42	3195.04	1614.17	2078.26	1503.07	Down
Bcl2l1	8172.73	-0.4048	0.09632	-4.2021	2.64E-05	0.00023	8701.71	10056.9	9176.42	7321.29	6983.5	6796.51	Down
Tpx2	232.265	1.28041	0.24027	5.32916	9.87E-08	1.46E-06	150.93	161.946	93.5785	342.116	305.191	339.825	Up
Foxs1	123.122	-0.9629	0.30362	-3.1713	0.00152	0.0079	113.198	177.278	197.661	87.0841	97.1543	66.3565	Down
Ttll9	640.776	-1.0148	0.24988	-4.0611	4.88E-05	0.0004	650.161	995.631	926.236	462.375	291.463	518.787	Down
Pdrg1	3236.23	-0.2799	0.08595	-3.2571	0.00113	0.00617	3417.22	3509.14	3721.18	2966.04	2949.48	2854.33	Down
Ccm2l	4539.95	-0.4276	0.11899	-3.5938	0.00033	0.00213	4606.27	5150.64	5866.8	3722.85	3936.86	3956.25	Down
Hck	3136.05	0.80794	0.15624	5.17125	2.33E-07	3.25E-06	2709.97	2084.21	2046.31	3754.98	4565.2	3655.64	Up
Tm9sf4	5100.22	0.49501	0.09389	5.27236	1.35E-07	1.95E-06	4366.33	4296.83	4038.2	5599.3	5968.66	6332.01	Up
Kif3b	2863.02	0.33109	0.12475	2.65399	0.00795	0.03167	2726.42	2520.22	2361.43	2972.26	2985.38	3612.41	Up
Asxl1	3838.86	0.20552	0.1031	1.99331	0.04623	0.12827	3721.01	3293.53	3682.98	3965.44	4408.91	3961.28	Up
Bpifa2	35.084	1.06507	0.44825	2.37608	0.0175	0.06001	12.5775	25.873	29.6014	45.6155	48.5772	48.2592	Up
Bpifa3	11.0919	5.07533	1.65523	3.06625	0.00217	0.01066	0	1.91652	0	6.2203	2.11205	56.3024	Up
Bpifb5	251.914	-2.0984	0.50429	-4.1612	3.17E-05	0.00027	334.756	507.877	382.908	58.0561	34.8488	193.037	Down
Snta1	1061	-0.9501	0.18856	-5.0387	4.69E-07	6.21E-06	1212.28	1690.37	1291.96	835.593	746.61	589.165	Down
E2f1	471.982	0.29124	0.14589	1.99625	0.04591	0.12759	433.441	432.175	407.735	518.358	578.702	461.479	Up
Pxmp4	2109.6	0.27421	0.11738	2.336	0.01949	0.06547	2104.32	1943.35	1681.55	2325.35	2334.87	2268.18	Up
Chmp4b	10412.9	0.42592	0.09512	4.47777	7.54E-06	7.67E-05	8960.03	8865.81	8834.58	12236.4	12708.2	10872.4	Up
Pigu	3831.52	0.79462	0.08183	9.71089	2.71E-22	2.91E-20	2828.01	2832.61	2746.24	4762.67	4757.39	5062.19	Up
Trp53inp2	14603.7	0.72107	0.16902	4.26623	1.99E-05	0.00018	9328.65	14000.2	9756.04	16846.6	19148.9	18541.6	Up
Ggt7	179.022	-0.6947	0.24871	-2.7933	0.00522	0.02247	163.508	272.145	228.217	145.14	140.451	124.67	Down
Acss2	1861.84	-0.7817	0.15373	-5.0848	3.68E-07	4.99E-06	2369.41	2765.53	1927.91	1372.61	1297.86	1437.72	Down
Myh7b	21.9243	-1.8919	0.59598	-3.1744	0.0015	0.00784	22.2525	51.746	29.6014	11.4039	9.50423	7.03781	Down
Ergic3	7765.38	0.26519	0.09592	2.76465	0.0057	0.02417	7440.09	6843.88	6877.07	9135.54	8183.14	8112.58	Up
Spag4	36.7538	-1.3166	0.49105	-2.6811	0.00734	0.02965	43.5376	36.4138	77.3455	25.9179	23.2326	14.0756	Down
Epb41l1	3209.58	0.65853	0.10892	6.04579	1.49E-09	3.19E-08	2732.22	2478.06	2258.3	3872.13	4095.27	3821.53	Up
Myl9	4288.71	-1.609	0.27467	-5.858	4.68E-09	9.16E-08	3528.48	8849.52	7001.2	2051.66	2312.7	1988.68	Down
Rab5if	1933.06	0.23202	0.10758	2.15676	0.03102	0.09408	1949.52	1685.58	1698.74	2124.23	2140.56	1999.74	Up
Rbl1	1284.41	0.62009	0.12545	4.94305	7.69E-07	9.77E-06	1149.39	961.133	927.191	1500.13	1606.21	1562.39	Up
Rpn2	18517.5	0.38795	0.07038	5.51245	3.54E-08	5.74E-07	16078.9	16344.1	15705	21347	20747.7	20882.2	Up
Src	3080.23	0.43843	0.12459	3.51911	0.00043	0.00274	2411.01	2443.56	2992.6	3379.69	3765.79	3488.74	Up
Blcap	3127.77	0.38828	0.09182	4.22871	2.35E-05	0.00021	2563.88	2713.79	2850.33	3574.6	3650.68	3413.34	Up
Platr27	9.2629	3.11096	0.91969	3.38262	0.00072	0.00422	1.935	1.91652	1.90977	19.6976	20.0645	10.054	Up
Tgm2	1547.08	0.41626	0.16986	2.45059	0.01426	0.05104	1560.58	1034.92	1380.76	1767.6	1900.85	1637.8	Up
Lbp	8863.58	1.1906	0.17577	6.77366	1.26E-11	3.89E-10	5398.66	6642.65	4160.42	12234.3	11274.1	13471.4	Up
Snhg11	6711.3	0.99871	0.09331	10.7031	9.85E-27	1.55E-24	4749.46	4445.36	4235.86	8454.42	9185.31	9197.41	Up
Ralgapb	5277.98	0.33309	0.09224	3.61104	0.0003	0.00201	4679.8	4465.49	4868.95	5539.17	5922.19	6192.26	Up
Actr5	813.895	-0.2604	0.12205	-2.1337	0.03286	0.09824	883.329	816.436	961.567	753.692	764.562	703.781	Down
Mafb	929.597	1.18247	0.19575	6.04078	1.53E-09	3.27E-08	681.121	610.411	414.419	1397.49	1192.25	1281.89	Up
Chd6	3751.24	-0.2189	0.07864	-2.7835	0.00538	0.02301	4002.55	4129.14	3974.22	3511.36	3380.34	3509.85	Down
Ptprt	229.311	0.95094	0.35264	2.69664	0.007	0.02855	110.295	211.775	147.052	356.63	171.076	379.036	Up
L3mbtl1	30.6577	1.42297	0.48432	2.93807	0.0033	0.01528	23.22	9.58259	17.1879	37.3218	44.3531	52.2808	Up
Tox2	679.309	-0.7399	0.18383	-4.0249	5.70E-05	0.00046	717.886	773.315	1058.01	516.284	543.853	466.506	Down
Jph2	1276.94	-1.8514	0.4984	-3.7147	0.0002	0.00141	1253.88	3368.28	1376.94	617.883	630.447	414.225	Down
Gdap1l1	20.1889	1.68053	0.71519	2.34978	0.01878	0.06363	5.80501	19.1652	3.81953	31.1015	40.129	21.1134	Up
Fitm2	2618.17	-0.3971	0.1517	-2.6179	0.00885	0.03451	2983.77	3525.43	2419.67	2272.48	2211.32	2296.34	Down
Hnf4a	151.962	3.5438	0.45349	7.81444	5.52E-15	2.80E-13	27.09	14.3739	30.5563	341.08	125.667	373.004	Up
Pkig	3501.75	-0.5368	0.12062	-4.45	8.59E-06	8.58E-05	3790.67	4317.91	4328.48	3092.52	2946.31	2534.62	Down
Ada	251.855	-1.3323	0.27397	-4.863	1.16E-06	1.40E-05	249.615	340.182	491.765	158.618	136.227	134.724	Down
Ccn5	2195.72	0.49662	0.22909	2.16782	0.03017	0.09218	2375.22	1661.62	1427.55	2418.66	3247.28	2043.98	Up
Rims4	9.85604	-2.6169	0.99235	-2.6371	0.00836	0.03299	7.74001	33.5391	9.54883	2.07343	4.2241	2.0108	Down
Slpi	3114.93	1.74195	0.50623	3.44101	0.00058	0.00351	2513.57	945.801	842.207	4779.26	4889.4	4719.35	Up
Matn4	4865.39	0.77851	0.1401	5.557	2.74E-08	4.56E-07	3550.73	3025.22	4174.75	6033.69	6549.47	5858.47	Up
Rbpjl	5215.59	1.41632	0.10521	13.462	2.62E-41	1.49E-38	2573.55	2937.06	3018.38	7976.49	7206.32	7581.73	Up
Sdc4	11919.8	0.6544	0.10783	6.06876	1.29E-09	2.81E-08	9357.67	8759.44	9668.19	14732.8	15877.3	13123.5	Up
Dbndd2	526.776	-0.4169	0.17533	-2.3778	0.01742	0.05977	553.411	542.374	711.388	400.172	444.587	508.733	Down
Pigt	4123.32	0.41016	0.08181	5.01376	5.34E-07	6.99E-06	3482.04	3709.42	3431.85	4664.18	4733.11	4719.35	Up
Gm14317	119.185	-0.9696	0.23169	-4.1848	2.85E-05	0.00025	159.638	138.948	174.744	76.717	87.6501	77.4159	Down
Wfdc2	62445.7	0.99802	0.15112	6.60425	4.00E-11	1.13E-09	37804.2	47070.6	40130.9	99402.4	75105.6	75160.8	Up
Spint3	19.9176	2.22204	0.93731	2.37065	0.01776	0.06078	3.87001	3.83303	13.3684	31.1015	62.3055	5.027	Up
Wfdc6a	74.156	-1.2121	0.33702	-3.5964	0.00032	0.00212	84.1726	85.285	141.323	45.6155	43.297	45.243	Down
Eppin	62.3644	-1.9015	0.5838	-3.2571	0.00113	0.00617	59.0176	102.534	133.684	7.25701	27.4567	44.2376	Down
Wfdc8	258.105	-1.2214	0.19724	-6.1926	5.92E-10	1.35E-08	428.603	312.392	342.803	165.875	154.18	144.778	Down
Wfdc6b	75.6395	-1.4945	0.33623	-4.4449	8.79E-06	8.77E-05	101.588	93.9093	139.413	50.7991	36.9609	31.1674	Down
Wfdc16	17.5383	-1.4955	0.73749	-2.0279	0.04257	0.12032	23.22	30.6643	23.8721	4.14686	4.2241	19.1026	Down
Wfdc11	4.85688	-2.5863	1.23028	-2.1022	0.03554	0.10449	6.77251	11.4991	6.68418	2.07343	2.11205	0	Down
Wfdc3	500.754	1.10454	0.28901	3.82186	0.00013	0.00097	347.333	310.476	296.014	819.006	379.113	852.58	Up
Snx21	1632.68	-0.6749	0.14671	-4.5998	4.23E-06	4.55E-05	1658.3	2152.25	2212.46	1264.79	1337.98	1170.29	Down
Neurl2	384.872	-0.7745	0.21829	-3.5479	0.00039	0.00249	358.943	573.997	524.231	270.583	322.088	259.393	Down
Ctsa	12989.1	0.95502	0.14286	6.68496	2.31E-11	6.83E-10	10140.4	8787.23	7593.23	16664.2	19203.8	15545.5	Up
Pltp	20889.8	-0.4128	0.10287	-4.0132	5.99E-05	0.00048	24353.9	21444.9	25776.1	17983.9	18476.2	17304	Down
Zfp335	826.27	0.27974	0.13488	2.07395	0.03808	0.11028	774.969	650.658	813.56	870.841	957.815	889.78	Up
Mmp9	585.637	-1.2619	0.58094	-2.1722	0.02984	0.09151	1238.4	803.021	438.291	355.594	500.556	177.956	Down

Cd40	1021.04	-0.3246	0.14028	-2.3141	0.02066	0.06841	1091.34	1099.12	1215.57	851.144	1048.63	820.407 Down
Cdh22	134.281	-1.146	0.37379	-3.066	0.00217	0.01067	148.995	278.853	126.999	98.488	95.0423	57.3078 Down
Slc35c2	2135.72	0.40447	0.14426	2.80387	0.00505	0.0219	2021.11	1801.53	1692.05	2369.93	2815.36	2114.36 Up
Zfp334	810.609	-0.3007	0.14665	-2.0503	0.04034	0.1153	895.906	866.266	922.417	733.995	614.607	830.461 Down
Slc13a3	168.035	0.83971	0.30833	2.72337	0.00646	0.02672	164.475	92.9511	104.082	223.931	157.348	265.426 Up
Slc2a10	470.298	-0.5201	0.13729	-3.7884	0.00015	0.00109	517.613	562.498	582.479	401.209	354.825	403.166 Down
Eya2	4442.56	0.64482	0.10642	6.05918	1.37E-09	2.96E-08	3254.67	3730.5	3412.75	5649.06	4978.1	5630.24 Up
Sulf2	2873.87	0.40064	0.19871	2.01616	0.04378	0.12303	2637.41	3049.18	1745.53	3335.11	3464.82	3011.18 Up
Arfgef2	6213.19	0.64042	0.08925	7.17533	7.21E-13	2.64E-11	4878.14	4780.75	4910.01	7389.71	8120.84	7199.68 Up
Znfx1	4790.94	0.39093	0.09895	3.95096	7.78E-05	0.00061	4435.03	3879.99	4122.23	5293.47	5762.73	5252.21 Up
Ptgis	5441.8	-0.5663	0.22877	-2.4752	0.01332	0.04829	8335.99	5846.34	5306.28	5159.73	4896.79	3105.68 Down
B4galt5	3146.82	0.29279	0.1048	2.79389	0.00521	0.02244	2993.45	2625.63	2866.56	3410.8	3711.93	3272.58 Up
Gm14319	46.7385	-1.4246	0.40143	-3.5489	0.00039	0.00248	56.1151	77.619	70.6613	29.028	15.8404	31.1674 Down
Ptpn1	5437.12	0.43943	0.08894	4.94056	7.79E-07	9.88E-06	4567.57	4576.64	4701.84	5947.64	6692.03	6136.97 Up
Nfatc2	379.967	0.37408	0.14687	2.54704	0.01086	0.04094	352.171	321.017	319.886	440.604	389.673	456.452 Up
Atp9a	5794.21	0.56949	0.09205	6.18701	6.13E-10	1.40E-08	4871.37	4677.26	4446.89	7012.35	7245.39	6511.98 Up
Gm14249	17.0075	2.72307	0.78608	3.46411	0.00053	0.00327	1.935	5.74955	5.7293	11.4039	38.0169	39.2106 Up
Zfp217	1760.02	0.51446	0.08783	5.85779	4.69E-09	9.16E-08	1429	1438.35	1481.02	2066.17	2124.72	2020.86 Up
Bcas1	591.089	0.46499	0.19661	2.365	0.01803	0.06156	460.531	595.079	434.472	686.306	561.805	808.342 Up
Fam209	42.037	-1.0674	0.3836	-2.7825	0.00539	0.02306	42.5701	65.1616	63.0223	29.028	24.2886	28.1512 Down
Gm14303	65.3555	-0.8246	0.28373	-2.9063	0.00366	0.01665	82.2376	85.285	83.0748	43.5421	51.7452	46.2484 Down
Bmp7	152.951	-1.0175	0.21822	-4.6626	3.12E-06	3.46E-05	191.565	183.027	239.676	105.745	97.1543	100.54 Down
Rbm38	76.1899	-0.9675	0.29008	-3.3352	0.00085	0.00486	102.555	108.283	91.6688	61.1662	41.185	52.2808 Down
Ctcflos	13.8708	-3.1692	1.09536	-2.8933	0.00381	0.01726	14.5125	55.579	4.77441	2.07343	5.28013	1.0054 Down
Pck1	615.677	-7.0305	1.19256	-5.8953	3.74E-09	7.43E-08	356.041	3076.97	232.991	13.4773	10.5603	4.0216 Down
Zbp1	1023.39	1.71527	0.18451	9.29614	1.46E-20	1.34E-18	531.158	546.207	356.171	1436.89	1579.81	1690.08 Up
Ctsz	12543.9	1.31082	0.16941	7.73749	1.01E-14	4.90E-13	8877.79	6689.6	6054.91	17152.5	20422.5	16066.3 Up
Tubb1	203.085	-1.1925	0.34399	-3.4667	0.00053	0.00324	154.8	416.843	275.961	130.626	125.667	114.616 Down
Atp5e	2386.67	-0.3332	0.13116	-2.5405	0.01107	0.04156	2482.61	2759.78	2740.51	2290.11	2249.33	1797.66 Down
Gm2007	3.5454	-5.2221	2.54559	-2.0514	0.04023	0.11503	20.3175	0	0.95488	0	0	0 Down
Zfp973	36.5908	-0.8772	0.42756	-2.0517	0.04019	0.11497	56.1151	31.6225	54.4283	21.771	27.4567	28.1512 Down
Zfp970	1506.6	-0.6511	0.19432	-3.3508	0.00081	0.00464	2218.48	1549.5	1755.07	1083.37	988.44	1444.76 Down
Zfp972	419.722	-0.5621	0.17571	-3.199	0.00138	0.00731	553.411	514.585	433.517	381.511	291.463	343.847 Down
Gm14326	884.233	-0.2849	0.10754	-2.6488	0.00808	0.03207	1028.45	922.803	962.522	788.941	798.355	804.321 Down
Phactr3	19.8944	-1.3334	0.60403	-2.2075	0.02728	0.0853	29.025	39.2886	17.1879	9.33044	8.4482	16.0864 Down
Lsm14b	2702.13	-0.3506	0.1271	-2.7583	0.00581	0.02456	2788.34	3254.25	3044.17	2453.91	2077.2	2594.94 Down
Rps21	3214.87	-0.515	0.139	-3.705	0.00021	0.00146	3941.6	3274.37	4131.78	2661.25	2932.58	2347.61 Down
Slco4a1	409.155	0.93961	0.21139	4.44489	8.79E-06	8.77E-05	206.078	305.685	329.435	578.487	559.693	475.555 Up
Ogfr	2362.25	0.21495	0.10745	2.00036	0.04546	0.12654	2134.31	2026.72	2398.67	2610.45	2569.31	2434.08 Up
Slc17a9	684.806	0.64169	0.13097	4.89941	9.61E-07	1.19E-05	564.053	557.707	483.171	777.537	882.837	843.531 Up
Nkain4	866.062	-0.4601	0.19351	-2.3778	0.01741	0.05977	1253.88	813.562	941.515	780.647	780.403	626.365 Down
Eef1a2	916.006	-5.8813	1.02844	-5.7187	1.07E-08	1.93E-07	1046.84	3377.86	979.71	31.1015	4.2241	56.3024 Down
Arfrp1	2998.32	-0.2031	0.09358	-2.1705	0.02997	0.09175	3267.25	3031.93	3327.77	2868.59	2849.16	2645.21 Down
Zgpat	835.583	0.34152	0.11459	2.98042	0.00288	0.01357	755.619	681.322	774.41	936.154	964.151	901.845 Up
Slc2a4rg-[illegible]	804.915	-0.2744	0.12685	-2.1628	0.03055	0.093	853.336	835.602	954.883	712.224	682.192	791.25 Down
Zbtb46	2264.43	-0.5856	0.18327	-3.1953	0.0014	0.00738	2329.74	2612.21	3211.27	1473.17	2178.58	1781.57 Down
Dnajc5	6942.53	0.27137	0.09414	2.88254	0.00394	0.01776	6430.01	5797.46	6646.94	7414.59	7856.83	7509.34 Up
Zfp512b	399.899	0.40391	0.17555	2.3009	0.0214	0.07029	339.593	298.018	395.322	465.485	401.29	499.684 Up
Sox18	4627.7	-0.9233	0.16552	-5.5782	2.43E-08	4.07E-07	5383.18	5978.58	6817.86	3176.5	3809.08	2600.97 Down
Tcea2	395.563	-0.5301	0.15186	-3.4906	0.00048	0.00301	489.556	481.046	431.607	357.667	318.92	294.582 Down
Rgs19	1467.39	0.54903	0.13691	4.01021	6.07E-05	0.00049	1243.24	1135.54	1195.51	1711.62	2003.28	1515.14 Up
Pcmtd2	4472.25	-0.3329	0.0832	-4.0014	6.30E-05	0.0005	5123.89	4891.91	4942.47	3862.8	3867.16	4145.27 Down
Hnf4g	13.1333	2.04455	0.99329	2.05836	0.03956	0.11352	5.80501	9.58259	0	15.5507	12.6723	35.189 Up
Il7	451.969	0.38301	0.15224	2.51584	0.01188	0.04399	376.358	405.343	395.322	441.641	530.125	563.024 Up
Gm16685	149.489	1.09275	0.2551	4.28361	1.84E-05	0.00017	108.36	109.241	68.7516	182.462	205.925	222.194 Up
Stmn2	3429.59	-0.5953	0.14924	-3.9886	6.65E-05	0.00053	3900	3607.84	4873.72	2840.6	2928.36	2427.04 Down
Hey1	3778.79	-0.6685	0.13133	-5.0898	3.58E-07	4.87E-06	4313.12	4736.67	4867.04	3284.32	2499.61	2971.96 Down
Mrps28	765.233	-0.364	0.17027	-2.1376	0.03255	0.09743	742.074	936.219	905.229	671.792	774.067	562.019 Down
Tpd52	7570.64	0.64043	0.10302	6.21629	5.09E-10	1.18E-08	6421.31	6023.61	5307.24	9159.38	9245.5	9266.78 Up
C030034L	119.459	-0.8461	0.26391	-3.2059	0.00135	0.00716	134.483	165.779	160.42	75.6803	71.8097	108.583 Down
Pag1	2972.64	0.48621	0.14393	3.37797	0.00073	0.00427	2841.55	2283.53	2304.13	3138.14	3952.7	3315.81 Up
Fabp5	18092	2.98005	0.1814	16.428	1.21E-60	2.36E-57	4192.18	4368.7	3649.56	30781.1	24958.1	40602.1 Up
Gm38335	3.27215	5.21133	1.7925	2.90731	0.00365	0.01661	0	0	0	4.14686	8.4482	7.03781 Up
Fabp4	2608.06	-2.7255	0.72775	-3.7451	0.00018	0.00128	2618.06	9118.79	1856.29	831.446	759.282	464.495 Down
Fabp12	309.599	0.84352	0.1961	4.3014	1.70E-05	0.00016	254.453	224.233	186.202	393.952	345.32	453.436 Up
Car13	549.981	0.6907	0.20059	3.44329	0.00057	0.00349	467.303	475.296	319.886	640.69	612.495	784.213 Up
Car3	4363.54	-6.7931	0.86487	-7.8544	4.02E-15	2.06E-13	5843.71	16089.2	4014.33	52.8725	151.012	30.162 Down
Sirpb1a	270.93	1.74306	0.24655	7.06992	1.55E-12	5.44E-11	154.8	132.24	86.8943	398.099	481.548	371.998 Up
Sirpb1b	302.014	1.1224	0.34522	3.25131	0.00115	0.00628	258.323	206.026	105.992	377.365	560.749	303.631 Up
Sirpb1c	594.015	2.241	0.30433	7.36384	1.79E-13	7.24E-12	288.315	217.525	116.496	926.824	1218.65	796.277 Up
Gm5150	259.271	1.4452	0.42877	3.37055	0.00075	0.00437	227.363	133.198	57.293	344.19	510.06	283.523 Up
Gm5844	105.452	-0.5343	0.25561	-2.0902	0.0366	0.10701	115.133	133.198	126.045	75.6803	77.0899	105.567 Down
Bhlhe22	179.098	0.96467	0.23582	4.09068	4.30E-05	0.00036	125.775	138.948	99.3078	206.306	284.071	220.183 Up
Cyp7b1	579.211	0.75248	0.14037	5.3607	8.29E-08	1.25E-06	446.018	442.715	405.825	643.801	747.666	789.24 Up
Trim55	208.096	-4.1902	0.48015	-8.7267	2.62E-18	2.01E-16	223.493	723.485	236.811	22.8077	15.8404	26.1404 Down
4632415L(	1155.25	-0.3946	0.112	-3.5231	0.00043	0.0027	1264.52	1237.11	1435.19	1001.47	975.767	1017.47 Down
Cp	29975.5	0.99889	0.25615	3.89965	9.63E-05	0.00073	17998.4	23078.7	18904.8	44680.4	23234.7	51956.1 Up
Cpa3	395.999	-1.2559	0.4093	-3.0685	0.00215	0.01059	204.143	821.228	649.32	209.417	277.735	214.15 Down
Cpb1	118.029	-1.213	0.36117	-3.3585	0.00078	0.00454	97.7176	184.944	211.984	71.5334	88.7061	53.2862 Down
Agtr1b	27.1	-1.4105	0.52257	-2.6991	0.00695	0.02838	40.6351	44.0799	33.4209	7.25701	21.1205	16.0864 Down
7530428D	2.58131	-3.7006	1.84154	-2.0095	0.04448	0.12448	6.77251	5.74955	1.90977	0	1.05603	0 Down
Gm43672	217.632	-0.6521	0.24663	-2.6439	0.0082	0.03245	249.615	321.017	227.262	134.773	212.261	160.864 Down
Naaladl2	723.065	0.61138	0.17063	3.58298	0.00034	0.00221	597.916	646.825	471.712	923.714	757.17	941.055 Up

Nlgn1	238.411	-3.743	1.1329	-3.3039	0.00095	0.00536	25.155	702.404	603.486	42.5053	33.7928	23.1242	Down
Ect2	331.933	0.91448	0.18905	4.83725	1.32E-06	1.58E-05	235.103	244.356	211.029	434.384	357.993	508.733	Up
Nceh1	7162.15	0.90271	0.13783	6.54954	5.77E-11	1.59E-09	4865.56	5205.26	4904.28	9268.24	10883.4	7846.15	Up
Fndc3b	5504.35	0.64266	0.10597	6.06443	1.32E-09	2.87E-08	4109.95	4544.06	4240.63	6167.42	7320.37	6643.69	Up
Tmem212	5313.08	-0.4872	0.21118	-2.307	0.02105	0.06942	5434.46	6884.13	6286.95	5221.94	2993.83	5057.17	Down
Pld1	2323.69	0.88668	0.12305	7.20588	5.77E-13	2.17E-11	1689.26	1687.49	1517.31	2875.85	2769.95	3402.28	Up
Tnik	1202.79	-0.6825	0.13551	-5.0366	4.74E-07	6.28E-06	1373.85	1585.92	1486.75	831.446	888.117	1050.64	Down
Eif5a2	1374.24	0.90469	0.14979	6.03957	1.55E-09	3.29E-08	1140.68	817.395	912.868	1761.38	1733.99	1879.09	Up
Mecom	4725.07	0.30803	0.09296	3.31371	0.00092	0.0052	4241.53	4461.65	3964.67	5149.37	5067.87	5465.36	Up
Lrrc34	980.086	-0.6025	0.21756	-2.7692	0.00562	0.0239	1101.98	1381.81	1061.83	837.666	549.133	948.093	Down
Prkci	7628.25	-0.2227	0.10509	-2.1188	0.03411	0.10117	8958.1	7427.46	8261.65	6857.88	7336.21	6928.22	Down
Kcnmb2	1404.89	-0.7565	0.28933	-2.6145	0.00894	0.03477	1700.87	2207.83	1386.49	1117.58	602.99	1413.59	Down
Zmat3	1930.61	0.71831	0.13574	5.29191	1.21E-07	1.77E-06	1558.64	1561.96	1258.54	2163.63	2471.1	2569.8	Up
Zfp639	2181.4	-0.2602	0.11132	-2.3374	0.01942	0.06525	2297.82	2224.12	2610.65	1941.77	2107.83	1906.24	Down
Mfn1	3917.2	-0.3211	0.11938	-2.6901	0.00714	0.029	3998.68	4981.03	4074.49	3493.73	3396.18	3559.12	Down
Actl6a	2159.54	0.33598	0.09988	3.36393	0.00077	0.00447	1875.99	1789.07	2062.55	2357.49	2432.03	2440.11	Up
Usp13	500.053	-0.6183	0.26214	-2.3586	0.01834	0.0624	637.583	801.104	378.134	425.053	364.329	394.117	Down
Ttc14	4944.32	-0.2153	0.09331	-2.3076	0.02102	0.06936	4970.06	5425.66	5542.14	4749.2	4355.05	4623.84	Down
Dnajc19	626.97	-0.3015	0.11863	-2.5418	0.01103	0.04142	668.544	699.529	708.523	565.01	591.374	528.841	Down
Gm38509	18.2218	1.8306	0.6457	2.83505	0.00458	0.02017	7.74001	8.62433	7.63906	40.4319	13.7283	31.1674	Up
1700017M	3.86029	-3.3502	1.58675	-2.1114	0.03474	0.10265	3.87001	8.62433	8.59395	2.07343	0	0	Down
Atp11b	3962.7	0.24118	0.08508	2.83475	0.00459	0.02018	3699.73	3577.18	3619.96	4073.26	4337.1	4469.01	Up
Gm15952	368.206	-0.4808	0.19215	-2.5025	0.01233	0.04537	393.773	446.549	446.885	267.473	273.511	381.047	Down
Ccdc144b	3.67331	-4.2448	2.09334	-2.0278	0.04258	0.12033	0	7.66607	13.3684	0	0	1.0054	Down
Acad9	960.766	0.33102	0.09765	3.38991	0.0007	0.00413	848.499	843.268	861.304	1052.27	1083.48	1075.78	Up
Ccna2	261.935	0.70134	0.27246	2.57409	0.01005	0.03836	226.395	215.608	156.601	337.969	222.821	412.214	Up
Bbs7	1057.1	-0.5281	0.19353	-2.7289	0.00635	0.02639	1053.61	1407.68	1284.32	806.565	709.649	1080.81	Down
Trpc3	204.185	-0.6727	0.25511	-2.6367	0.00837	0.033	173.183	282.686	296.969	149.287	152.068	170.918	Down
Gm12531	8.95925	-1.5879	0.76948	-2.0636	0.03906	0.11241	14.5125	12.4574	13.3684	3.11015	5.28013	5.027	Down
Cetn4	1199.07	-0.7115	0.26577	-2.6773	0.00742	0.02991	1144.55	1721.03	1601.34	881.208	594.542	1251.72	Down
Gm20755	246.024	-1.4613	0.42067	-3.4737	0.00051	0.00317	189.63	429.3	464.073	88.1208	91.8742	213.145	Down
Ankrd50	3462.26	-0.3719	0.15507	-2.3981	0.01648	0.05726	3149.22	3944.19	4624.5	2934.94	3205.04	2915.66	Down
C230034O	30.567	-2.3465	0.69453	-3.3785	0.00073	0.00427	21.285	41.2051	90.7139	9.33044	15.8404	5.027	Down
Slc25a31	33.3109	-1.1286	0.47086	-2.3968	0.01654	0.05741	32.895	61.3286	42.9697	17.6242	16.8964	28.1512	Down
Hspa4l	7330.48	-0.3608	0.18138	-1.989	0.0467	0.12933	7006.65	8336.85	9383.63	5987.03	5273.79	7994.95	Down
Pgrmc2	4487.91	-0.3235	0.0954	-3.3913	0.0007	0.00411	4664.32	5280.01	5022.68	4188.33	3867.16	3904.98	Down
1700052H	11.3274	-2.1759	0.99796	-2.1804	0.02923	0.09012	11.61	39.2886	4.77441	4.14686	2.11205	6.03241	Down
Jade1	2469.74	-0.3599	0.0998	-3.6061	0.00031	0.00205	2572.59	2923.65	2832.18	2240.34	2177.52	2072.13	Down
Sclt1	881.182	-0.5426	0.19466	-2.7873	0.00531	0.0228	820.441	1066.54	1248.03	675.939	633.615	842.526	Down
D3Ertd751	1014.22	-0.4844	0.14813	-3.2701	0.00108	0.00592	1030.39	1198.78	1319.65	784.794	802.579	949.098	Down
Pcdh18	2118.99	-0.3198	0.16013	-1.9972	0.0458	0.12733	2091.74	2273.95	2692.77	1708.51	2238.77	1708.18	Down
Slc7a11	435.471	1.22737	0.30899	3.97218	7.12E-05	0.00056	394.741	162.904	224.397	524.578	570.254	735.953	Up
Gm6209	45.0985	3.23658	0.50708	6.38273	1.74E-10	4.45E-09	10.6425	11.4991	3.81953	94.3411	93.9863	56.3024	Up
Gm9442	13.5678	-1.2807	0.65316	-1.9608	0.0499	0.13661	26.1225	12.4574	19.0977	8.29373	7.39218	8.04321	Down
Ndufc1	787.97	-0.4052	0.14237	-2.8464	0.00442	0.01954	811.734	1033	848.891	696.673	699.089	638.43	Down
5031434O	276.278	-0.5259	0.15665	-3.3572	0.00079	0.00456	302.828	347.848	327.525	234.298	225.989	219.177	Down
Foxo1	3287.85	-0.2851	0.09474	-3.0098	0.00261	0.01251	3685.21	3738.17	3411.8	2791.88	3067.75	3032.29	Down
Gm42901	6.3268	-2.0893	1.04756	-1.9945	0.0461	0.12803	9.67501	16.2904	4.77441	1.03672	3.16808	3.0162	Down
Lhfp	7461.88	-0.3924	0.12222	-3.2107	0.00132	0.00707	7926.74	8554.37	8930.06	6507.47	7206.32	5646.33	Down
Nhlrc3	1911.98	0.68785	0.13925	4.93968	7.83E-07	9.91E-06	1603.15	1486.26	1304.37	2223.76	2689.7	2164.63	Up
Stoml3	103.486	3.57624	0.50231	7.11963	1.08E-12	3.87E-11	20.3175	13.4156	14.3232	203.196	77.0899	292.572	Up
6430500D	5.30783	5.91015	1.59874	3.69677	0.00022	0.0015	0	0	0	12.4406	6.33615	13.0702	Up
Frem2	103.551	-2.1135	0.33532	-6.3029	2.92E-10	7.15E-09	122.873	229.982	151.826	44.5788	34.8488	37.1998	Down
Gm30735	10.3209	-2.7038	1.03265	-2.6183	0.00884	0.0345	9.67501	26.8312	17.1879	6.2203	0	2.0108	Down
Gm10727	20.7975	-2.9483	0.80105	-3.6805	0.00023	0.00158	42.5701	50.7877	17.1879	2.07343	2.11205	10.054	Down
Exosc8	1390.26	-0.3329	0.11745	-2.8343	0.00459	0.02019	1637.98	1381.81	1629.99	1234.73	1276.73	1180.34	Down
Alg5	2024.45	0.20439	0.08336	2.45181	0.01421	0.0509	1861.47	1913.64	1868.71	2203.02	2155.35	2144.52	Up
Smad9	634.651	-0.644	0.15576	-4.1343	3.56E-05	0.0003	770.131	775.231	776.32	475.853	586.094	424.279	Down
Rfxap	1012.25	-0.3066	0.1401	-2.1887	0.02862	0.0887	1192.93	1002.34	1163.05	1018.05	818.42	878.72	Down
Ccna1	282.838	3.50394	0.36242	9.66815	4.12E-22	4.29E-20	61.9201	48.8712	26.7367	576.414	310.471	672.613	Up
Spg20	2576.04	-0.3685	0.11567	-3.1855	0.00144	0.0076	3123.09	2589.21	2997.38	2392.74	2147.96	2205.85	Down
Dclk1	274.343	0.85727	0.23624	3.62873	0.00028	0.0019	186.728	185.902	212.939	329.676	270.342	460.474	Up
Nbea	2242.36	0.22646	0.1136	1.99341	0.04622	0.12825	1875.02	2043.97	2281.22	2317.06	2420.41	2516.52	Up
Tm4sf1	17064.2	-0.3053	0.08631	-3.5376	0.0004	0.00257	18838.2	18076.6	19674.4	15258.4	16008.3	14529	Down
Tm4sf4	9.75055	2.03194	0.91612	2.21798	0.02656	0.08357	1.935	4.79129	4.77441	16.5875	5.28013	25.135	Up
Gm26671	757.999	-0.6512	0.14453	-4.5058	6.61E-06	6.81E-05	869.784	895.014	1014.09	600.258	515.34	653.511	Down
Ankub1	205.223	0.69388	0.30872	2.2476	0.0246	0.07866	135.45	223.274	111.721	289.244	191.141	280.507	Up
Rnf13	9941.53	0.41984	0.08429	4.98113	6.32E-07	8.19E-06	8980.35	8215.15	8319.89	10969.5	11552.9	11611.4	Up
Pfn2	1597.4	-0.9051	0.16208	-5.5845	2.34E-08	3.94E-07	2050.14	2326.65	1871.57	1035.68	965.207	1335.17	Down
Serp1	17180.3	0.35841	0.09729	3.68404	0.00023	0.00157	16083.7	14418.9	14668.9	20676.3	18221.7	19012.1	Up
Gm8234	38.3626	-1.3961	0.58413	-2.39	0.01685	0.05833	37.7326	70.9111	58.2479	19.6976	7.39218	36.1944	Down
4930593A	56.0143	-0.6687	0.32086	-2.0841	0.03715	0.10823	60.9526	75.7024	69.7065	40.4319	38.0169	51.2754	Down
Med12l	181.638	0.63919	0.20782	3.07572	0.0021	0.01038	119.97	164.82	141.323	202.16	232.326	229.231	Up
Gm5709	2.54082	4.84858	2.08652	2.32376	0.02014	0.06711	0	0	0	2.07343	2.11205	11.0594	Up
Sucnr1	11.705	-4.4413	1.17565	-3.7777	0.00016	0.00114	12.5775	41.2051	13.3684	2.07343	0	1.0054	Down
P2ry1	320.786	-1.1148	0.20606	-5.41	6.30E-08	9.75E-07	450.856	515.543	350.442	198.013	181.636	228.226	Down
Rap2b	525.498	0.5771	0.15549	3.71149	0.00021	0.00143	483.751	416.843	364.765	593.001	664.24	630.386	Up
Mme	12422.3	0.85507	0.10831	7.89462	2.91E-15	1.51E-13	9406.05	8572.58	8556.71	14309.8	17102.3	16586.1	Up
Plch1	1016.85	1.1686	0.15092	7.74296	9.71E-15	4.72E-13	560.183	715.819	602.531	1402.68	1253.5	1566.41	Up
Slc33a1	2973.55	0.23581	0.07819	3.0159	0.00256	0.0123	2710.94	2781.82	2700.41	3220.04	3240.94	3187.12	Up
Gm37359	159.665	-0.9763	0.35264	-2.7684	0.00563	0.02395	176.085	252.022	207.21	100.561	61.2495	160.864	Down
Veph1	174.835	2.10485	0.26266	8.01355	1.11E-15	6.16E-14	65.7901	77.619	54.4283	324.492	311.527	215.156	Up
Shox2	39.4487	-4.4734	0.9155	-4.8863	1.03E-06	1.27E-05	54.1801	146.614	25.7818	2.07343	0	8.04321	Down

Mfsd1	7063.39	0.71267	0.12043	5.91798	3.26E-09	6.56E-08	5936.59	5462.07	4661.74	8859.77	8291.91	9168.25	Up
Ift80	2604.08	0.24333	0.09152	2.65884	0.00784	0.03127	2304.59	2333.36	2517.07	2881.03	2713.99	2874.44	Up
Smc4	3608.5	0.38238	0.08912	4.29083	1.78E-05	0.00016	3261.45	2941.85	3195.99	3986.17	4092.1	4173.42	Up
Trim59	611.755	0.52128	0.20525	2.5397	0.01109	0.04161	652.096	424.509	430.652	731.921	789.907	641.446	Up
Arl14	3.95442	5.48483	1.87954	2.91817	0.00352	0.01614	0	0	0	16.5875	2.11205	5.027	Up
Ppm1l	1499.51	-0.4154	0.11364	-3.6558	0.00026	0.00172	1793.75	1629.04	1718.79	1345.66	1349.6	1160.23	Down
B3galnt1	634.653	-0.5257	0.17167	-3.0625	0.00219	0.01076	834.954	713.903	698.019	541.166	595.598	424.279	Down
Sptssb	1712.87	2.27076	0.28568	7.94863	1.89E-15	1.01E-13	782.709	502.128	479.351	3634.73	1701.26	3177.07	Up
Bche	717.622	-0.5848	0.22161	-2.6388	0.00832	0.03286	661.771	1144.16	777.275	535.982	653.68	532.862	Down
Fnip2	152.506	0.73346	0.27742	2.64388	0.0082	0.03245	115.133	104.45	124.135	132.7	194.309	244.312	Up
Ppid	3921.08	-0.2868	0.11367	-2.5229	0.01164	0.04331	3796.48	4494.23	4637.87	3532.09	3394.07	3671.72	Down
Etfdh	2950.63	-0.4512	0.21195	-2.1287	0.03328	0.09921	2977.97	4670.55	2576.27	2354.38	2580.93	2543.66	Down
Rxfp1	153.309	-5.7049	1.17339	-4.8619	1.16E-06	1.41E-05	130.613	667.906	104.082	2.07343	2.11205	13.0702	Down
Gask1b	3911.98	-0.3054	0.14715	-2.0756	0.03793	0.10996	4414.71	4610.18	3948.44	3420.13	4118.5	2959.9	Down
Pdgfc	206.243	0.87187	0.3709	2.35072	0.01874	0.0635	156.735	214.65	65.8869	277.84	262.95	259.393	Up
Tdo2	141.415	0.76869	0.28411	2.70558	0.00682	0.02793	100.62	95.8259	117.451	133.736	161.572	239.285	Up
Gucy1b1	7492.11	-0.3499	0.11128	-3.1447	0.00166	0.00854	7675.19	8692.36	8821.21	6032.65	7140.84	6590.4	Down
Gucy1a1	8633.22	-0.4411	0.1618	-2.7262	0.00641	0.02657	8140.56	10411.5	11276.2	6302.2	8361.61	7307.25	Down
Rbm46	211.802	-0.5351	0.25418	-2.1053	0.03527	0.10389	274.77	195.485	281.69	128.553	193.253	197.059	Down
Rbm46os	189.517	-1.0748	0.2107	-5.101	3.38E-07	4.61E-06	257.355	225.191	288.375	117.149	140.451	108.583	Down
Fgg	513.969	3.90663	0.9387	4.16172	3.16E-05	0.00027	25.155	144.697	22.9172	751.619	543.853	1595.57	Up
Fga	374.798	4.47389	1.21573	3.67999	0.00023	0.00159	7.74001	83.3685	5.7293	447.861	397.066	1307.02	Up
Sfrp2	147.162	1.75282	0.5826	3.00863	0.00262	0.01255	96.7501	73.7859	31.5111	431.274	174.244	75.4051	Up
Gm7115	35.3345	1.13174	0.54989	2.05813	0.03958	0.11357	39.6676	16.2904	10.5037	41.4686	52.8013	51.2754	Up
Tlr2	2899.73	1.98824	0.23383	8.50281	1.85E-17	1.30E-15	1629.27	1007.13	866.079	4350.06	5464.93	4080.92	Up
Mnd1	113.865	-0.7707	0.2971	-2.594	0.00949	0.03656	102.555	154.28	173.789	89.1576	95.0423	68.3673	Down
Fhdc1	2242.4	-0.8459	0.15959	-5.3005	1.15E-07	1.69E-06	2865.74	2742.54	3036.53	1978.05	1370.72	1460.85	Down
Gm37240	568.82	-0.3996	0.15519	-2.5752	0.01002	0.03827	539.866	716.777	684.651	477.926	489.996	503.706	Down
Lrba	3007.16	0.25737	0.11844	2.173	0.02978	0.09135	2793.18	2813.45	2612.56	3084.23	3027.62	3711.94	Up
Cd1d1	1023.05	0.51923	0.13462	3.85714	0.00011	0.00086	838.824	953.467	730.485	1175.64	1212.32	1227.59	Up
Kirrel	1110.37	-0.515	0.10314	-4.9933	5.93E-07	7.73E-06	1294.52	1274.48	1350.2	921.64	955.703	865.65	Down
Etv3	2884.36	0.27796	0.10215	2.72103	0.00651	0.02688	2694.49	2465.6	2662.21	3088.38	2981.16	3414.34	Up
Arhgef11	2236.68	0.58043	0.14821	3.91627	8.99E-05	0.00069	2160.43	1571.54	1646.22	2628.07	2825.92	2587.9	Up
Pear1	2526.95	-0.6837	0.0905	-7.5546	4.20E-14	1.89E-12	2998.29	3142.13	3203.63	2019.52	1944.14	1853.96	Down
Rrnad1	1276.93	-0.2889	0.11303	-2.5557	0.0106	0.04012	1420.29	1340.6	1452.38	1139.35	1060.25	1248.71	Down
Gm19439	635.89	0.42588	0.1293	3.29367	0.00099	0.00553	511.808	571.122	545.238	783.757	668.464	734.948	Up
Iqgap3	153.063	1.22695	0.3041	4.03474	5.47E-05	0.00044	118.035	98.7006	58.2479	215.637	177.412	250.345	Up
Rhbg	32.3967	-1.0655	0.45451	-2.3443	0.01906	0.06428	46.4401	40.2469	44.8795	29.028	12.6723	21.1134	Down
Glmp	5215.01	0.60555	0.0977	6.1982	5.71E-10	1.31E-08	4433.09	3971.98	4003.82	6373.73	6590.65	5916.78	Up
Tmem79	370.864	0.591	0.18507	3.19339	0.00141	0.00743	355.073	252.022	280.736	422.98	492.108	422.268	Up
Bglap3	368.319	5.75035	0.7471	7.69689	1.39E-14	6.58E-13	7.74001	26.8312	5.7293	957.925	381.225	830.461	Up
Slc25a44	1926.34	0.36004	0.10541	3.41575	0.00064	0.0038	1709.58	1764.15	1587.97	2003.97	2228.21	2264.16	Up
Sema4a	3964.01	1.42568	0.15869	8.98384	2.61E-19	2.14E-17	2329.74	2388.94	1733.11	5509.11	6535.74	5287.4	Up
Lmna	8475.2	0.46323	0.15245	3.03859	0.00238	0.01153	7486.53	6246.89	7644.79	9578.22	11575.1	8319.69	Up
Gm37584	32.7989	-0.9166	0.40622	-2.2564	0.02405	0.07724	42.5701	35.4556	50.6088	22.8077	23.2326	22.1188	Down
Rab25	2179.75	0.38178	0.11695	3.26451	0.0011	0.00603	1911.78	1992.22	1775.13	2706.87	2229.27	2463.23	Up
Ssr2	6819.01	0.33055	0.11011	3.002	0.00268	0.01278	5691.81	5977.62	6454.05	8311.35	7538.97	6940.28	Up
Dap3	3076.7	-0.2411	0.08877	-2.7163	0.0066	0.02716	3268.22	3397.99	3333.5	2981.59	2693.92	2784.96	Down
Fdps	147.748	1.96711	0.32606	6.03298	1.61E-09	3.42E-08	55.1476	88.1598	37.2404	208.38	270.342	227.221	Up
Pklr	505.926	3.7046	0.34916	10.6099	2.68E-26	4.10E-24	89.0101	81.452	45.8344	1014.94	516.396	1287.92	Up
Hcn3	36.1475	1.81654	0.50041	3.63007	0.00028	0.00189	9.67501	23.9565	14.3232	38.3585	60.1934	70.3781	Up
Gba	2998.27	0.62872	0.11365	5.53188	3.17E-08	5.22E-07	2522.28	2311.32	2231.56	3704.19	3932.64	3287.66	Up
Muc1	34411.1	1.88099	0.10102	18.6208	2.18E-77	9.40E-74	15440.4	15457.7	13187.9	53430.3	56173.2	52777.5	Up
Trim46	1042.14	1.42176	0.11194	12.7016	5.79E-37	2.19E-34	561.151	587.413	550.967	1410.97	1604.1	1538.26	Up
Dpm3	451.691	-0.5811	0.16937	-3.431	0.0006	0.00362	512.776	515.543	595.847	408.466	374.889	302.626	Down
Slc50a1	2988.85	-0.2964	0.11575	-2.5608	0.01044	0.03963	3158.89	3253.29	3471.95	2850.97	2833.32	2364.7	Down
Efna4	245.139	0.68899	0.21241	3.24373	0.00118	0.00641	214.785	181.111	167.105	327.602	337.928	242.302	Up
Zbtb7b	2810.75	0.42852	0.09834	4.35756	1.32E-05	0.00012	2561.94	2347.73	2279.31	3295.72	3324.37	3055.41	Up
Gm15417	102.765	-0.7936	0.31105	-2.5513	0.01073	0.04057	136.418	149.488	105.037	98.488	72.8658	54.2916	Down
Cks1b	431.088	0.35066	0.17395	2.01593	0.04381	0.12309	362.813	449.423	324.66	517.321	446.699	485.609	Up
Shc1	5193.93	0.35754	0.07714	4.63506	3.57E-06	3.91E-05	4663.36	4464.53	4532.83	5758.96	5994	5749.89	Up
Pbxip1	8367.28	0.65066	0.09823	6.62402	3.50E-11	9.96E-10	6601.26	6801.72	6132.26	9709.88	11031.2	9927.33	Up
Pmvk	5045.74	1.22131	0.20488	5.96108	2.51E-09	5.12E-08	3258.54	3550.35	2278.35	6124.92	6258.01	8804.3	Up
Kcnn3	1214.65	2.5064	0.25741	9.73719	2.09E-22	2.27E-20	520.516	314.309	255.909	1889.93	2497.5	1809.72	Up
Adar	637.479	0.78277	0.20014	3.91118	9.18E-05	0.0007	596.948	377.554	431.607	823.152	713.873	881.737	Up
4632404H	432.055	-0.3522	0.17486	-2.0145	0.04396	0.12339	563.086	465.714	424.923	334.859	386.505	417.241	Down
Ube2q1	6811.55	0.22479	0.10501	2.14067	0.0323	0.09688	6694.14	5882.75	6268.81	7462.28	7825.15	6736.19	Up
She	3639.32	-0.396	0.1501	-2.6385	0.00833	0.03288	3970.63	3522.56	4913.83	3086.3	3429.97	2912.65	Down
Il6ra	2728.14	-0.7646	0.11449	-6.6778	2.43E-11	7.16E-10	3748.1	3164.17	3391.74	2018.49	1879.73	2166.64	Down
Ubap2l	1072.91	0.46319	0.16796	2.75775	0.00582	0.02459	1079.73	711.028	915.733	1244.06	1228.16	1258.76	Up
1700094D	1863.93	-0.7109	0.22888	-3.106	0.0019	0.00954	2020.14	2686	2236.34	1505.31	945.143	1790.62	Down
Tpm3	10613.1	1.09968	0.24985	4.40134	1.08E-05	0.0001	7323.02	6547.78	6389.12	16047.3	18907.1	8464.47	Up
Rps27	6784.92	-0.409	0.13995	-2.9222	0.00348	0.01597	8390.17	6289.05	8541.43	5915.5	5960.21	5613.15	Down
Creb3l4	211.155	0.7307	0.21948	3.32917	0.00087	0.00495	179.955	157.154	139.413	250.885	222.821	316.701	Up
Slc39a1	4611.94	0.83153	0.12989	6.40189	1.53E-10	3.97E-09	3842.92	3048.22	3064.22	5684.31	6361.5	5670.46	Up
Chtop	8535.11	-0.2257	0.10207	-2.2114	0.02701	0.0846	8731.7	8930.01	9942.24	7918.44	8346.82	7341.44	Down
S100a1	7295.43	0.2571	0.12391	2.07484	0.038	0.11008	6185.24	7604.74	6151.36	8531.13	7486.16	7813.98	Up
S100a13	2061.48	-0.3724	0.13832	-2.692	0.0071	0.02889	2093.67	2182.91	2701.36	1847.43	1897.68	1645.84	Down
S100a14	913.358	1.437	0.22293	6.44586	1.15E-10	3.02E-09	614.363	407.26	456.434	1309.37	1662.18	1030.54	Up
S100a16	7448.46	-0.6149	0.16269	-3.7795	0.00016	0.00113	7765.17	8570.67	10700.4	6095.89	6595.93	4962.66	Down
S100a7a	72.1059	-0.7983	0.28416	-2.8093	0.00497	0.0216	100.62	78.5772	95.4883	47.6889	55.9693	54.2916	Down
Lor	35.773	-1.3704	0.46352	-2.9565	0.00311	0.01454	67.7251	46.9547	40.1051	12.4406	24.2886	23.1242	Down
Sprr1a	28.9756	2.70811	0.75131	3.60451	0.00031	0.00206	11.61	0.95826	10.5037	63.2397	64.4175	23.1242	Up

Ivl	16.9876	2.29009	0.84222	2.71912	0.00655	0.02699	6.77251	3.83303	6.68418	32.1382	46.4651	6.03241	Up
Gm4202	274.606	0.48575	0.18335	2.64935	0.00806	0.03202	223.493	251.064	211.984	292.354	296.743	371.998	Up
S100a11	26066.1	0.80204	0.09548	8.40004	4.46E-17	2.99E-15	18977.5	18416.8	19610.4	35989.6	32542.5	30859.8	Up
Them4	536.566	-0.3753	0.14471	-2.5936	0.0095	0.03658	548.573	651.616	617.809	428.164	461.483	511.749	Down
Them5	81.1106	1.33342	0.39705	3.35835	0.00078	0.00455	49.3426	50.7877	38.1953	91.231	78.1459	178.961	Up
C2cd4d	4.01421	-4.3763	1.60348	-2.7293	0.00635	0.02637	7.74001	8.62433	6.68418	1.03672	0	0	Down
Celf3	73.1939	-0.8774	0.29587	-2.9654	0.00302	0.01418	105.458	104.45	74.4809	50.7991	50.6892	53.2862	Down
Cgn	6589.12	0.5861	0.11601	5.05202	4.37E-07	5.84E-06	5815.65	4799.92	5190.74	7657.18	8596.05	7475.16	Up
Gm26279	2.56964	-3.6989	1.78092	-2.077	0.03781	0.10971	5.80501	3.83303	4.77441	0	0	1.0054	Down
Rfx5	298.225	0.80087	0.23367	3.42737	0.00061	0.00366	280.575	171.528	200.525	327.602	372.777	436.344	Up
Pi4kb	2559.66	0.23166	0.09069	2.5544	0.01064	0.04025	2443.91	2209.74	2410.12	2809.5	2752	2732.68	Up
Zfp687	1186.19	0.39678	0.12771	3.10689	0.00189	0.00952	1080.7	952.509	1038.91	1403.71	1449.92	1191.4	Up
Pip5k1a	2834.52	0.31764	0.0969	3.27802	0.00105	0.00578	2726.42	2375.52	2469.33	3086.3	3197.64	3151.93	Up
Tmod4	160.148	-0.5689	0.23224	-2.4497	0.0143	0.05111	162.54	237.648	173.789	124.406	127.779	134.724	Down
Scnm1	1644.44	-0.2325	0.0982	-2.3682	0.01788	0.06112	1707.64	1779.49	1842.92	1597.58	1452.03	1486.99	Down
Tnfaip8l2	536.788	1.1446	0.1696	6.74886	1.49E-11	4.55E-10	379.261	345.931	277.871	721.554	819.476	676.635	Up
Sema6c	201	-0.8891	0.27347	-3.251	0.00115	0.00628	290.25	304.726	188.112	176.242	133.059	113.61	Down
Cdc42se1	7451.89	0.30367	0.08467	3.58629	0.00034	0.00219	7021.16	6532.45	6457.87	8441.98	8337.32	7920.55	Up
Bnipl	540.247	0.27843	0.13978	1.99198	0.04637	0.12864	540.833	479.129	444.975	617.883	554.413	604.246	Up
Cers2	19269.7	0.45577	0.10524	4.33097	1.48E-05	0.00014	17543.7	14809.9	16399.2	23003.7	23096.3	20765.5	Up
Ctsk	5855.21	2.46109	0.20257	12.1494	5.78E-34	1.69E-31	2255.25	1880.1	1264.26	9875.76	10932	8923.94	Up
Ctss	17954.6	2.67845	0.25975	10.3118	6.23E-25	8.20E-23	7190.47	4342.83	3021.25	29935.2	32508.7	30729.1	Up
Golph3l	2854.54	0.35206	0.09466	3.71935	0.0002	0.00139	2548.4	2617.96	2357.61	3321.64	3059.31	3222.31	Up
Adamtsl4	4392.77	-0.5074	0.17125	-2.963	0.00305	0.01428	3868.07	5777.34	5826.7	3813.04	3407.79	3663.68	Down
Ecm1	6044.63	-0.4361	0.1572	-2.7741	0.00554	0.0236	6710.59	6351.34	7791.84	6109.37	5025.62	4278.99	Down
Rprd2	2189.38	0.27906	0.09045	3.08538	0.00203	0.0101	1952.42	2009.47	1972.79	2375.12	2528.12	2298.35	Up
C920021L	203.601	-0.5242	0.23789	-2.2034	0.02757	0.08603	211.883	233.815	275.006	172.095	126.723	202.086	Down
Car14	1177.01	-0.691	0.15652	-4.4148	1.01E-05	9.90E-05	1537.36	1258.19	1565.05	884.319	1035.96	781.196	Down
Anp32e	5629.03	-0.2545	0.10482	-2.4283	0.01517	0.05357	5628.92	5986.24	6757.71	4957.58	5214.65	5229.09	Down
Plekho1	4080.6	-0.4587	0.09087	-5.048	4.47E-07	5.95E-06	4484.37	4631.26	5056.1	3385.91	3434.19	3491.76	Down
Gm20627	207.796	-0.6475	0.32081	-2.0183	0.04356	0.12256	235.103	247.231	278.826	130.626	106.659	248.334	Down
Gm42743	203.268	-0.6668	0.29945	-2.2266	0.02597	0.0821	296.055	203.151	249.224	118.186	127.779	225.21	Down
Fcgr1	325.023	1.10643	0.193	5.73265	9.89E-09	1.79E-07	219.623	224.233	174.744	444.751	378.057	508.733	Up
Hjv	320.713	-5.9055	1.35525	-4.3575	1.32E-05	0.00012	341.528	1219.86	331.344	12.4406	0	19.1026	Down
Gm15441	77.4674	-0.9631	0.3592	-2.6813	0.00733	0.02964	83.2051	131.281	92.6236	59.0928	64.4175	34.1836	Down
Ankrd34a	9.59454	-1.7262	0.87304	-1.9773	0.04801	0.13224	18.3825	19.1652	6.68418	2.07343	4.2241	7.03781	Down
Itga10	162.722	-0.5189	0.21313	-2.4348	0.0149	0.05279	176.085	223.274	175.698	144.104	121.443	135.729	Down
Pias3	2121.46	-0.2941	0.09527	-3.0873	0.00202	0.01006	2401.34	2221.24	2388.16	1921.03	1984.27	1812.74	Down
Polr3c	1658.85	0.20487	0.0922	2.22195	0.02629	0.08292	1566.38	1560.05	1497.26	1827.73	1800.52	1701.14	Up
Rnf115	3417.98	0.29877	0.0853	3.50245	0.00046	0.00289	3148.25	3061.64	2985.92	3773.65	3919.97	3618.44	Up
Acp6	1429.4	-0.4066	0.09817	-4.1424	3.44E-05	0.00029	1580.9	1597.42	1710.2	1181.86	1270.4	1235.64	Down
Bcl9	1725.32	0.29537	0.11379	2.59576	0.00944	0.03643	1444.48	1610.83	1592.74	1997.75	1723.43	1982.65	Up
Olfr1402	31.412	-2.168	0.79807	-2.7165	0.0066	0.02715	93.8476	35.4556	24.827	21.771	10.5603	2.0108	Down
Fmo5	2048.99	-0.5296	0.1087	-4.8721	1.10E-06	1.35E-05	2661.6	2330.49	2270.71	1734.43	1614.66	1682.04	Down
Prkab2	725.766	-0.4463	0.13188	-3.3841	0.00071	0.0042	809.799	869.141	832.658	560.863	592.43	689.705	Down
Pde4dip	4080.86	-0.7504	0.28112	-2.6692	0.0076	0.03051	3587.5	8112.62	3656.25	3137.1	3135.34	2856.34	Down
Hmgcs2	2344	-2.7382	0.18229	-15.021	5.32E-51	4.79E-48	3483.97	4695.47	4051.57	756.803	512.172	564.03	Down
Phgdh	881.762	1.17705	0.19463	6.04763	1.47E-09	3.16E-08	648.226	576.872	397.231	1196.37	1333.76	1138.11	Up
Zfp697	1749.71	-0.5184	0.11563	-4.4835	7.34E-06	7.48E-05	2000.79	2145.54	2035.81	1281.38	1560.81	1473.92	Down
Hsd3b3	2.0786	-4.4523	2.03824	-2.1844	0.02893	NA	2.9025	5.74955	3.81953	0	0	0	Down
M6pr-ps	47.0668	0.90984	0.44419	2.04832	0.04053	0.11571	51.2776	22.0399	24.827	63.2397	49.6332	71.3835	Up
Man1a2	4318.26	0.30893	0.09868	3.13068	0.00174	0.00888	3979.33	3973.9	3619.96	4478.61	4777.46	5080.29	Up
Vtcn1	941.898	1.41879	0.28976	4.89639	9.76E-07	1.21E-05	318.308	732.11	487.945	1650.45	1034.9	1427.67	Up
Trim45	318.864	-0.5546	0.16068	-3.4518	0.00056	0.00339	331.853	403.427	402.961	261.252	250.278	263.415	Down
Ttf2	637.17	0.56149	0.12879	4.35969	1.30E-05	0.00012	514.711	562.498	466.938	741.252	783.571	754.051	Up
Cd2	1158.49	1.02128	0.28903	3.5335	0.00041	0.00261	1156.16	507.877	630.223	1472.14	1896.62	1287.92	Up
Atp1a1	47314.1	0.59814	0.10198	5.86519	4.49E-09	8.82E-08	36679.9	41550.1	34702.4	57754.4	54815.1	58382.6	Up
Mab21l3	59.2764	1.68444	0.46454	3.62602	0.00029	0.00191	24.1875	17.2487	42.9697	105.745	54.9133	110.594	Up
Casq2	1144.34	-5.4967	0.98341	-5.5895	2.28E-08	3.84E-07	1188.09	4468.36	1060.87	43.5421	12.6723	92.4969	Down
A230001N	39.5995	-1.2643	0.52458	-2.4102	0.01594	0.05577	34.8301	72.8277	60.1576	30.0648	10.5603	29.1566	Down
Ngf	22.3997	-3.175	0.60483	-5.2494	1.53E-07	2.20E-06	43.5376	42.1634	35.3307	3.11015	4.2241	6.03241	Down
Ampd1	41.0569	-2.3593	0.51762	-4.558	5.16E-06	5.44E-05	40.6351	102.534	63.0223	9.33044	13.7283	17.0918	Down
Olfml3	5253.55	-0.257	0.10351	-2.4833	0.01302	0.0474	5462.51	5675.77	6022.45	4573.99	5243.17	4543.41	Down
Hipk1	522.026	0.52836	0.21292	2.48152	0.01308	0.04756	506.003	360.305	416.329	541.166	531.181	777.175	Up
Bcl2l15	140.184	3.92799	0.52979	7.41429	1.22E-13	5.11E-12	17.415	13.4156	21.0074	183.499	493.164	112.605	Up
Ptpn22	743.92	0.7985	0.21734	3.67402	0.00024	0.00162	625.973	479.129	524.231	877.062	1229.21	727.91	Up
Magi3	653.854	0.8316	0.19405	4.28558	1.82E-05	0.00017	566.956	386.178	458.344	708.077	836.372	967.196	Up
Slc16a1	3315.37	0.93059	0.20039	4.64381	3.42E-06	3.76E-05	2718.68	2544.18	1582.24	4568.81	4525.07	3953.24	Up
Ppm1j	486.216	1.27911	0.24817	5.15419	2.55E-07	3.55E-06	226.395	354.556	270.232	726.738	842.708	496.668	Up
Capza1	4343.29	0.56607	0.093	6.08653	1.15E-09	2.53E-08	3596.2	3273.41	3636.19	5015.63	5424.8	5113.47	Up
Wnt2b	256.307	-0.5301	0.16908	-3.1351	0.00172	0.00877	331.853	301.851	275.006	204.233	201.701	223.199	Down
Kcnd3	141.051	-1.276	0.29165	-4.3749	1.21E-05	0.00012	161.573	255.855	181.428	69.46	105.603	72.3889	Down
Inka2	2462.55	0.84111	0.19007	4.42528	9.63E-06	9.47E-05	1721.19	1585.92	1986.16	2852.01	2567.2	4062.82	Up
Gm5547	96.0981	2.58033	0.31736	8.13054	4.27E-16	2.53E-14	29.025	34.4973	19.0977	188.682	139.395	165.891	Up
Tmigd3	45.1571	1.71212	0.35574	4.81288	1.49E-06	1.77E-05	18.3825	22.9982	21.9623	73.6068	68.6416	65.3511	Up
I830077J0	389.415	1.34436	0.22315	6.02439	1.70E-09	3.57E-08	257.355	236.69	166.15	463.412	657.904	554.981	Up
Atp5pb	13880.6	0.38166	0.11891	3.2097	0.00133	0.00709	11286.9	13878.5	11000.3	15908.4	15959.7	15249.9	Up
Pifo	1610.15	-0.6657	0.2484	-2.6798	0.00737	0.02975	1774.4	2290.24	1861.07	1333.22	777.235	1624.73	Down
Chil3	59837.7	4.36498	0.89167	4.8953	9.82E-07	1.21E-05	11396.2	3948.98	1271.9	107165	174262	60981.6	Up
Chil4	32.7736	3.65554	0.82495	4.43125	9.37E-06	9.26E-05	10.6425	3.83303	0	60.1295	91.8742	30.162	Up
Gm6522	58.8943	3.64894	1.39497	2.61579	0.0089	0.03468	24.1875	0.95826	0.95488	96.4146	180.58	50.27	Up
Chil6	20.3873	1.82748	0.81083	2.25383	0.02421	0.07763	11.61	3.83303	11.4586	51.8358	7.39218	36.1944	Up
Gm38244	81.1135	2.25276	0.36112	6.23829	4.42E-10	1.04E-08	18.3825	24.9147	41.06	150.324	139.395	112.605	Up

2010016I1	28.0123	1.80155	0.48687	3.70024	0.00022	0.00148	11.61	15.3321	10.5037	43.5421	54.9133	32.1728	Up
Dennd2d	2885.89	1.01793	0.11969	8.50469	1.82E-17	1.29E-15	1789.88	1956.76	1977.56	3729.07	3519.73	4342.33	Up
Cept1	7151.66	0.32443	0.08504	3.81504	0.00014	0.00099	6124.28	6283.3	6645.03	7650.96	8269.73	7936.63	Up
Dram2	9325.13	0.50274	0.09688	5.18935	2.11E-07	2.97E-06	7417.83	7884.55	7847.23	10433.5	11923.6	10444.1	Up
Cd53	4382.98	1.36308	0.16975	8.03012	9.74E-16	5.41E-14	2892.83	2384.15	2084.51	5921.72	7503.06	5511.61	Up
Gm27008	56.3803	1.69608	0.35011	4.84448	1.27E-06	1.52E-05	34.8301	22.9982	21.9623	92.2677	90.8182	75.4051	Up
Kcna2	550.363	-1.6255	0.18405	-8.8319	1.03E-18	8.07E-17	771.099	837.518	885.176	334.859	251.334	222.194	Down
Rbm15	1163.86	-0.2521	0.10703	-2.3555	0.0185	0.06282	1229.69	1220.82	1345.43	1102.03	1006.39	1078.8	Down
Slc6a17	51.5358	-2.0018	0.49688	-4.0286	5.61E-05	0.00045	48.3751	135.114	63.9772	18.6609	17.9524	25.135	Down
Ahcyl1	8323.94	0.2582	0.0708	3.64707	0.00027	0.00178	7440.09	7607.62	7695.4	9094.07	9043.8	9062.68	Up
Csf1	2458.42	0.51256	0.10616	4.82812	1.38E-06	1.64E-05	2173.98	1945.27	1959.42	2754.55	3115.27	2802.05	Up
Gstm5	456.465	-0.7543	0.16879	-4.4691	7.85E-06	7.92E-05	518.581	665.99	534.734	347.3	368.553	303.631	Down
Gstm7	371.327	-0.9449	0.25713	-3.6747	0.00024	0.00161	382.163	664.073	420.148	238.445	216.485	306.647	Down
Gstm6	348.938	-1.2595	0.24984	-5.0411	4.63E-07	6.14E-06	386.033	652.574	438.291	216.674	165.796	234.258	Down
Gm12494	18.4215	-1.3682	0.57837	-2.3656	0.018	0.06151	27.09	27.7895	24.827	13.4773	5.28013	12.0648	Down
Gstm3	70.6854	-1.1141	0.30702	-3.6287	0.00028	0.0019	113.198	86.2433	90.7139	50.7991	35.9049	47.2538	Down
Gstm2	10911.4	-0.9951	0.12863	-7.7362	1.02E-14	4.94E-13	15519.7	13600.6	14476	7565.95	6215.77	8090.46	Down
Gstm1	27320.1	-1.2035	0.12641	-9.5209	1.72E-21	1.72E-19	44298	33645.4	36349.5	17236.4	16150.9	16240.2	Down
Ampd2	1480.63	0.51203	0.12913	3.96522	7.33E-05	0.00058	1271.3	1164.28	1226.07	1632.83	1992.72	1596.58	Up
Gnai3	8687.55	0.3736	0.10448	3.57585	0.00035	0.00227	7727.43	6802.68	8176.66	9408.2	9979.44	10030.9	Up
Amigo1	1016.48	-0.5078	0.12199	-4.1624	3.15E-05	0.00027	1213.25	1094.33	1272.86	867.731	877.557	773.153	Down
Atxn7l2	385.56	-0.3232	0.1548	-2.0876	0.03684	0.10753	423.766	428.342	433.517	297.537	383.337	346.863	Down
Sypl2	42.4532	-2.8499	0.55326	-5.1511	2.59E-07	3.60E-06	58.0501	118.824	46.7893	14.514	9.50423	7.03781	Down
Sort1	24354.8	0.8552	0.13582	6.29664	3.04E-10	7.40E-09	20431.7	16356.5	15233.2	29826.3	33630.2	30650.7	Up
Mybphl	1216.14	-6.3068	1.67337	-3.7689	0.00016	0.00117	1349.66	4576.64	1279.54	16.5875	0	74.3997	Down
Psrc1	279.366	2.03967	0.27943	7.29933	2.89E-13	1.13E-11	151.898	104.45	71.6162	441.641	518.508	388.085	Up
Celsr2	1015.63	-0.2751	0.11478	-2.397	0.01653	0.05738	1022.65	1110.62	1203.15	928.897	936.694	891.791	Down
1700013F(	705.776	-0.8137	0.22983	-3.5403	0.0004	0.00255	713.049	1093.37	892.816	539.092	377.001	619.327	Down
Stxbp3	4419.18	0.20108	0.08801	2.28471	0.02233	0.07273	4142.84	3910.65	4281.69	4896.41	4543.02	4740.47	Up
Fndc7	494.004	1.73925	0.30959	5.61791	1.93E-08	3.30E-07	167.378	325.808	190.022	770.28	512.172	998.363	Up
Henmt1	24.085	1.66862	0.63757	2.61717	0.00887	0.03455	13.545	3.83303	17.1879	44.5788	42.241	23.1242	Up
Fam102b	2307.03	0.79499	0.17528	4.53566	5.74E-06	5.98E-05	1910.82	1597.42	1552.64	3023.06	3466.93	2291.31	Up
Gm19391	79.9904	-0.7579	0.31094	-2.4374	0.01479	0.05248	117.068	80.4937	104.082	45.6155	62.3055	70.3781	Down
Vav3	898.207	-0.264	0.11087	-2.3815	0.01724	0.05931	956.859	932.386	1051.33	813.822	798.355	836.494	Down
Amy1	731.841	-1.672	0.19002	-8.7991	1.38E-18	1.07E-16	887.199	1354.98	1100.03	308.941	375.945	363.955	Down
Rnpc3	1099.03	-0.2496	0.11783	-2.1181	0.03416	0.1013	1241.3	1073.25	1267.13	1001.47	969.431	1041.6	Down
S1pr1	15575	-0.6282	0.10486	-5.9911	2.08E-09	4.31E-08	17685	18404.3	20651.3	13135.2	11721.9	11852.7	Down
A930005H	301.111	-0.4773	0.17126	-2.7867	0.00532	0.02283	365.716	307.601	378.134	227.041	258.726	269.447	Down
Slc30a7	3913.28	0.71801	0.09009	7.96958	1.59E-15	8.61E-14	3109.55	2774.16	2993.56	4809.32	5001.34	4791.74	Up
Lrrc39	206.645	-1.7484	0.45295	-3.8601	0.00011	0.00085	179.955	597.953	177.608	88.1208	87.6501	108.583	Down
Sass6	154.082	0.51177	0.22358	2.28898	0.02208	0.07213	120.938	137.031	123.18	210.453	148.9	183.988	Up
Agl	1216.16	-0.4967	0.21542	-2.3056	0.02113	0.06962	1324.51	1887.77	1058.01	1033.61	1098.27	894.807	Down
Frrs1	475.105	1.28612	0.23549	5.46137	4.72E-08	7.53E-07	291.218	290.352	247.315	673.865	873.333	474.549	Up
Palmd	4812.15	-0.5437	0.12679	-4.2886	1.80E-05	0.00017	5750.83	5912.46	5461.93	3317.49	4296.97	4133.2	Down
4930455H	21.815	-1.4618	0.61221	-2.3877	0.01695	0.05862	39.6676	25.873	30.5563	13.4773	4.2241	17.0918	Down
Snx7	3777.94	1.67131	0.1245	13.4241	4.37E-41	2.42E-38	1963.06	1735.41	1717.83	5775.54	6378.39	5097.38	Up
Gm37928	4.23014	5.58379	1.87707	2.97474	0.00293	0.0138	0	0	0	3.11015	3.16808	19.1026	Up
Dpyd	431.484	-0.9248	0.2208	-4.1886	2.81E-05	0.00025	578.566	596.037	521.366	293.391	216.485	383.058	Down
Gm4332	332.039	-0.5316	0.16603	-3.202	0.00136	0.00725	435.376	353.597	388.637	244.665	291.463	278.496	Down
Ptbp2	2031.34	-0.366	0.11472	-3.1902	0.00142	0.0075	2212.68	2371.69	2278.35	1600.69	1963.15	1761.46	Down
Rwdd3	591.136	-0.3859	0.18833	-2.049	0.04047	0.11556	556.313	717.736	735.26	549.459	413.962	574.084	Down
Tlcd4	4631.27	1.55445	0.12288	12.6506	1.11E-36	4.04E-34	2542.59	2325.69	2189.55	6193.34	6824.04	7712.43	Up
Cnn3	9479.91	-0.5604	0.11743	-4.7719	1.83E-06	2.12E-05	10113.3	11035.3	12745.8	7289.15	8024.74	7671.21	Down
Slc44a3	5098.85	0.38082	0.10496	3.62812	0.00029	0.0019	4710.76	4375.41	4203.39	5754.81	5320.26	6228.46	Up
A530020G	1948.91	0.454	0.14247	3.18665	0.00144	0.00757	1715.38	1699.95	1519.22	2136.67	1980.05	2642.19	Up
A730020M	38.47	-1.1047	0.52992	-2.0845	0.03711	0.10814	53.2126	75.7024	28.6465	13.4773	30.6247	29.1566	Down
Abcd3	15846.3	1.14428	0.12781	8.95301	3.46E-19	2.82E-17	11304.3	9734.95	8576.76	21505.6	20738.2	23217.7	Up
Bcar3	3416.8	-0.4389	0.12855	-3.4144	0.00064	0.00382	3762.61	3476.56	4558.61	2980.56	2824.87	2897.57	Down
Fnbp1l	8498.99	-0.23	0.11072	-2.0771	0.0378	0.10969	8532.4	8634.87	10357.6	7611.57	7850.49	8007.01	Down
Pde5a	9117.22	-0.5281	0.15273	-3.4575	0.00055	0.00333	9037.43	10773.7	12490.8	6878.61	8502.06	7020.71	Down
4933405D	22.0605	-1.7862	0.57846	-3.0878	0.00202	0.01004	28.0575	41.2051	33.4209	7.25701	6.33615	16.0864	Down
Myoz2	1595.33	-5.9361	1.32021	-4.4964	6.91E-06	7.09E-05	1958.22	5544.48	1915.5	27.9913	2.11205	123.664	Down
Synpo2	1349.41	-1.6107	0.2624	-6.1385	8.33E-10	1.86E-08	1414.49	3019.47	1665.32	626.176	726.545	644.462	Down
Snhg8	327.804	-0.6218	0.22134	-2.8094	0.00496	0.02159	359.911	359.347	472.667	262.289	311.527	201.08	Down
Ank2	212.426	-1.1684	0.44467	-2.6275	0.0086	0.03373	203.175	514.585	164.24	124.406	182.692	85.4591	Down
Tifa	1325.78	0.65957	0.19063	3.45997	0.00054	0.00331	977.176	1142.24	964.432	1621.42	1236.61	2012.81	Up
Ap1ar	1.88621	4.41731	2.12293	2.08076	0.03746	NA	0	0	0	5.18358	2.11205	4.0216	Up
Fam241a	1563.82	0.56572	0.14271	3.96412	7.37E-05	0.00058	1250.98	1274.48	1257.58	2049.59	2018.06	1532.23	Up
Pitx2	39.8362	-4.7974	0.79776	-6.0136	1.81E-09	3.79E-08	85.1401	110.2	35.3307	0	6.33615	2.0108	Down
Enpep	10278.8	0.80098	0.14268	5.61395	1.98E-08	3.37E-07	8635.92	6578.45	7275.25	12066.3	14815	12302.1	Up
Egf	115.252	-1.5485	0.44611	-3.4711	0.00052	0.0032	111.263	295.144	108.857	59.0928	73.9218	43.2322	Down
6330410L	10.8543	-6.8369	1.70895	-4.0006	6.32E-05	0.00051	1.935	47.9129	15.2781	0	0	0	Down
Gar1	603.224	-0.5097	0.17599	-2.8963	0.00378	0.01712	687.894	579.746	858.44	476.889	501.612	514.765	Down
Cfi	378.522	3.17365	0.18694	16.9768	1.22E-64	2.92E-61	76.4326	72.8277	77.3455	587.818	689.585	767.121	Up
Pla2g12a	1664.57	-0.4638	0.11823	-3.9232	8.74E-05	0.00067	1924.36	2100.5	1764.62	1476.28	1426.69	1294.96	Down
Ostc	3999.72	0.23564	0.08911	2.64425	0.00819	0.03243	3592.33	3529.27	3899.74	4464.1	4282.18	4230.73	Up
Rpl34	5398.78	-0.2926	0.13629	-2.147	0.03179	0.09582	5999.48	5070.15	6763.44	5075.76	5053.08	4430.8	Down
Lef1	369.131	-0.8363	0.2339	-3.5754	0.00035	0.00227	344.431	517.46	557.652	230.151	315.752	249.339	Down
Hadh	5560.59	-0.6565	0.12746	-5.1511	2.59E-07	3.60E-06	6951.5	7593.24	5868.71	4589.54	4040.35	4320.21	Down
Cyp2u1	179.779	-1.2373	0.28093	-4.4041	1.06E-05	0.0001	208.98	350.723	197.661	113.002	108.771	99.5347	Down
Sgms2	1003.62	-0.3656	0.18488	-1.9775	0.04798	0.13219	937.509	1322.4	1130.58	801.381	776.179	1053.66	Down
Dkk2	55.975	-1.845	0.48456	-3.8075	0.00014	0.00102	48.3751	136.073	78.3004	30.0648	16.8964	26.1404	Down
Tbck	1787.35	-0.3495	0.12148	-2.8771	0.00401	0.01803	1782.14	2099.54	2126.52	1519.83	1697.03	1499.05	Down

Npnt	24025.1	-0.2338	0.10564	-2.2135	0.02686	0.08435	24452.6	25986.1	27464.3	21807.3	24151.3	20289	Down
Arhgef38	3187.43	0.56073	0.10379	5.40265	6.57E-08	1.01E-06	2624.83	2581.55	2520.89	3958.18	3422.58	4016.58	Up
Gm43254	179.639	-0.35	0.17622	-1.986	0.04703	0.1301	201.24	201.234	201.48	152.397	162.628	158.853	Down
Cxxc4	140.556	-1.7174	0.30005	-5.7238	1.04E-08	1.87E-07	169.313	279.812	197.661	72.5701	48.5772	75.4051	Down
Cenpe	111.666	0.93232	0.28273	3.29757	0.00098	0.00547	71.5951	89.1181	69.7065	168.985	107.715	162.875	Up
Bdh2	59.2349	-1.1292	0.30173	-3.7426	0.00018	0.00129	82.2376	82.4102	79.2553	41.4686	34.8488	35.189	Down
Slc9b1	62.596	-1.1716	0.41024	-2.856	0.00429	0.01906	78.3676	94.8676	86.8943	18.6609	47.5211	49.2646	Down
Manba	2119.04	0.64793	0.15603	4.15257	3.29E-05	0.00028	1900.17	1662.58	1390.31	2555.5	2909.35	2296.34	Up
Slc39a8	18980.4	1.23118	0.14832	8.30057	1.04E-16	6.53E-15	13751.1	9734.95	10533.3	24791	27627.7	27444.4	Up
Ddit4l	423.02	-0.4054	0.18899	-2.1451	0.03195	0.09619	488.588	549.082	408.69	382.548	302.023	407.187	Down
Gm31678	3.76325	2.77168	1.37823	2.01104	0.04432	0.12411	0.9675	1.91652	0	6.2203	8.4482	5.027	Up
H2az1	8002.92	0.68647	0.1207	5.68753	1.29E-08	2.28E-07	6255.86	6319.72	5826.7	10997.5	8572.81	10045	Up
Dnajb14	2567.26	0.28193	0.10271	2.74501	0.00605	0.02533	2422.62	2211.66	2317.5	2610.45	2830.15	3011.18	Up
Lamtor3	3542.48	0.24557	0.11875	2.06794	0.03865	0.1115	3438.5	3102.84	3183.58	3724.92	4322.31	3482.71	Up
Dapp1	1172.25	0.97546	0.23404	4.16791	3.07E-05	0.00027	1072.96	685.155	613.035	1602.76	1806.86	1252.73	Up
1110002E	87.0708	-2.4113	0.52611	-4.5833	4.58E-06	4.88E-05	113.198	264.479	62.0674	34.2116	25.3446	23.1242	Down
Mttp	306.694	-0.9052	0.24048	-3.7641	0.00017	0.00119	357.008	439.841	402.961	230.151	146.788	263.415	Down
Trmt10a	439.562	-0.4608	0.19618	-2.3487	0.01884	0.06375	420.863	563.456	543.328	373.218	305.191	431.317	Down
Adh6b	58.1239	-1.4604	0.60048	-2.4321	0.01501	0.05308	43.5376	102.534	109.812	13.4773	20.0645	59.3187	Down
Rap1gds1	3650.3	0.30043	0.1494	2.01091	0.04434	0.12413	3583.63	3084.63	3146.34	3789.2	4811.25	3486.73	Up
Unc5c	701.634	-0.4908	0.12588	-3.8992	9.65E-05	0.00074	860.109	774.273	825.019	624.103	592.43	533.868	Down
Bmpr1b	1050.34	-0.7998	0.287	-2.7866	0.00533	0.02283	799.156	1617.54	1586.06	926.824	547.021	825.434	Down
Pdlim5	7448.07	-0.6663	0.18784	-3.5471	0.00039	0.00249	6638.03	11289.2	9486.76	5585.82	5490.28	6198.3	Down
Gbp5	974.024	0.85917	0.23517	3.65341	0.00026	0.00174	865.914	567.289	643.591	947.558	1628.39	1191.4	Up
Gbp3	1807.95	0.60117	0.16126	3.72798	0.00019	0.00136	1513.17	1550.46	1246.12	1918.96	2604.16	2014.82	Up
Gbp2	4041.81	0.35496	0.17906	1.98231	0.04744	0.13102	3838.08	3371.15	3431.85	3826.52	5845.1	3938.16	Up
Kyat3	547.675	-0.4792	0.24208	-1.9794	0.04777	0.13179	518.581	780.981	613.99	488.293	324.2	560.008	Down
Gtf2b	3080.27	0.29038	0.12093	2.40131	0.01634	0.05681	2817.36	2797.16	2699.45	3877.32	3073.03	3217.28	Up
4930519L(	2.55447	4.85583	1.97773	2.45525	0.01408	0.05051	0	0	0	3.11015	3.16808	9.04861	Up
Gm42705	9.95634	2.5628	1.04918	2.44267	0.01458	0.05187	1.935	5.74955	0.95488	23.8845	23.2326	4.0216	Up
Lmo4	4493.01	0.35562	0.1156	3.07631	0.0021	0.01036	3906.77	3863.7	4055.39	5053.99	5622.28	4455.94	Up
Hs2st1	12474.6	0.44574	0.11804	3.77624	0.00016	0.00114	11898.3	9424.47	10365.3	13561.3	14515.1	15083	Up
Clca3b	207.474	5.79383	2.61281	2.21748	0.02659	0.08365	0	22.0399	0	427.127	8.4482	787.229	Up
Clca2	18.939	1.25578	0.63638	1.9733	0.04846	0.13324	5.80501	14.3739	13.3684	16.5875	24.2886	39.2106	Up
Bcl10	2992.82	0.41911	0.09445	4.4372	9.11E-06	9.04E-05	2735.13	2487.64	2460.73	3566.3	3337.04	3370.1	Up
Syde2	1422.74	-0.483	0.12426	-3.887	0.0001	0.00077	1759.89	1691.33	1524.95	1317.67	1102.49	1140.12	Down
Mcoln3	1091.66	2.97088	0.66753	4.45057	8.56E-06	8.58E-05	468.271	155.238	117.451	1550.93	2773.12	1484.98	Up
Mcoln2	254.592	1.23414	0.23728	5.20114	1.98E-07	2.81E-06	202.208	137.989	115.541	365.961	336.872	368.982	Up
Ssx2ip	2552.34	0.3805	0.11588	3.28365	0.00102	0.00569	2393.6	2180.04	2079.73	2865.48	2644.29	3150.93	Up
Gng5	6392.26	0.43112	0.10082	4.27608	1.90E-05	0.00017	5586.35	5117.1	5629.03	7881.11	7340.43	6799.53	Up
Ttll7	5328.39	0.54531	0.11426	4.77266	1.82E-06	2.12E-05	4019	4297.79	4682.75	5686.39	6670.91	6613.53	Up
Gm42647	40.8059	3.01871	0.5967	5.05897	4.22E-07	5.64E-06	10.6425	11.4991	4.77441	69.46	34.8488	113.61	Up
Gm31121	15.4072	1.75412	0.80041	2.19153	0.02841	0.0882	9.67501	1.91652	9.54883	40.4319	14.7844	16.0864	Up
Rpsa-ps1(	1735.64	-0.3953	0.11788	-3.353	0.0008	0.00461	1842.12	1879.15	2194.32	1544.71	1544.97	1408.57	Down
Ifi44	814.001	1.11592	0.3544	3.14876	0.00164	0.00844	540.833	693.779	307.472	1162.16	609.327	1570.44	Up
Ptgfr	879.449	-1.5382	0.25924	-5.9333	2.97E-09	6.01E-08	1229.69	1817.82	877.537	453.045	519.564	379.036	Down
Gm43618	47.4971	-2.209	0.46402	-4.7605	1.93E-06	2.24E-05	66.7576	114.991	52.5186	14.514	21.1205	15.081	Down
Dnajb4	5778.04	-0.4291	0.10623	-4.0391	5.37E-05	0.00044	6139.76	7116.99	6636.44	5140.04	4535.63	5099.39	Down
Nexn	2692.88	-0.582	0.26068	-2.2327	0.02557	0.08106	2285.24	4860.29	2540.94	1957.32	2228.21	2285.28	Down
Miga1	2822.68	0.40966	0.13989	2.92845	0.00341	0.0157	2333.61	2390.86	2549.54	2968.12	2873.45	3820.52	Up
Usp33	2135.06	0.91631	0.16582	5.52589	3.28E-08	5.39E-07	1619.6	1208.36	1608.98	2491.23	2633.73	3248.45	Up
Pigk	2748.38	0.28513	0.10104	2.82194	0.00477	0.02087	2524.21	2635.21	2273.58	2921.47	3121.61	3014.19	Up
St6galnac	1334.13	-0.746	0.17435	-4.2787	1.88E-05	0.00017	1300.32	1835.07	1879.21	1081.29	1020.12	888.774	Down
Acadm	5942.55	-0.9167	0.20314	-4.5126	6.40E-06	6.60E-05	6269.41	10587.8	6451.19	4299.26	3888.29	4159.34	Down
Slc44a5	46.9285	-2.2261	0.58278	-3.8198	0.00013	0.00098	31.9275	135.114	64.932	12.4406	20.0645	17.0918	Down
Tyw3	277.922	-0.4333	0.20736	-2.0895	0.03667	0.10714	387.968	272.145	297.923	206.306	261.894	241.296	Down
Cryz	784.335	-0.467	0.18679	-2.5003	0.01241	0.04563	908.484	945.801	876.582	612.699	526.957	835.488	Down
Tnni3k	193.75	-2.9175	0.55133	-5.2917	1.21E-07	1.77E-06	216.72	624.785	185.247	26.9546	25.3446	83.4483	Down
Zranb2	5604.01	-0.2036	0.09303	-2.1883	0.02865	0.08875	5847.58	5847.29	6301.27	4910.92	5525.12	5191.89	Down
Ptger3	521.286	1.9011	0.61435	3.09448	0.00197	0.00985	169.313	396.719	94.5334	662.461	733.938	1070.75	Up
Cth	888.258	1.19255	0.19076	6.25169	4.06E-10	9.59E-09	568.891	577.83	475.532	1318.7	938.807	1449.79	Up
Ankrd13c	4357.61	0.26887	0.0827	3.25096	0.00115	0.00628	3934.83	3971.02	3952.26	4534.6	4961.21	4791.74	Up
Depdc1a	42.7761	1.15014	0.53253	2.15976	0.03079	0.09356	27.09	39.2886	13.3684	79.8271	32.7368	64.3457	Up
Wls	13966.9	0.57332	0.09524	6.01982	1.75E-09	3.66E-08	12315.3	10675	10692.8	16769.9	16635.6	16712.8	Up
AI838599	1206.37	-0.6779	0.22722	-2.9835	0.00285	0.01347	1596.38	1478.59	1378.85	884.319	1255.61	644.462	Down
Gm11783	11.9073	-1.6023	0.8036	-1.9939	0.04616	0.12814	11.61	18.2069	23.8721	5.18358	10.5603	2.0108	Down
Lyn	7745.52	0.64462	0.1082	5.95761	2.56E-09	5.22E-08	6502.58	6211.43	5416.1	8899.17	9760.84	9683.02	Up
Chchd7	1185.83	-0.9873	0.1278	-7.725	1.12E-14	5.33E-13	1426.1	1629.04	1673.91	788.941	868.053	728.916	Down
Penk	165.981	-2.2264	0.34535	-6.4467	1.14E-10	3.01E-09	208.98	345.931	265.457	71.5334	71.8097	32.1728	Down
Ubxn2b	1343.6	-0.2458	0.10005	-2.4569	0.01402	0.05033	1446.41	1483.38	1443.78	1194.3	1181.69	1312.05	Down
4930430E	360.983	4.65425	0.41347	11.2566	2.15E-29	4.14E-27	40.6351	34.4973	7.63906	709.114	852.212	521.803	Up
Sdcbp	30804.5	0.90057	0.09828	9.1634	5.03E-20	4.36E-18	22403.5	21257.1	20810.7	39014.7	43857.8	37483.4	Up
Tox	109.551	0.57295	0.25985	2.20489	0.02746	0.08577	105.458	90.0763	68.7516	120.259	132.003	140.756	Up
Car8	14573.7	1.71717	0.11403	15.0586	3.03E-51	2.97E-48	7464.27	6880.3	6048.23	20876.3	23467	22706	Up
Clvs1	44.193	-2.597	0.75194	-3.4537	0.00055	0.00338	55.1476	123.615	48.699	14.514	22.1765	1.0054	Down
Asph	4283.03	-0.3972	0.15569	-2.5512	0.01074	0.04057	4039.32	5923.95	4643.6	3747.73	3484.88	3858.73	Down
4930412C	414.443	-0.4192	0.18146	-2.3099	0.02089	0.06898	431.506	571.122	420.148	359.74	322.088	382.052	Down
Gdf6	85.9496	1.09366	0.51946	2.10541	0.03526	0.10388	87.0751	30.6643	46.7893	171.058	128.835	51.2754	Up
2610301B	2594.66	0.22713	0.08125	2.79532	0.00518	0.02236	2402.31	2339.11	2431.13	2747.3	2805.86	2842.27	Up
Plekhf2	3098.97	0.27427	0.09012	3.04354	0.00234	0.01136	2964.42	2710.91	2740.51	3511.36	3372.95	3293.69	Up
Gm11827	63.3365	-1.2899	0.33291	-3.8746	0.00011	0.0008	79.3351	112.116	78.3004	40.4319	30.6247	39.2106	Down
Esrp1	3531.04	0.43569	0.12269	3.55128	0.00038	0.00246	2811.56	3218.79	2975.42	4255.72	3548.25	4376.51	Up
Gem	1522.84	0.53369	0.23855	2.23722	0.02527	0.0804	959.761	1385.64	1387.44	1585.14	2444.7	1374.38	Up

Pdp1	1698.47	-0.2379	0.11005	-2.1617	0.03064	0.09319	1766.66	1933.77	1814.28	1438.96	1547.08	1690.08	Down
Tmem67	1025.3	-0.3797	0.17786	-2.1346	0.0328	0.09807	1220.02	1159.49	1099.07	885.355	696.977	1090.86	Down
Rbm12b2	934.948	-0.2941	0.14254	-2.063	0.03911	0.11252	961.696	994.672	1133.45	918.53	728.658	872.688	Down
Gm11839	28.2575	-1.084	0.54822	-1.9773	0.04801	0.13224	44.5051	31.6225	39.1502	20.7343	7.39218	26.1404	Down
Rbm12b1	514.583	-0.392	0.14839	-2.6419	0.00824	0.03262	602.753	519.376	630.223	418.833	427.69	488.625	Down
Pip4p2	2527.34	0.31401	0.09379	3.34802	0.00081	0.00468	2251.38	2326.65	2181.91	2785.66	2962.15	2656.27	Up
Gm11837	65.3992	-1.8879	0.33316	-5.6665	1.46E-08	2.55E-07	83.2051	119.782	105.992	26.9546	24.2886	32.1728	Down
Tmem64	3005.69	0.21955	0.08421	2.60725	0.00913	0.0354	2814.46	2826.86	2690.86	3160.95	3352.88	3188.13	Up
Decr1	3314.85	-0.404	0.14071	-2.8709	0.00409	0.01833	3792.61	4390.74	3144.43	2925.61	2840.71	2795.01	Down
Gm11867	31.1914	2.2901	0.86087	2.66022	0.00781	0.03116	16.4475	12.4574	2.86465	83.974	63.3615	8.04321	Up
Cngb3	13.271	-2.18	0.7594	-2.8707	0.0041	0.01834	16.4475	32.5808	16.233	6.2203	2.11205	6.03241	Down
Cpne3	6876.94	0.50389	0.0849	5.93508	2.94E-09	5.95E-08	5941.43	5507.11	5615.67	7699.69	8307.75	8190	Up
Wwp1	695.326	-0.4746	0.14436	-3.2874	0.00101	0.00564	792.384	837.518	796.372	561.9	512.172	671.608	Down
Atp6v0d2	6303.27	2.89168	0.58492	4.9437	7.67E-07	9.75E-06	2612.25	1209.32	669.373	10211.7	13775.9	9341.18	Up
Ttpa	661.163	-0.4313	0.18634	-2.3148	0.02062	0.06833	641.453	772.356	864.169	529.762	482.604	676.635	Down
Nkain3	39.6807	2.46974	0.76705	3.2198	0.00128	0.00689	1.935	26.8312	7.63906	55.9827	40.129	105.567	Up
Pnisr	8972.44	-0.273	0.1034	-2.6398	0.00829	0.03277	9207.71	9573	10675.6	7677.92	8046.91	8653.49	Down
Coq3	874.626	-0.3644	0.13021	-2.7983	0.00514	0.0222	945.249	1106.79	901.409	732.958	796.243	765.11	Down
Faxc	42.1612	-0.9363	0.38025	-2.4623	0.0138	0.04975	56.1151	53.6625	56.3381	20.7343	36.9609	29.1566	Down
Mms22l	228.606	0.54691	0.25166	2.17325	0.02976	0.0913	210.915	220.399	126.045	252.959	299.911	261.404	Up
Ndufaf4	1468.59	-0.3478	0.13531	-2.5704	0.01016	0.0387	1438.67	1839.86	1655.77	1180.82	1341.15	1355.28	Down
Fut9	51.7851	-1.8041	0.86124	-2.0947	0.03619	0.10604	27.09	177.278	37.2404	17.6242	4.2241	47.2538	Down
Bach2	978.116	-0.3224	0.13137	-2.4538	0.01413	0.05068	983.949	1158.53	1118.17	807.602	959.927	840.515	Down
Ankrd6	360.703	-0.9312	0.23208	-4.0123	6.01E-05	0.00048	462.466	452.298	505.133	276.803	167.908	299.609	Down
Gm11942	99.4112	-0.999	0.31271	-3.1946	0.0014	0.0074	120.938	151.405	125.09	82.9373	72.8658	43.2322	Down
Gabrr2	55.4137	-1.0961	0.45035	-2.4339	0.01494	0.05288	82.2376	42.1634	102.172	25.9179	32.7368	47.2538	Down
Gm12751	540.078	0.3584	0.15279	2.34568	0.01899	0.06413	476.978	421.634	521.366	678.012	573.422	569.057	Up
Akirin2	3165.37	-0.3054	0.09906	-3.083	0.00205	0.01016	3270.15	3680.67	3546.44	2765.96	3003.34	2725.64	Down
Smim8	588.792	-0.3202	0.14678	-2.1812	0.02917	0.08998	625.973	731.151	604.441	578.487	489.996	502.7	Down
Mob3b	3184.62	0.3131	0.11931	2.62412	0.00869	0.03401	2478.74	2983.06	3059.44	3415.98	3439.47	3731.04	Up
Aco1	4670.55	0.74461	0.1439	5.17447	2.29E-07	3.20E-06	4202.83	3124.88	3146.34	5533.99	6097.49	5917.79	Up
Ddx58	4169.01	0.62776	0.08985	6.98652	2.82E-12	9.41E-11	3510.1	3146.92	3171.17	4979.35	5090.04	5116.49	Up
Topors	2702.93	0.41171	0.11217	3.67051	0.00024	0.00164	2491.32	2221.24	2246.84	3130.88	3321.2	2806.07	Up
Tmem215	16.29	-3.4275	0.80562	-4.2545	2.09E-05	0.00019	35.7976	27.7895	25.7818	4.14686	4.2241	0	Down
B4galt1	7032.63	0.34208	0.08499	4.02516	5.69E-05	0.00046	6311.98	5991.03	6305.09	7654.07	8305.64	7627.98	Up
Nfx1	3944.77	-0.2175	0.08284	-2.6251	0.00866	0.03394	4228.95	4085.06	4410.6	3594.29	3611.61	3738.08	Down
Aqp7	39.6833	-2.7439	0.79153	-3.4666	0.00053	0.00324	28.0575	159.071	20.0525	8.29373	10.5603	12.0648	Down
Myorg	1214.72	0.32492	0.11156	2.91266	0.00358	0.01638	1035.23	1122.12	1078.06	1253.39	1457.32	1342.21	Up
1110017D	1870.99	-0.6677	0.23626	-2.8261	0.00471	0.02064	1679.58	2675.46	2534.26	1656.67	1019.06	1660.92	Down
Dnaic1	2046.53	-0.7653	0.25475	-3.0043	0.00266	0.01271	1767.63	3051.1	2912.39	1595.51	1063.42	1889.15	Down
Enho	443.12	-0.8171	0.25675	-3.1823	0.00146	0.00767	361.846	604.661	729.531	348.337	294.631	319.717	Down
Cntfr	385.595	-2.1423	0.40626	-5.2733	1.34E-07	1.94E-06	548.573	854.767	483.171	121.296	65.4736	240.291	Down
Il11ra1	3074.11	-0.3941	0.11359	-3.4692	0.00052	0.00322	3877.75	3250.41	3345.91	2821.94	2621.05	2527.58	Down
Ccl27a	394.068	-1.3063	0.26163	-4.9929	5.95E-07	7.75E-06	781.741	443.674	458.344	232.224	189.029	259.393	Down
Gm13307	2.88556	-4.9252	2.41256	-2.0415	0.0412	0.11721	6.77251	10.5408	0	0	0	0	Down
Gm17167	63.6761	-2.6437	0.57383	-4.607	4.08E-06	4.41E-05	192.533	75.7024	61.1125	29.028	11.6163	12.0648	Down
Dnajb5	1343.97	-0.6496	0.16596	-3.9142	9.07E-05	0.0007	1282.91	1846.56	1795.18	1031.53	992.664	1114.99	Down
Vcp	5071.54	0.48066	0.09113	5.27461	1.33E-07	1.93E-06	4520.17	4046.73	4136.55	5701.94	5920.08	6103.79	Up
Unc13b	3938.28	0.20713	0.0952	2.1757	0.02958	0.09087	3396.9	3791.83	3779.43	4025.57	4290.63	4345.34	Up
Cd72	522.897	1.10753	0.29175	3.79614	0.00015	0.00106	404.416	298.018	292.194	586.781	495.276	1060.7	Up
Arhgef39	162.021	0.59476	0.25316	2.34937	0.01881	0.06369	146.093	139.906	101.218	214.6	216.485	153.826	Up
Gm12454	150.494	0.79273	0.2447	3.23961	0.0012	0.00648	118.035	109.241	103.127	221.857	205.925	144.778	Up
Car9	239.948	2.48411	0.3628	6.8471	7.54E-12	2.40E-10	107.393	48.8712	62.0674	581.598	363.273	276.485	Up
Tpm2	2305.61	-1.6635	0.20617	-8.069	7.09E-16	4.02E-14	2596.77	4249.88	3667.71	1207.77	1219.71	891.791	Down
Creb3	4540.67	0.28403	0.12534	2.266	0.02345	0.07574	3764.55	4123.39	4397.24	5374.33	5244.22	4340.32	Up
Npr2	5926.57	-0.5772	0.10734	-5.3772	7.56E-08	1.15E-06	7292.06	6494.12	7503.47	5127.6	4678.19	4463.98	Down
Spag8	864.525	-0.5355	0.20281	-2.6403	0.00828	0.03274	903.646	1165.24	1000.72	816.932	516.396	784.213	Down
Hint2	1177.36	-0.2888	0.11929	-2.4213	0.01547	0.05436	1247.11	1331.02	1306.28	1181.86	1010.62	987.304	Down
Tmem8b	741.335	-0.5041	0.15196	-3.3171	0.00091	0.00515	882.361	787.689	938.65	521.468	645.231	672.613	Down
Reck	3419.39	-0.8382	0.11116	-7.5397	4.71E-14	2.10E-12	4587.89	4210.59	4358.09	2485.01	2675.97	2198.81	Down
Glipr2	1799.23	0.93465	0.17095	5.46739	4.57E-08	7.29E-07	999.429	1442.18	1266.17	2220.65	2722.43	2144.52	Up
Grhpr	901.215	-0.3803	0.13077	-2.9081	0.00364	0.01658	1085.54	1042.59	930.056	780.647	713.873	854.591	Down
Polr1e	371.175	-0.3757	0.14934	-2.5156	0.01188	0.044	394.741	464.755	398.186	307.905	333.704	327.761	Down
Tomm5	1183.94	-0.3101	0.1221	-2.54	0.01109	0.04159	1290.65	1383.73	1257.58	1154.9	1068.7	948.093	Down
Frmpd1	376.12	-0.299	0.1484	-2.015	0.0439	0.12326	385.066	409.176	450.705	347.3	312.584	351.89	Down
Shb	1927.33	0.41455	0.10135	4.09013	4.31E-05	0.00036	1518.01	1701.87	1736.93	2197.84	2257.78	2151.56	Up
Aldh1b1	426.05	-2.6053	0.41066	-6.3442	2.24E-10	5.58E-09	549.541	1276.4	369.54	159.654	106.659	94.5077	Down
Igfbpl1	16.0615	-4.4858	0.93916	-4.7764	1.78E-06	2.08E-05	38.7001	31.6225	21.9623	2.07343	0	2.0108	Down
Stra6l	399.403	3.68275	0.32659	11.2762	1.72E-29	3.34E-27	89.0101	40.2469	43.9246	708.077	965.207	549.954	Up
Tmod1	2038.11	-0.2896	0.12145	-2.3844	0.01711	0.05901	2418.75	2360.19	1947.01	1898.23	1805.8	1798.66	Down
Tstd2	685.981	0.43489	0.17516	2.48281	0.01303	0.04745	668.544	497.336	584.388	724.664	715.985	924.969	Up
Ncbp1	381.139	0.56001	0.17745	3.15583	0.0016	0.00827	366.683	273.104	284.555	416.76	454.091	491.641	Up
Xpa	1184.41	-0.2289	0.11411	-2.0064	0.04482	0.12525	1357.4	1154.7	1322.51	1056.41	1101.43	1113.98	Down
Nans	316.463	0.38533	0.18508	2.08197	0.03735	0.10862	231.233	270.229	321.796	334.859	391.785	348.874	Up
Trim14	1425.55	0.30125	0.09761	3.08617	0.00203	0.01008	1254.85	1256.28	1320.6	1490.8	1623.11	1607.64	Up
Coro2a	2000.25	0.25929	0.11849	2.18838	0.02864	0.08875	2017.24	1779.49	1666.27	2033	2292.63	2212.89	Up
Tbc1d2	1310.82	1.31748	0.2367	5.56614	2.60E-08	4.34E-07	974.274	581.663	696.11	1775.89	2378.17	1458.84	Up
Tgfbr1	4230.38	0.97438	0.10553	9.23325	2.63E-20	2.37E-18	3095.04	2583.47	2882.79	5376.41	5770.12	5674.48	Up
Alg2	2023.78	0.23394	0.11182	2.09219	0.03642	0.10657	1987.25	1846.56	1746.48	2357.49	2020.18	2184.74	Up
Nr4a3	548.546	-1.1427	0.23447	-4.8734	1.10E-06	1.34E-05	576.631	656.407	1032.23	312.051	359.049	354.907	Down
Stx17	3555.22	0.5996	0.08292	7.23094	4.80E-13	1.83E-11	2953.78	2752.12	2774.89	4277.49	4215.65	4357.41	Up
Erp44	5493.13	0.33554	0.08185	4.09958	4.14E-05	0.00035	5090.99	4757.75	4722.85	6236.88	6167.19	5983.14	Up
Tmeff1	2011	-0.3448	0.10316	-3.3426	0.00083	0.00476	2313.3	2130.21	2307	1904.45	1735.05	1676	Down

Cavin4	75.1003	-1.4352	0.56717	-2.5305	0.01139	0.04254	51.2776	219.441	58.2479	45.6155	33.7928	42.2268 Down
Plppr1	6.76355	-2.7828	1.08439	-2.5662	0.01028	0.03911	4.83751	13.4156	17.1879	2.07343	1.05603	2.0108 Down
Acnat1	22.9992	-2.7961	0.93666	-2.9852	0.00283	0.01341	6.77251	77.619	36.2855	4.14686	2.11205	11.0594 Down
Zfp189	267.297	-0.4075	0.17377	-2.3451	0.01902	0.0642	341.528	266.396	306.517	238.445	219.653	231.242 Down
Aldob	15.7123	-1.9356	0.74623	-2.5939	0.00949	0.03656	10.6425	40.2469	23.8721	5.18358	5.28013	9.04861 Down
Nipsnap3b	2380.5	0.4257	0.09724	4.3777	1.20E-05	0.00012	2120.76	1993.18	1981.38	2648.81	2917.8	2621.08 Up
Abca1	3085.78	-0.6239	0.12873	-4.8468	1.25E-06	1.51E-05	3869.04	3647.13	3712.58	2563.8	2049.75	2672.36 Down
4930412L0	15.523	-4.4461	1.08095	-4.1131	3.90E-05	0.00033	16.4475	27.7895	44.8795	0	0	4.0216 Down
Fsd1l	2687.67	-0.4529	0.19673	-2.3021	0.02133	0.07012	2796.08	3428.65	3093.82	2339.87	1648.46	2819.14 Down
Tal2	31.0253	1.2664	0.53169	2.38187	0.01723	0.05928	14.5125	16.2904	23.8721	57.0194	22.1765	52.2808 Up
Klf4	9930.99	-0.5459	0.1113	-4.9053	9.33E-07	1.16E-05	12365.6	12094.2	10903.8	8882.58	7956.1	7383.66 Down
Ctnnal1	1038.77	-0.2464	0.12381	-1.9903	0.04656	0.12905	1211.31	1147.99	1022.68	980.733	877.557	992.331 Down
Epb41l4b	6350.17	-0.7269	0.11439	-6.3549	2.09E-10	5.27E-09	8400.82	7435.13	7915.02	5351.53	4428.97	4569.55 Down
Pakap	115.296	-1.3423	0.26581	-5.05	4.42E-07	5.90E-06	147.06	181.111	168.059	72.5701	48.5772	74.3997 Down
Txn1	11543.3	0.34882	0.10977	3.17755	0.00149	0.00778	10945.3	9378.48	10139.9	13344.6	13695.6	11756.2 Up
Ecpas	5016.86	0.26708	0.08502	3.14134	0.00168	0.00861	4523.07	4608.27	4529.96	5213.64	5785.96	5440.22 Up
Ptgr1	3599.42	0.28116	0.11589	2.42612	0.01526	0.05379	3380.45	2901.61	3467.18	4190.41	4000.22	3656.64 Up
Shoc1	13.6665	1.52327	0.72361	2.10509	0.03528	0.10393	12.5775	2.87478	5.7293	22.8077	16.8964	21.1134 Up
Susd1	307.184	0.91315	0.25444	3.58889	0.00033	0.00217	269.933	231.899	137.503	383.585	393.897	426.29 Up
Ptbp3	8945.83	0.37486	0.0935	4.00904	6.10E-05	0.00049	7859.98	7342.18	8168.07	9541.93	10659.5	10103.3 Up
Hsdl2	2344.24	-0.3159	0.14852	-2.1272	0.0334	0.09947	2635.47	3037.68	2126.52	2038.18	2157.46	2070.12 Down
Snx30	1670.53	0.40848	0.1507	2.71052	0.00672	0.02756	1223.89	1458.47	1624.26	1799.74	2155.35	1761.46 Up
Slc31a2	2883.56	0.3523	0.10685	3.2971	0.00098	0.00547	2716.74	2360.19	2522.8	2994.04	3346.54	3361.06 Up
Fkbp15	3755.46	0.58955	0.11928	4.94268	7.71E-07	9.78E-06	3230.49	2640	3125.33	4452.69	4833.43	4250.83 Up
Wdr31	681.163	-0.5858	0.23171	-2.5282	0.01146	0.04281	846.564	894.055	712.343	631.36	362.217	640.44 Down
4933430I1	28.6505	1.57323	0.49812	3.15832	0.00159	0.00821	14.5125	15.3321	13.3684	34.2116	62.3055	32.1728 Up
Rgs3	3010.25	-0.2724	0.11214	-2.4294	0.01512	0.05342	3006.99	3365.4	3508.24	2674.73	2948.42	2557.74 Down
Orm1	151.076	1.50355	0.46075	3.26328	0.0011	0.00605	89.0101	95.8259	51.5637	240.518	88.7061	340.831 Up
Orm3	56.927	-1.5213	0.40799	-3.7287	0.00019	0.00135	81.2701	82.4102	89.759	19.6976	22.1765	46.2484 Down
Orm2	35.8848	1.55502	0.71262	2.18213	0.0291	0.08985	15.48	20.1234	19.0977	36.2851	13.7283	110.594 Up
Atp6v1g1	9638.44	0.54902	0.08315	6.60281	4.03E-11	1.14E-09	7622.94	7824.18	8031.52	11854.8	11667	10830.2 Up
Tmem268	1931.59	1.48058	0.10878	13.6109	3.45E-42	2.01E-39	1118.43	1011.92	927.191	2829.2	2834.37	2868.41 Up
Tnfsf15	276.285	1.38245	0.28135	4.91367	8.94E-07	1.12E-05	217.688	103.492	138.458	346.263	403.402	448.409 Up
Tnc	790.75	1.58483	0.18308	8.6564	4.87E-18	3.66E-16	333.788	418.759	433.517	1094.77	1435.14	1028.53 Up
Pappa	117.232	-1.2284	0.28776	-4.2689	1.96E-05	0.00018	116.1	193.568	183.338	64.2764	69.6977	76.4105 Down
Astn2	1318.62	-0.3321	0.08998	-3.6909	0.00022	0.00153	1477.37	1453.68	1478.16	1180.82	1158.46	1163.25 Down
Tlr4	2790.08	0.9842	0.09151	10.7546	5.64E-27	9.28E-25	1845.03	1994.14	1781.81	3641.98	3761.56	3715.96 Up
Brinp1	168.709	1.31721	0.27511	4.78791	1.69E-06	1.98E-05	79.3351	118.824	91.6688	192.829	309.415	220.183 Up
Cdk5rap2	845.689	-0.2834	0.12188	-2.3256	0.02004	0.06692	863.979	925.678	995.943	780.647	708.593	799.294 Down
Megf9	3529.04	0.27811	0.13065	2.12874	0.03328	0.09921	3631.03	2875.73	3063.26	3857.62	3544.02	4202.58 Up
Rasef	3118	0.5944	0.11223	5.29607	1.18E-07	1.73E-06	2584.2	2378.4	2491.29	3328.89	3976.99	3948.21 Up
2310002L0	109.998	-8.332	1.60548	-5.1897	2.11E-07	2.97E-06	91.9126	490.628	75.4357	0	0	2.0108 Down
Dmac1	819.953	-0.2834	0.13548	-2.0916	0.03647	0.1067	840.759	958.259	901.409	750.582	814.196	654.516 Down
Ptprd	2326.39	-0.5744	0.11687	-4.915	8.88E-07	1.11E-05	2539.69	3088.47	2722.37	1755.16	1907.18	1945.45 Down
Lurap1l	3959.77	0.35182	0.12202	2.88331	0.00394	0.01773	3863.23	3324.2	3250.42	4562.59	4763.73	3994.46 Up
Mpdz	1722.18	-0.696	0.1174	-5.9287	3.05E-09	6.17E-08	2032.72	2189.62	2166.63	1221.25	1473.16	1249.71 Down
Gm5860	26.603	-1.2	0.58762	-2.0421	0.04114	0.11711	24.1875	41.2051	45.8344	25.9179	7.39218	15.081 Down
Nfib	9915.18	-0.2549	0.08377	-3.0434	0.00234	0.01136	10355.2	10946.2	11065.2	8845.26	9502.12	8777.15 Down
Ttc39b	3037.41	0.40575	0.08692	4.66786	3.04E-06	3.38E-05	2672.24	2478.06	2688.95	3376.58	3501.78	3506.84 Up
Snapc3	1019.05	-0.286	0.11242	-2.5439	0.01096	0.0412	1164.87	1042.59	1151.59	898.833	973.655	882.742 Down
Gm12551	5.30179	2.86209	1.25822	2.27472	0.02292	0.07433	1.935	0.95826	0.95488	18.6609	5.28013	4.0216 Up
Plin2	12721.2	0.93304	0.16286	5.72901	1.01E-08	1.83E-07	9369.28	8721.11	8145.15	16342.8	20382.3	13366.8 Up
Dennd4c	1985.23	0.40122	0.1405	2.85569	0.00429	0.01908	1994.02	1656.83	1481.98	2268.33	2390.84	2119.39 Up
Acer2	20005.4	-0.6995	0.09808	-7.1318	9.91E-13	3.56E-11	25517.9	22394.5	26374.8	15505.1	14826.6	15413.8 Down
Slc24a2	99.9118	-1.8267	0.48065	-3.8005	0.00014	0.00105	132.548	260.646	74.4809	29.028	46.4651	56.3024 Down
Cdkn2a	97.8764	1.42441	0.32152	4.43021	9.41E-06	9.30E-05	53.2126	43.1216	63.0223	173.132	155.236	99.5347 Up
Gm12648	50.7915	2.19355	0.45841	4.78508	1.71E-06	2.00E-05	8.70751	28.7478	17.1879	72.5701	96.0983	81.4375 Up
Tek	14521.7	-0.4841	0.15657	-3.0917	0.00199	0.00993	16433	14817.6	19555	10831.6	14321.8	11171 Down
Gm12708	24.158	1.23392	0.5177	2.38348	0.01715	0.05907	14.5125	15.3321	13.3684	29.028	48.5772	24.1296 Up
Hook1	1637.99	0.56716	0.12273	4.62113	3.82E-06	4.15E-05	1376.75	1312.81	1270.95	2079.65	1707.59	2080.17 Up
Cyp2j9	373.951	-0.6552	0.1754	-3.7356	0.00019	0.00132	450.856	476.255	444.975	281.987	345.32	244.312 Down
Nfia	3864.47	-0.3027	0.10188	-2.9707	0.00297	0.01396	4178.64	4625.51	4000.96	3273.95	3535.57	3572.19 Down
Patj	3918.8	0.49034	0.1205	4.06927	4.72E-05	0.00039	3718.11	2923.65	3135.84	4468.25	4743.67	4523.3 Up
Kank4	1784.45	-0.6255	0.1246	-5.0201	5.16E-07	6.78E-06	2170.11	2152.25	2173.31	1371.58	1597.77	1241.67 Down
Atg4c	984.569	0.64382	0.13153	4.89476	9.84E-07	1.22E-05	854.304	698.571	752.448	1151.79	1303.14	1147.16 Up
Foxd3	3.35894	-5.1446	1.95096	-2.6369	0.00837	0.03299	3.87001	14.3739	1.90977	0	0	0 Down
Itgb3bp	478.073	-0.2852	0.13195	-2.1616	0.03065	0.09319	512.776	515.543	547.148	400.172	461.483	431.317 Down
Pgm1	5150.81	1.11595	0.14516	7.68785	1.50E-14	7.03E-13	3612.65	3538.85	2605.88	7023.75	6834.6	7289.16 Up
Ror1	671.557	0.44855	0.15155	2.95983	0.00308	0.0144	539.866	581.663	582.479	647.947	825.812	851.575 Up
Cachd1	1632.46	0.38922	0.14679	2.65147	0.00801	0.03185	1508.33	1256.28	1476.25	2088.98	1579.81	1885.13 Up
Raver2	3104.55	-0.5744	0.14231	-4.0365	5.43E-05	0.00044	3622.33	3400.86	4120.32	2260.04	2908.29	2315.44 Down
E130102H	226.943	-0.5133	0.25625	-2.0031	0.04516	0.12598	278.64	183.027	338.983	190.756	194.309	175.945 Down
0610043K	110.907	-0.8233	0.29983	-2.7458	0.00604	0.02529	130.613	132.24	162.33	52.8725	91.8742	95.5131 Down
Ak4	171.158	-0.525	0.21864	-2.4013	0.01634	0.05681	193.5	241.481	170.924	149.287	132.003	139.751 Down
Lepr	4880.33	-0.4008	0.12464	-3.2156	0.0013	0.00697	5775.02	4884.24	6002.39	3827.55	4540.91	4251.84 Down
Sgip1	513.141	-0.4167	0.14839	-2.8083	0.00498	0.02165	577.598	644.908	537.599	412.613	487.884	418.247 Down
Gm12709	155.214	-0.8747	0.23866	-3.6649	0.00025	0.00167	167.378	194.527	240.63	99.5247	125.667	103.556 Down
Insl5	78.1738	-0.6677	0.29375	-2.2731	0.02302	0.07461	86.1076	95.8259	105.992	50.7991	54.9133	75.4051 Down
Gm44037	45.8987	-1.0469	0.51861	-2.0187	0.04351	0.12245	91.9126	25.873	67.7967	23.8445	33.7928	32.1728 Down
Oma1	2060.23	0.3311	0.10802	3.06531	0.00217	0.01069	1964.03	1703.78	1806.64	2395.85	2347.54	2143.51 Up
Gm12718	16.2248	1.61121	0.57985	2.77867	0.00546	0.02332	7.74001	8.62433	7.63906	19.6976	28.5127	25.135 Up
Dab1	137.533	1.0888	0.42908	2.53751	0.01116	0.04183	59.0176	154.28	50.6088	215.637	142.563	203.091 Up
Fyb2	277.948	2.47238	0.20774	11.9015	1.16E-32	2.92E-30	102.555	88.1598	63.9772	489.33	455.147	468.517 Up

Plpp3	11542.9	-0.8817	0.10341	-8.5256	1.52E-17	1.09E-15	13439.6	15113.7	16339	8029.36	8153.57	8181.95	Down
Hspd1-ps	12.7882	-2.1129	0.73115	-2.8899	0.00385	0.01743	13.545	28.7478	20.0525	5.18358	3.16808	6.03241	Down
Gm12724	3.69547	-3.3028	1.5924	-2.0741	0.03807	0.11025	5.80501	9.58259	4.77441	0	0	2.0108	Down
Dhcr24	1631.83	1.42228	0.12644	11.249	2.34E-29	4.47E-27	857.206	973.591	829.793	2154.3	2377.11	2598.96	Up
Lexm	10.9669	2.10091	1.04421	2.01195	0.04423	0.1239	0	5.74955	6.68418	25.9179	6.33615	21.1134	Up
Ttc22	233.445	1.67623	0.25533	6.56496	5.20E-11	1.44E-09	74.4976	143.739	115.541	327.602	363.273	376.02	Up
Acot11	212.943	0.94741	0.22486	4.21335	2.52E-05	0.00022	184.793	131.281	120.315	271.62	264.006	305.642	Up
Cyb5rl	815.825	-0.4966	0.14446	-3.4378	0.00059	0.00355	833.019	999.464	1032.23	713.261	606.159	710.818	Down
Tceanc2	879.194	0.38569	0.1221	3.15889	0.00158	0.0082	833.986	734.984	718.072	1002.5	1047.58	938.039	Up
Lrrc42	1688.44	0.27002	0.11345	2.38014	0.01731	0.05953	1608.95	1494.88	1488.66	1850.54	2009.62	1678.01	Up
Dio1	378.333	1.5904	0.35172	4.52181	6.13E-06	6.35E-05	210.915	225.191	129.864	666.608	290.407	747.013	Up
Yipf1	2334.56	0.27047	0.10675	2.53358	0.01129	0.04224	2298.78	2106.25	1944.14	2665.4	2474.27	2518.53	Up
Ndc1	685.964	-0.3055	0.13659	-2.2364	0.02532	0.0805	773.034	823.144	678.922	585.744	591.374	663.565	Down
4933424M	32.3582	1.34613	0.50505	2.66534	0.00769	0.03079	27.09	13.4156	14.3232	30.0648	55.9693	53.2862	Up
Lrp8	239.949	1.81618	0.35867	5.0637	4.11E-07	5.51E-06	153.833	98.7006	65.8869	290.28	535.405	295.588	Up
Magoh	2103.4	-0.2944	0.13745	-2.1415	0.03224	0.09674	2019.18	2275.86	2656.48	1992.57	1940.97	1735.32	Down
Czib	3097.15	-0.446	0.12965	-3.44	0.00058	0.00353	3319.5	3334.74	4062.07	2370.97	2791.08	2704.53	Down
Scp2	247.576	0.63353	0.22452	2.82171	0.00478	0.02088	227.363	204.109	150.871	295.464	259.782	347.869	Up
Echdc2	1989.17	-0.2149	0.09399	-2.2865	0.02222	0.07248	2244.6	2012.34	2154.22	1823.58	1875.5	1824.8	Down
Txndc12	2833.13	0.30775	0.08683	3.54436	0.00039	0.00252	2645.15	2436.85	2514.21	3114.29	3218.77	3069.49	Up
Osbpl9	14069	0.36645	0.08956	4.0916	4.28E-05	0.00036	12811.7	11794.2	12269.3	14961.9	16778.1	15798.9	Up
Ttc39aos1	86.4409	1.36513	0.31008	4.40246	1.07E-05	0.0001	49.3426	62.2868	33.4209	113.002	129.891	130.702	Up
Rnf11	10144.3	0.23589	0.09201	2.56365	0.01036	0.03935	9134.18	9788.61	9027.46	10575.5	11745.1	10594.9	Up
Agbl4	285.168	-1.0954	0.27863	-3.9312	8.45E-05	0.00065	275.738	524.167	365.72	186.609	143.619	215.156	Down
Slc5a9	114.873	1.31575	0.57964	2.26994	0.02321	0.07515	25.155	140.864	31.5111	168.985	125.667	197.059	Up
Trabd2b	1143.26	-0.479	0.1608	-2.9791	0.00289	0.01362	1221.95	1606.04	1165.91	915.42	1043.35	906.872	Down
Foxd2	39.416	-0.867	0.39857	-2.1753	0.02961	0.09094	58.0501	38.3303	56.3381	23.8445	33.7928	26.1404	Down
Cmpk1	5340.11	0.31711	0.09258	3.4254	0.00061	0.00369	4655.62	4866.04	4744.81	5763.1	6387.9	5623.21	Up
Stil	91.74	1.54946	0.35108	4.41342	1.02E-05	9.94E-05	43.5376	64.2033	32.466	129.589	107.715	172.929	Up
Tal1	1089.21	-0.3852	0.1436	-2.6822	0.00732	0.02958	1175.51	1091.46	1434.23	884.319	991.608	958.147	Down
Pdzk1ip1	1365.69	0.73772	0.15075	4.89361	9.90E-07	1.22E-05	1219.05	936.219	916.688	1604.84	1666.41	1850.94	Up
Cyp4a12a	36.9113	3.95014	0.69941	5.64783	1.62E-08	2.82E-07	6.77251	2.87478	3.81953	125.443	44.3531	38.2052	Up
Cyp4a32	704.148	-1.1166	0.16816	-6.6402	3.13E-11	9.02E-10	828.181	1036.84	1026.5	506.954	377.001	449.414	Down
Cyp4b1	33590.1	-0.958	0.11525	-8.3123	9.38E-17	5.94E-15	45829.6	40612	46607.8	25259.6	22556.7	20675.1	Down
Mknk1	2843.07	0.27776	0.09312	2.98274	0.00286	0.01349	2720.61	2440.68	2549.54	3054.16	3054.03	3239.4	Up
Uqcrh	5117.88	-0.2799	0.11641	-2.4046	0.01619	0.05646	4918.78	6198.02	5721.66	4803.1	4509.23	4556.48	Down
Lrrc41	1205.47	0.32858	0.15437	2.12853	0.03329	0.09922	1279.04	929.511	997.853	1442.07	1310.53	1273.84	Up
Rad54l	57.9214	0.92136	0.31649	2.9112	0.0036	0.01644	43.5376	42.1634	34.3758	85.0107	77.0899	65.3511	Up
Tspan1	13099.5	1.2122	0.19953	6.07526	1.24E-09	2.70E-08	7169.19	9948.64	6578.19	19059	14338.7	21503.5	Up
Pik3r3	2309.05	1.18176	0.13426	8.80172	1.35E-18	1.05E-16	1177.45	1572.5	1488.66	3191.01	3149.07	3275.6	Up
Ipp	391.587	0.49204	0.19852	2.47855	0.01319	0.04791	387.968	333.474	254.954	511.101	427.69	434.333	Up
C530005A	66.7911	-0.7416	0.32603	-2.2747	0.02292	0.07433	90.9451	89.1181	70.6613	63.2397	48.5772	38.2052	Down
Ccdc17	6117.08	-0.6673	0.19501	-3.4218	0.00062	0.00373	6194.91	8590.79	7735.51	5512.22	3512.34	5156.7	Down
Akr1a1	28678.3	0.45541	0.0851	5.35172	8.71E-08	1.30E-06	24957.7	24416.4	23193.2	33586.5	34582.7	31333.3	Up
Prdx1	28990.2	0.38971	0.0899	4.33481	1.46E-05	0.00014	27187.8	24437.5	23669.6	32981	33016.6	32648.4	Up
Ccdc163	731.17	-0.4488	0.12234	-3.6681	0.00024	0.00165	848.499	777.148	906.184	620.993	641.007	593.187	Down
Mutyh	184.574	-0.5451	0.2042	-2.6694	0.0076	0.0305	247.68	203.151	206.255	171.058	142.563	136.735	Down
Zswim5	313.697	0.35839	0.16381	2.1879	0.02868	0.08881	286.38	248.189	290.284	317.235	359.049	381.047	Up
Tctex1d4	1471.68	-0.5914	0.22319	-2.6496	0.00806	0.032	1565.42	2181	1561.23	1293.82	831.092	1397.51	Down
Plk3	1249.02	0.7821	0.24489	3.19364	0.0014	0.00742	1071.99	814.52	868.943	1598.62	2101.49	1038.58	Up
Kif2c	66.9503	0.99143	0.34647	2.86148	0.00422	0.01878	49.3426	48.8712	36.2855	74.6435	73.9218	118.637	Up
Dmap1	1567.88	-0.2872	0.12724	-2.2574	0.02399	0.07708	1590.57	1804.4	1775.13	1545.74	1446.75	1244.69	Down
Atp6v0b	2567.36	0.31882	0.09891	3.22322	0.00127	0.00682	2422.62	2204.95	2226.79	2918.36	2961.1	2670.34	Up
St3gal3	1394.2	0.32592	0.1101	2.96025	0.00307	0.01438	1137.78	1351.14	1223.2	1573.73	1527.01	1552.34	Up
Kdm4a	3012.27	0.28851	0.08219	3.5102	0.00045	0.00282	2682.88	2748.29	2705.18	3231.44	3267.34	3438.47	Up
Ptprf	20496.2	0.90401	0.14309	6.31788	2.65E-10	6.53E-09	16984.5	13145.4	12700.9	25667	25126	29353.7	Up
Szt2	3992.94	0.69159	0.08759	7.89541	2.89E-15	1.51E-13	3030.21	2984.02	3147.29	4780.3	4802.8	5213	Up
Med8	3213.87	0.48431	0.10146	4.77339	1.81E-06	2.11E-05	2728.35	2614.13	2695.63	3978.92	3861.88	3404.29	Up
Elovl1	46610.5	1.92474	0.20388	9.44046	3.71E-21	3.59E-19	26653.7	15797.9	15852	73877.4	77868.1	69614	Up
Cdc20	308.801	0.4577	0.15545	2.9444	0.00324	0.01502	266.063	262.563	252.089	391.879	326.312	353.901	Up
Mpl	111.78	-1.648	0.30583	-5.3887	7.10E-08	1.08E-06	119.003	213.692	175.698	58.0561	55.9693	48.2592	Down
Tie1	12937.9	-0.3617	0.11494	-3.147	0.00165	0.00848	14221.3	13153.1	16279.8	11561.5	11821.1	10590.9	Down
Tmem125	885.031	0.34712	0.16233	2.13836	0.03249	0.09729	681.121	888.306	767.726	1148.68	913.462	910.893	Up
Cfap57	1393.95	-0.7253	0.20375	-3.56	0.00037	0.00239	1577.99	1742.11	1891.62	1161.12	737.106	1253.73	Down
Ebna1bp2	2455.84	-0.2255	0.10361	-2.1765	0.02952	0.09079	2590	2463.68	2888.52	2277.66	2200.76	2314.43	Down
Olfr1342	105.567	1.49425	0.43276	3.45283	0.00055	0.00338	30.96	77.619	57.293	124.406	236.55	106.572	Up
Ccdc30	2235.21	-0.4166	0.19611	-2.124	0.03367	0.10012	2292.98	2766.49	2607.79	2135.63	1363.33	2245.06	Down
Ppcs	721.659	0.30748	0.13328	2.307	0.02105	0.06942	696.601	580.705	657.914	851.144	740.274	803.315	Up
Zmynd12	580.392	-0.7477	0.20913	-3.5752	0.00035	0.00227	646.291	828.894	707.568	431.274	328.424	539.9	Down
Frg2f1	164.541	-0.4339	0.20125	-2.156	0.03108	0.09417	172.215	208.9	186.202	152.397	127.779	139.751	Down
Foxo6	150.271	-0.8531	0.2258	-3.7779	0.00016	0.00114	223.493	186.86	169.969	121.296	103.49	96.5185	Down
Foxo6os	15.216	-5.4417	1.24911	-4.3565	1.32E-05	0.00013	24.1875	46.9547	18.1428	0	0	2.0108	Down
Scmh1	3302.34	0.23559	0.08975	2.62507	0.00866	0.03394	3067.95	2915.98	3115.78	3600.51	3724.6	3389.21	Up
Ctps	6322.25	1.85822	0.14187	13.0977	3.39E-39	1.56E-36	3273.06	2359.23	2568.63	9696.4	9637.29	10398.9	Up
Gm8439	33.8264	4.05307	0.58574	6.91961	4.53E-12	1.47E-10	5.80501	1.91652	3.81953	57.0194	77.0899	57.3078	Up
Gm12860	27.3287	5.05189	0.81427	6.20421	5.50E-10	1.27E-08	0	3.83303	0.95488	43.5421	63.3615	52.2808	Up
Cited4	282.109	1.62922	0.28927	5.63212	1.78E-08	3.06E-07	94.8151	176.32	142.278	393.952	556.525	328.766	Up
Kcnq4	352.463	-0.5378	0.14841	-3.6236	0.00029	0.00193	426.668	441.757	383.863	271.62	287.239	303.631	Down
Rims3	203.229	1.3789	0.35627	3.87036	0.00011	0.00082	167.378	97.7424	73.526	255.032	405.514	220.183	Up
Ppt1	5486.31	0.53833	0.11229	4.79394	1.64E-06	1.93E-05	4953.61	4340.91	4128.91	6031.61	6763.84	6698.99	Up
Cap1	16489.2	0.50259	0.10686	4.7031	2.56E-06	2.90E-05	14934.4	13133.9	12869	18923.2	20847	18227.9	Up
Mfsd2a	4079.34	2.9561	0.22103	13.3743	8.54E-41	4.61E-38	1107.79	1067.5	618.764	7019.6	8381.67	6280.74	Up
Mycl	853.508	-0.9162	0.19989	-4.5838	4.57E-06	4.87E-05	1172.61	1250.53	924.327	709.114	456.203	608.268	Down

Hpcal4	497.39	-1.0229	0.33874	-3.0198	0.00253	0.01217	418.928	1059.83	521.366	251.922	322.088	410.204	Down
Macf1	35414.9	-0.4494	0.1069	-4.2041	2.62E-05	0.00023	37857.4	40720.2	44082.2	30435.9	31939.5	27454.5	Down
Gm12922	14.6873	-1.5233	0.62742	-2.4278	0.01519	0.05361	28.0575	20.1234	17.1879	9.33044	7.39218	6.03241	Down
Akirin1	5275.64	0.46794	0.0765	6.11657	9.56E-10	2.12E-08	4372.14	4401.28	4508.96	5955.93	6267.51	6148.03	Up
Rhbdl2	221.346	2.3805	0.3573	6.66239	2.69E-11	7.90E-10	43.5376	117.866	52.5186	345.226	436.138	332.788	Up
Gm12905	649.421	-0.3156	0.12963	-2.4345	0.01491	0.05283	744.009	718.694	698.019	590.928	518.508	626.365	Down
Rragc	5951.43	0.4192	0.11254	3.72494	0.0002	0.00137	5591.19	4841.12	4846.03	6520.94	7436.53	6472.77	Up
Utp11	2190.04	-0.2558	0.10874	-2.3522	0.01866	0.06328	2206.87	2426.31	2518.03	1930.36	1906.13	2152.56	Down
Fhl3	187.989	0.45907	0.22042	2.08273	0.03728	0.10848	124.808	176.32	173.789	199.049	241.83	212.14	Up
Sf3a3	2052.2	-0.2337	0.11368	-2.056	0.03978	0.11401	2213.64	2001.8	2438.77	1812.18	1911.41	1935.4	Down
Gm12915	7.10805	6.32965	1.54	4.11017	3.95E-05	0.00033	0	0	0	10.3672	23.2326	9.04861	Up
Inpp5b	7283.14	0.50184	0.08732	5.74685	9.09E-09	1.67E-07	6103.97	5735.18	6247.8	8501.07	8931.86	8178.94	Up
Cdca8	129.235	0.7575	0.27636	2.74095	0.00613	0.02559	108.36	111.158	68.7516	147.214	148.9	191.026	Up
Dnali1	4026.65	-0.8018	0.21523	-3.7255	0.00019	0.00136	4522.1	5671.93	5159.23	3151.62	1984.27	3670.72	Down
Zc3h12a	1217.76	1.34886	0.16703	8.07566	6.71E-16	3.84E-14	799.156	665.99	594.892	1784.19	1483.72	1978.63	Up
Sh3d21	1306.9	-0.3638	0.16931	-2.149	0.03163	0.09546	1508.33	1192.07	1712.11	1292.78	1041.24	1094.88	Down
Col8a2	1787.08	-0.5397	0.17014	-3.1718	0.00152	0.0079	2143.02	2063.13	2146.58	1485.61	1124.67	1759.45	Down
Tekt2	538.74	-0.9574	0.2053	-4.6633	3.11E-06	3.45E-05	564.053	782.897	786.824	394.989	291.463	412.214	Down
Clspn	72.7447	1.00491	0.36483	2.75443	0.00588	0.02478	67.7251	44.0799	33.4209	101.598	76.0338	113.61	Up
AU04032(	3586.23	0.51443	0.09567	5.37688	7.58E-08	1.15E-06	3033.12	2921.73	2905.71	3933.3	4522.96	4200.56	Up
Zmym1	429.993	-0.8066	0.24314	-3.3174	0.00091	0.00515	456.661	540.458	644.546	325.529	219.653	393.112	Down
Gjb3	210.89	1.68267	0.1958	8.59391	8.41E-18	6.21E-16	108.36	88.1598	104.082	326.565	288.295	349.88	Up
Gjb4	44.4222	2.93636	0.52165	5.62898	1.81E-08	3.12E-07	19.35	5.74955	5.7293	81.9006	83.426	70.3781	Up
A3galt2	76.3819	-0.9108	0.37174	-2.45	0.01428	0.05108	68.6926	87.2015	143.232	44.5788	62.3055	52.2808	Down
Trim62	1632.88	0.43846	0.11974	3.66181	0.00025	0.00169	1392.23	1415.35	1352.11	1811.14	2115.22	1711.19	Up
Azin2	1890.01	-0.4519	0.17088	-2.6445	0.00818	0.03241	2633.54	2059.3	1858.2	1718.87	1355.94	1714.21	Down
Rnf19b	2906.04	0.36249	0.11575	3.13172	0.00174	0.00886	2814.46	2307.49	2506.57	3274.99	3453.2	3079.54	Up
Fndc5	913.384	-3.9563	0.68602	-5.767	8.07E-09	1.49E-07	1185.19	3184.29	779.184	119.222	68.6416	143.772	Down
C77080	4937.78	0.87058	0.1306	6.66592	2.63E-11	7.72E-10	3746.17	3074.09	3654.34	6309.45	7101.77	5740.84	Up
Zbtb8os	1191.32	0.24111	0.10756	2.24177	0.02498	0.07964	1069.09	1120.2	1086.66	1192.22	1370.72	1309.03	Up
Bsdc1	4024.1	0.2598	0.09762	2.66126	0.00778	0.03111	3826.47	3508.18	3653.38	4553.26	4552.53	4050.76	Up
Fam229a	8.50267	2.29569	0.90078	2.54854	0.01082	0.04082	0.9675	4.79129	2.86465	18.6609	12.6723	11.0594	Up
Lck	615.656	-0.5324	0.16774	-3.1738	0.0015	0.00786	808.831	588.371	786.824	517.321	466.763	525.825	Down
Txlna	5276.3	0.23309	0.08235	2.83053	0.00465	0.0204	4941.03	4625.51	4986.4	5555.76	5831.37	5717.71	Up
E330017L	29.5904	-3.7268	0.78808	-4.7289	2.26E-06	2.57E-05	31.9275	102.534	30.5563	5.18358	6.33615	1.0054	Down
Gm12963	178.498	-0.5835	0.18244	-3.1984	0.00138	0.00732	209.948	205.067	227.262	145.14	147.844	135.729	Down
Pef1	4757.86	0.33468	0.10386	3.22246	0.00127	0.00683	4150.58	4054.39	4420.15	5665.65	5437.47	4818.89	Up
Tinagl1	7563.01	-0.3778	0.15726	-2.402	0.0163	0.05676	8736.54	7473.46	9432.33	7055.89	7355.22	5324.6	Down
Serinc2	2254.78	1.20718	0.1918	6.29387	3.10E-10	7.52E-09	1488.98	1453.68	1145.86	3495.81	3594.71	2349.62	Up
Fabp3	2234.91	-3.1855	0.5824	-5.4696	4.51E-08	7.21E-07	2709	7406.38	1966.1	403.282	434.026	490.636	Down
Sdc3	1567.69	0.42537	0.18744	2.26935	0.02325	0.07523	1600.25	978.382	1436.14	1699.18	1789.96	1902.22	Up
Laptm5	9613.63	1.49353	0.25547	5.84625	5.03E-09	9.74E-08	6687.37	5027.98	3401.29	14061	17953.5	10550.7	Up
Ptpru	3703.27	-0.978	0.13332	-7.3361	2.20E-13	8.80E-12	5023.27	4822.92	4891.87	2694.42	2064.53	2722.63	Down
Epb41	8276.91	-0.3662	0.09494	-3.8573	0.00011	0.00086	9068.39	9955.35	8941.52	7687.25	6977.16	7031.77	Down
Gm37711	30.6495	-3.0599	0.63581	-4.8126	1.49E-06	1.77E-05	52.2451	53.6625	58.2479	1.03672	12.6723	6.03241	Down
Oprd1	12.4261	-2.3554	1.05904	-2.2241	0.02614	0.08253	25.155	9.58259	27.6916	2.07343	0	10.054	Down
Ythdf2	2727.68	0.38581	0.09525	4.05068	5.11E-05	0.00042	2458.42	2187.7	2449.27	3019.95	3106.83	3143.89	Up
Gm28874	24.1008	1.56587	0.5372	2.91486	0.00356	0.01629	17.415	6.70781	12.4135	34.2116	30.6247	43.2322	Up
Snhg12	415.628	-0.5388	0.20201	-2.6672	0.00765	0.03066	543.736	368.93	564.336	330.712	364.329	321.728	Down
Med18	267.09	-0.3539	0.16208	-2.1837	0.02899	0.08959	327.015	280.77	291.239	241.555	240.774	221.188	Down
Sesn2	1563.37	0.79773	0.15844	5.03493	4.78E-07	6.32E-06	1354.5	1053.13	1017.91	2254.86	1866	1833.85	Up
Ptafr	380.405	0.83854	0.27489	3.0505	0.00228	0.01115	403.448	223.274	191.931	489.33	486.828	487.619	Up
Rpa2	760.489	-0.3376	0.11613	-2.9068	0.00365	0.01664	851.401	820.269	875.628	703.93	621.999	689.705	Down
Themis2	1343.65	0.92987	0.24725	3.76083	0.00017	0.00121	1220.99	954.426	599.666	1659.78	2087.76	1539.27	Up
Stx12	8683.99	0.3704	0.09503	3.89777	9.71E-05	0.00074	7660.68	7261.68	7803.3	9679.82	10541.2	9157.19	Up
Fgr	1452.95	1.67328	0.24506	6.82814	8.60E-12	2.72E-10	824.311	803.021	453.569	1955.25	2756.23	1925.34	Up
Ahdc1	976.67	-0.2995	0.09823	-3.0488	0.0023	0.01119	1089.41	1070.37	1073.29	898.833	856.437	871.683	Down
Cd164l2	200.469	2.18398	0.23148	9.43496	3.91E-21	3.77E-19	72.5626	73.7859	70.6613	288.207	290.407	407.187	Up
Map3k6	3024.11	-1.4023	0.17954	-7.8102	5.71E-15	2.89E-13	3751	4854.54	4558.61	2080.69	1430.91	1468.89	Down
Tmem222	2282.82	-0.2077	0.0933	-2.2259	0.02602	0.08219	2394.57	2553.76	2391.98	2133.56	2209.21	2013.82	Down
Wdtc1	2212.47	-0.2712	0.09324	-2.9085	0.00363	0.01657	2400.37	2346.78	2512.3	1899.26	2033.9	2082.19	Down
Slc9a1	2828.5	0.4222	0.10044	4.20351	2.63E-05	0.00023	2391.66	2376.48	2484.61	2962.93	3314.86	3440.48	Up
Tent5b	835.256	-0.5449	0.21227	-2.5669	0.01026	0.03904	1042	984.132	947.244	919.567	557.581	561.014	Down
Nudc	6064.42	-0.4317	0.0897	-4.8128	1.49E-06	1.77E-05	6702.85	7070.99	7121.52	5383.67	4828.15	5279.36	Down
Nr0b2	8.82205	-4.6241	1.77039	-2.6119	0.009	0.035	8.70751	42.1634	0	0	1.05603	1.0054	Down
Rps6ka1	4462.36	0.38679	0.13811	2.8006	0.0051	0.02208	4425.35	3763.08	3414.66	5272.74	5414.24	4484.09	Up
Crybg2	281.902	-0.8938	0.24628	-3.6294	0.00028	0.00189	399.578	368.93	331.344	182.462	144.675	264.42	Down
Sh3bgrl3	4010.9	0.52021	0.16663	3.12198	0.0018	0.00911	3654.25	3130.63	3101.46	4852.87	5602.21	3724	Up
Cep85	10371.7	2.52985	0.84756	2.98485	0.00284	0.01342	4705.93	3745.83	733.35	11041	33143.4	8860.6	Up
Cnksr1	1474.87	0.28972	0.09633	3.00746	0.00263	0.01259	1311.93	1337.73	1332.06	1709.54	1614.66	1543.29	Up
Pdik1l	1100.9	0.31614	0.14758	2.14222	0.03218	0.09663	1110.69	909.387	922.417	1135.2	1135.23	1392.48	Up
Trim63	160.026	-5.6873	1.2326	-4.6141	3.95E-06	4.28E-05	147.06	657.365	137.503	1.03672	2.11205	15.081	Down
Slc30a2	33.4583	-1.4516	0.41219	-3.5218	0.00043	0.00271	55.1476	46.9547	44.8795	20.7343	17.9524	15.081	Down
Extl1	178.619	-1.4641	0.20939	-6.9924	2.70E-12	9.04E-11	267.03	290.352	229.172	104.708	93.9863	86.4645	Down
Pafah2	2135.75	0.407	0.10507	3.87354	0.00011	0.00081	1709.58	1939.52	1860.11	2474.64	2552.41	2278.24	Up
Ldlrap1	2245.26	-0.2174	0.08779	-2.4767	0.01326	0.04812	2391.66	2365.94	2484.61	2102.46	2133.17	1993.71	Down
Rsrp1	32007	-0.3749	0.09347	-4.011	6.05E-05	0.00049	36987.6	33560.1	37880.2	28618.5	26296.1	28699.2	Down
Syf2	4816.82	-0.295	0.1025	-2.8779	0.004	0.01799	5594.09	5001.15	5327.29	4603.02	4390.95	3984.4	Down
Runx3	205.677	0.47236	0.22962	2.05709	0.03968	0.11379	210.915	153.321	152.781	205.27	231.27	280.507	Up
Clic4	38761.3	-0.4218	0.1294	-3.2594	0.00112	0.00612	40505.4	43687	48969.3	30895.2	37858.5	30652.7	Down
Ncmap	168.273	1.46765	0.30186	4.86199	1.16E-06	1.41E-05	99.6527	108.283	60.1576	290.28	269.286	181.978	Up
Stpg1	370.847	-1.0579	0.20742	-5.1003	3.39E-07	4.63E-06	447.953	582.621	472.667	269.546	185.86	266.431	Down
Ifnlr1	1726.28	0.48835	0.12025	4.06123	4.88E-05	0.0004	1370.95	1437.39	1502.03	2020.56	2226.1	1800.67	Up

Myom3	442.343	0.77045	0.188	4.0981	4.17E-05	0.00035	352.171	368.93	259.728	553.606	620.943	498.679	Up
Pnrc2	7759.13	0.32295	0.12312	2.62296	0.00872	0.03411	7292.06	7136.15	6254.48	9162.49	9161.02	7548.55	Up
Cnr2	573.281	0.6258	0.17419	3.59256	0.00033	0.00214	521.483	364.138	466.938	651.058	762.45	673.619	Up
Fuca1	10633.3	1.48018	0.13025	11.3638	6.33E-30	1.25E-27	6063.33	5950.79	4820.25	16227.7	16641.9	14095.7	Up
Hmgcl	2676.5	0.27027	0.11003	2.45642	0.01403	0.05037	2703.2	2347.73	2228.7	2969.15	2936.81	2873.44	Up
Gale	1278.46	0.95917	0.17886	5.36267	8.20E-08	1.24E-06	692.731	1060.79	851.756	1815.29	1756.17	1494.03	Up
Eloa	293.448	0.57907	0.19814	2.92245	0.00347	0.01597	274.77	205.067	226.307	342.116	306.247	406.182	Up
Id3	9267.17	-0.3872	0.18901	-2.0487	0.04049	0.11562	9531.82	11551.8	10426.4	9271.35	9061.75	5759.94	Down
Asap3	1027.02	0.45095	0.11619	3.88131	0.0001	0.00079	827.214	837.518	938.65	1260.65	1126.78	1171.29	Up
Ephb2	221.37	-0.7803	0.30191	-2.5844	0.00975	0.03742	216.72	354.556	268.322	153.434	112.995	222.194	Down
C1qb	3078.84	1.0509	0.20744	5.06601	4.06E-07	5.45E-06	2143.98	2365.94	1503.94	3817.19	3493.33	5148.66	Up
C1qc	2532.42	0.93418	0.22821	4.09355	4.25E-05	0.00035	1906.95	2106.25	1206.97	3155.76	2725.6	4092.99	Up
C1qa	2766.61	0.89713	0.21898	4.09693	4.19E-05	0.00035	2226.22	2179.08	1394.13	3267.73	3020.23	4512.24	Up
Zbtb40	879.763	-0.3188	0.12504	-2.5496	0.01078	0.04072	915.256	952.509	1061.83	735.032	844.82	769.132	Down
Wnt4	1552.92	-0.8443	0.20216	-4.1763	2.96E-05	0.00026	1754.08	2006.59	2223.92	990.064	886.005	1456.83	Down
2810405F	53.1627	-1.2041	0.41174	-2.9244	0.00345	0.01587	56.1151	72.8277	93.5785	25.9179	24.2886	46.2484	Down
Rap1gap	4670.43	1.19024	0.12966	9.17996	4.31E-20	3.80E-18	3315.63	2637.13	2585.82	6878.61	6368.89	6236.5	Up
Alpl	733.469	0.58695	0.22328	2.62878	0.00857	0.03365	427.636	580.705	750.538	956.889	733.938	951.109	Up
Ddost	10228.2	0.30288	0.08934	3.39034	0.0007	0.00412	9353.8	8866.77	9254.72	11982.4	11342.8	10568.8	Up
Pink1	7183.12	-0.5415	0.12401	-4.3666	1.26E-05	0.00012	8412.43	9337.27	7796.62	6122.84	6273.85	5155.7	Down
Mul1	1485.76	-0.234	0.09185	-2.5479	0.01084	0.04087	1673.78	1573.46	1570.78	1361.21	1377.06	1358.3	Down
Camk2n1	3948.57	-0.3395	0.08608	-3.9447	7.99E-05	0.00062	4442.77	4511.48	4278.83	3564.23	3588.37	3305.76	Down
Pla2g5	119.094	2.02465	0.67575	2.99614	0.00273	0.01299	15.48	112.116	13.3684	227.041	140.451	206.107	Up
Gm13030	102.353	5.97509	0.59851	9.98332	1.80E-23	2.11E-21	5.80501	3.83303	0	242.592	124.611	237.275	Up
Otud3	449.058	0.59524	0.19368	3.07332	0.00212	0.01044	337.658	376.596	359.036	484.146	457.259	679.651	Up
Tmco4	1085.67	0.46729	0.12967	3.60373	0.00031	0.00207	1037.16	879.681	817.38	1269.98	1223.93	1285.91	Up
Pqlc2	859.187	0.43824	0.13334	3.28671	0.00101	0.00565	776.904	768.523	643.591	942.375	1066.59	957.142	Up
4933427I2	8.07422	-2.2923	0.95363	-2.4037	0.01623	0.05657	6.77251	18.2069	15.2781	3.11015	1.05603	4.0216	Down
Arhgef10l	1405.69	0.51124	0.19525	2.6184	0.00883	0.03449	1051.67	1469.01	956.793	1704.36	1862.83	1389.46	Up
Sdhb	5194.6	-0.616	0.18624	-3.3078	0.00094	0.0053	5353.19	8219.94	5288.14	4395.68	4155.46	3755.17	Down
Mfap2	2056.83	-0.9744	0.18742	-5.1991	2.00E-07	2.84E-06	2613.22	2245.2	3320.13	1666	1216.54	1279.88	Down
Crocc	726.622	-0.4247	0.18569	-2.287	0.02219	0.07239	796.254	737.859	964.432	691.489	491.052	678.646	Down
Necap2	4660.49	0.24893	0.08349	2.98168	0.00287	0.01352	4381.81	4132.01	4264.51	5259.26	4866.16	5059.18	Up
Cplane2	176.485	-0.7983	0.34365	-2.323	0.02018	0.06722	267.03	163.862	241.585	114.039	82.37	190.021	Down
Arhgef19	955.073	-0.4192	0.17881	-2.3445	0.01905	0.06426	1092.31	1027.25	1159.23	930.971	618.831	901.845	Down
Gm13074	93.1541	4.01286	0.53969	7.43542	1.04E-13	4.40E-12	6.77251	11.4991	14.3232	207.343	68.6416	250.345	Up
Hspb7	1904.86	-3.2179	0.57129	-5.6328	1.77E-08	3.06E-07	2331.68	6171.19	1817.14	418.833	411.85	278.496	Down
B330016D	189.371	-1.0177	0.25902	-3.9289	8.53E-05	0.00066	182.858	269.271	308.427	106.782	135.171	133.718	Down
Tmem82	162.524	0.49792	0.23433	2.12486	0.0336	0.09998	143.19	110.2	150.871	224.967	168.964	176.951	Up
Slc25a34	131.397	-1.5896	0.37453	-4.2443	2.19E-05	0.0002	204.143	274.062	113.631	75.6803	46.4651	74.3997	Down
Plekhm2	2992.47	0.41508	0.10949	3.79092	0.00015	0.00108	2535.82	2562.38	2596.33	3310.23	3811.2	3138.86	Up
Dnajc16	1941.86	0.28726	0.11086	2.5911	0.00957	0.03684	1771.5	1747.86	1728.34	1938.66	2114.16	2350.63	Up
Casp9	875.744	0.72357	0.2038	3.55031	0.00038	0.00247	853.336	509.794	618.764	1001.47	1214.43	1056.68	Up
Efhd2	6789.82	0.59746	0.11673	5.11824	3.08E-07	4.23E-06	5790.5	5603.9	4816.43	8145.48	8820.98	7561.62	Up
Fhad1os2	859.516	-0.7074	0.2033	-3.4793	0.0005	0.00312	890.101	1187.28	1121.03	786.867	493.164	678.646	Down
Tmem51	1223.07	0.70325	0.15934	4.41351	1.02E-05	9.94E-05	809.799	1058.88	923.372	1438.96	1756.17	1351.26	Up
Kazn	2651.45	0.36813	0.12674	2.90461	0.00368	0.01673	2399.4	2530.76	2014.8	2775.29	3192.36	2996.09	Up
Pdpn	5787.24	0.52759	0.17116	3.08236	0.00205	0.01018	5603.77	3693.13	4925.29	7416.67	6004.56	7080.03	Up
Pramef12	65.1106	-1.1691	0.5952	-1.9642	0.0495	0.13575	64.8226	91.9928	113.631	70.4967	9.50423	40.216	Down
AAdacl4fn	3.51249	-5.2091	2.46213	-2.1157	0.03437	0.10179	0	19.1652	1.90977	0	0	0	Down
Tnfrsf1b	2882.84	0.88856	0.11912	7.4596	8.68E-14	3.71E-12	2221.38	2058.34	1786.59	3615.03	3980.16	3635.53	Up
Tnfrsf8	106.654	1.47006	0.34257	4.29128	1.78E-05	0.00016	37.7326	63.2451	68.7516	182.462	108.771	178.961	Up
Gm13230	8.20678	2.91435	1.07771	2.70421	0.00685	0.02803	1.935	3.83303	0	22.8077	11.6163	9.04861	Up
Znf41-ps	133.637	1.08792	0.2811	3.87019	0.00011	0.00082	107.393	76.6607	72.5711	158.618	157.348	229.231	Up
Zfp992	212.062	1.01321	0.48615	2.08416	0.03715	0.10823	110.295	248.189	63.0223	176.242	440.363	234.258	Up
Gm13245	12.9533	1.42213	0.70421	2.01949	0.04344	0.12227	8.70751	2.87478	9.54883	17.6242	15.8404	23.1242	Up
Zfp981	39.8518	2.2994	0.75872	3.03065	0.00244	0.0118	13.545	22.0399	4.77441	44.5788	133.059	21.1134	Up
Zfp991	306.687	1.55709	0.20596	7.56035	4.02E-14	1.82E-12	173.183	134.156	159.465	513.174	367.497	492.646	Up
Mfn2	4184.85	-0.5206	0.15776	-3.2996	0.00097	0.00543	4373.11	6130.94	4291.24	3480.26	3331.76	3501.81	Down
2510039C	4621.61	0.25252	0.10409	2.42589	0.01527	0.05381	4182.51	4345.7	4126.05	5121.38	5422.69	4531.34	Up
Nppa	1009.54	-9.9469	1.73783	-5.7238	1.04E-08	1.87E-07	295.088	5691.1	64.932	1.03672	1.05603	4.0216	Down
Agtrap	3488.37	0.40151	0.12244	3.27912	0.00104	0.00576	3206.3	2586.34	3225.59	4157.23	3818.59	3936.14	Up
Fbxo6	2527.13	0.39327	0.13472	2.91921	0.00351	0.0161	2326.84	2205.91	2021.49	2974.34	3187.08	2447.15	Up
Fbxo44	629.976	0.49748	0.20417	2.43661	0.01483	0.05258	655.966	398.636	512.772	784.794	650.512	777.175	Up
Masp2	135.962	-1.1187	0.24433	-4.5786	4.68E-06	4.98E-05	167.378	162.904	228.217	90.1943	87.6501	79.4267	Down
Cort	36.1333	-1.2524	0.48264	-2.5949	0.00946	0.0365	58.0501	27.7895	66.8418	23.8445	22.1765	18.0972	Down
Pgd	11291.3	0.65925	0.15298	4.30931	1.64E-05	0.00015	10788.6	7874.97	7602.78	13209.8	14523.5	13747.9	Up
Kif1b	7397.24	-0.3066	0.08228	-3.7263	0.00019	0.00136	7972.21	8549.58	8019.11	6366.47	6725.83	6750.26	Down
Rbp7	32.3297	-3.6195	0.96458	-3.7525	0.00018	0.00125	21.285	139.906	18.1428	2.07343	10.5603	2.0108	Down
Ctnnbip1	385.381	0.52014	0.14998	3.46812	0.00052	0.00323	338.626	306.643	304.608	438.531	501.612	422.268	Up
Clstn1	14124.3	0.2456	0.11921	2.06016	0.03938	0.11315	14919.8	11750.2	12105.1	15258.4	15489.8	15222.8	Up
Pik3cd	1756.61	0.50807	0.10033	5.0641	4.10E-07	5.50E-06	1503.5	1440.26	1407.5	1989.46	2210.26	1988.68	Up
Tmem201	1213.54	-0.5065	0.116	-4.3662	1.26E-05	0.00012	1280.97	1509.26	1482.93	965.182	1026.46	1016.46	Down
Slc25a33	541.49	-0.4086	0.18262	-2.2372	0.02527	0.0804	600.818	684.197	568.155	489.33	368.553	537.889	Down
Gm16188	233.692	-1.0359	0.29012	-3.5707	0.00036	0.00231	383.131	259.688	299.833	181.425	96.0983	181.978	Down
Spsb1	1593.97	-0.6012	0.1316	-4.5684	4.91E-06	5.20E-05	1968.87	1872.44	1923.13	1235.77	1107.77	1455.82	Down
H6pd	10496.5	0.33826	0.11593	2.91786	0.00352	0.01616	10206.2	9049.79	8558.62	11978.2	12556.1	10630.1	Up
Gm37795	244.134	1.07926	0.18771	5.74968	8.94E-09	1.64E-07	166.41	154.28	149.917	280.95	344.264	368.982	Up
Gpr157	476.708	0.3525	0.16395	2.15001	0.03155	0.0953	435.376	409.176	411.555	521.468	626.223	456.452	Up
Eno1	6420.98	0.56839	0.14662	3.87665	0.00011	0.0008	5427.68	5630.73	4458.35	8693.9	6591.71	7723.49	Up
Slc45a1	2.88177	-4.9234	1.85	-2.6613	0.00778	0.03111	5.80501	7.66607	3.81953	0	0	0	Down
Camta1	1814.75	-0.3869	0.14964	-2.5858	0.00972	0.03731	1788.91	2468.47	1912.63	1513.61	1544.97	1659.92	Down
Thap3	2070.89	-0.4749	0.10739	-4.4219	9.78E-06	9.60E-05	2585.16	2203.99	2436.86	1817.36	1693.86	1688.07	Down

Tnfrsf25	405.215	-0.6535	0.1954	-3.3446	0.00082	0.00473	423.766	545.249	517.547	306.868	260.838	377.025	Down
Espn	606.912	-0.6885	0.12007	-5.7343	9.79E-09	1.78E-07	771.099	776.19	699.929	461.339	459.371	473.544	Down
Hes2	105.184	-2.5866	0.44251	-5.8454	5.05E-09	9.79E-08	300.893	117.866	122.225	29.028	36.9609	24.1296	Down
Acot7	8256.26	1.00889	0.15718	6.41888	1.37E-10	3.57E-09	6783.15	4926.41	4735.26	10475	11791.6	10826.2	Up
Rnf207	129.585	-3.5911	0.55644	-6.4538	1.09E-10	2.88E-09	170.28	436.008	111.721	25.9179	8.4482	25.135	Down
Chd5	135.284	1.07318	0.30497	3.51892	0.00043	0.00274	109.328	90.0763	62.0674	173.132	232.326	144.778	Up
Kcnab2	570.025	0.68529	0.1609	4.25905	2.05E-05	0.00019	504.068	437.924	369.54	686.306	664.24	758.072	Up
Nphp4	609.49	-0.5715	0.22698	-2.5179	0.01181	0.04378	589.208	915.137	681.786	441.641	411.85	617.316	Down
Ajap1	24.4624	1.31105	0.59897	2.18885	0.02861	0.08868	7.74001	16.2904	18.1428	39.3952	17.9524	47.2538	Up
A430005L	941.394	-0.2584	0.11276	-2.2917	0.02192	0.0717	1055.54	978.382	1042.73	842.85	808.915	919.942	Down
Dffb	593.794	-0.4035	0.15676	-2.5744	0.01004	0.03834	752.716	711.028	565.291	515.248	482.604	535.879	Down
Trp73	1203.09	-0.7032	0.26192	-2.6848	0.00726	0.02937	1234.53	1759.36	1478.16	1041.9	554.413	1150.18	Down
Arhgef16	1794.52	0.61178	0.09986	6.12636	8.99E-10	2.00E-08	1388.36	1406.72	1463.84	2318.1	2046.58	2143.51	Up
Prdm16os	293.568	-0.9407	0.28367	-3.3161	0.00091	0.00516	424.733	328.683	404.87	165.875	144.675	292.572	Down
Gm27202	75.9813	-1.7181	0.34046	-5.0463	4.50E-07	6.00E-06	131.58	99.6589	118.405	48.7256	25.3446	32.1728	Down
Gm13110	165.25	-0.9083	0.25077	-3.6221	0.00029	0.00194	185.76	197.401	263.548	113.002	137.283	94.5077	Down
Prxl2b	350.079	-0.6209	0.16078	-3.8617	0.00011	0.00084	480.848	390.97	401.051	290.28	260.838	276.485	Down
Pank4	1897.75	-0.3007	0.10627	-2.8298	0.00466	0.02044	1990.15	2164.71	2129.39	1810.11	1729.77	1562.39	Down
Morn1	1107.29	-0.4697	0.16479	-2.8503	0.00437	0.01935	1267.43	1392.35	1198.38	976.586	740.274	1068.74	Down
Faap20	1586.72	-0.2233	0.11181	-1.9972	0.0458	0.12733	1553.81	1818.77	1755.07	1531.23	1468.93	1392.48	Down
Cfap74	2120.07	-0.7465	0.2081	-3.5873	0.00033	0.00218	2323.94	2988.81	2657.44	1767.6	1103.55	1879.09	Down
Tmem52	87.2048	-1.0097	0.26061	-3.8744	0.00011	0.0008	119.97	114.991	114.586	50.7991	67.5856	55.297	Down
Nadk	10309.8	0.43352	0.08506	5.0966	3.46E-07	4.70E-06	9158.37	8543.83	8614.95	11300.2	12384	11857.7	Up
Cdk11b	3417.7	-0.3611	0.09762	-3.6987	0.00022	0.00149	3539.12	3974.86	4015.28	2943.24	3109.99	2923.71	Down
Mmp23	1104.92	0.55082	0.20575	2.67716	0.00742	0.02991	924.931	828.894	935.785	1707.47	974.711	1257.76	Up
Mib2	2560.46	-0.2081	0.10381	-2.0048	0.04499	0.12561	2861.87	2562.38	2810.22	2549.28	2252.5	2326.5	Down
Fndc10	331.525	0.44001	0.1535	2.86648	0.00415	0.01855	284.445	286.519	273.097	425.053	360.105	359.934	Up
Ssu72	3540.01	0.25913	0.08632	3.00194	0.00268	0.01278	3256.61	3247.54	3164.48	3959.22	3970.66	3641.56	Up
Tmem240	60.1589	-1.1351	0.39108	-2.9023	0.0037	0.01683	102.555	73.7859	71.6162	50.7991	39.0729	23.1242	Down
Atad3a	1742.39	-0.2531	0.11913	-2.1243	0.03365	0.10008	1722.15	1979.76	1982.34	1487.69	1736.11	1546.31	Down
Vwa1	670.618	0.29368	0.14962	1.9628	0.04967	0.1361	574.696	587.413	645.501	675.939	857.493	682.667	Up
Tmem88b	92.1917	-0.8432	0.25173	-3.3497	0.00081	0.00466	118.035	109.241	127.954	69.46	58.0814	70.3781	Down
Ankrd65	1339.43	-0.5357	0.24818	-2.1586	0.03088	0.09373	1282.91	1979.76	1493.44	1161.12	733.938	1385.44	Down
Mxra8	18782.5	-0.5803	0.09101	-6.3759	1.82E-10	4.63E-09	21292.8	24263.1	21972.8	14974.3	15532	14659.8	Down
Cptp	1102.37	0.77557	0.09835	7.88606	3.12E-15	1.62E-13	810.766	836.56	791.598	1412.01	1414.02	1349.25	Up
Acap3	1672.76	0.25671	0.11427	2.2466	0.02467	0.07879	1577.03	1441.22	1554.55	1931.4	1895.57	1636.79	Up
C1qtnf12	731.394	-0.307	0.14385	-2.1345	0.0328	0.09807	922.996	766.607	737.17	701.857	649.456	610.278	Down
Gm37090	533.378	-0.319	0.1462	-2.182	0.02911	0.08986	514.711	602.745	658.869	470.669	464.651	488.625	Down
Rnf223	16.8024	-1.386	0.58163	-2.383	0.01717	0.05912	17.415	28.7478	26.7367	10.3672	9.50423	8.04321	Down
Agrn	14771.9	0.53013	0.0977	5.42592	5.77E-08	9.01E-07	12473	11387	12403.9	17914.4	18397	16056.3	Up
Isg15	608.679	0.94614	0.18397	5.1428	2.71E-07	3.75E-06	480.848	373.721	393.412	879.135	645.231	879.726	Up
AW01173	450.953	-0.462	0.16203	-2.8514	0.00435	0.0193	497.296	477.213	592.982	361.814	424.522	351.89	Down
Perm1	489.898	-1.5513	0.3797	-4.0858	4.39E-05	0.00036	471.173	1288.86	431.607	214.6	257.67	275.48	Down
Pex1	427.482	0.28945	0.13933	2.07736	0.03777	0.10963	406.351	367.971	380.043	450.971	448.811	510.744	Up
Mterf1a	273.194	-0.5113	0.15922	-3.2113	0.00132	0.00706	331.853	321.017	310.337	217.71	247.11	211.134	Down
Cyp51	4808.72	1.69422	0.14177	11.9508	6.43E-33	1.71E-30	2383.92	2571.97	1855.34	7146.08	7759.67	7135.33	Up
Mterf1b	176.284	-0.5423	0.20401	-2.6585	0.00785	0.0313	217.688	202.193	207.21	170.021	129.891	130.702	Down
Fzd1	3184.85	0.47842	0.11157	4.28792	1.80E-05	0.00017	2788.34	2641.92	2554.31	3433.6	4123.78	3567.16	Up
Cldn12	2733.3	0.73916	0.09708	7.61382	2.66E-14	1.23E-12	2209.77	1982.64	1951.78	3311.27	3461.65	3482.71	Up
Steap2	2571.51	1.43978	0.15676	9.18472	4.13E-20	3.65E-18	1400.94	1612.75	1142.04	3382.8	3736.22	4154.32	Up
Steap1	356.477	1.37532	0.20206	6.80645	1.00E-11	3.13E-10	164.475	252.022	178.563	547.386	483.66	512.754	Up
Steap4	2244.42	1.40447	0.2735	5.13519	2.82E-07	3.90E-06	1127.14	1739.24	825.974	2770.1	2735.11	4268.93	Up
Dbf4	523.523	0.63519	0.13042	4.87048	1.11E-06	1.36E-05	436.343	411.093	382.908	674.902	613.551	622.343	Up
Slc25a40	888.164	0.2364	0.10139	2.33171	0.01972	0.06608	829.149	823.144	794.463	949.632	948.311	984.287	Up
Abcb4	59.0149	-1.3878	0.52042	-2.6667	0.00766	0.03068	54.1801	155.238	46.7893	33.1749	28.5127	36.1944	Down
Crot	2958.34	0.49455	0.0975	5.07235	3.93E-07	5.29E-06	2533.89	2529.8	2305.09	3483.37	3268.4	3629.5	Up
Tmem243	7125.89	1.20553	0.11654	10.3447	4.42E-25	5.91E-23	4912.97	4037.14	3981.86	10082.1	9527.46	10213.9	Up
9330182L	779.42	0.56448	0.17997	3.13645	0.00171	0.00873	792.384	543.333	550.967	907.126	893.397	989.314	Up
Sema3a	3486.26	0.3545	0.09981	3.55191	0.00038	0.00246	3329.17	2883.4	2967.78	4044.23	3789.02	3903.97	Up
Gm43719	904.978	0.25208	0.10929	2.30651	0.02108	0.06948	850.434	765.649	862.259	990.064	984.216	977.25	Up
Pclo	303.922	-0.7898	0.30037	-2.6293	0.00856	0.03361	240.908	454.215	460.254	216.674	168.964	282.518	Down
Gm9758	15.2353	4.51007	0.96424	4.67733	2.91E-06	3.24E-05	1.935	1.91652	0	39.3952	19.0085	29.1566	Up
Speer4e	19.5008	3.33335	0.81285	4.10082	4.12E-05	0.00034	3.87001	4.79129	1.90977	43.5421	11.6163	51.2754	Up
Gm10354	45.8995	5.3234	1.61327	3.29976	0.00097	0.00543	0	6.70781	0	110.929	40.129	117.632	Up
Gm17019	5.96883	3.51766	1.21995	2.88344	0.00393	0.01772	0	1.91652	0.95488	10.3672	9.50423	13.0702	Up
Cacna2d1	2231.48	-0.6457	0.11513	-5.6089	2.04E-08	3.46E-07	2714.81	2922.69	2530.44	1631.79	1906.13	1683.04	Down
Hgf	461.01	0.5715	0.20532	2.78348	0.00538	0.02301	443.116	322.933	346.622	434.384	617.775	601.23	Up
Cd36	70373.6	-0.3615	0.1346	-2.686	0.00723	0.02928	84283.9	72883.2	80268.4	63174.4	69674.4	51957.1	Down
Gm43032	36.0674	-0.8771	0.40444	-2.1687	0.03011	0.09204	39.6676	59.412	41.06	24.8812	23.2326	28.1512	Down
4921504A	60.3888	-0.871	0.33499	-2.6001	0.00932	0.03606	69.6601	93.9093	70.6613	33.1749	50.6892	44.2376	Down
Magi2	412.493	-0.9802	0.21389	-4.583	4.58E-06	4.89E-05	504.068	635.325	503.223	299.611	206.981	325.75	Down
Gm29254	4.33784	-3.5432	1.43617	-2.4671	0.01362	0.04919	5.80501	10.5408	7.63906	1.03672	0	1.0054	Down
Ptpn12	8294.56	0.61897	0.12216	5.06701	4.04E-07	5.43E-06	6648.67	6052.36	6924.81	9753.42	11358.6	9029.51	Up
Gsap	8295.35	1.54885	0.1306	11.8599	1.91E-32	4.53E-30	4849.12	3773.62	4055.39	11622.6	13538.2	11933.1	Up
Fam185a	527.771	-0.2791	0.14012	-1.9918	0.04639	0.12867	589.208	588.371	558.606	491.403	419.242	519.792	Down
Fbxl13	1163.65	-0.5828	0.27169	-2.145	0.03195	0.09619	1172.61	1514.05	1500.12	1051.23	535.405	1208.49	Down
Armc10	3162.1	0.84846	0.11251	7.54131	4.65E-14	2.08E-12	2397.47	2115.84	2261.16	3750.84	4464.88	3982.39	Up
Napepld	1054.39	0.95887	0.21622	4.4346	9.22E-06	9.13E-05	805.929	744.567	598.712	1511.53	991.608	1673.99	Up
Pmpcb	3212.2	-0.2569	0.11827	-2.1719	0.02986	0.09156	3320.47	3926.94	3244.69	3048.98	3005.45	2726.65	Down
5031425E	2456.89	-0.3469	0.10456	-3.3176	0.00091	0.00514	2699.33	2558.55	2994.51	2122.16	2251.45	2115.36	Down
Kmt2e	3313.05	0.29102	0.13551	2.14763	0.03174	0.09571	3304.99	2588.26	3047.03	3422.2	3448.98	4066.85	Up
Rint1	603.044	0.48403	0.11771	4.11201	3.92E-05	0.00033	516.646	481.046	510.862	703.93	675.856	729.921	Up
Tomm7	1061.79	-0.4483	0.10874	-4.123	3.74E-05	0.00032	1220.99	1160.45	1294.82	940.301	896.566	857.607	Down

2700038G	74.1017	-0.7987	0.29443	-2.7128	0.00667	0.02739	96.7501	76.6607	108.857	54.9459	59.1374	48.2592	Down
Klhl7	2874.74	-0.2521	0.10658	-2.365	0.01803	0.06156	2964.42	3108.59	3302.94	2627.04	2417.24	2828.19	Down
Kcnh2	854.593	-1.3511	0.18805	-7.1848	6.73E-13	2.49E-11	1085.54	1566.75	1031.27	501.77	485.772	456.452	Down
Nos3	339.038	-1.1628	0.21854	-5.3209	1.03E-07	1.53E-06	344.431	499.253	562.426	210.453	218.597	199.069	Down
Abcb8	1423.37	0.32912	0.12467	2.63993	0.00829	0.03277	1220.02	1400.02	1164.96	1629.72	1671.69	1453.81	Up
Slc4a2	2071.93	0.8168	0.117	6.98105	2.93E-12	9.75E-11	1654.43	1356.89	1490.57	2724.49	2479.55	2725.64	Up
Asb10	66.2244	-4.4446	0.79025	-5.6242	1.86E-08	3.19E-07	62.8876	281.728	35.3307	5.18358	3.16808	9.04861	Down
Chpf2	830.341	0.57056	0.12822	4.44995	8.59E-06	8.58E-05	661.771	625.743	717.117	1059.52	1019.06	898.828	Up
Wdr86	80.218	1.07496	0.40879	2.62962	0.00855	0.03358	81.2701	46.9547	26.7367	115.075	107.715	103.556	Up
Xrcc2	223.286	-0.3902	0.18564	-2.102	0.03556	0.10453	282.51	236.69	240.63	181.425	216.485	181.978	Down
Actr3b	89.8452	-1.7211	0.42187	-4.0795	4.51E-05	0.00037	93.8476	209.859	109.812	35.2483	59.1374	31.1674	Down
Gm21663	2.91372	4.00117	1.72657	2.31741	0.02048	0.06802	0	0	0.95488	6.2203	5.28013	5.027	Up
Gm7347	2.89039	5.03347	2.39233	2.10401	0.03538	0.10411	0	0	0	8.29373	0	9.04861	Up
Gm10220	10.7445	5.94442	1.44302	4.11943	3.80E-05	0.00032	0	0.95826	0	10.3672	17.9524	35.189	Up
Gm7361	149.028	3.00941	1.06399	2.82841	0.00468	0.0205	11.61	77.619	9.54883	245.702	99.2664	450.42	Up
Dpp6	338.671	-2.0378	0.27615	-7.3791	1.59E-13	6.59E-12	422.798	727.318	484.126	128.553	100.322	168.907	Down
Paxip1	2847.57	-0.2524	0.11593	-2.1773	0.02945	0.09065	3279.83	3112.42	2896.16	2679.91	2320.09	2797.03	Down
Insig1	7221.08	1.15878	0.16176	7.16359	7.86E-13	2.85E-11	3947.41	5094.1	4360.95	9749.28	11835.9	8338.79	Up
Cnpy1	248.864	3.95944	0.33249	11.9085	1.07E-32	2.71E-30	19.35	44.0799	26.7367	559.827	328.424	514.765	Up
9530036C	1012.93	0.26873	0.12694	2.11697	0.03426	0.10152	995.559	853.808	907.139	1195.33	1118.33	1007.41	Up
Il6	262.094	3.357	0.75737	4.43242	9.32E-06	9.22E-05	9.67501	78.5772	51.5637	380.475	516.396	535.879	Up
Hadhb	6647.4	-0.5555	0.21407	-2.5948	0.00946	0.0365	6717.36	10898.3	6118.89	5498.74	5828.2	4822.91	Down
Adgrf3	54.4899	2.08332	0.35992	5.7883	7.11E-09	1.33E-07	19.35	25.873	17.1879	75.6803	101.378	87.4699	Up
Selenoi	1838.54	0.20633	0.0891	2.31574	0.02057	0.06821	1658.3	1710.49	1753.16	1978.05	1910.35	2020.86	Up
Drc1	2507.19	-0.8273	0.23732	-3.486	0.00049	0.00305	2828.01	3558.01	3235.14	1985.31	1137.34	2299.35	Down
Fam166c	1060.1	-0.8265	0.21722	-3.8048	0.00014	0.00103	1078.76	1545.67	1442.83	885.355	554.413	853.585	Down
Cib4	52.4489	-1.5936	0.37711	-4.2259	2.38E-05	0.00021	100.62	61.3286	74.4809	22.8077	24.2886	31.1674	Down
Gm9899	205.481	-2.0648	0.24832	-8.3149	9.19E-17	5.83E-15	274.77	397.677	322.75	69.46	69.6977	98.5293	Down
Kcnk3	1565.95	-1.0633	0.24202	-4.3934	1.12E-05	0.00011	1597.34	2984.02	1773.22	1090.63	1111.99	838.504	Down
Slc35f6	1970.73	0.27729	0.11146	2.4877	0.01286	0.04693	1777.3	1854.23	1714.01	2131.49	2369.72	1977.62	Up
Dpysl5	416.389	-1.2247	0.18142	-6.751	1.47E-11	4.49E-10	553.411	513.627	682.741	219.784	250.278	278.496	Down
Mapre3	4735.15	-0.284	0.11554	-2.4579	0.01397	0.05021	4961.35	5484.11	5153.5	4791.7	4134.34	3885.87	Down
Tmem214	3749.09	0.24033	0.09857	2.43822	0.01476	0.05239	3386.26	3376.9	3549.3	4387.38	4023.46	3771.26	Up
Tcf23	438.693	-1.0277	0.27672	-3.7137	0.0002	0.00142	754.651	518.418	492.72	324.492	355.881	185.999	Down
Trim54	154.714	-6.3461	1.01414	-6.2576	3.91E-10	9.27E-09	147.06	642.992	126.999	4.14686	1.05603	6.03241	Down
Nrbp1	6213.07	-0.2136	0.08189	-2.6081	0.0091	0.03533	6392.28	6687.69	6936.27	5884.4	5781.74	5596.06	Down
Krtcap3	1576.31	0.53928	0.12147	4.43948	9.02E-06	8.95E-05	1268.39	1348.27	1238.48	2087.95	1822.7	1692.09	Up
Fndc4	291.624	-0.6881	0.1856	-3.7074	0.00021	0.00145	379.261	399.594	300.788	245.702	212.261	212.14	Down
Gpn1	1364.61	-0.2016	0.0987	-2.0426	0.04109	0.11701	1372.88	1493.93	1512.53	1296.93	1255.61	1255.75	Down
2900076G	7.96621	-2.0356	0.95679	-2.1275	0.03338	0.09942	9.67501	17.2487	11.4586	2.07343	6.33615	1.0054	Down
Fosl2	4720.07	0.40268	0.1021	3.9439	8.02E-05	0.00063	4176.7	4001.69	4018.15	5151.44	5924.3	5048.12	Up
Plb1	716.964	0.4802	0.19675	2.44062	0.01466	0.05208	760.456	459.964	575.794	851.144	871.221	783.207	Up
Pisd	1592.15	0.28051	0.11055	2.53734	0.01117	0.04184	1555.74	1399.06	1358.8	1865.05	1680.14	1694.1	Up
Gm20671	153.742	-0.4591	0.20063	-2.2883	0.02212	0.07222	189.63	160.029	184.292	133.736	134.115	120.648	Down
Depdc5	1407.93	0.41578	0.15117	2.75036	0.00595	0.02502	1423.19	1067.5	1128.67	1461.77	1748.78	1617.69	Up
Slc5a1	1494.26	3.07522	0.19099	16.1018	2.48E-58	4.11E-55	341.528	348.806	260.683	2412.44	2286.29	3315.81	Up
Spon2	699.84	-2.2865	0.75911	-3.0121	0.00259	0.01244	316.373	1367.44	1800.91	235.334	344.264	134.724	Down
Fam53a	1884.69	-0.316	0.10293	-3.0702	0.00214	0.01054	1960.16	2197.29	2113.16	1649.41	1780.46	1607.64	Down
Gm9903	19.3329	-1.8038	0.59088	-3.0527	0.00227	0.01108	19.35	41.2051	29.6014	8.29373	9.50423	8.04321	Down
Tacc3	337.439	0.48868	0.16497	2.96221	0.00305	0.0143	316.373	254.897	271.187	420.907	402.346	358.928	Up
Fgfr3	9899.19	-0.9802	0.13115	-7.474	7.78E-14	3.38E-12	12022.2	12970	14423.5	7304.7	5738.44	6936.26	Down
Letm1	3654.53	0.23376	0.10134	2.3067	0.02107	0.06946	3291.44	3650.01	3135.84	3803.71	4110.05	3936.14	Up
Nsd2	2271.17	0.29633	0.13388	2.21335	0.02687	0.08436	2089.8	1863.81	2162.81	2491.23	2189.14	2830.2	Up
Nelfa	1552.59	-0.2186	0.09478	-2.3059	0.02112	0.06958	1712.48	1640.54	1656.72	1447.26	1493.22	1365.33	Down
Gm42847	4.02812	-3.4038	1.60625	-2.1191	0.03408	0.1011	3.87001	12.4574	5.7293	0	2.11205	0	Down
Nat8l	197.324	-1.2834	0.24486	-5.2411	1.60E-07	2.29E-06	256.388	360.305	222.488	122.332	109.827	112.605	Down
Zfyve28	320.492	0.67516	0.15355	4.39699	1.10E-05	0.00011	264.128	243.398	232.991	365.961	421.354	395.123	Up
Sh3bp2	1147.43	1.35003	0.22152	6.09445	1.10E-09	2.42E-08	815.604	613.286	510.862	1611.06	2031.79	1301.99	Up
Htt	1034.83	0.75663	0.14882	5.08431	3.69E-07	5.00E-06	724.659	751.275	832.658	1088.55	1379.17	1432.7	Up
Rgs12	3080.77	-0.2795	0.11084	-2.5213	0.01169	0.04346	3114.39	3243.71	3776.56	2714.12	2801.64	2834.23	Down
Hgfac	777.275	2.13032	0.14105	15.1034	1.54E-51	1.66E-48	304.763	287.478	275.006	1339.44	1106.71	1350.25	Up
Dok7	183.112	-1.5506	0.36409	-4.2589	2.05E-05	0.00019	172.215	413.009	233.946	89.1576	69.6977	120.648	Down
Acox3	3674.62	0.87572	0.14077	6.22103	4.94E-10	1.14E-08	2915.08	2652.46	2209.6	4592.65	5284.35	4393.6	Up
Htra3	963.841	-1.6317	0.22704	-7.187	6.62E-13	2.45E-11	1208.41	1904.06	1259.49	537.019	511.116	362.95	Down
Ablim2	465.088	-0.7479	0.18607	-4.0195	5.83E-05	0.00047	510.841	705.278	532.825	313.088	389.673	338.82	Down
Sorcs2	4151.95	1.41968	0.13917	10.2013	1.96E-24	2.48E-22	2632.57	2090.92	2054.91	5740.3	5693.03	6699.99	Up
Tada2b	1583.29	0.25121	0.12156	2.06662	0.03877	0.11177	1582.83	1325.27	1429.46	1777.97	1573.48	1810.73	Up
Tbc1d14	3493.1	0.74334	0.117	6.35351	2.10E-10	5.30E-09	2908.31	2564.3	2365.24	4087.77	4581.04	4451.92	Up
D5Ertd579	542.679	0.45703	0.18834	2.42656	0.01524	0.05375	491.491	424.509	456.434	499.697	618.831	765.11	Up
Man2b2	5220.05	0.96477	0.13344	7.22984	4.84E-13	1.84E-11	4131.23	3460.27	3019.34	6938.74	6831.43	6939.28	Up
Ppp2r2c	884.943	-0.7486	0.27164	-2.7557	0.00586	0.02471	873.654	1102.96	1352.11	679.049	417.13	884.753	Down
Evc	794.145	-0.6913	0.13699	-5.0459	4.51E-07	6.00E-06	1113.59	911.304	917.642	643.801	591.374	587.154	Down
Evc2	856.971	-0.5528	0.11619	-4.7576	1.96E-06	2.26E-05	1032.32	985.09	1039.87	725.701	724.433	634.408	Down
Cytl1	1108.54	-1.5498	0.13529	-11.455	2.21E-30	4.49E-28	1542.2	1865.73	1549.77	600.258	574.478	518.787	Down
Nsg1	2390.28	-0.716	0.17091	-4.1893	2.80E-05	0.00025	2423.59	2853.69	3637.15	1791.44	1998	1637.8	Down
Lyar	966.168	-0.2933	0.11611	-2.526	0.01154	0.043	1116.5	997.547	1078.06	805.528	916.63	882.742	Down
Otop1	32.9226	-8.4375	2.51595	-3.3536	0.0008	0.0046	15.48	178.236	3.81953	0	0	0	Down
Wdr1	10251	0.54809	0.09418	5.81937	5.91E-09	1.13E-07	7935.45	8242.94	8802.11	11610.2	12997.6	11918	Up
Rab28	3965.47	-0.2841	0.10559	-2.6904	0.00714	0.02898	4046.09	4814.29	4203.39	3690.71	3555.64	3482.71	Down
Cpeb2	1646.3	-0.2849	0.10662	-2.6716	0.00755	0.03034	1697	1928.97	1799	1405.79	1481.6	1565.41	Down
C1qtnf7	2150.97	-0.6048	0.107	-5.652	1.59E-08	2.76E-07	2743.83	2460.81	2581.05	1734.43	1814.25	1571.44	Down
Cc2d2a	1978.91	-0.4162	0.16059	-2.5916	0.00955	0.03679	2288.14	2408.1	2091.19	1784.19	1344.32	1957.52	Down
Fbxl5	9339.93	0.5927	0.08927	6.63966	3.14E-11	9.04E-10	7704.21	7320.14	7319.18	11288.8	11888.7	10518.5	Up

Bst1	2836.5	2.20842	0.49363	4.47387	7.68E-06	7.79E-05	1639.91	829.852	557.652	4473.43	5684.58	3833.59	Up
Cd38	2392.79	-0.2426	0.11861	-2.0453	0.04083	0.11637	2821.23	2333.36	2625.93	2021.6	2310.58	2244.05	Down
Fgfbp1	812.021	-0.9006	0.25078	-3.5911	0.00033	0.00215	1340.96	934.302	897.59	631.36	381.225	686.689	Down
Prom1	6291.63	1.0494	0.29423	3.56659	0.00036	0.00234	3458.82	4773.09	4065.89	8491.74	4574.7	12385.5	Up
Gm42984	309.993	-0.7274	0.1682	-4.3247	1.53E-05	0.00014	353.138	439.841	366.675	235.334	219.653	245.318	Down
Ldb2	4125.74	-0.3885	0.14047	-2.7654	0.00569	0.02413	4071.25	4577.6	5384.58	3255.29	3934.75	3530.97	Down
Lap3	4429.66	0.86951	0.12306	7.0658	1.60E-12	5.59E-11	3109.55	3559.93	2731.92	5589.97	5690.92	5895.67	Up
Med28	3491.98	-0.203	0.10134	-2.0027	0.04521	0.12605	3607.81	3717.09	3886.37	3220.04	3513.4	3007.15	Down
Ncapg	78.8255	1.11057	0.35344	3.14218	0.00168	0.00859	47.4076	53.6625	48.699	92.2677	77.0899	153.826	Up
Lcorl	754.68	0.32585	0.1459	2.23336	0.02552	0.08097	677.251	634.367	698.019	772.353	769.842	976.244	Up
Kcnip4	1067.33	-0.6002	0.2394	-2.5072	0.01217	0.04489	945.249	1596.46	1316.78	1067.82	833.204	644.462	Down
Adgra3	3794.71	-0.4366	0.08857	-4.93	8.22E-07	1.04E-05	4430.19	4275.75	4387.69	3126.74	3434.19	3113.73	Down
Ppargc1a	405.749	-1.5403	0.3115	-4.9448	7.62E-07	9.70E-06	383.131	849.017	579.614	195.939	157.348	269.447	Down
Sod3	12274.8	-0.4677	0.14996	-3.1191	0.00181	0.00918	13127.1	14036.6	15578	10577.6	11916.2	8413.19	Down
Ccdc149	667.388	0.45438	0.14303	3.17686	0.00149	0.00779	597.916	577.83	513.727	869.805	719.153	725.899	Up
Sepsecs	2603.33	0.35976	0.14467	2.48675	0.01289	0.04702	2575.49	2040.13	2225.83	2892.44	2568.25	3317.82	Up
Pi4k2b	14231.1	1.0766	0.16903	6.36927	1.90E-10	4.83E-09	11427.2	7476.33	8560.53	19458.1	17535.3	20929.4	Up
Slc34a2	158978	1.63363	0.16781	9.73487	2.14E-22	2.31E-20	98074.7	64674.8	69736.1	230305	259557	231523	Up
Sel1l3	837.861	1.987	0.18963	10.478	1.09E-25	1.57E-23	397.643	313.351	301.743	1081.29	1580.87	1352.26	Up
Cckar	4531.6	-0.9408	0.25938	-3.627	0.00029	0.00191	7084.05	5099.85	5693.01	3691.75	1819.53	3801.42	Down
Pcdh7	625.474	-1.244	0.29261	-4.2513	2.13E-05	0.00019	692.731	1315.69	630.223	405.356	420.298	288.55	Down
Gm9954	42.8403	3.15861	0.5966	5.29435	1.19E-07	1.75E-06	1.935	14.3739	9.54883	60.1295	107.715	63.3403	Up
Nwd2	511.316	2.71599	0.38154	7.11857	1.09E-12	3.89E-11	66.7576	110.2	228.217	806.565	1157.4	698.754	Up
0610040J(	2219.01	0.9635	0.15913	6.05474	1.41E-09	3.04E-08	1184.22	1691.33	1637.62	3001.29	2715.04	3084.57	Up
Tbc1d1	2103.14	-0.4106	0.14866	-2.762	0.00574	0.02433	2111.09	2903.52	2186.68	1821.51	1756.17	1839.88	Down
Tlr1	317.705	0.49348	0.19837	2.48775	0.01286	0.04693	303.795	254.897	232.991	306.868	426.634	381.047	Up
Tlr6	148.074	0.8332	0.40311	2.06693	0.03874	0.11173	148.995	119.782	50.6088	158.618	256.614	153.826	Up
Fam114a1	2326.69	-0.4614	0.10034	-4.5984	4.26E-06	4.58E-05	2573.55	2912.15	2601.1	1898.23	2029.68	1945.45	Down
Tmem156	299.597	0.43626	0.20621	2.11563	0.03438	0.1018	299.925	229.982	233.946	314.125	413.962	305.642	Up
Klhl5	18429.2	-0.3994	0.11626	-3.4352	0.00059	0.00358	19183.6	20593.9	23113.9	15451.2	17411.7	14820.6	Down
Klb	19.6113	-1.7032	0.81349	-2.0937	0.03629	0.10628	8.70751	53.6625	27.6916	5.18358	6.33615	16.0864	Down
Rpl9	19921.9	-0.2762	0.10594	-2.6071	0.00913	0.0354	22757.6	19935.6	22775.9	18873.4	18662.1	16526.8	Down
N4bp2os	170.347	-0.4425	0.19826	-2.232	0.02562	0.08116	217.688	198.36	172.834	144.104	137.283	151.816	Down
Chrna9	116.74	-0.9573	0.3185	-3.0057	0.00265	0.01265	129.645	117.866	214.849	89.1576	65.4736	83.4483	Down
Rbm47	2775.61	0.82883	0.12469	6.64726	2.99E-11	8.65E-10	1734.73	2242.33	2021.49	3408.72	3680.25	3566.16	Up
Nsun7	579.844	-0.6005	0.21864	-2.7465	0.00602	0.02525	490.523	759.899	846.026	432.311	444.587	505.717	Down
Apbb2	2742.99	-0.2497	0.09385	-2.661	0.00779	0.03113	2850.26	2914.06	3174.99	2429.03	2585.15	2504.45	Down
Uchl1	347.894	-1.0261	0.20601	-4.981	6.32E-07	8.19E-06	518.581	524.167	357.126	230.151	249.222	208.118	Down
Limch1	32505.9	-0.5676	0.10023	-5.6625	1.49E-08	2.61E-07	40071	36683.1	39702.1	26895.5	27855.8	23828	Down
Gm33167	6.31766	6.16176	1.56557	3.93579	8.29E-05	0.00064	0	0	0	8.29373	9.50423	20.108	Up
Tmem33	5923.88	0.3432	0.07673	4.47261	7.73E-06	7.83E-05	5192.58	5159.26	5315.83	6407.94	6780.74	6686.92	Up
Bend4	255.321	0.41088	0.17212	2.38717	0.01698	0.05868	206.078	208.9	242.54	304.794	305.191	264.42	Up
Atp8a1	1465.03	0.47209	0.14976	3.15234	0.00162	0.00835	1194.86	1134.58	1353.07	1521.9	1584.04	2001.75	Up
Kctd8	347.218	-0.5489	0.21454	-2.5584	0.01051	0.03986	456.661	412.051	368.585	239.481	362.217	244.312	Down
Yipf7	105.53	-5.3272	0.67382	-7.906	2.66E-15	1.39E-13	117.068	397.677	103.127	4.14686	2.11205	9.04861	Down
Guf1	1316.28	-0.2712	0.1078	-2.5161	0.01186	0.04396	1340.96	1555.25	1422.78	1173.56	1178.52	1226.59	Down
Gabra4	60.2886	-0.7598	0.32972	-2.3044	0.0212	0.06979	64.8226	66.1198	96.4432	43.5421	48.5772	42.2268	Down
Gabrb1	82.0614	-0.7654	0.33404	-2.2913	0.02195	0.07176	86.1076	139.906	84.0297	50.7991	59.1374	72.3889	Down
Corin	450.233	-2.432	0.622	-3.91	9.23E-05	0.00071	475.043	1428.76	375.269	109.892	162.628	149.805	Down
Nipal1	520.562	1.27494	0.22062	5.77888	7.52E-09	1.40E-07	406.351	268.312	238.721	659.351	761.394	789.24	Up
Txk	154.416	-0.7926	0.29144	-2.7197	0.00654	0.02696	167.378	215.608	204.345	74.6435	148.9	115.621	Down
Tec	860.251	0.6672	0.13902	4.79924	1.59E-06	1.88E-05	749.814	635.325	609.215	1055.38	1141.56	970.212	Up
Sgcb	2400.77	-0.744	0.12299	-6.0492	1.46E-09	3.14E-08	2839.62	3417.15	2762.48	1876.46	1804.75	1704.15	Down
Gm6116	51.2211	1.99902	0.41004	4.87515	1.09E-06	1.33E-05	22.2525	21.0817	18.1428	96.4146	97.1543	52.2808	Up
Pdgfra	11460.8	-1.0511	0.13011	-8.0789	6.54E-16	3.75E-14	14814.4	14005	17562.2	7896.66	7845.21	6641.68	Down
Kdr	11852.9	-0.4824	0.1529	-3.1548	0.00161	0.00829	13763.7	11688.8	15996.2	8885.69	11317.4	9465.85	Down
Srd5a3	2017.95	0.46559	0.11393	4.08674	4.37E-05	0.00036	1670.88	1800.57	1613.75	2334.68	2551.36	2136.48	Up
Tmem165	6056.02	0.27631	0.07763	3.55938	0.00037	0.00239	5613.44	5485.07	5334.93	6614.25	6816.64	6471.77	Up
Gm7467	12.4743	-2.4709	0.82617	-2.9907	0.00278	0.0132	17.415	30.6643	15.2781	4.14686	6.33615	1.0054	Down
C530008M	591.624	1.58536	0.33555	4.72471	2.30E-06	2.62E-05	252.518	455.173	179.518	759.913	1203.87	698.754	Up
Aasdh	912.258	-0.2302	0.11169	-2.0614	0.03926	0.11285	1001.36	934.302	1018.86	855.291	876.501	787.229	Down
Srp72	5567.21	0.3754	0.08877	4.2287	2.35E-05	0.00021	5181.94	4685.88	4673.2	6361.29	6119.67	6381.28	Up
Thegl	107.235	-0.5835	0.26705	-2.1851	0.02888	0.08937	122.873	145.655	117.451	97.4513	65.4736	94.5077	Down
Hopx	28046.6	-0.5793	0.14721	-3.9352	8.31E-05	0.00064	32532.2	33851.4	34424.5	25532.2	24094.3	17844.9	Down
Rest	1551.02	0.39931	0.13019	3.06721	0.00216	0.01063	1502.53	1219.86	1291	1708.51	1655.85	1928.36	Up
Adgrl3	1969.14	-0.3803	0.1089	-3.4923	0.00048	0.00299	2446.81	2147.46	2087.37	1784.19	1678.02	1670.98	Down
Tecrl	205.732	-1.9437	0.40944	-4.7473	2.06E-06	2.37E-05	292.185	531.834	155.646	89.1576	98.2104	67.3619	Down
Stap1	421.745	0.36577	0.15881	2.30318	0.02127	0.07	413.123	362.222	330.389	428.164	507.948	488.625	Up
Tmprss11a	6.75961	-3.586	1.23027	-2.9148	0.00356	0.01629	7.74001	19.1652	10.5037	1.03672	2.11205	0	Down
Sult1d1	2860.76	-2.743	0.87121	-3.1485	0.00164	0.00845	3071.82	8009.13	3852.95	551.533	191.141	1487.99	Down
Sult1e1	4.49262	-3.6129	1.74028	-2.076	0.03789	0.10988	3.87001	19.1652	1.90977	0	0	2.0108	Down
Jchain	5434.12	2.17045	0.29074	7.46536	8.31E-14	3.58E-12	2359.74	2499.14	1067.56	7339.95	8020.51	11317.8	Up
Rufy3	1438.22	0.38641	0.09378	4.1203	3.78E-05	0.00032	1290.65	1202.61	1247.08	1664.97	1587.21	1636.79	Up
Mob1b	5949.49	0.49001	0.1108	4.42257	9.75E-06	9.57E-05	5391.89	4933.12	4521.37	6637.05	6714.21	7499.29	Up
Dck	876.228	0.83647	0.17003	4.91956	8.67E-07	1.09E-05	739.171	657.365	490.81	1097.88	1152.12	1120.02	Up
Gm9958	112.316	-0.7577	0.24345	-3.1123	0.00186	0.00936	148.995	139.906	134.638	69.46	82.37	98.5293	Down
Gm43449	39.8079	2.02753	0.50202	4.03875	5.37E-05	0.00044	18.3825	13.4156	15.2781	72.5701	32.7368	86.4645	Up
Afm	362.968	1.31607	0.21248	6.19396	5.87E-10	1.34E-08	212.85	182.069	229.172	613.736	397.066	542.916	Up
Rassf6	607.016	0.83777	0.12493	6.70607	2.00E-11	5.97E-10	416.026	475.296	415.374	788.941	758.226	788.234	Up
Ppbp	1151.34	-1.636	0.30696	-5.3298	9.83E-08	1.46E-06	951.054	2527.89	1747.44	497.624	685.36	498.679	Down
Pf4	652.816	-0.6908	0.18311	-3.7728	0.00016	0.00116	690.796	865.308	862.259	566.047	534.349	398.139	Down
Cxcl3	170.977	5.01859	0.48282	10.3942	2.63E-25	3.67E-23	5.80501	17.2487	7.63906	258.142	211.205	525.825	Up
Cxcl15	115297	0.82904	0.11314	7.32723	2.35E-13	9.39E-12	90463.3	72913.9	85780	145999	155454	141174	Up

Gm43085	17787.6	1.34943	0.12506	10.7901	3.84E-27	6.46E-25	9775.63	10066.5	10237.3	24756.8	29545.5	22344	Up
Cxcl1	367.718	2.20499	0.2466	8.94148	3.84E-19	3.11E-17	83.2051	162.904	147.052	651.058	583.982	578.105	Up
Cxcl2	228.368	3.38851	0.46012	7.36443	1.78E-13	7.23E-12	75.4651	26.8312	17.1879	435.421	522.733	292.572	Up
Epgn	22.4873	2.59321	0.58896	4.403	1.07E-05	0.0001	2.9025	6.70781	9.54883	34.2116	44.3531	37.1998	Up
Gm19610	8.37538	2.46921	0.94567	2.61107	0.00903	0.03506	1.935	4.79129	0.95488	14.514	19.0085	9.04861	Up
Ereg	80.1182	2.88261	0.70827	4.06993	4.70E-05	0.00039	1.935	13.4156	42.0148	131.663	191.141	100.54	Up
Areg	1241.48	1.02685	0.23082	4.44861	8.64E-06	8.62E-05	1105.85	557.707	788.733	1561.29	1821.64	1613.67	Up
Parm1	5229.19	2.03643	0.19215	10.5981	3.04E-26	4.59E-24	1643.78	2489.56	2015.76	8450.27	9975.22	6800.53	Up
Thap6	861.572	0.58864	0.14891	3.95307	7.72E-05	0.0006	607.591	717.736	739.079	1052.27	1147.9	904.861	Up
G3bp2	7455.71	0.35853	0.08292	4.32355	1.54E-05	0.00014	6668.99	6609.11	6324.19	8096.75	8235.94	8799.27	Up
Uso1	6423.28	0.30403	0.08222	3.6976	0.00022	0.00149	5569.91	5620.19	6056.82	7207.25	6960.26	7125.28	Up
Naaa	7401.97	0.74189	0.10219	7.25987	3.87E-13	1.49E-11	6110.74	5346.13	5162.1	9355.32	9489.44	8948.07	Up
Cxcl9	76.9487	1.33851	0.5661	2.36442	0.01806	0.06163	63.8551	52.7042	14.3232	111.965	55.9693	162.875	Up
Art3	2048.58	-1.9848	0.31574	-6.2863	3.25E-10	7.85E-09	2284.27	5398.83	2129.39	836.63	724.433	917.931	Down
Scarb2	23108.6	0.56145	0.1275	4.4036	1.06E-05	0.0001	21890.7	17350.2	16763	26630.1	28095.6	27922	Up
Fam47e	1971.78	-0.7915	0.23587	-3.3558	0.00079	0.00458	2203.97	2708.04	2586.78	1644.23	907.126	1780.56	Down
Sowahb	445.639	1.22641	0.21141	5.801	6.59E-09	1.24E-07	329.918	245.314	225.352	545.313	750.834	577.1	Up
Ccng2	3079.47	-0.2916	0.11703	-2.4919	0.01271	0.04646	3055.37	3667.26	3446.17	2924.58	2842.82	2540.65	Down
Cxcl13	585.825	1.5801	0.58863	2.68437	0.00727	0.0294	205.11	488.712	187.157	909.2	472.043	1252.73	Up
Gm7993	656.13	-1.1139	0.29334	-3.7973	0.00015	0.00106	478.913	1176.74	1037	409.503	401.29	433.328	Down
Anxa3	24934.7	0.59388	0.11009	5.39452	6.87E-08	1.05E-06	18756	20320.8	20544.3	29879.2	33139.1	26968.9	Up
Bmp2k	545.945	0.39321	0.14504	2.71109	0.00671	0.02752	437.311	510.752	467.893	552.57	673.744	633.403	Up
Antxr2	4715.07	-0.2915	0.1088	-2.6789	0.00739	0.02982	5006.82	5609.65	4952.98	4024.53	4063.59	4632.89	Down
Prdm8	97.1205	0.99255	0.32038	3.09808	0.00195	0.00975	55.1476	76.6607	63.0223	118.186	173.188	96.5185	Up
Cfap299	1183.44	-0.6012	0.22825	-2.6339	0.00844	0.03321	1129.07	1751.7	1398.9	1054.34	673.744	1092.87	Down
Bmp3	2720.83	-0.7058	0.10141	-6.9592	3.42E-12	1.13E-10	3375.61	3498.6	3245.65	2140.82	2170.13	1894.18	Down
Prkg2	1085.88	-0.9982	0.23691	-4.2134	2.52E-05	0.00022	1424.16	1388.52	1528.77	758.876	951.479	463.49	Down
Gm43251	25.809	-1.8842	0.53703	-3.5086	0.00045	0.00284	36.7651	29.706	55.3832	7.25701	12.6723	13.0702	Down
Vamp9	32.2256	-1.1203	0.42729	-2.6218	0.00875	0.0342	35.7976	51.746	44.8795	24.8812	17.9524	18.0972	Down
Hnrnpdl	11770	-0.3484	0.08477	-4.11	3.96E-05	0.00033	12827.1	12888.6	13837.2	9959.73	10816.9	10290.3	Down
Coq2	3823.37	-0.2264	0.10027	-2.2581	0.02394	0.07697	3875.81	4524.9	3967.54	3560.08	3528.18	3483.71	Down
Hpse	656.745	0.36086	0.15376	2.34695	0.01893	0.06397	519.548	675.572	529.96	788.941	708.593	717.856	Up
Abraxas1	516.186	-0.3432	0.13037	-2.6327	0.00847	0.03331	538.898	620.952	571.975	442.678	475.211	447.403	Down
Arhgap24	1738.25	-0.3151	0.13693	-2.3012	0.02138	0.07026	1684.42	2146.5	1950.83	1511.53	1706.54	1429.68	Down
Mapk10	529.541	-4.7908	0.76416	-6.2694	3.62E-10	8.64E-09	574.696	2048.76	443.066	34.2116	23.2326	53.2862	Down
Slc10a6	2762.77	-0.3171	0.1195	-2.6538	0.00796	0.03168	2778.66	3005.1	3411.8	2643.63	2369.72	2367.72	Down
Aff1	6596.98	0.35595	0.08324	4.27636	1.90E-05	0.00017	5912.4	5555.98	5893.54	7112.91	7421.75	7685.28	Up
Klhl8	641.794	-0.345	0.14193	-2.4308	0.01507	0.05324	717.886	734.984	701.839	507.991	538.573	649.489	Down
Hsd17b11	8927.89	0.62092	0.13577	4.5732	4.80E-06	5.10E-05	7866.75	6247.85	6992.61	10577.6	12205.5	9676.98	Up
Nudt9	3260.66	0.42395	0.10027	4.22829	2.35E-05	0.00021	2885.09	2740.62	2729.06	3824.44	3965.38	3419.37	Up
Spp1	32631.9	1.69907	0.21046	8.07292	6.86E-16	3.91E-14	19877.3	15180.7	11044.2	53726.8	54363.1	41599.5	Up
Pkd2	8246.48	-0.409	0.10329	-3.9599	7.50E-05	0.00059	10218.8	8938.64	9065.66	7253.9	7418.58	6583.36	Down
Zfp951	187.512	-0.5635	0.20204	-2.789	0.00529	0.02271	218.655	204.109	248.27	129.589	161.572	162.875	Down
Gm32051	9.24038	-1.9611	0.83965	-2.3357	0.01951	0.06552	9.67501	21.0817	13.3684	5.18358	2.11205	4.0216	Down
Gm42141	27.6484	1.2588	0.58347	2.15744	0.03097	0.09399	5.80501	24.9147	18.1428	39.3952	46.4651	31.1674	Up
Lrrc8c	7178.54	0.93242	0.1503	6.20375	5.51E-10	1.27E-08	5368.67	4112.85	5327.29	8595.41	10787.3	8879.7	Up
Lrrc8d	2890.81	0.55208	0.11156	4.94878	7.47E-07	9.53E-06	2391.66	2295.03	2346.15	3305.05	3844.99	3161.99	Up
Cdc7	287.855	-0.4794	0.19238	-2.4919	0.01271	0.04646	307.665	313.351	384.818	209.417	233.382	278.496	Down
Tgfbr3	6030.57	-0.933	0.07948	-11.739	8.04E-32	1.83E-29	7725.5	7937.26	8083.08	4069.11	4310.7	4057.8	Down
Brdt	257.966	-0.5086	0.1945	-2.6147	0.00893	0.03477	348.301	308.559	252.089	192.829	222.821	223.199	Down
Lpcat2b	21.9729	-1.5808	0.52738	-2.9974	0.00272	0.01294	26.1225	30.6643	42.0148	13.4773	9.50423	10.054	Down
Dipk1a	1365.31	0.4413	0.15756	2.80082	0.0051	0.02207	1225.82	1243.82	1004.54	1554.04	1817.42	1346.23	Up
Mtf2	310.065	0.41599	0.17321	2.40165	0.01632	0.05679	284.445	231.899	280.736	317.235	357.993	388.085	Up
Tmed5	5419.39	0.36623	0.12248	2.9901	0.00279	0.01322	4902.33	4769.25	4533.78	5672.91	7028.9	5609.13	Up
Gm43081	9.91287	-1.9259	0.79938	-2.4092	0.01599	0.05587	20.3175	8.62433	18.1428	4.14686	4.2241	4.0216	Down
Gm42517	87.6954	-0.8806	0.2667	-3.3017	0.00096	0.0054	116.1	106.367	118.405	64.2764	70.7537	50.27	Down
Gm10419	253.189	-1.7743	0.19916	-8.9091	5.15E-19	4.16E-17	417.961	370.846	386.728	90.1943	126.723	126.681	Down
Pde6b	74.9005	-0.663	0.2896	-2.2893	0.02206	0.0721	88.0426	96.7841	90.7139	57.0194	46.4651	70.3781	Down
Atp5k	258.872	-0.5759	0.18284	-3.1495	0.00164	0.00842	305.73	357.43	266.412	204.233	213.317	206.107	Down
AC158912	49.0576	1.00171	0.44846	2.23368	0.0255	0.08092	45.4726	28.7478	23.8721	77.7537	39.0729	79.4267	Up
Mfsd7a	524.286	1.01716	0.14261	7.13237	9.87E-13	3.55E-11	391.838	312.392	336.119	678.012	706.481	720.872	Up
Gak	6424.31	0.4755	0.09995	4.75737	1.96E-06	2.26E-05	5706.32	5169.81	5248.99	7535.89	7967.71	6917.16	Up
Fgfrl1	1311.05	-0.3498	0.15962	-2.1913	0.02843	0.08824	1502.53	1679.83	1225.11	1227.47	1223.93	1007.41	Down
Gm20629	84.5609	-1.4662	0.31624	-4.6364	3.55E-06	3.89E-05	131.58	122.657	118.405	31.1015	43.297	60.3241	Down
Vmn2r10	13.117	-2.2866	0.7723	-2.9607	0.00307	0.01436	28.0575	10.5408	26.7367	3.11015	4.2241	6.03241	Down
Vmn2r13	4.3434	-3.5565	1.57143	-2.2633	0.02362	0.07614	12.5775	3.83303	7.63906	0	0	2.0108	Down
Crlf2	1223.72	0.86623	0.14897	5.81464	6.08E-09	1.15E-07	831.084	896.93	872.763	1573.73	1840.65	1327.13	Up
Zfp932	981.554	-0.2453	0.11609	-2.113	0.0346	0.10235	1087.47	987.006	1120.08	882.245	855.381	957.142	Down
Plcxd1	38.1438	1.41312	0.445	3.17556	0.0015	0.00782	29.025	11.4991	21.9623	57.0194	58.0814	51.2754	Up
Zfp605	175.554	0.57345	0.2478	2.31416	0.02066	0.06841	136.418	110.2	176.653	217.71	233.382	178.961	Up
Chfr	639.585	0.50449	0.16803	3.00239	0.00268	0.01277	588.241	485.837	512.772	695.636	664.24	890.785	Up
Ankle2	2490.12	0.24617	0.11327	2.1733	0.02976	0.0913	2553.24	2102.42	2179.04	2681.98	2640.06	2783.96	Up
Pxmp2	444.574	-0.5275	0.19094	-2.7627	0.00573	0.02429	447.953	613.286	513.727	406.393	302.023	384.063	Down
Pole	134.909	0.52871	0.23851	2.21667	0.02665	0.08376	129.645	100.617	101.218	166.911	134.115	176.951	Up
P2rx2	101.11	1.84098	0.39426	4.66945	3.02E-06	3.36E-05	27.09	69.9529	35.3307	119.222	171.076	183.988	Up
Lrcol1	48.338	2.67131	0.3949	6.76455	1.34E-11	4.11E-10	11.61	12.4574	15.2781	68.4232	89.7622	92.4969	Up
Galnt9	65.194	-1.5322	0.4829	-3.1729	0.00151	0.00788	155.768	61.3286	73.526	22.8077	48.5772	29.1566	Down
Ttc28	1749.29	-0.5869	0.12527	-4.6852	2.80E-06	3.13E-05	2019.18	2073.67	2207.69	1407.86	1559.75	1227.59	Down
Mn1	295.925	-1.0603	0.15947	-6.6488	2.95E-11	8.58E-10	372.488	421.634	405.825	201.123	198.533	175.945	Down
Miat	20.5348	-2.0857	0.73738	-2.8286	0.00468	0.02049	24.1875	48.8712	26.7367	6.2203	2.11205	15.081	Down
Tpst2	3421.3	-0.3239	0.09956	-3.2532	0.00114	0.00625	3626.2	3680.67	4104.09	3067.64	3160.68	2888.52	Down
Hps4	760.192	0.22919	0.11035	2.0769	0.03781	0.10971	704.341	685.155	710.433	833.52	775.123	852.58	Up
Sez6l	29.3989	1.54245	0.75992	2.02976	0.04238	0.11989	14.5125	10.5408	20.0525	12.4406	25.3446	93.5023	Up

Myo18b	242.063	-3.6721	0.48972	-7.4983	6.46E-14	2.85E-12	259.29	795.355	292.194	40.4319	15.8404	49.2646 Down
Grk3	6040.09	0.67137	0.11379	5.89998	3.64E-09	7.25E-08	4135.1	5040.44	4803.06	7420.81	6991.94	7849.16 Up
Crybb3	158.245	-0.7589	0.30428	-2.4941	0.01263	0.04625	159.638	226.149	211.029	73.6068	137.283	141.762 Down
2900026A	3298.49	-0.3818	0.1353	-2.8221	0.00477	0.02087	3226.62	3620.3	4350.45	2760.77	2840.71	2992.07 Down
Sgsm1	820.191	0.92033	0.14778	6.22762	4.74E-10	1.10E-08	620.168	596.037	485.081	1119.65	1131	969.206 Up
Cmklr1	2091.01	-0.57	0.16413	-3.473	0.00051	0.00318	2365.54	2698.46	2432.09	1670.15	2027.57	1352.26 Down
Iscu	5244.66	-0.2599	0.10169	-2.5556	0.0106	0.04013	5405.43	6094.53	5647.18	5075.76	4811.25	4433.82 Down
Selplg	2609.83	0.7298	0.14667	4.97583	6.50E-07	8.39E-06	2320.07	1903.1	1667.23	3078.01	3494.39	3196.17 Up
Coro1c	5892.57	0.68873	0.13374	5.14972	2.61E-07	3.62E-06	4945.87	3898.2	4692.29	7075.59	8080.71	6662.79 Up
Ssh1	3572.86	0.4288	0.09666	4.43614	9.16E-06	9.08E-05	3081.49	2908.32	3147.29	4067.04	4379.34	3853.7 Up
Dao	67.0422	4.1737	0.45777	9.11745	7.69E-20	6.56E-18	3.87001	10.5408	6.68418	136.846	146.788	97.5239 Up
Usp30	1473.67	0.22686	0.09915	2.28804	0.02213	0.07224	1453.19	1303.23	1317.74	1582.03	1609.38	1576.47 Up
Foxn4	72.5523	1.04431	0.36547	2.85743	0.00427	0.01898	52.2451	56.5373	33.4209	111.965	66.5296	114.616 Up
Myo1h	585.355	-0.9081	0.19587	-4.6362	3.55E-06	3.89E-05	604.688	898.847	787.778	429.2	343.208	448.409 Down
Ube3b	6320.4	0.23122	0.07976	2.89878	0.00375	0.017	5944.33	5692.06	5808.55	6579	7084.87	6813.6 Up
Mmab	1288.84	0.24069	0.10901	2.20791	0.02725	0.08526	1152.29	1261.07	1131.54	1390.24	1318.98	1478.94 Up
Mvk	1600.09	0.80651	0.11508	7.00834	2.41E-12	8.17E-11	1159.07	1200.7	1132.49	2167.77	2125.78	1814.75 Up
Fam222a	337.679	-0.7084	0.16633	-4.2592	2.05E-05	0.00019	420.863	447.507	388.637	279.913	220.709	268.442 Down
Trpv4	895.767	-0.7998	0.19799	-4.0396	5.36E-05	0.00044	860.109	1423.97	1129.63	597.148	658.96	704.786 Down
Oasl2	3606.08	0.84601	0.18637	4.53935	5.64E-06	5.89E-05	2785.44	2902.57	2046.31	4883.97	3686.58	5331.64 Up
Oasl1	293.501	0.74881	0.19632	3.81417	0.00014	0.001	247.68	204.109	205.3	418.833	302.023	383.058 Up
Hnf1a	12.5457	2.19791	1.01952	2.15584	0.0311	0.09419	6.77251	6.70781	0	35.2483	8.4482	18.0972 Up
Hnf1aos1	46.251	2.22149	0.51489	4.31454	1.60E-05	0.00015	22.2525	13.4156	13.3684	80.8638	38.0169	109.589 Up
Unc119b	4299.16	-0.2378	0.10497	-2.2654	0.02349	0.07583	4379.88	4830.58	4747.68	4269.2	3627.45	3940.17 Down
Mlec	12073.6	0.21845	0.06966	3.13605	0.00171	0.00874	11098.2	11210.7	11175	12727.8	13157	13073.2 Up
Gm13830	116.458	-0.8631	0.28183	-3.0623	0.0022	0.01077	129.645	143.739	177.608	76.717	65.4736	105.567 Down
Pla2g1b	480.088	0.55627	0.18638	2.98465	0.00284	0.01342	433.441	301.851	430.652	603.369	592.43	518.787 Up
Sirt4	980.444	-0.2593	0.12312	-2.106	0.0352	0.10379	1064.25	1004.26	1136.31	972.439	886.005	819.402 Down
Bicdl1	1143.99	0.67721	0.11388	5.94658	2.74E-09	5.57E-08	935.574	841.351	864.169	1474.21	1297.86	1450.79 Up
Cit	388.396	-0.4026	0.18772	-2.1445	0.032	0.09628	408.286	527.042	391.502	343.153	290.407	369.988 Down
Prkab1	1345.02	0.48533	0.0971	4.99843	5.78E-07	7.55E-06	1140.68	1117.33	1104.8	1602.76	1485.83	1618.7 Up
Ccdc60	687.27	-0.5252	0.26274	-1.9991	0.04559	0.12684	631.778	962.092	839.342	590.928	368.553	730.926 Down
Hspb8	5326.59	-0.4945	0.15729	-3.1439	0.00167	0.00855	5680.2	7676.61	5334.93	4243.28	4740.5	4284.01 Down
Gstm2-ps	859.297	-1.407	0.19232	-7.3157	2.56E-13	1.02E-11	1469.63	1105.83	1168.78	460.302	380.169	571.068 Down
Taok3	4183.35	0.80708	0.11518	7.00711	2.43E-12	8.21E-11	3242.1	2809.61	3076.63	4935.8	5886.29	5149.66 Up
Pebp1	8172.2	-0.2349	0.09754	-2.4085	0.01602	0.05595	8712.35	9221.32	8574.85	7999.3	6886.34	7639.04 Down
BC051077	6.60054	2.29195	0.97319	2.35509	0.01852	0.06287	1.935	2.87478	1.90977	13.4773	6.33615	13.0702 Up
Vsig10	1927.82	0.40234	0.08663	4.64436	3.41E-06	3.75E-05	1657.33	1627.12	1697.78	2136.67	2204.98	2243.05 Up
Wsb2	4522.58	0.37873	0.0964	3.92886	8.53E-05	0.00066	4093.5	3880.95	3822.4	5218.83	5399.46	4720.36 Up
Rfc5	771.052	0.31205	0.12725	2.45238	0.01419	0.05084	748.846	618.077	697.065	894.686	832.148	835.488 Up
Gm15728	268.142	-0.5415	0.23395	-2.3144	0.02065	0.06838	336.691	348.806	268.322	224.967	162.628	267.437 Down
Nos1	17.8579	-3.0863	1.01817	-3.0312	0.00244	0.01178	12.5775	66.1198	17.1879	0	4.2241	7.03781 Down
Tesc	128.153	-0.6535	0.29834	-2.1903	0.0285	0.08843	107.393	198.36	164.24	110.929	104.547	83.4483 Down
Tbx3os1	616.857	-0.7942	0.19939	-3.9833	6.80E-05	0.00054	641.453	744.567	961.567	399.136	425.578	528.841 Down
Tbx3	5245.8	-0.4087	0.14906	-2.7419	0.00611	0.02554	5463.48	5465.91	7022.21	4088.81	5132.28	4302.11 Down
1700021F	62.6614	-1.5555	0.37751	-4.1205	3.78E-05	0.00032	91.9126	108.283	80.2102	31.1015	44.3531	20.108 Down
Gm43050	268.932	-0.9517	0.16451	-5.7853	7.24E-09	1.35E-07	344.431	346.89	372.404	200.086	165.796	183.988 Down
Tbx5	1828.92	-0.7775	0.16022	-4.8525	1.22E-06	1.47E-05	1996.92	2827.82	2105.52	1320.78	1444.64	1277.86 Down
Sds	60.0612	-1.9409	0.51719	-3.7528	0.00017	0.00125	55.1476	169.612	61.1125	22.8077	29.5687	22.1188 Down
Plbd2	3425.72	0.25446	0.11948	2.12966	0.0332	0.09905	3498.49	3032.89	2841.73	3735.29	3962.21	3483.71 Up
Tpcn1	2750.93	-0.3445	0.08021	-4.2948	1.75E-05	0.00016	3027.31	3069.3	3136.79	2467.38	2410.91	2393.86 Down
Rita1	430.771	-0.2861	0.13155	-2.1746	0.02966	0.09107	472.141	484.879	463.118	398.099	362.217	404.171 Down
Cfap73	216.295	-0.4995	0.20171	-2.4764	0.01327	0.04816	231.233	273.104	255.909	187.646	146.788	203.091 Down
Dtx1	688.28	-0.4206	0.19553	-2.1514	0.03145	0.09506	852.369	820.269	691.335	636.544	442.475	686.689 Down
Oas2	1953.15	1.41116	0.56218	2.51013	0.01207	0.04456	949.119	1808.23	444.975	3059.35	2199.7	3257.5 Up
Oas3	353.762	2.19218	0.38603	5.6788	1.36E-08	2.40E-07	94.8151	217.525	68.7516	565.01	443.531	732.937 Up
Oas1c	80.2777	0.92553	0.2753	3.36184	0.00077	0.0045	57.0826	63.2451	45.8344	105.745	97.1543	112.605 Up
Oas1h	99.754	1.20245	0.4289	2.80361	0.00505	0.02191	41.6026	91.9928	47.7441	120.259	90.8182	206.107 Up
Oas1g	393.104	1.67794	0.22558	7.43835	1.02E-13	4.32E-12	170.28	236.69	154.691	648.984	478.379	669.597 Up
Oas1a	1239.76	1.47295	0.21038	7.00154	2.53E-12	8.52E-11	572.761	887.348	509.907	1946.95	1691.75	1829.83 Up
Oas1d	1.88621	4.41731	2.12293	2.08076	0.03746	NA	0	0	0	5.18358	2.11205	4.0216 Up
Rph3a	12.4011	4.63282	1.06625	4.34495	1.39E-05	0.00013	0.9675	1.91652	0	25.9179	28.5127	17.0918 Up
Naa25	212.381	0.59337	0.17897	3.31556	0.00091	0.00517	176.085	159.071	172.834	231.188	256.614	278.496 Up
Erp29	3862.53	0.40125	0.0979	4.09847	4.16E-05	0.00035	3210.17	3312.7	3463.36	4518.01	4621.17	4049.75 Up
Tmem116	1588.87	0.85442	0.15617	5.47092	4.48E-08	7.16E-07	1363.21	1021.5	1010.27	2091.06	2190.2	1856.98 Up
Aldh2	44851.6	-0.5248	0.11026	-4.7593	1.94E-06	2.25E-05	57017.8	50874	50868.5	40367.6	33428.5	36553.4 Down
Acad12	998.686	-0.729	0.17708	-4.1165	3.85E-05	0.00033	1111.66	1542.8	1082.84	831.446	686.416	736.959 Down
Brap	2851.09	0.23815	0.09681	2.46001	0.01389	0.04998	2833.81	2468.47	2546.67	3065.57	3119.5	3072.51 Up
Cux2	1045.34	1.45226	0.12173	11.9303	8.23E-33	2.11E-30	568.891	595.079	514.682	1422.37	1635.78	1535.25 Up
Ppp1cc	5562.31	0.23585	0.08725	2.70324	0.00687	0.02809	5361.89	4826.75	5137.27	6163.28	6057.36	5827.3 Up
Hvcn1	188.117	2.0753	0.33578	6.18059	6.39E-10	1.45E-08	111.263	55.579	49.6539	284.06	372.777	255.372 Up
Tctn1	566.198	0.50285	0.13924	3.61134	0.0003	0.00201	467.303	460.922	477.441	704.967	579.758	706.797 Up
Rad9b	242.86	-0.506	0.2248	-2.2507	0.0244	0.07808	250.583	317.184	287.42	217.71	151.012	233.253 Down
Gpn3	2137.26	0.61339	0.12683	4.83622	1.32E-06	1.58E-05	1868.25	1567.71	1632.85	2716.2	2737.22	2301.36 Up
Arpc3	9693.68	0.47562	0.09633	4.93716	7.93E-07	1.00E-05	7733.24	7883.59	8713.31	11284.7	11873.9	10673.3 Up
Atp2a2	39750.3	-1.0926	0.46355	-2.357	0.01842	0.06264	38236.6	88718.5	35409.9	23994.8	25590.7	26551.6 Down
Gm10064	18.8484	-1.4924	0.66646	-2.2393	0.02514	0.08007	24.1875	34.4973	24.827	7.25701	4.2241	18.0972 Down
P2rx4	7749.39	0.43072	0.12238	3.5196	0.00043	0.00273	5822.42	7463.88	6517.08	8856.66	8420.75	9415.58 Up
Camkk2	1658.67	0.47504	0.12426	3.82295	0.00013	0.00097	1447.38	1349.23	1367.39	1731.32	2179.64	1877.08 Up
Anapc5	14150	-0.2362	0.08522	-2.7713	0.00558	0.02377	14540.6	15126.1	16250.2	13028.4	13284.8	12670.1 Down
Rnf34	1811.91	0.20914	0.09465	2.20954	0.02714	0.08497	1642.82	1699.95	1699.69	2051.66	1846.99	1930.37 Up
Kdm2b	706.611	0.49978	0.132	3.78639	0.00015	0.0011	595.013	612.327	549.058	750.582	825.812	906.872 Up
Orai1	1959.09	0.36537	0.14564	2.50881	0.01211	0.04471	1827.61	1510.22	1799	2338.83	2409.85	1869.04 Up

Morn3	307.794	-0.7475	0.20864	-3.5827	0.00034	0.00221	357.008	379.47	421.103	235.334	176.356	277.491	Down
Rhof	802.954	1.28841	0.16381	7.86545	3.68E-15	1.90E-13	523.418	385.22	490.81	1023.24	1261.95	1133.09	Up
AI480526	271.446	-0.3883	0.17048	-2.2773	0.02277	0.0739	280.575	299.935	342.803	222.894	228.101	254.366	Down
Psmd9	1828.6	-0.2297	0.09395	-2.4448	0.01449	0.05163	1972.74	2044.92	1904.04	1738.57	1605.16	1706.17	Down
Wdr66	2434.9	-0.6488	0.21036	-3.0841	0.00204	0.01013	2616.12	3260.95	3043.21	2029.89	1311.58	2347.61	Down
Bcl7a	1796.03	-0.6313	0.09091	-6.9448	3.79E-12	1.24E-10	2213.64	2163.75	2171.4	1412.01	1347.49	1467.89	Down
Lrrc43	710.601	-0.6894	0.21512	-3.2046	0.00135	0.00719	870.751	902.68	858.44	610.626	369.609	651.5	Down
Gm15751	111.155	-1.1857	0.30381	-3.9028	9.51E-05	0.00073	125.775	153.321	184.292	72.5701	47.5211	83.4483	Down
Vps33a	2197.56	0.20446	0.0962	2.12542	0.03355	0.09988	2175.91	1910.77	2039.63	2381.34	2361.27	2316.44	Up
Kntc1	105.954	1.39901	0.27406	5.1048	3.31E-07	4.53E-06	60.9526	66.1198	47.7441	179.352	126.723	154.832	Up
Hcar2	710.094	0.73089	0.26142	2.79582	0.00518	0.02233	591.143	680.364	330.389	806.565	1051.8	800.299	Up
Ccdc62	637.604	-0.6083	0.19262	-3.1578	0.00159	0.00822	700.471	803.021	806.876	572.267	375.945	567.046	Down
Hip1r	4732.6	0.22821	0.10252	2.22598	0.02602	0.08219	4071.25	4195.26	4810.7	5003.19	5092.15	5223.06	Up
Cdk2ap1	2707.44	-0.2882	0.11393	-2.5294	0.01142	0.04267	2709.97	2891.07	3329.68	2421.77	2514.4	2377.77	Down
Kmt5a	5080.66	0.41633	0.09864	4.22081	2.43E-05	0.00022	4331.5	4448.24	4277.88	5977.7	6169.3	5279.36	Up
Rilpl2	1359.51	0.52078	0.12105	4.30215	1.69E-05	0.00016	1209.38	1071.33	1069.47	1595.51	1743.5	1467.89	Up
Rilpl1	2294.85	-0.5716	0.12654	-4.5175	6.26E-06	6.46E-05	2427.46	3133.51	2669.85	1898.23	1856.49	1783.58	Down
Tmed2	9479.34	0.24396	0.07435	3.28098	0.00103	0.00573	8547.88	8871.56	8619.73	10392	10465.2	9979.61	Up
Eif2b1	1893.24	0.21866	0.10034	2.17928	0.02931	0.09031	1739.57	1735.41	1775.13	2205.09	1934.64	1969.58	Up
Atp6v0a2	1740.86	0.61701	0.09949	6.20148	5.59E-10	1.28E-08	1398.04	1438.35	1286.23	2055.81	2071.92	2194.79	Up
Dnah10	3681.53	-0.617	0.18826	-3.2776	0.00105	0.00579	4017.07	4678.22	4676.06	3147.47	2106.77	3463.61	Down
Rflna	162.737	-0.6028	0.28773	-2.0952	0.03616	0.10596	141.255	177.278	270.232	119.222	125.667	142.767	Down
Scarb1	4640.75	0.46235	0.14248	3.24495	0.00117	0.00638	4491.14	3564.72	3654.34	4865.31	6112.27	5156.7	Up
Bri3bp	795.743	0.29511	0.13594	2.17096	0.02993	0.09169	756.586	764.69	622.584	895.722	913.462	821.413	Up
Aacs	2571.35	0.67554	0.12488	5.40974	6.31E-08	9.75E-07	1865.34	2272.99	1801.86	3221.08	3253.61	3013.19	Up
Gm42953	4.31168	2.99059	1.45235	2.05913	0.03948	0.11337	1.935	0.95826	0	8.29373	12.6723	2.0108	Up
Tmem132	461.605	-0.981	0.207	-4.7394	2.14E-06	2.46E-05	703.374	468.588	666.508	315.162	266.118	349.88	Down
Adgrd1	569.888	-0.7126	0.22958	-3.1039	0.00191	0.00959	608.558	928.553	586.298	507.991	434.026	353.901	Down
Mmp17	114.112	-0.6397	0.2551	-2.5077	0.01215	0.04483	166.41	113.075	137.503	87.0841	97.1543	83.4483	Down
Zfp11	429.422	-0.306	0.15448	-1.9808	0.04761	0.1314	446.018	474.338	504.178	396.025	333.704	422.268	Down
Mrps17	3930.51	-0.3185	0.11158	-2.8549	0.00431	0.01912	4055.77	4399.37	4633.09	3696.93	3154.35	3643.57	Down
Nipsnap2	6086.73	-0.5422	0.17851	-3.0374	0.00239	0.01157	6593.52	9168.62	5889.72	5171.14	4409.96	5287.4	Down
Phkg1	268.523	-2.3462	0.40363	-5.8128	6.14E-09	1.16E-07	302.828	780.981	262.593	99.5247	69.6977	95.5131	Down
Zbed5	298.389	-0.588	0.16679	-3.5255	0.00042	0.00268	339.593	345.931	389.592	266.436	217.541	231.242	Down
Nupr1l	115.155	-0.7825	0.24131	-3.2426	0.00118	0.00642	148.995	139.906	148.007	102.635	74.9778	76.4105	Down
Vkorc1l1	3299.91	0.31313	0.10333	3.03024	0.00244	0.01181	3110.52	2749.24	2969.69	3546.6	3932.64	3490.75	Up
Gusb	4919.18	1.19895	0.11116	10.7861	4.00E-27	6.70E-25	3271.12	2986.89	2697.54	6638.09	7290.8	6630.62	Up
Asl	1384.74	0.36729	0.10122	3.62852	0.00029	0.0019	1245.17	1241.9	1141.09	1541.6	1631.56	1507.1	Up
Tpst1	2319.92	-0.505	0.09557	-5.2841	1.26E-07	1.84E-06	2841.55	2552.8	2771.07	1927.25	1977.94	1848.93	Down
Tmem248	5795.26	0.24894	0.08299	2.99974	0.0027	0.01285	5290.3	5148.72	5450.47	6594.55	6113.33	6174.17	Up
Sbds	7223.43	-0.2047	0.09156	-2.2356	0.02538	0.08062	8067.03	7124.65	8013.38	6618.39	6848.32	6668.82	Down
Tyw1	978.545	0.32988	0.16333	2.01973	0.04341	0.12222	1050.71	832.727	718.072	1114.47	1105.66	1049.64	Up
Castor2	1613.91	-0.4238	0.12238	-3.4631	0.00053	0.00328	1632.17	2041.09	1874.44	1398.53	1416.13	1321.1	Down
Ncf1	324.633	2.2379	0.37236	6.01001	1.86E-09	3.86E-08	185.76	77.619	77.3455	484.146	731.826	391.101	Up
Syna	42.5405	-1.0681	0.37008	-2.8862	0.0039	0.01759	62.8876	48.8712	61.1125	29.028	22.1765	31.1674	Down
Rfc2	3244.11	-0.2582	0.10488	-2.4623	0.01381	0.04975	3370.78	3518.73	3711.63	3181.68	2711.87	2969.95	Down
Lat2	285.539	0.36227	0.1561	2.32075	0.0203	0.0675	230.265	249.147	270.232	318.272	311.527	333.793	Up
Eln	5777.6	-1.3527	0.44147	-3.0641	0.00218	0.01072	4101.24	11478	9332.07	3415.98	4031.9	2306.39	Down
Cldn4	1152.28	2.36543	0.184	12.8555	8.01E-38	3.32E-35	385.066	405.343	333.254	2353.34	1535.46	1901.21	Up
Cldn3	10103.6	0.407	0.12124	3.35714	0.00079	0.00456	8443.39	9045.96	8573.89	12867.7	11749.3	9941.4	Up
Abhd11	1807.1	0.28316	0.09737	2.90811	0.00364	0.01658	1584.77	1689.41	1616.62	2054.77	2036.02	1861	Up
Mlxipl	257.267	-2.4725	0.34294	-7.2097	5.61E-13	2.12E-11	318.308	674.614	315.111	72.5701	65.4736	97.5239	Down
Nsun5	708.519	-0.308	0.11582	-2.6598	0.00782	0.03119	814.636	738.817	798.282	644.837	604.047	650.494	Down
Ccl24	34.1809	-2.5396	0.62716	-4.0494	5.14E-05	0.00042	65.7901	81.452	27.6916	14.514	11.6163	4.0216	Down
Gm43091	117.999	-1.1411	0.32956	-3.4624	0.00054	0.00328	119.97	171.528	195.751	66.3498	53.8573	100.54	Down
Por	23339.6	-0.4074	0.17476	-2.3309	0.01976	0.06616	27197.4	29039.1	23602.8	21672.5	15163.5	23362.5	Down
Styxl1	300.303	-0.6342	0.25339	-2.5029	0.01232	0.04535	387.001	404.385	304.608	240.518	163.684	301.62	Down
Mdh2	9633.01	-0.3964	0.18146	-2.1845	0.02893	0.08943	9474.74	14280	9089.53	8722.93	8446.09	7784.82	Down
Srrm3	7.70874	-3.7831	1.39478	-2.7123	0.00668	0.02743	3.87001	18.2069	21.0074	0	3.16808	0	Down
Hspb1	6506.16	-0.8242	0.17659	-4.6671	3.05E-06	3.39E-05	7182.73	7661.28	10102.7	5121.38	5153.4	3815.5	Down
Ywhag	8296.4	0.20747	0.09341	2.2211	0.02634	0.08304	7860.95	7770.52	7471	8366.3	9610.89	8698.73	Up
Rasa4	1100.38	-0.6061	0.15172	-3.9952	6.46E-05	0.00052	1151.33	1337.73	1495.35	970.366	873.333	774.159	Down
Lrwd1	1688.88	-0.3122	0.11852	-2.634	0.00844	0.03321	1828.58	1887.77	1896.4	1686.74	1353.82	1479.95	Down
Orai2	1536.94	0.83208	0.11131	7.4751	7.71E-14	3.36E-12	1081.67	1016.71	1218.43	1998.79	1930.41	1975.61	Up
Sh2b2	330.826	0.99856	0.2235	4.46779	7.90E-06	7.96E-05	255.42	220.399	186.202	429.2	543.853	349.88	Up
Cux1	4648.19	0.22675	0.0968	2.34257	0.01915	0.06455	4465.99	4338.04	4046.79	5175.29	5201.98	4661.04	Up
Col26a1	84.7577	-0.8064	0.31295	-2.5768	0.00997	0.03813	115.133	109.241	99.3078	60.1295	43.297	81.4375	Down
4933404C	211.849	-0.5213	0.18395	-2.8337	0.0046	0.02022	277.673	222.316	249.224	172.095	165.796	183.988	Down
Plod3	5591.32	0.60943	0.12101	5.03621	4.75E-07	6.28E-06	4660.45	4225.92	4396.28	6628.76	7639.29	5997.22	Up
Ap1s1	3902.21	0.51173	0.11227	4.5581	5.16E-06	5.44E-05	3374.65	3165.13	3111.96	4717.06	4968.6	4075.9	Up
Serpine1	1990.22	0.61772	0.2027	3.0475	0.00231	0.01123	1634.11	1833.15	1244.21	2328.46	2979.05	1922.33	Up
CAAA010	175.14	4.9646	1.5647	3.17288	0.00151	0.00788	2.9025	29.706	0	337.969	180.58	499.684	Up
Slc12a9	1013.97	0.27069	0.12486	2.168	0.03016	0.09217	972.339	874.89	910.003	1062.63	1233.44	1030.54	Up
Ephb4	7070.03	-0.3976	0.14446	-2.7523	0.00592	0.02491	8019.62	7056.62	9037.97	6302.2	6780.74	5223.06	Down
Zan	72.2878	-0.6822	0.30443	-2.2408	0.02504	0.0798	107.393	84.3268	75.4357	60.1295	60.1934	46.2484	Down
Tfr2	47.4809	1.38911	0.33808	4.10883	3.98E-05	0.00033	26.1225	25.873	26.7367	67.3865	63.3615	75.4051	Up
Pcolce	7301.65	-0.2032	0.09123	-2.2271	0.02594	0.08204	7485.56	7891.26	8067.81	7194.81	6789.19	6381.28	Down
Irs3	175.591	-0.5326	0.19866	-2.6809	0.00734	0.02967	178.988	229.982	213.894	142.03	148.9	139.751	Down
Agfg2	1193.03	0.31799	0.12824	2.47972	0.01315	0.04778	1054.58	1126.91	1004.54	1319.74	1471.04	1181.35	Up
Mepce	3720.54	-0.2092	0.09535	-2.194	0.02824	0.0878	4046.09	3698.88	4224.4	3569.41	3397.23	3387.2	Down
Zcwpw1	119.852	-0.5174	0.23667	-2.1861	0.02881	0.08918	141.255	137.031	145.142	81.9006	97.1543	116.627	Down
Pilra	674.191	1.6192	0.25875	6.25787	3.90E-10	9.26E-09	448.921	341.14	203.39	954.815	1103.55	993.336	Up
Cyp3a13	731.059	0.38375	0.19126	2.00643	0.04481	0.12525	817.539	559.623	526.14	822.116	797.299	863.639	Up

Azgp1	480.04	-0.4498	0.19379	-2.321	0.02029	0.06747	530.191	622.868	509.907	460.302	312.584	444.387	Down
Ap4m1	650.674	0.29908	0.12398	2.41242	0.01585	0.05548	576.631	561.54	612.08	732.958	759.282	661.554	Up
Gm454	3.52256	-5.2131	1.73106	-3.0115	0.0026	0.01246	7.74001	7.66607	5.7293	0	0	0	Down
Nxpe5	70.0654	1.0283	0.51682	1.98969	0.04663	0.12919	49.3426	62.2868	26.7367	66.3498	52.8013	162.875	Up
Pvrig	8.16181	2.42293	0.91792	2.63959	0.0083	0.03279	4.83751	1.91652	0.95488	14.514	12.6723	14.0756	Up
Fam20c	916.947	0.45787	0.19642	2.33109	0.01975	0.06615	913.321	789.605	614.945	1153.86	1175.36	854.591	Up
6330403L(	561.253	-0.4259	0.13859	-3.0728	0.00212	0.01046	592.111	665.99	672.238	522.505	477.323	437.349	Down
Sun1	5558.65	-0.2896	0.07938	-3.6487	0.00026	0.00177	5886.28	6099.32	6358.57	5056.06	4953.81	4997.85	Down
Adap1	466.789	0.72494	0.23778	3.0488	0.0023	0.01119	362.813	434.091	258.773	612.699	676.912	455.447	Up
Zfand2a	2356.13	0.69054	0.0984	7.01786	2.25E-12	7.64E-11	1846.96	1767.03	1794.22	2790.84	3142.73	2795.01	Up
Micall2	691.643	0.82282	0.12371	6.65144	2.90E-11	8.47E-10	476.978	539.5	482.216	933.044	878.613	839.51	Up
Tmem184	1852.68	0.35895	0.10238	3.50597	0.00045	0.00286	1622.5	1628.08	1619.48	2264.19	2022.29	1959.53	Up
Elfn1	947.194	0.58784	0.12654	4.64541	3.39E-06	3.74E-05	827.214	690.904	752.448	1226.43	1099.32	1086.84	Up
Snx8	1572.23	0.4879	0.13633	3.57895	0.00034	0.00224	1523.81	1187.28	1215.57	1747.9	1942.03	1816.76	Up
Eif3b	9903.32	0.22095	0.10642	2.07629	0.03787	0.10983	9673.08	8533.29	9232.76	10726.9	11508.6	9745.35	Up
Amz1	1007.48	1.60002	0.19533	8.19151	2.58E-16	1.55E-14	625.006	476.255	398.186	1444.15	1754.06	1347.24	Up
Card11	1055.88	1.28412	0.2826	4.54394	5.52E-06	5.78E-05	921.061	468.588	454.524	1462.81	1825.87	1202.46	Up
Ap5z1	1381.68	0.32554	0.11626	2.80021	0.00511	0.0221	1187.12	1286.94	1205.06	1546.78	1671.69	1392.48	Up
Radil	1206.51	0.34473	0.15218	2.2653	0.02349	0.07583	1113.59	927.594	1147.77	1313.52	1549.19	1187.38	Up
Tnrc18	2943.95	0.45512	0.0983	4.62985	3.66E-06	3.99E-05	2612.25	2296.95	2540.94	3391.1	3540.85	3281.63	Up
Fscn1	645.212	0.79197	0.12405	6.38403	1.72E-10	4.43E-09	484.718	447.507	485.081	881.208	782.515	790.245	Up
Rbakdn	10.2051	-2.2981	1.02793	-2.2357	0.02537	0.08062	18.3825	7.66607	24.827	8.29373	1.05603	1.0054	Down
Zfp12	1234.27	-0.3349	0.13219	-2.5335	0.01129	0.04225	1343.86	1264.9	1522.08	1030.5	1031.74	1212.51	Down
Kdelr2	5321.39	0.22008	0.09675	2.27489	0.02291	0.07432	4747.53	4796.08	5205.07	6077.23	5742.67	5359.79	Up
Daglb	834.91	0.73878	0.17888	4.12998	3.63E-05	0.00031	778.839	580.705	517.547	1084.4	1089.82	958.147	Up
Cyth3	10899.8	-0.3642	0.1281	-2.8434	0.00446	0.0197	11070.2	11502.9	14232.5	8886.73	9791.47	9915.26	Down
D130017N	161.584	-0.5615	0.23235	-2.4167	0.01566	0.05494	169.313	229.982	178.563	113.002	149.956	128.691	Down
Eif2ak1	2826.33	0.21591	0.08813	2.45006	0.01428	0.05108	2716.74	2567.17	2561.95	3088.38	2901.96	3121.77	Up
Pms2	73.7404	0.66928	0.2999	2.23169	0.02564	0.0812	53.2126	58.4538	59.2027	91.231	70.7537	109.589	Up
Rsph10b	643.508	-0.9101	0.18514	-4.9154	8.86E-07	1.11E-05	728.529	891.181	900.455	499.697	349.544	491.641	Down
Ccz1	4352.08	0.35953	0.09621	3.73684	0.00019	0.00132	3816.79	4030.44	3590.36	4995.93	5074.2	4604.74	Up
Ocm	20.8616	3.05734	0.66824	4.57524	4.76E-06	5.05E-05	2.9025	5.74955	4.77441	31.1015	25.3446	55.297	Up
Lmtk2	4863.54	0.57627	0.11815	4.87758	1.07E-06	1.32E-05	3464.62	3979.65	4270.24	5702.97	6261.17	5502.56	Up
Bhlha15	204.709	1.54458	0.45723	3.37811	0.00073	0.00427	145.125	115.949	52.5186	484.146	151.012	279.501	Up
Gm43267	14.5915	-1.8991	0.66274	-2.8655	0.00416	0.01859	17.415	26.8312	24.827	4.14686	5.28013	9.04861	Down
2900089D	279.458	0.35108	0.1685	2.08351	0.0372	0.10833	279.608	236.69	220.578	315.162	322.088	302.626	Up
Nptx2	37.0851	-2.0664	0.7161	-2.8856	0.00391	0.01762	53.2126	76.6607	49.6539	11.4039	29.5687	2.0108	Down
Smurf1	3463.51	0.2356	0.11046	2.1328	0.03294	0.09843	3217.91	2923.65	3402.25	3701.08	4041.41	3494.77	Up
Arpc1b	12994.4	1.19147	0.11869	10.0384	1.03E-23	1.24E-21	8639.79	7590.37	7512.06	17827.4	20076.1	16320.7	Up
1700001J(	87.6608	-0.7355	0.26308	-2.7959	0.00518	0.02233	126.743	101.575	100.263	63.2397	71.8097	62.3349	Down
Rnf6	3186.56	0.30223	0.10848	2.78616	0.00533	0.02286	3143.41	2657.25	2761.52	3521.72	3348.66	3686.8	Up
Cdk8	4423.57	0.36741	0.07997	4.5942	4.34E-06	4.66E-05	3769.39	3843.58	3977.09	4878.78	4939.03	5133.58	Up
Rpl21	5647.83	-0.2065	0.08366	-2.4685	0.01357	0.04905	6215.23	5697.81	6241.11	5246.82	5215.71	5270.31	Down
Flt3	292.556	0.84246	0.24885	3.38544	0.00071	0.00418	267.998	165.779	194.796	389.805	293.575	443.382	Up
Flt1	8895.81	-0.329	0.12567	-2.6175	0.00886	0.03454	10182	8597.5	10937.2	7642.67	8566.48	7449.02	Down
Gm15407	2.40222	-4.6608	2.01488	-2.3132	0.02071	0.06851	4.83751	7.66607	1.90977	0	0	0	Down
Gm15406	9.12826	-1.4816	0.75018	-1.9751	0.04826	0.13278	14.5125	11.4991	14.3232	5.18358	4.2241	5.027	Down
Gm15411	163.95	-0.751	0.28486	-2.6365	0.00838	0.03301	267.998	180.153	169.014	140.993	90.8182	134.724	Down
Alox5ap	1000.17	1.20618	0.22248	5.42142	5.91E-08	9.21E-07	719.821	638.2	456.434	1338.4	1738.22	1109.96	Up
Medag	473.187	-0.6794	0.20906	-3.2499	0.00115	0.00629	448.921	572.08	726.666	337.969	407.626	345.858	Down
Gm15997	277.122	1.23823	0.40831	3.03253	0.00243	0.01174	111.263	245.314	138.458	453.045	184.804	529.846	Up
Wdr95	1527.55	1.02739	0.29792	3.44859	0.00056	0.00343	714.016	1458.47	844.116	2406.22	1329.54	2412.96	Up
Fry	2881.28	-0.6615	0.11992	-5.516	3.47E-08	5.64E-07	3510.1	3821.54	3259.97	2393.78	1996.94	2305.38	Down
Gm43597	323.198	-0.4651	0.20514	-2.267	0.02339	0.0756	326.048	362.222	436.381	232.224	255.558	326.755	Down
Pds5b	3186.8	-0.2877	0.09415	-3.0553	0.00225	0.01099	3568.15	3382.65	3559.8	2688.2	2877.67	3044.35	Down
Gm36447	20.4709	-0.9845	0.49828	-1.9758	0.04818	0.1326	26.1225	28.7478	26.7367	11.4039	13.7283	16.0864	Down
Stard13	5918.24	-0.3478	0.10417	-3.339	0.00084	0.00481	6643.83	6059.07	7181.67	5059.17	5493.44	5072.25	Down
Vmn2r-ps	5.46025	-3.2805	1.36281	-2.4071	0.01608	0.05613	8.70751	8.62433	12.4135	0	0	3.0162	Down
Gm20559	3073.51	0.5902	0.13071	4.51552	6.32E-06	6.52E-05	2746.74	2065.05	2548.58	3757.06	3718.27	3605.37	Up
Samd9l	5728.77	0.76848	0.10938	7.02595	2.13E-12	7.25E-11	4695.28	3788	4231.09	7225.91	7105.99	7326.36	Up
Hepacam2	427.661	0.40938	0.16113	2.54067	0.01106	0.04155	361.846	332.516	407.735	547.386	473.099	443.382	Up
Vps50	1986.15	0.2835	0.09179	3.08855	0.00201	0.01002	1853.73	1778.53	1742.66	2081.73	2250.39	2209.87	Up
Gng11	2758.1	-0.5227	0.18197	-2.8725	0.00407	0.01826	2700.3	3237.96	3818.58	2418.66	2571.42	1801.68	Down
Peg10	154.81	-0.8607	0.29558	-2.9121	0.00359	0.0164	179.955	182.069	236.811	101.598	151.012	77.4159	Down
Gm44250	9008	0.6347	0.13353	4.75332	2.00E-06	2.31E-05	8278.91	6271.8	6623.07	10511.3	10650	11712.9	Up
Pon3	5173.08	0.83552	0.14719	5.67668	1.37E-08	2.42E-07	4491.14	3348.16	3307.71	6413.12	6374.17	7104.16	Up
Pon2	9376.5	0.62476	0.0939	6.65352	2.86E-11	8.36E-10	7179.83	7465.79	7486.28	11175.8	12311.1	10640.2	Up
Pdk4	1668.58	-2.0175	0.31389	-6.4275	1.30E-10	3.38E-09	1648.62	4164.59	2215.33	850.107	602.99	529.846	Down
Dync1i1	1925.73	-1.0199	0.27455	-3.7149	0.0002	0.00141	2093.67	3126.8	2518.03	1397.49	742.386	1676	Down
Gm20619	16.2854	-1.4823	0.67851	-2.1847	0.02891	0.08942	16.4475	40.2469	15.2781	8.29373	7.39218	10.054	Down
Sem1	2637.27	-0.2871	0.11199	-2.5636	0.01036	0.03935	2696.43	2790.45	3209.36	2430.06	2455.26	2242.04	Down
Gm5873	130.296	2.03032	0.26078	7.7855	6.94E-15	3.44E-13	59.0176	53.6625	41.06	199.049	244.998	183.998	Up
C1galt1	4449.75	0.53184	0.20718	2.56699	0.01026	0.03904	3394	2841.24	4680.84	4615.46	6396.35	4770.63	Up
Col28a1	66.1699	1.1242	0.36227	3.10321	0.00191	0.00961	42.5701	39.2886	42.9697	61.1662	123.555	87.4699	Up
Rpa3	777.446	-0.2864	0.13446	-2.1298	0.03319	0.09905	809.799	794.396	958.702	715.334	733.938	652.505	Down
Ndufa4	5071.2	-0.3747	0.12561	-2.9833	0.00285	0.01347	5090.99	6321.63	5765.58	4817.62	4405.74	4025.63	Down
Thsd7a	273.297	-0.6534	0.32462	-2.0128	0.04413	0.12372	314.438	443.674	244.45	258.142	126.723	252.356	Down
Tmem168	3319.35	0.42197	0.09529	4.42846	9.49E-06	9.35E-05	2877.35	2672.58	2962.05	3616.06	3989.66	3798.4	Up
Gpr85	76.6312	1.43703	0.35754	4.01923	5.84E-05	0.00047	54.1801	30.6643	39.1502	99.5247	146.788	89.4807	Up
2610001J(	2963.05	0.2854	0.10654	2.67869	0.00739	0.02983	2774.79	2507.76	2730.01	3305.05	3480.66	2980.01	Up
1110019D	451.126	0.55354	0.13614	4.06601	4.78E-05	0.00039	390.871	373.721	332.299	528.725	552.301	528.841	Up
Ppp1r3a	749.57	-0.734	0.2607	-2.8154	0.00487	0.02124	925.899	1277.36	605.396	632.397	580.814	475.555	Down
Foxp2	571.32	-0.4888	0.23572	-2.0735	0.03813	0.11038	528.256	827.935	645.501	405.356	406.57	614.3	Down

Mdfic	5602.24	0.33892	0.09876	3.43193	0.0006	0.00361	5182.91	4846.87	4811.65	6113.51	6784.96	5873.55	Up
Tfec	683.78	2.4674	0.28974	8.51582	1.65E-17	1.18E-15	316.373	180.153	131.774	1120.69	1343.26	1010.43	Up
Gm4876	155.763	-0.7875	0.30448	-2.5865	0.0097	0.03725	155.768	150.447	285.51	108.855	120.387	113.61	Down
D830026I1	715.45	-0.6719	0.20245	-3.3187	0.0009	0.00512	826.246	682.28	1128.67	576.414	572.366	506.722	Down
Met	4958.3	1.26762	0.1791	7.07762	1.47E-12	5.15E-11	2948.94	2295.03	3486.28	6110.4	8117.67	6791.48	Up
Capza2	12165.3	0.40755	0.10914	3.73409	0.00019	0.00133	10371.6	10184.4	10818.8	13223.3	15586.9	12806.8	Up
St7	1218.52	0.3501	0.1252	2.79637	0.00517	0.02231	1027.49	1022.46	1164	1377.8	1483.72	1235.64	Up
Wnt2	751.698	-0.6878	0.22351	-3.0773	0.00209	0.01033	890.101	761.816	1130.58	494.513	745.554	487.619	Down
Cftr	678.294	-0.726	0.2293	-3.1661	0.00154	0.00803	927.834	758.941	849.846	452.008	384.393	696.743	Down
Cttnbp2	320.374	-0.4392	0.18337	-2.3954	0.0166	0.05758	378.293	403.427	324.66	229.114	285.127	301.62	Down
Lsm8	1172.69	-0.3164	0.13132	-2.4092	0.01599	0.05587	1243.24	1214.11	1444.74	1100.99	1094.04	939.044	Down
Kcnd2	94.3972	-2.6715	0.6002	-4.451	8.55E-06	8.56E-05	108.36	304.726	76.3906	39.3952	27.4567	10.054	Down
Tspan12	5911.64	0.28768	0.102	2.82032	0.0048	0.02094	5413.17	5160.22	5398.91	6318.78	7150.35	6028.38	Up
Cped1	6590.86	-0.4323	0.07857	-5.5018	3.76E-08	6.08E-07	7672.29	7579.83	7460.5	5458.31	5854.6	5519.65	Down
Ptprz1	891.135	-1.3146	0.26563	-4.9492	7.45E-07	9.51E-06	867.849	1653.95	1291.96	580.561	364.329	588.16	Down
Aass	793.028	0.95528	0.14079	6.78523	1.16E-11	3.60E-10	481.816	522.251	614.945	1043.97	984.216	1110.97	Up
Cadps2	2527.76	-0.4075	0.11558	-3.5255	0.00042	0.00268	2833.81	2802.91	3010.75	2054.77	2020.18	2444.13	Down
Ndufa5	1095.74	-0.6779	0.13621	-4.977	6.46E-07	8.35E-06	1179.38	1512.13	1354.02	885.355	854.325	789.24	Down
Asb15	49.625	-2.4037	0.63901	-3.7616	0.00017	0.00121	49.3426	166.737	34.3758	14.514	12.6723	20.108	Down
Lmod2	568.809	-4.5222	0.38976	-11.603	4.00E-31	8.71E-29	816.571	1877.23	576.749	42.5053	47.5211	52.2808	Down
Gpr37	21.5408	-3.3739	1.20152	-2.808	0.00498	0.02166	2.9025	108.283	6.68418	2.07343	5.28013	4.0216	Down
Grm8	59.1468	-0.905	0.38807	-2.332	0.0197	0.06604	67.7251	108.283	55.3832	40.4319	33.7928	49.2646	Down
Snd1	4582.59	0.45374	0.13113	3.46014	0.00054	0.00331	4234.75	3723.79	3644.79	5302.8	5966.54	4622.83	Up
Lep	171.518	-7.9472	1.12131	-7.0874	1.37E-12	4.82E-11	212.85	662.157	149.917	2.07343	2.11205	0	Down
Prrt4	357.297	-1.1965	0.23465	-5.0992	3.41E-07	4.65E-06	384.098	463.797	644.546	194.903	251.334	205.102	Down
Impdh1	7287.68	-0.2412	0.12201	-1.9772	0.04802	0.13224	8210.22	7182.15	8294.11	6972.95	7203.15	5863.5	Down
Hilpda	4749.32	-0.5342	0.13789	-3.8746	0.00011	0.0008	5292.23	5737.09	5826.7	4345.91	4085.76	3208.23	Down
Fam71f2	315.527	-0.9521	0.20585	-4.6252	3.74E-06	4.08E-05	315.405	460.922	471.712	220.82	210.149	214.15	Down
Ccdc136	617.688	1.75151	0.14428	12.14	6.48E-34	1.84E-31	256.388	320.058	272.142	893.649	1013.78	950.104	Up
Flnc	668.187	-2.1034	0.36246	-5.8031	6.51E-09	1.23E-07	591.143	1853.27	807.831	230.151	291.463	235.264	Down
Atp6v1f	4107.48	0.3548	0.10984	3.23006	0.00124	0.00668	3669.73	3634.68	3510.15	4621.68	5079.48	4129.18	Up
Kcp	385.548	-0.5177	0.26027	-1.9889	0.04671	0.12935	300.893	569.206	491.765	368.034	319.976	263.415	Down
Irf5	1217.89	0.51295	0.1388	3.69556	0.00022	0.0015	1137.78	963.05	910.003	1420.3	1568.2	1308.03	Up
Tnpo3	2277.19	0.34182	0.11303	3.02399	0.00249	0.01202	2136.24	1841.77	2048.22	2485.01	2395.07	2756.81	Up
Tspan33	321.347	1.16606	0.21105	5.52519	3.29E-08	5.40E-07	188.663	241.481	164.24	511.101	381.225	441.371	Up
Ahcyl2	7003.1	1.19496	0.17201	6.94699	3.73E-12	1.22E-10	3475.27	4589.1	4709.48	8947.89	11740.9	8555.96	Up
Strip2	259.104	-1.0165	0.32547	-3.1231	0.00179	0.00908	254.453	545.249	240.63	172.095	175.3	166.897	Down
Rncr4	10.2303	1.96597	0.83544	2.35321	0.01861	0.06315	5.80501	4.79129	1.90977	13.4773	25.3446	10.054	Up
Cpa2	16.9232	1.9308	0.68672	2.81162	0.00493	0.02147	4.83751	5.74955	10.5037	13.4773	33.7928	33.1782	Up
Cpa5	7.72326	6.4509	1.45669	4.42847	9.49E-06	9.35E-05	0	0	0	14.514	13.7283	18.0972	Up
Cpa1	15.312	-1.4389	0.62413	-2.3055	0.02114	0.06962	15.48	21.0817	30.5563	7.25701	8.4482	9.04861	Down
Cep41	651.665	-0.306	0.12587	-2.4309	0.01506	0.05323	660.804	735.943	764.861	582.634	555.469	610.278	Down
Plxna4	2432.4	0.92932	0.19373	4.79711	1.61E-06	1.90E-05	1836.32	1672.16	1516.35	3013.73	4102.66	2453.18	Up
Chchd3	3489.71	-0.3593	0.13342	-2.693	0.00708	0.02882	3508.16	4591.98	3665.8	3138.14	3141.68	2892.54	Down
Exoc4	3379.43	0.31334	0.08724	3.59162	0.00033	0.00215	3121.16	3042.47	2878.02	3605.7	3842.88	3786.34	Up
Lrguk	1451.88	-0.5982	0.2712	-2.2057	0.02741	0.08563	1572.19	2037.26	1636.67	1149.72	691.697	1623.72	Down
Gm14546	2.72606	3.90057	1.81871	2.14469	0.03198	0.09625	0	0	0.95488	7.25701	2.11205	6.03241	Up
Akr1b8	4126.28	1.17413	0.21819	5.38128	7.40E-08	1.13E-06	3421.09	2413.85	1767.49	5620.04	6192.53	5342.7	Up
Akr1b10	1551.11	0.32422	0.14515	2.23365	0.02551	0.08092	1487.05	1501.59	1143.95	1672.22	1867.05	1634.78	Up
Agbl3	972.537	-0.3727	0.13815	-2.6977	0.00698	0.02846	1014.91	1167.16	1110.53	802.418	773.011	967.196	Down
Gm43748	69.5521	-1.3103	0.46251	-2.833	0.00461	0.02026	75.4651	141.822	80.2102	40.4319	20.0645	59.3187	Down
Tmem140	608.21	-0.3126	0.14714	-2.1246	0.03362	0.10002	639.518	607.536	774.41	524.578	572.366	530.852	Down
Wdr91	1999.95	0.55433	0.13545	4.09264	4.26E-05	0.00036	1637.01	1439.3	1784.68	2310.84	2662.24	2165.63	Up
Slc13a4	323.992	-0.6984	0.27805	-2.5119	0.01201	0.04437	551.476	321.017	330.389	278.877	182.692	279.501	Down
Mtpn	13802.8	0.43077	0.08037	5.35968	8.34E-08	1.25E-06	11868.3	11822	11581.8	14972.3	16226.9	16345.8	Up
9330158H	87.9307	-3.5189	0.52421	-6.7129	1.91E-11	5.73E-10	81.2701	288.436	115.541	13.4773	14.7844	14.0756	Down
Chrm2	1156.72	-1.6862	0.50219	-3.3577	0.00079	0.00455	1516.07	2843.15	935.785	566.047	408.682	670.602	Down
Ptn	62.57	-1.8	0.59803	-3.0099	0.00261	0.01251	61.9201	157.154	72.5711	53.9092	14.7844	15.081	Down
D630045J	571.807	-0.5558	0.13402	-4.1472	3.37E-05	0.00029	663.706	652.574	725.711	432.311	448.811	507.727	Down
Zc3hav1l	378.282	-0.5264	0.17348	-3.0343	0.00241	0.01167	472.141	427.383	440.201	255.032	321.032	353.901	Down
Zc3hav1	5297.03	0.40143	0.09529	4.21277	2.52E-05	0.00022	4689.48	4194.3	4810.7	5859.52	6029.9	6198.3	Up
Fmc1	283.552	-0.8375	0.18016	-4.6485	3.34E-06	3.68E-05	356.041	397.677	337.074	175.205	230.214	205.102	Down
Tbxas1	934.268	1.71151	0.22607	7.57071	3.71E-14	1.69E-12	565.988	397.677	347.577	1485.61	1691.75	1117	Up
Parp12	4665.39	0.42844	0.10151	4.22075	2.43E-05	0.00022	4379.88	3819.62	3733.59	5394.03	5451.2	5214.01	Up
Slc37a3	2803.48	0.36748	0.09653	3.80706	0.00014	0.00102	2579.36	2376.48	2389.12	3329.93	3165.96	2980.01	Up
Rab19	687.116	1.04313	0.16368	6.3729	1.85E-10	4.72E-09	512.776	420.676	413.464	939.265	1041.24	795.272	Up
Dennd2a	1372.11	-0.4764	0.16131	-2.9534	0.00314	0.01466	1486.08	1558.13	1745.53	959.999	1376	1106.95	Down
Braf	4009.41	0.28395	0.10224	2.77736	0.00548	0.0234	3779.06	3381.69	3687.76	4118.87	4367.72	4721.36	Up
Mrps33	848.783	-0.3022	0.14143	-2.1366	0.03263	0.09765	874.621	1000.42	936.74	763.023	854.325	663.565	Down
Tmem178	79.8049	-0.8433	0.29631	-2.8458	0.00443	0.01956	115.133	114.991	77.3455	53.9092	59.1374	58.3133	Down
Ssbp1	1614.63	-0.3367	0.12366	-2.7229	0.00647	0.02674	1716.35	1719.12	1970.88	1516.72	1485.83	1278.87	Down
Clec5a	531.696	2.85096	0.25368	11.2384	2.64E-29	4.99E-27	168.345	116.908	103.127	756.803	1216.54	828.45	Up
Mgam	86.3316	2.26249	0.4353	5.19757	2.02E-07	2.86E-06	28.0575	48.8712	12.4135	151.361	142.563	134.724	Up
Prss2	4.09705	3.56254	1.57737	2.25854	0.02391	0.0769	0	1.91652	0	11.4039	4.2241	7.03781	Up
Trbc1	403.431	-0.4463	0.14974	-2.9804	0.00288	0.01357	465.368	416.843	513.727	336.933	356.937	330.777	Down
Trbc2	376.546	-0.6024	0.16434	-3.6658	0.00025	0.00166	517.613	390.011	454.524	296.501	302.023	298.604	Down
Gstk1	3351.02	-0.7252	0.1042	-6.96	3.40E-12	1.12E-10	4371.17	3933.65	4223.45	2481.9	2361.27	2734.69	Down
Tmem139	460.71	0.45406	0.13219	3.43491	0.00059	0.00358	364.748	387.136	414.419	528.725	555.469	513.76	Up
Clcn1	43.5118	-1.6799	0.52872	-3.1773	0.00149	0.00778	72.5626	94.8676	31.5111	14.514	28.5127	19.1026	Down
Zyx	9545.33	-0.4044	0.14652	-2.7602	0.00578	0.02445	10881.5	10581.1	11160.7	8107.12	9789.36	6752.27	Down
Epha1	1228.08	-0.3213	0.12412	-2.5888	0.00963	0.03703	1524.78	1216.03	1352.11	1083.37	1078.2	1113.98	Down
Olfr457	17.7704	-1.5688	0.66132	-2.3722	0.01768	0.06056	30.96	20.1234	28.6465	3.11015	13.7283	10.054	Down
Olfr455	37.9512	-1.4939	0.43457	-3.4375	0.00059	0.00355	65.7901	43.1216	59.2027	15.5507	16.8964	27.1458	Down

Tcaf3	7.8745	-4.4699	1.64691	-2.7141	0.00665	0.0273	27.09	0.95826	17.1879	0	0	2.0108	Down
Arhgef5	3971.02	0.22915	0.09288	2.46718	0.01362	0.04919	3853.56	3397.03	3718.31	4319.99	4369.83	4167.39	Up
Tpk1	778.122	0.48099	0.11234	4.28175	1.85E-05	0.00017	614.363	663.115	671.283	947.558	878.613	893.801	Up
Cntnap2	77.7308	0.73845	0.27579	2.67761	0.00741	0.02989	55.1476	65.1616	54.4283	104.708	103.49	83.4483	Up
Pdia4	7166.53	0.50315	0.08655	5.81358	6.12E-09	1.16E-07	5741.15	5721.76	6325.14	8453.38	8214.82	8542.89	Up
Zfp783	187.78	-0.544	0.20558	-2.6462	0.00814	0.03229	234.135	230.94	203.39	139.957	137.283	180.972	Down
Zfp956	766.187	-0.3023	0.11941	-2.532	0.01134	0.04239	844.629	839.435	854.62	724.664	620.943	712.829	Down
Zfp467	3004.43	0.35121	0.1033	3.39982	0.00067	0.004	2721.58	2696.54	2503.7	3288.46	3159.63	3656.64	Up
Zfp862-ps	689.918	-0.3949	0.13484	-2.9287	0.0034	0.01569	770.131	835.602	745.764	573.304	549.133	665.575	Down
Atp6v0e2	1951.5	-0.4732	0.12945	-3.6558	0.00026	0.00172	2205.9	2392.77	2207.69	1812.18	1402.4	1688.07	Down
Rarres2	3239.87	-0.5184	0.16083	-3.223	0.00127	0.00682	4659.49	3366.36	3421.35	2921.47	2670.69	2399.89	Down
Gm28053	109.438	-0.658	0.29642	-2.2197	0.02644	0.08327	94.8151	166.737	140.368	73.6068	86.5941	94.5077	Down
4833403J1	121.631	-0.6227	0.25954	-2.3992	0.01643	0.05711	124.808	153.321	164.24	77.7537	116.163	93.5023	Down
Gimap3	2114.39	-0.5766	0.10276	-5.6113	2.01E-08	3.41E-07	2674.17	2330.49	2589.64	1629.72	1713.93	1748.39	Down
Tmem176	27372.9	0.45834	0.11619	3.94478	7.99E-05	0.00062	21913.9	24548.7	22720.5	33079.5	27705.9	34269.1	Up
Tmem176	28008.4	0.61006	0.12029	5.07159	3.95E-07	5.31E-06	22089	22834.3	21596.6	35117.7	28965.7	37447.2	Up
Gpnmb	4247.16	2.98722	0.57375	5.20646	1.92E-07	2.73E-06	1622.5	681.322	550.013	7066.26	10093.5	5469.38	Up
Gm19253	8.79588	4.11393	1.18051	3.48489	0.00049	0.00306	0.9675	0	1.90977	20.7343	21.1205	8.04321	Up
Igf2bp3	304.554	-0.9921	0.20825	-4.7639	1.90E-06	2.20E-05	383.131	382.345	450.705	173.132	181.636	256.377	Down
Cycs	3692.73	-0.4929	0.19917	-2.4748	0.01333	0.04833	3377.55	5767.76	3807.12	3088.38	3266.29	2849.31	Down
C530044C	12.3288	-1.7008	0.8281	-2.0538	0.03999	0.11452	13.545	28.7478	14.3232	2.07343	4.2241	11.0594	Down
Nfe2l3	431.037	-0.5519	0.22616	-2.4405	0.01467	0.05209	446.986	576.872	513.727	433.347	251.334	363.955	Down
Snx10	2331.36	1.14722	0.18406	6.23271	4.58E-10	1.07E-08	1677.65	1551.42	1121.99	2962.93	3781.63	2892.54	Up
Skap2	6643.97	0.42983	0.08074	5.32347	1.02E-07	1.51E-06	5757.6	5486.03	5740.76	7462.28	7956.1	7461.08	Up
Hoxa1	79.4462	-0.8065	0.40801	-1.9767	0.04808	0.13238	73.5301	131.281	98.3529	68.4232	73.9218	31.1674	Down
Hotairm1	233.064	-0.4324	0.21032	-2.056	0.03978	0.11401	222.525	306.643	274.051	167.948	208.037	219.177	Down
Gm27477	41.2901	-1.156	0.40564	-2.8498	0.00437	0.01937	56.1151	58.4538	56.3381	26.9546	33.7928	16.0864	Down
Hoxa7	315.776	-0.7338	0.29814	-2.4614	0.01384	0.04984	438.278	275.978	468.847	316.198	235.494	159.859	Down
Tril	712.016	-1.1312	0.23253	-4.8648	1.15E-06	1.39E-05	1041.03	852.85	1038.91	488.293	554.413	296.593	Down
Fkbp14	1720.33	-0.5743	0.20772	-2.7649	0.00569	0.02415	1410.62	2549.93	2214.37	1426.52	1341.15	1379.41	Down
Mturn	2288.44	-0.5853	0.12239	-4.7826	1.73E-06	2.02E-05	3110.52	2443.56	2685.13	1868.16	1841.71	1781.57	Down
Znrf2	4068.39	0.34484	0.11315	3.04769	0.00231	0.01123	3883.55	3641.38	3228.46	4282.67	4864.05	4510.23	Up
Crhr2	65.5083	-4.1737	0.66924	-6.2364	4.48E-10	1.05E-08	81.2701	245.314	45.8344	7.25701	6.33615	7.03781	Down
Inmt	79733.4	-1.2481	0.15448	-8.0795	6.50E-16	3.74E-14	122568	98016.4	116078	48686.2	54365.2	38685.8	Down
Mindy4	639.679	-0.4237	0.13355	-3.1728	0.00151	0.00788	822.376	695.696	680.832	558.79	536.461	543.922	Down
Adcyap1r1	325.962	-0.735	0.25317	-2.9033	0.00369	0.01679	506.003	446.549	269.277	240.518	225.989	267.437	Down
Pde1c	346.718	1.07579	0.33503	3.21101	0.00132	0.00707	130.613	330.599	208.164	354.557	455.147	601.23	Up
Gm10209	14.9751	-1.7487	0.67241	-2.6007	0.0093	0.03601	34.8301	18.2069	16.233	7.25701	5.28013	8.04321	Down
Fkbp9	8749.34	-0.2702	0.10046	-2.6895	0.00716	0.02903	10258.4	9589.29	8850.81	8105.04	8246.5	7446	Down
Herc6	1006.49	0.40216	0.13684	2.93898	0.00329	0.01524	908.484	794.396	898.545	1003.54	1172.19	1261.78	Up
Vopp1	2215.58	0.26296	0.10596	2.48163	0.01308	0.04756	1989.18	1861.9	2191.46	2443.54	2486.94	2320.47	Up
Abcg2	1942.78	-0.313	0.14907	-2.0996	0.03576	0.10503	2563.88	1947.18	1947.01	1648.38	1891.34	1658.91	Down
Fam13a	3702.93	-1.0342	0.19143	-5.4026	6.57E-08	1.01E-06	4837.51	5126.68	4964.44	2584.53	1723.43	2981.01	Down
Tigd2	1367.49	0.59049	0.11602	5.08949	3.59E-07	4.87E-06	1185.19	1046.42	1042.73	1641.12	1757.23	1532.23	Up
Snca	503.328	-0.995	0.31318	-3.1772	0.00149	0.00778	1015.88	560.581	434.472	325.529	416.074	267.437	Down
A730020E	216.395	-0.4194	0.20051	-2.0915	0.03648	0.10671	222.525	282.686	237.766	194.903	158.404	202.086	Down
Hpgds	339.461	1.8819	0.31128	6.04578	1.49E-09	3.19E-08	203.175	146.614	84.9846	560.863	409.738	631.392	Up
Tnip3	287.359	2.28936	0.19258	11.8877	1.37E-32	3.40E-30	86.1076	116.908	89.759	520.431	462.539	448.409	Up
Ndnf	10573.6	1.23343	0.13504	9.13386	6.61E-20	5.66E-18	7056.96	5900	5973.75	13507.4	17072.8	13930.8	Up
Prdm5	489.455	-0.6009	0.18273	-3.2883	0.00101	0.00562	488.588	708.153	572.93	401.209	413.962	351.89	Down
2610300M	18.1324	2.49988	0.76323	3.27539	0.00106	0.00583	8.70751	4.79129	2.86465	12.4406	32.7368	47.2538	Up
Gng12	6596.33	0.41176	0.09152	4.49924	6.82E-06	7.00E-05	6102.03	5501.36	5380.77	7554.55	7350.99	7688.3	Up
Gadd45a	643.625	0.72317	0.23482	3.07964	0.00207	0.01026	405.383	554.832	496.539	1078.18	706.481	620.332	Up
A430010J1	6.65316	-1.9375	0.93878	-2.0638	0.03903	0.11235	7.74001	10.5408	13.3684	4.14686	2.11205	2.0108	Down
Gm42889	46.094	1.47698	0.43165	3.42171	0.00062	0.00373	37.7326	16.2904	19.0977	59.0928	74.9778	69.3727	Up
Igkv1-133	78.413	4.88811	0.83488	5.85485	4.77E-09	9.30E-08	5.80501	6.70781	2.86465	126.479	17.9524	310.669	Up
Igkv14-12	112.47	1.66049	0.60884	2.72731	0.00639	0.0265	48.3751	45.0382	68.7516	355.594	46.4651	110.594	Up
Igkv17-12	65.2941	1.52878	0.55091	2.77502	0.00552	0.02355	30.96	43.1216	26.7367	183.499	60.1934	47.2538	Up
Igkv9-120	88.1714	1.61693	0.56541	2.85978	0.00424	0.01887	80.3026	35.4556	14.3232	116.112	195.365	87.4699	Up
Igkv1-117	427.646	2.95862	0.75462	3.92067	8.83E-05	0.00068	144.158	87.2015	61.1125	337.969	420.298	1515.14	Up
Igkv14-11	70.0729	1.39335	0.47832	2.91302	0.00358	0.01636	21.285	26.8312	67.7967	111.965	73.9218	118.637	Up
Igkv1-110	442.059	3.05081	0.93167	3.27456	0.00106	0.00584	135.45	67.0781	83.0748	317.235	1814.25	235.264	Up
Igkv16-10	51.5354	1.71755	0.68752	2.49818	0.01248	0.04582	29.025	29.706	13.3684	168.985	36.9609	31.1674	Up
Igkv10-96	166.347	2.4212	0.92211	2.62571	0.00865	0.03389	96.7501	34.4973	25.7818	317.235	461.483	62.3349	Up
Igkv10-94	36.3072	1.51144	0.64435	2.34569	0.01899	0.06413	11.61	29.706	15.2781	29.028	31.6808	100.54	Up
Igkv19-93	56.4805	1.0113	0.41724	2.42377	0.01536	0.05408	38.7001	37.3721	36.2855	116.112	59.1374	51.2754	Up
Igkv4-91	110.586	2.25171	0.46344	4.85874	1.18E-06	1.43E-05	38.7001	22.9982	53.4734	128.553	116.163	303.631	Up
Igkv12-89	259.111	3.1878	0.46723	6.82277	8.93E-12	2.82E-10	67.7251	20.1234	65.8869	406.393	738.162	256.377	Up
Igkv1-88	40.4197	3.7025	0.6295	5.88161	4.06E-09	8.05E-08	9.67501	3.83303	3.81953	70.4967	39.0729	115.621	Up
Igkv4-86	74.8569	4.20775	1.8879	2.2288	0.02583	0.08172	7.74001	11.4991	3.81953	313.088	112.995	0	Up
Igkv13-84	16.2986	3.04393	0.74057	4.11027	3.95E-05	0.00033	4.83751	1.91652	3.81953	22.8077	43.297	21.1134	Up
Igkv4-79	125.212	4.4164	1.41756	3.11549	0.00184	0.00927	9.67501	18.2069	5.7293	76.717	10.5603	630.386	Up
Igkv4-70	69.4571	2.40437	0.79372	3.02925	0.00245	0.01184	14.5125	41.2051	10.5037	165.875	13.7283	170.918	Up
Igkv4-69	15.1991	2.6543	1.16396	2.28041	0.02258	0.07336	5.80501	3.83303	2.86465	70.4967	3.16808	5.027	Up
Igkv4-58	20.6692	2.44677	0.90527	2.70282	0.00688	0.02811	7.74001	2.87478	8.59395	15.5507	79.2019	10.054	Up
Igkv4-50	30.025	1.98018	0.90314	2.19254	0.02834	0.08806	1.935	30.6643	3.81953	79.8271	32.7368	31.1674	Up
Igkv5-48	121.813	2.18264	0.60506	3.60734	0.00031	0.00204	81.2701	20.1234	30.5563	373.218	136.227	89.4807	Up
Igkv12-46	251.824	3.19598	1.3978	2.28644	0.02223	0.07248	25.155	90.0763	33.4209	1094.77	253.446	14.0756	Up
Igkv12-44	62.6314	2.27416	0.4236	5.36861	7.93E-08	1.20E-06	25.155	15.3321	23.8721	120.259	128.835	62.3349	Up
Igkv5-43	44.9292	1.37537	0.52426	2.62344	0.0087	0.03407	29.9925	28.7478	16.233	45.6155	108.771	40.216	Up
Igkv8-30	564.05	4.05332	1.30424	3.1078	0.00188	0.00949	71.5951	101.575	19.0977	71.5334	560.749	2559.75	Up
Igkv8-28	65.4602	2.06598	0.51651	3.9999	6.34E-05	0.00051	8.70751	44.0799	22.9172	114.039	82.37	120.648	Up
Igkv8-27	30.5318	2.30521	0.7209	3.19767	0.00139	0.00734	21.285	2.87478	6.68418	30.0648	76.0338	46.2484	Up

Igkv6-25	129.223	3.94989	1.29668	3.04614	0.00232	0.01127	23.22	10.5408	13.3684	25.9179	52.8013	649.489	Up
Igkv8-24	47.7944	1.96455	0.53033	3.70438	0.00021	0.00146	16.4475	12.4574	29.6014	62.203	45.4091	120.648	Up
Igkv8-19	42.3669	2.25371	0.85475	2.6367	0.00837	0.033	9.67501	5.74955	28.6465	19.6976	29.5687	160.864	Up
Igkv6-17	76.1644	1.48513	0.55179	2.69145	0.00711	0.02892	66.7576	29.706	23.8721	57.0194	86.5941	193.037	Up
Igkv6-13	25.6719	2.43661	0.70675	3.44762	0.00057	0.00344	6.77251	12.4574	4.77441	65.3131	49.6332	15.081	Up
Igkv3-10	35.0124	1.71594	0.7381	2.3248	0.02008	0.06702	18.3825	18.2069	12.4135	59.0928	92.9302	9.04861	Up
Igkv3-5	112.125	4.00348	1.16689	3.4309	0.0006	0.00362	25.155	3.83303	10.5037	503.844	57.0254	72.3889	Up
Igkv3-4	68.2151	1.60901	0.74139	2.17025	0.02999	0.09177	53.2126	26.8312	21.0074	230.151	55.9693	22.1188	Up
Igkv3-3	5.1826	3.29109	1.42406	2.31106	0.02083	0.0688	0.9675	0	1.90977	10.3672	15.8404	2.0108	Up
Igkv3-1	24.2253	2.47974	0.78381	3.1637	0.00156	0.00808	5.80501	12.4574	3.81953	39.3952	71.8097	12.0648	Up
Gm30211	595.523	0.52241	0.25753	2.02854	0.04251	0.12018	682.089	435.049	349.487	581.598	812.084	712.829	Up
Eif2ak3	2294.91	0.30813	0.08857	3.47901	0.0005	0.00312	2078.19	2040.13	2033.9	2669.54	2497.5	2450.16	Up
Thnsl2	790.932	-0.5092	0.19914	-2.5569	0.01056	0.03999	894.939	1066.54	825.974	654.168	502.668	801.304	Down
Smyd1	824.763	-1.6816	0.36794	-4.5705	4.87E-06	5.16E-05	935.574	2182.91	654.095	335.896	410.794	429.306	Down
Cd8b1	207.79	-0.6023	0.20625	-2.9202	0.0035	0.01606	250.583	209.859	291.239	151.361	164.74	178.961	Down
Gm44174	11.3496	-1.9019	0.78707	-2.4164	0.01567	0.05497	18.3825	12.4574	22.9172	2.07343	4.2241	8.04321	Down
Cd8a	252.303	-0.7084	0.21361	-3.3165	0.00091	0.00516	308.633	285.561	344.713	198.013	228.101	148.799	Down
Gm44172	2.07186	4.55162	2.08063	2.18761	0.0287	NA	0	0	0	2.07343	6.33615	4.0216	Up
Reep1	946.763	-0.5038	0.15243	-3.3049	0.00095	0.00535	964.599	1242.86	1123.9	752.656	712.817	883.747	Down
Atoh8	1252.13	-0.7632	0.13802	-5.5293	3.22E-08	5.29E-07	1378.69	1566.75	1781.81	998.357	913.462	873.693	Down
Sftpb	173205	0.76828	0.19112	4.01986	5.82E-05	0.00047	166072	94825.4	123543	228154	204422	222216	Up
Tmem150	1921.91	-0.2462	0.11037	-2.2308	0.02569	0.08133	2050.14	2178.12	2028.17	1934.51	1710.76	1629.75	Down
Rnf181	12070	0.52062	0.10629	4.89835	9.66E-07	1.20E-05	10250.7	9179.16	10316.6	15580.8	13698.8	13394	Up
Vamp5	1928.71	-0.4337	0.10976	-3.9507	7.79E-05	0.00061	2235.9	2087.09	2326.09	1762.42	1652.68	1508.1	Down
Vamp8	15450.5	0.26005	0.11222	2.31724	0.02049	0.06803	15015.6	12864.6	14305.1	18370.6	16726.4	15420.8	Up
Ggcx	3554.82	0.61357	0.10474	5.85813	4.68E-09	9.16E-08	3091.17	2572.92	2766.3	4343.84	4210.37	4344.34	Up
Mat2a	24150.5	-0.5203	0.15206	-3.4212	0.00062	0.00374	23664.1	31814.2	29896.4	20084.3	22249.4	17194.4	Down
Capg	3679.26	1.36363	0.21623	6.30625	2.86E-10	7.00E-09	2648.05	1787.15	1742.66	5254.08	6531.52	4112.09	Up
Retsat	2045.58	0.61722	0.14228	4.33814	1.44E-05	0.00014	1767.63	1715.28	1360.71	2492.26	2686.53	2251.09	Up
Tgoln1	16447.6	1.63474	0.13053	12.5235	5.55E-36	1.93E-33	8426.94	7894.13	7717.36	24000	28971	21676.4	Up
Gm20560	26.2932	-1.1731	0.50244	-2.3348	0.01955	0.06564	22.2525	42.1634	44.8795	16.5875	14.7844	17.0918	Down
Dnah6	5753.45	-0.7595	0.221	-3.4365	0.00059	0.00356	6403.89	8026.37	7271.43	4515.93	2840.71	5462.34	Down
Eva1a	546.478	-0.3861	0.16893	-2.2855	0.02228	0.07262	508.906	619.035	729.531	486.22	464.651	470.528	Down
Sema4f	1279.19	0.36347	0.14538	2.50017	0.01241	0.04563	1231.63	929.511	1195.51	1511.53	1465.76	1341.2	Up
Dqx1	705.36	-0.5905	0.17533	-3.3679	0.00076	0.00441	755.619	860.516	927.191	642.764	449.867	596.203	Down
Pcgf1	725.389	-0.326	0.14005	-2.3275	0.01994	0.06667	838.824	730.193	851.756	648.984	706.481	576.095	Down
Mogs	2043.38	0.27599	0.11441	2.41227	0.01585	0.0555	1991.12	1695.16	1859.16	2335.72	2319.03	2060.07	Up
Rtkn	615.153	0.94905	0.18285	5.19031	2.10E-07	2.96E-06	497.296	321.975	440.201	772.353	842.708	816.386	Up
Wdr54	318.235	-0.7786	0.19382	-4.0173	5.89E-05	0.00047	434.408	390.97	380.998	277.84	186.916	238.28	Down
1700003E	848.989	-0.6023	0.15287	-3.9399	8.15E-05	0.00063	1142.62	1017.67	910.958	699.783	582.926	739.975	Down
Mob1a	11812.7	0.22577	0.09316	2.42352	0.01537	0.05411	11329.4	10227.5	11113.9	12215.6	13553	12436.8	Up
Bola3	1112.15	-0.3896	0.14057	-2.7718	0.00558	0.02375	1074.89	1433.55	1275.72	985.917	976.823	925.974	Down
Actg2	1268.38	-1.9559	0.50425	-3.8788	0.0001	0.00079	1115.53	2764.58	2170.45	544.276	712.817	302.626	Down
Cd207	130.092	0.9114	0.40392	2.2564	0.02405	0.07724	115.133	45.9964	109.812	240.518	139.395	129.697	Up
Nagk	2824.41	0.37197	0.10532	3.53193	0.00041	0.00262	2432.3	2564.3	2390.07	3255.29	3410.96	2893.54	Up
Paip2b	3494.98	0.5846	0.10017	5.83618	5.34E-09	1.03E-07	2935.4	2573.88	2879.93	4411.23	4122.72	4046.74	Up
Gm44127	59.9381	-1.0591	0.30288	-3.4968	0.00047	0.00295	87.0751	84.3268	71.6162	37.3218	39.0729	40.216	Down
Cyp26b1	2080.76	-1.6796	0.30859	-5.4428	5.25E-08	8.29E-07	2956.68	2792.37	3765.1	1033.61	1434.08	502.7	Down
Spr-ps1	37.9797	-1.0444	0.44478	-2.3482	0.01887	0.0638	31.9275	68.9946	52.5186	25.9179	26.4006	22.1188	Down
Smyd5	1203.24	0.37686	0.13575	2.77602	0.0055	0.02349	904.614	1044.5	1191.69	1410.97	1348.54	1319.09	Up
Pradc1	856.369	0.25784	0.11235	2.29505	0.02173	0.07119	811.734	739.776	788.733	886.392	929.302	982.277	Up
Nat8f7	103.166	-1.2518	0.31426	-3.9834	6.79E-05	0.00054	165.443	112.116	158.511	50.7991	50.6892	81.4375	Down
Nat8f4	288.399	0.63484	0.2573	2.46727	0.01361	0.04918	313.47	187.819	176.653	374.254	305.191	373.004	Up
Gm10443	106.974	-0.5127	0.24164	-2.1217	0.03386	0.10057	126.743	109.241	141.323	94.3411	89.7622	80.4321	Down
Snrpg	901.244	-0.335	0.12172	-2.7523	0.00592	0.02491	1047.8	905.554	1062.78	813.822	818.42	759.078	Down
Pcyox1	5489.28	0.74493	0.16029	4.64732	3.36E-06	3.70E-05	5073.58	3667.26	3567.44	6794.64	7454.48	6378.26	Up
Gm43936	23.4685	-1.4254	0.51929	-2.7449	0.00605	0.02534	23.22	34.4973	44.8795	13.4773	12.6723	12.0648	Down
Gm28719	71.1589	-1.1947	0.39983	-2.988	0.00281	0.0133	55.1476	121.699	120.315	47.6889	34.8488	47.2538	Down
Gm44386	28.2994	-3.7982	0.72934	-5.2077	1.91E-07	2.72E-06	38.7001	90.0763	29.6014	5.18358	4.2241	2.0108	Down
1600020E	1089.38	-0.6451	0.12654	-5.0979	3.43E-07	4.68E-06	1209.38	1315.69	1461.93	868.768	786.739	893.801	Down
Snrnp27	1756.35	-0.2391	0.11891	-2.0111	0.04431	0.12411	1737.63	1974.97	1991.89	1657.71	1716.04	1459.84	Down
Anxa4	15032	0.79054	0.11164	7.08088	1.43E-12	5.05E-11	12290.2	9975.47	10774.9	19275.7	19841.7	18033.9	Up
Gfpt1	7442.33	1.00849	0.08322	12.1185	8.43E-34	2.36E-31	4835.57	5103.69	4887.09	9446.55	10120.9	10260.1	Up
D6Ertd527	163.01	2.86249	0.29358	9.75018	1.84E-22	2.01E-20	54.1801	31.6225	32.466	233.261	338.984	287.545	Up
Gkn3	81.1053	1.18386	0.37337	3.17075	0.00152	0.00792	36.7651	72.8277	39.1502	86.0474	115.107	136.735	Up
Arhgap25	1122.62	0.46592	0.16033	2.90598	0.00366	0.01666	1121.33	809.729	897.59	1339.44	1401.35	1166.27	Up
Prokr1	36.4132	1.75761	0.5001	3.51454	0.00044	0.00278	9.67501	15.3321	24.827	40.4319	73.9218	54.2916	Up
Aplf	466.752	0.47044	0.13956	3.37074	0.00075	0.00437	366.683	379.47	427.788	505.917	558.637	562.019	Up
1810020C	432.02	-0.551	0.25699	-2.1439	0.03204	0.09637	474.076	571.122	495.584	364.924	224.933	461.479	Down
Efcc1	170.655	-0.4327	0.21797	-1.9849	0.04716	0.13039	170.28	195.485	222.488	137.883	129.891	167.902	Down
Gp9	146.69	-1.6336	0.35048	-4.661	3.15E-06	3.48E-05	136.418	292.269	236.811	68.4232	92.9302	53.2862	Down
Cnbp	22640.3	-0.2811	0.09637	-2.9169	0.00354	0.0162	24303.6	23862.6	26350.9	20912.6	21517.6	18894.5	Down
Copg1	3810.25	0.42308	0.08388	5.04398	4.56E-07	6.06E-06	3405.61	3154.59	3206.5	4446.47	4254.73	4393.6	Up
Rab7	1105.68	0.44311	0.13441	3.2968	0.00098	0.00548	1064.25	813.562	933.875	1295.89	1235.55	1290.93	Up
Rpn1	13913.7	0.32065	0.08193	3.91378	9.09E-05	0.0007	11949.6	12220.7	12951.1	16050.4	15280.7	15029.7	Up
Gata2	6923	-1.1235	0.19462	-5.773	7.79E-09	1.45E-07	7071.47	10090.5	11308.7	4932.69	4530.35	3604.36	Down
Sec61a1	7253.67	0.55811	0.11109	5.02389	5.06E-07	6.66E-06	6505.48	5299.17	5799	9106.51	8379.56	8432.3	Up
Kbtbd12	49.7179	-2.0125	0.76175	-2.6419	0.00824	0.03262	49.3426	171.528	18.1428	23.8445	26.4006	9.04861	Down
Abtb1	2066.44	-0.4198	0.12858	-3.265	0.00109	0.00602	2284.27	2343.9	2466.46	1760.34	2005.39	1538.26	Down
Mcm2	902.502	0.55203	0.1573	3.50943	0.00045	0.00283	888.166	656.407	651.23	1093.74	1070.81	1054.67	Up
4933427D	97.2252	-1.4578	0.34692	-4.2022	2.64E-05	0.00023	161.573	137.989	127.954	59.0928	67.5856	29.1566	Down
Gm1965	35.714	0.86743	0.40714	2.13057	0.03312	0.09889	27.09	21.0817	27.6916	35.2483	54.9133	48.2592	Up
Plxna1	584.453	1.20547	0.25141	4.79493	1.63E-06	1.92E-05	439.246	235.732	385.773	672.829	788.851	984.287	Up

Chchd6	1516.83	-0.5755	0.15697	-3.6661	0.00025	0.00166	1601.21	2112	1733.11	1305.23	1044.41	1305.01	Down
Txnrd3	1393.34	0.32997	0.11458	2.87981	0.00398	0.01789	1371.92	1184.41	1147.77	1534.34	1590.37	1531.23	Up
Vmn1r42	34.2195	2.07605	0.52585	3.94796	7.88E-05	0.00062	10.6425	21.0817	7.63906	71.5334	40.129	54.2916	Up
Uroc1	10.7091	1.9169	0.73584	2.60504	0.00919	0.0356	4.83751	3.83303	4.77441	17.6242	21.1205	12.0648	Up
Cfap100	3802.07	-0.6623	0.17421	-3.8018	0.00014	0.00104	4231.85	4962.82	4784.92	3356.89	2213.43	3262.53	Down
Gm44117	21.9597	-1.6414	0.54739	-2.9985	0.00271	0.0129	24.1875	44.0799	31.5111	13.4773	8.4482	10.054	Down
Klf15	3732.52	-1.5131	0.20895	-7.2414	4.44E-13	1.70E-11	4962.32	5852.09	5770.36	2352.31	1310.53	2147.54	Down
Gm44421	151.306	-1.844	0.26877	-6.8609	6.84E-12	2.18E-10	210.915	225.191	274.051	79.8271	46.4651	71.3835	Down
Aldh1l1	1063.46	1.13223	0.20532	5.51437	3.50E-08	5.69E-07	534.061	864.349	600.621	1353.95	1331.65	1696.11	Up
Slc41a3	678.76	-0.7524	0.2571	-2.9264	0.00343	0.01579	639.518	1207.41	708.523	444.751	599.822	472.538	Down
Fbln2	809.149	-1.2999	0.28625	-4.5414	5.59E-06	5.84E-05	1124.24	1400.97	927.191	505.917	624.111	272.464	Down
Wnt7a	731.529	0.85601	0.30369	2.81875	0.00482	0.02104	683.056	368.93	509.907	928.897	1304.19	594.192	Up
Xpc	2225.12	-0.4237	0.09014	-4.7005	2.60E-06	2.92E-05	2438.1	2580.59	2629.75	1957.32	1905.07	1839.88	Down
Gm45218	48.0793	-1.3218	0.5439	-2.4303	0.01509	0.0533	117.068	60.3703	28.6465	27.9913	23.2326	31.1674	Down
Slc6a6	12757.2	0.4619	0.14278	3.23515	0.00122	0.00657	12540.8	10469.9	9185.97	14405.2	16259.6	13681.5	Up
Grip2	392.81	-0.8088	0.27235	-2.9696	0.00298	0.014	334.756	695.696	469.802	286.134	323.144	247.329	Down
Fgd5	4435.04	-0.443	0.1398	-3.1687	0.00153	0.00797	4541.45	4882.33	5907.86	3654.42	4177.64	3446.51	Down
Prickle2	5369.04	-0.2539	0.11927	-2.1291	0.03325	0.09914	6050.75	5400.75	6069.24	4588.5	5530.4	4574.57	Down
Adamts9	1656.64	-0.8451	0.27939	-3.0249	0.00249	0.01199	1239.37	2178.12	2967.78	1010.8	1407.68	1136.1	Down
9530026P	1227.78	-0.9114	0.1801	-5.0607	4.18E-07	5.59E-06	1831.48	1327.19	1650.99	690.453	955.703	910.893	Down
Slc25a26	547.455	-0.4618	0.16868	-2.7376	0.00619	0.02583	586.306	753.191	563.381	488.293	477.323	416.236	Down
Suclg2	3557.68	-0.4426	0.13147	-3.3667	0.00076	0.00443	3735.52	4560.35	4001.91	3149.54	2644.29	3254.48	Down
Tafa1	185.856	-1.1496	0.3631	-3.166	0.00155	0.00803	142.223	281.728	344.713	78.7904	130.947	136.735	Down
Tafa4	47.612	4.45273	0.66686	6.67714	2.44E-11	7.18E-10	1.935	7.66607	2.86465	150.324	67.5856	55.297	Up
Uba3	4266.33	0.26914	0.07817	3.443	0.00058	0.00349	3852.59	3881.91	3873.96	4560.51	4831.32	4597.7	Up
Arl6ip5	7853.42	0.27862	0.09084	3.06724	0.00216	0.01063	7264	7001.04	7026.98	8576.75	9208.54	8043.21	Up
Lmod3	94.241	-4.1615	0.61507	-6.766	1.32E-11	4.09E-10	114.165	344.015	77.3455	5.18358	12.6723	12.0648	Down
Mitf	886.792	0.31981	0.13993	2.28556	0.02228	0.07262	839.791	845.184	681.786	902.98	1027.51	1023.5	Up
Gm20696	308.69	-0.5373	0.15955	-3.3673	0.00076	0.00442	357.976	351.681	386.728	266.436	266.118	223.199	Down
Tpt1-ps3	6345.56	-0.2427	0.1067	-2.2743	0.02295	0.07439	7071.47	6495.08	7067.09	5836.71	6327.7	5275.34	Down
Rybp	1221.21	0.32733	0.15487	2.11358	0.03455	0.10223	1257.75	889.264	1102.89	1293.82	1333.76	1449.79	Up
Gxylt2	159.824	-0.792	0.34067	-2.325	0.02007	0.06701	180.923	304.726	122.225	121.296	116.163	113.61	Down
Cntn4	56.9314	-1.2857	0.40423	-3.1805	0.00147	0.00771	93.8476	93.9093	54.4283	43.5421	32.7368	23.1242	Down
Sumf1	2959.94	0.31096	0.1039	2.99293	0.00276	0.01311	2725.45	2605.51	2595.37	3153.69	3602.1	3077.53	Up
Bhlhe40	6853.93	1.14662	0.2028	5.65401	1.57E-08	2.73E-07	5635.7	4077.39	3082.36	9108.59	10073.4	9146.13	Up
Arl8b	7106.07	0.27651	0.08361	3.30711	0.00094	0.00531	6711.56	6438.54	6131.3	7746.34	8028.96	7579.72	Up
Edem1	4551.97	0.66669	0.08427	7.9113	2.55E-15	1.33E-13	3470.43	3448.77	3636.19	5528.81	5836.65	5390.96	Up
Lmcd1	807.661	-0.8351	0.21539	-3.8772	0.00011	0.0008	778.839	1141.29	1185.01	509.027	717.041	514.765	Down
Cav3	154.537	-1.1436	0.40804	-2.8028	0.00507	0.02196	152.865	357.43	127.954	89.1576	120.387	79.4267	Down
Srgap3	263.998	-0.4522	0.2089	-2.1646	0.03042	0.09272	322.178	314.309	278.826	223.931	175.3	269.447	Down
Gt(ROSA)2	2850.06	1.65256	0.30851	5.35657	8.48E-08	1.27E-06	953.956	942.927	2229.65	4071.18	5217.82	3684.79	Up
Lhfpl4	214.224	-1.4348	0.28287	-5.0724	3.93E-07	5.29E-06	264.128	334.432	339.938	106.782	79.2019	160.864	Down
Camk1	3422.33	0.43902	0.12262	3.58041	0.00034	0.00223	3022.47	2746.37	2947.72	3993.43	4382.51	3441.49	Up
Arpc4	4267.12	0.58444	0.13132	4.45033	8.57E-06	8.58E-05	3843.88	3166.09	3233.23	5169.07	5634.95	4555.47	Up
Cidec	969.04	-1.5319	0.71476	-2.1432	0.0321	0.09652	1157.13	2566.22	596.802	401.209	776.179	316.701	Down
Jagn1	1520.47	0.26649	0.10493	2.53959	0.0111	0.04162	1395.14	1319.52	1426.6	1786.26	1617.83	1577.47	Up
Vhl	1800.27	0.27096	0.11068	2.44811	0.01436	0.05128	1812.13	1537.05	1545.96	1981.16	1996.94	1928.36	Up
Irak2	3489.4	0.21489	0.1068	2.01201	0.04422	0.1239	2961.52	3310.78	3417.53	3598.44	4027.68	3620.45	Up
Tatdn2	2319.74	0.32231	0.12759	2.52618	0.01153	0.04299	2227.19	1859.02	2098.83	2304.62	2823.81	2604.99	Up
Ghrl	48.2687	-1.5075	0.53848	-2.7995	0.00512	0.02214	90.9451	37.3721	85.9395	41.4686	14.7844	19.1026	Down
Gm44053	55.2443	-1.1682	0.33305	-3.5077	0.00045	0.00284	72.5626	74.7442	82.1199	25.9179	35.9049	40.216	Down
Atp2b2	475.704	-1.6534	0.22987	-7.1928	6.35E-13	2.37E-11	803.994	657.365	704.704	216.674	166.852	304.636	Down
Slc6a1	51.9286	3.04975	0.62799	4.85636	1.20E-06	1.44E-05	3.87001	24.9147	4.77441	107.818	89.7622	80.4321	Up
Atg7	3019.28	0.27207	0.11007	2.47174	0.01345	0.04868	3026.34	2607.42	2572.45	3381.77	3410.96	3116.74	Up
Pparg	996.804	0.74435	0.24343	3.05772	0.00223	0.01091	986.851	756.066	492.72	1207.77	1442.53	1094.88	Up
Tsen2	406.346	-0.4977	0.13594	-3.661	0.00025	0.00169	511.808	457.089	458.344	335.896	342.152	332.788	Down
Mkrn2os	151.028	3.2459	0.25365	12.7968	1.71E-37	6.95E-35	28.0575	34.4973	23.8721	261.252	261.894	296.593	Up
Raf1	2037.18	0.29579	0.1186	2.49399	0.01263	0.04626	2021.11	1627.12	1839.1	2180.21	2202.87	2352.64	Up
Tmem40	249.154	1.116	0.314	3.55416	0.00038	0.00244	97.7176	239.565	134.638	339.006	363.273	320.723	Up
Cand2	276.016	-1.4406	0.31218	-4.6146	3.94E-06	4.27E-05	314.438	596.995	298.878	121.296	141.507	182.983	Down
Mbd4	184.79	0.58525	0.1912	3.06093	0.00221	0.01081	135.45	153.321	154.691	199.049	224.933	241.296	Up
Ift122	1351.36	-0.4072	0.14944	-2.725	0.00643	0.02662	1556.71	1587.83	1478.16	1259.61	940.919	1284.9	Down
Tmcc1	2389.6	0.26213	0.10154	2.58161	0.00983	0.03768	2283.3	2171.41	2064.46	2640.52	2749.89	2428.04	Up
9530062K	88.6836	-0.5668	0.25406	-2.2311	0.02568	0.08129	94.8151	112.116	110.766	72.5701	64.4175	77.4159	Down
Washc2	2720	0.22305	0.11363	1.96301	0.04965	0.13607	2825.1	2358.27	2347.1	2929.76	3005.45	2854.33	Up
Marchf8	4996.01	0.24651	0.08694	2.83555	0.00457	0.02014	4803.64	4322.7	4584.39	5536.06	5276.96	5452.29	Up
Alox5	878.742	1.23431	0.28848	4.2786	1.88E-05	0.00017	727.561	535.667	309.382	1252.35	1475.27	972.223	Up
Rassf4	2339.34	0.87761	0.16883	5.19822	2.01E-07	2.85E-06	1936.94	1677.91	1332.06	2786.69	3473.27	2829.2	Up
Cxcl12	13281.2	0.31037	0.12862	2.41312	0.01582	0.0554	10614.5	11399.4	13560.3	13596.5	15997.7	14519	Up
Zfp637	1192.9	-0.2441	0.1056	-2.3115	0.02081	0.06876	1362.24	1217.95	1300.55	1143.5	1063.42	1069.75	Down
Fxyd4	140.452	1.13489	0.31537	3.59858	0.00032	0.0021	52.2451	102.534	108.857	224.967	172.132	181.978	Up
Gm4875	33.6008	0.90635	0.44965	2.01566	0.04384	0.12313	28.0575	14.3739	27.6916	43.5421	51.7452	36.1944	Up
Ret	1245.63	-0.4266	0.1285	-3.32	0.0009	0.0051	1483.18	1301.32	1501.08	1133.13	944.087	1110.97	Down
Zfp248	300.085	-0.322	0.14815	-2.1738	0.02972	0.09122	312.503	343.057	344.713	261.252	274.567	264.42	Down
Zfp9	590.648	-0.6609	0.14854	-4.449	8.63E-06	8.61E-05	628.876	776.19	765.816	421.943	460.427	490.636	Down
Cacna1c	2655.36	0.42923	0.14294	3.00289	0.00267	0.01276	1978.54	2571.97	2239.2	2670.58	3211.37	3260.52	Up
Dcp1b	1237.85	-0.211	0.10488	-2.012	0.04422	0.1239	1322.57	1340.6	1321.56	1228.51	1068.7	1145.15	Down
Cacna2d4	567.732	-0.7645	0.16239	-4.7081	2.50E-06	2.83E-05	822.376	615.202	706.613	386.695	457.259	418.247	Down
Adipor2	11140.1	0.43638	0.11685	3.73463	0.00019	0.00133	9446.68	10587.8	8369.55	13024.3	13274.2	12138.2	Up
Erc1	116.461	0.66175	0.26417	2.50505	0.01224	0.04513	81.2701	102.534	86.8943	122.332	127.779	177.956	Up
Rad52	880.109	-0.2676	0.11656	-2.2954	0.02171	0.07114	882.361	981.257	1020.77	831.446	784.627	780.191	Down
Il17ra	2095.18	0.9215	0.16386	5.62355	1.87E-08	3.20E-07	1475.44	1532.26	1335.88	2551.36	3369.78	2306.39	Up
Atp6v1e1	8696.58	0.48273	0.10166	4.74848	2.05E-06	2.36E-05	7617.14	7411.17	6736.7	10459.4	10608.8	9346.21	Up

Bcl2l13	1595.48	0.2344	0.10648	2.20147	0.0277	0.08643	1503.5	1559.09	1335.88	1686.74	1760.39	1727.28	Up
Bid	714.394	0.67971	0.12052	5.63966	1.70E-08	2.94E-07	520.516	548.124	578.659	886.392	928.246	824.429	Up
Mical3	1923.52	-0.3177	0.15603	-2.0361	0.04174	0.1185	1788.91	2233.7	2380.52	1585.14	1966.32	1586.52	Down
Tuba8	954.028	0.6026	0.18921	3.18482	0.00145	0.00761	730.464	858.6	683.696	1346.69	1224.99	879.726	Up
Slc6a13	33.5898	-3.9314	0.72518	-5.4213	5.92E-08	9.21E-07	48.3751	111.158	29.6014	6.2203	3.16808	3.0162	Down
Iqsec3	4.16478	-5.4547	2.2511	-2.4231	0.01539	0.05416	7.74001	17.2487	0	0	0	0	Down
M6pr	14580.2	0.63932	0.08416	7.59641	3.04E-14	1.40E-12	11680.6	11072.7	11451	17735.1	18702.2	16839.5	Up
Apobec1	1498.66	0.87558	0.10882	8.04588	8.56E-16	4.81E-14	1000.4	1143.2	1028.41	1837.06	2024.4	1958.52	Up
Gdf3	9.35977	4.81773	1.35633	3.55203	0.00038	0.00246	1.935	0	0	17.6242	8.4482	28.1512	Up
Dppa3	8.77042	6.634	1.43847	4.61184	3.99E-06	4.32E-05	0	0	0	22.8077	13.7283	16.0864	Up
Slc2a3	497.215	-0.8937	0.18846	-4.7422	2.11E-06	2.43E-05	672.414	736.901	529.96	350.41	396.01	297.599	Down
C3ar1	453.565	1.45136	0.25609	5.66736	1.45E-08	2.54E-07	312.503	258.73	157.556	645.874	576.59	770.137	Up
Clec4a3	774.114	1.37999	0.1886	7.31691	2.54E-13	1.01E-11	461.498	501.169	326.57	1234.73	1118.33	1002.38	Up
Clec4a2	412.305	1.36398	0.22737	5.99898	1.99E-09	4.13E-08	300.893	228.066	163.285	574.341	624.111	583.133	Up
Clec4n	1554.97	3.38465	0.67237	5.03393	4.81E-07	6.34E-06	518.581	188.777	107.902	2968.12	3075.15	2471.28	Up
Clec4d	446.946	0.92553	0.3958	2.33839	0.01937	0.06509	476.978	295.144	152.781	429.2	869.109	458.463	Up
Clec4e	487.066	2.05235	0.38707	5.30229	1.14E-07	1.68E-06	277.673	206.026	84.0297	538.056	1066.59	750.029	Up
Cd163	549.952	-1.0772	0.30936	-3.4821	0.0005	0.00309	863.979	991.798	382.908	376.328	356.937	327.761	Down
Clstn3	236.222	-1.0285	0.3193	-3.2212	0.00128	0.00686	265.095	424.509	261.638	164.838	97.1543	204.096	Down
C1rl	752.033	1.04014	0.22608	4.6008	4.21E-06	4.53E-05	612.428	498.294	365.72	1029.46	807.859	1198.44	Up
C1ra	4281.78	0.40389	0.16451	2.45506	0.01409	0.05053	4359.56	3548.43	3151.11	5446.91	4096.32	5088.33	Up
C1s1	2006.2	-0.2972	0.09417	-3.1558	0.0016	0.00827	2238.8	2185.79	2211.51	1879.57	1837.48	1684.05	Down
Lpcat3	4165.31	0.8274	0.10022	8.25582	1.51E-16	9.34E-15	3051.5	3140.21	2815.95	5169.07	5726.83	5088.33	Up
Emg1	2639.93	-0.2602	0.10746	-2.4214	0.01546	0.05435	2685.78	2754.99	3191.22	2448.72	2376.06	2382.8	Down
Ptpn6	2772.79	0.86253	0.16988	5.07725	3.83E-07	5.16E-06	2375.22	1821.65	1706.38	3395.24	4170.24	3168.02	Up
Eno2	315.103	-0.7894	0.20374	-3.8744	0.00011	0.0008	324.113	477.213	396.276	260.216	219.653	213.145	Down
Lrrc23	4641.45	-0.9397	0.2209	-4.2538	2.10E-05	0.00019	5393.82	6958.87	5952.74	3507.21	2109.94	3926.09	Down
Spsb2	876.061	0.40047	0.17194	2.32906	0.01986	0.06643	879.459	723.485	662.689	1018.05	1134.17	838.504	Up
Gpr162	171.824	-0.4613	0.18712	-2.4653	0.01369	0.04939	187.695	215.608	193.841	142.03	151.012	140.756	Down
Acrbp	612.67	-0.5412	0.15837	-3.4173	0.00063	0.00378	686.926	682.28	809.741	446.825	474.155	576.095	Down
Chd4	10059.4	0.21864	0.0881	2.48162	0.01308	0.04756	9794.02	8675.12	9426.6	10806.7	11085.1	10568.8	Up
Gapdh	2531.25	0.40978	0.16851	2.43183	0.01502	0.05311	2198.16	2582.51	1741.71	2922.5	3182.86	2559.75	Up
Ltbr	5145.83	0.3163	0.10342	3.05853	0.00222	0.01089	4600.47	4255.63	4895.68	5940.38	5888.4	5294.44	Up
Plekhg6	535.669	0.26973	0.13386	2.01502	0.0439	0.12326	471.173	479.129	507.043	599.222	529.069	628.376	Up
Cd9	29837.3	0.67733	0.1449	4.67441	2.95E-06	3.29E-05	24176.9	21704.6	22995.5	37182.9	42915.8	30048.4	Up
Vwf	19442	-0.5571	0.13379	-4.1643	3.12E-05	0.00027	21203.8	26322.4	21924.1	17697.8	14214.1	15290.1	Down
Ano2	42.3466	1.15899	0.48727	2.37854	0.01738	0.05968	16.4475	25.873	36.2855	40.4319	48.5772	86.4645	Up
Kcna5	36.5878	-2.8462	0.71195	-3.9978	6.39E-05	0.00051	29.025	120.741	42.9697	3.11015	11.6163	12.0648	Down
Kcna6	15.3971	-2.3216	0.80815	-2.8728	0.00407	0.01825	45.4726	11.4991	20.0525	4.14686	3.16808	8.04321	Down
Rad51ap1	76.7403	0.98424	0.29927	3.28884	0.00101	0.00561	58.0501	46.9547	49.6539	111.965	79.2019	114.616	Up
Tigar	860.563	0.22023	0.11092	1.98557	0.04708	0.1302	770.131	833.685	781.094	892.612	979.992	905.866	Up
Ccnd2	3172	0.65112	0.13115	4.96477	6.88E-07	8.83E-06	2476.8	2262.45	2665.08	3338.23	4150.18	4139.24	Up
Cracr2a	2945.3	0.35297	0.1686	2.09356	0.0363	0.10628	2940.24	2482.85	2337.55	3182.72	2732.99	3995.46	Up
Prmt8	1955.83	1.07263	0.15821	6.77998	1.20E-11	3.73E-10	1379.66	1230.4	1171.64	2411.4	2330.65	3211.25	Up
Tspan11	4362.09	0.9577	0.15004	6.38285	1.74E-10	4.45E-09	2650.95	2907.36	3337.32	5954.9	4828.15	6493.88	Up
Tulp3	2332.63	-0.4486	0.09857	-4.5509	5.34E-06	5.60E-05	2710.94	2716.66	2649.8	1983.24	1824.81	2110.34	Down
Gm10069	355.466	-0.765	0.25183	-3.0375	0.00239	0.01157	374.423	581.663	386.728	273.693	199.589	316.701	Down
Pzp	45.6795	-1.0339	0.47611	-2.1715	0.02989	0.09161	42.5701	62.2868	79.2553	15.5507	42.241	32.1728	Down
Gm44009	72.9919	5.09383	1.906	2.67253	0.00753	0.03027	0	12.4574	0	157.581	72.8658	195.048	Up
Klrb1c	99.1682	-0.9486	0.42106	-2.2529	0.02426	0.07775	58.0501	181.111	152.781	68.4232	61.2495	73.3943	Down
Gm44511	34.7966	1.70561	0.46425	3.67387	0.00024	0.00162	14.5125	20.1234	14.3232	52.8725	70.7537	36.1944	Up
Gm44066	175.253	0.65729	0.22768	2.88697	0.00389	0.01755	132.548	141.822	133.684	261.252	171.076	211.134	Up
Gm44223	6.10992	2.73114	1.22148	2.23592	0.02536	0.08058	0.9675	3.83303	0	14.514	5.28013	12.0648	Up
Clec2d	4187.59	-0.3785	0.18723	-2.0215	0.04323	0.12176	4164.13	3876.16	6160.9	3554.9	4003.39	3366.08	Down
Gm47861	5.95838	4.14104	1.43759	2.88055	0.00397	0.01786	0	1.91652	0	8.29373	8.4482	17.0918	Up
Clec12a	1202.46	1.47967	0.13822	10.7053	9.61E-27	1.52E-24	625.973	729.235	549.058	1731.32	1718.15	1861	Up
Clec9a	2730.8	-0.228	0.09707	-2.3485	0.01885	0.06376	2802.85	2898.73	3136.79	2409.33	2505.95	2631.13	Down
Clec7a	6518.24	2.95797	0.64407	4.59262	4.38E-06	4.69E-05	2602.58	1215.07	641.681	10231.3	16349.4	8069.35	Up
Olr1	1552.05	3.30629	0.538	6.14557	7.97E-10	1.78E-08	478.913	227.107	148.962	2389.63	3567.25	2500.43	Up
Gabarapl1	9273.25	-0.4855	0.12392	-3.9178	8.94E-05	0.00069	12393.7	9821.19	10242.1	7944.35	8060.64	7177.56	Down
Klre1	145.044	-0.7648	0.35615	-2.1474	0.03176	0.09575	128.678	273.104	146.097	78.7904	110.883	132.713	Down
Klrd1	366.584	-0.4646	0.21396	-2.1713	0.02991	0.09164	347.333	516.501	411.555	261.252	299.911	362.95	Down
Klrc2	102.6	-0.8811	0.37937	-2.3225	0.0202	0.06728	91.9126	183.986	123.18	66.3498	49.6332	100.54	Down
Klrc1	60.2106	-0.8946	0.38862	-2.3019	0.02134	0.07014	56.1151	89.1181	89.759	39.3952	29.5687	57.3078	Down
Klra2	377.6	1.41481	0.21144	6.6912	2.21E-11	6.56E-10	160.605	253.939	203.39	471.706	579.758	596.203	Up
Ybx3	6649.71	-0.4525	0.13998	-3.2327	0.00123	0.00662	6783.15	8254.44	8014.33	5603.45	6385.79	4857.09	Down
Etv6	2477.55	0.41674	0.17709	2.35324	0.01861	0.06315	1637.01	2180.04	2549.54	2684.06	2651.68	3162.99	Up
Bcl2l14	682.72	1.80759	0.11987	15.0798	2.20E-51	2.26E-48	299.925	311.434	298.878	1054.34	1012.73	1119.01	Up
Gm38910	533.889	0.31742	0.12713	2.49683	0.01253	0.04596	487.621	488.712	449.75	594.038	626.223	556.992	Up
Mansc1	996.968	0.69113	0.157	4.402	1.07E-05	0.0001	664.674	818.353	804.966	1268.94	1045.47	1379.41	Up
Gpr19	290.922	-0.5012	0.21234	-2.3604	0.01826	0.06216	297.023	346.89	379.089	259.179	185.86	277.491	Down
Cdkn1b	5158.72	-0.2698	0.10381	-2.5991	0.00935	0.03616	6030.44	5382.54	5505.85	4763.71	4969.66	4300.1	Down
Hebp1	1293.4	0.97366	0.25964	3.75	0.00018	0.00126	1257.75	805.896	554.787	1670.15	1849.1	1622.72	Up
Gm36640	55.8544	-1.1528	0.38418	-3.0006	0.00269	0.01282	58.0501	93.9093	79.2553	41.4686	23.2326	39.2106	Down
Emp1	13614.3	-0.3474	0.15316	-2.2683	0.02331	0.07538	15773.2	13935	16028.7	10882.4	14637.6	10429	Down
Plbd1	3285.3	0.64611	0.1573	4.10762	4.00E-05	0.00034	2723.52	2769.37	2192.41	3905.31	3431.03	4690.2	Up
Gucy2c	105.039	0.93317	0.4145	2.25129	0.02437	0.07799	49.3426	55.579	111.721	137.883	88.7061	187.005	Up
H2aj	1975.14	0.48921	0.1796	2.72385	0.00645	0.0267	1378.69	1470.93	2080.69	2474.64	2056.08	2389.84	Up
Eps8	4503.49	1.02765	0.10623	9.67343	3.91E-22	4.09E-20	3061.17	3178.54	2652.66	6105.22	5825.04	6198.3	Up
Dera	982.092	0.74689	0.10362	7.20763	5.69E-13	2.15E-11	766.261	737.859	696.11	1244.06	1239.77	1208.49	Up
Mgst1	25329.5	-0.4429	0.16313	-2.7153	0.00662	0.02722	31899.5	28686.4	26977.4	23760.5	16562.7	24090.4	Down
Pik3c2g	477.23	2.65975	0.26715	9.95594	2.38E-23	2.73E-21	81.2701	144.697	165.195	922.677	654.736	894.807	Up
Gm28523	155.318	-0.6416	0.2975	-2.1565	0.03104	0.09411	213.818	176.32	177.608	96.4146	174.244	93.5023	Down

Pde3a	4933.71	-0.6426	0.16909	-3.8003	0.00014	0.00105	4623.69	6590.9	6829.32	3460.56	4179.75	3918.05 Down
Slco1a4	6.5861	-3.5619	1.20017	-2.9679	0.003	0.01407	8.70751	14.3739	13.3684	0	1.05603	2.0108 Down
Slco1a5	641.235	0.95595	0.29808	3.20698	0.00134	0.00714	269.933	509.794	529.005	970.366	563.918	1004.4 Up
Gys2	22.8963	-6.0325	1.33332	-4.5244	6.06E-06	6.28E-05	25.155	95.8259	14.3232	2.07343	0	0 Down
Ldhb	13710.2	-0.8573	0.21233	-4.0377	5.40E-05	0.00044	17365.7	18641	16997.9	12383.6	6468.16	10404.9 Down
Abcc9	889.34	-0.521	0.24604	-2.1177	0.0342	0.10138	908.484	1302.27	933.875	487.256	880.725	823.423 Down
Etnk1	167.938	0.86523	0.20794	4.16103	3.17E-05	0.00027	120.938	102.534	133.684	208.38	203.813	238.28 Up
Sox5	606.736	-0.6242	0.16234	-3.8452	0.00012	0.00089	615.331	796.313	796.372	507.991	428.746	495.663 Down
Lrmp	651.788	0.36751	0.18331	2.0048	0.04498	0.12561	666.609	603.703	437.336	726.738	784.627	691.716 Up
Casc1	1344.53	-0.885	0.23982	-3.6903	0.00022	0.00153	1436.74	1954.85	1841.97	1111.36	608.271	1113.98 Down
Lmntd1	340.656	-1.2595	0.18748	-6.7181	1.84E-11	5.55E-10	416.026	571.122	454.524	186.609	218.597	197.059 Down
Tuba3b	4.47424	-5.5583	1.85277	-3	0.0027	0.01284	2.9025	20.1234	3.81953	0	0	0 Down
Sspn	2076.33	-0.6683	0.16605	-4.0249	5.70E-05	0.00046	2792.21	2905.44	1948.92	1601.73	1624.17	1585.52 Down
Pthlh	70.6176	-1.7282	0.33843	-5.1065	3.28E-07	4.49E-06	96.7501	125.532	103.127	43.5421	30.6247	24.1296 Down
Ergic2	1353.17	0.34381	0.13235	2.59767	0.00939	0.03627	1185.19	1115.41	1277.63	1372.61	1449.92	1718.23 Up
Tmtc1	5428.8	-0.5747	0.11083	-5.1856	2.15E-07	3.02E-06	7154.67	6534.37	5799	4514.9	4338.15	4231.73 Down
Caprin2	424.057	-0.5571	0.1541	-3.6155	0.0003	0.00198	498.263	479.129	537.599	318.272	319.976	391.101 Down
Dennd5b	1720.56	-0.3233	0.11648	-2.7751	0.00552	0.02355	1838.25	1768.95	2130.34	1457.62	1560.81	1567.42 Down
AU018091	36.9635	2.08613	0.41787	4.99227	5.97E-07	7.77E-06	18.3825	12.4574	11.4586	63.2397	54.9133	61.3295 Up
Cacng7	170.983	-0.4741	0.20773	-2.2822	0.02248	0.07306	214.785	202.193	179.518	121.296	156.292	151.816 Down
Cacng6	11.6075	6.05516	1.41355	4.28367	1.84E-05	0.00017	0.9675	0	0	24.8812	32.7368	11.0594 Up
Leng1	621.409	0.40187	0.1117	3.59772	0.00032	0.00211	526.321	528.959	550.967	716.371	698.033	707.802 Up
Tmc4	4810.61	1.51539	0.09751	15.5404	1.85E-54	2.49E-51	2661.6	2515.43	2303.18	7137.79	6861	7384.67 Up
Mboat7	2315.51	0.3008	0.13419	2.24152	0.02499	0.07968	2103.35	1929.93	2191.46	2537.88	2912.52	2217.91 Up
Pirb	2092.25	1.68509	0.21874	7.70372	1.32E-14	6.27E-13	1218.08	1070.37	689.425	2853.04	3805.92	2916.67 Up
Gm15922	50.3911	2.58063	0.50309	5.1296	2.90E-07	4.01E-06	25.155	7.66607	10.5037	74.6435	112.995	71.3835 Up
Gm15448	40.1177	1.48142	0.44087	3.36024	0.00078	0.00452	29.025	19.1652	15.2781	65.3131	69.6977	42.2268 Up
Pira2	31.7493	2.92863	0.52822	5.54433	2.95E-08	4.88E-07	11.61	6.70781	3.81953	47.6889	63.3615	57.3078 Up
Lair1	620.026	1.44681	0.2655	5.44931	5.06E-08	8.00E-07	477.946	303.768	216.758	872.915	1003.22	845.542 Up
Cdc42ep5	418.191	-0.5743	0.20223	-2.8398	0.00451	0.01991	507.938	479.129	513.727	354.557	404.458	249.339 Down
Lilra5	1059.23	2.28322	0.27006	8.45437	2.81E-17	1.95E-15	524.386	327.724	231.082	1582.03	2062.42	1627.74 Up
Gm15931	188.375	2.31051	0.38663	5.97605	2.29E-09	4.71E-08	105.458	48.8712	35.3307	296.501	390.729	253.361 Up
Gp6	106.868	-1.3603	0.3652	-3.7247	0.0002	0.00137	102.555	226.149	132.729	47.6889	70.7537	61.3295 Down
Eps8l1	4648.15	0.86928	0.10863	8.0024	1.22E-15	6.70E-14	3099.87	3213.04	3553.12	6515.76	5605.38	5901.7 Up
Tnnt1	156.38	-1.9562	0.4004	-4.8857	1.03E-06	1.27E-05	149.963	322.933	273.097	34.2116	88.7061	69.3727 Down
Tnni3	4921.85	-4.0363	0.57834	-6.9791	2.97E-12	9.87E-11	5656.98	15873.6	6304.14	562.937	385.449	748.018 Down
Syt5	1841.26	-0.4507	0.19641	-2.2948	0.02174	0.07122	2384.89	2116.79	1878.25	1632.83	1142.62	1892.16 Down
Ptprh	57.3761	0.88223	0.41172	2.1428	0.03213	0.09656	56.1151	24.9147	40.1051	76.717	54.9133	91.4915 Up
Ppp6r1	7241.9	0.36937	0.09135	4.04339	5.27E-05	0.00043	6813.15	6107.94	6038.68	8069.8	8024.74	8397.11 Up
Tmem150	106.174	1.55933	0.28789	5.41638	6.08E-08	9.44E-07	57.0826	49.8294	54.4283	152.397	200.645	122.659 Up
Tmem238	680.666	0.34792	0.13854	2.51122	0.01203	0.04444	632.746	563.456	600.621	812.785	806.803	667.586 Up
Shisa7	89.8414	-0.933	0.33036	-2.8241	0.00474	0.02076	144.158	117.866	91.6688	47.6889	82.37	55.297 Down
Nat14	291.174	-1.0649	0.16452	-6.4725	9.64E-11	2.56E-09	413.123	376.596	392.457	172.095	181.636	211.134 Down
Ssc5d	191.934	-0.489	0.2366	-2.0669	0.03875	0.11173	262.193	180.153	230.127	191.792	142.563	144.778 Down
Sbk2	308.856	-1.9131	0.47437	-4.0329	5.51E-05	0.00045	375.391	910.346	178.563	104.708	159.46	124.67 Down
Sbk3	913.349	-4.3291	0.59661	-7.256	3.99E-13	1.53E-11	1405.78	2950.48	864.169	71.5334	65.4736	122.659 Down
Ccdc106	62.6572	-0.6952	0.32075	-2.1673	0.03021	0.09222	78.3676	82.4102	71.6162	35.2483	57.0254	51.2754 Down
Zim1	10.3678	-2.3189	1.12103	-2.0686	0.03859	0.1114	9.67501	31.6225	10.5037	8.29373	2.11205	0 Down
Peg3	538.667	-1.5369	0.21486	-7.1527	8.51E-13	3.07E-11	639.518	895.972	867.989	339.006	270.342	219.177 Down
Gm45844	108.989	-2.3925	0.30419	-7.8652	3.68E-15	1.90E-13	183.825	160.029	205.3	38.3585	43.297	23.1242 Down
Gm6842	5.12726	2.43442	1.16499	2.08964	0.03665	0.10712	0.9675	2.87478	0.95488	12.4406	9.50423	4.0216 Up
Zfp419	13.6627	2.22254	0.97657	2.27587	0.02285	0.07415	10.6425	3.83303	0	25.9179	28.5127	13.0702 Up
Zfp606	820.678	-0.3689	0.14045	-2.6263	0.00863	0.03384	885.264	909.387	980.665	682.159	644.175	822.418 Down
Zscan18	492.399	-0.4488	0.15993	-2.8061	0.00502	0.02177	563.086	581.663	560.516	350.41	417.13	481.587 Down
Rps5	24545.7	-0.3037	0.10431	-2.9121	0.00359	0.0164	27559.3	25667.9	28132.8	22942.5	23251.6	19719.9 Down
Zfp446	725.702	-0.322	0.11485	-2.8035	0.00506	0.02192	758.521	810.687	849.846	662.461	626.223	646.473 Down
Mzf1	160.119	-0.54	0.24256	-2.2264	0.02599	0.08214	178.02	212.733	178.563	153.434	98.2104	139.751 Down
Zswim9	424.231	-0.4604	0.13963	-3.2971	0.00098	0.00547	494.393	498.294	481.261	389.805	355.881	325.75 Down
Mrip-ps	41.3172	1.13074	0.47477	2.38167	0.01723	0.0593	27.09	18.2069	32.466	48.7256	36.9609	84.4537 Up
Selenow	5587.81	-0.4604	0.12716	-3.6204	0.00029	0.00195	6604.17	6317.8	6493.2	4933.73	5214.65	3963.29 Down
Zfp541	3.91073	2.83685	1.35973	2.08634	0.03695	0.10778	0.9675	1.91652	0	7.25701	5.28013	8.04321 Up
Meis3	832.527	0.43294	0.13969	3.09926	0.00194	0.00971	751.749	715.819	657.914	1024.28	1016.95	828.45 Up
Inafm1	1351.93	-0.577	0.11664	-4.9469	7.54E-07	9.60E-06	1549.94	1672.16	1633.8	1075.07	1193.31	987.304 Down
Ap2s1	3285.76	0.20021	0.09198	2.17663	0.02951	0.09076	3044.73	2998.39	3131.06	3615.03	3652.79	3272.58 Up
Prkd2	4693.03	-0.3229	0.1172	-2.7552	0.00587	0.02474	4857.82	4903.41	5886.85	3937.45	4396.23	4176.44 Down
Ptgir	428.87	1.35352	0.24716	5.47632	4.34E-08	6.96E-07	302.828	210.817	210.074	620.993	774.067	454.441 Up
Calm3	13450.5	0.4081	0.07566	5.39396	6.89E-08	1.06E-06	11632.3	11852.7	11197	15679.3	15425.4	14916.1 Up
Pnmal2	170.979	-0.9521	0.29132	-3.2682	0.00108	0.00596	247.68	190.693	237.766	131.663	144.675	73.3943 Down
Ccdc8	349.16	-0.6067	0.21169	-2.8659	0.00416	0.01857	488.588	390.011	385.773	306.868	312.584	211.134 Down
Hif3a	363.679	-1.659	0.30636	-5.4151	6.13E-08	9.50E-07	458.596	678.447	520.411	205.27	97.1543	222.194 Down
Psg16	12.8637	-1.5469	0.77249	-2.0024	0.04524	0.12609	7.74001	22.9982	26.7367	8.29373	7.39218	4.0216 Down
Mill2	2835.73	-1.0356	0.13838	-7.4839	7.22E-14	3.16E-12	3348.52	4134.89	3952.26	1642.16	2084.59	1851.95 Down
Pglyrp1	1882.58	1.91434	0.47518	4.02863	5.61E-05	0.00045	729.496	1161.41	477.441	3699	1721.32	3506.84 Up
Nova2	1193.76	-0.7251	0.20903	-3.4691	0.00052	0.00322	1162.94	1304.19	1995.71	893.649	846.932	959.152 Down
Foxa3	35.0258	1.05972	0.44973	2.35638	0.01845	0.06272	18.3825	32.5808	17.1879	42.5053	41.185	58.3133 Up
Dmwd	2451.71	-0.7815	0.12294	-6.3571	2.06E-10	5.20E-09	3075.69	3000.31	3223.68	1884.75	1969.49	1556.36 Down
Dmpk	2927.13	-1.1082	0.1987	-5.5773	2.44E-08	4.09E-07	3511.06	5235.93	3250.42	2130.45	1750.89	1684.05 Down
Six5	1393.76	-0.2964	0.12779	-2.3194	0.02038	0.0677	1428.03	1500.63	1680.59	1390.24	1161.63	1201.45 Down
Qpctl	868.721	0.48105	0.13778	3.4915	0.00048	0.003	821.409	629.576	724.756	1014.94	981.048	1040.59 Up
Snrpd2	1550.42	-0.2393	0.11854	-2.019	0.04349	0.1224	1643.78	1522.67	1869.66	1473.17	1409.79	1383.43 Down
Gpr4	734.267	-0.4628	0.13971	-3.3126	0.00092	0.00522	833.986	836.56	882.312	612.699	700.145	539.9 Down
Opa3	2063.83	0.34783	0.10393	3.34676	0.00082	0.0047	1670.88	1945.27	1832.42	2357.49	2355.99	2220.93 Up
Rtn2	951.148	-0.6693	0.14973	-4.4701	7.82E-06	7.89E-05	1134.88	1210.28	1158.27	778.574	822.644	602.235 Down

D830036(	34.9095	-1.4292	0.46234	-3.0913	0.00199	0.00994	54.1801	65.1616	33.4209	22.8077	14.7844	19.1026	Down
Ercc1	1033.84	-0.4001	0.1248	-3.206	0.00135	0.00716	1093.28	1193.03	1242.3	924.751	949.367	800.299	Down
Cd3eap	367.682	0.47774	0.15972	2.99115	0.00278	0.01318	328.95	280.77	312.247	411.576	479.436	393.112	Up
Ckm	1309.24	-6.1989	1.09741	-5.6486	1.62E-08	2.81E-07	1251.95	5165.01	1333.02	15.5507	8.4482	81.4375	Down
Clasrp	1966.4	-0.2779	0.09883	-2.8118	0.00493	0.02146	2152.69	2030.55	2282.17	1801.81	1829.04	1702.14	Down
Relb	1671.21	0.61604	0.11586	5.317	1.05E-07	1.56E-06	1370.95	1215.07	1373.12	1857.79	2175.41	2034.93	Up
Clptm1	8601.09	0.32243	0.10574	3.04926	0.00229	0.01118	8275.04	7344.09	7312.49	9721.28	10167.4	8786.2	Up
Nectin2	3366.55	0.94703	0.13142	7.20595	5.76E-13	2.17E-11	2415.85	2091.88	2391.03	4436.11	4987.61	3876.83	Up
Bcam	36901.3	0.66087	0.11931	5.53896	3.04E-08	5.02E-07	32359.1	24859.1	28564.4	46392	44816.7	44416.6	Up
Cblc	1348.5	1.78813	0.15347	11.6517	2.25E-31	5.00E-29	595.013	622.868	598.712	2320.17	2265.17	1689.07	Up
Bcl3	1215.61	0.58084	0.19843	2.92727	0.00342	0.01575	720.789	989.881	1211.75	1294.86	1506.95	1569.43	Up
Igsf23	75.8333	-1.1978	0.34895	-3.4325	0.0006	0.00361	98.6851	143.739	74.4809	44.5788	42.241	51.2754	Down
Ceacam2(	13.2624	4.30119	1.02919	4.1792	2.93E-05	0.00026	1.935	1.91652	0	18.6609	15.8404	41.2214	Up
Nlrp9b	21.9136	4.21405	0.77243	5.45559	4.88E-08	7.75E-07	1.935	4.79129	0	37.3218	41.185	46.2484	Up
Zfp109	119.71	0.79116	0.28966	2.73137	0.00631	0.02622	94.8151	79.5355	88.8041	147.214	109.827	198.064	Up
Zfp61	959.211	0.35366	0.10937	3.23365	0.00122	0.0066	889.134	790.563	846.981	1119.65	1061.31	1047.63	Up
Zfp94	299.871	-0.4258	0.17729	-2.4019	0.01631	0.05677	376.358	321.975	333.254	272.656	216.485	278.496	Down
Kcnn4	404.451	-0.4493	0.15618	-2.8771	0.00401	0.01803	459.563	500.211	441.156	370.108	296.743	358.928	Down
Plaur	4004.48	0.56281	0.15555	3.61809	0.0003	0.00197	3212.1	2877.65	3609.46	4934.77	5461.76	3931.12	Up
AC16116(	16.8336	-1.3857	0.58137	-2.3836	0.01715	0.05907	29.025	19.1652	24.827	9.33044	11.6163	7.03781	Down
Irgq	1709.89	0.3024	0.11008	2.74697	0.00601	0.02522	1672.81	1469.01	1452.38	1916.89	1784.68	1963.55	Up
Cd177	6232.75	3.85131	1.15646	3.33025	0.00087	0.00494	910.419	1136.49	376.224	12407.4	749.778	21816.2	Up
Ceacam1(	548.159	5.26199	0.97766	5.38221	7.36E-08	1.12E-06	27.09	30.6643	25.7818	1148.68	119.331	1937.41	Up
Rps19	10127.9	-0.8239	0.14431	-5.7096	1.13E-08	2.02E-07	13588.6	10972.1	14270.7	7684.14	7918.08	6334.03	Down
Rabac1	11198.9	-0.4826	0.11831	-4.079	4.52E-05	0.00037	12812.6	13014.1	13336.8	9903.75	10122	8004	Down
Grik5	1236.73	0.23769	0.10924	2.17578	0.02957	0.09086	1183.25	1053.13	1168.78	1391.27	1360.16	1263.79	Up
Pafah1b3	1199.8	0.89153	0.13353	6.67682	2.44E-11	7.19E-10	905.581	812.603	803.057	1642.16	1680.14	1355.28	Up
4732471J(	286.776	0.83709	0.17053	4.90864	9.17E-07	1.14E-05	224.46	180.153	212.939	337.969	378.057	387.079	Up
Dmac2	1405.32	-0.346	0.11674	-2.9637	0.00304	0.01425	1697	1557.17	1464.79	1293.82	1282.01	1137.11	Down
Tgfb1	3792.22	0.51169	0.16189	3.16078	0.00157	0.00815	3384.32	2928.44	3067.08	4254.68	5449.09	3669.71	Up
Axl	6796.93	0.91516	0.16185	5.65434	1.56E-08	2.73E-07	5380.28	4615.93	4135.6	8236.71	10678.5	7734.55	Up
Cyp2s1	16001.6	-0.7684	0.20753	-3.7027	0.00021	0.00147	18465.7	20989.7	21039.9	14333.6	7922.3	13258.2	Down
Cyp2a4	203.298	1.7744	0.49208	3.60589	0.00031	0.00205	125.775	80.4937	69.7065	283.023	109.827	550.96	Up
Cyp2b19	159.859	-1.7227	0.31669	-5.4396	5.34E-08	8.43E-07	228.33	254.897	253.044	92.2677	40.129	90.4861	Down
Cyp2g1	59.7954	-2.0432	0.36872	-5.5415	3.00E-08	4.95E-07	80.3026	89.1181	119.36	26.9546	16.8964	26.1404	Down
Cyp2f2	107674	-1.4023	0.19028	-7.37	1.71E-13	7.00E-12	155440	160530	152751	69793.8	41148	66382.6	Down
Numbl	1081.05	-0.5806	0.14033	-4.1375	3.51E-05	0.0003	1427.06	1128.83	1331.11	920.604	895.51	783.207	Down
Ltbp4	22964.6	-0.5953	0.09464	-6.2902	3.17E-10	7.68E-09	26545.3	28112.4	28251.2	18974	19171.1	16733.9	Down
Shkbp1	1560.76	0.59936	0.14463	4.14418	3.41E-05	0.00029	1384.49	1162.37	1176.42	1856.76	2142.68	1641.82	Up
Sptbn4	30.1271	0.85525	0.43368	1.97209	0.0486	0.13354	26.1225	17.2487	21.0074	33.1749	36.9609	46.2484	Up
Blvrb	1363.32	0.48298	0.20816	2.32024	0.02033	0.06756	1455.12	1072.29	884.222	1642.16	1811.08	1315.06	Up
Prx	9641.3	-0.3539	0.15089	-2.3457	0.01899	0.06413	9468.94	9796.28	13188.8	7845.87	8884.34	8663.54	Down
Pld3	8910.24	1.51579	0.1785	8.49188	2.03E-17	1.43E-15	5646.34	4351.45	3853.91	13053.3	15410.6	11145.9	Up
Akt2	7592.1	0.21498	0.09179	2.3422	0.01917	0.06458	7218.53	6974.21	6889.48	8069.8	8755.51	7645.07	Up
Zfp607b	127.686	-0.6292	0.26334	-2.3895	0.01687	0.05836	132.548	145.655	187.157	111.965	79.2019	109.589	Down
Zfp60	1034.96	-0.2322	0.11145	-2.0833	0.03723	0.10837	1081.67	1061.75	1210.79	921.64	954.647	979.26	Down
Zfp626	814.301	-0.3545	0.11184	-3.1695	0.00153	0.00795	946.216	849.017	946.289	721.554	714.929	707.802	Down
Zfp780b	685.798	-0.2809	0.12585	-2.2316	0.02564	0.08121	820.441	685.155	751.493	609.589	616.719	631.392	Down
Fcgbp	1118.09	2.43187	0.29392	8.27402	1.30E-16	8.09E-15	257.355	522.251	269.277	2037.15	1343.26	2279.24	Up
Eid2b	442.452	-0.3056	0.13524	-2.26	0.02382	0.07665	495.361	506.919	465.028	393.952	423.466	369.988	Down
Dll3	5.90852	-5.9596	1.61154	-3.6981	0.00022	0.00149	4.83751	16.2904	14.3232	0	0	0	Down
Supt5	1799.5	0.33307	0.14142	2.35512	0.01852	0.06287	1759.89	1347.31	1671.05	2041.29	1826.92	2150.55	Up
Rps16	15471.9	-0.267	0.12909	-2.0681	0.03863	0.11148	17224.4	15211.4	18262.1	14986.8	15012.5	12134.2	Down
Paf1	4202.41	-0.2547	0.10219	-2.4919	0.01271	0.04646	4161.22	4704.09	4851.76	3902.2	3657.02	3938.16	Down
Fbxo27	59.7952	-0.856	0.37826	-2.2631	0.02363	0.07616	54.1801	86.2433	90.7139	31.1015	42.241	54.2916	Down
Ech1	6499.34	-0.5095	0.17229	-2.9576	0.0031	0.01449	6991.17	9359.31	6555.27	5949.71	5622.28	4518.27	Down
Lgals4	881.461	-1.0921	0.13321	-8.1979	2.45E-16	1.47E-14	1123.27	1292.69	1184.05	629.287	519.564	539.9	Down
Actn4	16256.9	0.32289	0.0985	3.27791	0.00105	0.00578	14738.9	13261.3	15335.4	17983.9	19011.6	17210.5	Up
Rasgrp4	551.87	0.67248	0.22464	2.99351	0.00276	0.01309	551.476	363.18	361.901	612.699	824.756	597.208	Up
Fam98c	637.25	0.30814	0.1174	2.62475	0.00867	0.03396	588.241	536.625	583.433	720.518	717.041	677.64	Up
Spred3	327.073	0.86253	0.22254	3.87584	0.00011	0.0008	266.063	205.067	225.352	369.071	357.993	538.895	Up
Psmd8	6779.33	0.3766	0.09166	4.10845	3.98E-05	0.00034	5816.62	6251.68	5629.99	7806.47	7921.25	7249.95	Up
Catsperg1	362.487	-0.6862	0.15761	-4.3535	1.34E-05	0.00013	458.596	401.51	481.261	253.995	281.959	297.599	Down
Kcnk6	1287.18	0.66909	0.1175	5.69462	1.24E-08	2.20E-07	1042	919.928	1019.81	1471.1	1712.87	1557.37	Up
2200002D	258.131	0.48244	0.16656	2.8964	0.00377	0.01712	204.143	213.692	228.217	330.712	292.519	279.501	Up
Spint2	18492.4	0.44785	0.09918	4.51541	6.32E-06	6.52E-05	15136.6	15648.4	16149.9	23464	20067.7	20488.1	Up
Ppp1r14a	1701.77	-1.0391	0.21788	-4.7689	1.85E-06	2.15E-05	2026.92	2378.4	2462.64	1255.46	1342.21	745.002	Down
Sipa1l3	4132.73	0.37638	0.09874	3.81173	0.00014	0.00101	3351.43	3513.93	3924.57	4651.74	4748.95	4605.74	Up
Zfp420	586.309	-0.5813	0.14217	-4.0887	4.34E-05	0.00036	714.016	623.826	770.59	465.485	500.556	443.382	Down
Zfp568	1239.46	0.3016	0.10854	2.77884	0.00546	0.02331	1060.38	1115.41	1155.41	1280.34	1364.38	1460.85	Up
Cox7a1	188.868	-2.7261	0.47031	-5.7964	6.78E-09	1.27E-07	206.078	571.122	207.21	25.9179	67.5856	55.297	Down
Capns1	19824.4	0.2034	0.09846	2.06577	0.03885	0.11196	17995.5	18711	18581.1	21801.1	22695	19162.9	Up
Clip3	289.234	-0.7405	0.24107	-3.0715	0.00213	0.0105	410.221	301.851	373.359	204.233	279.847	165.891	Down
Tyrobp	2456.87	1.49325	0.25701	5.81018	6.24E-09	1.18E-07	1741.5	1237.11	885.176	3492.7	4594.77	2789.99	Up
Nfkbid	389.183	0.76254	0.23241	3.28095	0.00103	0.00573	354.106	268.312	243.495	465.485	614.607	389.09	Up
Hspb6	1298.11	-1.6486	0.51567	-3.1971	0.00139	0.00735	1154.23	3395.11	1355.93	647.947	499.5	735.953	Down
Upk1a	140.424	-1.9022	0.3583	-5.3091	1.10E-07	1.62E-06	334.756	140.864	189.067	64.2764	62.3055	51.2754	Down
Dmkn	5084.99	1.00779	0.21823	4.61808	3.87E-06	4.21E-05	2858.97	4137.76	3136.79	6064.79	5321.31	8990.29	Up
Ffar2	281.473	2.27444	0.26691	8.52123	1.58E-17	1.13E-15	118.035	101.575	69.7065	348.337	556.525	494.657	Up
Ffar3	2.07387	4.55283	2.03693	2.23514	0.02541	NA	0	0	0	4.14686	5.28013	3.0162	Up
Cd22	1199.06	1.18682	0.28605	4.14892	3.34E-05	0.00029	1131.98	564.414	499.404	1478.36	1914.57	1605.63	Up
Mag	157.909	1.43742	0.21105	6.81078	9.71E-12	3.05E-10	89.9776	92.9511	72.5711	246.738	227.045	218.172	Up
Lsr	1349.17	1.08789	0.12308	8.83866	9.69E-19	7.68E-17	968.469	840.393	781.094	1923.11	1797.36	1784.59	Up

Fxyd5	5014.59	0.77451	0.1466	5.283	1.27E-07	1.85E-06	3807.12	3560.89	3731.68	6266.95	7498.84	5222.05	Up
Fxyd7	151.921	-1.5777	0.32129	-4.9104	9.09E-07	1.13E-05	152.865	227.107	302.698	88.1208	83.426	57.3078	Down
Fxyd1	2011.47	-1.3696	0.13366	-10.247	1.22E-24	1.57E-22	2519.37	3236.04	2945.81	1080.26	1217.6	1069.75	Down
Hpn	1380.59	0.61625	0.12883	4.78359	1.72E-06	2.02E-05	1163.9	1138.41	968.251	1761.38	1513.28	1738.34	Up
Gm12762	39.0838	1.37665	0.67298	2.04559	0.0408	0.11631	12.5775	37.3721	15.2781	43.5421	22.1765	103.556	Up
Gm12766	121.669	0.64977	0.25581	2.54001	0.01108	0.04159	85.1401	117.866	81.165	129.589	158.404	157.848	Up
Kctd15	921.831	-0.5164	0.12614	-4.0942	4.24E-05	0.00035	1161	1097.21	996.898	743.325	824.756	707.802	Down
Chst8	299.644	-0.554	0.27455	-2.0179	0.0436	0.12264	357.976	253.939	457.389	239.481	303.079	185.999	Down
Cebpa	7632.71	0.68444	0.18454	3.70888	0.00021	0.00144	7179.83	5126.68	5259.49	10311.2	10461	7458.06	Up
Slc7a10	1885.87	-1.1406	0.1675	-6.8097	9.78E-12	3.06E-10	2107.22	2530.76	3146.34	1191.19	1261.95	1077.79	Down
Lrp3	663.174	-0.4736	0.1181	-4.0104	6.06E-05	0.00049	782.709	735.943	794.463	585.744	553.357	526.83	Down
Gpatch1	1110.52	-0.2645	0.10745	-2.4619	0.01382	0.04978	1117.46	1265.86	1252.81	1007.69	994.776	1024.5	Down
Cep89	971.112	-0.5803	0.11896	-4.8781	1.07E-06	1.31E-05	1118.43	1187.28	1185.96	799.308	701.201	834.483	Down
Nudt19	888.211	-0.3951	0.1278	-3.0918	0.00199	0.00993	955.891	1125.95	945.334	807.602	764.562	729.921	Down
Pdcd5	1005.72	-0.529	0.15383	-3.4391	0.00058	0.00354	1124.24	1092.41	1347.34	863.584	903.958	702.775	Down
Zfp536	27.5909	-1.7453	0.53278	-3.2758	0.00105	0.00582	28.0575	56.5373	42.9697	12.4406	8.4482	17.0918	Down
Uri1	3579.29	0.25565	0.08864	2.88412	0.00393	0.01769	3327.24	3282.04	3179.76	3663.75	3981.22	4041.71	Up
Gm30684	23.0584	3.7462	0.66897	5.59992	2.14E-08	3.63E-07	1.935	4.79129	2.86465	51.8358	31.6808	45.243	Up
Ccne1	124.641	0.94494	0.27274	3.4646	0.00053	0.00326	108.36	86.2433	61.1125	152.397	165.796	173.934	Up
Plekhf1	469.337	-0.6695	0.16266	-4.1158	3.86E-05	0.00033	534.061	662.157	532.825	388.768	324.2	374.009	Down
Zfp939	250.482	-0.4561	0.19385	-2.353	0.01862	0.06316	313.47	260.646	295.059	182.462	248.166	203.091	Down
Gm37494	3153.53	-0.3407	0.12946	-2.6314	0.0085	0.03341	3370.78	3281.08	3920.75	2530.62	2699.2	3118.75	Down
Vstm2b	66.2574	-0.9373	0.39527	-2.3713	0.01773	0.06069	80.3026	104.45	76.3906	24.8812	61.2495	50.27	Down
AI987944	940.55	0.31781	0.10128	3.13799	0.0017	0.00869	862.044	825.061	825.019	1011.83	1068.7	1050.64	Up
Gm9294	131.401	-0.6168	0.27531	-2.2403	0.02507	0.07988	164.475	120.741	191.931	88.1208	124.611	98.5293	Down
Siglecf	852.448	2.16827	0.54757	3.95981	7.50E-05	0.00059	500.198	264.479	166.15	1240.95	1952.59	990.32	Up
Nkg7	271.394	-0.9364	0.2791	-3.355	0.00079	0.00458	314.438	442.715	312.247	130.626	252.39	175.945	Down
Cd33	2021.17	1.49516	0.20572	7.26794	3.65E-13	1.41E-11	1396.1	955.384	824.064	2606.3	3357.1	2988.05	Up
Siglece	296.987	0.94413	0.24659	3.82878	0.00013	0.00095	256.388	204.109	148.962	315.162	434.026	423.274	Up
Klk14	156.951	1.41755	0.30881	4.59032	4.43E-06	4.74E-05	107.393	48.8712	100.263	210.453	234.438	240.291	Up
Klk8	407.853	-0.5044	0.2255	-2.237	0.02528	0.08042	506.003	574.955	354.262	369.071	364.329	278.496	Down
Klk1b3	8.19956	2.6662	1.30668	2.04044	0.04131	0.11749	0	0	6.68418	17.6242	15.8404	9.04861	Up
Shank1	77.4515	-1.3241	0.37695	-3.5126	0.00044	0.0028	97.7176	89.1181	145.142	33.1749	63.3615	36.1944	Down
1700008C	21.6915	1.10523	0.47966	2.30419	0.02121	0.06982	14.5125	13.4156	13.3684	32.1382	29.5687	27.1458	Up
Lrrc4b	47.0954	-1.9273	0.50116	-3.8457	0.00012	0.00089	83.2051	49.8294	90.7139	8.29373	26.4006	24.1296	Down
Josd2	2031.15	-0.3019	0.13325	-2.266	0.02345	0.07574	2207.84	2541.3	1979.47	1819.44	1956.82	1682.04	Down
5430431A	110.023	-2.2986	0.64529	-3.5621	0.00037	0.00237	88.0426	386.178	74.4809	25.9179	22.1765	63.3403	Down
Mybpc2	381.858	2.38282	0.26295	9.06184	1.28E-19	1.08E-17	110.295	152.363	105.992	485.183	578.702	858.612	Up
Napsa	112.565	2.15257	0.33262	6.47164	9.69E-11	2.57E-09	60.9526	26.8312	36.2855	173.132	171.076	207.113	Up
Kcnc3	1792.51	1.28603	0.11357	11.3237	1.00E-29	1.96E-27	976.209	1103.91	1047.51	2430.06	2777.35	2420	Up
Vrk3	2756.77	0.32713	0.10192	3.2098	0.00133	0.00709	2377.15	2622.75	2336.6	3116.37	3210.32	2877.46	Up
Atf5	3145.15	-0.8712	0.18618	-4.6794	2.88E-06	3.21E-05	4212.5	4198.13	3789.93	2515.07	2557.69	1597.58	Down
Ap2a1	2271.96	0.38118	0.12002	3.17593	0.00149	0.00781	2216.55	1928.97	1775.13	2641.55	2628.45	2441.11	Up
Prrg2	1465.89	0.42301	0.1135	3.72707	0.00019	0.00136	1353.53	1227.53	1176.42	1762.42	1717.1	1558.37	Up
Rcn3	1793.37	-0.5533	0.15098	-3.6645	0.00025	0.00167	2155.59	2469.43	1774.17	1525.01	1505.89	1330.15	Down
Slc17a7	394.991	-5.7025	1.23603	-4.6136	3.96E-06	4.29E-05	383.131	1621.37	320.841	2.07343	6.33615	36.1944	Down
Gfy	68.4055	-2.4895	0.34745	-7.1652	7.77E-13	2.83E-11	101.588	107.325	139.413	23.8445	22.1765	16.0864	Down
Ccdc155	440.631	-2.0664	0.1477	-13.991	1.78E-44	1.13E-41	697.569	733.068	703.749	183.499	149.956	175.945	Down
Dkkl1	302.875	-0.7748	0.20316	-3.8135	0.00014	0.001	427.636	363.18	356.171	174.168	239.718	256.377	Down
Tead2	860.276	-0.4743	0.1391	-3.4099	0.00065	0.00388	1084.57	865.308	1051.33	750.582	740.274	669.597	Down
Trpm4	3001.15	0.24741	0.09167	2.69897	0.00696	0.02838	2720.61	2774.16	2738.6	3471.96	3077.26	3224.32	Up
Hrc	757.609	-5.2532	0.88418	-5.9413	2.83E-09	5.74E-08	933.639	2744.45	751.493	27.9913	12.6723	75.4051	Down
Ppfia3	137.691	1.02772	0.27884	3.68569	0.00023	0.00156	97.7176	108.283	65.8869	223.931	158.404	171.924	Up
Ntf5	410.659	0.89275	0.14817	6.02527	1.69E-09	3.56E-08	302.828	281.728	277.871	545.313	577.646	478.571	Up
Ruvbl2	3543.49	-0.3869	0.11663	-3.3172	0.00091	0.00515	3657.16	4263.29	4127	3288.46	2773.12	3151.93	Down
Gys1	2678.99	0.55441	0.13029	4.25508	2.09E-05	0.00019	2113.02	2511.6	1886.85	3210.71	3138.51	3213.26	Up
Ftl1	44842.9	0.42887	0.18526	2.31492	0.02062	0.06832	47193.8	33495.9	33989.1	51414.9	61080.5	41883	Up
Bcat2	2511	-0.4325	0.10717	-4.0352	5.45E-05	0.00044	2793.18	2757.87	3102.41	2272.48	2156.4	1983.66	Down
Fut1	21.5422	2.89087	0.80275	3.6012	0.00032	0.00208	3.87001	5.74955	5.7293	77.7537	20.0645	16.0864	Up
Rasip1	2212.34	-0.5225	0.14568	-3.5865	0.00034	0.00219	2496.15	2278.74	3050.85	1691.92	1996.94	1759.45	Down
Fut2	251.543	1.582	0.44128	3.58504	0.00034	0.0022	61.9201	240.523	75.4357	375.291	378.057	378.031	Up
Sec1	286.566	2.67455	0.35851	7.46014	8.64E-14	3.71E-12	117.068	78.5772	37.2404	433.347	618.831	434.333	Up
Car11	231.814	-0.6702	0.31475	-2.1293	0.03323	0.0991	355.073	160.987	338.029	162.764	210.149	163.88	Down
Fam83e	1615.8	2.1811	0.26106	8.3549	6.55E-17	4.28E-15	440.213	885.431	425.878	2663.32	2675.97	2603.99	Up
Sult2b1	193.384	0.66555	0.24313	2.73741	0.00619	0.02585	174.15	158.113	116.496	249.849	193.253	268.442	Up
Kcnj14	28.0265	-2.3257	0.51176	-4.5445	5.51E-06	5.76E-05	41.6026	49.8294	48.699	12.4406	10.5603	5.027	Down
Kcnj11	217.016	-2.512	0.88074	-2.8522	0.00434	0.01925	165.443	809.729	132.729	82.9373	34.8488	76.4105	Down
Tph1	13.5064	2.21408	1.09285	2.02596	0.04277	0.12072	0	12.4574	1.90977	26.9546	10.5603	29.1566	Up
Saa3	787.796	4.40977	0.30982	14.2331	5.71E-46	3.97E-43	95.7826	45.9964	70.6613	1506.35	982.104	2025.88	Up
Saa4	13.4158	3.98123	1.13656	3.50287	0.00046	0.00289	0	4.79129	0	19.6976	14.7844	41.2214	Up
Saa1	14.4759	2.71015	0.86852	3.12042	0.00181	0.00915	3.87001	0	7.63906	31.1015	21.1205	23.1242	Up
Ldha	25613.7	0.55305	0.09856	5.61145	2.01E-08	3.41E-07	19374.2	22751.9	20164.3	30513.7	31485.4	29392.9	Up
Uevld	962.656	0.33032	0.15781	2.09308	0.03634	0.10638	1022.65	775.231	761.042	999.394	1063.42	1154.2	Up
Tmem86a	911.984	0.45573	0.2304	1.97798	0.04793	0.13209	1005.23	710.07	592.027	1142.46	1200.7	821.413	Up
Ptpn5	68.8434	1.1751	0.34592	3.39705	0.00068	0.00403	48.3751	26.8312	51.5637	87.0841	107.715	91.4915	Up
Gm44969	6.34898	6.16824	1.51856	4.06191	4.87E-05	0.0004	0	0	0	14.514	9.50423	14.0756	Up
Zdhhc13	1390.42	0.31658	0.09225	3.43175	0.0006	0.00361	1273.23	1250.53	1191.69	1549.89	1544.97	1532.23	Up
Csrp3	2263.68	-5.7776	1.16885	-4.943	7.69E-07	9.77E-06	2284.27	8405.84	2648.85	44.5788	9.50423	189.015	Down
Htatip2	1488.65	0.32262	0.13563	2.37869	0.01737	0.05967	1473.5	1383.73	1111.48	1642.16	1681.19	1639.81	Up
Prmt3	893.768	-0.2934	0.12558	-2.3367	0.01945	0.06535	926.866	993.714	1032.23	785.831	886.005	737.964	Down
Gm45235	23.8707	-1.1541	0.5548	-2.0803	0.0375	0.10896	27.09	29.706	42.0148	7.25701	20.0645	17.0918	Down
Svip	1376.37	-0.2665	0.12726	-2.0941	0.03625	0.10619	1685.39	1505.42	1318.69	1247.17	1218.65	1282.89	Down
Siglech	186.05	-0.7941	0.38218	-2.0777	0.03774	0.10955	337.658	235.732	134.638	83.974	158.404	165.891	Down

Cyfip1	6572.3	0.22657	0.10654	2.12655	0.03346	0.09962	6116.54	5610.6	6444.5	6831.96	7711.1	6719.09	Up
Nipa2	1995.83	0.94504	0.10717	8.81846	1.16E-18	9.07E-17	1390.3	1355.94	1347.34	2446.65	2885.06	2549.7	Up
Atp10a	1847.67	0.7774	0.13185	5.89625	3.72E-09	7.39E-08	1406.75	1451.76	1226.07	2200.95	2635.84	2164.63	Up
Ube3a	1255.15	0.26851	0.13201	2.03409	0.04194	0.11898	1147.46	1063.67	1205.06	1211.92	1367.55	1535.25	Up
Snhg14	530.997	-0.4066	0.15922	-2.5538	0.01065	0.0403	530.191	709.111	576.749	460.302	435.082	474.549	Down
Gm10297	372.88	-0.4039	0.13341	-3.0274	0.00247	0.01191	425.701	438.882	409.645	320.345	319.976	322.734	Down
Klf13	6043.79	-0.2569	0.08161	-3.1481	0.00164	0.00846	6674.79	6334.09	6732.88	5581.68	5295.97	5643.32	Down
Gm32633	420.732	0.53004	0.18251	2.90412	0.00368	0.01675	419.896	293.227	319.886	469.632	487.884	533.868	Up
Fan1	316.157	0.39648	0.19885	1.99388	0.04617	0.12814	286.38	223.274	309.382	316.198	348.488	413.22	Up
Mcee	1660.08	-0.4054	0.1341	-3.0227	0.00251	0.01207	1763.76	2038.22	1873.48	1573.73	1231.33	1479.95	Down
Apba2	92.2007	-0.809	0.30333	-2.667	0.00765	0.03066	99.6527	135.114	117.451	47.6889	72.8658	80.4321	Down
Pcsk6	242.371	-0.8327	0.24954	-3.3369	0.00085	0.00484	322.178	312.392	296.969	162.764	125.667	234.258	Down
Snrpa1	1302.74	-0.3515	0.11603	-3.029	0.00245	0.01185	1449.32	1352.1	1580.33	1143.5	1214.43	1076.78	Down
Chsy1	1328.94	0.58589	0.1508	3.88514	0.0001	0.00077	1260.65	983.173	944.379	1648.38	1682.25	1454.82	Up
Lrrk1	4439.04	0.31145	0.11631	2.67782	0.00741	0.02988	4301.51	3513.93	4069.71	4746.09	5269.57	4733.43	Up
Aldh1a3	87.4026	-1.5958	0.3565	-4.4765	7.59E-06	7.71E-05	147.06	163.862	83.0748	46.6522	48.5772	35.189	Down
Cers3	88.6006	1.37622	0.29745	4.62678	3.71E-06	4.05E-05	40.6351	58.4538	48.699	109.892	156.292	117.632	Up
Adamts17	282.498	-1.6271	0.36683	-4.4355	9.18E-06	9.10E-05	197.37	596.037	486.99	158.618	138.339	117.632	Down
Igf1r	4453.39	-0.6954	0.08358	-8.32	8.80E-17	5.61E-15	5331.9	5572.27	5614.71	3257.36	3518.68	3425.4	Down
Pgpep1l	32.6774	-1.4386	0.65456	-2.1978	0.02797	0.08709	49.3426	78.5772	15.2781	12.4406	25.3446	15.081	Down
Gm44889	2.9217	5.04816	2.47186	2.04225	0.04113	0.1171	0	0	0	14.514	0	3.0162	Up
Gm29683	151.874	-1.1061	0.24291	-4.5534	5.28E-06	5.54E-05	213.818	182.069	226.307	73.6068	110.883	104.562	Down
Nr2f2	4323	-0.209	0.10441	-2.0015	0.04534	0.12631	4638.2	4619.76	4648.37	4043.19	4374.06	3614.42	Down
B130024G	356.486	-0.5896	0.2177	-2.7083	0.00676	0.02772	367.651	429.3	487.945	221.857	351.656	280.507	Down
Gm44808	93.836	-1.154	0.3616	-3.1913	0.00142	0.00747	97.7176	161.946	128.909	58.0561	36.9609	79.4267	Down
Rgma	822.64	-0.3733	0.18065	-2.0662	0.03881	0.11186	1161.97	845.184	778.23	739.178	748.722	662.559	Down
1810026B	352.992	-0.4856	0.15917	-3.0511	0.00228	0.01113	384.098	421.634	429.697	265.399	331.592	285.534	Down
Slco3a1	7706.69	-0.4554	0.11088	-4.107	4.01E-05	0.00034	9203.84	8797.77	8737.18	6725.18	7058.47	5717.71	Down
Akap13	12158.8	0.36982	0.12512	2.95578	0.00312	0.01456	11749.3	9986.97	10090.2	12630.3	15472.9	13023	Up
AU020206	11774.6	0.32429	0.15163	2.13876	0.03246	0.09724	12100.5	10312.8	8956.8	13171.5	14734.7	11371.1	Up
Klhl25	1441.23	-0.3357	0.14606	-2.2984	0.02154	0.07065	1432.87	1528.42	1862.98	1327	1356.99	1139.12	Down
Ntrk3	168.106	-1.1461	0.32967	-3.4765	0.00051	0.00314	163.508	352.639	178.563	101.598	108.771	103.556	Down
Aen	1460.23	0.39094	0.18187	2.14952	0.03159	0.09538	1321.61	1076.12	1393.17	1349.8	1598.82	2021.86	Up
Abhd2	12055.3	0.58117	0.10944	5.31051	1.09E-07	1.61E-06	10229.4	8626.24	10122.7	13764.5	15414.8	14174.1	Up
Fanci	35.6236	1.03494	0.42171	2.45418	0.01412	0.05064	25.155	16.2904	28.6465	57.0194	45.4091	41.2214	Up
Ticrr	23.7139	1.10522	0.49596	2.22845	0.02585	0.08177	15.48	19.1652	10.5037	38.3585	30.6247	28.1512	Up
Plin1	532.01	-6.3107	1.18271	-5.3358	9.51E-08	1.41E-06	522.451	2200.16	429.697	17.6242	21.1205	1.0054	Down
Wdr93	751.725	-1.2042	0.2459	-4.897	9.73E-07	1.20E-05	861.076	1185.37	1099.07	464.449	305.191	595.197	Down
Anpep	5520.69	0.66186	0.12767	5.18417	2.17E-07	3.04E-06	4589.83	4314.08	3924.57	6074.12	6525.18	7696.34	Up
Zfp710	2109.22	0.51146	0.12188	4.19637	2.71E-05	0.00024	1821.81	1671.2	1724.52	2242.42	2799.52	2395.87	Up
Idh2	9132.92	-0.3266	0.13179	-2.4782	0.0132	0.04794	10414.2	11191.5	8881.37	8870.14	7222.16	8218.15	Down
Cib1	3924.52	0.90713	0.09676	9.37487	6.93E-21	6.47E-19	2795.11	2779.91	2614.47	5500.81	4820.76	5036.05	Up
Gdpgp1	392.296	0.53391	0.19066	2.80024	0.00511	0.0221	360.878	280.77	319.886	388.768	547.021	456.452	Up
Ttll13	175.252	-1.1724	0.23322	-5.0271	4.98E-07	6.55E-06	218.655	261.605	248.27	108.855	83.426	130.702	Down
Ngrn	1959.95	-0.2767	0.13629	-2.0304	0.04231	0.11976	2021.11	2360.19	2060.64	2034.04	1679.08	1604.62	Down
Man2a2	2367.14	1.22953	0.1488	8.2632	1.42E-16	8.82E-15	1675.71	1331.02	1239.44	3050.02	3640.12	3266.55	Up
Fes	2785.2	0.72774	0.13182	5.52053	3.38E-08	5.53E-07	1983.38	2090.92	2217.24	3363.11	3998.11	3058.43	Up
Furin	5716.21	0.75466	0.13913	5.42406	5.83E-08	9.09E-07	4337.31	4241.25	4184.3	6950.14	8492.56	6091.72	Up
Iqgap1	31119.6	0.90271	0.12882	7.0073	2.43E-12	8.21E-11	24664.5	19674	20729.6	38505.7	45093.3	38050.4	Up
Gm44899	148.886	-0.5367	0.25953	-2.0679	0.03865	0.1115	170.28	147.572	211.029	99.5247	115.107	149.805	Down
Alpk3	190.802	-2.3742	0.46144	-5.1452	2.67E-07	3.71E-06	220.59	571.122	168.059	42.5053	58.0814	84.4537	Down
Pde8a	2375.85	-0.2567	0.11863	-2.1635	0.0305	0.0929	2431.33	2457.93	2870.38	2144.97	2330.65	2019.85	Down
2900076A	519.046	-0.6863	0.21011	-3.2663	0.00109	0.00599	549.541	808.77	562.426	335.896	399.178	458.463	Down
Gm37829	71.5431	-3.0801	0.55835	-5.5164	3.46E-08	5.64E-07	86.1076	227.107	70.6613	22.8077	9.50423	13.0702	Down
Fsd2	449.688	-2.769	0.42989	-6.4411	1.19E-10	3.11E-09	435.376	1406.72	510.862	157.581	74.9778	112.605	Down
Homer2	238.575	-1.1172	0.224	-4.9874	6.12E-07	7.95E-06	292.185	348.806	338.983	153.434	114.051	183.988	Down
Btbd1	1203.83	0.25012	0.10797	2.31664	0.02052	0.06809	1080.7	1188.24	1030.32	1278.27	1305.25	1340.2	Up
Tm6sf1	764.483	0.67447	0.17744	3.80118	0.00014	0.00105	691.764	534.708	540.464	777.537	1058.14	984.287	Up
Hdgfl3	634.837	-0.3245	0.12981	-2.4996	0.01243	0.04567	762.391	659.282	696.11	606.479	543.853	540.906	Down
Adamtsl3	761.864	-0.3216	0.14645	-2.1959	0.0281	0.08744	808.831	930.469	800.192	631.36	625.167	775.164	Down
Stard5	1958.52	1.21117	0.14485	8.36159	6.19E-17	4.06E-15	1370.95	1179.62	994.033	2693.39	2926.25	2586.9	Up
Cfap161	1383.35	-1.2266	0.22795	-5.3809	7.41E-08	1.13E-06	1691.19	2187.7	1936.5	904.016	549.133	1031.54	Down
Mesd	3234.79	-0.2801	0.11508	-2.4339	0.01494	0.05288	3263.38	3651.92	3727.86	3084.23	3058.25	2623.09	Down
Abhd17c	6031.46	0.70413	0.11107	6.33948	2.31E-10	5.75E-09	4723.34	4599.64	4441.16	7531.74	8239.11	6653.74	Up
Arnt2	228.309	2.50989	0.46594	5.38674	7.17E-08	1.10E-06	59.9851	117.866	26.7367	234.298	502.668	428.301	Up
Gm2115	515.903	1.88394	0.24317	7.74755	9.37E-15	4.58E-13	246.713	206.984	206.255	547.386	858.549	1029.53	Up
Folh1	56.8953	-1.1338	0.50357	-2.2514	0.02436	0.07797	80.3026	114.991	39.1502	44.5788	42.241	20.108	Down
Nox4	4617.11	-0.6004	0.13739	-4.37	1.24E-05	0.00012	6475.49	5434.28	4783.01	3421.16	3836.54	3752.16	Down
Grm5	21.4058	-2.0666	0.595	-3.4733	0.00051	0.00317	35.7976	28.7478	39.1502	13.4773	4.2241	7.03781	Down
Ctsc	24205.5	1.49844	0.20686	7.24379	4.36E-13	1.67E-11	17030.9	9220.36	11714.5	32852.5	37121.4	37293.3	Up
Gm44751	755.374	1.35038	0.26263	5.14171	2.72E-07	3.77E-06	373.456	534.708	368.585	1527.08	841.652	886.764	Up
Rab38	621.738	0.4628	0.18896	2.44922	0.01432	0.05115	667.576	438.882	462.163	720.518	745.554	695.737	Up
Tmem135	3150.67	0.59182	0.10944	5.40782	6.38E-08	9.85E-07	2352.96	2735.83	2451.18	3527.94	4026.62	3809.46	Up
Fzd4	7294.17	-0.4003	0.08635	-4.6355	3.56E-06	3.90E-05	7775.81	8366.56	8756.28	6325	6270.68	6270.69	Down
Prss23	2930.43	-0.8969	0.21827	-4.1094	3.97E-05	0.00033	3810.02	4231.67	3397.47	2558.61	2213.43	1371.37	Down
Me3	397.795	-1.9205	0.38275	-5.0177	5.23E-07	6.86E-06	470.206	1019.59	398.186	238.445	145.732	114.616	Down
Ccdc81	911.046	-1.1731	0.20135	-5.8264	5.66E-09	1.08E-07	1177.45	1372.23	1237.53	448.898	494.22	735.953	Down
Picalm	16301.5	0.2266	0.08995	2.51913	0.01176	0.04366	14083	15438.5	15550.3	16663.1	18213.3	17860.9	Up
Sytl2	1349.89	-1.0175	0.17237	-5.9031	3.57E-09	7.13E-08	1709.58	2013.3	1698.74	974.513	693.809	1009.42	Down
Ccdc89	170.208	-0.5111	0.22016	-2.3214	0.02027	0.06742	210.915	209.859	179.518	131.663	120.387	168.907	Down
Dlg2	90.0945	-1.1862	0.29796	-3.981	6.86E-05	0.00055	130.613	144.697	100.263	66.3498	44.3531	54.2916	Down
Ccdc90b	1093.81	-0.2272	0.11052	-2.0556	0.03982	0.11408	1088.44	1264.9	1185.96	1005.61	988.44	1029.53	Down
Ddias	79.4386	0.83882	0.3089	2.71553	0.00662	0.02721	65.7901	45.0382	60.1576	105.745	80.2579	119.643	Up

Prcp	2956.7	0.57124	0.12065	4.73467	2.19E-06	2.51E-05	2596.77	2224.12	2315.59	3769.5	3680.25	3153.94	Up
Gm15501	171.372	-0.7613	0.22586	-3.3709	0.00075	0.00437	249.615	212.733	184.292	143.067	130.947	107.578	Down
Gab2	2629.19	0.40344	0.13705	2.94383	0.00324	0.01503	2066.58	2524.05	2201.01	2802.24	3419.41	2761.84	Up
Usp35	630.478	0.5908	0.15768	3.74673	0.00018	0.00127	587.273	479.129	443.066	722.591	827.924	722.883	Up
Alg8	792.499	0.41961	0.11188	3.75068	0.00018	0.00125	630.811	711.986	691.335	910.237	899.734	910.893	Up
Thrsp	1197.59	-1.8125	0.63405	-2.8586	0.00426	0.01892	1154.23	3439.19	999.762	752.656	381.225	458.463	Down
Kctd14	446.037	0.61821	0.16598	3.72456	0.0002	0.00137	405.383	316.225	334.209	483.11	549.133	588.16	Up
Aamdc	943.249	-0.364	0.13424	-2.7111	0.00671	0.02752	1212.28	1020.55	952.018	801.381	844.82	828.45	Down
Pak1	2288.44	1.09572	0.10217	10.7243	7.83E-27	1.26E-24	1468.67	1430.68	1477.2	3156.8	3334.93	2862.38	Up
Myo7a	1461.46	0.97801	0.2435	4.01641	5.91E-05	0.00048	1311.93	890.222	750.538	1546.78	2481.66	1787.6	Up
Capn5	1844.41	0.33126	0.1669	1.98475	0.04717	0.13041	1398.04	1622.33	1880.16	1995.68	2408.79	1761.46	Up
B3gnt6	55.0679	-0.9145	0.34872	-2.6224	0.00873	0.03415	60.9526	67.0781	87.8492	46.6522	31.6808	36.1944	Down
Tsku	1717.39	-1.3376	0.30534	-4.3806	1.18E-05	0.00011	3921.28	1735.41	1726.43	995.247	767.73	1158.22	Down
Gm44507	8.4658	-2.1478	0.98967	-2.1702	0.02999	0.09177	25.155	8.62433	7.63906	4.14686	4.2241	1.0054	Down
Gm15506	174.458	-0.6372	0.24609	-2.5892	0.00962	0.03698	161.573	236.69	238.721	136.846	156.292	116.627	Down
Wnt11	1677.42	-0.8146	0.18637	-4.3706	1.24E-05	0.00012	2225.25	2259.57	1931.73	1392.31	892.341	1363.32	Down
Uvrag	3154.88	0.30406	0.10517	2.89127	0.00384	0.01735	3113.42	2623.71	2733.83	3425.31	3591.54	3441.49	Up
Dgat2	1635.01	-1.4977	0.52283	-2.8646	0.00418	0.01864	2223.32	3928.86	1092.39	662.461	1023.29	879.726	Down
Map6	2392.72	0.49419	0.16378	3.01743	0.00255	0.01225	1914.69	1866.69	2179.04	2602.16	3432.08	2361.69	Up
Gdpd5	1337.84	0.35466	0.15676	2.26243	0.02367	0.07625	959.761	1308.02	1254.72	1516.72	1631.56	1356.29	Up
Gm10605	81.5491	-0.7954	0.29478	-2.6983	0.00697	0.02842	127.71	85.285	97.3981	62.203	64.4175	52.2808	Down
Xrra1	181.618	-0.8321	0.21321	-3.9029	9.50E-05	0.00073	204.143	270.229	223.443	143.067	115.107	133.718	Down
Chrdl2	34.2556	-1.4945	0.4568	-3.2716	0.00107	0.0059	40.6351	67.0781	43.9246	16.5875	23.2326	14.0756	Down
Pold3	2188.03	0.23718	0.0942	2.51779	0.01181	0.04379	2000.79	1953.89	2071.14	2241.38	2366.55	2494.4	Up
Pgm2l1	2150.32	0.44849	0.19393	2.3126	0.02074	0.0686	1833.42	1965.39	1657.68	2496.41	1834.32	3114.73	Up
P4ha3	122.991	1.23615	0.26151	4.72697	2.28E-06	2.59E-05	74.4976	66.1198	79.2553	212.527	165.796	139.751	Up
Ucp3	401.601	1.52233	0.33771	4.50777	6.55E-06	6.75E-05	276.705	226.149	119.36	554.643	820.532	412.214	Up
Ucp2	45321	0.38468	0.16444	2.3394	0.01931	0.06498	42721	39788.8	35432.8	56716.7	57652.6	39613.8	Up
Dnajb13	1622.88	-0.3365	0.14578	-2.3083	0.02098	0.06925	1756.98	1861.9	1815.23	1579.95	1165.85	1557.37	Down
Arhgef17	4453.31	-0.2717	0.11288	-2.4069	0.01609	0.05616	5231.28	4353.37	5029.37	4146.86	4198.76	3760.2	Down
P2ry6	941.371	0.73513	0.14167	5.18915	2.11E-07	2.97E-06	745.944	700.487	673.192	1138.31	1351.71	1038.58	Up
P2ry2	1385.27	1.39593	0.15278	9.13684	6.43E-20	5.53E-18	911.386	658.324	719.027	1935.55	2168.02	1919.31	Up
Fchsd2	481.252	0.75989	0.19418	3.91332	9.10E-05	0.0007	412.156	320.058	339.938	478.963	654.736	681.662	Up
Folr2	133.022	-0.6587	0.28253	-2.3315	0.01973	0.0661	145.125	201.234	142.278	131.663	81.314	96.5185	Down
Folr1	21.267	1.9639	0.82078	2.39273	0.01672	0.05798	19.35	4.79129	1.90977	49.7624	31.6808	20.108	Up
Lamtor1	2802.53	0.37147	0.13165	2.82155	0.00478	0.02088	2469.06	2594.01	2267.85	3263.58	3508.12	2712.57	Up
Lrrc51	64.3341	-0.8721	0.42337	-2.06	0.0394	0.11318	94.8151	66.1198	88.8041	29.028	34.8488	72.3889	Down
Il18bp	759.863	1.05724	0.1151	9.18513	4.11E-20	3.65E-18	506.971	491.587	481.261	1094.77	984.216	1000.37	Up
Art5	35.9041	-3.4115	0.69252	-4.9262	8.38E-07	1.05E-05	64.8226	103.492	28.6465	2.07343	6.33615	10.054	Down
Art1	78.7384	-6.5289	1.69274	-3.857	0.00011	0.00086	93.8476	326.766	46.7893	0	0	5.027	Down
Chrna10	9.7202	-2.2145	0.84125	-2.6323	0.00848	0.03335	10.6425	24.9147	12.4135	3.11015	4.2241	3.0162	Down
Pgap2	2235.88	0.27382	0.11658	2.34869	0.01884	0.06375	2258.15	1936.64	1878.25	2307.73	2568.25	2466.25	Up
Stim1	4514.51	-0.2584	0.08232	-3.139	0.0017	0.00867	4845.25	5155.43	4752.45	4176.93	4073.09	4083.94	Down
Rrm1	2018.9	0.27801	0.13984	1.98814	0.0468	0.12953	2143.02	1738.28	1593.7	2337.79	2150.07	2150.55	Up
Trim68	1746.8	-0.2364	0.11725	-2.0162	0.04377	0.12302	1897.27	1879.15	1892.58	1728.21	1405.57	1678.01	Down
Olfr78	19.2053	-1.5107	0.68058	-2.2198	0.02644	0.08327	14.5125	33.5391	37.2404	4.14686	13.7283	12.0648	Down
Trim6	253.601	1.48553	0.20078	7.39874	1.37E-13	5.74E-12	122.873	153.321	124.135	408.466	398.122	314.69	Up
Trim30a	2164.83	0.4711	0.12794	3.68236	0.00023	0.00157	2067.55	1778.53	1597.52	2434.21	2468.99	2642.19	Up
Smpd1	6137.55	0.24019	0.08666	2.77156	0.00558	0.02376	5852.42	5784.05	5247.08	6636.02	6632.9	6672.85	Up
Trim3	7423.75	-0.5312	0.18672	-2.8448	0.00444	0.01962	6291.66	9585.46	10448.3	6163.28	6397.4	5656.39	Down
Tpp1	11111.5	0.50639	0.09659	5.24274	1.58E-07	2.27E-06	9771.76	8781.48	8990.22	12607.5	14001.8	12516.2	Up
Syt9	78.4959	-1.2003	0.34656	-3.4634	0.00053	0.00327	97.7176	125.532	105.037	35.2483	39.0729	68.3673	Down
Olfml1	754.716	-0.5188	0.11624	-4.4636	8.06E-06	8.10E-05	835.921	946.76	884.222	643.801	609.327	608.268	Down
Cyb5r2	39.062	-1.2515	0.54939	-2.278	0.02272	0.0738	34.8301	92.9511	37.2404	15.5507	31.6808	22.1188	Down
Gm10156	224.426	0.58896	0.21282	2.76744	0.00565	0.02401	174.15	213.692	149.917	303.758	237.606	267.437	Up
Olfr510	6.97766	4.38165	1.36004	3.22171	0.00127	0.00685	0	0.95826	0.95488	14.514	6.33615	19.1026	Up
Stk33	2146.8	-0.8803	0.25875	-3.4022	0.00067	0.00397	2407.14	3064.51	2875.15	1673.26	888.117	1972.6	Down
1700095J(	146.808	-0.9375	0.38988	-2.4046	0.01619	0.05646	178.988	201.234	198.616	108.855	44.3531	148.799	Down
Trim66	49.3392	-1.5229	0.40976	-3.7167	0.0002	0.00141	46.4401	91.0346	82.1199	29.028	24.2886	23.1242	Down
Rpl27a	18884.1	-0.2596	0.13036	-1.9914	0.04644	0.12877	20492.6	18066.1	23176.9	18212	17973.6	15383.6	Down
Tmem9b	5369.64	0.23725	0.097	2.4458	0.01445	0.05152	4708.83	4982.94	5095.26	5819.09	6214.71	5396.99	Up
Scube2	1229.36	-1.0292	0.12723	-8.0892	6.01E-16	3.48E-14	1611.86	1505.42	1833.38	823.152	749.778	852.58	Down
Tmem41b	3279.41	0.57615	0.09874	5.83505	5.38E-09	1.03E-07	2471.97	2652.46	2774.89	3724.92	4152.29	3899.95	Up
Swap70	4430.55	-0.3883	0.09217	-4.2132	2.52E-05	0.00022	5300.94	4755.84	5013.14	3653.39	3916.8	3943.18	Down
Gm28863	29.7809	2.38047	0.64698	3.67933	0.00023	0.00159	10.6425	2.87478	15.2781	59.0928	27.4567	63.3403	Up
Lyve1	9666.32	-1.4406	0.15625	-9.2204	2.96E-20	2.65E-18	14532.8	11819.2	16031.5	6039.91	4929.53	4644.95	Down
Mrvi1	443.301	-0.9418	0.28978	-3.2502	0.00115	0.00629	408.286	864.349	476.487	295.464	354.825	260.399	Down
1700012D	151.302	-0.6581	0.29084	-2.2629	0.02364	0.07618	174.15	143.739	237.766	87.0841	139.395	125.675	Down
Galnt18	7453.14	-0.5077	0.11685	-4.345	1.39E-05	0.00013	7695.51	9147.54	9410.37	6431.79	6312.92	5720.73	Down
Dkk3	5176.21	-0.719	0.22168	-3.2432	0.00118	0.00641	4709.8	9010.51	5599.43	3959.22	4100.55	3677.76	Down
2310014F(	392.428	-0.7294	0.14794	-4.9303	8.21E-07	1.04E-05	469.238	491.587	507.998	316.198	261.894	307.653	Down
Tead1	7852.33	-0.2622	0.08482	-3.0911	0.00199	0.00995	8186.03	8566.83	8938.66	6969.84	7444.98	7007.64	Down
Rassf10	355.576	-0.8063	0.33258	-2.4244	0.01533	0.05401	417.961	503.086	436.381	331.749	123.555	320.723	Down
Arntl	1019.52	-1.2253	0.62376	-1.9643	0.04949	0.13574	431.506	1916.52	1936.5	603.369	620.943	608.268	Down
Spon1	11603.8	-0.394	0.08413	-4.6832	2.82E-06	3.16E-05	13401.8	13144.4	12989.3	10459.4	10227.6	9400.5	Down
Rras2	4655.05	-0.6392	0.14748	-4.3346	1.46E-05	0.00014	4672.06	5970.91	6366.2	3579.78	4003.39	3337.93	Down
Copb1	8746.18	0.21711	0.07524	2.88543	0.00391	0.01762	8012.85	7884.55	8370.5	9553.34	9303.58	9352.24	Up
Cyp2r1	130.208	0.66836	0.24817	2.69313	0.00708	0.02881	126.743	88.1598	86.8943	160.691	147.844	170.918	Up
Calca	154.174	-2.373	0.23848	-9.9508	2.50E-23	2.85E-21	299.925	250.106	225.352	52.8725	47.5211	49.2646	Down
Sox6	583.796	-0.3589	0.12948	-2.7715	0.00558	0.02376	630.811	706.237	631.178	492.44	493.164	548.949	Down
Plekha7	1383.57	0.31039	0.12535	2.47612	0.01328	0.04817	1245.17	1374.14	1086.66	1579.95	1474.21	1541.28	Up
Pik3c2a	1625.67	0.80903	0.13798	5.86351	4.53E-09	8.90E-08	1255.82	1004.26	1284.32	1930.36	2040.24	2239.03	Up
Nucb2	3423.59	0.96997	0.11353	8.54356	1.30E-17	9.42E-16	2603.55	2187.7	2151.35	4724.31	4465.93	4408.68	Up

Gm45151	70.3455	0.58189	0.27547	2.11236	0.03466	0.10247	61.9201	49.8294	57.293	82.9373	87.6501	82.4429	Up
Xylt1	248.528	0.38632	0.17212	2.24454	0.0248	0.07914	217.688	216.566	211.984	275.766	316.808	252.356	Up
Arl6ip1	12452.9	0.59282	0.15242	3.88934	0.0001	0.00076	10012.7	8646.37	11130.1	16037	16504.6	12386.5	Up
Syt17	492.122	-0.7043	0.27646	-2.5475	0.01085	0.04092	667.576	630.534	531.87	314.125	257.67	550.96	Down
Gm44652	32.9052	-1.1229	0.49996	-2.2459	0.02471	0.07891	32.895	53.6625	48.699	31.1015	19.0085	12.0648	Down
Tmc5	1807.57	1.64872	0.53968	3.05498	0.00225	0.011	836.889	1352.1	433.517	2957.75	1612.55	3652.62	Up
Gde1	15810	0.81176	0.11303	7.18201	6.87E-13	2.53E-11	12535.9	11448.3	10443.6	19388.7	19089.8	21953.9	Up
Vps35l	3879.99	0.30622	0.09367	3.26921	0.00108	0.00594	3707.47	3358.7	3343.04	4403.97	4368.78	4098.01	Up
Iqck	913.389	-0.4466	0.18933	-2.3589	0.01833	0.06238	1060.38	1088.58	1012.18	893.649	563.918	861.629	Down
Gp2	7089.32	4.48075	1.24712	3.59287	0.00033	0.00214	222.525	1502.55	98.3529	10751.8	4692.98	25267.7	Up
Umod	18.2594	3.54431	0.89839	3.94519	7.97E-05	0.00062	4.83751	2.87478	0.95488	19.6976	15.8404	65.3511	Up
Acsm5	57.9339	-1.5576	0.5516	-2.8239	0.00474	0.02077	101.588	114.991	42.9697	34.2116	11.6163	42.2268	Down
Acsm1	602.084	-0.5073	0.25581	-1.983	0.04736	0.13083	648.226	843.268	629.268	551.533	317.864	622.343	Down
Thumpd1	1845.29	-0.2217	0.08687	-2.5527	0.01069	0.04042	1960.16	1957.72	2042.49	1696.07	1740.33	1675	Down
Ppp1ccb	634.677	0.36422	0.15369	2.36985	0.0178	0.06088	642.421	541.416	481.261	713.261	671.632	758.072	Up
Acsm3	9.46833	1.69152	0.8435	2.00536	0.04492	0.12549	1.935	8.62433	2.86465	16.5875	13.7283	13.0702	Up
Anks4b	10.4362	4.37554	1.15755	3.78001	0.00016	0.00113	0	2.87478	0	23.8445	14.7844	21.1134	Up
Abca15	10.5739	-5.8134	1.36395	-4.2622	2.02E-05	0.00018	20.3175	21.0817	21.0074	1.03672	0	0	Down
Mosmo	2581.68	-0.407	0.08119	-5.0133	5.35E-07	7.00E-06	2922.82	2916.94	2990.69	2268.33	2175.41	2215.9	Down
Eef2k	1668.33	-0.3362	0.1353	-2.485	0.01296	0.04722	2129.47	1790.99	1665.32	1475.25	1365.44	1583.51	Down
Cdr2	1670.49	-0.7918	0.16234	-4.8777	1.07E-06	1.32E-05	2040.46	1834.11	2478.88	1111.36	1140.51	1417.62	Down
Usp31	1702.56	-0.2737	0.1079	-2.5364	0.0112	0.04193	1789.88	1770.86	2030.08	1539.52	1477.38	1607.64	Down
Ndufab1	2101.75	-0.4513	0.14465	-3.1203	0.00181	0.00915	2066.58	2842.2	2374.79	1882.68	1672.74	1771.52	Down
Tnrc6a	3930.77	-0.2415	0.10051	-2.4027	0.01627	0.0567	4078.99	4112.85	4585.35	3570.45	3420.47	3816.5	Down
Arhgap17	3344.98	0.41108	0.10785	3.81178	0.00014	0.00101	2962.49	2769.37	2882.79	3653.39	4234.66	3567.16	Up
3100003L(	6.25574	2.46803	1.14565	2.15427	0.03122	0.09448	0	3.83303	1.90977	7.25701	8.4482	16.0864	Up
Gm30717	6.93857	3.29232	1.41426	2.32794	0.01992	0.0666	3.87001	0	0	5.18358	8.4482	24.1296	Up
Il4ra	6666	0.32212	0.13621	2.36481	0.01804	0.06159	5121.95	6058.11	6594.42	6868.24	8301.42	7051.88	Up
Gtf3c1	4766.23	0.50881	0.09367	5.43187	5.58E-08	8.78E-07	4079.95	3752.54	3970.4	5756.88	5815.53	5222.05	Up
Gsg1l	110.742	-1.593	0.36576	-4.3552	1.33E-05	0.00013	234.135	102.534	162.33	44.5788	67.5856	53.2862	Down
Sbk1	786.356	-0.3641	0.16397	-2.2203	0.0264	0.08317	856.239	1056.96	741.944	731.921	669.52	661.554	Down
Lat	525.707	-0.7498	0.14631	-5.1246	2.98E-07	4.11E-06	581.468	679.405	717.117	414.686	366.441	395.123	Down
Tufm	2559.91	-0.2051	0.10192	-2.012	0.04422	0.1239	2620.96	2875.73	2728.1	2490.19	2192.31	2452.17	Down
Eif3c	11412.7	0.24751	0.07826	3.16281	0.00156	0.0081	10751.8	9975.47	10581.1	12550.5	12102.1	12515.2	Up
Apobr	752.153	1.19293	0.20362	5.85857	4.67E-09	9.15E-08	547.606	452.298	373.359	1010.8	1267.23	861.629	Up
Il27	49.639	0.82385	0.34549	2.38461	0.0171	0.05899	29.9925	38.3303	39.1502	57.0194	76.0338	57.3078	Up
Nupr1	9311.25	0.5501	0.15483	3.55303	0.00038	0.00245	6352.61	8706.74	7612.33	12766.1	10087.2	10342.6	Up
2510046G	53.1342	-0.7469	0.33045	-2.2601	0.02381	0.07663	63.8551	67.0781	68.7516	40.4319	47.5211	31.1674	Down
Gm6939	81.5636	-1.3738	0.29588	-4.6431	3.43E-06	3.77E-05	102.555	106.367	144.187	50.7991	42.241	43.2322	Down
Ypel3	5117.85	-0.4344	0.13023	-3.336	0.00085	0.00485	6240.38	5759.13	5648.13	4737.79	4654.96	3666.7	Down
Tbx6	230.952	-0.5853	0.17028	-3.4373	0.00059	0.00355	285.413	250.106	296.014	184.535	181.636	188.01	Down
Fam57b	407.787	-0.5737	0.28584	-2.0069	0.04476	0.12512	635.648	478.171	349.487	335.896	420.298	227.221	Down
Hirip3	911.94	-0.3077	0.14267	-2.1568	0.03102	0.09408	1125.2	856.683	1044.64	854.254	782.515	808.342	Down
Sez6l2	97.7459	-0.7382	0.24589	-3.0022	0.00268	0.01277	116.1	111.158	139.413	71.5334	72.8658	75.4051	Down
Cdiptos	627.892	-0.5207	0.24288	-2.1437	0.03206	0.09642	477.946	890.222	851.756	571.23	460.427	515.771	Down
Mvp	7317.85	0.48581	0.09092	5.34318	9.13E-08	1.36E-06	6054.62	5995.82	6241.11	8418.13	9165.24	8032.15	Up
Tbc1d10b	2872.47	0.21741	0.10093	2.15408	0.03123	0.09449	2657.73	2527.89	2783.48	3145.4	3259.95	2860.37	Up
Septin1	732.882	-0.2707	0.13665	-1.9809	0.04761	0.1314	899.776	750.317	754.357	623.066	658.96	710.818	Down
Dctpp1	269.874	0.38097	0.18785	2.02807	0.04255	0.12028	271.868	222.316	209.119	288.207	343.208	284.528	Up
Sephs2	1477.65	0.53305	0.14569	3.65887	0.00025	0.0017	1380.62	1221.78	1020.77	1819.44	1822.7	1600.6	Up
Itgal	3156.75	0.4022	0.14052	2.86214	0.00421	0.01875	2862.84	2985.93	2309.86	3270.84	3978.05	3532.98	Up
Zfp764	646.58	-0.3389	0.12858	-2.6358	0.00839	0.03306	768.196	646.825	751.493	581.598	572.366	559.003	Down
Gm45184	25.3209	-1.797	0.58216	-3.0868	0.00202	0.01007	31.9275	41.2051	44.8795	17.6242	4.2241	12.0648	Down
Bcl7c	1080.73	-0.2937	0.11083	-2.6502	0.00804	0.03196	1148.42	1127.87	1294.82	965.182	956.759	991.325	Down
Fbxl19	1705.08	0.50604	0.09958	5.08194	3.74E-07	5.05E-06	1391.27	1325.27	1510.62	2035.07	1990.61	1977.62	Up
Setd1a	157.697	0.65889	0.24131	2.73044	0.00632	0.02629	143.19	93.9093	129.864	176.242	184.804	218.172	Up
Vkorc1	212.629	-0.4655	0.21964	-2.1193	0.03406	0.10107	282.51	249.147	208.164	200.086	190.085	145.783	Down
Kat8	1478.31	-0.295	0.12059	-2.446	0.01445	0.05151	1633.14	1450.8	1802.82	1347.73	1284.13	1351.26	Down
Prss8	5772.84	0.35186	0.1438	2.44687	0.01441	0.0514	5455.74	4683.01	5078.07	7189.62	6919.08	5311.53	Up
Prss36	287.844	-0.6307	0.1649	-3.8246	0.00013	0.00096	320.243	387.136	341.848	230.151	236.55	211.134	Down
Pycard	306.972	0.40232	0.16334	2.46312	0.01377	0.04966	282.51	275.02	235.856	319.308	361.161	367.977	Up
Trim72	159.562	-3.5406	0.60219	-5.8796	4.11E-09	8.14E-08	158.67	596.995	126.045	21.771	12.6723	41.2214	Down
Itgax	6091.45	2.74899	0.28746	9.56299	1.14E-21	1.16E-19	2478.74	1250.53	1003.58	9928.63	12240.4	9646.82	Up
Itgad	120.188	2.24251	0.24933	8.99418	2.38E-19	1.96E-17	44.5051	44.0799	37.2404	174.168	206.981	214.15	Up
Tgfb1i1	3757.85	-0.4038	0.17535	-2.3029	0.02128	0.07003	3435.6	3999.77	5405.59	3226.26	3461.65	3018.21	Down
Rgs10	929.775	0.9059	0.15442	5.86644	4.45E-09	8.76E-08	719.821	582.621	638.817	1045.01	1372.83	1219.55	Up
Gm40457	26.593	-5.2363	1.11299	-4.7047	2.54E-06	2.87E-05	27.09	113.075	15.2781	3.11015	0	1.0054	Down
Sec23ip	3467.77	0.20055	0.07874	2.54712	0.01086	0.04094	3300.15	3216.87	3164.48	3664.79	3674.97	3785.33	Up
Fgfr2	14711.1	0.7695	0.09937	7.74383	9.65E-15	4.71E-13	11666.1	10428.7	10540	18143.6	17543.7	19944.1	Up
Dhx32	2462.37	0.42363	0.09953	4.25636	2.08E-05	0.00019	2059.81	2225.08	2025.31	2663.32	2989.61	2811.1	Up
Fank1	1334.4	-0.7816	0.25648	-3.0476	0.00231	0.01123	1395.14	1954.85	1712.11	1088.55	604.047	1251.72	Down
Dock1	3909.07	0.61368	0.1316	4.66323	3.11E-06	3.45E-05	3510.1	2731.04	3028.89	4284.75	5001.34	4898.31	Up
Ptpre	2430.06	0.3183	0.16102	1.97673	0.04807	0.13238	2095.61	2155.12	2238.25	2434.21	3350.77	2306.39	Up
Mki67	570.511	0.92671	0.21156	4.38032	1.19E-05	0.00011	366.683	492.545	320.841	800.345	606.159	836.494	Up
Gm6249	2.56193	-4.7537	1.99293	-2.3853	0.01707	0.05892	4.83751	8.62433	1.90977	0	0	0	Down
9430038I(	445.171	0.3902	0.1693	2.30481	0.02118	0.06973	361.846	401.51	392.457	493.477	592.43	429.306	Up
Tcerg1l	41.2633	-1.4253	0.41045	-3.4726	0.00052	0.00318	44.5051	78.5772	57.293	22.8077	24.2886	20.108	Down
Mapk1ip1	2579.23	-0.3338	0.11375	-2.9342	0.00334	0.01544	2657.73	3087.51	2883.75	2241.38	2123.67	2481.33	Down
Bnip3	612.585	-0.3929	0.13539	-2.9018	0.00371	0.01686	705.309	697.612	683.696	574.341	463.595	550.96	Down
Dpysl4	24.073	-2.5842	0.75605	-3.418	0.00063	0.00378	25.155	81.452	17.1879	9.33044	5.28013	6.03241	Down
Lrrc27	493.8	-0.3526	0.15239	-2.3135	0.02069	0.06847	483.751	630.534	547.148	438.531	444.587	418.247	Down
Inpp5a	6117.77	-0.649	0.12731	-5.0975	3.44E-07	4.68E-06	6861.52	7536.7	8014.33	4823.84	5312.86	4157.33	Down
Nkx6-2	46.3147	-2.9404	0.41868	-7.023	2.17E-12	7.38E-11	80.3026	94.8676	70.6613	9.33044	12.6723	10.054	Down

Cfap46	2253.35	-0.5964	0.22797	-2.6162	0.00889	0.03465	2435.2	3044.39	2658.39	2100.39	1155.29	2126.42	Down
Adam8	7713.36	1.74184	0.58786	2.96303	0.00305	0.01428	2423.59	6105.07	2123.66	14467.4	7378.45	13782	Up
Zfp511	1295.01	-0.4747	0.10411	-4.5599	5.12E-06	5.40E-05	1496.72	1511.17	1510.62	1170.45	1025.4	1055.67	Down
Msx3	11.3352	1.77698	0.78711	2.25759	0.02397	0.07706	3.87001	9.58259	1.90977	18.6609	16.8964	17.0918	Up
Echs1	5515.75	-0.3307	0.15958	-2.0722	0.03824	0.11062	5284.49	7676.61	5474.34	5005.26	4717.27	4936.52	Down
Paox	787.375	0.37734	0.10578	3.56729	0.00036	0.00233	701.439	683.238	670.328	917.494	866.997	884.753	Up
Mtg1	777.923	-0.2668	0.13316	-2.0039	0.04508	0.12578	791.416	926.636	830.748	673.865	778.291	666.581	Down
Sirt3	1298.92	-0.4172	0.14466	-2.8842	0.00392	0.01769	1395.14	1620.42	1440.92	1240.95	939.863	1156.21	Down
Cox8b	98.3802	-4.1752	0.57997	-7.1989	6.07E-13	2.26E-11	106.425	351.681	101.218	6.2203	12.6723	12.0648	Down
Pkp3	1430	0.32347	0.09713	3.3302	0.00087	0.00494	1288.71	1218.91	1303.42	1662.89	1559.75	1546.31	Up
Ano9	298.834	1.12273	0.22608	4.96596	6.84E-07	8.79E-06	242.843	175.361	146.097	449.935	410.794	367.977	Up
Rnh1	12267.4	0.50697	0.10566	4.79819	1.60E-06	1.89E-05	10597	10062.7	9741.72	15000.2	15332.4	12870.1	Up
Lrrc56	1254.1	-0.7123	0.18496	-3.8513	0.00012	0.00087	1493.82	1715.28	1463.84	1051.23	707.537	1092.87	Down
Irf7	2411.48	1.33678	0.16017	8.34582	7.07E-17	4.59E-15	1496.72	1426.85	1180.24	3574.6	2852.32	3938.16	Up
Cdhr5	321.97	3.82175	0.28702	13.3152	1.89E-40	9.71E-38	26.1225	62.2868	39.1502	602.332	513.228	688.7	Up
Eps8l2	1929.23	0.33607	0.12247	2.74405	0.00607	0.02539	1738.6	1522.67	1855.34	2333.65	2055.03	2070.12	Up
Taldo1	11399.3	0.45541	0.14612	3.11665	0.00183	0.00925	10676.4	9642.96	8525.19	13967.7	14578.4	11005.1	Up
Chid1	1674.1	0.39809	0.10563	3.76862	0.00016	0.00118	1563.48	1392.35	1377.9	1864.02	1996.94	1849.94	Up
Muc5ac	613.709	1.28474	0.31398	4.09181	4.28E-05	0.00036	316.373	522.251	232.991	797.234	643.119	1170.29	Up
Tollip	6466.28	0.2088	0.09103	2.29384	0.0218	0.07135	6304.24	5853.04	5840.06	6690.96	7373.17	6736.19	Up
Brsk2	22.4501	-1.6232	0.61003	-2.6609	0.00779	0.03113	29.025	27.7895	44.8795	4.14686	14.7844	14.0756	Down
Mob2	2914.08	-0.3606	0.12995	-2.7745	0.00553	0.02358	2790.27	3317.49	3721.18	2530.62	2567.2	2557.74	Down
Dusp8	1883.23	-0.4704	0.15533	-3.0283	0.00246	0.01188	2116.89	1886.81	2559.09	1500.13	1448.87	1787.6	Down
Gm32786	40.8409	1.60821	0.51542	3.12019	0.00181	0.00915	22.2525	25.873	12.4135	93.3044	35.9049	55.297	Up
Gm39115	5.28124	3.95638	1.70257	2.32377	0.02014	0.06711	0	1.91652	0	18.6609	1.05603	10.054	Up
Gm7579	4.3153	5.61018	1.65059	3.39889	0.00068	0.00401	0	0	0	8.29373	10.5603	7.03781	Up
Krtap5-5	158.605	4.53375	0.40602	11.1664	5.96E-29	1.10E-26	12.5775	22.9982	3.81953	273.693	337.928	300.615	Up
Gm40460	24.6513	2.82851	0.67147	4.2124	2.53E-05	0.00022	4.83751	11.4991	1.90977	41.4686	57.0254	31.1674	Up
Ifitm10	899.586	-0.7782	0.15615	-4.9837	6.24E-07	8.10E-06	933.639	1268.73	1206.97	675.939	695.921	616.311	Down
Ctsd	6960.05	1.81175	0.24681	7.3406	2.13E-13	8.54E-12	4165.09	2887.23	2205.78	9038.09	14052.5	9411.56	Up
Syt8	83.8491	1.60216	0.3942	4.06436	4.82E-05	0.0004	21.285	62.2868	41.06	137.883	110.883	129.697	Up
Lsp1	3600.7	0.42864	0.15727	2.72547	0.00642	0.02661	3407.54	3123.92	2677.49	3814.08	4929.53	3651.62	Up
Tnnt3	107.509	-1.6139	0.44911	-3.5936	0.00033	0.00213	84.1726	281.728	120.315	53.9092	49.6332	55.297	Down
Mrpl23	1313.12	-0.2986	0.11431	-2.612	0.009	0.03499	1342.89	1486.26	1516.35	1274.12	1132.06	1127.05	Down
Ins2	14.1114	-2.1509	0.73762	-2.916	0.00355	0.01624	19.35	17.2487	32.466	7.25701	6.33615	2.0108	Down
Tspan32	451.272	0.42834	0.20638	2.07549	0.03794	0.10998	474.076	362.222	317.976	461.339	611.439	480.582	Up
R74862	162.563	-0.5335	0.24112	-2.2127	0.02692	0.08447	183.825	154.28	238.721	120.259	142.563	135.729	Down
Kcnq1	2322.5	0.96037	0.11793	8.14377	3.83E-16	2.28E-14	1677.65	1665.45	1387.44	2898.66	3123.72	3182.09	Up
Cdkn1c	1610.26	-0.3652	0.1837	-1.9881	0.0468	0.12953	1617.66	1846.56	1974.7	1792.48	1239.77	1190.39	Down
Slc22a18	1356.03	0.79953	0.13118	6.095	1.09E-09	2.41E-08	938.476	1135.54	894.725	1742.72	1788.91	1635.79	Up
Tnfrsf26	413.117	2.02417	0.21582	9.37893	6.66E-21	6.25E-19	201.24	136.073	151.826	587.818	801.523	600.224	Up
Tnfrsf23	41.2399	1.42051	0.46126	3.07962	0.00207	0.01026	31.9275	22.9982	12.4135	48.7256	55.9693	75.4051	Up
Mrgpre	269.748	0.5685	0.26287	2.16264	0.03057	0.09303	275.738	228.066	148.007	318.272	375.945	272.464	Up
Dhcr7	1737.25	1.69648	0.19728	8.59948	8.01E-18	5.96E-16	904.614	949.634	603.486	2492.26	3105.77	2367.72	Up
Shank2	1205.02	-0.4532	0.22625	-2.0029	0.04519	0.12602	1290.65	1415.35	1472.43	1073	672.688	1306.02	Down
Cttn	8919	0.29214	0.07608	3.84009	0.00012	0.00091	8030.26	7834.72	8191.94	9849.84	10086.1	9521.15	Up
Ano1	6415.35	0.5449	0.17018	3.20197	0.00136	0.00725	6617.71	4426.2	4610.17	7893.55	7931.81	7012.67	Up
Ccnd1	4051.73	0.70379	0.17278	4.07343	4.63E-05	0.00038	3770.35	2501.06	2976.37	4527.34	5697.26	4837.99	Up
Tpcn2	2858.33	0.50403	0.15068	3.34498	0.00082	0.00472	2107.22	2237.53	2747.2	3113.26	3848.16	3096.63	Up
Tex45	15.7847	-1.5048	0.62168	-2.4205	0.0155	0.05447	22.2525	20.1234	27.6916	5.18358	7.39218	12.0648	Down
Zfp358	1390.72	-0.4293	0.09411	-4.5619	5.07E-06	5.36E-05	1577.03	1646.29	1565.05	1226.43	1174.3	1155.21	Down
Pnpla6	3233.66	0.32782	0.08954	3.66137	0.00025	0.00169	2755.44	2789.49	3058.49	3604.66	3632.73	3561.13	Up
Camsap3	1599.01	1.11631	0.11967	9.32821	1.08E-20	9.93E-19	1020.71	949.634	1058.01	2119.05	2427.8	2018.84	Up
Stxbp2	6437.48	0.83215	0.1144	7.27406	3.49E-13	1.36E-11	5070.68	4382.12	4439.25	8317.57	8914.97	7500.29	Up
Retn	171.806	-5.1617	1.01508	-5.085	3.68E-07	4.98E-06	148.995	581.663	272.142	1.03672	17.9524	9.04861	Down
Mcemp1	1873.35	2.43339	0.51385	4.73559	2.18E-06	2.50E-05	950.086	496.378	309.382	2779.44	4085.76	2619.07	Up
Fcor	38.8242	1.80341	0.41425	4.35342	1.34E-05	0.00013	18.3825	17.2487	16.233	47.6889	77.0899	56.3024	Up
Fcer2a	986.433	1.59224	0.22961	6.93466	4.07E-12	1.33E-10	679.186	420.676	374.314	1367.43	1393.95	1683.04	Up
Cd209a	278.901	-1.5167	0.33755	-4.4933	7.01E-06	7.18E-05	228.33	427.383	584.388	154.471	111.939	166.897	Down
Cd209d	55.4236	-2.1844	0.4181	-5.2246	1.75E-07	2.49E-06	69.6601	124.574	78.3004	25.9179	19.0085	15.081	Down
Cd209b	114.751	-2.1685	0.4108	-5.2786	1.30E-07	1.89E-06	130.613	263.521	169.014	23.8445	62.3055	39.2106	Down
Cd209f	253.854	-3.2137	0.32638	-9.8464	7.11E-23	7.90E-21	413.123	642.033	319.886	37.3218	45.4091	65.3511	Down
Cd209g	93.9469	-3.054	0.47531	-6.4252	1.32E-10	3.43E-09	150.93	250.106	102.172	11.4039	16.8964	32.1728	Down
Lrrc8e	831.792	0.81842	0.13543	6.04308	1.51E-09	3.23E-08	688.861	539.5	577.704	1037.75	1057.08	1089.85	Up
Ctxn1	1846.77	-0.4461	0.18128	-2.4607	0.01387	0.04991	2028.85	2304.61	2056.82	1729.24	1157.4	1803.69	Down
Timm44	2252.11	-0.2322	0.0979	-2.3721	0.01769	0.06056	2357.8	2545.13	2395.8	2128.38	2152.18	1933.39	Down
Cers4	3072.3	-0.5285	0.10363	-5.0998	3.40E-07	4.64E-06	3758.74	3360.61	3767.01	2368.9	2679.14	2499.43	Down
Vmn2r-ps	9.39636	-1.994	0.85587	-2.3298	0.01981	0.06633	11.61	15.3321	18.1428	1.03672	4.2241	6.03241	Down
Shcbp1	60.937	0.96547	0.39846	2.42299	0.01539	0.05417	36.7651	56.5373	30.5563	96.4146	53.8573	91.4915	Up
Gm5605	4.30286	5.60649	1.70333	3.2915	0.001	0.00556	0	0	0	4.14686	11.6163	10.054	Up
Efnb2	6610.45	-0.5297	0.12967	-4.0849	4.41E-05	0.00037	7830.96	6719.31	8881.37	5609.67	5061.53	5559.87	Down
Arglu1	8549.29	-0.2968	0.07999	-3.711	0.00021	0.00143	9002.6	9494.43	9779.91	7585.65	7849.44	7583.74	Down
Abhd13	2955.48	0.3147	0.10284	3.05997	0.00221	0.01084	2832.84	2482.85	2587.73	3160.95	3180.75	3487.74	Up
Myo16	55.167	-2.1495	0.5501	-3.9074	9.33E-05	0.00071	37.7326	98.7006	133.684	30.0648	13.7283	17.0918	Down
9530052E	120.607	-0.6313	0.21417	-2.9476	0.0032	0.01491	147.06	137.031	155.646	96.4146	93.9863	93.5023	Down
Col4a1	10569.2	0.69563	0.14767	4.71059	2.47E-06	2.80E-05	9177.72	6910.96	8119.37	12944.4	14783.3	11479.7	Up
Rab20	1001.96	1.07785	0.11751	9.17213	4.64E-20	4.05E-18	710.146	591.246	631.178	1342.55	1362.27	1374.38	Up
E230013L	103.963	0.54762	0.27594	1.98458	0.04719	0.13045	71.5951	106.367	75.4357	104.708	130.947	134.724	Up
Ankrd10	3389.89	-0.3276	0.08214	-3.9879	6.67E-05	0.00053	3694.89	3686.42	3937.94	2981.59	3021.29	3017.21	Down
Gm45592	25.3134	-1.4603	0.54096	-2.6995	0.00694	0.02835	31.9275	40.2469	39.1502	9.33044	22.1765	9.04861	Down
Sox1ot	8.93498	2.19636	0.9389	2.3393	0.01932	0.06498	3.87001	4.79129	0.95488	13.4773	7.39218	23.1242	Up
Tubgcp3	4276.2	-0.2045	0.08594	-2.3795	0.01734	0.0596	4520.17	4540.23	4676.06	3858.66	3854.49	4207.6	Down
4931415C	39.712	-0.8066	0.38362	-2.1027	0.03549	0.10436	43.5376	45.9964	62.0674	33.1749	25.3446	28.1512	Down

Atp11a	3285.22	1.9273	0.20387	9.45374	3.27E-21	3.19E-19	1774.4	1189.2	1140.13	4438.18	4936.92	6232.48	Up
Mcf2l	4676.29	-0.4498	0.14615	-3.0774	0.00209	0.01033	6043.01	4932.16	5223.21	4212.18	3294.8	4352.38	Down
F7	2945.26	3.01055	0.25899	11.6244	3.10E-31	6.82E-29	944.281	577.83	428.742	4990.75	6151.35	4578.6	Up
F10	869.437	2.53043	0.19431	13.0226	9.10E-39	4.01E-36	297.99	290.352	181.428	1467.99	1528.07	1450.79	Up
Proz	1043.75	2.04312	0.34087	5.99379	2.05E-09	4.25E-08	409.253	561.54	252.089	1595.51	997.944	2446.14	Up
Gm17023	34.4516	1.02536	0.46449	2.20753	0.02728	0.0853	19.35	22.9982	25.7818	50.7991	27.4567	60.3241	Up
Cul4a	5692.36	0.28829	0.08227	3.5041	0.00046	0.00288	5168.39	5282.88	4925.29	6033.69	6322.42	6421.5	Up
Lamp1	12504.1	0.73891	0.13384	5.52104	3.37E-08	5.52E-07	11124.3	8367.51	8618.77	15123.6	16119.2	15671.2	Up
Grtp1	1356.32	-0.3728	0.13984	-2.666	0.00768	0.03074	1491.89	1654.91	1444.74	1095.81	1371.78	1078.8	Down
2810030D	167.19	-0.6558	0.26866	-2.4409	0.01465	0.05208	208.013	174.403	231.082	107.818	174.244	107.578	Down
Adprhl1	202.258	-4.5564	0.84501	-5.3922	6.96E-08	1.07E-06	146.093	838.476	179.518	19.6976	12.6723	17.0918	Down
Tmco3	3505.93	0.42551	0.09038	4.70819	2.50E-06	2.83E-05	3046.66	3122.96	2808.31	4118.87	3903.07	4035.68	Up
Tfdp1	2853.72	0.24434	0.0857	2.85119	0.00436	0.0193	2498.09	2721.45	2618.29	3101.85	3134.28	3048.38	Up
Tmem255	39.7042	-1.776	0.42606	-4.1684	3.07E-05	0.00027	54.1801	77.619	52.5186	18.6609	22.1765	13.0702	Down
Coprs	407.063	0.40668	0.18036	2.25487	0.02414	0.07746	317.34	415.884	317.021	412.613	466.763	512.754	Up
Fbxo25	1680.86	0.31508	0.09607	3.27989	0.00104	0.00575	1525.75	1550.46	1418	1803.89	1860.72	1926.35	Up
Erich1	604.662	0.31554	0.11981	2.63375	0.00844	0.03322	567.923	544.291	504.178	665.572	687.473	658.538	Up
Gm10699	30.4991	-1.1782	0.5582	-2.1107	0.0348	0.10277	51.2776	39.2886	36.2855	9.33044	32.7368	14.0756	Down
Cln8	1485.15	0.28411	0.14016	2.0271	0.04265	0.12048	1204.54	1440.26	1373.12	1553	1867.05	1472.91	Up
Myom2	990.965	-4.291	0.74811	-5.7357	9.71E-09	1.77E-07	1401.91	3523.52	731.44	79.8271	62.3055	146.789	Down
Agpat5	1648.16	-0.2527	0.0928	-2.7235	0.00646	0.02672	1823.74	1752.66	1799.95	1467.99	1578.76	1465.87	Down
Defb1	8.73783	-2.6267	1.2987	-2.0225	0.04312	0.12153	6.77251	35.4556	2.86465	3.11015	4.2241	0	Down
Atp7b	3402.82	-0.768	0.19354	-3.968	7.25E-05	0.00057	4411.81	4576.64	3874.91	2644.66	1814.25	3094.62	Down
Alg11	1396.03	0.2212	0.11109	1.99111	0.04647	0.12884	1318.7	1228.49	1320.6	1384.02	1501.67	1622.72	Up
Nek5	2503.3	-0.5675	0.22829	-2.4858	0.01293	0.04714	2671.27	3157.46	3139.65	2237.23	1293.63	2520.54	Down
Ckap2	145.368	1.23275	0.31518	3.91132	9.18E-05	0.0007	98.6851	103.492	58.2479	214.6	147.844	249.339	Up
Thsd1	2191.57	-0.3643	0.14041	-2.5948	0.00946	0.0365	2344.26	2166.62	2889.48	1875.42	2051.86	1821.79	Down
Mrps31	1009.21	-0.2976	0.11369	-2.6174	0.00886	0.03454	1101.98	1205.49	1031.27	886.392	929.302	900.839	Down
Slc20a2	2152.31	-0.4171	0.19218	-2.1701	0.03	0.09178	2172.04	3223.58	1988.07	1760.34	2004.34	1765.48	Down
Vdac3	6328.36	-0.3467	0.11185	-3.0996	0.00194	0.00971	6454.2	7949.71	6851.28	5794.2	5475.49	5445.25	Down
Polb	2057.09	-0.3647	0.11059	-3.2975	0.00098	0.00547	2117.86	2268.2	2561	1781.08	1824.81	1789.61	Down
Plat	3604.04	-0.6946	0.25453	-2.7289	0.00635	0.02639	3090.2	3757.33	6518.03	2563.8	3193.42	2501.44	Down
Ank1	105.501	-1.2042	0.51968	-2.3172	0.02049	0.06803	91.9126	284.603	64.932	65.3131	53.8573	72.3889	Down
Sfrp1	1320.29	0.27637	0.1364	2.0261	0.04275	0.12069	1164.87	1374.14	1043.69	1382.98	1450.98	1505.09	Up
Ido1	91.2878	-0.9383	0.41078	-2.2843	0.02236	0.07279	92.8801	147.572	119.36	37.3218	100.322	50.27	Down
Adam3	277.378	-1.1109	0.23475	-4.7324	2.22E-06	2.53E-05	301.86	344.015	491.765	176.242	157.348	193.037	Down
Adam9	10932.2	0.72898	0.11647	6.25903	3.87E-10	9.20E-09	8303.1	8216.11	8163.29	12392.9	15620.7	12897.3	Up
Tacc1	11550.6	-0.342	0.08893	-3.8454	0.00012	0.00089	12478.8	13289.1	12971.1	9812.52	10913	9838.85	Down
5430421F	21.4432	-2.6746	0.81455	-3.2835	0.00103	0.00569	22.2525	64.2033	24.827	1.03672	5.28013	11.0594	Down
Gm31045	33.8144	-2.4145	0.48978	-4.9298	8.23E-07	1.04E-05	48.3751	60.3703	62.0674	16.5875	8.4482	7.03781	Down
Fgfr1	3208.08	-0.4243	0.10643	-3.9863	6.71E-05	0.00053	3462.69	4044.81	3521.61	2712.05	2868.16	2639.18	Down
Letm2	544.18	-0.7609	0.20307	-3.7471	0.00018	0.00127	591.143	688.988	773.455	375.291	329.48	506.722	Down
D830025C	19.3158	-1.96	0.61393	-3.1926	0.00141	0.00744	34.8301	27.7895	29.6014	3.11015	9.50423	11.0594	Down
Pomk	1045.43	0.33498	0.11369	2.94629	0.00322	0.01496	982.981	903.638	887.086	1148.68	1249.28	1100.91	Up
Erlin2	4502.75	0.34133	0.09166	3.72378	0.0002	0.00137	3793.57	4079.31	4044.88	4759.56	5025.62	5313.54	Up
Brf2	564.255	-0.2849	0.13851	-2.0566	0.03973	0.11391	637.583	642.992	578.659	469.632	566.03	490.636	Down
Rab11fip1	7949	0.33136	0.13653	2.42703	0.01522	0.05371	7402.35	6224.85	7492.97	8389.1	10193.8	7990.93	Up
Got1l1	141.257	-1.4282	0.39962	-3.5739	0.00035	0.00228	99.6527	215.608	302.698	80.8638	61.2495	87.4699	Down
Adrb3	858.373	-1.6824	0.19637	-8.5676	1.06E-17	7.78E-16	1145.52	1446.97	1333.97	388.768	514.284	320.723	Down
Eif4ebp1	1731.09	-0.4626	0.14732	-3.1401	0.00169	0.00864	1846.96	2096.67	2074.96	1656.67	1503.78	1207.49	Down
5430430B	89.8851	-0.9686	0.33643	-2.8791	0.00399	0.01793	111.263	83.3685	162.33	54.9459	57.0254	70.3781	Down
Gm26795	3.04317	-3.9589	1.84138	-2.15	0.03156	0.0953	1.935	11.4991	3.81953	0	0	1.0054	Down
Unc5d	87.559	1.11141	0.28218	3.93871	8.19E-05	0.00064	62.8876	48.8712	54.4283	129.589	133.059	96.5185	Up
Dusp26	73.3706	-1.9063	0.48435	-3.9358	8.29E-05	0.00064	101.588	107.325	138.458	22.8077	55.9693	14.0756	Down
Rnf122	829.328	0.4227	0.15367	2.75074	0.00595	0.025	737.236	624.785	763.906	966.219	1063.42	820.407	Up
Fut10	370.338	-0.6075	0.26019	-2.335	0.01954	0.06562	541.801	370.846	428.742	322.419	361.161	197.059	Down
Nrg1	830.096	-0.6486	0.15572	-4.1653	3.11E-05	0.00027	921.061	993.714	1125.81	644.837	738.162	556.992	Down
Purg	757.601	-0.3167	0.12321	-2.5707	0.01015	0.03869	919.126	812.603	789.688	652.094	665.296	706.797	Down
Tex15	549.05	0.45583	0.17445	2.61289	0.00898	0.03492	544.703	380.429	464.073	639.654	579.758	685.683	Up
Gsr	6038.41	0.31268	0.15529	2.01357	0.04406	0.12357	5881.44	5646.06	4632.14	6984.35	7553.75	5532.72	Up
Rbpms	9708.39	-0.5314	0.09277	-5.728	1.02E-08	1.84E-07	10921.2	12359.6	11148.3	8135.11	8073.31	7612.9	Down
Dctn6	4280.85	-0.2694	0.0853	-3.1588	0.00158	0.0082	4689.48	4695.47	4653.14	4073.26	3902.01	3671.72	Down
Gm19410	15.0168	-2.6428	0.96128	-2.7493	0.00597	0.02507	7.74001	56.5373	13.3684	6.2203	4.2241	2.0108	Down
Prag1	499.343	0.76509	0.2083	3.67296	0.00024	0.00162	351.203	363.18	395.322	590.928	806.803	488.625	Up
Gm6213	61.4643	-2.4853	0.61597	-4.0348	5.47E-05	0.00044	105.458	60.3703	147.052	38.3585	9.50423	8.04321	Down
Msr1	840.551	2.85815	0.24098	11.8605	1.90E-32	4.53E-30	248.648	212.733	149.917	1088.55	1793.13	1550.33	Up
Micu3	851.22	-0.4486	0.13231	-3.3904	0.0007	0.00412	909.451	932.386	1105.75	712.224	686.416	761.088	Down
Mtmr7	909.138	0.46717	0.15637	2.98763	0.00281	0.01331	800.124	739.776	749.583	1178.75	1131	855.596	Up
Slc7a2	4149.59	0.43028	0.13276	3.24091	0.00119	0.00646	3655.22	3525.43	3425.16	4339.69	5611.72	4340.32	Up
Mtus1	12644	-0.648	0.11418	-5.675	1.39E-08	2.44E-07	14311.3	16008.7	15990.5	9169.75	9343.71	11040.3	Down
Fgl1	416.665	5.01651	0.67269	7.45741	8.82E-14	3.77E-12	21.285	46.9547	6.68418	786.867	889.173	749.024	Up
Asah1	14298.5	0.49756	0.15295	3.25301	0.00114	0.00625	13049.7	11210.7	11310.6	17563	19207	13450.3	Up
Frg1	1754.41	-0.2481	0.09375	-2.647	0.00812	0.03223	1864.38	1920.35	1929.82	1572.7	1694.92	1544.3	Down
Gm6180	367.959	0.35302	0.14814	2.38308	0.01717	0.05912	294.12	323.891	351.397	431.274	413.962	393.112	Up
Fat1	5109.4	0.33572	0.10021	3.35005	0.00081	0.00465	4451.47	4475.07	4626.41	5388.85	5445.92	6268.67	Up
Cyp4v3	1850.92	1.00113	0.17841	5.61124	2.01E-08	3.41E-07	1537.36	1055.04	1107.66	2304.62	2273.62	2827.19	Up
Tlr3	2156.71	0.339	0.10011	3.38641	0.00071	0.00417	1866.31	1833.15	2013.85	2297.36	2560.86	2368.72	Up
1700029J(	1292.15	-0.7302	0.1849	-3.949	7.85E-05	0.00061	1312.9	1828.36	1695.87	1065.74	777.235	1072.76	Down
Ufsp2	2135.94	0.29999	0.08242	3.63991	0.00027	0.00182	1919.52	1948.14	1876.34	2352.31	2369.72	2349.62	Up
Ankrd37	558.552	0.85403	0.24491	3.48719	0.00049	0.00304	477.946	429.3	286.465	920.604	615.663	621.338	Up
Snx25	9647.14	0.71761	0.13153	5.45602	4.87E-08	7.73E-07	8507.24	6372.42	7008.84	11787.5	11531.8	12675.1	Up
Slc25a4	13757.5	-1.0354	0.23967	-4.32	1.56E-05	0.00015	14812.4	27093.8	13571.8	9008.02	9585.54	8473.52	Down
Gm39168	10.0949	-1.5472	0.75948	-2.0372	0.04163	0.11824	15.48	19.1652	10.5037	6.2203	3.16808	6.03241	Down

Acsl1	5387.34	-0.641	0.26156	-2.4507	0.01426	0.05104	5700.52	9320.02	4674.15	4118.87	3225.1	5285.39	Down
Cenpu	77.0675	0.7556	0.35081	2.15386	0.03125	0.09453	73.5301	34.4973	63.9772	106.782	97.1543	86.4645	Up
Primpol	252.788	0.36938	0.17143	2.15467	0.03119	0.0944	244.778	203.151	213.894	263.326	302.023	289.555	Up
Casp3	940.706	0.50734	0.15151	3.34853	0.00081	0.00467	912.354	727.318	691.335	1009.76	1176.41	1127.05	Up
Stox2	1369.38	-0.593	0.16045	-3.6958	0.00022	0.0015	1516.07	1825.48	1599.43	1066.78	915.574	1292.95	Down
Trappc11	3445.18	0.21722	0.084	2.58601	0.00971	0.03729	3228.55	3087.51	3242.78	3593.26	3850.27	3668.71	Up
Gm8623	17.6532	-1.1451	0.53812	-2.128	0.03334	0.09932	23.22	23.9565	25.7818	9.33044	10.5603	13.0702	Down
Gm9892	734.687	0.47958	0.12858	3.72997	0.00019	0.00135	551.476	642.992	646.456	892.612	872.277	802.31	Up
Neil3	101.835	0.7316	0.33756	2.16735	0.03021	0.09222	88.0426	96.7841	44.8795	133.736	109.827	137.74	Up
Spcs3	5224.89	0.24193	0.10067	2.40324	0.01625	0.05663	4495.98	4742.42	5124.86	5612.78	6023.57	5349.74	Up
Hpgd	38772.5	-0.5588	0.12978	-4.3057	1.66E-05	0.00015	41275.5	49890.8	47399.4	35195.5	31233	27640.5	Down
Hand2os1	87.024	-3.7453	0.52174	-7.1785	7.05E-13	2.59E-11	144.158	229.982	111.721	22.8077	8.4482	5.027	Down
5033428I2	3.19375	-5.0721	1.83791	-2.7597	0.00579	0.02448	2.9025	7.66607	8.59395	0	0	0	Down
Galnt7	1034.12	0.83227	0.12481	6.66846	2.58E-11	7.60E-10	737.236	776.19	718.072	1177.71	1436.19	1359.3	Up
Mfap3l	2677.12	1.21747	0.13515	9.00803	2.10E-19	1.73E-17	1795.68	1656.83	1377.9	3419.09	3968.54	3844.65	Up
Clcn3	4212.08	0.22918	0.09437	2.42846	0.01516	0.05355	3627.16	4028.52	3979	4312.74	4613.77	4711.31	Up
B230317F.	5.6363	-2.8552	1.17763	-2.4245	0.01533	0.054	6.77251	12.4574	10.5037	2.07343	0	2.0108	Down
Palld	5125.39	-1.0374	0.19317	-5.3705	7.85E-08	1.19E-06	4966.19	8193.11	7518.75	3058.31	3781.63	3234.37	Down
Gm45418	27.4175	-1.9398	0.73316	-2.6458	0.00815	0.03232	25.155	88.1598	17.1879	15.5507	7.39218	11.0594	Down
Ddx60	679.751	0.55866	0.16841	3.31734	0.00091	0.00515	601.786	586.454	461.208	760.949	737.106	931.001	Up
Spock3	23.615	-2.4672	0.55224	-4.4675	7.91E-06	7.97E-05	32.895	51.746	35.3307	8.29373	7.39218	6.03241	Down
Cpe	2114.47	-1.7266	0.4668	-3.6987	0.00022	0.00149	2741.9	5086.44	1914.54	1238.88	774.067	931.001	Down
Msmo1	6128.52	1.15351	0.15267	7.55541	4.18E-14	1.88E-12	3989.01	4281.5	3132.97	8022.11	9591.88	7753.65	Up
Klhl2	2905.05	-0.2213	0.08691	-2.5459	0.0109	0.04105	2961.52	3228.37	3192.17	2709.98	2696.03	2642.19	Down
Tmem192	796.235	0.34766	0.13079	2.65817	0.00786	0.03133	735.301	654.491	712.343	851.144	992.664	831.467	Up
Gm10663	19.9331	3.23395	0.78028	4.14458	3.40E-05	0.00029	0	3.83303	7.63906	31.1015	33.7928	43.2322	Up
Smim31	763.33	1.9323	0.23224	8.32028	8.78E-17	5.61E-15	233.168	427.383	290.284	1015.98	1282.01	1331.15	Up
Marchf1	935.766	1.13786	0.20142	5.64925	1.61E-08	2.80E-07	733.366	584.538	436.381	1221.25	1444.64	1194.42	Up
Npy1r	790.025	0.66419	0.19425	3.41918	0.00063	0.00376	674.349	688.03	471.712	895.722	871.221	1139.12	Up
Naf1	1484.74	-0.3986	0.11531	-3.4563	0.00055	0.00335	1610.89	1683.66	1771.31	1258.57	1177.47	1406.56	Down
Psd3	3042.68	-0.5125	0.14127	-3.6276	0.00029	0.0019	3232.42	3938.44	3561.71	2186.43	2462.65	2874.44	Down
Sh2d4a	2885.58	0.92434	0.1455	6.35304	2.11E-10	5.31E-09	2316.2	1702.83	1955.6	3943.67	3993.89	3401.27	Up
Csgalnact	1185.72	-0.509	0.12089	-4.2107	2.55E-05	0.00023	1269.36	1396.18	1512.53	948.595	1047.58	940.05	Down
Lpl	16320	-0.6124	0.28095	-2.1797	0.02928	0.09026	17838.8	27151.3	14207.7	15364.1	15306	8052.26	Down
Slc18a1	208.917	1.4949	0.20762	7.2003	6.01E-13	2.25E-11	99.6527	127.448	101.218	290.28	283.015	351.89	Up
Atp6v1b2	11610.7	0.84773	0.09628	8.80453	1.31E-18	1.02E-16	8903.92	8146.16	7832.9	14527.5	15836.2	14417.4	Up
D130040H	144.058	0.65861	0.29966	2.19786	0.02796	0.08709	125.775	118.824	90.7139	137.883	147.844	243.307	Up
Gm43263	4.67158	-3.6379	1.52165	-2.3907	0.01681	0.05822	6.77251	13.4156	5.7293	0	2.11205	0	Down
Zfp869	2762.16	-0.2432	0.10358	-2.3481	0.01887	0.0638	2929.59	2875.73	3177.85	2705.83	2532.35	2351.63	Down
Zfp963	353.469	-0.4642	0.19019	-2.4408	0.01465	0.05208	375.391	446.549	407.735	336.933	235.494	318.712	Down
Gm20422	113.786	-0.5804	0.23039	-2.5191	0.01177	0.04366	144.158	126.49	138.458	103.672	84.482	85.4591	Down
Gmip	2006.09	0.39877	0.11997	3.32383	0.00089	0.00504	1899.21	1632.87	1659.59	2241.38	2491.16	2112.35	Up
Pbx4	329.247	-0.597	0.23371	-2.5543	0.01064	0.04025	454.726	321.017	413.464	336.933	229.158	220.183	Down
Cilp2	134.36	-5.336	0.67431	-7.9133	2.51E-15	1.32E-13	146.093	528.001	112.676	6.2203	2.11205	11.0594	Down
Yjefn3	117.621	-0.9243	0.34344	-2.6914	0.00712	0.02892	104.49	190.693	167.105	81.9006	55.9693	105.567	Down
Tm6sf2	475.162	-0.5671	0.27675	-2.0493	0.04044	0.11552	480.848	584.538	636.907	418.833	229.158	500.69	Down
Ncan	43.8276	-2.6932	0.43172	-6.2382	4.43E-10	1.04E-08	83.2051	75.7024	68.7516	10.3672	16.8964	8.04321	Down
Slc25a42	875.542	-0.5864	0.11925	-4.9172	8.78E-07	1.10E-05	1135.85	1014.8	1002.63	656.241	712.817	730.926	Down
Kxd1	2408.42	-0.2221	0.08711	-2.5501	0.01077	0.04068	2494.22	2688.87	2597.28	2281.81	2196.53	2191.77	Down
Ell	2415.7	0.27243	0.09888	2.75519	0.00587	0.02474	2060.78	2135	2369.06	2587.64	2664.35	2677.38	Up
Isyna1	3692.11	0.57478	0.09077	6.33221	2.42E-10	6.00E-09	2846.39	3073.14	2979.23	4482.76	4174.47	4596.69	Up
Ssbp4	3416.51	0.23757	0.09289	2.55757	0.01054	0.03992	3170.5	3122.01	3114.83	3838.96	3834.43	3418.36	Up
Lrrc25	245.076	0.83946	0.16661	5.03845	4.69E-07	6.22E-06	169.313	187.819	169.969	303.758	338.984	300.615	Up
Gdf15	430.938	1.73713	0.40119	4.32996	1.49E-05	0.00014	302.828	184.944	108.857	730.885	909.238	348.874	Up
Mpv17l2	1463.62	0.3168	0.11593	2.73276	0.00628	0.02613	1359.34	1328.15	1223.2	1786.26	1556.58	1528.21	Up
Ifi30	4958.37	1.03585	0.15776	6.56602	5.17E-11	1.43E-09	3951.28	2984.02	2817.86	7139.86	6924.36	5932.87	Up
Arrdc2	1082.9	-0.5355	0.13278	-4.0327	5.51E-05	0.00045	1245.17	1443.14	1156.36	939.265	886.005	827.445	Down
Kcnn1	113.92	-1.0036	0.41822	-2.3996	0.01641	0.05706	216.72	149.488	89.759	79.8271	103.49	44.2376	Down
Myo9b	4435.12	0.22757	0.09547	2.38354	0.01715	0.05907	4270.55	3769.79	4217.72	4767.86	4955.93	4628.87	Up
Ushbp1	5801.99	-0.3247	0.13919	-2.3329	0.01965	0.0659	6083.65	5669.06	7603.73	5183.58	5489.22	4782.69	Down
Ankle1	42.391	0.81563	0.39605	2.05939	0.03946	0.11331	24.1875	43.1216	24.827	53.9092	57.0254	51.2754	Up
Ano8	1291.46	0.30108	0.10025	3.00329	0.00267	0.01274	1125.2	1125.95	1220.34	1482.5	1399.23	1395.5	Up
Plvap	8508.16	-0.9362	0.15785	-5.9312	3.01E-09	6.08E-08	12136.3	8918.51	12472.7	6326.04	6024.62	5170.78	Down
Ccdc194	62.1397	2.62941	0.81062	3.24371	0.00118	0.00641	22.2525	11.4991	18.1428	26.9546	29.5687	264.42	Up
Slc27a1	3010.57	-0.3104	0.13253	-2.3424	0.01916	0.06456	3298.21	3803.33	2898.07	2828.16	2737.22	2498.42	Down
Colgalt1	5830.34	0.52848	0.11937	4.42735	9.54E-06	9.39E-05	5090.99	4539.27	4692.29	7338.91	7319.31	6001.24	Up
Jak3	3218.27	0.60659	0.11971	5.06736	4.03E-07	5.42E-06	2598.71	2321.86	2733.83	3530.02	4248.39	3876.83	Up
B3gnt3	2561.76	0.45774	0.16323	2.80425	0.00504	0.02188	2051.1	2144.58	2280.26	3125.7	3458.48	2310.41	Up
Gm5373	24.3747	2.56622	0.60309	4.25509	2.09E-05	0.00019	9.67501	4.79129	6.68418	32.1382	30.6247	62.3349	Up
Zfp961	1117.73	-0.2541	0.12722	-1.9977	0.04575	0.12722	1282.91	1204.53	1160.18	1113.43	1046.52	898.828	Down
Gm45734	140.006	0.5075	0.25105	2.02156	0.04322	0.12176	146.093	93.9093	106.947	179.352	147.844	165.891	Up
Rab8a	4659.05	0.42595	0.09369	4.54613	5.46E-06	5.73E-05	4269.58	3771.71	3887.33	5405.44	5477.6	5142.63	Up
Hsh2d	212.828	0.52875	0.21727	2.43363	0.01495	0.0529	218.655	152.363	151.826	264.363	233.382	256.377	Up
Cib3	40.6156	0.71774	0.35747	2.00783	0.04466	0.12492	29.9925	29.706	32.466	54.9459	43.297	53.2862	Up
Fam32a	5865.57	0.20934	0.10137	2.06521	0.0389	0.11206	5632.79	5050.02	5639.54	6707.55	5875.73	6287.78	Up
Klf2	17824.6	-0.7169	0.22072	-3.2478	0.00116	0.00633	20699.7	27117.8	18674.6	16217.3	14926.9	9311.02	Down
Calr3	153.89	-1.0247	0.29238	-3.5048	0.00046	0.00287	183.825	260.646	174.744	95.3779	76.0338	132.713	Down
1700030K	891.546	-0.3429	0.11817	-2.9018	0.00371	0.01686	969.436	1093.37	928.146	795.161	792.019	771.142	Down
Med26	53.1782	-0.9526	0.35299	-2.6988	0.00696	0.02839	72.5626	67.0781	70.6613	45.6155	38.0169	25.135	Down
Tmem38a	1709.75	-1.5884	0.24238	-6.5532	5.63E-11	1.55E-09	2159.46	3704.63	1834.33	861.511	869.109	829.456	Down
Hmox1	3317.15	1.1822	0.19798	5.97137	2.35E-09	4.83E-08	2556.14	1805.36	1726.43	4622.72	5471.27	3720.99	Up
Mcm5	667.793	0.71927	0.16164	4.44992	8.59E-06	8.58E-05	576.631	511.71	425.878	852.18	745.554	894.807	Up
Rasd2	813.177	2.01237	0.60357	3.33411	0.00086	0.00488	600.818	201.234	167.105	1266.87	1611.49	1031.54	Up

Gm10649	23.0725	-1.1302	0.48252	-2.3422	0.01917	0.06458	27.09	31.6225	36.2855	16.5875	14.7844	12.0648	Down
Arhgap10	756.292	-0.7313	0.13479	-5.4256	5.77E-08	9.02E-07	933.639	1039.71	858.44	547.386	615.663	542.916	Down
4933431K	104.659	-0.8334	0.36407	-2.2892	0.02207	0.07211	105.458	193.568	103.127	60.1295	100.322	65.3511	Down
Ttc29	1815.64	-1.0131	0.22033	-4.5979	4.27E-06	4.59E-05	2220.42	2620.84	2443.55	1133.13	852.212	1623.72	Down
Lsm6	1736.93	-0.255	0.11343	-2.2477	0.02459	0.07865	1734.73	1901.19	2033.9	1651.49	1627.34	1472.91	Down
Zfp827	807.852	0.67765	0.14619	4.63529	3.56E-06	3.90E-05	687.894	600.828	575.794	965.182	1123.61	893.801	Up
Smad1	2021.34	0.38832	0.09196	4.22257	2.42E-05	0.00022	1827.61	1661.62	1763.67	2250.71	2276.79	2347.61	Up
Tpd52-ps	105.555	0.58364	0.22897	2.54901	0.0108	0.04078	85.1401	76.6607	91.6688	122.332	128.835	128.691	Up
Hhip	2314.42	0.67686	0.15668	4.32009	1.56E-05	0.00015	2143.98	1595.5	1604.2	2857.19	3126.89	2558.75	Up
Gm31105	116.909	-0.7351	0.25981	-2.8292	0.00467	0.02046	178.02	131.281	128.909	101.598	79.2019	82.4429	Down
Inpp4b	1600.04	0.35957	0.12197	2.94796	0.0032	0.0149	1309.03	1515.97	1379.81	1775.89	1973.71	1645.84	Up
Gm9655	13.3007	1.38673	0.68392	2.02761	0.0426	0.12037	7.74001	4.79129	9.54883	26.9546	12.6723	18.0972	Up
Tbc1d9	2122.73	0.73196	0.10393	7.04274	1.89E-12	6.51E-11	1584.77	1656.83	1545	2439.39	2680.19	2830.2	Up
Scoc	2571.5	-0.3247	0.09993	-3.2493	0.00116	0.0063	2783.5	2735.83	3059.44	2205.09	2415.13	2229.98	Down
Ndufb7	2283.15	-0.232	0.11039	-2.1013	0.03562	0.10466	2316.2	2590.17	2492.24	2088.98	2278.9	1932.38	Down
Gipc1	2871.93	0.2935	0.104	2.82207	0.00477	0.02087	2668.37	2597.84	2476.01	3295.72	3321.2	2872.43	Up
Pkn1	1383.77	0.55681	0.14699	3.78814	0.00015	0.0011	1259.69	954.426	1145.86	1508.42	1819.53	1614.67	Up
Asf1b	183.774	0.77094	0.22271	3.46157	0.00054	0.00329	165.443	134.156	107.902	234.298	217.541	243.307	Up
1700067K	52.038	-1.5579	0.38262	-4.0718	4.66E-05	0.00038	64.8226	81.452	86.8943	25.9179	17.9524	35.189	Down
Gm10643	187.104	-0.5041	0.21505	-2.3442	0.01907	0.06428	186.728	264.479	207.21	145.14	154.18	164.886	Down
Cc2d1a	1605.31	0.24264	0.08978	2.70254	0.00688	0.02813	1460.93	1436.43	1514.44	1748.94	1772.01	1699.13	Up
4930432K	35.5167	-1.1187	0.40364	-2.7714	0.00558	0.02376	43.5376	45.9964	56.3381	26.9546	22.1765	18.0972	Down
Gm26532	941.213	-0.514	0.23838	-2.156	0.03108	0.09417	1297.42	912.262	1111.48	664.535	1062.36	599.219	Down
Gm50464	53.2178	-0.6824	0.33621	-2.0296	0.0424	0.11993	59.9851	67.0781	69.7065	32.1382	40.129	50.27	Down
Nacc1	4321.68	0.25043	0.097	2.5818	0.00983	0.03767	3720.04	4119.55	4002.87	4429.89	4988.66	4669.08	Up
Dand5	314.08	-0.3723	0.15614	-2.3843	0.01711	0.05901	358.943	377.554	326.57	261.252	296.743	263.415	Down
Calr	31561.8	0.48497	0.10211	4.74957	2.04E-06	2.35E-05	25181.2	25352.6	28384.8	36288.2	39687.5	34476.2	Up
Gcdh	985.058	-0.4066	0.10439	-3.8952	9.81E-05	0.00075	1152.29	1152.79	1063.74	858.401	861.717	821.413	Down
Dnase2a	1215.81	0.99363	0.21352	4.65345	3.26E-06	3.60E-05	1049.74	719.652	669.373	1690.88	1871.28	1293.95	Up
Junb	10538.5	0.59108	0.24178	2.44467	0.0145	0.05164	7827.09	10021.5	7379.33	13027.4	16829.9	8145.76	Up
Hook2	1715.95	0.3968	0.10581	3.75013	0.00018	0.00126	1552.84	1428.76	1462.88	2064.1	1798.41	1988.68	Up
Tnpo2	656.283	0.55148	0.17018	3.24054	0.00119	0.00646	588.241	436.008	572.93	720.518	740.274	879.726	Up
Wdr83	1142.49	-0.2268	0.1039	-2.1826	0.02906	0.08979	1258.72	1282.15	1155.41	1082.33	1052.86	1023.5	Down
Man2b1	7382.97	0.86824	0.13601	6.3834	1.73E-10	4.44E-09	5983.03	5232.09	4463.12	9706.77	10154.7	8758.05	Up
Mylk3	930.744	-4.3688	0.76147	-5.7373	9.62E-09	1.75E-07	1088.44	3395.11	843.162	76.717	43.297	137.74	Down
Itfg1	5251.21	0.31457	0.10114	3.11014	0.00187	0.00942	4953.61	4854.54	4234.91	5633.51	5833.48	5997.22	Up
Phkb	2964.01	0.27621	0.11933	2.3146	0.02063	0.06835	2667.4	2788.53	2587.73	3020.99	3026.57	3692.84	Up
Gm10638	41.4222	-0.9561	0.40443	-2.3642	0.01807	0.06165	39.6676	60.3703	63.9772	21.771	29.5687	33.1782	Down
Adcy7	4823.6	0.66264	0.12311	5.38241	7.35E-08	1.12E-06	4193.15	3471.77	3539.75	5607.6	6505.12	5624.21	Up
Brd7	5702.62	0.59208	0.13397	4.41947	9.89E-06	9.70E-05	5226.44	4090.81	4328.48	6624.61	7580.15	6365.19	Up
Snx20	591.953	0.94088	0.18193	5.17162	2.32E-07	3.25E-06	472.141	389.053	355.216	725.701	921.91	687.694	Up
Cyld	3048.63	0.27486	0.09333	2.94488	0.00323	0.01501	2816.4	2705.16	2755.79	3098.74	3451.09	3464.61	Up
Tox3	842.953	0.27858	0.13467	2.0687	0.03857	0.11139	862.044	747.442	676.057	889.502	934.582	948.093	Up
Aktip	3424.79	-0.3882	0.08338	-4.6563	3.22E-06	3.56E-05	3885.49	3723.79	4039.15	2938.05	3003.34	2958.89	Down
Fto	3730.44	-0.2181	0.09234	-2.3615	0.0182	0.06202	3891.29	4320.79	3823.35	3465.74	3450.03	3431.43	Down
Gm36325	8.15446	6.53028	1.5716	4.15518	3.25E-05	0.00028	0	0	0	10.3672	7.39218	31.1674	Up
Irx3	2720.49	0.65226	0.11301	5.77171	7.85E-09	1.46E-07	2211.71	2135.96	1999.52	3589.11	3404.63	2982.02	Up
Irx3os	414.162	1.10357	0.13336	8.27495	1.29E-16	8.06E-15	254.453	263.521	271.187	589.891	544.909	561.014	Up
Gm36670	6.8726	3.27564	1.34485	2.43569	0.01486	0.05268	3.87001	0	0	18.6609	12.6723	6.03241	Up
Crnde	1117.51	0.40263	0.14428	2.79062	0.00526	0.0226	1110.69	854.767	922.417	1348.77	1157.4	1311.04	Up
Irx5	2213.29	0.50399	0.09217	5.4678	4.56E-08	7.28E-07	1839.22	1921.31	1731.2	2652.96	2589.37	2545.67	Up
Mmp2	3428.51	0.50178	0.17965	2.7931	0.00522	0.02248	2281.37	3228.37	3005.02	4136.5	3276.85	4642.94	Up
Lpcat2	1035.02	0.73312	0.20398	3.59399	0.00033	0.00213	863.011	883.514	586.298	1213.99	1110.94	1552.34	Up
Slc6a2	5167.86	-0.4524	0.17553	-2.5774	0.00995	0.03807	7314.31	5191.85	5408.46	5078.87	4291.69	3721.99	Down
Ces1c	23.8811	4.14321	0.70576	5.87054	4.34E-09	8.56E-08	1.935	2.87478	2.86465	37.3218	36.9609	61.3295	Up
Ces1d	43534.2	-0.5934	0.16783	-3.5354	0.00041	0.00259	61359.9	50751.3	44976.9	35631.9	28362.7	40122.5	Down
Ces1e	815.668	-0.6702	0.19958	-3.358	0.00078	0.00455	1012.97	1036.84	955.838	642.764	459.371	786.223	Down
Ces1f	914.531	-2.8988	0.78006	-3.7161	0.0002	0.00141	2996.35	1129.79	712.343	234.298	87.6501	326.755	Down
Gnao1	1596.89	-0.7837	0.12652	-6.1939	5.87E-10	1.34E-08	1783.11	2040.13	2237.29	1143.5	1248.22	1129.07	Down
Amfr	12823.4	0.34563	0.09358	3.69339	0.00022	0.00152	12117	10909.8	10857	13960.4	15160.3	13935.9	Up
Bbs2	1791.08	-0.5289	0.1289	-4.1036	4.07E-05	0.00034	1963.06	2084.21	2300.31	1528.12	1287.29	1583.51	Down
Mt3	21.4796	-5.3493	1.03792	-5.1539	2.55E-07	3.55E-06	39.6676	63.2451	22.9172	1.03672	0	2.0108	Down
Gm45774	4.18838	-3.4786	1.56684	-2.2202	0.02641	0.08319	9.67501	3.83303	9.54883	2.07343	0	0	Down
Mt1	2146.61	-0.572	0.24182	-2.3653	0.01801	0.06154	2584.2	2778.95	2336.6	1963.54	2162.74	1053.66	Down
Ap3s1-ps:	35.2135	0.86469	0.43494	1.98807	0.0468	0.12953	27.09	15.3321	32.466	49.7624	45.4091	41.2214	Up
9330175E	112.512	0.67336	0.29129	2.31168	0.0208	0.06873	79.3351	100.617	80.2102	97.4513	162.628	154.832	Up
Nlrc5	1480.24	0.45684	0.16084	2.84027	0.00451	0.01989	1268.39	1352.1	1122.94	1390.24	1964.21	1783.58	Up
Cpne2	1205.9	1.05964	0.10761	9.84738	7.04E-23	7.86E-21	768.196	814.52	762.951	1620.39	1736.11	1533.24	Up
Arl2bp	7053.46	-0.3859	0.11794	-3.2718	0.00107	0.00589	6942.79	8221.86	8808.79	6364.4	6030.96	5951.97	Down
Pllp	306.722	-0.5318	0.21053	-2.526	0.01154	0.043	272.835	407.26	407.735	270.583	237.606	244.312	Down
Ccl22	430.804	1.05897	0.32005	3.30882	0.00094	0.00528	317.34	143.739	377.179	644.837	625.167	476.56	Up
Coq9	2527.73	-0.6247	0.2153	-2.9016	0.00371	0.01686	2551.3	4259.46	2389.12	2027.82	1940.97	1997.73	Down
Polr2c	2443.21	-0.3808	0.08558	-4.4493	8.61E-06	8.60E-05	2753.51	2760.74	2776.8	2149.11	2190.2	2028.9	Down
Ccdc102a	1046.87	-0.543	0.13014	-4.1727	3.01E-05	0.00026	1101.98	1256.28	1366.44	910.237	826.868	819.402	Down
Adgrg1	8077.21	0.86607	0.11248	7.69983	1.36E-14	6.44E-13	6499.67	5347.08	5322.52	10387.9	10628.9	10277.2	Up
Kifc3	1743.13	0.40185	0.14038	2.8626	0.0042	0.01874	1676.68	1272.57	1556.46	1957.32	2145.84	1849.94	Up
Tepp	71.0547	-1.4898	0.37726	-3.9489	7.85E-05	0.00061	135.45	79.5355	99.3078	32.1382	51.7452	28.1512	Down
Cfap20	3646.69	-0.3242	0.08041	-4.0318	5.54E-05	0.00045	3979.33	4206.76	3978.04	3246.99	3193.42	3275.6	Down
4933406B	477.786	-1.3255	0.16974	-7.8089	5.77E-15	2.91E-13	601.786	764.69	682.741	238.445	271.399	307.653	Down
Ndrg4	650.227	-2.9711	0.70907	-4.1901	2.79E-05	0.00024	576.631	2357.32	526.14	164.838	145.732	130.702	Down
Slc38a7	1155.02	0.35341	0.15778	2.23985	0.0251	0.07996	1188.09	882.556	972.071	1194.3	1460.48	1232.62	Up
Got2	3981.07	-0.3936	0.17354	-2.2679	0.02334	0.07546	4328.6	5686.31	3547.39	3533.13	3345.49	3445.51	Down
Impdh2-p	691.188	-0.2471	0.11873	-2.0813	0.03741	0.10877	769.164	717.736	763.906	589.891	637.839	668.592	Down

Cdh11	5092.3	-0.3424	0.12533	-2.7316	0.0063	0.02621	4806.55	6076.32	6198.14	4430.92	4515.56	4526.31 Down
Cdh5	26475.9	-0.3799	0.13605	-2.7921	0.00524	0.02253	28120.4	28347.2	33356.9	22188.8	26523.1	20319.2 Down
Gm45663	37.4515	-0.8354	0.40061	-2.0854	0.03703	0.10796	44.5051	40.2469	59.2027	26.9546	31.6808	22.1188 Down
Bean1	24.1495	-1.3297	0.6604	-2.0134	0.04407	0.12359	26.1225	55.579	21.9623	5.18358	17.9524	18.0972 Down
Cklf	534.39	0.45435	0.1666	2.72724	0.00639	0.0265	527.288	406.302	419.194	559.827	687.473	606.257 Up
Ces2c	35.1256	3.23416	0.84755	3.81589	0.00014	0.00099	14.5125	5.74955	0	47.6889	105.603	37.1998 Up
Ces2g	1630.27	-0.5974	0.13332	-4.4812	7.42E-06	7.55E-05	2178.81	1798.65	1911.68	1167.34	1436.19	1288.92 Down
Elmo3	2072.27	0.39774	0.08419	4.72423	2.31E-06	2.62E-05	1754.08	1839.86	1771.31	2367.86	2333.82	2366.71 Up
Lrrc29	329.357	0.47715	0.15866	3.00744	0.00263	0.01259	307.665	257.772	260.683	398.099	396.01	355.912 Up
Lrrc36	3427.01	-0.6173	0.19725	-3.1294	0.00175	0.00891	3512.03	4578.56	4357.13	3199.31	1964.21	2950.85 Down
Tppp3	28798	-0.6098	0.11457	-5.3221	1.03E-07	1.52E-06	34279.5	31520	38584.9	24553.6	21397.2	22452.6 Down
Zdhhc1	9389.61	-0.2743	0.11603	-2.364	0.01808	0.06165	9116.77	10684.6	11037.5	8840.08	7807.2	8851.55 Down
Hsd11b2	489.59	3.43436	0.31782	10.8061	3.22E-27	5.56E-25	76.4326	111.158	61.1125	1049.16	1108.83	530.852 Up
Atp6v0d1	8804.1	0.76258	0.11165	6.83031	8.47E-12	2.68E-10	6968.91	6555.45	6065.42	11110.5	12069.3	10055 Up
Agrp	129.894	-0.8349	0.27216	-3.0677	0.00216	0.01062	190.598	141.822	167.105	103.672	67.5856	108.583 Down
Pard6a	398.931	-0.3356	0.16283	-2.0608	0.03932	0.113	415.058	501.169	419.194	383.585	313.64	360.939 Down
Enkd1	604.775	-0.6045	0.14463	-4.1794	2.92E-05	0.00026	735.301	690.904	762.951	501.77	412.906	524.819 Down
Tsnaxip1	1138.51	-0.9238	0.18632	-4.9581	7.12E-07	9.12E-06	1377.72	1629.04	1466.7	825.226	590.318	942.061 Down
Thap11	1718.82	-0.2015	0.10075	-1.9998	0.04552	0.12667	1913.72	1721.99	1880.16	1668.08	1597.77	1531.23 Down
Slc12a4	1634.4	0.52371	0.15148	3.45722	0.00055	0.00334	1585.73	1162.37	1274.77	1898.23	2106.77	1778.55 Up
Gm45752	52.2306	1.19882	0.32988	3.63413	0.00028	0.00186	31.9275	32.5808	30.5563	70.4967	84.482	63.3403 Up
Esrp2	2954.65	0.35923	0.12558	2.86052	0.00423	0.01883	2332.65	2442.6	2990.69	3394.21	3344.43	3223.32 Up
Pla2g15	3126.23	1.44493	0.22237	6.49797	8.14E-11	2.18E-09	2143.02	1543.75	1352.11	4393.6	5783.85	3541.02 Up
Slc7a6	1828.29	-0.3494	0.17063	-2.0478	0.04058	0.11578	1752.15	2037.26	2356.65	1517.75	1387.62	1918.3 Down
Smpd3	437.742	0.47348	0.18943	2.49944	0.01244	0.04568	398.611	360.305	340.893	549.459	397.066	580.116 Up
Gm10073	552.345	-0.5189	0.1818	-2.8541	0.00432	0.01916	632.746	593.162	725.711	491.403	511.116	359.934 Down
Cdh1	29220.6	0.59747	0.07313	8.17001	3.08E-16	1.84E-14	22530.2	23792.6	23442.4	34831.6	34689.4	36037.6 Up
Has3	335.742	0.78641	0.3215	2.44609	0.01444	0.0515	247.68	264.479	227.262	447.861	224.933	602.235 Up
Cog8	1590.05	0.29091	0.09401	3.09462	0.00197	0.00985	1494.79	1380.85	1415.14	1758.27	1793.13	1698.12 Up
Tmed6	75.9839	1.32859	0.41415	3.20798	0.00134	0.00712	64.8226	38.3303	26.7367	124.406	73.9218	127.686 Up
Terf2	2142.84	-0.219	0.08751	-2.503	0.01231	0.04534	2366.51	2212.62	2336.6	2002.94	1934.64	2003.76 Down
Cyb5b	17245.9	0.92417	0.09946	9.29183	1.52E-20	1.39E-18	12711	11556.6	11443.3	20962.4	24158.7	22643.6 Up
Nqo1	529.792	-1.4506	0.67191	-2.159	0.03085	0.09368	1508.33	451.34	367.63	362.851	209.093	279.501 Down
Nob1	1261.36	-0.4414	0.10953	-4.0301	5.57E-05	0.00045	1406.75	1377.98	1573.65	1041.9	1116.22	1051.65 Down
Zfp821	1249.01	-0.2939	0.10094	-2.9112	0.0036	0.01644	1339.99	1344.44	1442.83	1174.6	1099.32	1092.87 Down
Ap1g1	1081.08	0.55867	0.17607	3.17303	0.00151	0.00788	972.339	769.482	881.357	1079.22	1218.65	1565.41 Up
Phlpp2	1259.39	0.31306	0.15084	2.07544	0.03795	0.10998	1353.53	992.756	1023.63	1357.06	1429.86	1399.52 Up
Marveld3	1033.18	0.74969	0.12205	6.14259	8.12E-10	1.81E-08	841.726	750.317	719.982	1230.58	1263.01	1393.49 Up
Hydin	2881.32	-0.708	0.25284	-2.8001	0.00511	0.02211	3135.67	4170.34	3417.53	2239.31	1360.16	2964.93 Down
Vac14	2217.33	0.46165	0.12392	3.72553	0.00019	0.00136	2068.52	1839.86	1688.23	2534.77	2785.79	2386.82 Up
Mtss2	2563.37	-0.4175	0.14958	-2.7913	0.00525	0.02257	2778.66	2592.09	3424.21	2043.37	2484.83	2057.05 Down
Il34	1278.62	0.78134	0.12223	6.39221	1.64E-10	4.21E-09	1008.14	893.097	920.507	1492.87	1783.63	1573.45 Up
St3gal2	2423.74	-0.4237	0.11505	-3.6824	0.00023	0.00157	2574.52	2650.54	3106.23	2123.19	1970.54	2117.37 Down
Aars	5454.84	0.36775	0.15788	2.32925	0.01985	0.06641	3997.72	4464.53	5827.65	6419.34	6380.51	5639.29 Up
Clec18a	4.83283	-3.691	1.50452	-2.4533	0.01416	0.05073	7.74001	13.4156	5.7293	0	2.11205	0 Down
Glg1	3945.21	0.39626	0.1303	3.04119	0.00236	0.01144	3918.38	3192.92	3109.1	4101.25	4726.77	4622.83 Up
Rfwd3	1784.05	0.26224	0.10835	2.42028	0.01551	0.05448	1724.09	1553.34	1589.88	1883.71	1846.99	2106.31 Up
Mlkl	637.257	0.72462	0.12466	5.81272	6.15E-09	1.16E-07	498.263	459.964	483.171	785.831	854.325	741.986 Up
Ldhd	941.196	0.76387	0.19649	3.88747	0.0001	0.00077	717.886	849.975	525.186	1322.85	1180.64	1050.64 Up
Tmem170	1484.6	0.35094	0.11205	3.132	0.00174	0.00885	1333.22	1281.19	1300.55	1754.12	1492.16	1746.38 Up
Mon1b	533.997	0.41092	0.1284	3.20046	0.00137	0.00728	499.231	442.715	433.517	602.332	599.822	626.365 Up
Vat1l	7.89795	-3.3654	1.50565	-2.2352	0.0254	0.08069	5.80501	33.5391	3.81953	0	4.2241	0 Down
Maf	1476.81	-0.2834	0.12791	-2.216	0.02669	0.08388	1635.08	1794.82	1434.23	1306.26	1426.69	1263.79 Down
Dynlrb2	3185.42	-0.4431	0.17748	-2.4966	0.01254	0.04598	3248.87	3925.99	3837.67	3043.8	2034.96	3021.23 Down
Atmin	2267.83	0.29959	0.11356	2.63803	0.00834	0.03291	2214.61	1798.65	2086.42	2495.38	2474.27	2537.63 Up
Pkd1l2	43.3072	-0.915	0.42297	-2.1634	0.03051	0.09292	36.7651	68.9946	63.9772	26.9546	38.0169	25.135 Down
Cmip	1674.54	0.32373	0.15029	2.15406	0.03124	0.09449	1613.79	1447.93	1400.81	1571.66	1835.37	2177.7 Up
Plcg2	1418.76	0.98601	0.21753	4.5327	5.82E-06	6.06E-05	1190.99	964.966	699.929	1656.67	2296.86	1703.15 Up
Sdr42e1	391.143	0.36053	0.16603	2.17151	0.02989	0.09161	366.683	340.182	320.841	383.585	430.858	504.711 Up
Hsd17b2	87.5501	0.92249	0.32476	2.84055	0.0045	0.01988	54.1801	81.452	45.8344	101.598	103.49	138.745 Up
Mphosph6	1512.53	0.31966	0.11358	2.81449	0.00489	0.02129	1284.84	1330.06	1421.82	1619.35	1840.65	1578.48 Up
Cdh13	1998.8	-0.7227	0.23693	-3.0502	0.00229	0.01116	2474.87	2810.57	2181.91	1710.58	1860.72	954.125 Down
Necab2	48.9522	-1.7812	0.3686	-4.8324	1.35E-06	1.61E-05	64.8226	86.2433	76.3906	19.6976	27.4567	19.1026 Down
Dnaaf1	949.999	-0.9802	0.22131	-4.4292	9.46E-06	9.33E-05	1118.43	1419.18	1245.17	741.252	432.97	742.991 Down
Gm32352	24.7526	-1.525	0.70032	-2.1776	0.02944	0.0906	11.61	65.1616	33.4209	10.3672	16.8964	11.0594 Down
Wfdc1	1912.1	-0.4997	0.17409	-2.8707	0.0041	0.01834	1766.66	2485.72	2467.42	1701.25	1702.31	1349.25 Down
Atp2c2	1363.45	-0.6317	0.29802	-2.1196	0.03404	0.10101	1352.57	2008.51	1610.89	1111.36	589.262	1508.1 Down
Cotl1	3295.65	1.02382	0.17842	5.73809	9.58E-09	1.75E-07	2542.59	2224.12	1752.21	4121.98	5263.23	3869.79 Up
Galnt2l	3403.8	-0.4834	0.13557	-3.5654	0.00036	0.00235	3596.2	4013.19	4296.97	2446.65	2897.73	3172.04 Down
Ttc13	2486.36	0.22326	0.10883	2.05141	0.04023	0.11503	2453.58	2175.25	2254.48	2527.51	2595.71	2911.64 Up
Fam89a	385.642	-0.6609	0.19159	-3.4494	0.00056	0.00342	405.383	520.334	491.765	249.849	294.631	351.89 Down
Trim67	19.561	-2.3215	0.93406	-2.4854	0.01294	0.04718	10.6425	77.619	9.54883	8.29373	4.2241	7.03781 Down
Gnpat	7619.15	0.32607	0.11509	2.83307	0.00461	0.02026	6810.24	7532.87	5942.24	8231.52	8654.13	8543.9 Up
Exoc8	1546.7	0.27752	0.10046	2.76247	0.00574	0.0243	1432.87	1337.73	1424.69	1595.51	1712.87	1776.54 Up
Sipa1l2	3190.93	-0.3976	0.10744	-3.7003	0.00022	0.00148	3384.32	3605.93	3893.06	2605.27	2970.6	2686.43 Down
4930567H	75.1397	-1.326	0.37677	-3.5194	0.00043	0.00273	84.1726	122.657	115.541	53.9092	24.2886	50.27 Down
Ntpcr	907.328	0.57013	0.13696	4.16289	3.14E-05	0.00027	808.831	730.193	652.185	1031.53	1033.85	1187.38 Up
Kcnk1	307.452	0.53122	0.23415	2.26873	0.02328	0.07531	319.275	187.819	247.315	351.447	417.13	321.728 Up
Slc35f3	462.845	-0.3479	0.14102	-2.4674	0.01361	0.04918	507.938	500.211	547.148	375.291	397.066	449.414 Down
Gm31718	201.1	0.84619	0.21933	3.85804	0.00011	0.00085	150.93	164.82	115.541	222.894	281.959	270.453 Up
Pard3	2957.28	0.25943	0.11114	2.33435	0.01958	0.0657	2924.76	2479.02	2672.72	3026.17	3195.53	3445.51 Up
Nrp1	6577.63	0.39066	0.14081	2.77439	0.00553	0.02359	6420.34	4802.79	5854.39	6719.99	7593.88	8074.37 Up
Gm21399	1057.71	0.3676	0.11567	3.17792	0.00148	0.00777	1017.81	859.558	893.77	1209.85	1165.85	1199.44 Up

Gm32856	305.657	1.07098	0.16854	6.3545	2.09E-10	5.28E-09	205.11	173.445	212.939	390.842	399.178	452.43	Up
Alkbh8	916.25	-0.2532	0.11225	-2.2556	0.0241	0.07734	959.761	986.048	1043.69	821.079	794.131	892.796	Down
Gucy1a2	219.71	-1.0838	0.18608	-5.8245	5.73E-09	1.09E-07	269.933	334.432	291.239	140.993	149.956	131.708	Down
Kbtbd3	572.345	-0.3903	0.12566	-3.1059	0.0019	0.00954	631.778	624.785	691.335	523.542	470.987	491.641	Down
Casp1	1152.71	1.00498	0.15253	6.58862	4.44E-11	1.24E-09	878.491	780.023	641.681	1683.63	1440.42	1492.01	Up
Casp4	1802.57	0.76655	0.11108	6.90068	5.18E-12	1.67E-10	1343.86	1349.23	1311.05	2151.19	2140.56	2519.53	Up
Dync2h1	1949.96	-0.5953	0.17307	-3.4395	0.00058	0.00353	2412.95	2413.85	2213.42	1642.16	1178.52	1838.88	Down
Mmp13	206.621	3.23247	0.48003	6.73392	1.65E-11	5.01E-10	54.1801	30.6643	34.3758	447.861	545.965	126.681	Up
Mmp12	1120.63	3.39573	0.19003	17.8698	2.03E-71	7.29E-68	252.518	178.236	152.781	2167.77	1890.29	2082.19	Up
Mmp3	1120.19	-1.0341	0.31037	-3.3317	0.00086	0.00492	1624.43	1308.02	1583.2	795.161	1043.35	366.971	Down
Mmp8	512.009	2.72184	0.39477	6.89476	5.40E-12	1.74E-10	224.46	126.49	53.4734	996.284	951.479	719.867	Up
Tmem123	15766.1	0.31465	0.08718	3.60906	0.00031	0.00203	14506.7	13202.9	14451.2	16691.1	17901.7	17842.8	Up
Birc3	4170.62	0.94679	0.09434	10.0359	1.06E-23	1.27E-21	2823.17	2988.81	2735.74	5215.72	5427.97	5832.33	Up
Yap1	7116.75	-0.2654	0.07829	-3.3903	0.0007	0.00412	7783.55	7524.25	8000.96	6298.05	6635.01	6458.7	Down
Cfap300	882.666	-0.8182	0.24786	-3.3012	0.00096	0.00541	1104.89	1155.66	1119.12	670.755	396.01	849.564	Down
Gm19324	52.0058	1.91983	0.39321	4.88248	1.05E-06	1.29E-05	15.48	18.2069	31.5111	72.5701	90.8182	83.4483	Up
1700128F	3.45015	5.28747	1.74675	3.02704	0.00247	0.01192	0	0	0	6.2203	8.4482	6.03241	Up
Gm32014	484.773	-0.3042	0.15137	-2.0093	0.0445	0.12451	526.321	485.837	594.892	390.842	458.315	452.43	Down
Gm16485	74.7843	-0.8715	0.31978	-2.7253	0.00642	0.02662	114.165	100.617	75.4357	45.6155	47.5211	65.3511	Down
Arhgap42	1948.6	-0.5558	0.13875	-4.0056	6.19E-05	0.0005	2010.47	2308.45	2639.3	1470.06	1548.13	1715.21	Down
Sesn3	2214.14	0.27836	0.10481	2.65588	0.00791	0.03152	1961.13	2082.3	1960.37	2311.88	2316.92	2652.25	Up
Amotl1	3723.02	-0.2341	0.0961	-2.4361	0.01485	0.05264	3794.54	4024.69	4254	3233.52	3556.69	3474.67	Down
Piwil4	733.372	1.71611	0.19804	8.66557	4.49E-18	3.39E-16	378.293	341.14	307.472	1128.98	865.941	1378.4	Up
Fut4	1099.97	1.61392	0.21905	7.36771	1.74E-13	7.08E-12	684.991	496.378	444.021	1737.54	1269.34	1967.57	Up
1700012B	177.947	-3.4661	0.30867	-11.229	2.93E-29	5.50E-27	234.135	340.182	404.87	26.9546	25.3446	36.1944	Down
Izumo1r	38.678	-1.0592	0.48839	-2.1687	0.03011	0.09204	81.2701	39.2886	36.2855	18.6609	26.4006	30.162	Down
Panx1	494.13	0.32212	0.15777	2.04171	0.04118	0.1172	475.043	420.676	422.058	547.386	476.267	623.349	Up
Hephl1	5.4363	-4.8323	2.11495	-2.2848	0.02232	0.07272	0	28.7478	2.86465	0	0	1.0054	Down
Smco4	1355.6	-0.3972	0.1213	-3.2745	0.00106	0.00584	1587.67	1548.55	1486.75	1310.41	1140.51	1059.69	Down
Slc36a4	2544.42	0.31938	0.09558	3.34134	0.00083	0.00477	2368.44	2262.45	2160.9	2673.69	2954.76	2846.29	Up
Fat3	1004.4	-0.8813	0.16997	-5.1851	2.16E-07	3.03E-06	1036.19	1477.63	1392.22	713.261	639.951	767.121	Down
Gm48069	30.7328	-1.1731	0.45574	-2.5741	0.01005	0.03836	52.2451	30.6643	44.8795	16.5875	16.8964	23.1242	Down
Fbxl12os	198.158	-0.546	0.22344	-2.4436	0.01454	0.05177	237.038	193.568	275.006	187.646	148.9	146.789	Down
Rdh8	2.39334	4.76113	2.10286	2.26413	0.02357	0.07603	0	0	0	1.03672	5.28013	8.04321	Up
Shfl	1771.9	0.31514	0.11423	2.75873	0.0058	0.02454	1613.79	1423.01	1700.65	1854.68	2004.34	2034.93	Up
Ppan	968.977	-0.347	0.13336	-2.602	0.00927	0.03589	1073.93	1008.09	1172.6	880.172	924.022	755.056	Down
Eif3g	4283.57	-0.2316	0.10067	-2.3008	0.0214	0.0703	4642.07	4428.11	4809.75	4249.5	3928.41	3643.57	Down
S1pr2	3197.14	0.70554	0.17866	3.94904	7.85E-05	0.00061	2931.53	1949.1	2411.08	3840	4590.54	3460.59	Up
Mrpl4	2964.4	-0.3138	0.13192	-2.3786	0.01738	0.05967	3089.23	3463.15	3303.89	2889.33	2804.8	2236.01	Down
Icam1	29894.6	1.30685	0.12784	10.2223	1.58E-24	2.02E-22	19763.2	14645.1	17223.2	42533.3	43484	41719.1	Up
Icam5	19.9436	1.92217	0.58321	3.29586	0.00098	0.00549	11.61	7.66607	5.7293	36.2851	22.1765	36.1944	Up
Tyk2	22.8205	0.97901	0.46849	2.08972	0.03664	0.10711	15.48	13.4156	17.1879	27.9913	31.6808	31.1674	Up
Pde4a	1099.68	-0.4637	0.11957	-3.8782	0.00011	0.00079	1394.17	1170.99	1259.49	897.796	976.823	898.828	Down
Ap1m2	2644.61	0.36808	0.0936	3.93248	8.41E-05	0.00065	2196.23	2284.49	2446.41	3094.6	2905.13	2940.8	Up
Slc44a2	20560.9	0.39726	0.12177	3.26228	0.00111	0.00607	19043.3	16474.4	17725.5	23502.3	26032.1	20587.6	Up
Gm16853	29.6013	-1.5964	0.52146	-3.0614	0.0022	0.0108	49.3426	50.7877	33.4209	15.5507	7.39218	21.1134	Down
Qtrt1	619.293	-0.2701	0.11989	-2.253	0.02426	0.07773	653.063	666.948	711.388	553.606	538.573	592.181	Down
Dnm2	10084.9	0.44784	0.09385	4.77172	1.83E-06	2.13E-05	8829.42	8380.93	8385.78	11435	12569.9	10908.6	Up
Tmed1	854.497	-0.3005	0.11899	-2.5257	0.01155	0.04302	915.256	951.551	962.522	808.638	797.299	691.716	Down
Smarca4	6462.7	0.32028	0.07755	4.12991	3.63E-05	0.00031	5751.8	5665.23	5827.65	6975.02	7452.37	7104.16	Up
Ldlr	6537.05	1.50552	0.12701	11.8539	2.05E-32	4.81E-30	3641.68	3514.89	3059.44	9304.52	10904.5	8797.26	Up
Dock6	6909.58	-0.3331	0.10288	-3.2383	0.0012	0.00651	7266.9	7345.05	8499.41	5954.9	6368.89	6022.35	Down
Tmem205	3175.25	-0.3543	0.10763	-3.292	0.00099	0.00556	3892.26	3403.73	3393.65	2977.45	2716.1	2668.33	Down
Plppr2	633.707	-0.4351	0.15651	-2.7797	0.00544	0.02326	851.401	621.91	712.343	556.716	528.013	531.857	Down
Rgl3	1530.52	0.20665	0.09092	2.27289	0.02303	0.07463	1436.74	1367.44	1459.06	1643.19	1659.02	1617.69	Up
Ccdc151	1076.87	-0.6299	0.21171	-2.9754	0.00293	0.01377	1114.56	1419.18	1391.26	1051.23	608.271	876.71	Down
Prkcsh	5173.01	0.24779	0.08535	2.90305	0.0037	0.0168	4763.01	4553.65	4872.77	5797.32	5703.59	5347.73	Up
Elavl3	3.51332	-5.2096	1.89503	-2.7491	0.00598	0.02508	1.935	13.4156	5.7293	0	0	0	Down
Cnn1	1782.05	-2.4113	0.33687	-7.1579	8.19E-13	2.97E-11	1609.92	4446.32	2943.9	628.25	695.921	367.977	Down
Elof1	7948.49	-0.4902	0.14058	-3.4872	0.00049	0.00304	9093.55	9900.73	8864.18	7205.18	5400.51	7226.82	Down
Pigyl	1369.83	0.35132	0.13807	2.54452	0.01094	0.04117	1241.3	1116.37	1253.76	1737.54	1542.85	1327.13	Up
Gm18227	13.8013	1.6392	0.70009	2.34143	0.01921	0.06467	2.9025	9.58259	7.63906	19.6976	15.8404	27.1458	Up
Zfp872	210.998	1.28808	0.22796	5.65052	1.60E-08	2.78E-07	119.003	122.657	126.045	305.831	362.217	230.237	Up
1810064F	894.007	1.28368	0.13902	9.23408	2.61E-20	2.36E-18	565.021	483.921	512.772	1376.76	1311.58	1113.98	Up
Zfp809	6019.89	0.67373	0.12924	5.21314	1.86E-07	2.65E-06	4617.88	4495.19	4804.97	7198.95	6433.31	8569.03	Up
Zfp810	1179.91	0.21771	0.0998	2.18138	0.02916	0.08996	1075.86	1141.29	1056.1	1249.24	1241.89	1315.06	Up
Bmper	1246.67	-0.6896	0.18615	-3.7047	0.00021	0.00146	1153.26	1773.74	1690.14	896.759	1060.25	905.866	Down
Tbx20	340.37	-5.8168	1.40052	-4.1533	3.28E-05	0.00028	342.496	1298.44	365.72	12.4406	0	23.1242	Down
B3gat1	11.3568	-2.0213	0.77214	-2.6177	0.00885	0.03452	10.6425	21.0817	22.9172	4.14686	6.33615	3.0162	Down
Glb1l2	2425.48	-0.7145	0.21706	-3.2917	0.001	0.00556	2425.53	3684.5	2932.45	2141.85	1330.59	2037.95	Down
Glb1l3	162.54	-0.5942	0.19739	-3.0101	0.00261	0.01251	204.143	186.86	195.751	128.553	116.163	143.772	Down
Vps26b	2274.1	0.23109	0.09287	2.48825	0.01284	0.04688	2179.78	2123.5	1973.74	2498.49	2494.33	2374.76	Up
Jam3	828.133	-1.083	0.16302	-6.6432	3.07E-11	8.85E-10	1040.06	1327.19	1008.36	534.945	473.099	585.143	Down
Snx19	3330.55	0.26716	0.08457	3.15919	0.00158	0.00819	3123.09	3001.27	2944.86	3495.81	3700.31	3717.97	Up
Gm31013	6.89067	-5.1828	1.63888	-3.1624	0.00156	0.00811	7.74001	28.7478	3.81953	1.03672	0	0	Down
Adamts15	3495.72	-0.7425	0.26638	-2.7873	0.00531	0.0228	2460.36	5505.2	5162.1	2525.44	2991.72	2329.51	Down
Zbtb44	2880.15	-0.2329	0.09569	-2.4343	0.01492	0.05284	3146.31	2943.77	3246.6	2540.99	2605.21	2798.03	Down
St14	5413.25	0.91962	0.08064	11.4044	3.97E-30	7.94E-28	3590.4	3773.62	3868.23	7239.39	6954.98	7052.89	Up
Barx2	513.716	-0.591	0.15965	-3.7018	0.00021	0.00147	550.508	705.278	596.802	413.65	370.665	445.393	Down
Gm3756	281.35	-0.3753	0.16808	-2.2328	0.02556	0.08106	306.698	287.478	359.036	255.032	236.55	243.307	Down
Arhgap32	1836.97	-0.3482	0.11836	-2.9416	0.00327	0.01513	1860.51	2176.21	2136.07	1515.68	1581.93	1751.41	Down
Kcnj5	233.531	-4.4196	0.49947	-8.8485	8.87E-19	7.07E-17	298.958	794.396	245.405	14.514	13.7283	34.1836	Down
Gm10698	631.239	0.34322	0.16306	2.10483	0.03531	0.10397	645.323	464.755	559.561	695.636	661.072	761.088	Up

St3gal4	3689.43	0.40991	0.09886	4.14656	3.38E-05	0.00029	3121.16	3291.62	3093.82	4022.46	4032.96	4574.57	Up
Tirap	1226.47	0.42332	0.12673	3.34023	0.00084	0.00479	1135.85	976.466	1031.27	1268.94	1415.07	1531.23	Up
Srpr	8859.89	0.2762	0.09321	2.96308	0.00305	0.01428	8290.52	7776.27	7976.14	10097.6	10079.8	8939.02	Up
Stt3a	9371.63	0.27778	0.07246	3.83345	0.00013	0.00093	8664.94	8461.42	8290.29	10227.2	10157.9	10428	Up
Ei24	1860.4	0.39684	0.14469	2.74273	0.00609	0.02548	1875.02	1499.67	1443.78	1895.12	2241.94	2206.85	Up
Fez1	62.4548	-1.0099	0.40326	-2.5044	0.01227	0.0452	64.8226	114.991	70.6613	42.5053	27.4567	54.2916	Down
Tmem218	567.864	-0.4613	0.15332	-3.0088	0.00262	0.01255	691.764	706.237	575.794	514.211	424.522	494.657	Down
Slc37a2	508.107	1.11648	0.31926	3.49705	0.00047	0.00294	405.383	307.601	249.224	625.14	1037.02	424.279	Up
Hepacam	189.204	-0.8089	0.23878	-3.3876	0.00071	0.00415	303.795	208.9	210.074	151.361	119.331	141.762	Down
Robo4	3647.55	-0.5148	0.14998	-3.4324	0.0006	0.00361	4130.26	3841.66	4902.37	2716.2	3501.78	2793	Down
Robo3	15.3526	-1.3591	0.60819	-2.2347	0.02544	0.08078	23.22	22.0399	21.0074	5.18358	11.6163	9.04861	Down
Msantd2	2593.23	-0.5497	0.12962	-4.2412	2.22E-05	0.0002	2747.7	3176.63	3320.13	2063.06	1909.29	2342.58	Down
Esam	13332.3	-0.2929	0.13138	-2.2297	0.02577	0.08156	13465.7	13436.7	17141.1	11740.8	12883.5	11325.8	Down
Vsig2	2203.8	0.43908	0.13639	3.21927	0.00129	0.00689	1878.89	1718.16	2015.76	2560.69	2856.55	2192.78	Up
Nrgn	1144.21	-1.2348	0.21682	-5.6949	1.23E-08	2.19E-07	1115.53	1711.45	1990.93	665.572	762.45	619.327	Down
Vwa5a	6505.29	0.44671	0.08798	5.07726	3.83E-07	5.16E-06	5889.18	5270.42	5358.8	7353.43	7643.51	7516.38	Up
Olfr920	303.091	0.34697	0.17435	1.99005	0.04659	0.12911	234.135	298.018	268.322	305.831	344.264	367.977	Up
Scn3b	3684.35	0.4962	0.16517	3.00411	0.00266	0.01271	3721.01	2835.49	2614.47	3901.16	4140.68	4893.29	Up
Ubash3b	573.687	0.54632	0.13335	4.09681	4.19E-05	0.00035	457.628	492.545	448.795	625.14	742.386	675.629	Up
Mir100hg	32.213	-1.7849	0.52384	-3.4073	0.00066	0.00391	42.5701	70.9111	36.2855	20.7343	13.7283	9.04861	Down
Sc5d	4546.85	0.60549	0.1154	5.24705	1.55E-07	2.22E-06	3432.7	3978.69	3407.98	5008.37	5848.27	5605.11	Up
Grik4	83.2255	1.24878	0.34119	3.66009	0.00025	0.0017	45.4726	54.6207	47.7441	92.2677	164.74	94.5077	Up
Arhgef12	9948.18	-0.3714	0.10922	-3.4005	0.00067	0.00399	10633.8	10607	12424	8391.18	9299.36	8333.77	Down
D630033C	4.95901	2.75688	1.28856	2.13951	0.03239	0.0971	0	1.91652	1.90977	14.514	7.39218	4.0216	Up
Trim29	1012.1	2.51262	0.24477	10.2654	1.01E-24	1.31E-22	375.391	311.434	218.668	1633.86	2208.15	1325.12	Up
Nectin1	2445.37	0.95589	0.14699	6.50328	7.86E-11	2.11E-09	1500.59	1633.83	1856.29	3215.89	3688.7	2776.92	Up
Thy1	882.862	-0.4601	0.15456	-2.9765	0.00292	0.01373	939.444	993.714	1134.4	732.958	642.063	854.591	Down
Gm10687	486.156	-0.7728	0.26992	-2.8632	0.00419	0.01871	391.838	686.113	761.997	288.207	430.858	357.923	Down
Mcam	11670.7	-0.4176	0.19431	-2.1489	0.03164	0.09546	10034.9	12741	17268.1	8876.36	10585.6	10518.5	Down
Cbl	3385.92	0.24743	0.09726	2.54402	0.01096	0.0412	3203.4	2942.81	3142.52	3489.59	3936.86	3600.34	Up
Ccdc153	9076.93	-0.6577	0.20013	-3.2862	0.00102	0.00565	9930.44	12086.5	11315.4	7918.44	4884.12	8326.73	Down
Nlrx1	769.571	0.8261	0.19291	4.28232	1.85E-05	0.00017	543.736	629.576	491.765	886.392	1234.49	831.467	Up
Hinfp	980.257	0.3937	0.12137	3.24377	0.00118	0.00641	905.581	794.396	842.207	1109.29	1029.62	1200.45	Up
C2cd2l	3092.8	0.63612	0.08985	7.07981	1.44E-12	5.08E-11	2539.69	2428.23	2297.45	3874.21	3761.56	3655.64	Up
Dpagt1	1307.46	0.22608	0.11205	2.0177	0.04362	0.12266	1200.67	1100.08	1314.87	1418.23	1422.47	1388.46	Up
Hmbs	1408.52	0.55649	0.097	5.73695	9.64E-09	1.76E-07	1155.2	1165.24	1100.03	1691.92	1737.16	1601.6	Up
Hyou1	7299.51	0.40162	0.12994	3.09072	0.002	0.00996	5602.8	5948.87	7318.22	8830.75	8136.68	7959.76	Up
Upk2	64.5399	-0.8887	0.35616	-2.4952	0.01259	0.04615	73.5301	107.325	70.6613	47.6889	32.7368	55.297	Down
Phldb1	3257.25	-0.3753	0.10201	-3.6785	0.00023	0.00159	3655.22	3478.48	3901.65	2699.61	3048.75	2759.83	Down
Arcn1	11780.7	0.23081	0.07177	3.2158	0.0013	0.00697	11012.1	10629	10879.9	12999.4	12556.1	12607.7	Up
Kmt2a	4086.7	-0.2127	0.09811	-2.1679	0.03016	0.09217	4476.63	4138.72	4547.15	3696.93	3598.93	4061.82	Down
Cd3d	260.078	-0.4367	0.16575	-2.6345	0.00843	0.03317	279.608	295.144	322.75	220.82	204.869	237.275	Down
Mpzl3	545.369	0.73406	0.1504	4.88063	1.06E-06	1.30E-05	471.173	355.514	402.006	664.535	683.248	695.737	Up
Jaml	179.101	0.5331	0.22551	2.36399	0.01808	0.06165	179.955	119.782	139.413	227.041	214.373	194.042	Up
Scn4b	203.838	-4.9724	1.03871	-4.7871	1.69E-06	1.99E-05	249.615	789.605	146.097	13.4773	2.11205	22.1188	Down
Gm39327	22.5952	1.76911	0.67092	2.63683	0.00837	0.033	9.67501	17.2487	3.81953	24.8812	52.8013	27.1458	Up
Tmprss4	1363.3	1.93034	0.16667	11.5819	5.09E-31	1.10E-28	709.179	494.461	496.539	2056.84	2223.99	2198.81	Up
Il10ra	1230	0.48746	0.16329	2.98515	0.00283	0.01341	1252.91	974.549	845.071	1406.82	1468.93	1431.69	Up
Tmprss13	164.781	-1.0815	0.4487	-2.4102	0.01594	0.05577	145.125	334.432	191.931	144.104	45.4091	127.686	Down
Fxyd6	1300.69	-0.5283	0.21154	-2.4974	0.01251	0.04589	1791.81	1548.55	1268.08	952.742	1382.34	860.623	Down
4833428L	598.391	2.83204	0.72935	3.88299	0.0001	0.00078	67.7251	292.269	82.1199	1280.34	605.103	1262.78	Up
Dscaml1	113.355	2.69224	0.40592	6.63252	3.30E-11	9.44E-10	19.35	44.0799	27.6916	266.436	122.499	200.075	Up
Tagln	195.149	-0.8808	0.30656	-2.8731	0.00406	0.01823	167.378	342.098	249.224	130.626	168.964	112.605	Down
Sidt2	6422.82	0.40188	0.11793	3.40771	0.00066	0.0039	6096.23	5138.18	5367.4	7176.15	8025.79	6733.17	Up
Zpr1	1893.16	-0.2676	0.10059	-2.6599	0.00782	0.03119	2010.47	1972.1	2222.01	1652.53	1762.51	1739.34	Down
Gm31557	9.49976	2.13793	0.88099	2.42674	0.01524	0.05373	2.9025	1.91652	5.7293	24.8812	9.50423	12.0648	Up
Gm5617	286.991	-0.4285	0.18323	-2.3388	0.01935	0.06505	297.023	377.554	313.202	237.408	274.567	222.194	Down
Zbtb16	1551.04	-1.3923	0.3038	-4.5829	4.59E-06	4.89E-05	1584.77	2420.56	2733.83	670.755	590.318	1306.02	Down
Usp28	954.069	-0.3914	0.13211	-2.9627	0.00305	0.01428	1051.67	1190.16	1006.45	779.61	784.627	911.899	Down
Ttc12	2274.34	-0.3972	0.17549	-2.2634	0.02361	0.07614	2377.15	2887.23	2492.24	2176.07	1497.44	2215.9	Down
Ncam1	121.382	-0.7325	0.31522	-2.3238	0.02014	0.06711	99.6527	202.193	152.781	103.672	85.5381	84.4537	Down
Plet1os	1311.23	1.73572	0.261	6.65018	2.93E-11	8.52E-10	680.154	660.24	476.487	1966.65	1320.03	2763.85	Up
Il18	1309.1	1.63895	0.2502	6.55065	5.73E-11	1.58E-09	897.841	550.999	460.254	1954.21	2346.49	1644.84	Up
Timm8b	916.647	0.26132	0.10409	2.51038	0.01206	0.04454	854.304	857.641	789.688	997.321	989.496	1011.43	Up
Pih1d2	1246.73	-0.4243	0.19069	-2.225	0.02608	0.08236	1339.02	1555.25	1392.22	1096.85	792.019	1305.01	Down
Dlat	3759.23	-0.3502	0.16847	-2.0785	0.03766	0.10937	3900	5312.59	3427.07	3327.86	3282.13	3305.76	Down
Dixdc1	2666.23	-0.3773	0.14352	-2.6288	0.00857	0.03365	3400.77	2850.82	2787.3	2400	1992.72	2565.78	Down
2310030G	2211.4	0.22843	0.08505	2.6858	0.00724	0.02929	2068.52	2064.09	1977.56	2426.95	2325.37	2405.92	Up
Hspb2	95.0751	-0.7116	0.33379	-2.132	0.033	0.09858	90.9451	161.946	101.218	63.2397	89.7622	63.3403	Down
Cryab	13404.5	-0.6983	0.13454	-5.1908	2.09E-07	2.95E-06	14655.7	19284	15820.5	10563.1	10788.4	9315.04	Down
Alg9	1810.68	0.47105	0.11616	4.05531	5.01E-05	0.00041	1534.46	1376.06	1642.4	2177.1	2178.58	1955.5	Up
Gm32742	364.21	1.40744	0.25515	5.51608	3.47E-08	5.64E-07	187.695	270.229	140.368	597.148	450.923	538.895	Up
Arhgap20	38.7921	1.56882	0.45685	3.434	0.00059	0.00359	29.025	15.3321	14.3232	57.0194	71.8097	45.243	Up
Fdx1	3658.67	-0.2818	0.13306	-2.1176	0.03421	0.10141	4212.5	3794.7	4037.24	3846.22	2910.41	3150.93	Down
Zc3h12c	797.08	0.37379	0.13417	2.78589	0.00534	0.02288	742.074	721.569	619.719	896.759	832.148	970.212	Up
Acat1	6222.77	-0.6952	0.15774	-4.4071	1.05E-05	0.0001	7140.16	9533.72	6407.26	4826.95	4675.02	4753.54	Down
Sln	2474.48	-6.1068	1.39971	-4.3629	1.28E-05	0.00012	2940.24	8907.01	2787.3	35.2483	2.11205	174.94	Down
Tnfaip8l3	538.923	0.56466	0.23256	2.428	0.01518	0.05359	569.858	364.138	370.495	550.496	590.318	788.234	Up
Gldn	3.99428	-5.3945	2.5016	-2.1564	0.03105	0.09412	0.9675	22.9982	0	0	0	0	Down
Dmxl2	2656.41	0.76582	0.12258	6.24771	4.17E-10	9.82E-09	2036.59	1972.1	1893.53	3223.15	3805.92	3007.15	Up
Sh2d7	3.93966	4.4575	1.85782	2.39932	0.01643	0.0571	0.9675	0	0	16.5875	1.05603	5.027	Up
Idh3a	5649	-0.4425	0.19031	-2.3253	0.02005	0.06696	5611.51	8662.66	5251.86	4746.09	4743.67	4878.21	Down
Rab7-ps1	287.619	0.40279	0.14667	2.74622	0.00603	0.02526	250.583	243.398	249.224	315.162	330.536	336.809	Up

Ireb2	4939.42	0.2243	0.09543	2.35042	0.01875	0.06354	4467.92	4519.15	4681.79	5053.99	5138.62	5775.02	Up
Hykk	613.777	-0.6019	0.22528	-2.6715	0.00755	0.03034	858.174	616.16	745.764	572.267	346.376	543.922	Down
Chrnb4	48.3616	2.55195	0.44091	5.78796	7.12E-09	1.33E-07	13.545	18.2069	10.5037	88.1208	103.49	56.3024	Up
Tmem266	37.2768	-0.9389	0.42451	-2.2117	0.02699	0.08458	48.3751	55.579	42.9697	32.1382	28.5127	16.0864	Down
Etfa	6203.19	-0.642	0.15465	-4.1509	3.31E-05	0.00028	6907.96	9296.07	6478.88	5133.82	4675.02	4727.39	Down
Isl2	7.45487	-1.9411	0.89072	-2.1792	0.02931	0.09031	10.6425	13.4156	11.4586	2.07343	2.11205	5.027	Down
Gm7435	115.244	-0.9255	0.24507	-3.7764	0.00016	0.00114	130.613	153.321	169.014	68.4232	87.6501	82.4429	Down
Pstpip1	647.047	0.38951	0.11623	3.3511	0.0008	0.00464	592.111	538.541	550.013	753.692	737.106	710.818	Up
Tspan3	4983.78	-0.5705	0.14402	-3.9615	7.45E-05	0.00059	5422.85	7057.57	5389.36	4276.45	3623.22	4133.2	Down
Snx33	2047.07	-0.2103	0.0958	-2.1953	0.02814	0.08755	2213.64	2128.29	2245.88	1846.39	2024.4	1823.8	Down
Ptpn9	545.109	0.55988	0.17231	3.24925	0.00116	0.0063	512.776	411.093	398.186	562.937	654.736	730.926	Up
Scamp5	764.054	1.57635	0.20953	7.52333	5.34E-14	2.37E-12	354.106	453.256	343.758	1032.57	1462.6	938.039	Up
Scamp2	4611.78	0.57583	0.09879	5.82902	5.58E-09	1.07E-07	3938.7	3617.43	3554.07	5629.37	5796.52	5134.58	Up
Cyp1a1	2785.81	-3.7702	0.90628	-4.1601	3.18E-05	0.00028	10659	1556.21	3358.32	377.365	165.796	598.214	Down
Edc3	1896.76	-0.3177	0.14439	-2.2004	0.02778	0.08662	1866.31	1980.72	2467.42	1624.53	1595.65	1845.92	Down
Sema7a	6182.48	-0.7021	0.11265	-6.2325	4.59E-10	1.07E-08	7208.85	7757.1	8007.65	5019.78	4164.96	4936.52	Down
Ccdc33	1632.88	-0.6067	0.22763	-2.6653	0.00769	0.03079	1531.55	2542.26	1840.06	1404.75	971.543	1507.1	Down
Stra6	34.0556	-1.6199	0.68142	-2.3773	0.01744	0.05985	17.415	75.7024	61.1125	9.33044	11.6163	29.1566	Down
Islr	614.993	-1.1724	0.14801	-7.9209	2.36E-15	1.24E-13	804.961	938.135	812.605	405.356	398.122	330.777	Down
Insyn1	615.894	0.60891	0.18943	3.21443	0.00131	0.00699	468.271	603.703	391.502	668.682	771.955	791.25	Up
Cd276	573.218	1.20725	0.1548	7.79897	6.24E-15	3.12E-13	381.196	336.349	321.796	749.546	913.462	736.959	Up
Neo1	3416.92	-0.3073	0.09877	-3.1114	0.00186	0.00939	3867.1	3814.83	3656.25	3206.56	3162.8	2794.01	Down
Hexa	8383.9	0.85852	0.17449	4.92027	8.64E-07	1.09E-05	7273.68	5649.89	4957.75	11077.3	12229.8	9114.96	Up
Gm20275	33.5508	1.28333	0.57766	2.22162	0.02631	0.08297	26.1225	9.58259	22.9172	76.717	33.7928	32.1728	Up
9230112J1	52.124	-0.8059	0.34727	-2.3207	0.0203	0.0675	80.3026	55.579	63.0223	40.4319	42.241	31.1674	Down
Gm17853	11.2409	-1.4013	0.71215	-1.9677	0.04911	0.13473	14.5125	16.2904	18.1428	8.29373	3.16808	7.03781	Down
Larp6	169.202	-0.7756	0.2192	-3.5385	0.0004	0.00257	239.94	207.942	192.886	140.993	129.891	103.556	Down
Rplp1	22476.8	-0.3059	0.1415	-2.1618	0.03063	0.09316	24217.5	22740.4	27594.2	21861.2	21685.5	16762	Down
Kif23	551.245	1.1235	0.22338	5.02946	4.92E-07	6.48E-06	385.066	400.552	254.954	884.319	611.439	771.142	Up
Itga11	392.63	-0.6429	0.27406	-2.3459	0.01898	0.06412	380.228	527.042	529.005	286.134	209.093	424.279	Down
Fem1b	4870.65	0.38631	0.09219	4.19012	2.79E-05	0.00024	4092.53	4012.23	4562.43	5472.82	5565.25	5518.65	Up
Calml4	1631.62	-0.5719	0.21555	-2.6531	0.00798	0.03173	1781.17	2141.71	1929.82	1492.87	880.725	1563.4	Down
2300009A	344.125	-0.4621	0.1876	-2.4631	0.01377	0.04966	416.026	355.514	424.923	245.702	277.735	344.852	Down
Iqch	21.5779	-1.2552	0.5795	-2.166	0.03031	0.09245	33.8626	22.9982	34.3758	20.7343	8.4482	9.04861	Down
Smad3	2826.94	-0.2392	0.09499	-2.5186	0.01178	0.04372	3057.3	3027.14	3097.64	2563.8	2783.68	2432.06	Down
Gm18541	123.91	0.57366	0.23585	2.43231	0.015	0.05306	107.393	105.408	85.9395	160.691	157.348	126.681	Up
Gm36033	179.01	-0.8855	0.23684	-3.7386	0.00019	0.00131	215.753	203.151	277.871	150.324	120.387	106.572	Down
Smad6	6728.91	-1.1947	0.16356	-7.3044	2.78E-13	1.09E-11	8257.63	9680.33	10160	4167.6	4832.37	3275.6	Down
Scarletltr	22.7301	-1.198	0.57894	-2.0694	0.03851	0.11126	24.1875	28.7478	42.0148	21.771	11.6163	8.04321	Down
Lctl	66.9157	-9.4608	1.73039	-5.4674	4.57E-08	7.29E-07	110.295	259.688	31.5111	0	0	0	Down
Map2k1	5322.17	0.5925	0.09395	6.30685	2.85E-10	6.98E-09	4397.29	4229.75	4106	6202.67	6889.51	6107.81	Up
Hacd3	9652.37	0.63195	0.10502	6.01728	1.77E-09	3.71E-08	8299.23	6885.09	7530.21	11521	12278.4	11400.2	Up
Dpp8	399.731	0.71188	0.18439	3.86064	0.00011	0.00085	311.535	265.438	332.299	417.796	494.22	577.1	Up
Gm47350	4360.99	-0.2803	0.1021	-2.7454	0.00604	0.02531	4656.58	4635.1	5058.01	3738.4	4287.46	3790.36	Down
Cilp	179.383	-1.3771	0.4552	-3.0253	0.00248	0.01198	163.508	405.343	208.164	140.993	114.051	44.2376	Down
Clpx	3516.67	-0.2565	0.10383	-2.4708	0.01348	0.04877	3507.19	4171.3	3807.12	3157.84	3143.79	3312.8	Down
Pdcd7	1123.16	-0.2887	0.09528	-3.0304	0.00244	0.0118	1259.69	1208.36	1237.53	1026.35	990.552	1016.46	Down
Rasl12	3612.65	-0.4019	0.14882	-2.7008	0.00692	0.02826	3528.48	4201.96	4607.31	2993	3567.25	2777.92	Down
Slc51b	80.1716	-0.5636	0.28625	-1.969	0.04895	0.13435	77.4001	102.534	106.947	55.9827	71.8097	66.3565	Down
Spg21	5857.63	0.77392	0.1098	7.04829	1.81E-12	6.26E-11	4737.85	4166.51	4064.94	7707.98	7670.97	6797.52	Up
Rbpms2	432.676	-1.0043	0.24967	-4.0222	5.76E-05	0.00047	455.693	792.48	484.126	266.436	337.928	259.393	Down
Zfp609	1968.29	0.24865	0.11764	2.11377	0.03453	0.1022	1858.57	1635.75	1903.08	1970.8	2133.17	2308.4	Up
Csnk1g1	1431.37	0.31276	0.12831	2.43757	0.01479	0.05247	1397.07	1193.03	1240.39	1413.04	1713.93	1630.76	Up
Ppib	4472.82	0.27559	0.09362	2.94381	0.00324	0.01503	4135.1	3793.75	4211.99	5099.61	4654.96	4941.55	Up
Snx1	7116.71	0.60169	0.12266	4.90516	9.34E-07	1.16E-05	5944.33	5280.01	5736.94	8331.05	9740.78	7667.19	Up
Ciao2a	3142.89	0.62665	0.0944	6.63811	3.18E-11	9.13E-10	2510.67	2525.01	2376.7	3873.17	4007.62	3564.15	Up
Fbxl22	298.728	-1.3452	0.43506	-3.0921	0.00199	0.00992	214.785	793.438	277.871	179.352	192.197	134.724	Down
Usp3	2917.66	0.5867	0.10308	5.69163	1.26E-08	2.23E-07	2548.4	2196.33	2252.57	3483.37	3640.12	3385.18	Up
Aph1c	909.554	0.34613	0.15074	2.29622	0.02166	0.071	938.476	734.984	729.531	1102.03	939.863	1012.44	Up
Rab8b	3740.24	0.29137	0.13761	2.11741	0.03422	0.10143	3703.6	3141.17	3246.6	3673.08	4769.01	3907.99	Up
Lactb	1107.52	0.2934	0.1081	2.71407	0.00665	0.0273	1040.06	1013.84	931.966	1162.16	1272.51	1224.58	Up
Tpm1	32339.6	-0.6561	0.23751	-2.7625	0.00574	0.0243	29615.2	58069.5	31023.2	24880.1	26081.7	24367.9	Down
Gm19299	305.167	-0.6134	0.22558	-2.7194	0.00654	0.02698	384.098	403.427	319.886	277.84	175.3	270.453	Down
Vps13c	1076.36	0.576	0.14808	3.88975	0.0001	0.00076	990.722	784.814	817.38	1150.75	1424.58	1289.93	Up
4930502A	60.0956	0.87987	0.42892	2.05134	0.04023	0.11504	66.7576	26.8312	33.4209	69.46	67.5856	96.5185	Up
Anxa2	42899.2	0.47517	0.11316	4.19919	2.68E-05	0.00024	34824.2	34863.4	38005.3	50925.6	54743.3	44033.5	Up
B230323A	113.753	8.46754	1.15699	7.31857	2.51E-13	9.97E-12	1.935	0	0	206.306	392.841	81.4375	Up
Gtf2a2	2960.02	-0.2657	0.09122	-2.9127	0.00358	0.01638	3043.76	3282.04	3369.78	2653.99	2631.62	2778.93	Down
Fam81a	919.202	-1.0223	0.18486	-5.5303	3.20E-08	5.26E-07	1229.69	1324.31	1142.04	591.965	474.155	753.045	Down
Myo1e	4521.43	0.42919	0.14191	3.0244	0.00249	0.01201	3259.51	3791.83	4509.91	5003.19	5566.31	4997.85	Up
Ccnb2	230.757	0.67255	0.169	3.97958	6.90E-05	0.00055	173.183	186.86	173.789	291.317	259.782	299.609	Up
Rnf111	2765.31	0.27556	0.0853	3.23036	0.00124	0.00667	2515.5	2555.68	2434.95	2914.21	3048.75	3122.78	Up
Adam10	7276.5	0.64264	0.10241	6.27512	3.49E-10	8.35E-09	6098.16	5311.63	5636.67	8242.93	9352.16	9017.44	Up
Lipc	55.6049	1.26598	0.35152	3.60142	0.00032	0.00208	34.8301	36.4138	26.7367	98.488	71.8097	65.3511	Up
Aqp9	212.123	1.47855	0.2401	6.15819	7.36E-10	1.66E-08	104.49	136.073	95.4883	278.877	383.337	274.474	Up
Myzap	8761.92	-0.8072	0.1	-8.0727	6.87E-16	3.91E-14	10439.3	11492.4	11521.6	6224.44	6888.45	6005.26	Down
4930509E	30.997	-1.1614	0.5424	-2.1411	0.03226	0.09679	32.895	57.4955	38.1953	22.8077	8.4482	26.1404	Down
Pigb	319.751	0.52607	0.15527	3.38817	0.0007	0.00415	240.908	282.686	262.593	398.099	383.337	350.885	Up
Pigbos1	252.971	-0.3873	0.182	-2.1283	0.03331	0.09926	267.03	275.978	317.021	229.114	238.662	190.021	Down
Rab27a	5913.04	1.26098	0.16591	7.60052	2.95E-14	1.36E-12	2996.35	3706.54	3742.19	7888.37	10123.1	7021.72	Up
Rsl24d1	1673.08	-0.2902	0.13166	-2.204	0.02752	0.08593	1647.65	1788.11	2086.42	1417.19	1624.17	1474.92	Down
Wdr72	350.292	-1.3231	0.42101	-3.1426	0.00167	0.00858	307.665	679.405	514.682	176.242	93.9863	329.771	Down
Fam214a	3361.86	0.30967	0.13472	2.2986	0.02153	0.07062	2939.27	3448.77	2619.24	4034.9	3601.05	3527.95	Up

Myo5a	1889	1.17077	0.17497	6.69121	2.21E-11	6.56E-10	1356.44	1105.83	1023.63	2421.77	3170.19	2256.12	Up
Myo5c	7863.81	0.35497	0.11995	2.95922	0.00308	0.01442	6058.49	7107.4	7537.85	9360.51	8172.58	8946.06	Up
Gnb5	911.855	-0.4063	0.1935	-2.0999	0.03574	0.10498	819.474	1233.28	1065.65	730.885	694.865	926.98	Down
Mapk6	3298.06	0.5978	0.11059	5.40554	6.46E-08	9.96E-07	2459.39	2794.28	2619.24	3864.88	4348.71	3701.89	Up
Tmod2	2686.19	-0.4661	0.17904	-2.6032	0.00924	0.03578	2385.86	3298.33	3664.84	1969.76	2444.7	2353.64	Down
Lysmd2	1976.95	-0.5065	0.14598	-3.4695	0.00052	0.00321	2172.04	2257.66	2531.39	1444.15	1927.25	1529.21	Down
Scg3	51.3327	-1.1113	0.38975	-2.8512	0.00436	0.0193	83.2051	70.9111	56.3381	30.0648	44.3531	23.1242	Down
Bmp5	1356.22	0.73733	0.12646	5.83039	5.53E-09	1.06E-07	1065.22	991.798	994.033	1475.25	1816.36	1794.64	Up
Gm26903	81.2249	1.0036	0.35748	2.80741	0.00499	0.02169	45.4726	68.9946	47.7441	133.736	70.7537	120.648	Up
Hmgcll1	1481.26	0.28365	0.12076	2.34895	0.01883	0.06373	1208.41	1431.64	1368.35	1691.92	1491.11	1696.11	Up
Mlip	250.69	-5.1938	0.82898	-6.2653	3.72E-10	8.85E-09	269.933	996.589	197.661	11.4039	8.4482	20.108	Down
Klhl31	231.687	-4.9292	0.82558	-5.9706	2.36E-09	4.85E-08	156.735	929.511	259.728	13.4773	10.5603	20.108	Down
Gclc	36013.9	1.44885	0.13949	10.3867	2.85E-25	3.93E-23	21075.1	17662.6	19195.1	45244.4	51775.9	61130.4	Up
Elovl5	17317.5	0.58335	0.10751	5.4261	5.76E-08	9.01E-07	13675.6	12475.6	15438.5	20969.7	20351.7	20993.8	Up
Fbxo9	4256.2	0.25934	0.09279	2.79502	0.00519	0.02237	4123.49	3748.71	3751.73	4591.61	4856.66	4464.99	Up
Cilk1	4759.58	0.35189	0.08886	3.9599	7.50E-05	0.00059	3961.92	4172.26	4411.56	5327.68	5508.23	5175.8	Up
Gsta1	15.7432	-1.8436	0.71882	-2.5648	0.01032	0.03925	17.415	41.2051	15.2781	8.29373	4.2241	8.04321	Down
Gm10639	6.16049	-2.3119	0.99404	-2.3257	0.02003	0.06692	13.545	9.58259	7.63906	2.07343	2.11205	2.0108	Down
Gsta2	341.737	-2.9782	0.45017	-6.6158	3.69E-11	1.05E-09	952.021	438.882	428.742	105.745	28.5127	96.5185	Down
Dppa5a	13.4775	-1.9579	0.68333	-2.8652	0.00417	0.01861	19.35	16.2904	28.6465	6.2203	6.33615	4.0216	Down
Cgas	190.169	0.42666	0.19167	2.22598	0.02602	0.08219	165.443	165.779	155.646	213.563	193.253	247.329	Up
Slc17a5	1992.32	0.46962	0.12373	3.79556	0.00015	0.00107	1857.6	1697.08	1458.11	2366.82	2217.65	2356.66	Up
Cd109	98.2239	-0.9201	0.33458	-2.7499	0.00596	0.02503	89.0101	135.114	161.375	53.9092	86.5941	63.3403	Down
Gm8116	514.584	0.42978	0.17594	2.44275	0.01458	0.05187	440.213	395.761	479.351	608.552	679.024	484.603	Up
Gm10635	9.69547	-2.5902	0.88587	-2.9239	0.00346	0.0159	9.67501	23.9565	16.233	2.07343	4.2241	2.0108	Down
Tmem30a	27446.7	0.98205	0.09652	10.1748	2.57E-24	3.20E-22	18475.4	17968.3	18905.7	33474.5	39503.8	36352.3	Up
4930429F:	339.35	-0.7717	0.21742	-3.5492	0.00039	0.00248	348.301	524.167	411.555	206.306	269.286	276.485	Down
Filip1	654.052	-0.4047	0.18719	-2.162	0.03062	0.09315	654.031	871.057	710.433	456.155	650.512	582.127	Down
Myo6	5258.02	0.20587	0.08464	2.43243	0.015	0.05305	4891.69	4863.16	4895.68	5291.4	5806.03	5800.16	Up
Gm9531	198.803	0.39742	0.18557	2.14165	0.03222	0.09673	175.118	172.487	167.105	207.343	256.614	214.15	Up
Gm26907	1.71342	4.27884	2.17645	1.96597	0.0493	NA	0	0	0	4.14686	2.11205	4.0216	Up
Mei4	33.961	-1.2015	0.55054	-2.1824	0.02908	0.08983	33.8626	79.5355	28.6465	22.8077	14.7844	24.1296	Down
Sh3bgrl2	3125.88	0.55502	0.17073	3.25095	0.00115	0.00628	2123.67	2903.52	2568.63	3234.55	3467.99	4456.94	Up
Gm36120	4.58809	3.73967	1.628	2.29709	0.02161	0.07086	0	1.91652	0	12.4406	2.11205	11.0594	Up
Ttk	69.7157	0.77016	0.34809	2.21256	0.02693	0.08447	52.2451	66.1198	36.2855	71.5334	85.5381	106.572	Up
Bckdhb	1087.61	-0.4042	0.12844	-3.1471	0.00165	0.00848	1127.14	1365.52	1224.16	880.172	1013.78	914.915	Down
4933431K	80.8612	-0.9031	0.30029	-3.0075	0.00263	0.01259	128.678	103.492	84.0297	58.0561	48.5772	62.3349	Down
Pgm3	876.244	0.52545	0.13762	3.81823	0.00013	0.00098	761.424	763.732	630.223	1085.44	939.863	1076.78	Up
Me1	8777.02	0.43362	0.20986	2.0662	0.03881	0.11186	7827.09	8781.48	5795.18	8828.67	8401.74	13028	Up
Snap91	44.9964	-3.8448	0.62491	-6.1526	7.62E-10	1.71E-08	57.0826	146.614	48.699	6.2203	6.33615	5.027	Down
Nt5e	6342.76	0.46815	0.16166	2.89589	0.00378	0.01714	6547.08	5131.48	4289.33	7023.75	7381.62	7683.27	Up
Syncrip	1670.92	0.31326	0.12587	2.4888	0.01282	0.04684	1545.1	1352.1	1573.65	1738.57	1753	2063.08	Up
Gm38214	6.00546	-1.9968	0.99825	-2.0003	0.04546	0.12654	10.6425	9.58259	8.59395	4.14686	1.05603	2.0108	Down
Gm10634	22.8593	1.676	0.54435	3.0789	0.00208	0.01028	14.5125	12.4574	5.7293	39.3952	35.9049	29.1566	Up
Gm26611	10.8861	1.95138	0.88808	2.19732	0.028	0.08716	1.935	1.91652	9.54883	17.6242	23.2326	11.0594	Up
Gm2396	4.84647	3.82176	1.62982	2.3449	0.01903	0.06422	0	1.91652	0	10.3672	14.7844	2.0108	Up
Bcl2a1a	436.351	2.04958	0.24772	8.27377	1.30E-16	8.09E-15	209.948	150.447	148.962	656.241	916.63	535.879	Up
4930579C	5.15819	2.81665	1.35097	2.08491	0.03708	0.10806	1.935	0	1.90977	13.4773	11.6163	2.0108	Up
Gm47403	19.9221	1.91995	0.6224	3.08474	0.00204	0.01012	7.74001	11.4991	5.7293	27.9913	46.4651	20.108	Up
Tmed3	4272.27	0.39651	0.09628	4.11841	3.81E-05	0.00032	3792.61	3706.54	3567.44	5250.97	4593.71	4722.37	Up
Rasgrf1	232.423	-2.1255	0.30236	-7.0297	2.07E-12	7.08E-11	460.531	283.645	390.547	93.3044	54.9133	111.599	Down
Ctsh	29710.7	1.02603	0.13333	7.69532	1.41E-14	6.65E-13	22962.7	17930.9	17815.3	43064.1	37907.1	38584.3	Up
Morf4l1	15352.9	-0.2365	0.07754	-3.0507	0.00228	0.01114	16454.3	16255.9	17116.3	13716.8	13921.6	14652.7	Down
Tbc1d2b	556.332	0.71335	0.26235	2.71912	0.00655	0.02699	603.721	314.309	346.622	574.341	732.882	766.115	Up
Plscr1	6881.6	0.4096	0.10503	3.89996	9.62E-05	0.00073	5275.79	6345.59	6112.21	7671.7	8074.37	7809.95	Up
Plscr4	4599.82	-0.5306	0.10619	-4.9965	5.84E-07	7.61E-06	5754.7	5041.4	5512.54	3519.65	4066.75	3703.9	Down
Gm38391	57.2539	-0.6751	0.32164	-2.0988	0.03583	0.10515	63.8551	80.4937	66.8418	51.8358	43.297	37.1998	Down
Plod2	3173.47	-0.6174	0.10506	-5.8767	4.19E-09	8.28E-08	4009.33	3486.14	4031.52	2629.11	2421.47	2463.23	Down
Dipk2a	6650.03	-0.3533	0.13254	-2.6653	0.00769	0.03079	8709.45	6514.24	7156.85	5569.24	5834.54	6115.85	Down
Paqr9	136.667	-3.2398	0.49978	-6.4824	9.03E-11	2.40E-09	215.753	431.216	94.5334	29.028	25.3446	24.1296	Down
Pcolce2	9830.08	-0.8225	0.12158	-6.7651	1.33E-11	4.10E-10	13470.5	12470.8	11734.6	6852.69	8038.47	6413.45	Down
Trpc1	962.732	-0.5119	0.13817	-3.7049	0.00021	0.00146	1131.01	1129.79	1134.4	679.049	901.846	800.299	Down
Pls1	1970.51	0.58194	0.12298	4.73209	2.22E-06	2.53E-05	1671.84	1427.81	1635.71	2385.48	2138.45	2563.77	Up
Gm16794	84.8489	-0.6278	0.25353	-2.4763	0.01328	0.04816	97.7176	107.325	104.082	67.3865	60.1934	72.3889	Down
Gk5	333.798	0.71395	0.18445	3.87066	0.00011	0.00081	260.258	208.9	289.33	389.805	459.371	395.123	Up
Atp1b3	11727.3	0.32079	0.09178	3.49502	0.00047	0.00296	10415.2	10369.3	10501.8	12621	14144.4	12312.1	Up
Pxylp1	956.531	-0.9281	0.2771	-3.3495	0.00081	0.00466	1250.01	1364.56	1147.77	588.855	409.738	978.255	Down
Gm10123	3733.54	0.24126	0.07588	3.17961	0.00147	0.00773	3417.22	3419.07	3429.94	4123.02	4025.57	3986.41	Up
Clstn2	1155.18	-0.3157	0.15203	-2.0765	0.03784	0.10979	1085.54	1342.52	1415.14	905.053	1092.99	1089.85	Down
Rbp1	2395.93	-0.9441	0.12865	-7.3379	2.17E-13	8.70E-12	2789.31	3152.67	3516.83	1650.45	1757.23	1509.11	Down
Rbp2	122.21	1.41113	0.37113	3.80225	0.00014	0.00104	40.6351	77.619	82.1199	220.82	112.995	199.069	Up
Copb2	8438.51	0.28051	0.07825	3.58454	0.00034	0.0022	7669.38	7783.94	7408.94	9574.07	8955.1	9239.63	Up
Mrps22	752.68	-0.2857	0.12493	-2.2871	0.02219	0.07239	837.856	785.772	857.485	688.379	615.663	730.926	Down
Pik3cb	2871.58	0.26513	0.12655	2.09512	0.03616	0.10596	2828.01	2274.91	2722.37	2993	3413.07	2998.11	Up
Cep70	1620.34	-0.4429	0.10528	-4.2065	2.59E-05	0.00023	1870.18	1777.57	1953.69	1451.4	1276.73	1392.48	Down
4930422N	161.449	-0.9879	0.3149	-3.1372	0.00171	0.00872	199.305	226.149	218.668	131.663	60.1934	132.713	Down
Gm37249	184.243	-0.7014	0.19985	-3.5097	0.00045	0.00283	216.72	251.064	216.758	158.618	123.555	138.745	Down
Mras	953.082	-0.3475	0.11462	-3.0319	0.00243	0.01176	1065.22	1026.3	1110.53	897.796	775.123	843.531	Down
Nme9	4163.07	-0.6583	0.20853	-3.1568	0.0016	0.00825	4369.24	5968.03	4952.98	3754.98	2271.51	3661.67	Down
Dzip1l	2100.93	-0.419	0.14343	-2.9213	0.00349	0.01602	2398.44	2399.48	2413.94	2028.85	1463.65	1901.21	Down
Cldn18	39662	0.45589	0.14451	3.15479	0.00161	0.00829	38970	28100.9	33270	46126.6	49784.2	41720.1	Up
Gm34397	30.7573	3.56461	0.62463	5.70678	1.15E-08	2.05E-07	0.9675	7.66607	5.7293	41.4686	63.3615	65.3511	Up
Ppp2r3a	1987.83	-0.862	0.17164	-5.0224	5.10E-07	6.71E-06	2349.09	3102.84	2242.07	1192.22	1476.32	1564.4	Down

Gm28979	3.20709	-4.0386	1.83497	-2.2009	0.02774	0.08652	3.87001	12.4574	1.90977	0	0	1.0054	Down
Ky	573.769	-1.023	0.38313	-2.6702	0.00758	0.03044	430.538	1225.61	651.23	359.74	237.606	537.889	Down
Cep63	2412.93	-0.3189	0.11629	-2.742	0.00611	0.02553	2386.83	2896.82	2751.97	2108.68	2055.03	2278.24	Down
Anapc13	657.29	-0.3047	0.11521	-2.6445	0.00818	0.03241	690.796	757.983	730.485	607.515	581.87	575.089	Down
Ryk	6268.66	-0.2429	0.08931	-2.7197	0.00653	0.02696	7182.73	6351.34	6851.28	5802.5	5825.04	5599.08	Down
Rab6b	1131.07	-1.0139	0.17139	-5.9156	3.31E-09	6.65E-08	1647.65	1240.94	1650.04	866.694	726.545	654.516	Down
Srprb	3988.56	0.2192	0.09172	2.38973	0.01686	0.05834	3799.38	3492.85	3766.06	4473.43	4330.76	4068.86	Up
Trf	41944.4	1.02172	0.18668	5.473	4.42E-08	7.09E-07	28957.3	28348.2	25743.6	59883.8	40342.3	68391.4	Up
Cpne4	24.6975	2.37015	0.80418	2.94729	0.00321	0.01492	6.77251	12.4574	4.77441	58.0561	58.0814	8.04321	Up
Col6a5	52.7479	1.34733	0.50797	2.65239	0.00799	0.03178	33.8626	12.4574	42.9697	106.782	58.0814	62.3349	Up
Col6a4	37.9793	3.51748	0.9441	3.72574	0.00019	0.00136	12.5775	0.95826	4.77441	120.259	80.2579	9.04861	Up
Twf2	2843.73	-0.2526	0.11888	-2.1249	0.03359	0.09998	2915.08	3345.28	3015.52	2675.76	2795.3	2315.44	Down
Tlr9	68.4292	0.74059	0.30568	2.42277	0.0154	0.0542	60.9526	52.7042	40.1051	76.717	86.5941	93.5023	Up
Poc1a	323.131	0.45331	0.1535	2.95312	0.00315	0.01467	289.283	285.561	243.495	384.622	374.889	360.939	Up
Dusp7	6102.26	-0.3607	0.14286	-2.525	0.01157	0.04309	7979.95	5732.3	6871.34	5654.25	4969.66	5406.04	Down
Rpl29	7046.3	-0.6241	0.22347	-2.7928	0.00523	0.02249	8957.13	7512.75	9171.65	4531.48	4315.98	7788.84	Down
Acy1	506.426	0.67002	0.2035	3.29254	0.00099	0.00555	416.993	340.182	415.374	633.433	754.002	478.571	Up
Abhd14a	793.692	-1.1684	0.15961	-7.32	2.48E-13	9.89E-12	1176.48	1015.75	1103.84	442.678	438.251	585.143	Down
Pcbp4	1638.44	-0.3503	0.1288	-2.72	0.00653	0.02694	1825.68	1716.24	1967.06	1346.69	1635.78	1339.19	Down
Parp3	3870.84	0.55955	0.10925	5.12191	3.02E-07	4.16E-06	3268.22	3015.64	3104.32	4700.47	4998.17	4138.23	Up
Mapkapk3	3457.31	0.86381	0.1035	8.34619	7.05E-17	4.59E-15	2434.23	2594.01	2328	4401.9	4812.31	4173.42	Up
Cish	436.826	0.3787	0.15021	2.52117	0.0117	0.04347	372.488	356.472	410.6	517.321	438.251	525.825	Up
6430571L	26.1499	-1.4292	0.51995	-2.7487	0.00598	0.0251	44.5051	44.0799	25.7818	16.5875	16.8964	9.04861	Down
Cacna2d2	35.3429	-2.5576	0.87781	-2.9137	0.00357	0.01634	24.1875	144.697	12.4135	11.4039	5.28013	14.0756	Down
Cyb561d2	687.438	0.66287	0.15675	4.22869	2.35E-05	0.00021	593.078	527.042	476.487	878.098	924.022	725.899	Up
Zmynd10	440.82	-0.727	0.23861	-3.0466	0.00231	0.01125	489.556	592.204	567.2	369.071	220.709	406.182	Down
Hyal2	5067.19	-0.7677	0.17233	-4.4548	8.40E-06	8.42E-05	5302.88	5902.87	7947.49	3894.94	4057.25	3297.71	Down
Lsmem2	132.79	-0.6843	0.32558	-2.1018	0.03557	0.10455	108.36	194.527	188.112	115.075	118.275	72.3889	Down
Slc38a3	93.4558	-5.2447	0.60287	-8.6996	3.33E-18	2.53E-16	152.865	300.893	92.6236	4.14686	3.16808	7.03781	Down
Sema3f	9742.19	-0.2798	0.09283	-3.0137	0.00258	0.01238	10916.3	9899.77	11235.2	8834.89	9115.61	8451.4	Down
Uba7	3381.44	0.58952	0.09769	6.03486	1.59E-09	3.38E-08	2755.44	2696.54	2647.89	4115.76	4333.93	3739.09	Up
Gm20661	8.75296	1.98651	1.01164	1.96364	0.04957	0.1359	6.77251	2.87478	0.95488	6.2203	10.5603	25.135	Up
Cdhr4	1298.1	0.87409	0.23383	3.73821	0.00019	0.00131	911.386	1074.21	763.906	1951.1	1120.44	1967.57	Up
Ip6k1	6845.77	-0.2311	0.09253	-2.4976	0.0125	0.04587	7473.95	6900.42	7804.26	6579	6204.15	6112.84	Down
Gmppb	967.372	0.59781	0.1228	4.86835	1.13E-06	1.37E-05	770.131	757.983	781.094	1293.82	1140.51	1060.7	Up
Rnf123	1189.04	0.23958	0.11054	2.16736	0.03021	0.09222	1138.75	1048.33	1084.75	1280.34	1199.64	1382.43	Up
Bsn	120.036	0.9058	0.26176	3.46049	0.00054	0.0033	77.4001	98.7006	74.4809	134.773	190.085	144.778	Up
Dag1	20124.8	0.50732	0.09338	5.43265	5.55E-08	8.75E-07	17391.8	17335.9	15139.7	24016.6	23476.5	23388.6	Up
Amt	931.367	-0.5358	0.16809	-3.1876	0.00143	0.00755	1063.28	1115.41	1128.67	702.893	638.895	939.044	Down
Rhoa	22840.5	0.2669	0.09572	2.78834	0.0053	0.02275	21987.4	19123	21090.5	24587.8	26238	24016	Up
Gpx1	19934.6	0.55137	0.1233	4.47187	7.75E-06	7.85E-05	17861	15716.4	14935.3	24385.6	25757.5	20951.5	Up
1700102P	137.726	-0.6009	0.24316	-2.4713	0.01346	0.04873	139.32	160.987	197.661	94.3411	121.443	112.605	Down
Klhdc8b	3327.5	-0.271	0.11879	-2.2813	0.02253	0.07322	3312.73	3507.23	4097.4	3089.41	2835.43	3122.78	Down
Qars	5309.13	0.29028	0.11743	2.47189	0.01344	0.04867	5251.6	4562.27	4516.6	6459.78	5496.61	5567.91	Up
Impdh2	2867.85	-0.2263	0.09574	-2.364	0.01808	0.06165	3143.41	3036.72	3096.69	2811.57	2667.52	2451.17	Down
4833445I(	39.6766	1.04339	0.36879	2.82921	0.00467	0.02046	23.22	25.873	28.6465	52.8725	60.1934	47.2538	Up
Wdr6	4592.67	0.55434	0.1181	4.69385	2.68E-06	3.01E-05	4127.36	3610.72	3425.16	5813.9	4992.89	5586.01	Up
P4htm	712.031	-0.7316	0.15785	-4.6345	3.58E-06	3.92E-05	995.559	803.979	867.034	575.377	455.147	575.089	Down
Arih2	3302.78	0.24906	0.07903	3.15139	0.00162	0.00838	3081.49	2953.35	3020.29	3603.62	3638.01	3519.91	Up
Prkar2a	5536.69	0.42136	0.11461	3.67654	0.00024	0.0016	4598.53	4493.27	5109.58	5801.46	7010.95	6206.34	Up
Celsr3	73.5614	0.71225	0.32531	2.18943	0.02857	0.08858	73.5301	50.7877	42.9697	83.974	106.659	83.4483	Up
Col7a1	662.365	0.51599	0.25062	2.05889	0.03951	0.11342	419.896	578.788	636.907	693.563	591.374	1053.66	Up
Pfkfb4	4541.74	1.17386	0.10646	11.0262	2.86E-28	5.18E-26	3009.9	2759.78	2599.19	6076.19	6786.02	6019.33	Up
Shisa5	17612.1	0.58367	0.10017	5.82671	5.65E-09	1.08E-07	14785.4	13250.8	14255.4	21157.3	22694	19529.9	Up
Fbxw22	4.46715	3.69607	1.51011	2.44755	0.01438	0.05134	0	1.91652	0	8.29373	10.5603	6.03241	Up
Fbxw23	3.25939	4.17079	1.67945	2.48343	0.01301	0.04739	0.9675	0	0	6.2203	6.33615	6.03241	Up
Fbxw15	10.0406	3.53245	1.05686	3.3424	0.00083	0.00476	0	3.83303	0.95488	21.771	10.5603	23.1242	Up
Fbxw26	25.2783	-2.9553	0.92485	-3.1954	0.0014	0.00738	31.9275	89.1181	13.3684	2.07343	2.11205	13.0702	Down
3000002C	152.815	-0.4243	0.21075	-2.0132	0.04409	0.12363	182.858	181.111	161.375	113.002	147.844	130.702	Down
Cdc25a	838.435	0.69758	0.13016	5.3593	8.35E-08	1.26E-06	674.349	574.955	669.373	1031.53	1116.22	964.179	Up
Scap	3409.03	0.45203	0.08894	5.08259	3.72E-07	5.04E-06	3053.43	2792.37	2792.08	3880.43	4053.03	3882.86	Up
Klhl18	1412.76	0.43922	0.11695	3.75567	0.00017	0.00123	1171.64	1094.33	1332.06	1575.81	1634.73	1667.96	Up
Myl3	130.725	-3.3965	1.06089	-3.2016	0.00137	0.00726	142.223	350.723	223.443	16.5875	2.11205	49.2646	Down
Prss50	14.7938	-1.7236	0.64514	-2.6717	0.00755	0.03034	20.3175	22.0399	25.7818	10.3672	4.2241	6.03241	Down
Tmie	296.083	-0.8504	0.22832	-3.7247	0.0002	0.00137	297.023	468.588	377.179	231.188	175.3	227.221	Down
Als2cl	6950.73	-0.2763	0.12107	-2.2825	0.02246	0.07302	6583.85	7713.02	8546.2	6398.61	6349.88	6112.84	Down
Lrrc2	320.891	-2.5661	0.71816	-3.5731	0.00035	0.00229	290.25	1103.91	253.044	65.3131	98.2104	114.616	Down
Rtp3	419.355	-0.7065	0.22965	-3.0763	0.0021	0.01036	431.506	524.167	604.441	230.151	355.881	369.988	Down
Ltf	21812	3.31775	0.95751	3.46498	0.00053	0.00326	856.239	9144.66	1927.91	40051.4	31896.2	46995.5	Up
Gm43814	138.277	-0.6712	0.29357	-2.2863	0.02224	0.07251	133.515	202.193	173.789	88.1208	142.563	89.4807	Down
Ccrl2	2339.37	-0.3854	0.14909	-2.5852	0.00973	0.03736	2390.7	2634.25	2924.81	2157.41	2243	1686.06	Down
Lrrfip2	5848.6	0.2646	0.09456	2.79812	0.00514	0.02221	5485.73	5270.42	5185.01	6016.06	6895.85	6238.51	Up
Stac	137.293	2.44776	0.35162	6.96138	3.37E-12	1.11E-10	25.155	66.1198	36.2855	221.857	268.23	206.107	Up
Arpp21	47.0273	2.08281	0.56203	3.70586	0.00021	0.00146	28.0575	15.3321	10.5037	117.149	73.9218	37.1998	Up
Pdcd6ip	12505.2	0.33605	0.08835	3.80364	0.00014	0.00104	11087.6	10926.1	11152.1	13542.6	15033.6	13289.4	Up
Clasp2	2771.07	-0.2779	0.09932	-2.7977	0.00515	0.02223	2884.12	3048.22	3178.81	2356.46	2665.41	2493.39	Down
4930520C	116.799	-0.7244	0.32755	-2.2117	0.02699	0.08458	108.36	192.61	135.593	116.112	69.6977	78.4213	Down
Glb1	2741.61	0.58429	0.11409	5.12128	3.03E-07	4.17E-06	2381.99	2255.74	1944.14	3310.23	3153.29	3404.29	Up
Cnot10	3976.72	-0.3124	0.09864	-3.1669	0.00154	0.00801	4297.64	4219.21	4699.93	3776.76	3374	3492.76	Down
Cmtm6	8145.84	0.66447	0.10925	6.08188	1.19E-09	2.60E-08	6816.05	6067.69	6023.4	9440.33	10974.2	9553.32	Up
Cmtm8	2743.15	0.2242	0.09586	2.33884	0.01934	0.06504	2622.9	2492.43	2476.01	3170.28	2824.87	2872.43	Up
Osbpl10	1063.3	0.53773	0.12321	4.36421	1.28E-05	0.00012	892.036	862.433	847.936	1171.49	1193.31	1412.59	Up
Tgfbr2	20836	0.60512	0.12933	4.67883	2.89E-06	3.22E-05	18928.2	15244.9	15414.7	24333.8	27767.1	23327.3	Up

D730003K	173.558	2.70848	0.41093	6.59108	4.37E-11	1.22E-09	35.7976	64.2033	38.1953	294.427	156.292	452.43 Up
Eomes	56.2707	-1.0665	0.41273	-2.5841	0.00976	0.03745	74.4976	95.8259	58.2479	21.771	38.0169	49.2646 Down
Itga9	1676.02	0.4645	0.10607	4.37901	1.19E-05	0.00011	1488.98	1382.77	1354.02	1953.17	1811.08	2066.1 Up
Gm2415	296.912	-0.5986	0.15686	-3.8159	0.00014	0.00099	389.903	334.432	348.532	243.628	240.774	224.204 Down
Ctdspl	8338.09	-0.2703	0.11073	-2.4413	0.01464	0.05203	10226.5	8287.98	8836.49	7735.97	7641.4	7300.22 Down
Scn5a	1569.34	-3.038	0.25018	-12.143	6.22E-34	1.79E-31	2191.39	3796.62	2406.3	306.868	273.511	441.371 Down
Scn10a	45.4079	-3.2609	0.65555	-4.9743	6.55E-07	8.44E-06	66.7576	132.24	47.7441	15.5507	2.11205	8.04321 Down
Rpsa	39063.6	-0.2055	0.10056	-2.0435	0.041	0.11681	42238.2	39142.9	44141.4	37788.3	37830	33240.6 Down
Myrip	1931.48	-0.8756	0.11716	-7.4739	7.79E-14	3.38E-12	2205.9	2701.33	2593.46	1393.35	1370.72	1324.11 Down
Eif1b	2508.48	-0.3148	0.08778	-3.5864	0.00034	0.00219	2770.92	2868.07	2704.23	2326.39	2217.65	2163.62 Down
Entpd3	493.31	-0.5461	0.21956	-2.4872	0.01288	0.04698	510.841	512.668	733.35	410.539	318.92	473.544 Down
Rpl14	11400.5	-0.2627	0.12363	-2.1252	0.03357	0.09993	13011.9	10869.5	13425.7	10565.2	11104.1	9426.64 Down
Gm39458	10.4355	3.29731	1.16876	2.82121	0.00478	0.0209	4.83751	0	0.95488	5.18358	28.5127	23.1242 Up
Gm34425	13.9245	2.50745	0.83844	2.99061	0.00278	0.0132	5.80501	4.79129	1.90977	8.29373	29.5687	33.1782 Up
Gm39460	3.74426	3.42164	1.5796	2.16615	0.0303	0.09243	0	1.91652	0	6.2203	5.28013	9.04861 Up
Ctnnb1	6431.76	0.83033	0.13778	6.02656	1.67E-09	3.53E-08	5314.49	4054.39	4522.33	7456.06	8193.7	9049.61 Up
Ulk4	1661.26	-0.4868	0.23113	-2.1063	0.03518	0.10373	1642.82	2271.07	1903.08	1502.2	926.134	1722.25 Down
Trak1	9660.99	0.21361	0.09745	2.19205	0.02838	0.08813	8487.89	9668.83	8684.66	10564.1	9793.58	10766.8 Up
Ss18l2	669.431	-0.3853	0.1446	-2.6648	0.0077	0.03083	755.619	789.605	729.531	658.315	580.814	502.7 Down
Zfp651	1379.91	-0.3591	0.13608	-2.6384	0.00833	0.03288	1699.9	1616.58	1335.88	1281.38	1114.11	1231.62 Down
Klhl40	11.7524	-2.734	1.0431	-2.621	0.00877	0.03427	5.80501	41.2051	14.3232	1.03672	2.11205	6.03241 Down
Hhatl	551.744	-1.964	0.30779	-6.381	1.76E-10	4.50E-09	531.158	1204.53	899.5	226.004	164.74	284.528 Down
Ccdc13	532.916	-0.6199	0.22662	-2.7355	0.00623	0.02597	587.273	747.442	602.531	443.714	296.743	519.792 Down
Ackr2	2248.69	-0.3069	0.11404	-2.6913	0.00712	0.02892	2630.64	2263.41	2566.73	1986.35	2165.91	1879.09 Down
Gask1a	383.142	-0.5349	0.16631	-3.2164	0.0013	0.00695	439.246	416.843	504.178	299.611	284.071	354.907 Down
Pomgnt2	796.554	-0.4477	0.13871	-3.2277	0.00125	0.00673	966.534	915.137	875.628	716.371	726.545	579.111 Down
Ano10	3194.78	0.33853	0.11051	3.06345	0.00219	0.01074	3123.09	2805.78	2536.17	3524.83	3633.78	3545.04 Up
Abhd5	2516.02	0.70912	0.15014	4.72316	2.32E-06	2.64E-05	2269.76	1675.99	1783.72	3031.36	3444.75	2890.53 Up
A730085K	113.567	0.76542	0.34609	2.21162	0.02699	0.08459	60.9526	108.283	83.0748	108.855	199.589	120.648 Up
Gm47135	17.9928	2.5895	0.74564	3.47284	0.00051	0.00318	5.80501	5.74955	3.81953	15.5507	54.9133	22.1188 Up
Tcaim	93.1987	0.54443	0.2639	2.06301	0.03911	0.11252	70.6276	78.5772	78.3004	122.332	88.7061	120.648 Up
Kif15	208.513	0.46351	0.21427	2.16324	0.03052	0.09294	165.443	155.238	205.3	250.885	202.757	271.458 Up
Zdhhc3	10542	0.50383	0.11701	4.30568	1.66E-05	0.00015	9400.24	7485.92	9272.87	12513.2	12385.1	12194.5 Up
Cdcp1	3168.37	1.13477	0.08525	13.3107	2.01E-40	1.01E-37	1969.83	2027.68	1950.83	4249.5	4552.53	4259.88 Up
Sacm1l	1251.73	0.54127	0.16265	3.32776	0.00088	0.00497	1102.95	966.883	989.259	1256.5	1397.12	1797.66 Up
Slc6a20a	573.297	2.10849	0.32739	6.44029	1.19E-10	3.13E-09	242.843	232.857	171.879	908.163	501.612	1382.43 Up
Fyco1	1052.48	0.39273	0.13393	2.93238	0.00336	0.01553	1041.03	874.89	814.515	1228.51	1138.4	1217.54 Up
Xcr1	275.31	1.36797	0.2137	6.40133	1.54E-10	3.98E-09	180.923	160.029	120.315	396.025	446.699	347.869 Up
Ccr1	916.111	1.68143	0.26686	6.30073	2.96E-10	7.21E-09	648.226	365.097	293.149	1441.04	1476.32	1272.84 Up
Ccr2	1829.5	0.58949	0.17986	3.27755	0.00105	0.00579	1391.27	1755.53	1235.62	1991.53	2628.45	1974.61 Up
Ccr5	708.321	2.03986	0.16323	12.4966	7.79E-36	2.58E-33	272.835	255.855	302.698	973.476	1131	1314.06 Up
2010315B	1521.81	0.35907	0.13743	2.61277	0.00898	0.03493	1321.61	1447.93	1230.84	1547.82	1623.11	1959.53 Up
H60c	313.712	0.8052	0.32987	2.44099	0.01465	0.05207	366.683	140.864	177.608	396.025	415.018	386.074 Up
Ppp1r14c	7293.75	0.33643	0.10602	3.17341	0.00151	0.00787	6097.19	6823.76	6420.63	7618.82	7861.05	8941.03 Up
Iyd	480.247	-0.7004	0.32264	-2.1708	0.02995	0.09172	525.353	552.915	705.658	387.732	189.029	520.798 Down
Plekhg1	1982.58	0.82615	0.10441	7.91226	2.53E-15	1.33E-13	1515.11	1320.48	1454.29	2404.14	2611.55	2589.91 Up
Akap12	6806.32	-0.5884	0.16141	-3.6454	0.00027	0.00179	6341.97	8465.26	9718.8	5255.11	5566.31	5490.49 Down
Gm21781	472.212	-0.4113	0.13948	-2.949	0.00319	0.01485	564.053	493.503	559.561	391.879	437.195	387.079 Down
Armt1	902.261	0.28862	0.12018	2.40147	0.01633	0.0568	899.776	764.69	772.5	969.329	1016.95	990.32 Up
Esr1	254.123	0.43004	0.20093	2.14019	0.03234	0.09698	170.28	247.231	232.037	272.656	299.911	302.626 Up
Syne1	6935.89	-0.4048	0.1101	-3.6769	0.00024	0.0016	7256.26	7927.67	8524.24	5579.6	5858.83	6468.75 Down
Ulbp1	342.335	0.92998	0.17516	5.30923	1.10E-07	1.62E-06	268.965	210.817	227.262	394.989	479.436	472.538 Up
Lrp11	467.702	0.6386	0.17171	3.71902	0.0002	0.00139	357.976	430.258	309.382	537.019	551.245	620.332 Up
Pcmt1	4993.39	-0.2144	0.08086	-2.6517	0.00801	0.03183	5368.67	5178.43	5544.05	4688.03	4694.03	4487.1 Down
Zc3h12d	268.47	0.65899	0.19638	3.35562	0.00079	0.00458	253.485	184.944	186.202	303.758	351.656	330.777 Up
Ust	464.182	-0.6547	0.21667	-3.0216	0.00251	0.0121	635.648	497.336	570.065	439.568	378.057	264.42 Down
Sash1	4968.92	-0.3742	0.12071	-3.1001	0.00193	0.0097	4943.93	5850.17	6034.86	4169.67	4708.82	4106.06 Down
Adgb	2259.82	-0.6247	0.20869	-2.9936	0.00276	0.01309	2492.28	3151.71	2581.05	1925.18	1245.05	2163.62 Down
Rab32	1477.82	1.59687	0.22215	7.18837	6.56E-13	2.43E-11	900.744	763.732	538.554	1949.03	2779.46	1935.4 Up
Grm1	34.9168	-4.8077	1.05313	-4.5651	4.99E-06	5.28E-05	15.48	169.612	17.1879	1.03672	3.16808	3.0162 Down
Fbxo30	2535.61	-0.5846	0.10829	-5.3985	6.72E-08	1.03E-06	2935.4	3202.5	2989.74	2016.41	1860.72	2208.87 Down
Epm2a	703.568	-0.347	0.16192	-2.1428	0.03213	0.09656	857.206	846.142	659.824	619.956	684.304	553.976 Down
Utrn	8545.15	-0.2635	0.08743	-3.0138	0.00258	0.01238	9242.54	9025.84	9701.61	7318.18	8093.38	7889.38 Down
Plagl1	1496.9	-0.5261	0.12676	-4.1499	3.33E-05	0.00029	1760.85	1657.79	1882.07	1288.64	1073.98	1318.08 Down
Phactr2	5113.83	-0.3693	0.10275	-3.5938	0.00033	0.00213	6013.02	6037.99	5243.26	4220.47	4550.41	4617.81 Down
Fuca2	6616.6	0.7821	0.09762	8.01142	1.13E-15	6.26E-14	4976.83	5198.55	4422.06	8228.41	8474.6	8399.12 Up
Pex3	2162.45	0.20293	0.08966	2.26328	0.02362	0.07614	1980.48	2117.75	1933.64	2288.03	2324.31	2330.52 Up
Aig1	3567.93	0.31764	0.08195	3.87612	0.00011	0.0008	3185.98	3263.83	3080.45	4059.78	3849.21	3968.32 Up
Adgrg6	3726.97	-0.4167	0.11595	-3.5935	0.00033	0.00213	3729.72	4449.19	4605.4	3325.78	3106.83	3144.89 Down
Gm47712	13.5027	-3.8823	0.97605	-3.9776	6.96E-05	0.00055	25.155	37.3721	13.3684	3.11015	0	2.0108 Down
Gm47770	11.7405	3.77214	1.00635	3.74833	0.00018	0.00126	2.9025	0.95826	0.95488	29.028	8.4482	28.1512 Up
Cited2	4869.48	0.84051	0.16843	4.9903	6.03E-07	7.84E-06	3376.58	3466.02	3626.65	6653.64	7361.55	4732.42 Up
Txlnb	719.206	-4.8878	0.93445	-5.2307	1.69E-07	2.42E-06	881.394	2843.15	449.75	20.7343	32.7368	87.4699 Down
Abracl	1580.53	0.39669	0.13837	2.86681	0.00415	0.01854	1249.04	1331.98	1512.53	1838.1	1981.1	1570.44 Up
Ccdc28a	749.152	-0.3142	0.13944	-2.2537	0.02422	0.07765	905.581	752.233	833.613	708.077	596.654	698.754 Down
Hebp2	58.8236	-1.0686	0.50907	-2.0991	0.03581	0.10514	63.8551	130.323	44.8795	40.4319	22.1765	51.2754 Down
Gm48297	3.09472	4.0922	1.79237	2.28312	0.02242	0.07293	0	0.95826	0	2.07343	9.50423	6.03241 Up
Arfgef3	3693.07	1.80182	0.13194	13.6564	1.85E-42	1.11E-39	1804.39	1502.55	1631.89	5013.56	6343.54	5862.49 Up
Gm48483	4.1516	-5.4504	1.85057	-2.9453	0.00323	0.01499	1.935	16.2904	6.68418	0	0	0 Down
Tnfaip3	1727.2	1.03015	0.0943	10.9248	8.78E-28	1.54E-25	1196.8	1098.16	1111.48	2290.11	2382.39	2284.27 Up
Olig3	176.475	-0.9687	0.35216	-2.7507	0.00595	0.025	133.515	305.685	261.638	136.846	83.426	137.74 Down
Slc35d3	124.477	-1.4517	0.28342	-5.1219	3.02E-07	4.16E-06	142.223	234.773	169.969	63.2397	61.2495	75.4051 Down
Mtfr2	61.7366	0.6521	0.30101	2.16636	0.03028	0.09239	45.4726	55.579	42.9697	76.717	81.314	68.3673 Up

Ahi1	859.124	-0.4294	0.14048	-3.0566	0.00224	0.01094	921.061	939.093	1098.12	682.159	695.921	818.396 Down
Myb	1257.45	-0.4982	0.18105	-2.7519	0.00593	0.02493	1411.58	1614.67	1391.26	1197.41	784.627	1145.15 Down
1700020N	3.09704	4.09246	1.71659	2.38406	0.01712	0.05903	0.9675	0	0	7.25701	6.33615	4.0216 Up
Sgk1	15790.1	0.56678	0.23423	2.41978	0.01553	0.05453	10642.5	14831	12709.5	21337.7	23149.1	12070.8 Up
E030030I(	354.323	0.52104	0.17084	3.04993	0.00229	0.01116	316.373	253.939	302.698	390.842	470.987	391.101 Up
Gm10824	113.931	-0.868	0.27092	-3.204	0.00136	0.00721	137.385	144.697	159.465	103.672	76.0338	62.3349 Down
Vnn3	1342.68	1.84311	0.52887	3.48502	0.00049	0.00306	724.659	564.414	466.938	2191.62	910.294	3198.18 Up
Vnn1	1732.73	1.63488	0.62851	2.60119	0.00929	0.03597	1380.62	557.707	593.937	2711.01	1224.99	3928.1 Up
Ccn2	9111.8	-0.5742	0.24169	-2.3756	0.01752	0.06007	12146	12582.9	7975.18	9841.54	6754.34	5370.85 Down
Enpp1	815.344	0.32982	0.11999	2.74877	0.00598	0.0251	706.276	690.904	770.59	897.796	853.268	973.228 Up
Med23	2001.93	0.22184	0.09397	2.36079	0.01824	0.06211	1846.96	1816.86	1881.12	2022.63	2247.22	2196.8 Up
Arg1	148.041	0.90462	0.3241	2.79116	0.00525	0.02257	113.198	119.782	76.3906	180.389	133.059	265.426 Up
Epb41l2	10434.7	-0.2475	0.09453	-2.6185	0.00883	0.03449	11574.2	11173.3	11235.2	8919.9	10375.4	9330.12 Down
Tmem200	414.157	-0.3839	0.16694	-2.2996	0.02147	0.07047	395.708	485.837	525.186	335.896	384.393	357.923 Down
Lama2	2346.19	-0.2892	0.09321	-3.1023	0.00192	0.00963	2537.76	2555.68	2647.89	2147.04	2208.15	1980.64 Down
9330159F	218.775	-1.9313	0.25381	-7.6093	2.76E-14	1.27E-12	267.03	384.262	388.637	114.039	80.2579	78.4213 Down
Echdc1	797.462	0.30774	0.13693	2.24738	0.02462	0.07869	718.854	637.242	782.049	808.638	946.199	891.791 Up
Hint3	443.467	0.49788	0.14826	3.35814	0.00078	0.00455	336.691	395.761	370.495	535.982	553.357	468.517 Up
Ncoa7	1935.76	0.81192	0.10998	7.38236	1.56E-13	6.45E-12	1477.37	1327.19	1410.36	2300.47	2680.19	2418.99 Up
Hey2	256.97	-0.8001	0.31996	-2.5007	0.0124	0.0456	224.46	380.429	374.314	156.544	270.342	135.729 Down
Hddc2	468.671	0.35333	0.12808	2.75861	0.0058	0.02454	413.123	398.636	423.013	502.807	517.452	556.992 Up
Rnf217	1254.5	-0.2543	0.10195	-2.4947	0.0126	0.04619	1303.22	1349.23	1441.87	1188.08	1122.55	1122.03 Down
Trdn	862.12	-5.0314	0.75854	-6.633	3.29E-11	9.42E-10	1000.4	3200.58	818.335	37.3218	30.6247	85.4591 Down
Rsph4a	2515.75	-0.586	0.22802	-2.5702	0.01016	0.03872	2895.73	3306.95	2857.01	2028.85	1330.59	2675.37 Down
Gm48018	41.7379	-1.0767	0.43449	-2.4782	0.01321	0.04794	43.5376	83.3685	42.9697	26.9546	27.4567	26.1404 Down
A830082N	120.759	-0.5312	0.26117	-2.0341	0.04194	0.11898	156.735	129.365	142.278	98.488	76.0338	121.654 Down
Dse	1748.33	0.42751	0.10921	3.91454	9.06E-05	0.0007	1384.49	1625.21	1463.84	1924.14	1999.06	2093.24 Up
Frk	1247.66	2.01347	0.13394	15.0324	4.51E-51	4.23E-48	542.768	522.251	421.103	1906.52	2062.42	2030.91 Up
Marcks	10053.5	-0.398	0.11635	-3.4209	0.00062	0.00374	9849.16	12145.9	12299.8	8801.72	8744.95	8479.55 Down
Lama4	3991.22	-0.7774	0.12668	-6.1363	8.45E-10	1.88E-08	4403.1	5124.77	5595.61	2780.47	3186.03	2857.35 Down
Fam229b	275.518	-0.8391	0.19522	-4.298	1.72E-05	0.00016	297.023	404.385	359.036	180.389	190.085	222.194 Down
Fyn	2044.98	-0.3433	0.12987	-2.6437	0.0082	0.03246	2113.02	2093.8	2654.57	1790.41	1858.6	1759.45 Down
Gm16364	308.842	-0.8731	0.17111	-5.1026	3.35E-07	4.58E-06	351.203	390.97	456.434	211.49	221.765	221.188 Down
Rev3l	2457.46	-0.2955	0.13355	-2.2125	0.02694	0.08448	2455.52	2785.66	2883.75	2321.21	1898.73	2399.89 Down
4930547N	126.394	-1.1021	0.27208	-4.0506	5.11E-05	0.00042	153.833	216.566	147.052	74.6435	70.7537	95.5131 Down
Mfsd4b5	132.193	-1.3638	0.29062	-4.6928	2.69E-06	3.02E-05	192.533	207.942	170.924	71.5334	50.6892	99.5347 Down
Mfsd4b1	322.387	-1.0108	0.21085	-4.7938	1.64E-06	1.93E-05	375.391	505.961	411.555	230.151	168.964	242.302 Down
Slc16a10	1171.06	-0.3871	0.1778	-2.177	0.02948	0.09071	1092.31	1527.46	1361.66	938.228	1202.81	903.855 Down
Amd1	5308.64	0.3939	0.09032	4.36133	1.29E-05	0.00012	4672.06	4801.83	4291.24	6221.33	6032.02	5833.34 Up
Cdk19	6468.3	0.62197	0.15921	3.90662	9.36E-05	0.00072	5602.8	5259.88	4423.02	9440.33	6839.88	7243.91 Up
Ddo	2367.88	-0.5577	0.1749	-3.1887	0.00143	0.00753	2496.15	2892.98	3070.9	2110.75	1466.82	2169.66 Down
Mettl24	606.827	-0.5915	0.12228	-4.8372	1.32E-06	1.58E-05	770.131	698.571	719.982	485.183	459.371	507.727 Down
Fig4	2679.5	0.80723	0.16684	4.83834	1.31E-06	1.57E-05	2425.53	1827.4	1593.7	3212.78	3458.48	3559.12 Up
Ak9	1436.73	-0.5689	0.24289	-2.3421	0.01917	0.06458	1116.5	1967.31	2065.41	1250.28	921.91	1298.98 Down
Mical1	1498.63	0.40801	0.16199	2.51867	0.01178	0.04371	1358.37	1137.45	1368.35	1610.02	2048.69	1468.89 Up
Ppil6	3195.29	-0.4887	0.23988	-2.0374	0.04161	0.11823	3235.32	4084.1	3874.91	2993	1648.46	3335.92 Down
Cd164	26773.6	0.45221	0.1073	4.2146	2.50E-05	0.00022	22686.9	22651.3	22496.1	30494	34507.7	27805.4 Up
Sesn1	3142.01	-0.6579	0.13764	-4.7797	1.76E-06	2.05E-05	3307.89	4498.07	3732.64	2473.6	2514.4	2325.49 Down
Armc2	896.403	-0.5495	0.22807	-2.4093	0.01598	0.05587	951.054	1198.78	1045.6	800.345	486.828	895.812 Down
Afg1l	1449.94	-0.2968	0.09462	-3.1364	0.00171	0.00873	1561.55	1614.67	1619.48	1241.99	1313.7	1348.24 Down
Ostm1	3290.79	0.45733	0.09739	4.69585	2.66E-06	2.98E-05	2854.13	2898.73	2567.68	3691.75	3983.33	3749.14 Up
Sobp	451.25	0.93008	0.20947	4.44023	8.99E-06	8.93E-05	247.68	408.218	275.961	582.634	599.822	593.187 Up
Pdss2	662.733	-0.2765	0.13727	-2.0143	0.04398	0.12343	680.154	764.69	733.35	528.725	621.999	647.478 Down
Crybg1	553.215	0.37139	0.13817	2.688	0.00719	0.02913	508.906	489.67	448.795	628.25	566.03	677.64 Up
Atg5	2387.56	0.37106	0.09326	3.9786	6.93E-05	0.00055	2113.02	2091.88	2041.54	2667.47	2860.77	2550.7 Up
Gm48065	8.88423	3.04494	0.91207	3.3385	0.00084	0.00482	1.935	0.95826	2.86465	14.514	17.9524	15.081 Up
Prep	6417.42	0.50915	0.09012	5.64949	1.61E-08	2.80E-07	5663.75	5232.09	4994.04	7520.34	7722.71	7371.6 Up
Popdc3	857.21	-0.3421	0.13721	-2.4932	0.01266	0.04632	1062.32	888.306	924.327	758.876	824.756	684.678 Down
Bves	195.958	-4.4845	0.53265	-8.4194	3.79E-17	2.57E-15	238.005	678.447	209.119	8.29373	13.7283	28.1512 Down
Hace1	1332.98	-0.3565	0.10851	-3.2854	0.00102	0.00566	1501.56	1407.68	1581.29	1092.7	1209.15	1205.48 Down
Gm46224	86.902	2.02478	0.34613	5.84984	4.92E-09	9.57E-08	44.5051	36.4138	21.9623	171.058	128.835	118.637 Up
Gm48786	13.9066	2.6349	1.02797	2.56322	0.01037	0.03939	9.67501	1.91652	0	13.4773	22.1765	36.1944 Up
Fam162b	326.672	-0.941	0.27282	-3.4492	0.00056	0.00342	320.243	368.93	599.666	202.16	199.589	269.447 Down
Gprc6a	150.878	-1.0704	0.22436	-4.7709	1.83E-06	2.13E-05	180.923	205.067	227.262	81.9006	104.547	105.567 Down
Ros1	73.4254	0.99876	0.31893	3.13164	0.00174	0.00886	51.2776	41.2051	54.4283	75.6803	121.443	96.5185 Up
Nus1	6962.49	0.76169	0.10337	7.36859	1.72E-13	7.04E-12	5239.99	5050.98	5206.98	8410.88	9713.32	8152.8 Up
Cep85l	2465.78	-0.643	0.14276	-4.5043	6.66E-06	6.84E-05	2481.64	3098.05	3439.49	1890.97	1880.78	2003.76 Down
Man1a	5784.66	0.85198	0.13715	6.2122	5.22E-10	1.21E-08	4426.32	4311.21	3636.19	6501.25	8388.01	7444.99 Up
Gja1	10047.1	2.17553	0.15109	14.3991	5.24E-47	3.90E-44	3676.51	4255.63	2993.56	15101.8	18060.1	16195 Up
Gm40652	19.4576	1.30844	0.59074	2.21494	0.02676	0.08407	11.61	7.66607	14.3232	19.6976	23.2326	40.216 Up
Hsf2	1311.14	-0.2158	0.09891	-2.182	0.02911	0.08986	1379.66	1377.02	1470.52	1221.25	1161.63	1256.75 Down
Pkib	416.452	1.06199	0.22802	4.6574	3.20E-06	3.54E-05	318.308	290.352	200.525	605.442	634.671	449.414 Up
Smpdl3a	9978.5	0.2834	0.12749	2.22295	0.02622	0.08272	10082.3	8886.89	8035.34	11056.6	11927.8	9882.09 Up
Gcc2	4332.85	0.32052	0.08762	3.65807	0.00025	0.00171	3870.97	3772.66	3916.93	4512.82	4979.16	4944.56 Up
Ccdc138	850.329	-0.5046	0.19547	-2.5815	0.00984	0.03769	904.614	1058.88	1029.36	727.775	522.733	858.612 Down
Sowahc	3146.54	0.78159	0.11832	6.6055	3.96E-11	1.12E-09	2590	2192.5	2160.9	3756.02	4275.85	3903.97 Up
Pla2g12b	19.7705	-1.3022	0.63945	-2.0365	0.0417	0.1184	19.35	20.1234	44.8795	9.33044	16.8964	8.04321 Down
Oit3	43.6225	-1.2242	0.36464	-3.3573	0.00079	0.00456	53.2126	56.5373	73.526	25.9179	26.4006	26.1404 Down
Spock2	16933.9	0.26932	0.1118	2.40897	0.016	0.05589	15150.1	13737.6	17186	18799.8	18914.5	17815.7 Up
Psap	59432.1	0.79724	0.15684	5.0832	3.71E-07	5.02E-06	49063	44245.7	36940.6	72728.7	88518.2	65096.7 Up
Slc29a3	971.891	0.49654	0.14455	3.43509	0.00059	0.00358	881.394	863.391	674.147	1093.74	1116.22	1202.46 Up
Sgpl1	4541.25	0.61757	0.11616	5.3166	1.06E-07	1.56E-06	4019.97	3432.48	3299.12	5169.07	5810.25	5516.63 Up
Tbata	554.251	-3.9495	0.37296	-10.59	3.33E-26	4.95E-24	711.114	1418.22	994.033	29.028	87.6501	85.4591 Down

Adamts14	276.509	-0.8493	0.20499	-4.1431	3.43E-05	0.00029	276.705	412.051	378.134	196.976	190.085	205.102	Down
Prf1	226.848	-0.6069	0.27972	-2.1698	0.03002	0.09182	229.298	302.81	289.33	158.618	251.334	129.697	Down
Ppa1	1784.22	0.49656	0.15672	3.16843	0.00153	0.00797	1220.02	1782.36	1438.05	1987.38	2128.95	2148.54	Up
Col13a1	3063.48	-0.4322	0.16735	-2.5826	0.0098	0.03759	3707.47	3439.19	3409.89	2753.52	3074.09	1996.73	Down
Gm5424	543.674	-0.6738	0.13451	-5.0091	5.47E-07	7.15E-06	621.136	648.741	735.26	403.282	420.298	433.328	Down
Hk1	5085.41	0.25892	0.12545	2.06404	0.03901	0.11232	4211.53	5287.67	4391.51	5322.5	5988.72	5310.53	Up
Hkdc1	1130.19	1.9779	0.18574	10.6487	1.77E-26	2.72E-24	502.133	533.75	337.074	1733.39	1951.53	1723.26	Up
Vps26a	5227.41	0.33692	0.1204	2.79837	0.00514	0.0222	5286.43	4333.25	4239.68	5723.71	6126	5655.38	Up
Srgn	9973.9	-0.3041	0.11428	-2.6606	0.0078	0.03113	10090.1	11979.2	10993.6	8880.51	9708.04	8192.01	Down
Stox1	469.826	-0.3665	0.15585	-2.3516	0.01869	0.06337	503.101	504.044	580.569	414.686	360.105	456.452	Down
Slc25a16	2841.34	0.30253	0.11271	2.68414	0.00727	0.02942	2755.44	2615.09	2263.07	3003.37	3243.05	3168.02	Up
Pbld2	718.97	2.481	0.24967	9.93728	2.87E-23	3.25E-21	254.453	210.817	190.022	1090.63	1680.14	887.769	Up
Pbld1	91.2045	1.09132	0.33031	3.30388	0.00095	0.00536	53.2126	68.0364	53.4734	126.479	161.572	84.4537	Up
Mypn	267.932	-4.4071	0.98546	-4.4721	7.75E-06	7.84E-05	240.908	959.217	335.164	23.8445	4.2241	44.2376	Down
Sirt1	2573.06	-0.294	0.09563	-3.0745	0.00211	0.01041	2866.71	2671.63	2964.91	2292.18	2222.93	2420	Down
Dnajc12	1139.94	-0.4196	0.13581	-3.0896	0.002	0.00999	1414.49	1219.86	1279.54	1020.13	846.932	1058.69	Down
Gm32255	3.40591	3.27139	1.62096	2.01818	0.04357	0.12257	0	1.91652	0	7.25701	4.2241	7.03781	Up
Egr2	660.307	1.75363	0.32663	5.36886	7.92E-08	1.20E-06	337.658	360.305	208.164	935.118	1514.34	606.257	Up
Rtkn2	16974.3	-0.4794	0.11232	-4.2682	1.97E-05	0.00018	20276.9	17605.1	21424.7	14990.9	13253.1	14294.8	Down
Arid5b	6761.23	-0.3233	0.08895	-3.6345	0.00028	0.00186	7065.66	7793.52	7687.76	5960.08	5800.75	6259.63	Down
Cabcoco1	469.978	-0.855	0.33078	-2.5848	0.00974	0.03739	445.051	759.899	611.125	360.777	179.524	463.49	Down
Rhobtb1	1170.19	-0.6843	0.18749	-3.6498	0.00026	0.00176	1515.11	1616.58	1196.47	793.088	790.963	1108.96	Down
Cdk1	312.949	1.23512	0.33725	3.66237	0.00025	0.00168	172.215	268.312	119.36	506.954	283.015	527.835	Up
Ank3	5448.7	0.66037	0.13874	4.75965	1.94E-06	2.24E-05	3567.18	4826.75	4275.01	6115.59	7045.8	6861.86	Up
Slc16a9	1845.93	-0.6182	0.12928	-4.7821	1.73E-06	2.03E-05	2257.18	1986.47	2462.64	1431.7	1589.32	1348.24	Down
Phyhipl	55.637	-0.8017	0.31654	-2.5327	0.01132	0.04231	67.7251	68.9946	75.4357	46.6522	33.7928	41.2214	Down
Bicc1	260.166	0.64615	0.27065	2.38743	0.01697	0.05866	282.51	160.029	166.15	299.611	276.679	376.02	Up
Cisd1	2372.01	-0.453	0.14733	-3.0746	0.00211	0.01041	2548.4	3235.08	2440.68	2151.19	1803.69	2053.03	Down
Ipmk	3235.42	0.27568	0.09241	2.98339	0.00285	0.01347	2934.43	2833.57	3013.61	3339.26	3743.61	3548.06	Up
Rsph14	582.233	-0.7661	0.23797	-3.2195	0.00128	0.00689	643.388	876.807	679.877	476.889	294.631	521.803	Down
Rab36	1523.79	-0.6031	0.17037	-3.5399	0.0004	0.00255	1739.57	1928.97	1844.83	1361.21	921.91	1346.23	Down
Bcr	5170.46	0.25324	0.10052	2.51921	0.01176	0.04366	5075.51	4296.83	4781.1	5739.26	5688.81	5441.23	Up
Specc1l	7813.38	-0.5254	0.07987	-6.5782	4.76E-11	1.33E-09	8896.18	9469.51	9295.78	6387.21	6620.22	6211.37	Down
Snrpd3	1760.28	-0.328	0.11725	-2.7976	0.00515	0.02224	1841.16	1891.6	2145.62	1513.61	1691.75	1477.94	Down
Lrrc75b	83.2509	-1.2673	0.33508	-3.7822	0.00016	0.00112	163.508	97.7424	91.6688	47.6889	49.6332	49.2646	Down
Ggt5	987.288	1.10262	0.1828	6.03198	1.62E-09	3.43E-08	741.106	502.128	638.817	1212.96	1562.92	1265.8	Up
Cabin1	4321.51	0.21319	0.10399	2.05018	0.04035	0.11531	3929.99	3756.37	4321.8	4623.75	4932.69	4364.45	Up
Gstt3	111.583	0.53278	0.23636	2.25412	0.02419	0.07758	95.7826	83.3685	94.5334	143.067	112.995	139.751	Up
Gstt1	6032.99	0.27881	0.09615	2.89971	0.00374	0.01695	5892.08	5157.35	5306.28	6577.96	6376.28	6888	Up
Gstt2	2149.51	0.58679	0.17261	3.39954	0.00067	0.004	2084.97	1608.92	1460.97	2745.22	2813.25	2183.73	Up
Derl3	133.417	0.79952	0.29627	2.69867	0.00696	0.0284	121.905	64.2033	105.992	196.976	162.628	148.799	Up
S100b	32.9317	1.31823	0.5769	2.28504	0.02231	0.07269	13.545	21.0817	21.9623	31.1015	27.4567	82.4429	Up
Pcnt	5436.91	0.47894	0.11853	4.04058	5.33E-05	0.00043	5131.63	4277.67	4218.67	5972.52	6754.34	6266.66	Up
Ybey	334.714	-0.3358	0.15117	-2.2213	0.02633	0.08303	357.976	370.846	391.502	305.831	314.696	267.437	Down
Lss	289.967	1.7945	0.23502	7.63564	2.25E-14	1.04E-12	170.28	125.532	93.5785	422.98	449.867	477.565	Up
Col6a2	9962.44	0.82108	0.13543	6.06274	1.34E-09	2.90E-08	8034.13	6451	7119.61	13784.2	13395.7	10990	Up
Col6a1	17000.1	1.03862	0.14198	7.3153	2.57E-13	1.02E-11	13193.8	9738.78	10463.6	24789.9	22691.9	21122.5	Up
Pcbp3	1391.7	-0.5459	0.1416	-3.8552	0.00012	0.00086	1585.73	1581.13	1788.5	1146.61	1282.01	966.19	Down
Itgb2	8262.12	2.02238	0.25667	7.87939	3.29E-15	1.70E-13	4435.03	3033.85	2323.23	11896.3	17543.7	10340.5	Up
Tspear	9.0824	-1.9266	0.92835	-2.0753	0.03796	0.10999	6.77251	24.9147	11.4586	6.2203	2.11205	3.0162	Down
1700009J(	6.80113	2.60198	1.08969	2.38783	0.01695	0.05861	3.87001	0	1.90977	14.514	8.4482	12.0648	Up
Lrrc3	54.2607	0.82857	0.3817	2.17073	0.02995	0.09172	50.3101	28.7478	38.1953	65.3131	88.7061	54.2916	Up
Gatd3a	1860.05	-0.5516	0.17148	-3.2169	0.0013	0.00695	2077.23	2763.62	1793.27	1576.84	1478.44	1470.9	Down
Trappc10	3689.22	0.21782	0.09786	2.22573	0.02603	0.08223	3562.34	3491.89	3179.76	3917.75	3790.08	4193.53	Up
Gm30122	252.142	-0.5477	0.17153	-3.1931	0.00141	0.00743	269.933	313.351	315.111	201.123	191.141	222.194	Down
Cstb	3612.78	1.00941	0.10434	9.67443	3.87E-22	4.07E-20	2520.34	2313.24	2360.47	4858.05	5198.81	4425.77	Up
Syde1	3900.72	-0.2934	0.11968	-2.4519	0.01421	0.05089	4069.31	3909.7	4909.05	3434.64	3620.05	3461.6	Down
Plpp2	1599.23	0.59536	0.13432	4.43231	9.32E-06	9.22E-05	1336.12	1272.57	1212.7	1965.61	2158.52	1649.86	Up
Mier2	1129.85	-0.3246	0.11847	-2.7399	0.00615	0.02567	1226.79	1247.65	1294.82	1114.47	926.134	969.206	Down
Bsg	37649	0.43041	0.09373	4.59218	4.39E-06	4.70E-05	31192.2	34223.2	30807.4	45690.1	40729.8	43251.3	Up
Hcn2	132.108	0.93082	0.35423	2.62773	0.0086	0.03372	79.3351	132.24	61.1125	211.49	184.804	123.664	Up
Polrmt	1166.74	-0.2366	0.09751	-2.4262	0.01526	0.05379	1306.13	1236.15	1244.21	1064.71	1105.66	1043.61	Down
Palm	1527.35	-0.5055	0.18228	-2.773	0.00555	0.02368	1821.81	1757.45	1797.09	1351.88	1505.89	929.996	Down
Misp	966.015	0.90343	0.18227	4.9565	7.18E-07	9.18E-06	551.476	831.769	635.952	1359.13	1106.71	1311.04	Up
Ptbp1	2027	0.42031	0.12159	3.45675	0.00055	0.00334	1882.76	1509.26	1809.5	2310.84	2236.66	2412.96	Up
Plppr3	287.794	0.73024	0.20827	3.5062	0.00045	0.00286	258.323	194.527	196.706	318.272	332.648	426.29	Up
Gm19810	8.10598	-2.2987	1.1256	-2.0422	0.04113	0.1171	24.1875	5.74955	10.5037	0	3.16808	5.027	Down
Cfd	1100.29	-6.8032	0.96822	-7.0265	2.12E-12	7.23E-11	1466.73	3308.87	1767.49	20.7343	35.9049	2.0108	Down
R3hdm4	3642.37	0.46815	0.09125	5.13036	2.89E-07	4.00E-06	3181.14	3079.84	2908.57	4417.45	4232.55	4034.67	Up
Arid3a	557.586	0.48224	0.1641	2.93871	0.0033	0.01525	431.506	453.256	510.862	571.23	760.338	618.322	Up
Cnn2	19190.9	0.22166	0.10914	2.03097	0.04226	0.11964	17907.5	16912.3	18338.5	20698	22784.8	18504.4	Up
Abca7	3805.32	0.52561	0.11215	4.68655	2.78E-06	3.11E-05	3005.06	2985.93	3367.87	4552.22	4842.93	4077.91	Up
Sbno2	5738.83	0.79151	0.12684	6.24025	4.37E-10	1.03E-08	3726.82	4175.13	4706.62	6786.34	7976.16	7061.94	Up
Cbarp	1069.72	-0.8263	0.15456	-5.346	8.99E-08	1.34E-06	1421.26	1156.62	1525.9	687.343	849.044	778.18	Down
Cirbp	3475.41	-0.3546	0.10759	-3.2955	0.00098	0.0055	3661.99	3944.19	4094.54	2993	3327.54	2831.21	Down
Ndufs7	3246.55	-0.4929	0.15322	-3.2167	0.0013	0.00695	3196.62	4474.11	3716.4	2904.88	2777.35	2409.95	Down
Rps15	20687.7	-0.2752	0.12844	-2.1427	0.03214	0.09657	23508.4	19483.3	24973.1	19638.5	19521.7	17001.3	Down
Apc2	163.402	-0.6974	0.25591	-2.7251	0.00643	0.02662	160.605	225.191	220.578	97.4513	148.9	127.686	Down
Pcsk4	361.347	-0.3557	0.1684	-2.1121	0.03468	0.10251	430.538	436.966	349.487	350.41	303.079	297.599	Down
Reep6	2190.76	-0.5998	0.23101	-2.5966	0.00941	0.03636	2869.61	2288.32	2761.52	1950.06	1120.44	2154.57	Down
Adamtsl5	2630.98	-0.3296	0.10272	-3.2081	0.00134	0.00712	2798.98	3084.63	2906.66	2445.61	2402.46	2147.54	Down
Plk5	386.547	-0.9363	0.24356	-3.8444	0.00012	0.0009	552.443	519.376	451.66	306.868	174.244	314.69	Down
Mknk2	10476.7	-0.2418	0.11617	-2.0819	0.03736	0.10864	12855.2	10568.6	10634.5	10124.6	9586.6	9090.83	Down

Mob3a	2407.62	0.29913	0.09268	3.22768	0.00125	0.00673	2200.1	2046.84	2229.65	2683.02	2741.44	2544.67	Up
Izumo4	1383.18	-0.5043	0.147	-3.4307	0.0006	0.00362	1335.15	1694.2	1838.15	1123.8	1119.39	1188.38	Down
Ap3d1	8015.89	0.21833	0.08913	2.44974	0.0143	0.05111	7437.18	7070.99	7723.09	8887.76	8852.66	8123.64	Up
Lingo3	7.24397	-3.2406	1.23472	-2.6246	0.00868	0.03397	3.87001	22.0399	13.3684	2.07343	2.11205	0	Down
Lsm7	757.862	-0.4509	0.16362	-2.7555	0.00586	0.02473	819.474	752.233	1054.19	619.956	677.968	623.349	Down
Gng7	228.981	-0.4255	0.16912	-2.5162	0.01186	0.04396	272.835	276.937	237.766	190.756	198.533	197.059	Down
Thop1	1241.82	-0.2502	0.12315	-2.0314	0.04221	0.11956	1323.54	1331.02	1393.17	1259.61	1007.45	1136.1	Down
Atcayos	17.3466	-3.7394	0.97041	-3.8534	0.00012	0.00087	10.6425	64.2033	21.9623	1.03672	4.2241	2.0108	Down
Zfr2	396.825	-0.5232	0.15906	-3.2896	0.001	0.0056	500.198	472.422	431.607	325.529	287.239	363.955	Down
Matk	142.022	0.54976	0.23771	2.31268	0.02074	0.06859	137.385	107.325	101.218	187.646	143.619	174.94	Up
Tjp3	4392.96	1.60065	0.17572	9.10889	8.32E-20	7.07E-18	1614.76	2580.59	2340.42	6981.24	6371	6469.75	Up
Mfsd12	482.274	1.13351	0.17511	6.47297	9.61E-11	2.56E-09	337.658	302.81	265.457	668.682	754.002	565.035	Up
Gm48551	51.2835	-1.007	0.3294	-3.057	0.00224	0.01093	68.6926	59.412	77.3455	36.2851	33.7928	32.1728	Down
Smim24	232.286	0.64376	0.24913	2.58402	0.00977	0.03745	216.72	194.527	132.729	332.786	234.438	282.518	Up
Celf5	39.3914	1.66151	0.49426	3.3616	0.00077	0.0045	31.9275	12.4574	12.4135	48.7256	65.4736	65.3511	Up
Ncln	4722.08	0.32254	0.11719	2.75237	0.00592	0.02491	4249.27	4003.6	4336.12	5137.96	5905.29	4700.25	Up
S1pr4	738.34	-0.5981	0.18701	-3.1981	0.00138	0.00733	834.954	825.061	1007.4	546.349	728.658	487.619	Down
Gna15	400.041	1.19637	0.19435	6.15581	7.47E-10	1.68E-08	274.77	223.274	231.082	578.487	645.231	447.403	Up
Tle2	3960.2	0.46011	0.08728	5.27169	1.35E-07	1.96E-06	3174.37	3295.45	3532.11	4628.94	4514.51	4615.8	Up
Tle6	1186.36	1.03929	0.13687	7.59334	3.12E-14	1.43E-12	754.651	734.026	841.252	1436.89	1537.57	1813.74	Up
BC024063	120.934	0.45248	0.21226	2.13172	0.03303	0.09864	109.328	95.8259	101.218	140.993	141.507	136.735	Up
1190007I(	519.883	-0.4067	0.12152	-3.3465	0.00082	0.0047	582.436	586.454	609.215	432.311	440.363	468.517	Down
Txnrd1	9366.49	0.41623	0.16688	2.49411	0.01263	0.04625	10194.6	6835.26	7044.17	10221	11017.5	10886.5	Up
Chst11	1090.89	-0.3077	0.13142	-2.3414	0.01921	0.06467	1167.77	1143.2	1309.14	949.632	1089.82	885.758	Down
Slc41a2	1323.39	1.29734	0.15143	8.56709	1.06E-17	7.79E-16	654.031	753.191	888.996	1826.69	2074.03	1743.37	Up
D10Wsu1(	1253.01	0.24026	0.1019	2.35777	0.01839	0.06252	1220.02	1093.37	1133.45	1323.89	1353.82	1393.49	Up
Washc4	2752.43	0.31559	0.10166	3.10424	0.00191	0.00958	2569.68	2299.82	2488.42	2962.93	2924.13	3269.56	Up
Nuak1	1448.78	-0.3655	0.13182	-2.7728	0.00556	0.02369	1438.67	1706.66	1748.39	1314.56	1343.26	1141.13	Down
Ckap4	2695.8	0.27598	0.10023	2.75364	0.00589	0.02482	2297.82	2504.89	2513.25	3001.29	3105.77	2751.78	Up
Tcp11l2	5406.08	-0.2582	0.11253	-2.2944	0.02177	0.07128	5270.95	6578.45	5816.19	4966.91	4789.08	5014.94	Down
Polr3b	2116.33	0.51221	0.09768	5.24372	1.57E-07	2.26E-06	1738.6	1644.37	1850.56	2404.14	2581.98	2478.31	Up
Rfx4	47.7209	2.41938	0.80802	2.9942	0.00275	0.01307	5.80501	37.3721	1.90977	60.1295	107.715	73.3943	Up
Btbd11	391.778	0.75836	0.20348	3.72687	0.00019	0.00136	350.236	260.646	262.593	540.129	399.178	537.889	Up
Pwp1	1515.97	-0.3214	0.10177	-3.1578	0.00159	0.00822	1708.61	1573.46	1770.35	1387.13	1299.97	1356.29	Down
Bpifc	5.9834	-2.262	1.0487	-2.1569	0.03101	0.09408	5.80501	7.66607	16.233	2.07343	2.11205	2.0108	Down
Syn3	257.124	-0.4214	0.17227	-2.4464	0.01443	0.05146	299.925	284.603	298.878	207.343	200.645	251.35	Down
1810014B	250.313	-0.5501	0.24624	-2.2338	0.02549	0.08092	324.113	342.098	226.307	220.82	156.292	232.248	Down
Hsp90b1	11037.8	0.47225	0.13848	3.41011	0.00065	0.00387	9435.07	8030.21	10276.4	11530.4	12453.7	14500.9	Up
Nt5dc3	1463.19	-0.4044	0.11141	-3.6298	0.00028	0.00189	1571.22	1794.82	1634.76	1172.53	1296.8	1309.03	Down
Stab2	16.8071	1.68176	0.66081	2.54502	0.01093	0.04113	5.80501	7.66607	10.5037	16.5875	20.0645	40.216	Up
1700113H	11.8637	-1.615	0.78143	-2.0667	0.03876	0.11177	7.74001	26.8312	19.0977	7.25701	4.2241	6.03241	Down
Ascl1	81.4801	-2.5726	0.40162	-6.4057	1.50E-10	3.88E-09	108.36	205.067	105.037	25.9179	26.4006	18.0972	Down
Parpbp	35.0411	0.91181	0.39189	2.32672	0.01998	0.06676	22.2525	29.706	21.0074	42.5053	47.5211	47.2538	Up
Dram1	29907	1.30652	0.14242	9.17384	4.56E-20	4.00E-18	20357.2	14179.4	17124.9	41787.9	42908.4	43084.4	Up
Gnptab	2269.75	1.49403	0.15591	9.5829	9.44E-22	9.65E-20	1376.75	1087.62	1103.84	2859.26	3384.56	3806.45	Up
Chpt1	7871.92	-0.3647	0.15399	-2.3685	0.01786	0.06108	8880.7	10555.2	7149.21	6513.69	6853.6	7279.1	Down
Spic	62.928	1.6696	0.42321	3.94506	7.98E-05	0.00062	36.7651	33.5391	20.0525	88.1208	63.3615	135.729	Up
Arl1	9438.62	0.21765	0.07097	3.06676	0.00216	0.01065	8857.48	8735.49	8591.08	10308.1	10075.5	10064.1	Up
Ano4	24.7214	-3.9112	0.77686	-5.0347	4.79E-07	6.32E-06	30.96	77.619	30.5563	3.11015	1.05603	5.027	Down
Gas2l3	177.136	1.78361	0.22156	8.05029	8.26E-16	4.65E-14	87.0751	82.4102	69.7065	277.84	311.527	234.258	Up
Nr1h4	26.5442	1.00981	0.48544	2.08019	0.03751	0.10896	20.3175	19.1652	13.3684	47.6889	29.5687	29.1566	Up
Scyl2	2949.02	0.45225	0.09329	4.84794	1.25E-06	1.50E-05	2627.73	2394.69	2449.27	3236.63	3487	3498.8	Up
1500026H	27.7436	-0.9449	0.44298	-2.1331	0.03292	0.09839	40.6351	37.3721	31.5111	17.6242	23.2326	16.0864	Down
Actr6	908.398	-0.3148	0.11059	-2.8463	0.00442	0.01955	1042	996.589	982.574	795.161	865.941	768.126	Down
Uhrf1bp1l	5440.04	0.54694	0.09823	5.56814	2.57E-08	4.30E-07	4658.52	4437.7	4167.11	6212	6232.66	6932.24	Up
Apaf1	1820.6	0.51993	0.13841	3.75641	0.00017	0.00123	1697.97	1329.1	1460.97	2093.13	2371.83	1970.59	Up
Ikbip	1404.61	-0.2448	0.11349	-2.1568	0.03102	0.09408	1604.12	1453.68	1512.53	1398.53	1270.4	1188.38	Down
Slc25a3	22582.5	-0.2453	0.12377	-1.9822	0.04745	0.13103	22498.3	28318.5	22677.5	21166.6	21393	19441.4	Down
Tmpo	7870.99	-0.2872	0.08214	-3.4958	0.00047	0.00296	8563.36	8284.15	9107.67	7223.84	7035.24	7011.67	Down
Nedd1	1453.56	-0.241	0.1036	-2.3267	0.01998	0.06676	1489.95	1633.83	1600.38	1256.5	1341.15	1399.52	Down
Cfap54	1857.31	-0.5095	0.24787	-2.0557	0.03981	0.11407	1979.51	2514.47	2052.04	1554.04	973.655	2070.12	Down
Cdk17	3788.52	-0.2357	0.10725	-2.1975	0.02798	0.08713	3775.19	4048.64	4467.9	3304.01	3670.74	3464.61	Down
Elk3	615.497	-0.3403	0.14665	-2.3205	0.02031	0.06752	595.981	699.529	767.726	511.101	558.637	560.008	Down
Amdhd1	20.8209	-1.9223	0.88247	-2.1783	0.02939	0.09049	22.2525	43.1216	33.4209	14.514	11.6163	0	Down
Ntn4	4828.77	-0.5185	0.1251	-4.1445	3.40E-05	0.00029	5911.43	4893.83	6256.39	3810.97	4226.21	3873.81	Down
Gm3571	565.021	1.86815	0.14623	12.7751	2.26E-37	8.86E-35	249.615	261.605	217.713	851.144	971.543	838.504	Up
Fgd6	2106.52	0.57344	0.12669	4.52636	6.00E-06	6.23E-05	1937.91	1519.8	1622.35	2492.26	2424.63	2642.19	Up
Ndufa12	1836.14	-0.313	0.14014	-2.2337	0.0255	0.08092	1773.43	2385.11	1945.1	1717.84	1618.89	1576.47	Down
2310039L	22.0106	-1.3095	0.66187	-1.9785	0.04787	0.13199	33.8626	36.4138	23.8721	18.6609	3.16808	16.0864	Down
Socs2	7388.3	0.46613	0.12375	3.76671	0.00017	0.00118	6403.89	6312.05	5899.27	9424.78	7325.65	8964.15	Up
Mrpl42	2292.52	-0.4352	0.15456	-2.8156	0.00487	0.02123	2186.55	3168	2552.4	2056.84	1907.18	1884.12	Down
Gm47718	4.08218	3.55752	1.75122	2.03145	0.04221	0.11956	0	1.91652	0	13.4773	1.05603	8.04321	Up
Btg1	9847.16	0.25617	0.09992	2.56368	0.01036	0.03935	8911.66	9176.28	8837.44	11369.7	11142.1	9645.82	Up
Dcn	8080.14	-1.6142	0.21663	-7.4516	9.22E-14	3.93E-12	13249	14552.1	8742.91	4723.28	4106.88	3106.69	Down
Lum	843.576	-1.4137	0.31962	-4.4231	9.73E-06	9.56E-05	929.769	1955.81	794.463	488.293	531.181	361.944	Down
Gm48768	127.373	-0.6413	0.21222	-3.022	0.00251	0.01209	147.06	165.779	152.781	97.4513	106.659	94.5077	Down
B530045E	212.662	-1.712	0.29447	-5.8139	6.10E-09	1.16E-07	257.355	294.185	425.878	85.0107	134.115	79.4267	Down
Kitl	12207	-0.4162	0.13456	-3.0929	0.00198	0.0099	12332.7	13646.6	15887.3	9373.98	10528.6	11472.6	Down
Nts	29.5507	-3.2859	0.76653	-4.2868	1.81E-05	0.00017	12.5775	68.9946	79.2553	6.2203	4.2241	6.03241	Down
Rassf9	2531.2	-0.4843	0.13168	-3.6782	0.00023	0.0016	2561.94	3251.37	3043.21	2208.2	1915.63	2206.85	Down
Alx1	26.0928	-1.3003	0.50188	-2.5908	0.00958	0.03685	38.7001	40.2469	32.466	16.5875	8.4482	20.108	Down
Slc6a15	377.198	-0.5366	0.19692	-2.7251	0.00643	0.02662	486.653	409.176	444.021	253.995	288.295	381.047	Down
Gm36283	307.804	-0.637	0.16458	-3.8702	0.00011	0.00082	360.878	352.639	410.6	222.894	233.382	266.431	Down

Csrp2	2402.08	-0.8377	0.14613	-5.7327	9.89E-09	1.79E-07	2751.57	3460.27	3029.84	1918.96	1475.27	1776.54	Down
Zdhhc17	2302.5	-0.2827	0.12085	-2.3393	0.01932	0.06498	2446.81	2436.85	2698.5	1823.58	2193.36	2215.9	Down
Gm40761	26.1788	-1.788	0.62305	-2.8698	0.00411	0.01837	22.2525	53.6625	45.8344	17.6242	12.6723	5.027	Down
Nap1l1	15210.9	-0.4056	0.09269	-4.3762	1.21E-05	0.00012	17327	17066.6	17611.9	13229.5	13996.6	12033.6	Down
Gm5176	45.5446	-1.3662	0.39772	-3.4351	0.00059	0.00358	73.5301	53.6625	69.7065	16.5875	30.6247	29.1566	Down
Phlda1	1885.18	0.91595	0.18499	4.9515	7.36E-07	9.41E-06	1169.71	1393.31	1354.98	2334.68	3108.94	1949.47	Up
Glipr1	1685.64	0.51395	0.15937	3.22489	0.00126	0.00678	1229.69	1539.92	1396.04	2220.65	1625.22	2102.29	Up
Glipr1l3	33.5375	1.45867	0.52874	2.75873	0.0058	0.02454	11.61	22.0399	20.0525	45.6155	28.5127	73.3943	Up
Kcnc2	156.513	2.70435	0.33739	8.01554	1.10E-15	6.08E-14	55.1476	28.7478	41.06	355.594	190.085	268.442	Up
Gm31182	4.23517	3.60861	1.65387	2.18192	0.02912	0.08986	1.935	0	0	8.29373	2.11205	13.0702	Up
Tph2	11.5767	2.05612	0.7749	2.65341	0.00797	0.0317	5.80501	5.74955	1.90977	14.514	26.4006	15.081	Up
Tbc1d15	4102.7	0.25817	0.11465	2.25183	0.02433	0.07794	4220.24	3476.56	3513.01	4281.64	4608.49	4516.26	Up
Rab21	6812.66	0.27288	0.08719	3.12964	0.00175	0.00891	6350.68	6101.23	6058.73	7334.76	7923.36	7107.18	Up
Tmem19	2290.35	0.2115	0.09184	2.30298	0.02128	0.07002	2036.59	2217.41	2114.11	2473.6	2538.68	2361.69	Up
Ptprb	30827.4	-0.4718	0.11386	-4.1435	3.42E-05	0.00029	35004.2	31993.4	40472.7	26420.7	26024.7	25048.6	Down
Kcnmb4	88.1341	-0.9877	0.27053	-3.6509	0.00026	0.00175	132.548	114.033	105.037	62.203	49.6332	65.3511	Down
Kcnmb4os	14.3398	-1.66	0.67482	-2.4599	0.0139	0.04998	28.0575	13.4156	23.8721	9.33044	6.33615	5.027	Down
5330438D	176.266	-0.7368	0.24345	-3.0266	0.00247	0.01193	261.225	170.57	229.172	153.434	123.555	119.643	Down
Lrrc10	108.986	-6.7394	1.80696	-3.7297	0.00019	0.00135	57.0826	465.714	125.09	0	0	6.03241	Down
Yeats4	2811.99	0.24349	0.08379	2.90596	0.00366	0.01666	2583.23	2536.51	2605.88	3046.91	3159.63	2939.79	Up
Lyz2	571618	1.79216	0.23378	7.66602	1.77E-14	8.30E-13	365884	192894	209643	851191	1021058	789036	Up
Gm48903	25.205	-3.4982	0.76595	-4.5672	4.94E-06	5.23E-05	22.2525	71.8694	44.8795	2.07343	2.11205	8.04321	Down
Cpm	9462.14	0.53732	0.09876	5.44076	5.31E-08	8.38E-07	8201.51	6987.62	7971.36	11031.7	11542.4	11038.3	Up
Slc35e3	2288.64	-0.3328	0.13517	-2.462	0.01382	0.04977	2580.33	2452.18	2622.11	2124.23	1693.86	2259.14	Down
Gm40773	72.564	-0.9789	0.28645	-3.4175	0.00063	0.00378	83.2051	93.9093	111.721	49.7624	47.5211	49.2646	Down
Mdm1	1225.73	-0.6419	0.18197	-3.5274	0.00042	0.00266	1273.23	1769.9	1439.01	1028.42	774.067	1069.75	Down
Gm38403	15.4676	-1.5403	0.69477	-2.217	0.02662	0.08373	20.3175	11.4991	37.2404	7.25701	8.4482	8.04321	Down
Gm47480	19.6713	-3.2304	0.65801	-4.9094	9.13E-07	1.14E-05	41.6026	33.5391	31.5111	2.07343	5.28013	4.0216	Down
Gm34045	63.9811	-0.6933	0.29396	-2.3584	0.01835	0.06244	80.3026	75.7024	81.165	57.0194	46.4651	43.2322	Down
Grip1os3	7.28225	-2.3362	0.95967	-2.4343	0.01492	0.05284	9.67501	17.2487	9.54883	1.03672	3.16808	3.0162	Down
Helb	236.496	0.48709	0.21591	2.25592	0.02408	0.07731	236.07	150.447	204.345	260.216	290.407	277.491	Up
Tmbim4	4385.28	0.31461	0.10078	3.12163	0.0018	0.00912	3890.32	4000.73	3835.76	5124.49	5065.75	4394.61	Up
Llph	1935.56	-0.2393	0.10402	-2.3005	0.02142	0.07034	2084	2033.42	2169.49	1856.76	1846.99	1622.72	Down
4921513I(	25.0111	-1.7683	0.5452	-3.2433	0.00118	0.00641	22.2525	49.8294	43.9246	12.4406	10.5603	11.0594	Down
Msrb3	5316.78	-0.4626	0.11488	-4.0267	5.66E-05	0.00046	5418.01	6295.76	6772.03	4306.52	4623.28	4485.09	Down
Wif1	445.592	-1.4791	0.21459	-6.8927	5.47E-12	1.76E-10	842.694	577.83	547.148	214.6	265.062	226.215	Down
Gns	18779.9	0.94892	0.10451	9.07995	1.09E-19	9.19E-18	13844.9	13077.4	11529.3	23785.4	25891.6	24550.9	Up
D930020E	208.789	-2.0873	0.24848	-8.4	4.46E-17	2.99E-15	374.423	307.601	332.299	81.9006	55.9693	100.54	Down
Gm35865	18.4364	-2.5077	0.60596	-4.1384	3.50E-05	0.0003	28.0575	30.6643	35.3307	4.14686	7.39218	5.027	Down
Gm48877	13.5726	-2.7621	1.12483	-2.4556	0.01407	0.05048	9.67501	39.2886	21.9623	0	9.50423	1.0054	Down
Ppm1h	1820.62	0.67907	0.09399	7.22527	5.00E-13	1.90E-11	1365.14	1455.59	1378.85	2140.82	2321.14	2262.15	Up
D630033A	77.8481	-0.9983	0.29861	-3.3431	0.00083	0.00475	123.84	84.3268	103.127	44.5788	54.9133	56.3024	Down
Usp15	3715.16	0.22574	0.08638	2.61343	0.00896	0.03488	3296.28	3418.11	3560.76	3867.99	4153.35	3994.46	Up
Tspan31	3171.87	0.2923	0.08641	3.38265	0.00072	0.00422	2987.64	2781.82	2785.39	3520.69	3571.48	3384.18	Up
Os9	10652.9	0.32073	0.08644	3.71033	0.00021	0.00143	9666.31	8893.6	9861.08	12261.2	11589.9	11645.6	Up
B4galnt1	4364.34	1.14669	0.11003	10.4212	1.98E-25	2.80E-23	2657.73	2993.6	2496.06	6380.99	5747.95	5909.75	Up
Slc26a10	473.037	0.54592	0.27386	1.99344	0.04621	0.12825	256.388	444.632	452.614	587.818	689.585	407.187	Up
Arhgef25	1882.23	-0.4173	0.12102	-3.4481	0.00056	0.00343	2091.74	2127.33	2238.25	1611.06	1797.36	1427.67	Down
Pip4k2c	431.686	0.81301	0.19302	4.21216	2.53E-05	0.00022	372.488	256.813	310.337	566.047	474.155	610.278	Up
Arhgap9	1140.36	0.59335	0.17299	3.42992	0.0006	0.00363	992.657	943.885	790.643	1284.49	1662.18	1168.28	Up
Ndufa4l2	1025.77	-0.7814	0.15978	-4.8901	1.01E-06	1.24E-05	1134.88	1319.52	1436.14	745.399	870.165	648.484	Down
Stat6	9644.07	0.3208	0.08836	3.63051	0.00028	0.00189	8357.28	8401.05	8970.17	10290.4	11370.2	10475.3	Up
Myo1a	43.6811	0.97608	0.42166	2.31483	0.02062	0.06833	31.9275	30.6643	25.7818	70.4967	34.8488	68.3673	Up
Gpr182	2432.57	-0.5363	0.18635	-2.8782	0.004	0.01797	3206.3	2178.12	3254.24	1904.45	2296.86	1755.43	Down
Sdr9c7	25.6789	-1.2537	0.46617	-2.6893	0.00716	0.02904	36.7651	37.3721	34.3758	18.6609	15.8404	11.0594	Down
Hsd17b6	3.09307	4.09163	1.90602	2.14669	0.03182	0.09588	0	0.95826	0	13.4773	2.11205	2.0108	Up
Atp5b	37167.2	-0.3684	0.1708	-2.1567	0.03103	0.09409	37312.7	53758.3	34588.7	32663.8	33103.2	31576.6	Down
Stat2	3354.46	0.49381	0.10844	4.5536	5.27E-06	5.54E-05	2712.87	2909.27	2735.74	3577.71	3883.01	4308.14	Up
Cnpy2	2962.29	0.21096	0.0889	2.37315	0.01764	0.06043	2786.4	2822.07	2629.75	3312.31	3162.8	3060.44	Up
Coq10a	2175.01	-0.3875	0.16002	-2.4216	0.01545	0.05434	2214.61	3059.72	2121.75	1869.2	1896.62	1888.14	Down
Ankrd52	550.787	0.44313	0.17766	2.49424	0.01262	0.04625	467.303	396.719	536.644	565.01	605.103	733.943	Up
Rnf41	2474.96	0.34909	0.08863	3.93877	8.19E-05	0.00064	2226.22	2155.12	2149.44	2747.3	2909.35	2662.3	Up
Esyt1	7474.01	0.36657	0.12502	2.93199	0.00337	0.01555	7264.97	5924.91	6398.67	8356.97	9264.51	7634.01	Up
Ikzf4	218.935	-0.6214	0.22981	-2.704	0.00685	0.02805	201.24	275.978	318.931	168.985	159.46	189.015	Down
Suox	1187.28	-0.2979	0.1141	-2.6113	0.00902	0.03506	1216.15	1405.77	1306.28	1126.91	1058.14	1010.43	Down
Rab5b	6151.41	0.37248	0.13956	2.6689	0.00761	0.03053	6398.09	4803.75	4883.27	6753.17	7353.1	6717.08	Up
Cdk2	1441.6	0.47005	0.1237	3.80008	0.00014	0.00105	1301.29	1062.71	1262.36	1705.4	1741.39	1576.47	Up
Dnajc14	3414.19	0.34062	0.11946	2.8513	0.00435	0.0193	3250.8	2636.17	3152.07	3576.67	3967.49	3901.96	Up
Gdf11	106.007	0.82592	0.31172	2.64953	0.00806	0.03201	60.9526	107.325	61.1125	117.149	145.732	143.772	Up
Cd63	15696.9	0.86935	0.11936	7.28356	3.25E-13	1.27E-11	12073.5	10575.3	10668	20644.1	22280	17940.4	Up
Itga7	4924.9	1.92341	0.17803	10.8038	3.30E-27	5.65E-25	1812.13	2519.26	1833.38	7554.55	9187.42	6642.68	Up
Vmn2r87	17.749	1.49393	0.63253	2.36185	0.01818	0.06196	14.5125	9.58259	3.81953	24.8812	29.5687	24.1296	Up
Gm35405	13.5031	1.3324	0.65321	2.03977	0.04137	0.11764	4.83751	7.66607	10.5037	19.6976	23.2326	15.081	Up
Gm12592	378.166	-0.7823	0.17202	-4.5476	5.43E-06	5.69E-05	424.733	493.503	516.592	239.481	283.015	311.674	Down
Pik3ip1	2226.5	-0.303	0.13175	-2.2997	0.02146	0.07046	2649.99	2617	2111.25	1918.96	2159.57	1902.22	Down
Limk2	6325.76	0.39126	0.11449	3.41735	0.00063	0.00378	5100.67	5937.37	5381.72	7345.13	6469.21	7720.47	Up
Selenom	1126.89	-0.7405	0.16963	-4.3657	1.27E-05	0.00012	1488.02	1355.94	1385.54	935.118	947.255	649.489	Down
Smtn	2821.14	-0.958	0.16084	-5.9564	2.58E-09	5.26E-08	2992.48	4532.56	3649.56	1931.4	1894.51	1926.35	Down
Osbp2	94.4581	-2.4477	0.42693	-5.7332	9.86E-09	1.79E-07	118.035	258.73	102.172	25.9179	32.7368	29.1566	Down
Gal3st1	952.649	0.44204	0.15332	2.88305	0.00394	0.01774	816.571	810.687	796.372	1257.54	897.622	1137.11	Up
Sec14l2	2193.38	1.39045	0.11929	11.6559	2.14E-31	4.81E-29	1324.51	1243.82	1065.65	3118.44	3070.92	3336.93	Up
Ccdc157	3675.12	-0.3117	0.14343	-2.1733	0.02976	0.0913	4024.81	3839.74	4347.58	3381.77	2708.71	3748.13	Down
Osm	780.961	3.19633	0.67999	4.70055	2.59E-06	2.92E-05	227.363	160.987	72.5711	1431.7	2204.98	588.16	Up

Lif	692.804	1.55681	0.26266	5.92714	3.08E-09	6.23E-08	275.738	384.262	394.367	964.146	1422.47	715.845 Up
Ascc2	5120.95	0.25927	0.09252	2.80233	0.00507	0.02199	4441.8	4633.18	4910.96	5351.53	5894.73	5493.51 Up
Uqcr10	1511.76	-0.2285	0.11421	-2.0006	0.04543	0.12649	1481.24	1749.78	1662.45	1397.49	1460.48	1319.09 Down
Nefh	36.9459	1.67761	0.39925	4.20195	2.65E-05	0.00023	17.415	19.1652	16.233	52.8275	49.6332	66.3565 Up
Ap1b1	5102.68	0.57064	0.08312	6.86529	6.64E-12	2.12E-10	4199.92	4133.93	3985.68	6365.44	5872.56	6058.55 Up
Gas2l1	4370.19	0.50843	0.10891	4.66826	3.04E-06	3.38E-05	3629.1	3683.55	3511.1	5252	5576.87	4568.54 Up
Xbp1	16234.1	0.25102	0.11057	2.27031	0.02319	0.0751	13745.3	14000.2	16730.5	17696.7	18200.6	17031.5 Up
Urgcp	1359.16	0.22129	0.10341	2.14005	0.03235	0.097	1315.8	1226.57	1223.2	1452.44	1387.62	1549.32 Up
Dbnl	6658.9	0.91482	0.12724	7.18958	6.50E-13	2.41E-11	4902.33	4379.24	4565.3	8639.99	9892.85	7573.68 Up
Pgam2	1275.88	-1.4615	0.62695	-2.3311	0.01975	0.06615	1041.03	3548.43	1026.5	698.746	825.812	514.765 Down
Polm	1307.82	0.43245	0.11124	3.88736	0.0001	0.00077	1033.29	1159.49	1146.81	1532.27	1574.53	1400.52 Up
Myl7	7096.79	-5.4179	1.14809	-4.7191	2.37E-06	2.69E-05	8983.25	23256	9368.36	218.747	28.5127	725.899 Down
Ykt6	5192.17	0.24559	0.09526	2.57799	0.00994	0.03803	5022.3	4816.21	4415.38	5438.61	5892.62	5567.91 Up
Ppia	27666.5	0.22866	0.07818	2.92489	0.00345	0.01585	25746.2	24806.4	25883.1	31282.9	29301.5	28978.7 Up
Myo1g	1992.87	0.72327	0.13032	5.54998	2.86E-08	4.73E-07	1585.73	1628.08	1296.73	2437.32	2672.8	2336.55 Up
Nacad	224.172	-0.4413	0.20703	-2.1315	0.03305	0.09869	237.038	227.107	310.337	205.27	195.365	169.913 Down
Ramp3	420.343	-1.085	0.20039	-5.4143	6.15E-08	9.53E-07	548.573	610.411	554.787	262.289	336.872	209.123 Down
Adcy1	185.974	-2.2356	0.29175	-7.6626	1.82E-14	8.51E-13	322.178	385.22	212.939	55.9827	79.2019	60.3241 Down
Igfbp3	5134.63	-2.0271	0.2412	-8.4043	4.30E-17	2.90E-15	7833.86	6211.43	10692.8	2521.29	2104.66	1443.76 Down
Tns3	13878.5	-0.2685	0.08114	-3.3085	0.00094	0.00529	14898.6	14756.2	15843.4	13040.8	12162.2	12569.5 Down
Gm11992	746.455	-0.714	0.24302	-2.9379	0.0033	0.01528	824.311	1049.29	909.049	618.919	364.329	712.829 Down
Abca13	46.0927	-1.1383	0.43651	-2.6078	0.00911	0.03536	70.6276	69.9529	49.6539	16.5875	28.5127	41.2214 Down
Vwc2	75.0732	-1.5898	0.37578	-4.2308	2.33E-05	0.00021	119.003	139.906	79.2553	48.7256	25.3446	38.2052 Down
Spata48	47.367	-1.238	0.42291	-2.9274	0.00342	0.01575	52.2451	82.4102	64.932	16.5875	34.8488	33.1782 Down
Ikzf1	1618.25	0.38884	0.1154	3.36954	0.00075	0.00439	1443.51	1412.47	1348.29	1735.46	2044.47	1725.27 Up
Fignl1	124.897	1.02389	0.29964	3.417	0.00063	0.00379	106.425	86.2433	54.4283	144.104	173.188	184.994 Up
Ddc	157.234	-1.6217	0.27674	-5.8602	4.62E-09	9.07E-08	180.923	315.267	215.804	74.6435	82.37	74.3997 Down
Grb10	3016.53	-0.6061	0.14968	-4.0495	5.13E-05	0.00042	2996.35	3595.39	4331.35	2446.65	2328.54	2400.9 Down
Cobl	3803.28	0.55757	0.11436	4.87555	1.09E-06	1.33E-05	3281.76	2893.94	3056.58	4337.62	4216.71	5033.04 Up
Vstm2a	309.525	2.66341	0.41933	6.35161	2.13E-10	5.35E-09	138.353	79.5355	35.3307	403.282	738.162	462.484 Up
Akt2-ps	306.258	0.68587	0.2253	3.04418	0.00233	0.01134	287.348	239.565	177.608	380.475	325.256	427.295 Up
Gm12663	277.26	0.5105	0.21003	2.43061	0.01507	0.05326	278.64	190.693	216.758	341.08	355.881	280.507 Up
Egfr	1644.96	-1.0105	0.1948	-5.1873	2.13E-07	3.00E-06	1571.22	2444.52	2580.09	1073	1029.62	1171.29 Down
Plek	5186.61	1.65012	0.19088	8.64492	5.38E-18	4.03E-16	2847.36	2637.13	2034.86	6698.22	9905.52	6996.58 Up
Cnrip1	428.219	-0.4802	0.14389	-3.3375	0.00085	0.00483	476.978	483.921	535.689	327.602	359.049	386.074 Down
Ppp3r1	75.6017	0.6734	0.27382	2.45924	0.01392	0.05005	62.8876	59.412	52.5186	81.9006	101.378	95.5131 Up
Etaa1os	15.5341	-1.2984	0.61364	-2.116	0.03435	0.10174	21.285	17.2487	27.6916	9.33044	11.6163	6.03241 Down
Meis1	1854.41	-0.3071	0.14842	-2.0689	0.03855	0.11133	1996.92	1962.51	2193.37	1471.1	1986.38	1516.14 Down
Spred2	2088.09	-0.2972	0.13461	-2.2077	0.02726	0.08529	2118.83	2277.78	2510.39	1662.89	2125.78	1832.85 Down
Actr2	15262.4	0.406	0.08918	4.5524	5.30E-06	5.57E-05	12984.8	12544.6	13857.3	16681.8	18335.8	17170.2 Up
Slc1a4	3497.08	-0.7193	0.19907	-3.6131	0.0003	0.002	3404.64	4368.7	5280.5	2727.6	2099.38	3101.66 Down
Lgalsl	2651.28	-0.4156	0.07977	-5.2107	1.88E-07	2.68E-06	3026.34	2988.81	3076.63	2260.04	2251.45	2304.38 Down
Mdh1	12913.5	-0.5905	0.22516	-2.6227	0.00872	0.03413	12028.9	22192.3	12339	10783.9	9891.79	10245 Down
Otx1	136.071	-1.7559	0.61656	-2.8479	0.0044	0.01947	103.523	373.721	152.781	49.7624	19.0085	117.632 Down
Ehbp1	1471.12	-0.4351	0.18019	-2.4145	0.01576	0.05522	1507.37	2165.66	1400.81	1213.99	1242.94	1295.96 Down
Tmem17	342.701	-0.3544	0.16576	-2.1377	0.03254	0.09742	349.268	384.262	420.148	339.006	283.015	280.507 Down
Pus10	1579.35	0.41356	0.09124	4.53246	5.83E-06	6.06E-05	1355.47	1361.69	1346.38	1727.17	1857.55	1827.82 Up
Rel	1151.31	0.34434	0.14531	2.36963	0.01781	0.06091	1149.39	1014.8	879.447	1217.1	1423.52	1223.57 Up
Ccdc85a	2385.78	-0.3944	0.11132	-3.5433	0.0004	0.00253	2655.79	2762.66	2710.91	2124.23	2229.27	1831.84 Down
Efemp1	8257.72	-0.2507	0.12368	-2.0267	0.04269	0.12057	9932.37	8406.8	8580.58	8390.14	7423.86	6812.6 Down
A630052C	367.635	-0.4896	0.14508	-3.3745	0.00074	0.00432	413.123	404.385	470.757	309.978	299.911	307.653 Down
Ccdc88a	1468.91	0.36797	0.12056	3.05215	0.00227	0.0111	1310.96	1217.95	1318.69	1553	1856.49	1556.36 Up
Gm12092	4.18538	-3.4985	1.57471	-2.2216	0.02631	0.08297	12.5775	5.74955	4.77441	0	0	2.0108 Down
Sptbn1	65420.3	-0.2392	0.08933	-2.6778	0.00741	0.02988	70011.3	66591.3	75892.2	59171.6	62126	58729.5 Down
Gm8098	213.126	-0.4395	0.21105	-2.0824	0.03731	0.10854	198.338	260.646	276.916	185.572	196.421	160.864 Down
Acyp2	942.175	-0.5837	0.14701	-3.9705	7.17E-05	0.00057	1014.91	1305.15	1070.42	783.757	794.131	684.678 Down
Psme4	5397.53	0.25508	0.09288	2.74625	0.00603	0.02526	4834.6	5282.88	4647.42	5679.13	5914.8	6026.37 Up
Stc2	534.883	1.46424	0.27007	5.42175	5.90E-08	9.20E-07	266.063	376.596	211.029	1011.83	766.674	577.1 Up
Bod1	2424.63	-0.3575	0.11012	-3.2463	0.00117	0.00636	2709.97	2792.37	2667.94	2319.13	2151.12	1907.25 Down
Cpeb4	5745.49	0.48702	0.09237	5.27243	1.35E-07	1.95E-06	4500.82	4924.49	4929.11	6330.19	6947.59	6840.75 Up
Nsg2	134.136	-0.8162	0.28912	-2.823	0.00476	0.02082	131.58	220.399	161.375	108.855	76.0338	106.572 Down
Rhbdf1	3955.58	-0.2644	0.10381	-2.5466	0.01088	0.04098	4335.37	3963.36	4652.19	3785.05	3458.48	3539.01 Down
Hba-a2	3014.2	-2.8163	0.8095	-3.4791	0.0005	0.00312	8371.79	3434.4	4030.56	337.969	1556.58	353.901 Down
Sh3pxd2b	2027.95	0.93283	0.155	6.0184	1.76E-09	3.69E-08	1131.01	1607	1444.74	2485.01	2609.44	2890.53 Up
Ubtd2	226.068	0.68257	0.23305	2.92889	0.0034	0.01569	210.915	150.447	159.465	268.509	231.27	335.804 Up
Stk10	1526.72	0.4719	0.16162	2.91987	0.0035	0.01607	1486.08	1258.19	1093.34	1690.88	2041.3	1590.54 Up
Fgf18	418.699	0.5562	0.25294	2.19897	0.02788	0.08687	441.181	241.481	334.209	509.027	587.15	399.144 Up
Gabrp	9558.5	-0.512	0.25188	-2.0327	0.04209	0.11934	8664.94	13983.9	11062.3	8182.8	5159.74	10297.3 Down
4930469K	56.9505	0.92183	0.32743	2.81536	0.00487	0.02124	34.8301	46.9547	36.2855	81.9006	62.3055	79.4267 Up
Dock2	1875.33	0.74743	0.17815	4.19549	2.72E-05	0.00024	1671.84	1343.48	1185.01	2158.44	2809.03	2084.2 Up
Spdl1	154.893	0.99134	0.21915	4.52357	6.08E-06	6.30E-05	104.49	94.8676	111.721	179.352	200.645	238.28 Up
Slit3	1442.97	-0.5381	0.1614	-3.3339	0.00086	0.00488	1807.29	1693.24	1626.17	1237.84	1362.27	931.001 Down
Pank3	6160.4	0.32842	0.101	3.25176	0.00115	0.00627	5761.47	5483.16	5142.04	6339.52	7292.91	6943.3 Up
Wwc1	14747.5	0.29452	0.106	2.77839	0.00546	0.02333	12887.1	12122	14732.9	16083.6	16817.2	15842.1 Up
Hmmr	103.243	1.43667	0.32807	4.3791	1.19E-05	0.00011	59.9851	68.0364	39.1502	133.736	122.499	196.053 Up
Nudcd2	1409.58	0.21077	0.1013	2.08068	0.03746	0.1089	1375.79	1279.28	1265.22	1542.63	1561.86	1432.7 Up
Ccng1	10617.1	0.39764	0.18593	2.13867	0.03246	0.09725	8618.5	11781.8	7089.05	11554.2	13267.9	11391.2 Up
C1qtnf2	927.459	-0.6617	0.19458	-3.4006	0.00067	0.00399	981.046	1331.98	1096.21	739.178	854.325	562.019 Down
Ccnjl	97.1898	-1.6431	0.42829	-3.8364	0.00012	0.00092	67.7251	175.361	198.616	57.0194	41.185	43.2322 Down
4933415A	10.0249	-2.1109	0.85812	-2.4599	0.0139	0.04998	8.70751	14.3739	25.7818	4.14686	2.11205	5.027 Down
Ttc1	2304.59	-0.2099	0.08148	-2.5758	0.01	0.03823	2497.12	2438.77	2479.83	2165.7	2145.84	2100.28 Down
Adra1b	177.892	-1.0783	0.41466	-2.6004	0.00931	0.03604	227.363	354.556	142.278	101.598	173.188	68.3673 Down
Il12b	59.2593	2.00061	0.36153	5.53379	3.13E-08	5.17E-07	29.9925	19.1652	21.9623	110.929	74.9778	98.5293 Up

Rnf145	7649.15	0.47013	0.13891	3.38434	0.00071	0.0042	7565.86	5445.78	6229.66	9170.79	8995.22	8487.59	Up
Ebf1	941.589	-0.6838	0.16131	-4.2393	2.24E-05	0.0002	1059.41	1332.94	1089.52	622.03	824.756	720.872	Down
Clint1	8269.69	0.79982	0.0773	10.3475	4.30E-25	5.79E-23	6128.15	6101.23	5873.48	10356.8	10881.3	10277.2	Up
Adam19	733.527	1.66246	0.16014	10.3815	3.01E-25	4.11E-23	372.488	386.178	297.923	981.77	1218.65	1144.15	Up
Cyfip2	2756.25	-0.3954	0.1951	-2.0265	0.04272	0.12062	3009.9	3722.83	2662.21	2532.7	1805.8	2804.06	Down
Havcr2	725.365	1.81398	0.27105	6.69254	2.19E-11	6.52E-10	466.336	244.356	253.044	1018.05	1398.18	972.223	Up
Timd4	117.427	-0.612	0.29647	-2.0643	0.03899	0.11226	179.955	128.407	117.451	95.3779	112.995	70.3781	Down
Gm12183	1848.74	0.35561	0.13306	2.6726	0.00753	0.03027	1688.29	1579.21	1598.47	2364.75	2075.09	1786.6	Up
Gm12184	276.542	-0.4186	0.17091	-2.4493	0.01431	0.05115	272.835	338.265	338.029	245.702	229.158	235.264	Down
Irgm1	3146.91	0.66337	0.11638	5.70028	1.20E-08	2.13E-07	2643.21	2378.4	2285.99	3677.23	4262.12	3634.52	Up
Gm5431	465.863	1.884	0.25558	7.37152	1.69E-13	6.96E-12	146.093	275.978	173.789	797.234	598.766	803.315	Up
Olfr1392	6.42077	2.00803	0.98923	2.02989	0.04237	0.11987	1.935	1.91652	3.81953	14.514	5.28013	11.0594	Up
Cnot6	6976.4	0.23341	0.07771	3.00349	0.00267	0.01274	6263.6	6336.01	6640.26	7406.3	7476.66	7735.55	Up
Gfpt2	919.674	-1.3005	0.16544	-7.8607	3.82E-15	1.96E-13	1131.98	1447.93	1344.48	552.57	597.71	443.382	Down
Rasgef1c	25.8143	-1.571	0.74586	-2.1063	0.03518	0.10373	12.5775	59.412	43.9246	18.6609	4.2241	16.0864	Down
Tbc1d9b	6778.25	0.22536	0.08096	2.78363	0.00538	0.02301	6228.77	6333.13	6187.64	7555.59	7429.14	6935.26	Up
Mgat4b	5893.13	0.52851	0.11599	4.5565	5.20E-06	5.48E-05	4901.36	4776.92	4798.29	7097.36	7713.21	6071.62	Up
Ltc4s	142.995	1.52511	0.45008	3.38851	0.0007	0.00415	97.7176	92.9511	30.5563	202.16	298.855	135.729	Up
Canx	21534.8	0.29283	0.09181	3.18974	0.00142	0.00751	18599.2	18875.8	20594.9	22664.7	25116.5	23357.5	Up
Hnrnph1	22577.6	-0.2493	0.11701	-2.1302	0.03316	0.09898	22872.7	22752.9	27943.7	19987.9	20094.1	21814.2	Down
Zfp354c	442.291	-0.5816	0.12753	-4.5602	5.11E-06	5.40E-05	508.906	535.667	546.193	346.263	353.768	362.95	Down
Zfp879	54.4468	-0.7272	0.35898	-2.0258	0.04278	0.12074	69.6601	81.452	52.5186	50.7991	39.0729	33.1782	Down
Zfp2	445.696	-0.2819	0.13976	-2.0166	0.04374	0.12295	506.003	458.048	503.223	366.997	427.69	412.214	Down
Zfp354b	239.559	-0.4329	0.20016	-2.163	0.03054	0.09298	288.315	262.563	275.006	215.637	161.572	234.258	Down
Clk4	6225.39	-0.2739	0.1032	-2.6538	0.00796	0.03168	6616.74	6630.19	7196.95	5602.41	5187.2	6118.87	Down
Col23a1	4760.78	-0.6082	0.09198	-6.6119	3.79E-11	1.07E-09	5773.08	5477.41	5998.57	3768.46	3577.81	3969.32	Down
Hnrnpab	4138.21	0.31741	0.0929	3.41664	0.00063	0.00379	3922.25	3543.64	3588.45	4728.46	4673.97	4372.49	Up
Ube2b	13433.5	-0.2841	0.09919	-2.8645	0.00418	0.01864	13572.1	15992.4	14691.8	11978.2	11730.3	12635.9	Down
Olfr1372-	284.541	1.08707	0.29988	3.62504	0.00029	0.00192	277.673	131.281	137.503	394.989	412.906	352.896	Up
Tcf7	1725.37	-0.6393	0.15695	-4.0734	4.63E-05	0.00038	1875.02	1849.44	2580.09	1323.89	1345.38	1378.4	Down
A630014C	50.0021	-1.0471	0.4942	-2.1187	0.03412	0.10118	41.6026	46.9547	113.631	36.2851	25.3446	36.1944	Down
Vdac1	10385.9	-0.2892	0.1403	-2.0613	0.03927	0.11287	11309.1	13337	9624.26	9407.16	8742.83	9895.16	Down
Zcchc10	700.939	-0.2758	0.13144	-2.098	0.0359	0.1053	823.344	710.07	769.636	608.552	693.809	600.224	Down
A430108G	222.984	-0.8393	0.24341	-3.4481	0.00056	0.00343	264.128	325.808	268.322	207.343	122.499	149.805	Down
Il4	742.979	-0.5285	0.1662	-3.1799	0.00147	0.00773	775.936	984.132	872.763	669.718	505.836	649.489	Down
Gm12216	930.02	-0.4955	0.17759	-2.79	0.00527	0.02265	904.614	1137.45	1222.25	684.232	918.742	712.829	Down
Slc22a5	2959.77	-0.3413	0.08134	-4.196	2.72E-05	0.00024	3304.02	3380.74	3239.92	2634.29	2658.02	2541.65	Down
Pdlim4	232.439	-1.8847	0.30677	-6.1437	8.06E-10	1.80E-08	317.34	535.667	244.45	96.4146	98.2104	102.551	Down
Csf2	72.272	1.90884	0.33887	5.63302	1.77E-08	3.05E-07	25.155	32.5808	33.4209	143.067	111.939	87.4699	Up
Acsl6	34.0087	-2.6709	0.80765	-3.307	0.00094	0.00531	21.285	119.782	35.3307	13.4773	2.11205	12.0648	Down
Cdc42se2	7387.49	0.46504	0.07648	6.08042	1.20E-09	2.62E-08	6412.6	6083.03	6125.57	8365.26	8626.67	8711.8	Up
Lyrm7	238.742	-0.7202	0.356	-2.0231	0.04306	0.12144	157.703	380.429	353.307	134.773	168.964	237.275	Down
Gpx3	59074.5	-0.6983	0.16725	-4.1749	2.98E-05	0.00026	61922	91719.7	65652	46415.8	48226.6	40510.6	Down
Tnip1	2639.26	0.59188	0.14652	4.03966	5.35E-05	0.00044	2443.91	1891.6	1980.43	3370.36	3381.39	2767.87	Up
Gm2a	6627.91	0.62141	0.11601	5.35639	8.49E-08	1.27E-06	5805.98	5285.75	4574.84	8297.87	7908.57	7894.41	Up
Atp5pb-p	329.765	0.35944	0.17638	2.03784	0.04157	0.11813	243.81	324.85	297.923	392.915	382.281	336.809	Up
Slc36a2	2276.37	-0.7169	0.14139	-5.0703	3.97E-07	5.34E-06	2594.84	3377.86	2518.98	1733.39	1733.99	1699.13	Down
Slc36a1	1698.92	0.54779	0.10227	5.35635	8.49E-08	1.27E-06	1426.1	1345.4	1369.3	1915.85	1964.21	2172.67	Up
Atox1	1948.81	0.37176	0.10905	3.4092	0.00065	0.00388	1698.93	1569.63	1828.6	2165.7	2332.76	2097.27	Up
Gria1	793.558	-1.8902	0.20687	-9.1371	6.42E-20	5.53E-18	950.086	1290.77	1508.71	313.088	397.066	301.62	Down
Larp1	5912.16	0.22657	0.11406	1.98643	0.04699	0.13002	4977.8	5401.7	5967.06	5832.56	6678.3	6615.54	Up
Mrpl22	879.34	-0.3354	0.13698	-2.4486	0.01434	0.05121	851.401	988.923	1102.89	789.977	787.795	755.056	Down
Zfp692	1327.71	-0.3498	0.12765	-2.7404	0.00614	0.02564	1295.48	1553.34	1614.71	1207.77	1188.03	1106.95	Down
Lypd8	97.0439	4.59282	1.17471	3.90977	9.24E-05	0.00071	20.3175	0.95826	1.90977	234.298	105.603	219.177	Up
2210407C	604.783	7.17226	0.79877	8.97916	2.73E-19	2.23E-17	9.67501	10.5408	4.77441	1235.77	2018.06	349.88	Up
Olfr323	9.947	6.81435	1.61003	4.23245	2.31E-05	0.00021	0	0	0	16.5875	39.0729	4.0216	Up
Olfr317	9.00159	-3.246	1.05758	-3.0693	0.00215	0.01057	5.80501	26.8312	16.233	2.07343	1.05603	2.0108	Down
Olfr316	6.7867	-3.137	1.13513	-2.7636	0.00572	0.02424	15.48	13.4156	7.63906	2.07343	2.11205	0	Down
Fam183b	2259.82	-0.6522	0.1722	-3.7871	0.00015	0.0011	2861.87	2632.34	2792.08	2192.65	1393.95	1686.06	Down
Gm12258	360.98	-0.7579	0.19696	-3.8481	0.00012	0.00089	424.733	392.886	543.328	233.261	306.247	265.426	Down
Zfp39	247.642	-0.6183	0.24735	-2.4995	0.01244	0.04568	226.395	327.724	345.668	156.544	193.253	236.269	Down
Rnf187	8294.44	-0.2897	0.10531	-2.7506	0.00595	0.025	8345.67	9223.24	9803.78	7335.8	7975.1	7083.05	Down
Obscn	1341.34	-4.2162	0.67173	-6.2766	3.46E-10	8.28E-09	1599.28	4659.05	1378.85	96.4146	99.2664	215.156	Down
Gjc2	91.4348	-1.086	0.38843	-2.796	0.00517	0.02233	164.475	81.452	126.999	83.974	46.4651	45.243	Down
Zfp867	712.908	-0.249	0.10984	-2.2666	0.02341	0.07566	787.546	743.609	791.598	666.608	653.68	634.408	Down
Nlrp3	324.495	1.35097	0.30855	4.37844	1.20E-05	0.00011	265.095	164.82	118.405	440.604	585.038	373.004	Up
Flcn	4408.77	0.20644	0.07598	2.71708	0.00659	0.02712	4148.65	4124.35	4008.6	4806.21	4708.82	4656.01	Up
Gm12714	5.60993	3.41897	1.21263	2.81946	0.00481	0.021	0.9675	0.95826	0.95488	10.3672	6.33615	14.0756	Up
Srebf1	6483.3	0.56794	0.08713	6.51851	7.10E-11	1.92E-09	5335.77	5351.87	4982.58	8151.7	7589.65	7488.23	Up
Tom1l2	4539.84	-0.282	0.11964	-2.3566	0.01844	0.06269	4301.51	5287.67	5356.89	3922.93	4216.71	4153.31	Down
Drc3	3558.14	-0.5349	0.24871	-2.1508	0.03149	0.09516	3498.49	4919.7	4212.94	3188.94	1804.75	3724	Down
Gid4	3211.4	-0.4806	0.09855	-4.8771	1.08E-06	1.32E-05	3943.54	3674.92	3605.64	2637.41	2870.28	2536.63	Down
Myo15	142.818	1.22925	0.27468	4.47523	7.63E-06	7.75E-05	81.2701	72.8277	102.172	208.38	149.956	242.302	Up
Flii	9013.45	0.21577	0.08538	2.52727	0.0115	0.0429	8282.78	7883.59	8855.58	9606.21	9815.76	9636.77	Up
Smcr8	2023.82	0.34499	0.10983	3.14113	0.00168	0.00862	1896.3	1604.12	1848.65	2211.31	2220.82	2361.69	Up
Shmt1	1150.41	0.53457	0.1196	4.46982	7.83E-06	7.90E-05	1039.1	854.767	925.282	1315.59	1360.16	1407.56	Up
Dhrs7b	1719.33	0.49159	0.12882	3.81614	0.00014	0.00099	1514.14	1452.72	1320.6	2025.74	2229.27	1773.53	Up
Natd1	1596.48	0.57452	0.16476	3.48695	0.00049	0.00304	1598.31	1156.62	1093.34	1955.25	1931.47	1843.91	Up
Tnfrsf13b	566.583	0.58445	0.14353	4.07199	4.66E-05	0.00038	490.523	424.509	444.975	641.727	760.338	637.424	Up
Usp22	4901.69	0.22386	0.08837	2.53325	0.0113	0.04227	4679.8	4221.13	4665.56	5165.96	5333.98	5343.71	Up
Aldh3a1	1959.31	-1.5965	0.65157	-2.4502	0.01428	0.05108	2508.73	4258.5	2067.32	1034.64	346.376	1540.27	Down
Slc47a1	363.623	-1.0247	0.31063	-3.2989	0.00097	0.00544	389.903	728.277	344.713	227.041	192.197	299.609	Down
Gm45605	130.556	-1.3824	0.31872	-4.3373	1.44E-05	0.00014	174.15	170.57	221.533	44.5788	74.9778	97.5239	Down

Mfap4	29697.3	-0.5728	0.15485	-3.6992	0.00022	0.00149	29173.1	42745	34631.7	25642.1	24049.9	21941.9	Down
Grap	4027.61	-0.4829	0.12707	-3.8	0.00014	0.00105	4584.02	4291.08	5210.8	3523.8	3580.98	2974.98	Down
Akap10	1600.87	0.32309	0.11455	2.82047	0.0048	0.02094	1502.53	1351.14	1413.23	1785.22	1929.36	1623.72	Up
Specc1	2754.69	0.50057	0.1351	3.70517	0.00021	0.00146	2444.88	2022.88	2376.7	2979.52	3672.86	3031.28	Up
Adora2b	1878.33	1.74545	0.10893	16.0232	8.80E-58	1.36E-54	811.734	861.475	915.733	2686.13	3056.14	2938.79	Up
Zswim7	479.913	0.49499	0.1605	3.08408	0.00204	0.01013	353.138	442.715	399.141	548.423	628.335	507.727	Up
Ncor1	2677.42	0.28891	0.13104	2.20476	0.02747	0.08579	2737.06	2217.41	2276.44	2862.37	2729.83	3241.41	Up
Ubb	36941.2	0.23797	0.09298	2.55936	0.01049	0.03978	32704.5	34176.3	34823.6	43336.8	37763.5	38842.7	Up
Trpv2	1366.15	1.08328	0.1873	5.78368	7.31E-09	1.36E-07	1049.74	802.062	776.32	1814.25	2230.33	1524.19	Up
Mmgt2	1160.82	0.42486	0.12514	3.39497	0.00069	0.00406	1049.74	923.761	999.762	1459.7	1328.48	1203.46	Up
Fbxw10	112.603	0.70044	0.28708	2.43989	0.01469	0.05217	85.1401	101.575	70.6613	178.315	118.275	121.654	Up
Pmp22	27538	-0.4536	0.11408	-3.9763	7.00E-05	0.00055	30074.8	30947	34474.1	24654.1	24433.3	20644.9	Down
Hmgb1-p	39.9153	0.79789	0.38962	2.04789	0.04057	0.11577	32.895	33.5391	21.0074	48.7256	58.0814	45.243	Up
Hs3st3b1	761.388	0.50094	0.20141	2.48717	0.01288	0.04698	660.804	557.707	673.192	966.219	654.736	1055.67	Up
2810001G	360.992	-0.8836	0.19946	-4.4298	9.43E-06	9.31E-05	557.281	367.013	480.306	270.583	254.502	236.269	Down
Gm12290	6.15015	-2.6092	1.14199	-2.2848	0.02233	0.07273	8.70751	15.3321	7.63906	3.11015	2.11205	0	Down
Arhgap44	3845.53	0.61979	0.09941	6.23449	4.53E-10	1.06E-08	3304.99	2892.98	2898.07	4827.99	4530.35	4618.81	Up
Myocd	91.9389	-2.4647	0.40756	-6.0474	1.47E-09	3.17E-08	91.9126	229.024	146.097	22.8077	30.6247	31.1674	Down
Gm12295	81.0559	-4.2924	0.64183	-6.6878	2.27E-11	6.70E-10	117.068	286.519	59.2027	7.25701	4.2241	12.0648	Down
Map2k4	3500.46	0.48976	0.08305	5.89736	3.69E-09	7.36E-08	2924.76	2853.69	2957.27	3942.63	4250.5	4073.88	Up
Dnah9	2841.91	-0.6198	0.24573	-2.5222	0.01166	0.04337	3106.65	4149.26	3073.77	2197.84	1481.6	3042.34	Down
Shisa6	30.5469	-1.1922	0.50611	-2.3556	0.01849	0.06282	23.22	45.9964	58.2479	18.6609	20.0645	17.0918	Down
Adprm	1789.65	-0.2556	0.11082	-2.3066	0.02108	0.06948	1758.92	1976.89	2107.43	1672.22	1638.95	1583.51	Down
Sco1	782.512	0.5845	0.11606	5.03637	4.74E-07	6.28E-06	654.998	610.411	613.035	872.915	970.487	973.228	Up
Myh8	6.905	-4.2379	1.58204	-2.6788	0.00739	0.02982	4.83751	29.706	4.77441	0	2.11205	0	Down
Myh13	72.2733	1.21744	0.38725	3.14382	0.00167	0.00855	25.155	44.0799	61.1125	92.2677	123.555	87.4699	Up
Dhrs7c	332.354	-5.5556	1.22945	-4.5188	6.22E-06	6.43E-05	400.546	1207.41	344.713	6.2203	1.05603	34.1836	Down
Pik3r5	1483.66	1.49038	0.1398	10.6611	1.55E-26	2.40E-24	907.516	756.066	673.192	2112.83	2295.8	2156.58	Up
Pik3r6	819.084	0.57353	0.14783	3.87956	0.0001	0.00079	732.399	667.906	574.84	895.722	1083.48	960.158	Up
Mfsd6l	458.062	-0.5009	0.2231	-2.2454	0.02474	0.07899	473.108	592.204	545.238	433.347	266.118	438.355	Down
Myh10	8256.12	-0.6634	0.12772	-5.1939	2.06E-07	2.91E-06	10130.7	10207.4	10026.3	6057.53	7474.55	5640.3	Down
Arhgef15	7129.37	-0.3544	0.13024	-2.7211	0.00651	0.02687	7887.07	7388.17	8726.67	5531.92	7115.5	6126.91	Down
Slc25a35	1117.07	0.58464	0.16934	3.45241	0.00056	0.00339	729.496	1100.08	851.756	1332.18	1289.41	1399.52	Up
Pfas	865.369	-0.2696	0.10621	-2.5382	0.01114	0.04175	919.126	922.803	995.943	784.794	799.411	770.137	Down
Aurkb	132.616	1.09621	0.27032	4.0553	5.01E-05	0.00041	85.1401	108.283	60.1576	192.829	176.356	172.929	Up
Tmem107	1661.3	-0.6737	0.17649	-3.8174	0.00013	0.00099	1944.68	2200.16	1982.34	1399.57	963.095	1477.94	Down
Vamp2	7444.25	-0.356	0.07854	-4.533	5.81E-06	6.05E-05	8545.94	8227.61	8300.8	6606.99	6719.49	6264.65	Down
Chd3	7579.16	0.89471	0.08229	10.8729	1.55E-27	2.70E-25	5383.18	5050.02	5471.48	9564.74	9880.17	10125.4	Up
Tmem88	579.021	-0.5424	0.18246	-2.9725	0.00295	0.01388	559.216	653.532	846.981	457.192	483.66	473.544	Down
Dnah2	1889.71	-0.4974	0.20101	-2.4747	0.01334	0.04835	1844.06	2297.9	2495.11	1738.57	1146.84	1815.75	Down
Atp1b2	485.76	-0.7736	0.14819	-5.2204	1.79E-07	2.55E-06	605.656	639.159	593.937	319.308	407.626	348.874	Down
Sat2	155.46	-0.554	0.24621	-2.2501	0.02444	0.07818	160.605	190.693	203.39	116.112	158.404	103.556	Down
Mpdu1	2842.8	0.3481	0.10512	3.31149	0.00093	0.00524	2538.72	2473.27	2492.24	3238.7	3438.42	2875.45	Up
Cd68	5412.79	2.94262	0.47995	6.13106	8.73E-10	1.94E-08	1971.77	1031.09	735.26	8830.75	12289	7618.93	Up
Eif4a1	15668.9	0.36697	0.11312	3.24406	0.00118	0.0064	14385.8	12143.1	14531.4	17821.1	18714.9	16417.2	Up
Polr2a	5594.46	0.28223	0.09714	2.90544	0.00367	0.01669	5282.56	4724.22	5140.13	5818.05	6525.18	6076.64	Up
Slc35g3	139.71	-1.494	0.40005	-3.7345	0.00019	0.00133	133.515	311.434	173.789	59.0928	53.8573	106.572	Down
Tmem102	848.44	0.97006	0.14655	6.61926	3.61E-11	1.02E-09	526.321	652.574	541.419	1180.82	1190.14	999.368	Up
Tnk1	697.924	0.37747	0.15909	2.37273	0.01766	0.06049	636.616	499.253	685.606	750.582	771.955	843.531	Up
Acap1	886.103	-0.345	0.12728	-2.711	0.00671	0.02753	1074.89	923.761	975.89	812.785	819.476	709.813	Down
Eif5a	18600.3	0.22603	0.11316	1.9974	0.04578	0.12731	16642	17705.7	17090.5	20537.3	22066.7	17559.3	Up
Slc2a4	673.429	-3.8037	0.65616	-5.7969	6.76E-09	1.27E-07	787.546	2433.02	550.013	92.2677	79.2019	98.5293	Down
Cldn7	4614.24	0.40545	0.11863	3.41789	0.00063	0.00378	3444.31	4285.33	4180.48	5485.26	5259.01	5031.03	Up
Gabarap	18008.6	0.40411	0.10452	3.86638	0.00011	0.00083	17078.3	14781.1	14648.9	20514.5	21628.5	19400.2	Up
Dlg4	1811.72	-0.453	0.11129	-4.0702	4.70E-05	0.00039	2291.04	1891.6	2098.83	1522.94	1540.74	1525.19	Down
Asgr1	228.101	-3.3313	0.42515	-7.8355	4.67E-15	2.38E-13	306.698	484.879	453.569	27.9913	20.0645	75.4051	Down
Asgr2	76.0117	0.88425	0.38325	2.30726	0.02104	0.06939	52.2451	46.9547	61.1125	118.186	54.9133	122.659	Up
Mgl2	1243.17	0.51717	0.1844	2.80457	0.00504	0.02187	778.839	1209.32	1079.97	1349.8	1400.29	1640.81	Up
Clec10a	243.23	-1.104	0.33402	-3.3054	0.00095	0.00534	321.21	480.088	194.796	170.021	119.331	173.934	Down
Slc16a11	4168.05	-0.5648	0.20191	-2.7973	0.00515	0.02225	4458.25	5998.7	4464.08	4147.9	2526.01	3413.34	Down
Bcl6b	1795.47	0.37846	0.18179	2.0818	0.03736	0.10864	1488.02	1361.69	1834.33	1666	1971.6	2451.17	Up
Alox12	511.131	-1.3539	0.23587	-5.74	9.47E-09	1.73E-07	507.938	959.217	737.17	278.877	303.079	280.507	Down
Alox12e	86.0271	3.02038	0.76352	3.95586	7.63E-05	0.0006	11.61	43.1216	1.90977	152.397	72.8658	234.258	Up
Arrb2	2130.25	0.51568	0.15076	3.42065	0.00062	0.00375	2083.03	1537.05	1640.49	2420.73	2764.67	2335.55	Up
Cxcl16	5042.21	0.97053	0.08822	11.0011	3.77E-28	6.78E-26	3458.82	3209.21	3554.07	6641.2	6900.07	6489.86	Up
Zmynd15	695.793	1.30501	0.2262	5.7694	7.96E-09	1.47E-07	406.351	321.017	475.532	929.934	775.123	1266.81	Up
Pld2	2708.77	0.63606	0.09551	6.65978	2.74E-11	8.03E-10	2232.99	2031.51	2098.83	3436.71	3332.82	3119.76	Up
Mink1	3447.07	0.27779	0.09595	2.89521	0.00379	0.01717	3028.28	2973.48	3346.86	3623.32	3902.01	3808.46	Up
Gp1ba	88.2659	-1.031	0.51273	-2.0108	0.04435	0.12415	46.4401	208.9	100.263	66.3498	43.297	64.3457	Down
Rnf167	5208.74	-0.5027	0.08649	-5.8125	6.16E-09	1.16E-07	6064.3	5987.2	6269.76	4456.84	4421.58	4052.77	Down
Pfn1	19603	0.64013	0.1286	4.97776	6.43E-07	8.32E-06	15736.4	14613.4	15621.9	24917.5	26649.9	20078.9	Up
Eno3	2477.93	-2.1987	0.30074	-7.311	2.65E-13	1.05E-11	2904.44	6595.69	2708.05	944.448	896.566	818.396	Down
Kif1c	11013.5	0.32692	0.08562	3.81832	0.00013	0.00098	9729.19	9963.02	9620.45	12310	12941.6	11516.9	Up
Scimp	372.697	1.99573	0.30606	6.52066	7.00E-11	1.90E-09	232.2	98.7006	117.451	535.982	673.744	578.105	Up
C1qbp	2171.68	-0.2181	0.09179	-2.3758	0.01751	0.06005	2396.5	2410.02	2200.05	2050.62	1983.22	1989.69	Down
Derl2	4950.88	0.28717	0.08476	3.38812	0.0007	0.00415	4351.82	4316.96	4710.44	5331.83	5561.03	5433.19	Up
Mis12	1330.14	0.28651	0.12671	2.2611	0.02375	0.07647	1282.91	1095.29	1217.48	1503.24	1309.47	1572.45	Up
6330403K	127.214	-0.7957	0.23536	-3.3809	0.00072	0.00424	190.598	148.53	145.142	89.1576	101.378	88.4753	Down
Nlrp1b	377.453	1.13809	0.23449	4.85343	1.21E-06	1.46E-05	222.525	274.062	211.029	405.356	473.099	678.646	Up
Wscd1	1488.62	-0.3964	0.17875	-2.2176	0.02658	0.08363	1479.31	1626.16	1969.92	1063.67	1544.97	1247.7	Down
Pimreg	76.3219	1.44798	0.42656	3.39452	0.00069	0.00407	29.9925	66.1198	26.7367	101.598	88.7061	144.778	Up
Pitpnm3	2774.03	-0.3801	0.15209	-2.499	0.01245	0.04571	2620.96	3274.37	3516.83	2420.73	2127.89	2683.41	Down
Med31	1273.9	-0.4082	0.13292	-3.0711	0.00213	0.01051	1433.84	1443.14	1481.98	1233.69	938.807	1111.97	Down

Xaf1	2738.33	0.38965	0.17986	2.16637	0.03028	0.09239	2836.71	2312.28	1963.24	3258.4	3519.73	2539.64 Up
Smtnl2	1469.86	-0.5318	0.16491	-3.2249	0.00126	0.00678	2101.41	1569.63	1542.14	1328.03	1210.21	1067.74 Down
Spns3	81.1136	1.53191	0.42543	3.6008	0.00032	0.00208	63.8551	39.2886	21.9623	148.25	87.6501	125.675 Up
Zzef1	3102.1	0.29948	0.10113	2.96152	0.00306	0.01433	2922.82	2714.75	2706.14	3257.36	3709.82	3301.74 Up
P2rx1	129.323	-0.7725	0.33616	-2.298	0.02156	0.07071	124.808	185.902	178.563	89.1576	135.171	62.3349 Down
Itgae	528.376	1.31495	0.27286	4.81905	1.44E-06	1.72E-05	451.823	208.9	248.27	793.088	719.153	749.024 Up
P2rx5	588.098	1.3789	0.15608	8.83436	1.01E-18	7.95E-17	282.51	344.015	353.307	843.887	937.751	767.121 Up
Emc6	4153.83	0.43433	0.09161	4.74135	2.12E-06	2.43E-05	3631.03	3478.48	3490.1	5062.28	4789.08	4472.02 Up
Tax1bp3	3799.82	0.77768	0.09722	7.99889	1.26E-15	6.87E-14	3006.03	2728.16	2665.08	4873.6	4967.54	4558.49 Up
Ctns	1514.3	0.42691	0.11977	3.56453	0.00036	0.00235	1428.03	1193.99	1253.76	1640.08	1714.99	1854.96 Up
Aspa	841.954	-0.4309	0.18977	-2.2706	0.02317	0.07505	865.914	1165.24	868.943	658.315	865.941	627.37 Down
Rap1gap2	652.64	-0.5926	0.20376	-2.9082	0.00364	0.01658	652.096	1031.09	671.283	499.697	523.789	537.889 Down
Sgsm2	1718.28	0.36302	0.09454	3.83992	0.00012	0.00091	1480.28	1477.63	1551.68	1845.35	2023.34	1931.38 Up
Rpa1	3518.86	-0.3084	0.09853	-3.1304	0.00175	0.00889	3940.63	4038.1	3702.08	3387.99	2997	3047.37 Down
Serpinf1	3032.99	0.65312	0.19822	3.29497	0.00098	0.00551	2054.97	2968.69	2050.13	4557.4	3344.43	3222.31 Up
Wdr81	2674.07	0.68866	0.11122	6.19192	5.94E-10	1.36E-08	2200.1	1939.52	2003.34	3450.19	3452.15	2999.11 Up
Rilp	1113.42	0.36601	0.12596	2.90581	0.00366	0.01667	861.076	1027.25	1030.32	1227.47	1337.98	1196.43 Up
Myo1c	19927	0.31084	0.14718	2.11195	0.03469	0.10253	20299.1	14560.7	18505.6	21955.6	24040.4	20200.5 Up
Doc2b	112.433	-1.62	0.30059	-5.3894	7.07E-08	1.08E-06	189.63	169.612	149.917	61.1662	35.9049	68.3673 Down
Vps53	2511.26	0.49474	0.0896	5.52188	3.35E-08	5.49E-07	2054.01	2119.67	2080.69	3058.31	2976.94	2777.92 Up
Nxn	1519.5	-0.5855	0.10275	-5.6984	1.21E-08	2.15E-07	1725.06	1843.69	1902.13	1273.09	1222.88	1150.18 Down
Abr	6438.48	0.53618	0.12289	4.36323	1.28E-05	0.00012	5809.85	5264.67	4692.29	7487.16	8332.04	7044.84 Up
Trarg1	689.768	-1.3522	0.20405	-6.6268	3.43E-11	9.79E-10	846.564	1257.24	869.898	446.825	382.281	335.804 Down
Ssh2	5019.81	-0.2389	0.11685	-2.0444	0.04091	0.11658	4752.37	5924.91	5626.17	4557.4	4431.08	4826.93 Down
Coro6	865.933	-1.446	0.2965	-4.8767	1.08E-06	1.32E-05	863.979	1986.47	950.108	509.027	466.763	419.252 Down
Git1	5073.85	-0.269	0.1357	-1.9823	0.04744	0.13102	4848.15	5441.95	6346.15	4550.15	5033.02	4223.69 Down
Trp53i13	1041.12	-0.5112	0.1442	-3.545	0.00039	0.00251	1280.97	1274.48	1115.3	901.943	944.087	729.921 Down
Taok1	132.062	0.54203	0.21155	2.56217	0.0104	0.0395	107.393	106.367	108.857	138.92	168.964	161.87 Up
Myo18a	6379.96	0.4399	0.0901	4.8824	1.05E-06	1.29E-05	5507.02	5068.23	5669.14	7518.26	7050.03	7467.11 Up
Phf12	3452.72	0.21477	0.0916	2.34453	0.01905	0.06426	3332.08	2991.68	3264.74	3665.83	3835.48	3626.48 Up
Flot2	5725.38	0.45949	0.11853	3.87673	0.00011	0.0008	5329.97	4682.05	4451.66	6463.92	7268.62	6156.07 Up
Traf4	1358.54	0.36891	0.10636	3.46838	0.00052	0.00323	1244.21	1182.49	1130.58	1613.13	1548.13	1432.7 Up
Tlcd1	895.185	0.27854	0.11451	2.43241	0.015	0.05305	869.784	804.937	752.448	1017.02	955.703	971.217 Up
2610507B	9433.22	0.33463	0.08486	3.94355	8.03E-05	0.00063	8261.5	8496.88	8274.06	9956.62	10469.4	11140.8 Up
Spag5	138.093	0.69812	0.21493	3.24815	0.00116	0.00632	113.198	107.325	95.4883	184.535	152.068	175.945 Up
Aldoc	1399.41	2.71923	0.24328	11.1776	5.25E-29	9.76E-27	521.483	329.641	255.909	2330.54	2774.18	2184.74 Up
Pigs	3385.02	0.63783	0.1116	5.71509	1.10E-08	1.96E-07	2926.69	2591.13	2428.27	4046.3	4363.5	3954.24 Up
Unc119	1809.21	1.19217	0.20796	5.73281	9.88E-09	1.79E-07	1371.92	1051.21	881.357	2567.95	3000.17	1982.65 Up
Slc13a2os	5.4408	4.00237	1.48291	2.69899	0.00696	0.02838	0	1.91652	0	10.3672	5.28013	15.081 Up
Slc13a2	30.3207	3.54134	0.61938	5.71756	1.08E-08	1.94E-07	2.9025	7.66607	3.81953	67.3865	33.7928	66.3565 Up
Slc46a1	373.125	0.38233	0.15105	2.53122	0.01137	0.04247	333.788	348.806	289.33	448.898	409.738	408.193 Up
Vtn	819.174	-1.0849	0.18527	-5.8557	4.75E-09	9.26E-08	982.014	968.799	1389.35	526.652	578.702	469.522 Down
Tmem199	1145.78	0.258	0.10375	2.48679	0.01289	0.04702	1075.86	1076.12	978.755	1259.61	1278.85	1205.48 Up
Tnfaip1	9683.56	0.29376	0.10378	2.8305	0.00465	0.0204	8579.8	7966	9557.42	10319.5	10574	11104.7 Up
Tmem97	1373.39	0.23155	0.09689	2.38972	0.01686	0.05834	1279.04	1308.98	1202.2	1525.01	1476.32	1448.78 Up
Lyrm9	431.368	-0.8695	0.13867	-6.2702	3.61E-10	8.60E-09	550.508	598.912	523.276	289.244	312.584	313.685 Down
Nos2	253.625	1.38903	0.34976	3.97136	7.15E-05	0.00057	218.655	100.617	101.218	355.594	475.211	270.453 Up
Lgals9	6852.19	0.39457	0.09829	4.01431	5.96E-05	0.00048	5817.59	5677.68	6267.85	7562.84	7390.07	8397.11 Up
Ksr1	1021.41	-0.4482	0.12936	-3.4645	0.00053	0.00326	1126.17	1120.2	1290.05	775.463	897.622	918.936 Down
Nf1	3599.89	0.37843	0.1431	2.64452	0.00818	0.03241	2913.15	3384.57	3093.82	3864.88	3546.13	4796.77 Up
Rab11fip4	881.283	-1.164	0.2196	-5.3007	1.15E-07	1.69E-06	1105.85	1355.94	1194.56	596.112	370.665	664.57 Down
Rnf135	1009.84	0.38182	0.10654	3.58375	0.00034	0.00221	878.491	855.725	896.635	1089.59	1217.6	1121.02 Up
Rhbdl3	363.669	-0.7828	0.20853	-3.7537	0.00017	0.00124	435.376	567.289	377.179	241.555	306.247	254.366 Down
Psmd11	3799.62	0.26851	0.08615	3.11682	0.00183	0.00925	3636.84	3278.2	3426.12	4138.57	4179.75	4138.23 Up
Myo1d	9900.53	0.4941	0.10414	4.74465	2.09E-06	2.40E-05	9021.95	7517.54	8125.1	11472.3	12087.3	11179.1 Up
Tmem98	3003.55	0.79157	0.13736	5.76286	8.27E-09	1.52E-07	2568.72	2097.63	1932.68	4058.74	3565.14	3798.4 Up
Asic2	52.3233	2.50356	0.37756	6.63089	3.34E-11	9.53E-10	18.3825	17.2487	11.4586	91.231	77.0899	98.5293 Up
Ccl7	150.968	1.70874	0.30707	5.56471	2.63E-08	4.36E-07	70.6276	52.7042	88.8041	244.665	285.127	163.88 Up
Ccl12	181.915	1.87699	0.25153	7.4624	8.50E-14	3.66E-12	99.6527	75.7024	58.2479	317.235	288.295	252.356 Up
Ccl8	219.788	1.40405	0.44857	3.13008	0.00175	0.00889	74.4976	204.109	83.0748	324.492	171.076	461.479 Up
Fndc8	16.8208	-2.58	0.66239	-3.8949	9.82E-05	0.00075	29.9925	36.4138	20.0525	6.2203	4.2241	4.0216 Down
Unc45b	975.36	-0.585	0.16085	-3.6369	0.00028	0.00184	1055.54	1348.27	1107.66	679.049	769.842	891.791 Down
Slfn5os	85.0732	-0.8598	0.34757	-2.4738	0.01337	0.04844	84.1726	145.655	99.3078	43.5421	63.3615	74.3997 Down
Slfn9	324.095	1.91012	0.29039	6.57775	4.78E-11	1.33E-09	199.305	88.1598	121.27	558.79	436.138	540.906 Up
Slfn8	337.17	0.94586	0.38113	2.48171	0.01308	0.04756	208.013	331.557	151.826	301.684	323.144	706.797 Up
Slfn10-ps	13.1193	2.28169	1.01745	2.24257	0.02492	0.07948	3.87001	0	9.54883	36.2851	17.9524	11.0594 Up
Slfn2	171.112	0.79224	0.25562	3.09932	0.00194	0.00971	160.605	125.532	89.759	216.674	204.869	229.231 Up
Slfn1	449.25	-0.686	0.20081	-3.416	0.00064	0.0038	618.233	606.578	437.336	315.162	405.514	312.68 Down
Gm11427	150.242	0.6784	0.25999	2.60933	0.00907	0.03523	121.905	96.7841	127.954	139.957	222.821	192.032 Up
Ap2b1	9076.13	0.43235	0.08043	5.37582	7.62E-08	1.16E-06	7740.01	7567.37	7871.1	10335	10921.4	10021.8 Up
1700020L	255.225	-1.0054	0.19976	-5.0332	4.82E-07	6.36E-06	287.348	402.469	332.299	184.535	166.852	157.848 Down
Mmp28	785.235	-0.5069	0.15225	-3.3292	0.00087	0.00495	769.164	941.968	1054.19	639.654	637.839	668.592 Down
Ccl5	481.982	-0.8281	0.16124	-5.1357	2.81E-07	3.89E-06	648.226	656.407	545.238	302.721	384.393	354.907 Down
Ccl9	1562.4	0.70929	0.22213	3.19309	0.00141	0.00743	920.094	1658.75	978.755	1939.7	2019.12	1857.98 Up
E230016K	1102.7	2.99426	0.55173	5.42701	5.73E-08	8.99E-07	440.213	166.737	130.819	1652.53	2331.7	1894.18 Up
Ccl3	225.811	1.62353	0.46983	3.45556	0.00055	0.00335	168.345	91.0346	72.5711	287.17	582.926	152.821 Up
Wfdc17	769.042	3.8153	0.35336	10.7973	3.55E-27	6.02E-25	60.9526	175.361	69.7065	1174.6	1337.98	1795.65 Up
Wfdc18	45.4576	3.50581	0.78406	4.47136	7.77E-06	7.86E-05	3.87001	14.3739	3.81953	168.985	26.4006	55.297 Up
Wfdc21	366.528	3.15036	0.67104	4.69473	2.67E-06	3.00E-05	146.093	45.0382	31.5111	600.258	815.252	561.014 Up
Heatr6	1455.49	0.27215	0.1175	2.31609	0.02055	0.06816	1464.8	1291.73	1199.33	1556.11	1649.51	1571.44 Up
Hnf1b	1416.69	0.68724	0.11255	6.10633	1.02E-09	2.26E-08	1014.91	1187.28	1054.19	1745.83	1827.98	1669.97 Up
Synrg	3783.4	0.22107	0.08865	2.4938	0.01264	0.04628	3678.44	3319.41	3484.37	4034.9	4201.92	3981.39 Up
Tada2a	1020.83	-0.2537	0.1087	-2.3336	0.01962	0.06578	1131.98	1037.79	1161.14	940.301	960.983	892.796 Down

Acaca	3012.71	0.60665	0.25921	2.34043	0.01926	0.06483	1668.94	3562.81	1933.64	3233.52	3630.62	4046.74	Up
Myo19	816.228	0.31265	0.14319	2.18345	0.029	0.08963	722.724	792.48	669.373	898.833	801.523	1012.44	Up
Znhit3	533.589	-0.3413	0.13802	-2.4726	0.01341	0.0486	633.713	543.333	612.08	499.697	478.379	434.333	Down
Car4	19271.1	0.95876	0.16891	5.6763	1.38E-08	2.43E-07	12426.6	12007	14846.5	24204.2	31279.5	20863.1	Up
Usp32	3279.71	0.40632	0.09238	4.39856	1.09E-05	0.00011	2709.97	2861.36	2891.39	3513.43	3791.13	3911.01	Up
Tbx2	12506.6	-0.597	0.12306	-4.8515	1.23E-06	1.48E-05	14760.2	14304.9	16108.9	10189.9	11048.1	8627.34	Down
Tbx4	4115.27	-0.5375	0.12271	-4.3804	1.18E-05	0.00011	5263.21	4347.62	5008.36	3405.61	3628.5	3038.32	Down
Brip1	112.632	0.93138	0.29816	3.1238	0.00179	0.00907	82.2376	98.7006	51.5637	136.846	142.563	163.88	Up
Brip1os	2585.58	0.41101	0.10682	3.84778	0.00012	0.00089	2149.79	2352.52	2157.08	2755.59	2888.23	3210.24	Up
Vmp1	12293.6	0.64673	0.07986	8.09798	5.59E-16	3.27E-14	9278.34	9388.06	10083.6	15094.6	15212	14705	Up
Cltc	19043.6	0.35397	0.10234	3.45872	0.00054	0.00332	15520.7	17087.7	17548.8	19835.5	21268.4	23000.6	Up
Gdpd1	4209.09	0.63583	0.12814	4.96209	6.97E-07	8.94E-06	3316.6	3495.73	3076.63	5817.01	4494.44	5054.15	Up
Smg8	1288.71	0.42592	0.12554	3.39257	0.00069	0.0041	1232.6	1000.42	1066.6	1571.66	1418.24	1442.75	Up
Prr11	110.934	0.68971	0.30948	2.22863	0.02584	0.08174	117.068	68.9946	68.7516	137.883	114.051	158.853	Up
Ppm1e	460.168	-0.5285	0.1605	-3.2928	0.00099	0.00554	465.368	599.87	565.291	345.226	399.178	386.074	Down
Septin4	9387.58	-0.6816	0.11775	-5.7887	7.09E-09	1.33E-07	10115.2	12066.4	12512.8	7101.5	7686.81	6842.76	Down
Mtmr4	2947.56	0.28899	0.12852	2.24848	0.02455	0.07851	2478.74	2548.97	2932.45	3217.97	2899.85	3607.38	Up
Rnf43	1091.35	0.62702	0.17517	3.57956	0.00034	0.00224	1074.89	705.278	793.508	1324.92	1264.06	1385.44	Up
Tspoap1	826.66	-0.8596	0.25889	-3.3202	0.0009	0.0051	811.734	1319.52	1066.6	555.68	418.186	788.234	Down
Epx	6.63286	-2.4439	0.99785	-2.4492	0.01432	0.05115	12.5775	13.4156	7.63906	1.03672	2.11205	3.0162	Down
Vezf1	5219.17	-0.2608	0.08231	-3.1687	0.00153	0.00797	5900.79	5400.75	5767.49	4706.69	4803.86	4735.44	Down
Cuedc1	2316.4	-0.4433	0.08417	-5.2668	1.39E-07	2.01E-06	2659.66	2601.67	2747.2	1998.79	1953.65	1937.41	Down
C030037D	187.206	-0.8739	0.24595	-3.553	0.00038	0.00245	221.558	227.107	277.871	155.507	144.675	96.5185	Down
Akap1	2385.57	0.22542	0.10895	2.06898	0.03855	0.11133	2203.97	2210.7	2183.82	2664.36	2757.28	2293.32	Up
Scpep1	713.378	0.60359	0.23695	2.54731	0.01086	0.04093	776.904	412.051	509.907	881.208	945.143	755.056	Up
Gm11496	23.826	1.4992	0.64884	2.31057	0.02086	0.06888	4.83751	16.2904	16.233	37.3218	19.0085	49.2646	Up
Ankfn1	228.956	-0.7157	0.26498	-2.7009	0.00691	0.02825	236.07	303.768	314.156	170.021	122.499	227.221	Down
Pctp	958.075	0.42926	0.11941	3.59477	0.00032	0.00213	743.041	889.264	817.38	1060.56	1136.28	1101.92	Up
Tmem100	27953.8	-0.6611	0.13606	-4.8589	1.18E-06	1.43E-05	29462.4	33079.1	40204.4	22324.6	22380.3	20271.9	Down
Mmd	3470.39	-0.4277	0.12874	-3.3221	0.00089	0.00507	3845.82	4545.02	3552.16	3092.52	3056.14	2730.67	Down
Tom1l1	3164.61	0.32274	0.11774	2.74109	0.00612	0.02559	3063.11	2793.32	2580.09	3283.28	3414.13	3853.7	Up
B230206L	163.646	-0.8064	0.23651	-3.4097	0.00065	0.00388	191.565	197.401	235.856	123.369	92.9302	140.756	Down
Luc7l3	7787.41	-0.3383	0.11707	-2.8892	0.00386	0.01745	8343.73	7895.09	9849.62	6508.5	7101.77	7025.74	Down
Cacna1g	33.9026	-1.2347	0.53116	-2.3246	0.02009	0.06705	23.22	60.3703	59.2027	19.6976	14.7844	26.1404	Down
Epn3	5386.67	0.41294	0.14768	2.79614	0.00517	0.02232	3931.93	4617.85	5312.97	6677.49	6386.84	5392.97	Up
Mycbpap	2382.84	-0.5388	0.23166	-2.3257	0.02004	0.06692	2421.66	3324.2	2722.37	1996.71	1311.58	2520.54	Down
Lrrc59	6804.79	0.61972	0.08213	7.5457	4.50E-14	2.01E-12	5183.87	5298.21	5613.76	8373.55	8377.45	7981.88	Up
Eme1	26.4632	1.20971	0.54549	2.21768	0.02658	0.08363	11.61	13.4156	22.9172	39.3952	22.1765	49.2646	Up
Gm11545	8.82936	2.17688	0.86115	2.52787	0.01148	0.04284	1.935	3.83303	3.81953	8.29373	19.0085	16.0864	Up
Sgca	148.169	-4.4553	0.95394	-4.6704	3.01E-06	3.35E-05	179.955	550.999	119.36	11.4039	3.16808	24.1296	Down
Pdk2	2374.66	-0.6159	0.16616	-3.7065	0.00021	0.00145	2728.35	3481.35	2412.03	2028.85	1951.53	1645.84	Down
Itga3	15310.5	0.56356	0.0899	6.26886	3.64E-10	8.66E-09	11852.9	12025.2	13194.6	17395.1	18713.8	18681.4	Up
Fam117a	415.635	0.45323	0.20653	2.19454	0.0282	0.0877	400.546	257.772	394.367	512.138	440.363	488.625	Up
Slc35b1	4225.75	0.27438	0.08965	3.06049	0.00221	0.01083	3709.4	3734.33	4031.52	4829.02	4606.38	4443.87	Up
Spop	16943.4	-0.3698	0.07664	-4.8251	1.40E-06	1.67E-05	18673.7	19047.3	19587.5	14961.9	15181.4	14208.3	Down
Nxph3	366.083	-1.1781	0.24322	-4.8436	1.27E-06	1.53E-05	377.326	607.536	538.554	241.555	172.132	259.393	Down
Abi3	353.523	-0.4482	0.1809	-2.4774	0.01324	0.04804	393.773	355.514	474.577	280.95	335.816	280.507	Down
Atp5g1	3293.3	-0.6888	0.23092	-2.9831	0.00285	0.01348	3195.66	5742.84	3256.15	2692.35	2697.09	2175.69	Down
Calcoco2	78.3152	-1.1031	0.46672	-2.3634	0.01811	0.06171	118.035	84.3268	118.405	40.4319	23.2326	85.4591	Down
Ttll6	1594.19	-0.6825	0.22281	-3.0632	0.00219	0.01075	1863.41	2015.22	2014.8	1369.5	793.075	1509.11	Down
Hoxb4	571.613	-0.8004	0.15813	-5.0618	4.15E-07	5.56E-06	739.171	639.159	800.192	397.062	472.043	382.052	Down
Skap1	388.293	-0.6716	0.14985	-4.4821	7.39E-06	7.53E-05	454.726	464.755	511.817	270.583	304.135	323.739	Down
Nfe2l1	13328.5	0.24355	0.07629	3.19242	0.00141	0.00745	12090.9	12298.3	12229.2	14212.3	15101.2	14039.4	Up
Copz2	1447.14	-0.4046	0.10006	-4.0433	5.27E-05	0.00043	1572.19	1717.2	1656.72	1308.34	1223.93	1204.47	Down
Cdk5rap3	2268.23	0.33294	0.10237	3.25246	0.00114	0.00626	1946.61	2125.42	1950.83	2700.64	2509.12	2376.77	Up
D030028A	26.8544	1.49224	0.46072	3.23889	0.0012	0.0065	14.5125	11.4991	16.233	34.2116	46.4651	38.2052	Up
Sp2	206.496	0.75613	0.1915	3.94837	7.87E-05	0.00062	134.483	164.82	161.375	278.877	267.174	232.248	Up
Lrrc46	792.906	-0.8242	0.22634	-3.6414	0.00027	0.00181	863.011	1157.58	1019.81	721.554	401.29	594.192	Down
Osbpl7	1699.76	0.82087	0.11931	6.87999	5.99E-12	1.92E-10	1180.35	1178.66	1327.29	2281.81	2279.96	1950.48	Up
Tbx21	101.9	-0.7204	0.31935	-2.2558	0.02408	0.07733	135.45	139.906	105.037	55.9827	106.659	68.3673	Down
Gm11592	63.4503	-0.9905	0.37885	-2.6146	0.00893	0.03477	71.5951	88.1598	93.5785	50.7991	24.2886	52.2808	Down
Arhgap23	2685.53	-0.2919	0.12989	-2.2471	0.02463	0.07873	3087.3	2583.47	3197.9	2280.77	2665.41	2298.35	Down
2410003L	51.5994	1.32457	0.35849	3.69486	0.00022	0.00151	32.895	30.6643	24.827	63.2397	65.4736	92.4969	Up
Cisd3	741.429	-0.3249	0.12211	-2.6606	0.0078	0.03113	756.586	885.431	831.703	671.792	630.447	672.613	Down
Lasp1	14564	0.34466	0.12443	2.76994	0.00561	0.02385	11070.2	13027.5	14399.6	16301.3	17177.3	15407.8	Up
B230217C	96.6305	-0.7913	0.28509	-2.7755	0.00551	0.02352	97.7176	141.822	127.954	80.8638	57.0254	74.3997	Down
Stac2	145.943	0.60278	0.19889	3.03068	0.00244	0.0118	119.97	114.033	113.631	185.572	180.58	161.87	Up
Ppp1r1b	130.453	-1.6711	0.35052	-4.7675	1.87E-06	2.16E-05	203.175	275.02	117.451	71.5334	61.2495	54.2916	Down
Stard3	4184.58	0.49835	0.122	4.0849	4.41E-05	0.00037	3701.66	3197.71	3507.28	5310.06	5081.59	4309.15	Up
Tcap	2352.69	-4.2936	0.58876	-7.2925	3.04E-13	1.19E-11	5109.38	6277.55	2044.4	200.086	127.779	356.917	Down
Pnmt	44.9368	-2.3313	0.52362	-4.4522	8.50E-06	8.52E-05	80.3026	94.8676	49.6539	16.5875	22.1765	6.03241	Down
Thra	3222.22	-0.4374	0.11046	-3.9601	7.49E-05	0.00059	3367.87	3857.95	3894.97	2720.34	2929.41	2562.77	Down
Msl1	485.169	0.33033	0.12618	2.61789	0.00885	0.03451	438.278	430.258	421.103	572.267	513.228	535.879	Up
Top2a	610.367	1.19072	0.21552	5.52475	3.30E-08	5.41E-07	352.171	441.757	321.796	869.805	643.119	1033.55	Up
Igfbp4	9296.91	-0.8815	0.13172	-6.6924	2.20E-11	6.52E-10	10695.7	14146.8	11313.5	6237.92	6579.04	6808.57	Down
Tns4	99.077	1.29869	0.33184	3.91358	9.09E-05	0.0007	50.3101	76.6607	44.8795	118.186	121.443	182.983	Up
Krt222	96.8116	-1.3743	0.50563	-2.718	0.00657	0.02705	90.9451	230.94	97.3981	43.5421	29.5687	88.4753	Down
A830036E	28.5295	3.15158	1.07778	2.92414	0.00345	0.01589	9.67501	0	7.63906	60.1295	88.7061	5.027	Up
Krt20	48.8266	5.41088	0.73292	7.38265	1.55E-13	6.45E-12	4.83751	0	1.90977	131.663	57.0254	97.5239	Up
Krt23	1597.14	1.27886	0.19622	6.51731	7.16E-11	1.94E-09	1026.52	1019.59	750.538	2059.95	2828.04	1898.2	Up
Krt34	11.4651	7.01941	1.4777	4.75022	2.03E-06	2.34E-05	0	0	0	26.9546	33.7928	8.04321	Up
Krt19	19382.6	0.71119	0.09638	7.37931	1.59E-13	6.59E-12	14625.7	14840.6	14632.6	25882.6	24444.9	21869.5	Up
Eif1	31413.6	-0.2716	0.09171	-2.9611	0.00307	0.01435	32957.9	34444.6	35681.1	29879.2	29157.9	26360.6	Down

Gm10039	321.438	-0.6075	0.26222	-2.3168	0.02052	0.06809	322.178	538.541	303.653	298.574	234.438	231.242	Down
Jup	18802.8	-0.2648	0.13109	-2.0197	0.04342	0.12222	19454.5	19225.5	22889.5	17517.4	18886	14843.7	Down
P3h4	1317.58	-0.4551	0.18203	-2.4999	0.01242	0.04565	1825.68	1329.1	1416.09	1262.72	1169.02	902.85	Down
Acly	15376.4	1.04451	0.13166	7.93363	2.13E-15	1.13E-13	11144.6	10200.7	8778.24	18506.4	22692.9	20935.5	Up
Ttc25	1233.09	-0.6143	0.19667	-3.1234	0.00179	0.00908	1379.66	1602.21	1493.44	1143.5	695.921	1083.82	Down
Cnp	3709.09	0.29612	0.09314	3.17929	0.00148	0.00774	3226.62	3548.43	3214.14	4233.95	4116.39	3915.03	Up
Nkiras2	2248.87	0.46027	0.14543	3.16499	0.00155	0.00805	2037.56	1832.19	1809.5	2727.6	2936.81	2149.55	Up
Dhx58	320.249	0.90082	0.20009	4.502	6.73E-06	6.91E-05	218.655	261.605	190.022	386.695	374.889	489.63	Up
Rab5c	13429.1	0.22542	0.09931	2.26971	0.02323	0.07518	12819.4	12207.3	12119.4	15021	15328.2	13079.3	Up
Kcnh4	60.5847	1.57303	0.50499	3.115	0.00184	0.00929	51.2776	17.2487	22.9172	103.672	115.107	53.2862	Up
Ghdc	953.043	0.27206	0.11714	2.32256	0.0202	0.06728	922.029	820.269	847.936	1071.96	1090.87	965.185	Up
Stat5a	1091.32	0.50545	0.12699	3.98036	6.88E-05	0.00055	907.516	964.966	833.613	1292.78	1391.84	1157.22	Up
Stat3	13730.7	0.44923	0.08531	5.26605	1.39E-07	2.01E-06	11280.1	11626.6	11923.6	14940.1	15955.5	16658.5	Up
Cavin1	22577	-0.4663	0.14824	-3.1455	0.00166	0.00852	24665.5	23988.1	29928.9	19435.3	21477.4	15966.8	Down
Atp6v0a1	9799.31	1.02669	0.15951	6.43633	1.22E-10	3.20E-09	7444.92	5827.17	6085.47	12525.6	15648.2	11264.5	Up
Naglu	1865.31	0.87475	0.1036	8.44373	3.07E-17	2.12E-15	1315.8	1368.39	1265.22	2443.54	2560.86	2238.02	Up
Mlx	2101.5	0.20361	0.09984	2.03927	0.04142	0.11777	1881.79	1977.85	2000.48	2327.43	2342.26	2079.17	Up
Psmc3ip	368.933	-0.5423	0.19958	-2.7173	0.00658	0.0271	431.506	458.048	423.013	348.337	224.933	327.761	Down
Ramp2	15762.8	-0.9444	0.14945	-6.3192	2.63E-10	6.49E-09	17756.6	19934.7	24544.3	11052.4	11713.4	9575.44	Down
Wnk4	1214	0.81179	0.15384	5.27693	1.31E-07	1.91E-06	904.614	793.438	945.334	1677.41	1683.3	1279.88	Up
Coa3	3115.08	0.2439	0.11355	2.14791	0.03172	0.09565	2713.84	3031.93	2811.18	3593.26	3526.07	3014.19	Up
Aoc3	455.485	-3.1784	0.40803	-7.7897	6.72E-15	3.34E-13	503.101	1475.72	482.216	72.5701	109.827	89.4807	Down
Ifi35	2965.34	-0.2712	0.12401	-2.1873	0.02872	0.08894	2876.38	3622.22	3231.32	2801.21	2488	2772.9	Down
Rnd2	571.841	-0.4037	0.14309	-2.8213	0.00478	0.0209	659.836	683.238	611.125	513.174	429.802	533.868	Down
Brca1	122.275	0.9544	0.26279	3.63175	0.00028	0.00188	94.8151	88.1598	66.8418	162.764	133.059	188.01	Up
Nbr1	8261.47	0.20354	0.08815	2.30907	0.02094	0.06912	8143.46	7695.78	7199.82	8634.81	8888.57	9006.38	Up
Tmem106	2314.66	0.97873	0.14981	6.5332	6.44E-11	1.76E-09	1746.34	1604.12	1324.42	3003.37	3467.99	2741.73	Up
Rdm1	1307.18	-0.4838	0.18142	-2.6667	0.00766	0.03068	1712.48	1215.07	1645.26	1126.91	1234.49	908.882	Down
Arl4d	3647.12	-1.2582	0.20999	-5.9915	2.08E-09	4.30E-08	4591.76	5254.13	5585.11	2745.22	2214.49	1492.01	Down
Sost	14.429	-4.3207	1.11536	-3.8738	0.00011	0.00081	10.6425	54.6207	17.1879	0	2.11205	2.0108	Down
Mpp3	367.052	-1.3695	0.2218	-6.1744	6.64E-10	1.50E-08	545.671	420.676	621.629	185.572	177.412	251.35	Down
Lsm12	1836.73	0.33056	0.09305	3.55252	0.00038	0.00245	1536.39	1698.99	1646.22	2069.28	2047.63	2021.86	Up
G6pc3	2634.54	0.28243	0.12943	2.18212	0.0291	0.08985	2153.66	2687.92	2290.76	3146.43	2868.16	2660.29	Up
Asb16	6.79122	-2.218	1.07172	-2.0695	0.03849	0.11123	3.87001	21.0817	8.59395	2.07343	2.11205	3.0162	Down
Tmub2	3011.25	0.22897	0.081	2.82673	0.0047	0.0206	2795.11	2754.99	2768.21	3357.92	3228.27	3162.99	Up
Rundc3a	318.226	1.78611	0.17906	9.97512	1.96E-23	2.27E-21	131.58	153.321	144.187	485.183	560.749	434.333	Up
Slc25a39	5581.69	0.28477	0.11581	2.45907	0.01393	0.05007	5486.7	5180.35	4430.66	6198.52	6404.79	5789.1	Up
Grn	13909.1	0.64561	0.12521	5.1561	2.52E-07	3.52E-06	11659.4	10499.6	10384.4	17370.2	18896.5	14644.7	Up
Itga2b	713.077	-1.2543	0.26208	-4.7859	1.70E-06	2.00E-05	602.753	1321.44	1090.48	414.686	430.858	418.247	Down
2810433D	94.4622	-0.9457	0.35201	-2.6865	0.00722	0.02925	169.313	87.2015	116.496	58.0561	83.426	52.2808	Down
Adam11	123.886	-1.6505	0.38295	-4.3101	1.63E-05	0.00015	171.248	286.519	105.992	67.3865	53.8573	58.3133	Down
Gjc1	1685.15	-0.6595	0.12891	-5.1159	3.12E-07	4.28E-06	1816	2193.45	2181.91	1269.98	1222.88	1426.66	Down
Higd1b	885.306	-0.2958	0.13398	-2.208	0.02725	0.08526	878.491	938.135	1110.53	792.051	819.476	773.153	Down
Eftud2	5634.7	-0.2294	0.08922	-2.5714	0.01013	0.03863	5921.11	5806.09	6518.03	5188.76	5273.79	5100.4	Down
Ccdc103	453.298	-0.4951	0.23458	-2.1108	0.03479	0.10277	452.791	587.413	550.967	403.282	260.838	464.495	Down
Nmt1	6223.29	0.48549	0.11219	4.32746	1.51E-05	0.00014	5704.39	4844	5009.32	7378.31	7725.88	6677.87	Up
Acbd4	1922.87	-0.3391	0.10609	-3.1968	0.00139	0.00736	1947.58	2257.66	2238.25	1651.49	1736.11	1706.17	Down
Fmnl1	2053.47	0.54531	0.18447	2.95616	0.00311	0.01455	2024.98	1566.75	1418	2314.99	2920.97	2075.15	Up
Plekhm1	3466.58	0.40614	0.1368	2.96888	0.00299	0.01403	3202.43	2522.14	3220.82	3819.26	4333.93	3700.88	Up
Gm884	8.28094	3.58039	1.3851	2.58493	0.00974	0.03738	0	3.83303	0	13.4773	4.2241	28.1512	Up
Rprml	129.898	-0.6895	0.28765	-2.3971	0.01653	0.05738	156.735	148.53	175.698	75.6803	137.283	85.4591	Down
Nsf	3561.62	0.71235	0.12957	5.49789	3.84E-08	6.21E-07	2979.9	2481.89	2637.39	4184.19	5003.45	4082.93	Up
Arf2	3601.14	0.36753	0.11334	3.24271	0.00118	0.00642	3435.6	2843.15	3155.89	4016.24	4323.37	3832.59	Up
Mapt	7644.88	-0.9141	0.16184	-5.6483	1.62E-08	2.81E-07	8495.63	10119.2	11351.6	4777.19	6329.82	4795.76	Down
Cdc27	2706.29	0.26379	0.1167	2.26044	0.02379	0.07658	2330.71	2622.75	2425.4	2733.82	2834.37	3290.68	Up
Myl4	9952.4	-5.1479	0.85941	-5.99	2.10E-09	4.33E-08	10983.1	35624.2	11469.1	455.118	199.589	983.282	Down
Gm11639	18.4617	2.63268	0.75386	3.49227	0.00048	0.00299	7.74001	6.70781	0.95488	24.8812	23.2326	47.2538	Up
Mrc2	4269.52	-0.4316	0.191	-2.2599	0.02383	0.07666	3587.5	5507.11	5615.67	3563.19	4136.45	3207.23	Down
Tanc2	3001.89	0.44933	0.12593	3.5682	0.00036	0.00233	2183.65	2828.78	2602.06	3381.77	3384.56	3630.5	Up
Cyb561	9345.42	0.39496	0.08037	4.9144	8.91E-07	1.11E-05	8169.58	7934.38	8118.41	11174.8	10283.6	10391.8	Up
Ace	23661.9	-0.3465	0.10771	-3.2166	0.0013	0.00695	27103.6	26222.7	26141.8	22643.9	21480.6	18378.7	Down
Ace3	248.908	-0.6315	0.24891	-2.5368	0.01119	0.04189	327.983	256.813	322.75	261.252	165.796	158.853	Down
Dcaf7	5692.97	0.21816	0.08633	2.52715	0.0115	0.04291	5385.11	4985.82	5418.96	6164.31	6313.98	5889.64	Up
Limd2	6468.28	0.6294	0.12639	4.97977	6.37E-07	8.24E-06	5454.77	4925.45	4857.49	8118.52	8692.14	6761.32	Up
Ccdc47	2463.67	0.57475	0.12756	4.5056	6.62E-06	6.81E-05	2139.15	1747.86	2051.09	2746.26	2850.21	3247.44	Up
Scn4a	106.416	-1.2797	0.34376	-3.7225	0.0002	0.00138	126.743	214.65	110.766	57.0194	76.0338	53.2862	Down
Icam2	8758.23	-0.6154	0.15735	-3.911	9.19E-05	0.0007	8716.22	10300.3	12778.2	7053.81	7406.96	6293.81	Down
Ern1	2569.08	0.37592	0.11124	3.37925	0.00073	0.00426	2441.01	2007.55	2260.21	2797.06	2997	2911.64	Up
Tex2	2431.05	0.25191	0.08829	2.85318	0.00433	0.0192	2303.62	2181.95	2172.36	2672.65	2716.1	2539.64	Up
Pecam1	34154.4	-0.314	0.11453	-2.7416	0.00611	0.02556	34978.1	36493.4	42097.9	29063.3	33157.1	29136.5	Down
Milr1	316.116	1.53663	0.2283	6.73087	1.69E-11	5.11E-10	178.988	187.819	119.36	471.706	540.685	398.139	Up
Smurf2	7798.57	-0.2366	0.10847	-2.1809	0.02919	0.09003	7957.7	8022.54	9329.21	6683.71	7356.27	7441.98	Down
Kpna2	2817.62	0.24564	0.12246	2.00589	0.04487	0.12537	2653.86	2234.66	2846.51	3152.65	2930.47	3087.59	Up
1810010H	4458.04	-0.7601	0.21413	-3.5499	0.00039	0.00247	4498.88	5549.28	6770.12	3943.67	2394.01	3592.3	Down
Pitpnc1	6981.01	-0.2854	0.13526	-2.1102	0.03484	0.10285	8342.77	6812.26	7852.96	5651.14	7191.53	6035.42	Down
Helz	1955.02	0.26431	0.10561	2.5028	0.01232	0.04535	1766.66	1640.54	1922.18	2109.72	2076.15	2214.9	Up
Cep112	288.367	-0.8687	0.20891	-4.1582	3.21E-05	0.00028	339.593	368.93	409.645	201.123	161.572	249.339	Down
Axin2	984.675	-0.3255	0.14895	-2.1854	0.02886	0.08932	961.696	1042.59	1281.45	845.96	939.863	836.494	Down
Gm11668	5.8422	3.47488	1.35808	2.55868	0.01051	0.03985	2.9025	0	0	12.4406	12.6723	7.03781	Up
Rgs9	1471.71	-0.7114	0.14066	-5.0578	4.24E-07	5.67E-06	1515.11	2016.18	1950.83	1104.1	1132.06	1111.97	Down
Slc16a6	816.235	0.89387	0.14496	6.1662	6.99E-10	1.58E-08	520.516	627.659	565.291	925.787	1133.12	1125.04	Up
Arsg	494.741	1.20703	0.1502	8.03615	9.27E-16	5.19E-14	324.113	313.351	259.728	658.315	741.33	671.608	Up
Prkar1a	22186.2	-0.2335	0.10022	-2.33	0.0198	0.0663	21513.4	25292.3	25127.7	20190	20964.2	20029.6	Down

Fam20a	650.654	-0.4251	0.1877	-2.2648	0.02353	0.07591	597.916	764.69	874.673	561.9	625.167	479.576	Down
Abca8b	1344.34	-0.4973	0.11545	-4.3074	1.65E-05	0.00015	1473.5	1614.67	1632.85	1125.87	1199.64	1019.48	Down
Abca8a	4344.8	-0.7688	0.14318	-5.3695	7.90E-08	1.20E-06	6157.18	5234.01	5036.05	3603.62	3303.25	2734.69	Down
Abca6	115.445	-0.7518	0.30204	-2.4892	0.0128	0.04679	118.035	170.57	146.097	68.4232	73.9218	115.621	Down
Kcnj16	5.45505	2.9084	1.39971	2.07785	0.03772	0.10953	1.935	1.91652	0	20.7343	2.11205	6.03241	Up
Kcnj2	1545.26	0.35452	0.1499	2.36509	0.01803	0.06156	1427.06	1250.53	1391.26	1607.95	2064.53	1530.22	Up
Slc39a11	1665.2	1.00544	0.1548	6.49503	8.30E-11	2.22E-09	1220.02	1213.16	888.996	2279.74	2049.75	2339.57	Up
Cdc42ep4	7486.98	-0.3946	0.09682	-4.076	4.58E-05	0.00038	8793.62	7922.88	8797.34	6873.43	6402.68	6131.94	Down
Sdk2	123.622	-0.8092	0.31776	-2.5466	0.01088	0.04098	121.905	201.234	148.962	82.9373	119.331	67.3619	Down
4932435C	2.40222	-4.6608	2.01488	-2.3132	0.02071	0.06851	4.83751	7.66607	1.90977	0	0	0	Down
Rpl38	1307.09	-0.461	0.13277	-3.4725	0.00052	0.00318	1550.9	1313.77	1677.73	1102.03	1159.52	1038.58	Down
Ttyh2	662.097	1.04036	0.18295	5.6866	1.30E-08	2.29E-07	382.163	440.799	476.487	832.483	1085.59	755.056	Up
Dnaic2	2333.8	-0.6101	0.22107	-2.7597	0.00579	0.02448	2483.58	3297.37	2679.4	1983.24	1260.89	2298.35	Down
Btbd17	110.54	1.87656	0.34937	5.3712	7.82E-08	1.19E-06	30.96	64.2033	46.7893	214.6	126.723	179.967	Up
Gpr142	25.4428	2.10489	0.53403	3.94149	8.10E-05	0.00063	6.77251	15.3321	6.68418	44.5788	39.0729	40.216	Up
Cd300a	734.204	0.91106	0.18151	5.01928	5.19E-07	6.81E-06	549.541	550.999	428.742	882.245	1153.18	840.515	Up
Cd300lb	426.684	0.76985	0.28737	2.67891	0.00739	0.02982	440.213	295.144	211.029	479.999	684.304	449.414	Up
Cd300ld	376.644	1.02426	0.24919	4.11041	3.95E-05	0.00033	293.153	276.937	174.744	473.779	621.999	419.252	Up
Cd300c2	1264.07	1.80438	0.23336	7.73218	1.06E-14	5.07E-13	716.919	571.122	400.096	1903.41	2399.29	1593.56	Up
Slc9a3r1	5475.54	0.35297	0.12086	2.92036	0.0035	0.01606	4917.81	5062.48	4446.89	6919.04	5562.09	5944.94	Up
Nat9	1628.7	-0.3376	0.11827	-2.8548	0.00431	0.01912	1721.19	2033.42	1700.65	1509.46	1392.9	1414.6	Down
Grin2c	158.821	0.84407	0.42787	1.97274	0.04853	0.13339	205.11	76.6607	59.2027	198.013	203.813	210.129	Up
Fdxr	494.327	-0.3317	0.12179	-2.7233	0.00646	0.02672	567.923	547.166	537.599	425.053	449.867	438.355	Down
Fads6	253.479	-0.9498	0.19917	-4.7687	1.85E-06	2.15E-05	375.391	335.391	291.239	200.086	168.964	149.805	Down
Jpt1	3750.02	0.31098	0.13614	2.2842	0.02236	0.07279	3194.69	3466.02	3381.24	4227.73	4744.72	3485.72	Up
Nup85	2249.2	-0.3415	0.09696	-3.5222	0.00043	0.00271	2449.71	2483.81	2608.74	2005.01	2091.99	1855.97	Down
Grb2	5547.49	0.37712	0.11219	3.36141	0.00078	0.0045	5309.65	4633.18	4536.65	6304.27	6712.1	5789.1	Up
Caskin2	7105.25	-0.4317	0.10952	-3.9419	8.08E-05	0.00063	7597.79	7699.61	9184.06	5950.75	6203.09	5996.21	Down
Llgl2	3520.97	0.34942	0.11397	3.06578	0.00217	0.01067	3055.37	2959.1	3275.25	4200.77	4137.51	3497.79	Up
Itgb4	1828.6	-0.7578	0.14423	-5.2539	1.49E-07	2.15E-06	2517.44	2165.66	2211.51	1424.45	1138.4	1514.13	Down
Trim47	1325.61	-0.2899	0.09189	-3.1549	0.00161	0.00829	1490.92	1452.72	1431.37	1175.64	1218.65	1184.36	Down
Acox1	10718	0.33218	0.1232	2.6964	0.00701	0.02856	10688	9638.17	8142.29	11734.6	12337.5	11767.2	Up
Ten1	894.563	0.34696	0.15332	2.26294	0.02364	0.07618	675.316	761.816	925.282	955.852	1071.87	977.25	Up
Evpl	682.79	-0.5576	0.13148	-4.2409	2.23E-05	0.0002	793.351	760.857	885.176	595.075	515.34	546.938	Down
Gm11739	24.7038	-1.1305	0.46676	-2.4221	0.01543	0.05429	31.9275	29.706	40.1051	17.6242	14.7844	14.0756	Down
Sphk1	2208.23	-0.9509	0.16574	-5.7374	9.62E-09	1.75E-07	2769.96	2648.63	3313.44	1809.07	1441.47	1266.81	Down
Cygb	1926.39	-0.2826	0.13399	-2.1088	0.03496	0.10317	2238.8	2321.86	1782.77	1792.48	1657.96	1764.48	Down
Prcd	530.737	1.01533	0.2042	4.97228	6.62E-07	8.52E-06	421.831	317.184	315.111	727.775	566.03	836.494	Up
St6galnac	726.395	0.53869	0.18837	2.85968	0.00424	0.01887	674.349	532.792	570.065	807.602	713.873	1059.69	Up
Gm11735	1210.47	2.02518	0.72382	2.7979	0.00514	0.02222	517.613	548.124	366.675	1330.11	694.865	3805.44	Up
St6galnac	283.365	1.88389	0.83216	2.26385	0.02358	0.07607	160.605	114.033	87.8492	281.987	126.723	928.99	Up
Mxra7	3153.2	-0.5007	0.11934	-4.1955	2.72E-05	0.00024	3604.91	3773.62	3705.9	2545.14	2961.1	2328.51	Down
Jmjd6	2212.44	-0.2748	0.10971	-2.5051	0.01224	0.04512	2398.44	2281.61	2587.73	2102.46	1833.26	2071.13	Down
Mettl23	1143.74	-0.2757	0.11859	-2.3245	0.0201	0.06705	1220.99	1274.48	1262.36	1128.98	1048.63	926.98	Down
Mfsd11	2131.54	0.23639	0.12027	1.96545	0.04936	0.1354	2013.37	2080.38	1777.99	2407.25	2433.08	2077.16	Up
Snhg20	950.696	-0.6324	0.14229	-4.4445	8.81E-06	8.78E-05	1194.86	1006.17	1266.17	773.39	790.963	672.613	Down
Gm11730	90.801	-0.7741	0.31452	-2.4613	0.01384	0.04984	128.678	85.285	129.864	50.7991	70.7537	79.4267	Down
Septin9	5932.17	0.46517	0.12742	3.6508	0.00026	0.00175	5535.08	4816.21	4600.63	6659.86	7720.6	6260.63	Up
Gm11734	185.495	-1.4462	0.33266	-4.3473	1.38E-05	0.00013	184.793	344.973	284.555	83.974	73.9218	140.756	Down
2900041M	44.054	-0.8771	0.43872	-1.9991	0.04559	0.12684	71.5951	57.4955	42.0148	43.5421	29.5687	20.108	Down
Tnrc6c	2554.56	-0.3122	0.092	-3.3936	0.00069	0.00408	2913.15	2746.37	2830.27	2149.11	2312.7	2375.76	Down
Tmc6	3492.99	-0.3325	0.11565	-2.8748	0.00404	0.01814	3727.78	4333.25	3619.96	3276.02	3118.44	2882.48	Down
Tha1	516.681	0.29184	0.13424	2.17402	0.0297	0.09119	420.863	484.879	487.945	553.606	599.822	552.97	Up
Socs3	5076.83	1.58994	0.14802	10.7412	6.52E-27	1.06E-24	2259.12	2542.26	2793.99	7251.83	8952.98	6660.78	Up
Pgs1	4457.28	0.78546	0.09642	8.14639	3.75E-16	2.23E-14	3276.93	3030.01	3512.06	5536.06	5818.7	5569.92	Up
Dnah17	79.1617	0.65804	0.28281	2.32679	0.01998	0.06676	56.1151	53.6625	74.4809	100.561	86.5941	103.556	Up
Timp2	26892.5	-0.2407	0.10476	-2.2973	0.0216	0.07083	31570.5	27630.4	28190.1	24690.4	26460.8	22812.5	Down
Lgals3bp	17119.8	1.68253	0.1999	8.4168	3.87E-17	2.62E-15	8928.1	9055.54	6415.86	29339.1	19386.5	29594	Up
Cant1	4328.89	0.36773	0.09503	3.86967	0.00011	0.00082	3946.44	3583.89	3809.98	4930.62	5123.84	4578.6	Up
Gm11747	626.753	-0.8721	0.16473	-5.2941	1.20E-07	1.75E-06	741.106	959.217	731.44	479.999	444.587	404.171	Down
Engase	641.523	-0.3778	0.17644	-2.1415	0.03223	0.09674	643.388	883.514	648.365	535.982	519.564	618.322	Down
Rbfox3	91.5542	-0.8827	0.25969	-3.399	0.00068	0.00401	113.198	108.283	134.638	58.0561	71.8097	63.3403	Down
Ccdc40	2780.52	-0.5856	0.23577	-2.4836	0.01301	0.04738	3077.62	3719.96	3214.14	2510.93	1386.56	2773.9	Down
Gaa	7096.1	0.34436	0.1296	2.65706	0.00788	0.03142	6760.9	6668.52	5330.16	7828.24	8575.98	7412.82	Up
Sgsh	498.925	1.00431	0.17297	5.80619	6.39E-09	1.21E-07	366.683	264.479	364.765	658.315	631.503	707.802	Up
Endov	1534.76	0.2523	0.10923	2.30986	0.0209	0.06898	1350.63	1315.69	1536.41	1698.14	1613.61	1694.1	Up
Gm11762	3.74635	3.42481	1.57987	2.16778	0.03018	0.09218	0	0	1.90977	5.18358	6.33615	9.04861	Up
Chmp6	1525.45	0.34251	0.15436	2.21886	0.0265	0.08343	1300.32	1441.22	1293.87	1719.91	1994.83	1402.53	Up
Ndufaf8	498.859	-0.366	0.14807	-2.4718	0.01345	0.04868	502.133	555.79	627.358	459.265	441.419	407.187	Down
Slc38a10	7251.23	0.35831	0.09646	3.71464	0.0002	0.00141	6942.79	6022.66	6100.75	7952.65	8325.7	8162.85	Up
2810410L	1121.96	-0.3412	0.16613	-2.0536	0.04001	0.11456	1187.12	1160.45	1414.18	984.88	1163.74	821.413	Down
Gm11770	1.91152	4.43453	2.2058	2.0104	0.04439	NA	0	0	0	5.18358	5.28013	1.0054	Up
Actg1	47182.3	0.44384	0.11477	3.86706	0.00011	0.00083	35294.5	40596.6	44052.6	52718	57905	52527.2	Up
0610009L	195.748	-0.8232	0.21919	-3.7556	0.00017	0.00123	206.078	275.978	268.322	119.222	152.068	152.821	Down
Oxld1	221.256	-0.4358	0.19502	-2.2348	0.02543	0.08077	240.908	297.06	225.352	199.049	172.132	193.037	Down
Ccdc137	1732.5	-0.221	0.11152	-1.9817	0.04752	0.13117	1896.3	1754.57	1944.14	1660.82	1453.09	1686.06	Down
Slc25a10	4959.09	0.33323	0.1061	3.14065	0.00169	0.00863	4573.38	4465.49	4127.96	5997.4	5063.64	5526.69	Up
Gcgr	92.5169	-2.4595	0.36375	-6.7616	1.36E-11	4.18E-10	190.598	113.075	166.15	32.1382	17.9524	35.189	Down
Mcrip1	3365.29	-0.2775	0.11247	-2.4677	0.0136	0.04913	3526.54	3604.97	3932.21	3252.18	3151.18	2724.64	Down
P4hb	37383.8	0.53497	0.08388	6.37759	1.80E-10	4.59E-09	31802.7	29332.3	30457.9	45981.5	44665.6	42063	Up
Anapc11	1861.4	-0.3588	0.09725	-3.69	0.00022	0.00153	2190.42	2062.17	2022.44	1712.65	1627.34	1553.34	Down
Pcyt2	4238.91	0.38362	0.11628	3.29915	0.00097	0.00544	3465.59	4004.56	3565.53	5136.93	4951.7	4309.15	Up
Myadml2	85.2794	-2.8922	0.55517	-5.2095	1.89E-07	2.69E-06	89.0101	288.436	73.526	20.7343	15.8404	24.1296	Down

Lrrc45	4782.06	-0.2965	0.14415	-2.057	0.03969	0.11382	4644.97	5471.66	5698.74	4690.1	3605.27	4581.61	Down
Cbr2	284462	-0.2791	0.13594	-2.0531	0.04007	0.11469	333171	298938	303566	296682	216221	258192	Down
Gm11771	234.326	-0.4208	0.2119	-1.986	0.04703	0.1301	277.673	298.018	229.172	233.261	164.74	203.091	Down
Rfng	1646.63	-0.2324	0.10708	-2.1704	0.02998	0.09176	1816	1650.12	1870.62	1521.9	1592.49	1428.67	Down
Fasn	38407.2	1.69596	0.17898	9.47589	2.64E-21	2.62E-19	17850.4	22763.4	13736.9	54575.8	61003.4	60513.1	Up
Slc16a3	1101.02	-0.2775	0.11478	-2.4178	0.01562	0.05481	1276.13	1224.65	1119.12	1029.46	927.19	1029.53	Down
Gm11775	37.8213	2.72843	0.63004	4.33054	1.49E-05	0.00014	8.70751	12.4574	8.59395	101.598	22.1765	73.3943	Up
Sectm1b	248.523	3.93796	0.43468	9.05954	1.31E-19	1.10E-17	42.5701	28.7478	20.0525	650.021	203.813	545.933	Up
Sectm1a	127.208	4.23912	0.50529	8.38949	4.88E-17	3.24E-15	12.5775	7.66607	18.1428	267.473	103.49	353.901	Up
Fn3k	196.965	-1.1641	0.22407	-5.1954	2.04E-07	2.89E-06	254.453	335.391	227.262	119.222	128.835	116.627	Down
Tbcd	2612.46	0.50709	0.09031	5.61491	1.97E-08	3.35E-07	2262.99	2173.33	2037.72	3031.36	3130.06	3039.33	Up
B3gntl1	360.329	0.47192	0.18498	2.55122	0.01073	0.04057	316.373	338.265	251.134	362.851	451.979	441.371	Up
Metrnl	1045.23	0.75024	0.2187	3.43038	0.0006	0.00363	920.094	842.309	575.794	1407.86	1504.84	1020.48	Up
Rab10	1315.08	0.38568	0.11041	3.49305	0.00048	0.00298	1162.94	1066.54	1191.69	1490.8	1395.01	1583.51	Up
Asxl2	2194.28	0.29191	0.11249	2.59496	0.00946	0.0365	2184.62	1918.43	1816.19	2301.51	2461.6	2483.34	Up
Dnmt3a	2144.52	0.37717	0.09512	3.96532	7.33E-05	0.00058	1980.48	1840.81	1776.08	2342.98	2480.6	2446.14	Up
Efr3b	706.346	0.56668	0.12971	4.36864	1.25E-05	0.00012	574.696	542.374	591.073	753.692	895.51	880.731	Up
Dnajc27	708.888	-0.4476	0.16463	-2.719	0.00655	0.02699	774.001	886.389	793.508	490.367	692.753	616.311	Down
Cenpo	401.409	0.3119	0.12905	2.41689	0.01565	0.05492	366.683	357.43	350.442	435.421	454.091	444.387	Up
Ncoa1	2995.2	0.53194	0.08378	6.34939	2.16E-10	5.42E-09	2537.76	2463.68	2346.15	3525.87	3577.81	3519.91	Up
Itsn2	7000.73	0.55426	0.09326	5.94325	2.79E-09	5.68E-08	5750.83	5425.66	5840.06	7949.54	8949.81	8088.45	Up
Fam228b	66.1375	-0.8649	0.33294	-2.5979	0.00938	0.03626	64.8226	85.285	105.992	46.6522	53.8573	40.216	Down
Fkbp1b	423.506	0.57573	0.17158	3.35545	0.00079	0.00458	378.293	356.472	285.51	532.872	536.461	451.425	Up
Mfsd2b	421.494	-0.6523	0.1987	-3.2827	0.00103	0.0057	416.026	556.748	572.93	322.419	278.791	382.052	Down
Ubxn2a	1904.27	0.51774	0.12028	4.30441	1.67E-05	0.00016	1719.25	1413.43	1566.01	2075.51	2294.74	2356.66	Up
2810032G	96.3616	1.3803	0.31327	4.40609	1.05E-05	0.0001	68.6926	49.8294	42.0148	126.479	117.219	173.934	Up
Apob	145.52	1.38217	0.43821	3.15415	0.00161	0.00831	79.3351	112.116	50.6088	115.075	340.04	175.945	Up
Ldah	2940.43	0.38694	0.0987	3.92039	8.84E-05	0.00068	2616.12	2641.92	2387.21	3460.56	3410.96	3125.79	Up
Gm48898	197.207	0.81745	0.39782	2.05484	0.03989	0.11427	124.808	233.815	69.7065	263.326	292.519	199.069	Up
Gm4755	33.066	1.15683	0.42912	2.69583	0.00702	0.0286	20.3175	20.1234	21.0074	33.1749	46.4651	57.3078	Up
Sdc1	21271.4	1.17454	0.11281	10.4114	2.20E-25	3.08E-23	14684.7	11682.1	12816.4	30285.6	29717.6	28441.8	Up
Wdr35	2025.55	-0.5012	0.1601	-3.1308	0.00174	0.00888	2278.47	2563.34	2280.26	1691.92	1350.66	1988.68	Down
Osr1	474.278	-0.7929	0.1748	-4.5358	5.74E-06	5.98E-05	506.971	641.075	656.005	357.667	383.337	300.615	Down
Gm5182	187.035	0.87281	0.31806	2.74417	0.00607	0.02539	201.24	101.575	93.5785	240.518	266.118	219.177	Up
Rdh14	1370.04	-0.3482	0.11926	-2.92	0.0035	0.01606	1537.36	1688.45	1377.9	1243.02	1211.26	1162.24	Down
Gm38407	89.8847	0.61274	0.26093	2.3483	0.01886	0.06379	79.3351	69.9529	63.9772	93.3044	115.107	117.632	Up
Gen1	80.2155	0.92448	0.36233	2.55148	0.01073	0.04056	49.3426	74.7442	42.0148	73.6068	110.883	130.702	Up
Vsnl1	735.243	-0.5616	0.16536	-3.3959	0.00068	0.00405	759.489	901.721	968.251	542.202	694.865	544.927	Down
Gm4294	211.524	-0.4779	0.22572	-2.1171	0.03425	0.1015	272.835	252.022	213.894	135.81	198.533	196.053	Down
Mycn	127.217	1.23593	0.3134	3.94364	8.03E-05	0.00063	65.7901	70.9111	90.7139	138.92	248.166	148.799	Up
Trib2	5241.39	-0.5145	0.14022	-3.669	0.00024	0.00164	5342.54	6418.42	6737.65	4148.94	3890.4	4910.38	Down
Lpin1	2461.7	0.39855	0.20149	1.97802	0.04793	0.13209	2461.32	2402.35	1507.76	2550.32	3209.26	2639.18	Up
Ntsr2	69.9892	1.46679	0.47593	3.08196	0.00206	0.01019	48.3751	49.8294	13.3684	117.149	108.771	82.4429	Up
Greb1	64.3076	-0.9725	0.30314	-3.208	0.00134	0.00712	96.7501	81.452	77.3455	47.6889	45.4091	37.1998	Down
E2f6	2067.77	-0.4859	0.09878	-4.9187	8.71E-07	1.09E-05	2309.43	2600.71	2328	1688.81	1719.21	1760.46	Down
Rock2	11069.6	-0.2957	0.1231	-2.4023	0.01629	0.05672	10519.6	12262.8	13818.1	9623.83	10060.8	10132.4	Down
Pqlc3	3744.4	0.51927	0.12549	4.13802	3.50E-05	0.0003	3325.3	2933.23	2974.46	4245.35	5007.67	3980.38	Up
Pdia6	14351.4	0.23303	0.10036	2.32207	0.02023	0.06734	12310.5	12663.4	14610.7	15670	15294.4	15559.6	Up
Atp6v1c2	5269.51	0.54981	0.13129	4.18784	2.82E-05	0.00025	4977.8	3673.01	4181.43	6212	6298.14	6274.71	Up
Gm36862	7.59958	-2.1841	0.98345	-2.2208	0.02636	0.08308	5.80501	21.0817	10.5037	2.07343	2.11205	4.0216	Down
Gm49329	835.343	-0.5778	0.13305	-4.3428	1.41E-05	0.00013	914.289	1007.13	1079.97	618.919	655.792	735.953	Down
5730507C	73.5292	1.33894	0.37497	3.57081	0.00036	0.00231	53.2126	41.2051	30.5563	85.0107	145.732	85.4591	Up
Gm10479	2.06943	4.55018	2.1549	2.11155	0.03473	NA	0	0	0	8.29373	2.11205	2.0108	Up
1700030C	215.2	-1.1759	0.41262	-2.8498	0.00438	0.01937	140.288	408.218	346.622	98.488	104.547	193.037	Down
Itgb1bp1	1435.23	0.24261	0.11282	2.15052	0.03151	0.0952	1388.36	1281.19	1274.77	1574.77	1670.63	1421.64	Up
Cpsf3	3724.74	0.42046	0.1153	3.64653	0.00027	0.00178	3563.31	3039.6	2954.41	4442.33	4361.38	3987.42	Up
Iah1	1569.84	0.76063	0.2414	3.15095	0.00163	0.00839	1689.26	872.015	934.83	1971.83	1863.88	2087.21	Up
Adam17	3228.77	0.61674	0.09121	6.76157	1.37E-11	4.18E-10	2560.01	2500.1	2586.78	3646.13	4030.85	4048.75	Up
Grhl1	521.453	-1.0662	0.16986	-6.277	3.45E-10	8.27E-09	741.106	729.235	647.411	344.19	276.679	390.096	Down
Klf11	3148.23	-0.3098	0.13755	-2.2522	0.02431	0.07789	3888.39	2897.77	3668.66	2658.14	2923.08	2853.33	Down
Rrm2	292.659	1.09542	0.22571	4.85327	1.21E-06	1.47E-05	185.76	211.775	162.33	474.816	302.023	419.252	Up
Mboat2	266.462	-0.6735	0.31916	-2.1103	0.03483	0.10285	233.168	505.961	243.495	227.041	210.149	178.961	Down
Id2	4916.65	1.08919	0.1615	6.7444	1.54E-11	4.67E-10	3463.66	3122.01	2846.51	6954.29	7780.79	5332.65	Up
Gm36723	137.299	0.87675	0.3188	2.75015	0.00596	0.02502	140.288	87.2015	63.0223	161.728	179.524	192.032	Up
Gm9866	3.60021	4.32213	1.66039	2.60308	0.00924	0.03579	0	0.95826	0	9.33044	5.28013	6.03241	Up
Gm31333	9.51557	2.68555	0.83368	3.22133	0.00128	0.00685	2.9025	2.87478	1.90977	14.514	14.7844	20.108	Up
Sntg2	129.549	-1.1727	0.3356	-3.4944	0.00048	0.00297	147.06	259.688	131.774	70.4967	70.7537	97.5239	Down
Acp1	2335.87	0.46594	0.2102	2.2167	0.02664	0.08376	2694.49	1652.04	1539.27	2820.9	2621.05	2687.44	Up
Sh3yl1	843.253	0.67403	0.1558	4.32618	1.52E-05	0.00014	727.561	693.779	528.05	1089.59	1000.06	1020.48	Up
Fam110c	367.78	0.98842	0.16294	6.06632	1.31E-09	2.84E-08	259.29	220.399	259.728	489.33	538.573	439.36	Up
Lamb1	5238.09	-0.4005	0.12652	-3.1653	0.00155	0.00804	5234.18	5904.79	6742.43	4858.05	4307.53	4381.54	Down
Dld	6274.33	-0.3112	0.12522	-2.4852	0.01295	0.04719	6180.4	8027.33	6637.39	5690.53	5739.5	5370.85	Down
Slc26a4	4875.53	4.96379	0.18975	26.1591	#######	#######	381.196	306.643	220.578	8796.53	9064.92	10483.3	Up
Bcap29	2819.68	0.77812	0.12919	6.02287	1.71E-09	3.60E-08	2167.2	2300.78	1763.67	3396.28	3628.5	3661.67	Up
Cog5	806.818	0.39074	0.12096	3.23032	0.00124	0.00667	751.749	663.115	679.877	852.18	939.863	954.125	Up
Hbp1	630.08	0.41649	0.13175	3.16126	0.00157	0.00814	579.533	490.628	549.058	755.766	732.882	672.613	Up
5430401H	27.5728	1.54581	0.51164	3.0213	0.00252	0.01211	11.61	17.2487	13.3684	44.5788	25.3446	53.2862	Up
Pik3cg	903.645	1.48994	0.20217	7.3696	1.71E-13	7.00E-12	588.241	477.213	358.081	1333.22	1505.89	1159.23	Up
Ccdc71l	2011.99	0.47706	0.12409	3.84444	0.00012	0.0009	1683.45	1690.37	1672.95	2205.09	2687.58	2132.46	Up
Sypl	15947.2	0.20976	0.09345	2.24452	0.0248	0.07914	16003.4	13753.9	14612.6	16914	17527.9	16871.6	Up
Efcab10	382.789	-1.064	0.20253	-5.2536	1.49E-07	2.15E-06	532.126	546.207	475.532	268.509	184.804	289.555	Down
Ahr	4808.16	0.36711	0.18555	1.97847	0.04788	0.13199	4722.37	3192.92	4683.7	5013.56	6397.4	4838.99	Up
Agr3	738.389	-0.5473	0.27918	-1.9605	0.04994	0.13669	738.204	1023.42	868.943	641.727	352.712	805.326	Down

Agr2	952.405	2.63061	0.84959	3.09633	0.00196	0.0098	140.288	564.414	89.759	1718.87	899.734	2301.36	Up
Tspan13	14631	-0.5622	0.11413	-4.9263	8.38E-07	1.05E-05	16613.9	16067.1	19658.2	11292.9	12522.3	11631.5	Down
Crppa	407.728	-0.426	0.14744	-2.8896	0.00386	0.01743	424.733	515.543	462.163	332.786	363.273	347.869	Down
Agmo	2746.78	0.51881	0.12655	4.09969	4.14E-05	0.00035	2217.51	2538.43	2018.62	3074.9	3116.33	3514.88	Up
Dgkb	198.185	-0.6042	0.20092	-3.0073	0.00264	0.01259	241.875	229.982	245.405	188.682	139.395	143.772	Down
Etv1	1172.34	-0.543	0.09621	-5.6441	1.66E-08	2.87E-07	1397.07	1413.43	1360.71	987.99	938.807	936.028	Down
Arl4a	2597.26	-0.8185	0.18263	-4.4816	7.41E-06	7.54E-05	3023.44	3888.61	3032.71	2239.31	1473.16	1926.35	Down
Scin	47.2392	-1.031	0.43207	-2.3861	0.01703	0.05881	69.6601	83.3685	37.2404	30.0648	36.9609	26.1404	Down
Lsmem1	50.5321	-1.4872	0.53853	-2.7616	0.00575	0.02435	37.7326	130.323	55.3832	32.1382	28.5127	19.1026	Down
Ifrd1	6101.07	-0.4011	0.12261	-3.2714	0.00107	0.0059	6469.68	6994.33	7366.92	5494.59	5718.38	4562.51	Down
Dock4	3819.93	-0.6296	0.10782	-5.8396	5.23E-09	1.01E-07	4529.84	4393.62	4997.86	2842.67	3262.06	2893.54	Down
Immp2l	279.864	-0.8184	0.38871	-2.1054	0.03525	0.10388	337.658	462.839	271.187	233.261	88.7061	285.534	Down
Dnajb9	4985.88	0.35855	0.10855	3.30313	0.00096	0.00537	4526.94	4370.62	4211.03	6176.75	5072.09	5557.86	Up
Stxbp6	1940.45	-0.7246	0.10901	-6.647	2.99E-11	8.65E-10	2204.94	2401.4	2646.94	1465.92	1464.71	1458.84	Down
Nova1	740.297	-1.1188	0.1923	-5.8181	5.95E-09	1.13E-07	852.369	1121.16	1067.56	416.76	579.758	404.171	Down
Scfd1	3249.59	0.25426	0.0821	3.09693	0.00196	0.00978	2990.55	3040.55	2860.83	3619.18	3500.72	3485.72	Up
Coch	118.213	-1.4717	0.38732	-3.7996	0.00014	0.00105	141.255	271.187	108.857	49.7624	72.8658	65.3511	Down
Ap4s1	1621.4	-0.3001	0.11032	-2.7204	0.00652	0.02692	1673.78	1863.81	1830.51	1387.13	1576.65	1396.5	Down
Heatr5a	3493.48	0.58866	0.12369	4.75925	1.94E-06	2.25E-05	2629.67	2670.67	3070.9	4047.34	4685.58	3856.72	Up
Nubpl	608.922	-0.7509	0.20443	-3.6733	0.00024	0.00162	687.894	988.923	614.945	483.11	418.186	460.474	Down
Akap6	517.127	-1.8027	0.40914	-4.4062	1.05E-05	0.0001	541.801	1406.72	463.118	249.849	144.675	296.593	Down
Snx6	5623.25	0.26085	0.10144	2.57153	0.01013	0.03863	5140.34	4801.83	5406.55	5686.39	6526.24	6178.19	Up
Cfl2	5927.72	-0.3652	0.09888	-3.693	0.00022	0.00152	6361.32	7335.47	6325.14	5246.82	5101.66	5195.91	Down
Baz1a	2362.61	0.87424	0.12838	6.80968	9.78E-12	3.06E-10	1861.47	1563.88	1578.42	3380.73	2790.02	3001.12	Up
2700097C	788.022	-0.4348	0.13651	-3.1852	0.00145	0.00761	887.199	861.475	969.206	640.69	612.495	757.067	Down
Ppp2r3c	1283.85	0.3351	0.10961	3.05712	0.00223	0.01093	1077.8	1209.32	1119.12	1336.33	1481.6	1478.94	Up
Prorp	662.181	0.54777	0.11088	4.94029	7.80E-07	9.89E-06	524.386	540.458	549.058	802.418	763.506	793.261	Up
Gm19990	461.278	0.52643	0.22191	2.37229	0.01768	0.06056	476.011	317.184	340.893	639.654	557.581	436.344	Up
Mbip	5336.66	0.71826	0.07835	9.16699	4.86E-20	4.23E-18	4032.55	3970.07	4102.18	6662.97	6837.76	6414.46	Up
Sfta3-ps	19865.8	0.3795	0.10538	3.60114	0.00032	0.00208	16789.1	16285.6	18729.1	21130.3	24337.2	21923.8	Up
Nkx2-1	14498.2	0.53215	0.1128	4.71742	2.39E-06	2.71E-05	12349.2	10659.7	12553.8	18665	16706.3	16055.2	Up
Gm48835	169.191	0.60681	0.30803	1.96999	0.04884	0.13406	128.678	137.031	136.548	189.719	291.463	131.708	Up
Mia2	7009.85	0.63211	0.10445	6.0516	1.43E-09	3.09E-08	5919.17	5585.69	4990.22	8529.06	8104.99	8929.97	Up
Fbxo33	2033.15	0.37191	0.14751	2.52137	0.01169	0.04346	1905.98	1643.41	1768.44	2319.13	1917.74	2644.2	Up
Fkbp3	1510.53	-0.276	0.13764	-2.0052	0.04494	0.12553	1616.69	1753.61	1593.7	1380.91	1166.91	1551.33	Down
Rpl17-ps3	569.396	-0.5459	0.12549	-4.3499	1.36E-05	0.00013	631.778	716.777	678.922	482.073	460.427	446.398	Down
Rps29	1657.16	-0.5285	0.12756	-4.1436	3.42E-05	0.00029	2165.27	1855.19	1851.52	1466.95	1385.51	1218.55	Down
Pole2	164.041	0.54848	0.2241	2.44744	0.01439	0.05135	155.768	124.574	119.36	165.875	218.597	200.075	Up
Nemf	4076.06	0.25192	0.0795	3.16895	0.00153	0.00796	3756.81	3660.55	3746.01	4550.15	4275.85	4467	Up
Gm9887	98.5308	0.70435	0.26997	2.60901	0.00908	0.03525	81.2701	81.452	62.0674	127.516	138.339	100.54	Up
Arf6	3632.94	0.60088	0.09526	6.30744	2.84E-10	6.96E-09	3010.86	2838.36	2812.13	4666.26	4352.94	4117.12	Up
5830428M	1784.8	0.62564	0.14699	4.25624	2.08E-05	0.00019	1132.94	1538.96	1539.27	2109.72	2209.21	2178.7	Up
Vcpkmt	813.439	0.97279	0.15722	6.18743	6.12E-10	1.39E-08	546.638	497.336	603.486	1209.85	911.35	1111.97	Up
Dmac2l	963.106	-0.3907	0.14545	-2.6859	0.00723	0.02929	999.429	1204.53	1074.24	937.191	730.77	832.472	Down
Cdkl1	262.627	-0.8881	0.24107	-3.684	0.00023	0.00157	304.763	359.347	359.036	219.784	126.723	206.107	Down
Sav1	2002.04	0.37937	0.1372	2.76503	0.00569	0.02415	1990.15	1527.46	1703.51	2123.19	2198.64	2469.26	Up
Pygl	4996.22	1.09244	0.2075	5.26474	1.40E-07	2.03E-06	3136.64	3658.63	2774.89	5283.1	9035.35	6088.71	Up
Trim9	94.254	-1.2644	0.5287	-2.3915	0.01678	0.05812	122.873	172.487	104.082	39.3952	21.1205	105.567	Down
Tmx1	5640.24	0.22096	0.08476	2.60682	0.00914	0.03543	5403.5	4910.12	5313.92	5947.64	6090.1	6176.18	Up
Gm32219	140.742	1.61746	0.29668	5.4518	4.99E-08	7.91E-07	88.0426	69.9529	49.6539	215.637	165.796	255.372	Up
Gm24474	59.8959	-2.0137	0.54585	-3.6891	0.00023	0.00154	75.4651	163.862	48.699	13.4773	32.7368	25.135	Down
Gm40437	146.864	-0.3932	0.19655	-2.0003	0.04547	0.12654	167.378	164.82	168.059	137.883	120.387	122.659	Down
Frmd6	4258.79	-0.4497	0.09455	-4.7559	1.98E-06	2.28E-05	5024.24	4548.85	5178.33	3561.12	3718.27	3521.92	Down
3110056K	990.472	-0.5432	0.18232	-2.9795	0.00289	0.01361	1286.78	939.093	1298.64	721.554	747.666	949.098	Down
Timm9	866.188	-0.3905	0.14097	-2.7705	0.0056	0.02382	853.336	1004.26	1090.48	697.71	797.299	754.051	Down
4930404H	15.3907	-2.201	0.95997	-2.2928	0.02186	0.07152	20.3175	43.1216	12.4135	0	8.4482	8.04321	Down
L3hypdh	349.062	-0.6738	0.2557	-2.6352	0.00841	0.03311	460.531	466.672	359.991	243.628	367.497	196.053	Down
Lrrc9	312.721	-0.6075	0.23178	-2.6209	0.00877	0.03427	389.903	328.683	414.419	260.216	178.468	304.636	Down
Gm4756	113.881	-0.5421	0.23225	-2.3341	0.01959	0.06572	131.58	143.739	129.864	89.1576	82.37	106.572	Down
Dhrs7	4099.53	0.23537	0.09788	2.4048	0.01618	0.05645	3961.92	3831.12	3504.42	4571.92	4549.36	4178.45	Up
Six1	821.167	-0.8913	0.30344	-2.9374	0.00331	0.0153	956.859	1250.53	994.033	579.524	312.584	833.477	Down
Six4	966.942	-1.0563	0.22993	-4.5941	4.35E-06	4.66E-05	1394.17	1381.81	1142.04	691.489	413.962	778.18	Down
Mnat1	1654.9	-0.4451	0.10322	-4.3124	1.61E-05	0.00015	2025.95	1795.78	1903.08	1409.93	1334.82	1459.84	Down
Trmt5	709.121	-0.4384	0.14375	-3.0499	0.00229	0.01116	748.846	922.803	776.32	622.03	636.783	547.943	Down
D830013C	45.26	-1.9596	0.65858	-2.9755	0.00292	0.01377	55.1476	137.989	22.9172	21.771	11.6163	22.1188	Down
Gm33929	2.92537	4.00637	1.74029	2.30213	0.02133	0.07012	0	0	0.95488	5.18358	7.39218	4.0216	Up
Tmem30b	2479	0.6693	0.1358	4.92853	8.28E-07	1.04E-05	1873.08	2162.79	1706.38	3349.63	2748.83	3033.29	Up
Syne2	7721.19	-0.2454	0.1051	-2.3348	0.01955	0.06564	9040.33	7545.33	8543.34	7139.86	6748	7310.27	Down
Akap5	11994.3	-0.37	0.16727	-2.2119	0.02697	0.08456	13295.4	12645.2	14630.7	10613.9	12641.7	8138.72	Down
Hspa2	2119.28	-0.5423	0.22571	-2.4025	0.01628	0.05671	2363.61	2428.23	2747.2	2034.04	1105.66	2036.94	Down
Sptb	374.178	-1.9799	0.32052	-6.1772	6.52E-10	1.48E-08	455.693	933.344	402.006	171.058	135.171	147.794	Down
Fntb	1055.58	-0.4078	0.13442	-3.0336	0.00242	0.0117	1164.87	1316.65	1129.63	999.394	915.574	807.337	Down
Fut8	3142.3	0.60654	0.10685	5.67668	1.37E-08	2.42E-07	2617.09	2587.3	2269.76	3703.15	3633.78	4042.72	Up
Atp6v1d	5239.15	0.21045	0.09057	2.32361	0.02015	0.06712	4796.87	5157.35	4618.77	5526.73	5857.77	5477.42	Up
Eif2s1	3183.96	0.20923	0.0808	2.58956	0.00961	0.03696	2963.46	2869.98	3026.98	3348.59	3475.38	3419.37	Up
9230116L(	37.3373	1.53192	0.46614	3.28641	0.00101	0.00565	14.5125	16.2904	26.7367	75.6803	48.5772	42.2268	Up
Arg2	565.987	0.71969	0.18901	3.80775	0.00014	0.00102	524.386	406.302	352.352	631.36	787.795	693.727	Up
Rdh11	1909.08	0.62818	0.09896	6.34793	2.18E-10	5.45E-09	1599.28	1465.18	1435.19	2335.72	2397.18	2221.94	Up
Zfyve26	1676.68	0.6095	0.12198	4.99656	5.84E-07	7.61E-06	1436.74	1220.82	1325.38	1933.48	2231.38	1912.27	Up
2310015A	846.472	-0.2589	0.10883	-2.3794	0.01734	0.0596	868.816	958.259	939.605	749.546	780.403	782.202	Down
Exd2	1818.72	-0.2317	0.10212	-2.2692	0.02325	0.07525	2131.41	1844.65	1917.4	1668.08	1672.74	1678.01	Down
Galnt16	166.169	-0.8976	0.31623	-2.8384	0.00453	0.01999	160.605	321.017	167.105	114.039	125.667	108.583	Down
Slc39a9	5404.58	0.20983	0.08652	2.42507	0.01531	0.05393	5161.62	4866.04	5009.32	5829.45	5493.44	6067.59	Up

Plekhd1	2992.8	1.00729	0.15986	6.30089	2.96E-10	7.21E-09	2032.72	1736.36	2196.23	3778.83	4772.18	3440.48	Up
Susd6	11448.6	0.74073	0.09743	7.60299	2.89E-14	1.33E-12	8842.96	8642.53	8232.05	13340.5	14077.9	15555.6	Up
Smoc1	354.42	-1.6847	0.66361	-2.5387	0.01113	0.04171	350.236	1046.42	225.352	170.021	140.451	194.042	Down
Slc8a3	41.4084	-1.2207	0.4121	-2.9621	0.00306	0.01431	58.0501	52.7042	63.0223	18.6609	35.9049	20.108	Down
Ttc9	4499.33	0.93271	0.10892	8.56363	1.09E-17	8.00E-16	2987.64	3358.7	2934.36	5460.38	5915.85	6339.05	Up
Gm49654	236.041	1.09446	0.24462	4.47405	7.68E-06	7.78E-05	198.338	117.866	135.593	293.391	303.079	367.977	Up
Map3k9	428.343	0.95343	0.20845	4.57397	4.79E-06	5.08E-05	350.236	212.733	312.247	575.377	596.654	522.808	Up
4930423D	175.804	0.83225	0.22304	3.73143	0.00019	0.00134	140.288	101.575	137.503	198.013	249.222	228.226	Up
Pcnx	5709.95	-0.2649	0.0834	-3.1762	0.00149	0.00781	6316.82	5978.58	6402.49	5309.02	5279.07	4973.72	Down
Rgs6	613.426	-0.8007	0.18627	-4.2985	1.72E-05	0.00016	683.056	923.761	731.44	366.997	483.66	491.641	Down
Dpf3	100.115	-0.8118	0.32179	-2.5227	0.01165	0.04333	82.2376	160.987	139.413	76.717	74.9778	66.3565	Down
Psen1	3813.43	0.23896	0.08935	2.67438	0.00749	0.03012	3717.14	3318.45	3459.54	4066	4105.83	4213.64	Up
Numb	8764.02	-0.3622	0.11649	-3.1092	0.00188	0.00945	9029.69	9257.74	11287.7	7556.62	7789.24	7663.17	Down
Riox1	1217.29	-0.4054	0.13353	-3.036	0.0024	0.01162	1268.39	1591.67	1301.51	987.99	1082.43	1071.76	Down
Acot1	2075.89	-0.7036	0.16629	-4.2312	2.32E-05	0.00021	2160.43	2650.54	2905.71	1825.66	1579.81	1333.16	Down
Pnma1	148.512	-0.6619	0.2332	-2.8385	0.00453	0.01999	155.768	206.026	184.292	104.708	104.547	135.729	Down
Elmsan1	3093.72	-0.2594	0.1265	-2.0505	0.04032	0.11526	3106.65	3123.92	3882.55	2756.63	2977.99	2714.58	Down
Zfp410	2171.28	-0.2062	0.10449	-1.9737	0.04841	0.13314	2169.14	2356.36	2453.09	2152.22	1928.3	1968.57	Down
Coq6	924.765	-0.3018	0.1472	-2.0499	0.04038	0.11537	867.849	1136.49	1058.97	900.906	794.131	790.245	Down
Entpd5	4510.57	-0.9314	0.19516	-4.7724	1.82E-06	2.12E-05	7345.27	5625.94	4783.01	3209.67	2491.16	3608.38	Down
Bbof1	654.652	-0.7828	0.24387	-3.2099	0.00133	0.00709	913.321	861.475	709.478	570.194	309.415	564.03	Down
Aldh6a1	166.031	-0.7143	0.25497	-2.8016	0.00509	0.02203	260.258	200.276	158.511	127.516	110.883	138.745	Down
Lin52	1415.12	-0.4023	0.10563	-3.8086	0.00014	0.00102	1644.75	1495.84	1693.01	1193.26	1188.03	1275.85	Down
Abcd4	1467.71	0.74853	0.17651	4.24079	2.23E-05	0.0002	1249.04	1056	980.665	1801.81	2218.71	1500.06	Up
Npc2	100601	1.54402	0.14675	10.5216	6.87E-26	9.94E-24	62454.2	47696.4	43985.7	146937	157371	145163	Up
Ltbp2	4398.48	0.2829	0.10167	2.78257	0.00539	0.02306	3733.59	3861.78	4310.34	5093.38	4746.83	4644.95	Up
Arel1	1513.65	0.31483	0.12899	2.44071	0.01466	0.05208	1480.28	1262.03	1305.32	1527.08	1643.18	1864.01	Up
Acyp1	405.973	-0.2931	0.13015	-2.2517	0.02434	0.07794	438.278	451.34	451.66	364.924	350.6	379.036	Down
Zc2hc1c	858.497	-0.3537	0.15784	-2.2411	0.02502	0.07975	913.321	1062.71	913.823	809.675	621.999	829.456	Down
Tmed10	14553	0.34002	0.07653	4.44315	8.87E-06	8.82E-05	13043.9	12839.7	12654.1	16678.7	16551.1	15550.5	Up
Jdp2	1996.9	-0.381	0.15899	-2.3967	0.01654	0.05742	2232.03	1890.64	2654.57	1952.14	1605.16	1646.85	Down
Batf	395.482	0.88358	0.14579	6.06051	1.36E-09	2.94E-08	254.453	278.853	300.788	504.881	489.996	543.922	Up
Flvcr2	966.975	0.84528	0.19778	4.27385	1.92E-05	0.00017	811.734	661.198	601.576	1285.53	1482.66	959.152	Up
Erg28	1926.14	0.21985	0.10159	2.16414	0.03045	0.0928	1639.91	1819.73	1879.21	2113.86	2099.38	2004.77	Up
Ift43	3507.44	-0.4427	0.11975	-3.6964	0.00022	0.0015	3685.21	4255.63	4183.34	3176.5	2644.29	3099.65	Down
Gm805	12.6952	-1.4357	0.71016	-2.0216	0.04321	0.12175	16.4475	12.4574	26.7367	7.25701	4.2241	9.04861	Down
Esrrb	32.6711	-2.5286	0.71474	-3.5378	0.0004	0.00257	43.5376	96.7841	26.7367	18.6609	5.28013	5.027	Down
Vash1	122.373	0.6294	0.25734	2.44574	0.01446	0.05152	119.97	82.4102	85.9395	168.985	135.171	141.762	Up
Ism2	27.4746	-2.3688	0.52132	-4.5438	5.52E-06	5.78E-05	39.6676	43.1216	55.3832	9.33044	5.28013	12.0648	Down
Sptlc2	13381.2	0.81039	0.10325	7.84866	4.21E-15	2.15E-13	10345.5	9068	9742.67	16292	18517.4	16321.7	Up
Oog1	34.3756	-2.8764	0.52432	-5.4859	4.11E-08	6.61E-07	68.6926	58.4538	54.4283	3.11015	9.50423	12.0648	Down
Gm3742	4.00981	-5.3998	1.78452	-3.0259	0.00248	0.01195	13.545	2.87478	7.63906	0	0	0	Down
Gm21038	3.05454	-3.9602	1.70559	-2.3219	0.02024	0.06736	4.83751	5.74955	6.68418	0	1.05603	0	Down
Gm2042	10.7598	-4.9294	1.30271	-3.784	0.00015	0.00111	31.9275	18.2069	12.4135	0	0	2.0108	Down
Gm2099	19.9997	-1.9422	0.60977	-3.1851	0.00145	0.00761	44.5051	31.6225	19.0977	6.2203	9.50423	9.04861	Down
Gm10436	26.2046	-2.9795	0.72741	-4.0961	4.20E-05	0.00035	64.8226	45.9964	28.6465	2.07343	12.6723	3.0162	Down
Gm47684	3.20794	-4.0355	1.7844	-2.2615	0.02373	0.07641	1.935	6.70781	9.54883	0	1.05603	0	Down
Dio2	93.0178	-2.0835	0.66265	-3.1442	0.00167	0.00855	46.4401	338.265	66.8418	29.028	44.3531	33.1782	Down
Tshr	64.2642	-2.6321	0.49198	-5.35	8.80E-08	1.31E-06	78.3676	184.944	68.7516	17.6242	14.7844	21.1134	Down
Sel1l	9089.2	0.38386	0.07074	5.42651	5.75E-08	9.00E-07	7761.3	7926.72	7973.27	10154.6	10318.4	10400.9	Up
Galc	2362.6	0.7646	0.13251	5.7701	7.92E-09	1.47E-07	1929.2	1704.74	1618.53	2589.72	3112.11	3221.3	Up
Gm47567	2.58379	4.87057	1.91275	2.54637	0.01088	0.041	0	0	0	7.25701	4.2241	4.0216	Up
Zc3h14	4220.45	0.28106	0.09624	2.92029	0.0035	0.01606	4010.29	3681.63	3740.28	4584.36	4365.61	4940.54	Up
Eml5	591.707	-0.4803	0.1877	-2.5591	0.01049	0.0398	617.266	650.658	800.192	498.66	402.346	581.122	Down
Efcab11	183.722	-0.6788	0.25741	-2.6372	0.00836	0.03298	202.208	254.897	221.533	157.581	98.2104	167.902	Down
Tdp1	731.275	-0.3402	0.14162	-2.4026	0.01628	0.05671	803.026	789.605	858.44	655.204	723.377	557.997	Down
Kcnk13	340.873	0.91691	0.27815	3.29645	0.00098	0.00548	272.835	218.483	216.758	386.695	636.783	313.685	Up
Calm1	59505.7	0.21607	0.08376	2.5796	0.00989	0.03787	54613.5	52443.6	58117	66092.7	63794.5	61972.9	Up
Ttc7b	1407.73	0.24743	0.11896	2.07994	0.03753	0.10902	1281.94	1271.61	1308.19	1431.7	1720.27	1432.7	Up
Dglucy	1042.96	0.49923	0.15614	3.19741	0.00139	0.00734	949.119	944.843	698.974	1302.12	1154.24	1208.49	Up
Ppp4r3a	1127.74	0.37864	0.17403	2.17564	0.02958	0.09087	1016.84	828.894	1096.21	1160.09	1134.17	1530.22	Up
Gm18795	12.551	-3.2408	1.23337	-2.6276	0.0086	0.03373	9.67501	53.6625	4.77441	5.18358	0	2.0108	Down
Catsperb	263.816	1.77207	0.22872	7.74775	9.35E-15	4.58E-13	147.06	114.033	97.3981	394.989	481.548	347.869	Up
Tc2n	2986.07	2.05653	0.14574	14.1109	3.26E-45	2.13E-42	1352.57	1057.92	1061.83	4465.14	5418.47	4560.5	Up
Fbln5	8197.58	-0.2098	0.10687	-1.9629	0.04966	0.13608	8246.01	9822.15	8309.39	7337.87	7675.19	7794.87	Down
Cpsf2	2221.57	0.32527	0.08903	3.65327	0.00026	0.00174	2047.23	1924.18	1945.1	2419.69	2567.2	2426.03	Up
Slc24a4	1362.61	1.10901	0.15077	7.35587	1.90E-13	7.68E-12	947.184	898.847	743.854	1855.72	1635.78	2094.25	Up
Lgmn	8144.33	0.94079	0.13259	7.09533	1.29E-12	4.58E-11	6510.32	5213.89	5013.14	11199.6	11043.9	9885.1	Up
Chga	14.067	-2.6753	0.78595	-3.4039	0.00066	0.00395	22.2525	18.2069	32.466	2.07343	7.39218	2.0108	Down
Unc79	446.714	-2.1006	0.3839	-5.4717	4.46E-08	7.14E-07	406.351	1259.15	507.998	134.773	189.029	182.983	Down
Asb2	671.084	-2.13	0.61836	-3.4447	0.00057	0.00347	508.906	2067.92	700.884	220.82	254.502	273.469	Down
Otub2	669.248	-0.5346	0.13492	-3.962	7.43E-05	0.00059	861.076	774.273	740.034	582.634	561.805	495.663	Down
Ifi27	7393.14	0.24602	0.10616	2.31751	0.02048	0.06801	6639.96	7318.22	6334.69	8684.57	7608.66	7772.75	Up
Ppp4r4	1220.05	-0.7773	0.14479	-5.3686	7.93E-08	1.20E-06	1300.32	1538.01	1784.68	897.796	898.678	900.839	Down
Serpina10	97.598	1.14593	0.45039	2.54434	0.01095	0.04118	53.2126	53.6625	75.4357	146.177	57.0254	200.075	Up
Serpina16	5.48897	-2.7897	1.30182	-2.1429	0.03212	0.09656	6.77251	3.83303	18.1428	2.07343	2.11205	0	Down
Serpina11	8.09968	3.54364	1.25632	2.82066	0.00479	0.02093	1.935	0	1.90977	10.3672	4.2241	30.162	Up
Gm28577	54.0714	4.15424	0.65592	6.33349	2.40E-10	5.96E-09	1.935	7.66607	7.63906	122.332	39.0729	145.783	Up
Serpina9	1272.3	2.12141	0.84198	2.51956	0.01175	0.04364	119.003	902.68	404.87	2547.21	1034.9	2625.1	Up
Serpina12	26.6925	1.71619	0.64089	2.67781	0.00741	0.02988	7.74001	11.4991	18.1428	43.5421	16.8964	62.3349	Up
Serpina3c	683.894	-0.8223	0.31176	-2.6375	0.00835	0.03296	450.856	1271.61	898.545	493.477	494.22	494.657	Down
Serpina3f	491.826	0.90474	0.37427	2.41738	0.01563	0.05486	490.523	344.015	192.886	365.961	906.07	651.5	Up
Serpina3g	542.459	0.69734	0.24254	2.8752	0.00404	0.01812	478.913	471.463	291.239	515.248	730.77	767.121	Up

Serpina3i	108.613	1.21288	0.41179	2.94539	0.00323	0.01499	28.0575	76.6607	91.6688	134.773	142.563	177.956	Up
Serpina3n	8286.8	0.92308	0.1311	7.04096	1.91E-12	6.57E-11	6276.18	5733.26	5158.28	11576	11585.7	9391.45	Up
Clmn	2051.01	1.1396	0.12343	9.23287	2.63E-20	2.37E-18	1249.04	1448.89	1143.95	2759.74	2743.55	2960.91	Up
Gm28875	372.493	-0.8289	0.18861	-4.3948	1.11E-05	0.00011	389.903	563.456	476.487	264.363	290.407	250.345	Down
Atg2b	1808.75	0.23894	0.08991	2.6575	0.00787	0.03138	1691.19	1666.41	1620.44	1892.01	1951.53	2030.91	Up
Setd3	8397.94	0.21447	0.08267	2.59427	0.00948	0.03654	7779.68	7636.36	7908.34	8871.18	9529.57	8662.53	Up
Ccdc85c	919.469	-0.33	0.11279	-2.9253	0.00344	0.01584	985.884	1082.83	1003.58	800.345	866.997	777.175	Down
Cyp46a1	85.8076	-0.6463	0.26959	-2.3972	0.01652	0.05737	110.295	103.492	100.263	74.6435	72.8658	53.2862	Down
Evl	382.041	1.25526	0.23989	5.23262	1.67E-07	2.39E-06	291.218	199.318	186.202	502.807	665.296	447.403	Up
Degs2	107.742	0.79813	0.358	2.22942	0.02579	0.0816	59.9851	108.283	67.7967	164.838	88.7061	156.843	Up
Gm34220	92.8478	-0.8478	0.24618	-3.4437	0.00057	0.00348	122.873	114.991	120.315	62.203	62.3055	74.3997	Down
Slc25a47	416.689	-0.7592	0.21011	-3.6133	0.0003	0.002	449.888	505.961	615.899	331.749	239.718	356.917	Down
Wdr25	345.389	-0.5094	0.1731	-2.9431	0.00325	0.01506	388.936	405.343	423.013	331.749	240.774	282.518	Down
Meg3	1280.85	1.26134	0.52614	2.39734	0.01651	0.05736	724.659	850.934	686.561	1221.25	3190.25	1011.43	Up
Rian	838.814	0.86921	0.33935	2.56138	0.01043	0.03958	520.516	649.699	610.17	757.839	1830.09	664.57	Up
Dio3os	30.6562	-2.1349	0.45743	-4.6672	3.05E-06	3.39E-05	52.2451	43.1216	54.4283	13.4773	11.6163	9.04861	Down
B930059L	26.0669	-0.9767	0.44977	-2.1716	0.02988	0.0916	34.8301	30.6643	38.1953	20.7343	16.8964	15.081	Down
Gm35558.	72.2531	-0.8753	0.28488	-3.0725	0.00212	0.01047	107.393	89.1181	84.0297	52.8725	53.8573	46.2484	Down
1700001K	151.28	-0.4202	0.19546	-2.1495	0.03159	0.09538	174.15	165.779	179.518	139.957	124.611	123.664	Down
Hsp90aa1	31981.6	-0.3834	0.19445	-1.9719	0.04862	0.13354	30938.8	32275.1	45406.6	26354.4	22989.7	33925.2	Down
Cinp	1371.84	-0.2524	0.10181	-2.479	0.01318	0.04786	1443.51	1427.81	1603.25	1257.54	1248.22	1250.72	Down
Ankrd9	380.17	-1.1725	0.17469	-6.712	1.92E-11	5.76E-10	545.671	565.373	468.847	262.289	240.774	198.064	Down
Gm48630	3.6799	-5.2762	1.90768	-2.7658	0.00568	0.02411	4.83751	15.3321	1.90977	0	0	0	Down
Traf3	2034.31	0.4634	0.11245	4.12105	3.77E-05	0.00032	1729.89	1669.29	1732.16	2181.25	2265.17	2628.12	Up
Cdc42bpb	5160.38	0.35337	0.11492	3.07487	0.00211	0.0104	4624.66	3978.69	4991.17	5758.96	5986.61	5622.2	Up
Lbhd2	7.65892	4.52125	1.40463	3.21881	0.00129	0.0069	0	1.91652	0	16.5875	6.33615	21.1134	Up
Tnfaip2	583.333	2.11144	0.27285	7.73858	1.01E-14	4.87E-13	308.633	174.403	174.744	761.986	1217.6	862.634	Up
Bag5	2764.79	-0.2672	0.13468	-1.9837	0.04728	0.13064	3043.76	2584.42	3431.85	2525.44	2650.62	2352.64	Down
Apopt1	1377.04	-0.2453	0.09811	-2.5001	0.01241	0.04563	1488.02	1544.71	1448.56	1269.98	1310.53	1200.45	Down
Zfyve21	3252.67	-0.3355	0.16078	-2.0863	0.03695	0.10778	4145.74	3267.66	3473.86	3393.17	2819.59	2415.98	Down
Atp5mpl	1209.85	-0.4487	0.12341	-3.6361	0.00028	0.00185	1231.63	1516.92	1440.92	1058.49	992.664	1018.47	Down
Aspg	272.404	-2.2545	0.38076	-5.9212	3.20E-09	6.44E-08	363.781	595.079	392.457	147.214	45.4091	90.4861	Down
A530016L	58.3539	-9.2633	1.3811	-6.7072	1.98E-11	5.94E-10	54.1801	251.064	44.8795	0	0	0	Down
Tmem179	28.9791	-5.358	1.25864	-4.257	2.07E-05	0.00019	17.415	137.989	14.3232	4.14686	0	0	Down
Adssl1	1610.83	-0.352	0.16748	-2.1019	0.03556	0.10453	1767.63	2204.95	1446.65	1364.32	1404.51	1476.93	Down
Siva1	1103.13	-0.5365	0.10967	-4.8918	9.99E-07	1.23E-05	1222.92	1335.81	1358.8	938.228	913.462	849.564	Down
Akt1	11083	0.22182	0.10147	2.18603	0.02881	0.08918	10593.2	9581.63	10522.8	11907.7	12860.3	11032.3	Up
Zbtb42	1425.32	0.39772	0.11406	3.48689	0.00049	0.00304	1209.38	1135.54	1345.43	1584.1	1570.31	1707.17	Up
Jag2	3014.31	-0.5738	0.17005	-3.374	0.00074	0.00433	2822.2	3634.68	4360.95	2501.6	2219.77	2546.68	Down
Gm26583	63.1794	-1.8808	0.4923	-3.8204	0.00013	0.00098	67.7251	107.325	123.18	13.4773	21.1205	46.2484	Down
Crip2	32640.3	-0.4529	0.12321	-3.6759	0.00024	0.00161	35269.3	36845	41051.4	29800.4	28940.4	23935.6	Down
Igha	8713.37	2.50232	0.54896	4.55829	5.16E-06	5.44E-05	1250.01	4164.59	2428.27	9014.24	16648.2	18774.9	Up
Ighg2b	548.275	1.83272	0.61927	2.95948	0.00308	0.01441	86.1076	257.772	377.179	1134.17	812.084	622.343	Up
Ighg1	317.537	3.79057	0.95535	3.96771	7.26E-05	0.00057	16.4475	63.2451	48.699	1308.34	230.214	238.28	Up
Ighg3	97.7729	1.45025	0.64997	2.23126	0.02566	0.08126	112.23	21.0817	23.8721	228.077	132.003	69.3727	Up
Ighm	16146.3	1.14248	0.52502	2.17606	0.02955	0.09081	16912.9	7475.38	5814.28	25800.7	16964	23910.4	Up
Ighv2-2	75.7062	4.29075	0.86201	4.97761	6.44E-07	8.32E-06	7.74001	2.87478	11.4586	183.499	237.606	11.0594	Up
Ighv5-4	19.8096	2.46115	0.92746	2.65364	0.00796	0.03168	9.67501	2.87478	5.7293	4.14686	40.129	56.3024	Up
Ighv5-6	13.0981	2.53776	0.97989	2.58983	0.0096	0.03694	4.83751	4.79129	1.90977	12.4406	48.5772	6.03241	Up
Ighv5-9	13.5224	3.70638	1.10756	3.34643	0.00082	0.0047	2.9025	1.91652	0.95488	27.9913	44.3531	3.0162	Up
Ighv5-9-1	23.5164	1.96685	0.87584	2.24567	0.02472	0.07895	3.87001	6.70781	18.1428	8.29373	73.9218	30.162	Up
Ighv2-6-8	12.1245	4.60494	1.64472	2.79983	0.00511	0.02212	0	0.95826	1.90977	0	10.5603	59.3187	Up
Ighv5-16	33.0551	4.45311	1.13236	3.93258	8.40E-05	0.00065	5.80501	0	2.86465	4.14686	52.8013	132.713	Up
Ighv4-1	74.9754	4.72725	1.45566	3.24749	0.00116	0.00633	10.6425	0	5.7293	29.028	89.7622	314.69	Up
Ighv3-1	8.38416	4.6594	1.74015	2.67758	0.00742	0.02989	0	0.95826	0.95488	1.03672	2.11205	45.243	Up
Ighv11-2	34.5939	1.73456	0.72658	2.3873	0.01697	0.05867	14.5125	7.66607	25.7818	99.5247	15.8404	44.2376	Up
Ighv14-3	17.3274	2.87405	0.94327	3.04691	0.00231	0.01124	2.9025	8.62433	0.95488	52.8725	8.4482	30.162	Up
Ighv9-1	26.0307	3.10047	1.13267	2.73731	0.00619	0.02585	3.87001	3.83303	8.59395	3.11015	126.723	10.054	Up
Ighv9-3	46.6578	1.97682	0.8057	2.45355	0.01415	0.05071	25.155	14.3739	17.1879	177.278	14.7844	31.1674	Up
Ighv14-4	31.8701	4.0947	0.70893	5.77592	7.65E-09	1.42E-07	4.83751	2.87478	2.86465	93.3044	60.1934	27.1458	Up
Ighv3-5	7.4254	3.04421	1.22674	2.48155	0.01308	0.04756	2.9025	1.91652	0	10.3672	25.3446	4.0216	Up
Ighv3-6	66.0478	2.21752	0.47184	4.69971	2.61E-06	2.93E-05	28.0575	26.8312	15.2781	126.479	53.8573	145.783	Up
Ighv9-4	8.60027	3.28342	1.32276	2.48225	0.01306	0.04751	2.9025	0.95826	0.95488	1.03672	10.5603	35.189	Up
Ighv12-3	16.2219	1.60887	0.59848	2.68828	0.00718	0.02911	11.61	5.74955	6.68418	22.8077	25.3446	25.135	Up
Ighv6-3	104.818	3.90179	0.62369	6.25598	3.95E-10	9.35E-09	21.285	5.74955	12.4135	293.391	240.774	55.297	Up
Ighv6-6	198.557	3.83937	0.54862	6.99821	2.59E-12	8.70E-11	24.1875	39.2886	14.3232	678.012	129.891	305.642	Up
Ighv1-7	10.0098	2.39498	0.93251	2.56832	0.01022	0.03892	1.935	5.74955	1.90977	27.9913	7.39218	15.081	Up
Ighv1-9	51.9533	3.51753	0.65427	5.37627	7.60E-08	1.15E-06	15.48	5.74955	3.81953	89.1576	156.292	41.2214	Up
Ighv1-12	40.5041	4.20928	0.64832	6.49256	8.44E-11	2.25E-09	0	7.66607	4.77441	57.0194	97.1543	76.4105	Up
Ighv1-36	3.16486	5.16207	2.57764	2.00264	0.04522	0.12605	0	0	0	1.03672	17.9524	0	Up
Ighv1-47	10.0976	2.24071	1.05211	2.12973	0.03319	0.09905	4.83751	2.87478	2.86465	31.1015	16.8964	2.0108	Up
Ighv1-50	13.9239	2.26804	0.93711	2.42024	0.01551	0.05448	1.935	5.74955	6.68418	8.29373	11.6163	49.2646	Up
Ighv1-52	116.864	3.99495	0.67659	5.90453	3.54E-09	7.08E-08	23.22	14.3739	3.81953	99.5247	109.827	450.42	Up
Ighv1-55	113.467	2.81794	0.43926	6.41516	1.41E-10	3.65E-09	36.7651	12.4574	35.3307	257.106	216.485	122.659	Up
Ighv1-56	9.81116	-3.3704	1.46018	-2.3082	0.02099	0.06925	1.935	46.9547	4.77441	4.14686	1.05603	0	Down
Ighv1-58	14.1012	4.05855	1.47087	2.75928	0.00579	0.02451	0	2.87478	1.90977	74.6435	3.16808	2.0108	Up
Ighv1-61	4.09473	2.42931	1.2178	1.99484	0.04606	0.12796	1.935	0.95826	0.95488	5.18358	9.50423	6.03241	Up
Ighv1-64	36.6291	0.98183	0.42885	2.28944	0.02205	0.0721	17.415	22.9982	33.4209	52.8725	53.8573	39.2106	Up
Ighv1-69	230.598	4.76585	1.26337	3.77234	0.00016	0.00116	23.22	16.2904	9.54883	45.6155	125.667	1163.25	Up
Ighv8-12	36.0753	1.80747	0.62557	2.88932	0.00386	0.01744	27.09	9.58259	11.4586	24.8812	55.9693	87.4699	Up
Ighv1-74	18.5374	2.64297	0.80125	3.29856	0.00097	0.00545	3.87001	4.79129	6.68418	8.29373	44.3531	43.2322	Up
Ighv1-75	14.0455	2.05288	0.99273	2.06793	0.03865	0.1115	9.67501	5.74955	0.95488	42.5053	5.28013	20.108	Up
Ighv1-80	39.2127	2.35203	0.82172	2.86234	0.00421	0.01875	26.1225	7.66607	4.77441	101.598	12.6723	82.4429	Up

Ighv1-81	25.7695	2.37695	0.56935	4.17486	2.98E-05	0.00026	7.74001	12.4574	4.77441	31.1015	42.241	56.3024 Up
Ighv1-82	76.5456	2.56369	0.6021	4.25791	2.06E-05	0.00019	42.5701	14.3739	9.54883	82.9373	87.6501	222.194 Up
Gm9260	123.466	-0.8642	0.38914	-2.2208	0.02636	0.08308	76.4326	190.693	211.029	85.0107	77.0899	100.54 Down
Vipr2	1420.9	-0.5078	0.16467	-3.0836	0.00205	0.01015	1553.81	1674.08	1777.04	1274.12	1327.42	918.936 Down
D430020J	39.9123	0.92544	0.36696	2.52192	0.01167	0.0434	29.9925	27.7895	24.827	60.1295	46.4651	50.27 Up
Ncapg2	354.891	0.60379	0.14839	4.06906	4.72E-05	0.00039	303.795	257.772	283.6	420.907	411.85	451.425 Up
Ptprn2	209.479	-0.8472	0.35748	-2.37	0.01779	0.06088	205.11	367.013	235.856	139.957	89.7622	219.177 Down
Rapgef5	11137.2	-0.4756	0.13584	-3.5013	0.00046	0.0029	11119.5	12555.1	15195.1	9046.38	9067.03	9839.86 Down
Dnah11	1357.64	-0.5328	0.21869	-2.4363	0.01484	0.05262	1492.85	1623.29	1700.65	1132.09	758.226	1438.73 Down
Gm49602	484.624	-0.5373	0.18486	-2.9067	0.00365	0.01664	581.468	480.088	659.824	434.384	418.186	333.793 Down
Ncoa4-ps	233.313	0.89292	0.2088	4.27645	1.90E-05	0.00017	192.533	132.24	165.195	301.684	334.76	273.469 Up
Macc1	970.376	0.37472	0.18185	2.06055	0.03935	0.11305	990.722	713.903	830.748	1038.79	944.087	1304 Up
Gm17177	7.237	6.35646	1.52368	4.17179	3.02E-05	0.00026	0	0	0	22.8077	10.5603	10.054 Up
Gm48530	16.3127	-1.4839	0.61134	-2.4272	0.01521	0.05369	31.9275	23.9565	16.233	7.25701	8.4482	10.054 Down
Gm16505	52.9847	-1.8328	0.52076	-3.5194	0.00043	0.00273	119.003	62.2868	66.8418	27.9913	32.7368	9.04861 Down
Gdi2	20276.7	0.29693	0.07601	3.9063	9.37E-05	0.00072	18934	18219.4	17439	22304.9	22286.4	22476.7 Up
Akr1c13	613.73	0.51732	0.18911	2.73552	0.00623	0.02597	514.711	591.246	408.69	823.152	615.663	728.916 Up
Akr1c19	841.83	2.25807	0.31342	7.20465	5.82E-13	2.18E-11	431.506	198.36	243.495	1311.45	1882.89	983.282 Up
Akr1c12	121.125	1.54925	0.44515	3.48029	0.0005	0.00311	33.8626	100.617	50.6088	221.857	106.659	213.145 Up
Gm40660	7.63114	-2.1861	0.95544	-2.288	0.02213	0.07224	21.285	8.62433	7.63906	3.11015	2.11205	3.0162 Down
Gm47507	34.6318	1.89039	0.43123	4.38369	1.17E-05	0.00011	13.545	15.3321	15.2781	44.5788	50.6892	68.3673 Up
Gm46401	26.5908	2.3054	0.54602	4.22216	2.42E-05	0.00022	4.83751	11.4991	10.5037	34.2116	41.185	57.3078 Up
Idi1	1948.4	1.44997	0.14479	10.0141	1.32E-23	1.56E-21	1119.4	1151.83	861.304	2628.07	2934.69	2995.09 Up
Gtpbp4	2879.53	-0.2582	0.10981	-2.3512	0.01871	0.06343	3358.2	2820.16	3231.32	2638.44	2495.39	2733.68 Down
Gm36264	31.9901	-1.4432	0.47484	-3.0392	0.00237	0.01151	60.9526	38.3303	41.06	23.8445	12.6723	15.081 Down
Chrm3	325.808	-0.8883	0.33284	-2.6687	0.00761	0.03054	464.401	528.001	276.916	244.665	136.227	304.636 Down
Ryr2	1883.47	-3.765	0.63354	-5.9428	2.80E-09	5.69E-08	2467.13	6306.3	1753.16	218.747	178.468	377.025 Down
Mtr	1764.2	-0.5179	0.16087	-3.2193	0.00128	0.00689	1875.99	2543.22	1813.32	1354.99	1438.31	1559.38 Down
Actn2	2166.53	-5.3324	1.11143	-4.7978	1.60E-06	1.89E-05	2279.43	8023.5	2381.48	81.9006	12.6723	220.183 Down
Heatr1	2810.75	0.78562	0.21696	3.62095	0.00029	0.00195	1802.46	2314.19	2074.96	2974.34	2835.43	4863.12 Up
Lgals8	3717.78	0.71387	0.18847	3.78767	0.00015	0.0011	3369.81	2422.48	2656.48	4533.56	5626.5	3697.86 Up
Gm30239	2.71533	-4.8379	1.97458	-2.4501	0.01428	0.05108	1.935	9.58259	4.77441	0	0	0 Down
Nid1	4928.83	-0.3759	0.10193	-3.6875	0.00023	0.00155	5055.2	5638.39	6008.12	4174.85	4398.35	4298.09 Down
Lyst	2788.84	0.6875	0.19462	3.53259	0.00041	0.00262	2446.81	1951.97	2010.98	3301.94	4375.11	2646.22 Up
Gng4	69.4027	-1.3148	0.37939	-3.4654	0.00053	0.00325	137.385	70.9111	88.8041	40.4319	30.6247	48.2592 Down
Gli3	256.015	-0.7788	0.18911	-4.118	3.82E-05	0.00032	311.535	280.77	378.134	176.242	195.365	194.042 Down
Gm5628	75.2789	-1.2523	0.31622	-3.9603	7.49E-05	0.00059	122.873	106.367	88.8041	49.7624	50.6892	33.1782 Down
Vdac3-ps:	1002.87	-0.434	0.1537	-2.8238	0.00475	0.02078	964.599	1334.85	1158.27	914.383	823.7	821.413 Down
Vps41	4052.42	0.22142	0.0895	2.47399	0.01336	0.04842	3823.57	3866.57	3535.93	4231.87	4550.41	4306.13 Up
Amph	1183.54	-1.1515	0.18313	-6.2878	3.22E-10	7.79E-09	1229.69	1716.24	1950.83	755.766	754.002	694.732 Down
Stard3nl	4653.16	0.33277	0.09974	3.33628	0.00085	0.00485	4036.42	4094.64	4225.36	5438.61	5422.69	4701.25 Up
Epdr1	1206.47	-0.4188	0.12773	-3.2791	0.00104	0.00576	1429	1488.18	1224.16	1015.98	971.543	1109.96 Down
Sfrp4	96.3052	-1.5197	0.40708	-3.7332	0.00019	0.00133	70.6276	195.485	162.33	45.6155	46.4651	57.3078 Down
Aoah	182.234	0.95838	0.39974	2.39753	0.01651	0.05734	109.328	176.32	85.9395	187.646	153.124	381.047 Up
Gpx6	27.8254	8.29951	3.90841	2.1235	0.03371	0.1002	0	0	0	68.4232	0	98.5293 Up
Gm11274	47.0268	-2.0691	0.72996	-2.8345	0.00459	0.02018	28.0575	79.5355	120.315	26.9546	3.16808	24.1296 Down
C230035I:	179.623	-0.4545	0.21918	-2.0738	0.0381	0.1103	200.273	189.735	232.991	181.425	143.619	129.697 Down
Gm46404	104.331	-0.5481	0.23269	-2.3556	0.01849	0.06282	132.548	120.741	118.405	88.1208	89.7622	76.4105 Down
H4c8	62.3154	-1.039	0.37999	-2.7343	0.00625	0.02603	69.6601	84.3268	97.3981	47.6889	50.6892	24.1296 Down
H1f3	46.6249	0.89557	0.4185	2.13996	0.03236	0.09701	19.35	31.6225	46.7893	61.1662	66.5296	54.2916 Up
H2bc8	56.7794	1.47351	0.32315	4.55977	5.12E-06	5.40E-05	25.155	32.5808	32.466	85.0107	74.9778	90.4861 Up
H2bc6	577.537	-0.5234	0.17434	-3.0025	0.00268	0.01277	684.024	665.032	694.2	487.256	559.693	375.015 Down
H2ac6	51.985	-0.8272	0.38547	-2.1458	0.03189	0.09605	43.5376	83.3685	72.5711	32.1382	39.0729	41.2214 Down
Ripor2	1439.83	-0.4084	0.1353	-3.0182	0.00254	0.01222	1753.11	1762.24	1411.32	1259.61	1311.58	1141.13 Down
Acot13	1750.2	-0.3724	0.16841	-2.2115	0.027	0.08459	1733.76	2425.35	1765.58	1584.1	1348.54	1643.83 Down
D130043K	232.986	-0.6898	0.33707	-2.0465	0.04071	0.11607	274.77	311.434	276.916	184.535	91.8742	258.388 Down
Dcdc2a	856.379	-1.3182	0.33246	-3.9651	7.34E-05	0.00058	731.431	1617.54	1318.69	469.632	306.247	694.732 Down
Gm11353	165.72	-0.4625	0.21526	-2.1484	0.03168	0.09556	208.013	192.61	175.698	136.846	118.275	162.875 Down
Gm40841	41.6793	-1.6329	0.43115	-3.7873	0.00015	0.0011	50.3101	87.2015	51.5637	23.8445	20.0645	17.0918 Down
2610307P	220.398	-0.6267	0.1769	-3.543	0.0004	0.00253	251.55	260.646	290.284	173.132	186.916	159.859 Down
Sox4	2404.27	0.43996	0.15761	2.79154	0.00525	0.02256	1816	1836.98	2468.37	2501.6	3030.79	2771.89 Up
E2f3	551.002	0.5702	0.15961	3.57244	0.00035	0.00229	397.643	529.917	402.961	651.058	656.848	667.586 Up
Mboat1	1181.08	0.42509	0.11485	3.70118	0.00021	0.00148	936.541	1078.04	1010.27	1433.78	1358.05	1269.82 Up
Agtr1a	906.848	-1.0861	0.10839	-10.02	1.25E-23	1.49E-21	1175.51	1221.78	1301.51	588.855	571.31	582.127 Down
Uqcrfs1	5359.5	-0.4786	0.2008	-2.3836	0.01714	0.05907	5641.5	8357.93	4721.9	4702.54	4492.33	4240.78 Down
1700018A	81.9123	-0.8204	0.2953	-2.7784	0.00546	0.02333	82.2376	120.741	110.766	68.4232	57.0254	52.2808 Down
Foxf2	1577.42	-0.4652	0.13853	-3.358	0.00079	0.00455	1864.38	1687.49	1936.5	1249.24	1536.52	1190.39 Down
Foxc1	472.593	-0.9031	0.21134	-4.2734	1.92E-05	0.00017	513.743	795.355	538.554	354.557	292.519	340.831 Down
Gmds	860.594	0.71907	0.13397	5.36754	7.98E-08	1.20E-06	647.258	702.404	601.576	1054.34	1182.75	975.239 Up
Mylk4	413.946	-5.8326	1.03246	-5.6492	1.61E-08	2.80E-07	336.691	1796.73	307.472	8.29373	6.33615	28.1512 Down
Wrnip1	2354.62	-0.2038	0.0904	-2.2543	0.02418	0.07756	2580.33	2403.31	2578.18	2256.93	2198.64	2110.34 Down
Serpinb1a	2009.27	0.82701	0.22068	3.74751	0.00018	0.00127	1859.54	1305.15	1181.19	2269.37	3274.73	2165.63 Up
Serpinb6b	10183.6	0.85078	0.10561	8.0559	7.89E-16	4.45E-14	7950.93	6535.32	7308.67	12673.9	13519.2	13113.4 Up
Serpinb9	12719.4	0.94971	0.1277	7.43703	1.03E-13	4.36E-12	9993.32	7555.87	8484.13	16091.9	17688.4	16502.6 Up
Gm6093	1.73029	4.29133	2.17467	1.97333	0.04846	NA	0	0	0	4.14686	4.2241	2.0108 Up
Ripk1	2696.61	0.45852	0.09739	4.70789	2.50E-06	2.83E-05	2347.16	2140.75	2327.05	2943.24	3270.51	3150.93 Up
Slc22a23	5441.62	0.64085	0.10602	6.04488	1.50E-09	3.21E-08	4682.71	3901.07	4173.79	6341.59	6852.55	6697.98 Up
Pxdc1	11609.7	-0.6952	0.12824	-5.4211	5.92E-08	9.21E-07	15482	11970.6	15609.5	8898.13	8698.48	8999.34 Down
Fam217a	66.8253	-0.7043	0.31133	-2.2622	0.02369	0.07629	65.7901	93.9093	88.8041	48.7256	45.4091	58.3133 Down
Gm16984	8.48337	1.76933	0.83459	2.11999	0.03401	0.10094	5.80501	3.83303	1.90977	14.514	14.7844	10.054 Up
Nrn1	506.679	-1.5058	0.74736	-2.0149	0.04392	0.12329	946.216	749.358	552.877	127.516	116.163	547.943 Down
Ly86	767.427	0.67209	0.1402	4.79383	1.64E-06	1.93E-05	601.786	636.284	537.599	947.558	843.764	1037.57 Up
Gm47731	15.749	-1.7524	0.688	-2.5471	0.01086	0.04094	18.3825	20.1234	34.3758	3.11015	8.4482	10.054 Down

Gm47732	17.756	-1.4301	0.66189	-2.1606	0.03073	0.0934	25.155	12.4574	40.1051	10.3672	7.39218	11.0594	Down
Gm47754	42.7721	1.25157	0.45946	2.72398	0.00645	0.02669	27.09	34.4973	14.3232	42.5053	72.8658	65.3511	Up
Rreb1	3055.5	0.31396	0.13072	2.40183	0.01631	0.05677	2659.66	2869.98	2643.12	3401.46	3848.16	2910.64	Up
Snrnp48	2548.58	-0.3206	0.11505	-2.787	0.00532	0.02282	2612.25	2848.9	3030.8	2062.03	2308.47	2429.05	Down
Gm47990	3.53676	-4.1847	1.679	-2.4924	0.01269	0.04642	7.74001	8.62433	3.81953	1.03672	0	0	Down
Bmp6	8020.69	-0.7406	0.15326	-4.8326	1.35E-06	1.61E-05	10150.1	9178.2	10777.8	5732	7238	5048.12	Down
Txndc5	11766.1	0.22261	0.08444	2.63641	0.00838	0.03302	11179.5	10588.8	10812.1	13438.9	12334.4	12242.8	Up
Bloc1s5	1002.58	-0.2701	0.12449	-2.17	0.03001	0.0918	989.754	1130.75	1167.82	926.824	957.815	842.526	Down
Slc35b3	2542.51	0.36299	0.09667	3.75504	0.00017	0.00124	2085.93	2327.61	2259.25	2858.23	2984.33	2739.72	Up
Gcnt2	560.36	0.68637	0.18131	3.78561	0.00015	0.0011	528.256	361.264	399.141	633.433	699.089	740.98	Up
A730081D	95.76	-0.8518	0.28835	-2.9541	0.00314	0.01463	138.353	109.241	122.225	53.9092	63.3615	87.4699	Down
Tmem14c	3697.14	0.44877	0.11002	4.07898	4.52E-05	0.00037	3065.04	3313.66	3001.2	4472.39	4505	3825.55	Up
Gcm2	19.4731	1.54311	0.65692	2.34899	0.01882	0.06373	18.3825	5.74955	5.7293	24.8812	36.9609	25.135	Up
Nedd9	8051.43	0.34386	0.12526	2.74504	0.00605	0.02533	7350.11	6665.65	7273.34	8582.97	10370.2	8066.33	Up
Tmem170	2599.18	0.41252	0.08389	4.91731	8.77E-07	1.10E-05	2277.5	2269.16	2143.71	2946.35	2944.2	3014.19	Up
Adtrp	130.055	2.6726	0.46276	5.77538	7.68E-09	1.43E-07	46.4401	46.9547	12.4135	249.849	280.903	143.772	Up
Phactr1	5294.52	-0.3949	0.11286	-3.4992	0.00047	0.00293	6558.69	5376.79	6108.39	4656.93	4773.23	4293.06	Down
Gfod1	1324.85	-0.3382	0.12143	-2.785	0.00535	0.02294	1409.65	1509.26	1519.22	1047.08	1292.58	1171.29	Down
Sirt5	756.788	-0.521	0.13554	-3.8437	0.00012	0.0009	929.769	957.3	788.733	600.258	668.464	596.203	Down
Nol7	3661.18	-0.2082	0.08553	-2.4338	0.01494	0.05288	3931.93	3801.41	4041.06	3482.33	3466.93	3243.42	Down
Mcur1	3685.64	0.24115	0.11037	2.18501	0.02889	0.08937	3171.47	3692.17	3271.43	3745.65	3940.03	4293.06	Up
Cd83	1327.17	0.99023	0.1826	5.42295	5.86E-08	9.14E-07	1139.72	775.231	751.493	1633.86	1804.75	1857.98	Up
Gm40932	7.70148	3.91324	1.34183	2.91635	0.00354	0.01623	0	1.91652	0.95488	30.0648	4.2241	9.04861	Up
5033430I1	61.3384	0.76295	0.32071	2.37894	0.01736	0.05965	53.2126	47.9129	35.3307	85.0107	79.2019	67.3619	Up
Stmnd1	1815.1	-0.855	0.26195	-3.2641	0.0011	0.00603	1883.73	3004.14	2125.57	1434.81	822.644	1619.7	Down
Cap2	881.758	-0.7985	0.29898	-2.6707	0.00757	0.03039	774.001	1741.16	844.116	644.837	535.405	751.034	Down
2010001K	669.83	-0.394	0.15734	-2.5044	0.01227	0.0452	676.284	800.146	805.921	630.323	489.996	616.311	Down
Rnf144b	2467.24	0.50869	0.13506	3.76648	0.00017	0.00118	2303.62	1744.99	2061.59	2681.98	3040.3	2970.96	Up
Id4	706.614	-0.8392	0.22252	-3.7713	0.00016	0.00116	696.601	1234.24	788.733	493.477	505.836	520.798	Down
Gm48648	2.56235	-4.7539	1.89966	-2.5025	0.01233	0.04537	5.80501	5.74955	3.81953	0	0	0	Down
6720427I0	439.423	-0.7451	0.2585	-2.8826	0.00394	0.01776	595.013	352.639	703.749	332.786	290.407	361.944	Down
Ptpdc1	825.584	-0.4005	0.14927	-2.6827	0.0073	0.02953	1006.2	948.676	863.214	746.435	786.739	602.235	Down
Fam120a	13762.7	0.34571	0.07629	4.53166	5.85E-06	6.08E-05	11693.2	12225.5	12445.9	14972.3	15589	15650.1	Up
Wnk2	221.331	-2.8604	0.3599	-7.9479	1.90E-15	1.01E-13	308.633	611.369	247.315	52.8725	46.4651	61.3295	Down
Ninj1	1550.4	-0.3801	0.11891	-3.1964	0.00139	0.00736	1838.25	1785.24	1636.67	1398.53	1437.25	1206.48	Down
Susd3	308.546	1.18667	0.28801	4.12019	3.79E-05	0.00032	214.785	203.151	147.052	427.127	577.646	281.512	Up
Fgd3	412.757	0.62705	0.18573	3.37616	0.00074	0.0043	367.651	342.098	263.548	468.596	568.142	466.506	Up
Bicd2	6445.77	-0.3329	0.12355	-2.6946	0.00705	0.0287	6354.55	7108.36	8095.5	5446.91	6137.62	5531.72	Down
Cenpp	488.646	-0.8995	0.16325	-5.5096	3.60E-08	5.83E-07	538.898	690.904	678.922	362.851	309.415	350.885	Down
Gm8739	109.344	-0.7153	0.30406	-2.3526	0.01864	0.06322	94.8151	163.862	148.962	81.9006	97.1543	69.3727	Down
Hist1h2al	116.853	-0.6077	0.26146	-2.3243	0.02011	0.06708	146.093	127.448	149.917	82.9373	77.0899	117.632	Down
Cks2	272.267	0.58907	0.21155	2.78453	0.00536	0.02296	247.68	222.316	182.383	389.805	277.735	313.685	Up
Sema4d	1638.28	0.66717	0.14422	4.62599	3.73E-06	4.07E-05	1378.69	1339.65	1079.97	1777.97	2130	2123.41	Up
Gadd45g	2062.11	0.76771	0.21731	3.53276	0.00041	0.00262	1099.08	1973.05	1505.85	2628.07	2974.82	2191.77	Up
Syk	3980.55	1.17992	0.18696	6.31106	2.77E-10	6.81E-09	3115.35	2332.4	1865.84	5048.81	5893.68	5627.23	Up
Auh	1942.38	-0.2358	0.11576	-2.0374	0.04161	0.11823	2104.32	2216.45	1981.38	1849.5	1905.07	1597.58	Down
Gm33424	81.5241	-0.7923	0.29066	-2.7259	0.00641	0.02658	108.36	105.408	96.4432	60.1295	45.4091	73.3943	Down
Gm48336	28.4534	-1.7046	0.57099	-2.9854	0.00283	0.0134	51.2776	45.9964	33.4209	5.18358	13.7283	21.1134	Down
Gm2762	9.16466	3.09997	1.08872	2.84735	0.00441	0.01949	0	1.91652	3.81953	22.8077	6.33615	20.108	Up
Hrh2	80.0121	1.16381	0.37033	3.14262	0.00167	0.00858	75.4651	38.3303	34.3758	105.745	124.611	101.545	Up
Gm2830	224.331	-0.8513	0.17999	-4.7295	2.25E-06	2.56E-05	277.673	298.977	289.33	182.462	145.732	151.816	Down
Faf2	3939.28	0.34523	0.09413	3.66766	0.00024	0.00165	3616.52	3559.93	3234.19	4209.07	4520.84	4495.15	Up
Hk3	405.014	1.51031	0.28175	5.36044	8.30E-08	1.25E-06	238.973	253.939	138.458	572.267	773.011	453.436	Up
Fgfr4	2755.39	-0.8996	0.18994	-4.7361	2.18E-06	2.49E-05	4309.25	2806.74	3646.7	1916.89	2245.11	1607.64	Down
Prelid1	7533.73	0.3577	0.10034	3.56478	0.00036	0.00235	6805.41	6389.67	6618.29	8795.5	8942.42	7651.1	Up
Mxd3	41.0415	1.1394	0.3938	2.89332	0.00381	0.01726	32.895	22.0399	21.9623	45.6155	64.4175	59.3187	Up
Lman2	10397.9	0.24137	0.09336	2.58536	0.00973	0.03735	9926.57	9599.84	9063.75	11537.6	11786.3	10473.3	Up
Pdlim7	2166.8	-0.6269	0.19105	-3.2815	0.00103	0.00573	1891.47	2944.73	3054.67	1695.03	1836.43	1578.48	Down
Dok3	1283.65	0.62813	0.16835	3.73101	0.00019	0.00134	1053.61	1094.33	877.537	1456.59	1877.61	1342.21	Up
Fam193b	7166.45	-0.292	0.10138	-2.88	0.00398	0.01788	7650.03	7364.22	8653.15	6575.89	6583.26	6172.16	Down
Tmed9	6574.69	0.28276	0.10643	2.65671	0.00789	0.03144	5719.87	5906.71	6170.45	7569.06	7642.46	6439.59	Up
Macroh2a	2923.05	0.44187	0.09903	4.46178	8.13E-06	8.17E-05	2678.04	2335.28	2423.49	3366.22	3252.56	3482.71	Up
Tifab	581.957	1.29978	0.20681	6.28473	3.28E-10	7.92E-09	354.106	366.055	288.375	757.839	1050.75	674.624	Up
Slc25a48	19.9953	-1.8669	0.65249	-2.8612	0.00422	0.0188	31.9275	45.9964	16.233	12.4406	6.33615	7.03781	Down
Tgfbi	5324.75	1.47349	0.11253	13.0945	3.54E-39	1.59E-36	3059.24	2934.19	2465.51	7724.57	7997.28	7767.73	Up
Trpc7	22.9696	3.2104	0.60761	5.28368	1.27E-07	1.84E-06	4.83751	4.79129	3.81953	36.2851	33.7928	54.2916	Up
Klhl3	288.405	-0.6039	0.17879	-3.3777	0.00073	0.00428	341.528	394.803	307.472	213.563	220.709	252.356	Down
Gm48295	3.3695	-4.1115	1.98716	-2.069	0.03854	0.11133	1.935	16.2904	0.95488	1.03672	0	0	Down
2210016F	2344.45	0.44492	0.12119	3.67129	0.00024	0.00163	2151.72	1979.76	1825.74	2764.92	2912.52	2432.06	Up
Ntrk2	2634.17	1.17496	0.24597	4.77674	1.78E-06	2.08E-05	1589.6	2131.17	1130.58	4696.32	3161.74	3095.63	Up
Golm1	3510.84	2.08088	0.13728	15.1575	6.76E-52	7.67E-49	1495.76	1409.6	1121.99	5257.19	5834.54	5945.94	Up
Isca1	4896.63	-0.2832	0.11171	-2.5352	0.01124	0.04206	5161.62	5913.41	5052.29	4615.46	4060.42	4576.58	Down
Tut7	2603.23	0.52207	0.10813	4.82838	1.38E-06	1.64E-05	2242.67	1969.22	2200.05	2844.75	3114.22	3248.45	Up
Gm19792	21.9764	2.0261	0.68795	2.94515	0.00323	0.015	14.5125	7.66607	3.81953	20.7343	55.9693	29.1566	Up
Gm3604	171.527	0.68495	0.19334	3.54271	0.0004	0.00253	137.385	131.281	126.045	188.682	217.541	228.226	Up
Gm48691	28.3583	1.64159	0.57168	2.87153	0.00408	0.0183	10.6425	20.1234	10.5037	32.1382	67.5856	29.1566	Up
Gm48701	32.8722	0.92257	0.44575	2.06971	0.03848	0.11122	14.5125	31.6225	21.9623	42.5053	45.4091	41.2214	Up
Fbp2	475.576	2.89565	0.67047	4.31886	1.57E-05	0.00015	111.263	123.615	103.127	321.382	1599.88	594.192	Up
Fbp1	254.672	2.8317	0.37628	7.52561	5.25E-14	2.33E-12	63.8551	63.2451	61.1125	201.123	536.461	602.235	Up
Zfp367	1626.85	-0.309	0.12384	-2.4955	0.01258	0.04612	1745.37	1883.94	1772.26	1472.14	1276.73	1610.65	Down
Cdc14b	1102.67	-0.4392	0.14173	-3.0987	0.00194	0.00973	1285.81	1412.47	1109.57	962.072	842.708	1003.39	Down
Prxl2c	2609.06	-0.6558	0.18866	-3.476	0.00051	0.00315	3274.02	2981.14	3321.08	2497.45	1477.38	2103.3	Down
Cdk20	440.231	-0.4364	0.16737	-2.6076	0.00912	0.03537	431.506	496.378	591.073	380.475	355.881	386.074	Down

Uqcrb	2746.42	-0.3272	0.14906	-2.1954	0.02814	0.08754	2648.05	3463.15	3058.49	2796.02	2214.49	2298.35 Down
Mterf3	2661.23	-0.3194	0.08329	-3.8345	0.00013	0.00093	2921.85	2958.14	2984.01	2352.31	2296.86	2454.18 Down
Ptdss1	1512.8	0.33851	0.09684	3.49562	0.00047	0.00296	1396.1	1264.9	1347.34	1709.54	1717.1	1641.82 Up
F630042J(	139.636	0.58634	0.23939	2.44929	0.01431	0.05115	92.8801	122.657	119.36	139.957	189.029	173.934 Up
Zfp874a	690.67	-0.4279	0.18473	-2.3164	0.02054	0.06813	786.579	790.563	800.192	572.267	461.483	732.937 Down
Zfp874b	452.971	-0.4194	0.15151	-2.7682	0.00564	0.02396	502.133	464.755	588.208	376.328	381.225	405.177 Down
Zfp58	239.82	-0.4283	0.16837	-2.5439	0.01096	0.0412	260.258	282.686	282.645	202.16	187.973	223.199 Down
Zfp738	342.781	0.44805	0.16051	2.79145	0.00525	0.02256	273.803	317.184	278.826	414.686	420.298	351.89 Up
Zfp65	742.314	0.35964	0.13963	2.57573	0.01	0.03823	710.146	643.95	596.802	742.289	864.885	895.812 Up
Zfp85os	113.332	0.64695	0.25286	2.55848	0.01051	0.03986	84.1726	81.452	99.3078	130.626	165.796	118.637 Up
1700001L	501.962	-0.4559	0.22548	-2.022	0.04318	0.12168	579.533	655.449	507.043	493.477	295.687	480.582 Down
Ube2ql1	41.153	-1.6134	0.39396	-4.0954	4.21E-05	0.00035	48.3751	69.9529	67.7967	18.6609	19.0085	23.1242 Down
Gm3772	3.05128	-5.0055	1.91365	-2.6157	0.00891	0.03469	11.61	3.83303	2.86465	0	0	0 Down
Gm40999	133.69	1.83488	0.29492	6.22161	4.92E-10	1.14E-08	48.3751	65.1616	62.0674	276.803	164.74	184.994 Up
Irx1	7349.15	1.16917	0.2531	4.61932	3.85E-06	4.18E-05	6535.47	3455.48	3581.77	10971.6	7919.13	11631.5 Up
Gm20554	118.193	0.68007	0.26986	2.52007	0.01173	0.04358	85.1401	75.7024	111.721	153.434	118.275	164.886 Up
Irx2	5658.57	0.54193	0.0967	5.60396	2.10E-08	3.55E-07	4553.06	4477.94	4793.51	6885.87	6164.02	7077.02 Up
Ndufs6	1037.23	-0.3464	0.12938	-2.6777	0.00741	0.02989	1131.01	1210.28	1142.04	1018.05	917.686	804.321 Down
Lpcat1	23395.2	0.89322	0.13857	6.4462	1.15E-10	3.02E-09	18734.7	14119	16273.1	30531.3	33364.1	27348.9 Up
Clptm1l	8443.78	0.46375	0.09323	4.97428	6.55E-07	8.44E-06	7321.08	7216.65	6756.75	9936.92	10316.3	9114.96 Up
Tert	111.042	0.70514	0.29199	2.41492	0.01574	0.05516	77.4001	65.1616	110.766	128.553	122.499	161.87 Up
Nkd2	1327.25	-0.3999	0.1907	-2.0969	0.03601	0.10558	1708.61	1545.67	1275.72	1432.74	1048.63	952.115 Down
Gm15912	151.517	-0.6375	0.22059	-2.8898	0.00385	0.01743	203.175	176.32	173.789	133.736	123.555	98.5293 Down
Slc9a3	5.16943	-2.6907	1.26153	-2.1329	0.03294	0.09843	3.87001	12.4574	10.5037	2.07343	2.11205	0 Down
Sdha	13185.3	-0.2753	0.13415	-2.0522	0.04015	0.11486	13558.6	17112.6	12647.4	12136.8	11563.5	12093 Down
Gm46430	789.8	-0.3201	0.11609	-2.757	0.00583	0.02464	821.409	903.638	906.184	672.829	692.753	741.986 Down
Erap1	3593.69	0.32454	0.09455	3.43235	0.0006	0.00361	3335.95	3127.76	3110.05	3807.86	3912.57	4267.93 Up
Cast	6067.82	0.31962	0.08577	3.72629	0.00019	0.00136	5454.77	5454.41	5286.23	6399.65	6683.58	7128.29 Up
Pcsk1	121.089	-2.1703	0.26742	-8.1156	4.83E-16	2.83E-14	219.623	160.029	214.849	48.7256	39.0729	44.2376 Down
Ell2	2495.15	0.51897	0.09414	5.51256	3.54E-08	5.74E-07	2009.5	2122.54	2021.49	2768.03	2953.7	3095.63 Up
Glrx	2521.65	1.09708	0.13304	8.24638	1.63E-16	1.00E-14	1705.71	1605.08	1508.71	3402.5	3916.8	2991.07 Up
Rhobtb3	514.998	-0.6268	0.14173	-4.4229	9.74E-06	9.56E-05	618.233	694.738	562.426	405.356	417.13	392.106 Down
Gm38604	40.3929	-0.9142	0.44537	-2.0527	0.0401	0.11477	39.6676	50.7877	67.7967	27.9913	38.0169	18.0972 Down
A830082K	139.977	-0.5321	0.21823	-2.438	0.01477	0.05241	160.605	174.403	161.375	107.818	134.115	101.545 Down
Arrdc3	9131.67	-0.6837	0.15975	-4.2798	1.87E-05	0.00017	9832.72	13662.9	10271.7	7829.28	6976.1	6217.4 Down
5430425K	263.385	-1.1724	0.26139	-4.4853	7.28E-06	7.43E-05	290.25	417.801	386.728	187.646	110.883	187.005 Down
Adgrv1	196.296	-1.161	0.33342	-3.482	0.0005	0.00309	313.47	305.685	194.796	151.361	69.6977	142.767 Down
Mblac2	908.727	-0.3612	0.12584	-2.8704	0.0041	0.01835	981.046	1140.33	944.379	814.859	761.394	810.353 Down
A230107N	27.298	-1.2082	0.4615	-2.6179	0.00885	0.03451	44.5051	37.3721	32.466	12.4406	16.8964	20.108 Down
Rasa1	1237.85	0.32201	0.15233	2.11397	0.03452	0.10217	1123.27	983.173	1194.56	1239.91	1272.51	1613.67 Up
Cox7c	1944.41	-0.3506	0.14935	-2.3472	0.01891	0.06394	1837.29	2499.14	2201.96	1896.15	1626.28	1605.63 Down
Edil3	868.123	-0.6607	0.14052	-4.7019	2.58E-06	2.91E-05	1007.17	963.05	1220.34	634.47	676.912	706.797 Down
Hapln1	44.0719	1.52675	0.46806	3.26185	0.00111	0.00608	20.3175	24.9147	22.9172	87.0841	33.7928	75.4051 Up
Atp6ap1l	6.99544	4.38442	1.88094	2.33097	0.01975	0.06616	0	1.91652	0	27.9913	0	12.0648 Up
Rps23	12028.6	-0.258	0.12388	-2.0831	0.03724	0.10839	13174.5	11527.9	14602.1	11362.4	11503.3	10001.7 Down
Ssbp2	1548.76	-0.3281	0.10756	-3.0508	0.00228	0.01114	1796.65	1758.4	1617.57	1393.35	1277.79	1448.78 Down
4833422C	396.055	-0.369	0.15571	-2.3699	0.0178	0.06088	433.441	489.67	416.329	326.565	325.256	385.069 Down
Ckmt2	1761.26	-5.5423	0.78729	-7.0398	1.93E-12	6.61E-11	1928.23	6808.43	1608.98	59.0928	41.185	121.654 Down
Ankrd34b	44.2568	2.01018	0.372	5.40377	6.53E-08	1.01E-06	17.415	16.2904	19.0977	62.203	78.1459	72.3889 Up
Fam151b	756.683	-0.3711	0.13794	-2.6899	0.00715	0.02901	817.539	922.803	820.244	663.498	586.094	729.921 Down
Mtx3	1215.79	-0.2694	0.12689	-2.1236	0.03371	0.1002	1197.77	1365.52	1423.73	1002.5	1128.89	1176.32 Down
Cmya5	963.201	-3.7855	0.58959	-6.4205	1.36E-10	3.53E-09	1170.68	3267.66	950.108	160.691	101.378	128.691 Down
Gm47155	102.504	-0.723	0.31558	-2.291	0.02196	0.0718	110.295	164.82	107.902	55.9827	85.5381	90.4861 Down
Jmy	2623.07	-0.2352	0.09565	-2.4586	0.01395	0.05013	2705.13	2753.08	3050.85	2354.38	2448.92	2426.03 Down
Arsb	2192.64	0.3427	0.10516	3.2588	0.00112	0.00614	2081.1	1886.81	1832.42	2608.38	2404.57	2342.58 Up
Lhfpl2	1000.52	1.87778	0.23367	8.03593	9.29E-16	5.19E-14	513.743	468.588	301.743	1423.41	1970.54	1325.12 Up
Scamp1	7655.73	0.70807	0.09098	7.78263	7.10E-15	3.51E-13	5834.03	5717.93	5889.72	8843.19	9500	10149.5 Up
Gm9776	139.609	0.66022	0.22917	2.8809	0.00397	0.01785	115.133	112.116	97.3981	140.993	189.029	182.983 Up
Ap3b1	4094.44	0.39676	0.0957	4.14603	3.38E-05	0.00029	3764.55	3366.36	3473.86	4584.36	4910.52	4467 Up
Tbca	2780.98	0.21473	0.099	2.16906	0.03008	0.09199	2619.03	2474.22	2629.75	3140.21	3069.87	2752.79 Up
Pde8b	2725.76	-0.3793	0.10648	-3.5625	0.00037	0.00237	3141.48	2778.95	3325.86	2335.72	2347.54	2425.03 Down
Zbed3	816.986	0.31119	0.10857	2.86616	0.00415	0.01857	763.359	701.445	722.846	938.228	891.285	884.753 Up
F2rl1	872.009	0.39579	0.19764	2.0026	0.04522	0.12605	792.384	659.282	807.831	1221.25	752.946	998.363 Up
Sv2c	51.9295	-1.0763	0.46647	-2.3074	0.02103	0.06938	81.2701	57.4955	72.5711	14.514	47.5211	38.2052 Down
Col4a3bp	5895.03	0.24306	0.08334	2.91667	0.00354	0.01621	5450.9	5323.13	5424.69	6741.76	6310.81	6118.87 Up
Hmgcr	4596.15	1.55202	0.1226	12.6592	9.96E-37	3.70E-34	2621.93	2317.07	2074.01	6703.4	7361.55	6498.91 Up
Hexb	2565.44	1.23614	0.17432	7.09141	1.33E-12	4.70E-11	1875.99	1431.64	1279.54	3099.78	3697.14	4008.53 Up
Enc1	1214.5	0.55033	0.19894	2.76636	0.00567	0.02407	911.386	1070.37	974.935	1302.12	1886.06	1142.14 Up
Lncenc1	2.411	4.77074	1.94119	2.45764	0.01399	0.05023	0	0	0	6.2203	4.2241	4.0216 Up
Gm5086	45.7308	0.8247	0.39413	2.09245	0.0364	0.10653	46.4401	24.9147	27.6916	50.7991	60.1934	64.3457 Up
Tnpo1	6736.21	0.2456	0.09653	2.54442	0.01095	0.04118	6130.09	5866.46	6496.07	6774.94	7599.16	7550.56 Up
A930014D	12.6518	3.88595	0.95239	4.08021	4.50E-05	0.00037	3.87001	0	0.95488	24.8812	20.0645	26.1404 Up
Zfp366	1991.92	-0.4097	0.11941	-3.4311	0.0006	0.00362	2087.87	2268.2	2462.64	1576.84	1695.98	1859.99 Down
BC001981	5.9948	-2.5646	1.27585	-2.0101	0.04442	0.12435	9.67501	18.2069	2.86465	3.11015	2.11205	0 Down
Naip2	1365.98	1.93341	0.1356	14.2581	3.99E-46	2.87E-43	660.804	531.834	507.998	2079.65	2240.89	2174.68 Up
Naip5	1402.06	1.53245	0.17283	8.86693	7.52E-19	6.03E-17	758.521	780.981	621.629	2347.12	1659.02	2245.06 Up
Naip3-ps1	79.635	1.29695	0.2849	4.5523	5.31E-06	5.57E-05	39.6676	49.8294	48.699	131.663	101.378	106.572 Up
Naip6	382.439	1.57948	0.14695	10.7484	6.03E-27	9.86E-25	208.98	186.86	179.518	596.112	548.077	575.089 Up
Naip1	25.901	1.60249	0.712	2.25068	0.02441	0.07808	19.35	17.2487	1.90977	30.0648	49.6332	37.1998 Up
Marveld2	1317.77	0.47187	0.10117	4.66391	3.10E-06	3.44E-05	1176.48	1092.41	1043.69	1547.82	1506.95	1539.27 Up
Ccdc125	779.699	0.77895	0.1648	4.72679	2.28E-06	2.59E-05	641.453	566.331	514.682	879.135	1162.68	913.909 Up
Ccnb1	132.384	1.12551	0.26129	4.30751	1.65E-05	0.00015	84.1726	96.7841	68.7516	193.866	143.619	207.113 Up
Pik3r1	5638.54	-0.3642	0.09403	-3.873	0.00011	0.00081	6126.22	6890.84	6022.45	4945.13	4859.83	4986.79 Down

Cd180	445.133	0.62899	0.23503	2.67622	0.00745	0.02997	454.726	306.643	287.42	485.183	665.296	471.533	Up
Nln	2250.58	0.21147	0.10364	2.04041	0.04131	0.11749	2263.95	2033.42	1960.37	2381.34	2521.79	2342.58	Up
Sgtb	416.698	-0.5134	0.14774	-3.4748	0.00051	0.00316	517.613	477.213	475.532	314.125	332.648	383.058	Down
Cwc27	463.824	-0.3835	0.15205	-2.5219	0.01167	0.0434	483.751	523.209	568.155	412.613	439.307	355.912	Down
Nt5el	2.95789	5.06444	2.01444	2.51407	0.01193	0.04413	0	0	0	8.29373	8.4482	1.0054	Up
Rnf180	459.935	1.94785	0.20832	9.35009	8.76E-21	8.11E-19	240.908	174.403	152.781	702.893	831.092	657.532	Up
Gm30411	15.6209	-2.8649	0.80291	-3.5682	0.00036	0.00233	12.5775	44.0799	25.7818	4.14686	2.11205	5.027	Down
Dimt1	930.672	-0.4036	0.11615	-3.4751	0.00051	0.00316	1065.22	1019.59	1095.25	729.848	841.652	832.472	Down
Ndufaf2	680.357	-0.4233	0.13156	-3.2173	0.00129	0.00694	731.431	813.562	793.508	615.809	519.564	608.268	Down
Elovl7	607.837	0.56094	0.13938	4.02471	5.70E-05	0.00046	514.711	515.543	443.066	706.003	793.075	674.624	Up
Pde4d	1749.31	-0.4246	0.12563	-3.38	0.00072	0.00425	1756.98	2164.71	2093.1	1404.75	1484.77	1591.55	Down
Rab3c	324.328	1.18969	0.25847	4.60285	4.17E-06	4.49E-05	248.648	183.027	161.375	439.568	575.534	337.815	Up
Gapt	303.118	3.16258	0.4092	7.72868	1.09E-14	5.19E-13	82.2376	60.3703	40.1051	469.632	882.837	283.523	Up
Plk2	4841.94	0.41347	0.13852	2.98483	0.00284	0.01342	3649.42	4329.41	4479.36	5540.21	6226.33	4826.93	Up
Gm33172	4.60544	2.63399	1.28155	2.05532	0.03985	0.11415	0	1.91652	1.90977	4.14686	11.6163	8.04321	Up
Mier3	1802.27	-0.2921	0.12101	-2.414	0.01578	0.05527	2048.2	1864.77	2039.63	1638.01	1440.42	1782.58	Down
Ddx4	167.209	2.13311	0.46619	4.57562	4.75E-06	5.05E-05	47.4076	112.116	26.7367	327.602	183.748	305.642	Up
Plpp1	6392.74	-0.6287	0.16917	-3.7163	0.0002	0.00141	8888.44	6978.04	7425.17	5028.07	5994	4042.72	Down
Dhx29	2007.99	0.49155	0.08471	5.80296	6.52E-09	1.23E-07	1683.45	1660.66	1663.41	2290.11	2407.74	2342.58	Up
Ccno	328.5	1.20857	0.1937	6.23944	4.39E-10	1.03E-08	182.858	245.314	167.105	487.256	434.026	454.441	Up
Mcidas	111.571	2.44708	0.25551	9.57717	9.97E-22	1.01E-19	39.6676	33.5391	30.5563	200.086	180.58	184.994	Up
Cdc20b	137.053	1.31	0.28581	4.58353	4.57E-06	4.88E-05	90.9451	90.0763	55.3832	164.838	184.804	236.269	Up
Gzma	899.518	-0.4687	0.22611	-2.073	0.03817	0.11048	814.636	1309.94	1008.36	682.159	954.647	627.37	Down
Hspb3	11.3829	-4.3883	1.3833	-3.1724	0.00151	0.00788	3.87001	52.7042	8.59395	2.07343	1.05603	0	Down
Fst	324.615	-1.0896	0.22332	-4.8789	1.07E-06	1.31E-05	437.311	480.088	407.735	263.326	153.124	206.107	Down
Itga1	11300.5	-0.2795	0.13753	-2.032	0.04215	0.11944	11043.1	12554.1	13577.5	8961.37	11676.5	9990.67	Down
Parp8	1315.59	0.35276	0.11213	3.14607	0.00165	0.0085	1206.47	1140.33	1120.08	1454.51	1367.55	1604.62	Up
Gm6416	14.4444	-3.4793	1.04581	-3.3269	0.00088	0.00499	13.545	46.9547	19.0977	1.03672	0	6.03241	Down
Emb	14562	0.79734	0.10542	7.56379	3.91E-14	1.77E-12	11438.8	10737.3	9735.99	18169.5	19820.5	17469.8	Up
Gm6421	351.177	0.34563	0.16111	2.14526	0.03193	0.09616	349.268	301.851	276.916	408.466	365.385	405.177	Up
Mrps30	1181.02	-0.2906	0.11891	-2.4436	0.01454	0.05177	1220.99	1347.31	1330.15	1153.86	1066.59	967.196	Down
Fgf10	1186.06	-0.8257	0.15413	-5.3572	8.45E-08	1.27E-06	1443.51	1468.05	1637.62	859.437	996.888	710.818	Down
Ccl28	367.256	2.80515	0.29106	9.63779	5.54E-22	5.71E-20	110.295	110.2	55.3832	538.056	569.198	820.407	Up
Hmgcs1	7988.07	1.12142	0.17509	6.40473	1.51E-10	3.90E-09	4797.84	5996.78	4297.93	10111.1	13227.8	9497.02	Up
D13Ertd6(	4.02092	-4.3775	1.65043	-2.6523	0.00799	0.03178	10.6425	3.83303	8.59395	0	1.05603	0	Down
B020031N	1.9063	4.43085	2.12145	2.08859	0.03674	NA	0	0	0	4.14686	5.28013	2.0108	Up
1700110I(	15.1062	4.93032	1.28314	3.84238	0.00012	0.0009	0	2.87478	0	61.1662	9.50423	17.0918	Up
Gm26898	12.4956	4.6463	1.06864	4.34787	1.37E-05	0.00013	0.9675	0	1.90977	30.0648	16.8964	25.135	Up
Gm3618	11.0415	3.14825	0.98909	3.18297	0.00146	0.00766	1.935	1.91652	2.86465	5.18358	22.1765	32.1728	Up
Gm10251	27.819	3.75801	0.83049	4.52505	6.04E-06	6.26E-05	0	4.79129	6.68418	27.9913	36.9609	90.4861	Up
Gm10339	5.14586	3.91509	1.44914	2.70167	0.0069	0.0282	0	1.91652	0	8.29373	11.6163	9.04861	Up
4933406F(	10.9774	1.96841	0.95743	2.05593	0.03979	0.11402	0	8.62433	4.77441	17.6242	13.7283	21.1134	Up
Flnb	6732.79	0.24057	0.10995	2.18803	0.02867	0.08879	6040.11	5634.56	6843.65	7275.67	6948.65	7654.12	Up
Dnase1l3	233.785	1.08349	0.27863	3.8886	0.0001	0.00077	184.793	123.615	141.323	215.637	385.449	351.89	Up
Fam107a	2472.97	-1.1721	0.13809	-8.4883	2.10E-17	1.47E-15	3536.22	3695.05	3046.08	1316.63	1560.81	1683.04	Down
Fhit	393.242	-0.9975	0.23193	-4.3009	1.70E-05	0.00016	363.781	619.993	588.208	283.023	267.174	237.275	Down
Sntn	2767.07	-0.7083	0.23451	-3.0204	0.00252	0.01215	3016.67	3983.48	3299.12	2043.37	1423.52	2836.24	Down
Ngly1	3348.96	-0.2017	0.09132	-2.2087	0.0272	0.08512	3520.74	3510.1	3717.36	2942.2	3124.78	3278.61	Down
Gm48163	48.2295	-0.7897	0.38272	-2.0635	0.03907	0.11242	56.1151	76.6607	50.6088	40.4319	25.3446	40.216	Down
Rarb	611.544	-1.3639	0.24782	-5.5035	3.72E-08	6.03E-07	757.554	902.68	982.574	283.023	260.838	482.592	Down
Thrb	1304.98	-0.3221	0.11576	-2.7826	0.00539	0.02306	1555.74	1500.63	1293.87	1145.57	1164.8	1169.28	Down
Nid2	481.724	-0.8674	0.19763	-4.3892	1.14E-05	0.00011	547.606	748.4	571.02	277.84	366.441	379.036	Down
Gng2	1159.32	-0.365	0.13539	-2.696	0.00702	0.02859	1178.42	1244.78	1492.48	991.1	967.319	1081.81	Down
Saysd1	732.982	-0.3765	0.13408	-2.8083	0.00498	0.02165	796.254	776.19	911.913	681.122	582.926	649.489	Down
Kcnk5	472.568	-0.4616	0.16417	-2.8114	0.00493	0.02148	550.508	508.835	583.433	411.576	332.648	448.409	Down
Nudt13	1436.44	-0.385	0.11846	-3.2499	0.00115	0.00629	1457.06	1747.86	1675.82	1249.24	1306.3	1182.35	Down
Dnajc9	1643.41	0.27354	0.1177	2.324	0.02013	0.06711	1593.47	1413.43	1457.15	1850.54	1925.13	1620.71	Up
Cfap70	1704.82	-0.7242	0.2303	-3.1447	0.00166	0.00854	1804.39	2306.53	2261.16	1470.06	837.428	1549.32	Down
Anxa7	11070.9	0.25598	0.08987	2.84835	0.00439	0.01944	10351.3	9668.83	10253.5	11710.7	12889.8	11551.1	Up
Mss51	281.398	-0.5666	0.20961	-2.7033	0.00687	0.02809	337.658	286.519	383.863	207.343	198.533	274.474	Down
Myoz1	15.532	-1.3717	0.66529	-2.0618	0.03923	0.11278	17.415	25.873	23.8721	6.2203	14.7844	5.027	Down
Synpo2l	266.826	-4.755	0.87066	-5.4614	4.72E-08	7.53E-07	304.763	985.09	253.999	16.5875	6.33615	34.1836	Down
Sec24c	423.293	0.69645	0.19359	3.59755	0.00032	0.00211	374.423	274.062	320.841	465.485	482.604	622.343	Up
Zswim8	6240.69	0.21402	0.07948	2.69268	0.00709	0.02884	5963.68	5561.73	5810.46	6761.46	6818.76	6528.07	Up
Camk2g	2762.8	0.28924	0.10804	2.67708	0.00743	0.02991	2529.05	2436.85	2494.15	2995.07	3350.77	2770.88	Up
Plau	522.34	0.58345	0.1679	3.47497	0.00051	0.00316	360.878	451.34	442.111	540.129	700.145	639.435	Up
Ap3m1	4183.67	0.34271	0.08804	3.89274	9.91E-05	0.00075	3886.45	3535.02	3645.74	4588.5	4856.66	4589.65	Up
Adk	7980.25	0.34683	0.11737	2.95497	0.00313	0.01459	7899.65	6196.1	6981.15	8742.62	8765.01	9296.94	Up
Dusp13	26.5671	1.46992	0.48424	3.03549	0.0024	0.01164	14.5125	16.2904	11.4586	41.4686	47.5211	28.1512	Up
Samd8	2280.36	0.30512	0.10375	2.9409	0.00327	0.01516	1982.41	1961.56	2176.18	2565.87	2649.57	2346.61	Up
Vdac2	14292.6	0.23389	0.10972	2.13174	0.03303	0.09864	12284.4	14298.2	12826.9	15872.1	16407.5	14066.6	Up
A430057N	27.8422	1.51883	0.4522	3.35879	0.00078	0.00454	16.4475	10.5408	16.233	38.3585	42.241	43.2322	Up
Lrmda	781.906	0.39478	0.13491	2.9262	0.00343	0.0158	650.161	746.483	630.223	929.934	803.635	931.001	Up
Gm10248	17.6549	3.49166	0.81434	4.2877	1.81E-05	0.00017	4.83751	2.87478	0.95488	31.1015	16.8964	49.2646	Up
Kcnma1	93.3513	-1.9697	0.36075	-5.46	4.76E-08	7.58E-07	104.49	200.276	141.323	36.2851	48.5772	29.1566	Down
Gm31392	12.2953	-2.0547	0.90684	-2.2658	0.02346	0.07576	16.4475	25.873	17.1879	6.2203	0	8.04321	Down
Dlg5	186.558	0.91479	0.26832	3.40929	0.00065	0.00388	142.223	125.532	120.315	212.527	187.973	330.777	Up
Rps24	24347.6	-0.2977	0.1161	-2.5644	0.01034	0.03928	26035.5	24002.5	30515.2	21863.3	22498.6	21170.7	Down
Zcchc24	4310.55	-0.6245	0.09537	-6.5477	5.84E-11	1.61E-09	5293.2	5172.68	5221.3	3213.82	3688.7	3273.59	Down
Plac9a	16.0087	-1.1481	0.58156	-1.9742	0.04836	0.13302	17.415	26.8312	21.9623	9.33044	8.4482	12.0648	Down
Pde12	1153.06	0.40893	0.09947	4.11114	3.94E-05	0.00033	1012.01	1009.05	951.063	1293.82	1366.5	1285.91	Up
Dnah12	2567.8	-0.4538	0.18511	-2.4513	0.01423	0.05096	2694.49	3292.58	2918.12	2220.65	1634.73	2646.22	Down
Asb14	970.463	-0.7173	0.23992	-2.99	0.00279	0.01322	970.404	1525.55	1124.85	817.969	515.34	868.666	Down

Appl1	3424.47	-0.2158	0.09707	-2.2231	0.02621	0.08269	3505.26	3870.41	3664.84	2990.93	3154.35	3361.06	Down
Hesx1	21.394	1.85203	0.60421	3.06519	0.00218	0.01069	10.6425	11.4991	5.7293	33.1749	20.0645	47.2538	Up
Il17rd	1644.87	-0.2933	0.11297	-2.5959	0.00944	0.03643	1962.09	1696.12	1776.08	1449.33	1585.09	1400.52	Down
Cacna2d3	138.279	-1.3516	0.40318	-3.3523	0.0008	0.00462	146.093	332.516	117.451	72.5701	87.6501	73.3943	Down
Cacna1d	1499.17	-0.3355	0.147	-2.2826	0.02245	0.073	1384.49	1699.95	1933.64	1309.37	1368.61	1298.98	Down
Tkt	12877.2	0.65374	0.14597	4.47866	7.51E-06	7.64E-05	10615.4	11214.5	8195.76	15072.8	17368.4	14796.5	Up
Prkcd	7038.9	0.42534	0.11063	3.84464	0.00012	0.0009	6092.36	5545.44	6388.17	8328.98	8581.26	7297.2	Up
Rft1	1339.9	0.3158	0.11834	2.66856	0.00762	0.03055	1247.11	1282.15	1052.28	1483.54	1496.39	1477.94	Up
Sfmbt1	1424.72	0.30399	0.13317	2.28269	0.02245	0.073	1400.94	1155.66	1269.04	1406.82	1594.6	1721.25	Up
Stimate	3129.92	0.6633	0.10923	6.07232	1.26E-09	2.75E-08	2678.04	2259.57	2330.87	3668.94	4025.57	3816.5	Up
Itih4	6260.02	1.56948	0.72041	2.17858	0.02936	0.09044	3269.19	3917.36	2279.31	6511.61	3475.38	18107.3	Up
Itih1	243.416	1.95486	0.41769	4.68013	2.87E-06	3.20E-05	104.49	68.9946	126.045	301.684	202.757	656.527	Up
Glt8d1	3291.15	-0.3851	0.09774	-3.9401	8.14E-05	0.00063	3589.43	3834.95	3759.37	2806.39	2681.25	3075.52	Down
Smim4	353.457	-0.9309	0.15278	-6.0934	1.11E-09	2.43E-08	458.596	426.425	506.088	229.114	248.166	252.356	Down
Nt5dc2	1936.73	-0.8857	0.20182	-4.3888	1.14E-05	0.00011	3123.09	2224.12	2192.41	1522.94	1512.23	1045.62	Down
Stab1	3869.49	-0.3266	0.13631	-2.3958	0.01658	0.05753	4087.69	3912.57	4916.69	3505.14	3048.75	3746.12	Down
Sema3g	6437.92	-0.6193	0.20686	-2.9938	0.00276	0.01308	5860.16	7015.41	10520.9	4629.97	5455.43	5145.64	Down
Dnah1	1516.71	-0.9596	0.19965	-4.8065	1.54E-06	1.82E-05	1758.92	2217.41	2033.9	1091.66	747.666	1250.72	Down
Capn7	401.857	0.85913	0.19825	4.33352	1.47E-05	0.00014	333.788	256.813	266.412	482.073	446.699	625.359	Up
Sh3bp5	4213.61	-0.5736	0.15311	-3.7463	0.00018	0.00127	4497.91	5097.94	5524.95	3179.61	4042.47	2938.79	Down
Mettl6	1642.32	-0.3295	0.14466	-2.2779	0.02273	0.07381	1692.16	1786.19	2009.07	1402.68	1276.73	1687.06	Down
Colq	1188.5	-1.8277	0.25777	-7.0902	1.34E-12	4.74E-11	1653.46	2071.76	1838.15	669.718	589.262	308.658	Down
Galnt15	1153.87	-1.5658	0.21324	-7.3429	2.09E-13	8.41E-12	1669.91	2037.26	1467.66	646.911	683.248	418.247	Down
Dph3	1550.03	-0.3008	0.11238	-2.6766	0.00744	0.02994	1552.84	1712.41	1867.75	1421.34	1386.56	1359.3	Down
Oxnad1	693.567	-0.4485	0.17618	-2.5458	0.0109	0.04105	714.984	935.26	751.493	563.973	509.004	686.689	Down
Ncoa4	1712.11	0.68435	0.16569	4.13037	3.62E-05	0.00031	1543.16	1215.07	1182.15	1912.74	2491.16	1928.36	Up
Ogdhl	90.4992	-1.7558	0.56234	-3.1224	0.00179	0.0091	51.2776	275.978	91.6688	44.5788	43.297	36.1944	Down
1700024G	1009.01	-1.2652	0.22634	-5.5901	2.27E-08	3.83E-07	1309.03	1566.75	1399.86	634.47	393.897	750.029	Down
3425401B	85.0349	-1.0476	0.4898	-2.1389	0.03244	0.09722	110.295	183.027	50.6088	45.6155	42.241	78.4213	Down
Tmem273	347.405	1.51317	0.20701	7.30953	2.68E-13	1.06E-11	191.565	181.111	168.059	448.898	648.4	446.398	Up
Vstm4	1526.12	-0.5499	0.20897	-2.6315	0.0085	0.03341	1358.37	2104.34	1977.56	1180.82	1510.12	1025.51	Down
Wdfy4	2011.31	1.05231	0.19935	5.27876	1.30E-07	1.89E-06	1644.75	1077.08	1204.11	2495.38	3296.91	2349.62	Up
Arhgap22	289.265	0.92774	0.28922	3.20772	0.00134	0.00713	203.175	123.615	271.187	321.382	437.195	379.036	Up
A630023A	24.4975	2.29228	0.64744	3.54051	0.0004	0.00255	1.935	12.4574	10.5037	32.1382	51.7452	38.2052	Up
Rpl23a-ps	1304.25	-0.3693	0.17248	-2.1408	0.03229	0.09686	1495.76	1221.78	1693.01	1148.68	1302.08	964.179	Down
Glud1	12749.8	0.25431	0.10347	2.45783	0.01398	0.05022	12498.2	11224.1	11164.5	13745.8	14985	12881.2	Up
Ldb3	1789.92	-4.5742	0.67238	-6.803	1.02E-11	3.19E-10	2067.55	6423.21	1816.19	129.589	91.8742	211.134	Down
Lrit2	3.36821	5.2546	2.46025	2.1358	0.0327	0.09783	0	0	0	0	2.11205	18.0972	Up
Cdhr1	1948.84	3.30155	0.85553	3.85907	0.00011	0.00085	208.013	750.317	118.405	3120.51	1940.97	5554.84	Up
Tspan14	4772.13	0.37419	0.10698	3.49793	0.00047	0.00294	4049.96	3991.15	4428.75	5257.19	5896.85	5008.91	Up
Prxl2a	3375.78	-0.4176	0.16706	-2.4997	0.01243	0.04567	3130.83	4495.19	3957.03	3116.37	2462.65	3092.61	Down
Dydc1	133.759	-0.8898	0.3278	-2.7145	0.00664	0.02728	174.15	177.278	169.969	105.745	51.7452	123.664	Down
Mat1a	1721.63	1.01277	0.25763	3.93105	8.46E-05	0.00065	1187.12	988.923	1247.08	2178.14	1471.04	3257.5	Up
Sftpa1	211905	0.91799	0.1577	5.82095	5.85E-09	1.12E-07	178407	125519	136095	266922	254067	310422	Up
Sftpd	88511.5	2.05954	0.15841	13.001	1.21E-38	5.21E-36	42643.6	28132.6	31974.3	142971	143770	141577	Up
Gm5930	81.7375	-1.7793	0.6108	-2.9131	0.00358	0.01636	247.68	92.9511	39.1502	52.8725	30.6247	27.1458	Down
Gm8229	90.8736	-1.3077	0.54721	-2.3898	0.01686	0.05834	254.453	68.9946	64.932	68.4232	41.185	47.2538	Down
AC160336	605.425	-1.8031	0.37172	-4.8506	1.23E-06	1.48E-05	1433.84	603.703	785.869	416.76	215.429	176.951	Down
Gm8267	66.2114	-5.081	1.92401	-2.6408	0.00827	0.0327	214.785	44.0799	126.999	11.4039	0	0	Down
Gm34060	25.8076	-5.6226	1.16984	-4.8063	1.54E-06	1.82E-05	68.6926	8.62433	74.4809	1.03672	0	2.0108	Down
Ero1l	2847.44	0.47683	0.12443	3.83222	0.00013	0.00094	2643.21	2085.17	2414.9	3385.91	3464.82	3090.6	Up
Gm15601	111.709	1.16595	0.26001	4.48425	7.32E-06	7.46E-05	87.0751	64.2033	55.3832	151.361	158.404	153.826	Up
Ddhd1	2501.2	1.52199	0.10537	14.4442	2.73E-47	2.10E-44	1341.92	1284.07	1249.94	3441.9	4027.68	3661.67	Up
A530076I1	45.9119	0.98999	0.38223	2.59002	0.0096	0.03693	34.8301	29.706	27.6916	47.6889	80.2579	55.297	Up
Bmp4	3411.26	0.5198	0.20431	2.54421	0.01095	0.04119	3214.04	2484.76	2710.91	3958.18	5141.79	2957.89	Up
Cdkn3	62.9704	0.76251	0.34913	2.18403	0.02896	0.08952	36.7651	55.579	47.7441	102.635	70.7537	64.3457	Up
Gmfb	10297.9	0.29214	0.09826	2.97325	0.00295	0.01386	9746.61	8560.12	9469.57	10612.9	11708.2	11689.8	Up
Gm34934	212.884	-0.7528	0.19611	-3.8387	0.00012	0.00091	239.94	278.853	282.645	151.361	183.748	140.756	Down
Gch1	3071.49	1.45111	0.09556	15.1848	4.46E-52	5.34E-49	1697.97	1674.08	1563.14	4292	4766.9	4434.82	Up
Wdhd1	318.774	0.55573	0.18857	2.94712	0.00321	0.01492	294.12	275.02	205.3	400.172	357.993	380.042	Up
Lgals3	11770.1	1.13398	0.19804	5.72592	1.03E-08	1.86E-07	9381.86	6316.84	6407.26	16572.9	18983.1	12958.6	Up
Dlgap5	103.469	0.84599	0.28772	2.94036	0.00328	0.01518	84.1726	84.3268	53.4734	115.075	130.947	152.821	Up
Fbxo34	1603.52	0.50175	0.10684	4.69609	2.65E-06	2.98E-05	1352.57	1322.4	1307.23	1975.98	1955.76	1707.17	Up
Ktn1	5528.52	-0.4207	0.10952	-3.8414	0.00012	0.00091	6031.4	6991.46	5964.2	4624.79	4507.12	5052.14	Down
Peli2	1539.98	-0.5969	0.10484	-5.6934	1.25E-08	2.21E-07	1782.14	1784.28	1995.71	1268.94	1234.49	1174.31	Down
Gm18962	2.40054	-4.6599	1.92551	-2.4201	0.01552	0.0545	4.83751	4.79129	4.77441	0	0	0	Down
Gm48935	1.88299	4.41505	2.21569	1.99262	0.0463	NA	0	0	0	6.2203	1.05603	4.0216	Up
ccdc198	72.3228	4.21976	0.50896	8.29098	1.12E-16	7.06E-15	10.6425	7.66607	3.81953	188.682	82.37	140.756	Up
Tlr11	47.7823	0.8384	0.35302	2.37495	0.01755	0.06015	41.6026	35.4556	25.7818	58.0561	65.4736	60.3241	Up
Tep1	2487.21	0.52559	0.10895	4.82411	1.41E-06	1.68E-05	1978.54	2228.91	1909.77	2801.21	3116.33	2888.52	Up
Gm26782	443.47	0.45489	0.14122	3.22115	0.00128	0.00686	357.008	409.176	356.171	479.999	540.685	517.781	Up
Klhl33	102.79	-0.8203	0.32977	-2.4876	0.01286	0.04694	141.255	153.321	99.3078	61.1662	59.1374	102.551	Down
Apex1	222.618	0.81947	0.17321	4.73123	2.23E-06	2.54E-05	169.313	158.113	155.646	274.73	310.471	267.437	Up
Rnase4	43833.1	0.95843	0.15967	6.00247	1.94E-09	4.04E-08	35625.3	27029.6	26703.3	63085.2	61817.6	48737.8	Up
Rnase1	8.41148	2.4812	1.10003	2.25557	0.0241	0.07734	1.935	1.91652	3.81953	15.5507	2.11205	25.135	Up
Rnase2b	24.7987	4.19698	0.7609	5.51585	3.47E-08	5.64E-07	5.80501	0	1.90977	51.8358	36.9609	52.2808	Up
Gm7107	77.1791	0.77275	0.26414	2.92555	0.00344	0.01583	62.8876	56.5373	51.5637	94.3411	98.2104	99.5347	Up
Ear6	166.406	4.96804	1.46889	3.38217	0.00072	0.00422	27.09	3.83303	0	309.978	461.483	196.053	Up
Mettl17	1359.17	-0.2772	0.12313	-2.2511	0.02438	0.07803	1331.28	1556.21	1580.33	1306.26	1240.83	1140.12	Down
Slc39a2	951.284	3.21927	0.53416	6.02675	1.67E-09	3.53E-08	306.698	114.991	131.774	1689.85	2344.38	1120.02	Up
Ndrg2	4817.98	-1.6629	0.21386	-7.7756	7.51E-15	3.69E-13	6767.67	9256.78	5945.1	2695.46	2518.62	1724.26	Down
Zfp219	3081.75	0.23198	0.10972	2.11428	0.03449	0.10211	2607.42	2844.11	3051.81	3499.95	3404.63	3082.56	Up
Rpgrip1	350.46	1.35336	0.16064	8.42467	3.62E-17	2.47E-15	195.435	218.483	177.608	467.559	504.78	538.895	Up

Supt16	1066.08	0.32719	0.16265	2.01156	0.04427	0.12399	972.339	827.935	1037	1024.28	1139.45	1395.5	Up
Sall2	537.871	-0.5646	0.15285	-3.6938	0.00022	0.00151	658.868	584.538	681.786	465.485	462.539	374.009	Down
Trac	382.218	-0.4962	0.23238	-2.1353	0.03274	0.09794	503.101	389.053	449.75	422.98	264.006	264.42	Down
Olfr49	5.28385	2.17166	1.07826	2.01404	0.04401	0.12348	0.9675	1.91652	2.86465	10.3672	10.5603	5.027	Up
Gm43305	34.928	2.64874	0.84468	3.13579	0.00171	0.00875	7.74001	18.2069	2.86465	19.6976	129.891	31.1674	Up
Slc7a7	2044	0.30481	0.11166	2.72964	0.00634	0.02635	1794.72	2005.64	1686.32	2373.04	2152.18	2252.1	Up
Mmp14	3276.1	0.35388	0.16037	2.2067	0.02733	0.08545	2234.93	3246.58	3147.29	3888.72	3664.41	3474.67	Up
Lrp10	11045.7	0.60534	0.09811	6.17023	6.82E-10	1.54E-08	9080.97	8419.26	8784.92	13895.1	13960.7	12133.2	Up
Acin1	1181.44	0.42449	0.18945	2.24064	0.02505	0.07982	1092.31	757.024	1177.37	1274.12	1254.56	1533.24	Up
Slc7a8	328.855	1.26255	0.30906	4.0851	4.41E-05	0.00036	215.753	249.147	115.541	409.503	606.159	377.025	Up
Slc22a17	1293.42	-0.2961	0.11282	-2.6248	0.00867	0.03396	1530.59	1388.52	1357.84	1159.05	1237.66	1086.84	Down
Efs	259.726	-0.519	0.22729	-2.2833	0.02241	0.0729	387.968	241.481	288.375	235.334	210.149	195.048	Down
Cmtm5	3.35417	-5.1427	1.92411	-2.6728	0.00752	0.03026	1.935	13.4156	4.77441	0	0	0	Down
Myh6	15719.5	-5.3632	1.52949	-3.5066	0.00045	0.00285	20960	53762.1	17357.9	485.183	7.39218	1744.37	Down
Mhrt	46.1383	-4.4523	1.92181	-2.3167	0.02052	0.06809	49.3426	162.904	52.5186	0	0	12.0648	Down
Myh7	256.059	-3.3251	0.4426	-7.5126	5.79E-14	2.56E-12	312.503	775.231	309.382	34.2116	30.6247	74.3997	Down
Ap1g2	1430.19	0.48706	0.11712	4.15876	3.20E-05	0.00028	1193.9	1198.78	1180.24	1765.53	1767.79	1474.92	Up
Pck2	2547.5	0.27179	0.13053	2.08225	0.03732	0.10856	2378.12	2252.87	2293.63	3209.67	2727.71	2423.02	Up
Dcaf11	2644.41	0.48046	0.10664	4.5052	6.63E-06	6.82E-05	2413.92	2023.84	2186.68	3000.26	3045.58	3196.17	Up
Fitm1	59.6201	-2.6398	0.48085	-5.4898	4.02E-08	6.47E-07	93.8476	157.154	57.293	13.4773	15.8404	20.108	Down
Psme1	7994.35	0.54901	0.08958	6.12891	8.85E-10	1.97E-08	6607.07	6175.02	6691.82	9879.9	9675.3	8937.01	Up
Emc9	585.204	-0.4679	0.18196	-2.5712	0.01013	0.03864	664.674	750.317	622.584	495.55	583.982	394.117	Down
Psme2	4360.64	0.45228	0.0833	5.42941	5.65E-08	8.89E-07	3698.76	3604.97	3744.1	4952.39	5285.41	4878.21	Up
Irf9	3514.52	0.25172	0.0923	2.72721	0.00639	0.0265	3223.71	3275.33	3127.24	3922.93	3553.53	3984.4	Up
Tgm1	13.5828	2.74914	0.71803	3.82874	0.00013	0.00095	2.9025	3.83303	3.81953	21.771	19.0085	30.162	Up
Nop9	1205.8	0.26299	0.1028	2.55833	0.01052	0.03986	1083.6	1090.5	1114.35	1382.98	1333.76	1229.61	Up
Cideb	145.498	2.05826	0.2284	9.01161	2.03E-19	1.68E-17	56.1151	59.412	53.4734	213.563	268.23	222.194	Up
Ltb4r2	17.6491	1.48356	0.66284	2.2382	0.02521	0.08023	12.5775	6.70781	8.59395	31.1015	34.8488	12.0648	Up
Adcy4	5892.66	-0.4591	0.15291	-3.0023	0.00268	0.01277	6236.51	6176.94	8053.48	4625.83	5705.71	4557.48	Down
Ripk3	756.283	0.65459	0.14861	4.40481	1.06E-05	0.0001	584.371	527.042	651.23	877.062	1044.41	853.585	Up
Nynrin	1221.6	-0.4651	0.14777	-3.1472	0.00165	0.00848	1246.14	1355.94	1648.13	1030.5	1108.83	940.05	Down
Khnyn	1749.14	0.31252	0.11272	2.77253	0.00556	0.0237	1638.95	1421.1	1621.39	1873.35	1863.88	2076.15	Up
Cma1	85.5252	-1.2538	0.52526	-2.3869	0.01699	0.05871	56.1151	210.817	94.5334	41.4686	76.0338	34.1836	Down
Mcpt4	32.9292	-3.6604	0.98503	-3.716	0.0002	0.00141	14.5125	145.655	22.9172	10.3672	2.11205	2.0108	Down
Gzmb	207.576	-0.7576	0.30615	-2.4745	0.01334	0.04836	234.135	357.43	190.977	111.965	190.085	160.864	Down
Parp4	3149.08	0.29139	0.09435	3.08835	0.00201	0.01002	3007.96	2690.79	2797.81	3330.97	3460.6	3606.37	Up
Mphosph8	1844.31	0.38835	0.12136	3.19994	0.00137	0.00729	1644.75	1619.46	1528.77	2081.73	1859.66	2331.52	Up
Gm8983	15.4579	1.51469	0.71576	2.1162	0.03433	0.1017	9.67501	8.62433	5.7293	14.514	40.129	14.0756	Up
Gja3	51.4168	-5.1953	0.92256	-5.6314	1.79E-08	3.07E-07	56.1151	211.775	32.466	0	2.11205	6.03241	Down
Gjb2	657.723	1.19161	0.17174	6.93853	3.96E-12	1.29E-10	336.691	465.714	399.141	1004.58	940.919	799.294	Up
Gjb6	1349.91	0.8623	0.22274	3.87139	0.00011	0.00081	661.771	1054.08	1158.27	1853.65	1942.03	1429.68	Up
Cryl1	1321.57	0.40825	0.12425	3.28566	0.00102	0.00566	1291.61	1068.46	1047.51	1499.09	1478.44	1544.3	Up
Ift88	2789.37	-0.3828	0.17861	-2.143	0.03211	0.09655	2916.05	3436.32	3119.6	2584.53	1816.36	2863.38	Down
Rcbtb1	2748.87	0.29635	0.07978	3.71445	0.0002	0.00141	2420.69	2505.85	2476.01	3000.26	3077.26	3013.19	Up
Phf11	54.1172	1.1326	0.34872	3.24789	0.00116	0.00633	27.09	32.5808	42.0148	88.1208	66.5296	68.3673	Up
Phf11a	98.8635	0.63426	0.25224	2.51452	0.01192	0.04409	77.4001	84.3268	70.6613	106.782	139.395	114.616	Up
Phf11d	2187.76	0.51056	0.0839	6.08522	1.16E-09	2.55E-08	1826.64	1832.19	1755.07	2529.59	2605.21	2577.85	Up
Atp8a2	110.557	-1.1543	0.28911	-3.9927	6.53E-05	0.00052	114.165	193.568	149.917	73.6068	70.7537	61.3295	Down
Gm29266	177.294	-1.1216	0.28616	-3.9196	8.87E-05	0.00068	161.573	295.144	272.142	122.332	92.9302	119.643	Down
Mipep	1766.63	-0.411	0.15835	-2.5953	0.00945	0.03647	1818.9	2404.27	1826.69	1567.51	1329.54	1652.88	Down
Tnfrsf19	2246.77	-0.4377	0.1751	-2.4999	0.01242	0.04565	2262.02	2799.07	2693.72	1765.53	2369.72	1590.54	Down
Sgcg	32.7334	-3.0071	0.7012	-4.2884	1.80E-05	0.00017	35.7976	112.116	26.7367	9.33044	7.39218	5.027	Down
1700109G	39.5494	0.9796	0.4564	2.14638	0.03184	0.09594	39.6676	20.1234	20.0525	61.1662	58.0814	38.2052	Up
Gm35419	5.80989	5.0386	1.5526	3.24527	0.00117	0.00638	0	0.95826	0	15.5507	5.28013	13.0702	Up
Rnaseh2b	935.729	0.37219	0.17709	2.10173	0.03558	0.10455	1029.42	735.943	681.786	1028.42	1076.09	1062.71	Up
Fam124a	918.595	-0.3301	0.13603	-2.4268	0.01523	0.05373	929.769	1029.17	1110.53	798.271	901.846	741.986	Down
Ctsb	42943.9	0.9748	0.10052	9.69777	3.08E-22	3.26E-20	29902.6	30604.9	26383.4	55923.6	60356.1	54492.7	Up
Fdft1	4809.04	1.15333	0.13768	8.37677	5.44E-17	3.59E-15	2950.88	3388.4	2609.69	6856.84	7158.8	5889.64	Up
Neil2	141.326	0.58657	0.22392	2.61955	0.0088	0.03439	118.035	121.699	99.3078	174.168	145.732	189.015	Up
Gata4	215.011	-2.0686	0.52605	-3.9323	8.41E-05	0.00065	259.29	642.033	140.368	133.736	63.3615	51.2754	Down
Gm49390	7.53102	-4.3998	1.50033	-2.9326	0.00336	0.01552	7.74001	29.706	5.7293	0	0	2.0108	Down
Gm5463	5.77534	-3.9973	1.55397	-2.5723	0.0101	0.03855	7.74001	21.0817	3.81953	0	0	2.0108	Down
Sox7	5348.05	-0.2603	0.12435	-2.0933	0.03632	0.10633	4973.93	6171.19	6342.33	5036.37	4930.58	4633.89	Down
Gm48433	3.71642	-2.6321	1.29498	-2.0325	0.0421	0.11935	5.80501	7.66607	5.7293	1.03672	1.05603	1.0054	Down
Scara5	658.844	-2.2581	0.26794	-8.4279	3.52E-17	2.41E-15	981.046	1555.25	733.35	240.518	199.589	243.307	Down
Pbk	106.095	1.52521	0.36182	4.21539	2.49E-05	0.00022	52.2451	61.3286	50.6088	188.682	87.6501	196.053	Up
Esco2	80.1542	0.89693	0.34928	2.56795	0.01023	0.03895	54.1801	75.7024	38.1953	107.818	82.37	122.659	Up
Ccdc25	1952.5	0.57379	0.09184	6.24761	4.17E-10	9.82E-09	1647.65	1558.13	1502.03	2289.07	2385.56	2332.53	Up
Scara3	463.9	-1.7011	0.27109	-6.275	3.50E-10	8.35E-09	501.166	965.925	661.734	236.371	166.852	251.35	Down
Clu	28554.8	0.78602	0.20593	3.817	0.00014	0.00099	20384.3	24886.9	17617.6	45902.7	26935	35602.3	Up
Ephx2	514.791	-1.748	0.37442	-4.6687	3.03E-06	3.37E-05	546.638	1381.81	451.66	226.004	273.511	209.123	Down
Ptk2b	2024.16	0.86677	0.20319	4.26578	1.99E-05	0.00018	1793.75	1337.73	1169.73	2662.29	3096.27	2085.2	Up
Trim35	6470.59	-0.2101	0.10324	-2.0348	0.04187	0.11884	7028.9	6513.28	7280.03	6068.93	6423.8	5508.59	Down
Adra1a	1337.73	-1.5956	0.22436	-7.112	1.14E-12	4.07E-11	1632.17	2228.91	2169.49	755.766	783.571	456.452	Down
Gm10032	21.0416	-1.3766	0.5301	-2.5969	0.00941	0.03633	24.1875	31.6225	35.3307	15.5507	9.50423	10.054	Down
Dpysl2	6700.26	-0.4062	0.13257	-3.0641	0.00218	0.01072	6807.34	7415.01	8689.43	5566.13	6390.01	5333.65	Down
Cdca2	115.523	1.10472	0.25599	4.3155	1.59E-05	0.00015	77.4001	76.6607	65.8869	157.581	186.916	128.691	Up
Kctd9	1972.61	0.28118	0.09875	2.84744	0.00441	0.01949	1798.59	1845.61	1698.74	2039.22	2277.85	2175.69	Up
Dock5	1993.19	0.64241	0.12539	5.12322	3.00E-07	4.14E-06	1753.11	1482.43	1434.23	2455.98	2588.32	2245.06	Up
Gm31227	19.3486	2.1682	0.55483	3.90784	9.31E-05	0.00071	7.74001	6.70781	6.68418	36.2851	28.5127	30.162	Up
Adam7	280.188	1.45657	0.26271	5.54443	2.95E-08	4.88E-07	202.208	117.866	128.909	334.859	492.108	405.177	Up
Adam28	1786.75	0.48081	0.182	2.64182	0.00825	0.03262	1423.19	1741.16	1311.05	2115.94	1656.9	2472.28	Up
Slc25a37	3702.04	-0.4863	0.08814	-5.5178	3.43E-08	5.60E-07	4533.71	4068.77	4358.09	3053.13	3130.06	3068.48	Down

Rhobtb2	995.368	-0.3312	0.10721	-3.089	0.00201	0.01001	1060.38	1109.66	1157.32	920.604	838.484	885.758	Down
Egr3	221.418	0.74932	0.2618	2.86222	0.00421	0.01875	151.898	208.9	134.638	270.583	345.32	217.167	Up
Bin3	1638.6	0.21302	0.09541	2.23253	0.02558	0.08108	1560.58	1442.18	1550.73	1812.18	1768.84	1697.12	Up
Pdlim2	7799.69	-0.4152	0.14071	-2.9506	0.00317	0.01478	9615.03	7868.26	9259.5	7584.61	6726.88	5743.86	Down
Sorbs3	15015.1	-0.8461	0.13023	-6.4975	8.17E-11	2.19E-09	17468.2	17764.2	22656.5	11241.1	10257.2	10703.5	Down
Gm49417	330.204	-0.5314	0.16597	-3.2017	0.00137	0.00726	374.423	369.888	426.833	283.023	235.494	291.566	Down
Phyhip	52.9764	-1.2519	0.41525	-3.0149	0.00257	0.01234	80.3026	91.0346	52.5186	44.5788	24.2886	25.135	Down
Bmp1	7760.24	1.17561	0.11164	10.5304	6.26E-26	9.12E-24	4442.77	5065.36	4779.19	10514.4	11867.6	9892.14	Up
Sftpc	1018493	1.10725	0.15674	7.06419	1.62E-12	5.64E-11	762454	510364	664492	1498180	1332686	1342780	Up
Lgi3	11855.9	0.72427	0.1641	4.41362	1.02E-05	9.94E-05	11054.7	7066.2	8701.85	15143.3	14673.5	14495.9	Up
Reep4	2251.3	0.5677	0.12257	4.63181	3.62E-06	3.96E-05	1683.45	1852.31	1905.95	2728.64	2952.65	2384.81	Up
Hr	366.602	-0.6233	0.29094	-2.1423	0.03217	0.09663	276.705	533.75	523.276	213.563	331.592	320.723	Down
Nudt18	1348.66	0.26308	0.10672	2.46524	0.01369	0.0494	1254.85	1233.28	1189.78	1514.64	1548.13	1351.26	Up
Fam160b2	1274.47	0.31641	0.11144	2.83916	0.00452	0.01995	1232.6	1066.54	1106.71	1486.65	1373.89	1380.42	Up
Dmtn	916.992	-0.7653	0.10868	-7.0417	1.90E-12	6.54E-11	1117.46	1137.45	1208.88	694.6	699.089	644.462	Down
Gfra2	920.929	-0.7304	0.18311	-3.9889	6.64E-05	0.00053	905.581	1330.06	1211.75	706.003	771.955	600.224	Down
Fndc3a	5242.99	0.48172	0.08587	5.61007	2.02E-08	3.44E-07	4514.36	4314.08	4298.88	5904.1	5971.82	6454.67	Up
Rcbtb2	1265.89	0.56303	0.14325	3.9304	8.48E-05	0.00066	1178.42	1026.3	861.304	1486.65	1475.27	1567.42	Up
Rb1	413.616	0.40766	0.19066	2.13818	0.0325	0.09732	445.051	294.185	327.525	468.596	466.763	479.576	Up
Sucla2	4850.65	-0.4613	0.13749	-3.3553	0.00079	0.00458	5104.54	6677.15	5077.11	3917.75	4270.57	4056.79	Down
Lrch1	3369.31	0.28256	0.13544	2.08623	0.03696	0.10779	3539.12	2686	2896.16	3448.12	3930.53	3715.96	Up
Lcp1	16178	1.45182	0.17966	8.08094	6.43E-16	3.71E-14	10810.9	7928.63	7245.65	20589.2	27544.3	22949.3	Up
Gtf2f2	1188.24	0.28412	0.11926	2.38228	0.01721	0.05922	1146.49	1074.21	994.033	1365.35	1355.94	1193.41	Up
Tsc22d1	21513.5	-0.4263	0.11974	-3.56	0.00037	0.00239	22256.4	27528.9	24221.6	20061.5	17668.4	17344.2	Down
Serp2	72.2791	-1.06	0.31018	-3.4175	0.00063	0.00378	113.198	99.6589	80.2102	39.3952	55.9693	45.243	Down
Lacc1	873.042	1.24959	0.16543	7.55365	4.23E-14	1.90E-12	508.906	598.912	443.066	1069.89	1246.11	1371.37	Up
Ccdc122	637.463	1.26382	0.18094	6.98484	2.85E-12	9.51E-11	369.586	448.465	306.517	968.293	788.851	943.066	Up
Epsti1	1652.38	1.58118	0.15787	10.0156	1.30E-23	1.54E-21	822.376	983.173	677.967	2565.87	2281.01	2583.88	Up
Tnfsf11	29.0261	1.09207	0.4999	2.1846	0.02892	0.08943	9.67501	25.873	20.0525	43.5421	33.7928	41.2214	Up
Dgkh	4027.47	-0.5546	0.16414	-3.3785	0.00073	0.00427	3853.56	5179.39	5343.52	2804.32	3366.61	3617.43	Down
Vwa8	2425.38	-0.2372	0.12001	-1.9763	0.04812	0.13245	2738.03	2832.61	2302.22	2351.27	2199.7	2128.43	Down
Rgcc	9104.69	-0.9385	0.20588	-4.5586	5.15E-06	5.43E-05	12817.5	11618.9	11461.5	7724.57	6787.08	4218.66	Down
Elf1	5792.06	0.20158	0.07391	2.72742	0.00638	0.0265	5376.41	5317.38	5470.52	6197.49	6082.71	6307.89	Up
Olfm4	243.126	3.88683	0.47081	8.25559	1.51E-16	9.34E-15	49.3426	27.7895	15.2781	713.261	221.765	431.317	Up
Pcdh17	1308.99	0.36997	0.16135	2.29301	0.02185	0.07149	1357.4	954.426	1114.35	1374.69	1629.45	1423.65	Up
Rps3a2	7125.98	-0.8593	0.22088	-3.8906	1.00E-04	0.00076	9122.57	7874.97	10565.8	4010.02	4204.04	6978.49	Down
Gm33203	3.08248	5.12607	1.85236	2.76732	0.00565	0.02401	0	0	0	3.11015	6.33615	9.04861	Up
Pcdh9	49.8068	-1.2643	0.51195	-2.4695	0.01353	0.04893	36.7651	120.741	53.4734	25.9179	33.7928	28.1512	Down
Klhl1	35.1195	2.65623	0.71898	3.69443	0.00022	0.00151	15.48	8.62433	4.77441	68.4232	16.8964	96.5185	Up
Dach1	2156.98	-0.3164	0.12172	-2.5996	0.00933	0.03611	2256.21	2389.9	2531.39	2135.63	1913.52	1715.21	Down
Bora	440.562	-0.4205	0.13629	-3.085	0.00204	0.01011	482.783	523.209	507.043	352.483	370.665	407.187	Down
Gm41230	259.789	-4.1793	0.24194	-17.274	7.39E-67	1.99E-63	440.213	544.291	492.72	32.1382	23.2326	26.1404	Down
Kctd12	6048.28	0.21807	0.08317	2.6221	0.00874	0.03418	5418.98	5584.73	5772.27	6252.43	6775.46	6485.84	Up
Cln5	3752.59	0.47267	0.13652	3.46226	0.00054	0.00328	3662.96	2821.11	2945.81	4152.05	4753.17	4180.46	Up
Fbxl3	7427.22	-0.236	0.09215	-2.5614	0.01042	0.03958	7426.54	8279.35	8394.38	6588.33	6904.29	6970.44	Down
Gm34907	335.782	1.03532	0.27502	3.76457	0.00017	0.00119	303.795	193.568	163.285	388.768	568.142	397.133	Up
Ednrb	3643.77	-0.8839	0.1056	-8.3697	5.78E-17	3.80E-15	4808.48	4368.7	5001.68	2372.01	2689.7	2622.09	Down
Ndfip2	3940.92	0.28674	0.10401	2.75689	0.00584	0.02464	3449.14	3687.38	3514.92	4288.89	4727.83	3977.37	Up
9330188P	80.7583	-2.5293	0.50088	-5.0496	4.43E-07	5.90E-06	147.06	117.866	148.007	7.25701	42.241	22.1188	Down
Mir17hg	433.68	-0.947	0.19283	-4.9109	9.07E-07	1.13E-05	562.118	461.881	689.425	302.721	268.23	317.707	Down
Gpc6	76.7954	-1.2338	0.37548	-3.286	0.00102	0.00565	93.8476	121.699	107.902	42.5053	27.4567	67.3619	Down
Abcc4	1430.73	1.03246	0.17328	5.95829	2.55E-09	5.21E-08	929.769	1143.2	745.764	1747.9	2017.01	2000.75	Up
Cldn10	2038.56	-1.1925	0.27448	-4.3445	1.40E-05	0.00013	2747.7	2751.16	3009.79	1680.52	675.856	1366.34	Down
Dzip1	830.678	-0.7175	0.23001	-3.1196	0.00181	0.00917	1043.93	1015.75	1039.87	654.168	413.962	816.386	Down
Dnajc3	13523.3	0.30005	0.08202	3.65827	0.00025	0.00171	11856.7	11804.8	12704.7	14340.9	15316.6	15116.2	Up
Gm49768	92.9539	-0.6511	0.30105	-2.1629	0.03055	0.093	93.8476	141.822	105.037	89.1576	66.5296	61.3295	Down
Uggt2	411.044	-0.3821	0.14037	-2.7221	0.00649	0.0268	479.881	460.922	454.524	354.557	388.617	327.761	Down
Gm4681	14.5635	-2.2233	0.76106	-2.9213	0.00349	0.01602	19.35	38.3303	14.3232	3.11015	4.2241	8.04321	Down
Rap2a	6367.85	0.36315	0.12397	2.92931	0.0034	0.01567	6380.67	4951.32	5379.81	7093.21	7544.25	6857.84	Up
Gm49008	2.55758	-4.7515	1.90569	-2.4933	0.01266	0.04632	3.87001	4.79129	6.68418	0	0	0	Down
Stk24	3735.73	0.28257	0.11572	2.44178	0.01462	0.05198	3594.27	3174.71	3344	3894.94	4578.93	3827.56	Up
Slc15a1	192.999	0.94972	0.24582	3.86355	0.00011	0.00084	172.215	101.575	121.27	231.188	270.342	261.404	Up
Gm49035	23.1402	-1.9487	0.71508	-2.7252	0.00643	0.02662	29.025	48.8712	32.466	4.14686	4.2241	20.108	Down
Dock9	11430.9	-0.2436	0.09865	-2.4692	0.01354	0.04897	11885.8	11708	13587	10702	10071.3	10631.1	Down
1810041H	39.7118	-0.8358	0.40938	-2.0416	0.04119	0.11721	60.9526	34.4973	57.293	26.9546	26.4006	32.1728	Down
Timm8a2	2.5722	-3.6975	1.80434	-2.0492	0.04044	0.11552	4.83751	2.87478	6.68418	1.03672	0	0	Down
Pcca	1578.76	-0.3069	0.11677	-2.6282	0.00858	0.03368	1731.83	1928.02	1578.42	1418.23	1362.27	1453.81	Down
Fgf14	120.849	-1.1577	0.34019	-3.4031	0.00067	0.00396	122.873	196.443	181.428	97.4513	46.4651	80.4321	Down
Ccdc152	1119.87	-0.5057	0.12101	-4.179	2.93E-05	0.00026	1265.49	1460.39	1216.52	961.036	924.022	891.791	Down
Ghr	6056.54	-0.3935	0.1237	-3.1811	0.00147	0.0077	6247.16	7788.73	6596.33	4902.63	5669.8	5134.58	Down
Fbxo4	1836.71	0.64552	0.11277	5.72398	1.04E-08	1.87E-07	1490.92	1481.47	1325.38	2269.37	2059.25	2393.86	Up
Plcxd3	55.273	-1.1641	0.35446	-3.2841	0.00102	0.00568	56.1151	88.1598	84.9846	32.1382	39.0729	31.1674	Down
C6	9474.43	1.49512	0.11958	12.5029	7.20E-36	2.46E-33	4854.92	5336.54	4694.2	12211.5	14882.6	14866.9	Up
C7	8873.68	0.31275	0.1247	2.50809	0.01214	0.0448	8654.3	7533.83	7558.85	8610.96	10023.8	10860.3	Up
Rpl37	4240.03	-0.412	0.14519	-2.8373	0.00455	0.02003	5281.59	4027.56	5214.62	3739.43	3930.53	3246.44	Down
Prkaa1	198.072	0.49823	0.20396	2.44283	0.01457	0.05187	180.923	137.031	174.744	218.747	218.597	258.388	Up
Ptger4	1058.73	0.42988	0.13837	3.10678	0.00189	0.00952	923.964	954.426	827.883	1243.02	1345.38	1057.68	Up
5430437J1	146.296	1.12539	0.29124	3.86415	0.00011	0.00083	120.938	94.8676	60.1576	196.976	181.636	223.199	Up
Dab2	5934.12	1.37815	0.19362	7.11794	1.10E-12	3.91E-11	4346.98	3007.02	2538.08	8317.57	9241.28	8153.8	Up
Osmr	5402.82	0.63802	0.1613	3.95544	7.64E-05	0.0006	3776.16	4202.92	4702.8	5750.66	5961.26	8023.1	Up
Lifr	7300.33	-0.5192	0.14389	-3.6085	0.00031	0.00203	10270	8053.21	7476.73	6100.04	6334.04	5567.91	Down
Egflam	3360.32	-0.4823	0.13536	-3.5631	0.00037	0.00236	3615.55	3786.08	4348.54	2863.41	3115.27	2433.07	Down
Cplane1	1764.51	-0.2349	0.10714	-2.1925	0.02834	0.08806	1932.1	1851.36	1940.32	1526.05	1558.69	1778.55	Down

Slc1a3	2459.28	0.69342	0.1883	3.68244	0.00023	0.00157	2152.69	1632.87	1852.47	2679.91	3861.88	2575.84	Up
Lmbrd2	3087.37	0.37869	0.0886	4.27408	1.92E-05	0.00017	2623.86	2686	2743.38	3424.27	3688.7	3358.04	Up
Capsl	2094.97	0.67371	0.16499	4.08325	4.44E-05	0.00037	1588.64	1853.27	1401.77	2830.23	2109.94	2785.97	Up
Rad1	763.683	-0.3567	0.13071	-2.7292	0.00635	0.02638	902.679	795.355	874.673	640.69	741.33	627.37	Down
C1qtnf3	109.37	-0.5068	0.24614	-2.0588	0.03951	0.11342	148.028	114.033	123.18	88.1208	81.314	101.545	Down
Adamts12	131.719	-1.1316	0.34962	-3.2366	0.00121	0.00654	99.6527	246.272	196.706	83.974	80.2579	83.4483	Down
Npr3	6344.99	-2.0505	0.43812	-4.6804	2.86E-06	3.20E-05	5149.04	10237.1	15281	2025.74	2898.79	2478.31	Down
Mtmr12	3372.37	-0.2478	0.0949	-2.6111	0.00903	0.03506	3632.97	3879.03	3471.95	3119.48	2932.58	3198.18	Down
Golph3	9629.82	0.22344	0.0717	3.11614	0.00183	0.00926	8777.17	8809.27	9070.43	10307	10260.3	10554.7	Up
Pdzd2	16599.1	-0.4634	0.15875	-2.9188	0.00351	0.01612	17540.8	18100.5	22084.5	11887	16668.3	13313.5	Down
Otulinl	2103.67	0.51183	0.1148	4.45851	8.25E-06	8.29E-05	1761.82	1822.61	1618.53	2576.24	2617.89	2224.95	Up
Dnah5	4169.1	-0.7259	0.29508	-2.4602	0.01389	0.04997	4427.29	6679.06	4483.18	2927.69	1855.44	4641.94	Down
Dap	6172.41	0.65453	0.13328	4.91095	9.06E-07	1.13E-05	4871.37	4689.72	4825.98	7648.89	8660.46	6338.05	Up
Ankrd33b	4166.03	-0.6164	0.14361	-4.2924	1.77E-05	0.00016	4362.46	4990.61	5775.13	2911.1	3596.82	3360.05	Down
Ropn1l	264.092	-0.4646	0.19658	-2.3636	0.0181	0.0617	293.153	349.764	275.961	242.592	184.804	238.28	Down
Cmbl	1977.82	-0.4414	0.19077	-2.3138	0.02068	0.06843	2371.35	2409.06	2053.95	1923.11	1211.26	1898.2	Down
Snhg18	802.26	-0.4474	0.11437	-3.9116	9.17E-05	0.0007	915.256	941.968	919.552	709.114	703.313	624.354	Down
Sema5a	1517.64	-0.2321	0.09596	-2.4189	0.01557	0.05464	1605.08	1623.29	1690.14	1349.8	1368.61	1468.89	Down
Gm32618	196.307	-0.4373	0.1974	-2.2153	0.02674	0.08401	198.338	222.316	256.863	152.397	168.964	178.961	Down
Sdc2	4983.05	-0.6497	0.10225	-6.3536	2.10E-10	5.30E-09	5730.51	5940.25	6588.69	3728.03	3781.63	4129.18	Down
Mtdh	6559.42	0.34763	0.12957	2.68299	0.0073	0.02951	6420.34	5497.53	5400.82	7050.7	8318.31	6668.82	Up
Rpl30	5723.27	-0.3301	0.13037	-2.5324	0.01133	0.04234	6761.87	5661.39	6702.32	5353.6	5441.7	4418.74	Down
Erich5	21.8301	-1.3984	0.55005	-2.5424	0.01101	0.04136	23.22	44.0799	27.6916	11.4039	9.50423	15.081	Down
Stk3	2328.78	0.31852	0.102	3.12262	0.00179	0.0091	1934.04	2058.34	2225.83	2463.24	2639.01	2652.25	Up
Gm33497	5.97952	3.05974	1.26116	2.42613	0.01526	0.05379	0.9675	2.87478	0	18.6609	6.33615	7.03781	Up
Osr2	54.903	1.4296	0.38224	3.74009	0.00018	0.0013	17.415	38.3303	33.4209	77.7537	76.0338	86.4645	Up
Vps13b	1239.16	0.45522	0.12187	3.73541	0.00019	0.00132	1137.78	989.881	1008.36	1388.16	1345.38	1565.41	Up
Rnf19a	4566.88	0.33055	0.12376	2.67089	0.00756	0.03039	4220.24	3491.89	4425.88	4824.88	5040.41	5398	Up
Ankrd46	6293.92	0.20101	0.10149	1.9806	0.04764	0.13145	6335.2	5416.08	5817.15	6830.92	6991.94	6372.23	Up
Gm46515	22.7009	-1.2003	0.5624	-2.1343	0.03282	0.09812	16.4475	41.2051	37.2404	14.514	13.7283	13.0702	Down
Ywhaz	21966.3	0.23193	0.08967	2.58657	0.00969	0.03725	18819.8	20330.4	21462.9	22990.2	24376.2	23817.9	Up
Gm10384	48.1607	0.92339	0.39792	2.32058	0.02031	0.06752	23.22	31.6225	44.8795	76.717	61.2495	51.2754	Up
Gm34590	32.5367	-0.9776	0.47636	-2.0522	0.04015	0.11486	35.7976	45.0382	48.699	25.9179	11.6163	28.1512	Down
Grhl2	1862.1	0.39145	0.1318	2.96993	0.00298	0.01399	1572.19	1661.62	1599.43	2272.48	1783.63	2283.27	Up
Ncald	2466.72	-0.331	0.14648	-2.2596	0.02385	0.07671	2519.37	3035.76	2689.91	2128.38	2540.8	1886.13	Down
Ubr5	6428.39	0.2089	0.08755	2.38603	0.01703	0.05881	6005.28	5938.33	5947.97	6530.27	6783.91	7364.56	Up
Gm41307	200.529	1.82838	0.26106	7.00361	2.49E-12	8.41E-11	113.198	83.3685	67.7967	289.244	379.113	270.453	Up
G930009F	229.553	0.9022	0.19809	4.55441	5.25E-06	5.52E-05	148.995	163.862	167.105	298.574	347.432	251.35	Up
Azin1	15898.8	1.07499	0.12831	8.37819	5.37E-17	3.56E-15	9419.59	10121.1	11164.5	18875.5	24199.9	21612.1	Up
Atp6v1c1	4341.42	0.42957	0.11686	3.67593	0.00024	0.00161	3733.59	3699.84	3665.8	4859.09	5642.34	4447.89	Up
Gm9522	51.608	-1.5173	0.5031	-3.0159	0.00256	0.0123	69.6601	67.0781	92.6236	27.9913	42.241	10.054	Down
Dcstamp	66.6322	2.46133	0.70161	3.50812	0.00045	0.00284	24.1875	9.58259	27.6916	54.9459	39.0729	244.312	Up
Lrp12	1469.34	1.92193	0.27337	7.0304	2.06E-12	7.05E-11	864.946	529.917	445.93	2207.17	3029.74	1738.34	Up
AU022793	163.388	1.98117	0.32481	6.0994	1.06E-09	2.35E-08	94.8151	55.579	47.7441	232.224	335.816	214.15	Up
Gm33251	6.87281	3.73236	1.19284	3.12897	0.00175	0.00892	1.935	0	0.95488	14.514	14.7844	9.04861	Up
Zfpm2	530.106	-0.4278	0.17013	-2.5148	0.01191	0.04407	577.598	703.362	543.328	502.807	460.427	393.112	Down
Abra	135.082	-2.6281	0.92596	-2.8382	0.00454	0.02	147.06	416.843	133.684	42.5053	63.3615	7.03781	Down
Angpt1	4905.13	-0.7245	0.10254	-7.065	1.61E-12	5.62E-11	5995.61	6368.59	5969.93	3573.56	4062.53	3460.59	Down
Gm17473	60.4127	-1.1429	0.50036	-2.2841	0.02237	0.0728	71.5951	98.7006	79.2553	13.4773	40.129	59.3187	Down
Nudcd1	1303.4	0.26268	0.11098	2.36693	0.01794	0.0613	1226.79	1227.53	1100.98	1511.53	1337.98	1415.6	Up
Ebag9	1606.28	0.28302	0.10631	2.66216	0.00776	0.03105	1405.78	1572.5	1369.3	1814.25	1785.74	1690.08	Up
Sybu	275.044	-1.1084	0.18952	-5.8486	4.96E-09	9.63E-08	362.813	434.091	330.389	189.719	177.412	155.837	Down
A930017N	84.2234	-1.8322	0.58982	-3.1065	0.00189	0.00953	35.7976	204.109	154.691	36.2851	22.1765	52.2808	Down
Aard	530.976	-0.9743	0.14945	-6.5195	7.05E-11	1.91E-09	613.396	742.65	755.312	356.63	335.816	382.052	Down
Med30	1446.1	-0.2468	0.11628	-2.1229	0.03376	0.1003	1607.02	1589.75	1511.58	1421.34	1368.61	1178.33	Down
Ext1	3265.96	0.41027	0.10011	4.09805	4.17E-05	0.00035	2704.17	2818.24	2891.39	3700.04	4021.34	3460.59	Up
Tnfrsf11b	244.056	0.97132	0.21115	4.60001	4.22E-06	4.55E-05	178.02	134.156	182.383	368.034	325.256	276.485	Up
Ccn3	672.472	-2.0423	0.25656	-7.9604	1.72E-15	9.23E-14	697.569	1472.84	1076.15	272.656	269.286	246.323	Down
Enpp2	3478.27	-0.2754	0.12364	-2.227	0.02595	0.08204	4217.34	3461.23	3748.87	2986.78	3475.38	2980.01	Down
Col14a1	1628.72	-0.3524	0.12148	-2.9011	0.00372	0.01688	1895.34	1960.6	1624.26	1438.96	1339.04	1514.13	Down
Mrpl13	1728.14	-0.3357	0.11073	-3.0318	0.00243	0.01176	1732.8	2035.34	2016.71	1578.92	1508	1497.04	Down
Slc22a22	101.832	-1.3623	0.27345	-4.9818	6.30E-07	8.17E-06	168.345	124.574	147.052	47.6889	55.9693	67.3619	Down
Derl1	8804.79	0.29314	0.10151	2.88781	0.00388	0.01751	7862.88	7852.93	8023.88	9753.42	10564.5	8771.12	Up
Tbc1d31	553.789	0.37412	0.17984	2.08027	0.0375	0.10896	517.613	438.882	490.81	693.563	493.164	688.7	Up
Atad2	1199.77	0.58523	0.16187	3.61549	0.0003	0.00198	1181.32	858.6	839.342	1443.11	1380.23	1496.04	Up
Wdyhv1	2306.08	-0.3256	0.13819	-2.3561	0.01847	0.06275	2430.36	2790.45	2475.06	2130.45	1732.94	2277.23	Down
Fam91a1	6316.35	0.51262	0.09816	5.22254	1.76E-07	2.52E-06	5383.18	4858.37	5375.99	6904.53	7705.82	7670.2	Up
Gm20712	166.193	-0.5661	0.20857	-2.7142	0.00664	0.0273	173.183	224.233	197.661	130.626	146.788	124.67	Down
Mtss1	4605.64	0.69352	0.10288	6.74137	1.57E-11	4.77E-10	3838.08	3507.23	3213.18	5542.28	5853.55	5679.51	Up
Gm36617	20.7264	-1.2908	0.64996	-1.9859	0.04704	0.13011	22.2525	41.2051	24.827	4.14686	15.8404	16.0864	Down
Sqle	6122.4	1.09751	0.13347	8.22262	1.99E-16	1.21E-14	4210.57	4266.17	3222.73	8072.91	8597.1	8364.94	Up
Washc5	3220.65	0.36141	0.10776	3.35374	0.0008	0.0046	3033.12	2738.7	2686.09	3547.64	3923.13	3395.24	Up
Trib1	2630.88	0.29971	0.14707	2.03795	0.04155	0.11811	2520.34	2283.53	2271.67	2935.98	3371.89	2401.9	Up
Gm2682	371.417	-0.6868	0.18498	-3.7128	0.0002	0.00142	423.766	515.543	435.427	247.775	267.174	338.82	Down
Myc	683.135	0.72465	0.19933	3.63535	0.00028	0.00185	537.931	478.171	529.005	809.675	1083.48	660.548	Up
Pvt1	216.731	0.58692	0.18403	3.18925	0.00143	0.00752	200.273	156.196	163.285	261.252	264.006	255.372	Up
Fam49b	4367.39	0.70449	0.11075	6.36119	2.00E-10	5.07E-09	3389.16	3435.36	3140.61	5048.81	6025.68	5164.74	Up
Adcy8	1580.36	-0.6225	0.16791	-3.7075	0.00021	0.00145	1734.73	1913.64	2099.79	1092.7	1533.35	1107.95	Down
Gm21798	14.9731	-2.9396	0.94386	-3.1144	0.00184	0.0093	4.83751	39.2886	35.3307	4.14686	4.2241	2.0108	Down
Efr3a	4261.62	0.23849	0.0951	2.50782	0.01215	0.04482	3856.46	3867.53	4006.69	4296.15	4585.26	4957.63	Up
Oc90	8.82646	2.56047	0.92924	2.75545	0.00586	0.02473	1.935	3.83303	1.90977	22.8077	7.39218	15.081	Up
Lrrc6	1071.08	-0.7386	0.23262	-3.1752	0.0015	0.00783	1322.57	1440.26	1255.67	881.208	515.34	1011.43	Down
Ccn4	142.555	0.94061	0.24593	3.82464	0.00013	0.00096	109.328	101.575	82.1199	150.324	204.869	207.113	Up

Ndrg1	556.126	-0.2918	0.13114	-2.2253	0.02606	0.0823	608.558	592.204	635.952	471.706	478.379	549.954	Down
St3gal1	8752.07	0.77364	0.1348	5.73928	9.51E-09	1.74E-07	6810.24	5991.99	6578.19	9310.75	11331.2	12490.1	Up
Khdrbs3	1291.55	-0.3227	0.13544	-2.3825	0.0172	0.0592	1277.1	1649.16	1379.81	1097.88	1211.26	1134.09	Down
Gm2999	92.0332	1.0179	0.2588	3.93319	8.38E-05	0.00065	72.5626	55.579	54.4283	118.186	127.779	123.664	Up
Dennd3	5754.64	-0.5317	0.16271	-3.2677	0.00108	0.00597	5354.15	6960.79	8094.54	4831.1	4956.98	4330.26	Down
Ptp4a3	7195.59	-0.5135	0.15632	-3.2848	0.00102	0.00567	9866.58	7273.18	8248.28	6194.38	6561.09	5030.02	Down
Mroh5	19.5467	-1.6648	0.83067	-2.0041	0.04505	0.12572	16.4475	25.873	46.7893	2.07343	20.0645	6.03241	Down
Them6	938.855	-0.5394	0.17392	-3.1018	0.00192	0.00965	1308.06	1011.92	1016.95	823.152	841.652	631.392	Down
Lypd2	1415.23	-2.7601	0.83245	-3.3156	0.00091	0.00517	1503.5	3869.45	2026.26	258.142	116.163	717.856	Down
Slurp2	80.9586	-4.1611	0.42649	-9.7566	1.73E-22	1.89E-20	175.118	144.697	140.368	9.33044	3.16808	13.0702	Down
Lynx1	1903.8	-0.7519	0.15809	-4.7559	1.98E-06	2.28E-05	2271.69	2855.61	2039.63	1554.04	1286.24	1415.6	Down
Ly6d	729.938	0.55364	0.21534	2.57104	0.01014	0.03865	794.319	471.463	508.953	874.988	935.638	794.267	Up
Gm17189	396.283	0.43889	0.21615	2.03051	0.0423	0.11975	267.03	412.051	330.389	450.971	384.393	532.862	Up
Ly6m	132.232	-1.6309	0.31829	-5.1238	2.99E-07	4.13E-06	154.8	280.77	164.24	59.0928	58.0814	76.4105	Down
Ly6e	62236.4	0.77656	0.09327	8.32557	8.39E-17	5.40E-15	46926.7	45145.5	45566.1	83412.1	80605.4	71762.5	Up
Ly6i	3807.19	5.88486	0.28297	20.7964	4.66E-96	5.03E-92	119.003	128.407	132.729	9524.31	4030.85	8907.85	Up
Ly6c2	2283.23	2.33086	0.17466	13.3451	1.26E-40	6.65E-38	863.979	824.102	583.433	3954.03	4145.96	3327.88	Up
Ly6g2	119.375	6.06208	0.51006	11.8851	1.41E-32	3.47E-30	1.935	5.74955	2.86465	265.399	250.278	190.021	Up
9030619P	3.20064	5.181	2.48007	2.08905	0.0367	0.10723	0	0	0	0	2.11205	17.0918	Up
Gpihbp1	7662.68	-0.822	0.18384	-4.4716	7.76E-06	7.86E-05	8306.97	9041.17	12017.2	6007.77	6166.13	4436.83	Down
2810039B	206.81	-0.4022	0.18421	-2.1833	0.02902	0.08965	210.915	230.94	264.503	172.095	177.412	184.994	Down
Rhpn1	480.793	0.619	0.24371	2.53988	0.01109	0.0416	360.878	403.427	373.359	768.206	390.729	588.16	Up
Naprt	872.111	0.65693	0.13055	5.0322	4.85E-07	6.39E-06	745.944	670.781	613.99	1027.39	1151.07	1023.5	Up
Eef1d	5805.86	-0.2437	0.08842	-2.7562	0.00585	0.02469	6376.8	6047.57	6460.74	5641.81	5177.69	5130.56	Down
Tsta3	1790.75	0.38689	0.10036	3.85498	0.00012	0.00086	1606.05	1430.68	1619.48	2074.47	1998	2015.83	Up
Zfp707	842.419	-0.2617	0.12773	-2.0491	0.04045	0.11555	864.946	862.433	1028.41	796.198	764.562	737.964	Down
Mapk15	4521.23	-0.5177	0.2078	-2.4914	0.01272	0.04652	4394.39	5762.01	5815.24	4610.28	2669.63	3875.82	Down
Fam83h	1251.08	0.44895	0.09733	4.61267	3.98E-06	4.30E-05	1021.68	1057.92	1094.3	1495.98	1412.96	1423.65	Up
Nrbp2	3070.62	0.48613	0.11398	4.26491	2.00E-05	0.00018	2526.15	2313.24	2835.05	3461.59	3513.4	3774.27	Up
Gm41349	156.151	-0.6969	0.23643	-2.9475	0.0032	0.01491	161.573	189.735	228.217	114.039	105.603	137.74	Down
Eppk1	820.315	-0.4677	0.20753	-2.2536	0.02422	0.07765	724.659	938.135	1193.6	587.818	707.537	770.137	Down
BC024139	67.1299	0.83088	0.29057	2.85947	0.00424	0.01888	48.3751	41.2051	55.3832	85.0107	81.314	91.4915	Up
Parp10	2079.96	0.30552	0.10551	2.89566	0.00378	0.01715	1881.79	1751.7	1947.96	2226.87	2481.66	2189.76	Up
Grina	9553.63	0.47706	0.13365	3.56954	0.00036	0.00232	9119.67	7742.73	7102.42	11163.4	12172.8	10020.8	Up
Oplah	3329.09	0.46651	0.14191	3.28727	0.00101	0.00564	3295.31	2681.21	2410.12	3824.44	3644.34	4119.13	Up
Gpaa1	2692.57	0.2973	0.09621	3.09022	0.002	0.00997	2385.86	2445.48	2416.81	3108.07	3063.53	2735.7	Up
Sharpin	2754.26	0.35504	0.11792	3.01087	0.0026	0.01248	2461.32	2420.56	2369.06	3312.31	3277.9	2684.42	Up
Mroh1	4896.18	0.73666	0.10277	7.16794	7.61E-13	2.77E-11	3895.16	3553.22	3569.35	6276.28	6484	5599.08	Up
Hsf1	2848.61	-0.2754	0.10533	-2.6151	0.00892	0.03474	3100.84	2922.69	3335.41	2629.11	2709.76	2393.86	Down
Slc39a4	17606.4	1.74771	0.21589	8.09545	5.71E-16	3.33E-14	8997.76	7916.17	7324.91	32171.4	17825.7	31402.7	Up
Tonsl	383.247	0.70316	0.15202	4.62532	3.74E-06	4.08E-05	312.503	275.978	286.465	523.542	464.651	436.344	Up
Cyhr1	7148.63	0.20269	0.08161	2.48356	0.01301	0.04738	6679.63	6680.02	6582.01	7913.25	7804.03	7232.85	Up
Ppp1r16a	4801	0.38473	0.10456	3.67951	0.00023	0.00159	3762.61	4241.25	4489.86	5665.65	5336.1	5310.53	Up
Gpt	3255.61	0.50792	0.16457	3.08624	0.00203	0.01008	3061.17	2606.46	2397.71	3908.42	3109.99	4449.9	Up
1110038F	1632.71	-0.3165	0.12716	-2.4886	0.01282	0.04684	1872.12	1646.29	1914.54	1423.41	1616.77	1323.11	Down
Mb	5193.38	-6.3364	1.4172	-4.471	7.78E-06	7.87E-05	5041.65	21636.5	4101.22	87.0841	4.2241	289.555	Down
Apol6	182.959	-2.3828	0.49908	-4.7745	1.80E-06	2.10E-05	179.955	570.164	170.924	55.9827	85.5381	35.189	Down
Apol7a	237.618	3.65614	0.36975	9.88817	4.69E-23	5.26E-21	46.4401	38.3303	20.0525	594.038	271.399	455.447	Up
Apol9a	154.695	1.82097	0.41999	4.33574	1.45E-05	0.00014	82.2376	92.9511	29.6014	297.537	158.404	267.437	Up
Apol9b	275.627	0.9058	0.26168	3.46146	0.00054	0.00329	214.785	230.94	129.864	385.658	289.351	403.166	Up
Myh9	22774	0.43172	0.12116	3.56318	0.00037	0.00236	18916.6	17430.7	21827.7	24229.1	28154.7	26085.1	Up
Txn2	4280.45	-0.2761	0.13113	-2.1054	0.03525	0.10388	4085.76	5409.37	4571.02	3955.07	4012.9	3648.6	Down
Ift27	1485.8	-0.4279	0.12074	-3.544	0.00039	0.00252	1612.82	1755.53	1745.53	1291.75	1125.72	1383.43	Down
Ncf4	875.775	1.12443	0.15256	7.37061	1.70E-13	6.98E-12	604.688	561.54	486.035	1155.94	1369.66	1076.78	Up
Csf2rb2	1216.76	2.45349	0.24085	10.1867	2.27E-24	2.85E-22	528.256	333.474	265.457	1785.22	2108.88	2279.24	Up
Csf2rb	3563.28	2.26307	0.21125	10.7127	8.87E-27	1.42E-24	1605.08	1186.32	894.725	5348.42	6853.6	5491.5	Up
Il2rb	497.282	-0.5194	0.1764	-2.9444	0.00324	0.01502	542.768	644.908	570.065	409.503	336.872	479.576	Down
C1qtnf6	650.726	-0.3112	0.12316	-2.5271	0.0115	0.04291	684.991	705.278	771.545	608.552	585.038	548.949	Down
Gm49490	25.1392	-3.0232	0.6171	-4.8991	9.63E-07	1.19E-05	29.9925	66.1198	38.1953	6.2203	5.28013	5.027	Down
Sstr3	32.6203	-3.5521	0.89289	-3.9782	6.94E-05	0.00055	34.8301	117.866	27.6916	0	5.28013	10.054	Down
Rac2	2994.76	0.60462	0.15183	3.98216	6.83E-05	0.00054	2877.35	2247.12	2004.3	3643.02	3756.28	3440.48	Up
Cyth4	2231.8	1.15846	0.15584	7.43345	1.06E-13	4.46E-12	1612.82	1260.11	1269.99	2794.99	3570.42	2882.48	Up
Elfn2	5.16612	4.86238	1.68806	2.88044	0.00397	0.01786	0.9675	0	0	2.07343	16.8964	11.0594	Up
Mfng	1746.15	-0.5189	0.13173	-3.9395	8.16E-05	0.00063	1910.82	1870.52	2389.12	1436.89	1471.04	1398.51	Down
Card10	2896.19	-0.3671	0.1183	-3.1029	0.00192	0.00962	2943.14	3277.24	3567.44	2393.78	2739.33	2456.19	Down
Cdc42ep1	7294.82	-0.3487	0.14409	-2.4204	0.01551	0.05448	8214.09	6969.42	9333.03	6656.75	6941.25	5654.37	Down
Gm26634	15.6568	-1.3256	0.61245	-2.1645	0.03043	0.09274	20.3175	16.2904	30.5563	9.33044	7.39218	10.054	Down
Triobp	983.5	0.27181	0.13861	1.96097	0.04988	0.13658	1009.1	777.148	887.086	1061.6	1141.56	1024.5	Up
H1f0	4853.63	0.56798	0.14333	3.96266	7.41E-05	0.00058	4553.06	3841.66	3336.36	5632.48	6356.22	5402.02	Up
Gcat	11.4311	2.79612	0.82274	3.39854	0.00068	0.00402	0.9675	3.83303	3.81953	14.514	25.3446	20.108	Up
Galr3	16.1224	3.53269	0.80876	4.36806	1.25E-05	0.00012	3.87001	3.83303	0	27.9913	35.9049	25.135	Up
Gm3924	70.2396	2.52407	0.32695	7.72002	1.16E-14	5.53E-13	18.3825	22.9982	21.0074	122.332	135.171	101.545	Up
Micall1	190.801	0.68577	0.20462	3.35146	0.0008	0.00464	163.508	126.49	148.962	220.82	218.597	266.431	Up
Gm10863	86.7787	0.76491	0.29435	2.59866	0.00936	0.03618	48.3751	77.619	66.8418	102.635	125.667	99.5347	Up
Baiap2l2	61.1914	-1.7948	0.48202	-3.7234	0.0002	0.00137	75.4651	142.781	66.8418	32.1382	13.7283	36.1944	Down
Pla2g6	1702.32	0.37808	0.11497	3.28856	0.00101	0.00562	1346.76	1635.75	1459.06	1994.64	1854.38	1923.33	Up
Tmem184	7102.38	-0.2378	0.1108	-2.1463	0.03185	0.09596	7130.49	7362.3	8566.25	6333.3	6919.08	6302.86	Down
Fam227a	1041.93	-0.4657	0.17409	-2.675	0.00747	0.03008	1071.99	1308.98	1245.17	902.98	695.921	1026.51	Down
Josd1	2396.03	0.25805	0.09363	2.75589	0.00585	0.02471	2321.04	2082.3	2143.71	2683.02	2548.19	2597.96	Up
Dnal4	1952.38	-0.2438	0.11535	-2.1139	0.03453	0.10218	2130.44	2183.87	2036.77	1965.61	1600.93	1796.65	Down
AC113595	176.786	1.12103	0.19754	5.67499	1.39E-08	2.44E-07	101.588	127.448	105.037	255.032	232.326	239.285	Up
Apobec3	8207.01	1.03104	0.08747	11.7872	4.55E-32	1.04E-29	5089.06	5769.68	5320.61	11019.3	11011.2	11032.3	Up
Cbx7	4652.63	-0.4828	0.10903	-4.4284	9.49E-06	9.35E-05	5882.41	4959.95	5429.46	4090.88	3932.64	3620.45	Down

Gm41361	86.8765	-1.0968	0.29812	-3.6789	0.00023	0.00159	121.905	89.1181	144.187	52.8725	53.8573	59.3187	Down
Mgat3	2967.35	-0.8762	0.26971	-3.2486	0.00116	0.00632	3094.07	4770.21	3661.02	2170.88	1276.73	2831.21	Down
Sgsm3	3388.72	0.3758	0.12418	3.0262	0.00248	0.01194	2811.56	3078.89	2959.18	4276.45	3324.37	3881.85	Up
Mrtfa	2013.67	0.44562	0.15005	2.96986	0.00298	0.01399	1774.4	1523.63	1817.14	2279.74	2703.42	1983.66	Up
Mchr1	12.0981	-1.4284	0.72264	-1.9766	0.04809	0.13239	27.09	11.4991	14.3232	7.25701	7.39218	5.027	Down
8430426J(	91.7154	-0.8028	0.27586	-2.9103	0.00361	0.01648	137.385	110.2	102.172	77.7537	65.4736	57.3078	Down
Slc25a17	5232.31	-0.2892	0.13724	-2.1072	0.0351	0.10356	5022.3	6558.32	5684.42	4761.64	4233.61	5133.58	Down
Chadl	107.867	-0.5661	0.27416	-2.0647	0.03896	0.11219	159.638	112.116	114.586	72.5701	89.7622	98.5293	Down
Rangap1	3383.79	0.26191	0.11839	2.21218	0.02695	0.08452	3275.96	2675.46	3280.98	3795.42	3751	3523.93	Up
Tob2	2864.98	-0.5584	0.12285	-4.5455	5.48E-06	5.74E-05	3336.91	3802.37	3098.59	2523.37	2240.89	2187.75	Down
Csdc2	294.656	-3.281	0.41053	-7.9921	1.33E-15	7.24E-14	389.903	897.888	315.111	53.9092	73.9218	37.1998	Down
Pmm1	1314.05	-0.6244	0.15042	-4.151	3.31E-05	0.00028	1879.86	1446.97	1455.24	1045.01	1115.16	942.061	Down
Snu13	4077.59	-0.2171	0.08964	-2.4218	0.01544	0.05432	4220.24	4317.91	4613.04	3778.83	3922.08	3613.41	Down
Srebf2	10576.2	0.45412	0.08379	5.41986	5.96E-08	9.27E-07	8812.97	8799.69	9163.06	11900.5	12964.8	11816.5	Up
Septin3	300.416	1.17419	0.21637	5.42678	5.74E-08	8.99E-07	224.46	167.695	161.375	337.969	463.595	447.403	Up
Wbp2nl	35.7384	1.25672	0.46645	2.69419	0.00706	0.02873	12.5775	22.9982	27.6916	52.8725	36.9609	61.3295	Up
Naga	6958.03	0.79615	0.08656	9.19725	3.67E-20	3.27E-18	5292.23	5083.56	4880.41	9064.01	9046.97	8381.02	Up
Ndufa6	2693.61	-0.2389	0.1028	-2.3243	0.02011	0.06708	2928.63	2891.07	2928.63	2707.9	2415.13	2290.3	Down
Cyp2d22	3516.92	-0.9751	0.09627	-10.128	4.13E-24	5.06E-22	4513.39	4509.57	4963.48	2507.82	2317.98	2289.3	Down
Cyp2d10	75.7297	5.14845	1.90004	2.70966	0.00674	0.02762	0	12.4574	0	230.151	97.1543	114.616	Up
Cyp2d34	43.1274	8.93162	1.3105	6.81544	9.40E-12	2.96E-10	0	0	0	148.25	61.2495	49.2646	Up
Gm49431	2.78112	3.92772	1.95979	2.00415	0.04505	0.12572	0	0	0.95488	3.11015	11.6163	1.0054	Up
Nfam1	1689.29	1.41761	0.22319	6.35153	2.13E-10	5.35E-09	1160.03	893.097	707.568	2133.56	3132.17	2109.33	Up
A4galt	1943.86	-0.7547	0.11834	-6.3775	1.80E-10	4.59E-09	2148.82	2634.25	2539.99	1497.02	1401.35	1441.74	Down
1700001L(	496.29	0.41922	0.14064	2.9808	0.00288	0.01356	429.571	386.178	458.344	606.479	530.125	567.046	Up
Ttll12	1197.47	0.29589	0.12491	2.36889	0.01784	0.06102	1164.87	995.631	1064.69	1395.42	1372.83	1191.4	Up
Scube1	1060.11	-0.3092	0.12965	-2.3846	0.0171	0.05899	1325.48	1069.42	1124.85	937.191	995.832	907.877	Down
Efcab6	840.507	-0.7197	0.25184	-2.8579	0.00426	0.01896	1031.36	1132.66	973.981	666.608	393.897	844.537	Down
Sult4a1	139.308	1.69725	0.30261	5.60879	2.04E-08	3.46E-07	84.1726	67.0781	45.8344	193.866	178.468	266.431	Up
Pnpla3	66.2196	-2.221	0.63809	-3.4807	0.0005	0.0031	56.1151	228.066	42.9697	31.1015	17.9524	21.1134	Down
Gm49513	70.3165	-1.6045	0.49968	-3.211	0.00132	0.00707	56.1151	189.735	71.6162	35.2483	38.0169	31.1674	Down
Samm50	3278.16	-0.2061	0.08874	-2.3223	0.02021	0.06731	3361.1	3630.84	3543.57	3090.45	3136.4	2906.61	Down
Parvb	3914.12	-0.6536	0.17541	-3.7263	0.00019	0.00136	4070.28	4952.28	5334.93	2754.55	3763.67	2609.02	Down
Parvg	947.606	1.38021	0.20758	6.64907	2.95E-11	8.57E-10	644.356	464.755	468.847	1262.72	1712.87	1132.08	Up
Prr5	223.838	-0.4376	0.21202	-2.064	0.03902	0.11233	312.503	212.733	247.315	198.013	198.533	173.934	Down
Arhgap8	1141.08	0.56123	0.20518	2.73528	0.00623	0.02597	1094.24	660.24	1011.22	1175.64	1458.37	1446.77	Up
Nup50	2957.27	0.32117	0.09319	3.44641	0.00057	0.00345	2771.89	2551.84	2564.82	3350.67	3114.22	3390.21	Up
Upk3a	110.189	1.21552	0.51091	2.37914	0.01735	0.05963	88.0426	73.7859	37.2404	158.618	59.1374	244.312	Up
Fam118a	869.779	-0.4332	0.13773	-3.1454	0.00166	0.00852	908.484	954.426	1135.36	750.582	690.641	779.186	Down
Ribc2	197.058	-0.938	0.27306	-3.4353	0.00059	0.00358	241.875	350.723	184.292	140.993	126.723	137.74	Down
Gm19277	9.98115	-3.1356	1.54999	-2.023	0.04307	0.12146	13.545	40.2469	0	2.07343	0	4.0216	Down
Fbln1	2957.93	-0.4662	0.16184	-2.8807	0.00397	0.01786	3600.07	3887.66	2807.36	2643.63	2685.47	2123.41	Down
Atxn10	10254	0.27761	0.09523	2.91522	0.00355	0.01627	10136.5	8707.7	8967.31	11310.6	11277.3	11124.8	Up
Wnt7b	755.516	1.04009	0.24016	4.33086	1.49E-05	0.00014	520.516	603.703	359.036	1181.86	754.002	1113.98	Up
Ppara	183.497	-1.0022	0.35673	-2.8095	0.00496	0.02159	152.865	294.185	287.42	133.736	73.9218	158.853	Down
Gtse1	133.733	1.32951	0.24187	5.49674	3.87E-08	6.25E-07	63.8551	91.0346	73.526	192.829	170.02	211.134	Up
Trmu	871.103	-0.2347	0.11225	-2.0908	0.03654	0.10686	928.801	888.306	1008.36	786.867	788.851	825.434	Down
Celsr1	2902.38	0.51537	0.12055	4.27494	1.91E-05	0.00017	2268.79	2486.68	2412.99	3227.3	3141.68	3876.83	Up
Tbc1d22a	2072.35	0.37431	0.118	3.17203	0.00151	0.00789	2004.66	1741.16	1669.14	2416.58	2428.86	2173.68	Up
Pim3	6211.74	-0.5178	0.19179	-2.6996	0.00694	0.02835	7400.42	7656.49	6886.62	6069.97	5667.69	3589.28	Down
Ttll8	262.441	-0.7083	0.20106	-3.5228	0.00043	0.00271	287.348	299.935	389.592	184.535	189.029	224.204	Down
Mlc1	5802.57	1.68297	0.20229	8.31972	8.82E-17	5.61E-15	3730.69	2094.75	2442.59	8536.32	9129.34	8881.71	Up
Mov10l1	42.9304	-5.9438	0.92965	-6.3936	1.62E-10	4.18E-09	68.6926	145.655	39.1502	2.07343	0	2.0108	Down
Panx2	57.9931	-1.9878	0.43119	-4.61	4.03E-06	4.35E-05	126.743	93.9093	57.293	17.6242	23.2326	29.1566	Down
Hdac10	1448.59	-0.3963	0.11093	-3.5724	0.00035	0.00229	1551.87	1589.75	1797.09	1275.16	1286.24	1191.4	Down
Mapk12	3530.56	-0.3415	0.10922	-3.1271	0.00177	0.00897	4184.44	3498.6	4156.61	3036.54	3131.12	3176.06	Down
Mapk11	1362.62	0.64565	0.15332	4.21106	2.54E-05	0.00023	936.541	1047.38	1204.11	1427.56	1845.93	1714.21	Up
Plxnb2	19757.7	0.83818	0.0815	10.2845	8.28E-25	1.08E-22	14954.7	13910.1	13658.6	25184.9	24800.8	26036.9	Up
Dennd6b	906.076	-0.262	0.11393	-2.2993	0.02149	0.07051	934.606	958.259	1071.38	835.593	835.316	801.304	Down
Adm2	40.3562	2.83825	0.98109	2.89296	0.00382	0.01727	1.935	19.1652	8.59395	176.242	21.1205	15.081	Up
Tymp	1951.22	-1.1207	0.17154	-6.5332	6.44E-11	1.76E-09	2326.84	2667.79	3025.07	1301.08	979.992	1406.56	Down
Odf3b	2232.64	-0.6768	0.18157	-3.7276	0.00019	0.00136	2375.22	2964.85	2900.93	1991.53	1306.3	1856.98	Down
Mapk8ip2	8.62713	-1.6685	0.84085	-1.9843	0.04722	0.13051	13.545	14.3739	11.4586	7.25701	2.11205	3.0162	Down
Shank3	7662.81	-0.5625	0.16799	-3.3485	0.00081	0.00467	7702.28	8463.34	11248.5	5383.67	6443.87	6735.18	Down
Rabl2	2123.75	-0.6872	0.17547	-3.9161	9.00E-05	0.00069	2317.17	2862.32	2681.31	1760.34	1250.33	1871.05	Down
Syt10	85.9069	-2.004	0.35626	-5.625	1.85E-08	3.18E-07	111.263	181.111	120.315	27.9913	28.5127	46.2484	Down
Alg10b	3485.53	0.23296	0.07606	3.06282	0.00219	0.01076	3202.43	3180.46	3231.32	3720.77	3763.67	3814.49	Up
Cpne8	2446.11	0.25836	0.12904	2.00218	0.04527	0.12615	2210.74	2022.88	2449.27	2401.03	2950.53	2642.19	Up
Kif21a	2791.43	-1.0695	0.21942	-4.8742	1.09E-06	1.33E-05	3229.52	4512.44	3601.82	1714.73	1326.37	2363.7	Down
Abcd2	2003.55	0.93288	0.23068	4.04413	5.25E-05	0.00043	1525.75	1531.3	1075.2	2273.52	3552.47	2063.08	Up
Lrrk2	14780.5	1.78862	0.18932	9.44763	3.47E-21	3.37E-19	7845.47	6155.85	5905.95	19526.5	19961	29288.3	Up
Cntn1	312.418	-0.9822	0.30386	-3.2324	0.00123	0.00663	235.103	561.54	447.84	191.792	228.101	210.129	Down
Gm26760	63.0559	-3.0658	0.87029	-3.5227	0.00043	0.00271	88.0426	196.443	53.4734	35.2483	2.11205	3.0162	Down
Pdzrn4	29.9493	-1.6514	0.65089	-2.5371	0.01118	0.04186	30.96	85.285	20.0525	18.6609	12.6723	12.0648	Down
Gm30085	8.03661	-3.8516	1.43108	-2.6914	0.00711	0.02892	4.83751	36.4138	3.81953	1.03672	2.11205	0	Down
Zcrb1	3840.44	-0.2774	0.09796	-2.8316	0.00463	0.02035	3849.69	4405.12	4370.5	3507.21	3544.02	3366.08	Down
Irak4	1295.8	0.63251	0.11854	5.33583	9.51E-08	1.41E-06	1108.76	938.135	1001.67	1546.78	1685.42	1494.03	Up
Twf1	517.145	0.76768	0.19316	3.97441	7.06E-05	0.00056	463.433	329.641	355.216	600.258	588.206	766.115	Up
Ano6	1527.9	0.5158	0.15139	3.40704	0.00066	0.00391	1423.19	1051.21	1298.64	1630.75	1791.02	1972.6	Up
Gm49172	2220.95	-0.3198	0.14417	-2.2182	0.02654	0.08355	2231.06	2467.52	2699.45	1950.06	2270.45	1707.17	Down
D030018L	1005.38	-1.7523	0.28441	-6.161	7.23E-10	1.63E-08	1091.34	1789.07	1771.31	561.9	278.791	539.9	Down
Slc38a4	1311.04	-1.5764	0.33695	-4.6784	2.89E-06	3.23E-05	1894.37	2071.76	1925.04	628.25	306.247	1040.59	Down
Amigo2	2094.21	-0.6596	0.19085	-3.4559	0.00055	0.00335	2624.83	2625.63	2443.55	1884.75	1834.32	1152.19	Down

Rpap3	1231.69	-0.2394	0.10571	-2.2651	0.02351	0.07586	1331.28	1371.27	1298.64	1163.2	1044.41	1181.35	Down
Rapgef3	3212.94	-0.6057	0.11859	-5.108	3.26E-07	4.46E-06	3759.71	3609.76	4263.55	2327.43	2760.45	2556.73	Down
Slc48a1	8892.78	-0.3157	0.13039	-2.4215	0.01546	0.05435	11430.1	8881.14	9274.78	7958.87	8428.14	7383.66	Down
Vdr	546.604	0.53461	0.16587	3.22309	0.00127	0.00682	536.963	404.385	398.186	642.764	615.663	681.662	Up
Pfkm	2033.16	-1.5021	0.27554	-5.4515	4.99E-08	7.91E-07	2487.45	4600.6	1927.91	1085.44	1054.97	1042.6	Down
Ccdc184	191.978	-1.5247	0.44708	-3.4103	0.00065	0.00387	128.678	350.723	375.269	120.259	125.667	51.2754	Down
Zfp641	1370.02	-0.7936	0.18527	-4.2832	1.84E-05	0.00017	1276.13	2061.21	1875.39	992.137	1015.9	999.368	Down
Cacnb3	1596.51	0.69	0.15362	4.49147	7.07E-06	7.23E-05	1436.74	1097.21	1131.54	2138.74	2045.52	1729.29	Up
Rnd1	724.664	0.55597	0.21933	2.53486	0.01125	0.0421	595.013	559.623	605.396	812.785	1145.79	629.381	Up
Ccdc65	1090.59	-0.826	0.1986	-4.159	3.20E-05	0.00028	1302.26	1548.55	1333.02	867.731	566.03	925.974	Down
Fkbp11	546.906	0.95741	0.25587	3.74183	0.00018	0.00129	521.483	307.601	286.465	810.712	585.038	770.137	Up
Wnt10b	642.275	-1.521	0.2443	-6.2261	4.78E-10	1.11E-08	666.609	1074.21	1117.21	317.235	267.174	411.209	Down
Rhebl1	379.292	-0.8043	0.16327	-4.9262	8.38E-07	1.05E-05	510.841	418.759	517.547	298.574	255.558	274.474	Down
Lmbr1l	1942.11	-0.3103	0.10447	-2.9697	0.00298	0.014	1990.15	2134.04	2326.09	1774.86	1742.44	1685.05	Down
Tuba1b	8777.08	-0.325	0.14924	-2.1778	0.02942	0.09058	7946.09	10188.2	11150.2	8175.54	8154.63	7047.86	Down
4930578M	62.3058	-1.1785	0.30047	-3.9223	8.77E-05	0.00068	88.0426	93.9093	77.3455	35.2483	39.0729	40.216	Down
Tuba1c	4059.98	0.82049	0.11775	6.968	3.21E-12	1.06E-10	2753.51	3014.68	3038.44	5340.12	5647.62	4565.53	Up
Prph	100.947	-1.6305	0.46581	-3.5004	0.00046	0.00291	200.273	157.154	100.263	45.6155	80.2579	22.1188	Down
Dnajc22	73.9216	2.82734	0.41774	6.76824	1.30E-11	4.03E-10	23.22	22.9982	8.59395	121.296	104.547	162.875	Up
Spats2	1527.63	0.73422	0.1059	6.93321	4.11E-12	1.34E-10	1072.96	1153.74	1214.61	1888.9	1823.76	2011.81	Up
Kcnh3	665.284	-0.5859	0.13817	-4.24	2.23E-05	0.0002	822.376	775.231	798.282	528.725	468.875	598.214	Down
1700120C	40.2807	-1.6784	0.58021	-2.8928	0.00382	0.01727	36.7651	108.283	39.1502	20.7343	11.6163	25.135	Down
Prpf40b	2698.13	-0.2911	0.1057	-2.7539	0.00589	0.02481	2679.98	3133.51	3094.78	2461.16	2338.04	2481.33	Down
Tmbim6	54428.6	0.34635	0.07808	4.43603	9.16E-06	9.08E-05	49190.7	46205.3	48383	62540.9	61801.8	58450	Up
Faim2	998.582	-1.33	0.15235	-8.73	2.55E-18	1.95E-16	1509.3	1378.93	1397.95	521.468	681.136	502.7	Down
Aqp2	33.9799	-1.9294	0.59301	-3.2536	0.00114	0.00624	62.8876	65.1616	33.4209	25.9179	8.4482	8.04321	Down
Racgap1	422.206	0.46188	0.17252	2.67724	0.00742	0.02991	322.178	424.509	318.931	463.412	478.379	525.825	Up
Cox14	2315.12	-0.2053	0.10188	-2.0152	0.04388	0.12325	2450.68	2578.67	2409.17	2295.29	2172.24	1984.66	Down
Larp4	3713.38	0.20966	0.09611	2.18143	0.02915	0.08996	3480.1	3220.71	3631.42	3938.48	3828.09	4181.46	Up
Tmprss12	27.1757	-2.0522	0.66662	-3.0785	0.00208	0.0103	31.9275	65.1616	34.3758	7.25701	4.2241	20.108	Down
Mettl7a1	31405.6	-0.5778	0.15388	-3.7546	0.00017	0.00124	46624.9	33908	32301.8	25582	24294.9	25722.2	Down
Mettl7a3	74.1305	-1.173	0.42063	-2.7888	0.00529	0.02272	94.8151	131.281	82.1199	59.0928	22.1765	55.297	Down
Slc11a2	4533.2	-0.4887	0.14902	-3.2796	0.00104	0.00576	5031.98	5789.8	5059.92	3623.32	3206.09	4488.11	Down
5330439K	39.9745	-1.1668	0.50035	-2.332	0.0197	0.06604	41.6026	76.6607	47.7441	14.514	21.1205	38.2052	Down
Csrnp2	436.934	-0.4769	0.14901	-3.2004	0.00137	0.00728	513.743	459.006	552.877	358.704	342.152	395.123	Down
Gm49492	261.88	-0.4338	0.19099	-2.2711	0.02314	0.07497	334.756	278.853	289.33	185.572	234.438	248.334	Down
Smagp	2375.91	-0.3711	0.15894	-2.3347	0.01956	0.06564	2339.42	2468.47	3231.32	1910.67	2315.86	1989.69	Down
Bin2	1311.61	0.78933	0.18992	4.15612	3.24E-05	0.00028	1102.95	985.09	796.372	1517.75	2052.91	1414.6	Up
Cela1	140.259	1.54994	0.22362	6.93126	4.17E-12	1.35E-10	81.2701	69.9529	63.0223	200.086	229.158	198.064	Up
Galnt6	1325.94	1.13214	0.15648	7.23487	4.66E-13	1.78E-11	706.276	990.839	795.417	1821.51	1679.08	1962.54	Up
Scn8a	43.5403	2.185	0.40365	5.41308	6.19E-08	9.59E-07	19.35	16.2904	11.4586	78.7904	76.0338	59.3187	Up
Fignl2	709.405	-0.4269	0.14808	-2.883	0.00394	0.01774	768.196	731.151	941.515	628.25	564.974	622.343	Down
Acvrl1	27407.9	-0.4829	0.14346	-3.3658	0.00076	0.00444	30846.8	27077.5	37932.7	22432.5	24542	21616.1	Down
Grasp	1584.71	-0.3877	0.12845	-3.0184	0.00254	0.01222	1763.76	1740.2	1884.94	1267.9	1576.65	1274.85	Down
Krt7	24875.7	0.36391	0.14276	2.54919	0.0108	0.04077	20767.4	19867.6	24629.3	30150.8	30258.3	23580.7	Up
Krt87	406.903	2.43801	0.30155	8.08481	6.23E-16	3.60E-14	124.808	167.695	87.8492	958.962	549.133	552.97	Up
Krt1	69.2862	1.4358	0.37373	3.84178	0.00012	0.0009	20.3175	49.8294	42.0148	99.5247	103.49	100.54	Up
Krt4	15.6963	1.96143	0.96487	2.03284	0.04207	0.1193	7.74001	11.4991	0	20.7343	40.129	14.0756	Up
Krt78	15.4328	1.93212	0.70951	2.72317	0.00647	0.02673	7.74001	9.58259	1.90977	18.6609	29.5687	25.135	Up
Tns2	15136.7	-0.7417	0.08991	-8.2499	1.59E-16	9.77E-15	19265.9	17610.9	19956.1	11494.1	11570.9	10922.7	Down
Igfbp6	7160.23	-1.3336	0.27737	-4.8079	1.53E-06	1.81E-05	13520.8	7809.81	9426.6	5361.89	4351.88	2490.38	Down
Soat2	84.4203	0.9239	0.36651	2.52082	0.01171	0.0435	55.1476	78.5772	41.06	128.553	127.779	75.4051	Up
Csad	2566.66	-0.5251	0.10043	-5.2286	1.71E-07	2.44E-06	2828.97	3194.83	3062.31	2196.8	1990.61	2126.42	Down
Zfp740	5992.02	-0.4061	0.07641	-5.3146	1.07E-07	1.57E-06	6817.02	6709.73	6963.01	5079.91	5068.92	5313.54	Down
Rarg	4104.46	-0.3959	0.11108	-3.564	0.00037	0.00236	4674.97	4186.63	5130.59	3626.43	3617.94	3390.21	Down
Gm9918	14.379	-1.5669	0.74453	-2.1045	0.03534	0.10404	34.8301	13.4156	16.233	4.14686	11.6163	6.03241	Down
Espl1	207.964	0.80062	0.19173	4.17573	2.97E-05	0.00026	141.255	159.071	154.691	298.574	262.95	231.242	Up
Amhr2	367.188	0.5507	0.18693	2.94612	0.00322	0.01496	269.933	329.641	294.104	429.2	514.284	365.966	Up
Prr13	6706.26	0.4411	0.12926	3.41241	0.00064	0.00384	5346.41	5947.91	5772.27	7781.59	8760.79	6628.61	Up
Pcbp2	11530	-0.2345	0.08411	-2.7875	0.00531	0.0228	13097.1	12538.8	11758.4	10819.2	10583.5	10382.8	Down
Gm28047	324.31	-0.3816	0.18275	-2.0879	0.03681	0.10748	341.528	354.556	404.87	240.518	275.623	328.766	Down
Calcoco1	6785.99	-0.2847	0.11325	-2.514	0.01194	0.04413	8138.62	7476.33	6745.29	6547.9	6111.22	5696.6	Down
Copz1	7640.54	0.31292	0.09872	3.16986	0.00153	0.00794	6379.7	6802.68	7262.84	8715.67	8781.91	7900.44	Up
Gpr84	3.79452	4.40051	1.69988	2.58871	0.00963	0.03703	0	0	0.95488	11.4039	7.39218	3.0162	Up
Zfp385a	505.022	0.53812	0.18982	2.83494	0.00458	0.02017	476.978	415.884	342.803	624.103	672.688	497.673	Up
Nckap1l	2384.68	1.24996	0.18887	6.61808	3.64E-11	1.03E-09	1803.42	1278.32	1153.5	3173.39	3846.04	3053.4	Up
Pde1b	741.809	0.41708	0.14622	2.85237	0.00434	0.01924	724.659	610.411	571.02	777.537	854.325	912.904	Up
Ppp1r1a	212.59	-1.6524	0.34691	-4.7632	1.91E-06	2.21E-05	268.965	496.378	202.435	97.4513	87.6501	122.659	Down
Gm41409	45.5307	0.79388	0.36187	2.19385	0.02825	0.08782	41.6026	32.5808	25.7818	53.9092	55.9693	63.3403	Up
Cluap1	947.903	-0.4598	0.12399	-3.7084	0.00021	0.00144	1081.67	1105.83	1105.75	859.437	702.257	832.472	Down
Slx4	868.307	0.24385	0.10867	2.24392	0.02484	0.07925	829.149	764.69	791.598	927.861	909.238	987.304	Up
Srl	1593.59	-4.4905	0.66404	-6.7625	1.36E-11	4.16E-10	1680.55	5939.29	1534.5	125.443	109.827	171.924	Down
Tfap4	281.249	-0.6381	0.16208	-3.9372	8.24E-05	0.00064	339.593	319.1	368.585	209.417	238.662	212.14	Down
Coro7	2040.14	0.46937	0.11039	4.2519	2.12E-05	0.00019	1707.64	1849.44	1576.51	2472.57	2218.71	2415.98	Up
Cdip1	3230.22	-0.2115	0.08394	-2.5193	0.01176	0.04366	3462.69	3445.9	3491.05	3132.96	2879.78	2968.95	Down
4930562C	968.084	-0.951	0.20596	-4.6172	3.89E-06	4.22E-05	1294.52	1285.98	1248.03	658.315	473.099	848.558	Down
Mgrn1	2746.08	0.39218	0.0868	4.51836	6.23E-06	6.44E-05	2431.33	2421.52	2272.62	3047.94	3086.76	3216.28	Up
Nudt16l1	1239.94	-0.2338	0.09796	-2.3863	0.01702	0.05878	1343.86	1339.65	1336.84	1178.75	1163.74	1076.78	Down
Smim22	296.114	0.34909	0.15937	2.19039	0.0285	0.08841	245.745	245.314	290.284	347.3	326.312	321.728	Up
Glyr1	7262.01	0.40415	0.10517	3.84305	0.00012	0.0009	6751.23	5763.93	6239.2	7685.17	8588.65	8543.9	Up
Sec14l5	568.949	-2.0394	0.16883	-12.08	1.35E-33	3.69E-31	794.319	1038.75	912.868	228.077	195.365	244.312	Down
Nagpa	1596.02	0.30356	0.15076	2.01347	0.04407	0.12359	1531.55	1249.57	1504.9	1733.39	2026.51	1530.22	Up
Rbfox1	109.994	-1.4245	0.4439	-3.2091	0.00133	0.0071	117.068	270.229	93.5785	43.5421	59.1374	76.4105	Down

Mettl22	486.665	-0.3301	0.16311	-2.0241	0.04296	0.12122	491.491	610.411	524.231	450.971	468.875	374.009	Down
Abat	422.433	-1.1678	0.31395	-3.7199	0.0002	0.00139	397.643	921.845	434.472	241.555	255.558	283.523	Down
Pmm2	2950.31	0.3839	0.09716	3.95127	7.77E-05	0.00061	2396.5	2543.22	2740.51	3410.8	3215.6	3395.24	Up
Atf7ip2	37.7214	4.10073	0.62353	6.57667	4.81E-11	1.34E-09	0	7.66607	4.77441	65.3131	79.2019	69.3727	Up
Nubp1	323.137	0.62766	0.17746	3.537	0.0004	0.00258	286.38	230.94	244.45	350.41	444.587	382.052	Up
Ciita	74.8483	1.30139	0.2844	4.57585	4.74E-06	5.04E-05	40.6351	40.2469	48.699	101.598	120.387	97.5239	Up
Clec16a	1981.48	0.43279	0.09725	4.45012	8.58E-06	8.58E-05	1601.21	1744.99	1713.06	2251.75	2397.18	2180.71	Up
Gspt1	171.658	0.45113	0.22241	2.02841	0.04252	0.1202	165.443	132.24	137.503	164.838	201.701	228.226	Up
Tnfrsf17	15.9031	1.5709	0.59189	2.65405	0.00795	0.03167	9.67501	8.62433	5.7293	24.8812	26.4006	20.108	Up
Snx29	368.349	0.44041	0.19885	2.21481	0.02677	0.08408	362.813	268.312	306.517	357.667	500.556	414.225	Up
Cpped1	2131.76	0.22161	0.1011	2.19188	0.02839	0.08814	2009.5	2079.42	1816.19	2207.17	2330.65	2347.61	Up
Shisa9	79.3305	-1.6382	0.28476	-5.7528	8.78E-09	1.62E-07	131.58	116.908	111.721	44.5788	38.0169	33.1782	Down
2310015D	13.9878	-3.2022	1.02149	-3.1349	0.00172	0.00877	6.77251	55.579	13.3684	2.07343	2.11205	4.0216	Down
Pla2g10	26.8762	1.364	0.56623	2.40893	0.016	0.05589	13.545	23.9565	7.63906	39.3952	27.4567	49.2646	Up
Pla2g10os	6.2784	2.20298	1.07308	2.05295	0.04008	0.11471	1.935	3.83303	0.95488	17.6242	5.28013	8.04321	Up
A930007A	389.786	0.97392	0.19615	4.96507	6.87E-07	8.82E-06	236.07	308.559	244.45	534.945	424.522	590.17	Up
Rrn3	2046.35	0.21386	0.09645	2.21736	0.0266	0.08366	1971.77	1831.23	1882.07	2125.27	2134.23	2333.54	Up
Pdxdc1	4035.16	0.47214	0.10163	4.64589	3.39E-06	3.73E-05	3601.04	3358.7	3182.62	4518.01	4514.51	5036.05	Up
Nde1	3339.55	-0.2528	0.0941	-2.6862	0.00723	0.02927	3667.8	3404.69	3821.44	3034.47	3168.08	2940.8	Down
Myh11	4175.16	-1.3097	0.21515	-6.0874	1.15E-09	2.52E-08	4125.43	7596.12	6128.44	2161.55	2755.17	2284.27	Down
Abcc1	2874.16	0.39633	0.15058	2.63206	0.00849	0.03336	2721.58	2547.05	2177.13	3249.07	2787.91	3762.21	Up
Pkp2	3683.5	0.35023	0.08988	3.89687	9.74E-05	0.00074	3330.14	3294.49	3090.96	4124.06	4320.2	3941.17	Up
Ppm1f	5639.11	-0.4102	0.14815	-2.7686	0.00563	0.02394	5592.16	5933.54	7780.39	4529.41	5084.76	4914.4	Down
Mapk1	9328.97	0.25611	0.08628	2.96835	0.00299	0.01405	9014.21	8235.27	8259.74	9816.66	10507.5	10140.5	Up
2610318N	28.0523	1.24547	0.57195	2.17758	0.02944	0.0906	11.61	26.8312	11.4586	35.2483	57.0254	26.1404	Up
Sdf2l1	1065.68	0.91729	0.11183	8.2023	2.36E-16	1.42E-14	681.121	746.483	785.869	1437.92	1404.51	1338.19	Up
Ccdc116	116.802	1.23082	0.2724	4.51845	6.23E-06	6.44E-05	74.4976	73.7859	61.1125	207.343	137.283	146.789	Up
Ydjc	148.881	0.9851	0.27405	3.5946	0.00032	0.00213	113.198	116.908	69.7065	227.041	198.533	167.902	Up
Ube2l3	320.7	0.52662	0.15617	3.37201	0.00075	0.00435	268.965	273.104	246.36	417.796	359.049	358.928	Up
Tmem191	134.514	-0.5966	0.22635	-2.636	0.00839	0.03305	161.573	140.864	183.338	118.186	105.603	97.5239	Down
Pi4ka	2247.24	0.49358	0.13807	3.5749	0.00035	0.00227	2184.62	1743.07	1672	2436.28	2730.88	2716.59	Up
Snap29	2481.59	0.50423	0.11977	4.21011	2.55E-05	0.00023	2247.51	1909.81	1999.52	2658.14	3164.91	2909.63	Up
Aifm3	292.484	1.08307	0.26184	4.13633	3.53E-05	0.0003	198.338	201.234	163.285	371.144	283.015	537.889	Up
Lztr1	5477.85	0.31058	0.09409	3.30076	0.00096	0.00542	5186.78	4618.81	4866.08	6074.12	5771.18	6350.11	Up
Lrrc74b	1818.84	-0.592	0.2876	-2.0585	0.03954	0.11348	1791.81	2538.43	2230.61	1639.05	787.795	1925.34	Down
P2rx6	1124.1	-0.396	0.14499	-2.7316	0.0063	0.02621	1305.16	1303.23	1224.16	1038.79	801.523	1071.76	Down
Slc7a4	64.9619	-1.3214	0.42619	-3.1005	0.00193	0.00969	76.4326	136.073	65.8869	23.8445	43.297	44.2376	Down
Ccdc74a	892.475	-0.4722	0.18605	-2.5383	0.01114	0.04175	1029.42	1058.88	1023.63	883.282	552.301	807.337	Down
Med15	3706.5	0.31725	0.09846	3.22197	0.00127	0.00684	3388.19	3292.58	3220.82	4251.57	4328.65	3757.18	Up
Klhl22	2072.3	0.32194	0.11844	2.71809	0.00657	0.02705	1931.13	1793.86	1800.91	2336.76	2529.18	2041.97	Up
AA914427	24.4473	-1.5595	0.49858	-3.1278	0.00176	0.00896	42.5701	33.5391	33.4209	8.29373	14.7844	14.0756	Down
Slc25a1	4552.29	0.70319	0.1462	4.80983	1.51E-06	1.79E-05	3277.89	4112.85	3002.15	5286.21	6168.24	5466.36	Up
Prodh	1953.69	-0.5581	0.09236	-6.0421	1.52E-09	3.25E-08	2252.34	2341.98	2386.25	1530.19	1659.02	1552.34	Down
4933432I(	24.403	-1.6669	0.50297	-3.3142	0.00092	0.00519	32.895	47.9129	30.5563	10.3672	11.6163	13.0702	Down
Zdhhc8	2991.05	-0.2064	0.08799	-2.3454	0.01901	0.06417	3245	3124.88	3243.74	2848.9	2858.66	2625.1	Down
Tango2	1689.84	-0.6576	0.13879	-4.7381	2.16E-06	2.47E-05	1941.78	2407.15	1856.29	1389.2	1320.03	1224.58	Down
Arvcf	1687.06	-0.5317	0.12425	-4.2792	1.88E-05	0.00017	2012.4	1971.14	1999.52	1458.66	1494.28	1186.37	Down
Comt	5257.12	0.24921	0.08491	2.93488	0.00334	0.01542	4745.59	5053.86	4613.04	5610.71	5886.29	5633.26	Up
Gnb1l	413.445	-0.3213	0.13673	-2.3502	0.01876	0.06356	464.401	440.799	472.667	341.08	390.729	370.993	Down
Tbx1	1560.17	-1.3947	0.22946	-6.0781	1.22E-09	2.66E-08	1921.46	2493.39	2367.15	976.586	562.862	1039.58	Down
4930588K	55.6463	-1.7876	0.41585	-4.2987	1.72E-05	0.00016	63.8551	96.7841	98.3529	25.9179	14.7844	34.1836	Down
Cldn5	17892.1	-0.8984	0.20902	-4.2983	1.72E-05	0.00016	16835.5	25254.9	27778.5	13026.3	14528.8	9928.33	Down
Gm28539	139.018	-0.9557	0.21504	-4.4442	8.82E-06	8.79E-05	197.37	191.652	161.375	98.488	88.7061	96.5185	Down
Iglc2	300.021	0.78636	0.18037	4.35965	1.30E-05	0.00012	240.908	195.485	224.397	371.144	336.872	431.317	Up
Iglv2	134.71	2.08458	0.44033	4.7341	2.20E-06	2.51E-05	21.285	44.0799	88.8041	230.151	181.636	242.302	Up
Olfr171	60.1268	1.14285	0.40249	2.83946	0.00452	0.01993	53.2126	21.0817	38.1953	73.6068	78.1459	96.5185	Up
Lamp3	58428.8	2.12091	0.17189	12.339	5.58E-35	1.72E-32	27284.5	17681.8	20565.3	84400.1	98227.3	102414	Up
Map6d1	23.1697	-1.5623	0.57901	-2.6983	0.00697	0.02842	47.4076	25.873	30.5563	6.2203	16.8964	12.0648	Down
Cyp2ab1	154.33	1.59135	0.31343	5.07723	3.83E-07	5.16E-06	102.555	65.1616	63.0223	170.021	302.023	223.199	Up
Abcc5	8409.58	0.53093	0.08439	6.29132	3.15E-10	7.64E-09	6877	6621.57	7139.66	9457.96	10148.4	10212.9	Up
Abcf3	2744.88	-0.2256	0.10171	-2.2182	0.02654	0.08355	2918.95	2845.07	3112.92	2629.11	2633.73	2329.51	Down
Eif4g1	14643.3	0.22511	0.08077	2.7872	0.00532	0.02281	13339.9	13133.9	14035.8	15122.6	15949.2	16278.4	Up
Fam131a	932.601	-0.4421	0.1392	-3.1761	0.00149	0.00781	1077.8	1079	1066.6	858.401	664.24	849.564	Down
Ephb3	2547.72	0.32288	0.12664	2.5497	0.01078	0.04072	2361.67	2118.71	2310.82	2939.09	3107.88	2448.15	Up
Ehhadh	953.292	0.37038	0.12556	2.94988	0.00318	0.01481	817.539	897.888	779.184	1084.4	1158.46	982.277	Up
Map3k13	593.6	0.95704	0.17879	5.35281	8.66E-08	1.30E-06	499.231	354.556	357.126	746.435	852.212	752.04	Up
Tmem41a	1263.03	-0.4564	0.16391	-2.7846	0.00536	0.02296	1221.95	1542.8	1618.53	1093.74	1193.31	907.877	Down
Etv5	9490.69	1.32846	0.26785	4.95971	7.06E-07	9.05E-06	7648.1	3509.14	5059.92	13737.5	16640.8	10348.6	Up
Dgkg	272.101	0.99527	0.29827	3.33686	0.00085	0.00484	264.128	150.447	130.819	336.933	445.643	304.636	Up
Dnajb11	5744.85	0.39769	0.0905	4.3944	1.11E-05	0.00011	4912	4746.26	5215.57	6684.74	6735.33	6175.17	Up
Kng2	3568.5	5.84825	0.70196	8.33128	8.00E-17	5.18E-15	136.418	194.527	34.3758	7014.42	9267.68	4763.59	Up
Kng1	219.751	6.59881	1.00632	6.55736	5.48E-11	1.52E-09	5.80501	7.66607	0	322.419	720.209	262.41	Up
Eif4a2	37065.8	0.31856	0.08957	3.55652	0.00038	0.00242	32616.4	31638.8	34714.8	39732.1	43792.3	39900.3	Up
Rfc4	419.712	0.58679	0.13944	4.20812	2.58E-05	0.00023	367.651	315.267	323.705	491.403	498.444	521.803	Up
Adipoq	595.583	-8.4315	0.89784	-9.3908	5.95E-21	5.61E-19	639.518	2386.06	537.599	2.07343	4.2241	4.0216	Down
B630019A	149.007	-0.4786	0.24376	-1.9634	0.0496	0.13595	178.988	181.111	160.42	92.2677	141.507	139.751	Down
Rtp4	1902.65	0.45076	0.14976	3.0099	0.00261	0.01251	1603.15	1793.86	1426.6	2249.67	1872.33	2470.27	Up
Bcl6	2831.49	-0.2758	0.12345	-2.234	0.02549	0.0809	2698.36	3409.48	3195.99	2409.33	2630.56	2645.21	Down
Lpp	5568.71	0.43515	0.09855	4.41559	1.01E-05	9.87E-05	4552.09	5015.53	4637.87	5953.86	6810.31	6442.61	Up
Uts2b	16.5974	-3.1065	1.35734	-2.2887	0.0221	0.07217	13.545	67.0781	8.59395	10.3672	0	0	Down
Fgf12	404.863	-3.5101	0.44048	-7.9689	1.60E-15	8.63E-14	402.481	1411.51	419.194	70.4967	59.1374	66.3565	Down
Mb21d2	578.344	-0.2648	0.1315	-2.0139	0.04402	0.1235	652.096	601.786	639.772	514.211	576.59	485.609	Down
Plaat1	46.1079	-2.8336	0.69277	-4.0902	4.31E-05	0.00036	32.895	171.528	38.1953	11.4039	10.5603	12.0648	Down

Atp13a4	424.42	1.17669	0.23319	5.04597	4.51E-07	6.00E-06	279.608	274.062	227.262	710.15	645.231	410.204	Up
Gm49636	1.71986	4.2836	2.17576	1.96878	0.04898	NA	0	0	0	2.07343	4.2241	4.0216	Up
4632428C	791.016	0.66395	0.15866	4.18484	2.85E-05	0.00025	645.323	606.578	584.388	891.576	1161.63	856.602	Up
9030404E	213.33	1.05018	0.2383	4.40697	1.05E-05	0.0001	146.093	123.615	147.052	233.261	368.553	261.404	Up
Hes1	5281.59	-0.3804	0.08448	-4.5033	6.69E-06	6.87E-05	5887.25	6311.09	5723.57	4513.86	4635.95	4617.81	Down
Cpn2	249.831	4.14936	0.90426	4.58866	4.46E-06	4.77E-05	48.3751	27.7895	3.81953	542.202	274.567	602.235	Up
Gp5	154.204	-1.4247	0.34054	-4.1836	2.87E-05	0.00025	134.483	294.185	245.405	105.745	76.0338	69.3727	Down
Atp13a3	16347.1	0.47125	0.10751	4.38313	1.17E-05	0.00011	14816.3	13293	12993.1	19381.4	17293.5	20305.1	Up
Acap2	6379.06	-0.2578	0.09587	-2.6892	0.00716	0.02905	6574.17	6839.09	7428.99	5715.41	6139.73	5576.96	Down
Gm41442	433.818	-0.9636	0.20495	-4.7016	2.58E-06	2.91E-05	532.126	468.588	719.982	278.877	274.567	328.766	Down
Ppp1r2	15960.4	-0.3905	0.11074	-3.5265	0.00042	0.00267	16185.3	18354.5	19782.3	13162.1	14645	13633.2	Down
Gm49767	220.867	-0.4891	0.18181	-2.6903	0.00714	0.02899	250.583	264.479	258.773	157.581	202.757	191.026	Down
Apod	385.5	-1.3343	0.32356	-4.1238	3.73E-05	0.00032	392.806	852.85	410.6	176.242	208.037	272.464	Down
Gm46560	6.28095	1.96648	0.95122	2.06732	0.0387	0.11164	1.935	1.91652	3.81953	8.29373	12.6723	9.04861	Up
Senp5	692.454	0.49554	0.15878	3.12098	0.0018	0.00913	643.388	519.376	561.471	729.848	765.618	935.023	Up
Pak2	7972.47	0.24856	0.08904	2.79153	0.00525	0.02256	7507.81	6904.25	7450	8225.3	9062.81	8684.65	Up
Nrros	2170.47	0.47617	0.13986	3.40466	0.00066	0.00394	1826.64	1800.57	1819.05	2422.8	2979.05	2174.68	Up
Smco1	26.4514	-5.2206	0.94979	-5.4966	3.87E-08	6.25E-07	36.7651	87.2015	30.5563	2.07343	2.11205	0	Down
Ubxn7	3366.18	-0.2107	0.08478	-2.4852	0.01295	0.04719	3661.03	3439.19	3734.55	3118.44	3068.81	3175.06	Down
Tm4sf19	4.10416	2.91885	1.34671	2.1674	0.0302	0.09222	0	0.95826	1.90977	6.2203	9.50423	6.03241	Up
Gm20056	19.3124	2.01078	0.55039	3.65334	0.00026	0.00174	6.77251	8.62433	7.63906	25.9179	32.7368	34.1836	Up
Tfrc	7523.36	1.10537	0.15213	7.26602	3.70E-13	1.43E-11	4861.69	4545.98	4915.74	8815.19	9403.91	12597.7	Up
Tnk2	2428.74	-0.2049	0.09271	-2.2102	0.02709	0.08484	2462.29	2605.51	2734.78	2265.22	2315.86	2188.76	Down
Muc4	3716.01	1.45467	0.19495	7.46167	8.54E-14	3.67E-12	1861.47	2522.14	1576.51	5287.25	4684.53	6364.19	Up
Muc20	3419.36	2.29412	0.21774	10.5361	5.89E-26	8.64E-24	820.441	1509.26	1144.9	5962.15	4758.45	6320.96	Up
Smbd1	73.9268	2.0477	0.29818	6.86723	6.55E-12	2.09E-10	24.1875	33.5391	28.6465	127.516	114.051	115.621	Up
Snx4	7539.23	0.50915	0.09382	5.42687	5.74E-08	8.99E-07	6331.33	5920.12	6415.86	8906.43	9391.23	8270.43	Up
Gm15657	553.575	-0.6819	0.20966	-3.2525	0.00114	0.00626	543.736	636.284	866.079	468.596	365.385	441.371	Down
Muc13	592.623	5.41365	0.63919	8.46961	2.46E-17	1.72E-15	18.3825	24.9147	38.1953	1876.46	465.707	1132.08	Up
Kalrn	1600.07	0.49598	0.13221	3.75146	0.00018	0.00125	1414.49	1305.15	1263.31	1808.03	2138.45	1670.98	Up
Ccdc14	537.802	-0.6096	0.13857	-4.3994	1.09E-05	0.00011	574.696	693.779	680.832	417.796	421.354	438.355	Down
Adcy5	1385.54	-0.8509	0.2072	-4.1068	4.01E-05	0.00034	1484.15	2264.37	1599.43	1117.58	1065.53	782.202	Down
Sec22a	1611.96	0.21336	0.10347	2.06214	0.03919	0.11271	1444.48	1515.01	1519.22	1710.58	1861.77	1620.71	Up
Pdia5	832.333	-0.6025	0.1524	-3.9532	7.71E-05	0.0006	900.744	989.881	1120.08	722.591	690.641	570.062	Down
Parp14	5020.24	0.54231	0.11121	4.87648	1.08E-06	1.32E-05	4162.19	3966.23	4134.64	5293.47	6006.67	6558.23	Up
Dtx3l	2437.3	1.03482	0.10903	9.49151	2.28E-21	2.26E-19	1702.8	1607.96	1485.8	3016.84	3414.13	3396.24	Up
Parp9	1335.78	0.90787	0.12601	7.205	5.80E-13	2.18E-11	1040.06	822.186	924.327	1681.55	1729.77	1816.76	Up
Fam162a	2271.32	0.29231	0.12884	2.26885	0.02328	0.0753	1908.88	2211.66	2005.25	2376.15	2840.71	2285.28	Up
Csta2	154.443	0.78708	0.2263	3.47808	0.00051	0.00313	109.328	128.407	102.172	193.866	225.989	166.897	Up
Cd86	568.092	1.07026	0.23716	4.51286	6.40E-06	6.59E-05	481.816	298.977	318.931	638.617	718.097	952.115	Up
Ildr1	1809.73	0.7571	0.16849	4.49337	7.01E-06	7.18E-05	1258.72	1639.58	1138.22	2407.25	1990.61	2424.02	Up
Golgb1	5797.31	0.37835	0.09497	3.98372	6.78E-05	0.00054	4992.31	4867	5265.22	6745.91	6061.59	6851.81	Up
Hcls1	1777.78	1.23833	0.20492	6.04307	1.51E-09	3.23E-08	1369.98	989.881	815.47	2272.48	2911.46	2307.39	Up
Fbxo40	234.982	-3.5063	0.81676	-4.293	1.76E-05	0.00016	198.338	923.761	173.789	31.1015	30.6247	52.2808	Down
Ndufb4	1975.88	-0.2378	0.10646	-2.2333	0.02553	0.08097	1973.7	2191.54	2249.7	1923.11	1726.6	1790.62	Down
Gsk3b	5569.96	0.54253	0.09952	5.4516	4.99E-08	7.91E-07	4915.87	4363.91	4324.66	6459.78	6979.27	6376.25	Up
Popdc2	1251.42	-1.9983	0.28301	-7.0609	1.65E-12	5.75E-11	1438.67	3096.13	1470.52	500.734	547.021	455.447	Down
Pla1a	209.119	1.01436	0.34456	2.94392	0.00324	0.01503	187.695	137.031	90.7139	337.969	327.368	173.934	Up
Cd80	319.576	0.67912	0.21541	3.15267	0.00162	0.00835	316.373	210.817	210.074	388.768	360.105	431.317	Up
B4galt4	4399.18	-0.2969	0.11506	-2.58	0.00988	0.03783	4279.26	5083.56	5187.88	3942.63	3739.39	4162.36	Down
Upk1b	3066.42	-0.6616	0.14901	-4.4397	9.01E-06	8.94E-05	3850.66	3792.79	3628.55	2773.21	2430.97	1922.33	Down
Lsamp	69.7195	-1.5144	0.46188	-3.2786	0.00104	0.00577	64.8226	160.029	84.9846	52.8725	27.4567	28.1512	Down
Zbtb20	5459.66	-0.5225	0.12563	-4.159	3.20E-05	0.00028	6006.25	6956.96	6349.97	4003.8	4367.72	5073.25	Down
Qtrt2	1039.91	-0.3907	0.10565	-3.6981	0.00022	0.00149	1143.59	1175.78	1220.34	856.327	892.341	951.109	Down
Ccdc191	1182.28	-0.4997	0.16778	-2.9785	0.0029	0.01365	1284.84	1383.73	1486.75	1023.24	773.011	1142.14	Down
Zdhhc23	1127.25	0.49206	0.12339	3.98798	6.66E-05	0.00053	892.036	885.431	1033.18	1250.28	1280.96	1421.64	Up
Atp6v1a	9285.86	0.71656	0.11613	6.1703	6.82E-10	1.54E-08	7860.95	6462.5	6754.84	10644	12080.9	11912	Up
Naa50	5884.18	0.35918	0.08858	4.055	5.01E-05	0.00041	5332.87	4946.53	5186.92	6292.87	6965.54	6580.35	Up
Sidt1	750.767	1.15869	0.11457	10.113	4.84E-24	5.90E-22	488.588	461.881	443.066	998.357	1077.15	1035.56	Up
Boc	2385.38	-0.3878	0.13946	-2.7805	0.00543	0.0232	2598.71	2955.27	2558.13	2412.44	1874.45	1913.28	Down
Nepro	416.637	0.4037	0.14642	2.75722	0.00583	0.02463	350.236	323.891	402.006	473.779	478.379	471.533	Up
Cd200r1	688.443	2.36491	0.28828	8.20338	2.34E-16	1.42E-14	341.528	174.403	155.646	1151.79	1340.1	967.196	Up
Gm19142	25.2215	2.08618	0.70148	2.97399	0.00294	0.01383	10.6425	16.2904	1.90977	35.2483	58.0814	29.1566	Up
Cd200r4	948.331	2.56321	0.57017	4.49552	6.94E-06	7.11E-05	488.588	182.069	152.781	1586.18	2091.99	1188.38	Up
Cd200r2	69.4143	3.79563	0.87185	4.35355	1.34E-05	0.00013	22.2525	3.83303	1.90977	95.3779	262.95	30.162	Up
Gm6030	47.1213	-0.9321	0.36239	-2.5721	0.01011	0.03857	74.4976	58.4538	52.5186	29.028	39.0729	29.1566	Down
Gcsam	158.583	0.83492	0.21986	3.79749	0.00015	0.00106	117.068	102.534	122.225	168.985	216.485	224.204	Up
BC016579	51.2336	2.33633	0.46968	4.97433	6.55E-07	8.44E-06	9.67501	15.3321	25.7818	111.965	60.1934	84.4537	Up
Tagln3	72.0922	-1.257	0.39148	-3.2109	0.00132	0.00707	81.2701	85.285	138.458	27.9913	42.241	57.3078	Down
Phldb2	12849.9	0.46894	0.12771	3.67191	0.00024	0.00163	10740.2	9573	12025.8	13618.3	14670.3	16471.5	Up
Gm7204	225.546	0.45588	0.18281	2.49369	0.01264	0.04628	211.883	190.693	168.059	283.023	250.278	249.339	Up
Morc1	177.493	1.8746	0.47714	3.92885	8.54E-05	0.00066	35.7976	133.198	59.2027	293.391	156.292	387.079	Up
Retnlb	8.49368	5.5969	1.47735	3.78847	0.00015	0.00109	0.9675	0	0	15.5507	26.4006	8.04321	Up
Retnla	45695.9	4.28463	0.24104	17.7759	1.09E-70	3.35E-67	5451.87	4085.06	3844.36	85946.9	116939	57908.1	Up
Cip2a	226.884	0.66159	0.1838	3.59943	0.00032	0.00209	192.533	177.278	157.556	260.216	265.062	308.658	Up
Gm9575	103.731	-0.5572	0.23415	-2.3797	0.01733	0.05958	116.1	128.407	126.045	93.3044	77.0899	81.4375	Down
Cblb	3009.02	-0.3153	0.09512	-3.3144	0.00092	0.00519	3126.96	3479.44	3403.2	2582.46	2658.02	2804.06	Down
Alcam	25522.5	0.40613	0.10445	3.88842	0.0001	0.00077	23347.7	20959	21554.6	28112.6	27189.5	31971.7	Up
Nfkbiz	2731.45	0.53597	0.17888	2.99617	0.00273	0.01299	1836.32	2840.28	2012.89	3510.32	2970.6	3218.29	Up
Nxpe3	847.872	-0.5911	0.16281	-3.6304	0.00028	0.00189	1132.94	891.181	1033.18	724.664	739.218	566.041	Down
Cep97	693.602	-0.5477	0.17642	-3.1045	0.00191	0.00958	807.864	844.226	819.29	566.047	443.531	680.656	Down
Trmt10c	1056.92	-0.2839	0.10275	-2.7634	0.00572	0.02425	1193.9	1123.08	1164.96	966.219	906.07	987.304	Down
Abi3bp	3356.16	-0.8762	0.14062	-6.2311	4.63E-10	1.08E-08	3805.18	4202.92	5027.46	2177.1	2324.31	2599.97	Down

Adgrg7	25.986	4.70399	0.78073	6.02508	1.69E-09	3.56E-08	1.935	3.83303	0	42.5053	43.297	64.3457	Up
Tmem45a	800.785	-0.6257	0.18448	-3.3918	0.00069	0.0041	787.546	1107.75	1019.81	664.535	702.257	522.808	Down
Cmss1	699.341	-0.8393	0.15054	-5.5752	2.47E-08	4.13E-07	797.221	1047.38	846.981	521.468	497.388	485.609	Down
Col8a1	554.415	-0.9167	0.14772	-6.2058	5.44E-10	1.25E-08	778.839	705.278	690.38	435.421	371.721	344.852	Down
Cpox	1380.31	0.45074	0.15613	2.88695	0.00389	0.01755	1374.82	1092.41	1032.23	1603.8	1416.13	1762.47	Up
Gpr15	40.2589	2.44708	0.4193	5.83606	5.34E-09	1.03E-07	11.61	11.4991	14.3232	54.9459	70.7537	78.4213	Up
Riox2	836.381	0.54202	0.12983	4.1749	2.98E-05	0.00026	592.111	717.736	733.35	991.1	992.664	991.325	Up
Gm9816	842.337	0.26153	0.12661	2.06556	0.03887	0.112	828.181	752.233	718.072	907.126	995.832	852.58	Up
Pros1	6836.53	0.2214	0.11094	1.99556	0.04598	0.12776	6894.42	5836.75	6207.69	7112.91	7990.94	6976.48	Up
Gm18870	146.569	-1.0218	0.28651	-3.5665	0.00036	0.00234	158.67	197.401	232.991	101.598	120.387	68.3673	Down
Cggbp1	8509.21	0.21208	0.09011	2.35367	0.01859	0.06309	7588.11	7818.43	8248.28	8827.64	8850.55	9722.23	Up
Chmp2b	5966.68	0.28435	0.09622	2.95516	0.00313	0.01459	5083.25	5611.56	5446.65	6596.62	6938.09	6123.9	Up
Vgll3	1715.91	-0.9266	0.13931	-6.6512	2.91E-11	8.47E-10	1994.02	2290.24	2461.69	1180.82	1320.03	1048.63	Down
Cadm2	148.078	-1.9359	0.2766	-6.9988	2.58E-12	8.67E-11	208.013	272.145	224.397	51.8358	49.6332	82.4429	Down
Gbe1	2234.91	-0.2747	0.11486	-2.3917	0.01677	0.05811	2420.69	2721.45	2199.1	1992.57	2008.56	2067.1	Down
Robo1	859.847	-0.5873	0.13674	-4.2951	1.75E-05	0.00016	974.274	1009.05	1114.35	723.628	599.822	737.964	Down
Robo2	1353.78	-0.257	0.11633	-2.2091	0.02717	0.08505	1343.86	1572.5	1505.85	1184.97	1195.42	1320.09	Down
Hspa13	2921.83	0.35013	0.10533	3.32403	0.00089	0.00504	2513.57	2399.48	2793.99	3430.49	3105.77	3287.66	Up
Samsn1	743.211	0.78279	0.23538	3.32568	0.00088	0.00501	696.601	564.414	378.134	901.943	1108.83	809.348	Up
Nrip1	1719.72	0.34247	0.11096	3.08656	0.00202	0.01008	1420.29	1674.08	1455.24	1859.87	1989.55	1919.31	Up
Mir99ahg	555.423	-0.716	0.13771	-5.1991	2.00E-07	2.84E-06	624.038	759.899	687.516	403.282	423.466	434.333	Down
E330011C	87.2881	0.82052	0.38531	2.1295	0.03321	0.09906	88.0426	40.2469	61.1125	131.663	74.9778	127.686	Up
Cxadr	250.49	0.66207	0.19737	3.35441	0.0008	0.00459	214.785	189.735	177.608	268.509	289.351	362.95	Up
Btg3	3521.82	0.29822	0.14067	2.12006	0.034	0.10094	3611.68	2703.25	3162.57	3718.7	3650.68	4284.01	Up
Chodl	29.3123	2.2997	0.90523	2.54047	0.01107	0.04156	0	22.9982	6.68418	36.2851	48.5772	61.3295	Up
Ncam2	47.9546	-1.7472	0.6392	-2.7335	0.00627	0.02609	146.093	50.7877	24.827	26.9546	17.9524	21.1134	Down
Mrpl39	2460.26	-0.3317	0.13217	-2.51	0.01207	0.04457	2566.78	3094.22	2564.82	2309.8	1934.64	2291.31	Down
Jam2	4897.53	-0.2981	0.13071	-2.2806	0.02257	0.07333	5384.15	5970.91	4849.85	4431.96	4851.38	3896.93	Down
Atp5j	5253.17	-0.4021	0.10454	-3.8464	0.00012	0.00089	5493.47	6509.45	5938.42	4725.35	4546.19	4306.13	Down
App	60023.1	0.3409	0.09831	3.46744	0.00053	0.00323	57892.4	49330.2	51670.6	65838.7	70072.6	65334	Up
Adamts5	1005.17	-1.313	0.21725	-6.0438	1.50E-09	3.22E-08	1148.42	1871.48	1280.5	580.561	479.436	670.602	Down
Rwdd2b	360.056	0.61382	0.155	3.96015	7.49E-05	0.00059	300.893	288.436	264.503	481.036	399.178	426.29	Up
Map3k7cl	128.335	-0.9886	0.33996	-2.9079	0.00364	0.01658	101.588	207.942	202.435	67.3865	97.1543	93.5023	Down
Bach1	4035.26	0.21604	0.10636	2.0313	0.04222	0.11956	4120.59	3556.1	3524.47	4118.87	4462.76	4428.79	Up
Gm49688	20.7675	4.1343	0.98984	4.17672	2.96E-05	0.00026	1.935	1.91652	2.86465	30.0648	7.39218	80.4321	Up
Tiam1	2147.55	-0.3189	0.14599	-2.1845	0.02892	0.08943	2384.89	2584.42	2182.86	1801.81	1682.25	2249.08	Down
Sod1	11376.8	-0.48	0.10226	-4.694	2.68E-06	3.01E-05	14195.2	12466	13095.3	10286.3	9130.4	9087.82	Down
Gm49708	495.621	-2.1785	0.7824	-2.7844	0.00536	0.02297	764.326	1431.64	239.676	240.518	209.093	88.4753	Down
Gm36169	37.6644	-1.5043	0.38222	-3.9356	8.30E-05	0.00064	54.1801	57.4955	55.3832	21.771	20.0645	17.0918	Down
Mrap	152.706	-3.2216	0.58028	-5.5518	2.83E-08	4.68E-07	108.36	583.58	135.593	23.8445	31.6808	33.1782	Down
Urb1	825.403	-0.3441	0.16493	-2.0864	0.03695	0.10778	876.556	889.264	1004.54	723.628	599.822	858.612	Down
Eva1c	1602.55	-0.5423	0.16567	-3.2733	0.00106	0.00586	1598.31	2001.8	2100.74	1458.66	1082.43	1373.38	Down
4932438H	25.8228	1.07105	0.5303	2.01972	0.04341	0.12222	20.3175	21.0817	8.59395	37.3218	26.4006	41.2214	Up
Gm15966	5.25746	3.31415	1.31238	2.52531	0.01156	0.04306	1.935	0	0.95488	8.29373	5.28013	15.081	Up
Ifnar2	1750.95	0.53016	0.09604	5.52007	3.39E-08	5.54E-07	1472.54	1486.26	1339.7	2097.28	2031.79	2078.16	Up
Il10rb	4918.27	0.45535	0.09353	4.86829	1.13E-06	1.37E-05	4423.42	4065.89	3956.08	5861.59	5797.58	5405.04	Up
Ifnar1	3685.02	0.31477	0.11591	2.71553	0.00662	0.02721	3266.28	2952.39	3635.24	3942.63	3962.21	4351.37	Up
Ifngr2	4619.14	0.76122	0.09247	8.23167	1.85E-16	1.13E-14	3441.4	3493.81	3348.77	5507.03	6245.33	5678.5	Up
Tmem50b	12518.4	0.33859	0.10804	3.13409	0.00172	0.00879	12312.4	10116.3	10739.6	14615.6	13748.4	13577.9	Up
Gart	1607.6	0.21056	0.09721	2.16593	0.03032	0.09245	1582.83	1414.39	1474.34	1696.07	1725.55	1752.41	Up
Kcne2	1665.34	0.60998	0.1832	3.32949	0.00087	0.00495	1423.19	1061.75	1470.52	1964.58	1680.14	2391.85	Up
Smim11	596.091	-0.3738	0.13703	-2.7275	0.00638	0.0265	618.233	653.532	746.718	516.284	549.133	492.646	Down
Runx1	4273.93	0.38032	0.09581	3.96932	7.21E-05	0.00057	3637.81	3907.78	3596.09	4880.86	4516.62	5104.42	Up
1700029J(	71.9923	1.05894	0.4181	2.53272	0.01132	0.04231	29.9925	70.9111	39.1502	126.479	95.0423	70.3781	Up
Cbr1	3384.52	-0.4202	0.19683	-2.135	0.03276	0.09799	4850.08	3777.46	2994.51	3384.88	2468.99	2831.21	Down
Gm5678	10.9757	-4.9341	1.59927	-3.0852	0.00203	0.01011	54.1801	3.83303	5.7293	0	2.11205	0	Down
Cbr3	1433.96	-0.759	0.32412	-2.3417	0.0192	0.06465	3002.16	1254.36	1151.59	1209.85	946.199	1039.58	Down
Dop1b	3002.68	0.33415	0.11129	3.00238	0.00268	0.01277	2925.72	2516.39	2527.58	3207.6	3251.5	3587.27	Up
Chaf1b	338.477	1.22494	0.2478	4.94328	7.68E-07	9.76E-06	276.705	146.614	185.247	424.017	501.612	496.668	Up
2410003I1	22.8334	2.45684	0.69879	3.51584	0.00044	0.00277	4.83751	13.4156	2.86465	38.3585	23.2326	54.2916	Up
Gm49619	10.8712	2.37385	0.89006	2.66705	0.00765	0.03066	0.9675	7.66607	1.90977	22.8077	14.7844	17.0918	Up
Sim2	4.30744	4.59091	1.59636	2.87587	0.00403	0.01809	0	0.95826	0	8.29373	10.5603	6.03241	Up
Hlcs	1208.06	0.33761	0.11205	3.01303	0.00259	0.0124	1123.27	1102	976.845	1280.34	1385.51	1380.42	Up
Ripply3	3804.76	-0.7681	0.16563	-4.6371	3.53E-06	3.88E-05	4248.3	4632.22	5502.04	2802.24	3323.31	2320.47	Down
Pigp	2476.99	-0.2456	0.12486	-1.9668	0.04921	0.135	2452.62	3033.85	2575.32	2439.39	2230.33	2130.44	Down
Gm31641	1080.77	0.53404	0.14372	3.71573	0.0002	0.00141	823.344	799.188	1026.5	1239.91	1211.26	1384.44	Up
Get1	1136.31	-0.2761	0.11878	-2.3242	0.02011	0.06708	1235.5	1328.15	1170.69	1019.09	951.479	1112.98	Down
Sh3bgr	286.527	-2.1758	0.3612	-6.0238	1.70E-09	3.58E-08	312.503	780.981	314.156	103.672	99.2664	108.583	Down
Bace2	1639.87	0.44359	0.20099	2.20708	0.02731	0.08538	1595.41	1473.8	1100.03	2363.71	1584.04	1722.25	Up
Mx1	328.239	1.38852	0.14847	9.35245	8.56E-21	7.96E-19	173.183	184.944	186.202	497.624	450.923	476.56	Up
Tmprss2	8232.35	0.52047	0.1554	3.34915	0.00081	0.00467	7519.42	5618.27	7152.07	9775.19	10975.3	8353.88	Up
Ripk4	2447.27	0.82574	0.14318	5.76725	8.06E-09	1.49E-07	1973.7	1490.09	1832.42	3035.5	3456.37	2895.55	Up
Scaf8	1979.89	0.2689	0.12034	2.23456	0.02545	0.08079	1936.94	1639.58	1811.41	2026.78	2089.87	2374.76	Up
Tiam2	345.259	-0.4002	0.16013	-2.4991	0.01245	0.04571	346.366	417.801	414.419	287.17	284.071	321.728	Down
Snx9	4393.21	0.22135	0.09132	2.42387	0.01536	0.05408	4270.55	3845.49	4054.43	4634.12	4956.98	4597.7	Up
Tmem181	975.472	0.47402	0.11366	4.17051	3.04E-05	0.00026	865.914	822.186	761.997	1111.36	1091.93	1199.44	Up
Dynlt1a	161.754	0.52789	0.24575	2.14807	0.03171	0.09563	121.905	168.654	106.947	190.756	172.132	210.129	Up
Ezr	24544.1	0.29305	0.09353	3.13334	0.00173	0.00881	20474.3	22248.8	23456.7	26883.1	25959.2	28242.7	Up
Rsph3b	1605.95	-0.4582	0.13612	-3.3663	0.00076	0.00443	1666.04	2043.01	1867.75	1412.01	1189.08	1457.83	Down
Rps6ka2	1779.27	-0.2035	0.10024	-2.0299	0.04236	0.11987	1788.91	1971.14	1953.69	1602.76	1616.77	1742.36	Down
Fndc1	790.829	-1.1721	0.15037	-7.7947	6.45E-15	3.22E-13	995.559	1076.12	1214.61	540.129	495.276	423.274	Down
Gm49673	5.62734	4.05532	1.4306	2.8347	0.00459	0.02018	0	0	1.90977	9.33044	8.4482	14.0756	Up
Fgfr1op	2543.2	0.2674	0.12356	2.16402	0.03046	0.09281	2120.76	2403.31	2400.58	3003.37	2443.64	2887.51	Up

Mpc1	886.309	0.27013	0.1306	2.06835	0.03861	0.11143	833.986	873.932	702.794	1003.54	972.599	931.001 Up
T2	964.136	-0.6707	0.24824	-2.7017	0.0069	0.0282	1007.17	1381.81	1164	938.228	475.211	818.396 Down
1700010I1	200.993	0.66568	0.25343	2.62665	0.00862	0.03381	123.84	188.777	153.736	238.445	198.533	302.626 Up
Gm7947	3.24762	5.20158	1.87895	2.76834	0.00563	0.02395	0	0	0	9.33044	2.11205	8.04321 Up
Slc22a1	99.0321	-1.5315	0.35633	-4.2979	1.72E-05	0.00016	113.198	215.608	112.676	59.0928	44.3531	49.2646 Down
Igf2r	4732.23	0.40697	0.09363	4.3464	1.38E-05	0.00013	4359.56	4033.31	3814.76	5239.56	5398.4	5547.8 Up
Airn	1426.97	-0.3203	0.13143	-2.437	0.01481	0.05253	1588.64	1403.85	1761.76	1171.49	1277.79	1358.3 Down
Mrgprh	5.75921	-5.9224	1.81675	-3.2599	0.00111	0.00612	6.77251	25.873	1.90977	0	0	0 Down
Acat2	2733.98	1.25162	0.14368	8.71106	3.01E-18	2.29E-16	1611.86	1836.02	1403.68	3835.85	4279.01	3437.47 Up
Gpr31c	9.32057	2.64841	1.09602	2.41639	0.01568	0.05497	3.87001	3.83303	0	5.18358	16.8964	26.1404 Up
Gm19283	5.41753	2.90467	1.28173	2.2662	0.02344	0.07572	0	1.91652	1.90977	12.4406	3.16808	13.0702 Up
Dact2	289.368	1.0742	0.17866	6.01255	1.83E-09	3.81E-08	202.208	174.403	182.383	404.319	435.082	337.815 Up
Thbs2	136.787	-0.8026	0.37999	-2.1121	0.03468	0.10251	167.378	260.646	93.5785	92.2677	120.387	86.4645 Down
Wdr27	608.261	-0.8297	0.19112	-4.3413	1.42E-05	0.00013	737.236	823.144	775.365	472.742	326.312	514.765 Down
1600012H	1495.74	0.30761	0.12783	2.4065	0.01611	0.05621	1455.12	1165.24	1390.31	1762.42	1575.59	1625.73 Up
Ermard	1140.34	-0.2528	0.10254	-2.4653	0.01369	0.04939	1236.47	1304.19	1179.28	1010.8	1068.7	1042.6 Down
Gm35455	29.8126	-1.1028	0.4643	-2.3751	0.01754	0.06014	49.3426	35.4556	37.2404	12.4406	24.2886	20.108 Down
Gm5091	6.7316	-6.1473	1.5462	-3.9757	7.02E-05	0.00056	17.415	16.2904	6.68418	0	0	0 Down
Gm35576	8.80757	-4.6348	1.46899	-3.1551	0.0016	0.00829	7.74001	35.4556	7.63906	0	0	2.0108 Down
Dll1	2699.26	-0.4491	0.14384	-3.1222	0.0018	0.00911	3400.77	2520.22	3427.07	2303.58	2254.61	2289.3 Down
Spaca6	1520.39	0.30717	0.13014	2.36032	0.01826	0.06216	1260.65	1274.48	1542.14	1642.16	1817.42	1585.52 Up
Fpr1	861.405	2.29901	0.65992	3.48376	0.00049	0.00307	538.898	210.817	123.18	1020.13	1673.8	1601.6 Up
Fpr3	13.392	1.66545	0.76976	2.16358	0.0305	0.0929	11.61	5.74955	1.90977	24.8812	21.1205	15.081 Up
Zfp53	997.011	0.31815	0.12354	2.57539	0.01001	0.03825	807.864	878.723	975.89	1171.49	1081.37	1066.73 Up
Gm50433	32.2734	-0.9547	0.46611	-2.0482	0.04055	0.11573	50.3101	33.5391	43.9246	13.4773	23.2326	29.1566 Down
Gm10509	73.028	0.9	0.35069	2.56638	0.01028	0.03909	67.7251	52.7042	32.466	85.0107	108.771	91.4915 Up
Zfp820	317.262	0.62057	0.16872	3.67805	0.00024	0.0016	284.445	224.233	241.585	355.594	386.505	411.209 Up
Zfp995	424.553	0.3336	0.16273	2.04996	0.04037	0.11536	373.456	375.637	378.134	433.347	428.746	557.997 Up
Gm7072	906.461	0.39037	0.13337	2.92696	0.00342	0.01577	782.709	849.975	720.937	943.411	1137.34	1004.4 Up
Zfp946	1493.81	0.58913	0.10951	5.37984	7.46E-08	1.13E-06	1121.33	1254.36	1203.15	1669.11	1907.18	1807.71 Up
Mmp25	121.842	0.72395	0.25783	2.80785	0.00499	0.02167	106.425	94.8676	74.4809	132.7	143.619	178.961 Up
Bicdl2	512.13	0.42797	0.16351	2.61736	0.00886	0.03454	389.903	444.632	475.532	675.939	564.974	521.803 Up
Cldn6	6.58115	-4.1923	1.37586	-3.0471	0.00231	0.01124	15.48	9.58259	12.4135	0	0	2.0108 Down
9530082P	475.791	0.54361	0.26752	2.03203	0.04215	0.11944	546.638	269.271	345.668	574.341	646.288	472.538 Up
Srrm2	23961.2	0.25699	0.09488	2.70865	0.00676	0.0277	21868.4	20029.5	23599.9	25779	25986.7	26503.4 Up
Prss32	5.30151	4.9021	1.54562	3.17161	0.00152	0.0079	0.9675	0	0	12.4406	6.33615	12.0648 Up
Sbp	4.10754	5.54036	1.71283	3.23462	0.00122	0.00658	0	0	0	10.3672	4.2241	10.054 Up
Prss30	441.173	4.15829	0.72618	5.72626	1.03E-08	1.85E-07	62.8876	45.9964	31.5111	1663.93	402.346	440.366 Up
Prss22	129.121	3.74067	0.60801	6.15226	7.64E-10	1.71E-08	30.96	21.0817	1.90977	352.483	195.365	172.929 Up
Amdhd2	1468.47	0.99099	0.10512	9.42692	4.22E-21	4.00E-19	1012.97	986.048	950.108	1838.1	2096.21	1927.35 Up
Atp6v0c	7175.92	0.66606	0.11987	5.55653	2.75E-08	4.57E-07	5012.63	6160.64	5471.48	8041.8	8961.43	9407.54 Up
Ccnf	161.396	1.12592	0.22167	5.07927	3.79E-07	5.12E-06	99.6527	105.408	99.3078	223.931	182.692	257.383 Up
Abca3	11884.6	1.78756	0.18248	9.79595	1.17E-22	1.30E-20	7010.52	4310.25	4695.16	18100	18636.7	18554.7 Up
D330041H	205.555	0.76057	0.2111	3.60281	0.00031	0.00207	122.873	183.986	150.871	269.546	258.726	247.329 Up
Dnase1l2	316.026	-0.5293	0.22891	-2.3125	0.02075	0.06861	362.813	344.015	413.464	245.702	195.365	334.798 Down
Pgp	2319.95	0.31359	0.10649	2.94467	0.00323	0.01501	2180.75	2089.96	1935.55	2650.88	2691.81	2370.74 Up
Nthl1	494.512	-0.4882	0.15336	-3.1837	0.00145	0.00764	492.458	607.536	632.132	430.237	406.57	398.139 Down
Slc9a3r2	21206	-0.3054	0.14304	-2.135	0.03276	0.09799	25656.2	19724.8	24945.4	19665.5	20640	16604.2 Down
Gfer	950.772	0.49719	0.15695	3.16782	0.00154	0.00798	696.601	818.353	850.801	1125.87	946.199	1266.81 Up
Noxo1	195.185	0.95126	0.24918	3.81756	0.00013	0.00099	122.873	134.156	142.278	234.298	201.701	335.804 Up
Tbl3	1700.67	0.33143	0.09862	3.36056	0.00078	0.00452	1401.91	1564.84	1551.68	1946.95	1877.61	1861 Up
Ndufb10	3868.87	-0.6202	0.14837	-4.1798	2.92E-05	0.00026	3792.61	5456.32	4814.52	3158.87	3013.9	2976.99 Down
Rpl3l	65.7203	-1.3332	0.50083	-2.6621	0.00777	0.03105	72.5626	153.321	56.3381	25.9179	57.0254	29.1566 Down
Msrb1	2447.41	0.97308	0.21254	4.5784	4.69E-06	4.98E-05	2026.92	1715.28	1213.66	3094.6	3929.47	2704.53 Up
Hs3st6	242.894	-1.2641	0.30375	-4.1617	3.16E-05	0.00027	238.005	400.552	390.547	144.104	97.1543	187.005 Down
Hagh	2155.23	0.24746	0.09443	2.62046	0.00878	0.03431	1956.29	1972.1	1984.25	2477.75	2194.42	2346.61 Up
Igfals	143.871	1.13845	0.28769	3.95726	7.58E-05	0.0006	72.5626	116.908	80.2102	173.132	171.076	249.339 Up
Nubp2	1381.77	0.26	0.12066	2.1549	0.03117	0.09436	1356.44	1187.28	1228.93	1454.51	1656.9	1406.56 Up
Mapk8ip3	5099.65	-0.3043	0.0898	-3.3882	0.0007	0.00415	5418.98	5429.49	6057.78	4598.87	4528.24	4564.52 Down
Jpt2	2152.38	0.80315	0.1225	6.55632	5.52E-11	1.53E-09	1427.06	1535.13	1742.66	2815.72	2511.23	2882.48 Up
Clcn7	3393.89	0.36766	0.08011	4.58948	4.44E-06	4.75E-05	3045.69	2918.86	2926.72	3754.98	3914.69	3802.43 Up
Ccdc154	521.151	-0.6691	0.1975	-3.3879	0.0007	0.00415	648.226	671.739	599.666	518.358	340.04	348.874 Down
BC003965	1205.96	-0.364	0.13997	-2.6007	0.0093	0.03601	1162.94	1473.8	1435.19	969.329	1063.42	1131.08 Down
Gnptg	3077.39	0.26138	0.09035	2.89299	0.00382	0.01727	2705.13	2923.65	2769.16	3437.75	3440.53	3188.13 Up
Baiap3	558.451	-0.7553	0.20485	-3.6874	0.00023	0.00155	774.969	619.035	710.433	416.76	315.752	513.76 Down
Ube2i	9161.75	0.45407	0.0924	4.91421	8.91E-07	1.11E-05	8293.42	7381.47	7520.66	10510.2	10203.3	11061.4 Up
Tpsb2	20.5217	-4.2341	1.40213	-3.0197	0.00253	0.01217	4.83751	99.6589	12.4135	6.2203	0	0 Down
Cacna1h	334.858	-1.1586	0.392	-2.9555	0.00312	0.01457	264.128	709.111	414.419	255.032	115.107	251.35 Down
Tekt4	1667.26	-0.6369	0.18068	-3.5252	0.00042	0.00268	1895.34	2214.54	1978.52	1619.35	1016.95	1278.87 Down
Ccdc78	2726.76	-0.4894	0.23443	-2.0876	0.03683	0.10753	2615.16	3652.88	3286.71	2725.53	1475.27	2604.99 Down
Metrn	233.896	-0.3925	0.19492	-2.0138	0.04403	0.12351	222.525	312.392	261.638	201.123	199.589	206.107 Down
Jmjd8	883.188	0.3491	0.15401	2.26672	0.02341	0.07565	893.971	723.485	713.298	966.219	889.173	1112.98 Up
Wdr90	1379.89	-0.4895	0.16316	-3.0003	0.0027	0.01283	1485.11	1556.21	1794.22	1245.1	920.854	1277.86 Down
Mcrip2	446.683	-0.8251	0.2477	-3.3309	0.00087	0.00493	446.018	804.937	462.163	325.529	314.696	326.755 Down
Pigq	509.444	0.92859	0.16319	5.69015	1.27E-08	2.25E-07	389.903	316.225	346.622	594.038	761.394	648.484 Up
Capn15	274.254	0.98527	0.17973	5.48208	4.20E-08	6.75E-07	204.143	155.238	192.886	337.969	382.281	373.004 Up
Rab11fip3	930.6	0.37419	0.17411	2.1491	0.03163	0.09546	902.679	671.739	857.485	911.273	1014.84	1225.58 Up
Tmem8	2599.89	0.22071	0.09214	2.39526	0.01661	0.0576	2569.68	2325.69	2308.91	2793.95	2811.14	2789.99 Up
Pdia2	509.101	-0.8017	0.2329	-3.4422	0.00058	0.0035	542.768	679.405	719.027	440.604	255.558	417.241 Down
Arhgdig	2008.69	-0.4647	0.17062	-2.7233	0.00646	0.02672	2230.09	2491.47	2266.89	1794.56	1291.52	1977.62 Down
Rgs11	1743.29	-0.6384	0.16922	-3.7724	0.00016	0.00116	2001.76	2195.37	2171.4	1661.86	1076.09	1353.27 Down
Fam234a	4535.89	0.23415	0.10638	2.20112	0.02773	0.08649	4503.72	4105.18	3896.88	5242.67	4887.29	4579.6 Up
Luc7l	4491.89	-0.2588	0.12405	-2.0865	0.03693	0.10778	4614.98	4935.99	5130.59	3935.37	3662.3	4672.1 Down

Neurl1b	1144.27	-0.8004	0.15613	-5.1264	2.95E-07	4.08E-06	1254.85	1414.39	1692.05	739.178	894.453	870.677	Down
Gm50269	69.0697	-0.8425	0.35901	-2.3468	0.01894	0.06399	82.2376	100.617	83.0748	53.9092	64.4175	30.162	Down
Gm34455	32.2214	-2.1775	0.66511	-3.2739	0.00106	0.00585	45.4726	58.4538	54.4283	22.8077	2.11205	10.054	Down
Atp6v0e	6332.17	0.49064	0.1105	4.44011	8.99E-06	8.93E-05	5150.98	5383.5	5262.36	7353.43	8222.21	6620.56	Up
Crebrf	5729.41	-0.2037	0.09741	-2.0913	0.03651	0.10677	6405.83	5924.91	6069.24	4986.6	5240	5749.89	Down
Nkx2-5	53.4122	-6.2727	1.02746	-6.1051	1.03E-09	2.27E-08	44.5051	215.608	56.3381	0	0	4.0216	Down
Cuta	2517.11	0.26905	0.09444	2.84893	0.00439	0.01941	2215.58	2268.2	2365.24	2857.19	2819.59	2576.84	Up
Syngap1	1645.01	-0.4979	0.1981	-2.5134	0.01196	0.04418	1820.84	1807.28	2150.4	1398.53	996.888	1696.11	Down
Itpr3	3770.71	0.32639	0.09691	3.36789	0.00076	0.00441	3438.5	3089.43	3510.15	4191.44	4040.35	4354.39	Up
Uqcc2	1036.83	-0.2888	0.14647	-1.9716	0.04866	0.13362	943.314	1197.82	1279.54	939.265	945.143	915.92	Down
Ip6k3	292.08	-1.2713	0.22434	-5.6669	1.45E-08	2.55E-07	341.528	492.545	404.87	161.728	208.037	143.772	Down
9630028I(	1199.97	-0.9256	0.22377	-4.1363	3.53E-05	0.0003	1776.33	1711.45	1228.93	1075.07	638.895	769.132	Down
Grm4	301.759	-1.2592	0.2133	-5.9034	3.56E-09	7.12E-08	343.463	495.42	438.291	166.911	157.348	209.123	Down
Nudt3	3302.71	-0.381	0.11399	-3.3428	0.00083	0.00475	3449.14	4161.72	3598	2861.34	3024.46	2721.62	Down
Spdef	258.522	1.14611	0.30563	3.75003	0.00018	0.00126	124.808	238.606	119.36	309.978	321.032	437.349	Up
Ilrun	9284.14	0.26127	0.09545	2.73716	0.0062	0.02586	9088.71	7912.34	8336.13	10081	10563.4	9723.23	Up
Tcp11	934.957	-0.3691	0.18257	-2.0214	0.04323	0.12176	1041.03	1102	1018.86	923.714	607.215	916.926	Down
Scube3	170.634	-1.2137	0.28245	-4.2972	1.73E-05	0.00016	156.735	281.728	276.916	94.3411	103.49	110.594	Down
Def6	538.054	0.4154	0.13928	2.98257	0.00286	0.01349	428.603	462.839	491.765	643.801	647.344	553.976	Up
Ppard	1263.34	0.27908	0.09772	2.85593	0.00429	0.01906	1191.96	1104.87	1127.72	1418.23	1396.07	1341.2	Up
Fkbp5	2102.52	-0.6809	0.18618	-3.6571	0.00026	0.00171	2432.3	3242.75	2094.06	1738.57	1379.17	1728.28	Down
E230001N	24.5139	-1.3077	0.51117	-2.5583	0.01052	0.03986	42.5701	38.3303	23.8721	17.6242	11.6163	13.0702	Down
Slc26a8	97.0604	-1.0372	0.26693	-3.8857	0.0001	0.00077	119.003	128.407	144.187	75.6803	51.7452	63.3403	Down
Mapk13	2086.75	1.69713	0.10596	16.0173	9.68E-58	1.39E-54	1021.68	1027.25	902.364	3120.51	3083.59	3365.08	Up
Bnip5	61.681	4.35054	0.58532	7.43272	1.06E-13	4.47E-12	7.74001	5.74955	3.81953	175.205	54.9133	122.659	Up
Pxt1	2.0828	-4.4549	2.07354	-2.1484	0.03168	NA	4.83751	5.74955	1.90977	0	0	0	Down
Stk38	8207.9	0.57837	0.08805	6.56871	5.08E-11	1.41E-09	6734.78	6542.99	6475.06	9599.99	10522.2	9372.35	Up
Srsf3	15125.1	-0.2102	0.10558	-1.9912	0.04646	0.12882	14662.5	16266.4	17746.5	13374.7	14492.9	14207.3	Down
Trp53cor1	13.1649	1.9413	0.70302	2.76137	0.00576	0.02437	5.80501	6.70781	3.81953	23.8445	12.6723	26.1404	Up
Rab44	1168.26	1.18706	0.20708	5.73237	9.90E-09	1.79E-07	891.069	531.834	716.162	1587.21	1879.73	1403.54	Up
Pi16	1147.59	-2.0644	0.28631	-7.2105	5.58E-13	2.11E-11	1397.07	2820.16	1339.7	533.909	407.626	387.079	Down
Mtch1	14011.8	0.20313	0.08182	2.48256	0.01304	0.04747	13515	12929.8	12636	15478.2	15209.9	14301.8	Up
Fgd2	1415.52	0.68918	0.14099	4.88809	1.02E-06	1.25E-05	1245.17	1050.25	955.838	1622.46	1718.15	1901.21	Up
Gm36486	57.7683	2.16018	0.43574	4.95754	7.14E-07	9.14E-06	19.35	17.2487	26.7367	68.4232	140.451	74.3997	Up
Pim1	2120.63	1.00656	0.16032	6.27835	3.42E-10	8.21E-09	1174.55	1620.42	1433.28	2781.51	3215.6	2498.42	Up
Mdga1	175.779	1.11451	0.29759	3.74511	0.00018	0.00128	97.7176	146.614	88.8041	224.967	312.584	183.988	Up
Btbd9	1318.21	0.23441	0.09003	2.60374	0.00922	0.03573	1193.9	1215.07	1225.11	1418.23	1418.24	1438.73	Up
Gm10503	102.008	-0.7999	0.31393	-2.5481	0.01083	0.04086	114.165	137.989	136.548	48.7256	98.2104	76.4105	Down
Abcg1	4874.63	1.53941	0.26864	5.73046	1.00E-08	1.81E-07	3773.26	1814.94	1898.31	7038.26	8554.86	6168.13	Up
Rsph1	3003.93	-0.5243	0.2344	-2.2368	0.0253	0.08045	3015.7	4023.73	3592.27	2756.63	1579.81	3055.41	Down
Slc37a1	3973.42	0.60506	0.11664	5.18719	2.13E-07	3.00E-06	3068.91	3325.16	3062.31	4823.84	5304.42	4255.86	Up
Pde9a	1614.96	-0.8043	0.14366	-5.5987	2.16E-08	3.65E-07	1779.24	2175.25	2206.73	1098.92	1333.76	1095.89	Down
Gm50105	127.005	1.2596	0.2715	4.63937	3.49E-06	3.84E-05	54.1801	81.452	88.8041	201.123	181.636	154.832	Up
4833413E	8.13001	2.02881	0.85408	2.37544	0.01753	0.06009	2.9025	4.79129	1.90977	10.3672	13.7283	15.081	Up
Gm30571	176.053	-2.3328	0.43205	-5.3994	6.69E-08	1.03E-06	199.305	399.594	282.645	50.7991	27.4567	96.5185	Down
Hsf2bp	152.92	1.3236	0.36262	3.65014	0.00026	0.00176	66.7576	120.741	74.4809	218.747	135.171	301.62	Up
Rrp1b	1142.81	-0.2434	0.11531	-2.1108	0.03479	0.10277	1176.48	1198.78	1341.61	981.77	1103.55	1054.67	Down
Ephx3	92.789	-0.5867	0.29007	-2.0227	0.0431	0.12153	108.36	113.075	112.676	58.0561	98.2104	66.3565	Down
Akap8	7711.27	-0.2948	0.0951	-3.0996	0.00194	0.00971	8335.99	8026.37	9126.77	6726.21	6770.18	7282.12	Down
Cyp4f16	1702.4	0.60417	0.13174	4.58603	4.52E-06	4.82E-05	1409.65	1434.51	1208.88	1988.42	2296.86	1876.08	Up
Cyp4f37	5.63534	3.42214	1.23859	2.76293	0.00573	0.02428	1.935	0.95826	0	8.29373	10.5603	12.0648	Up
Cyp4f40	32.1807	1.63439	0.55662	2.93627	0.00332	0.01536	13.545	25.873	7.63906	55.9827	32.7368	57.3078	Up
Cyp4f15	1050.18	-1.3491	0.34744	-3.883	0.0001	0.00078	1116.5	1799.61	1608.98	689.416	267.174	819.402	Down
Zfp763	600.433	-0.419	0.12221	-3.4283	0.00061	0.00365	645.323	686.113	729.531	531.835	511.116	498.679	Down
Adamts10	7119.81	-0.3886	0.11278	-3.4454	0.00057	0.00347	7666.48	7608.57	8943.43	5742.37	6616	6141.99	Down
Myo1f	2341.08	1.09993	0.18606	5.91157	3.39E-09	6.80E-08	1759.89	1506.38	1202.2	2828.16	3871.39	2878.46	Up
Gm17251	137.556	-0.8739	0.21396	-4.0844	4.42E-05	0.00037	178.02	191.652	164.24	105.745	98.2104	87.4699	Down
Angptl4	3656.7	-1.2335	0.15847	-7.7839	7.03E-15	3.48E-13	4243.46	5366.25	5783.73	2510.93	2079.31	1956.51	Down
Kank3	2820.87	-0.4371	0.15892	-2.7502	0.00596	0.02502	3355.3	2640	3739.32	2218.57	2690.75	2281.25	Down
Rps28	1516.93	-0.4246	0.13628	-3.1158	0.00183	0.00927	1785.04	1514.05	1916.45	1377.8	1336.93	1171.29	Down
Kifc1	112.93	0.72331	0.33658	2.14899	0.03164	0.09546	101.588	96.7841	57.293	166.911	97.1543	157.848	Up
Tapbp	14614	0.35577	0.09871	3.60411	0.00031	0.00206	11764.8	13276.7	13421.8	15734.2	17402.2	16084.4	Up
Rps18	13048.5	-0.3208	0.14306	-2.2428	0.02491	0.07947	12169.2	14559.8	16751.5	11131.2	10923.5	12755.5	Down
AA388235	9.87561	-2.0721	0.97575	-2.1236	0.03371	0.1002	4.83751	13.4156	29.6014	6.2203	3.16808	2.0108	Down
H2-K1	75866.3	0.76362	0.15228	5.01448	5.32E-07	6.97E-06	49360	60291.7	59081.5	86179.1	85482.1	114804	Up
Ring1	1471.76	-0.4613	0.14767	-3.1237	0.00179	0.00907	1446.41	1653.95	2014.8	1229.54	1205.98	1279.88	Down
Col11a2	162.559	0.49797	0.2163	2.3022	0.02132	0.07012	145.125	125.532	133.684	226.004	171.076	173.934	Up
H2-DMa	3623.88	0.80305	0.13636	5.88928	3.88E-09	7.69E-08	2992.48	2437.81	2491.29	4782.37	5007.67	4031.66	Up
H2-DMb2	1044.63	0.75632	0.34118	2.21679	0.02664	0.08376	1141.65	607.536	581.524	1589.29	1640.01	707.802	Up
H2-DMb1	3494.95	0.95211	0.12733	7.47741	7.58E-14	3.31E-12	2224.29	2385.11	2536.17	4272.31	4268.45	5283.38	Up
Psmb9	2338.6	0.40365	0.13973	2.88875	0.00387	0.01746	2275.56	1867.65	1897.35	2508.85	3035.02	2447.15	Up
Tap1	3719.42	0.5902	0.13811	4.27351	1.92E-05	0.00017	3188.88	3079.84	2638.34	4184.19	5153.4	4071.87	Up
Psmb8	729.709	0.72941	0.1907	3.82483	0.00013	0.00096	667.576	509.794	469.802	961.036	1018.01	752.04	Up
Tap2	3577.45	0.23798	0.12068	1.97199	0.04861	0.13354	3532.35	3010.85	3305.8	3898.05	4241	3476.68	Up
H2-Ab1	22338.9	0.81567	0.08073	10.1035	5.33E-24	6.46E-22	16329.5	16097.8	16133.7	27411.8	27863.2	30197.2	Up
H2-Aa	33376.4	0.82878	0.1417	5.84872	4.95E-09	9.63E-08	22188.7	24116.5	25829.6	38968.1	38225	50930.6	Up
Gm20513	14.1902	1.50917	0.72719	2.07534	0.03796	0.10999	13.545	2.87478	5.7293	24.8812	19.0085	19.1026	Up
BC051142	397.12	0.77243	0.24087	3.20682	0.00134	0.00714	229.298	359.347	291.239	567.084	371.721	564.03	Up
Btnl4	24.1216	5.62227	0.99091	5.67387	1.40E-08	2.45E-07	0	1.91652	0.95488	64.2764	45.4091	32.1728	Up
Btnl6	9.79943	3.4878	1.00807	3.45989	0.00054	0.00331	2.9025	1.91652	0	17.6242	24.2886	12.0648	Up
Notch4	4817.85	-0.5894	0.18025	-3.2699	0.00108	0.00593	4616.92	5400.75	7347.82	3463.67	4121.67	3956.25	Down
Rnf5	1422.29	0.21877	0.10421	2.09939	0.03578	0.10508	1330.31	1348.27	1265.22	1590.32	1582.98	1416.61	Up
Prrt1	210.805	-0.3908	0.17506	-2.2322	0.0256	0.08113	262.193	233.815	221.533	179.352	187.973	179.967	Down

C4b	17209.3	1.52116	0.25886	5.87639	4.19E-09	8.28E-08	13410.5	7381.47	5887.81	26037.1	24110.1	26429	Up
Stk19-ps1	3.24832	3.19151	1.52257	2.09614	0.03607	0.10575	0.9675	0.95826	0	7.25701	5.28013	5.027	Up
Cyp21a1	7.2945	6.36684	1.55461	4.09545	4.21E-05	0.00035	0	0	0	12.4406	24.2886	7.03781	Up
C4a	169.385	0.7865	0.30985	2.53834	0.01114	0.04175	164.475	85.285	123.18	245.702	241.83	155.837	Up
Ehmt2	7575.47	0.42849	0.12211	3.50901	0.00045	0.00283	6911.83	6224.85	6239.2	9283.79	9289.86	7503.31	Up
Slc44a4	3863.41	1.30125	0.16728	7.77902	7.31E-15	3.60E-13	1957.26	2823.99	1909.77	5714.38	5247.39	5527.69	Up
Neu1	3737.44	1.14361	0.16118	7.09528	1.29E-12	4.58E-11	2550.33	2309.4	2127.48	5268.59	6044.69	4124.15	Up
Hspa1b	794.01	-0.6805	0.30927	-2.2003	0.02779	0.08663	752.716	844.226	1336.84	559.827	390.729	879.726	Down
Hspa1a	191.087	-1.326	0.45034	-2.9445	0.00323	0.01502	170.28	317.184	332.299	77.7537	55.9693	193.037	Down
Vwa7	212.505	0.68115	0.18783	3.62636	0.00029	0.00191	177.053	137.989	174.744	250.885	261.894	272.464	Up
Msh5	325.123	0.35884	0.15925	2.25332	0.02424	0.07769	287.348	272.145	295.059	373.218	400.234	322.734	Up
Clic1	13704.6	0.68224	0.07565	9.01795	1.92E-19	1.60E-17	10356.1	10489.1	10724.3	17381.6	17018.9	16257.3	Up
Mpig6b	161.784	-1.3996	0.33351	-4.1967	2.71E-05	0.00024	145.125	334.432	224.397	91.231	74.9778	100.54	Down
Ly6g6c	92.668	-0.7435	0.34403	-2.1613	0.03067	0.09324	154.8	109.241	84.0297	88.1208	66.5296	53.2862	Down
Ly6g6d	208.365	0.45245	0.20232	2.23625	0.02534	0.08052	166.41	165.779	195.751	209.417	232.326	280.507	Up
Ly6g6e	111.247	1.44143	0.30111	4.78709	1.69E-06	1.99E-05	59.9851	78.5772	41.06	182.462	162.628	142.767	Up
Aif1	342.386	0.99983	0.15469	6.46333	1.02E-10	2.71E-09	248.648	213.692	222.488	425.053	489.996	454.441	Up
Tnf	174.351	2.12471	0.30486	6.9694	3.18E-12	1.05E-10	81.2701	74.7442	39.1502	230.151	324.2	296.593	Up
Lta	31.21	1.06615	0.432	2.46794	0.01359	0.04912	26.1225	17.2487	17.1879	43.5421	35.9049	47.2538	Up
H2-D1	107139	0.91335	0.22145	4.1244	3.72E-05	0.00032	96530.5	64395	62016.8	157249	160573	102070	Up
H2-Q2	781.148	1.25132	0.2174	5.75592	8.62E-09	1.59E-07	628.876	378.512	379.089	1183.93	1092.99	1023.5	Up
H2-Q4	11571.1	0.29406	0.08441	3.48366	0.00049	0.00307	10304.9	10620.4	10262.1	12362.8	13572	12304.1	Up
H2-Q7	6295.72	0.33332	0.14836	2.24672	0.02466	0.07877	5085.19	5830.05	5799.96	6228.59	6373.11	8457.43	Up
Tcf19	388.11	0.52112	0.19768	2.63616	0.00839	0.03303	395.708	296.102	264.503	496.587	462.539	413.22	Up
Cdsn	172.658	1.29117	0.35183	3.66984	0.00024	0.00164	80.3026	155.238	64.932	240.518	299.911	195.048	Up
2300002M	72.9866	9.69043	1.31557	7.36597	1.76E-13	7.16E-12	0	0	0	176.242	215.429	46.2484	Up
Sfta2	1241.44	0.97541	0.19888	4.90465	9.36E-07	1.16E-05	1067.15	623.826	820.244	1804.92	1566.09	1566.41	Up
Gm20483	26.0834	1.3931	0.53004	2.62831	0.00858	0.03368	11.61	8.62433	22.9172	34.2116	35.9049	43.2322	Up
Vars2	577.476	1.39499	0.14798	9.42704	4.22E-21	4.00E-19	343.463	273.104	338.029	783.757	843.764	882.742	Up
Gtf2h4	683.736	-0.343	0.14949	-2.2943	0.02177	0.07129	656.933	861.475	775.365	571.23	645.231	592.181	Down
Ddr1	5416.96	0.31636	0.11748	2.69286	0.00708	0.02883	4996.18	4943.66	4536.65	6247.25	5259.01	6519.02	Up
4833427F	81.9435	0.85701	0.37831	2.26536	0.02349	0.07583	75.4651	50.7877	48.699	132.7	63.3615	120.648	Up
Ier3	9866.09	0.66759	0.15923	4.19259	2.76E-05	0.00024	8184.09	6617.73	8067.81	11534.5	14538.3	10254.1	Up
Tubb5	14541.4	0.30684	0.11913	2.57562	0.01001	0.03823	12158.6	12433.4	14410.1	16652.8	17186.8	14406.4	Up
Prr3	1131.29	-0.2399	0.11562	-2.0747	0.03802	0.11011	1152.29	1204.53	1318.69	1018.05	984.216	1109.96	Down
A930015D	1231.62	-0.543	0.18999	-2.8583	0.00426	0.01894	1246.14	1634.79	1501.08	793.088	1231.33	983.282	Down
Gm10499	59.6216	-1.0075	0.34199	-2.9459	0.00322	0.01497	63.8551	72.8277	102.172	37.3218	44.3531	37.1998	Down
Rpp21	804.707	-0.2619	0.12589	-2.0807	0.03747	0.1089	883.329	813.562	935.785	795.161	689.585	710.818	Down
Trim26	4100.47	0.33227	0.11608	2.86258	0.0042	0.01874	3860.33	3444.94	3585.59	4831.1	4848.21	4032.66	Up
Trim15	299.016	0.79347	0.17034	4.65812	3.19E-06	3.53E-05	190.598	218.483	247.315	364.924	369.609	403.166	Up
Trim10	70.5002	0.90554	0.45659	1.98328	0.04734	0.13077	73.5301	53.6625	20.0525	104.708	86.5941	84.4537	Up
Trim31	37.9242	4.10589	0.75236	5.45738	4.83E-08	7.68E-07	3.87001	5.74955	2.86465	78.7904	116.163	20.108	Up
Znrd1	1275.57	-0.4521	0.12726	-3.5526	0.00038	0.00245	1459.96	1414.39	1546.91	1196.37	1087.71	948.093	Down
H2-M6-ps	20.4493	-1.253	0.58397	-2.1456	0.03191	0.0961	23.22	42.1634	21.0074	13.4773	14.7844	8.04321	Down
2410137M	14.3889	-2.1938	0.8669	-2.5306	0.01139	0.04254	4.83751	33.5391	32.466	5.18358	5.28013	5.027	Down
H2-M5	168.273	-1.1264	0.27757	-4.0581	4.95E-05	0.0004	155.768	267.354	269.277	96.4146	118.275	102.551	Down
Mog	55.4044	-1.5447	0.37631	-4.1048	4.05E-05	0.00034	66.7576	74.7442	105.992	32.1382	31.6808	21.1134	Down
H2-M2	241.742	3.52162	0.28856	12.2043	2.95E-34	8.83E-32	40.6351	41.2051	34.3758	428.164	318.92	587.154	Up
Olfr110	2.06421	4.5469	2.10632	2.15869	0.03087	NA	0	0	0	7.25701	2.11205	3.0162	Up
Olfr111	3.58197	5.3433	1.79413	2.9782	0.0029	0.01366	0	0	0	4.14686	5.28013	12.0648	Up
Gm20541	2.05499	4.54113	2.0815	2.18167	0.02913	NA	0	0	0	2.07343	4.2241	6.03241	Up
Esp8	2.05577	4.54157	2.17783	2.08537	0.03704	NA	0	0	0	7.25701	1.05603	4.0216	Up
Crisp2	41.761	2.15022	0.39164	5.49029	4.01E-08	6.46E-07	11.61	18.2069	16.233	66.3498	71.8097	66.3565	Up
Cd2ap	7182.04	0.32518	0.09546	3.40633	0.00066	0.00392	6249.09	6670.44	6208.65	7364.83	8202.15	8397.11	Up
Tnfrsf21	3466.1	0.39192	0.11896	3.29467	0.00099	0.00551	2862.84	3333.78	2797.81	4244.31	3927.36	3630.5	Up
Slc25a27	589.688	-0.6456	0.16952	-3.8086	0.00014	0.00102	753.684	644.908	760.087	453.045	386.505	539.9	Down
Rcan2	2213.86	-0.5822	0.22984	-2.5332	0.0113	0.04227	2292.98	3737.21	1933.64	1797.67	1882.89	1638.8	Down
Enpp5	6952.72	0.32903	0.1084	3.03534	0.0024	0.01164	6606.1	6368.59	5515.4	7561.81	7472.44	8192.01	Up
Runx2	570.834	0.66333	0.1731	3.83205	0.00013	0.00094	471.173	449.423	404.87	710.15	816.308	573.078	Up
Nfkbie	819.841	1.3856	0.16122	8.59471	8.35E-18	6.19E-16	535.996	453.256	372.404	1248.21	1127.84	1181.35	Up
Slc35b2	2283.18	0.44909	0.13816	3.25043	0.00115	0.00629	2151.72	1950.06	1690.14	2840.6	2734.05	2332.53	Up
Slc29a1	2888.87	-0.4729	0.15724	-3.0078	0.00263	0.01258	3589.43	3053.01	3431.85	2534.77	2784.74	1939.42	Down
Tmem63b	5456.57	0.35969	0.1156	3.11148	0.00186	0.00939	5358.02	4325.58	4656.01	6474.29	6071.09	5854.45	Up
Xpo5	2555.3	0.27183	0.08732	3.11313	0.00185	0.00934	2334.58	2202.08	2409.17	2760.77	2810.08	2815.12	Up
Polr1c	2900.4	0.41904	0.10354	4.04707	5.19E-05	0.00042	2493.25	2422.48	2530.44	3575.63	3362.38	3018.21	Up
Yipf3	4618.19	0.21388	0.10029	2.13256	0.03296	0.09847	4269.58	4354.33	4205.3	5256.15	5139.68	4484.09	Up
Lrrc73	285.102	-0.7473	0.19304	-3.8715	0.00011	0.00081	324.113	381.387	366.675	228.077	171.076	239.285	Down
Klc4	2631.66	0.28824	0.11039	2.6111	0.00903	0.03506	2481.64	2378.4	2248.75	3135.03	2922.02	2624.1	Up
Gnmt	404.154	-0.584	0.21592	-2.7047	0.00684	0.028	488.588	509.794	456.434	288.207	256.614	425.285	Down
2310039H	295.367	-0.3328	0.16173	-2.0579	0.0396	0.11362	306.698	368.93	312.247	270.583	251.334	262.41	Down
Bicral	2591.32	-0.2701	0.10597	-2.5484	0.01082	0.04083	2798.98	2659.17	3041.3	2182.29	2434.14	2432.06	Down
Tbcc	838.707	-0.2424	0.11548	-2.099	0.03582	0.10514	887.199	956.342	883.267	784.794	806.803	713.835	Down
Foxp4	3617.01	0.21372	0.09743	2.19352	0.02827	0.08787	3366.91	3103.8	3577.95	3941.59	3971.71	3740.09	Up
Trem1	436.487	2.07704	0.66603	3.11853	0.00182	0.00919	296.055	118.824	86.8943	748.509	1012.73	355.912	Up
Treml2	303.482	-0.4353	0.19891	-2.1885	0.02863	0.08873	335.723	397.677	313.202	248.812	307.303	218.172	Down
Trem2	619.282	2.5299	0.30899	8.18777	2.66E-16	1.59E-14	276.705	162.904	108.857	1069.89	1259.84	837.499	Up
Treml1	122.689	-1.4114	0.45459	-3.1048	0.0019	0.00957	83.2051	288.436	163.285	58.0561	91.8742	51.2754	Down
Oard1	2211.24	-0.2225	0.08968	-2.4812	0.0131	0.0476	2413.92	2346.78	2383.39	2115.94	2074.03	1933.39	Down
Apobec2	183.077	-3.1262	0.51532	-6.0665	1.31E-09	2.84E-08	222.525	597.953	165.195	20.7343	13.7368	59.3187	Down
Mocs1	2243.27	0.58731	0.14557	4.0346	5.47E-05	0.00044	2065.62	1745.95	1566.96	2675.76	2984.33	2421.01	Up
Rftn1	2170.66	0.41504	0.11908	3.48537	0.00049	0.00306	1652.49	1854.23	2074.96	2507.82	2389.79	2544.67	Up
Plcl2	727.043	0.64678	0.1945	3.32531	0.00088	0.00501	691.764	575.913	432.562	850.107	989.496	822.418	Up
Tbc1d5	1857.06	0.31766	0.09014	3.52392	0.00043	0.0027	1642.82	1670.24	1647.17	1975.98	2151.12	2055.04	Up

Satb1	813.837	-0.7185	0.10814	-6.6447	3.04E-11	8.77E-10	1023.62	1025.34	988.304	583.671	635.727	626.365 Down
Sgo1	75.9454	0.88074	0.30731	2.86592	0.00416	0.01857	60.9526	53.6625	45.8344	90.1943	82.37	122.659 Up
Sult1c2	36.7958	2.67907	0.83045	3.22604	0.00126	0.00676	12.5775	17.2487	0	98.488	41.185	51.2754 Up
Sult1c1	83.7876	4.44617	2.05894	2.15945	0.03082	0.0936	0.9675	21.0817	0	327.602	5.28013	147.794 Up
Stap2	3090.93	0.41382	0.09669	4.28007	1.87E-05	0.00017	2555.17	2581.55	2814.99	3605.7	3662.3	3325.87 Up
Ubxn6	669.156	0.30573	0.14206	2.15203	0.03139	0.09492	684.991	556.748	553.832	704.967	781.459	732.937 Up
Plin4	1167.44	-3.0161	0.6144	-4.909	9.16E-07	1.14E-05	1351.6	3843.58	1038.91	200.086	239.718	330.777 Down
Plin5	533.437	-0.5606	0.28578	-1.9618	0.04979	0.1364	531.158	913.22	463.118	463.412	319.976	509.738 Down
Lrg1	36392.2	3.79641	0.2583	14.6978	6.66E-49	5.75E-46	6343.91	5534.9	2781.57	66464.9	76270.4	60957.5 Up
Sema6b	784.027	0.51702	0.16966	3.04745	0.00231	0.01123	737.236	630.534	567.2	912.31	1067.64	789.24 Up
Tnfaip8l1	155.212	1.22204	0.21027	5.81192	6.18E-09	1.17E-07	95.7826	80.4937	103.127	231.188	218.597	202.086 Up
Mydgf	2615.59	0.47664	0.1061	4.49224	7.05E-06	7.21E-05	2220.42	2111.04	2230.61	3290.54	3074.09	2766.86 Up
Dpp9	5639.37	0.70271	0.10929	6.42949	1.28E-10	3.34E-09	3903.87	4423.32	4550.02	7148.16	7375.28	6435.57 Up
Ticam1	939.565	0.32725	0.12166	2.68984	0.00715	0.02901	835.921	828.894	835.523	1167.34	988.44	981.271 Up
Plin3	4530.24	0.21776	0.09864	2.20762	0.02727	0.0853	4336.34	4146.39	4084.03	5137.96	5028.79	4447.89 Up
Arrdc5	5.63893	-3.3222	1.29353	-2.5683	0.01022	0.03892	14.5125	11.4991	4.77441	1.03672	0	2.0108 Down
Uhrf1	343.75	0.89582	0.22656	3.95402	7.68E-05	0.0006	240.908	243.398	236.811	463.412	319.976	557.997 Up
Gm20219	604.894	-0.6042	0.20051	-3.0131	0.00259	0.0124	570.826	772.356	846.026	561.9	409.738	468.517 Down
Rpl36	3867.54	-0.2526	0.12047	-2.097	0.03599	0.10555	4403.1	3702.71	4509.91	3707.3	3633.78	3248.45 Down
Catsperd	208.621	1.50291	0.20998	7.15744	8.22E-13	2.97E-11	113.198	114.033	99.3078	339.006	252.39	333.793 Up
Vmac	1748.22	0.48605	0.13506	3.59882	0.00032	0.0021	1666.04	1400.02	1303.42	2212.35	2003.28	1904.23 Up
Gm17949	18.59	-1.316	0.57762	-2.2784	0.0227	0.07374	16.4475	32.5808	30.5563	9.33044	10.5603	12.0648 Down
Crb3	2102.39	0.60355	0.12139	4.97209	6.62E-07	8.52E-06	1802.46	1529.38	1674.86	2748.33	2562.97	2296.34 Up
Dennd1c	1466.64	0.54651	0.12093	4.51939	6.20E-06	6.42E-05	1342.89	1090.5	1142.99	1708.51	1804.75	1710.19 Up
Tubb4a	1278.46	-1.2357	0.20542	-6.0155	1.79E-09	3.75E-08	1335.15	1981.68	2067.32	799.308	866.997	620.332 Down
Gm11110	131.717	0.52748	0.26499	1.99053	0.04653	0.12899	119.003	87.2015	117.451	130.626	193.253	142.767 Up
Tnfsf14	132.647	0.87615	0.29136	3.00711	0.00264	0.0126	123.84	91.9928	64.932	159.654	158.404	197.059 Up
C3	35078.9	0.83923	0.19248	4.36002	1.30E-05	0.00012	31337.4	24592.7	19533.1	45672.5	37363.2	51974.2 Up
Gpr108	4831.77	0.33183	0.08251	4.02178	5.78E-05	0.00047	4336.34	4261.38	4237.77	5595.16	5437.47	5122.52 Up
Vav1	1197.44	1.2813	0.19066	6.72035	1.81E-11	5.48E-10	855.271	697.612	541.419	1598.62	1949.42	1542.28 Up
Adgre1	1949.81	1.99847	0.29415	6.79406	1.09E-11	3.39E-10	1145.52	767.565	428.742	2611.49	3466.93	3278.61 Up
Cntnap5c	124.291	2.18173	0.36579	5.96448	2.45E-09	5.03E-08	67.7251	41.2051	25.7818	196.976	248.166	165.891 Up
Efna5	325.389	0.62025	0.19597	3.16504	0.00155	0.00805	287.348	277.895	204.345	351.447	394.953	436.344 Up
Man2a1	5657.87	0.58477	0.10049	5.81897	5.92E-09	1.13E-07	4544.35	4712.72	4322.75	6484.66	7406.96	6475.79 Up
Tmem232	1548.71	-0.6744	0.29925	-2.2535	0.02423	0.07767	1591.54	2446.43	1674.86	1236.8	671.632	1670.98 Down
Ndufv2	5259.16	-0.3242	0.14234	-2.2777	0.02274	0.07383	5180.97	7050.87	5311.06	4799.99	4584.21	4627.86 Down
Gm20742	3.51388	-5.2098	1.91531	-2.7201	0.00653	0.02694	1.935	14.3739	4.77441	0	0	0 Down
Themis3	39.0081	1.95536	0.4158	4.70264	2.57E-06	2.90E-05	10.6425	19.1652	18.1428	59.0928	69.6977	57.3078 Up
Arhgap28	1155.59	-0.7796	0.18548	-4.2032	2.63E-05	0.00023	1201.64	1305.15	1874.44	833.52	892.341	826.44 Down
Tgif1	1571.96	0.67252	0.09007	7.46667	8.22E-14	3.56E-12	1201.64	1250.53	1184.05	1962.5	1896.62	1936.4 Up
Myl12b	616.302	-0.365	0.14203	-2.5697	0.01018	0.03877	671.446	669.823	740.034	556.716	589.262	470.528 Down
Myom1	1777.06	-2.227	0.51008	-4.3659	1.27E-05	0.00012	1741.5	5142.97	1901.17	629.287	644.175	603.241 Down
Emilin2	864.888	0.70504	0.25239	2.79346	0.00521	0.02247	658.868	843.268	470.757	1085.44	1332.7	798.288 Up
Ndc80	105.553	0.60048	0.28288	2.12277	0.03377	0.10031	92.8801	97.7424	61.1125	143.067	109.827	128.691 Up
Pcare	21.9153	1.60205	0.61168	2.61912	0.00882	0.03443	3.87001	15.3321	13.3684	25.9179	33.7928	39.2106 Up
Clip4	6529.56	1.27193	0.17848	7.1264	1.03E-12	3.69E-11	3513	4814.29	3145.38	8065.65	9407.07	10232 Up
Ypel5	4118.24	0.4773	0.10216	4.67208	2.98E-06	3.32E-05	3704.56	3205.38	3419.44	4814.51	5037.24	4528.33 Up
Lbh	10486.8	-0.3416	0.13049	-2.618	0.00884	0.03451	12125.7	11773.2	11268.6	9093.03	10714.4	7945.68 Down
Capn13	121.264	-1.3657	0.50395	-2.7101	0.00673	0.02759	88.0426	235.732	200.525	55.9827	31.6808	115.621 Down
Ehd3	2841.46	1.70398	0.17648	9.65521	4.67E-22	4.85E-20	1316.77	1426.85	1260.45	3774.68	3716.15	5553.83 Up
Memo1	2477.98	0.29751	0.09531	3.12152	0.0018	0.00912	2084.97	2347.73	2237.29	2793.95	2766.79	2637.17 Up
Slc30a6	1197.03	0.20677	0.09551	2.16491	0.03039	0.09267	1148.42	1096.25	1089.52	1256.5	1312.64	1278.87 Up
Nlrc4	272.553	1.57372	0.21013	7.4893	6.92E-14	3.04E-12	160.605	147.572	103.127	406.393	382.281	435.339 Up
Ltbp1	4508.86	-0.3394	0.14949	-2.2702	0.0232	0.07512	4312.15	4907.24	5890.67	3744.62	4476.49	3721.99 Down
Fez2	3685.42	-0.2487	0.11545	-2.1542	0.03123	0.09449	3859.36	4090.81	4056.34	3328.89	3768.95	3008.16 Down
Vit	306.058	1.55023	0.27851	5.5662	2.60E-08	4.34E-07	148.028	151.405	168.059	502.807	280.903	585.143 Up
Strn	2348.13	0.24877	0.09048	2.74938	0.00597	0.02507	2089.8	2192.5	2156.13	2443.54	2681.25	2525.57 Up
Gm6548	1190.73	0.69671	0.09524	7.31515	2.57E-13	1.02E-11	921.061	912.262	892.816	1471.1	1433.03	1514.13 Up
Eif2ak2	1294.77	0.75396	0.14939	5.04692	4.49E-07	5.98E-06	1060.38	851.892	979.71	1461.77	1541.8	1873.06 Up
Sult6b1	95.3308	0.82414	0.24508	3.36273	0.00077	0.00449	68.6926	72.8277	64.932	135.81	115.107	114.616 Up
Ndufaf7	1849.37	-0.4311	0.10213	-4.221	2.43E-05	0.00022	1983.38	2144.58	2243.02	1614.17	1496.39	1614.67 Down
Prkd3	4556.76	-0.2937	0.11305	-2.5977	0.00938	0.03627	5346.41	4450.15	5260.45	3960.25	3975.94	4347.35 Down
Cdc42ep3	3012.99	0.69284	0.18041	3.84034	0.00012	0.00091	2688.69	2147.46	2073.05	3798.53	4438.47	2931.75 Up
Rmdn2	3598.48	0.58395	0.15203	3.84106	0.00012	0.00091	3270.15	2307.49	3062.31	4236.02	4637.01	4077.91 Up
Cyp1b1	906.72	-1.7369	0.17002	-10.216	1.68E-24	2.15E-22	1558.64	1483.38	1142.99	378.401	402.346	474.549 Down
Atl2	4429.01	0.29706	0.08662	3.42963	0.0006	0.00363	4151.55	4000.73	3771.79	4767.86	4846.1	5036.05 Up
Srsf7	8689.33	-0.3576	0.10222	-3.4985	0.00047	0.00293	9375.09	9204.07	10703.3	7986.86	7273.9	7592.79 Down
Gemin6	227.231	-0.3848	0.16871	-2.2807	0.02257	0.07333	267.998	250.106	253.999	179.352	203.813	208.118 Down
Arhgef33	190.386	-0.8572	0.23351	-3.6709	0.00024	0.00163	223.493	252.98	259.728	126.479	107.715	171.924 Down
Tmem178	1152.6	-0.695	0.15553	-4.4685	7.88E-06	7.94E-05	1440.61	1343.48	1490.57	799.308	1064.47	777.175 Down
Gm19689	28.6609	2.42472	0.97669	2.48259	0.01304	0.04747	18.3825	8.62433	0	60.1295	70.7537	14.0756 Up
Mta3	2291.22	0.48633	0.12212	3.98246	6.82E-05	0.00054	2054.01	2009.47	1662.45	2678.87	2806.92	2535.62 Up
4933433H	4.43149	3.68762	1.58686	2.32385	0.02013	0.06711	0	0	1.90977	3.11015	9.50423	12.0648 Up
Gm19696	31.7212	1.23441	0.59278	2.08241	0.03731	0.10854	30.96	18.2069	7.63906	49.7624	27.4567	56.3024 Up
Zfp36l2	7311.68	0.47359	0.14203	3.33451	0.00085	0.00487	6514.19	6058.11	5794.23	8433.68	9944.59	7125.28 Up
Plekhh2	32.4876	0.89607	0.42958	2.08591	0.03699	0.10786	21.285	18.2069	28.6465	49.7624	33.7928	43.2322 Up
Abcg8	16.7898	2.58464	0.67924	3.80522	0.00014	0.00103	3.87001	7.66607	2.86465	23.8845	24.2886	38.2052 Up
Slc3a1	834.274	-0.5884	0.14769	-3.9839	6.78E-05	0.00054	879.459	1164.28	962.522	688.379	628.335	682.667 Down
Camkmt	815.078	-0.2593	0.11585	-2.238	0.02522	0.08027	893.004	836.56	934.83	697.71	758.226	770.137 Down
Prkce	2327.84	-0.3639	0.16719	-2.1763	0.02954	0.0908	2231.06	2773.2	2855.1	1723.02	2435.19	1949.47 Down
Epas1	129152	-0.5583	0.10325	-5.4071	6.41E-08	9.88E-07	147201	143225	171077	103118	108296	101995 Down
Rhoq	462.537	0.50452	0.20661	2.4419	0.01461	0.05197	458.596	404.385	284.555	504.881	519.564	603.241 Up
Mcfd2	8161.35	0.65048	0.11976	5.43139	5.59E-08	8.80E-07	6902.16	6242.1	5911.68	10366.1	10738.7	8807.31 Up

Ttc7	4171.26	0.99422	0.14956	6.64781	2.97E-11	8.62E-10	3221.78	2704.21	2438.77	5454.16	6301.3	4907.36	Up
Epcam	6475.54	0.61285	0.15627	3.9216	8.80E-05	0.00068	4891.69	5045.23	5424.69	8243.96	6160.85	9086.81	Up
Gm31499	3.80228	4.40243	1.69503	2.59726	0.0094	0.0363	0.9675	0	0	4.14686	12.6723	5.027	Up
Gm31615	45.6397	-1.177	0.48058	-2.4491	0.01432	0.05115	33.8626	81.452	74.4809	25.9179	38.0169	20.108	Down
Ston1	3830.35	-0.5709	0.15891	-3.5928	0.00033	0.00214	4509.52	5060.56	4165.2	3321.64	3467.99	2457.2	Down
Lhcgr	22.7995	-2.1846	0.6494	-3.364	0.00077	0.00447	21.285	48.8712	42.0148	11.4039	3.16808	10.054	Down
Nrxn1	63.5485	-0.8912	0.37661	-2.3663	0.01797	0.0614	69.6601	110.2	67.7967	54.9459	47.5211	31.1674	Down
Gm20939	165.473	0.68097	0.21425	3.17846	0.00148	0.00776	138.353	120.741	122.225	194.903	238.662	177.956	Up
Crem	1654.92	-0.4867	0.19132	-2.5437	0.01097	0.04121	1472.54	1896.39	2425.4	1269.98	1339.04	1526.2	Down
Bambi	1453.36	-0.5531	0.16485	-3.3551	0.00079	0.00458	1970.8	1537.05	1677.73	1343.58	1220.77	970.212	Down
Map3k8	1381.33	0.23237	0.08957	2.59437	0.00948	0.03654	1280.97	1253.4	1276.68	1484.58	1474.21	1518.16	Up
Jcad	16171.8	-0.379	0.10236	-3.7025	0.00021	0.00147	16872.3	17670.3	20308.4	14172.9	14100.1	13906.7	Down
Gm10556	138.995	8.75797	1.13295	7.73021	1.07E-14	5.14E-13	1.935	0	0	496.587	139.395	196.053	Up
Gm26682	18.302	-2.203	0.58918	-3.7391	0.00018	0.00131	28.0575	29.706	32.466	4.14686	7.39218	8.04321	Down
Zfp438	461.362	-0.398	0.1347	-2.955	0.00313	0.01459	520.516	527.042	526.14	430.237	401.29	362.95	Down
Gm10125	244.699	-0.6106	0.17774	-3.4356	0.00059	0.00357	280.575	299.935	306.517	180.389	222.821	177.956	Down
Zeb1	4593.53	-0.2694	0.12353	-2.1812	0.02917	0.08998	5031.01	4726.13	5306.28	3834.81	4769.01	3893.92	Down
Epc1	2830.95	-0.2924	0.11665	-2.5067	0.01219	0.04495	3169.53	2775.12	3406.07	2451.83	2481.66	2701.51	Down
Mkx	24.7106	-1.3152	0.51858	-2.5362	0.0112	0.04195	44.5051	26.8312	34.3758	15.5507	17.9524	9.04861	Down
Armc4	1167.62	-0.5548	0.24588	-2.2566	0.02403	0.0772	1214.21	1589.75	1364.53	949.632	617.775	1269.82	Down
Greb1l	277.498	-2.5944	0.20884	-12.423	1.96E-35	6.40E-33	455.693	450.382	522.321	61.1662	93.9863	81.4375	Down
Esco1	2039.68	0.40068	0.1076	3.7239	0.0002	0.00137	1754.08	1684.62	1836.24	2342.98	2125.78	2494.4	Up
Gata6os	817.974	-0.3038	0.12918	-2.352	0.01867	0.06331	879.459	879.681	952.018	789.977	758.226	648.484	Down
Rbbp8	2481.31	1.11683	0.15626	7.1473	8.85E-13	3.19E-11	1876.95	1300.36	1521.13	3605.7	3467.99	3115.74	Up
Gm6277	225.111	-0.4452	0.2213	-2.0119	0.04423	0.12391	275.738	259.688	243.495	185.572	148.9	237.275	Down
Tmem241	848.345	0.24067	0.10575	2.27575	0.02286	0.07416	769.164	796.313	767.726	962.072	905.014	889.78	Up
Gm25289	2.23579	-4.5576	2.18815	-2.0828	0.03727	NA	0.9675	9.58259	2.86465	0	0	0	Down
Rmc1	2806.69	0.44976	0.09672	4.65028	3.31E-06	3.66E-05	2317.17	2318.99	2481.74	3325.78	3378.23	3018.21	Up
Npc1	2572.08	1.00652	0.1326	7.59034	3.19E-14	1.45E-12	1927.26	1538.96	1662.45	3148.51	3793.24	3362.06	Up
Ankrd29	1069.13	-0.3422	0.16877	-2.0274	0.04262	0.12041	1021.68	1144.16	1419.91	952.742	1052.86	823.423	Down
Lama3	6744.25	0.65441	0.15687	4.17157	3.03E-05	0.00026	6075.91	4288.21	5356.89	7637.49	9335.26	7771.75	Up
Ttc39c	810.877	-0.6046	0.13847	-4.3661	1.26E-05	0.00012	854.304	1097.21	983.529	635.507	624.111	670.602	Down
Zfp521	632.938	-0.5136	0.14737	-3.485	0.00049	0.00306	830.116	688.03	715.207	462.375	544.909	556.992	Down
8430422H	2.07587	4.55412	2.08044	2.18902	0.0286	NA	0	0	0	6.2203	4.2241	2.0108	Up
Taf4b	547.313	0.61747	0.13028	4.73949	2.14E-06	2.46E-05	394.741	458.048	443.066	641.727	651.568	694.732	Up
Kctd1	937.899	0.90639	0.12844	7.05688	1.70E-12	5.91E-11	631.778	710.07	615.899	1342.55	1186.97	1140.12	Up
A830021F	7.64166	2.79435	1.28408	2.17615	0.02954	0.0908	3.87001	1.91652	0	24.8812	2.11205	13.0702	Up
Aqp4	1539.18	1.4974	0.28455	5.2623	1.42E-07	2.05E-06	614.363	1147.99	653.14	2633.26	1506.95	2679.39	Up
Gm7665	791.8	0.65478	0.10957	5.97619	2.28E-09	4.71E-08	607.591	633.409	604.441	1014.94	928.246	962.169	Up
Cdh2	323.899	-1.4813	0.32088	-4.6163	3.91E-06	4.24E-05	351.203	738.817	340.893	142.03	177.412	193.037	Down
Gm15328	2.23579	-4.5576	2.18815	-2.0828	0.03727	NA	0.9675	9.58259	2.86465	0	0	0	Down
Dsc2	285.206	-1.1683	0.40097	-2.9138	0.00357	0.01634	284.445	668.865	231.082	163.801	127.779	235.264	Down
Dsg1a	10.9223	3.37784	0.99789	3.38497	0.00071	0.00419	0	3.83303	1.90977	10.3672	24.2886	25.135	Up
Gm16090	138.235	-0.6849	0.26219	-2.6123	0.00899	0.03498	142.223	179.194	190.022	116.112	79.2019	122.659	Down
Mep1b	4.27627	3.62899	1.52498	2.37969	0.01733	0.05958	0	1.91652	0	10.3672	6.33615	7.03781	Up
Dtna	2378.61	-0.4491	0.1344	-3.3417	0.00083	0.00477	2426.49	3200.58	2610.65	1930.36	2003.28	2100.28	Down
Zscan30	502.828	-0.4651	0.17553	-2.6499	0.00805	0.03199	540.833	557.707	651.23	408.466	359.049	499.684	Down
Ino80c	2352.8	0.32728	0.09381	3.48894	0.00048	0.00302	1970.8	2170.46	2119.84	2526.48	2718.21	2611.03	Up
Galnt1	7052.92	0.31482	0.07919	3.97562	7.02E-05	0.00056	6217.16	6326.42	6315.6	7481.98	8127.17	7849.16	Up
Gm38410	27.1281	0.98025	0.48257	2.03132	0.04222	0.11956	22.2525	20.1234	12.4135	36.2851	27.4567	44.2376	Up
Fhod3	217.889	-2.878	0.36809	-7.8187	5.34E-15	2.71E-13	292.185	609.453	249.224	53.9092	42.241	60.3241	Down
Tpgs2	943.884	-0.2604	0.10207	-2.551	0.01074	0.04058	1064.25	1032.04	990.214	840.777	874.389	861.629	Down
AW55491:	1070.74	-0.2812	0.10981	-2.5612	0.01043	0.03959	1189.06	1229.45	1105.75	924.751	1024.34	951.109	Down
Celf4	248.311	0.795	0.27352	2.90654	0.00365	0.01664	211.883	191.652	141.323	291.317	234.438	419.252	Up
Syt4	3.1939	-5.0721	1.91524	-2.6483	0.00809	0.03211	1.935	11.4991	5.7293	0	0	0	Down
Wdr33	4501.11	-0.2906	0.0963	-3.0176	0.00255	0.01224	4677.87	4860.29	5320.61	3884.57	4046.69	4216.65	Down
Lims2	6788.8	-0.7429	0.0784	-9.4752	2.66E-21	2.62E-19	8220.86	8762.32	8513.74	5215.72	4998.17	5021.98	Down
Bin1	2321.89	-0.3673	0.10567	-3.476	0.00051	0.00315	2506.8	2459.85	2880.88	2055.81	2042.35	1985.67	Down
Gypc	977.775	-0.8705	0.14851	-5.8613	4.59E-09	9.01E-08	1339.99	1384.68	1067.56	713.261	730.77	630.386	Down
Stard4	2931.5	0.98655	0.13968	7.06313	1.63E-12	5.67E-11	2039.49	1960.6	1899.26	3645.09	4621.17	3423.39	Up
Nrep	2285.36	-0.5535	0.18305	-3.024	0.00249	0.01202	2199.13	2690.79	3265.7	1827.73	1568.2	2160.61	Down
Apc	6469.6	0.59564	0.09421	6.32267	2.57E-10	6.35E-09	5147.11	4960.91	5350.21	7216.58	7929.69	8213.12	Up
Brd8dc	210.086	-0.5321	0.2586	-2.0578	0.03961	0.11363	189.63	229.024	326.57	150.324	167.908	197.059	Down
Gfra3	12.9546	-1.9075	0.9065	-2.1043	0.03535	0.10407	12.5775	39.2886	9.54883	4.14686	2.11205	10.054	Down
Cdc25c	22.1833	1.56386	0.52096	3.0019	0.00268	0.01278	13.545	9.58259	10.5037	37.3218	38.0169	24.1296	Up
Kdm3b	4012.11	0.24883	0.09054	2.74812	0.00599	0.02514	3880.65	3434.4	3685.85	4359.39	4437.42	4274.96	Up
Etf1	8694.85	0.20467	0.08259	2.47821	0.0132	0.04794	8330.19	7878.8	8028.66	9099.26	9039.58	9792.6	Up
Sil1	1556	0.20687	0.09548	2.16649	0.03027	0.09239	1514.14	1398.1	1421.82	1667.04	1614.66	1720.24	Up
1700066B	108.222	0.83595	0.29374	2.84586	0.00443	0.01956	64.8226	66.1198	102.172	114.039	158.404	143.772	Up
Sting1	3027.59	1.87617	0.11504	16.3082	8.63E-60	1.55E-56	1299.35	1331.98	1257.58	4485.87	5351.94	4438.84	Up
Cxxc5	941.258	-0.7301	0.16676	-4.3782	1.20E-05	0.00011	1070.06	1440.26	1013.13	744.362	677.968	701.77	Down
Igip	3725.7	-0.2419	0.111	-2.1792	0.02931	0.09031	4484.37	3818.66	3809.03	3236.63	3416.24	3589.28	Down
Cystm1	6690.86	0.6258	0.08092	7.73347	1.05E-14	5.03E-13	5169.36	5205.26	5411.32	8194.2	8392.23	7772.75	Up
Pfdn1	1648.79	0.2066	0.09895	2.08793	0.0368	0.10748	1479.31	1479.55	1633.8	1800.78	1793.13	1706.17	Up
Hbegf	2864.88	0.94929	0.18491	5.13385	2.84E-07	3.93E-06	1933.07	1727.74	2203.87	3154.73	4802.8	3367.09	Up
Cd14	6801.79	1.71631	0.12403	13.8383	1.50E-43	9.23E-41	2745.77	3599.22	3176.9	10273.9	10866.5	10148.5	Up
Dnd1	74.7268	-0.6215	0.28878	-2.1522	0.03138	0.0949	92.8801	76.6607	102.172	50.7991	66.5296	59.3187	Down
Diaph1	10403.6	0.41525	0.11035	3.7629	0.00017	0.0012	9386.7	8260.19	9102.9	11828.9	13005	10838.2	Up
Fchsd1	260.865	0.51122	0.17852	2.86367	0.00419	0.01869	233.168	188.777	223.443	340.043	286.183	293.577	Up
Arap3	11102.8	-0.4534	0.14654	-3.094	0.00197	0.00987	11516.2	11481.9	15501.6	8674.2	9874.89	9568.4	Down
Pcdh1	5829.55	-0.3848	0.13397	-2.8719	0.00408	0.01829	6257.8	5762.97	7786.12	4958.61	5178.75	5033.04	Down
Dele1	2564.14	-0.2837	0.08598	-3.3	0.00097	0.00543	2763.18	2909.27	2773.93	2232.05	2371.83	2334.54	Down
Gnpda1	1477.61	0.72752	0.13999	5.19679	2.03E-07	2.87E-06	1210.34	1206.45	921.462	1874.38	1874.45	1778.55	Up

Spry4	1863.94	0.32984	0.12276	2.68683	0.00721	0.02922	1452.22	1800.57	1702.56	1946.95	2125.78	2155.58	Up
Arhgap26	208.744	0.37338	0.18719	1.99467	0.04608	0.128	202.208	170.57	172.834	257.106	237.606	212.14	Up
Gm41715	17.0097	-1.9048	0.87024	-2.1889	0.02861	0.08868	13.545	56.5373	10.5037	5.18358	4.2241	12.0648	Down
Dcp2	2846.65	0.23723	0.10695	2.21804	0.02655	0.08357	2696.43	2494.35	2648.85	2868.59	2987.5	3384.18	Up
Mcc	4651.37	-0.3031	0.10476	-2.8934	0.00381	0.01726	5323.19	4653.3	5438.06	4027.64	4395.18	4070.87	Down
Kcnn2	134.913	-1.1611	0.337	-3.4453	0.00057	0.00347	224.46	219.441	115.541	92.2677	61.2495	96.5185	Down
Trim36	333.3	0.6667	0.15102	4.41465	1.01E-05	9.90E-05	253.485	240.523	278.826	388.768	434.026	404.171	Up
Ticam2	237.971	1.39179	0.21345	6.52034	7.01E-11	1.91E-09	153.833	137.989	102.172	337.969	380.169	315.696	Up
Eif1a	6097.63	0.40115	0.09338	4.29582	1.74E-05	0.00016	4965.22	5124.77	5675.82	6811.22	7140.84	6867.89	Up
Cdo1	11345	-0.6085	0.09315	-6.5321	6.48E-11	1.77E-09	14394.5	14138.1	12574.9	9132.43	9107.16	8722.86	Down
Atg12	3453.81	0.24853	0.10099	2.46094	0.01386	0.04988	3280.8	3009.89	3180.71	3860.73	3440.53	3950.22	Up
Ap3s1	2675.57	0.28161	0.11517	2.4451	0.01448	0.0516	2271.69	2712.83	2261.16	3018.92	2992.78	2796.02	Up
Sema6a	2959.95	-0.3213	0.12748	-2.5204	0.01172	0.04355	3608.78	3041.51	3214.14	2472.57	2969.54	2453.18	Down
1700065C	4.92645	3.84978	1.56082	2.46651	0.01364	0.04925	0	1.91652	0	11.4039	3.16808	13.0702	Up
Hsd17b4	7953.93	0.23024	0.09688	2.3765	0.01748	0.05996	7829.02	7465.79	6666.99	8756.1	8521.07	8484.58	Up
Snx2	7181.41	0.31442	0.09622	3.26782	0.00108	0.00597	6531.6	6462.5	6211.51	7738.05	8663.63	7481.19	Up
Snx24	1303.22	0.86834	0.15412	5.63411	1.76E-08	3.04E-07	990.722	927.594	848.891	1706.43	1944.14	1401.53	Up
Ppic	7215.77	0.77388	0.09153	8.45486	2.79E-17	1.94E-15	5661.82	4986.78	5328.25	9355.32	8766.07	9196.4	Up
Prdm6	1256.67	-0.9651	0.18158	-5.3152	1.07E-07	1.57E-06	1478.34	1653.95	1853.43	810.712	1052.86	690.71	Down
Aldh7a1	3463.11	-0.2559	0.09638	-2.6547	0.00794	0.03162	3801.31	3775.54	3731.68	3176.5	2911.46	3382.17	Down
Lmnb1	1139.28	0.2904	0.13177	2.2039	0.02753	0.08594	1086.5	1007.13	981.62	1202.59	1134.17	1423.65	Up
4930511M	11.518	-1.7984	0.73319	-2.4529	0.01417	0.05078	11.61	21.0817	21.0074	4.14686	4.2241	7.03781	Down
Gm19519	26.4389	1.41817	0.51539	2.75164	0.00593	0.02494	12.5775	19.1652	11.4586	24.8812	43.297	47.2538	Up
Slc12a2	6529.35	-0.3929	0.1495	-2.6283	0.00858	0.03368	5993.67	8449.92	7795.66	5484.23	5328.7	6123.9	Down
Slc27a6	266.087	0.83578	0.31183	2.68026	0.00736	0.02972	282.51	139.906	150.871	302.721	432.97	287.545	Up
Adamts19	51.8428	-3.4053	0.58353	-5.8356	5.36E-09	1.03E-07	79.3351	158.113	46.7893	12.4406	6.33615	8.04321	Down
Chsy3	434.834	0.65712	0.2468	2.66256	0.00775	0.03102	343.463	437.924	231.082	615.809	470.987	509.738	Up
Gm4951	302.934	1.85056	0.41179	4.49394	6.99E-06	7.16E-05	212.85	88.1598	93.5785	328.639	744.498	349.88	Up
Gm4841	43.2834	2.40175	0.44003	5.45811	4.81E-08	7.66E-07	19.35	13.4156	8.59395	79.8271	58.0814	80.4321	Up
Iigp1	1779.25	1.43203	0.23964	5.97566	2.29E-09	4.71E-08	1237.43	848.059	801.147	1912.74	3388.79	2487.36	Up
BC023105	62.3377	2.06502	0.43028	4.79928	1.59E-06	1.88E-05	39.6676	17.2487	15.2781	89.1576	95.0423	117.632	Up
Synpo	5074.76	-0.3597	0.17747	-2.0269	0.04267	0.12052	4301.51	5751.47	7059.45	4268.16	4452.2	4615.8	Down
Cd74	97993.1	1.53009	0.15473	9.88893	4.65E-23	5.25E-21	61620.2	44976.8	44625.5	152627	153380	130728	Up
Camk2a	128.876	-0.9562	0.2941	-3.2513	0.00115	0.00628	196.403	201.234	112.676	85.0107	83.426	94.5077	Down
Slc6a7	21.9963	-1.4663	0.58134	-2.5223	0.01166	0.04337	22.2525	50.7877	23.8721	12.4406	10.5603	12.0648	Down
Pdgfrb	5593.45	-0.4567	0.17262	-2.6455	0.00816	0.03233	5577.65	6506.58	7329.68	4196.63	5825.04	4125.16	Down
Hmgxb3	1428.53	0.37146	0.12596	2.94901	0.00319	0.01485	1349.66	1147.04	1240.39	1492.87	1557.64	1783.58	Up
Arhgef37	158.275	-0.734	0.33139	-2.215	0.02676	0.08406	143.19	298.977	150.871	131.663	120.387	104.562	Down
Bvht	1567.06	-0.4825	0.18623	-2.5909	0.00957	0.03684	1904.04	1821.65	1754.12	1200.52	1686.47	1035.56	Down
Grpel2	3447.92	0.23733	0.09614	2.46867	0.01356	0.04903	3339.82	3013.72	3141.56	3699	3547.19	3946.2	Up
Ablim3	4286.88	-0.4681	0.10629	-4.4037	1.06E-05	0.0001	4878.14	4663.84	5386.49	3505.14	3869.28	3418.36	Down
Adrb2	4366.66	-0.5068	0.16446	-3.0815	0.00206	0.01021	5266.11	5115.18	4995.95	4452.69	3498.61	2871.42	Down
Apcdd1	402.248	-0.451	0.1802	-2.5029	0.01232	0.04535	530.191	386.178	477.441	377.365	332.648	309.663	Down
Wdr7	1668.04	0.43709	0.10642	4.10731	4.00E-05	0.00034	1515.11	1345.4	1391.26	1833.95	2043.41	1879.09	Up
Amd-ps3	90.6965	0.80863	0.26203	3.08597	0.00203	0.01009	69.6601	54.6207	73.526	114.039	106.659	125.675	Up
Alpk2	248.514	2.40954	0.39928	6.03468	1.59E-09	3.38E-08	61.9201	137.989	36.2855	425.053	385.449	444.387	Up
Malt1	5425.29	2.53169	0.15217	16.6376	3.72E-62	8.03E-59	1889.53	1490.09	1419.91	8318.61	8918.13	10515.5	Up
Gm41760	31.7178	-0.8358	0.41738	-2.0026	0.04522	0.12605	37.7326	43.1216	41.06	23.8445	27.4567	17.0918	Down
Oacyl	35.3794	1.05264	0.43624	2.41298	0.01582	0.05541	14.5125	29.706	24.827	41.4686	46.4651	55.297	Up
Sec11c	4419.11	0.8842	0.08711	10.1507	3.29E-24	4.05E-22	3055.37	3173.75	3088.09	6054.42	5686.7	5456.31	Up
Gm15958	27.8865	-1.0338	0.45273	-2.2834	0.02241	0.0729	37.7326	40.2469	34.3758	18.6609	23.2326	13.0702	Down
Gm9926	59.3489	7.52567	1.11243	6.7651	1.33E-11	4.10E-10	1.935	0	0	172.095	106.659	75.4051	Up
Gm50162	1.8822	4.41447	2.21576	1.99231	0.04634	NA	0	0	0	1.03672	4.2241	6.03241	Up
Impa2	371.431	0.74647	0.22318	3.34471	0.00082	0.00473	327.015	296.102	209.119	466.522	541.741	388.085	Up
Psmg2	1316.94	-0.2422	0.09748	-2.4845	0.01297	0.04727	1355.47	1450.8	1475.29	1232.66	1208.09	1179.34	Down
Ptpn2	2293.53	0.33828	0.10111	3.34578	0.00082	0.00471	1916.62	2091.88	2069.23	2618.74	2380.28	2684.42	Up
Ldlrad4	2191.43	0.42321	0.10148	4.17053	3.04E-05	0.00026	1896.3	1967.31	1753.16	2409.33	2662.24	2460.22	Up
Tcf4	6770.98	-0.2815	0.08419	-3.344	0.00083	0.00474	7465.24	7254.02	7569.36	5763.1	6325.59	6248.57	Down
4930448D	32.7376	-1.0488	0.47472	-2.2092	0.02716	0.08503	37.7326	41.2051	53.4734	32.1382	14.7844	17.0918	Down
Rab27b	1825.2	0.56453	0.14722	3.83449	0.00013	0.00093	1245.17	1490.09	1682.5	2000.86	2143.73	2388.83	Up
Gm45879	2.88119	3.98624	1.8166	2.19434	0.02821	0.08773	0	0	0.95488	3.11015	3.16808	10.054	Up
Mex3c	3867.28	0.22547	0.09835	2.29259	0.02187	0.07154	3542.99	3298.33	3855.82	4211.14	4065.7	4229.72	Up
Ska1	35.8628	1.35508	0.41249	3.2851	0.00102	0.00567	17.415	26.8312	16.233	54.9459	46.4651	53.2862	Up
Myo5b	4682.23	0.55058	0.10321	5.33446	9.58E-08	1.42E-06	4087.69	3879.03	3431.85	5413.73	5547.3	5733.8	Up
Acaa2	8578.89	-0.624	0.17746	-3.5162	0.00044	0.00276	10825.4	12571.4	7820.49	7364.83	6606.49	6284.76	Down
Lipg	2414.95	-0.5242	0.11875	-4.4142	1.01E-05	9.92E-05	2499.06	3053.01	2994.51	2002.94	2056.08	1884.12	Down
9030625G	4.33925	-3.5199	1.68778	-2.0855	0.03702	0.10795	2.9025	3.83303	17.1879	0	2.11205	0	Down
Rpl17	12201.1	-0.216	0.10912	-1.9793	0.04778	0.13179	13269.3	11752.1	14316.6	11803	11364.9	10700.5	Down
Dym	3282.09	0.32342	0.11117	2.9092	0.00362	0.01654	3099.87	2987.85	2659.35	3757.06	3833.37	3355.02	Up
2010010A	2.70518	3.89014	1.87486	2.0749	0.038	0.11008	0	0	0.95488	3.11015	2.11205	10.054	Up
Smad7	3708.72	-0.4105	0.16127	-2.5456	0.01091	0.04106	3905.8	4061.1	4731.44	3545.57	3483.83	2524.56	Down
Zbtb7c	303.887	-0.5411	0.19371	-2.7934	0.00522	0.02247	387.968	405.343	287.42	257.106	228.101	257.383	Down
Smad2	3495.89	0.35791	0.10988	3.25726	0.00112	0.00617	3403.67	2822.07	2967.78	4013.13	3753.11	4015.57	Up
Gm7276	4.31634	-5.5065	1.66418	-3.3088	0.00094	0.00528	5.80501	11.4991	8.59395	0	0	0	Down
St8sia5	12.6199	-1.9763	0.85823	-2.3028	0.02129	0.07003	11.61	32.5808	16.233	2.07343	3.16808	10.054	Down
Haus1	513.203	-0.6233	0.1263	-4.9351	8.01E-07	1.01E-05	585.338	620.952	660.779	409.503	405.514	397.133	Down
Atp5a1	32285.9	-0.3605	0.16192	-2.2267	0.02597	0.0821	33647.8	45487.6	29762.7	28729.5	28561.3	27526.9	Down
Pstpip2	591.289	0.91645	0.20944	4.37565	1.21E-05	0.00012	429.571	493.503	305.563	693.563	897.622	727.91	Up
Epg5	1330.02	0.20223	0.09929	2.03672	0.04168	0.11835	1219.05	1218.91	1272.86	1345.66	1487.94	1435.71	Up
Slc14a1	308.777	-0.427	0.21268	-2.0077	0.04468	0.12494	314.438	408.218	339.938	244.665	219.653	325.75	Down
Slc14a2	324.163	-0.9122	0.41345	-2.2062	0.02737	0.08555	217.688	605.619	446.885	196.976	130.947	346.863	Down
Pard6g	2023.95	-0.6225	0.12542	-4.9636	6.92E-07	8.88E-06	2192.36	2360.19	2809.27	1579.95	1609.38	1592.55	Down
Kcng2	98.3393	-4.9454	0.65135	-7.5925	3.14E-14	1.43E-12	82.2376	376.596	112.676	4.14686	6.33615	8.04321	Down

D330025C	11.8578	-5.0626	1.32841	-3.8111	0.00014	0.00101	13.545	41.2051	14.3232	2.07343	0	0	Down
Sall3	2.87253	-4.919	2.46188	-1.9981	0.04571	0.12713	0	13.4156	3.81953	0	0	0	Down
Mbp	3906.42	0.67489	0.12077	5.58832	2.29E-08	3.86E-07	3441.4	2888.19	2697.54	4662.11	4863	4886.25	Up
Gm41793	8.61284	-1.8333	0.84979	-2.1574	0.03098	0.09399	18.3825	7.66607	14.3232	3.11015	3.16808	5.027	Down
Zfp516	1742.11	0.23726	0.11656	2.03553	0.0418	0.11864	1667.01	1548.55	1582.24	1798.7	1749.83	2106.31	Up
4933401L	39.1089	1.12094	0.38801	2.88897	0.00387	0.01746	24.1875	23.9565	25.7818	41.4686	54.9133	64.3457	Up
Zadh2	3407.98	0.24295	0.11044	2.19981	0.02782	0.08672	3413.35	3031.93	2920.03	3458.48	3672.86	3951.23	Up
Cndp2	6751.23	0.89374	0.12892	6.93235	4.14E-12	1.35E-10	5457.68	4479.86	4235.86	8757.14	9362.72	8214.13	Up
Socs6	2069.21	0.25477	0.10649	2.3925	0.01673	0.05801	1763.76	2021.93	1875.39	2191.62	2161.68	2400.9	Up
Cd226	384.091	-0.5256	0.19004	-2.7656	0.00568	0.02411	376.358	550.999	432.562	299.611	305.191	339.825	Down
Tmx3	7251.97	0.27517	0.07841	3.50915	0.00045	0.00283	6391.31	6570.78	6725.24	7685.17	8192.64	7946.69	Up
Gm45871	746.937	0.71841	0.20477	3.50836	0.00045	0.00284	523.418	596.037	574.84	897.796	700.145	1189.39	Up
Kmt5b	131.979	0.52556	0.24987	2.1033	0.03544	0.10424	122.873	82.4102	119.36	168.985	139.395	158.853	Up
Tcirg1	6309.69	0.74657	0.10235	7.29455	3.00E-13	1.17E-11	4815.25	4562.27	4760.09	8195.24	8408.07	7117.23	Up
Unc93b1	5944.64	0.56486	0.09836	5.74272	9.32E-09	1.71E-07	5061	4925.45	4400.1	7013.38	7430.19	6837.73	Up
Aldh3b3	6.12149	-2.976	1.23768	-2.4045	0.01619	0.05647	2.9025	20.1234	9.54883	1.03672	2.11205	1.0054	Down
Aldh3b2	28.2678	-1.647	0.51484	-3.199	0.00138	0.00731	38.7001	51.746	38.1953	11.4039	8.4482	21.1134	Down
Doc2g	378.821	-2.3984	0.67569	-3.5495	0.00039	0.00247	300.893	1273.53	336.119	118.186	123.555	120.648	Down
Ndufv1	5297.59	-0.5569	0.15103	-3.6875	0.00023	0.00155	5514.76	7721.65	5686.33	4470.32	4118.5	4273.96	Down
Gstp1	6481.83	-0.4949	0.08847	-5.5941	2.22E-08	3.74E-07	7919.97	7326.85	7501.56	5694.68	5238.94	5208.98	Down
Gstp2	213.626	-0.4677	0.22341	-2.0936	0.03629	0.10628	313.47	206.984	223.443	170.021	185.86	181.978	Down
Gstp3	492.512	1.10674	0.2237	4.9475	7.52E-07	9.58E-06	301.86	351.681	283.6	781.684	474.155	762.094	Up
Pitpnm1	3057.57	0.37711	0.11099	3.39773	0.00068	0.00403	2783.5	2577.72	2619.24	3421.16	3806.97	3136.85	Up
Coro1b	6220.73	0.7364	0.14234	5.17366	2.30E-07	3.21E-06	5340.61	4209.63	4449.75	7749.45	8751.28	6823.66	Up
Carns1	350.034	-0.6234	0.26069	-2.3915	0.01678	0.05812	408.286	565.373	299.833	230.151	322.088	274.474	Down
Ppp1ca	9989.27	0.27819	0.10674	2.60614	0.00916	0.03549	9964.3	8442.26	8680.84	11160.2	11418.8	10269.2	Up
Ssh3	3872.09	0.59517	0.09551	6.23139	4.62E-10	1.08E-08	3241.13	2853.69	3158.75	4838.35	4603.21	4537.37	Up
Grk2	6983.14	0.23006	0.11156	2.06223	0.03919	0.1127	7064.7	5967.08	6250.66	7668.59	8001.5	6946.31	Up
Rhod	627.271	0.36025	0.16094	2.23846	0.02519	0.08019	476.978	622.868	548.103	779.61	710.705	625.359	Up
A930001C	60.8087	2.20022	0.42575	5.1679	2.37E-07	3.31E-06	16.4475	19.1652	29.6014	85.0107	72.8658	141.762	Up
Syt12	149.522	2.039	0.30304	6.72854	1.71E-11	5.18E-10	44.5051	63.2451	67.7967	166.911	287.239	267.437	Up
Pcx	5297.98	0.92269	0.15849	5.8216	5.83E-09	1.11E-07	3294.34	4465.49	3217.96	6165.35	7341.49	7303.23	Up
Sptbn2	161.919	-0.5945	0.27317	-2.1762	0.02954	0.0908	181.89	183.027	219.623	158.618	86.5941	141.762	Down
Gm21992	1484.39	-0.2863	0.11375	-2.5171	0.01183	0.04387	1548	1551.42	1794.22	1275.16	1337.98	1399.52	Down
Dpp3	2942.69	0.57813	0.08489	6.81048	9.73E-12	3.05E-10	2471	2298.86	2312.73	3589.11	3479.6	3504.83	Up
Peli3	451.571	0.8118	0.20666	3.92821	8.56E-05	0.00066	347.333	321.975	314.156	615.809	423.466	686.689	Up
Npas4	23.5131	-2.0248	0.5373	-3.7685	0.00016	0.00118	33.8626	43.1216	36.2855	5.18358	10.5603	12.0648	Down
Slc29a2	123.96	-1.0638	0.24797	-4.29	1.79E-05	0.00016	155.768	187.819	159.465	64.2764	93.9863	82.4429	Down
Rin1	517.962	0.61299	0.16218	3.77969	0.00016	0.00113	374.423	392.886	461.208	568.12	714.929	596.203	Up
Cd248	321.234	-1.0132	0.21029	-4.8178	1.45E-06	1.73E-05	342.496	531.834	414.419	206.306	231.27	201.08	Down
Tmem151	553.544	0.7627	0.32753	2.32865	0.01988	0.0665	637.583	295.144	298.878	807.602	800.467	481.587	Up
Rab1b	9422.17	0.44116	0.11599	3.80354	0.00014	0.00104	8655.27	8047.46	7275.25	10862.7	11803.2	9889.12	Up
Klc2	1823.85	0.28202	0.10384	2.71594	0.00661	0.02718	1579.93	1667.37	1691.1	2168.81	1914.57	1921.32	Up
D330050I	42.618	-0.8399	0.42821	-1.9614	0.04984	0.13647	41.6026	73.7859	48.699	37.3218	21.1205	33.1782	Down
Ctsw	263.129	-0.556	0.17715	-3.1387	0.0017	0.00868	271.868	347.848	319.886	200.086	224.933	214.15	Down
Efemp2	3042.89	0.30725	0.08989	3.41794	0.00063	0.00378	2689.65	2675.46	2794.94	3396.28	3523.96	3177.07	Up
Ap5b1	289.739	0.87005	0.19929	4.36585	1.27E-05	0.00012	226.395	178.236	210.074	344.19	447.755	331.782	Up
Sipa1	4771.32	-0.3239	0.13788	-2.3493	0.01881	0.06369	5084.22	4709.84	6119.84	4139.61	4684.53	3889.9	Down
Ltbp3	6194.97	-0.2511	0.09511	-2.6402	0.00829	0.03275	6817.98	6375.29	7005.02	6047.16	5362.5	5561.88	Down
Scyl1	4996.48	0.28906	0.11081	2.60862	0.00909	0.03529	4828.8	4121.47	4542.38	5813.9	5641.29	5031.03	Up
Neat1	131154	0.31483	0.12192	2.58227	0.00982	0.03762	100430	123707	126563	135696	152439	148090	Up
Frmd8os	16.4006	-2.0053	0.61544	-3.2584	0.00112	0.00614	28.0575	31.6225	19.0977	7.25701	6.33615	6.03241	Down
Frmd8	3027.29	0.20839	0.09744	2.13861	0.03247	0.09725	2953.78	2650.54	2822.63	3266.69	3404.63	3065.47	Up
Slc25a45	1543.05	0.61428	0.11906	5.1592	2.48E-07	3.46E-06	1224.86	1155.66	1277.63	1710.58	2069.81	1819.78	Up
Cdc42ep2	1633.84	-0.486	0.14277	-3.404	0.00066	0.00395	1960.16	1593.58	2165.67	1350.84	1302.08	1430.69	Down
Capn1	3738.91	0.60121	0.14797	4.06309	4.84E-05	0.0004	3514.93	2678.33	2719.51	4620.64	4891.51	4008.53	Up
Tm7sf2	1320.68	1.37764	0.11097	12.4143	2.18E-35	7.03E-33	751.749	780.981	669.373	1853.65	1983.22	1885.13	Up
Cdca5	66.1793	1.32683	0.34797	3.81309	0.00014	0.001	28.0575	43.1216	42.0148	80.8638	82.37	120.648	Up
Map4k2	6629.81	-0.481	0.12359	-3.8917	9.95E-05	0.00076	6919.57	7329.72	8925.29	5358.78	5580.04	5665.43	Down
Gm14966	252.719	-0.5801	0.22569	-2.5705	0.01015	0.0387	230.265	339.224	338.983	181.425	233.382	193.037	Down
Pygm	2507.85	-2.1325	0.3033	-7.0311	2.05E-12	7.03E-11	2719.65	6636.9	2896.16	971.403	989.496	833.477	Down
Rasgrp2	2464.34	-0.7728	0.13856	-5.5774	2.44E-08	4.09E-07	2838.65	2902.57	3585.59	1913.78	1917.74	1627.74	Down
Nrxn2	502.77	-1.3274	0.20533	-6.4648	1.01E-10	2.69E-09	673.381	683.238	800.192	330.712	319.976	209.123	Down
Rps6ka4	1655.83	0.54354	0.1937	2.80613	0.00501	0.02177	1671.84	1022.46	1348.29	2050.62	2141.62	1700.13	Up
Ccdc88b	686.112	0.82585	0.18728	4.40962	1.04E-05	0.0001	606.623	436.966	441.156	852.18	1010.62	769.132	Up
Esrra	2102.62	0.53107	0.13622	3.89858	9.68E-05	0.00074	1714.41	1909.81	1535.45	2390.67	2793.19	2272.21	Up
Kcnk4	67.2856	-1.0207	0.43496	-2.3466	0.01895	0.06402	68.6926	100.617	101.218	48.7256	21.1205	63.3403	Down
Trpt1	557.092	-0.4505	0.20324	-2.2164	0.02666	0.08381	545.671	720.611	663.644	565.01	483.66	363.955	Down
Fermt3	1793.19	0.51577	0.17317	2.97847	0.0029	0.01365	1566.38	1549.5	1312.01	2027.82	2586.21	1717.22	Up
Macrod1	1239.93	-0.783	0.17728	-4.4166	1.00E-05	9.83E-05	1339.02	1957.72	1408.45	957.925	816.308	960.158	Down
Mark2	4211.18	0.45581	0.10678	4.26879	1.97E-05	0.00018	3839.05	3281.08	3534.02	4806.21	5201.98	4604.74	Up
2700081C	1851.85	-0.3715	0.10664	-3.4842	0.00049	0.00307	2049.17	1980.72	2237.29	1616.24	1517.51	1710.19	Down
Atl3	7637.99	0.29399	0.09221	3.18831	0.00143	0.00753	7216.59	6383.92	6986.88	8051.14	8621.39	8568.03	Up
Lgals12	387.24	-0.8432	0.34455	-2.4472	0.0144	0.05137	432.473	688.988	370.495	361.814	149.956	319.717	Down
Wdr74	1258.43	-0.2459	0.11485	-2.1414	0.03224	0.09675	1299.35	1322.4	1474.34	1188.08	1200.7	1065.72	Down
Taf6l	414.059	0.5634	0.16667	3.38031	0.00072	0.00425	346.366	313.351	342.803	503.844	560.749	417.241	Up
Ttc9c	2290.81	0.29212	0.08732	3.34525	0.00082	0.00472	2111.09	1975.93	2092.15	2509.89	2467.93	2587.9	Up
Hnrnpul2	3468.44	0.38875	0.12093	3.21463	0.00131	0.00699	3304.99	2594.96	3111.96	3959.22	3803.8	4035.68	Up
Bscl2	4461.35	0.8982	0.11599	7.74359	9.66E-15	4.71E-13	3082.46	3436.32	2828.36	5686.39	6261.17	5473.4	Up
Ganab	10732.6	0.27684	0.08189	3.38067	0.00072	0.00424	10201.3	9382.31	9534.51	11392.5	11863.4	12021.6	Up
Eml3	1589.4	0.30405	0.10274	2.95939	0.00308	0.01441	1467.7	1359.77	1439.96	1753.09	1866	1649.86	Up
Mta2	5367.8	0.24024	0.11515	2.0863	0.03695	0.10778	5303.84	4306.41	5155.41	5785.91	6074.26	5580.98	Up
Scgb1a1	923667	-2.0527	0.17287	-11.874	1.61E-32	3.91E-30	1451237	1285220	1729191	428354	289199	358799	Down

Asrgl1	936.066	-0.3797	0.15367	-2.471	0.01347	0.04876	1145.52	1138.41	891.861	738.142	803.635	898.828	Down
Stxbp3-ps	127.054	0.52717	0.23568	2.23676	0.0253	0.08045	125.775	99.6589	86.8943	152.397	146.788	150.81	Up
Gm29231	25.3368	4.9392	0.82547	5.98348	2.18E-09	4.50E-08	0.9675	2.87478	0.95488	32.1382	51.7452	63.3403	Up
Gm50321	138.915	-1.3728	0.37966	-3.6158	0.0003	0.00198	190.598	303.768	106.947	68.4232	81.314	82.4429	Down
Incenp	649.123	1.02741	0.16353	6.28252	3.33E-10	8.02E-09	511.808	411.093	359.036	908.163	849.044	855.596	Up
Fth1	145261	0.5598	0.14411	3.88447	0.0001	0.00078	131241	115392	105648	172660	199106	147519	Up
Best1	438.992	0.67894	0.21377	3.17598	0.00149	0.00781	325.08	322.933	364.765	407.429	529.069	684.678	Up
Rab3il1	960.619	1.15404	0.1774	6.50548	7.74E-11	2.09E-09	666.609	660.24	460.254	1217.1	1293.63	1465.87	Up
Fads3	10964.7	0.35912	0.11052	3.24939	0.00116	0.0063	10595.1	8930.01	9295.78	12730.9	12878.2	11358	Up
Fads2	4973.91	1.44552	0.12531	11.5359	8.69E-31	1.82E-28	3058.27	2618.92	2337.55	7003.02	7572.76	7252.96	Up
Fads1	13891	1.08394	0.08672	12.499	7.56E-36	2.55E-33	8963.9	9158.08	8592.99	17829.4	19923	18878.4	Up
Myrf	2812.79	0.39569	0.12199	3.24355	0.00118	0.00641	2517.44	2205.91	2564.82	3147.47	3533.46	2907.62	Up
Dagla	983.434	0.4287	0.12328	3.47756	0.00051	0.00313	871.719	816.436	826.929	1250.28	1106.71	1028.53	Up
Syt7	2671.97	1.19549	0.1473	8.11592	4.82E-16	2.83E-14	1863.41	1446.97	1562.19	3299.87	4218.82	3640.56	Up
Lrrc10b	2395.77	-0.5714	0.18657	-3.0624	0.0022	0.01076	2671.27	3240.83	2680.36	2132.52	1426.69	2222.94	Down
Cyb561a3	1174.71	1.24631	0.21432	5.81522	6.06E-09	1.15E-07	914.289	619.993	555.742	1582.03	1951.53	1424.65	Up
Tkfc	393.078	0.86874	0.1451	5.98727	2.13E-09	4.40E-08	281.543	289.394	263.548	477.926	554.413	491.641	Up
Slc15a3	1161.94	1.92064	0.30474	6.30253	2.93E-10	7.15E-09	745.944	371.804	338.983	1768.64	2351.77	1394.49	Up
Tmem132	624.026	0.44519	0.13817	3.22215	0.00127	0.00684	528.256	555.79	501.314	767.17	758.226	633.403	Up
Tmem109	6733.26	-0.5619	0.12551	-4.4772	7.56E-06	7.68E-05	7607.46	8002.42	8474.59	5739.26	5935.92	4639.93	Down
Prpf19	4925.2	-0.2027	0.08262	-2.4535	0.01415	0.05071	5165.49	5486.03	5160.19	4504.53	4746.83	4488.11	Down
Ms4a15	45.1311	-2.5731	0.59192	-4.3471	1.38E-05	0.00013	43.5376	73.7859	114.586	15.5507	4.2241	19.1026	Down
AW11201	3377.64	0.95731	0.13572	7.05341	1.75E-12	6.04E-11	2374.25	2282.57	2232.52	5013.56	3712.99	4649.98	Up
Ms4a8a	1892.72	2.00683	0.23572	8.51358	1.69E-17	1.20E-15	1024.58	718.694	519.456	2983.67	3504.95	2604.99	Up
Ms4a14	109.195	1.90366	0.399	4.77102	1.83E-06	2.13E-05	40.6351	52.7042	44.8795	132.7	108.771	275.48	Up
Ms4a7	548.295	2.36018	0.26674	8.8484	8.88E-19	7.07E-17	224.46	180.153	131.774	761.986	748.722	1242.68	Up
Ms4a4b	767.961	-0.539	0.1343	-4.0136	5.98E-05	0.00048	960.729	822.186	946.289	675.939	632.559	570.062	Down
Ms4a6b	1125.17	-0.2579	0.11157	-2.3112	0.02082	0.06879	1191.96	1330.06	1154.45	1026.35	989.496	1058.69	Down
Ms4a4d	2704.43	-0.9682	0.1283	-7.5458	4.50E-14	2.01E-12	3655.22	3485.19	3597.04	1957.32	1988.5	1543.29	Down
Ms4a6d	1237.62	2.49666	0.20519	12.1677	4.62E-34	1.36E-31	443.116	408.218	266.412	1933.48	2433.08	1941.43	Up
Olfr1419	5.79406	3.01244	1.16841	2.57824	0.00993	0.03801	0	1.91652	1.90977	7.25701	11.6163	12.0648	Up
Patl1	2852.21	0.26262	0.09298	2.82454	0.00473	0.02074	2756.41	2427.27	2596.33	3099.78	3145.9	3087.59	Up
Osbp	5711.61	0.20263	0.07385	2.74381	0.00607	0.02541	5353.19	5188.97	5391.27	6149.8	6143.96	6042.46	Up
Mpeg1	17883.4	2.4367	0.25649	9.50002	2.10E-21	2.10E-19	8057.35	5034.69	3637.15	27385.9	35782.4	27403.2	Up
Lpxn	612.487	0.89847	0.18777	4.78485	1.71E-06	2.00E-05	545.671	361.264	376.224	778.574	828.98	784.213	Up
Psat1	1040.23	0.65116	0.17414	3.73933	0.00018	0.0013	617.266	914.179	896.635	1345.66	1224.99	1242.68	Up
Gcnt1	593.801	0.80275	0.21835	3.67639	0.00024	0.00161	392.806	571.122	334.209	776.5	821.588	666.581	Up
Ostf1	8811.42	0.48753	0.11991	4.06595	4.78E-05	0.00039	7374.3	7524.25	7111.01	10547.5	11436.8	8874.67	Up
Carnmt1	708.426	-0.5645	0.21336	-2.6456	0.00815	0.03233	946.216	804.937	784.914	466.522	487.884	760.083	Down
Trpm6	3729.42	0.76058	0.1277	5.95594	2.59E-09	5.27E-08	2852.19	2495.31	2958.23	4539.78	4253.67	5277.35	Up
Aldh1a1	85779.2	-0.8954	0.19929	-4.4929	7.02E-06	7.19E-05	129348	113657	91721.3	67740.1	43656.1	68552.3	Down
Aldh1a7	9652.79	-0.5044	0.18751	-2.6901	0.00714	0.029	12530.1	10264.9	11175	8769.58	5780.68	9396.48	Down
Gda	3517.45	1.83093	0.22786	8.03528	9.34E-16	5.20E-14	1834.38	1713.37	1082.84	4700.47	6970.82	4802.8	Up
Gm3443	50.6702	1.2891	0.34215	3.76764	0.00016	0.00118	24.1875	27.7895	36.2855	67.3865	74.9778	73.3943	Up
1110059E	3324.53	0.37339	0.12466	2.99533	0.00274	0.01302	2772.86	2850.82	3066.13	3963.36	4064.64	3229.35	Up
Abhd17b	4141.32	0.23658	0.11693	2.02331	0.04304	0.12141	3785.83	3829.2	3792.79	4344.88	4012.9	5082.3	Up
Cemip2	8270.21	-0.341	0.12901	-2.6429	0.00822	0.03253	7832.89	9519.34	10376.7	7250.79	6981.38	7660.15	Down
Trpm3	671.514	-1.1765	0.35158	-3.3463	0.00082	0.00471	966.534	896.93	930.056	321.382	215.429	698.754	Down
Klf9	12631.2	-0.8875	0.13488	-6.5804	4.69E-11	1.31E-09	15660	16613.3	16922.4	9555.41	7293.97	9742.33	Down
Mamdc2	5745.16	-0.3627	0.14758	-2.4576	0.01399	0.05023	7616.17	5591.44	6182.87	5096.5	5480.77	4503.19	Down
1700028P	393.308	-0.6466	0.18871	-3.4265	0.00061	0.00367	463.433	508.835	467.893	348.337	236.55	334.798	Down
Ptar1	3765	-0.3761	0.09098	-4.1341	3.56E-05	0.0003	4176.7	4067.81	4514.69	3176.5	3262.06	3392.22	Down
Fam189a2	10374.2	-0.5511	0.21117	-2.6096	0.00907	0.03521	9073.23	13598.6	14323.2	9083.7	9739.72	6426.52	Down
Tjp2	7364.15	0.39124	0.08622	4.53778	5.69E-06	5.93E-05	6563.53	6090.69	6460.74	8022.11	8771.35	8276.46	Up
Pip5k1b	2514.65	0.56015	0.12414	4.51224	6.41E-06	6.61E-05	2039.49	2069.84	1988.07	2679.91	3433.14	2877.46	Up
Tmem252	1723.98	-0.9186	0.14519	-6.3267	2.50E-10	6.19E-09	2302.65	2495.31	1967.06	1350.84	1070.81	1157.22	Down
Pgm5	1055.14	-1.3603	0.18851	-7.216	5.35E-13	2.03E-11	1345.79	1859.02	1351.16	559.827	702.257	512.754	Down
Gm10053	243.234	-0.8195	0.32564	-2.5165	0.01185	0.04393	201.24	482.004	248.27	171.058	186.916	169.913	Down
Cbwd1	826.075	0.32858	0.11702	2.80787	0.00499	0.02167	756.586	744.567	696.11	991.1	872.277	895.812	Up
Dock8	4296.96	0.59818	0.10369	5.76872	7.99E-09	1.48E-07	3292.41	3668.21	3295.3	4894.34	5586.37	5045.1	Up
Kank1	2100.57	-0.2921	0.14147	-2.0646	0.03896	0.11219	2381.99	2621.8	1933.64	2002.94	1874.45	1788.61	Down
Dmrt3	21.762	-4.6271	1.30231	-3.553	0.00038	0.00245	7.74001	100.617	17.1879	0	0	5.027	Down
Dmrt2	209.351	-0.9241	0.4216	-2.1918	0.0284	0.08815	157.703	380.429	284.555	158.618	69.6977	205.102	Down
Vldlr	2881.18	-0.5389	0.18116	-2.9746	0.00293	0.0138	3035.05	4199.09	3005.02	2614.6	2507	1926.35	Down
Gm50114	23.6805	-5.4855	1.08952	-5.0348	4.78E-07	6.32E-06	17.415	79.5355	42.0148	0	2.11205	1.0054	Down
Kcnv2	19.888	-4.803	1.04479	-4.5971	4.28E-06	4.60E-05	28.0575	71.8694	15.2781	0	2.11205	2.0108	Down
Glis3	1027.75	-0.3614	0.1793	-2.0158	0.04382	0.12312	1110.69	1188.24	1168.78	907.126	692.753	1098.9	Down
Slc1a1	111.001	0.76418	0.27274	2.80188	0.00508	0.02201	94.8151	68.9946	83.0748	114.039	135.171	169.913	Up
4430402I1	1045.11	-0.5292	0.25902	-2.0431	0.04105	0.11693	1007.17	1469.01	1227.98	977.623	531.181	1057.68	Down
Ak3	972.829	-0.4194	0.17383	-2.4129	0.01583	0.05542	1188.09	951.551	1200.29	697.71	811.027	988.309	Down
Jak2	4451.87	0.31451	0.09783	3.21477	0.00131	0.00699	4183.48	3833.03	3889.24	4605.09	4941.14	5259.25	Up
Cd274	1916.47	0.92287	0.23923	3.8577	0.00011	0.00085	1453.19	1463.26	1054.19	2234.12	3452.15	1841.89	Up
Pdcd1lg2	67.806	1.4945	0.3332	4.48526	7.28E-06	7.43E-05	31.9275	30.6643	43.9246	95.3779	122.499	82.4429	Up
A930007I1	397.361	0.56308	0.18833	2.9898	0.00279	0.01323	325.08	332.516	304.608	473.779	568.142	380.042	Up
Mlana	225.141	-1.0683	0.47714	-2.239	0.02516	0.08012	204.143	577.83	132.729	115.075	133.059	188.01	Down
Il33	25033.3	2.86339	0.249	11.4997	1.32E-30	2.74E-28	8488.86	5057.69	4599.67	40813.4	35188.9	56051.1	Up
Trpd52l3	6.31123	6.16042	1.57122	3.92079	8.83E-05	0.00068	0	0	0	10.3672	7.39218	20.108	Up
Prkg1	1976.24	-0.8398	0.14929	-5.6255	1.85E-08	3.17E-07	2207.84	2809.61	2589.64	1220.21	1591.43	1438.73	Down
Asah2	1945.49	0.47681	0.1411	3.37917	0.00073	0.00426	1685.39	1762.24	1433.28	2218.57	2011.73	2561.76	Up
Sgms1	8154.6	-0.3241	0.1177	-2.7535	0.0059	0.02483	7930.61	9170.54	10098.8	6937.7	7454.48	7335.4	Down
Rpl9-ps6	982.407	-0.3166	0.12376	-2.5579	0.01053	0.03989	1172.61	979.34	1117.21	870.841	922.966	831.467	Down
Papss2	2428.73	-0.7505	0.1236	-6.0724	1.26E-09	2.75E-08	2654.82	3296.41	3188.35	1829.8	1900.85	1702.14	Down
Lipo3	3058.27	0.37082	0.0847	4.37796	1.20E-05	0.00011	2745.77	2621.8	2634.52	3336.15	3579.93	3431.43	Up

Ankrd22	84.5759	2.64005	0.49943	5.28617	1.25E-07	1.82E-06	24.1875	37.3721	8.59395	102.635	206.981	127.686	Up
Acta2	10648.8	-1.7675	0.30307	-5.8319	5.48E-09	1.05E-07	8342.77	21483.2	19560.8	5045.7	5738.44	3721.99	Down
Fas	5365.48	-0.6286	0.12965	-4.8488	1.24E-06	1.49E-05	5998.51	6749.02	6801.63	4481.72	3589.43	4572.56	Down
Ch25h	1310.13	2.56822	0.24207	10.6093	2.70E-26	4.10E-24	478.913	337.307	317.976	2393.78	2779.46	1553.34	Up
Gm26902	97.8602	1.76254	0.33276	5.29673	1.18E-07	1.73E-06	62.8876	34.4973	36.2855	159.654	173.188	120.648	Up
Lipa	4907.72	1.11383	0.20639	5.39687	6.78E-08	1.04E-06	4027.71	2643.84	2634.52	6158.09	8201.09	5781.05	Up
Ifit2	1920.73	0.32518	0.13594	2.39203	0.01676	0.05808	1858.57	1658.75	1598.47	1988.42	1958.93	2461.22	Up
Ifit3	2865.75	0.90288	0.21797	4.14227	3.44E-05	0.00029	2738.03	1708.58	1545	4255.72	3436.31	3510.86	Up
Ifit1bl1	668.157	1.21902	0.14996	8.12887	4.33E-16	2.56E-14	465.368	358.389	380.998	987.99	869.109	947.088	Up
Ifit3b	617.058	0.42793	0.17575	2.43492	0.01489	0.05278	606.623	534.708	437.336	810.712	648.4	664.57	Up
Ifit1	1653.71	1.33105	0.19883	6.69454	2.16E-11	6.45E-10	891.069	930.469	1000.72	2511.96	1686.47	2901.59	Up
Kif20b	173.53	0.50064	0.21635	2.31402	0.02067	0.06841	151.898	148.53	130.819	171.058	199.589	239.285	Up
Htr7	39.9162	1.82172	0.46607	3.90868	9.28E-05	0.00071	21.285	16.2904	15.2781	50.7991	44.3531	91.4915	Up
Ankrd1	5112.52	-2.3236	0.90249	-2.5747	0.01003	0.03832	2850.26	19462.2	3255.2	1287.6	1454.15	2365.71	Down
Gm32027	4.95883	-5.7066	1.61771	-3.5276	0.00042	0.00266	7.74001	14.3739	7.63906	0	0	0	Down
Hectd2os	48.8264	-1.7059	0.67819	-2.5154	0.01189	0.04402	23.22	148.53	52.5186	23.8445	12.6723	32.1728	Down
Btaf1	3187.67	0.29951	0.10322	2.9017	0.00371	0.01686	2779.63	2768.41	3026.02	3337.19	3397.23	3817.51	Up
Cpeb3	1412.31	1.02339	0.16745	6.11156	9.87E-10	2.18E-08	1061.35	1013.84	719.027	1808.03	2009.62	1862	Up
Marchf5	5250.84	0.46822	0.08414	5.565	2.62E-08	4.36E-07	4512.43	4421.41	4284.56	6275.24	6226.33	5785.08	Up
Kif11	271.092	0.71506	0.24103	2.96664	0.00301	0.01412	229.298	226.149	160.42	370.108	254.502	386.074	Up
Exoc6	2125.99	0.2153	0.08754	2.45929	0.01392	0.05005	1954.35	1916.52	2031.99	2228.94	2354.94	2269.19	Up
Myof	10719.1	1.02318	0.12289	8.32577	8.38E-17	5.40E-15	7311.41	6825.68	7071.86	14301.5	16320.9	12483.1	Up
Cep55	104.836	1.27963	0.30169	4.2416	2.22E-05	0.0002	77.4001	55.579	50.6088	151.361	115.107	178.961	Up
Rbp4	1078	-1.4459	0.1263	-11.448	2.40E-30	4.84E-28	1562.51	1678.87	1489.62	569.157	641.007	526.83	Down
Fra10ac1	1095.58	-0.2464	0.10519	-2.3428	0.01914	0.06451	1164.87	1173.87	1227.98	941.338	1056.03	1009.42	Down
Slc35g1	1012.17	2.31343	0.18382	12.585	2.55E-36	9.03E-34	342.496	416.843	257.818	1755.16	1740.33	1560.38	Up
Hells	280.449	0.82898	0.18099	4.58017	4.65E-06	4.95E-05	219.623	214.65	171.879	379.438	322.088	375.015	Up
Cyp2c68	6.46006	5.19613	1.65018	3.14884	0.00164	0.00844	0	0.95826	0	16.5875	2.11205	19.1026	Up
Cyp2c69	2.06943	4.55018	2.1549	2.11155	0.03473	NA	0	0	0	8.29373	2.11205	2.0108	Up
Pdlim1	8471.79	0.65595	0.15387	4.26302	2.02E-05	0.00018	6835.4	5685.35	7214.14	10098.6	12151.7	8845.52	Up
Sorbs1	819.849	-0.4932	0.19959	-2.4712	0.01346	0.04873	865.914	1143.2	867.034	611.662	578.702	852.58	Down
Aldh18a1	1130.86	0.71699	0.1744	4.11128	3.93E-05	0.00033	694.666	849.017	1022.68	1563.37	1408.74	1246.7	Up
Entpd1	7244	0.25673	0.10506	2.44376	0.01454	0.05176	6543.21	6218.14	7042.26	7321.29	7788.19	8550.93	Up
Cc2d2b	145.898	2.06312	0.34913	5.90929	3.44E-09	6.89E-08	53.2126	75.7024	40.1051	155.507	312.584	238.28	Up
Ccnj	1488.84	1.05407	0.10149	10.3864	2.86E-25	3.93E-23	991.689	905.554	1006.45	1970.8	2091.99	1966.56	Up
Zfp518a	1364.44	0.36306	0.10896	3.33201	0.00086	0.00491	1144.55	1235.2	1201.24	1406.82	1586.15	1612.66	Up
Blnk	915.299	0.7189	0.17436	4.12308	3.74E-05	0.00032	811.734	725.402	538.554	1116.54	1073.98	1225.58	Up
Tm9sf3	21417.4	0.28054	0.07691	3.64769	0.00026	0.00177	19094.6	19393.2	19536.9	22359.9	24042.5	24077.3	Up
Pik3ap1	2985.95	1.39328	0.22058	6.31654	2.67E-10	6.58E-09	2277.5	1415.35	1247.08	4158.27	4848.21	3969.32	Up
Arhgap19	411.385	0.59996	0.18676	3.2125	0.00132	0.00704	399.578	302.81	278.826	452.008	535.405	499.684	Up
Pgam1	3093.21	0.32728	0.10515	3.11258	0.00185	0.00936	2831.88	2930.35	2469.33	3405.61	3542.97	3379.15	Up
Ubtd1	1041.67	0.32095	0.12028	2.66848	0.00762	0.03055	1001.36	954.426	823.109	1145.57	1154.24	1171.29	Up
Pi4k2a	4056.01	0.40028	0.09093	4.40219	1.07E-05	0.0001	3567.18	3386.49	3536.89	4739.86	4796.47	4309.15	Up
Avpi1	2839.12	1.0886	0.12376	8.79641	1.41E-18	1.09E-16	2018.21	1640.54	1789.45	4211.14	3770.01	3605.37	Up
Marveld1	13419.3	0.33304	0.12437	2.67788	0.00741	0.02988	13516	10244.7	11871.1	15086.3	15389.5	14408.4	Up
Zfyve27	2881.13	0.24203	0.08476	2.85551	0.0043	0.01908	2764.15	2583.47	2572.45	3104.96	3169.13	3092.61	Up
Sfrp5	62.2227	-4.539	0.6608	-6.869	6.46E-12	2.07E-10	86.1076	205.067	66.8418	6.2203	1.05603	8.04321	Down
Loxl4	134.886	1.10412	0.39379	2.80386	0.00505	0.0219	139.32	52.7042	64.932	188.682	224.933	138.745	Up
Pyroxd2	324.403	-0.5009	0.18527	-2.7035	0.00686	0.02808	379.261	383.303	378.134	251.922	227.045	326.755	Down
Hps1	989.749	0.26878	0.12899	2.08378	0.03718	0.10829	1004.27	822.186	867.034	1011.83	1136.28	1096.89	Up
Got1	2285.78	-1.1076	0.26762	-4.1388	3.49E-05	0.0003	2250.41	4735.71	2381.48	1567.51	1271.45	1508.1	Down
Entpd7	341.803	0.40689	0.17216	2.3635	0.0181	0.06171	336.691	258.73	286.465	380.475	361.161	427.295	Up
Cpn1	691.499	0.38534	0.17411	2.21315	0.02689	0.08439	590.176	608.494	600.621	744.362	644.175	961.163	Up
Erlin1	2570.83	0.28333	0.13345	2.12307	0.03375	0.10027	2465.19	2155.12	2337.55	2852.01	2432.03	3183.1	Up
Bloc1s2	1980.26	0.83964	0.12285	6.83466	8.22E-12	2.61E-10	1423.19	1314.73	1521.13	2593.86	2760.45	2268.18	Up
Scd3	48.5738	-1.0922	0.46802	-2.3337	0.01961	0.06578	34.8301	67.0781	96.4432	25.9179	38.0169	29.1566	Down
Scd2	57598.4	0.84283	0.07126	11.827	2.83E-32	6.57E-30	40258.7	40904.2	42546.7	73218.1	73575.4	75087.4	Up
Scd4	119.646	-1.0005	0.32257	-3.1017	0.00192	0.00965	106.425	222.316	149.917	81.9006	72.8658	84.4537	Down
Scd1	422704	3.30031	0.24903	13.2524	4.37E-40	2.14E-37	63194.3	110719	59812	650092	679542	972864	Up
Gm50337	102.381	1.4133	0.41401	3.41368	0.00064	0.00383	24.1875	69.9529	73.526	135.81	128.835	181.978	Up
Wnt8b	6.93515	3.29876	1.21427	2.71667	0.00659	0.02714	1.935	0	1.90977	10.3672	5.28013	22.1188	Up
Ndufb8	1645.62	-0.3716	0.15701	-2.3669	0.01794	0.0613	1605.08	2276.82	1687.28	1447.26	1445.7	1411.58	Down
Slf2	119.357	0.96178	0.33905	2.83672	0.00456	0.02007	94.8151	60.3703	87.8492	130.626	118.275	224.204	Up
Twnk	609.518	-0.3146	0.13521	-2.327	0.01997	0.06675	598.883	711.986	716.162	545.313	564.974	519.792	Down
Pdzd7	228.327	-0.4271	0.18089	-2.361	0.01823	0.06209	265.095	265.438	254.954	205.27	213.317	165.891	Down
Gm35867	100.113	-0.7083	0.23321	-3.0371	0.00239	0.01158	132.548	120.741	119.36	72.5701	76.0338	79.4267	Down
Fbxw4	1004.71	0.45187	0.14243	3.17256	0.00151	0.00788	816.571	779.064	950.108	1122.76	1302.08	1057.68	Up
Oga	2529.52	0.3528	0.13686	2.57789	0.00994	0.03803	2437.14	1923.23	2305.09	2619.78	2730.88	3160.98	Up
Kcnip2	136.904	-1.8155	0.40624	-4.469	7.86E-06	7.92E-05	211.883	308.559	119.36	55.9827	41.185	84.4537	Down
Nfkb2	2324.29	0.56861	0.10185	5.58282	2.37E-08	3.97E-07	1927.26	1705.7	1983.29	2725.53	2828.04	2775.91	Up
Psd	847.464	0.58539	0.19179	3.05227	0.00227	0.01109	620.168	624.785	788.733	936.154	851.156	1263.79	Up
Mfsd13a	1150.35	1.29405	0.12967	9.97972	1.87E-23	2.18E-21	696.601	615.202	687.516	1472.14	1807.92	1622.72	Up
Arl3	3022.2	-0.4509	0.09537	-4.7284	2.26E-06	2.58E-05	3364	3573.35	3534.98	2619.78	2370.78	2670.34	Down
Wbp1l	6251.8	0.40923	0.09011	4.54147	5.59E-06	5.84E-05	5131.63	5716.01	5265.22	7140.9	7392.18	6864.88	Up
Cnnm2	1201.98	0.6436	0.13219	4.86861	1.12E-06	1.37E-05	963.631	1002.34	848.891	1504.27	1304.19	1588.53	Up
Gm50431	521.511	0.44998	0.16334	2.75493	0.00587	0.02475	467.303	477.213	378.134	565.01	560.749	680.656	Up
Atp5md	1082.16	-0.3293	0.12808	-2.5714	0.01013	0.03863	1137.78	1313.77	1164	1028.42	860.661	988.309	Down
Pdcd11	53.7863	0.79137	0.36307	2.17967	0.02928	0.09026	49.3426	25.873	42.9697	73.6068	67.5856	63.3403	Up
Calhm2	687.97	-0.5846	0.14044	-4.1626	3.15E-05	0.00027	833.019	767.565	875.628	567.084	601.934	482.592	Down
Sh3pxd2a	5105.16	-0.3516	0.14223	-2.4721	0.01343	0.04866	4694.32	6151.06	6327.05	4316.88	4834.48	4307.14	Down
Col17a1	287.002	-1.1989	0.22929	-5.2286	1.71E-07	2.44E-06	401.513	415.884	381.953	130.626	229.158	162.875	Down
Sfr1	4664.47	-0.2184	0.08235	-2.6525	0.00799	0.03178	5092.93	4900.53	5057.06	4460.99	4357.16	4118.12	Down
Cfap43	6287.81	-0.5351	0.20738	-2.5801	0.00988	0.03783	6853.78	8201.74	7266.66	5726.82	3478.55	6199.3	Down

Cfap58	1029.95	-0.7074	0.23439	-3.0182	0.00254	0.01222	1302.26	1398.1	1132.49	823.152	514.284	1009.42	Down
Xpnpep1	3977.39	0.21458	0.08888	2.41418	0.01577	0.05526	3572.02	3936.53	3537.84	4305.48	4272.68	4239.78	Up
Add3	9339.28	-0.4617	0.09254	-4.989	6.07E-07	7.89E-06	10012.7	10816.8	11633.3	7848.98	7893.79	7830.06	Down
Dusp5	658.925	0.64191	0.2651	2.42141	0.01546	0.05435	582.436	542.374	419.194	865.658	1040.18	503.706	Up
Nutf2-ps1	151.244	0.51508	0.23284	2.2122	0.02695	0.08452	118.035	145.655	109.812	177.278	204.869	151.816	Up
Smc3	3381.01	-0.2785	0.09562	-2.9121	0.00359	0.0164	3687.15	3501.48	3930.3	2900.73	3119.5	3146.9	Down
Rbm20	397.39	-2.0928	0.354	-5.9118	3.38E-09	6.80E-08	517.613	1036.84	377.179	154.471	118.275	179.967	Down
Pdcd4	2386.24	-0.2928	0.12862	-2.2766	0.02281	0.07404	2846.39	2586.34	2450.23	2268.33	1861.77	2304.38	Down
Adra2a	140.449	-1.7764	0.35067	-5.0656	4.07E-07	5.46E-06	153.833	293.227	205.3	79.8271	40.129	70.3781	Down
Gm50186	134.742	-1.9086	0.26578	-7.181	6.92E-13	2.54E-11	222.525	171.528	244.45	62.203	45.4091	62.3349	Down
Gpam	10846.8	0.91602	0.11714	7.81976	5.29E-15	2.69E-13	8305.03	7167.77	7070.91	13077.1	13879.3	15580.7	Up
Acsl5	910.602	1.07142	0.20042	5.34585	9.00E-08	1.34E-06	740.139	502.128	519.456	1031.53	1237.66	1432.7	Up
Zdhhc6	2578.49	0.25715	0.09404	2.73464	0.00624	0.02601	2284.27	2380.31	2383.39	2633.26	2822.76	2966.94	Up
Tcf7l2	1493.03	0.80226	0.17461	4.59445	4.34E-06	4.66E-05	1234.53	1129.79	900.455	1837.06	2236.66	1619.7	Up
Nrap	429.401	-2.4369	0.68011	-3.5831	0.00034	0.00221	380.228	1441.22	353.307	107.818	152.068	141.762	Down
Casp7	2259.19	0.73603	0.11271	6.53032	6.56E-11	1.79E-09	1867.28	1618.5	1599.43	2751.44	3014.95	2703.52	Up
Plekhs1	87.4424	1.60887	0.4136	3.88988	0.0001	0.00076	34.8301	59.412	35.3307	149.287	72.8658	172.929	Up
Nhlrc2	8554.16	-0.275	0.08797	-3.1264	0.00177	0.00899	9010.34	9433.1	9657.69	7359.65	8200.04	7664.17	Down
Gm32492	28.02	-3.5243	0.64924	-5.4283	5.69E-08	8.94E-07	50.3101	73.7859	30.5563	3.11015	6.33615	4.0216	Down
Gm50100	43.2411	-0.7871	0.38448	-2.0472	0.04064	0.11592	53.2126	66.1198	44.8795	26.9546	40.129	28.1512	Down
Tdrd1	5.76776	2.63433	1.08007	2.43903	0.01473	0.05228	1.935	1.91652	0.95488	6.2203	9.50423	14.0756	Up
Trub1	705.138	-0.3594	0.12052	-2.982	0.00286	0.01351	787.546	836.56	753.403	577.451	650.512	625.359	Down
Atrnl1	4167.37	0.42838	0.12633	3.39088	0.0007	0.00412	4062.54	3176.63	3420.39	4451.66	4988.66	4904.35	Up
Gm16277	126.674	-1.1045	0.25274	-4.3702	1.24E-05	0.00012	201.24	171.528	146.097	90.1943	66.5296	84.4537	Down
Gfra1	1476.34	-1.23	0.24684	-4.983	6.26E-07	8.13E-06	2162.37	2082.3	1966.1	810.712	580.814	1255.75	Down
Eno4	2552.49	-0.8738	0.21508	-4.0627	4.85E-05	0.0004	2889.93	3799.5	3218.91	1986.35	1235.55	2184.74	Down
Shtn1	4055.84	1.29354	0.12251	10.5586	4.63E-26	6.85E-24	2471.97	2239.45	2339.46	5403.36	6584.32	5296.45	Up
Pdzd8	2962.76	0.32586	0.1306	2.49512	0.01259	0.04615	2949.91	2246.16	2692.77	3112.22	3337.04	3438.47	Up
Eif3a	9376.23	0.25275	0.0935	2.70313	0.00687	0.0281	8323.42	8198.86	9148.73	9621.76	10444.1	10520.5	Up
Sfxn4	553.221	-0.577	0.1493	-3.8648	0.00011	0.00083	642.421	614.244	730.485	498.66	420.298	413.22	Down
Zfp950	3706.3	-0.3346	0.09744	-3.4336	0.0006	0.00359	3818.73	4123.39	4460.26	3279.13	3252.56	3303.75	Down
Gm6020	13.2394	2.42147	0.76854	3.15072	0.00163	0.00839	5.80501	5.74955	0.95488	18.6609	21.1205	27.1458	Up
Csf2ra	3267.94	1.2983	0.15416	8.42195	3.70E-17	2.52E-15	2107.22	1869.56	1691.1	4725.35	5346.66	3867.78	Up
Gm18999	5.63613	3.42227	1.2511	2.73541	0.00623	0.02597	1.935	0.95826	0	13.4773	7.39218	10.054	Up
Nudt10	111.788	-0.8279	0.26981	-3.0686	0.00215	0.01059	143.19	139.906	146.097	70.4967	65.4736	105.567	Down
Bmp15	14.1998	-1.3707	0.62401	-2.1966	0.02805	0.08731	17.415	25.873	18.1428	6.2203	9.50423	8.04321	Down
Clcn5	1055.49	1.56258	0.16528	9.45408	3.26E-21	3.19E-19	623.071	518.418	460.254	1435.85	1817.42	1477.94	Up
2010204K	268.857	-1.2283	0.2073	-5.9252	3.12E-09	6.30E-08	357.976	322.933	449.75	176.242	138.339	167.902	Down
Cacna1f	20.9634	1.57139	0.56143	2.79891	0.00513	0.02217	6.77251	14.3739	10.5037	40.4319	29.5687	24.1296	Up
Plp2	6952.06	0.45493	0.11902	3.82214	0.00013	0.00097	6252.96	5580.9	5760.81	8502.11	8612.94	7002.62	Up
Praf2	384.669	-0.3714	0.16471	-2.2552	0.02412	0.07741	400.546	426.425	474.577	339.006	374.889	292.572	Down
Tfe3	2785.69	0.25168	0.09908	2.54014	0.01108	0.04159	2681.91	2372.65	2575.32	2933.91	3194.48	2955.88	Up
Hdac6	1030.33	0.37457	0.1088	3.44272	0.00058	0.00349	956.859	836.56	898.545	1140.39	1195.42	1154.2	Up
Gata1	25.8302	-1.2656	0.56327	-2.247	0.02464	0.07873	29.9925	51.746	27.6916	8.29373	22.1765	15.081	Down
Was	583.432	0.85776	0.22151	3.87231	0.00011	0.00081	516.646	406.302	321.796	699.783	916.63	639.435	Up
Rbm3	8055.39	0.27125	0.10663	2.54383	0.01096	0.0412	6810.24	7839.51	7251.38	9296.23	8052.19	9082.79	Up
Slc38a5	1162.01	-0.7681	0.14436	-5.3208	1.03E-07	1.53E-06	1271.3	1531.3	1589.88	913.347	908.182	758.072	Down
Xk	289.831	-0.3886	0.18166	-2.1393	0.03241	0.09713	316.373	358.389	311.292	224.967	233.382	294.582	Down
Cybb	7831.94	2.16964	0.28464	7.6223	2.49E-14	1.16E-12	3937.73	2803.86	1803.77	9393.68	17209	11843.6	Up
Dynlt3	10276.9	0.25699	0.07826	3.28376	0.00102	0.00569	9499.9	9085.25	9506.81	10794.3	11576.2	11199.2	Up
Sytl5	23.3786	-1.7101	0.54468	-3.1395	0.00169	0.00866	33.8626	35.4556	38.1953	9.33044	6.33615	17.0918	Down
Srpx	468.599	-1.2524	0.21462	-5.8352	5.37E-09	1.03E-07	497.296	828.894	654.095	304.794	266.118	260.399	Down
Tspan7	27277.7	-0.8231	0.1227	-6.7086	1.96E-11	5.89E-10	33774.5	31960.8	38829.4	20150.6	21325.4	17625.7	Down
Mid1ip1	9689.12	0.816	0.1027	7.94584	1.93E-15	1.02E-13	6917.64	7220.48	6920.99	12606.5	13353.4	11115.7	Up
Atp6ap2	7991.78	0.43721	0.09298	4.70199	2.58E-06	2.91E-05	7063.73	6719.31	6586.78	9236.1	9777.74	8567.02	Up
Gm14634	65.4825	-1.249	0.32148	-3.8852	0.0001	0.00077	97.7176	101.575	77.3455	37.3218	31.6808	47.2538	Down
Usp9x	2795.62	0.36759	0.14264	2.57703	0.00997	0.0381	2721.58	2231.78	2370.97	2737.97	3112.11	3599.34	Up
Cask	2537.11	0.34825	0.10214	3.40972	0.00065	0.00388	2417.79	2054.51	2224.88	2778.4	2877.67	2869.41	Up
Maob	3138.11	-0.5543	0.11839	-4.6815	2.85E-06	3.19E-05	4026.74	3624.13	3550.25	2624.96	2258.84	2743.74	Down
Ndp	15.7896	-1.8364	0.627	-2.9289	0.0034	0.01569	27.09	26.8312	20.0525	8.29373	8.4482	4.0216	Down
Efhc2	119.559	-1.4289	0.31261	-4.5709	4.86E-06	5.15E-05	156.735	218.483	148.007	62.203	46.4651	85.4591	Down
Slc9a7	70.903	0.89048	0.37431	2.37898	0.01736	0.05965	72.5626	33.5391	42.9697	97.4513	103.49	75.4051	Up
Rp2	3188.37	0.61743	0.09483	6.51101	7.46E-11	2.01E-09	2559.04	2652.46	2337.55	3736.32	3936.86	3907.99	Up
Rgn	53.2207	0.93602	0.43646	2.14459	0.03199	0.09626	53.2126	34.4973	21.9623	60.1295	57.0254	92.4969	Up
Ndufb11	4257.06	-0.2662	0.10948	-2.4313	0.01505	0.05319	4359.56	4911.08	4675.11	4190.41	3888.29	3517.9	Down
Usp11	1292.8	-0.2846	0.09696	-2.935	0.00334	0.01542	1374.82	1398.1	1486.75	1136.24	1178.52	1182.35	Down
Syn1	92.6668	1.15935	0.26912	4.30788	1.65E-05	0.00015	65.7901	51.746	54.4283	149.287	115.107	119.643	Up
Elk1	680.541	0.27501	0.1154	2.38301	0.01717	0.05912	641.453	575.913	630.223	745.399	760.338	729.921	Up
A230072C	304.928	-0.4172	0.18355	-2.2732	0.02301	0.07461	328.95	321.017	396.276	229.114	256.614	297.599	Down
Slc6a14	8450.43	1.00368	0.13529	7.4187	1.18E-13	4.95E-12	6677.7	4941.74	5252.81	10920.8	11315.3	11594.3	Up
Klhl13	924.548	-0.7269	0.20302	-3.5805	0.00034	0.00223	867.849	1464.22	1125.81	701.857	757.17	630.386	Down
Gm2223	128.098	0.47577	0.22581	2.10692	0.03512	0.10362	99.6527	106.367	115.541	144.104	132.003	170.918	Up
Dock11	963.588	0.45993	0.12512	3.67589	0.00024	0.00161	863.011	841.351	729.531	1035.68	1164.8	1147.16	Up
Il13ra1	5890.55	0.53994	0.20293	2.66077	0.0078	0.03113	3638.77	6421.29	4342.81	6827.81	6476.6	7636.02	Up
Zcchc12	52.1048	-1.0513	0.41803	-2.5149	0.01191	0.04407	46.4401	73.7859	90.7139	31.1015	25.3446	45.243	Down
Gm14569	212.04	1.13991	0.26492	4.30283	1.69E-05	0.00016	179.955	118.824	98.3529	294.427	326.312	254.366	Up
Pgrmc1	17843.9	-0.7069	0.11106	-6.3652	1.95E-10	4.95E-09	24067.6	20125.3	22198.2	14246.5	12434.7	13991.2	Down
Slc25a5	16398.4	0.26154	0.09721	2.6905	0.00713	0.02898	15577.7	15633	13537.4	18075.1	18314.6	17252.7	Up
Rpl39	2265.23	-0.2584	0.11431	-2.2608	0.02377	0.07652	2497.12	2227.95	2677.49	2123.19	2134.23	1931.38	Down
Akap14	799.346	-1.2496	0.23838	-5.2421	1.59E-07	2.28E-06	1068.12	1198.78	1109.57	466.522	313.64	639.435	Down
Tmem255	62.9023	-0.8081	0.3571	-2.2629	0.02364	0.07618	101.588	56.5373	82.1199	53.9092	38.0169	45.243	Down
Lamp2	30451.2	0.60385	0.09699	6.22619	4.78E-10	1.11E-08	26207.7	24012	22289.8	35547.9	38035.9	36613.7	Up
Gm7598	41.2246	-0.9049	0.45088	-2.007	0.04475	0.12512	56.1151	54.6207	50.6088	22.8077	17.9524	45.243	Down

Cul4b	1857.95	0.6328	0.10075	6.28117	3.36E-10	8.08E-09	1537.36	1389.48	1443.78	2157.41	2387.67	2231.99	Up
C1galt1c1	3415.4	0.7364	0.09892	7.44414	9.76E-14	4.14E-12	2516.47	2479.02	2690.86	4442.33	4421.58	3942.18	Up
Ap3s1-ps:	175.42	0.83278	0.4161	2.00139	0.04535	0.12632	158.67	46.9547	172.834	243.628	212.261	218.172	Up
Gria3	1551.07	-0.6792	0.11717	-5.797	6.75E-09	1.27E-07	1737.63	2044.92	1946.05	1144.53	1284.13	1149.17	Down
Sh2d1a	52.8478	-0.841	0.32243	-2.6084	0.0091	0.03531	59.0176	74.7442	69.7065	41.4686	36.9609	35.189	Down
Dcaf12l1	112.353	0.54993	0.26198	2.09912	0.03581	0.10514	89.0101	100.617	84.0297	147.214	102.434	150.81	Up
Prr32	13.7566	-7.1786	1.49287	-4.8086	1.52E-06	1.80E-05	17.415	54.6207	10.5037	0	0	0	Down
Ocrl	2390.93	0.32914	0.09573	3.43833	0.00059	0.00354	2266.86	2045.88	2045.36	2600.08	2757.28	2630.13	Up
Xpnpep2	883.652	-0.8719	0.15409	-5.6581	1.53E-08	2.67E-07	1053.61	1338.69	1036.05	589.891	687.473	596.203	Down
Sash3	1219.16	0.70449	0.16271	4.32961	1.49E-05	0.00014	1105.85	835.602	840.297	1530.19	1674.86	1328.13	Up
Zdhhc9	2916.42	1.28052	0.11996	10.6744	1.34E-26	2.10E-24	1908.88	1559.09	1634.76	3978.92	4439.53	3977.37	Up
Aifm1	2121	-0.2764	0.13263	-2.0836	0.03719	0.10831	2356.83	2629.46	1984.25	1982.2	1929.36	1843.91	Down
Rab33a	34.6592	-1.778	0.72425	-2.455	0.01409	0.05053	25.155	94.8676	41.06	7.25701	8.4482	31.1674	Down
Arhgap36	14.4624	-2.1918	0.79215	-2.7669	0.00566	0.02404	29.025	31.6225	10.5037	6.2203	7.39218	2.0108	Down
Igsf1	174.699	-1.7702	0.89493	-1.978	0.04793	0.13209	208.013	550.04	52.5186	53.9092	78.1459	105.567	Down
Rap2c	3492.97	0.47587	0.09651	4.93073	8.19E-07	1.04E-05	2894.76	2897.77	2973.51	3794.38	4388.84	4008.53	Up
Mbnl3	178.257	0.76106	0.22988	3.3107	0.00093	0.00525	166.41	107.325	123.18	220.82	238.662	213.145	Up
Gpc4	1620.93	0.48808	0.15876	3.07443	0.00211	0.01041	1374.82	1525.55	1147.77	2011.23	1605.16	2061.07	Up
Gm14586	93.103	-0.5007	0.24649	-2.0313	0.04222	0.11956	95.7826	113.075	118.405	77.7537	79.2019	74.3997	Down
Hprt	7799.84	0.4595	0.09382	4.89797	9.68E-07	1.20E-05	6633.19	6621.57	6449.28	9187.38	9614.05	8293.55	Up
C430049B	348.209	-1.019	0.30044	-3.3919	0.00069	0.0041	281.543	596.037	521.366	289.244	186.916	214.15	Down
Fam122b	645.149	0.60999	0.17536	3.47842	0.0005	0.00313	565.988	503.086	463.118	674.902	940.919	722.883	Up
Gm14597	442.761	-0.5997	0.13828	-4.3371	1.44E-05	0.00014	506.003	537.583	556.697	347.3	381.225	327.761	Down
Rtl8a	1374.99	-0.8932	0.20168	-4.4286	9.48E-06	9.35E-05	1376.75	1858.06	2127.48	952.742	1142.62	792.256	Down
Rtl8b	1514.19	-0.7203	0.15875	-4.5371	5.70E-06	5.94E-05	1546.07	1983.6	2123.66	1118.62	1274.62	1038.58	Down
Ct55	20.8674	1.67829	0.65424	2.56523	0.01031	0.03921	11.61	14.3739	3.81953	42.5053	33.7928	19.1026	Up
Xlr	382.806	0.90266	0.21254	4.24702	2.17E-05	0.00019	322.178	224.233	253.999	589.891	496.332	410.204	Up
Zfp449	250.324	-0.4714	0.19015	-2.4792	0.01317	0.04784	283.478	300.893	288.375	181.425	196.421	251.35	Down
Mmgt1	2945.68	0.25185	0.0998	2.52367	0.01161	0.04324	2860.9	2640.96	2565.77	3028.25	3395.12	3183.1	Up
Slc9a6	4217.31	0.54324	0.11808	4.60056	4.21E-06	4.54E-05	3705.53	3113.38	3478.64	4753.34	5513.51	4739.46	Up
Fhl1	46098.6	-0.7813	0.10744	-7.2726	3.53E-13	1.37E-11	54601.9	57637.3	62616.4	34979.8	36064.3	30691.9	Down
Htatsf1	2620.49	-0.3362	0.09867	-3.4079	0.00065	0.0039	2708.04	2959.1	3106.23	2246.56	2385.56	2317.45	Down
Rbmx	3003.05	-0.2486	0.09567	-2.5987	0.00936	0.03618	3274.99	3173.75	3334.45	2711.01	2945.25	2578.85	Down
Gm364	8.5676	3.27463	1.07046	3.05908	0.00222	0.01087	3.87001	0	0.95488	12.4406	20.0645	14.0756	Up
F9	21.3886	2.42355	0.62104	3.90242	9.52E-05	0.00073	5.80501	11.4991	2.86465	37.3218	30.6247	40.216	Up
Gm5637	203.413	1.18582	0.21625	5.4837	4.17E-08	6.69E-07	130.613	112.116	129.864	298.574	322.088	227.221	Up
Mamld1	486.291	-0.3088	0.13865	-2.227	0.02595	0.08204	553.411	530.875	529.96	467.559	449.867	386.074	Down
Mtm1	1428.98	0.34525	0.12517	2.75823	0.00581	0.02456	1413.52	1255.32	1107.66	1624.53	1568.2	1604.62	Up
Hmgb3	944.75	0.84211	0.10961	7.68304	1.55E-14	7.28E-13	667.576	707.195	655.05	1142.46	1233.44	1262.78	Up
Prrg3	8013.15	1.21175	0.14722	8.23065	1.86E-16	1.14E-14	5866.93	4145.43	4486.04	11671.3	10789.4	11119.7	Up
Cnga2	179.644	-0.8532	0.2754	-3.098	0.00195	0.00975	298.958	169.612	225.352	121.296	109.827	152.821	Down
Gabre	832.736	-1.7439	0.22339	-7.8063	5.89E-15	2.96E-13	1280.97	1141.29	1425.64	436.457	256.614	455.447	Down
Gabra3	477.531	-1.1172	0.22166	-5.0401	4.65E-07	6.17E-06	833.019	643.95	484.126	330.712	299.911	273.469	Down
Gabrq	101.668	-2.0347	0.3376	-6.027	1.67E-09	3.53E-08	227.363	119.782	143.232	42.5053	35.9049	41.2214	Down
Nsdhl	2999.98	1.24537	0.1157	10.7639	5.10E-27	8.46E-25	1850.83	1918.43	1570.78	3991.36	4298.02	4370.48	Up
Zfp185	847.526	0.41324	0.12589	3.28243	0.00103	0.00571	772.066	665.99	742.899	887.429	1005.34	1011.43	Up
Zfp275	1582.92	-0.4033	0.12079	-3.3386	0.00084	0.00482	1617.66	1786.19	2004.3	1302.12	1389.73	1397.51	Down
Bgn	44525.7	-0.3136	0.12109	-2.5897	0.00961	0.03695	52978.4	51394.3	43664.9	42452.5	40970.6	35693.7	Down
Pnck	252.187	-1.1247	0.298	-3.7742	0.00016	0.00115	297.023	386.178	354.262	218.747	95.0423	161.87	Down
Abcd1	3326.96	0.41983	0.12929	3.24713	0.00117	0.00634	3043.76	2836.45	2658.39	3750.84	4322.31	3350	Up
Plxnb3	320.446	1.80309	0.26495	6.80534	1.01E-11	3.15E-10	135.45	183.986	108.857	567.084	363.273	564.03	Up
Srpk3	283.21	-0.4881	0.2223	-2.1955	0.02813	0.08752	312.503	383.303	296.014	261.252	268.23	177.956	Down
Idh3g	5616.63	-0.3597	0.13515	-2.6617	0.00777	0.03108	5789.53	7451.42	5698.74	5052.95	5075.26	4631.88	Down
Ssr4	3751.76	0.36181	0.0999	3.62163	0.00029	0.00194	3304.99	3379.78	3166.39	4550.15	4209.32	3899.95	Up
Pdzd4	285.316	-0.6368	0.19851	-3.2081	0.00134	0.00712	374.423	324.85	342.803	216.674	182.692	270.453	Down
Renbp	3920.58	1.10639	0.14861	7.44495	9.70E-14	4.13E-12	2912.18	2216.45	2331.82	5398.18	5946.48	4718.35	Up
Dnase1l1	2554.76	0.41949	0.10265	4.08657	4.38E-05	0.00036	2346.19	2039.17	2172.36	2980.56	3023.4	2766.86	Up
Atp6ap1	14998.8	0.33702	0.09977	3.37813	0.00073	0.00427	13176.4	13569.9	13017.9	16762.7	18230.2	15235.8	Up
Plxna3	473.326	-0.4447	0.1567	-2.8376	0.00455	0.02002	535.996	512.668	588.208	416.76	442.475	343.847	Down
Ikbkg	3528.54	-0.2997	0.1303	-2.3003	0.02143	0.07037	4478.56	3605.93	3597.04	3057.28	2997	3435.45	Down
Mpp1	4340.47	0.43273	0.12258	3.53011	0.00042	0.00264	4031.58	3663.42	3387.92	4866.34	5543.08	4550.44	Up
Fundc2	2712.29	-0.3538	0.10737	-3.295	0.00098	0.00551	3050.53	3310.78	2768.21	2374.08	2464.76	2305.38	Down
Cmc4	884.364	-0.4307	0.12777	-3.3705	0.00075	0.00437	1066.19	994.672	985.439	668.682	768.786	822.418	Down
Rab39b	56.3479	-1.0587	0.34618	-3.0582	0.00223	0.0109	59.9851	88.1598	80.2102	34.2116	44.3531	31.1674	Down
Pls3	18690.2	0.25021	0.08246	3.03429	0.00241	0.01167	17183.8	16216.6	17820	19714.2	20936.8	20269.9	Up
Prrg1	1329.18	-0.5331	0.14372	-3.7095	0.00021	0.00144	1367.08	1614.67	1734.07	1066.78	1210.21	982.277	Down
Cfap47	493.234	-0.4336	0.15085	-2.8742	0.00405	0.01817	569.858	593.162	537.599	412.613	369.609	476.56	Down
Tmem47	2304.47	-0.6384	0.12261	-5.2069	1.92E-07	2.73E-06	2507.76	2946.65	2963.96	1732.35	1978.99	1697.12	Down
Actr3-ps	536.026	0.81936	0.14758	5.55179	2.83E-08	4.68E-07	440.213	356.472	366.675	636.544	726.545	689.705	Up
Dmd	2973.06	-0.7133	0.11941	-5.9736	2.32E-09	4.77E-08	3215.97	4039.06	3825.26	2333.65	2215.54	2208.87	Down
Gk	2411.95	1.1176	0.12862	8.68911	3.65E-18	2.76E-16	1435.77	1769.9	1359.75	3276.02	3285.29	3344.97	Up
5430427C	190.842	0.90853	0.25456	3.56896	0.00036	0.00232	168.345	129.365	100.263	265.399	274.567	207.113	Up
Pdk3	1972.79	0.60001	0.11019	5.44506	5.18E-08	8.19E-07	1644.75	1588.79	1471.47	2231.01	2587.26	2313.43	Up
Eif2s3x	2120.08	0.374	0.11881	3.14799	0.00164	0.00846	2053.04	1701.87	1785.63	2222.72	2445.75	2511.49	Up
Apoo	1028.17	-0.4305	0.18409	-2.3383	0.01937	0.06509	952.989	1497.76	1090.48	906.09	890.229	831.467	Down
Arhgef9	286.254	-0.778	0.18798	-4.1387	3.49E-05	0.0003	325.08	418.759	340.893	200.086	238.662	194.042	Down
Asb12	8.83159	-4.6136	1.38514	-3.3308	0.00087	0.00493	12.5775	29.706	8.59395	0	2.11205	0	Down
Eda2r	179.179	0.46162	0.20395	2.26338	0.02361	0.07614	151.898	169.612	130.819	190.756	202.757	229.231	Up
Ar	551.805	-0.8187	0.16546	-4.948	7.50E-07	9.56E-06	672.414	681.322	759.132	477.926	360.105	359.934	Down
Yipf6	8275.75	0.63329	0.09271	6.83099	8.43E-12	2.67E-10	6424.21	6530.53	6509.44	9579.25	9675.3	10935.7	Up
Stard8	5670.24	-0.6063	0.10842	-5.5918	2.25E-08	3.79E-07	6184.27	6990.5	7358.33	4218.4	4721.49	4548.43	Down
Efnb1	5254.99	-0.662	0.14319	-4.6234	3.78E-06	4.11E-05	5481.86	6419.37	7418.49	4241.2	4324.42	3644.58	Down
Pja1	3241.83	-0.3381	0.11522	-2.9344	0.00334	0.01544	3269.19	3572.39	4018.15	2974.34	2910.41	2706.54	Down

Tmem28	594.996	1.6456	0.17741	9.27572	1.76E-20	1.61E-18	328.95	267.354	268.322	819.006	1073.98	812.364	Up
Eda	237.565	-0.602	0.226	-2.6638	0.00773	0.03091	359.911	259.688	239.676	213.563	181.636	170.918	Down
Awat1	350.02	10.0935	1.03966	9.70845	2.78E-22	2.96E-20	1.935	0	0	645.874	618.831	833.477	Up
Kif4	139.192	0.72966	0.23512	3.10339	0.00191	0.00961	123.84	104.45	85.9395	188.682	156.292	175.945	Up
Gdpd2	697.963	-2.6767	0.27836	-9.616	6.84E-22	7.03E-20	932.671	1377.02	1312.01	159.654	130.947	275.48	Down
Tex11	507.897	0.92697	0.27417	3.38094	0.00072	0.00424	516.646	262.563	271.187	590.928	765.618	640.44	Up
Foxo4	2229.27	-0.3089	0.09323	-3.3128	0.00092	0.00521	2572.59	2306.53	2521.85	1995.68	2005.39	1973.6	Down
Gjb1	333.105	2.15919	0.44408	4.86221	1.16E-06	1.41E-05	72.5626	233.815	59.2027	541.166	650.512	441.371	Up
Zmym3	2391.83	0.20038	0.09293	2.15624	0.03106	0.09415	2173.98	2360.19	2143.71	2615.63	2586.21	2471.28	Up
Itgb1bp2	361.442	-3.2483	0.41678	-7.7936	6.51E-15	3.24E-13	408.286	1127.87	425.878	72.5701	90.8182	43.2322	Down
Ogt	13346.1	-0.2405	0.08376	-2.8716	0.00408	0.0183	14129.4	13844	15394.6	12192.8	12120	12395.6	Down
Nhsl2	950.891	-0.4768	0.124	-3.8452	0.00012	0.00089	1094.24	1011.92	1213.66	748.509	822.644	814.375	Down
Ercc6l	86.6081	0.87442	0.37374	2.33963	0.0193	0.06495	69.6601	37.3721	76.3906	146.177	84.482	105.567	Up
Cited1	19.7511	1.97679	0.62302	3.17291	0.00151	0.00788	9.67501	8.62433	5.7293	18.6609	29.5687	46.2484	Up
Phka1	2988.95	0.89485	0.08777	10.1958	2.07E-24	2.61E-22	2155.59	2116.79	1999.52	3761.21	3883.01	4017.58	Up
Gm9109	1.91679	-4.3355	2.1141	-2.0508	0.04029	NA	1.935	4.79129	4.77441	0	0	0	Down
Gm6206	411.194	0.38709	0.17495	2.21257	0.02693	0.08447	382.163	330.599	356.171	448.898	550.189	399.144	Up
Rlim	4934.09	0.42894	0.10511	4.08091	4.49E-05	0.00037	4020.94	4040.02	4556.7	5441.72	6105.94	5439.22	Up
Gm24993	11.4829	1.47348	0.70056	2.10328	0.03544	0.10424	5.80501	4.79129	7.63906	22.8077	14.7844	13.0702	Up
Nexmif	815.815	1.39064	0.13443	10.3444	4.44E-25	5.91E-23	506.971	393.844	450.705	1158.01	1185.92	1199.44	Up
Abcb7	2236.02	0.26302	0.10873	2.41906	0.01556	0.05463	1907.91	2229.87	1960.37	2364.75	2571.42	2381.79	Up
Magee2	20.8438	1.44782	0.62359	2.32173	0.02025	0.06738	4.83751	18.2069	10.5037	32.1382	22.1765	37.1998	Up
5530601H	1414	-0.242	0.11216	-2.1577	0.03095	0.09394	1427.06	1531.3	1638.58	1204.66	1342.21	1340.2	Down
Atrx	7962.96	0.36939	0.08284	4.45907	8.23E-06	8.27E-05	6830.56	6779.68	7237.06	8701.16	8859	9370.34	Up
Atp7a	291.166	0.7891	0.22562	3.4975	0.00047	0.00294	217.688	220.399	202.435	298.574	326.312	481.587	Up
Tlr13	951.018	1.90902	0.26616	7.17249	7.36E-13	2.69E-11	512.776	429.3	257.818	1381.94	1918.8	1205.48	Up
Cysltr1	391.749	0.50527	0.17707	2.85344	0.00432	0.01919	307.665	329.641	334.209	371.144	512.172	495.663	Up
Rtl3	7.85638	-4.4464	1.38942	-3.2002	0.00137	0.00729	7.74001	23.9565	13.3684	2.07343	0	0	Down
Lpar4	81.4877	-0.5899	0.27451	-2.1488	0.03165	0.09547	89.0101	107.325	97.3981	53.9092	73.9218	67.3619	Down
P2ry10b	338.315	1.46375	0.2506	5.8409	5.19E-09	1.00E-07	210.915	196.443	132.729	462.375	627.279	400.15	Up
Itm2a	2031.14	1.38759	0.14214	9.76198	1.64E-22	1.80E-20	1181.32	1101.04	1087.61	2446.65	3041.35	3328.88	Up
2610002M	1545.78	0.25225	0.09062	2.78377	0.00537	0.023	1365.14	1431.64	1436.14	1640.08	1707.59	1694.1	Up
Gm379	36.1053	2.59521	0.46506	5.58034	2.40E-08	4.03E-07	15.48	6.70781	8.59395	64.2764	61.2495	60.3241	Up
Brwd3	1018.07	0.93745	0.10614	8.83197	1.03E-18	8.07E-17	721.756	685.155	688.471	1273.09	1409.79	1330.15	Up
Hmgn5	1386.15	-0.2269	0.10523	-2.1559	0.03109	0.09418	1374.82	1553.34	1556.46	1284.49	1303.14	1244.69	Down
Gm6377	255.459	1.11964	0.26444	4.23403	2.30E-05	0.00021	158.67	170.57	153.736	295.464	487.884	266.431	Up
Rps6ka6	172.183	-0.8951	0.22718	-3.9399	8.15E-05	0.00063	218.655	208.9	244.45	117.149	97.1543	146.789	Down
Zfp711	140.29	-0.8111	0.34787	-2.3316	0.01972	0.06609	141.255	193.568	201.48	97.4513	60.1934	147.794	Down
Nap1l3	281.282	-0.3792	0.17292	-2.1929	0.02832	0.08799	303.795	327.724	322.75	224.967	224.933	283.523	Down
Pcdh19	298.587	-0.6103	0.17626	-3.4623	0.00054	0.00328	306.698	371.804	403.915	221.857	243.942	243.307	Down
Tspan6	738.363	-0.2789	0.12191	-2.288	0.02213	0.07224	822.376	865.308	740.989	655.204	651.568	694.732	Down
Srpx2	341.346	-0.4066	0.17006	-2.3908	0.01681	0.05822	377.326	401.51	388.637	335.896	247.11	297.599	Down
Tmem35a	194.284	-0.9014	0.314	-2.8706	0.0041	0.01834	306.698	214.65	237.766	178.315	147.844	80.4321	Down
Btk	815.257	0.96537	0.22527	4.28529	1.82E-05	0.00017	743.041	513.627	400.096	992.137	1186.97	1055.67	Up
Rpl36a	4289.95	-0.3211	0.10609	-3.0265	0.00247	0.01194	4954.58	4267.13	5074.25	3804.75	3942.14	3696.86	Down
Gla	1049.34	1.11824	0.20567	5.43705	5.42E-08	8.54E-07	841.726	520.334	623.539	1346.69	1694.92	1268.82	Up
Armcx4	1972.56	-0.6431	0.15333	-4.1942	2.74E-05	0.00024	2485.51	1957.72	2772.03	1444.15	1529.12	1646.85	Down
Armcx1	1437.78	-0.4457	0.11939	-3.7334	0.00019	0.00133	1668.94	1485.3	1820.01	1211.92	1266.17	1174.31	Down
Armcx6	146.172	-0.7855	0.20529	-3.8263	0.00013	0.00096	195.435	165.779	193.841	111.965	102.434	107.578	Down
Zmat1	820.489	-0.5112	0.16318	-3.1331	0.00173	0.00882	832.051	953.467	1107.66	598.185	668.464	763.099	Down
Nxf7	28.3362	1.26292	0.59878	2.10917	0.03493	0.1031	25.155	8.62433	16.233	63.2397	30.6247	26.1404	Up
Bhlhb9	662.844	-0.5993	0.1335	-4.4886	7.17E-06	7.32E-05	895.906	730.193	769.636	524.578	525.901	530.852	Down
Arxes2	699.338	-1.1594	0.21012	-5.5177	3.43E-08	5.60E-07	863.979	1200.7	833.613	523.542	420.298	353.901	Down
Arxes1	29.6037	-2.2532	0.63803	-3.5316	0.00041	0.00263	38.7001	79.5355	28.6465	12.4406	4.2241	14.0756	Down
Bex4	637.326	-1.2805	0.2609	-4.9082	9.19E-07	1.14E-05	1076.83	606.578	1025.54	455.118	279.847	380.042	Down
Tceal8	2458.39	-0.582	0.1192	-4.8829	1.05E-06	1.29E-05	2726.42	2877.65	3238.96	2116.97	1802.64	1987.68	Down
Tceal9	11963.7	1.2927	0.10219	12.6496	1.12E-36	4.04E-34	6494.84	7178.32	7133.93	18348.8	16940.8	15685.3	Up
Bex3	4017.27	1.77001	0.09473	18.6857	6.48E-78	3.49E-74	1768.59	1859.98	1836.24	6452.52	6434.36	5751.9	Up
Tceal1	811.455	-0.4353	0.1307	-3.3304	0.00087	0.00494	911.386	1026.3	861.304	711.187	634.671	723.889	Down
Plp1	244.932	-0.8686	0.25179	-3.4496	0.00056	0.00342	215.753	391.928	341.848	175.205	167.908	176.951	Down
Slc25a53	492.315	-0.2743	0.13808	-1.9863	0.047	0.13004	541.801	511.71	563.381	401.209	477.323	458.463	Down
Nrk	7.25602	2.45666	1.03615	2.37095	0.01774	0.06074	1.935	0.95826	3.81953	12.4406	5.28013	19.1026	Up
Serpina7	100.228	-1.4314	0.30223	-4.7362	2.18E-06	2.49E-05	140.288	171.528	126.999	40.4319	51.7452	70.3781	Down
4930513C	18.7756	-1.524	0.60949	-2.5004	0.0124	0.04562	25.155	36.4138	21.9623	10.3672	13.7283	5.027	Down
Pwwp3b	270.047	-1.3319	0.23487	-5.6709	1.42E-08	2.49E-07	303.795	366.055	489.855	139.957	144.675	175.945	Down
Platr21	308.281	-0.7475	0.21006	-3.5586	0.00037	0.0024	339.593	335.391	484.126	242.592	242.886	205.102	Down
Radx	141.615	2.33817	0.29451	7.93925	2.03E-15	1.08E-13	53.2126	55.579	31.5111	199.049	285.127	225.21	Up
Rnf128	3535.9	1.85715	0.18271	10.1643	2.86E-24	3.55E-22	1535.42	1905.98	1147.77	5437.57	5946.48	5242.16	Up
Tbc1d8b	1190	0.69829	0.11842	5.89691	3.70E-09	7.37E-08	971.371	811.645	939.605	1471.1	1415.07	1531.23	Up
Cldn2	397.475	1.89306	0.29068	6.51256	7.39E-11	1.99E-09	147.06	226.149	132.729	554.643	464.651	859.618	Up
Morc4	232.696	-0.7993	0.22158	-3.6074	0.00031	0.00204	265.095	301.851	319.886	139.957	155.236	214.15	Down
Nup62cl	347.752	0.94313	0.21231	4.44228	8.90E-06	8.85E-05	194.468	271.187	248.27	525.615	365.385	481.587	Up
Tsc22d3	17487.1	-1.0866	0.16575	-6.5557	5.54E-11	1.53E-09	24143	23767.7	23423.3	13737.5	8668.91	11182.1	Down
Vsig1	131.401	2.06029	0.36826	5.59465	2.21E-08	3.73E-07	33.8626	56.5373	62.0674	278.877	128.835	228.226	Up
Psmd10	1567.69	0.32499	0.1036	3.13706	0.00171	0.00872	1425.13	1473.8	1276.68	1719.91	1756.17	1754.42	Up
Atg4a	1981.25	0.24771	0.09944	2.49114	0.01273	0.04654	1791.81	1937.6	1705.42	2120.08	2103.6	2228.97	Up
Col4a6	1031.64	-1.2786	0.16063	-7.9596	1.73E-15	9.26E-14	1307.09	1762.24	1313.92	598.185	564.974	643.457	Down
Col4a5	4545.02	-0.4515	0.08379	-5.3892	7.08E-08	1.08E-06	5441.23	5067.27	5243.26	3908.42	3691.86	3918.05	Down
Acsl4	15937.3	1.34447	0.14101	9.53425	1.51E-21	1.52E-19	10000.1	8260.19	8757.23	20675.2	21095.2	26836.2	Up
Tmem164	7024.53	0.84889	0.1182	7.18177	6.88E-13	2.53E-11	5435.42	4660.97	4950.11	8256.41	10047	8797.26	Up
Ammecr1	605.698	0.45182	0.12747	3.54465	0.00039	0.00251	472.141	515.543	547.148	660.388	718.097	720.872	Up
Chrdl1	1046.94	-1.0746	0.18821	-5.7092	1.14E-08	2.03E-07	1383.53	1767.99	1107.66	648.984	736.05	637.424	Down
Capn6	125.902	1.64938	0.25562	6.45258	1.10E-10	2.90E-09	75.4651	56.5373	50.6088	203.196	201.701	167.902	Up

Lhfpl1	24.1467	2.71205	0.58168	4.66241	3.13E-06	3.46E-05	3.87001	7.66607	7.63906	53.9092	29.5687	42.2268	Up
Htr2c	299.355	2.388	0.72711	3.28424	0.00102	0.00568	178.02	82.4102	27.6916	440.604	664.24	403.166	Up
Il13ra2	8.3061	2.45767	0.90478	2.71632	0.0066	0.02716	1.935	3.83303	1.90977	17.6242	8.4482	16.0864	Up
Gm5946	7.30675	-2.1012	0.98084	-2.1423	0.03217	0.09663	11.61	16.2904	7.63906	5.18358	2.11205	1.0054	Down
Pfkfb1	155.971	-0.6852	0.32696	-2.0956	0.03612	0.10588	156.735	284.603	135.593	108.855	140.451	109.589	Down
Tro	112.286	-0.834	0.34728	-2.4016	0.01632	0.05679	94.8151	169.612	167.105	106.782	77.0899	58.3133	Down
Maged2	2455.99	-0.3544	0.11043	-3.2098	0.00133	0.00709	2454.55	2857.53	2956.32	2155.33	2202.87	2109.33	Down
Hsd17b10	3534.56	0.62643	0.09715	6.44835	1.13E-10	2.98E-09	2995.38	2730.08	2611.6	4295.11	4427.91	4147.28	Up
Kdm5c	4346.94	0.44177	0.09629	4.58778	4.48E-06	4.79E-05	3614.59	3456.44	3988.55	4875.67	5136.51	5009.91	Up
Kantr	806.19	-0.2596	0.12018	-2.1598	0.03079	0.09356	869.784	823.144	942.469	725.701	778.291	697.748	Down
Gpr173	57.3939	-0.7298	0.37204	-1.9617	0.0498	0.1364	54.1801	86.2433	74.4809	38.3585	33.7928	57.3078	Down
Mageh1	706.104	-0.455	0.13986	-3.2533	0.00114	0.00625	859.141	706.237	884.222	625.14	579.758	582.127	Down
Rragb	218.267	-0.3962	0.16482	-2.4036	0.01623	0.05658	254.453	238.606	251.134	191.792	180.58	193.037	Down
Kctd12b	816.622	0.66852	0.17938	3.72695	0.00019	0.00136	747.879	641.075	503.223	952.742	1127.84	926.98	Up
Sat1	16565.1	0.65552	0.10615	6.17556	6.59E-10	1.50E-08	11821.9	13928.3	12845.1	21101.3	20962.1	18731.6	Up
Prdx4	2215.92	0.2928	0.11583	2.52779	0.01148	0.04284	2034.66	1948.14	1992.84	2638.44	2144.79	2536.63	Up
Ptchd1	9.6016	2.00056	0.83027	2.40953	0.01597	0.05586	4.83751	3.83303	2.86465	12.4406	9.50423	24.1296	Up
Phex	319.111	1.97874	0.28174	7.02324	2.17E-12	7.38E-11	176.085	127.448	84.0297	486.22	425.578	615.305	Up
Mbtps2	1448.66	0.20626	0.1015	2.03215	0.04214	0.11944	1354.5	1361.69	1319.65	1441.04	1632.62	1582.5	Up
Smpx	1132.37	-6.6589	1.74155	-3.8235	0.00013	0.00097	1113.59	4423.32	1190.74	6.2203	0	60.3241	Down
Klhl34	12.93	-2.1506	0.95508	-2.2518	0.02434	0.07794	19.35	29.706	14.3232	4.14686	0	10.054	Down
Cnksr2	119.827	-1.4955	0.46369	-3.2253	0.00126	0.00678	166.41	185.902	178.563	70.4967	20.0645	97.5239	Down
Rps6ka3	5116.4	0.42207	0.11058	3.81695	0.00014	0.00099	4170.9	4257.54	4691.34	5267.55	6206.26	6104.79	Up
Bclaf3	1195.27	-0.3271	0.12154	-2.691	0.00712	0.02895	1282.91	1254.36	1453.33	973.476	1084.54	1123.03	Down
Sh3kbp1	2359.17	-0.4992	0.21841	-2.2855	0.02228	0.07262	2083.03	3789.91	2416.81	1791.44	2158.52	1915.29	Down
Scml2	28.6267	-1.025	0.46534	-2.2028	0.02761	0.08615	36.7651	43.1216	35.3307	22.8077	11.6163	22.1188	Down
Rai2	336.941	-0.6917	0.18778	-3.6838	0.00023	0.00157	421.831	448.465	378.134	241.555	310.471	221.188	Down
Syap1	6013.99	0.3487	0.09285	3.75551	0.00017	0.00123	5042.62	5450.58	5378.86	6325	7136.62	6750.26	Up
Ctps2	2543.47	0.35142	0.0993	3.53914	0.0004	0.00256	2371.35	2158.96	2175.22	2882.07	2993.83	2679.39	Up
Ace2	1705.22	-0.9083	0.22953	-3.9571	7.59E-05	0.0006	2570.65	1989.34	2115.07	1246.13	790.963	1519.16	Down
Bmx	784.186	-0.935	0.14876	-6.2849	3.28E-10	7.92E-09	1150.36	1004.26	934.83	583.671	470.987	561.014	Down
Pir	2353.1	-0.4866	0.21549	-2.2581	0.02394	0.07697	3121.16	2864.24	2253.52	2150.15	1373.89	2355.65	Down
Vegfd	3502.34	-0.5003	0.15897	-3.147	0.00165	0.00848	4697.22	4415.66	3197.9	3050.02	2829.09	2824.17	Down
Piga	1004.51	0.52369	0.1359	3.85338	0.00012	0.00087	743.041	845.184	884.222	1051.23	1277.79	1225.58	Up
Fancb	256.036	-0.4617	0.19735	-2.3393	0.01932	0.06498	305.73	255.855	328.48	186.609	211.205	248.334	Down
Gpm6b	4126.23	-0.5928	0.12742	-4.6521	3.29E-06	3.62E-05	4443.73	5407.45	5035.1	3067.64	3688.7	3114.73	Down
Ofd1	1577.74	0.98237	0.1703	5.76865	7.99E-09	1.48E-07	1154.23	1015.75	1011.22	1669.11	2523.9	2092.24	Up
Rab9	3368.8	0.68365	0.10305	6.63406	3.27E-11	9.37E-10	2621.93	2465.6	2667.94	4169.67	4470.16	3817.51	Up
Tceanc	474.771	0.3874	0.14409	2.68861	0.00718	0.02909	443.116	377.554	413.464	526.652	587.15	500.69	Up
Egfl6	5266.72	1.44904	0.19333	7.49525	6.62E-14	2.91E-12	3689.08	2534.59	2247.79	6952.22	7524.18	8652.48	Up
Gm15226	4.12364	5.54539	1.68813	3.28493	0.00102	0.00567	0	0	0	5.18358	9.50423	10.054	Up
Rpl7a-ps1	148.495	-0.5034	0.19629	-2.5649	0.01032	0.03925	179.955	182.069	160.42	123.369	122.499	122.659	Down
Tmsb4x	131885	0.3596	0.15445	2.32823	0.0199	0.06656	113561	103289	129749	155408	168667	120637	Up
Tlr8	382.538	2.41836	0.31008	7.79914	6.23E-15	3.12E-13	178.02	115.949	67.7967	545.313	727.601	660.548	Up
Tlr7	909.704	1.86226	0.34768	5.35619	8.50E-08	1.27E-06	546.638	431.216	199.571	1109.29	2023.34	1148.17	Up
Prps2	5318.81	0.47899	0.15904	3.0118	0.0026	0.01245	5355.12	4167.47	3809.03	5728.89	7012.01	5840.37	Up
Arhgap6	237.768	-0.5668	0.17776	-3.1888	0.00143	0.00753	274.77	311.434	265.457	171.058	203.813	200.075	Down
BC022960	56.7774	-0.8659	0.34268	-2.527	0.01151	0.04291	87.0751	54.6207	78.3004	43.5421	35.9049	41.2214	Down

Supplementary Table 2 DEGs between the Pten$^{d/d}$ELF3^{OV} vs Pten$^{d/d}$ groups

gene_id	baseMean	log2FoldC	lfcSE	stat	pvalue	padj	Ptendd1	Ptendd2	Ptendd3	PtenddOv	PtenddOv	PtenddOv	change
Rp1	1625.55	-0.9888	0.3159	-3.1302	0.00175	0.01688	2664.44	1344.5	2476.53	1165.53	673.815	1428.49	Down
Sox17	4730.42	0.61845	0.15263	4.05209	5.08E-05	0.00106	3323.45	4289.98	3581.76	6197.83	6106.58	4882.91	Up
Rgs20	51.2066	-1.8771	0.53789	-3.4898	0.00048	0.00629	68.5449	39.4572	133.524	16.3154	19.5757	29.8224	Down
Alkal1	1171.76	0.79955	0.15188	5.26434	1.41E-07	7.46E-06	872.479	978.538	714.402	1595.85	1452.72	1416.56	Up
Adhfe1	422.573	-0.434	0.18905	-2.2959	0.02168	0.10773	555.214	516.889	384.978	340.585	344.12	393.655	Down
Sgk3	1476.66	0.39711	0.13551	2.93053	0.00338	0.0281	1183.87	1463.86	1176.38	1628.48	1799.93	1607.42	Up
Snhg6	257.629	-0.3835	0.17915	-2.1406	0.03231	0.14237	297.681	270.282	307.008	259.008	205.029	206.768	Down
Ppp1r42	292.419	-1.1795	0.37391	-3.1546	0.00161	0.01583	500.378	201.232	515.578	203.943	123.636	209.751	Down
Cops5	3610.11	-0.2358	0.11889	-1.9834	0.04732	0.18428	3943.29	3626.12	4144.12	3583.28	2963.14	3400.74	Down
Cspp1	3188.79	-0.3897	0.19576	-1.9905	0.04653	0.18212	3780.74	2761.02	4308.83	2886.81	2304.78	3090.59	Down
A830018L	308.334	-0.9297	0.27445	-3.3874	0.00071	0.00834	421.061	310.725	481.466	200.884	154.545	281.324	Down
Eya1	498.688	-0.736	0.25959	-2.8352	0.00458	0.03511	779.453	698.392	391.8	347.723	378.12	396.637	Down
4930444P	546.156	-1.3795	0.32368	-4.262	2.03E-05	0.0005	1057.55	467.568	842.078	350.782	206.06	352.898	Down
Ly96	403.038	0.38197	0.19265	1.98272	0.0474	0.18447	389.727	354.128	306.033	457.852	534.725	375.762	Up
Il17a	14.4037	4.39884	1.5102	2.91276	0.00358	0.02934	1.95843	0	1.94925	39.7689	0	42.7454	Up
Il17f	14.2294	2.51275	1.06845	2.35178	0.01868	0.0977	7.8337	1.97286	2.92388	39.7689	2.0606	30.8164	Up
Mcm3	1159.78	0.35522	0.12931	2.74701	0.00601	0.04281	913.605	992.348	1147.14	1257.31	1358.96	1289.32	Up
Efhc1	2816.22	-0.8527	0.26986	-3.1597	0.00158	0.01562	4159.7	2556.83	4158.73	2188.31	1317.75	2516.01	Down
Gsta3	645.954	-0.8468	0.30142	-2.8093	0.00497	0.03716	699.158	596.79	1194.89	497.621	326.605	560.66	Down
Ogfrl1	2340.83	0.96604	0.11833	8.16386	3.24E-16	1.10E-13	1410.07	1579.27	1766.02	3059.14	3155.8	3074.68	Up
Smap1	764.536	0.2906	0.11591	2.507	0.01218	0.07136	700.137	710.229	653	799.456	895.329	829.061	Up
Gm28437	1804.82	0.3457	0.16993	2.03437	0.04191	0.17006	1452.17	1621.69	1694.88	1718.22	2484.05	1857.93	Up
Gm9898	21.1125	-1.0891	0.49716	-2.1906	0.02848	0.13045	27.418	24.6607	34.112	14.276	10.303	15.9053	Down
4931428L	311.727	-0.9841	0.27833	-3.5357	0.00041	0.0055	409.311	303.82	529.223	252.889	151.454	223.668	Down
Gm37724	5.21138	2.80528	1.16036	2.41759	0.01562	NA	1.95843	0	1.94925	9.17743	8.24239	9.94079	Up
Rab23	1938.28	-0.5697	0.19133	-2.9776	0.00291	0.02507	2476.43	1821.94	2650.01	1612.17	1284.78	1784.37	Down
Bag2	1129.84	-0.6028	0.26776	-2.2513	0.02436	0.11669	1624.51	826.628	1636.4	1002.38	739.754	949.345	Down
Bend6	160.685	1.32114	0.28289	4.67021	3.01E-06	0.0001	73.441	113.439	88.6911	175.391	288.484	224.662	Up
Ccdc115	1155.18	0.41525	0.15694	2.64591	0.00815	0.05324	1034.05	1110.72	825.509	1452.07	1206.48	1302.24	Up
Ptpn18	933.549	0.49358	0.1295	3.81145	0.00014	0.00241	853.874	696.419	775.803	1154.32	1089.03	1031.85	Up
Fam168b	7921.8	0.2115	0.0844	2.50594	0.01221	0.07151	7590.86	7534.35	6901.34	8388.17	8597.84	8518.26	Up
Plekhb2	10401.1	0.51426	0.12286	4.1858	2.84E-05	0.00065	8974.48	9364.18	7361.36	12670	12202.9	11833.5	Up
Uggt1	5364.94	0.20696	0.08173	2.53233	0.01133	0.06771	4916.63	5193.55	4832.2	5629.85	5898.46	5718.93	Up
Neurl3	1889.9	0.43633	0.21233	2.05489	0.03989	0.16477	1477.63	2137.59	1203.66	2063.9	2124.48	2332.11	Up
Cnnm4	608.627	-0.3916	0.15203	-2.5756	0.01001	0.06193	781.412	595.803	694.909	559.823	505.877	513.939	Down
Fam178b	78.7025	-0.6375	0.31104	-2.0494	0.04042	0.16592	98.9005	117.385	71.1478	71.38	58.727	54.6743	Down
Zap70	553.48	-0.671	0.22074	-3.0399	0.00237	0.02148	650.197	642.166	747.539	476.207	298.787	505.986	Down
Vwa3b	1277.95	-0.8885	0.3036	-2.9265	0.00343	0.02835	1951.57	1122.56	1904.42	989.123	541.937	1158.1	Down
Mgat4a	1901.29	0.32548	0.13905	2.3407	0.01925	0.09967	1807.63	1405.66	1849.84	2087.36	2081.2	2176.04	Up
Tsga10	2200.89	-0.6774	0.23842	-2.8411	0.0045	0.03457	2969.95	1890.99	3264.03	1666.21	1342.48	2071.66	Down
Aff3	1712.65	-0.2998	0.117	-2.5622	0.0104	0.0637	1782.17	1878.16	2009.68	1471.45	1445.51	1688.94	Down
Npas2	753.999	-2.322	0.33606	-6.9094	4.87E-12	6.67E-10	1517.78	1624.65	627.66	221.278	220.484	312.141	Down
Rnf149	2955.05	0.72603	0.12975	5.59572	2.20E-08	1.46E-06	2243.38	2411.82	2025.28	3243.71	3948.1	3858.02	Up
Il1r1	2620.76	-0.2963	0.12796	-2.3154	0.02059	0.1044	3041.43	3083.58	2541.83	2145.48	2502.59	2409.65	Down
Il1rl1	159.401	0.64621	0.31623	2.04344	0.04101	0.16746	143.944	154.869	74.0717	229.436	175.151	178.934	Up
Il18rap	727.935	0.85703	0.15625	5.48514	4.13E-08	2.55E-06	482.752	537.604	533.121	967.709	1058.12	788.304	Up
Slc9a4	823.918	1.54044	0.24609	6.25956	3.86E-10	3.65E-08	547.38	404.436	312.855	1229.78	1488.78	960.28	Up
Mrps9	2123.5	-0.3238	0.11357	-2.8509	0.00436	0.03379	2232.61	2610.09	2239.69	1823.25	1985.39	1849.98	Down
Fhl2	828.739	-1.2826	0.64188	-1.9981	0.0457	0.1798	1779.23	497.161	1247.52	534.331	244.181	670.009	Down
Nck2	2183.45	-0.3239	0.10746	-3.014	0.00258	0.02296	2506.78	2211.58	2564.24	1922.16	1871.02	2024.94	Down
Col5a2	2942.34	0.4926	0.16806	2.93113	0.00338	0.02809	2491.12	2891.23	1952.18	3673.01	3537.01	3109.48	Up
Dnah7b	952.12	-1.1209	0.28645	-3.9132	9.11E-05	0.00172	1429.65	935.135	1548.68	657.716	377.089	764.446	Down
Dnah7c	935.287	-1.8088	0.29405	-6.1512	7.69E-10	6.96E-08	2101.39	1083.1	1181.25	424.201	301.877	519.903	Down
Tmeff2	517.396	-0.7844	0.23581	-3.3265	0.00088	0.0099	682.511	802.954	478.542	353.841	458.483	328.046	Down
Nabp1	3562.66	1.00603	0.18023	5.58184	2.38E-08	1.55E-06	2460.76	2405.9	2238.72	5019.04	5673.85	3577.69	Up
Gm553	101.01	-0.7114	0.25915	-2.745	0.00605	0.04302	130.235	124.29	121.828	77.4983	59.7573	92.4493	Down
Gm5527	31.2563	-1.1233	0.52178	-2.1528	0.03133	0.13895	26.4387	59.1858	42.8836	14.276	28.8484	15.9053	Down
Osgepl1	575.483	-0.3704	0.16002	-2.3146	0.02064	0.10448	538.567	726.999	681.264	492.522	525.452	488.093	Down
Dnah7a	1264.56	-0.9576	0.2769	-3.4581	0.00054	0.00687	1795.88	1163	2049.64	898.369	580.058	1100.44	Down
Hecw2	2199.2	-0.387	0.18905	-2.047	0.04066	0.1665	3114.88	2030.07	2332.28	1661.12	1923.57	2133.29	Down
Pgap1	623.644	0.52969	0.14476	3.65912	0.00025	0.00384	445.542	577.061	508.755	727.057	715.027	768.423	Up
Ankrd44	4762.28	-0.3836	0.10984	-3.4924	0.00048	0.00624	5375.88	5264.57	5534.91	3745.41	4085.13	4567.79	Down
Hspe1	2211.68	-0.3432	0.14935	-2.2983	0.02154	0.10725	2729.07	2085.31	2606.15	2138.34	1960.66	1750.57	Down
Rftn2	938.89	-0.6764	0.20482	-3.3022	0.00096	0.01064	1122.18	1455.97	886.911	691.367	811.875	665.039	Down
Gm10561	101.907	-1.0609	0.38314	-2.7689	0.00562	0.04074	143.944	193.34	76.0209	61.1829	78.3027	58.6506	Down
Spats2l	1230.73	-0.3256	0.15445	-2.1082	0.03501	0.15062	1562.82	1171.88	1372.28	1020.73	1218.84	1037.82	Down
Aox1	4629.92	-0.6052	0.18525	-3.2667	0.00109	0.01178	4921.52	4700.34	7139.14	3732.16	3302.11	3984.27	Down
Aox3	5462.49	-0.6092	0.2211	-2.7552	0.00587	0.04202	6765.38	5524.01	7507.55	5020.06	2978.59	4979.34	Down
Flacc1	131.274	-0.7326	0.22682	-3.2299	0.00124	0.01305	154.716	147.964	189.078	100.952	87.5754	107.36	Down
Casp8	3648.53	0.44511	0.15324	2.90462	0.00368	0.02989	2934.7	3628.09	2707.51	4095.18	4707.43	3818.26	Up
Tmem237	1334.68	-0.393	0.12745	-3.0839	0.00204	0.01909	1434.55	1437.23	1674.41	1062.54	1148.78	1250.55	Down
Mpp4	23.1054	-1.5289	0.59833	-2.5554	0.01061	0.06467	31.3348	35.5115	36.0612	18.3549	13.3939	3.97631	Down
Als2	419.824	0.45321	0.13599	3.33269	0.00086	0.00974	354.475	351.169	357.688	452.753	475.998	526.862	Up
Gm26813	90.3033	0.87446	0.37672	2.32122	0.02027	0.10319	50.9191	74.9687	65.3	124.405	157.636	68.5914	Up
Gm973	215.305	-1.1914	0.48239	-2.4698	0.01352	0.07722	384.831	168.679	345.018	92.7941	64.9088	235.597	Down
Ica1l	209.403	-0.8581	0.25086	-3.4208	0.00062	0.00767	269.284	208.137	332.348	125.425	145.272	175.952	Down
Eif4a-ps4	2478.96	0.24956	0.10231	2.43931	0.01472	0.08234	2443.14	2228.34	2123.71	2677.77	2799.32	2601.5	Up
Pard3b	1550.35	-0.2207	0.109	-2.0252	0.04285	0.17231	1593.18	1699.62	1713.39	1309.31	1509.39	1477.2	Down
Nrp2	4018.6	0.59575	0.15136	3.93593	8.29E-05	0.00159	2939.6	3768.16	2893.67	4382.73	5393.61	4733.8	Up
Ino80dos	207.162	-0.5502	0.22205	-2.4778	0.01322	0.07589	288.868	202.218	247.555	160.095	149.393	194.839	Down
Gm39653	5.24652	-2.3814	1.10111	-2.1627	0.03056	NA	7.8337	4.93215	13.6448	1.01971	2.0606	1.98816	Down
Dytn	13.7553	-2.6854	0.808	-3.3235	0.00089	0.00998	29.3764	14.7964	27.2896	1.01971	3.0909	6.95855	Down

Mdh1b	666.874	-1.3022	0.32974	-3.9491	7.85E-05	0.00152	1231.85	578.048	1037	424.201	240.06	490.081	Down
Fzd5	1659.53	0.25297	0.11703	2.16167	0.03064	0.13706	1419.86	1681.86	1441.47	1852.82	1848.36	1712.8	Up
Pikfyve	1872.74	0.24543	0.09702	2.52959	0.01142	0.06802	1642.14	1749.93	1749.46	1914	2087.38	2093.53	Up
Rpe	2411.03	0.25714	0.08941	2.87578	0.00403	0.03185	2216.94	2219.47	2153.93	2511.56	2608.72	2755.59	Up
Erbb4	50.4863	-2.3514	0.68657	-3.4248	0.00062	0.00758	89.1084	37.4843	126.702	30.5914	4.12119	14.9112	Down
Spag16	4798.94	-0.6987	0.28734	-2.4315	0.01503	0.08356	7073.83	3684.31	7058.25	3721.96	2559.26	4696.03	Down
Fn1	29144.7	0.77098	0.21534	3.58021	0.00034	0.00484	19677.3	28153.7	16781.1	33325.3	44332.7	32597.8	Up
Mreg	439.031	1.08643	0.17894	6.07165	1.27E-09	1.10E-07	253.616	334.4	255.352	548.607	667.633	574.577	Up
Tmem169	8.24346	1.67974	0.82081	2.04645	0.04071	0.16655	2.93764	3.94572	4.87314	12.2366	17.5151	7.95263	Up
Igfbp2	7871.73	-1.4301	0.22748	-6.2868	3.24E-10	3.10E-08	8503.48	11881.5	14061.9	4775.32	4830.04	3178.07	Down
Igfbp5	20121	-0.974	0.19118	-5.0948	3.49E-07	1.65E-05	25822.8	21612.7	32564.2	15316.1	11008.7	14401.2	Down
Pinc	3.11342	-5.0599	1.95044	-2.5942	0.00948	NA	1.95843	12.8236	3.89851	0	0	0	Down
C530043A	795.824	-2.7416	0.76123	-3.6015	0.00032	0.00455	1942.76	629.342	1581.82	162.135	74.1815	384.708	Down
Gm29186	34.3438	-2.074	0.45843	-4.5241	6.06E-06	0.00018	69.5241	42.4165	54.5791	9.17743	15.4545	14.9112	Down
6030407C	18.9939	-1.2625	0.52907	-2.3863	0.01702	0.09177	26.4387	31.5657	22.4164	9.17743	14.4242	9.94079	Down
Rufy4	302.878	2.48487	0.19968	12.4444	1.50E-35	4.06E-32	80.2954	97.6565	97.4627	464.99	601.694	475.17	Up
Cxcr1	364.215	3.87703	0.33258	11.6576	2.10E-31	3.97E-28	51.8983	52.2808	35.0866	521.074	1018.97	505.986	Up
Arpc2	20047.8	0.31563	0.12473	2.53053	0.01139	0.06793	17386.9	19541.2	16662.2	21998.3	24748.8	19949.2	Up
Pnkd	1899.97	-0.3444	0.12382	-2.7815	0.00541	0.03957	1966.26	2323.04	2087.65	1826.31	1656.72	1539.83	Down
Catip	2389.68	-0.9914	0.32576	-3.0433	0.00234	0.02129	3617.21	1684.82	4237.68	1887.49	1108.6	1802.26	Down
Slc11a1	673.702	0.67689	0.21507	3.14733	0.00165	0.01612	406.373	654.989	494.136	704.623	953.026	829.061	Up
Vil1	153.538	1.14204	0.31785	3.59305	0.00033	0.00466	57.7736	122.317	107.209	222.298	172.06	239.573	Up
Stk36	1793.02	-0.8749	0.23126	-3.7833	0.00015	0.00262	2611.56	1604.92	2745.53	1256.29	1044.72	1495.09	Down
Cfap65	2497.19	-0.6782	0.33983	-1.9957	0.04597	0.18058	3670.09	1806.15	3744.52	2033.31	1110.66	2618.4	Down
Ihh	101.276	-1.4718	0.287	-5.1281	2.93E-07	1.43E-05	150.799	130.209	165.687	62.2026	38.121	60.6388	Down
Slc23a3	122.448	0.78843	0.32535	2.42334	0.01538	0.08497	102.817	68.0636	98.4374	150.918	207.09	107.36	Up
Atg9a	710.216	0.4021	0.11034	3.64411	0.00027	0.004	647.26	594.817	593.548	812.713	807.754	805.204	Up
Tuba4a	4065.95	0.28766	0.08165	3.52299	0.00043	0.00567	3817.95	3534.38	3633.41	4478.59	4511.68	4419.67	Up
Dnajb2	5428.61	-0.348	0.11555	-3.0114	0.0026	0.02304	6562.68	5609.83	6068.03	4961.93	4326.22	5042.96	Down
Obsl1	575.875	-0.5754	0.22142	-2.5987	0.00936	0.05876	732.451	482.364	852.799	464.99	456.422	466.223	Down
Inha	379.941	-0.9587	0.33655	-2.8486	0.00439	0.03395	365.246	382.735	757.285	255.948	184.423	334.01	Down
Stk11ip	2049.74	-0.3664	0.11561	-3.1688	0.00153	0.01526	2389.28	2075.45	2460.93	1854.86	1834.96	1682.97	Down
Slc4a3	971.963	-0.6289	0.16504	-3.8105	0.00014	0.00242	1179.95	978.538	1383	704.623	823.208	762.458	Down
Epha4	875.244	0.5771	0.1606	3.59351	0.00033	0.00466	680.553	817.75	609.142	1005.44	1168.36	970.221	Up
Sgpp2	967.347	0.90721	0.13654	6.64439	3.04E-11	3.88E-09	712.867	710.229	595.497	1143.1	1304.36	1338.03	Up
Farsb	3408.16	0.28021	0.09721	2.88252	0.00395	0.03137	3269.59	3124.02	2841.04	3717.88	3820.35	3676.1	Up
Mogat1	250.409	2.25219	0.30806	7.31079	2.66E-13	4.83E-11	71.4825	91.738	97.4627	485.384	251.393	504.992	Up
5730419F	10.6538	-3.005	1.03482	-2.9039	0.00369	0.02995	6.85449	24.6607	25.3403	0	3.0909	3.97631	Down
Acsl3	3563.69	-0.4882	0.23895	-2.0433	0.04102	0.16747	4881.38	2907.99	4693.81	2966.35	2289.32	3643.3	Down
Ap1s3	848.869	0.52934	0.17611	3.00569	0.00265	0.02337	668.802	798.022	617.914	1027.87	831.451	1149.15	Up
Mrpl44	1148.78	-0.3081	0.10274	-2.9984	0.00271	0.0238	1291.58	1288.28	1232.9	1040.11	1073.57	966.244	Down
Serpine2	7613.49	-0.2016	0.08272	-2.4374	0.01479	0.08265	8110.82	7965.42	8357.43	7154.32	7349.12	6743.83	Down
2310015K	1.96259	-4.3943	2.07674	-2.116	0.03435	NA	1.95843	5.91858	3.89851	0	0	0	Down
Dock10	3356.29	0.53977	0.17473	3.08919	0.00201	0.01884	2506.78	3140.79	2559.37	3270.23	4782.65	3877.9	Up
Daw1	1105.56	-0.9763	0.32654	-2.99	0.00279	0.0243	1696.98	929.217	1771.87	927.941	428.604	878.765	Down
Fbxo36	1594.64	-1.0576	0.22147	-4.7754	1.79E-06	6.53E-05	2523.43	1553.63	2385.89	1111.49	827.33	1166.05	Down
Gm29284	59.7288	-1.5264	0.47623	-3.2051	0.00135	0.01388	72.4617	47.3486	146.194	32.6309	30.909	28.8283	Down
C130026I2	516.168	-0.9867	0.29352	-3.3616	0.00077	0.00897	609.07	493.215	956.109	350.782	252.423	435.406	Down
AC147806	98.107	-0.5981	0.28314	-2.1124	0.03465	0.14947	135.131	85.8194	133.524	79.5378	72.1209	82.5085	Down
A630001G	1007.39	0.36516	0.14871	2.45542	0.01407	0.07957	727.555	976.565	937.591	1124.75	1171.45	1106.41	Up
Gm28100	9.56673	-1.8793	0.95757	-1.9626	0.0497	0.19018	11.7506	17.7557	15.594	6.11829	6.18179	0	Down
Gm18180	76.5214	-2.0171	0.81424	-2.4773	0.01324	0.07593	152.757	89.7651	125.727	5.09857	8.24239	77.5381	Down
Spata3	27.384	-2.3929	0.62671	-3.8182	0.00013	0.00236	73.441	24.6607	39.9597	7.138	6.18179	12.923	Down
2810459M	157.36	0.88948	0.27877	3.19067	0.00142	0.01433	83.2331	94.6972	153.016	209.042	214.302	189.869	Up
Psmd1	4941.91	0.26303	0.07667	3.43075	0.0006	0.00746	4616.01	4451.76	4410.19	5297.42	5482.22	5393.87	Up
Armc9	695.517	-0.241	0.11727	-2.0548	0.0399	0.16477	758.89	702.338	799.194	628.144	627.452	657.086	Down
B3gnt7	1839.99	1.71377	0.15889	10.7857	4.02E-27	5.85E-24	895.98	819.723	863.52	2950.04	3238.23	2272.46	Up
Tex44	10.9508	-3.5837	0.99761	-3.5923	0.00033	0.00467	22.5219	10.8507	27.2896	0	2.0606	2.98224	Down
Efhd1	276.397	2.16175	0.20321	10.6383	1.98E-26	2.49E-23	111.63	107.521	83.8179	387.492	471.877	496.045	Up
Ngef	759.419	-0.945	0.25709	-3.6758	0.00024	0.00366	920.46	687.541	1390.79	585.316	486.301	486.104	Down
Neu2	132.312	-1.0086	0.35374	-2.8514	0.00435	0.03377	173.321	103.575	253.403	76.4786	90.6663	96.4256	Down
Inpp5d	2764	0.29347	0.10992	2.66987	0.00759	0.05041	2531.26	2298.38	2621.75	2908.23	3293.86	2930.54	Up
Sag	88.9882	-1.0065	0.28277	-3.5594	0.00037	0.0051	121.422	96.6701	138.397	48.9463	63.8785	64.6151	Down
Dgkd	5889.28	0.28655	0.11386	2.51658	0.01185	0.06991	4721.76	5592.07	5605.08	6556.77	6775.24	6084.75	Up
Usp40	1663.33	-0.2715	0.10192	-2.6635	0.00773	0.05107	1721.46	1869.28	1867.39	1415.36	1554.72	1551.76	Down
Spp2	391.687	-0.8948	0.35763	-2.502	0.01235	0.07199	626.696	332.427	569.182	293.678	149.393	378.744	Down
Gm29538	322.575	-1.5056	0.26136	-5.7606	8.38E-09	6.29E-07	493.523	353.142	584.776	180.49	125.696	197.822	Down
Gm28888	9.52821	-1.7464	0.79015	-2.2102	0.02709	0.12556	12.7298	12.8236	18.5179	4.07886	2.0606	6.95855	Down
Gm19589	13.7373	-2.8439	0.94066	-3.0233	0.0025	0.02243	27.418	10.8507	34.112	4.07886	0	5.96447	Down
Arl4c	5735.53	-0.4417	0.12175	-3.6281	0.00029	0.0042	6971.02	7004.64	5844.84	4720.26	4587.92	5284.52	Down
Gm28342	11.0267	-2.6289	1.03459	-2.541	0.01105	0.06648	7.8337	29.5929	19.4925	3.05914	6.18179	0	Down
Iqca	2162.39	-1.0718	0.32747	-3.2729	0.00106	0.01157	3505.58	1871.26	3415.09	1405.17	813.936	1963.31	Down
Mlph	4230.95	1.20904	0.18648	6.48353	8.96E-11	9.69E-09	2923.93	1917.62	2823.5	5600.27	6689.73	5430.65	Up
Prlh	16.2352	1.28188	0.61187	2.095	0.03617	0.15426	7.8337	5.91858	14.6194	28.552	20.606	19.8816	Up
Lrrfip1	10464.4	0.31574	0.13371	2.36143	0.0182	0.09612	9575.72	10320	8075.76	10974.2	12800.4	11040.2	Up
Rbm44	2.94231	-3.9334	1.69361	-2.3225	0.02021	NA	6.85449	4.93215	4.87314	0	0	0.99408	Down
Ramp1	1745.08	-1.0239	0.17646	-5.8025	6.53E-09	5.02E-07	2596.87	2055.72	2366.4	1159.42	926.238	1365.86	Down
Espnl	99.6773	-1.5592	0.38084	-4.0941	4.24E-05	0.00091	185.071	111.467	150.093	52.0055	28.8484	70.5796	Down
1700020N	51.3246	-1.4639	0.51395	-2.8483	0.0044	0.03395	77.3578	45.3758	103.31	37.7295	12.3636	31.8105	Down
Hes6	4695.66	-0.7623	0.29182	-2.6121	0.009	0.05725	5003.78	3873.71	8846.69	4360.3	2962.11	3127.37	Down
Gm28382	101.104	-1.1445	0.54184	-2.1122	0.03467	0.14952	214.448	45.3758	157.89	65.2618	39.1513	84.4967	Down
Traf3ip1	5687.24	-0.751	0.26194	-2.8671	0.00414	0.03243	8030.52	4854.22	8520.19	4634.6	2942.53	5141.37	Down
Ankmy1	243.945	-0.9727	0.43827	-2.2194	0.02646	0.12377	453.376	140.073	376.206	176.411	98.9087	218.697	Down

Kif1a	1458.73	-0.5432	0.159	-3.4163	0.00063	0.00775	1509.95	1572.37	2108.12	1210.4	1175.57	1175.99	Down
Crocc2	1630.32	-0.8477	0.26304	-3.2226	0.00127	0.01328	2311.92	1427.36	2548.65	1370.5	824.239	1299.26	Down
Mterf4	1039.84	-0.4149	0.13134	-3.1587	0.00158	0.01566	1235.77	1142.29	1187.1	923.862	775.815	974.197	Down
Bok	1707.75	-0.9528	0.23136	-4.1183	3.82E-05	0.00083	2627.23	1498.39	2630.52	1258.33	999.389	1232.66	Down
Pdcd1	48.9583	1.29202	0.51225	2.52226	0.01166	0.06907	37.2101	19.7286	28.2642	46.9069	45.3331	116.307	Up
St8sia4	1384.52	0.6385	0.26423	2.41648	0.01567	0.08621	794.142	1539.82	915.175	1537.73	2069.87	1450.36	Up
Gm7135	52.5934	-0.6913	0.34889	-1.9814	0.04755	0.18493	77.3578	46.3622	71.1478	37.7295	41.2119	41.7513	Down
Gm28403	155.528	-0.6345	0.22892	-2.7719	0.00557	0.04045	202.697	202.218	162.763	108.09	111.272	146.13	Down
Tnfrsf11a	757.749	0.87139	0.1234	7.06167	1.65E-12	2.62E-10	530.733	587.912	488.288	987.084	964.359	988.114	Up
A530053N	35.5098	-1.5089	0.61172	-2.4667	0.01364	0.07773	82.2539	35.5115	39.9597	13.2563	9.27269	32.8046	Down
Ralb	4052.55	0.26576	0.10252	2.59241	0.00953	0.05967	3607.42	3912.18	3521.33	4384.77	4738.34	4151.27	Up
Cfap221	789.494	-1.2121	0.27037	-4.4833	7.35E-06	0.00022	1299.42	800.981	1208.54	463.97	333.817	630.246	Down
Sctr	4.73405	-4.6507	1.63634	-2.8421	0.00448	NA	12.7298	2.95929	11.6955	1.01971	0	0	Down
Marco	3830.48	3.43543	0.26955	12.745	3.32E-37	1.26E-33	484.71	813.804	646.178	4479.61	8065.18	8493.41	Up
Actr3	18207.8	0.48883	0.10638	4.59517	4.32E-06	0.00014	15133.7	15994	14329	20402.5	23353.8	20033.7	Up
Gpr39	458.255	0.43008	0.13933	3.08672	0.00202	0.01896	363.288	413.314	394.724	492.522	569.755	515.927	Up
Lypd1	66.0603	0.83946	0.28934	2.90131	0.00372	0.03013	42.1061	51.2943	48.7314	79.5378	77.2724	97.4197	Up
Map3k19	724.164	-1.0068	0.378	-2.6636	0.00773	0.05107	1186.81	582.98	1131.54	497.621	245.211	700.825	Down
Zranb3	438.556	1.00493	0.18779	5.35133	8.73E-08	4.98E-06	266.346	287.051	321.627	543.508	707.815	504.992	Up
Cd55b	120.936	-1.1353	0.34069	-3.3323	0.00086	0.00975	102.817	173.612	222.215	84.6363	61.8179	80.5204	Down
Pigr	39238.8	1.4526	0.47487	3.05895	0.00222	0.02047	25274.5	10987.8	26737.9	63752.6	32496.6	76183.2	Up
Dyrk3	822.225	-0.667	0.20305	-3.285	0.00102	0.01115	1135.89	762.51	1128.62	657.716	538.846	709.772	Down
Rab7b	470.952	0.56403	0.159	3.54727	0.00039	0.00531	357.413	408.382	374.257	567.981	631.573	486.104	Up
Slc26a9	6765.46	0.53185	0.24677	2.15529	0.03114	0.13833	6135.75	6563.7	3897.53	7670.3	10284.4	6041.02	Up
Pm20d1	708.167	-0.419	0.17341	-2.4163	0.01568	0.08622	874.437	825.642	730.97	555.745	529.573	732.636	Down
Elk4	61.9466	0.7592	0.36406	2.0854	0.03703	0.15677	44.0646	30.5793	63.3508	82.5969	84.4845	66.6033	Up
Mfsd4a	2023.21	2.21143	0.29454	7.50806	6.00E-14	1.26E-11	857.79	373.857	923.947	3671.99	3089.87	3221.81	Up
Cdk18	1908.22	0.2825	0.13107	2.15535	0.03113	0.13833	1582.41	1850.54	1732.89	2229.1	2220.29	1834.07	Up
Gm29630	1.69804	4.23483	2.14613	1.97324	0.04847	NA	0	0	0	4.07886	4.12119	1.98816	Up
Klhdc8a	844.468	0.33032	0.12563	2.6294	0.00855	0.05523	676.636	798.022	769.956	1001.36	924.178	896.659	Up
Tmcc2	7059.18	-0.365	0.14384	-2.5377	0.01116	0.06699	8285.12	8824.6	6732.73	5928.62	6871.06	5712.97	Down
Ppp1r15b	7027.72	0.20989	0.08015	2.61873	0.00883	0.05645	6548.97	6251.99	6751.24	7401.09	7511.91	7701.13	Up
Plekha6	4375.59	0.28913	0.14624	1.97707	0.04803	0.18622	3881.6	3403.18	4531.04	4594.84	4481.8	5361.07	Up
Prelp	987.831	0.49233	0.14229	3.46014	0.00054	0.00683	894.021	859.18	709.529	1053.37	1222.96	1187.92	Up
Chil1	131589	1.08366	0.16555	6.5456	5.93E-11	6.96E-09	69816.9	78559.3	104729	189802	170553	176076	Up
Ppfia4	510.901	0.67612	0.18459	3.66286	0.00025	0.00379	366.226	488.283	325.526	640.381	633.634	611.358	Up
Tmem183	4312.77	0.214	0.08228	2.60083	0.0093	0.05851	4032.4	4063.1	3884.86	4568.32	4820.77	4507.15	Up
Cyb5r1	2990.38	0.42933	0.13676	3.13922	0.00169	0.01651	2494.05	2768.91	2382.96	3389.53	3900.71	3006.09	Up
Klhl12	1323.57	-0.2448	0.12204	-2.0058	0.04488	0.17769	1395.38	1309.98	1601.31	1178.79	1260.06	1195.88	Down
Rabif	2458.6	-0.6167	0.20603	-2.9931	0.00276	0.0241	3429.2	3378.52	2120.79	2030.25	2103.87	1688.94	Down
Ube2t	128.997	-0.5321	0.23451	-2.2692	0.02326	0.11296	148.84	144.019	164.712	126.445	87.5754	102.39	Down
Lgr6	368.382	-2.3333	0.92013	-2.5358	0.01122	0.06729	1101.61	84.8329	657.873	101.971	157.636	106.366	Down
Ptpn7	395.828	0.41116	0.14147	2.90627	0.00366	0.02981	347.621	355.115	316.754	423.182	448.18	484.116	Up
Gpr37l1	30.538	-1.8192	0.57322	-3.1736	0.00151	0.01503	41.1269	25.6472	76.0209	12.2366	10.303	17.8934	Down
Rnpep	3533.29	0.40545	0.10796	3.75551	0.00017	0.00286	3067.87	2901.09	3150.97	4111.49	4320.04	3648.27	Up
Lmod1	259.707	-0.4644	0.21382	-2.1719	0.02986	0.13461	282.992	282.119	338.196	219.239	262.726	172.97	Down
Shisa4	366.39	-0.7883	0.26002	-3.0317	0.00243	0.02193	436.729	424.165	531.172	231.475	373.998	200.804	Down
Csrp1	21118.4	0.37285	0.08206	4.54388	5.52E-06	0.00017	18207.5	18197.7	18808.4	22938.5	25181.5	23376.8	Up
Tnnt2	976.134	-2.9052	1.26036	-2.3051	0.02116	0.10633	2634.08	77.9279	2455.09	240.653	77.2724	371.785	Down
Tmem9	2311.15	-0.2459	0.11265	-2.1833	0.02902	0.13197	2442.16	2459.17	2621.75	2317.81	1923.57	2102.48	Down
Kif21b	1196.91	0.5854	0.24308	2.40831	0.01603	0.08767	734.41	1330.69	806.991	1262.41	1672.17	1374.81	Up
Camsap2	320.656	0.7635	0.27402	2.78627	0.00533	0.03916	292.785	198.272	222.215	333.447	323.514	553.702	Up
Gm19705	198.648	-0.5139	0.1857	-2.7673	0.00565	0.04091	250.678	248.58	201.748	160.095	162.787	167.999	Down
Ptprc	6217.51	0.67179	0.17332	3.87608	0.00011	0.00196	4153.82	5960.01	4272.77	6896.33	8498.93	7523.19	Up
Rgs2	5237.37	0.88539	0.32708	2.70694	0.00679	0.04656	2294.3	5042.63	3699.69	6892.25	9352.02	4143.32	Up
Rgs1	667.408	1.45134	0.31516	4.60501	4.12E-06	0.00013	222.281	497.161	352.815	1287.9	935.511	708.778	Up
Rgs18	314.714	-0.6689	0.15119	-4.4242	9.68E-06	0.00027	404.415	362.02	392.775	224.337	247.272	257.466	Down
Ptgs2os2	593.029	0.67729	0.17651	3.83709	0.00012	0.00222	495.482	417.26	456.126	684.229	877.814	627.264	Up
Fam129a	4019.39	0.42887	0.10109	4.24261	2.21E-05	0.00053	3534.96	3457.44	3286.44	4611.15	4956.77	4269.57	Up
Tsen15	906.245	-0.2947	0.10994	-2.6809	0.00734	0.04925	1021.32	1022.93	951.236	863.699	783.027	795.263	Down
Arpc5	16018.4	0.32752	0.11357	2.88392	0.00393	0.03129	13677.6	14622.8	14323.1	17941.9	19703.4	15841.6	Up
Ncf2	2202.36	0.91275	0.19454	4.69179	2.71E-06	9.28E-05	1405.17	1926.5	1252.4	2851.12	3291.8	2487.18	Up
Lamc2	13416	0.62654	0.15115	4.14506	3.40E-05	0.00076	8686.6	12409.3	10547.4	15622	17405.9	15824.7	Up
Lamc1	7425.54	0.27036	0.08608	3.14078	0.00168	0.01647	6703.69	6993.79	6497.84	8123.05	8472.14	7762.76	Up
Shcbp1l	50.58	0.7192	0.31654	2.27208	0.02308	0.11232	41.1269	37.4843	36.0612	56.0843	71.0906	61.6329	Up
Dhx9	4888.07	0.30838	0.11793	2.61489	0.00893	0.05693	4701.2	3949.66	4452.1	5080.22	5248.34	5896.87	Up
Npl	459.725	-0.792	0.23738	-3.3364	0.00085	0.00966	627.675	412.328	708.554	344.664	285.393	379.738	Down
Rnasel	1598.9	0.9486	0.12516	7.57884	3.49E-14	7.67E-12	997.818	1189.63	1086.71	2029.23	2305.81	1984.18	Up
Gm28286	74.0414	3.62427	0.47284	7.66492	1.79E-14	4.23E-12	7.8337	11.8372	13.6448	85.6561	203.999	121.278	Up
Cacna1e	305.503	-0.5243	0.14861	-3.5281	0.00042	0.00562	355.454	381.748	344.043	235.554	262.726	253.49	Down
Ier5	3663.39	-0.367	0.11123	-3.2993	0.00097	0.01073	4408.42	3719.83	4252.3	3130.52	3077.5	3391.8	Down
Tdrd5	960.098	2.82054	0.37191	7.58401	3.35E-14	7.46E-12	198.78	324.535	191.027	1510.2	2661.26	874.789	Up
Nphs2	40.5964	4.17801	0.5616	7.43951	1.01E-13	1.97E-11	4.89606	5.91858	1.94925	68.3209	97.8784	64.6151	Up
Axdnd1	561.325	1.78886	0.3507	5.10083	3.38E-07	1.61E-05	213.468	373.857	168.611	757.648	1255.93	598.435	Up
Soat1	21203.4	0.47892	0.21411	2.23678	0.0253	0.1197	17245.9	23193.9	12708.2	24695.5	27235.9	22141.1	Up
Abl2	2118.19	0.27536	0.10219	2.69469	0.00705	0.04777	1827.21	1932.42	1990.19	2300.48	2476.84	2182	Up
Fam20b	4758.62	0.38533	0.07789	4.94726	7.53E-07	3.15E-05	4020.65	4116.37	4243.53	5419.78	5469.85	5281.54	Up
Pappa2	248.654	0.55607	0.2043	2.72176	0.00649	0.04514	186.05	222.933	194.925	262.067	358.544	267.407	Up
Tnn	601.842	-1.0416	0.23627	-4.4087	1.04E-05	0.00029	631.592	703.324	1095.48	442.556	353.392	384.708	Down
Ankrd45	1284.61	-0.9507	0.34105	-2.7876	0.00531	0.03904	1904.57	1040.68	2134.43	1005.44	486.301	1136.23	Down
Gm37357	4.74809	-3.0708	1.42674	-2.1523	0.03137	NA	17.6258	3.94572	3.89851	0	1.0303	1.98816	Down
Dnm3	488.693	-0.5789	0.19123	-3.0272	0.00247	0.02218	489.606	544.509	722.199	427.261	368.847	379.738	Down
Vamp4	3218.07	0.39918	0.12246	3.25962	0.00112	0.012	2727.11	3071.74	2528.18	3475.19	3985.19	3521.03	Up

Fmo6	234.721	-2.5195	1.12071	-2.2481	0.02457	0.11723	613.966	38.4708	546.766	56.0843	26.7878	126.248	Down
Kifap3	2840.99	-0.3848	0.13511	-2.8479	0.0044	0.03396	3491.87	2713.67	3447.26	2332.09	2549.99	2511.04	Down
Ccdc181	5137.09	-0.6399	0.24461	-2.6162	0.00889	0.05679	7324.51	4312.67	7137.2	4348.06	2968.29	4731.81	Down
Blzf1	2194.4	-0.2617	0.1267	-2.0659	0.03884	0.16198	2429.43	2120.82	2628.57	2002.72	1854.54	2130.31	Down
Nme7	1373.81	-0.4028	0.16475	-2.4446	0.0145	0.08152	1743.98	1295.18	1653.94	1199.18	1034.42	1316.16	Down
Atp1b1	39982.9	0.72678	0.12209	5.95258	2.64E-09	2.17E-07	32442.3	25839.5	32076.9	51767.9	49711.9	48058.7	Up
Dpt	594.471	0.45474	0.16172	2.81192	0.00492	0.03694	575.777	489.269	439.557	648.539	766.542	647.145	Up
Sft2d2	7507.06	0.22538	0.08378	2.69017	0.00714	0.04824	6789.86	6875.41	7100.16	8348.41	8268.15	7660.37	Up
Mpzl1	2515.83	0.40536	0.10715	3.78322	0.00015	0.00262	2134.68	2262.87	2096.42	2907.21	3074.41	2619.4	Up
Dusp27	9.80178	-3.1293	1.55191	-2.0164	0.04375	0.17475	35.2517	0	17.5433	2.03943	0	3.97631	Down
Gpa33	17.8888	1.40974	0.68313	2.06364	0.03905	0.16245	9.79213	4.93215	14.6194	38.7492	13.3939	25.846	Up
Rgs4	667.125	0.93565	0.18775	4.98357	6.24E-07	2.70E-05	406.373	579.034	388.876	932.019	835.572	860.872	Up
Ccdc190	23.2226	-1.5492	0.53675	-2.8863	0.0039	0.03114	46.023	27.62	30.2134	7.138	14.4242	13.9171	Down
Hsd17b7	1467	0.61757	0.15253	4.04892	5.15E-05	0.00107	1172.12	1209.36	1091.58	1650.92	2118.29	1559.71	Up
Uap1	4380.75	0.28394	0.08358	3.39738	0.00068	0.00815	4076.46	3908.23	3868.3	4949.7	4871.25	4610.54	Up
Uhmk1	8172.45	0.24422	0.09278	2.63209	0.00849	0.05495	7427.33	7899.33	7120.63	8368.8	9053.23	9165.4	Up
Sh2d1b1	717.347	1.30128	0.26661	4.88092	1.06E-06	4.17E-05	366.226	565.224	310.906	894.29	1310.54	856.896	Up
Olfml2b	540.569	-0.3306	0.1416	-2.3347	0.01956	0.10058	663.906	536.618	606.218	472.128	460.543	503.998	Down
Fcrlb	53.1814	1.01071	0.42508	2.37767	0.01742	0.09335	52.8775	26.6336	26.3149	92.7941	61.8179	58.6506	Up
Fcgr2b	3053.17	0.94307	0.17702	5.32758	9.95E-08	5.52E-06	2266.88	2315.15	1686.11	3622.03	3677.14	4751.7	Up
Fcgr4	918.891	1.23154	0.28631	4.30134	1.70E-05	0.00043	460.23	759.551	426.887	1146.16	1745.33	975.191	Up
Fcgr3	3612.47	1.03695	0.20455	5.0695	3.99E-07	1.84E-05	2518.54	2928.71	1654.92	4828.35	5071.13	4673.16	Up
Cfap126	3235.71	-1.0283	0.26277	-3.9133	9.10E-05	0.00172	4816.75	3039.19	5171.37	2374.92	1440.36	2571.68	Down
Pcp4l1	9452.37	-0.9983	0.21809	-4.5774	4.71E-06	0.00015	14430.7	9463.81	13900.1	6671.99	4767.19	7480.44	Down
Tomm40l	343.213	0.31565	0.15008	2.10326	0.03544	0.15195	323.14	304.807	289.464	353.841	416.241	371.785	Up
Fcer1g	2911.52	1.05343	0.17391	6.05733	1.38E-09	1.19E-07	1745.94	2252.02	1682.21	3715.84	4643.56	3429.57	Up
Usp21	2184.8	-0.2708	0.1337	-2.0253	0.04284	0.17231	2631.14	2086.3	2450.21	2161.8	1861.75	1917.58	Down
Klhdc9	158.504	-1.015	0.32454	-3.1273	0.00176	0.01701	254.595	129.222	252.428	104.011	85.5148	125.254	Down
Arhgap30	2119.02	0.77767	0.17764	4.37775	1.20E-05	0.00032	1336.63	1917.62	1429.78	2469.75	3067.2	2493.15	Up
Tstd1	246.702	-0.5214	0.18017	-2.8937	0.00381	0.03067	327.057	287.051	258.276	221.278	193.696	192.851	Down
F11r	14098.4	0.20853	0.07872	2.649	0.00807	0.05284	12659.3	13154	13430.4	15311	15483.3	14552.3	Up
Cd244a	497.945	0.33441	0.13555	2.46705	0.01362	0.0777	402.456	484.337	434.684	546.567	573.876	545.749	Up
Slamf7	369.782	0.73531	0.1751	4.19948	2.68E-05	0.00062	266.346	246.607	319.678	462.951	413.15	509.962	Up
Cd48	1548.63	0.63759	0.19982	3.19077	0.00142	0.01433	1111.41	1546.72	977.551	1875.26	2131.69	1649.18	Up
Cd84	2721	1.31072	0.17918	7.31528	2.57E-13	4.72E-11	1484.49	1829.83	1376.17	3574.1	4706.4	3355.02	Up
Gm10521	35.0383	2.12358	0.49286	4.30867	1.64E-05	0.00042	15.6674	14.7964	8.77165	44.8675	81.3936	44.7335	Up
Slamf6	400.208	0.52854	0.23843	2.21673	0.02664	0.12442	328.036	419.233	235.86	450.714	541.937	425.466	Up
Ncstn	7415.82	0.36305	0.07733	4.69466	2.67E-06	9.19E-05	6406.99	6487.75	6568.01	8610.47	8384.57	8037.12	Up
Igsf8	2458.57	0.35916	0.13173	2.7264	0.0064	0.04472	1988.78	2027.11	2446.31	2696.13	3009.5	2583.61	Up
Kcnj10	103.161	0.65604	0.30279	2.16667	0.03026	0.13592	82.2539	100.616	57.503	122.366	106.121	150.106	Up
Slamf9	701.689	0.78477	0.18643	4.2094	2.56E-05	0.0006	423.02	615.532	507.781	783.141	872.663	1008	Up
Cfap45	901.486	-0.7567	0.2694	-2.8087	0.00497	0.03721	1274.94	752.646	1370.33	744.392	485.271	781.346	Down
4933439K	272.267	-0.7747	0.37714	-2.0543	0.03995	0.16492	474.918	215.042	341.12	191.706	120.545	290.271	Down
Aim2	815.243	1.22284	0.21584	5.66538	1.47E-08	1.04E-06	449.459	579.034	438.582	1119.65	1426.96	877.771	Up
Ifi207	954.981	0.46731	0.17346	2.69407	0.00706	0.04781	981.171	722.066	701.732	1213.46	1018.97	1092.49	Up
Ifi203-ps	367.439	0.72028	0.2262	3.18429	0.00145	0.01459	244.803	353.142	234.885	431.339	397.695	542.767	Up
Ifi202b	5.72835	-2.9089	1.40597	-2.0689	0.03855	NA	22.5219	1.97286	5.84776	2.03943	0	1.98816	Down
Fmn2	254.972	-0.5553	0.2633	-2.1089	0.03495	0.15039	220.323	408.382	281.667	225.357	182.363	211.739	Down
Rgs7	19.9611	-1.4829	0.63904	-2.3206	0.02031	0.10332	43.0854	21.7015	23.3911	16.3154	10.303	4.97039	Down
Kmo	321.874	0.35956	0.16998	2.11529	0.03441	0.14878	255.575	309.739	280.693	327.328	351.332	406.578	Up
1700016C	68.376	-1.1352	0.38025	-2.9854	0.00283	0.0246	89.1084	72.9958	119.879	38.7492	29.8787	59.6447	Down
Catspere2	1831.46	0.48464	0.12835	3.77578	0.00016	0.00268	1320.96	1634.51	1624.7	2077.16	2069.87	2261.53	Up
Gm38300	133.359	-0.6976	0.23169	-3.011	0.0026	0.02307	141.986	179.53	173.484	115.228	86.5451	103.384	Down
Efcab2	569.075	-0.6626	0.23095	-2.8691	0.00412	0.03229	777.495	517.876	797.245	447.655	350.301	523.879	Down
Kif26b	996.815	-0.8864	0.18092	-4.8991	9.63E-07	3.85E-05	1422.8	1018	1440.5	727.057	614.058	758.482	Down
Smyd3	1223.4	-0.8811	0.19526	-4.5123	6.41E-06	0.00019	1546.18	1294.2	1917.09	869.817	716.057	997.061	Down
Sccpdh	2194.77	-0.5591	0.15478	-3.6121	0.0003	0.00441	2799.57	2391.11	2653.91	1818.15	1464.05	2041.84	Down
Parp1	3201.12	0.25454	0.12398	2.05303	0.04007	0.16523	2696.75	3209.84	2851.76	3186.61	3817.26	3444.48	Up
Lin9	316.259	0.45242	0.20854	2.16949	0.03005	0.13517	216.406	245.621	339.17	356.9	363.695	375.762	Up
Pycr2	2262.28	-0.3943	0.10801	-3.6504	0.00026	0.00394	2687.94	2454.24	2566.19	2114.89	1944.17	1806.24	Down
Ccdc121	1413.33	-0.9428	0.31923	-2.9535	0.00314	0.02659	2332.48	1158.07	2087.65	1124.75	582.119	1194.88	Down
Enah	3734.81	-0.5495	0.23617	-2.3266	0.01999	0.10219	4719.81	3067.8	5525.16	3382.39	2368.66	3345.07	Down
Taf1a	935.116	-0.3559	0.16171	-2.2005	0.02777	0.12807	1190.72	891.732	1067.22	833.107	724.3	903.617	Down
Gm37986	26.2439	-1.2021	0.44139	-2.7235	0.00646	0.04498	40.1477	33.5386	36.0612	19.3746	14.4242	13.9171	Down
Hhipl2	23.4102	-1.0167	0.51705	-1.9664	0.04926	0.18906	31.3348	28.6065	34.112	11.2169	11.3333	23.8579	Down
1700112H	2.045	4.50264	2.13944	2.10459	0.03533	NA	0	0	0	2.03943	8.24239	1.98816	Up
Gm37800	36.5121	1.07358	0.40826	2.62965	0.00855	0.05522	20.5635	21.7015	28.2642	52.0055	59.7573	36.7809	Up
Mtarc1	77.7603	-1.7089	0.42295	-4.0405	5.33E-05	0.00111	115.547	86.8058	154.966	48.9463	18.5454	41.7513	Down
Bpnt1	2757.51	-0.2351	0.11048	-2.1278	0.03336	0.14575	3164.82	2835.99	2944.35	2504.42	2341.87	2753.6	Down
Lyplal1	478.402	-0.5568	0.24221	-2.2989	0.02151	0.10714	533.671	472.5	702.706	414.004	282.302	465.229	Down
Tgfb2	1880.35	0.53875	0.13338	4.03914	5.36E-05	0.00111	1312.15	1659.17	1628.6	2131.2	2180.11	2370.88	Up
Spata17	1158.67	-0.8797	0.2627	-3.3486	0.00081	0.00932	1734.19	1069.29	1700.72	893.27	559.452	995.073	Down
Esrrg	381.573	-1.0499	0.35691	-2.9417	0.00326	0.02732	566.964	356.101	620.838	243.712	135.999	365.821	Down
A230020J	71.7473	-1.0866	0.35407	-3.0688	0.00215	0.01993	109.672	63.1315	119.879	48.9463	39.1513	49.7039	Down
Nek2	1941.82	-0.896	0.30339	-2.9532	0.00314	0.02659	2658.56	1716.39	3203.6	1367.44	849.996	1854.95	Down
1700034H	103.261	-0.8216	0.388	-2.1177	0.0342	0.14823	126.318	121.331	148.143	62.2026	43.2725	118.295	Down
Rd3	386.035	-1.776	0.41399	-4.29	1.79E-05	0.00045	723.638	235.757	833.306	216.18	174.12	133.207	Down
Gm10516	286.622	-0.4416	0.1993	-2.2156	0.02672	0.12471	374.059	270.282	345.993	257.988	260.666	210.745	Down
Cr2	224.834	0.69488	0.30026	2.31429	0.02065	0.10452	122.402	239.702	153.016	267.165	342.059	224.662	Up
Acbd7	33.7856	-1.0606	0.53584	-1.9792	0.04779	0.18562	48.9606	31.5657	56.5284	28.552	9.27269	27.8342	Down
Olah	61.7805	-1.0767	0.44515	-2.4187	0.01558	0.08591	69.5241	59.1858	122.803	39.7689	24.7272	54.6743	Down
Meig1	755.053	-1.2366	0.30673	-4.0315	5.54E-05	0.00114	1198.56	625.396	1356.68	488.443	321.453	539.785	Down
Frmd4a	3777.47	0.46176	0.1349	3.42306	0.00062	0.00761	2744.73	3345.97	3443.36	4207.34	4822.83	4100.57	Up

Phyh	3860.32	-0.3062	0.1045	-2.9297	0.00339	0.02814	4131.3	4057.18	4616.81	3411.97	3280.47	3664.17	Down
Gm13391	54.5833	1.2692	0.4198	3.02333	0.0025	0.02243	50.9191	22.6879	22.4164	72.3998	85.5148	73.5618	Up
Gm38386	3.89948	3.45058	1.60452	2.15053	0.03151	NA	1.95843	0	0	5.09857	12.3636	3.97631	Up
Gata3	280.125	0.68181	0.26399	2.58269	0.0098	0.06083	144.923	288.037	212.469	374.235	339.998	321.087	Up
Itih5	354.584	-0.8307	0.26749	-3.1057	0.0019	0.01799	550.318	294.942	516.552	294.698	220.484	250.508	Down
E030013I1	138.63	-0.787	0.27254	-2.8875	0.00388	0.03106	158.632	198.272	169.585	118.287	116.424	70.5796	Down
C1ql3	2.6924	3.84952	1.79709	2.14208	0.03219	NA	0	0	0.97463	2.03943	6.18179	6.95855	Up
Vim	44312	0.4945	0.13591	3.63831	0.00027	0.00407	37024	39358.5	33991.1	52542.8	58660	44295.1	Up
Hacd1	910.257	-0.2396	0.11318	-2.1165	0.0343	0.14845	957.67	1007.14	992.171	766.826	878.845	858.884	Down
Stamos	46.5851	-1.1347	0.37654	-3.0134	0.00258	0.02298	64.628	54.2536	73.097	33.6506	33.9998	19.8816	Down
Mrc1	9410.05	1.22423	0.17362	7.05132	1.77E-12	2.79E-10	6127.91	6114.88	4680.16	12743.4	15681.1	11112.8	Up
Slc39a12	90.515	1.1638	0.24742	4.70371	2.55E-06	8.87E-05	63.6488	52.2808	51.6552	127.464	127.757	120.284	Up
Gm13266	12.5801	1.81946	0.74046	2.45719	0.014	0.07925	5.87528	5.91858	4.87314	31.6112	10.303	16.8993	Up
Mllt10	823.513	0.20299	0.10343	1.96264	0.04969	0.19018	778.474	734.89	783.6	885.113	890.178	868.825	Up
Pip4k2a	2123.13	0.3742	0.12916	2.89715	0.00377	0.03042	1664.66	2066.57	1816.71	2268.87	2609.75	2312.23	Up
Armc3	838.133	-1.0502	0.29219	-3.5942	0.00033	0.00466	1293.54	759.551	1338.16	626.105	352.362	659.074	Down
Msrb2	1112.62	-0.4475	0.1307	-3.424	0.00062	0.0076	1343.48	1326.75	1181.25	1050.31	863.39	910.576	Down
Enkur	2640.56	-0.8338	0.27461	-3.0362	0.0024	0.02168	3957.98	2226.37	3964.78	2104.69	1298.18	2291.35	Down
Apbb1ip	1538.23	0.9632	0.22648	4.25299	2.11E-05	0.00051	956.691	1288.28	883.987	1833.45	2640.65	1626.31	Up
Il1f9	187.358	0.56554	0.27259	2.07466	0.03802	0.15939	142.965	117.385	192.976	215.16	180.302	275.36	Up
Il1rn	809.896	1.08455	0.23186	4.67762	2.90E-06	9.82E-05	429.874	704.311	422.988	985.045	1271.39	1045.77	Up
Cacna1b	121.025	0.60548	0.25631	2.36228	0.01816	0.09609	105.755	77.9279	104.285	173.352	124.666	140.165	Up
Pnpla7	2077.76	0.90423	0.16894	5.35232	8.68E-08	4.96E-06	1227.93	1500.36	1613.01	2606.39	3187.74	2331.11	Up
Entpd8	399.685	1.19719	0.39223	3.05222	0.00227	0.02085	324.119	137.114	267.048	605.711	295.696	768.423	Up
AL732309	542.278	-0.645	0.28229	-2.285	0.02231	0.10976	646.28	904.556	433.709	364.038	522.361	382.72	Down
Tor4a	2768.67	0.25282	0.1104	2.29011	0.02202	0.10883	2582.18	2686.05	2311.82	2876.62	3223.8	2931.54	Up
Tubb4b	26899.6	-0.3245	0.14521	-2.2348	0.02543	0.12023	33723.1	25852.3	30161.8	26080.2	20830.6	24749.6	Down
Cysrt1	63.7112	-0.9475	0.32026	-2.9584	0.00309	0.0263	84.2123	77.9279	89.6657	43.8477	31.9393	54.6743	Down
Rnf208	192.9	-1.1763	0.26489	-4.4408	8.96E-06	0.00026	230.115	267.322	305.058	121.346	83.4542	150.106	Down
Lrrc26	714.528	-0.7405	0.35429	-2.09	0.03662	0.15559	1199.54	403.45	1078.91	626.105	452.301	526.862	Down
Man1b1	4868.2	0.23086	0.08013	2.88098	0.00396	0.03149	4615.03	4339.3	4484.26	5114.89	5293.67	5362.06	Up
Dpp7	2000.29	0.68429	0.12192	5.61267	1.99E-08	1.35E-06	1646.06	1607.88	1349.86	2505.44	2563.38	2329.13	Up
Fut7	44.9033	1.08529	0.37099	2.92543	0.00344	0.02843	28.3972	31.5657	26.3149	62.2026	75.2118	45.7276	Up
Tmem250	3581.3	-0.3102	0.08238	-3.7652	0.00017	0.00277	3941.33	4077.9	3875.12	3289.6	3207.32	3096.55	Down
Gm13553	2.17873	4.59654	2.06064	2.23064	0.0257	NA	0	0	0	3.05914	2.0606	7.95263	Up
Ccdc187	3664.94	-0.7488	0.29456	-2.5422	0.01102	0.06633	5085.05	3047.08	5653.81	2907.21	1726.78	3569.74	Down
Dnlz	1197.31	0.43826	0.1135	3.86122	0.00011	0.00205	1074.2	1047.59	928.82	1406.19	1309.51	1417.56	Up
Card9	128.099	0.66557	0.25387	2.62166	0.00875	0.0561	84.2123	123.304	89.6657	154.997	178.242	138.177	Up
Inpp5e	1699.41	-0.2944	0.12899	-2.2822	0.02248	0.11025	2027.95	1637.47	1951.2	1508.16	1441.39	1630.29	Down
Surf4	12694	0.23145	0.09715	2.38237	0.0172	0.09251	11694.7	12344.2	10994.8	14305.6	14010	12814.7	Up
Dbhos	3.10708	-5.0572	1.79731	-2.8137	0.0049	NA	3.91685	8.87787	5.84776	0	0	0	Down
Brd3os	411.001	-0.7819	0.18419	-4.2449	2.19E-05	0.00053	552.276	414.3	592.573	317.131	289.514	300.212	Down
Olfm1	1822.87	0.31686	0.10711	2.95824	0.00309	0.0263	1710.68	1626.62	1533.09	1991.5	2166.72	1908.63	Up
Ppp1r26	716.619	-0.3492	0.14967	-2.3333	0.01963	0.10086	872.479	673.731	862.545	655.677	611.997	623.287	Down
1700007K	3715.05	-1.1547	0.29929	-3.8581	0.00011	0.00207	6177.85	3618.22	5585.59	2640.04	1330.12	2938.5	Down
Mrps2	1213.09	-0.2742	0.10838	-2.5299	0.01141	0.068	1392.44	1235.01	1356.68	1062.54	1079.75	1152.14	Down
Gbgt1	92.4576	1.20883	0.28719	4.20913	2.56E-05	0.0006	72.4617	43.4029	51.6552	133.583	117.454	136.189	Up
Gm10134	412.731	0.8084	0.16733	4.83128	1.36E-06	5.20E-05	321.182	321.576	257.302	546.567	562.543	467.217	Up
Ralgds	4629.75	0.23194	0.1023	2.26718	0.02338	0.11338	4543.55	4221.92	4009.62	4971.11	5318.4	4713.92	Up
Spaca9	1410.74	-0.778	0.26155	-2.9745	0.00293	0.02526	2159.16	1167.93	2019.43	1146.16	776.845	1194.88	Down
Ak8	1173.46	-1.0496	0.28591	-3.6711	0.00024	0.00371	1771.4	933.162	2042.82	763.766	614.058	915.546	Down
Barhl1	201.59	-0.6815	0.29764	-2.2895	0.02205	0.10895	259.491	179.53	306.033	176.411	107.151	180.922	Down
Cfap77	1123.27	-0.9617	0.31035	-3.0988	0.00194	0.01836	1861.48	914.42	1677.33	856.561	490.422	939.404	Down
Coq4	974.282	-0.5199	0.14748	-3.5255	0.00042	0.00564	1236.75	1031.81	1175.4	889.191	694.421	818.127	Down
Cercam	167.068	0.54223	0.27106	2.00044	0.04545	0.17923	114.568	181.503	112.082	165.194	215.332	213.727	Up
Wdr34	1792.54	-0.8556	0.2253	-3.7974	0.00015	0.00251	2568.48	1716.39	2642.21	1424.54	959.208	1444.4	Down
Tbc1d13	2272.68	0.28376	0.12968	2.18809	0.02866	0.13095	1944.72	2227.36	1977.52	2390.21	2817.87	2278.43	Up
Endog	2846.11	-0.9601	0.22389	-4.2882	1.80E-05	0.00045	3950.14	2736.36	4592.44	2311.69	1548.54	1937.46	Down
Kyat1	1248.09	-0.5876	0.1108	-5.3028	1.14E-07	6.22E-06	1517.78	1399.74	1578.9	1023.79	934.481	1033.84	Down
Sh3glb2	3605.43	-0.2673	0.12724	-2.1005	0.03569	0.15275	3740.59	3739.55	4335.14	3670.97	3052.77	3093.57	Down
Crat	4149.4	0.22055	0.11046	1.99654	0.04587	0.18033	3659.32	4261.38	3577.86	4562.2	4560.1	4275.53	Up
Ier5l	315.137	-0.4747	0.17874	-2.6561	0.00791	0.05199	381.893	346.237	371.333	235.554	314.241	241.561	Down
Gm14487	22.3781	-1.5892	0.69962	-2.2715	0.02312	0.11243	50.9191	11.8372	38.0105	6.11829	15.4545	11.9289	Down
Gm14488	32.0009	-1.1764	0.53171	-2.2125	0.02693	0.12526	62.6696	20.715	49.706	18.3549	22.6666	17.8934	Down
Tor1b	3349.95	0.22593	0.07821	2.88867	0.00387	0.03099	3132.5	3072.73	3059.36	3525.15	3633.86	3676.1	Up
BC005624	3693.17	-0.341	0.12845	-2.6551	0.00793	0.05207	4524.94	3639.93	4218.19	3246.77	2999.2	3529.97	Down
Ncs1	823.364	-0.9146	0.30489	-2.9996	0.0027	0.02374	1175.06	674.718	1378.12	576.139	393.574	742.577	Down
Hmcn2	247.916	-1.1996	0.3501	-3.4263	0.00061	0.00755	372.101	206.164	458.075	173.352	97.8784	179.928	Down
Ass1	1057.17	-0.4018	0.20096	-1.9995	0.04556	0.17944	1058.53	999.253	1552.58	850.442	860.299	1021.91	Down
Lamc3	649.322	0.43906	0.18014	2.43731	0.0148	0.08265	447.5	545.496	660.797	817.811	701.633	722.695	Up
Nup214	2543.32	0.39107	0.13522	2.89218	0.00383	0.03077	2128.81	2508.49	1964.85	2702.24	3139.32	2816.22	Up
Plpp7	139.1	-0.8656	0.23684	-3.6548	0.00026	0.00389	198.78	164.734	175.433	81.5772	94.7875	119.289	Down
Prrc2b	4486.81	0.31	0.12444	2.4911	0.01273	0.07372	4212.57	4123.28	3684.09	4423.52	4971.19	5506.2	Up
Uck1	2663.16	-0.4519	0.12338	-3.6626	0.00025	0.00379	3186.36	2910.95	3133.43	2451.39	1963.75	2333.1	Down
Swi5	4206.73	-0.3032	0.13165	-2.3027	0.0213	0.10668	4959.71	4024.63	4956.95	4032.97	3432.95	3833.17	Down
Lcn2	60861.3	1.42916	0.28331	5.04456	4.55E-07	2.06E-05	26509.2	29688.6	42685.8	92226.1	54227.7	119830	Up
Ptges2	1349.16	-0.2782	0.1387	-2.0054	0.04492	0.17779	1605.91	1492.47	1338.16	1349.08	1077.69	1231.66	Down
St6galnac	1548.33	-0.3512	0.16255	-2.1608	0.03071	0.13723	1650.95	2069.53	1487.28	1252.21	1324.96	1505.03	Down
Ak1	1628.06	-0.9053	0.13917	-6.5056	7.74E-11	8.72E-09	2183.64	1848.57	2336.18	1126.78	1037.51	1235.64	Down
Fpgs	1108.43	-0.2099	0.10561	-1.9869	0.04693	0.18321	1232.83	1181.74	1152.01	1023.79	1088	972.209	Down
Sh2d3c	3961.33	-0.3035	0.10038	-3.0237	0.0025	0.02242	4321.27	4199.23	4609.01	3765.81	3296.96	3575.7	Down
Ttc16	1925.53	-0.9908	0.29121	-3.4023	0.00067	0.00803	2991.49	1588.15	3106.14	1520.39	875.754	1471.24	Down
Cfap157	1043.86	-0.723	0.22333	-3.2371	0.00121	0.01279	1513.86	939.081	1447.32	854.521	632.603	875.783	Down

Lmx1b	75.4899	-1.0896	0.5219	-2.0877	0.03682	0.1562	151.778	36.4979	119.879	57.104	33.9998	53.6802	Down
C130021I2	216.545	-1.6393	0.43908	-3.7335	0.00019	0.00305	521.92	132.182	329.424	129.504	94.7875	91.4552	Down
C230014C	162.626	1.05906	0.20888	5.07004	3.98E-07	1.84E-05	103.797	93.7108	118.905	194.766	235.938	228.638	Up
AI182371	527.733	-0.7221	0.27874	-2.5907	0.00958	0.05987	693.283	852.275	425.912	378.314	324.544	492.069	Down
Ggta1	3567.12	0.45273	0.15147	2.98895	0.0028	0.02436	3096.27	3358.79	2580.81	3901.43	4717.74	3747.68	Up
Ttll11	336.15	-0.5354	0.14428	-3.711	0.00021	0.00327	384.831	384.708	423.963	260.027	275.09	288.283	Down
Morn5	322.286	-1.0659	0.29724	-3.5862	0.00034	0.00476	503.315	269.295	536.045	239.633	161.757	223.668	Down
Ptgs1	8340.23	0.65076	0.14269	4.56053	5.10E-06	0.00016	5684.33	7739.53	6047.56	9882.06	10636.8	10051.1	Up
Strbp	3600.98	-0.5052	0.19387	-2.6059	0.00916	0.05801	4582.72	3456.45	4636.3	3134.6	2300.66	3495.18	Down
Nek6	3998.24	0.5799	0.17759	3.26534	0.00109	0.01183	3078.64	3868.78	2668.53	4717.2	5507.98	4148.29	Up
Nr6a1	1186.22	0.27848	0.10031	2.77628	0.0055	0.04006	1067.34	1037.72	1111.08	1272.6	1363.08	1265.46	Up
Olfml2a	1331.31	-0.613	0.22074	-2.7768	0.00549	0.04002	1812.52	1386.92	1630.55	1064.58	756.239	1337.04	Down
Wdr38	180.424	-0.6711	0.3273	-2.0504	0.04032	0.16567	248.72	173.612	242.682	165.194	80.3633	171.976	Down
Kynu	615.224	1.63866	0.21488	7.62586	2.42E-14	5.59E-12	266.346	357.088	273.87	836.166	1174.54	783.334	Up
Rnd3	828.555	0.5295	0.14455	3.663	0.00025	0.00379	678.594	752.646	603.294	1045.21	1013.81	877.771	Up
Nmi	1700.62	0.28912	0.12607	2.29335	0.02183	0.10825	1455.11	1698.63	1438.55	1906.87	1997.75	1706.83	Up
Arl5a	3930.19	0.22687	0.09019	2.51537	0.01189	0.07008	3447.81	3801.7	3615.87	4259.35	4377.74	4078.7	Up
Rprm	756.344	1.27349	0.23628	5.38978	7.05E-08	4.17E-06	412.249	525.767	389.851	1028.89	1393.99	787.31	Up
Galnt13	570.937	1.98479	0.20715	9.5814	9.57E-22	7.88E-19	236.969	219.974	233.911	960.571	1096.24	677.962	Up
Nr4a2	1092.48	0.53357	0.18845	2.83142	0.00463	0.03541	843.102	955.85	879.114	1329.71	1562.96	984.138	Up
Gpd2	2264.19	0.25808	0.12889	2.00233	0.04525	0.17868	2272.75	2104.05	1809.88	2276	2572.66	2549.81	Up
Cytip	6906.11	0.25402	0.11203	2.26752	0.02336	0.11334	6943.6	5997.49	5957.9	8066.96	7350.15	7120.58	Up
Upp2	906.996	-0.4055	0.16916	-2.397	0.01653	0.08976	1133.93	829.587	1137.39	780.082	715.027	845.961	Down
Ccdc148	360.74	-0.7023	0.28778	-2.4406	0.01466	0.08216	533.671	325.522	481.466	280.422	186.484	356.874	Down
Cd302	4507.59	0.55085	0.13029	4.22796	2.36E-05	0.00056	3320.51	4146.95	3504.76	5163.84	5046.4	5863.08	Up
Ly75	2794.68	1.29536	0.15693	8.25452	1.53E-16	5.34E-14	1563.8	1621.69	1668.56	3430.32	4841.37	3642.3	Up
Gm13580	48.2106	-1.2461	0.57312	-2.1742	0.02969	0.13412	85.1915	33.5386	84.7926	33.6506	11.3333	40.7572	Down
Tank	2954.76	0.23882	0.09756	2.44789	0.01437	0.08101	2772.15	2834.01	2526.23	3160.1	3092.96	3343.09	Up
Fign	560.947	-1.113	0.24991	-4.4534	8.45E-06	0.00024	662.927	600.736	1037.98	359.959	290.544	413.537	Down
Slc38a11	444.626	-2.0765	0.78036	-2.661	0.00779	0.05139	1211.29	231.811	713.427	182.529	75.2118	253.49	Down
Scn3a	598.955	-0.5374	0.19686	-2.7297	0.00634	0.0444	766.724	762.51	598.421	395.649	588.3	482.128	Down
Galnt3	3052.83	0.70912	0.18213	3.89353	9.88E-05	0.00185	2793.69	1743.02	2415.13	3750.51	4015.07	3599.56	Up
Stk39	2206.97	0.3236	0.13461	2.40401	0.01622	0.08846	2114.12	1896.9	1870.31	2274.98	2821.99	2263.52	Up
Cers6	1477.22	0.85807	0.09529	9.0047	2.16E-19	1.05E-16	1007.61	1098.88	1044.8	1865.06	1948.29	1898.69	Up
Dhrs9	224.938	-1.4562	0.47095	-3.092	0.00199	0.01871	623.759	151.91	213.443	114.208	134.969	111.337	Down
Lrp2	13561.7	0.57212	0.24114	2.37252	0.01767	0.09426	11520.4	14300.3	6901.34	14644.1	17201.9	16801.9	Up
Bbs5	1049.74	-0.4604	0.17173	-2.6811	0.00734	0.04923	1335.65	991.362	1320.62	852.482	793.33	1005.01	Down
Klhl41	94.9657	-0.9321	0.42833	-2.1762	0.02954	0.13362	160.591	72.0094	141.321	43.8477	54.6058	97.4197	Down
Ccdc173	666.766	-0.9523	0.30151	-3.1584	0.00159	0.01567	1027.19	596.79	1013.61	463.97	293.635	605.394	Down
Mettl5os	10.3823	-1.5013	0.72842	-2.0611	0.03929	0.16307	19.5843	9.8643	16.5687	6.11829	6.18179	3.97631	Down
Myo3b	162.135	-0.6425	0.23632	-2.7187	0.00655	0.04552	161.57	219.974	211.494	132.563	104.06	143.147	Down
Rpl9-ps7	193.307	-0.725	0.32264	-2.2473	0.02462	0.11743	329.995	240.689	152.042	136.642	120.545	179.928	Down
Sp5	434.893	0.87508	0.3357	2.60671	0.00914	0.05794	336.849	410.355	173.484	637.322	667.633	383.714	Up
Cybrd1	1053.9	-0.8507	0.1694	-5.0222	5.11E-07	2.26E-05	1332.71	1583.22	1152.01	669.953	733.572	851.925	Down
Gm13647	5.07598	-3.7998	1.44334	-2.6327	0.00847	NA	7.8337	7.89144	12.6702	0	2.0606	0	Down
Itga6	6279.44	-0.222	0.10973	-2.0228	0.0431	0.17303	7311.78	6411.79	6561.19	5376.96	5754.22	6260.71	Down
Cir1	659.775	0.31564	0.12301	2.56598	0.01029	0.06325	585.569	554.373	623.761	744.392	684.118	766.435	Up
Scrn3	2033.33	-0.3883	0.09698	-4.0034	6.24E-05	0.00125	2395.15	2148.44	2372.24	1802.86	1724.72	1756.54	Down
Gpr155	2764.26	-0.9539	0.27518	-3.4665	0.00053	0.00673	3335.2	2311.2	5292.23	2141.4	1622.72	1882.78	Down
Gm13657	155.948	-0.6737	0.21071	-3.1973	0.00139	0.01415	188.009	188.408	198.824	108.09	109.212	143.147	Down
Ttc30b	2075.12	-0.459	0.23107	-1.9865	0.04698	0.18333	2241.42	1904.8	3061.3	1923.18	1295.09	2024.94	Down
Pde11a	22.4036	-1.5919	0.48769	-3.2641	0.0011	0.01186	33.2932	32.5522	35.0866	14.276	9.27269	9.94079	Down
Osbpl6	6310.67	-0.4314	0.18888	-2.2839	0.02238	0.10995	7226.59	5857.42	8657.61	6235.56	4404.53	5482.34	Down
Ttn	640.806	-2.645	1.25511	-2.1074	0.03509	0.15086	1828.19	115.412	1371.3	114.208	33.9998	381.726	Down
Zfp385b	507.17	0.54034	0.21343	2.53171	0.01135	0.06776	376.997	518.862	344.043	500.68	684.118	618.317	Up
Itga4	879.71	0.36334	0.17594	2.06516	0.03891	0.16209	942.982	744.754	620.838	964.65	989.087	1015.95	Up
Ppp1r1c	3.74864	-5.3284	1.97526	-2.6976	0.00699	NA	0.97921	5.91858	15.594	0	0	0	Down
Nup35	1641.4	-0.2158	0.10764	-2.0048	0.04499	0.17799	1920.24	1651.28	1720.22	1567.3	1503.21	1486.15	Down
Gm4735	1033.25	-0.8119	0.12671	-6.4076	1.48E-10	1.54E-08	1348.38	1289.26	1311.85	688.308	847.936	713.748	Down
Itgav	3964.92	0.40803	0.08925	4.5719	4.83E-06	0.00015	3453.68	3536.35	3233.81	4370.5	4693.01	4502.18	Up
Gm13711	12.3588	6.11812	1.77112	3.45438	0.00055	0.00694	0.97921	0	0	62.2026	1.0303	9.94079	Up
Calcrl	39176.3	0.51786	0.16399	3.1579	0.00159	0.01569	26237	38719.3	31702.7	46249.2	50213.7	41936.2	Up
Tnks1bp1	8309.85	0.22265	0.10849	2.0522	0.04015	0.16534	7311.78	7134.85	8563.08	8758.33	8902.81	9188.27	Up
Agbl2	1952.71	-0.8608	0.29317	-2.9362	0.00332	0.02769	2772.15	1811.08	2972.61	1494.9	831.451	1834.07	Down
C1qtnf4	83.6047	-0.9969	0.26043	-3.8279	0.00013	0.0023	124.36	102.589	107.209	62.2026	53.5755	51.6921	Down
Slc39a13	318.064	0.32901	0.15773	2.08588	0.03699	0.15664	310.41	281.132	254.378	339.565	345.15	377.75	Up
Spi1	2380.27	1.00988	0.2408	4.19386	2.74E-05	0.00064	1395.38	2075.45	1267.99	3060.16	4022.29	2460.34	Up
Madd	3065.07	0.23364	0.11214	2.08351	0.0372	0.15704	2728.09	3121.06	2603.23	3163.16	3343.32	3431.56	Up
Acp2	1440.6	0.53051	0.10232	5.18482	2.16E-07	1.08E-05	1182.89	1176.81	1176.38	1574.44	1757.69	1775.42	Up
1110051M	2403.42	-0.3838	0.13956	-2.7498	0.00596	0.04253	2647.79	2402.94	3112.96	2136.3	1896.78	2223.75	Down
F2	55.357	-1.7307	0.52072	-3.3238	0.00089	0.00998	100.859	34.525	119.879	25.4929	19.5757	31.8105	Down
Creb3l1	1239.16	0.5855	0.18324	3.1952	0.0014	0.0142	1202.47	968.674	802.118	1574.44	1595.93	1291.31	Up
Slc35c1	2132.58	0.2724	0.1154	2.36045	0.01825	0.09622	2103.35	1819.96	1872.26	2472.81	2150.23	2376.84	Up
Prdm11	636.094	0.27544	0.11389	2.41844	0.01559	0.08595	586.548	551.414	588.675	669.953	719.148	700.825	Up
Gm27027	27.4206	-1.0417	0.52271	-1.9928	0.04628	0.18146	41.1269	33.5386	36.0612	8.15772	25.7575	19.8816	Down
Hsd17b12	6633.98	0.30768	0.09548	3.2223	0.00127	0.01328	6200.38	5855.45	5731.78	7147.18	7879.72	6989.37	Up
Mir670hg	25.173	-1.3176	0.65309	-2.0174	0.04365	0.17454	38.1893	39.4572	30.2134	7.138	7.21209	28.8283	Down
B230118H	1290.73	-0.6397	0.18123	-3.53	0.00042	0.00559	1697.95	1268.55	1750.43	1129.84	844.845	1052.73	Down
Prr5l	1344	0.52851	0.25881	2.0421	0.04114	0.16782	806.871	1521.07	973.653	1535.69	1949.32	1277.39	Up
Pamr1	1028.77	0.45388	0.18468	2.45769	0.01398	0.07919	960.608	885.814	758.26	1307.27	1332.18	928.469	Up
Slc1a2	174.518	-0.7212	0.20117	-3.5848	0.00034	0.00477	193.884	231.811	226.114	142.76	114.363	138.177	Down
Cd44	15387.1	0.94966	0.15436	6.15208	7.65E-10	6.96E-08	10833	11239.4	9421.72	18773	24320.2	17735.4	Up
BC016548	5.39144	-4.8457	1.5047	-3.2204	0.00128	NA	7.8337	10.8507	12.6702	0	0	0.99408	Down

Elf5	1647.9	-0.3629	0.13286	-2.7318	0.0063	0.04429	1783.15	2082.35	1696.83	1571.38	1331.15	1422.53	Down
Cd59a	1712.84	-0.7655	0.22629	-3.3829	0.00072	0.00844	2068.1	2118.85	2283.55	1128.82	1744.3	933.44	Down
Dcdc5	423.55	-0.7417	0.31713	-2.3387	0.01935	0.09986	612.008	304.807	673.467	372.196	233.878	344.945	Down
Gm14015	20.5509	-1.6803	0.60578	-2.7738	0.00554	0.04026	35.2517	23.6743	35.0866	4.07886	10.303	14.9112	Down
Mpped2	218.675	-0.519	0.19751	-2.6276	0.0086	0.0554	238.928	299.875	233.911	188.647	191.636	159.053	Down
Mettl15	366.699	-0.5757	0.15196	-3.7885	0.00015	0.00258	423.999	403.45	489.263	306.934	282.302	294.247	Down
Ccdc34	120.328	-0.7625	0.3418	-2.2308	0.02569	0.12109	176.258	96.6701	181.281	115.228	69.03	83.5026	Down
Bbox1	973.274	-1.5213	0.39181	-3.8828	0.0001	0.00192	1767.48	837.479	1726.06	512.917	244.181	751.523	Down
Fibin	2148.44	-0.3419	0.15657	-2.1835	0.029	0.13197	2134.68	2823.16	2247.49	2019.04	2010.11	1656.13	Down
A26c3	84.0155	-1.5432	0.26351	-5.8562	4.73E-09	3.75E-07	118.485	125.277	131.575	47.9266	37.0907	43.7395	Down
Lpcat4	2192.61	0.48504	0.19878	2.44006	0.01468	0.08222	2036.76	1323.79	2121.76	2870.5	2388.23	2414.62	Up
Arhgap11	597.426	0.76099	0.15397	4.94251	7.71E-07	3.20E-05	383.851	496.174	450.278	683.209	774.784	796.257	Up
Dph6	1279.49	-0.2932	0.11667	-2.5128	0.01198	0.07048	1541.28	1288.28	1397.62	1128.82	1124.06	1196.87	Down
Gm13986	56.6568	-0.7908	0.36222	-2.1833	0.02902	0.13197	71.4825	59.1858	84.7926	48.9463	27.8181	47.7158	Down
Thbs1	9963.2	0.72598	0.29113	2.49371	0.01264	0.07332	8083.4	8751.6	5688.9	11576.8	18025.1	7653.41	Up
Fsip1	615.875	-0.8349	0.30312	-2.7544	0.00588	0.04209	850.936	525.767	991.196	478.246	287.453	561.654	Down
Gpr176	24.7845	1.43379	0.53664	2.67181	0.00754	0.05019	17.6258	10.8507	11.6955	21.414	46.3634	40.7572	Up
Bmf	2106.75	0.73023	0.11983	6.09397	1.10E-09	9.69E-08	1441.4	1785.44	1527.24	2558.46	2705.56	2622.38	Up
Bub1b	472.034	0.63655	0.22557	2.82197	0.00477	0.03605	335.87	488.283	284.591	521.074	581.088	621.299	Up
Plcb2	1304.24	1.21929	0.2438	5.00124	5.70E-07	2.51E-05	731.472	962.755	656.899	1618.29	2459.32	1396.68	Up
Ccdc9b	2233.54	0.3314	0.11248	2.94622	0.00322	0.02699	1860.5	1942.28	2131.51	2344.32	2690.11	2432.51	Up
Phgr1	319.963	0.56838	0.28226	2.01364	0.04405	0.17551	214.448	349.196	209.545	358.94	490.422	297.229	Up
Gchfr	85.214	-0.6286	0.27344	-2.2989	0.02151	0.10714	114.568	80.8872	115.006	65.2618	66.9694	68.5914	Down
Ppp1r14d	676.05	0.49176	0.16659	2.95199	0.00316	0.02666	534.65	495.188	655.924	886.132	713.997	770.411	Up
Rhov	173.149	-0.9794	0.32841	-2.9822	0.00286	0.02479	197.801	194.327	297.261	117.267	74.1815	158.058	Down
Vps18	1748.69	0.3567	0.14359	2.48412	0.01299	0.07489	1474.69	1662.13	1463.89	1916.04	2264.6	1710.81	Up
Chp1	13683.6	0.4525	0.13258	3.41307	0.00064	0.00783	11947.4	12392.5	10325.2	15200.9	17910.7	14324.7	Up
Itpka	1075.68	-0.9094	0.25007	-3.6365	0.00028	0.00409	1375.79	1043.64	1792.34	803.535	546.058	892.683	Down
Ltk	383.5	-0.7779	0.20616	-3.7734	0.00016	0.0027	517.024	392.599	543.842	290.619	232.847	324.07	Down
Mapkbp1	743.254	0.27971	0.13894	2.01311	0.0441	0.17562	744.202	581.007	689.061	792.318	813.936	839.002	Up
Pla2g4f	1631.88	0.77314	0.25583	3.02214	0.00251	0.02248	1042.86	1667.07	904.454	1790.62	2585.02	1801.27	Up
Tmem87a	4263.98	0.22828	0.08777	2.60074	0.0093	0.05851	3883.56	3844.12	4054.45	4423.52	4527.13	4851.1	Up
Snap23	5151.6	0.25395	0.10914	2.32692	0.01997	0.10219	4640.49	5119.57	4338.07	5235.22	5830.46	5745.77	Up
Epb42	51.6404	-1.3563	0.48712	-2.7842	0.00537	0.03931	43.0854	118.372	61.4015	30.5914	20.606	35.7868	Down
Tgm5	12.0037	-1.9125	0.9021	-2.1201	0.034	0.14768	22.5219	25.6472	8.77165	1.01971	4.12119	9.94079	Down
Tubgcp4	453.567	0.37944	0.15304	2.47934	0.01316	0.07565	410.29	380.762	391.8	444.596	522.361	571.595	Up
Map1a	608.474	-0.5615	0.17017	-3.2998	0.00097	0.01072	774.557	583.966	817.712	510.877	465.695	498.033	Down
Ppip5k1	2323.21	0.21538	0.09537	2.25845	0.02392	0.11517	2242.4	2062.62	2145.15	2553.37	2575.75	2359.94	Up
Ckmt1	8718.67	1.38451	0.2067	6.6982	2.11E-11	2.75E-09	4618.95	4267.29	5601.18	13003.4	15611.1	9210.14	Up
Strc	118.398	-0.9009	0.44485	-2.0253	0.04284	0.17231	176.258	70.0365	216.367	89.7349	54.6058	103.384	Down
Frmd5	185.854	-1.4374	0.43167	-3.3298	0.00087	0.00982	447.5	121.331	245.606	89.7349	90.6663	120.284	Down
Spg11	1273.81	0.30145	0.09883	3.05032	0.00229	0.02095	1135.89	1175.82	1112.05	1358.26	1382.66	1478.19	Up
Gatm	428.784	0.6577	0.18974	3.46636	0.00053	0.00673	302.577	388.653	307.008	486.404	467.755	620.305	Up
Dtwd1	505.353	-0.4075	0.16242	-2.5092	0.0121	0.07101	558.151	495.188	675.417	439.497	424.483	439.383	Down
Atp8b4	442.138	0.3793	0.17697	2.14335	0.03209	0.14163	364.267	343.278	445.405	456.832	572.846	470.199	Up
Slc27a2	930.275	0.81474	0.32468	2.50938	0.01209	0.07099	668.802	368.925	985.348	1176.75	933.45	1448.37	Up
Gabpb1	2895.16	0.48077	0.13944	3.44786	0.00057	0.00709	2330.53	2692.95	2228	3343.65	3795.62	2980.25	Up
Usp50	78.9124	1.22039	0.34468	3.54061	0.0004	0.00542	40.1477	55.2401	46.7821	150.918	97.8784	82.5085	Up
Sppl2a	8365.76	0.33496	0.09647	3.47219	0.00052	0.00662	7090.48	7706.97	7399.37	8679.81	9925.9	9392.05	Up
Blvra	1612.69	0.53565	0.14382	3.7244	0.0002	0.00314	1330.75	1335.63	1283.58	2000.68	2140.96	1584.56	Up
Tmem127	5462.67	0.20759	0.08491	2.44491	0.01449	0.0815	4998.88	5221.17	4991.07	5553.37	5969.55	6042.01	Up
Prom2	137.621	-1.1936	0.42422	-2.8136	0.0049	0.03679	186.05	137.114	251.454	87.6955	39.1513	124.26	Down
Nphp1	2901.04	-0.7092	0.24914	-2.8466	0.00442	0.03408	4045.13	2445.36	4309.8	2270.91	1663.93	2671.09	Down
Mtln	724.456	-0.2495	0.11773	-2.1197	0.03403	0.14769	816.663	760.537	783.6	712.781	642.906	630.246	Down
Bub1	208.853	-0.8337	0.30522	-2.7316	0.0063	0.04429	253.616	366.952	182.255	139.701	125.696	184.899	Down
Acoxl	2140.87	-2.2782	0.53162	-4.2853	1.82E-05	0.00045	3339.12	4721.05	2589.58	673.012	353.392	1169.04	Down
Morrbid	491.261	0.37611	0.17228	2.18319	0.02902	0.13197	440.646	492.228	349.891	541.469	594.482	528.85	Up
Mertk	3597.03	0.64905	0.11679	5.55751	2.74E-08	1.77E-06	2730.05	2553.87	3119.78	4289.94	4651.8	4236.76	Up
Zc3h8	572.546	-0.4363	0.19709	-2.2135	0.02686	0.12502	687.407	537.604	750.463	470.089	413.15	576.566	Down
Vinac1	65.586	-0.8915	0.29824	-2.9891	0.0028	0.02436	73.441	82.8601	99.412	41.8083	44.3028	51.6921	Down
Ttl	2021.3	-0.2321	0.09122	-2.5441	0.01096	0.06603	2218.9	2166.2	2165.62	1905.85	1756.66	1914.6	Down
Il1a	15.0411	1.19467	0.5621	2.12538	0.03355	0.14631	8.81291	9.8643	8.77165	20.3943	18.5454	23.8579	Up
Sirpa	9772.01	1.11212	0.20688	5.37567	7.63E-08	4.48E-06	6240.52	7629.05	4675.29	12775	15974.8	11337.5	Up
Cpxm1	919.775	0.49532	0.14493	3.41769	0.00063	0.00773	732.451	767.442	790.423	1200.2	910.784	1117.34	Up
Mrps26	1008.54	-0.3006	0.13718	-2.1915	0.02842	0.13027	1161.35	1048.57	1129.59	938.138	993.208	780.352	Down
Lzts3	859.026	0.42879	0.21805	1.96646	0.04925	0.18906	897.938	555.36	743.641	1057.44	794.36	1105.42	Up
4930402H	4397.3	-0.2352	0.11316	-2.0787	0.03764	0.15832	5073.3	4323.52	4868.26	4030.93	3759.56	4328.22	Down
Adam33	679.755	0.92283	0.17179	5.37183	7.79E-08	4.54E-06	432.812	576.075	399.597	859.62	910.784	899.641	Up
Siglec1	1091.22	1.76572	0.21129	8.35701	6.43E-17	2.39E-14	500.378	587.912	399.597	1548.95	2103.87	1406.62	Up
Gm14232	26.3866	-1.1303	0.47266	-2.3914	0.01679	0.09082	34.2724	28.6065	45.8075	12.2366	17.5151	19.8816	Down
Spef1	2926.01	-0.5061	0.15992	-3.1651	0.00155	0.01541	3806.2	2787.65	3708.46	2539.09	2141.99	2572.68	Down
Ap5s1	1273.47	0.32525	0.13779	2.36041	0.01825	0.09622	1081.05	1182.73	1127.64	1463.29	1574.3	1211.78	Up
Smox	1822.69	-0.3664	0.14112	-2.5965	0.00942	0.05907	2093.56	1799.25	2266.01	1697.83	1409.45	1670.05	Down
Gm14280	33.0297	-1.1349	0.42746	-2.6551	0.00793	0.05207	48.9606	35.5115	51.6552	24.4732	22.6666	14.9112	Down
5330413P	303.922	-1.1508	0.27139	-4.2406	2.23E-05	0.00054	471.001	316.644	469.77	183.549	137.03	245.537	Down
Slc23a2	16914.8	-0.4825	0.20496	-2.354	0.01857	0.09735	20138.5	15295.6	23717.6	13651.9	11519.8	17165.7	Down
Gm14051	380.383	-1.7337	0.4728	-3.6668	0.00025	0.00375	554.234	310.725	889.835	148.878	81.3936	297.229	Down
Gpcpd1	5754.29	0.72461	0.13551	5.34707	8.94E-08	5.06E-06	4019.67	4312.67	4684.06	6859.62	8255.78	6393.91	Up
Lrrn4	450.511	0.59752	0.26836	2.22659	0.02598	0.12215	343.704	488.283	243.657	630.184	457.453	539.785	Up
Bmp2	665.421	0.92959	0.2177	4.27008	1.95E-05	0.00048	422.041	503.079	449.303	745.412	1154.96	717.725	Up
Ankef1	175.427	-1.302	0.35072	-3.7124	0.00021	0.00326	345.662	141.059	262.175	89.7349	90.6663	123.266	Down
Macrod2	353.213	-0.286	0.14059	-2.0341	0.04195	0.17006	398.54	392.599	373.282	314.072	299.817	340.969	Down
Bfsp1	272.669	0.85193	0.18403	4.62939	3.67E-06	0.00012	164.508	215.042	203.697	328.348	388.423	335.999	Up

Dstn	39648.8	0.25551	0.09423	2.7115	0.0067	0.04609	36141.8	33666.8	38632.3	43111.5	44790.2	41550.5 Up
Rrbp1	14389	0.31712	0.08709	3.64114	0.00027	0.00404	12300.9	13561.4	12579.5	16194.1	16376.6	15321.7 Up
Snx5	8114.12	0.28225	0.11118	2.53868	0.01113	0.06683	7412.64	7814.5	6741.5	8847.05	9628.14	8240.91 Up
Slc24a3	2046.2	-0.7378	0.31048	-2.3762	0.01749	0.09358	3084.52	1789.38	2801.08	1442.9	937.572	2221.77 Down
Cfap61	1900.1	-1.0358	0.30211	-3.4285	0.00061	0.00751	3016.95	1635.5	3010.62	1279.74	803.633	1654.15 Down
Ralgapa2	6196.97	0.81449	0.14158	5.75268	8.78E-09	6.54E-07	3744.51	4560.26	5173.32	7637.66	8250.63	7815.45 Up
Gm14114	18.992	-1.3221	0.53454	-2.4734	0.01338	0.07656	24.4803	29.5929	27.2896	12.2366	13.3939	6.95855 Down
Xrn2	5319.38	0.21738	0.09063	2.39852	0.01646	0.08952	4658.12	5211.31	4888.73	5573.76	5921.13	5663.27 Up
Nkx2-2	22.1763	-2.32	0.65308	-3.5524	0.00038	0.00522	28.3972	54.2536	28.2642	5.09857	4.12119	12.923 Down
Nkx2-2os	20.3979	-2.3272	0.60294	-3.8597	0.00011	0.00206	23.5011	46.3622	32.1627	6.11829	9.27269	4.97039 Down
9030622C	616.354	-0.6309	0.20269	-3.1129	0.00185	0.01764	688.387	764.483	794.321	556.764	353.392	540.779 Down
Cd93	14429.5	0.28343	0.10893	2.60202	0.00927	0.05846	12288.1	13883	12878.7	15578.2	17313.1	14635.8 Up
Gzf1	2632.22	0.3767	0.11451	3.28965	0.001	0.01103	2399.07	2433.52	2038.92	2971.45	3105.32	2845.05 Up
Cst8	290.123	-1.5634	0.38022	-4.1118	3.93E-05	0.00085	398.54	629.342	272.896	123.386	97.8784	218.697 Down
Zfp120	913.251	0.39275	0.10852	3.61925	0.0003	0.00432	742.243	834.519	792.372	1007.48	1058.12	1044.78 Up
Pygb	7570.83	0.28167	0.11715	2.40445	0.0162	0.08844	7290.24	6015.25	7196.65	8584.98	8544.27	7793.58 Up
Abhd12	5124.66	0.48628	0.17987	2.70344	0.00686	0.04689	4004	5162.97	3640.23	5900.07	6967.91	5072.78 Up
Ninl	542.622	1.08666	0.14395	7.54896	4.39E-14	9.43E-12	335.87	385.694	320.652	761.727	775.815	675.973 Up
Gm14150	132.17	-0.4782	0.2179	-2.1944	0.0282	0.12942	166.466	155.856	139.372	115.228	95.8178	120.284 Down
Angpt4	34.6738	-1.1711	0.38419	-3.0483	0.0023	0.02106	50.9191	44.3893	48.7314	24.4732	21.6363	17.8934 Down
Fam110a	1582.95	-0.2661	0.12451	-2.1375	0.03256	0.14314	1688.16	1908.74	1588.64	1489.8	1503.21	1319.14 Down
Tbc1d20	6810.2	0.33111	0.09542	3.46992	0.00052	0.00666	6035.87	6131.65	5928.66	7909.93	7905.48	6949.6 Up
6820408C	2159.24	-0.8712	0.29277	-2.9756	0.00292	0.02519	3154.04	1805.17	3417.04	1724.34	985.996	1868.87 Down
Defb23	308.607	-0.6793	0.29247	-2.3226	0.0202	0.10291	478.835	265.35	395.699	283.481	164.848	263.431 Down
Defb20	13.0081	-1.9236	0.66915	-2.8746	0.00404	0.03191	23.5011	18.7422	19.4925	6.11829	7.21209	2.98224 Down
Tpx2	287.228	0.36576	0.18344	1.99388	0.04617	0.18109	222.281	296.915	233.911	336.506	297.756	335.999 Up
Ttll9	655.028	-1.0967	0.26631	-4.1181	3.82E-05	0.00083	995.859	652.03	1030.18	454.793	284.362	512.945 Down
Hck	3331.72	0.51541	0.16349	3.15254	0.00162	0.01589	2373.61	3281.85	2573.02	3693.41	4453.98	3614.47 Up
Tm9sf4	5464.32	0.21134	0.1008	2.09659	0.03603	0.15394	5324.96	4762.48	5107.05	5507.48	5823.25	6260.71 Up
Bpifa1	26111	-4.2795	1.99906	-2.1407	0.03229	0.14235	106173	234.77	42586.3	1113.53	42.2422	6516.18 Down
Bpifa5	24.1813	-3.2136	1.24784	-2.5754	0.01001	0.06195	82.2539	0	48.7314	3.05914	3.0909	7.95263 Down
Bpifb5	259.296	-2.1752	0.77368	-2.8115	0.00493	0.03698	628.655	321.576	323.576	57.104	33.9998	190.863 Down
Trp53inp2	15996.1	0.33809	0.09277	3.64443	0.00027	0.004	13860.8	14736.3	13793.9	16570.4	18682.4	18332.8 Up
Acss2	1551.87	-0.3851	0.13053	-2.9502	0.00318	0.02674	1841.9	1528.97	1902.47	1350.1	1266.24	1421.53 Down
Procr	455.929	-0.4	0.13814	-2.8955	0.00379	0.03052	529.754	505.052	521.426	428.28	354.423	396.637 Down
Cnbd2	522.104	0.64647	0.2475	2.612	0.009	0.05725	416.165	394.572	410.318	487.424	894.299	529.844 Up
Epb41l1	3417.25	0.37668	0.14482	2.6009	0.0093	0.05851	3201.05	2423.66	3296.19	3808.64	3995.5	3778.49 Up
Gm14168	76.2814	0.64506	0.31242	2.06474	0.03895	0.16216	68.5449	69.0501	40.9343	101.971	91.6966	85.4908 Up
Soga1	758.029	0.32798	0.13604	2.41083	0.01592	0.08718	646.28	649.071	721.224	798.437	946.844	786.316 Up
Tldc2	16.847	-1.3457	0.6233	-2.159	0.03085	0.13755	21.5427	22.6879	28.2642	11.2169	13.3939	3.97631 Down
Rbl1	1388.24	0.29389	0.11981	2.45306	0.01416	0.08	1131.97	1384.95	1225.11	1475.53	1567.08	1544.8 Up
Platr27	10.2798	1.9358	0.98678	1.96173	0.04979	0.19047	4.89606	7.89144	0	19.3746	19.5757	9.94079 Up
Tgm2	1452.84	0.57272	0.15375	3.72497	0.0002	0.00313	1363.06	1160.04	981.45	1738.61	1854.54	1619.35 Up
Mafb	1073.65	0.52931	0.24556	2.15552	0.03112	0.13833	642.364	1241.91	752.412	1374.58	1163.21	1267.45 Up
L3mbtl1	33.3981	0.93929	0.45427	2.0677	0.03867	0.16139	21.5427	32.5522	14.6194	36.7097	43.2725	51.6921 Up
Gdap1l1	21.1566	1.31888	0.57151	2.30773	0.02101	0.10588	12.7298	16.7693	6.82239	30.5914	39.1513	20.8756 Up
Fitm2	2445.15	-0.2652	0.09389	-2.8241	0.00474	0.03591	2820.13	2661.39	2526.23	2235.22	2157.44	2270.48 Down
Serinc3	55871.7	0.21825	0.11036	1.97763	0.04797	0.18606	51579.1	47824.1	55557.7	64216.5	61163.7	54889 Up
Ada	171.771	-0.5277	0.21232	-2.4853	0.01294	0.07468	237.949	178.544	192.002	156.016	132.908	133.207 Down
Ccn5	2023.64	0.72655	0.21679	3.35134	0.0008	0.00926	1227.93	1862.38	1483.38	2378.99	3168.17	2020.96 Up
Slpi	3747.15	0.76045	0.10336	7.35712	1.88E-13	3.53E-11	2569.45	3050.04	2726.03	4700.89	4770.28	4666.2 Up
Neurl2	361.3	-0.6697	0.19723	-3.3954	0.00069	0.00819	431.833	367.938	531.172	266.146	314.241	256.472 Down
Ctsa	13699.9	0.67162	0.13428	5.00155	5.69E-07	2.51E-05	10044.8	11992	9665.38	16390.9	18736	15370.4 Up
Zfp334	845.387	-0.4511	0.15633	-2.8856	0.00391	0.03117	992.922	905.542	1031.16	721.958	599.634	821.109 Down
Slc2a10	505.985	-0.7348	0.12895	-5.698	1.21E-08	8.72E-07	642.364	632.301	621.812	394.63	346.18	398.625 Down
Trp53rkb	183.361	-0.3972	0.18184	-2.1843	0.02894	0.13187	212.489	210.11	202.722	176.411	147.333	151.1 Down
Arfgef2	6745.65	0.29662	0.09238	3.21085	0.00132	0.01366	5788.13	6260.87	6114.81	7268.53	7923	7118.6 Up
Znfx1	4943.41	0.23237	0.10961	2.12004	0.034	0.14768	4078.42	4713.16	4846.82	5206.66	5622.34	5193.07 Up
B4galt5	3138.44	0.24472	0.12174	2.01019	0.04441	0.17633	3196.15	2573.59	2848.84	3354.86	3621.5	3235.73 Up
Gm20431	50.2959	-0.9059	0.43367	-2.0889	0.03672	0.15583	54.8359	56.2265	85.7672	27.5323	24.7272	52.6862 Down
A530013C	120.728	0.72178	0.25406	2.84102	0.0045	0.03457	94.0044	85.8194	93.5642	187.628	139.09	124.26 Up
1200007C	142.879	-1.2555	0.43939	-2.8574	0.00427	0.03331	185.071	328.481	90.6403	92.7941	91.6966	68.5914 Down
Kcng1	61.1535	0.77403	0.39373	1.96587	0.04931	0.1892	26.4387	65.1044	43.8582	85.6561	77.2724	68.5914 Up
Nfatc2	356.002	0.53815	0.16013	3.36067	0.00078	0.00899	262.429	290.01	318.703	433.379	380.18	451.312 Up
Atp9a	6340.34	0.21027	0.08615	2.44067	0.01466	0.08216	5887.03	5705.51	6044.64	6897.35	7068.88	6438.65 Up
Sall4	2.28855	-4.6161	1.94821	-2.3694	0.01782 NA		5.87528	4.93215	2.92388	0	0	0 Down
Zfp217	1800.16	0.37765	0.08463	4.46255	8.10E-06	0.00024	1554.99	1582.23	1560.38	2032.29	2072.96	1998.1 Up
Cbln4	13.3687	-4.6761	1.2285	-3.8064	0.00014	0.00245	27.418	8.87787	40.9343	0	0	2.98224 Down
Fam210b	2425.52	-0.3432	0.12109	-2.834	0.0046	0.0352	2951.35	2824.15	2362.5	2116.93	2115.2	2183 Down
Aurka	256.932	-0.3294	0.16021	-2.0563	0.03976	0.16445	299.639	278.173	280.693	216.18	217.393	249.514 Down
Bmp7	145.261	-0.943	0.21023	-4.4858	7.26E-06	0.00022	217.385	182.489	173.484	104.011	94.7875	99.4079 Down
Pck1	117.512	-4.6215	1.37233	-3.3676	0.00076	0.00884	608.091	20.715	48.7314	13.2563	10.303	3.97631 Down
1700010B	2.79016	-3.8492	1.82854	-2.1051	0.03529 NA		1.95843	9.8643	3.89851	1.01971	0	0 Down
Ctsz	13927.2	0.77054	0.13778	5.59271	2.24E-08	1.48E-06	9908.65	11577.7	9395.41	16871.2	19924.9	15885.4 Up
Fam217b	579.904	0.29994	0.13915	2.15544	0.03113	0.13833	559.13	517.876	482.441	609.79	706.785	603.406 Up
Cdh26	494.683	-3.542	1.2113	-2.9241	0.00345	0.02851	2205.19	81.8737	446.379	45.8872	53.5755	135.195 Down
Lsm14b	2750.02	-0.4384	0.19714	-2.224	0.02615	0.12272	3826.76	2357.57	3309.83	2413.67	2026.6	2565.72 Down
Slco4a1	466.297	0.3866	0.17494	2.20986	0.02711	0.12561	333.912	446.853	431.76	569.001	546.058	470.199 Up
Slc17a9	708.708	0.45704	0.12863	3.55311	0.00038	0.00522	639.426	544.509	608.167	764.786	861.33	834.032 Up
Dnajc5	6911.89	0.22973	0.07374	3.11534	0.00184	0.01752	6360.97	6397	6330.2	7293	7665.42	7424.77 Up
Zfp512b	388.525	0.44393	0.15623	2.84148	0.00449	0.03455	323.14	316.644	347.942	457.852	391.513	494.057 Up
Sox18	3599.13	-0.3722	0.15898	-2.3413	0.01922	0.09958	4194.95	4266.31	3721.13	3124.41	3716.29	2571.68 Down
Rgs19	1532.93	0.33862	0.15646	2.16426	0.03044	0.13649	1341.52	1528.97	1190.99	1683.55	1954.48	1498.08 Up

Polr3k	2521.23	-0.2816	0.09817	-2.8684	0.00413	0.03234	2692.84	2907.01	2699.72	2424.88	2182.17	2220.77	Down
Hey1	3253.09	-0.3421	0.15329	-2.2314	0.02565	0.12103	3788.57	3155.59	3966.73	3230.46	2438.72	2938.5	Down
Mrps28	754.592	-0.3747	0.15011	-2.4958	0.01257	0.07299	865.624	835.506	854.748	660.775	755.209	555.69	Down
Tpd52	8173.3	0.31568	0.07168	4.40436	1.06E-05	0.00029	7434.18	7250.26	7163.51	9009.18	9020.26	9162.42	Up
Pag1	2804.59	0.62975	0.12681	4.96616	6.83E-07	2.88E-05	2092.58	2353.62	2159.77	3086.68	3856.41	3278.47	Up
Fabp5	24474.4	0.86387	0.20452	4.22397	2.40E-05	0.00057	15968	20592.7	15514.1	30276.4	24350.1	40144.9	Up
Gm38335	3.3765	4.19391	1.66302	2.52186	0.01167	NA	0.97921	0	0	4.07886	8.24239	6.95855	Up
4632432E	137.911	-0.7862	0.35925	-2.1884	0.02864	0.13089	221.302	194.327	108.184	122.366	66.9694	114.319	Down
1700029B	23.792	-1.09	0.50601	-2.1541	0.03123	0.13861	36.2309	40.4436	20.4672	13.2563	15.4545	16.8993	Down
Fabp12	493.336	-0.6076	0.2127	-2.8568	0.00428	0.03334	581.652	734.89	470.745	387.492	336.908	448.329	Down
Ralyl	267.194	-1.3758	0.44521	-3.0904	0.002	0.01881	420.082	195.313	541.893	199.864	70.0603	175.952	Down
Car3	1543.65	-5.3008	1.39129	-3.81	0.00014	0.00242	8080.46	331.44	620.838	52.0055	147.333	29.8224	Down
Car2	5061.67	-0.4911	0.17399	-2.8226	0.00476	0.03604	5465.97	7470.23	4808.81	4257.31	4200.53	4167.18	Down
Sirpb1a	303.697	1.05174	0.30701	3.42572	0.00061	0.00756	175.279	291.983	125.727	391.571	469.816	367.809	Up
Sirpb1b	321.688	0.7758	0.32849	2.36168	0.01819	0.09611	197.801	344.264	169.585	371.176	547.088	300.212	Up
Sirpb1c	672.326	1.33336	0.24883	5.35847	8.39E-08	4.84E-06	346.641	507.025	292.388	911.625	1188.96	787.31	Up
Gm5150	292.396	0.8076	0.30583	2.64073	0.00827	0.05384	185.071	291.983	160.814	338.545	497.634	280.33	Up
Bhlhe22	180.616	0.85461	0.36503	2.34119	0.01922	0.09958	125.339	192.354	68.2239	202.923	277.15	217.703	Up
Cyp7b1	611.89	0.48776	0.15732	3.10051	0.00193	0.01827	474.918	472.5	580.878	633.243	729.451	780.352	Up
Trim55	52.7004	-1.9852	0.52264	-3.7984	0.00015	0.00251	123.381	36.4979	92.5896	22.4337	15.4545	25.846	Down
Cpb1	104.192	-0.9877	0.45681	-2.1621	0.03061	0.13702	120.443	227.865	67.2493	70.3603	86.5451	52.6862	Down
Nlgn1	348.862	-4.3532	1.22136	-3.5642	0.00036	0.00505	1280.81	26.6336	688.087	41.8083	32.9696	22.8638	Down
Nceh1	8047.22	0.40305	0.13962	2.88666	0.00389	0.03112	6882.89	7267.03	6641.11	9116.25	10618.3	7757.79	Up
Tnfsf10	6210.75	0.5545	0.28093	1.97379	0.04841	0.18691	3125.65	6760	5209.38	6094.84	9597.23	6477.42	Up
Fndc3b	5879.72	0.35147	0.13557	2.59252	0.00953	0.05967	5064.49	5891.94	4544.69	6066.28	7142.03	6568.87	Up
Tmem212	6307.35	-0.9247	0.2573	-3.5938	0.00033	0.00466	9022.47	5916.6	9847.63	5136.3	2920.9	5000.22	Down
Pld1	2619.49	0.38293	0.12148	3.1522	0.00162	0.0159	2219.88	2258.92	2343	2828.69	2702.47	3363.96	Up
Eif5a2	1457.9	0.60822	0.14342	4.24077	2.23E-05	0.00054	968.441	1201.47	1295.28	1732.5	1691.75	1857.93	Up
Lrrc34	1202.16	-1.0976	0.2939	-3.7345	0.00019	0.00305	1981.93	1005.17	1928.79	823.93	535.755	937.416	Down
Slc7a14	66.8827	-1.8475	0.67415	-2.7404	0.00614	0.04344	120.443	58.1993	135.473	13.2563	10.303	63.621	Down
Kcnmb2	1661.01	-1.1571	0.33099	-3.496	0.00047	0.00617	2920.01	1460.9	2499.92	1099.25	588.3	1397.67	Down
Ndufb5	2723	-0.2372	0.0959	-2.4733	0.01339	0.07656	3072.77	2800.47	2965.79	2626.79	2381.02	2491.16	Down
Gm9791	116.567	0.46408	0.21528	2.15575	0.0311	0.13833	103.797	90.7515	99.412	133.583	126.727	145.135	Up
Fxr1	1008.13	0.31948	0.14806	2.15772	0.03095	0.13787	997.818	836.492	856.697	1080.9	1002.48	1274.41	Up
Sox2ot	183.921	-0.8834	0.37752	-2.34	0.01928	0.09967	326.078	142.046	247.555	125.425	84.4845	177.94	Down
1700017M	4.58273	-3.6469	1.47695	-2.4692	0.01354	NA	9.79213	5.91858	9.74627	2.03943	0	0	Down
Exosc9	836.909	0.22335	0.10411	2.1452	0.03194	0.14102	747.139	778.293	791.397	887.152	885.026	932.446	Up
Bbs7	1064.31	-0.5849	0.22955	-2.5479	0.01084	0.06552	1354.25	956.837	1520.42	793.338	692.361	1068.63	Down
Cetn4	1276.55	-0.8899	0.31662	-2.8107	0.00494	0.03705	2008.37	1035.75	1930.74	866.758	580.058	1237.63	Down
Gm43439	19.471	-1.1966	0.54554	-2.1934	0.02828	0.12973	25.4595	27.62	28.2642	17.3352	7.21209	10.9349	Down
Gm20755	209.621	-1.1689	0.37232	-3.1395	0.00169	0.01651	328.036	224.906	317.728	86.6758	89.636	210.745	Down
Intu	1197.82	-0.4761	0.23528	-2.0236	0.04302	0.17286	1681.31	968.674	1531.14	1029.91	809.815	1166.05	Down
Slc25a31	44.8808	-1.7499	0.39842	-4.3921	1.12E-05	0.0003	74.4202	55.2401	77.9702	17.3352	16.4848	27.8342	Down
Hspa4l	7990.79	-0.615	0.21775	-2.8241	0.00474	0.03591	11748.6	7359.75	9897.34	5888.85	5145.31	7904.91	Down
1700052H	18.3398	-3.0124	0.75152	-4.0085	6.11E-05	0.00123	52.8775	18.7422	26.3149	4.07886	2.0606	5.96447	Down
D3Ertd751	961.183	-0.3927	0.13739	-2.8583	0.00426	0.03325	1170.16	1005.17	1098.4	771.924	783.027	938.41	Down
Slc7a11	471.337	0.80833	0.18141	4.45595	8.35E-06	0.00024	319.223	346.237	362.561	515.976	556.361	727.665	Up
Gm6209	48.506	2.23773	0.47434	4.71759	2.39E-06	8.46E-05	11.7506	11.8372	27.2896	92.7941	91.6966	55.6684	Up
Gm37199	3.04158	5.07663	1.79274	2.83177	0.00463	NA	0	0	0	4.07886	7.21209	6.95855	Up
Gm5103	54.8611	-0.7726	0.34529	-2.2376	0.02525	0.1195	81.2747	55.2401	71.1478	44.8675	30.909	45.7276	Down
Gm30173	8.38466	-1.6528	0.84163	-1.9638	0.04955	0.18981	16.6466	7.89144	13.6448	2.03943	4.12119	5.96447	Down
Nhlrc3	2045.08	0.38625	0.13687	2.82202	0.00477	0.03605	1670.54	1970.89	1677.33	2187.29	2624.17	2140.25	Up
Frem2	91.4235	-1.9205	0.301	-6.3805	1.76E-10	1.82E-08	172.341	112.453	149.118	43.8477	33.9998	36.7809	Down
Sertm1	2.12426	-4.5087	2.13135	-2.1154	0.03439	NA	0.97921	5.91858	5.84776	0	0	0	Down
Nbea	2210.73	0.21646	0.09621	2.24987	0.02446	0.11691	2140.56	2046.84	1948.28	2279.06	2361.44	2488.18	Up
Tm4sf1	18757.4	-0.5863	0.12034	-4.8726	1.10E-06	4.32E-05	23947.6	19465.2	24139.6	15008.2	15618.3	14365.4	Down
Tm4sf4	45.238	-2.2784	0.56839	-4.0084	6.11E-05	0.00123	65.6073	53.2672	106.234	16.3154	5.15149	24.852	Down
Gm26671	694.821	-0.482	0.19169	-2.5146	0.01192	0.07019	633.551	877.922	918.099	590.415	502.786	646.151	Down
BB187690	283.371	-0.8195	0.31701	-2.5851	0.00973	0.06054	395.602	211.096	478.542	250.85	178.242	185.893	Down
Ankub1	346.65	-0.8313	0.35149	-2.3651	0.01803	0.09563	509.191	226.879	595.497	284.5	186.484	277.348	Down
Pfn2	1602.92	-0.9498	0.21312	-4.4567	8.32E-06	0.00024	2155.25	1599	2582.76	1018.7	941.693	1320.14	Down
Gm42513	11.2639	-2.8902	1.22125	-2.3666	0.01795	0.09533	19.5843	2.95929	37.0358	2.03943	0	5.96447	Down
4921539H	10.6282	-3.5382	1.24348	-2.8454	0.00444	0.0342	43.0854	3.94572	11.6955	0	2.0606	2.98224	Down
Gm8234	51.3435	-1.9755	0.55944	-3.5313	0.00041	0.00557	79.3162	57.2129	109.158	19.3746	7.21209	35.7868	Down
4930593A	59.8265	-0.8582	0.30924	-2.775	0.00552	0.04015	77.3578	85.8194	68.2239	39.7689	37.0907	50.698	Down
P2ry1	242.941	-0.5248	0.20427	-2.5693	0.01019	0.06282	265.367	254.499	340.145	194.766	177.211	225.656	Down
Rap2b	535.829	0.44713	0.12498	3.57775	0.00035	0.00486	428.895	464.608	466.846	583.277	648.058	623.287	Up
Lekr1	461.111	-0.439	0.15507	-2.8308	0.00464	0.03543	480.793	523.794	587.7	380.354	360.604	433.418	Down
Gm37359	185.493	-1.3228	0.35729	-3.7024	0.00021	0.00336	290.826	207.15	297.261	98.9123	59.7573	159.053	Down
Veph1	243.01	0.42539	0.21365	1.99103	0.04648	0.18197	207.593	197.286	217.342	319.171	303.938	212.733	Up
Mlf1	2811.85	-0.9303	0.28506	-3.2635	0.0011	0.01186	4440.73	2329.95	4294.21	2143.44	1317.75	2345.03	Down
Mfsd1	7921.43	0.25628	0.11005	2.32875	0.01987	0.10187	7819.01	6553.84	7286.31	8714.48	8089.9	9065	Up
Smc4	3632.36	0.30366	0.11295	2.68848	0.00718	0.04845	2892.59	3551.15	3310.81	3920.8	3992.41	4126.42	Up
Sptssb	2265.77	0.68269	0.29724	2.29681	0.02163	0.10754	1509.95	1385.93	2322.54	3575.12	1659.81	3141.29	Up
Bche	689.195	-0.5304	0.17508	-3.0296	0.00245	0.02206	663.906	877.922	901.53	527.193	637.755	526.862	Down
Gm37464	607.256	-0.7883	0.24699	-3.1918	0.00141	0.0143	924.377	585.939	797.245	499.66	319.393	516.921	Down
Wdr49	797.774	-0.7435	0.31521	-2.3587	0.01834	0.09644	1145.68	591.858	1259.22	757.648	406.968	625.275	Down
Fnip2	155.709	0.59251	0.28151	2.10476	0.03531	0.15156	122.402	139.087	111.108	130.524	189.575	241.561	Up
Ppid	3790.15	-0.2428	0.10337	-2.3484	0.01885	0.09825	4455.42	3861.87	4007.67	3474.17	3311.38	3630.37	Down
Map9	1127.39	-0.7834	0.19611	-3.9944	6.49E-05	0.0013	1534.43	1108.75	1635.42	813.732	706.785	965.25	Down
Npy2r	6.25881	3.10107	1.37163	2.26086	0.02377	NA	3.91685	0	0	7.138	18.5454	7.95263	Up
Fgg	655.335	1.3928	0.3957	3.51984	0.00043	0.00573	290.826	516.889	276.794	739.293	530.604	1577.6	Up
Fga	428.359	2.23656	0.42262	5.29217	1.21E-07	6.51E-06	126.318	166.707	156.915	440.517	387.392	1292.3	Up

Plrg1	3212.13	-0.2689	0.11206	-2.3998	0.01641	0.08926	3799.35	3169.4	3563.24	2984.71	2752.96	3003.11 Down
Sfrp2	132.054	2.43613	0.55031	4.42686	9.56E-06	0.00027	37.2101	49.3215	37.0358	424.201	169.999	74.5559 Up
Gm7115	37.2248	0.83168	0.36502	2.27845	0.0227	0.11091	28.3972	24.6607	27.2896	40.7886	51.5149	50.698 Up
Tlr2	3367.6	1.05661	0.14886	7.09787	1.27E-12	2.05E-10	2120.97	2420.7	2018.45	4278.72	5331.79	4034.96 Up
Fhdc1	1912.68	-0.5136	0.21199	-2.4229	0.0154	0.08504	2170.91	1791.36	2786.46	1945.62	1337.33	1444.4 Down
Cd1d1	915.106	0.87468	0.11006	7.94712	1.91E-15	5.64E-13	689.366	640.193	608.167	1156.36	1182.78	1213.77 Up
Fcrls	386.819	0.4708	0.18398	2.55894	0.0105	0.0642	271.242	390.626	310.906	442.556	480.119	425.466 Up
Etv3	2882.93	0.22517	0.10698	2.10483	0.03531	0.15156	2626.25	2806.39	2542.8	3037.73	2908.53	3375.89 Up
Arhgef11	2330.42	0.37749	0.09815	3.8462	0.00012	0.00216	2149.37	2014.29	1918.07	2584.98	2757.08	2558.76 Up
Lrrc71	2789.23	-0.6674	0.25218	-2.6464	0.00814	0.05318	3861.04	2293.45	4114.88	2517.68	1608.3	2340.06 Down
Bcan	1242.56	-2.9382	0.57498	-5.1101	3.22E-07	1.55E-05	1768.46	3353.86	1472.66	232.495	158.666	469.205 Down
1700113A	76.832	-0.6357	0.31149	-2.0409	0.04126	0.1682	99.8797	107.521	73.097	73.4195	48.424	58.6506 Down
Glmp	5610.71	0.29541	0.10094	2.92644	0.00343	0.02835	5094.84	5361.24	4658.72	6269.21	6430.09	5850.15 Up
Bglap3	398.773	3.05237	0.46104	6.62058	3.58E-11	4.42E-09	135.131	34.525	87.7165	942.217	371.938	821.109 Up
Sema4a	4527.65	0.74705	0.16887	4.42379	9.70E-06	0.00027	3247.07	4091.71	2804	5418.76	6376.52	5227.86 Up
Fdps	175.225	0.95388	0.23011	4.1453	3.39E-05	0.00076	103.797	145.992	108.184	204.963	263.756	224.662 Up
Hcn3	41.7161	0.97903	0.41595	2.35369	0.01859	0.09738	35.2517	22.6879	26.3149	37.7295	58.727	69.5855 Up
Gba	3166.61	0.376	0.11251	3.34181	0.00083	0.00951	2808.38	2891.23	2569.12	3643.44	3836.83	3250.64 Up
Muc1	43172.6	0.68126	0.12052	5.65252	1.58E-08	1.10E-06	32059.4	37841.4	29592.6	52554.1	54804.7	52183.2 Up
Trim46	1236.03	0.60453	0.11058	5.4668	4.58E-08	2.79E-06	923.398	1021.94	997.044	1387.83	1565.02	1520.94 Up
Efna3	340.228	-0.6397	0.21063	-3.0372	0.00239	0.02165	318.244	508.011	417.14	255.948	261.696	280.33 Down
Adam15	217.255	0.48974	0.21367	2.29206	0.0219	0.10849	199.759	185.449	156.915	254.929	296.726	209.751 Up
Shc1	5351.03	0.20605	0.07614	2.7064	0.0068	0.04659	5060.57	4837.45	5010.56	5664.52	5847.97	5685.14 Up
Pbxip1	8923.07	0.36402	0.09204	3.95508	7.65E-05	0.00149	7666.26	8092.67	7650.82	9550.65	10762.5	9815.53 Up
Pmvk	8321.1	-0.4815	0.18711	-2.5736	0.01007	0.06223	10881	10058.6	8151.78	6024.48	6105.55	8705.15 Down
Kcnn3	1352.81	1.58242	0.28278	5.59603	2.19E-08	1.46E-06	852.894	791.117	387.902	1858.94	2436.66	1789.34 Up
Adar	675.753	0.50432	0.14779	3.41229	0.00064	0.00785	516.045	549.441	611.091	809.654	696.482	871.807 Up
Atp8b2	1480.74	0.24436	0.10007	2.4418	0.01461	0.08204	1413	1297.15	1356.68	1691.71	1569.14	1556.73 Up
Ubap2l	1078.52	0.38659	0.13381	2.88908	0.00386	0.03099	1076.15	853.262	875.215	1223.66	1198.24	1244.59 Up
1700094D	2166.25	-1.0803	0.2922	-3.697	0.00022	0.00342	3529.08	1880.13	3415.09	1480.63	922.117	1770.45 Down
Tpm3	10220.2	1.18614	0.25476	4.65598	3.22E-06	0.00011	6159.25	6298.35	6263.93	15784.2	18446.5	8369.15 Up
Nup210l	228.556	-0.437	0.19597	-2.2298	0.02576	0.12134	252.637	253.512	282.642	215.16	157.636	209.751 Down
Slc39a1	4964.07	0.4914	0.1198	4.102	4.10E-05	0.00089	4246.85	4488.25	3645.11	5591.1	6206.52	5606.6 Up
Dennd4b	1397.91	0.50233	0.16432	3.05703	0.00224	0.02057	1067.34	1308.99	1094.51	1555.07	1940.05	1421.53 Up
Gm43595	370.375	-0.5924	0.17806	-3.3272	0.00088	0.00988	436.729	486.31	413.242	293.678	249.332	342.957 Down
S100a7a	67.5173	-0.6873	0.26978	-2.5477	0.01084	0.06555	83.2331	82.8601	83.8179	46.9069	54.6058	53.6802 Down
C2cd4d	2.62323	-3.7545	1.90823	-1.9675	0.04912	NA	8.81291	4.93215	0.97463	1.01971	0	0 Down
Oaz3	50.1227	-0.8429	0.32375	-2.6034	0.00923	0.05831	64.628	62.1451	66.2747	31.6112	43.2725	32.8046 Down
Rfx5	316.862	0.51166	0.18669	2.74073	0.00613	0.04344	238.928	250.553	294.337	322.23	363.695	431.43 Up
Tnfaip8l2	601.141	0.6086	0.22059	2.75901	0.0058	0.04175	434.77	627.369	366.46	709.722	799.512	669.015 Up
Ctsk	7186.07	1.0696	0.15857	6.74533	1.53E-11	2.01E-09	4975.38	3733.64	5204.51	9713.8	10665.6	8823.44 Up
Ctss	20849.5	1.44804	0.19683	7.35693	1.88E-13	3.53E-11	11554.7	13988.6	8009.49	29444.3	31716.7	30383 Up
Car14	1031.17	-0.4138	0.17641	-2.3459	0.01898	0.09871	965.504	1340.56	1228.03	869.817	1010.72	772.399 Down
Sf3b4	1840.97	-0.3037	0.10374	-2.9272	0.00342	0.02831	2102.37	1924.52	2074.98	1752.89	1641.27	1549.77 Down
Bola1	782.95	-0.5639	0.14976	-3.7656	0.00017	0.00277	958.649	857.207	986.323	722.978	552.24	620.305 Down
H2bc21	469.559	-0.6441	0.13826	-4.659	3.18E-06	0.00011	517.024	596.79	604.269	374.235	349.271	375.762 Down
Acp6	1315.79	-0.2372	0.1047	-2.2653	0.02349	0.11362	1410.07	1343.52	1517.49	1162.47	1239.45	1221.72 Down
Hmgcs2	1104.23	-1.4207	0.17932	-7.9225	2.33E-15	6.78E-13	1569.68	1764.72	1489.23	744.392	499.695	557.678 Down
Phgdh	899.771	1.00509	0.15191	6.61651	3.68E-11	4.51E-09	648.239	646.111	500.958	1176.75	1301.27	1125.3 Up
Spag17	1134.7	-0.6285	0.2896	-2.1704	0.02998	0.13499	1514.84	943.027	1676.36	976.887	571.816	1125.3 Down
Gm43464	19.6241	-1.2725	0.50556	-2.517	0.01183	0.06986	30.3556	24.6607	28.2642	9.17743	12.3636	12.923 Down
Trim45	312.157	-0.5451	0.1408	-3.8714	0.00011	0.00198	381.893	362.02	367.434	256.968	244.181	260.449 Down
Ptgfrn	13580	0.37931	0.11698	3.24264	0.00118	0.01259	11772.1	12391.5	11251.1	14673.7	17314.2	14077.1 Up
Cd2	1261.31	0.60971	0.21754	2.80272	0.00507	0.03776	909.689	1273.48	812.839	1448	1850.42	1273.41 Up
Casq2	154.145	-2.4075	1.09007	-2.2086	0.0272	0.12588	413.228	40.4436	324.551	42.828	12.3636	91.4552 Down
Vangl1	1874.48	-0.2389	0.11795	-2.0252	0.04285	0.17231	2087.68	1824.89	2175.37	1774.3	1605.2	1779.4 Down
Gm16160	66.1731	-1.609	0.37914	-4.2439	2.20E-05	0.00053	96.9421	117.385	84.7926	30.5914	20.606	46.7217 Down
A230001N	50.9573	-1.7853	0.46985	-3.7996	0.00014	0.0025	60.7112	74.9687	101.361	29.5717	10.303	28.8283 Down
Ngf	10.509	-1.9236	0.9667	-1.9899	0.04661	0.18237	33.2932	3.94572	12.6702	3.05914	4.12119	5.96447 Down
Nr1h5	64.2331	-0.8318	0.40221	-2.068	0.03864	0.16131	62.6696	74.9687	109.158	57.104	27.8181	53.6802 Down
Ampd1	28.9425	-1.7641	0.47468	-3.7165	0.0002	0.00321	52.8775	34.525	46.7821	9.17743	13.3939	16.8993 Down
Bcl2l15	138.648	3.71415	0.55992	6.63339	3.28E-11	4.11E-09	16.6466	29.5929	12.6702	180.49	481.149	111.337 Up
Ptpn22	787.136	0.51891	0.26386	1.96661	0.04923	0.18906	484.71	599.749	856.697	862.679	1199.27	719.713 Up
Magi3	710.299	0.46156	0.16235	2.8429	0.00447	0.03442	569.902	649.071	574.055	696.465	815.996	956.304 Up
Gm5546	34.2544	-0.8873	0.43015	-2.0628	0.03913	0.16261	36.2309	56.2265	40.9343	31.6112	21.6363	18.8875 Down
Slc16a1	3650.3	0.49663	0.11058	4.49133	7.08E-06	0.00021	2866.16	3264.1	2954.1	4493.88	4414.83	3908.72 Up
Ppm1j	515.032	0.93307	0.23951	3.89576	9.79E-05	0.00183	377.976	403.45	280.693	714.82	822.178	491.075 Up
Capza1	4476.4	0.40063	0.09477	4.22748	2.36E-05	0.00057	3832.64	4111.44	3632.44	4933.38	5292.64	5055.88 Up
Gm5547	107.37	1.61403	0.2933	5.503	3.73E-08	2.32E-06	57.7736	37.4843	63.3508	185.588	135.999	164.023 Up
Tmigd3	47.7287	1.30821	0.38052	3.43799	0.00059	0.0073	17.6258	36.4979	28.2642	72.3998	66.9694	64.6151 Up
I830077J0	416.162	0.95294	0.23077	4.12936	3.64E-05	0.0008	252.637	364.979	232.936	455.813	641.876	548.731 Up
Atp5pb	14131.3	0.26637	0.07479	3.56163	0.00037	0.00508	13231.1	12764.4	12495.7	15647.5	15570.9	15078.2 Up
Pifo	1878.07	-1.0463	0.31483	-3.3234	0.00089	0.00998	3060.04	1569.41	2962.87	1311.35	758.3	1606.43 Down
Chil5	44.7065	-1.2061	0.35363	-3.4108	0.00065	0.00788	52.8775	62.1451	72.1224	23.4534	27.8181	29.8224 Down
Chil3	62780.1	3.03493	0.32484	9.34289	9.37E-21	6.12E-18	16194.2	14476.8	10290.1	105408	170017	60294.8 Up
Chil4	35.0061	2.5067	0.62138	4.03409	5.48E-05	0.00113	9.79213	16.7693	4.87314	59.1435	89.636	29.8224 Up
Gm6522	61.1376	2.79812	0.54637	5.12132	3.03E-07	1.48E-05	18.605	17.7557	9.74627	94.8335	176.181	49.7039 Up
Gm38244	92.3358	1.31497	0.2621	5.01698	5.25E-07	2.32E-05	49.9399	58.1993	50.6806	147.859	135.999	111.337 Up
2010016I1	29.5332	1.38737	0.50874	2.72705	0.00639	0.04464	8.81291	17.7557	22.4164	42.828	53.5755	31.8105 Up
Dennd2d	3336.72	0.4019	0.12382	3.24594	0.00117	0.01248	2744.73	3043.14	2837.14	3667.91	3433.98	4293.43 Up
Dram2	9744.69	0.29594	0.14332	2.06496	0.03893	0.16211	8397.73	10270.7	7577.73	10262.4	11633.1	10326.5 Up
Cd53	4635.23	1.01251	0.1935	5.23257	1.67E-07	8.69E-06	2866.16	3839.18	2511.61	5824.61	7320.27	5449.54 Up
Gm27008	58.4949	1.38733	0.31265	4.43733	9.11E-06	0.00026	27.418	36.4979	33.1373	90.7546	88.6057	74.5559 Up

Kcna2	339.561	-0.6464	0.20901	-3.0928	0.00198	0.01869	450.438	431.07	361.587	329.368	245.211	219.691	Down
Csf1	2272.26	0.73621	0.16767	4.39086	1.13E-05	0.00031	1405.17	2090.24	1618.86	2709.38	3039.38	2770.5	Up
Gstm7	313.53	-0.596	0.25325	-2.3535	0.0186	0.09738	476.877	279.16	376.206	234.534	211.211	303.194	Down
Gstm6	277.518	-0.8033	0.23486	-3.4204	0.00063	0.00767	379.935	272.255	406.42	213.12	161.757	231.62	Down
Gstm2	9098.01	-0.6213	0.13266	-4.6838	2.82E-06	9.60E-05	11104.3	10456.2	11522	7441.88	6064.34	7999.35	Down
Gstm1	19622.5	-0.4999	0.1506	-3.3196	0.0009	0.0101	22619.8	18982.9	27363.6	16953.8	15757.4	16057.4	Down
Ampd2	1461.64	0.49422	0.12945	3.81774	0.00013	0.00237	1280.81	1191.61	1168.58	1606.05	1944.17	1578.6	Up
Atxn7l2	378.354	-0.3202	0.1578	-2.0295	0.04241	0.17118	398.54	440.934	421.039	292.658	373.998	342.957	Down
Sort1	26614.8	0.45952	0.11261	4.08047	4.49E-05	0.00096	21472.2	24944.8	20818	29337.2	32810.9	30305.5	Up
1700013F(	757.491	-1.0064	0.29548	-3.406	0.00066	0.00798	1246.54	613.559	1174.43	530.252	367.817	612.352	Down
Henmt1	25.5087	1.2575	0.51106	2.46057	0.01387	0.07874	12.7298	19.7286	12.6702	43.8477	41.2119	22.8638	Up
Fam102b	2373.56	0.61733	0.16433	3.7566	0.00017	0.00285	1926.11	1868.3	1825.48	2973.49	3382.47	2265.5	Up
Slc25a24	2735.98	0.21466	0.09387	2.28666	0.02222	0.10951	2482.3	2539.07	2576.91	2895.99	3135.2	2786.4	Up
Amy1	563.332	-1.189	0.19481	-6.1034	1.04E-09	9.18E-08	875.416	605.668	868.393	303.875	366.786	359.856	Down
A930005H	284.724	-0.3804	0.15747	-2.4159	0.01569	0.08629	316.286	311.712	338.196	223.318	252.423	266.413	Down
Slc30a7	4343.24	0.29287	0.08351	3.50693	0.00045	0.00597	3803.26	4098.62	3809.82	4730.46	4879.49	4737.78	Up
Dbt	1609.88	-0.2858	0.13087	-2.1841	0.02896	0.13189	1800.77	1810.1	1695.85	1333.79	1349.69	1669.06	Down
Frrs1	516.119	0.83432	0.23859	3.49694	0.00047	0.00615	411.269	376.816	324.551	662.815	852.057	469.205	Up
Snx7	4874.23	0.46191	0.1941	2.37977	0.01732	0.093	4266.43	4996.27	3038.89	5680.83	6223	5039.98	Up
Dpyd	393.413	-0.7541	0.31156	-2.4205	0.0155	0.08554	397.56	375.83	708.554	288.579	211.211	378.744	Down
Rwdd3	610.528	-0.5087	0.22248	-2.2864	0.02223	0.10957	711.888	554.373	884.962	540.449	403.877	567.619	Down
Tlcd4	5944.13	0.41428	0.14432	2.87049	0.0041	0.03219	5219.2	5670.98	4399.47	6091.78	6657.79	7625.58	Up
A730020M	35.1661	-0.95	0.4776	-1.9891	0.04669	0.18257	40.1477	37.4843	61.4015	13.2563	29.8787	28.8283	Down
Synpo2	763.286	-0.4161	0.12846	-3.2395	0.0012	0.01271	947.878	843.397	826.484	615.908	708.845	637.204	Down
Ugt8a	293.223	-2.1116	0.40299	-5.2398	1.61E-07	8.42E-06	537.588	278.173	613.041	142.76	53.5755	134.201	Down
Arsj	46.8425	-0.6886	0.32808	-2.0989	0.03583	0.1532	54.8359	54.2536	64.3254	39.7689	36.0604	31.8105	Down
Camk2d	4624.56	0.2744	0.13992	1.96109	0.04987	0.19052	3823.83	4620.44	4113.9	4763.09	5831.49	4594.63	Up
Gm35585	14.3441	4.38249	1.46216	2.99727	0.00272	0.02387	0	3.94572	0	22.4337	57.6967	1.98816	Up
Fam241a	1606.36	0.41025	0.15529	2.64181	0.00825	0.05372	1357.19	1264.6	1516.52	2015.98	1968.9	1514.98	Up
Enpep	11038.5	0.4721	0.19124	2.46869	0.01356	0.07739	9962.51	10962.2	6820.44	11868.5	14454.1	12163.5	Up
Cfi	465.605	1.35766	0.19488	6.96668	3.25E-12	4.62E-10	208.572	292.97	282.642	578.178	672.785	758.482	Up
Lef1	322.735	-0.5652	0.2182	-2.5903	0.00959	0.05988	371.122	467.568	316.754	226.377	308.059	246.531	Down
Hadh	4897.42	-0.3881	0.09686	-4.0068	6.15E-05	0.00124	5543.32	5357.3	5756.15	4514.28	3941.92	4271.56	Down
Cyp2u1	130.315	-0.5628	0.23173	-2.4289	0.01515	0.08398	149.82	132.182	184.205	111.149	106.121	98.4138	Down
4930534D	2.2914	-4.6177	2.02167	-2.2841	0.02236	NA	1.95843	7.89144	3.89851	0	0	0	Down
Sgms2	1139.44	-0.7157	0.25452	-2.8121	0.00492	0.03694	1789.02	923.298	1536.99	788.24	757.269	1041.79	Down
Gm29865	29.4142	-1.4227	0.55324	-2.5716	0.01012	0.0625	43.0854	50.3079	35.0866	19.3746	22.6666	5.96447	Down
Gm43254	186.764	-0.4921	0.1846	-2.6658	0.00768	0.05085	208.572	244.635	201.748	149.898	158.666	157.064	Down
Gm26691	15.154	-1.7033	0.63411	-2.6861	0.00723	0.04867	28.3972	16.7693	24.3657	6.11829	10.303	4.97039	Down
Cxxc4	129.071	-1.5874	0.39466	-4.0222	5.77E-05	0.00117	185.071	108.507	287.515	71.38	47.3937	74.5559	Down
Manba	2239.88	0.38998	0.12243	3.18527	0.00145	0.01456	1908.49	1875.2	2033.07	2513.6	2838.47	2270.48	Up
Slc39a8	22755.6	0.43471	0.16183	2.68623	0.00723	0.04867	16327.4	23864.7	17866.9	24384.4	26954.7	27135.4	Up
Ddit4l	523.169	-0.9439	0.20118	-4.6918	2.71E-06	9.28E-05	709.929	556.346	799.194	376.275	294.665	402.602	Down
H2az1	8734.6	0.32167	0.12789	2.51524	0.0119	0.07008	7331.37	7810.55	8152.76	10817.1	8363.96	9931.84	Up
Dnajb14	2584.64	0.20588	0.10017	2.05524	0.03986	0.16471	2442.16	2402.94	2356.65	2567.64	2761.2	2977.27	Up
Lamtor3	3475.69	0.24887	0.12206	2.03898	0.04145	0.16869	3346.95	3242.39	2940.45	3663.84	4217.01	3443.49	Up
Dapp1	1215.2	0.75456	0.16272	4.63711	3.53E-06	0.00012	868.562	872.004	972.678	1576.48	1762.84	1238.62	Up
Mttp	307.759	-0.9489	0.25864	-3.6687	0.00024	0.00374	380.914	355.115	480.491	226.377	143.211	260.449	Down
Tspan5	1916.78	0.45737	0.19872	2.30163	0.02136	0.10682	1758.67	1710.47	1377.15	2261.73	2709.68	1682.97	Up
Bmpr1b	1200.46	-1.1276	0.27108	-4.1596	3.19E-05	0.00072	1613.74	1157.08	2170.49	911.625	533.695	816.138	Down
Kyat3	542.638	-0.4967	0.22444	-2.213	0.0269	0.12516	634.53	578.048	692.96	480.286	316.302	553.702	Down
Gm43646	39.0328	-0.8577	0.38399	-2.2337	0.0255	0.12048	51.8983	46.3622	52.6299	36.7097	24.7272	21.8697	Down
Lmo4	4454.67	0.32479	0.1229	2.64278	0.00822	0.05362	4222.37	3704.04	3939.44	4971.11	5485.31	4405.76	Up
Clca3a1	319.837	0.54428	0.25915	2.10022	0.03571	0.15281	276.138	216.028	288.49	473.148	259.635	405.584	Up
Bcl10	3134.81	0.21248	0.08849	2.40124	0.01634	0.08901	2782.92	2981.98	2948.25	3507.82	3255.74	3332.15	Up
Wdr63	2980.02	-0.7548	0.28438	-2.6542	0.00795	0.05219	4749.18	2469.03	4008.64	2446.3	1471.27	2735.7	Down
Mcoln3	1185.32	2.01241	0.27434	7.33557	2.21E-13	4.10E-11	442.604	592.844	377.181	1525.49	2705.56	1468.25	Up
Mcoln2	277.988	0.77781	0.16276	4.77881	1.76E-06	6.45E-05	183.113	211.096	220.266	359.959	328.665	364.827	Up
Adgrl4	10511	0.36697	0.15858	2.31412	0.02066	0.10452	8036.4	9643.34	9864.2	11152.6	14125.4	10244	Up
Gipc2	4400.76	-0.544	0.17892	-3.0406	0.00236	0.02145	5490.45	4061.13	6110.91	3636.3	3141.38	3964.39	Down
Dnajb4	5249.04	-0.2248	0.10982	-2.0466	0.04069	0.16654	6008.45	5732.14	5230.82	5055.75	4425.13	5041.97	Down
Usp33	2381.03	0.4433	0.1545	2.86921	0.00412	0.03229	2094.54	2193.82	1766.02	2450.37	2569.56	3211.87	Up
Acadm	4578.66	-0.3379	0.10455	-3.2315	0.00123	0.01299	5464.01	5144.23	4728.89	4228.76	3793.56	4112.5	Down
Slc44a5	32.5753	-1.5913	0.56643	-2.8094	0.00496	0.03716	63.6488	20.715	62.3761	12.2366	19.5757	16.8993	Down
Cryz	762.996	-0.4395	0.19389	-2.2666	0.02342	0.1135	875.416	857.207	902.505	602.652	514.119	826.079	Down
Erich3	2484.28	-0.7829	0.31623	-2.4757	0.0133	0.07616	3817.95	1871.26	3737.7	1831.41	1189.99	2457.36	Down
Negr1	147.474	0.63917	0.31083	2.05633	0.03975	0.16445	129.256	105.548	111.108	175.391	246.241	117.301	Up
Ptger3	580.572	1.19888	0.29541	4.05836	4.94E-05	0.00104	331.953	489.269	235.86	651.598	716.057	1058.69	Up
Lyn	7902.26	0.50955	0.08275	6.15743	7.39E-10	6.76E-08	6502.95	6544.96	6515.38	8753.23	9523.05	9573.97	Up
Penk	112.49	-1.5461	0.49533	-3.1214	0.0018	0.01727	197.801	69.0501	235.86	70.3603	70.0603	31.8105	Down
Fam110b	186.776	-0.6357	0.24396	-2.6059	0.00916	0.05801	221.302	283.105	177.382	152.957	158.666	127.242	Down
4930430E	418.548	2.13208	0.22805	9.34928	8.82E-21	5.96E-18	181.154	138.1	147.169	697.485	831.451	515.927	Up
Sdcbp	33706.3	0.49286	0.0997	4.94337	7.68E-07	3.19E-05	27265.2	29529.8	27217.4	38374.9	42789.3	37061.2	Up
Tox	95.4178	1.05253	0.24008	4.38399	1.17E-05	0.00031	59.732	67.0772	59.4523	118.287	128.787	139.171	Up
Car8	19695.1	0.33326	0.15983	2.08511	0.03706	0.15681	16622.1	21242.8	14426.4	20534	22895.3	22450.3	Up
Gdf6	86.7241	0.97185	0.48514	2.00326	0.04515	0.17836	52.8775	82.8601	39.9597	168.253	125.696	50.698	Up
Trp53inp1	7036.01	-0.3859	0.1904	-2.0268	0.04269	0.17194	8106.9	6106	9701.44	6045.89	5339.01	6916.8	Down
Ccne2	95.5513	0.65804	0.25788	2.55175	0.01072	0.06512	64.628	70.0365	87.7165	130.524	105.09	115.313	Up
Cdh17	34.5359	-0.9685	0.42566	-2.2754	0.02288	0.11158	57.7736	32.5522	46.7821	21.414	27.8181	20.8756	Down
Tmem67	1086.36	-0.5645	0.24284	-2.3245	0.0201	0.10257	1576.53	928.23	1383.97	870.837	679.997	1078.58	Down
Osgin2	1730.14	-0.2802	0.13716	-2.0426	0.04109	0.1677	2017.18	1664.11	2011.63	1597.89	1398.11	1691.92	Down
Gm11867	92.9299	-1.4118	0.64788	-2.1791	0.02932	0.13294	153.736	164.734	86.7418	82.5969	61.8179	7.95263	Down
Cpne3	7200.26	0.29163	0.08636	3.37701	0.00073	0.00861	6307.11	6796.5	6321.43	7573.42	8105.36	8097.76	Up

Slc7a13	8.88562	1.52912	0.73566	2.07856	0.03766	0.15832	4.89606	3.94572	4.87314	14.276	13.3939	11.9289	Up
Atp6v0d2	7010.85	1.80797	0.19163	9.43481	3.92E-21	2.96E-18	3486	3354.85	2503.82	10044.2	13440.2	9235.98	Up
Ggh	1978.96	0.30002	0.11216	2.67482	0.00748	0.04986	1893.8	1804.18	1623.73	2213.8	2292.41	2045.81	Up
Coq3	838.284	-0.3001	0.10732	-2.796	0.00517	0.03834	965.504	899.624	910.302	720.938	776.845	756.494	Down
Faxc	40.8077	-0.9043	0.42795	-2.1132	0.03458	0.14932	50.9191	38.4708	70.1732	20.3943	36.0604	28.8283	Down
Klhl32	406.422	-0.6052	0.28479	-2.125	0.03359	0.14635	580.673	316.644	574.055	323.25	247.272	396.637	Down
Map3k7	3588.84	0.25355	0.08416	3.01257	0.00259	0.02302	3335.2	3314.4	3173.39	3830.05	3815.2	4064.79	Up
Casp8ap2	925.983	0.29066	0.11599	2.50596	0.01221	0.07151	852.894	827.614	818.687	1019.71	936.541	1100.44	Up
Ankrd6	362.726	-0.9793	0.29121	-3.3629	0.00077	0.00895	511.149	340.318	592.573	272.264	163.817	296.235	Down
Spaca1	23.6272	-1.0247	0.49619	-2.0652	0.03891	0.16209	39.1685	25.6472	30.2134	11.2169	20.606	14.9112	Down
Cfap206	4249.86	-0.8774	0.28892	-3.0368	0.00239	0.02166	6432.45	3855.95	6223	3106.05	1858.66	4023.04	Down
Smim8	575.336	-0.3053	0.13383	-2.2809	0.02255	0.11051	615.925	621.451	670.544	569.001	478.058	497.039	Down
C9orf72	1301.63	0.34714	0.13531	2.56564	0.0103	0.06325	1118.26	1254.74	1064.29	1364.38	1641.27	1366.86	Up
Aco1	5071.61	0.387	0.09837	3.93416	8.35E-05	0.0016	4070.59	4568.16	4547.61	5443.24	5948.94	5851.15	Up
Ddx58	4511.42	0.29704	0.1293	2.29729	0.0216	0.10747	3529.08	4655.95	3960.89	4897.69	4966.04	5058.87	Up
Topors	2777.94	0.26401	0.11551	2.28554	0.02228	0.10969	2495.03	2728.46	2349.83	3079.54	3240.29	2774.47	Up
B4galt1	7175.5	0.22124	0.09757	2.26741	0.02337	0.11335	7123.77	6220.43	6534.88	7528.56	8103.3	7542.07	Up
1110017D	2304.93	-1.1647	0.32376	-3.5973	0.00032	0.00461	4306.58	1702.58	3554.47	1629.5	994.238	1642.22	Down
Dnaic1	2270.76	-1.0319	0.35355	-2.9187	0.00351	0.02889	4355.54	1475.7	3318.61	1569.34	1037.51	1867.87	Down
Enho	369.526	-0.4257	0.18361	-2.3184	0.02043	0.10378	506.253	375.83	388.876	342.624	287.453	316.117	Down
Cntfr	328.579	-1.8811	0.39938	-4.7101	2.48E-06	8.64E-05	588.507	508.998	453.202	119.307	63.8785	237.585	Down
Il11ra2	12.7433	-3.5462	1.2622	-2.8095	0.00496	0.03716	50.9191	3.94572	15.594	0	1.0303	4.97039	Down
Vcp	5243.15	0.3112	0.09646	3.22623	0.00125	0.01314	5040.99	4550.4	4448.2	5608.43	5775.85	6035.05	Up
Atp8b5	1272.37	-1.7291	0.81022	-2.1341	0.03283	0.14412	2709.48	718.121	2437.54	417.063	227.696	1124.3	Down
Fam166b	1487.34	-0.6861	0.25656	-2.6743	0.00749	0.04992	2284.5	1377.06	1842.05	1279.74	776.845	1363.88	Down
Cd72	544.066	0.86857	0.33551	2.58883	0.00963	0.06009	256.554	498.147	400.572	577.159	483.21	1048.75	Up
Gm12454	155.11	0.60958	0.24987	2.43959	0.0147	0.0823	139.048	106.534	122.803	218.219	200.908	143.147	Up
Car9	266.786	1.58155	0.3037	5.20762	1.91E-07	9.83E-06	158.632	125.277	116.955	572.06	354.423	273.372	Up
Npr2	5201.22	-0.2936	0.12798	-2.294	0.02179	0.10812	5687.27	6433.49	5065.14	5043.51	4564.22	4413.71	Down
Spag8	951.566	-0.8001	0.2573	-3.1097	0.00187	0.01778	1416.92	836.492	1373.25	803.535	503.816	775.381	Down
Hint2	1172.03	-0.3226	0.12488	-2.5833	0.00979	0.06074	1384.61	1271.51	1251.42	1162.47	985.996	976.185	Down
Melk	342.277	-0.9531	0.45936	-2.0748	0.038	0.15939	728.534	246.607	379.13	212.101	118.484	368.803	Down
Grhpr	938.401	-0.5241	0.142	-3.6908	0.00022	0.00349	1073.22	1011.09	1236.8	767.845	696.482	844.967	Down
Frmpd1	383.332	-0.3923	0.14264	-2.7505	0.00595	0.04249	463.168	428.11	414.217	341.604	304.968	347.927	Down
Exosc3	1155.88	-0.2742	0.13385	-2.0487	0.04049	0.16608	1310.19	1353.38	1132.52	1057.44	1135.39	946.363	Down
Igfbpl1	5.23416	-2.7603	1.33691	-2.0647	0.03895	NA	13.709	1.97286	11.6955	2.03943	0	1.98816	Down
Stra6l	403.063	3.2061	0.30321	10.5737	3.95E-26	4.67E-23	92.046	95.6837	48.7314	696.465	941.693	543.761	Up
Ccdc180	3425.57	-0.6553	0.25734	-2.5463	0.01089	0.06574	5036.09	2883.33	4651.9	2838.89	1890.6	3252.62	Down
Trim14	1443.53	0.20614	0.10335	1.99457	0.04609	0.18094	1274.94	1328.72	1418.08	1466.35	1583.57	1589.53	Up
Coro2a	1760.01	0.63543	0.32118	1.97842	0.04788	0.18583	1057.55	831.56	2246.52	1999.66	2236.78	2187.97	Up
Tbc1d2	1492.85	0.67619	0.19365	3.49186	0.00048	0.00625	1048.74	1256.71	1142.26	1746.77	2320.23	1442.41	Up
Tgfbr1	4485.55	0.67047	0.10636	6.30388	2.90E-10	2.89E-08	3610.36	3660.64	3113.93	5288.24	5629.55	5610.58	Up
Cavin4	56.001	-0.8556	0.38893	-2.1998	0.02782	0.12818	97.9213	47.3486	71.1478	44.8675	32.9696	41.7513	Down
Plppr1	6.21348	-2.6702	1.23923	-2.1548	0.03118	NA	7.8337	1.97286	22.4164	2.03943	1.0303	1.98816	Down
Acnat1	26.1779	-3.0307	0.7904	-3.8344	0.00013	0.00224	82.2539	19.7286	38.0105	4.07886	2.0606	10.9349	Down
Nipsnap3l	2468.88	0.24877	0.11742	2.11856	0.03413	0.14802	2358.92	2403.93	2006.76	2605.37	2846.71	2591.56	Up
4930412L(	15.5328	-4.4671	0.98882	-4.5176	6.26E-06	0.00019	27.418	33.5386	28.2642	0	0	3.97631	Down
Fsd1l	2899.42	-0.6759	0.23275	-2.9038	0.00369	0.02995	4034.36	2728.46	3936.52	2301.5	1608.3	2787.4	Down
Tal2	31.0474	1.18693	0.54402	2.18177	0.02913	0.13237	25.4595	18.7422	12.6702	56.0843	21.6363	51.6921	Up
Epb41l4b	5807.71	-0.5567	0.15885	-3.5044	0.00046	0.00601	6564.64	5929.43	8249.25	5263.77	4321.07	4518.09	Down
Pakap	110.747	-1.2949	0.28041	-4.6179	3.88E-06	0.00012	175.279	130.209	166.661	71.38	47.3937	73.5618	Down
Svep1	2741.88	0.30543	0.13866	2.20276	0.02761	0.12744	2149.37	2806.39	2402.46	2764.45	3176.41	3152.22	Up
Ecpas	4957.58	0.24881	0.09004	2.76338	0.00572	0.0413	4365.33	4502.06	4725.97	5128.15	5645.01	5378.96	Up
Susd1	331.678	0.5522	0.16491	3.34855	0.00081	0.00932	249.699	308.752	248.53	377.295	384.301	421.489	Up
Ptbp3	8898.95	0.33415	0.09822	3.40221	0.00067	0.00803	7722.07	8446.8	7450.05	9385.46	10399.8	9989.5	Up
Slc31a2	2767.28	0.43117	0.11543	3.73523	0.00019	0.00304	2410.82	2528.22	2131.51	2944.94	3265.02	3323.2	Up
Fkbp15	3754.21	0.52728	0.09119	5.78217	7.37E-09	5.58E-07	3099.21	3119.09	3008.67	4379.68	4715.68	4202.96	Up
Prpf4	1339.79	-0.2303	0.11419	-2.0167	0.04372	0.17471	1581.43	1379.03	1379.1	1176.75	1229.15	1293.3	Down
Rnf183	29.7429	-0.8807	0.43024	-2.047	0.04066	0.1665	29.3764	44.3893	41.909	18.3549	19.5757	24.852	Down
Wdr31	776.526	-0.9245	0.26475	-3.4919	0.00048	0.00625	1170.16	761.524	1119.85	621.006	353.392	633.228	Down
4933430I1	28.4104	1.51403	0.61537	2.46035	0.01388	0.07876	7.8337	26.6336	9.74627	33.6506	60.7876	31.8105	Up
Zfp618	123.807	1.13652	0.26348	4.31349	1.61E-05	0.00041	98.9005	69.0501	64.3254	158.056	158.666	193.845	Up
Gm11212	45.3376	-0.6599	0.32712	-2.0173	0.04367	0.17455	54.8359	55.2401	56.5284	30.5914	36.0604	38.7691	Down
Whrn	508.051	-0.3637	0.14253	-2.5521	0.01071	0.0651	552.276	602.708	560.411	476.207	390.483	466.223	Down
Atp6v1g1	10493.8	0.2084	0.07939	2.62487	0.00867	0.05574	9802.9	9761.71	9646.86	11660.4	11382.7	10708.2	Up
Tmem268	2436.46	0.42743	0.10946	3.90472	9.43E-05	0.00177	1985.84	2307.26	1941.46	2782.8	2765.32	2836.11	Up
Tnfsf15	281.539	1.20248	0.24126	4.98427	6.22E-07	2.70E-05	120.443	200.245	191.027	340.585	393.574	443.359	Up
8030451A	33.6695	-1.0467	0.40714	-2.5708	0.01015	0.06256	39.1685	39.4572	57.503	20.3943	21.6363	23.8579	Down
Tlr4	3034.4	0.58579	0.12426	4.71433	2.43E-06	8.53E-05	2684.02	2503.56	2092.52	3582.26	3669.92	3674.11	Up
Brinp1	197.962	0.56757	0.24609	2.3064	0.02109	0.10605	153.736	182.489	142.296	189.667	301.877	217.703	Up
2310002L(	6.03808	-4.0938	1.98938	-2.0578	0.03961	NA	27.418	0	6.82239	0	0	1.98816	Down
Ttc39b	3140.13	0.24092	0.09675	2.49003	0.01277	0.0739	2889.66	3069.77	2676.33	3321.21	3416.47	3467.35	Up
Ccdc171	2624.85	0.66254	0.24429	2.71209	0.00669	0.04607	1453.15	2707.75	1936.58	3389.53	3743.07	2518.99	Up
Sh3gl2	231.577	-0.65	0.28503	-2.2805	0.02258	0.11055	326.078	173.612	348.917	181.509	183.393	175.952	Down
Gm12551	5.23369	2.80898	1.24683	2.2529	0.02427	NA	1.95843	0.98643	0.97463	18.3549	5.15149	3.97631	Up
Dennd4c	1960.14	0.38436	0.10226	3.75869	0.00017	0.00283	1793.92	1694.69	1613.01	2231.14	2332.6	2095.52	Up
Hacd4	910.581	0.31294	0.13567	2.3066	0.02108	0.10605	712.867	868.058	855.723	929.98	1056.06	1040.8	Up
Mrpl48-ps	324.421	-0.5535	0.24398	-2.2684	0.0233	0.11313	392.664	400.49	364.511	360.979	210.181	217.703	Down
Gm49890	83.3072	-0.8518	0.33589	-2.536	0.01121	0.06728	85.1915	135.141	101.361	69.3406	41.2119	67.5973	Down
Dmrta1	16.1545	-1.4955	0.55357	-2.7016	0.0069	0.04705	21.5427	24.6607	25.3403	8.15772	9.27269	7.95263	Down
Gm12648	52.3634	1.8412	0.36947	4.98338	6.25E-07	2.70E-05	23.5011	15.7829	29.2388	71.38	93.7572	80.5204	Up
Ift74	2227.91	-0.5883	0.19188	-3.0661	0.00217	0.02006	3048.29	2151.4	2828.37	1850.78	1425.93	2062.71	Down
Mysm1	1999.38	0.24331	0.10268	2.36967	0.0178	0.0948	1786.08	1800.23	1907.35	2174.03	2017.32	2311.23	Up

Gm12708	23.1635	1.34784	0.53304	2.5286	0.01145	0.06807	10.7713	11.8372	16.5687	28.552	47.3937	23.8579 Up
Gm12695	383.744	-1.2314	0.37926	-3.2468	0.00117	0.01246	628.655	311.712	674.442	250.85	124.666	312.141 Down
L1td1	29.6733	-1.2109	0.56606	-2.1392	0.03242	0.14268	28.3972	35.5115	60.4269	27.5323	9.27269	16.8993 Down
Atg4c	1013.55	0.4767	0.11503	4.14396	3.41E-05	0.00076	820.58	899.624	822.585	1132.9	1271.39	1134.24 Up
Jak1	10188.1	0.26393	0.09936	2.65644	0.0079	0.05197	9304.48	9843.58	8628.38	10439.8	11592.9	11319.6 Up
E130102H	162.425	0.38034	0.18985	2.00339	0.04514	0.17836	155.695	135.141	132.549	187.628	189.575	173.964 Up
Leprot	5900.79	0.27219	0.11166	2.43769	0.01478	0.08264	5254.46	5574.31	5208.41	6621.01	6997.79	5748.76 Up
Lepr	3814.93	0.24112	0.11564	2.08506	0.03706	0.15681	3628.96	3195.05	3666.55	3764.79	4430.28	4203.96 Up
Insl5	93.0283	-1.0927	0.28418	-3.8452	0.00012	0.00216	119.464	113.439	147.169	49.966	53.5755	74.5559 Down
Wdr78	3046.85	-0.6632	0.23462	-2.8265	0.00471	0.0358	4276.22	2800.47	4128.52	2489.12	1698.96	2887.8 Down
Oma1	2072.61	0.25516	0.10842	2.35346	0.0186	0.09738	1883.03	2029.09	1757.25	2356.56	2290.35	2119.38 Up
Gm12718	16.5822	1.39222	0.55115	2.52602	0.01154	0.06846	8.81291	9.8643	8.77165	19.3746	27.8181	24.852 Up
Dab1	260.046	-0.8691	0.32225	-2.6969	0.007	0.04751	435.75	198.272	374.257	212.101	139.09	200.804 Down
Fyb2	289.339	1.99853	0.26647	7.49993	6.39E-14	1.33E-11	120.443	72.9958	153.991	481.305	444.059	463.241 Up
Gm12729	21.2869	-1.1582	0.50805	-2.2796	0.02263	0.11067	23.5011	32.5522	32.1627	16.3154	9.27269	13.9171 Down
Pcsk9	27.39	-1.0473	0.47652	-2.1978	0.02796	0.12865	37.2101	41.43	32.1627	12.2366	15.4545	25.846 Down
Dhcr24	1997.87	0.49304	0.14432	3.41624	0.00063	0.00775	1890.86	1456.96	1631.53	2118.97	2319.2	2569.69 Up
Acot11	242.517	0.39691	0.16325	2.43132	0.01504	0.08356	207.593	205.177	215.393	267.165	257.575	302.2 Up
Cyb5rl	855.165	-0.6515	0.12272	-5.3083	1.11E-07	6.07E-06	1059.51	1038.71	1037	701.564	591.391	702.814 Down
Hspb11	766.854	-0.4883	0.17752	-2.7507	0.00595	0.04249	1022.3	762.51	901.53	617.947	553.27	743.571 Down
Dio1	777.436	-0.8322	0.38536	-2.1596	0.03081	0.13749	1253.39	549.441	1184.17	655.677	283.332	738.6 Down
Lrp8	260.152	1.25539	0.28283	4.43874	9.05E-06	0.00026	164.508	160.788	135.473	285.52	522.361	292.259 Up
Podn	1750.47	0.46475	0.09493	4.89591	9.79E-07	3.90E-05	1419.86	1546.72	1446.35	2054.73	1957.57	2077.62 Up
Echdc2	2008.42	-0.2871	0.11742	-2.4448	0.01449	0.0815	2027.95	2480.87	2113.97	1793.68	1829.81	1804.25 Down
Shisal2a	311.806	-0.8085	0.25636	-3.154	0.00161	0.01584	382.872	332.427	475.618	286.54	161.757	231.62 Down
Rab3b	138.868	1.05848	0.25469	4.15604	3.24E-05	0.00073	93.0252	68.0636	109.158	211.081	168.969	182.91 Up
Ttc39a	4203.18	-0.4773	0.22329	-2.1377	0.03254	0.14311	5916.4	3456.45	5303.92	3615.91	2846.71	4079.7 Down
Agbl4	301.91	-1.249	0.34472	-3.6233	0.00029	0.00427	525.837	221.947	527.273	183.549	140.121	212.733 Down
Pdzk1ip1	1489.95	0.36644	0.12207	3.00187	0.00268	0.02359	1308.23	1194.57	1402.49	1578.52	1625.81	1830.1 Up
Cyp4a12a	37.6689	3.2441	0.6401	5.06814	4.02E-07	1.85E-05	6.85449	8.87787	5.84776	123.386	43.2725	37.775 Up
Cyp4b1	24933.6	-0.2905	0.10641	-2.7303	0.00633	0.04437	28017.2	26796.4	27493.3	24845.4	22007.2	20442.2 Down
Mknk1	2776.13	0.29845	0.09526	3.13312	0.00173	0.01675	2361.86	2628.83	2479.45	3004.08	2979.62	3202.92 Up
Rad54l	59.783	0.72397	0.34623	2.09103	0.03653	0.15529	30.3556	51.2943	53.6045	83.6166	75.2118	64.6151 Up
Gpbp1l1	2096.7	0.33814	0.10561	3.20187	0.00137	0.01399	1909.46	1787.41	1859.59	2236.23	2234.72	2552.79 Up
Ccdc17	6806.38	-0.9471	0.21871	-4.3305	1.49E-05	0.00038	9321.13	6948.41	10621.5	5421.82	3426.77	5098.63 Down
Akr1a1	29987.1	0.25064	0.07824	3.20342	0.00136	0.01393	27048.8	27621	27496.2	33035.7	33740.2	30980.5 Up
Ptch2	225.105	0.56599	0.25909	2.18455	0.02892	0.13185	158.632	237.73	148.143	257.988	322.483	225.656 Up
Tctex1d4	1824.11	-1.1099	0.26172	-4.241	2.23E-05	0.00054	2708.5	1723.29	3047.66	1272.6	810.845	1381.77 Down
Plk3	1282	0.61183	0.26738	2.28827	0.02212	0.10919	827.435	1274.47	940.515	1572.4	2050.29	1026.88 Up
Armh1	817.712	-0.8677	0.29795	-2.9121	0.00359	0.02939	1183.87	839.452	1146.16	604.691	334.847	797.251 Down
Med8	3350.97	0.28634	0.10495	2.72847	0.00636	0.04454	3173.63	2928.71	2956.04	3913.67	3767.8	3365.95 Up
Elovl1	57067.4	0.79959	0.18039	4.43259	9.31E-06	0.00026	39096	52944.6	32896.6	72665.9	75971.1	68830 Up
Mpl	94.9461	-1.3641	0.31067	-4.3909	1.13E-05	0.00031	114.568	112.453	183.23	57.104	54.6058	47.7158 Down
Cfap57	1407.95	-0.786	0.29354	-2.6776	0.00742	0.04954	2217.92	1090	2038.92	1142.08	719.148	1239.62 Down
Ebna1bp2	2393.3	-0.2029	0.09726	-2.0858	0.03699	0.15664	2675.21	2620.94	2387.84	2240.31	2147.14	2288.37 Down
Ccdc30	2637.5	-0.8485	0.25751	-3.2949	0.00098	0.01087	3912.93	2325.01	3936.52	2100.61	1330.12	2219.78 Down
Zmynd12	643.177	-1.0132	0.28277	-3.5831	0.00034	0.00479	1044.82	552.401	983.399	424.201	320.423	533.82 Down
Rimkla	49.3395	-1.7319	0.53804	-3.2189	0.00129	0.01339	68.5449	99.6294	59.4523	19.3746	9.27269	39.7631 Down
Foxo6	134.301	-0.6347	0.22079	-2.8747	0.00404	0.03191	143.944	176.571	169.585	119.307	100.969	95.4315 Down
Foxo6os	3.75589	-3.3535	1.69226	-1.9817	0.04751	NA	1.95843	5.91858	12.6702	0	0	1.98816 Down
Ctps	7856.09	0.70589	0.18807	3.75339	0.00017	0.00287	4911.73	7793.78	5209.38	9537.39	9402.5	10281.8 Up
Gm8439	32.4737	4.7746	0.69907	6.82991	8.50E-12	1.14E-09	2.93764	3.94572	0	56.0843	75.2118	56.6625 Up
Gm12860	32.5924	1.99519	0.44682	4.46534	7.99E-06	0.00023	16.6466	12.8236	9.74627	42.828	61.8179	51.6921 Up
Cited4	274.133	1.6893	0.25438	6.64083	3.12E-11	3.93E-09	110.651	152.897	125.727	387.492	542.967	325.064 Up
Kcnq4	251.847	0.35355	0.15501	2.2808	0.02256	0.11052	225.219	216.028	222.215	267.165	280.241	300.212 Up
Rims3	215.837	1.00415	0.33784	2.9723	0.00296	0.02541	148.84	190.381	91.615	250.85	395.635	217.703 Up
Gm12892	164.921	-5.3695	1.26545	-4.2432	2.20E-05	0.00053	717.763	232.797	15.594	10.1971	7.21209	5.96447 Down
Ppt1	5700.31	0.34832	0.10894	3.19737	0.00139	0.01415	5283.83	5205.39	4557.36	5932.7	6599.06	6623.55 Up
Cap1	17026.7	0.33445	0.109	3.06832	0.00215	0.01995	14842.9	16443.8	13899.2	18612.9	20339.1	18022.6 Up
Ndufs5	1689.89	-0.2504	0.12509	-2.002	0.04528	0.17877	2081.81	1710.47	1716.32	1611.15	1486.72	1532.87 Down
Rhbdl2	282.026	0.87127	0.32432	2.68641	0.00722	0.04866	267.325	107.521	223.19	339.565	425.513	329.04 Up
Gm12905	703.903	-0.5607	0.21555	-2.6011	0.00929	0.05851	1040.9	612.573	863.52	581.237	505.877	619.311 Down
Rragc	6063.91	0.29885	0.10254	2.91452	0.00356	0.0292	5224.1	5767.65	5322.44	6414.01	7255.36	6399.88 Up
Utp11	2168.93	-0.2757	0.11442	-2.4098	0.01596	0.08739	2548.89	2376.31	2201.68	1898.71	1859.69	2128.32 Down
Fhl3	176.013	0.62958	0.23586	2.66927	0.0076	0.05043	174.3	127.249	113.057	195.785	235.938	209.751 Up
Inpp5b	7559.1	0.31752	0.13031	2.43658	0.01483	0.08275	6727.19	7670.48	5794.16	8361.66	8714.26	8086.83 Up
Dnali1	4017	-0.833	0.26579	-3.1342	0.00172	0.01671	6092.66	3613.29	5730.81	3099.93	1935.93	3629.38 Down
Zc3h12a	1304.48	0.95133	0.15104	6.29847	3.01E-10	2.95E-08	930.252	817.75	920.048	1754.93	1447.57	1956.35 Up
Oscp1	1531.85	-0.8689	0.26659	-3.2592	0.00112	0.012	2272.75	1615.77	2050.62	1173.69	668.664	1409.6 Down
Col8a2	2077.63	-0.9261	0.22317	-4.15	3.32E-05	0.00075	2928.83	2052.76	3186.06	1461.25	1097.27	1739.64 Down
Tekt2	532.786	-0.9697	0.19637	-4.938	7.89E-07	3.25E-05	617.883	683.596	814.788	388.511	284.362	407.572 Down
Zmym1	402.046	-0.6895	0.26482	-2.6036	0.00922	0.05829	613.966	400.49	474.643	320.19	214.302	388.685 Down
Gjb3	242.196	0.91021	0.34009	2.67642	0.00744	0.04966	127.298	267.322	110.133	321.21	281.271	345.939 Up
Gjb4	55.7727	1.16762	0.41365	2.82276	0.00476	0.03604	24.4803	54.2536	24.3657	80.5575	81.3936	69.5855 Up
Azin2	2054.96	-0.6947	0.14205	-4.8904	1.01E-06	4.00E-05	2760.4	2397.02	2463.86	1690.69	1322.9	1694.9 Down
Fndc5	255.599	-1.8867	0.47621	-3.962	7.43E-05	0.00146	596.341	144.019	466.846	117.267	66.9694	142.153 Down
S100pbp	1821.62	-0.378	0.1505	-2.5118	0.01201	0.0706	2064.18	2108	2004.81	1456.15	1390.9	1905.65 Down
C77080	5805.53	0.23146	0.10936	2.11645	0.03431	0.14845	5468.9	5221.17	5332.19	6205.98	6928.76	5676.19 Up
Sync	64.0911	0.84723	0.38324	2.21068	0.02706	0.12553	46.023	64.1179	27.2896	80.5575	72.1209	94.4375 Up
Zbtb8a	504.469	0.63771	0.19391	3.28866	0.00101	0.01105	456.313	309.739	418.115	657.716	650.118	534.814 Up
Bsdc1	3978.23	0.24002	0.1004	2.39067	0.01682	0.09092	3877.68	3528.46	3537.9	4478.59	4441.62	4005.14 Up
Hdac1	2921.9	0.55316	0.14095	3.92448	8.69E-05	0.00165	2483.28	2252.02	2370.29	3858.6	2893.08	3674.11 Up
Serinc2	2553.23	0.61526	0.21984	2.79866	0.00513	0.03813	2360.88	1527.98	2161.72	3438.48	3507.14	2323.16 Up

Fabp3	582.348	-0.7459	0.37316	-1.9988	0.04563	0.1796	1052.65	307.766	828.433	396.669	423.453	485.11	Down
Sdc3	1484.15	0.55508	0.11413	4.86335	1.15E-06	4.48E-05	1121.2	1299.13	1186.12	1671.31	1746.36	1880.8	Up
Laptm5	9743.2	1.32456	0.20315	6.52	7.03E-11	7.98E-09	5865.48	6102.05	4713.3	13830.4	17516.1	10431.9	Up
Ptpru	3012.3	-0.5429	0.20055	-2.7068	0.00679	0.04656	3822.85	2689.99	4204.54	2650.24	2014.23	2691.96	Down
Srsf4	1005.67	0.3137	0.12652	2.47938	0.01316	0.07565	923.398	807.886	959.033	1041.13	1111.69	1190.91	Up
Gm12992	94.9062	0.75779	0.23911	3.16928	0.00153	0.01524	67.5657	66.0908	77.9702	120.326	110.242	127.242	Up
Rcc1	748.517	-0.3043	0.15319	-1.9863	0.047	0.18333	768.682	940.067	772.879	606.73	653.209	749.535	Down
Ptafr	376.435	0.81057	0.21443	3.78003	0.00016	0.00265	250.678	355.115	214.418	481.305	474.968	482.128	Up
Themis2	1343.38	0.85562	0.21241	4.02806	5.62E-05	0.00115	743.222	1223.17	902.505	1632.56	2036.9	1521.93	Up
Stx12	8871.58	0.24395	0.11862	2.05656	0.03973	0.1644	7924.77	9061.34	7383.78	9521.08	10284.4	9054.07	Up
Fgr	1585.78	1.11963	0.24199	4.62669	3.72E-06	0.00012	874.437	1352.39	771.905	1923.18	2689.08	1903.66	Up
Cd164l2	258.191	0.74213	0.24879	2.98288	0.00286	0.02476	171.362	244.635	163.737	283.481	283.332	402.602	Up
Sytl1	682.469	-0.4553	0.15972	-2.8507	0.00436	0.03379	761.828	728.971	877.165	579.198	494.543	653.11	Down
Wdtc1	2184.19	-0.2833	0.09716	-2.9155	0.00355	0.02912	2344.24	2523.29	2326.44	1868.12	1984.35	2058.74	Down
Nudc	5943.63	-0.4246	0.13272	-3.199	0.00138	0.01409	7747.53	5924.5	6763.91	5295.38	4710.52	5219.91	Down
Rps6ka1	4318.08	0.43716	0.11726	3.72809	0.00019	0.00311	3613.3	3455.46	3937.49	5186.27	5282.34	4433.59	Up
Crybg2	245.193	-0.6108	0.30201	-2.0224	0.04313	0.17313	348.6	209.123	331.373	179.47	141.151	261.443	Down
Cd52	1615.18	0.26208	0.12732	2.0585	0.03954	0.16384	1472.74	1560.53	1373.25	1890.55	1844.23	1549.77	Up
Ubxn11	2646.43	-0.6598	0.26605	-2.4799	0.01314	0.07561	3525.17	2286.54	3912.15	2190.35	1385.75	2578.64	Down
Sh3bgrl3	3839.37	0.6109	0.16263	3.75633	0.00017	0.00285	3166.77	2866.56	3081.77	4773.29	5465.73	3682.07	Up
Cep85	9017.66	4.5954	0.6559	7.00623	2.45E-12	3.65E-10	759.869	533.658	855.723	10860	32335.9	8760.81	Up
Grrp1	913.292	0.34556	0.16684	2.0712	0.03834	0.1604	829.393	733.904	849.875	915.704	1235.33	915.546	Up
Trim63	20.9296	-2.5766	1.13728	-2.2656	0.02348	0.11357	70.5033	3.94572	33.1373	1.01971	2.0606	14.9112	Down
Slc30a2	36.0748	-1.6321	0.39036	-4.1811	2.90E-05	0.00067	58.7528	49.3215	55.5538	20.3943	17.5151	14.9112	Down
Ncmap	166.55	1.42481	0.31269	4.55662	5.20E-06	0.00016	88.1291	62.1451	120.854	285.52	262.726	179.928	Up
Stpg1	360.147	-1.0312	0.26325	-3.9173	8.96E-05	0.0017	551.297	335.386	564.309	265.126	181.333	263.431	Down
Ifnlr1	1785.94	0.3145	0.12173	2.58349	0.00978	0.06074	1535.41	1540.8	1699.75	1987.42	2171.87	1780.39	Up
Myom3	447.266	0.65972	0.15321	4.30611	1.66E-05	0.00042	336.849	378.789	324.551	544.528	605.815	493.063	Up
Gm13000	14.7787	-1.96	0.75077	-2.6107	0.00904	0.05744	16.6466	28.6065	25.3403	3.05914	3.0909	11.9289	Down
Pnrc2	7298.29	0.46775	0.13808	3.38742	0.00071	0.00834	6808.47	5357.3	6210.33	9012.24	8937.84	7463.54	Up
Fuca1	12002.4	0.83405	0.10687	7.8045	5.97E-15	1.53E-12	8216.57	9174.78	8488.03	15961.6	16236.5	13937	Up
Gale	1492.89	0.32171	0.12645	2.54416	0.01095	0.06603	1413	1228.1	1340.11	1785.52	1713.39	1477.2	Up
Zfp46	1319.49	0.56298	0.14102	3.99219	6.55E-05	0.00131	1084.97	1024.9	1085.73	1661.12	1737.08	1323.12	Up
Ephb2	289.055	-1.382	0.38977	-3.5456	0.00039	0.00534	537.588	206.164	509.73	150.918	110.242	219.691	Down
C1qb	3417.62	0.57038	0.19613	2.90823	0.00363	0.02965	2402.99	3300.59	2548.65	3754.59	3408.23	5090.68	Up
C1qc	2757.48	0.54269	0.21422	2.53336	0.0113	0.0676	1928.07	2839.93	1966.8	3104.01	2659.2	4046.89	Up
C1qa	2955.79	0.57871	0.18728	3.08999	0.002	0.01881	2176.79	2707.75	2228	3214.14	2946.65	4461.42	Up
Wnt4	1702.68	-1.0812	0.21981	-4.9187	8.71E-07	3.54E-05	2610.58	1912.69	2414.15	973.828	864.42	1440.42	Down
Rap1gap	4976.66	0.83749	0.13447	6.22818	4.72E-10	4.42E-08	3788.57	3005.65	3919.95	6765.81	6213.73	6166.27	Up
Alpl	649.217	1.00141	0.17958	5.57639	2.46E-08	1.59E-06	501.357	409.368	386.927	941.197	716.057	940.398	Up
2310026L	39.403	-0.901	0.41193	-2.1872	0.02873	0.13118	53.8567	53.2672	46.7821	24.4732	39.1513	18.8875	Down
Kif17	118.065	-0.9438	0.30416	-3.1029	0.00192	0.01814	198.78	103.575	163.737	76.4786	79.333	86.4848	Down
Ubxn10	4312.07	-0.8271	0.28152	-2.938	0.0033	0.02756	6749.71	3708.98	6087.52	3181.51	2072.96	4071.75	Down
Pla2g5	136.929	1.13175	0.50947	2.22141	0.02632	0.12342	157.653	31.5657	68.2239	223.318	137.03	203.786	Up
Gm13030	137.295	1.37715	0.53011	2.59785	0.00938	0.05886	132.194	24.6607	72.1224	238.613	121.575	234.603	Up
Akr7a5	1812.89	-0.4396	0.11357	-3.8702	0.00011	0.00199	1997.59	2231.3	2032.1	1590.76	1402.24	1623.33	Down
Arhgef10l	1321.79	0.66823	0.18413	3.62903	0.00028	0.00419	1001.73	1223.17	838.179	1676.41	1817.45	1373.82	Up
Rcc2	4833.42	-0.299	0.09436	-3.1685	0.00153	0.01526	5433.65	5124.5	5439.39	4474.51	4023.32	4505.16	Down
Crocc	775.693	-0.6256	0.19212	-3.256	0.00113	0.01212	942.003	806.899	1075.01	680.15	479.089	671.003	Down
Arhgef19	1016.46	-0.6129	0.18969	-3.2309	0.00123	0.01302	1239.68	1114.67	1333.29	915.704	603.755	891.688	Down
Gm13056	140.54	-0.81	0.24522	-3.3031	0.00096	0.01061	186.05	167.693	183.23	128.484	80.3633	97.4197	Down
Gm13074	115.627	1.5644	0.46154	3.38953	0.0007	0.00831	55.8151	50.3079	69.1985	203.943	66.9694	247.526	Up
Fam131c	150.759	-1.4918	0.30185	-4.9423	7.72E-07	3.20E-05	216.406	215.042	235.86	112.169	51.5149	73.5618	Down
Fblim1	3125.14	0.23792	0.10501	2.26579	0.02346	0.11357	2631.14	3054.97	2918.03	3548.61	3235.14	3362.97	Up
Tmem82	160.025	0.49168	0.22193	2.21547	0.02673	0.12472	128.277	149.937	120.854	221.278	164.848	174.958	Up
Plekhm2	2973.75	0.37616	0.12314	3.05469	0.00225	0.02072	2539.1	2822.18	2403.43	3255.95	3718.35	3103.51	Up
Casp9	937.01	0.41715	0.12404	3.3631	0.00077	0.00895	810.788	828.601	768.006	985.045	1184.84	1044.78	Up
Efhd2	6876.38	0.48931	0.10406	4.70238	2.57E-06	8.89E-05	5325.94	5871.23	5966.67	8011.9	8606.08	7476.46	Up
Fhad1os2	954.049	-0.9796	0.24457	-4.0053	6.19E-05	0.00124	1442.38	906.529	1449.27	773.964	481.149	671.003	Down
Fhad1	3060	-0.6784	0.30395	-2.232	0.02561	0.12092	4261.53	2388.15	4649.95	2475.87	1492.9	3091.58	Down
Tmem51	1360.17	0.27245	0.13354	2.04023	0.04133	0.16839	1225	1265.59	1205.61	1415.36	1713.39	1336.04	Up
Gm13080	4.54561	2.57451	1.16488	2.21011	0.0271	NA	1.95843	0.98643	0.97463	5.09857	10.303	7.95263	Up
Tnfrsf1b	3104.49	0.53909	0.17241	3.12686	0.00177	0.01703	2319.76	3174.33	2099.35	3555.75	3883.19	3594.59	Up
Tnfrsf8	110.727	1.19667	0.33507	3.57142	0.00036	0.00494	47.9814	69.0501	84.7926	179.47	106.121	176.946	Up
Gm13230	7.7722	3.44526	1.0706	3.21805	0.00129	0.01339	1.95843	0.98643	0.97463	22.4337	11.3333	8.94671	Up
Gm13212	133.605	0.79361	0.32151	2.46841	0.01357	0.07743	62.6696	132.182	98.4374	137.662	194.726	175.952	Up
Zfp992	201.497	1.15643	0.3762	3.07396	0.00211	0.01965	86.1707	139.087	149.118	173.352	429.634	231.62	Up
Zfp981	39.2642	2.24414	1.01528	2.21037	0.02708	0.12554	23.5011	0	17.5433	43.8477	129.818	20.8756	Up
Zfp993	13.5257	2.86115	1.08627	2.63391	0.00844	0.05469	5.87528	3.94572	0	6.11829	45.3331	19.8816	Up
Zfp989	1.87755	4.37919	2.22186	1.97096	0.04873	NA	0	0	0	3.05914	7.21209	0.99408	Up
Zfp991	367.943	0.65569	0.20116	3.25954	0.00112	0.012	256.554	272.255	328.449	504.759	358.544	487.098	Up
Zfp534	3.23847	5.16645	2.43214	2.12424	0.03365	NA	0	0	0	0	15.4545	3.97631	Up
Plod1	2802.25	0.39548	0.18818	2.10162	0.03559	0.15239	2670.31	2342.77	2248.47	2830.73	4079.98	2641.27	Up
Nppb	10.3631	-1.8884	0.915	-2.0638	0.03904	0.16243	24.4803	7.89144	16.5687	1.01971	8.24239	3.97631	Down
Nppa	88.4571	-6.4414	1.83765	-3.5052	0.00046	0.006	489.606	1.97286	33.1373	1.01971	1.0303	3.97631	Down
Fbxo44	816.7	-0.3252	0.16064	-2.0246	0.04291	0.17249	772.599	1012.08	940.515	771.924	634.664	768.423	Down
Masp2	117.099	-0.8319	0.21925	-3.7944	0.00015	0.00254	136.111	153.883	159.839	88.7152	85.5148	78.5322	Down
Gm572	17.8679	-2.9475	1.0783	-2.7335	0.00627	0.04412	47.0022	8.87787	38.9851	2.03943	10.303	0	Down
Cort	34.0279	-1.1651	0.41532	-2.8054	0.00503	0.03753	35.2517	56.2265	49.706	23.4534	21.6363	17.8934	Down
Cenps	102.787	-0.5506	0.24028	-2.2914	0.02194	0.10854	114.568	127.249	124.752	85.6561	70.0603	94.4375	Down
Pik3cd	1754.01	0.45194	0.21462	2.10579	0.03522	0.15124	1163.3	2009.36	1271.89	1956.83	2156.41	1966.29	Up
Tmem201	1065.73	-0.2182	0.1056	-2.0665	0.03878	0.16178	1086.93	1212.32	1139.34	949.355	1001.45	1005.01	Down
Gm16188	255.183	-1.2545	0.31106	-4.0329	5.51E-05	0.00114	332.932	281.132	464.897	178.45	93.7572	179.928	Down

Spsb1	1556.5	-0.5849	0.22812	-2.5638	0.01035	0.06351	2339.34	1297.15	1966.8	1215.5	1080.78	1439.43	Down
H6pd	9746.54	0.52914	0.13927	3.79944	0.00015	0.0025	6910.3	9184.65	7841.85	11781.8	12250.2	10510.4	Up
Slc2a5	33.4066	-2.9215	0.79479	-3.6758	0.00024	0.00366	110.651	10.8507	55.5538	9.17743	8.24239	5.96447	Down
Eno1	6594.77	0.41628	0.12989	3.20487	0.00135	0.01388	5722.52	5574.31	5652.84	8551.33	6431.12	7636.51	Up
Thap3	1882.97	-0.2765	0.10685	-2.588	0.00965	0.06019	1904.57	2081.37	2202.66	1787.56	1652.6	1669.06	Down
Tnfrsf25	386.244	-0.579	0.19471	-2.9738	0.00294	0.02531	457.292	513.93	417.14	301.836	254.484	372.779	Down
Espn	595.18	-0.6838	0.15725	-4.3484	1.37E-05	0.00036	773.578	610.6	816.738	453.773	448.18	468.211	Down
Hes2	44.3356	-1.0057	0.38113	-2.6388	0.00832	0.05407	50.9191	74.9687	51.6552	28.552	36.0604	23.8579	Down
Gpr153	1277.22	0.55562	0.21358	2.60144	0.00928	0.05851	1064.4	1257.7	780.676	1596.87	1733.99	1229.68	Up
Rnf207	39.9298	-1.6262	0.5672	-2.867	0.00414	0.03243	60.7112	33.5386	86.7418	25.4929	8.24239	24.852	Down
Chd5	135.116	0.99679	0.26431	3.77131	0.00016	0.00271	97.9213	94.6972	77.9702	170.292	226.666	143.147	Up
Nphp4	728.321	-1.0149	0.29274	-3.4668	0.00053	0.00672	1096.72	584.953	1241.68	434.399	401.816	610.364	Down
Cep104	3576.97	-0.2839	0.11938	-2.3783	0.01739	0.09324	4190.05	3681.36	3912.15	3279.4	2882.78	3516.06	Down
Trp73	1272.68	-0.8679	0.28456	-3.0499	0.00229	0.02097	1698.93	1253.75	1980.44	1024.81	540.907	1137.23	Down
Prdm16os	249.018	-0.6007	0.28814	-2.0849	0.03708	0.15684	298.66	288.037	313.83	163.154	141.151	289.277	Down
Gm27202	64.7796	-1.4439	0.35031	-4.1217	3.76E-05	0.00082	108.693	88.7787	86.7418	47.9266	24.7272	31.8105	Down
Gm13110	168.385	-0.9892	0.29732	-3.3271	0.00088	0.00988	165.487	201.232	305.058	111.149	133.939	93.4434	Down
Ttc34	259.426	-0.9932	0.46061	-2.1564	0.03105	0.13824	356.433	196.299	483.415	198.844	70.0603	251.502	Down
Mmel1	58.1143	-1.1573	0.59012	-1.9612	0.04986	0.19052	112.609	42.4165	85.7672	44.8675	11.3333	51.6921	Down
Prxl2b	305.419	-0.3253	0.14616	-2.2256	0.02604	0.12234	332.932	331.44	354.764	285.52	254.484	273.372	Down
Morn1	1249.49	-0.7962	0.21934	-3.6299	0.00028	0.00419	1840.92	1175.82	1740.68	960.571	722.239	1056.71	Down
Cfap74	2295.04	-0.9609	0.24782	-3.8776	0.00011	0.00195	3446.83	2225.39	3424.84	1738.61	1076.66	1857.93	Down
Tmem52	102.035	-1.3729	0.28612	-4.7983	1.60E-06	5.91E-05	149.82	114.426	177.382	49.966	65.9391	54.6743	Down
Nadk	10792.9	0.22707	0.10864	2.09004	0.03661	0.15559	9885.15	10919.8	9030.9	11114.9	12082.3	11724.2	Up
Tmem88b	96.1002	-0.973	0.25892	-3.7581	0.00017	0.00284	118.485	114.426	149.118	68.3209	56.6664	69.5855	Down
Ankrd65	1709.2	-1.1222	0.26421	-4.2475	2.16E-05	0.00052	2482.3	1729.21	2815.7	1142.08	716.057	1369.84	Down
Cptp	1251.9	0.26715	0.09551	2.7971	0.00516	0.03826	1093.78	1174.84	1140.31	1388.85	1379.57	1334.05	Up
Pusl1	550.286	0.26328	0.12387	2.12541	0.03355	0.14631	505.274	503.079	492.187	612.849	633.634	554.696	Up
Acap3	1554.74	0.43833	0.15554	2.81819	0.00483	0.03637	1133.93	1272.49	1554.53	1899.73	1849.39	1618.36	Up
Ttll10	615.114	-0.3617	0.17573	-2.058	0.03959	0.16397	763.786	558.319	753.387	531.271	491.452	592.471	Down
Agrn	15557.7	0.29602	0.13281	2.22888	0.02582	0.1215	11887.6	15495.8	14518	17620.7	17948.8	15875.4	Up
Cdk6	2346.22	0.25556	0.10661	2.39727	0.01652	0.08972	2013.26	2292.46	2111.04	2545.21	2708.65	2406.66	Up
Gatad1	3970.96	-0.2233	0.0814	-2.7437	0.00608	0.0431	4431.92	4238.69	4162.63	3587.36	3655.5	3749.66	Down
Akap9	4999.6	-0.3416	0.15407	-2.2169	0.02663	0.12441	6173.94	4635.23	5956.92	4415.37	3920.29	4895.84	Down
Cyp51	6281.05	0.43373	0.09618	4.50963	6.49E-06	0.00019	5454.22	5617.72	4959.88	7028.89	7570.63	7054.98	Up
Cdk14	4604.68	0.24468	0.10408	2.3508	0.01873	0.09789	3880.62	4395.53	4369.25	5333.11	4808.4	4841.16	Up
Cldn12	2833.41	0.54177	0.09227	5.87149	4.32E-09	3.45E-07	2340.32	2172.12	2410.25	3256.97	3377.32	3443.49	Up
Cfap69	5077.18	-0.6618	0.2765	-2.3933	0.0167	0.09047	7072.85	4228.82	7363.31	4229.78	2585.02	4983.32	Down
Steap4	2583.26	0.708	0.26824	2.6394	0.00831	0.054	2643.87	1877.18	1364.48	2724.68	2668.47	4220.86	Up
Gm15731	11.5329	-1.5895	0.74517	-2.133	0.03292	0.14425	21.5427	14.7964	15.594	10.1971	3.0909	3.97631	Down
Adam22	1458.57	-0.3634	0.13211	-2.7504	0.00595	0.04249	1766.5	1432.3	1725.09	1186.95	1280.66	1359.9	Down
Abcb1a	1876.55	0.48113	0.13075	3.67978	0.00023	0.00362	1605.91	1632.54	1460.97	2272.94	2389.26	1897.7	Up
Crot	3002.76	0.38516	0.11946	3.22415	0.00126	0.01321	2694.79	2310.22	2807.9	3426.24	3188.77	3588.62	Up
Tmem243	8953.86	0.26385	0.10544	2.50227	0.01234	0.07199	8010.94	8903.51	7497.81	9916.73	9295.35	10098.8	Up
Hgf	468.702	0.45181	0.21411	2.11016	0.03484	0.15003	375.038	479.405	333.323	427.261	602.725	594.459	Up
Sema3c	28089.5	0.36017	0.12697	2.83672	0.00456	0.03497	21921.6	27118.9	24763.3	28765.1	34601.5	31366.2	Up
Gm43032	35.2052	-0.8611	0.3729	-2.3092	0.02093	0.10549	48.9606	46.3622	40.9343	24.4732	22.6666	27.8342	Down
Gm29254	4.91438	-3.7619	1.3784	-2.7292	0.00635	NA	8.81291	11.8372	6.82239	1.01971	0	0.99408	Down
Ptpn12	8157.17	0.61416	0.11645	5.27425	1.33E-07	7.12E-06	6641.02	6547.92	6150.87	9593.48	11081.9	8927.82	Up
Gsap	10160.4	0.57134	0.1392	4.10449	4.05E-05	0.00088	7360.74	9560.48	7602.09	11432	13208.4	11798.7	Up
Fbxl13	1409.07	-1.0516	0.33001	-3.1867	0.00144	0.0145	2296.25	1193.58	2213.38	1033.99	522.361	1194.88	Down
Armc10	3467.64	0.44166	0.11307	3.90624	9.37E-05	0.00176	2945.47	3129.94	2747.47	3689.33	4356.1	3937.55	Up
Napepld	1663.57	-0.5149	0.24241	-2.1241	0.03366	0.14653	2038.72	1480.63	2352.75	1486.74	967.45	1655.14	Down
5031425E	2284.87	-0.202	0.08332	-2.4249	0.01531	0.08473	2428.45	2442.4	2462.88	2087.36	2196.6	2091.54	Down
AI506816	1217.49	0.67111	0.24945	2.6904	0.00714	0.04822	800.996	682.609	1334.26	1349.08	1658.78	1479.19	Up
Pus7	944.805	-0.4827	0.13664	-3.5326	0.00041	0.00556	1130.99	1057.45	1115.95	708.702	752.118	903.617	Down
Rint1	601.009	0.43616	0.1204	3.6227	0.00029	0.00427	495.482	496.174	540.918	692.386	659.391	721.701	Up
2700038G	76.1065	-0.8984	0.32594	-2.7564	0.00584	0.04194	119.464	109.494	68.2239	54.0449	57.6967	47.7158	Down
Fam126a	2765.02	-0.4011	0.09524	-4.2108	2.54E-05	0.0006	3140.34	3141.78	3158.77	2376.96	2226.47	2545.84	Down
Klhl7	2804.87	-0.2322	0.11808	-1.9669	0.04919	0.18905	3177.55	2767.92	3145.12	2583.96	2358.35	2796.34	Down
Gm5575	5.0608	-4.7499	1.59082	-2.9858	0.00283	NA	7.8337	4.93215	16.5687	0	1.0303	0	Down
Kcnh2	575.502	-0.52	0.12222	-4.2543	2.10E-05	0.00051	667.823	649.071	717.326	493.542	473.937	451.312	Down
Nos3	231.917	-0.3277	0.16208	-2.0217	0.0432	0.17339	268.304	266.336	239.758	207.002	213.272	196.828	Down
Slc4a2	2333.07	0.32904	0.12364	2.66132	0.00778	0.05136	2311.92	1870.27	2022.35	2679.81	2419.14	2694.95	Up
Tmub1	617.703	-0.2944	0.1278	-2.3037	0.02124	0.10646	636.488	696.419	708.554	594.494	555.331	514.933	Down
Asb10	11.834	-1.6489	0.72749	-2.2665	0.02342	0.1135	23.5011	12.8236	17.5433	5.09857	3.0909	8.94671	Down
Iqca1l	6.71169	-4.234	1.33937	-3.1612	0.00157	NA	11.7506	13.81	12.6702	2.03943	0	0	Down
Chpf2	850.98	0.42353	0.12103	3.49927	0.00047	0.0061	693.283	751.659	735.844	1042.15	994.238	888.706	Up
Dpp6	307.406	-1.8924	0.3861	-4.9013	9.52E-07	3.82E-05	656.073	226.879	570.157	126.445	97.8784	167.005	Down
Paxip1	2968.17	-0.4042	0.16991	-2.3792	0.01735	0.0931	3552.58	2686.05	3905.33	2635.96	2263.57	2765.53	Down
Gm5551	3.21955	4.12042	1.72943	2.38254	0.01719	NA	0	0.98643	0	10.1971	5.15149	2.98224	Up
Gm26608	14.5028	-1.2841	0.59322	-2.1647	0.03041	0.13641	22.5219	15.7829	23.3911	7.138	8.24239	9.94079	Down
Insig1	8592.06	0.40627	0.15716	2.58517	0.00973	0.06054	7348.01	8174.54	6647.93	9589.4	11547.6	8244.89	Up
Cnpy1	545.045	-0.4537	0.22932	-1.9785	0.04788	0.18583	553.255	634.274	702.706	550.646	320.423	508.968	Down
Rnf32	1336.41	-0.5717	0.20232	-2.8255	0.00472	0.03585	1585.35	1269.53	1938.53	1121.69	873.693	1229.68	Down
Drc1	2793.54	-1.0984	0.28002	-3.9227	8.76E-05	0.00166	4450.52	2723.53	4251.32	1952.75	1109.63	2273.46	Down
Fam166c	1116.01	-0.977	0.24829	-3.9349	8.32E-05	0.00159	1633.33	1065.34	1741.66	870.837	540.907	843.973	Down
Cib4	44.9982	-1.3261	0.39706	-3.3399	0.00084	0.00956	86.1707	53.2672	53.6045	22.4337	23.6969	30.8164	Down
Gm9899	114.054	-0.9463	0.31352	-3.0184	0.00254	0.02268	196.822	111.467	142.296	68.3209	67.9997	97.4197	Down
Kcnk3	1159.71	-0.4113	0.14504	-2.836	0.00457	0.03503	1348.38	1259.67	1363.5	1072.74	1084.9	829.061	Down
Slc35f6	1869.11	0.39162	0.10886	3.59735	0.00032	0.00461	1582.41	1667.07	1601.31	2096.53	2311.99	1955.35	Up
Dpysl5	299.28	-0.5265	0.20352	-2.587	0.00968	0.06033	292.785	419.233	347.942	216.18	244.181	275.36	Down
Mapre3	4824.67	-0.378	0.12277	-3.0787	0.00208	0.01936	5281.87	5184.67	5892.6	4713.12	4033.62	3842.11	Down

Agbl5	1512.92	-0.4185	0.20547	-2.0368	0.04167	0.16936	1870.3	1366.21	1956.08	1362.34	1007.63	1514.98	Down
Tcf23	220.159	0.85298	0.28937	2.94767	0.0032	0.0269	132.194	186.435	152.042	319.171	347.211	183.905	Up
Slc5a6	526.021	0.2773	0.13401	2.06918	0.03853	0.16098	491.565	449.812	485.364	593.474	613.028	522.885	Up
Trim54	18.29	-3.1533	1.30357	-2.419	0.01556	0.08588	48.9606	0	49.706	4.07886	1.0303	5.96447	Down
Eif2b4	1798.66	-0.2087	0.08844	-2.36	0.01828	0.09622	1928.07	1865.34	1992.14	1680.49	1688.66	1637.25	Down
Ift172	4674.42	-0.5428	0.20003	-2.7135	0.00666	0.04594	6146.52	4152.87	6331.18	3966.69	3129.02	4320.27	Down
Fndc4	271.243	-0.5571	0.19357	-2.8782	0.004	0.03169	314.327	379.775	274.845	241.672	207.09	209.751	Down
Gm20671	148.671	-0.4195	0.21036	-1.994	0.04615	0.18107	166.466	148.951	194.925	131.543	130.848	119.289	Down
Slc5a1	1716.63	1.70493	0.18191	9.37237	7.09E-21	4.97E-18	787.287	895.678	734.869	2372.88	2230.6	3278.47	Up
Fam53a	1801.02	-0.2431	0.11205	-2.1698	0.03002	0.13517	1788.04	1959.05	2110.07	1622.37	1737.08	1589.53	Down
Gm9903	14.5185	-1.2826	0.61997	-2.0688	0.03856	0.16109	29.3764	15.7829	16.5687	8.15772	9.27269	7.95263	Down
Fgfr3	7469.58	-0.3581	0.18268	-1.9603	0.04996	0.19078	8823.69	6498.6	9853.48	7184.91	5598.64	6858.15	Down
Gm1673	116.507	-0.8134	0.29582	-2.7497	0.00597	0.04253	138.069	118.372	189.078	104.011	67.9997	81.5144	Down
Nat8l	236.857	-1.6756	0.20377	-8.2232	1.98E-16	6.82E-14	391.685	294.942	395.699	120.326	107.151	111.337	Down
Poln	740.658	-0.7031	0.24612	-2.8566	0.00428	0.03335	976.275	648.084	1128.62	621.006	433.756	636.21	Down
Gm43458	15.4767	-1.4126	0.66385	-2.1279	0.03335	0.14575	25.4595	10.8507	31.1881	10.1971	7.21209	7.95263	Down
Tnip2	1771.94	0.24308	0.11595	2.09635	0.03605	0.154	1615.7	1619.72	1633.48	1816.11	2152.29	1794.31	Up
Sh3bp2	1213.76	0.99926	0.20387	4.90137	9.52E-07	3.82E-05	755.952	968.674	703.681	1584.64	1982.29	1287.33	Up
Grk4	732.474	-0.4144	0.19039	-2.1767	0.0295	0.13353	811.767	686.555	1012.64	598.573	564.604	720.707	Down
Htt	1150.29	0.32076	0.14313	2.24109	0.02502	0.11887	1003.69	1056.47	1008.74	1070.7	1345.57	1416.56	Up
Hgfac	985.786	0.77417	0.16092	4.811	1.50E-06	5.68E-05	615.925	757.578	808.941	1317.47	1079.75	1335.05	Up
Dok7	135.623	-0.9695	0.29657	-3.2691	0.00108	0.0117	215.427	142.046	181.281	87.6955	67.9997	119.289	Down
Acox3	3895.83	0.58291	0.11537	5.05249	4.36E-07	1.99E-05	2975.83	3356.82	3025.24	4517.34	5155.61	4344.12	Up
Htra3	644.224	-0.8396	0.24429	-3.437	0.00059	0.00732	1093.78	725.026	660.797	528.212	498.664	358.862	Down
Sh3tc1	2441.63	0.35692	0.15834	2.2542	0.02418	0.11618	2041.66	2421.68	1959.98	2635.96	3218.65	2371.87	Up
Ablim2	392.57	-0.381	0.15545	-2.4509	0.01425	0.08044	424.978	482.364	424.937	307.954	380.18	335.004	Down
Afap1	1785.95	0.41578	0.16607	2.50362	0.01229	0.07181	1519.74	1819.96	1251.42	1926.24	2243.99	1954.36	Up
Sorcs2	5415.53	0.28123	0.14233	1.97589	0.04817	0.18643	4377.08	4640.16	5650.89	5646.16	5554.34	6624.54	Up
Ccdc96	1114.21	-0.7885	0.28224	-2.7936	0.00521	0.03858	1762.58	962.755	1508.72	825.969	562.543	1062.67	Down
Tbc1d14	3858.16	0.32988	0.11781	2.80015	0.00511	0.03799	3580	3650.78	3026.22	4020.74	4469.43	4401.78	Up
Man2b2	5884.62	0.44432	0.07924	5.60747	2.05E-08	1.38E-06	4942.09	4829.56	5185.02	6824.95	6665	6861.13	Up
Ppp2r2c	977.273	-1.0053	0.3084	-3.2597	0.00112	0.012	1508.97	864.112	1540.89	667.913	406.968	874.789	Down
Crmp1	30.7477	0.82126	0.40774	2.01416	0.04399	0.17537	18.605	25.6472	22.4164	44.8675	39.1513	33.7987	Up
Stx18	1251.17	-0.2066	0.10319	-2.0019	0.0453	0.17881	1409.09	1283.34	1329.39	1117.61	1149.81	1217.75	Down
Otop1	6.85307	-6.1986	1.85369	-3.3439	0.00083	NA	34.2724	1.97286	4.87314	0	0	0	Down
Wdr1	10561.7	0.38469	0.08974	4.28664	1.81E-05	0.00045	9080.24	9555.54	8849.62	11419.8	12680.9	11783.8	Up
Clnk	74.1434	-0.8599	0.41489	-2.0726	0.03821	0.15999	125.339	57.2129	104.285	33.6506	59.7573	64.6151	Down
Gm40293	19.8032	-2.3851	1.10812	-2.1524	0.03137	0.13907	68.5449	1.97286	29.2388	4.07886	2.0606	12.923	Down
Gm42528	487.166	0.53545	0.19351	2.76713	0.00566	0.04091	340.766	466.581	385.952	551.666	679.997	498.033	Up
Cc2d2a	1953.03	-0.4251	0.18916	-2.2473	0.02462	0.11743	2337.38	1859.42	2519.41	1754.93	1311.57	1935.47	Down
Fbxl5	9775.04	0.37377	0.09901	3.77511	0.00016	0.00268	8869.71	8789.09	7888.63	11103.7	11599.1	10400	Up
Bst1	3068.84	1.55453	0.21403	7.26322	3.78E-13	6.75E-11	1755.73	1773.6	1147.14	4400.07	5546.1	3790.42	Up
Fgfbp1	686.512	-0.5495	0.25887	-2.1227	0.03378	0.14689	796.1	655.976	995.094	621.006	371.938	678.956	Down
Gm42984	282.716	-0.5502	0.17599	-3.1261	0.00177	0.01705	346.641	288.037	373.282	231.475	214.302	242.555	Down
Slit2	2700.21	0.46467	0.15186	3.05985	0.00221	0.02043	1848.75	2572.61	2385.89	2957.17	3342.29	3094.57	Up
5730480H	503.07	-0.3268	0.13638	-2.3966	0.01655	0.08982	528.775	537.604	613.041	473.148	424.483	441.371	Down
Adgra3	3480.63	-0.2597	0.08989	-2.8895	0.00386	0.03096	3801.3	3672.48	3905.33	3075.46	3350.53	3078.66	Down
Ppargc1a	268.048	-0.7002	0.28996	-2.4149	0.01574	0.08644	340.766	237.73	417.14	192.726	153.514	266.413	Down
Pi4k2b	17310	0.2793	0.11224	2.4885	0.01283	0.0742	16215.8	16108.4	14595	19139	17108.1	20693.7	Up
5033403H	86.5403	-0.9175	0.42463	-2.1608	0.03071	0.13723	120.443	150.924	68.2239	49.966	41.2119	88.473	Down
Slc34a2	203865	0.46193	0.19025	2.42806	0.01518	0.08407	181862	208195	124454	226529	253234	228915	Up
Sel1l3	1139.82	0.44523	0.1738	2.5617	0.01042	0.06377	1032.09	1009.12	854.748	1063.56	1542.36	1337.04	Up
Cckar	4781.7	-1.0911	0.29689	-3.6751	0.00024	0.00367	5754.83	5117.6	8652.74	3631.2	1775.2	3758.61	Down
Tbc1d19	3218.9	-0.4902	0.14524	-3.3753	0.00074	0.00864	3883.56	3331.17	4067.12	2885.79	2273.87	2871.89	Down
Gm43266	28.5573	-1.1282	0.47572	-2.3716	0.01771	0.09442	29.3764	38.4708	49.706	12.2366	22.6666	18.8875	Down
Gm9954	43.6924	2.68418	0.54649	4.91171	9.03E-07	3.66E-05	3.91685	15.7829	15.594	59.1435	105.09	62.6269	Up
Nwd2	547.541	1.95959	0.24441	8.01752	1.08E-15	3.40E-13	201.718	190.381	279.718	793.338	1129.21	690.885	Up
0610040J(	2555.1	0.37305	0.12657	2.94728	0.00321	0.02692	2414.74	1962.01	2303.04	2952.07	2648.9	3049.83	Up
Tlr6	151.419	0.67365	0.27698	2.43216	0.01501	0.08347	118.485	129.222	102.336	156.016	250.363	152.094	Up
Fam114a1	2100.15	-0.2431	0.08432	-2.8831	0.00394	0.03133	2267.86	2274.71	2287.45	1867.1	1980.23	1923.54	Down
Tmem156	277.669	0.64071	0.19129	3.34935	0.00081	0.00931	214.448	234.77	201.748	308.974	403.877	302.2	Up
Wdr19	3186.14	-0.9884	0.20595	-4.7993	1.59E-06	5.90E-05	4497.52	3326.24	4886.78	2362.68	1631.99	2411.63	Down
Klb	19.0555	-1.6782	0.78348	-2.142	0.03219	0.14204	53.8567	12.8236	20.4672	5.09857	6.18179	15.9053	Down
Chrna9	124.112	-1.1248	0.28264	-3.9797	6.90E-05	0.00137	125.339	185.449	199.799	87.6955	63.8785	82.5085	Down
9130230L:	1266.96	-0.9531	0.29625	-3.2171	0.00129	0.01343	1446.3	1322.8	2243.59	1066.62	524.422	998.055	Down
Nsun7	622.447	-0.8056	0.14849	-5.4253	5.78E-08	3.47E-06	890.104	753.632	731.945	425.221	433.756	500.022	Down
Uchl1	317.203	-0.8628	0.21406	-4.0307	5.56E-05	0.00114	310.41	438.961	478.542	226.377	243.15	205.774	Down
Gm15477	34.0321	-1.0038	0.41425	-2.4232	0.01538	0.08497	56.7943	43.4029	36.0612	18.3549	24.7272	24.852	Down
Kctd8	359.586	-0.6764	0.23498	-2.8784	0.004	0.03169	539.546	404.436	383.029	235.554	353.392	241.561	Down
Yipf7	16.5284	-2.475	0.88835	-2.7861	0.00533	0.03917	39.1685	6.90501	38.0105	4.07886	2.0606	8.94671	Down
Corin	175.993	-0.628	0.31372	-2.0016	0.04532	0.17885	232.073	135.141	273.87	108.09	158.666	148.118	Down
Tec	875.407	0.53888	0.13997	3.85007	0.00012	0.00213	656.073	808.872	676.391	1038.07	1113.75	959.286	Up
Ociad2	1537.98	-0.818	0.24586	-3.3271	0.00088	0.00988	1963.32	2525.26	1399.56	1162.47	854.117	1323.12	Down
Sgcb	1972.45	-0.3064	0.11932	-2.5679	0.01023	0.06299	2270.79	1932.42	2340.08	1845.68	1760.78	1684.96	Down
Spata18	1139.04	-0.7514	0.35193	-2.1351	0.03275	0.14384	1692.08	897.651	1697.8	770.904	491.452	1284.35	Down
Lnx1	333.639	-0.704	0.34296	-2.0526	0.04011	0.1653	401.477	312.698	526.299	277.362	138.06	345.939	Down
Gm6116	52.9703	1.65693	0.40439	4.09733	4.18E-05	0.0009	23.5011	31.5657	21.4418	94.8335	94.7875	51.6921	Up
Cep135	947.479	-0.4258	0.16258	-2.6188	0.00882	0.05645	1135.89	923.298	1199.77	794.358	715.027	916.54	Down
C530008N	626.468	1.18902	0.24674	4.81884	1.44E-06	5.49E-05	455.334	372.87	317.728	747.451	1174.54	690.885	Up
Thegl	139.486	-1.2048	0.27843	-4.3269	1.51E-05	0.00039	228.157	149.937	205.646	95.8532	63.8785	93.4434	Down
Stap1	400.09	0.48435	0.24523	1.97512	0.04826	0.18666	246.762	453.758	300.185	421.142	495.574	483.122	Up
Tmprss11(	2.84572	4.98188	1.95582	2.54721	0.01086	NA	0	0	0	4.07886	2.0606	10.9349	Up
Ugt2a3	7.03403	-2.5793	1.07881	-2.3909	0.01681	NA	9.79213	5.91858	20.4672	1.01971	1.0303	3.97631	Down

Sult1b1	29.3146	-2.2163	1.11719	-1.9839	0.04727	0.18417	106.734	1.97286	36.0612	9.17743	2.0606	19.8816 Down
Sult1d1	1910.72	-2.074	0.96098	-2.1582	0.03091	0.13777	4621.88	985.443	3656.8	542.488	186.484	1471.24 Down
Rufy3	1473.12	0.25218	0.0973	2.59187	0.00955	0.05972	1413	1333.65	1287.48	1637.66	1548.54	1618.36 Up
Dck	952.431	0.46243	0.10465	4.41868	9.93E-06	0.00028	754.973	829.587	818.687	1079.88	1124.06	1107.4 Up
Slc4a4	1393.81	-0.581	0.182	-3.1923	0.00141	0.01428	1731.25	1595.06	1686.11	964.65	961.268	1424.51 Down
Alb	74.2416	-0.9489	0.38159	-2.4867	0.01289	0.07441	78.337	142.046	73.097	38.7492	53.5755	59.6447 Down
Ppbp	732.929	-0.7341	0.25914	-2.833	0.00461	0.03526	633.551	882.855	1229.98	489.463	668.664	493.063 Down
Cxcl3	190.934	2.56416	0.39591	6.47659	9.38E-11	1.01E-08	68.5449	58.1993	38.9851	253.909	206.06	519.903 Up
Cxcl1	401.589	1.50471	0.18606	8.08708	6.11E-16	1.96E-13	188.009	182.489	257.302	640.381	569.755	571.595 Up
Cxcl2	303.243	1.05235	0.39013	2.6974	0.00699	0.04746	100.859	311.712	179.331	428.28	509.998	289.277 Up
Epgn	25.9872	1.43002	0.50628	2.82455	0.00473	0.03591	10.7713	21.7015	9.74627	33.6506	43.2725	36.7809 Up
Ereg	104.682	0.96515	0.31553	3.0588	0.00222	0.02047	60.7112	76.9415	75.0463	129.504	186.484	99.4079 Up
Areg	2684.03	-1.1896	0.14259	-8.3427	7.26E-17	2.64E-14	3487.96	4352.13	3355.64	1535.69	1777.26	1595.5 Down
Btc	414.788	0.30975	0.15453	2.00443	0.04502	0.17808	364.267	353.142	393.749	467.029	507.937	402.602 Up
Parm1	5528.44	1.55951	0.1764	8.8409	9.49E-19	4.38E-16	3277.43	2508.49	2616.87	8311.7	9732.2	6723.95 Up
Thap6	868.186	0.49782	0.13968	3.56387	0.00037	0.00505	758.89	738.836	661.772	1035.01	1119.93	894.671 Up
Ppef2	329.172	-0.6096	0.1944	-3.136	0.00171	0.01663	430.854	341.305	421.039	273.284	214.302	294.247 Down
Naaa	8387.35	0.24661	0.1123	2.19602	0.02809	0.12915	8490.75	6846.81	7679.09	9201.91	9258.26	8847.3 Up
Scarb2	25258.6	0.20736	0.10113	2.05047	0.04032	0.16567	22253.6	25827.7	22258.5	26193.4	27411.1	27607.5 Up
Fam47e	2186.67	-1.055	0.29931	-3.5247	0.00042	0.00565	3395.91	1914.66	3546.67	1617.27	885.026	1760.51 Down
Ccdc158	180.216	-0.5849	0.28068	-2.084	0.03716	0.15698	240.886	215.042	192.976	128.484	105.09	198.816 Down
Cxcl13	667.186	0.87965	0.32753	2.68568	0.00724	0.04872	374.059	534.645	500.958	894.29	460.543	1238.62 Up
Gm7993	706.887	-1.3037	0.31991	-4.0753	4.59E-05	0.00097	1255.35	499.133	1264.09	402.787	391.513	428.448 Down
Anxa3	26948.1	0.26996	0.12359	2.18435	0.02894	0.13187	22219.3	26798.3	24284.8	29389.2	32331.8	26665.2 Up
Cfap299	1438.37	-1.0772	0.2756	-3.9087	9.28E-05	0.00175	2349.13	1256.71	2249.44	1037.05	657.33	1080.56 Down
Gm43251	18.2853	-1.253	0.61999	-2.021	0.04328	0.17352	24.4803	14.7964	38.0105	7.138	12.3636	12.923 Down
Gm42133	21.8456	-1.9258	0.61799	-3.1162	0.00183	0.01748	37.2101	25.6472	40.9343	13.2563	3.0909	10.9349 Down
Cds1	5979.46	-0.646	0.13857	-4.6621	3.13E-06	0.00011	8047.17	6455.2	7386.7	4422.5	4274.71	5290.49 Down
Arhgap24	1385.54	0.28451	0.13505	2.1067	0.03514	0.15104	1120.22	1235.01	1392.74	1486.74	1664.96	1413.58 Up
Mapk10	85.9403	-1.8987	0.60105	-3.159	0.00158	0.01566	161.57	36.4979	208.57	33.6506	22.6666	52.6862 Down
Aff1	6720.32	0.24029	0.08505	2.82541	0.00472	0.03585	6402.09	5982.7	6101.17	6996.26	7240.94	7598.74 Up
Hsd17b11	8996.81	0.52857	0.11641	4.54066	5.61E-06	0.00017	7610.44	7029.3	7460.77	10404.2	11908.2	9568.01 Up
Spp1	30258.3	2.08987	0.22498	9.28927	1.55E-20	9.80E-18	8838.37	9992.53	15703.2	52845.7	53038.7	41131 Up
Gm32051	12.2894	-2.49	0.83341	-2.9877	0.00281	0.02444	12.7298	13.81	36.0612	5.09857	2.0606	3.97631 Down
Lrrc8b	4413.86	0.28088	0.139	2.02068	0.04331	0.17361	3827.74	4357.06	3771.81	4204.28	5535.79	4786.49 Up
Lrrc8c	7848.12	0.52209	0.17201	3.03533	0.0024	0.02172	6460.85	7676.4	5192.81	8454.46	10524.5	8779.7 Up
Zfp326	1839.06	0.21362	0.10491	2.03615	0.04174	0.16953	1710.68	1660.16	1738.74	1848.74	1931.81	2144.23 Up
Brdt	247.842	-0.4529	0.16948	-2.6726	0.00753	0.0501	305.514	261.404	292.388	189.667	217.393	220.685 Down
Lpcat2b	27.9596	-2.0589	0.52241	-3.9412	8.11E-05	0.00156	27.418	55.2401	52.6299	13.2563	9.27269	9.94079 Down
Gm42669	55.4033	-1.0888	0.43044	-2.5294	0.01143	0.06802	98.9005	44.3893	82.8433	25.4929	36.0604	44.7335 Down
Mtf2	306.255	0.39908	0.17458	2.28599	0.02225	0.10965	262.429	234.77	295.312	312.033	349.271	383.714 Up
Tmed5	5315.09	0.37085	0.13317	2.78482	0.00536	0.03929	4537.67	5072.22	4297.13	5579.88	6857.67	5545.96 Up
Gm42517	84.1759	-0.8292	0.31281	-2.6509	0.00803	0.05263	107.713	80.8872	134.499	63.2223	69.03	49.7039 Down
Gm10419	229.32	-1.6208	0.31654	-5.1204	3.05E-07	1.48E-05	315.307	230.825	492.187	88.7152	123.636	125.254 Down
Pde6b	85.7717	-1.0062	0.33877	-2.9703	0.00298	0.02553	128.277	77.9279	137.422	56.0843	45.3331	69.5855 Down
Mfsd7a	535.477	0.85498	0.12168	7.02657	2.12E-12	3.23E-10	381.893	398.518	363.536	666.894	689.27	712.754 Up
Gak	6735.6	0.26033	0.09131	2.85099	0.00436	0.03379	6067.2	6269.75	6051.46	7412.31	7773.6	6839.26 Up
Dgkq	443.906	0.32062	0.15435	2.07724	0.03778	0.15871	395.602	349.196	439.557	509.857	452.301	516.921 Up
Gm20629	73.7951	-1.2272	0.3916	-3.1337	0.00173	0.01673	108.693	69.0501	132.549	30.5914	42.2422	59.6447 Down
Vmn2r10	11.5006	-2.0849	0.7131	-2.9237	0.00346	0.02851	14.6882	19.7286	21.4418	3.05914	4.12119	5.96447 Down
Vmn2r12	31.763	-1.2819	0.51181	-2.5047	0.01226	0.07166	39.1685	34.525	61.4015	16.3154	11.3333	27.8342 Down
Crlf2	1339.48	0.46156	0.15807	2.91991	0.0035	0.02881	1015.44	1228.1	1137.39	1547.93	1795.81	1312.18 Up
A430073D	271.387	0.317	0.1606	1.97389	0.04839	0.18691	231.094	225.892	268.023	299.796	314.241	289.277 Up
Zfp605	175.16	0.51745	0.21756	2.37846	0.01739	0.09323	120.443	150.924	160.814	214.14	227.696	176.946 Up
Chfr	643.851	0.42367	0.17314	2.44698	0.01441	0.08116	607.112	497.161	545.791	684.229	648.058	880.754 Up
Galnt9	55.6544	-1.2548	0.37468	-3.3491	0.00081	0.00931	81.2747	81.8737	72.1224	22.4337	47.3937	28.8283 Down
Chek2	1110.44	-0.828	0.2735	-3.0274	0.00247	0.02218	1766.5	927.244	1568.18	893.27	576.967	930.458 Down
Ttc28	1533.27	-0.302	0.12723	-2.3738	0.01761	0.09402	1792.94	1626.62	1659.79	1384.77	1521.75	1213.77 Down
Mn1	231.254	-0.54	0.17786	-3.0362	0.0024	0.02168	301.598	243.648	276.794	197.825	193.696	173.964 Down
Sez6l	79.7939	-1.4314	0.64827	-2.2081	0.02724	0.126	93.0252	162.761	93.5642	12.2366	24.7272	92.4493 Down
Sgsm1	917.034	0.43518	0.12669	3.43515	0.00059	0.00736	844.081	733.904	761.184	1101.29	1103.45	958.292 Up
Tmem119	1980.77	0.41489	0.17001	2.44037	0.01467	0.08218	1502.11	1872.24	1719.24	2466.69	2519.08	1805.25 Up
Selplg	2681.68	0.56369	0.16562	3.40352	0.00067	0.00802	2199.31	2571.62	1722.17	3027.53	3409.26	3160.18 Up
Coro1c	6059.84	0.52168	0.10966	4.75712	1.96E-06	7.08E-05	5070.36	5141.27	4716.22	6959.55	7883.84	6587.76 Up
Ssh1	3665.66	0.28595	0.11692	2.44572	0.01446	0.08138	2967.01	3617.24	3326.4	4000.34	4272.65	3810.3 Up
Dao	72.8342	2.57591	0.34555	7.45462	9.01E-14	1.79E-11	17.6258	25.6472	19.4925	134.602	143.211	96.4256 Up
Foxn4	142.213	-0.9697	0.32793	-2.9572	0.0031	0.02635	159.612	150.924	254.378	110.129	64.9088	113.325 Down
Myo1h	731.273	-1.4088	0.24282	-5.8016	6.57E-09	5.03E-07	1044.82	751.659	1390.79	422.162	334.847	443.359 Down
Fam222a	379.623	-1.0088	0.17407	-5.7955	6.81E-09	5.19E-07	563.047	453.758	504.857	275.323	215.332	265.419 Down
Tchp	1040.41	-0.3922	0.17442	-2.2484	0.02455	0.11717	1242.62	994.321	1306	959.552	743.875	996.067 Down
1500011B	642.725	-0.3091	0.14159	-2.1832	0.02902	0.13197	680.553	663.867	789.448	628.144	561.513	532.826 Down
Oasl1	423.777	-0.4249	0.18864	-2.2526	0.02428	0.11641	421.061	505.052	531.172	411.965	294.665	378.744 Down
Hnf1aos1	115.827	-1.0621	0.42563	-2.4952	0.01259	0.07305	118.485	134.154	217.342	79.5378	37.0907	108.355 Down
Unc119b	4268.92	-0.2649	0.11621	-2.2798	0.02262	0.11066	5037.07	4332.4	4609.99	4199.19	3539.08	3895.79 Down
Dynll1	12085.2	-0.2793	0.12167	-2.2958	0.02169	0.10774	14930.1	11864.8	12959.6	11614.6	10720.3	10421.9 Down
Gm13830	122.909	-1.0176	0.26898	-3.7832	0.00015	0.00262	158.632	148.951	186.154	75.4589	63.8785	104.378 Down
Cit	428.485	-0.683	0.22118	-3.0881	0.00201	0.01889	515.066	407.395	661.772	337.526	283.332	365.821 Down
Prkab1	1338.87	0.44159	0.17534	2.51851	0.01179	0.06963	1347.4	881.868	1177.35	1576.48	1449.63	1600.47 Up
C330018A	35.2949	-1.0863	0.43744	-2.4832	0.01302	0.07504	43.0854	44.3893	56.5284	27.5323	13.3939	26.8401 Down
Ccdc60	819.232	-0.9669	0.30421	-3.1785	0.00148	0.01486	1219.12	707.27	1325.49	581.237	359.574	722.695 Down
Hspb8	4734.04	-0.2378	0.09257	-2.5688	0.0102	0.06289	5306.35	4918.34	5145.06	4173.69	4625.01	4235.77 Down
Gstm2-ps	586.338	-0.6171	0.19419	-3.1777	0.00148	0.0149	720.701	628.356	780.676	452.753	370.907	564.637 Down
Taok3	4598.11	0.39892	0.14308	2.78807	0.0053	0.03904	4150.88	4405.39	3342.97	4854.86	5742.88	5091.67 Up

Pebp1	8467.6	-0.3727	0.1378	-2.7046	0.00684	0.04679	9659.93	8206.11	10799.8	7868.12	6718.58	7553.01	Down
Vsig10	1985.76	0.24929	0.10696	2.33064	0.01977	0.1014	1718.52	1736.12	1989.21	2101.63	2151.26	2217.79	Up
Wsb2	4640.46	0.23826	0.11846	2.0113	0.04429	0.17616	4290.91	4668.77	3814.69	5133.24	5267.92	4667.2	Up
Gm15728	273.934	-0.6325	0.24417	-2.5906	0.00958	0.05987	383.851	277.187	338.196	221.278	158.666	264.425	Down
Tesc	148.094	-1.0194	0.22326	-4.5659	4.97E-06	0.00015	223.261	192.354	179.331	109.109	102	82.5085	Down
Hrk	71.6743	-1.9697	0.4285	-4.5969	4.29E-06	0.00014	136.111	64.1179	142.296	34.6703	32.9696	19.8816	Down
Tbx3os1	515.041	-0.4027	0.1612	-2.4983	0.01248	0.07258	585.569	615.532	558.461	392.59	415.21	522.885	Down
Sdsl	74.6073	-0.8486	0.39498	-2.1485	0.03168	0.14016	74.4202	80.8872	132.549	62.2026	32.9696	64.6151	Down
Slc8b1	1445.53	0.30319	0.14167	2.14015	0.03234	0.14246	1214.22	1274.47	1393.72	1576.48	1821.57	1392.7	Up
Rita1	450.911	-0.4474	0.14367	-3.1142	0.00184	0.01757	563.047	473.486	524.349	391.571	353.392	399.62	Down
Cfap73	245.704	-0.839	0.2848	-2.9459	0.00322	0.027	364.267	204.191	377.181	184.568	143.211	200.804	Down
Dtx1	950.242	-1.1907	0.25529	-4.6641	3.10E-06	0.0001	1328.79	939.081	1696.83	626.105	431.695	678.956	Down
Oas1f	11.494	-2.7012	0.90995	-2.9686	0.00299	0.02565	14.6882	17.7557	27.2896	4.07886	5.15149	0	Down
Oas1a	1648.91	0.25035	0.11741	2.13218	0.03299	0.14445	1385.59	1600.98	1532.11	1915.02	1650.54	1809.22	Up
Rph3a	12.8403	3.35755	0.9011	3.72604	0.00019	0.00312	1.95843	0	4.87314	25.4929	27.8181	16.8993	Up
Naa25	206.818	0.62663	0.21388	2.92975	0.00339	0.02814	187.03	129.222	171.534	227.396	250.363	275.36	Up
Aldh2	39496	-0.2448	0.11666	-2.0979	0.03591	0.15346	41498.1	40647.8	46368.9	39705.7	32614.1	36141.7	Down
Acad12	849.551	-0.3786	0.14715	-2.5729	0.01009	0.06232	934.169	868.058	1078.91	817.811	669.694	728.66	Down
Cux2	1258.96	0.56965	0.1487	3.83101	0.00013	0.00227	901.855	1191.61	947.338	1399.05	1595.93	1517.96	Up
Hvcn1	192.296	1.79463	0.34995	5.12823	2.92E-07	1.43E-05	95.9628	117.385	44.8329	279.402	363.695	252.496	Up
Fam216a	1505.64	-0.6053	0.17107	-3.5382	0.0004	0.00546	1856.59	1539.82	2054.51	1278.72	988.056	1316.16	Down
Gpn3	2179.23	0.48098	0.11207	4.29161	1.77E-05	0.00045	1793.92	1891.97	1771.87	2671.65	2670.53	2275.45	Up
Arpc3	9923.84	0.33743	0.08863	3.80714	0.00014	0.00244	8999.94	8978.48	8327.22	11099.6	11584.7	10553.1	Up
Ift81	3264.1	-0.5027	0.18808	-2.6728	0.00752	0.05009	4159.7	3036.23	4285.44	2943.92	2178.05	2981.24	Down
Gm10064	19.0596	-1.5436	0.6713	-2.2995	0.02148	0.10712	22.5219	25.6472	37.0358	7.138	4.12119	17.8934	Down
P2rx4	7913.18	0.30464	0.10545	2.88882	0.00387	0.03099	7689.76	6808.34	6744.42	8711.42	8215.6	9309.55	Up
Camkk2	1654.9	0.42184	0.1515	2.78446	0.00536	0.0393	1358.17	1627.61	1258.24	1702.92	2126.54	1855.94	Up
Morn3	372.997	-1.2022	0.26356	-4.5613	5.08E-06	0.00016	644.322	374.843	540.918	231.475	172.06	274.366	Down
Rhof	942.036	0.54964	0.23946	2.29531	0.02172	0.10783	592.424	1067.32	634.482	1006.46	1231.21	1120.33	Up
Psmd9	1835.38	-0.2855	0.12663	-2.2548	0.02415	0.11604	2106.29	1755.84	2187.06	1710.06	1566.05	1686.95	Down
Wdr66	2544.45	-0.7886	0.2841	-2.7758	0.00551	0.04008	3712.2	2052.76	3904.36	1996.6	1279.63	2321.17	Down
Bcl7a	1619.6	-0.421	0.12762	-3.2985	0.00097	0.01075	1730.27	1728.22	2104.22	1388.85	1314.66	1451.35	Down
Lrrc43	773.049	-0.9177	0.28284	-3.2446	0.00118	0.01251	1139.8	683.596	1209.51	600.612	360.604	644.163	Down
Gm15751	99.1436	-0.9779	0.33327	-2.9344	0.00334	0.02784	149.82	94.6972	150.093	71.38	46.3634	82.5085	Down
Gm49027	59.8569	-0.9364	0.42625	-2.1969	0.02803	0.12893	64.628	63.1315	108.184	41.8083	24.7272	56.6625	Down
Ccdc62	640.516	-0.6586	0.22271	-2.9572	0.0031	0.02635	696.22	706.284	950.262	562.883	366.786	560.66	Down
Arl6ip4	2164.19	-0.2312	0.10109	-2.2873	0.02218	0.10938	2192.46	2334.88	2484.32	2069	1999.81	1904.65	Down
Pitpnm2	8901.15	-0.2169	0.08274	-2.622	0.00874	0.05607	9531.66	9770.59	9405.15	7908.91	8669.96	8120.63	Down
Cdk2ap1	2612.88	-0.2408	0.08286	-2.9057	0.00366	0.02986	2754.53	2842.89	2893.67	2382.05	2453.14	2351	Down
Rilpl1	1984.72	-0.2489	0.09303	-2.6757	0.00746	0.04975	2043.62	2200.72	2222.15	1867.1	1811.26	1763.5	Down
Tctn2	2284.42	-0.4812	0.21665	-2.2213	0.02633	0.12343	2998.35	1899.86	3087.62	2082.26	1557.81	2080.61	Down
Atp6v0a2	1904.24	0.2537	0.09276	2.73496	0.00624	0.04403	1800.77	1696.66	1714.37	2022.09	2021.45	2170.07	Up
Dnah10	4268.3	-0.99	0.26784	-3.6963	0.00022	0.00343	6934.78	3669.52	6429.62	3095.85	2055.45	3424.6	Down
Ccdc92	1452.15	-0.5111	0.18366	-2.7829	0.00539	0.03944	1614.72	1443.15	2062.31	1359.28	1010.72	1222.72	Down
Glt1d1	136.774	-0.8911	0.36932	-2.4129	0.01582	0.08678	156.674	197.286	179.331	64.242	69.03	154.082	Down
Tmem132	422.546	-0.8228	0.20499	-4.0136	5.98E-05	0.00121	661.948	463.622	494.136	309.993	259.635	345.939	Down
Gm6139	149.614	-0.5563	0.214	-2.5995	0.00934	0.05867	151.778	202.218	180.306	128.484	121.575	113.325	Down
Zfp11	464.075	-0.5442	0.16048	-3.3908	0.0007	0.0083	545.422	506.038	600.37	389.531	325.574	417.513	Down
Mrps17	4055.44	-0.4422	0.14187	-3.1167	0.00183	0.01746	5093.86	3965.45	4956.95	3636.3	3077.5	3602.54	Down
Nipsnap2	5853.59	-0.4883	0.14099	-3.4637	0.00053	0.00677	7212.88	5863.34	7428.61	5086.34	4302.53	5227.86	Down
Psph	718.567	-0.2859	0.12523	-2.2826	0.02245	0.11021	802.954	761.524	804.068	714.82	603.755	624.281	Down
Phkg1	130.634	-1.0077	0.39849	-2.5287	0.01145	0.06807	223.261	83.8465	216.367	97.8926	67.9997	94.4375	Down
Zbed5	308.116	-0.7049	0.24098	-2.925	0.00344	0.02845	468.064	271.268	406.42	262.067	212.241	228.638	Down
Nupr1l	113.995	-0.7989	0.25598	-3.1208	0.0018	0.01728	125.339	164.734	144.245	100.952	73.1512	75.55	Down
Vkorc1l1	3292.78	0.2631	0.09168	2.86976	0.00411	0.03225	3044.37	2939.56	2996	3488.44	3836.83	3451.44	Up
Gusb	5425.08	0.70947	0.10635	6.67132	2.54E-11	3.26E-09	4411.35	4196.27	3744.52	6529.23	7113.18	6555.95	Up
Castor2	1533.74	-0.3389	0.12674	-2.6736	0.0075	0.04999	1718.52	1509.24	1911.24	1375.6	1381.63	1306.22	Down
Ncf1	350.704	1.58014	0.25518	6.19225	5.93E-10	5.45E-08	190.946	173.612	162.763	476.207	713.997	386.697	Up
Syna	39.3049	-0.9336	0.3659	-2.5517	0.01072	0.06512	47.0022	51.2943	56.5284	28.552	21.6363	30.8164	Down
Rfc2	3443.37	-0.4558	0.12527	-3.6382	0.00027	0.00407	4173.4	3536.35	4238.65	3129.51	2645.81	2936.51	Down
Eif4h	24748.9	-0.2868	0.10469	-2.7399	0.00615	0.04349	26553.3	29974.6	25075.2	22502	21443.6	22944.3	Down
Limk1	1390.98	-0.3154	0.13639	-2.3128	0.02073	0.10478	1633.33	1560.53	1433.68	1200.2	1106.54	1411.59	Down
Eln	4350.39	-0.7877	0.32412	-2.4304	0.01508	0.08371	6886.8	6798.47	2842.99	3359.96	3933.68	2280.42	Down
Cldn4	1381.48	1.13263	0.24432	4.63578	3.56E-06	0.00012	789.245	654.989	1152.01	2314.75	1498.05	1879.8	Up
Bud23	1855.75	-0.2094	0.08312	-2.5194	0.01176	0.06954	2016.2	1983.71	1970.7	1709.04	1737.08	1717.77	Down
Mlxipl	144.412	-1.4535	0.32026	-4.5385	5.67E-06	0.00017	291.805	155.856	187.128	71.38	63.8785	96.4256	Down
Hip1	4369.56	0.42545	0.17818	2.38772	0.01695	0.09143	3533	4479.38	3177.28	4749.83	5912.88	4365	Up
Ccl24	20.2752	-1.64	0.70194	-2.3364	0.01947	0.10033	50.9191	17.7557	23.3911	14.276	11.3333	3.97631	Down
Gm43091	111.881	-1.0632	0.39136	-2.7167	0.00659	0.04571	169.404	87.7922	196.875	65.2618	52.5452	99.4079	Down
Styxl1	381.695	-1.1999	0.30709	-3.9072	9.34E-05	0.00176	619.842	345.25	630.584	236.574	159.696	298.224	Down
Mdh2	8887.46	-0.2326	0.08864	-2.6245	0.00868	0.05576	9861.65	9688.71	9257.01	8579.88	8240.33	7697.15	Down
Hspb1	5234.49	-0.3445	0.15057	-2.2878	0.02215	0.10926	5684.33	5474.68	6410.12	5037.39	5027.86	3772.53	Down
Zp3	11.9772	-2.0288	0.76129	-2.6649	0.0077	0.05096	21.5427	11.8372	24.3657	5.09857	2.0606	6.95855	Down
Dtx2	1233.76	-0.361	0.16025	-2.2525	0.02429	0.11641	1474.69	1246.85	1440.5	1120.67	890.178	1229.68	Down
Lrwd1	1853	-0.5871	0.14872	-3.9479	7.88E-05	0.00153	2245.33	1937.35	2492.12	1659.08	1320.84	1463.28	Down
Sh2b2	321.555	1.04212	0.23529	4.42914	9.46E-06	0.00027	223.261	241.675	165.687	422.162	530.604	345.939	Up
Col26a1	128.535	-1.6949	0.34454	-4.9193	8.69E-07	3.54E-05	187.03	142.046	260.225	59.1435	42.2422	80.5204	Down
Ift22	1995.62	-0.4894	0.16024	-3.0539	0.00226	0.02075	2560.64	1905.78	2526.23	1775.32	1466.11	1739.64	Down
4933404C	220.672	-0.6609	0.18825	-3.5109	0.00045	0.0059	286.909	229.838	294.337	169.273	161.757	181.916	Down
Znhit1	1830.88	-0.3284	0.12598	-2.6069	0.00914	0.05792	2245.33	1835.75	2034.05	1761.05	1545.45	1563.69	Down
Plod3	5803.81	0.41572	0.13648	3.0461	0.00232	0.02115	4793.25	5568.4	4558.33	6520.06	7453.18	5929.68	Up
Ap1s1	4127.22	0.26534	0.11214	2.36619	0.01797	0.0954	3561.4	3887.52	3797.15	4639.7	4847.55	4029.99	Up
Serpine1	1794.77	0.95096	0.18611	5.1096	3.23E-07	1.55E-05	1102.59	1180.76	1387.87	2290.28	2906.47	1900.68	Up

Slc12a9	993.524	0.27852	0.12767	2.18166	0.02913	0.13237	859.749	864.112	969.754	1045.21	1203.39	1018.93	Up
Pcolce	7214.23	-0.2181	0.09241	-2.3599	0.01828	0.09622	7785.72	8055.18	7434.46	7076.82	6623.79	6309.42	Down
Pilra	791.089	0.77788	0.2208	3.52307	0.00043	0.00567	512.128	784.212	452.227	939.157	1076.66	982.15	Up
Cyp3a13	658.312	0.69309	0.18277	3.79217	0.00015	0.00255	415.186	468.554	625.711	808.634	777.875	853.913	Up
Azgp1	324.317	0.67689	0.28455	2.37879	0.01737	0.09317	223.261	180.517	345.018	452.753	304.968	439.383	Up
Gm454	3.91227	-5.3899	2.32712	-2.3161	0.02055	NA	17.6258	0	5.84776	0	0	0	Down
Nxpe5	67.3874	1.13579	0.48585	2.33775	0.0194	0.10004	43.0854	48.3351	35.0866	65.2618	51.5149	161.041	Up
Fam20c	914.752	0.40455	0.17606	2.29779	0.02157	0.10738	677.615	823.669	860.596	1134.94	1146.72	844.967	Up
Dnaaf5	2421.66	-0.574	0.16886	-3.3995	0.00067	0.00809	3142.29	2342.77	3206.52	2098.57	1670.11	2069.67	Down
Adap1	472.003	0.61497	0.20507	2.99884	0.00271	0.02379	315.307	426.138	377.181	602.652	660.421	450.318	Up
Zfand2a	2512.34	0.39981	0.10366	3.85672	0.00011	0.00208	2119.02	2303.31	2076.93	2745.07	3066.17	2763.54	Up
Micall2	770.628	0.36773	0.16324	2.25261	0.02428	0.11641	602.216	604.681	811.865	917.743	857.208	830.056	Up
Snx8	1558.03	0.45825	0.11402	4.01914	5.84E-05	0.00119	1188.76	1360.29	1388.84	1719.24	1894.72	1796.3	Up
Iqce	2033.58	-0.4004	0.18325	-2.1848	0.02891	0.13181	2448.03	1778.53	2715.31	1827.33	1546.48	1885.77	Down
Amz1	1127.34	0.95654	0.18967	5.0431	4.58E-07	2.07E-05	745.181	936.122	618.888	1420.46	1711.33	1332.07	Up
Card11	1130.51	0.89319	0.21817	4.09407	4.24E-05	0.00091	765.744	982.484	625.711	1438.82	1781.39	1188.92	Up
Sdk1	204.442	0.61292	0.19616	3.12451	0.00178	0.01713	146.882	150.924	187.128	274.303	231.817	235.597	Up
Ap5z1	1369.49	0.29644	0.12052	2.4596	0.01391	0.07888	1159.39	1317.87	1210.49	1521.41	1630.96	1376.8	Up
Tnrc18	3023.83	0.3075	0.08987	3.4217	0.00062	0.00765	2591	2679.14	2838.11	3335.49	3454.59	3244.67	Up
Actb	99624.7	0.2171	0.09813	2.21239	0.02694	0.12526	93653.9	92480.7	90294.3	106896	116239	98184.1	Up
Rnf216	2746.48	0.20905	0.10309	2.02784	0.04258	0.1716	2766.28	2381.24	2496.02	2837.87	2975.5	3022	Up
Daglb	903.773	0.39179	0.1374	2.85135	0.00435	0.03377	749.098	883.841	712.453	1066.62	1063.27	947.357	Up
Rac1	21525	0.22911	0.09695	2.36313	0.01812	0.09597	19747.8	20362.9	19347.3	23030.3	25155.8	21505.9	Up
Rsph10b	701.446	-1.132	0.24341	-4.6504	3.31E-06	0.00011	986.067	695.433	1208.54	491.503	341.029	486.104	Down
2900089D	271.935	0.38278	0.19425	1.97049	0.04878	0.18785	206.614	212.082	289.464	309.993	314.241	299.218	Up
Smurf1	3372.8	0.26313	0.09624	2.73416	0.00625	0.04409	3033.6	3007.62	3156.82	3640.38	3942.95	3455.42	Up
Arpc1b	14257.1	0.72221	0.11465	6.29943	2.99E-10	2.94E-08	10641.1	11496.8	10145.9	17535	19587	16136.9	Up
Usp12	2793.19	0.26339	0.0999	2.63642	0.00838	0.05438	2607.64	2662.37	2346.9	2988.78	2972.41	3181.05	Up
Rasl11a	1452.92	-0.929	0.27456	-3.3837	0.00072	0.00843	2108.25	2464.1	1143.24	1023.79	1158.06	820.115	Down
Katnal1	735.344	-0.2792	0.1414	-1.9747	0.04831	0.18675	792.183	741.795	884.962	663.834	599.634	729.654	Down
Gm15406	10.5284	-1.7869	0.78242	-2.2838	0.02238	0.10996	7.8337	17.7557	23.3911	5.09857	4.12119	4.97039	Down
Hmgb1	6540.61	0.26003	0.08801	2.95456	0.00313	0.0265	6132.81	5588.12	6137.23	7278.72	7205.91	6900.89	Up
Alox5ap	1084.76	0.77672	0.20256	3.8346	0.00013	0.00224	758.89	942.04	697.833	1316.45	1695.87	1097.46	Up
Hsph1	8778.79	-0.7351	0.30139	-2.4391	0.01472	0.08237	15212.1	7520.54	10172.2	6140.72	4478.71	9148.5	Down
Gm20005	43.5964	-1.4894	0.50051	-2.9759	0.00292	0.02518	76.3786	48.3351	68.2239	28.552	9.27269	30.8164	Down
B3glct	729.573	0.44839	0.17341	2.58565	0.00972	0.06049	502.336	652.03	696.859	847.383	928.299	750.529	Up
Gm42906	26.1208	-1.1022	0.54338	-2.0285	0.04251	0.17142	37.2101	41.43	28.2642	8.15772	25.7575	15.9053	Down
Gm43196	98.797	0.59462	0.30009	1.98148	0.04754	0.18493	58.7528	85.8194	91.615	92.7941	123.636	140.165	Up
Gm20559	3178.53	0.41198	0.14945	2.75661	0.00584	0.04193	2197.35	3076.67	2909.26	3695.45	3627.68	3564.77	Up
Samd9l	6194.52	0.42231	0.11227	3.76159	0.00017	0.0028	4663.99	5706.5	5512.49	7107.41	6932.88	7243.85	Up
Pon1	6558.67	-1.6427	0.73438	-2.2368	0.0253	0.1197	7866.02	6388.12	15552.1	3759.69	947.875	4838.18	Down
Gm44250	9872.66	0.26305	0.10277	2.55965	0.01048	0.06411	8522.09	9652.21	8751.18	10338.9	10390.6	11581	Up
Pon3	6010.75	0.24362	0.11718	2.07907	0.03761	0.15832	4912.71	5929.43	5671.36	6307.96	6218.88	7024.16	Up
Pon2	10261.6	0.25743	0.09895	2.60153	0.00928	0.05851	9110.6	9903.75	9030.9	10992.5	12011.2	10520.3	Up
Dync1i1	1722.03	-0.808	0.34776	-2.3234	0.02016	0.10275	2906.3	1255.72	2414.15	1374.58	724.3	1657.13	Down
Slc25a13	894.279	-0.3918	0.16564	-2.3656	0.018	0.09552	998.797	852.275	1193.92	748.471	723.27	848.943	Down
C1galt1	4485.58	0.44089	0.15425	2.85828	0.00426	0.03325	4113.67	3644.86	3657.78	4539.77	6240.52	4716.9	Up
Nxph1	29.6001	1.35199	0.5836	2.31661	0.02052	0.10413	14.6882	15.7829	19.4925	30.5914	74.1815	22.8638	Up
Tmem168	3412.15	0.2738	0.09511	2.87875	0.00399	0.03167	2948.41	3268.04	3051.56	3556.77	3892.47	3755.63	Up
2610001J(	2961.82	0.23025	0.10123	2.27461	0.02293	0.11169	2616.46	2773.84	2787.43	3250.85	3395.86	2946.45	Up
Ppp1r3a	461.711	0.57772	0.25374	2.27681	0.0228	0.11126	278.096	323.549	509.73	622.026	566.664	470.199	Up
Tfec	833.529	1.10216	0.16985	6.48902	8.64E-11	9.49E-09	583.611	557.333	448.329	1102.31	1310.54	999.049	Up
Met	5399.91	0.81261	0.14631	5.55387	2.79E-08	1.79E-06	4085.28	4212.05	3457	6010.2	7919.9	6715	Up
Capza2	12135.7	0.35599	0.11053	3.22077	0.00128	0.01334	10587.2	11109.2	10241.4	13006.5	15207.2	12662.6	Up
Cftr	835.325	-1.2154	0.2891	-4.2041	2.62E-05	0.00061	1310.19	1412.57	780.676	444.596	375.029	688.896	Down
Gm20186	43.0831	3.07538	1.0262	2.99687	0.00273	0.02389	1.95843	8.87787	16.5687	17.3352	198.848	14.9112	Up
Ptprz1	729.418	-0.9276	0.32086	-2.8908	0.00384	0.03085	1174.08	530.699	1163.7	571.04	355.453	581.536	Down
Aass	854.084	0.59768	0.19039	3.13917	0.00169	0.01651	581.652	589.885	867.418	1026.85	960.238	1098.46	Up
Cadps2	2441.93	-0.3631	0.12112	-2.9974	0.00272	0.02387	2851.47	2758.06	2633.44	2021.07	1970.96	2416.6	Down
Iqub	1376.93	-0.8675	0.34091	-2.5446	0.01094	0.06599	2223.79	1052.52	2060.36	1088.04	560.482	1276.4	Down
Lmod2	113.886	-1.9581	0.70232	-2.788	0.0053	0.03904	282.013	19.7286	241.708	41.8083	46.3634	51.6921	Down
Gpr37	55.1832	-4.8409	0.91403	-5.2962	1.18E-07	6.41E-06	227.177	13.81	78.9448	2.03943	5.15149	3.97631	Down
Lep	31.1957	-5.4839	2.03777	-2.6911	0.00712	0.04817	172.341	0.98643	9.74627	2.03943	2.0606	0	Down
Rbm28	3140.18	0.23582	0.10525	2.24064	0.02505	0.11895	2725.15	3055.96	2871.25	3133.58	3566.89	3488.22	Up
Ccdc136	745.574	0.75299	0.1422	5.29521	1.19E-07	6.43E-06	547.38	627.369	491.212	878.994	989.087	939.404	Up
Tnpo3	2263.35	0.30576	0.10649	2.87138	0.00409	0.03214	2109.22	1980.75	1983.37	2444.26	2336.72	2725.76	Up
Cpa2	15.9579	2.2429	0.7949	2.8216	0.00478	0.03606	3.91685	10.8507	1.94925	13.2563	32.9696	32.8046	Up
Cpa5	7.59388	6.39689	1.4459	4.42416	9.68E-06	NA	0	0	0	14.276	13.3939	17.8934	Up
Cep41	663.976	-0.3986	0.13899	-2.8679	0.00413	0.03237	844.081	680.636	740.717	573.08	541.937	603.406	Down
Podxl	18160.8	0.41948	0.16575	2.53085	0.01138	0.06789	12734.7	17225	16657.4	17917.4	23871	20559.5	Up
Gm13849	58.761	-1.0998	0.44098	-2.4939	0.01263	0.0733	79.3162	97.6565	63.3508	38.7492	54.6058	18.8875	Down
Plxna4	2514.65	0.72171	0.19638	3.67495	0.00024	0.00367	1837.98	2098.14	1759.2	2964.31	4002.71	2425.55	Up
Lrguk	1786.34	-1.0989	0.31812	-3.4545	0.00055	0.00694	2939.6	1630.57	2736.75	1130.86	674.845	1605.44	Down
Akr1b8	4795.76	0.49922	0.13873	3.59861	0.00032	0.0046	3418.43	3943.75	4560.28	5527.87	6041.67	5282.53	Up
Akr1b10	1575.2	0.21838	0.10421	2.09556	0.03612	0.15417	1470.78	1396.78	1500.93	1644.8	1821.57	1616.37	Up
Agbl3	965.992	-0.3989	0.16077	-2.4814	0.01309	0.07538	1110.43	951.905	1233.88	789.259	754.178	956.304	Down
Gm43748	68.9534	-1.3241	0.43458	-3.0469	0.00231	0.02111	104.776	74.9687	115.981	39.7689	19.5757	58.6506	Down
Stra8	11.1116	-4.992	1.26331	-3.9515	7.77E-05	0.00151	14.6882	19.7286	30.2134	2.03943	0	0	Down
9330158H	24.3805	-1.3319	0.55143	-2.4153	0.01572	0.08638	40.1477	17.7557	46.7821	13.2563	14.4242	13.9171	Down
Ptn	81.545	-2.3052	0.58794	-3.9208	8.82E-05	0.00167	131.215	71.0229	204.672	53.0252	14.4242	14.9112	Down
D630045J	547.565	-0.4921	0.14149	-3.4778	0.00051	0.0065	589.486	687.541	643.254	425.221	437.877	502.01	Down
Zc3hav1	5435.38	0.26063	0.08966	2.9069	0.00365	0.02977	4640.49	5074.19	5122.64	5763.43	5883	6128.49	Up
Ttc26	1403.45	-0.6961	0.29361	-2.3709	0.01775	0.09457	1986.82	1139.33	2080.83	1101.29	707.815	1404.63	Down

Klrg2	368.175	-0.539	0.22447	-2.4011	0.01634	0.08901	427.916	374.843	505.832	305.914	228.726	365.821	Down
Tbxas1	969.948	1.39472	0.20075	6.94742	3.72E-12	5.25E-10	531.713	624.41	447.354	1461.25	1650.54	1104.42	Up
Slc37a3	2892.36	0.21104	0.1058	1.99468	0.04608	0.18094	2751.59	2828.09	2463.86	3275.32	3088.83	2946.45	Up
Rab19	773.749	0.50826	0.15272	3.32815	0.00087	0.00987	579.694	696.419	640.33	923.862	1015.87	786.316	Up
Tmem178	69.3941	-0.5585	0.28052	-1.9908	0.04651	0.18206	95.9628	79.9008	72.1224	53.0252	57.6967	57.6566	Down
Clec5a	568.867	2.05301	0.22516	9.11806	7.65E-20	4.26E-17	190.946	254.499	217.342	744.392	1186.9	819.121	Up
Mgam	104.031	1.05302	0.27358	3.84904	0.00012	0.00214	50.9191	85.8194	66.2747	148.878	139.09	133.207	Up
Prss2	4.36738	2.5103	1.27423	1.97006	0.04883	NA	1.95843	0	1.94925	11.2169	4.12119	6.95855	Up
Trbc1	389.411	-0.4015	0.17934	-2.2388	0.02517	0.1193	380.914	533.658	415.191	331.407	348.241	327.052	Down
Gstk1	2999.07	-0.5015	0.12	-4.1791	2.93E-05	0.00067	3274.49	3860.89	3410.22	2441.2	2303.75	2703.89	Down
Casp2	2269.85	0.2011	0.09635	2.08723	0.03687	0.15632	1983.89	2218.48	2133.46	2363.7	2400.6	2518.99	Up
Tcaf1	2332.87	0.27475	0.12708	2.16199	0.03062	0.13704	2205.19	2236.24	1892.73	2397.35	2830.23	2435.49	Up
Cntnap2	74.5423	0.83299	0.27374	3.04296	0.00234	0.02131	55.8151	57.2129	47.7567	102.991	100.969	82.5085	Up
Zfp777	1554.9	-0.3197	0.08939	-3.5766	0.00035	0.00487	1748.87	1697.65	1732.89	1426.58	1375.45	1347.97	Down
Sspo	372.669	-0.7049	0.32051	-2.1993	0.02786	0.12829	533.671	278.173	574.055	268.185	209.151	372.779	Down
Zfp862-ps	1613.17	-2.1717	0.12733	-17.056	3.14E-65	2.97E-61	2488.18	2813.3	2619.8	563.902	535.755	658.08	Down
Atp6v0e2	2060.32	-0.646	0.16661	-3.8774	0.00011	0.00195	2708.5	2075.45	2758.2	1782.46	1368.24	1669.06	Down
Rarres2	2922.01	-0.302	0.11602	-2.6028	0.00925	0.05837	3307.78	3022.42	3349.79	2873.56	2605.62	2372.87	Down
AI854703	68.1951	-0.9768	0.36115	-2.7048	0.00684	0.04679	93.0252	76.9415	101.361	27.5323	55.6361	54.6743	Down
Aoc1	3791.75	-1.1268	0.26217	-4.2978	1.72E-05	0.00043	4980.28	3657.68	6966.64	2945.96	1752.54	2447.42	Down
Gpnmb	4673.63	1.92787	0.25378	7.59651	3.04E-14	6.85E-12	2320.73	2102.08	1413.21	6950.38	9847.59	5407.79	Up
Gm19253	9.78866	2.32118	0.8866	2.61808	0.00884	0.05652	1.95843	1.97286	5.84776	20.3943	20.606	7.95263	Up
Npy	25.3672	-1.3166	0.54518	-2.4149	0.01574	0.08644	35.2517	20.715	52.6299	11.2169	16.4848	15.9053	Down
Osbpl3	2258.89	0.66578	0.25843	2.57622	0.00999	0.06186	2088.66	1021.94	2129.56	2713.46	2689.08	2910.66	Up
Snx10	2539.53	0.71295	0.13835	5.15338	2.56E-07	1.26E-05	1933.95	1821.94	2017.48	2914.35	3689.5	2859.96	Up
Skap2	6677.5	0.35419	0.10504	3.37195	0.00075	0.00872	5860.59	6389.1	5336.08	7339.91	7762.27	7377.06	Up
Jazf1	541.993	-0.6463	0.21979	-2.9405	0.00328	0.02741	681.532	543.523	759.235	477.227	315.271	475.17	Down
Gm16499	36.1125	-1.6457	0.63435	-2.5943	0.00948	0.05938	46.023	23.6743	94.5388	15.2957	10.303	26.8401	Down
4921529L0	2.69964	4.90474	2.05428	2.38757	0.01696	NA	0	0	0	10.1971	1.0303	4.97039	Up
Scrn1	2123.74	-0.7153	0.18608	-3.8438	0.00012	0.00217	3149.15	1960.04	2809.85	1554.05	1579.45	1689.93	Down
Plekha8	1305.56	-0.3581	0.16284	-2.1994	0.02785	0.12829	1581.43	1341.54	1477.53	1138	947.875	1346.98	Down
Znrf2	4160.05	0.2171	0.09323	2.32872	0.01987	0.10187	3988.33	3761.26	3793.25	4212.44	4745.55	4459.44	Up
Mindy4	637.593	-0.4594	0.14955	-3.072	0.00213	0.01976	780.433	623.424	810.89	549.626	523.392	537.796	Down
Vopp1	2142.54	0.31386	0.08566	3.66396	0.00025	0.00378	1937.86	1871.26	1921.97	2403.47	2426.35	2294.33	Up
Fam13a	3404.48	-0.8864	0.30241	-2.931	0.00338	0.02809	4288.95	2715.64	6251.26	2542.15	1681.45	2947.44	Down
Tigd2	1433.51	0.36627	0.11034	3.31944	0.0009	0.0101	1218.14	1339.57	1199.77	1614.21	1714.42	1514.98	Up
A730020E	233.34	-0.645	0.22794	-2.8295	0.00466	0.03555	317.265	218.987	317.728	191.706	154.545	199.81	Down
Ccser1	1612.01	-0.4125	0.11996	-3.4385	0.00058	0.0073	1945.7	1646.35	1930.74	1362.34	1316.72	1470.24	Down
Grid2	43.9827	-0.9145	0.45002	-2.0322	0.04213	0.17057	51.8983	51.2943	69.1985	47.9266	23.6969	19.8816	Down
Hpgds	424.142	0.70139	0.21483	3.26492	0.00109	0.01184	278.096	329.467	361.587	551.666	399.756	624.281	Up
Tnip3	358.204	0.9212	0.19188	4.80101	1.58E-06	5.88E-05	198.78	250.553	293.363	511.897	451.271	443.359	Up
Gng12	6634.19	0.33521	0.07957	4.2127	2.52E-05	0.0006	5745.04	6065.56	5790.26	7430.66	7171.91	7601.72	Up
Igkv1-133	76.9738	5.13892	0.86679	5.92868	3.05E-09	2.48E-07	4.89606	4.93215	2.92388	124.405	17.5151	307.17	Up
Igkv17-12	66.3875	1.17044	0.33876	3.45507	0.00055	0.00693	37.2101	45.3758	39.9597	119.307	67.9997	88.473	Up
Igkv14-12	96.8183	2.72183	0.69359	3.92427	8.70E-05	0.00165	42.1061	20.715	13.6448	349.762	45.3331	109.349	Up
Igkv14-10	31.0785	2.30667	0.98495	2.34192	0.01918	0.09947	17.6258	5.91858	7.79702	25.4929	124.666	4.97039	Up
Igkv4-90	8.22237	-2.3347	0.98093	-2.3801	0.01731	0.09296	14.6882	11.8372	14.6194	5.09857	3.0909	0	Down
Igkv12-89	239.998	4.3662	1.0779	4.05065	5.11E-05	0.00107	54.8359	4.93215	6.82239	399.728	720.179	253.49	Up
Igkv1-88	48.2018	1.71753	0.65907	2.60601	0.00916	0.05801	23.5011	5.91858	38.0105	69.3406	38.121	114.319	Up
Igkv4-79	120.464	5.69034	1.55939	3.64909	0.00026	0.00396	8.81291	3.94572	0.97463	75.4589	10.303	623.287	Up
Igkv4-70	66.5871	2.67876	0.81933	3.26945	0.00108	0.01169	27.418	20.715	5.84776	163.154	13.3939	168.993	Up
Igkv4-69	14.862	2.71725	1.20194	2.26071	0.02378	0.11467	5.87528	3.94572	1.94925	69.3406	3.0909	4.97039	Up
Igkv4-63	39.33	3.7194	1.06461	3.49367	0.00048	0.00622	5.87528	4.93215	5.84776	16.3154	6.18179	196.828	Up
Igkv5-43	45.4912	1.21376	0.60375	2.01037	0.04439	0.17629	42.1061	10.8507	29.2388	44.8675	106.121	39.7631	Up
Igkv7-33	4.71954	-5.6608	1.90171	-2.9767	0.00291	NA	8.81291	0.98643	18.5179	0	0	0	Down
Igkv8-30	583.046	3.16957	1.33374	2.37646	0.01748	0.09355	140.027	27.62	182.255	70.3603	547.088	2530.92	Up
Igkv6-25	136.027	2.89065	1.30744	2.21092	0.02704	0.12551	54.8359	19.7286	22.4164	25.4929	51.5149	642.175	Up
Igkv6-23	61.7174	-0.8309	0.39244	-2.1174	0.03423	0.14826	98.9005	55.2401	82.8433	36.7097	61.8179	34.7927	Down
Igkv6-13	26.1653	2.11619	0.65198	3.2458	0.00117	0.01248	9.79213	10.8507	8.77165	64.242	48.424	14.9112	Up
Igkv3-7	73.4179	-1.8083	0.88287	-2.0482	0.04054	0.16615	242.845	32.5522	67.2493	76.4786	14.4242	6.95855	Down
Igkv3-5	110.495	3.95409	0.76495	5.16908	2.35E-07	1.16E-05	19.5843	10.8507	9.74627	495.581	55.6361	71.5737	Up
Gm30211	563.925	0.65465	0.23976	2.73042	0.00633	0.04437	302.577	501.106	510.705	572.06	792.3	704.802	Up
Eif2ak3	2310.94	0.2303	0.09995	2.30426	0.02121	0.10646	2159.16	2231.3	1990.19	2625.77	2436.66	2422.57	Up
Thnsl2	1039.11	-1.1613	0.21184	-5.482	4.20E-08	2.57E-06	1465.88	1190.62	1651.99	643.44	490.422	792.281	Down
Gm44172	2.03292	4.49493	2.05833	2.18377	0.02898	NA	0	0	0	2.03943	6.18179	3.97631	Up
Capg	3897.66	1.00427	0.18861	5.32449	1.01E-07	5.60E-06	2514.62	2960.28	2304.99	5167.92	6372.4	4065.78	Up
Retsat	2151.4	0.37946	0.11933	3.17985	0.00147	0.0148	1970.18	1935.37	1704.62	2451.39	2621.08	2225.74	Up
Tgoln1	19100.8	0.82772	0.16325	5.07044	3.97E-07	1.84E-05	13703.1	15967.3	11630.2	23606.4	28265.2	21432.3	Up
Gm20560	25.9068	-1.1777	0.43224	-2.7246	0.00644	0.04488	33.2932	36.4979	38.0105	16.3154	14.4242	16.8993	Down
Dnah6	5804.83	-0.8164	0.27174	-3.0046	0.00266	0.02344	8685.62	5160.01	8369.12	4441.88	2771.5	5400.83	Down
Suclg1	6586.87	-0.3639	0.11633	-3.1279	0.00176	0.01698	7995.27	7592.55	6651.83	5779.74	5353.43	6148.38	Down
Gm20383	44.5541	-1.4492	0.57032	-2.541	0.01105	0.06648	64.628	40.4436	90.6403	29.5717	8.24239	33.7987	Down
Eva1a	646.345	-0.8289	0.11706	-7.0812	1.43E-12	2.29E-10	787.287	820.709	873.266	478.246	453.331	465.229	Down
Tacr1	436.974	-1.5345	0.6941	-2.2108	0.02705	0.12552	1007.61	242.662	698.808	229.436	112.303	331.028	Down
2310069B	23.0325	-1.4868	0.54755	-2.7154	0.00662	0.04576	37.2101	31.5657	33.1373	8.15772	8.24239	19.8816	Down
M1ap	332.593	-1.0881	0.40155	-2.7098	0.00673	0.04623	233.053	596.79	527.273	148.878	324.544	165.017	Down
Wbp1	4134.21	-0.2254	0.08848	-2.5472	0.01086	0.06561	4518.09	4543.49	4307.85	4030.93	3704.95	3699.96	Down
Ino80b	1036.57	-0.4725	0.14619	-3.2321	0.00123	0.01297	1124.14	1146.23	1344.01	989.123	807.754	808.186	Down
Rtkn	678.19	0.50799	0.17536	2.89691	0.00377	0.03042	588.507	651.044	440.532	759.688	822.178	807.192	Up
Wdr54	333.566	-0.9224	0.24667	-3.7394	0.00018	0.003	480.793	321.576	507.781	273.284	182.363	235.597	Down
1700003E	982.3	-0.9735	0.191	-5.0967	3.46E-07	1.64E-05	1519.74	1017.01	1368.38	688.308	568.725	731.642	Down
Gm21284	20.2581	-1.4561	0.55803	-2.6094	0.00907	0.05756	23.5011	22.6879	42.8836	9.17743	12.3636	10.9349	Down
Clec4f	6.43245	2.4765	1.23037	2.01281	0.04413	NA	0	1.97286	3.89851	13.2563	16.4848	2.98224	Up

Paip2b	3832.96	0.21708	0.09106	2.38383	0.01713	0.09225	3460.54	3718.84	3456.03	4338.89	4022.29	4001.17 Up
Gm44127	52.3935	-0.8019	0.34055	-2.3546	0.01854	0.09728	75.3994	49.3215	75.0463	36.7097	38.121	39.7631 Down
Spr-ps1	39.6409	-1.1722	0.36678	-3.1958	0.00139	0.0142	59.732	59.1858	45.8075	25.4929	25.7575	21.8697 Down
Cct7	9871.37	-0.2571	0.10748	-2.3924	0.01674	0.09065	11979.7	10107.9	10158.5	9262.07	8780.2	8939.75 Down
Nat8f7	82.358	-0.8036	0.4	-2.0091	0.04453	0.17657	74.4202	83.8465	155.94	49.966	49.4543	80.5204 Down
Alms1-ps2	27.512	1.20302	0.55837	2.15453	0.0312	0.13856	23.5011	16.7693	9.74627	35.69	23.6969	55.6684 Up
Nat8f1	116.809	-0.5941	0.22424	-2.6494	0.00806	0.05282	140.027	148.951	132.549	102.991	95.8178	80.5204 Down
Add2	12.0047	-2.0268	0.71103	-2.8505	0.00436	0.0338	19.5843	20.715	17.5433	2.03943	6.18179	5.96447 Down
Pcyox1	5721.21	0.52672	0.12512	4.20981	2.56E-05	0.0006	4325.18	5306	4433.58	6683.21	7272.88	6306.43 Up
2310040G	106.132	-0.6041	0.26472	-2.2821	0.02248	0.11025	112.609	125.277	146.194	104.011	73.1512	75.55 Down
Gm43936	21.7913	-1.3115	0.49885	-2.629	0.00856	0.05526	29.3764	39.4572	24.3657	13.2563	12.3636	11.9289 Down
1600020E	966.999	-0.3954	0.12035	-3.2854	0.00102	0.01114	1077.13	1042.66	1176.38	854.521	767.572	883.736 Down
Anxa4	16508.7	0.38815	0.08376	4.63426	3.58E-06	0.00012	13749.1	14773.8	14380.6	18959.6	19358.3	17830.8 Up
Gfpt1	8643.96	0.3781	0.11533	3.27844	0.00104	0.01137	8254.76	7629.05	6669.37	9291.64	9874.38	10144.6 Up
Arhgap25	1082.7	0.52973	0.18771	2.82211	0.00477	0.03605	845.061	1103.81	709.529	1317.47	1367.21	1153.13 Up
Prokr1	39.0335	1.27038	0.41842	3.03617	0.0024	0.02168	26.4387	23.6743	18.5179	39.7689	72.1209	53.6802 Up
1810020C	507.212	-0.9568	0.31706	-3.0178	0.00255	0.02271	717.763	442.907	847.926	358.94	219.454	456.282 Down
Gp9	142.789	-1.6175	0.37155	-4.3535	1.34E-05	0.00035	144.923	179.53	321.627	67.3012	90.6663	52.6862 Down
Hmces	1610.39	-0.2524	0.10979	-2.299	0.02151	0.10714	1761.6	1635.5	1855.69	1521.41	1367.21	1520.94 Down
H1f10	46.8282	-1.2274	0.41306	-2.9715	0.00296	0.02546	58.7528	61.1586	76.9956	38.7492	16.4848	28.8283 Down
Gm5577	116.403	-0.9664	0.37103	-2.6046	0.0092	0.05817	121.422	122.317	218.317	71.38	55.6361	109.349 Down
Rab7	1166.87	0.21132	0.09626	2.19533	0.02814	0.12925	1097.7	1053.51	1093.53	1274.64	1205.45	1276.4 Up
Gata2	4728.19	-0.2753	0.13848	-1.9879	0.04682	0.18302	5281.87	5015.99	5235.7	4851.8	4419.98	3563.77 Down
Ruvbl1	2404.34	-0.5307	0.18143	-2.9252	0.00344	0.02844	3171.67	2342.77	3010.62	2105.71	1580.48	2214.81 Down
Sec61a1	7824.09	0.24617	0.11808	2.08471	0.0371	0.15684	7841.54	7332.13	6300.97	8957.18	8175.42	8337.34 Up
Plxna1	608.595	0.94761	0.1809	5.23841	1.62E-07	8.44E-06	414.207	440.934	391.8	661.795	769.633	973.203 Up
Chchd6	1670.22	-0.8392	0.1971	-4.2576	2.07E-05	0.0005	2468.6	1604.92	2354.7	1283.82	1018.97	1290.31 Down
Vmn1r42	37.4881	1.40252	0.41684	3.36467	0.00077	0.00891	18.605	20.715	22.4164	70.3603	39.1513	53.6802 Up
Cfap100	4109.59	-0.8784	0.28876	-3.0422	0.00235	0.02134	5809.67	3114.16	7046.56	3301.84	2159.51	3225.78 Down
Klf15	2641.17	-0.8258	0.32182	-2.566	0.01029	0.06325	3773.89	1867.31	4490.11	2313.73	1278.6	2123.35 Down
1810044D	51.0454	-0.8487	0.32544	-2.6077	0.00911	0.05782	66.5865	64.1179	66.2747	33.6506	30.909	44.7335 Down
Chchd4	1657.14	-0.3178	0.09525	-3.3364	0.00085	0.00966	1944.72	1787.41	1784.54	1492.86	1492.9	1440.42 Down
Xpc	2020.61	-0.2188	0.09314	-2.349	0.01882	0.09813	2180.71	2270.76	2069.13	1925.22	1858.66	1819.16 Down
Gm45218	45.932	-1.2639	0.5321	-2.3753	0.01753	0.09371	47.9814	33.5386	113.057	27.5323	22.6666	30.8164 Down
Slc6a6	12462.3	0.48079	0.14125	3.40392	0.00066	0.00801	8923.57	10334.8	11955.8	14168.9	15863.5	13527.4 Up
Grip2	198.109	1.27607	0.27575	4.62767	3.70E-06	0.00012	110.651	155.856	80.8941	281.441	315.271	244.543 Up
9530026P	950.391	-0.3447	0.15538	-2.2182	0.02654	0.12405	1035.03	1078.17	1076.96	679.13	932.42	900.635 Down
Kbtbd8	275.046	-0.718	0.29572	-2.428	0.01518	0.08407	366.226	362.02	298.236	174.371	144.242	305.182 Down
Tafa4	64.5261	1.17756	0.46335	2.54138	0.01104	0.06644	40.1477	47.3486	31.1881	147.859	65.9391	54.6743 Up
Eogt	5425.77	0.41943	0.08802	4.76512	1.89E-06	6.83E-05	4796.18	4567.17	4564.18	6344.67	6435.24	5847.17 Up
Tmf1	2719.59	0.21956	0.08868	2.47588	0.01329	0.07615	2627.23	2421.68	2490.17	2831.75	3000.23	2946.45 Up
Arl6ip5	7731.98	0.27159	0.10639	2.55289	0.01068	0.06502	6744.82	7635.95	6638.19	8436.1	8984.2	7952.63 Up
Gxylt2	150.488	-0.6938	0.33228	-2.0881	0.03679	0.15609	247.741	105.548	204.672	119.307	113.333	112.331 Down
Crbn	722.029	0.3559	0.15894	2.23928	0.02514	0.11922	672.719	617.505	610.117	676.071	826.299	929.463 Up
Lrrn1	15.1537	-1.8953	0.71548	-2.6489	0.00807	0.05284	25.4595	33.5386	12.6702	3.05914	8.24239	7.95263 Down
Sumf1	2938.71	0.27674	0.11337	2.44091	0.01465	0.08216	2465.66	2835	2672.43	3101.97	3514.35	3042.87 Up
Bhlhe40	7755.72	0.57331	0.22298	2.57113	0.01014	0.06255	5459.11	4615.5	8629.35	8959.22	9828.02	9043.13 Up
Edem1	5014.24	0.2732	0.08845	3.0889	0.00201	0.01885	4718.83	4607.61	4296.16	5438.14	5694.46	5330.25 Up
Lmcd1	722.902	-0.6209	0.18256	-3.4013	0.00067	0.00805	984.109	804.927	839.154	500.68	699.573	508.968 Down
Srgap3	267.734	-0.5283	0.2668	-1.9801	0.04769	0.18528	300.618	240.689	407.394	220.258	171.03	266.413 Down
Gt(ROSA)2	3020.87	1.24164	0.16072	7.72531	1.12E-14	2.67E-12	1993.68	1667.07	1726.06	4004.42	5090.7	3643.3 Up
Setd5	5540.08	0.21423	0.07409	2.89136	0.00384	0.03083	5128.14	5167.9	5092.43	6033.65	6016.94	5801.44 Up
Lhfpl4	223.891	-1.5527	0.29105	-5.3347	9.57E-08	5.36E-06	340.766	280.146	381.079	105.031	77.2724	159.053 Down
Ogg1	803.007	-0.4692	0.15396	-3.0477	0.00231	0.0211	952.774	783.225	1061.37	649.558	658.361	712.754 Down
Camk1	3332.23	0.46867	0.13017	3.60028	0.00032	0.00457	2667.38	3055.96	2663.66	3927.94	4275.74	3402.73 Up
Arpc4	4515.71	0.32918	0.11081	2.97068	0.00297	0.02551	3966.79	4128.21	3913.13	5084.3	5497.67	4504.17 Up
Ttll3	2354.13	-0.5512	0.23322	-2.3636	0.0181	0.09591	2918.05	2038.95	3438.49	2121.01	1436.24	2172.06 Down
Gm44280	34.8128	-0.9627	0.42052	-2.2894	0.02206	0.10896	50.9191	35.5115	51.6552	23.4534	17.5151	29.8224 Down
Ghrl	69.8324	-2.2188	0.50131	-4.426	9.60E-06	0.00027	64.628	127.249	153.016	40.7886	14.4242	18.8875 Down
Slc6a1	53.3581	2.53825	0.39353	6.45003	1.12E-10	1.18E-08	20.5635	9.8643	16.5687	106.05	87.5754	79.5263 Up
Pparg	1004.38	0.64707	0.15177	4.26351	2.01E-05	0.00049	818.622	699.379	830.382	1187.97	1407.39	1082.55 Up
Mkrn2os	207.364	0.87777	0.3075	2.85456	0.00431	0.03351	176.258	82.8601	179.331	256.968	255.514	293.253 Up
Efcab12	929.158	-0.8507	0.30455	-2.7933	0.00522	0.03858	1253.39	790.13	1542.83	814.752	419.331	754.506 Down
Ift122	1441.16	-0.6068	0.18465	-3.286	0.00102	0.01112	1857.57	1430.32	1931.71	1238.95	917.996	1270.43 Down
Rho	69.3002	-0.8933	0.39782	-2.2455	0.02474	0.11796	120.443	66.0908	83.8179	46.9069	31.9393	66.6033 Down
Tmcc1	2365.67	0.23786	0.119	1.99876	0.04563	0.1796	1962.34	2392.09	2158.8	2597.21	2682.9	2400.7 Up
9530062K	91.0946	-0.6718	0.30511	-2.2018	0.02768	0.12773	105.755	83.8465	146.194	71.38	62.8482	76.544 Down
Washc2	2661.79	0.23564	0.0835	2.82182	0.00478	0.03605	2363.82	2461.14	2509.67	2881.71	2932.23	2822.19 Up
Zfand4	697.733	-0.7838	0.28188	-2.7807	0.00542	0.03964	936.127	635.261	1076.96	509.857	348.241	679.95 Down
Alox5	922.145	0.93457	0.24005	3.8932	9.89E-05	0.00185	642.364	801.967	456.126	1231.82	1439.33	961.274 Up
Zfp422	2824.09	-0.2074	0.08284	-2.5036	0.01229	0.07181	3008.14	2947.45	3124.66	2599.25	2585.02	2680.04 Down
Rassf4	2462.34	0.61045	0.13779	4.43028	9.41E-06	0.00027	1811.54	2163.24	1872.26	2740.99	3388.65	2797.34 Up
Cxcl12	12334.6	0.49875	0.14143	3.52639	0.00042	0.00564	9487.59	12016.7	9166.37	13373.6	15608	14355.5 Up
Gm4640	30.145	1.5853	0.70214	2.25781	0.02396	0.11533	5.87528	29.5929	9.74627	34.6703	73.1512	27.8342 Up
Fxyd4	247.771	-0.689	0.26857	-2.5653	0.01031	0.06328	237.949	262.39	417.14	221.278	167.939	179.928 Down
Gm4875	32.4543	0.97533	0.43534	2.24041	0.02506	0.11897	29.3764	15.7829	20.4672	42.828	50.4846	35.7868 Up
Rasgef1a	5579.82	0.43883	0.12676	3.46197	0.00054	0.0068	4335.95	5143.24	4733.76	6017.34	7226.51	6022.13 Up
Ret	1207.14	-0.3906	0.12388	-3.1527	0.00162	0.01589	1384.61	1308.99	1415.16	1114.55	921.087	1098.46 Down
Cacna1c	2719.86	0.29247	0.1429	2.04667	0.04069	0.16654	2444.12	2740.3	2151	2626.79	3133.14	3223.8 Up
Dcp1b	1223.88	-0.227	0.11056	-2.0533	0.04004	0.16518	1338.58	1356.34	1265.07	1208.36	1042.66	1132.26 Down
Cacna2d4	468.92	-0.3437	0.16355	-2.1017	0.03558	0.15239	599.278	455.73	518.502	380.354	446.119	413.537 Down
Il17ra	2182.13	0.6876	0.18621	3.69259	0.00022	0.00347	1553.03	1978.78	1483.38	2509.52	3287.68	2280.42 Up
Slc25a18	391.874	-0.9968	0.26174	-3.8085	0.00014	0.00243	543.463	366.952	655.924	319.171	212.241	253.49 Down

Bid	787.591	0.28147	0.14322	1.96527	0.04938	0.18935	634.53	683.596	814.788	871.856	905.632	815.144 Up
Tuba8	952.012	0.5453	0.18428	2.95915	0.00308	0.02625	713.846	776.32	832.332	1324.61	1195.15	869.819 Up
M6pr	15299.9	0.40758	0.08477	4.80781	1.53E-06	5.75E-05	13659	13057.4	12742.3	17444.3	18246.6	16649.8 Up
Apobec1	1504.06	0.79064	0.17985	4.39599	1.10E-05	0.0003	867.582	1365.22	1073.06	1806.93	1975.08	1936.46 Up
Gdf3	10.5364	2.44634	1.08905	2.2463	0.02468	0.11763	7.8337	1.97286	0	17.3352	8.24239	27.8342 Up
Slc2a3	433.31	-0.6193	0.18164	-3.4094	0.00065	0.0079	454.355	520.835	599.396	344.664	386.362	294.247 Down
Gm5112	72.8393	-0.6863	0.26684	-2.5719	0.01011	0.06247	92.046	87.7922	89.6657	63.2223	54.6058	49.7039 Down
C3ar1	490.642	0.99302	0.19898	4.99065	6.02E-07	2.64E-05	278.096	395.558	310.906	635.282	562.543	761.464 Up
Clec4a3	870.357	0.77582	0.18429	4.20983	2.56E-05	0.0006	610.05	788.157	527.273	1214.48	1091.09	991.096 Up
Clec4a2	473.29	0.68426	0.19007	3.60005	0.00032	0.00457	282.013	436.002	371.333	564.922	608.906	576.566 Up
Clec4n	1976.25	1.25899	0.15057	8.3616	6.19E-17	2.34E-14	1261.23	1245.86	987.297	2919.44	3000.23	2443.45 Up
Clec4d	402.492	1.31728	0.30703	4.2904	1.78E-05	0.00045	223.261	284.092	184.205	422.162	847.936	453.3 Up
Clec4e	575.475	1.01796	0.32168	3.1645	0.00155	0.01543	392.664	509.984	238.784	529.232	1040.6	741.583 Up
Clstn3	249.568	-1.1787	0.33426	-3.5262	0.00042	0.00564	391.685	231.811	415.191	162.135	94.7875	201.798 Down
C1ra	4293.9	0.33831	0.15249	2.21859	0.02651	0.12395	3482.08	3642.88	4253.27	5357.58	3996.53	5031.03 Up
Gm15884	116.861	-0.7337	0.28188	-2.6029	0.00924	0.05837	183.113	116.399	138.397	75.4589	82.4239	105.372 Down
Ptpn6	3087.58	0.40057	0.14854	2.69681	0.007	0.04751	2488.18	3002.69	2494.07	3339.57	4068.65	3132.34 Up
Eno2	369.035	-1.171	0.24073	-4.8644	1.15E-06	4.47E-05	576.756	355.115	601.345	255.948	214.302	210.745 Down
Lrrc23	5357.14	-1.2768	0.27725	-4.6052	4.12E-06	0.00013	8873.63	5086.03	8793.09	3449.7	2058.54	3881.88 Down
Pianp	35.9489	0.94952	0.44045	2.15581	0.0311	0.13833	18.605	34.525	20.4672	60.1632	40.1816	41.7513 Up
Acrbp	572.45	-0.4153	0.15222	-2.7283	0.00637	0.04454	620.821	660.908	681.264	439.497	462.604	569.607 Down
Gapdh	2609.79	0.25171	0.11547	2.17992	0.02926	0.13281	2450.97	2297.39	2399.53	2874.58	3105.32	2530.92 Up
Vamp1	934.017	0.27354	0.13577	2.01468	0.04394	0.17523	777.495	935.135	824.535	962.611	1127.15	977.179 Up
Cd9	31236.4	0.44832	0.1493	3.00271	0.00268	0.02356	28421.7	25996.4	24847.1	36573.1	41870.3	29710 Up
Vwf	14002.2	0.30244	0.14081	2.14789	0.03172	0.14026	11677.1	14324.9	11617.6	17407.6	13867.8	15117.9 Up
Dyrk4	78.1906	-1.3612	0.37689	-3.6115	0.0003	0.00442	110.651	82.8601	144.245	46.9069	27.8181	56.6625 Down
Ccnd2	3441.18	0.30909	0.13024	2.37325	0.01763	0.09413	3283.3	2866.56	3072.03	3283.48	4049.07	4092.62 Up
Tulp3	2470.63	-0.6305	0.16301	-3.8675	0.00011	0.00201	3119.77	2410.83	3475.52	1950.71	1780.36	2086.57 Down
Fkbp4	11346.5	-0.2447	0.11358	-2.1544	0.03121	0.13858	13603.2	11825.3	11490.9	10474.5	9643.59	11041.2 Down
Gm10069	323.155	-0.58	0.22994	-2.5223	0.01166	0.06907	440.646	325.522	395.699	269.205	194.726	313.135 Down
Gm44009	83.2196	2.38224	0.47798	4.98402	6.23E-07	2.70E-05	40.1477	22.6879	17.5433	154.997	71.0906	192.851 Up
Gm44511	40.6705	0.84566	0.40538	2.08609	0.03697	0.15663	27.418	27.62	32.1627	52.0055	69.03	35.7868 Up
BC064078	1294.53	-0.7185	0.26139	-2.7487	0.00598	0.04264	1584.37	1233.04	2013.58	1098.23	1185.87	652.116 Down
Gm47861	5.54991	5.945	1.57742	3.76881	0.00016	NA	0	0	0	8.15772	8.24239	16.8993 Up
Clec12a	1539.43	0.37763	0.14303	2.64012	0.00829	0.05392	1141.76	1338.58	1536.99	1702.92	1676.3	1840.04 Up
Clec7a	7453.9	1.66355	0.29082	5.72031	1.06E-08	7.80E-07	3626.02	4661.87	2442.42	10063.6	15951.1	7978.47 Up
Olr1	1803.67	1.72082	0.21472	8.01417	1.11E-15	3.44E-13	765.744	1048.57	704.656	2350.44	3480.35	2472.27 Up
Klra11-ps	2.37639	3.65461	1.85048	1.97495	0.04827	NA	0.97921	0	0	4.07886	7.21209	1.98816 Up
Klra3	234.264	2.10877	0.87898	2.39912	0.01643	0.0894	95.9628	77.9279	90.6403	308.974	764.481	67.5973 Up
Klra2	427.479	0.77578	0.22521	3.44468	0.00057	0.00716	283.972	410.355	251.454	463.97	565.634	589.489 Up
Etv6	2514.74	0.31117	0.11762	2.64558	0.00816	0.05324	2277.65	2307.26	2149.05	2640.04	2587.08	3127.37 Up
Bcl2l14	878.141	0.55109	0.16133	3.41597	0.00064	0.00776	578.715	748.7	809.915	1037.05	988.056	1106.41 Up
Hebp1	1347.62	0.73522	0.15908	4.62159	3.81E-06	0.00012	1006.63	1191.61	836.23	1642.76	1804.05	1604.44 Up
Gm36640	48.5243	-0.8855	0.42583	-2.0796	0.03756	0.15821	71.4825	40.4436	76.9956	40.7886	22.6666	38.7691 Down
Dynlt1-ps	961.057	-0.6465	0.18463	-3.5014	0.00046	0.00606	1229.89	912.447	1376.17	826.989	687.209	733.63 Down
Grin2b	6.57569	-3.1121	1.35755	-2.2925	0.02188	NA	19.5843	13.81	1.94925	1.01971	3.0909	0 Down
Gm43969	4.7622	-2.5858	1.21001	-2.137	0.0326	NA	7.8337	5.91858	10.7209	2.03943	2.0606	0 Down
BC049715	108.355	-1.1533	0.36826	-3.1316	0.00174	0.01681	166.466	102.589	179.331	74.4392	86.5451	40.7572 Down
Pde6h	51.8649	-1.2753	0.47977	-2.6582	0.00785	0.05174	54.8359	59.1858	106.234	36.7097	15.4545	38.7691 Down
Dera	1119.3	0.23265	0.1176	1.97838	0.04789	0.18583	933.19	1025.89	1128.62	1223.66	1209.57	1194.88 Up
Pik3c2g	484.512	2.35279	0.27541	8.5428	1.31E-17	5.51E-15	151.778	218.001	106.234	907.546	638.785	884.73 Up
Plekha5	2214.73	-0.2541	0.11723	-2.1673	0.03021	0.13576	2572.39	2167.19	2488.22	2106.73	1878.23	2075.64 Down
Slco1a4	7.04648	-3.6934	1.24067	-2.9769	0.00291	NA	5.87528	20.715	12.6702	0	1.0303	1.98816 Down
Gys2	4.91241	-3.7532	1.84922	-2.0296	0.04239	NA	23.5011	2.95929	0.97463	2.03943	0	0 Down
Ldhb	13964	-0.9345	0.25286	-3.6959	0.00022	0.00343	16348.9	15503.7	23152.3	12180.5	6310.58	10287.7 Down
Gm31108	17.9089	-2.1963	0.81554	-2.693	0.00708	0.04791	58.7528	18.7422	10.7209	8.15772	4.12119	6.95855 Down
Sox5	595.991	-0.6225	0.16604	-3.7493	0.00018	0.00291	807.851	607.641	752.412	499.66	418.301	490.081 Down
Lrmp	631.34	0.41451	0.20617	2.01054	0.04437	0.17627	396.581	663.867	563.335	714.82	765.512	683.926 Up
Casc1	1408.1	-1.0216	0.2982	-3.426	0.00061	0.00755	2051.45	1227.12	2381.99	1093.13	593.452	1101.44 Down
Lmntd1	314.167	-1.1284	0.20351	-5.5447	2.94E-08	1.88E-06	529.754	363.993	399.597	183.549	213.272	194.839 Down
Tuba3b	2.76832	-4.891	2.44718	-1.9986	0.04565	NA	8.81291	0	7.79702	0	0	0 Down
Itpr2	2382.38	0.289	0.14481	1.99573	0.04596	0.18058	1940.8	2280.63	2212.4	2538.07	2292.41	3029.95 Up
Med21	1802.17	-0.2509	0.09732	-2.5782	0.00993	0.06155	2018.16	1929.46	1927.81	1753.91	1602.11	1581.58 Down
Mrps35	1251.73	-0.2083	0.10213	-2.04	0.04135	0.16842	1345.44	1308.99	1371.3	1220.6	1174.54	1089.51 Down
Klhl42	1344.72	-0.416	0.10994	-3.7842	0.00015	0.00262	1497.22	1437.23	1677.33	1174.71	1143.63	1138.22 Down
Gm15762	9.55792	4.82428	1.48617	3.24612	0.00117	0.01248	0	0	1.94925	24.4732	3.0909	27.8342 Up
Ergic2	1362.77	0.26572	0.13264	2.00331	0.04514	0.17836	1275.91	1268.55	1168.58	1350.1	1414.6	1698.88 Up
Caprin2	384.537	-0.3559	0.16327	-2.1798	0.02927	0.13281	454.355	393.585	447.354	313.052	312.18	386.697 Down
Amn1	1565.45	-0.3281	0.09542	-3.4382	0.00059	0.0073	1771.4	1688.77	1767.97	1416.38	1316.72	1431.47 Down
Bicd1	488.104	-0.3457	0.16182	-2.1363	0.03266	0.14348	523.879	502.093	613.041	398.709	401.816	489.087 Down
AU018091	40.8577	1.3608	0.45164	3.01303	0.00259	0.023	14.6882	36.4979	17.5433	62.2026	53.5755	60.6388 Up
Leng1	640.689	0.24599	0.1191	2.06535	0.03889	0.16209	604.174	613.559	540.918	704.623	681.027	699.831 Up
Tmc4	5732.6	0.65146	0.12939	5.03493	4.78E-07	2.15E-05	4862.77	3785.92	4730.84	7020.74	6693.85	7301.51 Up
Pirb	2314.77	1.06794	0.18646	5.72751	1.02E-08	7.50E-07	1469.8	1797.27	1218.28	2806.26	3713.2	2883.82 Up
Gm15922	49.4001	2.59207	0.40756	6.35998	2.02E-10	2.07E-08	15.6674	14.7964	11.6955	73.4195	110.242	70.5796 Up
Gm15448	41.5904	1.20338	0.44065	2.7309	0.00632	0.04434	16.6466	35.5115	23.3911	64.242	67.9997	41.7513 Up
Pira2	38.5026	1.33318	0.43561	3.06046	0.00221	0.0204	32.314	14.7964	18.5179	46.9069	61.8179	56.6625 Up
Gm14548	31.0964	0.94649	0.40269	2.35043	0.01875	0.09795	20.5635	24.6607	18.5179	36.7097	46.3634	39.7631 Up
Lair1	694.379	0.84062	0.21692	3.87529	0.00011	0.00196	372.101	647.098	473.669	858.6	978.784	836.02 Up
Lilra5	1196.59	1.37098	0.23801	5.7601	8.41E-09	6.29E-07	646.28	887.787	467.821	1556.08	2012.17	1609.41 Up
Gm15931	211.916	1.4071	0.24168	5.82209	5.81E-09	4.52E-07	113.589	135.141	99.412	291.638	381.21	250.508 Up
Gp6	80.3234	-0.7905	0.38553	-2.0504	0.04032	0.16567	74.4202	77.9279	153.016	46.9069	69.03	60.6388 Down
Tnni3	1120.8	-1.5985	0.60802	-2.6291	0.00856	0.05526	2381.45	621.451	2052.57	553.705	376.059	739.594 Down

Syt5	2368.44	-1.0668	0.26111	-4.0857	4.39E-05	0.00094	3517.33	2155.35	3946.27	1606.05	1114.78	1870.86	Down
Hspbp1	1920.5	-0.3184	0.11544	-2.7578	0.00582	0.04183	2267.86	1957.08	2169.52	1747.79	1806.11	1574.62	Down
Tmem150	112.487	1.16661	0.31517	3.70159	0.00021	0.00337	72.4617	85.8194	49.706	149.898	195.757	121.278	Up
Fam71e2	13.3494	-1.6586	0.65782	-2.5214	0.01169	0.06922	14.6882	28.6065	17.5433	6.11829	6.18179	6.95855	Down
Isoc2a	1240.48	0.39912	0.17707	2.25397	0.0242	0.11619	957.67	1292.22	960.008	1399.05	1607.27	1226.69	Up
Nat14	250.118	-0.7676	0.20126	-3.8143	0.00014	0.00239	369.163	269.295	307.008	169.273	177.211	208.756	Down
Sbk3	159.184	-1.4529	0.6455	-2.2508	0.0244	0.11677	281.034	36.4979	382.054	70.3603	63.8785	121.278	Down
Epp13	61.3277	-2.1244	0.52736	-4.0285	5.61E-05	0.00115	118.485	49.3215	131.575	23.4534	11.3333	33.7987	Down
Zfp446	690.218	-0.2358	0.11824	-1.9945	0.0461	0.18095	800.017	733.904	705.63	651.598	610.967	639.192	Down
Mzf1	174.089	-0.7767	0.23995	-3.2369	0.00121	0.01279	245.782	213.069	200.773	150.918	95.8178	138.177	Down
Nlrp5-ps	68.3043	0.80789	0.33256	2.42929	0.01513	0.08391	34.2724	57.2129	57.503	73.4195	99.9389	87.4789	Up
Zfp541	3.7025	3.36527	1.55123	2.16942	0.03005	NA	0	1.97286	0	7.138	5.15149	7.95263	Up
Meis3	818.657	0.42921	0.13599	3.15613	0.0016	0.01577	669.782	687.541	735.844	1007.48	992.177	819.121	Up
C5ar2	61.3805	0.88329	0.42654	2.07084	0.03837	0.16044	48.9606	52.2808	28.2642	47.9266	84.4845	106.366	Up
Inafm1	1168.27	-0.2535	0.12579	-2.0151	0.04389	0.1751	1194.64	1241.91	1375.2	1057.44	1164.24	976.185	Down
Bbc3	1024.74	-0.5688	0.11311	-5.0289	4.93E-07	2.20E-05	1300.39	1127.49	1244.6	852.482	824.239	799.239	Down
Ptgir	386.135	1.85607	0.28615	6.48626	8.80E-11	9.57E-09	150.799	227.865	122.803	610.809	755.209	449.323	Up
Qpctl	901.153	0.30092	0.10194	2.95202	0.00316	0.02666	800.017	799.994	822.585	998.301	957.147	1028.87	Up
Eml2	6364.45	-0.3403	0.13435	-2.5327	0.01132	0.06768	7735.78	6014.26	7584.55	5961.25	5251.43	5639.41	Down
Ckm	170.153	-3.1389	1.35134	-2.3228	0.02019	0.10288	515.066	20.715	381.079	15.2957	8.24239	80.5204	Down
Exoc3l2	193.431	0.46809	0.23045	2.03118	0.04224	0.17074	125.339	165.72	195.9	249.83	207.09	216.709	Up
Relb	1759.11	0.37637	0.16095	2.33845	0.01936	0.09988	1549.11	1792.34	1251.42	1827.33	2122.41	2012.01	Up
Cblc	1607.14	0.82401	0.15651	5.26498	1.40E-07	7.45E-06	1236.75	1166.95	1076.96	2282.12	2209.99	1670.05	Up
Nlrp4e	90.3711	-1.0074	0.4036	-2.496	0.01256	0.07296	133.173	158.815	70.1732	69.3406	39.1513	71.5737	Down
Lypd5	67.434	-0.9319	0.34211	-2.7241	0.00645	0.04491	70.5033	80.8872	114.031	57.104	44.3028	37.775	Down
Kcnn4	417.45	-0.5692	0.20944	-2.7177	0.00657	0.04559	513.107	386.68	596.472	364.038	289.514	354.886	Down
Plaur	3844.38	0.64504	0.17975	3.58848	0.00033	0.00473	3508.52	3035.24	2453.14	4853.84	5328.7	3886.85	Up
AC16116	14.707	-1.1472	0.58341	-1.9664	0.04925	0.18906	23.5011	19.7286	17.5433	9.17743	11.3333	6.95855	Down
Ceacam1(	620.059	2.50116	1.15449	2.16645	0.03028	0.13596	307.473	45.3758	205.646	1129.84	116.424	1915.59	Up
4732471J(	303.112	0.56113	0.17967	3.12311	0.00179	0.01719	224.24	221.947	288.49	332.427	368.847	382.72	Up
B3gnt8	275.089	0.71644	0.21656	3.30835	0.00094	0.01045	185.071	223.92	215.393	333.447	419.331	273.372	Up
Tmem91	28.7265	-1.1834	0.51119	-2.315	0.02062	0.10443	44.0646	48.3351	27.2896	24.4732	10.303	17.8934	Down
B9d2	819.192	-0.3836	0.15751	-2.4352	0.01489	0.08302	936.127	826.628	1019.46	803.535	716.057	613.346	Down
Tgfb1	3772.5	0.466	0.18933	2.46137	0.01384	0.07863	2926.87	3806.63	2771.84	4184.91	5316.34	3628.39	Up
Axl	6545.68	0.99747	0.16559	6.02391	1.70E-09	1.45E-07	4117.59	5052.49	3936.52	8101.63	10418.4	7647.45	Up
Gm29763	32.4854	-1.2494	0.41413	-3.0169	0.00255	0.02277	39.1685	51.2943	46.7821	18.3549	15.4545	23.8579	Down
Cyp2s1	15906.4	-0.7922	0.24602	-3.2201	0.00128	0.01336	20808.3	15615.2	24078.2	14098.6	7729.3	13108.9	Down
Gm30146	27.1169	-2.0625	0.50012	-4.1239	3.72E-05	0.00082	45.0438	39.4572	46.7821	15.2957	6.18179	9.94079	Down
Cyp2b19	125.528	-1.2823	0.36751	-3.489	0.00048	0.0063	214.448	135.141	184.205	90.7546	39.1513	89.4671	Down
Cyp2g1	46.8781	-1.6249	0.45313	-3.586	0.00034	0.00476	93.0252	41.43	77.9702	26.5126	16.4848	25.846	Down
Cyp2f2	74984.2	-0.6593	0.26231	-2.5134	0.01196	0.07039	84666.7	67459.9	123349	68649.2	40145.6	65635	Down
Numbl	947.76	-0.2951	0.14589	-2.023	0.04307	0.17298	963.545	969.66	1199.77	905.507	873.693	774.387	Down
Shkbp1	1654.79	0.33609	0.15717	2.1384	0.03248	0.14292	1245.56	1619.72	1523.34	1826.31	2090.48	1623.33	Up
Blvrb	1393.86	0.34718	0.16569	2.09534	0.03614	0.15417	1063.43	1255.72	1361.55	1615.23	1766.96	1300.25	Up
Pld3	9800.22	0.96632	0.15898	6.07816	1.22E-09	1.06E-07	6634.17	7491.93	5780.51	12839.2	15035.1	11020.4	Up
9530053A	805.143	-0.6348	0.26913	-2.3588	0.01833	0.09644	1083.01	630.329	1225.11	757.648	495.574	639.192	Down
Eid2b	439.97	-0.3371	0.14399	-2.3412	0.01922	0.09958	447.5	534.645	491.212	387.492	413.15	365.821	Down
Supt5	1778.96	0.31469	0.13776	2.28435	0.02235	0.10989	1790	1419.47	1547.71	2007.82	1782.42	2126.33	Up
Acp7	104.593	-1.6185	0.56526	-2.8633	0.00419	0.03279	218.364	59.1858	195.9	46.9069	23.6969	83.5026	Down
Gm44618	31.3042	-1.3607	0.50116	-2.715	0.00663	0.04576	66.5865	33.5386	35.0866	19.3746	12.3636	20.8756	Down
Lgals4	650.759	-0.4357	0.14288	-3.0497	0.00229	0.02097	702.096	726.999	815.763	618.967	506.907	533.82	Down
Actn4	16055.1	0.30598	0.08914	3.43255	0.0006	0.00742	15077.9	14383.1	13615.5	17689	18548.5	17016.6	Up
Catsperg1	315.007	-0.3865	0.16409	-2.3552	0.01851	0.09719	385.81	317.63	367.434	249.83	275.09	294.247	Down
Kcnk6	1328.08	0.49264	0.13162	3.74279	0.00018	0.00298	1203.45	1133.41	973.653	1446.98	1671.14	1539.83	Up
Dpf1	51.5435	-0.9261	0.40814	-2.2691	0.02326	0.11297	76.3786	40.4436	85.7672	40.7886	36.0604	29.8224	Down
Zfp74	1107.97	-0.4229	0.19757	-2.1404	0.03232	0.14241	1464.9	955.85	1386.89	935.079	832.481	1072.61	Down
Zfp568	1243.01	0.23726	0.10907	2.17526	0.02961	0.13386	1096.72	1144.26	1182.22	1259.35	1331.15	1444.4	Up
Syne4	925.638	-0.5009	0.20492	-2.4443	0.01451	0.08156	1198.56	826.628	1229.01	892.251	643.937	763.452	Down
Tyrobp	2652.39	1.02751	0.24373	4.21576	2.49E-05	0.00059	1642.14	2281.61	1313.8	3435.42	4482.83	2758.57	Up
Zbtb32	414.716	-0.6206	0.26662	-2.3275	0.01994	0.10206	319.223	520.835	667.62	331.407	313.211	335.999	Down
Upk1a	105.453	-1.3909	0.34532	-4.028	5.62E-05	0.00115	133.173	105.548	219.291	63.2223	60.7876	50.698	Down
Tmem147	2545.67	-0.2404	0.08815	-2.7272	0.00639	0.04464	2768.23	2834.01	2669.5	2415.7	2343.93	2242.64	Down
Ffar2	347.733	0.94974	0.29741	3.19336	0.00141	0.01426	331.953	216.028	163.737	342.624	542.967	489.087	Up
Cd22	1180.47	1.17577	0.19222	6.11668	9.55E-10	8.57E-08	584.59	897.651	691.011	1454.11	1867.93	1587.54	Up
Mag	171.151	0.97046	0.18661	5.20056	1.99E-07	1.01E-05	122.402	116.399	108.184	242.692	221.514	215.715	Up
Lsr	1608.94	0.35007	0.11028	3.17445	0.0015	0.015	1535.41	1313.92	1394.69	1891.57	1753.57	1764.49	Up
Fxyd5	5058.83	0.67099	0.16656	4.02863	5.61E-05	0.00115	4301.68	4040.42	3367.34	6164.18	7316.15	5163.24	Up
Fxyd1	1223.31	-0.2854	0.12058	-2.3674	0.01792	0.09518	1352.29	1232.05	1447.32	1062.54	1187.93	1057.7	Down
Scn1b	1438.32	-0.7073	0.19411	-3.6439	0.00027	0.004	1800.77	1364.23	2187.06	1105.37	973.632	1198.86	Down
4931406P	1214.62	0.49422	0.14986	3.29791	0.00097	0.01076	1054.61	945	1026.28	1301.16	1281.69	1679	Up
Kctd15	820.677	-0.2662	0.13226	-2.013	0.04412	0.17563	949.836	802.954	935.642	731.136	804.663	699.831	Down
Cep89	1085.8	-0.8783	0.23855	-3.6816	0.00023	0.0036	1664.66	913.434	1641.27	786.2	684.118	825.085	Down
Nudt19	938.615	-0.5751	0.21758	-2.6431	0.00822	0.0536	1227.93	775.334	1366.43	794.358	745.936	721.701	Down
Pdcd5	1011.32	-0.586	0.21496	-2.7261	0.00641	0.04472	1423.78	867.072	1350.83	849.423	881.935	694.861	Down
Dpy19l3	1085.9	-0.7101	0.17186	-4.1317	3.60E-05	0.0008	1609.83	1322.8	1111.08	828.008	749.027	894.671	Down
Tshz3	457.301	-0.3349	0.15173	-2.2071	0.0273	0.12624	559.13	459.676	511.679	421.142	364.726	427.454	Down
Zfp536	21.9025	-1.3294	0.59097	-2.2495	0.02448	0.11694	47.9814	20.715	25.3403	12.2366	8.24239	16.8993	Down
Gm30684	22.0851	4.42855	0.83705	5.29065	1.22E-07	6.55E-06	4.89606	0.98643	0	50.9857	30.909	44.7335	Up
AI987944	916.016	0.34681	0.10971	3.16123	0.00157	0.01557	779.453	865.099	774.829	995.242	1042.66	1038.81	Up
Zfp715	2122.84	0.34811	0.10535	3.30423	0.00095	0.01057	1729.29	1993.57	1881.03	2261.73	2471.69	2399.71	Up
Siglecf	1013.79	1.05327	0.3528	2.98547	0.00283	0.0246	753.994	883.841	340.145	1220.6	1905.02	979.167	Up
Nkg7	230.038	-0.6	0.29249	-2.0512	0.04025	0.16558	217.385	337.359	276.794	128.484	246.241	173.964	Down
Cd33	2106.44	1.1933	0.15424	7.73665	1.02E-14	2.48E-12	1392.44	1366.21	1086.71	2563.56	3275.32	2954.4	Up

Zfp658	470.277	0.67579	0.16947	3.9877	6.67E-05	0.00133	345.662	412.328	328.449	535.35	537.816	662.056 Up
Siglece	307.54	0.73271	0.20735	3.53374	0.00041	0.00554	209.552	271.268	212.469	309.993	423.453	418.507 Up
2310002F	230.178	-0.748	0.36388	-2.0558	0.03981	0.16457	357.413	210.11	298.236	144.8	105.09	265.419 Down
Klk1b3	7.94249	2.82089	1.29547	2.1775	0.02944	0.13336	0	5.91858	0	17.3352	15.4545	8.94671 Up
Lrrc4b	30.7002	-1.1295	0.51817	-2.1799	0.02927	0.13281	48.9606	39.4572	38.0105	8.15772	25.7575	23.8579 Down
5430431A	58.7236	-1.1431	0.55509	-2.0592	0.03947	0.16363	84.2123	40.4436	117.93	25.4929	21.6363	62.6269 Down
Napsa	149.528	0.61012	0.27874	2.18883	0.02861	0.13081	125.339	148.951	80.8941	170.292	166.908	204.78 Up
Ptov1	7075.93	-0.231	0.09243	-2.4993	0.01245	0.07249	7623.17	7878.61	7421.79	6984.03	6354.88	6193.11 Down
Fuz	783.581	-0.6027	0.15534	-3.88	0.0001	0.00193	983.13	797.035	1054.55	677.091	587.27	602.412 Down
Prmt1	3447.21	-0.2222	0.09296	-2.3906	0.01682	0.09092	3751.36	3698.12	3687.02	3420.12	3085.74	3040.89 Down
Fcgrt	2106	0.40189	0.10421	3.85674	0.00011	0.00208	1751.81	1946.23	1745.56	2345.34	2542.78	2304.27 Up
Flt3l	6.73084	-2.4926	1.04146	-2.3934	0.01669	NA	19.5843	6.90501	7.79702	1.01971	3.0909	1.98816 Down
Slc17a7	39.7621	-2.1429	1.0835	-1.9777	0.04796	0.18606	118.485	4.93215	71.1478	2.03943	6.18179	35.7868 Down
Gfy	48.211	-1.9054	0.3585	-5.315	1.07E-07	5.88E-06	81.2747	67.0772	79.9194	23.4534	21.6363	15.9053 Down
Ccdc155	366.625	-1.7624	0.22471	-7.8428	4.41E-15	1.21E-12	496.461	475.459	727.072	180.49	146.302	173.964 Down
Dkkl1	398.768	-1.3962	0.19966	-6.993	2.69E-12	3.94E-10	576.756	512.943	644.229	171.312	233.878	253.49 Down
Ppfia3	236.628	-0.6836	0.3151	-2.1696	0.03004	0.13517	342.724	171.639	360.612	220.258	154.545	169.987 Down
AC151602	1344.52	-0.8321	0.29654	-2.8062	0.00501	0.03745	2073.97	1077.18	2014.55	1060.5	647.027	1193.89 Down
Ruvbl2	3817.48	-0.6127	0.13768	-4.4501	8.58E-06	0.00025	4747.22	3998	5103.15	3234.54	2705.56	3116.44 Down
Ftl1	44920.1	0.36191	0.15701	2.305	0.02117	0.10633	36564.8	38296.2	43084.4	50571.7	59592.5	41411.3 Up
Ppp1r15a	2178.49	0.45144	0.15517	2.90929	0.00362	0.02958	1783.15	1976.8	1761.15	2910.27	2578.84	2060.72 Up
Sec1	369.121	0.95005	0.20657	4.59911	4.24E-06	0.00014	244.803	225.892	284.591	426.241	603.755	429.442 Up
Car11	142.191	0.69357	0.35126	1.97455	0.04832	0.18675	94.0044	164.734	67.2493	160.095	205.029	162.035 Up
Fam83e	2034.73	0.82581	0.28627	2.8847	0.00392	0.03124	1728.31	781.252	1893.7	2619.65	2610.78	2574.66 Up
Sult2b1	307.216	-0.7083	0.22466	-3.1529	0.00162	0.01589	401.477	441.92	300.185	245.751	188.545	265.419 Down
Grin2d	226.583	0.71406	0.22767	3.13633	0.00171	0.01663	134.152	216.028	164.712	265.126	309.09	270.389 Up
Emp3	1684.46	0.41417	0.14774	2.80333	0.00506	0.0377	1477.63	1509.24	1345.96	1998.64	2171.87	1603.45 Up
Ccdc114	2830.04	-0.6411	0.24372	-2.6306	0.00852	0.0551	3552.58	2482.84	4310.78	2584.98	1616.54	2432.51 Down
Abcc6	33.6722	-2.232	0.45477	-4.908	9.20E-07	3.71E-05	70.5033	49.3215	46.7821	12.2366	9.27269	13.9171 Down
Saa3	921.61	2.03143	0.3781	5.37266	7.76E-08	4.54E-06	262.429	227.865	596.472	1481.65	958.178	2003.07 Up
Saa4	16.3444	1.66624	0.66684	2.49872	0.01246	0.07256	7.8337	6.90501	8.77165	19.3746	14.4242	40.7572 Up
Saa1	16.5959	1.53816	0.58525	2.62822	0.00858	0.05534	6.85449	11.8372	6.82239	30.5914	20.606	22.8638 Up
Tmem86a	895.972	0.45382	0.22217	2.04265	0.04109	0.1677	607.112	703.324	958.059	1123.73	1171.45	812.162 Up
Mrgprb1	3.92471	-4.3682	1.8018	-2.4243	0.01534	NA	16.6466	1.97286	3.89851	0	1.0303	0 Down
Nell1	83.1204	-2.0016	0.74716	-2.6789	0.00739	0.04945	251.658	23.6743	123.778	27.5323	14.4242	57.6566 Down
Gm45235	23.5911	-1.1671	0.54975	-2.123	0.03375	0.14684	27.418	28.6065	41.909	7.138	19.5757	16.8993 Down
Cyfip1	6368.5	0.26976	0.09783	2.75747	0.00583	0.04185	5826.32	5980.72	5517.37	6719.92	7523.24	6643.43 Up
Nipa2	2232.25	0.45417	0.12123	3.74647	0.00018	0.00294	2068.1	1824.89	1758.23	2406.53	2814.78	2520.98 Up
Nipa1	315.824	-0.4524	0.22532	-2.0077	0.04468	0.17701	434.77	261.404	398.623	266.146	259.635	274.366 Down
Gm34121	82.1835	-0.8375	0.37382	-2.2403	0.02507	0.11898	121.422	71.0229	123.778	61.1829	39.1513	76.544 Down
Snhg14	540.639	-0.4948	0.16766	-2.9514	0.00316	0.02669	565.006	576.075	756.311	452.753	424.483	469.205 Down
A230057D	1314.77	-0.5691	0.20302	-2.8034	0.00506	0.0377	1410.07	1240.93	2061.34	1123.73	1011.75	1040.8 Down
A330076H	336.108	-0.4949	0.24555	-2.0154	0.04386	0.17506	373.08	282.119	524.349	286.54	278.181	272.378 Down
Mtmr10	314.879	0.41224	0.16059	2.56709	0.01026	0.0631	296.701	264.363	249.505	330.388	389.453	358.862 Up
Mcee	1638.56	-0.4156	0.1624	-2.5588	0.0105	0.0642	2048.51	1569.41	2000.91	1547.93	1201.33	1463.28 Down
Tjp1	16395.9	0.29478	0.10565	2.7903	0.00527	0.03886	13501.4	15563.9	15114.5	17049.6	19371.7	17774.1 Up
Aldh1a3	65.5561	-1.0512	0.38943	-2.6994	0.00695	0.04729	109.672	52.2808	103.31	45.8872	47.3937	34.7927 Down
Cers3	91.564	1.12721	0.28114	4.00938	6.09E-05	0.00123	47.9814	62.1451	62.3761	108.09	152.484	116.307 Up
Adamts17	169.414	-0.5809	0.27744	-2.0939	0.03627	0.15453	230.115	239.702	139.372	156.016	134.969	116.307 Down
Gm44889	28.6469	-3.1637	0.936	-3.38	0.00072	0.00852	74.4202	23.6743	56.5284	14.276	0	2.98224 Down
4933436H	66.128	-3.2581	0.55386	-5.8826	4.04E-09	3.25E-07	194.863	51.2943	113.057	16.3154	10.303	10.9349 Down
Gm44808	126.004	-1.766	0.38059	-4.6401	3.48E-06	0.00011	210.531	124.29	249.505	57.104	36.0604	78.5322 Down
Gm45007	6.73334	-2.4925	0.99284	-2.5105	0.01206	NA	11.7506	10.8507	11.6955	4.07886	1.0303	0.99408 Down
Fam174b	10331.5	-0.2268	0.09795	-2.3152	0.0206	0.10443	11004.4	11788.8	10632.2	10202.2	9353.05	9008.34 Down
Gm10619	123.954	-0.6446	0.22368	-2.882	0.00395	0.03141	143.944	137.114	172.509	88.7152	103.03	98.4138 Down
St8sia2	1041.15	-0.7191	0.21248	-3.3842	0.00071	0.00841	1590.24	954.864	1341.09	835.146	668.664	856.896 Down
Akap13	11992.9	0.35601	0.14802	2.40514	0.01617	0.08832	8988.19	12083.8	10489.9	12423.2	15095.9	12876.3 Up
Isg20	2714.94	-0.4787	0.17495	-2.7362	0.00621	0.04389	3332.26	2671.25	3480.39	2493.2	1816.42	2496.13 Down
Mfge8	13094	0.31146	0.11212	2.77795	0.00547	0.03991	10737.1	13129.4	11191.6	14080.2	14953.8	14471.8 Up
Polg	2298.52	0.2531	0.10453	2.4214	0.01546	0.08535	2182.67	2094.19	2015.53	2584.98	2291.38	2622.38 Up
Plin1	147.449	-4.4417	1.54601	-2.873	0.00407	0.03201	733.43	20.715	91.615	17.3352	20.606	0.99408 Down
Wdr93	871.584	-1.5327	0.30257	-5.0657	4.07E-07	1.87E-05	1535.41	832.547	1518.47	456.832	297.756	588.494 Down
Mesp1	272.516	-0.8763	0.2263	-3.8721	0.00011	0.00198	343.704	436.988	277.769	173.352	217.393	185.893 Down
Mesp2	206.654	-0.7572	0.25897	-2.924	0.00346	0.02851	236.969	339.332	202.722	133.583	177.211	150.106 Down
Zfp710	2140.03	0.40062	0.12578	3.18501	0.00145	0.01457	1731.25	1962.01	1841.07	2205.64	2731.32	2368.89 Up
Cib1	4593.75	0.27553	0.11301	2.43817	0.01476	0.08255	4248.8	3780.98	4439.43	5410.61	4703.31	4979.34 Up
Gdpgp1	397.381	0.42728	0.19463	2.19533	0.02814	0.12925	315.307	394.572	307.008	382.393	533.695	451.312 Up
Ttll13	192.484	-1.3974	0.27095	-5.1574	2.50E-07	1.23E-05	293.764	211.096	332.348	107.07	81.3936	129.23 Down
Ngrn	2104.17	-0.5019	0.146	-3.4381	0.00059	0.0073	2737.88	2206.64	2455.09	2000.68	1638.17	1586.55 Down
Man2a2	2498.31	0.90907	0.10662	8.52638	1.51E-17	6.08E-15	1767.48	1690.74	1750.43	3000	3551.44	3229.76 Up
Fes	2713.36	0.75874	0.13566	5.59307	2.23E-08	1.48E-06	2084.74	2109.97	1852.77	3307.96	3900.71	3023.99 Up
Furin	5923.28	0.55475	0.14214	3.90284	9.51E-05	0.00178	4966.57	4853.23	4574.9	6836.17	8285.66	6023.12 Up
Iqgap1	35080.8	0.39306	0.10963	3.58531	0.00034	0.00477	30237.1	32214.8	28542	37874.3	43994.8	37621.9 Up
Gm37829	33.0462	-1.7837	0.54654	-3.2636	0.0011	0.01186	61.6904	28.6065	63.3508	22.4337	9.27269	12.923 Down
Fsd2	307.912	-2.1511	0.37814	-5.6886	1.28E-08	9.14E-07	616.904	267.322	623.761	154.997	73.1512	111.337 Down
Homer2	228.66	-1.0625	0.28254	-3.7604	0.00017	0.00282	361.33	348.21	218.317	150.918	111.272	181.916 Down
Tm6sf1	703.659	0.9325	0.19562	4.76694	1.87E-06	6.78E-05	469.043	581.993	400.572	764.786	1032.36	973.203 Up
Saxo2	3986.76	-1.3687	0.3268	-4.1883	2.81E-05	0.00065	7551.69	3427.84	6263.93	2315.77	1365.15	2996.15 Down
Gm16638	140.303	-0.6382	0.26284	-2.4281	0.01518	0.08407	126.318	187.422	198.824	113.188	94.7875	121.278 Down
Stard5	2128.54	0.77556	0.10646	7.28498	3.22E-13	5.80E-11	1500.15	1520.09	1689.03	2649.22	2854.96	2557.76 Up
Cfap161	1415.78	-1.307	0.27894	-4.6857	2.79E-06	9.53E-05	2348.15	1388.89	2312.79	889.191	535.755	1019.92 Down
Tlnrd1	2555.72	0.38917	0.10991	3.54068	0.0004	0.00542	2162.1	2333.89	2143.21	2784.84	3181.56	2728.75 Up
Arnt2	280.141	1.09297	0.35474	3.08103	0.00206	0.01923	235.99	189.394	111.108	230.456	490.422	423.477 Up

Fah	2406.32	-0.5624	0.1572	-3.5778	0.00035	0.00486	3238.26	2773.84	2596.41	2294.36	1756.66	1778.41	Down
Vmn2r71	1.95865	-4.3918	2.07682	-2.1147	0.03446	NA	1.95843	3.94572	5.84776	0	0	0	Down
Folh1	61.2136	-1.323	0.48768	-2.7128	0.00667	0.04601	51.8983	74.9687	135.473	43.8477	41.2119	19.8816	Down
Nox4	3279.96	0.28777	0.13486	2.13379	0.03286	0.14414	2610.58	2842.89	3408.27	3365.06	3743.07	3709.9	Up
Grm5	21.5539	-2.1086	0.62139	-3.3933	0.00069	0.00824	26.4387	48.3351	30.2134	13.2563	4.12119	6.95855	Down
Gm44751	1372.55	-0.6541	0.23828	-2.7448	0.00605	0.04303	1849.73	1590.12	1595.46	1502.04	821.148	876.777	Down
Prss23	2407.17	-0.4797	0.22607	-2.1217	0.03386	0.14716	3057.1	2461.14	2892.69	2516.66	2159.51	1355.92	Down
Me3	245.914	-1.0082	0.29494	-3.4183	0.00063	0.00772	360.35	274.227	350.866	234.534	142.181	113.325	Down
Ccdc81	807.31	-0.9509	0.28236	-3.3675	0.00076	0.00884	1491.34	849.316	851.824	441.537	482.18	727.665	Down
Hikeshi	1452.76	-0.2351	0.11836	-1.9863	0.047	0.18333	1638.22	1572.37	1501.9	1467.37	1322.9	1213.77	Down
Picalm	15849.8	0.2598	0.09559	2.71794	0.00657	0.04557	15115.1	14806.3	13358.2	16389.9	17769.6	17659.8	Up
Sytl2	1227.81	-0.8458	0.20526	-4.1205	3.78E-05	0.00083	1514.84	1898.88	1319.65	958.532	676.906	998.055	Down
Ccdc89	194.353	-0.8613	0.33639	-2.5604	0.01046	0.064	369.163	159.802	223.19	129.504	117.454	167.005	Down
Ankrd42	1619.35	-0.719	0.21982	-3.2708	0.00107	0.01165	2184.62	1557.57	2302.07	1319.51	913.875	1438.43	Down
Prcp	2999.21	0.45888	0.1249	3.6741	0.00024	0.00368	2403.97	2793.57	2381.01	3707.68	3590.59	3118.42	Up
Gm15501	149.566	-0.4796	0.22627	-2.1197	0.03403	0.14769	192.905	181.503	148.143	140.721	127.757	106.366	Down
Gab2	2658.16	0.30821	0.14632	2.10646	0.03516	0.1511	2113.14	2704.79	2307.92	2756.29	3336.11	2730.73	Up
Usp35	675.273	0.29641	0.13326	2.22421	0.02613	0.12269	556.193	656.962	605.244	710.741	807.754	714.742	Up
Ndufc2	5476.27	-0.2801	0.07945	-3.5256	0.00042	0.00564	6057.41	5905.75	6055.36	5097.55	4992.83	4748.71	Down
Thrsp	1435.22	-2.1701	0.3297	-6.5821	4.64E-11	5.56E-09	3368.49	1466.82	2210.45	740.313	371.938	453.3	Down
Rsf1	37.9559	-1.1429	0.36649	-3.1187	0.00182	0.01737	53.8567	47.3486	55.5538	26.5126	20.606	23.8579	Down
Pak1	2582.98	0.54242	0.11333	4.78639	1.70E-06	6.24E-05	2279.61	2063.61	1965.82	3105.03	3253.68	2830.14	Up
Gdpd4	10.5013	2.44277	1.15639	2.11242	0.03465	0.14947	6.85449	0	2.92388	19.3746	29.8787	3.97631	Up
Myo7a	1413.45	1.04326	0.18659	5.59121	2.25E-08	1.49E-06	909.689	926.257	934.668	1521.41	2421.2	1767.47	Up
Capn5	1762.68	0.4212	0.14248	2.95626	0.00311	0.02641	1487.42	1460.9	1573.05	1962.95	2350.11	1741.63	Up
B3gnt6	52.7677	-0.8574	0.3528	-2.4304	0.01508	0.08371	68.5449	81.8737	53.6045	45.8872	30.909	35.7868	Down
Acer3	4034.36	0.34533	0.10634	3.24736	0.00116	0.01245	3411.58	3885.55	3364.41	4261.39	4595.13	4688.07	Up
Tsku	1178.75	-0.5475	0.2783	-1.9673	0.04915	0.189	1300.39	931.19	1967.77	978.926	749.027	1145.18	Down
Lrrc32	2802.63	0.40222	0.09596	4.19158	2.77E-05	0.00064	2340.32	2505.53	2397.58	2977.57	3310.35	3284.44	Up
Wnt11	1844.81	-1.06	0.28471	-3.723	0.0002	0.00315	2614.5	1548.69	3317.63	1369.48	870.602	1347.97	Down
Uvrag	3198.08	0.20535	0.08039	2.55437	0.01064	0.06482	2980.72	3036.23	2895.62	3369.14	3504.05	3402.73	Up
Gdpd5	1345.32	0.27866	0.11449	2.43385	0.01494	0.0832	1238.7	1242.9	1165.65	1491.84	1591.81	1341.01	Up
Slco2b1	932.633	0.53347	0.16937	3.14971	0.00163	0.01602	740.285	670.772	875.215	1065.6	1280.66	963.262	Up
Neu3	371.143	-0.3613	0.18064	-2.0002	0.04548	0.17928	424.978	473.486	353.79	309.993	298.787	365.821	Down
Xrra1	175.851	-0.7981	0.22882	-3.4878	0.00049	0.00632	222.281	182.489	265.099	140.721	112.303	132.212	Down
Ucp3	397.929	1.46799	0.34265	4.28428	1.83E-05	0.00046	251.658	261.404	120.854	545.547	800.542	407.572	Up
Dnajb13	1763.22	-0.5852	0.22691	-2.5788	0.00991	0.06145	2728.09	1557.57	2062.31	1554.05	1137.45	1539.83	Down
Plekhb1	10035.8	-0.3794	0.09992	-3.7974	0.00015	0.00251	11654.6	11102.3	11287.2	8900.07	7933.3	9337.38	Down
Relt	384.824	0.41238	0.2056	2.00569	0.04489	0.17771	262.429	329.467	398.623	418.083	500.725	399.62	Up
P2ry6	952.495	0.62328	0.21668	2.87653	0.00402	0.0318	635.509	991.362	622.787	1119.65	1318.78	1026.88	Up
P2ry2	1558.02	0.78599	0.1252	6.27779	3.43E-10	3.26E-08	1200.51	1220.21	1010.69	1903.81	2115.2	1897.7	Up
Fchsd2	495.68	0.58383	0.20072	2.90867	0.00363	0.02962	440.646	331.44	418.115	471.108	638.785	673.985	Up
Stard10	5007.35	-0.215	0.10593	-2.0295	0.04241	0.17118	5738.19	4941.03	5459.86	4933.38	4554.95	4416.69	Down
Art5	13.2954	-1.7603	0.83818	-2.1001	0.03572	0.15281	24.4803	7.89144	29.2388	2.03943	6.18179	9.94079	Down
Art1	10.7589	-3.5747	1.54677	-2.3111	0.02083	0.10511	25.4595	0.98643	33.1373	0	0	4.97039	Down
Chrna10	9.68227	-2.2393	0.85631	-2.6151	0.00892	0.05693	19.5843	6.90501	21.4418	3.05914	4.12119	2.98224	Down
Trim6	289.984	0.78633	0.21979	3.57759	0.00035	0.00486	191.926	266.336	180.306	401.768	388.423	311.147	Up
Trim34a	296.263	2.20553	0.45117	4.8885	1.02E-06	4.02E-05	117.506	115.412	83.8179	164.174	661.452	635.216	Up
Trim5	182.22	2.81334	0.90309	3.11524	0.00184	0.01752	51.8983	37.4843	46.7821	53.0252	636.724	267.407	Up
Trim12a	411.005	10.2956	1.5971	6.44641	1.15E-10	1.20E-08	0	0.98643	0.97463	0	1321.87	1142.2	Up
Trim30d	293.946	4.5649	1.101	4.14614	3.38E-05	0.00076	15.6674	22.6879	33.1373	39.7689	827.33	825.085	Up
Cckbr	69.7548	-2.7991	0.76078	-3.6793	0.00023	0.00363	261.45	19.7286	84.7926	14.276	14.4242	23.8579	Down
Hpx	237.666	-0.6482	0.31568	-2.0534	0.04004	0.16518	326.078	222.933	321.627	178.45	117.454	259.454	Down
Trim3	6652.4	-0.299	0.12508	-2.3907	0.01682	0.09092	7811.18	7869.74	6337.03	6062.21	6241.55	5592.69	Down
Tpp1	11540.2	0.31935	0.1218	2.62193	0.00874	0.05607	10066.3	11560	9178.07	12400.8	13660.7	12375.3	Up
Gvin1	54.7368	1.09336	0.52653	2.07656	0.03784	0.15885	23.5011	32.5522	48.7314	33.6506	116.424	73.5618	Up
Gm8995	1217.79	0.65463	0.24405	2.68235	0.00731	0.0491	789.245	1023.91	1025.31	972.808	1704.11	1791.33	Up
Gm1966	60.2539	5.68669	1.72762	3.29163	0.001	0.01097	1.95843	4.93215	0	3.05914	188.545	163.029	Up
Syt9	85.0814	-1.3973	0.3733	-3.7432	0.00018	0.00297	134.152	88.7787	147.169	34.6703	38.121	67.5973	Down
Olfr482	38.547	-1.4506	0.57507	-2.5224	0.01166	0.06907	71.4825	30.5793	67.2493	35.69	12.3636	13.9171	Down
Stk33	1893.45	-0.6282	0.29504	-2.1292	0.03324	0.14535	2416.7	1708.5	2772.81	1645.82	866.481	1950.38	Down
Trim66	39.9122	-1.1302	0.46792	-2.4154	0.01572	0.08638	49.9399	32.5522	81.8687	28.552	23.6969	22.8638	Down
BC051019	3884.44	-0.5657	0.26739	-2.1155	0.03439	0.14874	5131.07	3307.5	5470.58	3663.84	2029.69	3703.94	Down
Scube2	875.888	-0.2681	0.12101	-2.2152	0.02675	0.12472	1014.46	900.61	956.109	809.654	731.512	842.979	Down
Tmem41b	3582.16	0.22182	0.11225	1.97623	0.04813	0.18643	3260.78	3634.99	3026.22	3663.84	4051.13	3856.03	Up
Gm28863	70.6222	-0.9038	0.41021	-2.2032	0.02758	0.12737	71.4825	84.8329	119.879	58.1237	26.7878	62.6269	Down
Galnt18	6843.81	-0.3374	0.08946	-3.7715	0.00016	0.00271	7697.59	7807.59	7415.94	6326.31	6159.12	5656.31	Down
Rassf10	471.034	-1.4325	0.40072	-3.5747	0.00035	0.0049	856.811	391.613	813.814	326.309	120.545	317.111	Down
Gm5599	16.174	-3.4581	0.96615	-3.5792	0.00034	0.00485	30.3556	10.8507	47.7567	5.09857	0	2.98224	Down
Gm33586	98.9379	-2.2737	0.6483	-3.5072	0.00045	0.00597	214.448	60.1722	217.342	38.7492	8.24239	54.6743	Down
Arntl	1134.84	-1.4758	0.56872	-2.5949	0.00946	0.0593	2228.69	2198.75	580.878	593.474	605.815	601.418	Down
Pik3c2a	1693.45	0.58896	0.10806	5.45033	5.03E-08	3.05E-06	1309.21	1385.93	1362.53	1898.71	1990.54	2213.81	Up
Nucb2	3745.23	0.55298	0.0847	6.52866	6.64E-11	7.66E-09	3104.1	3062.86	2941.43	4646.84	4357.13	4359.03	Up
Gm45151	67.5542	0.66533	0.27949	2.3805	0.01729	0.09287	52.8775	44.3893	59.4523	81.5772	85.5148	81.5144	Up
4732496C	379.747	0.4494	0.18874	2.38113	0.01726	0.09277	354.475	300.861	307.982	451.734	355.453	507.974	Up
Gm4366	749.191	-0.2832	0.11711	-2.418	0.01561	0.08601	821.559	878.909	767.032	695.446	654.24	677.962	Down
Itpripl2	7482.39	0.41773	0.11614	3.59671	0.00032	0.00462	6027.05	5959.02	7233.68	8744.06	8776.08	8154.43	Up
Vps35l	3882.52	0.24792	0.10947	2.26465	0.02353	0.11376	3919.79	3469.27	3260.13	4331.75	4262.34	4051.86	Up
Iqck	954.814	-0.5959	0.25462	-2.3403	0.01927	0.09967	1375.79	808.872	1263.12	878.994	550.179	851.925	Down
Acsm5	48.6963	-1.243	0.56749	-2.1903	0.0285	0.13047	58.7528	41.43	105.26	33.6506	11.3333	41.7513	Down
Acsm1	681.305	-0.8356	0.30082	-2.7777	0.00548	0.03993	1017.4	586.926	1015.56	542.488	310.12	615.335	Down
Dcun1d3	1918.26	-0.2177	0.10402	-2.0933	0.03632	0.15469	2112.16	1981.74	2094.47	1829.37	1629.93	1861.91	Down
Lyrm1	3157.77	-0.6455	0.22717	-2.8414	0.00449	0.03455	4088.21	2704.79	4764.95	2785.86	1985.39	2617.41	Down

Abca15	11.4554	-5.9562	1.41741	-4.2022	2.64E-05	0.00062	10.7713	34.525	22.4164	1.01971	0	0	Down
Vwa3a	5304.49	-0.8741	0.26622	-3.2832	0.00103	0.01121	7515.46	4632.27	8444.17	4386.81	2573.69	4274.54	Down
Gm5737	324.324	-0.7738	0.34069	-2.2714	0.02312	0.11243	465.126	297.902	464.897	265.126	129.818	323.076	Down
Cdr2	1428.56	-0.4604	0.20343	-2.2631	0.02363	0.11407	2121.95	1373.11	1468.76	1093.13	1112.72	1401.65	Down
Mfsd13b	45.9867	-1.1662	0.4994	-2.3353	0.01953	0.10045	40.1477	54.2536	96.4881	37.7295	16.4848	30.8164	Down
Gga2	1651.93	0.24369	0.11059	2.20365	0.02755	0.12728	1419.86	1522.06	1596.44	1785.52	1671.14	1916.58	Up
Ubfd1	2485.94	-0.2402	0.08867	-2.7092	0.00674	0.0463	2732.98	2737.34	2607.13	2249.49	2205.87	2382.81	Down
Ndufab1	1983.88	-0.3489	0.10346	-3.3726	0.00074	0.00871	2341.3	2139.57	2187.06	1851.8	1631.99	1751.57	Down
Arhgap17	3349.01	0.34768	0.10882	3.19492	0.0014	0.0142	3052.21	2983.95	2805.95	3593.48	4131.5	3526.99	Up
Gsg1l	77.5598	-0.8989	0.30418	-2.9552	0.00312	0.02647	81.2747	112.453	109.158	43.8477	65.9391	52.6862	Down
1700123J1	28.3065	-1.7116	0.63017	-2.7162	0.0066	0.04576	47.0022	18.7422	64.3254	15.2957	17.5151	6.95855	Down
Sbk1	762.304	-0.3291	0.12828	-2.5656	0.0103	0.06325	791.204	821.696	933.693	719.919	653.209	654.104	Down
Gm30928	164.749	-0.4298	0.18802	-2.2857	0.02227	0.10968	191.926	172.625	202.722	140.721	146.302	134.201	Down
Lat	444.916	-0.3885	0.13459	-2.8861	0.0039	0.03114	480.793	530.699	501.933	407.886	357.514	390.673	Down
Apobr	802.631	0.83057	0.21463	3.86981	0.00011	0.00199	516.045	726.999	490.238	994.222	1236.36	851.925	Up
Nupr1	13799.4	-0.6209	0.17441	-3.5602	0.00037	0.00509	20560.5	14961.2	14650.6	12556.8	9841.41	10226.1	Down
Sult1a1	3757.07	0.50024	0.22533	2.21999	0.02642	0.12367	3149.15	2305.29	3881.94	5481.99	4044.95	3679.08	Up
Gm6939	71.8547	-1.1499	0.30632	-3.7539	0.00017	0.00287	89.1084	123.304	84.7926	49.966	41.2119	42.7454	Down
Gdpd3	1368.02	0.86595	0.23713	3.65184	0.00026	0.00393	1274.94	860.167	772.879	1747.79	2162.6	1389.72	Up
Hirip3	879.875	-0.2594	0.10545	-2.46	0.0139	0.07883	974.317	938.095	963.906	840.245	763.451	799.239	Down
Kctd13	404.476	-0.4747	0.24162	-1.9647	0.04945	0.18952	335.87	525.767	549.69	274.303	357.514	383.714	Down
Cdiptos	757.853	-0.9924	0.28025	-3.5411	0.0004	0.00541	1184.85	583.966	1257.27	561.863	449.21	509.962	Down
Mvp	7644.61	0.28142	0.09811	2.86841	0.00413	0.03234	6525.47	6886.27	7292.16	8280.08	8941.96	7941.69	Up
Zg16	14.6749	-1.39	0.66718	-2.0833	0.03722	0.15704	10.7713	28.6065	24.3657	9.17743	6.18179	8.94671	Down
Zfp768	1210.51	-0.2279	0.10908	-2.089	0.0367	0.1558	1334.67	1236.98	1345.96	1069.68	1197.21	1078.58	Down
B130055M	75.995	-0.743	0.31501	-2.3586	0.01835	0.09644	80.2954	119.358	85.7672	45.8872	67.9997	56.6625	Down
Ccdc189	1637.52	-0.6549	0.20026	-3.2704	0.00107	0.01166	2247.29	1509.24	2252.36	1439.84	1062.24	1314.17	Down
Ctf1	374.099	-0.462	0.17776	-2.5989	0.00935	0.05874	404.415	425.151	470.745	325.289	358.544	260.449	Down
Setd1a	160.867	0.52467	0.22665	2.31492	0.02062	0.10443	157.653	112.453	125.727	173.352	180.302	215.715	Up
Stx1b	429.21	-0.8619	0.31739	-2.7155	0.00662	0.04576	768.682	303.82	588.675	350.782	273.029	290.271	Down
Itgax	6504.56	2.00563	0.1555	12.8981	4.62E-38	2.18E-34	2672.27	2925.75	2183.17	9765.81	11942.2	9538.18	Up
Itgad	118.255	2.23198	0.426	5.23936	1.61E-07	8.42E-06	69.5241	36.4979	18.5179	171.312	201.938	211.739	Up
9130023H	287.784	-0.4349	0.22056	-1.9717	0.04865	0.18754	257.533	419.233	315.779	237.594	241.09	255.478	Down
Inpp5f	1879.85	0.22295	0.08744	2.5499	0.01078	0.06532	1703.83	1783.46	1717.29	2027.19	2076.05	1971.26	Up
Gm39094	1116.57	-2.8577	0.7361	-3.8822	0.0001	0.00192	569.902	3809.59	1507.75	244.732	220.484	346.933	Down
Fank1	1328.07	-0.8073	0.32981	-2.4476	0.01438	0.08104	2307.03	1034.76	1728.99	1070.7	589.331	1237.63	Down
Adam12	286.209	-1.4183	0.41583	-3.4107	0.00065	0.00788	197.801	435.015	616.939	129.504	113.333	224.662	Down
Ebf3	24.1608	-1.6319	0.69508	-2.3477	0.01889	0.09836	64.628	13.81	31.1881	10.1971	8.24239	16.8993	Down
9430038I0	435.787	0.40082	0.20039	2.00014	0.04548	0.17928	382.872	436.988	307.008	485.384	577.997	424.472	Up
Mapk1ip1	2434.68	-0.2272	0.11374	-1.9974	0.04578	0.18002	2550.85	2522.3	2804.98	2204.62	2071.93	2453.39	Down
Stk32c	365.09	0.94396	0.34394	2.74456	0.00606	0.04305	259.491	366.952	122.803	505.779	483.21	452.306	Up
Nkx6-2	22.2421	-1.6959	0.72479	-2.3398	0.01929	0.09971	64.628	25.6472	11.6955	9.17743	12.3636	9.94079	Down
Cfap46	2603.87	-0.9636	0.29269	-3.2923	0.00099	0.01095	3992.25	2225.39	4110	2065.94	1127.15	2102.48	Down
Adgra1	21.8424	-1.7315	0.81989	-2.1119	0.0347	0.14958	59.732	5.91858	35.0866	8.15772	8.24239	13.9171	Down
Kndc1	3100.22	-0.6374	0.28775	-2.2152	0.02675	0.12472	4510.25	2420.7	4391.67	2754.25	1594.9	2929.55	Down
Gm16201	285.828	-1.1873	0.36881	-3.2192	0.00129	0.01339	408.332	228.852	554.563	168.253	115.393	239.573	Down
Tubgcp2	1893.67	-0.2243	0.08835	-2.5392	0.01111	0.06674	2019.14	1996.53	2106.17	1792.66	1698.96	1748.58	Down
Echs1	5639.01	-0.4314	0.08094	-5.3293	9.86E-08	5.49E-06	6617.52	6320.05	6490.04	4923.18	4602.34	4880.93	Down
5830411N	58.4449	0.84419	0.2934	2.87729	0.00401	0.03175	46.023	40.4436	38.9851	69.3406	79.333	76.544	Up
Sirt3	1295.17	-0.4527	0.17267	-2.622	0.00874	0.05607	1647.04	1241.91	1601.31	1220.6	916.966	1143.19	Down
Cox8b	19.5932	-1.5187	0.67386	-2.2538	0.02421	0.1162	48.9606	14.7964	23.3911	6.11829	12.3636	11.9289	Down
B4galnt4	149.682	-0.8547	0.36544	-2.3389	0.01934	0.09986	205.635	114.426	258.276	75.4589	106.121	138.177	Down
Ano9	323.591	0.71685	0.23671	3.02841	0.00246	0.02213	201.718	205.177	327.475	442.556	400.786	363.833	Up
Ptdss2	1791.41	-0.2194	0.11105	-1.9761	0.04814	0.18643	2025.99	1796.29	1959.98	1689.67	1527.93	1748.58	Down
Lrrc56	1332.79	-0.8883	0.25169	-3.5293	0.00042	0.00559	1963.32	1190.62	2037.95	1033.99	690.3	1080.56	Down
Cdhr5	456.239	0.88102	0.2444	3.60491	0.00031	0.0045	288.868	244.635	429.811	592.454	500.725	680.944	Up
Eps8l2	1918.85	0.29707	0.11092	2.67813	0.0074	0.04948	1838.96	1677.92	1649.07	2295.38	2004.96	2046.81	Up
Pnpla2	9164	0.26958	0.12414	2.17153	0.02989	0.13468	8936.3	8294.89	7699.56	10360.3	10894.4	8798.59	Up
Muc5ac	631.639	1.07286	0.33643	3.18898	0.00143	0.0144	438.687	227.865	554.563	784.161	627.452	1157.11	Up
Muc5b	27914.1	1.00096	0.35758	2.79923	0.00512	0.03808	26638.5	7664.56	21500.3	39779.1	31341.7	40560.4	Up
Gm20501	1.96138	-4.3935	2.02848	-2.1659	0.03032	NA	2.93764	4.93215	3.89851	0	0	0	Down
Gm32786	37.1082	2.1403	0.52324	4.09047	4.30E-05	0.00092	17.6258	12.8236	10.7209	91.7743	35.0301	54.6743	Up
Gm39115	4.88766	5.76115	1.88103	3.06276	0.00219	NA	0	0	0	18.3549	1.0303	9.94079	Up
Gm7579	4.23654	5.5542	1.63328	3.40063	0.00067	NA	0	0	0	8.15772	10.303	6.95855	Up
Krtap5-5	175.652	2.50571	0.25792	9.71493	2.60E-22	2.24E-19	36.2309	58.1993	63.3508	269.205	329.696	297.229	Up
Gm40460	26.103	2.11359	0.53194	3.97335	7.09E-05	0.0014	6.85449	8.87787	13.6448	40.7886	55.6361	30.8164	Up
Ctsd	7395.39	1.35573	0.20647	6.56614	5.16E-11	6.11E-09	4623.84	4484.31	3358.57	8889.87	13710.2	9305.57	Up
Lsp1	3542.87	0.42181	0.17769	2.37389	0.0176	0.09402	2840.7	3636.97	2608.1	3751.53	4809.43	3610.49	Up
Tspan32	400.688	0.79591	0.22719	3.50325	0.00046	0.00603	220.323	363.006	295.312	453.773	596.543	475.17	Up
Cd81	9994.53	0.37175	0.16939	2.19459	0.02819	0.1294	8462.36	10147.4	7531.92	11101.6	13211.5	9512.34	Up
Kcnq1	2646.54	0.40438	0.12508	3.23307	0.00122	0.01294	2152.31	2591.35	2090.58	2851.12	3047.62	3146.26	Up
Slc22a18	1551.5	0.26244	0.09756	2.6899	0.00715	0.04826	1424.75	1345.49	1461.94	1714.14	1745.33	1617.37	Up
Phlda2	83.9406	-0.8468	0.34043	-2.4873	0.01287	0.07432	108.693	124.29	90.6403	82.5969	56.6664	40.7572	Down
Tnfrsf26	437.516	1.54089	0.30966	4.97607	6.49E-07	2.78E-05	234.032	310.725	126.702	578.178	781.997	593.465	Up
E230032D	21.6607	1.65071	0.55302	2.98491	0.00284	0.02463	11.7506	12.8236	6.82239	44.8675	28.8484	24.852	Up
Tnfrsf23	38.813	1.66626	0.43684	3.81438	0.00014	0.00239	17.6258	12.8236	25.3403	47.9266	54.6058	74.5559	Up
Dhcr7	2144.06	0.63367	0.15345	4.12958	3.63E-05	0.0008	1695.02	1867.31	1479.48	2451.39	3030.11	2341.05	Up
Shank2	1326.82	-0.7233	0.29704	-2.4349	0.0149	0.08304	1829.17	1074.22	2054.51	1055.4	656.3	1291.31	Down
Ano1	6894.96	0.24488	0.08977	2.72797	0.00637	0.04455	6286.55	6279.61	6367.24	7764.11	7738.57	6933.7	Up
Ccnd1	4262.8	0.4565	0.15742	2.89991	0.00373	0.03019	3590.77	3026.37	4164.58	4453.09	5558.46	4783.51	Up
Pex11g	369.462	-0.3425	0.16032	-2.1365	0.03264	0.14344	390.706	378.789	469.77	345.683	327.635	304.188	Down
Mcoln1	3035.61	0.21087	0.09847	2.14156	0.03223	0.14213	2658.56	2796.53	2987.23	3215.16	3427.8	3128.37	Up
Stxbp2	7318.24	0.3087	0.11497	2.68498	0.00725	0.04878	6335.51	7154.57	6124.56	8181.17	8697.78	7415.83	Up

Retn	80.2769	-4.047	1.49537	-2.7064	0.0068	0.04659	386.789	11.8372	55.5538	1.01971	17.5151	8.94671	Down
Mcemp1	2192.16	1.27639	0.2498	5.10969	3.23E-07	1.55E-05	1315.08	1630.57	897.632	2733.86	3986.22	2589.57	Up
Fcor	42.5466	1.19806	0.54973	2.17937	0.0293	0.13289	43.0854	24.6607	9.74627	46.9069	75.2118	55.6684	Up
Fcer2a	1030.39	1.2688	0.27767	4.5695	4.89E-06	0.00015	378.955	869.044	565.284	1345	1359.99	1664.09	Up
Cd209b	72.6658	-1.3476	0.49635	-2.7151	0.00663	0.04576	143.944	117.385	51.6552	23.4534	60.7876	38.7691	Down
Gm16589	105.236	0.5361	0.26784	2.00156	0.04533	0.17885	84.2123	86.8058	86.7418	94.8335	126.727	152.094	Up
Cd209f	94.8288	-1.5382	0.29575	-5.2011	1.98E-07	1.01E-05	154.716	132.182	136.448	36.7097	44.3028	64.6151	Down
Cd209g	36.2289	-1.4053	0.5184	-2.7109	0.00671	0.04611	68.5449	52.2808	37.0358	11.2169	16.4848	31.8105	Down
Lrrc8e	955.393	0.26601	0.10539	2.52406	0.0116	0.0688	834.289	852.275	916.15	1020.73	1031.33	1077.58	Up
Ctxn1	2143.84	-0.8384	0.2408	-3.4818	0.0005	0.00642	2911.2	1982.72	3355.64	1700.88	1129.21	1783.38	Down
Myo16	30.7028	-1.0549	0.51808	-2.0361	0.04174	0.16953	29.3764	36.4979	58.4776	29.5717	13.3939	16.8993	Down
Col4a1	11062.2	0.46647	0.15226	3.0637	0.00219	0.0202	10109.4	9988.59	7769.73	12732.2	14423.1	11350.4	Up
Rab20	1159.78	0.44235	0.11941	3.70445	0.00021	0.00335	931.231	1098.88	920.048	1320.53	1329.08	1358.91	Up
E230013L	98.5946	0.67761	0.29222	2.31882	0.0204	0.10369	65.6073	99.6294	62.3761	102.991	127.757	133.207	Up
Tubgcp3	4239.78	-0.228	0.1023	-2.2289	0.02582	0.1215	4749.18	4230.8	4742.54	3795.38	3760.59	4160.22	Down
4931415C	40.3079	-0.8789	0.43047	-2.0418	0.04117	0.16792	69.5241	32.5522	54.5791	32.6309	24.7272	27.8342	Down
Atp11a	3955.67	0.87106	0.15601	5.58326	2.36E-08	1.55E-06	2800.55	2982.96	2606.15	4365.4	4816.65	6162.29	Up
F7	3597.78	1.32796	0.18045	7.35905	1.85E-13	3.53E-11	2173.85	2351.65	1623.73	4908.91	6001.49	4527.03	Up
F10	1107.72	0.94017	0.16623	5.65598	1.55E-08	1.09E-06	923.398	729.958	623.761	1443.92	1490.84	1434.46	Up
Proz	1346.37	0.67087	0.29164	2.30036	0.02143	0.10699	1105.53	1099.87	911.277	1569.34	973.632	2418.59	Up
Gm17023	32.8737	1.16581	0.51831	2.24927	0.0245	0.11694	23.5011	25.6472	11.6955	49.966	26.7878	59.6447	Up
Pcid2	446.321	0.32029	0.16017	1.99968	0.04553	0.1794	409.311	417.26	364.511	470.089	447.15	569.607	Up
Lamp1	13836.6	0.32015	0.10113	3.16567	0.00155	0.01538	13037.2	12761.4	11124.4	14875.6	15726.5	15494.7	Up
Adprhl1	43.6768	-2.1332	0.52478	-4.065	4.80E-05	0.00101	110.651	36.4979	66.2747	19.3746	12.3636	16.8993	Down
Tmem255	29.5589	-1.2349	0.53557	-2.3057	0.02112	0.10618	65.6073	29.5929	29.2388	18.3549	21.6363	12.923	Down
Cfap97d2	786.636	-1.1039	0.30431	-3.6277	0.00029	0.0042	1241.64	664.854	1314.77	533.311	338.968	626.269	Down
Cln8	1403.51	0.41034	0.1365	3.00602	0.00265	0.02336	1306.27	1179.77	1129.59	1527.53	1821.57	1456.33	Up
Kbtbd11	2914.96	-0.4807	0.17371	-2.7673	0.00565	0.04091	3508.52	2933.64	3746.47	2439.16	1978.17	2883.82	Down
Myom2	258.332	-2.1529	0.41602	-5.175	2.28E-07	1.13E-05	661.948	270.282	333.323	78.518	60.7876	145.135	Down
Atp7b	3416.85	-0.8146	0.28094	-2.8995	0.00374	0.03022	4657.14	2759.04	5653.81	2601.29	1770.05	3059.77	Down
Nek5	2975.8	-0.9988	0.29458	-3.3906	0.0007	0.0083	4374.14	2587.4	4938.44	2200.54	1262.12	2492.15	Down
Vdac3	5897.46	-0.207	0.08406	-2.4629	0.01378	0.07837	6393.28	6061.61	6504.66	5699.19	5342.1	5383.93	Down
Polb	1925.86	-0.2382	0.08618	-2.764	0.00571	0.04124	2119.02	2123.78	2010.66	1751.87	1780.36	1769.46	Down
Ikbkb	4249.07	0.29599	0.07807	3.79135	0.00015	0.00256	3784.66	3810.58	3848.8	4703.94	4810.46	4535.98	Up
Adam5	132.742	-0.7648	0.25721	-2.9733	0.00295	0.02533	173.321	129.222	198.824	110.129	86.5451	98.4138	Down
Adam9	11644.4	0.43684	0.12893	3.38817	0.0007	0.00834	9416.11	10818.2	9449.99	12189.7	15240.2	12752	Up
Plekha2	1447.33	0.28426	0.12989	2.18837	0.02864	0.13089	1490.36	1166.95	1258.24	1590.76	1628.9	1548.77	Up
Gm31045	18.6244	-1.3493	0.64138	-2.1037	0.0354	0.15181	23.5011	16.7693	39.9597	16.3154	8.24239	6.95855	Down
Adgra2	1955.22	0.37024	0.09783	3.78466	0.00015	0.00262	1608.85	1754.86	1753.35	2131.2	2311.99	2171.07	Up
Got1l1	143.564	-1.4926	0.2597	-5.7473	9.07E-09	6.73E-07	209.552	174.598	251.454	79.5378	59.7573	86.4848	Down
Adrb3	521.606	-0.6831	0.21381	-3.1949	0.0014	0.0142	723.638	659.921	544.817	382.393	501.755	317.111	Down
Nudc-ps1	662.882	-1.2562	0.35288	-3.5597	0.00037	0.0051	886.188	1096.91	820.636	266.146	249.332	658.08	Down
Gm3985	10.1572	-3.1811	1.33087	-2.3902	0.01684	0.09099	42.1061	10.8507	1.94925	0	2.0606	3.97631	Down
Tex15	799.475	-0.6427	0.22494	-2.8572	0.00427	0.03332	867.582	1307.02	749.488	629.164	565.634	677.962	Down
Gtf2e2	1569.34	-0.3553	0.16287	-2.1818	0.02913	0.13237	1913.38	1670.03	1701.7	1360.3	1135.39	1635.26	Down
Gm29243	7.55744	2.05055	0.99897	2.05265	0.04011	NA	6.85449	0.98643	0.97463	12.2366	12.3636	11.9289	Up
Gm45627	21.6218	2.40621	0.89202	2.69748	0.00699	0.04746	0	9.8643	10.7209	42.828	48.424	17.8934	Up
Mfhas1	1992.3	0.33528	0.11591	2.89256	0.00382	0.03075	1593.18	1904.8	1787.47	2290.28	2294.47	2083.59	Up
Lonrf1	901.24	0.35156	0.172	2.04399	0.04095	0.16728	843.102	628.356	904.454	1022.77	925.208	1083.55	Up
Trmt9b	178.28	-1.2156	0.44119	-2.7552	0.00587	0.04202	331.953	298.888	116.955	91.7743	70.0603	160.047	Down
Dlc1	8935.05	0.4592	0.1828	2.51201	0.012	0.0706	6212.13	9511.15	6851.63	9750.51	11641.3	9643.56	Up
Tusc3	5857.52	-0.3521	0.16407	-2.1463	0.03185	0.14068	8048.15	5787.38	5871.15	5140.38	4677.55	5620.52	Down
Msr1	962.09	1.61667	0.24503	6.59779	4.17E-11	5.03E-09	490.586	580.021	348.917	1070.7	1749.45	1532.87	Up
Slc7a2	3704.26	0.77696	0.16757	4.63672	3.54E-06	0.00012	2332.48	2656.45	3201.65	4268.53	5475.01	4291.44	Up
Fgl1	489.644	2.09934	0.27808	7.54948	4.37E-14	9.43E-12	182.134	113.439	260.225	773.964	867.511	740.589	Up
Asah1	13826.6	0.55149	0.2363	2.33389	0.0196	0.10077	7437.12	12631.2	13578.5	17275	18739.1	13298.8	Up
F11	134.502	-0.9939	0.37356	-2.6605	0.0078	0.05144	151.778	128.236	257.302	83.6166	61.8179	124.26	Down
Klkb1	197.548	-1.1458	0.30197	-3.7943	0.00015	0.00254	303.556	174.598	338.196	143.78	98.9087	126.248	Down
Cyp4v3	2144.04	0.38285	0.14754	2.59486	0.00946	0.0593	1903.59	2038.95	1641.27	2266.83	2218.23	2795.35	Up
Fam149a	3308.21	-0.6272	0.20543	-3.0533	0.00226	0.02079	4438.77	3193.07	4417.01	2716.52	1983.32	3100.53	Down
Tlr3	2171.5	0.26014	0.11956	2.17587	0.02956	0.13368	1859.53	2213.55	1855.69	2259.69	2498.47	2342.05	Up
Sorbs2	1984.18	-1.0023	0.26427	-3.7928	0.00015	0.00255	2741.8	1926.5	3272.8	1375.6	899.451	1688.94	Down
Ccdc110	101.206	-0.7809	0.30829	-2.533	0.01131	0.06763	119.464	98.643	165.687	90.7546	70.0603	62.6269	Down
1700029J	1356.12	-0.8779	0.22226	-3.95	7.81E-05	0.00151	1717.54	1333.65	2218.25	1048.27	758.3	1060.68	Down
Ufsp2	2164.74	0.20236	0.10065	2.01054	0.04437	0.17627	1882.05	2178.04	1979.47	2313.73	2311.99	2323.16	Up
Ankrd37	575.005	0.67344	0.30439	2.21242	0.02694	0.12526	297.681	382.735	649.102	905.507	600.664	614.341	Up
Lrp2bp	253.393	-0.5415	0.1865	-2.9034	0.00369	0.02997	324.119	256.472	320.652	205.982	186.484	226.65	Down
Slc25a4	9606.06	-0.2235	0.08505	-2.6281	0.00859	0.05534	10438.4	10185.9	10421.7	8860.3	9352.02	8378.09	Down
Stox2	1431.18	-0.7362	0.15613	-4.7155	2.41E-06	8.50E-05	1817.42	1721.32	1827.43	1049.29	893.269	1278.38	Down
Wwc2	6462.35	0.28478	0.08365	3.40442	0.00066	0.00801	5523.74	6048.79	5907.22	7067.64	7252.27	6974.45	Up
Dctd	47.863	0.92868	0.36378	2.55286	0.01068	0.06502	35.2517	26.6336	37.0358	67.3012	47.3937	73.5618	Up
Gm9892	745.685	0.36986	0.12252	3.01873	0.00254	0.02267	619.842	706.284	625.711	877.975	851.027	793.275	Up
Aga	1536.68	0.49666	0.14153	3.50919	0.00045	0.00593	1308.23	1252.77	1263.12	1961.93	1936.96	1497.08	Up
Spcs3	5198.12	0.2025	0.09783	2.06985	0.03847	0.16079	4583.69	5145.22	4772.75	5520.74	5876.82	5289.49	Up
Cep44	1026	-0.2319	0.11692	-1.983	0.04737	0.1844	1101.61	1075.21	1148.11	979.946	853.087	998.055	Down
Hmgb2	1609.52	0.39746	0.12935	3.07281	0.00212	0.01971	1258.29	1462.88	1446.35	1984.37	1869.99	1635.26	Up
Galnt7	1103.8	0.52157	0.16923	3.08197	0.00206	0.01918	1039.92	733.904	945.388	1158.4	1401.21	1343.99	Up
AW04620	133.922	0.82498	0.24763	3.33148	0.00086	0.00977	112.609	72.0094	105.26	167.233	183.393	163.029	Up
Gm45356	5.70669	2.58501	1.13916	2.26922	0.02326	NA	2.93764	0	1.94925	12.2366	6.18179	10.9349	Up
Mfap3l	3377.75	0.25781	0.11884	2.16943	0.03005	0.13517	3394.93	2868.54	2966.77	3363.02	3871.86	3801.36	Up
Msmo1	7418.43	0.34649	0.1352	2.56278	0.01038	0.06364	6743.84	7104.27	5747.38	7890.55	9358.2	7666.33	Up
Gm10663	23.9242	1.51386	0.48376	3.12937	0.00175	0.01691	15.6674	10.8507	10.7209	30.5914	32.9696	42.7454	Up
Smim31	792.081	1.58802	0.29719	5.34347	9.12E-08	5.13E-06	602.216	261.404	322.602	999.321	1250.78	1316.16	Up

Marchf1	972.563	0.89154	0.12559	7.09859	1.26E-12	2.05E-10	673.698	709.243	660.797	1201.22	1409.45	1180.97	Up
Npy1r	763.272	0.73047	0.17717	4.12307	3.74E-05	0.00082	486.669	636.247	599.396	881.034	849.996	1126.29	Up
Naf1	1502.07	-0.4707	0.17917	-2.6274	0.0086	0.05541	2111.18	1395.8	1728.01	1237.93	1148.78	1390.72	Down
Sh2d4a	3054.06	0.63232	0.13952	4.53199	5.84E-06	0.00018	2058.31	2689.01	2438.52	3879	3896.59	3362.97	Up
Ints10	1827.41	-0.4057	0.12607	-3.2182	0.00129	0.01339	2118.04	2013.3	2116.89	1531.61	1402.24	1782.38	Down
Lpl	9136.64	1.17668	0.24811	4.74263	2.11E-06	7.54E-05	6260.11	4605.64	5947.18	15112.2	14933.1	7961.57	Up
Gm33103	4.55505	3.05012	1.31207	2.32465	0.02009	NA	0	1.97286	0.97463	12.2366	6.18179	5.96447	Up
Slc18a1	262.005	0.45768	0.17276	2.64916	0.00807	0.05284	219.344	216.028	227.088	285.52	276.12	347.927	Up
Atp6v1b2	13685.6	0.20682	0.10411	1.98659	0.04697	0.18332	12542.7	13851.4	11724.8	14289.3	15450.4	14255.1	Up
Zfp930	1012.34	-0.3997	0.14716	-2.7159	0.00661	0.04576	1177.99	1183.72	1093.53	783.141	808.784	1026.88	Down
Gm3365	206.28	0.44006	0.20662	2.1298	0.03319	0.14524	144.923	172.625	207.596	224.337	257.575	230.626	Up
Zfp868	2099.09	-1.1178	0.2887	-3.8717	0.00011	0.00198	2970.93	3012.56	2638.32	913.665	997.329	2061.72	Down
Zfp869	2252.07	0.30044	0.09858	3.04754	0.00231	0.0211	2042.64	2035	1977.52	2661.46	2470.66	2325.15	Up
Zfp963	380.188	-0.6807	0.20053	-3.3944	0.00069	0.00822	516.045	483.351	405.445	331.407	229.757	315.123	Down
Gm20422	74.0628	0.61574	0.27321	2.25374	0.02421	0.1162	52.8775	65.1044	57.503	101.971	82.4239	84.4967	Up
Zfp866	34.5704	1.93226	0.49107	3.93477	8.33E-05	0.00159	18.605	5.91858	18.5179	55.0646	55.6361	53.6802	Up
Gmip	1956.01	0.42373	0.1369	3.09515	0.00197	0.01856	1527.57	1917.62	1567.2	2204.62	2430.47	2088.56	Up
Lpar2	447.053	0.69196	0.21792	3.17523	0.0015	0.01499	295.722	303.82	425.912	613.868	599.634	443.359	Up
Cilp2	26.487	-2.8671	0.7745	-3.7019	0.00021	0.00337	52.8775	15.7829	71.1478	6.11829	2.0606	10.9349	Down
Tm6sf2	578.625	-1.0499	0.3016	-3.4813	0.0005	0.00643	782.391	598.763	960.008	411.965	223.575	495.051	Down
Mef2b	1236.19	0.65982	0.17886	3.68912	0.00023	0.00351	992.922	1058.44	823.56	1651.94	1682.48	1207.81	Up
Armc6	553.067	-0.7277	0.16662	-4.3673	1.26E-05	0.00033	715.805	702.338	651.051	381.373	367.817	500.022	Down
Upf1	3609.37	0.2642	0.08389	3.14937	0.00164	0.01603	3257.84	3157.56	3423.87	3945.28	4002.71	3868.95	Up
Tmem59l	375.523	0.71886	0.2411	2.98157	0.00287	0.02483	231.094	375.83	244.631	495.581	520.301	385.702	Up
Crlf1	1987.45	-0.3658	0.15709	-2.3285	0.01988	0.10187	2009.34	2638.7	2066.21	1929.3	1546.48	1734.67	Down
Rex1bd	1834.97	-0.3377	0.10467	-3.226	0.00126	0.01314	2081.81	2123.78	1940.48	1698.85	1660.84	1504.04	Down
Isyna1	4784.59	-0.2674	0.11617	-2.3023	0.02132	0.1067	4832.41	5829.8	5018.36	4409.25	4072.77	4544.93	Down
Lsm4	1506.65	0.2942	0.14942	1.96893	0.04896	0.18839	1440.42	1327.73	1292.36	1700.88	1894.72	1383.76	Up
Gm3336	71.4723	1.42038	0.2987	4.75515	1.98E-06	7.13E-05	37.2101	42.4165	37.0358	120.326	84.4845	107.36	Up
Pde4c	601.233	-1.0192	0.24962	-4.0828	4.45E-05	0.00095	956.691	733.904	725.123	303.875	342.059	545.749	Down
Rab3a	1493.98	-0.3552	0.15577	-2.2802	0.0226	0.11061	1817.42	1640.43	1573.05	1544.87	1111.69	1276.4	Down
Ifi30	5525.79	0.54005	0.12028	4.49014	7.12E-06	0.00021	4113.67	4822.65	4573.93	7022.78	6755.67	5866.06	Up
Pik3r2	2887.33	-0.2312	0.1009	-2.2918	0.02192	0.10853	3122.71	3029.33	3202.63	2888.85	2537.63	2542.85	Down
2010320M	565.038	-0.366	0.12359	-2.9612	0.00306	0.02615	594.382	633.288	681.264	503.739	491.452	486.104	Down
Gm10654	3.57382	5.3082	1.78305	2.97704	0.00291	NA	0	0	0	8.15772	10.303	2.98224	Up
Haus8	530.27	0.9127	0.21659	4.21387	2.51E-05	0.00059	312.369	399.504	391.8	799.456	766.542	511.95	Up
Myo9b	4261.12	0.29859	0.09246	3.2292	0.00124	0.01308	3574.13	3863.84	4027.16	4689.67	4835.19	4576.74	Up
Ocel1	1015.08	-0.7501	0.15597	-4.8088	1.52E-06	5.73E-05	1326.83	1362.26	1130.57	675.051	713.997	881.748	Down
Abhd8	1126.09	-0.373	0.111	-3.3604	0.00078	0.00899	1217.16	1380.02	1215.36	988.104	1005.57	950.339	Down
Plvap	6735.27	-0.4306	0.17382	-2.4772	0.01324	0.07595	8191.11	9000.18	6007.6	6222.3	5877.85	5112.55	Down
Ccdc194	294.331	-2.1933	1.06054	-2.0681	0.03863	0.16129	466.105	298.888	684.188	26.5126	28.8484	261.443	Down
Tmem221	329.992	2.87726	0.30065	9.56998	1.07E-21	8.43E-19	97.9213	71.0229	68.2239	740.313	634.664	367.809	Up
Fam129c	422.134	-1.013	0.24732	-4.0961	4.20E-05	0.0009	441.625	626.383	625.711	205.982	279.211	353.892	Down
Colgalt1	5318.84	0.80439	0.11678	6.88787	5.66E-12	7.71E-10	3715.13	4029.56	3875.12	7218.56	7141	5933.65	Up
Unc13a	516.332	-0.3727	0.18081	-2.0613	0.03928	0.16306	587.528	636.247	524.349	378.314	433.756	537.796	Down
Jak3	3262.44	0.49504	0.13293	3.72413	0.0002	0.00314	2539.1	3074.7	2510.64	3472.13	4144.89	3833.17	Up
B3gnt3	1933.11	1.60757	0.23261	6.91112	4.81E-12	6.64E-10	1031.11	672.745	1161.76	3074.44	3374.23	2284.39	Up
Gm18860	57.6406	4.70939	1.47603	3.19058	0.00142	0.01433	7.8337	1.97286	2.92388	160.095	171.03	1.98816	Up
Gm5373	24.7622	2.27453	0.59569	3.81832	0.00013	0.00236	5.87528	6.90501	12.6702	31.6112	29.8787	61.6329	Up
Zfp617	920.354	0.48078	0.1129	4.25849	2.06E-05	0.0005	701.116	802.954	801.144	1074.78	1071.51	1070.62	Up
Cyp4f18	301.802	1.11239	0.22393	4.96749	6.78E-07	2.87E-05	196.822	222.933	153.016	361.999	375.029	501.016	Up
Tpm4	3740.3	0.42569	0.11606	3.66802	0.00024	0.00375	3466.41	3017.49	3093.47	3874.92	4498.28	4491.25	Up
Cib3	97.2298	-1.5439	0.42751	-3.6114	0.0003	0.00442	188.009	66.0908	180.306	54.0449	42.2422	52.6862	Down
Ap1m1	3934.47	-0.2088	0.07893	-2.6457	0.00815	0.05324	4274.26	4237.7	4144.12	3765.81	3611.2	3573.71	Down
Calr3	184.132	-1.4284	0.34798	-4.1047	4.05E-05	0.00088	330.974	159.802	314.805	93.8138	74.1815	131.218	Down
Slc35e1	2845.41	0.24372	0.11744	2.07532	0.03796	0.1593	2721.23	2796.53	2299.15	3071.38	3223.8	2960.37	Up
Large1	2137.7	0.4358	0.14093	3.09233	0.00199	0.01871	1700.89	2076.43	1674.41	2349.42	2741.62	2283.4	Up
Hmgxb4	288.797	0.57967	0.20408	2.84045	0.00451	0.03462	259.491	206.164	229.037	286.54	339.998	411.549	Up
Hmox1	3608.72	0.74583	0.16762	4.44944	8.61E-06	0.00025	2394.18	2968.17	2726.03	4546.91	5337.98	3679.08	Up
Mcm5	729.253	0.34803	0.17232	2.01965	0.04342	0.17393	538.567	629.342	757.285	838.206	727.391	884.73	Up
Rasd2	934.757	1.11641	0.26618	4.1942	2.74E-05	0.00064	377.976	697.406	694.909	1246.09	1572.24	1019.92	Up
Ttc29	1944.65	-1.1924	0.28851	-4.1329	3.58E-05	0.0008	3454.66	1806.15	2855.66	1114.55	831.451	1605.44	Down
Slc10a7	1603.67	0.23218	0.10835	2.14297	0.03212	0.14173	1450.21	1455.97	1518.47	1590.76	1868.96	1737.65	Up
Smad1	2049.72	0.28587	0.08914	3.20715	0.00134	0.0138	1868.34	1899.86	1773.82	2213.8	2221.32	2321.17	Up
Hhip	2255.11	0.70714	0.17005	4.15831	3.21E-05	0.00073	1344.46	1874.22	1920.99	2810.33	3050.71	2529.93	Up
Ucp1	27.7141	-3.0998	0.99953	-3.1013	0.00193	0.01824	120.443	14.7964	13.6448	4.07886	11.3333	1.98816	Down
Pkn1	1389.43	0.47999	0.12541	3.82751	0.00013	0.0023	1108.47	1262.63	1110.1	1483.69	1775.2	1596.49	Up
Adgre5	7975.76	0.23968	0.0982	2.44082	0.01465	0.08216	7685.84	7387.37	6871.12	8047.59	8908.99	8953.67	Up
1700067K	40.8647	-1.1041	0.4466	-2.4723	0.01343	0.07676	56.7943	38.4708	72.1224	25.4929	17.5151	34.7927	Down
Gm10643	202.913	-0.7386	0.20943	-3.5264	0.00042	0.00564	302.577	214.055	244.631	142.76	150.424	163.029	Down
Gm26887	86.1008	-0.885	0.26409	-3.3511	0.0008	0.00926	123.381	105.548	106.234	49.966	63.8785	67.5973	Down
Mir27a	39.1237	1.24824	0.52375	2.38328	0.01716	0.09232	17.6258	18.7422	33.1373	45.8872	86.5451	32.8046	Up
Cacna1a	520.285	-0.3412	0.16931	-2.0152	0.04388	0.1751	644.322	474.473	625.711	437.458	490.422	449.323	Down
Gcdh	914.776	-0.2606	0.10922	-2.3861	0.01703	0.09178	980.192	945.986	1065.27	844.324	840.724	812.162	Down
Dnase2a	1334.44	0.55813	0.19006	2.93659	0.00332	0.02767	1010.55	1291.24	936.617	1663.15	1825.69	1279.38	Up
Junb	10274.6	0.61418	0.27305	2.24929	0.02449	0.11694	6323.76	10571.6	7464.67	12813.7	16419.9	8054.02	Up
Tnpo2	677.199	0.38462	0.17171	2.23996	0.02509	0.11904	676.636	494.201	591.599	708.702	722.239	869.819	Up
Fbxw9	1850.22	-0.3879	0.12528	-3.0965	0.00196	0.01849	2101.39	1889.01	2302.07	1709.04	1478.48	1621.34	Down
Man2b1	8037.4	0.48338	0.11711	4.12755	3.67E-05	0.00081	6523.52	7433.73	6152.82	9547.59	9907.35	8659.42	Up
Gm42031	22.4819	1.72658	0.66443	2.59858	0.00936	0.05876	4.89606	5.91858	20.4672	27.5323	43.2725	32.8046	Up
Mylk3	180.152	-1.7033	0.59105	-2.8818	0.00395	0.03141	347.621	72.0094	407.394	75.4589	42.2422	136.189	Down
Abcc12	37.5315	-0.802	0.3912	-2.0501	0.04036	0.16573	43.0854	53.2672	46.7821	33.6506	19.5757	28.8283	Down
Heatr3	1463.59	-0.2629	0.12615	-2.0842	0.03714	0.15696	1734.19	1484.58	1571.1	1212.44	1323.93	1455.33	Down

Nkd1	8517.64	-0.256	0.11868	-2.1573	0.03098	0.13798	8848.17	10514.4	8451.97	7938.48	7417.12	7935.73 Down
Snx20	631.829	0.61452	0.2134	2.87973	0.00398	0.03159	409.311	635.261	453.202	713.8	899.451	679.95 Up
Cyld	3009.96	0.25977	0.10425	2.49168	0.01271	0.07365	2582.18	2912.93	2724.08	3047.93	3367.02	3425.59 Up
Sall1	14.0003	-1.8373	0.81078	-2.2661	0.02345	0.11356	28.3972	13.81	23.3911	10.1971	7.21209	0.99408 Down
Tox3	823.058	0.29909	0.10538	2.83824	0.00454	0.03483	725.597	762.51	726.097	874.915	911.814	937.416 Up
Gm19935	5784.9	-1.2373	0.3108	-3.9811	6.86E-05	0.00136	10318.9	5119.57	8933.43	3576.14	2137.87	4623.46 Down
Rbl2	660.024	0.33851	0.15288	2.21418	0.02682	0.12496	614.946	529.713	604.269	674.032	688.239	848.943 Up
Fto	3769.66	-0.2922	0.10687	-2.7341	0.00625	0.04409	4600.34	3854.97	3995	3408.91	3365.99	3392.79 Down
Gm45332	283.226	-1.6994	0.42626	-3.9867	6.70E-05	0.00133	447.5	654.989	196.875	129.504	87.5754	182.91 Down
Irx5	2745.12	-0.2045	0.09996	-2.0455	0.04081	0.16685	2785.86	3191.1	2841.04	2609.45	2526.29	2517.01 Down
Ces1b	195.968	-1.0896	0.49466	-2.2028	0.02761	0.12744	221.302	144.019	434.684	117.267	58.727	199.81 Down
Ces1d	39882.8	-0.4191	0.19722	-2.1251	0.03358	0.14635	40476.7	39048.8	57381.2	35047.6	27671.8	39670.7 Down
Ces1e	823.676	-0.7312	0.25065	-2.9174	0.00353	0.02899	1025.24	786.184	1272.86	632.223	448.18	777.369 Down
Ces1f	564.319	-2.1037	0.89794	-2.3428	0.01914	0.09928	1455.11	194.327	1097.43	230.456	85.5148	323.076 Down
Ces1g	446.891	-0.9707	0.39824	-2.4375	0.01479	0.08265	355.454	486.31	933.693	404.827	171.03	330.034 Down
Gnao1	1317.09	-0.3615	0.11125	-3.2491	0.00116	0.01238	1544.22	1536.86	1362.53	1124.75	1217.81	1116.35 Down
Bbs2	1664.51	-0.3887	0.13174	-2.9509	0.00317	0.02672	1968.22	1739.08	1955.1	1503.06	1255.93	1565.67 Down
C78859	17.3233	-1.2702	0.56395	-2.2524	0.0243	0.11641	17.6258	23.6743	32.1627	10.1971	11.3333	8.94671 Down
Ap3s1-ps:	33.5922	0.98769	0.44469	2.22109	0.02635	0.12345	15.6674	18.7422	33.1373	48.9463	44.3028	40.7572 Up
Ccl22	452.287	0.78077	0.27669	2.82184	0.00477	0.03605	375.038	205.177	418.115	634.263	609.937	471.193 Up
Coq9	2187.76	-0.3088	0.08699	-3.5496	0.00039	0.00527	2440.2	2479.88	2343	1994.56	1893.69	1975.23 Down
Ccdc102a	972.674	-0.4039	0.11291	-3.5777	0.00035	0.00486	1153.51	1044.63	1125.69	895.31	806.724	810.174 Down
Adgrg1	9402.1	0.26081	0.06957	3.74868	0.00018	0.00292	8467.25	8638.16	8558.2	10217.5	10370	10161.5 Up
Drc7	3224.57	-0.974	0.31218	-3.1201	0.00181	0.0173	4947.96	2834.01	5038.82	2311.69	1273.45	2941.48 Down
Katnb1	1453.51	-0.3364	0.13357	-2.5186	0.01178	0.06963	1678.37	1407.64	1780.64	1343.98	1198.24	1312.18 Down
Kifc3	1689.78	0.44664	0.12312	3.62766	0.00029	0.0042	1297.46	1570.4	1422.96	1925.22	2093.57	1829.1 Up
Cfap20	3838.67	-0.498	0.10152	-4.9048	9.35E-07	3.76E-05	4903.9	4190.35	4389.72	3193.75	3115.62	3238.71 Down
4933406B	452.075	-1.2483	0.24608	-5.0728	3.92E-07	1.83E-05	582.632	475.459	850.85	234.534	264.787	304.188 Down
Ccdc113	2456.31	-0.9156	0.26717	-3.4272	0.00061	0.00754	3717.09	2272.73	3642.18	1922.16	1120.96	2062.71 Down
Slc38a7	1172.57	0.24714	0.12402	1.99284	0.04628	0.18146	1076.15	1111.71	1029.21	1174.71	1424.9	1218.74 Up
Got2	3637.82	-0.2033	0.09587	-2.1203	0.03398	0.14764	4168.51	3676.42	3836.13	3475.19	3263.99	3406.71 Down
Gm45895	37.3238	1.004	0.46685	2.15057	0.03151	0.13961	12.7298	30.5793	31.1881	46.9069	60.7876	41.7513 Up
Bean1	22.1124	-1.1853	0.58346	-2.0315	0.04221	0.17067	32.314	33.5386	26.3149	5.09857	17.5151	17.8934 Down
Cklf	541.513	0.35026	0.14032	2.49606	0.01256	0.07296	471.001	505.052	452.227	550.646	670.724	599.429 Up
Cmtm3	2910.89	0.28862	0.14677	1.96645	0.04925	0.18906	2724.17	2987.9	2150.03	3268.19	3274.29	3060.77 Up
Cdh16	326.78	0.91415	0.32082	2.84938	0.00438	0.03391	255.575	257.458	166.661	482.325	549.149	249.514 Up
Rrad	312.113	0.87689	0.23202	3.77943	0.00016	0.00265	217.385	274.227	168.611	410.945	460.543	340.969 Up
Ces2c	34.7188	3.11232	0.83301	3.73622	0.00019	0.00303	0	12.8236	8.77165	46.9069	103.03	36.7809 Up
Fhod1	647.3	0.63762	0.16577	3.84639	0.00012	0.00216	606.133	426.138	487.314	765.806	818.057	780.352 Up
Slc9a5	454.04	0.32255	0.14844	2.17292	0.02979	0.13443	352.517	432.056	425.912	535.35	487.331	491.075 Up
Lrrc36	3707.56	-0.8378	0.26278	-3.1883	0.00143	0.01442	5285.79	3137.83	5840.94	3146.84	1916.36	2917.62 Down
Tppp3	27588.1	-0.5482	0.15948	-3.4374	0.00059	0.00731	37662.5	25990.4	34649	24150.9	20875.9	22199.8 Down
Zdhhc1	9821.2	-0.4341	0.16078	-2.7	0.00693	0.04725	11620.3	9040.63	13202.3	8695.11	7617	8751.87 Down
Hsd11b2	544.246	2.07344	0.2902	7.14489	9.01E-13	1.50E-10	228.157	167.693	230.987	1031.95	1081.81	524.873 Up
Atp6v0d1	9970.89	0.26432	0.11475	2.30347	0.02125	0.1065	8956.86	9820.89	8402.26	10928.3	11775.3	9941.78 Up
Agrp	118.024	-0.6526	0.2988	-2.1842	0.02895	0.13187	126.318	125.277	181.281	101.971	65.9391	107.36 Down
Enkd1	618.8	-0.6989	0.19717	-3.5445	0.00039	0.00535	832.331	595.803	869.368	493.542	402.847	518.909 Down
Gfod2	1641.94	-0.2968	0.12559	-2.363	0.01813	0.09597	1858.55	1642.41	1929.76	1556.08	1325.99	1538.83 Down
Tsnaxip1	1211	-1.0929	0.235	-4.6506	3.31E-06	0.00011	1813.5	1222.19	1911.24	811.693	575.937	931.452 Down
Slc12a4	1619.85	0.49244	0.10645	4.62583	3.73E-06	0.00012	1334.67	1362.26	1341.09	1867.1	2055.45	1758.52 Up
Gm45752	51.571	1.17309	0.32918	3.56362	0.00037	0.00505	35.2517	27.62	32.1627	69.3406	82.4239	62.6269 Up
1810019D	2699.85	-0.4314	0.11413	-3.7796	0.00016	0.00265	2972.89	3449.54	2879.05	2406.53	2257.38	2233.69 Down
Pla2g15	3389.75	0.97029	0.20448	4.74508	2.08E-06	7.47E-05	2214	2698.87	1959.98	4321.55	5642.94	3501.14 Up
Tango6	546.29	-0.4939	0.1368	-3.6107	0.00031	0.00442	677.615	610.6	628.635	448.675	414.18	498.033 Down
Cyb5b	19587.1	0.38609	0.12861	3.00197	0.00268	0.02359	17011.9	19114	14819.2	20618.6	23570.1	22388.6 Up
Nqo1	439.428	-1.1037	0.41829	-2.6386	0.00832	0.05407	376.997	354.128	1068.19	356.9	203.999	276.354 Down
Nob1	1180.87	-0.3181	0.09764	-3.2577	0.00112	0.01205	1298.44	1357.33	1275.79	1024.81	1089.03	1039.81 Down
Phlpp2	1219.43	0.36098	0.12887	2.80109	0.00509	0.03792	922.418	1137.35	1143.24	1334.81	1395.02	1383.76 Up
Hydin	3365.78	-1.0878	0.32093	-3.3895	0.0007	0.00831	5774.42	2797.51	5161.63	2202.58	1327.02	2931.54 Down
Vac14	2311.02	0.26626	0.11643	2.28687	0.0222	0.10948	2015.22	2298.38	1981.42	2493.2	2717.93	2359.94 Up
Il34	1434.28	0.31061	0.12956	2.39734	0.01651	0.08972	1148.62	1375.08	1317.7	1468.39	1740.17	1555.73 Up
Aars	5480.94	0.29443	0.10245	2.87393	0.00405	0.03194	5027.28	5137.33	4606.09	6314.07	6225.06	5575.79 Up
Glg1	4005.45	0.28912	0.10001	2.89091	0.00384	0.03085	3734.72	3582.71	3498.91	4033.99	4611.62	4570.77 Up
Mlkl	664.471	0.50686	0.13081	3.87494	0.00011	0.00196	500.378	586.926	559.436	772.944	833.511	733.63 Up
Fa2h	14.1562	1.64912	0.81273	2.0291	0.04245	0.17123	7.8337	2.95929	9.74627	19.3746	8.24239	36.7809 Up
Ldhd	1005.07	0.45987	0.17429	2.63856	0.00833	0.05407	810.788	714.175	1013.61	1301.16	1151.87	1038.81 Up
Tmem231	1466.96	-0.6584	0.24308	-2.7087	0.00675	0.04635	2229.67	1279.4	1879.08	1138	880.905	1394.69 Down
Mon1b	530.129	0.37701	0.12164	3.09937	0.00194	0.01834	488.627	445.866	449.303	592.454	585.21	619.311 Up
Vat1l	6.89511	-3.1848	1.47134	-2.1646	0.03042	NA	25.4595	7.89144	3.89851	0	4.12119	0 Down
Dynlrb2	3658.1	-0.8116	0.24236	-3.3485	0.00081	0.00932	5364.13	3207.87	5410.16	2993.88	1985.39	2987.21 Down
1700030J2	415.632	-0.8361	0.29667	-2.8183	0.00483	0.03637	633.551	331.44	633.508	303.875	224.605	366.815 Down
Cmip	1646.15	0.32349	0.15876	2.03755	0.04159	0.16912	1470.78	1329.71	1586.69	1545.89	1790.66	2153.17 Up
Plcg2	1508.35	0.66801	0.18189	3.67269	0.00024	0.00369	1024.26	1368.18	1103.28	1629.5	2240.9	1683.97 Up
Necab2	38.6084	-1.3579	0.38052	-3.5685	0.00036	0.00498	48.9606	57.2129	60.4269	19.3746	26.7878	18.8875 Down
Dnaaf1	1093.3	-1.309	0.27877	-4.6958	2.66E-06	9.15E-05	1744.96	1053.51	1875.18	729.096	422.422	734.624 Down
Taf1c	509.809	-0.3214	0.1567	-2.051	0.04027	0.16559	594.382	535.631	569.182	485.384	381.21	493.063 Down
Atp2c2	1626.42	-1.0627	0.35499	-2.9937	0.00276	0.02409	2727.11	1309.98	2562.3	1093.13	574.907	1491.12 Down
Cotl1	3604.5	0.5959	0.14674	4.06096	4.89E-05	0.00103	2835.8	3089.5	2686.07	4054.39	5135.01	3826.21 Up
Pgbd5	53.8642	-1.8496	0.38244	-4.8362	1.32E-06	5.10E-05	92.046	85.8194	75.0463	19.3746	33.9998	16.8993 Down
Agt	94.0479	-1.6224	0.35636	-4.5526	5.30E-06	0.00016	156.674	96.6701	172.509	43.8477	60.7876	33.7987 Down
Arv1	634.806	-0.7244	0.16792	-4.3139	1.60E-05	0.00041	738.326	740.809	893.733	436.438	437.877	561.654 Down
4930567H	68.7432	-1.1774	0.4971	-2.3685	0.01786	0.095	156.674	59.1858	70.1732	53.0252	23.6969	49.7039 Down
Map3k21	352.251	-0.4255	0.19752	-2.154	0.03124	0.13861	443.583	316.644	451.252	308.974	270.968	322.081 Down

Slc35f3	509.467	-0.628	0.18259	-3.4391	0.00058	0.00729	611.029	512.943	731.945	369.137	387.392	444.353	Down
Coa6	814.272	-0.251	0.11848	-2.1182	0.03416	0.14809	937.107	842.411	875.215	798.437	706.785	725.677	Down
Gm26759	101.492	1.17107	0.4319	2.71142	0.0067	0.04609	43.0854	73.9822	70.1732	211.081	138.06	72.5677	Up
Gm31718	225.933	0.35918	0.17881	2.00873	0.04457	0.17661	191.926	195.313	206.621	219.239	275.09	267.407	Up
Itgb1	42711.2	0.23117	0.09535	2.42449	0.01533	0.0848	38672	42272.5	36945.2	44179.1	48056.2	46142.1	Up
Ccdc7b	18.9833	1.86995	0.68902	2.71393	0.00665	0.04589	2.93764	5.91858	15.594	26.5126	37.0907	25.846	Up
2610044C	806.803	0.50511	0.13851	3.64668	0.00027	0.00398	601.237	656.962	742.666	938.138	1022.06	879.759	Up
Gm32856	291.624	1.20862	0.17825	6.7805	1.20E-11	1.58E-09	181.154	196.299	151.067	384.433	389.453	447.335	Up
Kbtbd3	554.932	-0.3558	0.15587	-2.2825	0.02246	0.11021	669.782	525.767	673.467	514.956	459.513	486.104	Down
Dync2h1	2087.21	-0.7927	0.23948	-3.3102	0.00093	0.0104	3080.6	1868.3	2991.13	1615.23	1149.81	1818.17	Down
Mmp13	251.437	1.421	0.47903	2.96641	0.00301	0.02578	99.8797	194.327	115.981	440.517	532.664	125.254	Up
Mmp12	1275.52	1.89935	0.20965	9.05971	1.31E-19	6.69E-17	456.313	722.066	439.557	2132.22	1844.23	2058.74	Up
Mmp8	623.628	1.22379	0.3863	3.16799	0.00153	0.01528	625.717	322.562	173.484	979.946	928.299	711.76	Up
Birc3	4446.42	0.6269	0.13239	4.7351	2.19E-06	7.82E-05	3788.57	3724.76	2972.61	5130.19	5295.73	5766.65	Up
Cfap300	1010.42	-1.1467	0.32086	-3.5739	0.00035	0.0049	1729.29	879.895	1567.2	659.756	386.362	839.996	Down
Cep126	3630.58	-0.8102	0.29881	-2.7112	0.0067	0.0461	5680.41	2926.74	5264.94	2775.66	1706.17	3429.57	Down
Gm19324	60.5083	1.00823	0.29277	3.44381	0.00057	0.00718	41.1269	40.4436	38.9851	71.38	88.6057	82.5085	Up
Trpc6	301.09	0.32827	0.15442	2.1258	0.03352	0.14625	249.699	293.956	257.302	322.23	351.332	332.022	Up
Gm32014	380.472	0.34942	0.15254	2.29074	0.02198	0.10868	333.912	362.02	307.982	384.433	447.15	447.335	Up
Pgr	212.918	0.69602	0.30716	2.26597	0.02345	0.11357	109.672	225.892	152.042	210.061	319.393	260.449	Up
Cep57	2487.23	-0.2177	0.10224	-2.129	0.03325	0.1454	2696.75	2475.94	2850.78	2391.23	2221.32	2287.37	Down
Gm47557	15.9818	-1.1836	0.59138	-2.0015	0.04534	0.17885	19.5843	20.715	26.3149	9.17743	6.18179	13.9171	Down
Sesn3	2787.6	-0.4189	0.13098	-3.1986	0.00138	0.0141	3140.34	3566.93	2861.51	2273.96	2260.47	2622.38	Down
Endod1	4001.91	-0.2846	0.11142	-2.5541	0.01065	0.06485	4590.55	4099.6	4495.96	3589.4	3309.32	3926.61	Down
1700012B	136.896	-3.0756	0.31011	-9.9176	3.49E-23	3.30E-20	249.699	184.462	300.185	26.5126	24.7272	35.7868	Down
4931406C	5678.16	-0.4855	0.22216	-2.1852	0.02887	0.13173	7220.71	4768.4	7884.73	4812.03	3759.56	5623.5	Down
Mtnr1b	1.85517	4.36368	2.09671	2.0812	0.03742	NA	0	0	0	2.03943	4.12119	4.97039	Up
Fat3	814.887	-0.4281	0.12731	-3.3628	0.00077	0.00895	890.104	949.932	964.881	701.564	624.361	758.482	Down
Angptl6	77.9885	-0.5656	0.25756	-2.1958	0.0281	0.12915	99.8797	83.8465	95.5135	67.3012	59.7573	61.6329	Down
Icam1	36514.6	0.42357	0.13724	3.08631	0.00203	0.01897	27591.3	37078.9	28907.4	41835.8	42424.6	41249.3	Up
Dnm2	10306.4	0.31647	0.09495	3.33299	0.00086	0.00974	9077.3	9591.06	8873.01	11247.5	12263.6	10785.8	Up
Tmed1	847.481	-0.3253	0.11855	-2.7437	0.00608	0.0431	911.647	972.62	943.439	795.378	777.875	683.926	Down
Ldlr	7954.16	0.56659	0.13063	4.33723	1.44E-05	0.00037	6891.7	6648.54	5695.72	9151.94	10638.9	8698.19	Up
Spc24	171.626	-0.5816	0.24639	-2.3605	0.01825	0.09622	165.487	211.096	240.733	158.056	110.242	144.141	Down
Plppr2	607.058	-0.3705	0.14493	-2.5562	0.01058	0.06461	593.403	762.51	697.833	547.587	515.149	525.868	Down
Ccdc151	1247.54	-1.0007	0.27285	-3.6674	0.00025	0.00375	1947.65	1101.84	1941.46	1033.99	593.452	866.836	Down
Elavl3	2.93951	-4.9774	1.79071	-2.7796	0.00544	NA	4.89606	5.91858	6.82239	0	0	0	Down
Elof1	9433.62	-0.9278	0.20754	-4.4706	7.80E-06	0.00023	13196.9	9104.75	14798.7	7087.02	5268.95	7145.44	Down
Zfp599	143.641	-0.5324	0.24501	-2.1732	0.02977	0.13438	183.113	165.72	160.814	126.445	87.5754	138.177	Down
Dpy19l2	51.042	-0.825	0.3911	-2.1095	0.0349	0.15024	65.6073	43.4029	86.7418	30.5914	39.1513	40.7572	Down
Gm48393	3.43868	-3.1931	1.60398	-1.9907	0.04651	NA	5.87528	3.94572	8.77165	2.03943	0	0	Down
B3gat1	8.89627	-1.6026	0.78999	-2.0286	0.0425	0.1714	14.6882	8.87787	16.5687	4.07886	6.18179	2.98224	Down
Glb1l2	2341.3	-0.6707	0.22341	-3.0021	0.00268	0.02359	2696.75	2408.86	3522.3	2106.73	1298.18	2015	Down
Glb1l3	163.728	-0.6525	0.26726	-2.4414	0.01463	0.08212	149.82	191.367	259.251	126.445	113.333	142.153	Down
Jam3	854.613	-1.185	0.26084	-4.5431	5.54E-06	0.00017	1476.65	732.917	1351.81	526.173	461.574	578.554	Down
Igsf9b	314	-0.9976	0.24597	-4.0559	4.99E-05	0.00105	478.835	466.581	309.931	163.154	233.878	231.62	Down
Barx2	529.448	-0.7029	0.2998	-2.3446	0.01905	0.09891	745.181	363.006	859.621	406.866	361.635	440.377	Down
Kcnj5	52.7578	-2.0521	0.79909	-2.5681	0.01023	0.06298	124.36	9.8643	120.854	14.276	13.3939	33.7987	Down
Gm32171	18.3574	-1.3109	0.6535	-2.006	0.04486	0.17765	20.5635	32.5522	25.3403	10.1971	17.5151	3.97631	Down
Kirrel3	158.413	0.58453	0.23493	2.48811	0.01284	0.07423	119.464	134.154	126.702	214.14	145.272	210.745	Up
St3gal4	3569.88	0.46324	0.1213	3.81886	0.00013	0.00236	3084.52	3228.58	2691.92	3956.49	3934.71	4523.06	Up
4930581F	64.0575	0.73686	0.32529	2.26522	0.0235	0.11362	47.9814	56.2265	39.9597	61.1829	86.5451	92.4493	Up
Ei24	1883.62	0.29852	0.11417	2.61472	0.00893	0.05694	1756.71	1698.63	1613.01	1864.04	2187.32	2182	Up
Tmem218	556.754	-0.4549	0.17468	-2.6041	0.00921	0.05822	764.765	577.061	589.65	505.779	414.18	489.087	Down
Hepacam	243.275	-1.3782	0.19126	-7.2061	5.76E-13	9.90E-11	379.935	309.739	364.511	148.878	116.424	140.165	Down
Msantd2	2314.69	-0.3069	0.14205	-2.1602	0.03075	0.13735	2793.69	2234.26	2651.96	2029.23	1862.78	2316.2	Down
Vsig2	2175.1	0.42232	0.13192	3.2013	0.00137	0.01401	1809.59	1983.71	1783.57	2518.7	2786.96	2168.09	Up
Nrgn	1084.2	-1.1604	0.14979	-7.747	9.41E-15	2.31E-12	1475.67	1320.83	1697.8	654.657	743.875	612.352	Down
Vwa5a	6889.81	0.20309	0.08628	2.35373	0.01859	0.09738	6032.93	6459.14	6724.93	7232.84	7457.3	7431.73	Up
Scn3b	3849.64	0.29241	0.12607	2.31939	0.02037	0.10356	3644.63	3297.63	3440.43	3837.19	4039.8	4838.18	Up
Gramd1b	2592.66	-0.4195	0.1954	-2.147	0.0318	0.14055	2712.42	2405.9	3782.53	2393.27	2354.23	1907.64	Down
Gm48284	6.88132	-3.1903	1.12604	-2.8332	0.00461	NA	9.79213	8.87787	18.5179	2.03943	2.0606	0	Down
Jhy	823.49	-1.5742	0.2931	-5.3708	7.84E-08	4.55E-06	1554.01	699.379	1445.37	483.345	349.271	409.56	Down
Crtam	36.6896	-0.8935	0.40749	-2.1928	0.02832	0.12991	33.2932	56.2265	53.6045	26.5126	22.6666	27.8342	Down
Ubash3b	554.882	0.60245	0.17761	3.39202	0.00069	0.00828	400.498	535.631	385.952	614.888	724.3	668.021	Up
Sorl1	10438.5	-0.3556	0.09617	-3.6981	0.00022	0.00341	11678.1	11379.5	12098	9520.06	9561.17	8394	Down
4930546K	12.7626	3.13296	0.7892	3.96978	7.19E-05	0.00141	3.91685	1.97286	1.94925	19.3746	18.5454	30.8164	Up
Sc5d	4255.61	0.78917	0.11583	6.81342	9.53E-12	1.27E-09	3178.52	3342.02	2839.09	4926.24	5705.79	5541.99	Up
Grik4	75.456	1.67733	0.35817	4.68309	2.83E-06	9.60E-05	30.3556	38.4708	38.9851	90.7546	160.727	93.4434	Up
Oaf	337.884	0.54784	0.20021	2.73629	0.00621	0.04389	301.598	274.227	247.555	412.985	470.846	320.093	Up
D630033C	4.89579	2.7006	1.30905	2.06302	0.03911	NA	0	0.98643	2.92388	14.276	7.21209	3.97631	Up
Trim29	1058.24	1.98874	0.21679	9.17342	4.58E-20	2.63E-17	421.061	494.201	362.561	1607.07	2154.35	1310.2	Up
Cbl	3306.49	0.26649	0.09015	2.95608	0.00312	0.02641	3016.95	2995.79	2993.08	3432.36	3840.95	3559.8	Up
Ccdc153	10234.1	-0.9664	0.27761	-3.4813	0.0005	0.00643	16371.5	8598.71	15647.6	7788.58	4765.13	8232.96	Down
Nlrx1	823.084	0.50649	0.19222	2.63491	0.00842	0.0546	599.278	762.51	678.341	871.856	1204.42	822.103	Up
C2cd2l	3336.47	0.31418	0.10654	2.94905	0.00319	0.02681	3283.3	2792.58	2847.86	3810.67	3669.92	3614.47	Up
Dpagt1	1271.85	0.25784	0.08967	2.87532	0.00404	0.03188	1155.47	1172.86	1147.14	1394.97	1387.81	1372.82	Up
H2ax	850.296	-0.2877	0.14302	-2.0114	0.04428	0.17616	964.525	874.963	964.881	875.935	676.906	744.565	Down
Gm47232	82.6956	-0.6102	0.2735	-2.231	0.02568	0.12108	99.8797	96.6701	103.31	64.242	52.5452	79.5263	Down
Phldb1	2546.4	0.27246	0.115	2.36931	0.01782	0.09485	2404.95	2074.46	2440.47	2655.34	2974.47	2728.75	Up
Ift46	4031.95	-0.3672	0.15781	-2.3267	0.01998	0.10219	4687.49	3756.32	5183.07	3903.47	3139.32	3522.02	Down
Mpzl3	575.285	0.47626	0.12046	3.95385	7.69E-05	0.0015	450.438	487.296	505.832	653.637	666.603	687.902	Up
Scn4b	28.1903	-1.8257	0.93076	-1.9615	0.04982	0.19052	63.6488	6.90501	61.4015	13.2563	2.0606	21.8697	Down

Tmprss4	1920.95	0.30363	0.11091	2.73772	0.00619	0.04372	1627.45	1890.99	1640.3	2023.11	2169.81	2174.05 Up
Cep164	2247.14	-0.3121	0.15359	-2.032	0.04216	0.1706	2478.39	2058.68	2930.7	2043.51	1900.9	2070.67 Down
Rnf214	1044.09	-0.2758	0.12532	-2.2005	0.02777	0.12807	1148.62	1018.98	1263.12	952.414	923.147	958.292 Down
Sidt2	6495.21	0.30624	0.12545	2.4411	0.01464	0.08216	5353.36	6526.22	5545.63	7058.47	7830.27	6657.34 Up
Apoa1	8.38247	-1.9765	0.97972	-2.0174	0.04366	0.17454	10.7713	5.91858	23.3911	5.09857	4.12119	0.99408 Down
Bud13	862.746	-0.2321	0.09976	-2.3266	0.01998	0.10219	931.231	924.285	940.515	778.042	790.239	812.162 Down
Gm5617	307.473	-0.6405	0.16762	-3.8211	0.00013	0.00235	391.685	336.372	395.699	233.515	267.878	219.691 Down
Drd2	17.4186	-2.1467	0.58888	-3.6455	0.00027	0.00399	32.314	27.62	25.3403	8.15772	4.12119	6.95855 Down
Ankk1	154.766	-1.4161	0.33819	-4.1872	2.82E-05	0.00065	189.967	178.544	307.008	105.031	54.6058	93.4434 Down
Ttc12	2596.6	-0.7568	0.22743	-3.3274	0.00088	0.00988	3492.85	2390.12	3904.36	2140.38	1460.96	2190.95 Down
Ncam1	123.674	-0.8152	0.25892	-3.1484	0.00164	0.01608	188.988	124.29	159.839	101.971	83.4542	83.5026 Down
Il18	1481.51	0.936	0.20357	4.59793	4.27E-06	0.00014	1106.51	1171.88	772.879	1922.16	2289.32	1626.31 Up
Pih1d2	1495.79	-0.8925	0.25381	-3.5164	0.00044	0.00579	2078.87	1362.26	2391.74	1078.86	772.724	1290.31 Down
Dixdc1	2590.88	-0.3472	0.14709	-2.3605	0.01825	0.09622	2790.76	2666.32	3246.48	2360.64	1944.17	2536.89 Down
1110032A	3243.49	-0.4355	0.15964	-2.7282	0.00637	0.04454	3940.35	3143.75	4104.16	2770.57	2371.75	3130.35 Down
Btg4	399.287	-1.0657	0.33106	-3.2192	0.00129	0.01339	746.16	318.617	556.512	247.791	191.636	335.004 Down
Gm32742	394.586	0.95022	0.288	3.29933	0.00097	0.01073	302.577	159.802	345.018	587.356	439.937	532.826 Up
Pou2af1	508.598	0.533	0.24363	2.18772	0.02869	0.13104	315.307	578.048	353.79	573.08	641.876	589.489 Up
2010007H	1179.38	0.5107	0.25486	2.00382	0.04509	0.17827	1225	607.641	1085.73	1280.76	1280.66	1596.49 Up
Arhgap20	35.355	2.05107	0.50785	4.03869	5.38E-05	0.00111	9.79213	22.6879	8.77165	56.0843	70.0603	44.7335 Up
Rdx	15975.8	0.21955	0.09735	2.2553	0.02411	0.11594	13655.1	15307.4	15325	16939.5	18162.1	16465.9 Up
4930550C	282.986	-0.5881	0.29536	-1.991	0.04648	0.18197	378.955	281.132	359.637	253.909	135.999	288.283 Down
Acat1	5256.33	-0.3234	0.08514	-3.7986	0.00015	0.00251	5826.32	5572.34	6130.41	4747.79	4561.13	4700 Down
Elmod1	1753.53	-0.9971	0.32378	-3.0795	0.00207	0.01931	2723.19	1458.93	2827.39	1147.18	722.239	1642.22 Down
Tnfaip8l3	765.427	-0.507	0.21856	-2.3197	0.02036	0.10351	1031.11	979.525	685.163	541.469	575.937	779.358 Down
Dmxl2	2720.34	0.60836	0.11955	5.08885	3.60E-07	1.70E-05	2180.71	2123.78	2160.75	3170.29	3713.2	2973.29 Up
Cib2	543.659	0.61429	0.21728	2.82726	0.00469	0.03577	452.396	508.998	327.475	690.347	745.936	536.802 Up
Idh3a	5137.21	-0.2425	0.0992	-2.4442	0.01452	0.08156	5867.44	5073.21	5762.97	4668.25	4628.1	4823.27 Down
Chrnb4	56.0798	1.38462	0.41541	3.33314	0.00086	0.00974	19.5843	40.4436	33.1373	86.6758	100.969	55.6684 Up
Etfa	5682.4	-0.4717	0.10768	-4.3805	1.18E-05	0.00032	6800.63	5987.63	7021.21	5049.63	4561.13	4674.16 Down
Isl2	8.69724	-2.2439	0.83114	-2.6998	0.00694	0.04726	13.709	13.81	15.594	2.03943	2.0606	4.97039 Down
Pstpip1	643.979	0.34726	0.17574	1.97594	0.04816	0.18643	476.877	697.406	526.299	741.333	719.148	702.814 Up
Tspan3	4650.46	-0.4426	0.13862	-3.1929	0.00141	0.01426	5584.45	4597.75	5892.6	4206.32	3534.95	4086.66 Down
Cspg4	979.9	0.74291	0.13202	5.62725	1.83E-08	1.26E-06	730.493	710.229	758.26	1357.24	1244.6	1078.58 Up
Gm47237	2.39019	4.72768	2.19821	2.15069	0.0315	NA	0	0	0	1.01971	11.3333	1.98816 Up
Ptpn9	554.517	0.43993	0.16096	2.73311	0.00627	0.04413	511.149	425.151	475.618	553.705	638.785	722.695 Up
Scamp5	872.65	0.8529	0.2179	3.91423	9.07E-05	0.00171	504.295	730.944	630.584	1015.64	1426.96	927.475 Up
Gm18103	893.048	-2.0509	1.03088	-1.9894	0.04665	0.18251	1023.28	1952.14	1341.09	77.4983	848.966	115.313 Down
Scamp2	4964.94	0.26695	0.08807	3.03116	0.00244	0.02196	4552.36	4447.81	4520.32	5537.05	5655.31	5076.76 Up
Cplx3	2.38843	4.72655	2.09595	2.25509	0.02413	NA	0	0	0	2.03943	10.303	1.98816 Up
Ccdc33	2023.97	-1.1238	0.26876	-4.1814	2.90E-05	0.00067	3156.98	1760.78	3406.32	1381.71	947.875	1490.12 Down
Stra6	60.8671	-2.6764	0.61882	-4.3251	1.52E-05	0.00039	125.339	39.4572	151.067	9.17743	11.3333	28.8283 Down
4930461G	1.80091	-4.2701	2.13776	-1.9975	0.04577	NA	2.93764	5.91858	1.94925	0	0	0 Down
Insyn1	1090.11	-0.9871	0.3736	-2.6421	0.00824	0.05369	1284.73	2401.96	660.797	657.716	753.148	782.34 Down
Cd276	681.995	0.44219	0.17651	2.50526	0.01224	0.07163	518.004	694.446	522.4	737.254	891.208	728.66 Up
Hexa	8956.36	0.54	0.14377	3.75609	0.00017	0.00285	7263.8	8021.65	6612.85	10895.7	11931.9	9012.32 Up
Celf6	24.4177	-1.1889	0.55765	-2.1319	0.03301	0.14451	47.9814	19.7286	34.112	10.1971	19.5757	14.9112 Down
Parp6	2389.01	-0.232	0.11737	-1.9768	0.04806	0.18628	2463.7	2851.77	2426.82	2154.66	2080.17	2356.96 Down
9230112J1	66.4806	-1.3616	0.31137	-4.3728	1.23E-05	0.00033	80.2954	95.6837	111.108	39.7689	41.2119	30.8164 Down
Uaca	2232.23	0.31141	0.13102	2.37675	0.01747	0.0935	1785.1	2257.94	1933.66	2544.19	2588.11	2284.39 Up
Kif23	639.261	0.47224	0.18636	2.53395	0.01128	0.06751	472.96	594.817	538.969	869.817	596.543	762.458 Up
Paqr5	204.364	-0.588	0.28245	-2.0817	0.03737	0.15753	305.514	169.666	261.2	156.016	134.969	198.816 Down
Itga11	383.608	-0.6258	0.27797	-2.2512	0.02437	0.1167	455.334	397.531	543.842	281.441	203.999	419.501 Down
Calml4	1936.13	-0.9993	0.28195	-3.5442	0.00039	0.00536	2925.89	1700.6	3116.86	1468.39	859.269	1545.79 Down
Smad6	4578.68	-0.3554	0.17212	-2.0651	0.03891	0.16209	5776.38	5145.22	4497.9	4099.25	4714.65	3238.71 Down
Lctl	15.6657	-7.3913	1.77426	-4.1659	3.10E-05	0.00071	84.2123	2.95929	6.82239	0	0	0 Down
Map2k1	5674.8	0.3126	0.1183	2.64245	0.00823	0.05366	5191.79	5507.24	4488.16	6100.95	6721.67	6039.03 Up
Megf11	499.573	-1.0429	0.39699	-2.627	0.00861	0.05545	1060.49	277.187	680.29	342.624	357.514	279.336 Down
Hacd3	10527.1	0.27511	0.10807	2.54564	0.01091	0.06585	9422.96	10501.5	8654.69	11332.1	11979.3	11271.9 Up
Dpp8	399.799	0.64669	0.18809	3.43819	0.00059	0.0073	352.517	302.834	279.718	410.945	482.18	570.601 Up
Clpx	3443.71	-0.247	0.09421	-2.6221	0.00874	0.05607	3963.85	3534.38	3715.28	3106.05	3067.2	3275.49 Down
Pdcd7	1079.77	-0.2305	0.10386	-2.2196	0.02644	0.12374	1151.55	1235.01	1111.08	1009.52	966.42	1005.01 Down
Plekho2	1742.23	0.42469	0.21463	1.97874	0.04785	0.18579	1554.01	1674.96	1233.88	1649.9	2615.93	1724.73 Up
Snx22	287.026	-1.1732	0.3896	-3.0114	0.0026	0.02304	413.228	590.871	189.078	147.859	152.484	228.638 Down
Ciao2a	3426.76	0.27118	0.11646	2.32857	0.01988	0.10187	2892.59	3466.31	2957.99	3809.65	3909.98	3524.01 Up
Usp3	3045.1	0.37782	0.09607	3.9326	8.40E-05	0.0016	2495.03	2829.08	2621.75	3426.24	3551.44	3347.06 Up
Gm10647	113.991	-0.8832	0.39203	-2.253	0.02426	0.11637	201.718	93.7108	148.143	78.518	51.5149	110.343 Down
Ppp1r2-ps	27.3296	-1.5163	0.5186	-2.9239	0.00346	0.02851	37.2101	43.4029	40.9343	18.3549	6.18179	17.8934 Down
Aph1c	809.795	0.69307	0.13158	5.26725	1.38E-07	7.38E-06	584.59	597.776	674.442	1083.96	916.966	1001.04 Up
Rab8b	3395.01	0.55775	0.15005	3.71705	0.0002	0.00321	2577.29	3137.83	2525.26	3612.85	4652.83	3863.98 Up
Gm19299	364.736	-1.0527	0.2299	-4.5788	4.68E-06	0.00015	571.86	438.961	465.872	273.284	171.03	267.407 Down
Vps13c	1065.8	0.5477	0.13684	4.00263	6.26E-05	0.00125	932.211	880.882	784.575	1131.88	1389.87	1275.4 Up
B230323A	111.774	7.40882	0.90787	8.16063	3.33E-16	1.11E-13	1.95843	1.97286	0	202.923	383.271	80.5204 Up
Fam81a	974.486	-1.181	0.2738	-4.3132	1.61E-05	0.00041	1565.76	863.126	1628.6	582.257	462.604	744.565 Down
Adam10	8057.67	0.23627	0.10096	2.34023	0.01927	0.09967	6947.51	7865.79	7384.75	8107.75	9124.32	8915.89 Up
Aqp9	202.52	1.63848	0.30418	5.38651	7.18E-08	4.23E-06	79.3162	142.046	74.0717	274.303	373.998	271.383 Up
Gm27188	32.0176	-1.0044	0.50426	-1.9919	0.04639	0.18179	42.1061	26.6336	59.4523	29.5717	15.4545	18.8875 Down
Mns1	546.714	-0.8277	0.30871	-2.6812	0.00734	0.04923	879.333	456.717	762.159	439.497	254.484	488.093 Down
Tex9	347.272	-0.5126	0.22813	-2.2471	0.02463	0.11745	399.519	327.495	498.035	300.816	228.726	329.04 Down
Pygo1	877.659	-0.4546	0.11692	-3.8882	0.0001	0.00188	1012.51	951.905	1079.89	713.8	787.148	720.707 Down
Rab27a	6921.5	0.53601	0.19825	2.70369	0.00686	0.04687	5664.75	6838.92	4447.22	7759.01	9876.44	6942.64 Up
Wdr72	323.11	-1.1886	0.48051	-2.4735	0.01338	0.07656	633.551	211.096	502.908	173.352	91.6966	326.058 Down
Myo5a	1947.32	0.95376	0.15888	6.00304	1.94E-09	1.61E-07	1272	1440.19	1266.04	2382.05	3092.96	2230.71 Up

Gnb5	995.654	-0.6619	0.16778	-3.9451	7.98E-05	0.00154	1360.13	1084.09	1216.33	718.899	677.936	916.54	Down
Gm7972	55.1458	-0.9424	0.36878	-2.5555	0.0106	0.06467	73.441	83.8465	60.4269	33.6506	27.8181	51.6921	Down
Tmod3	12510.4	0.2103	0.07886	2.66678	0.00766	0.05075	11472.5	11853.9	11474.3	12804.6	13733.9	13723.3	Up
Bmp5	1477.15	0.3707	0.13575	2.73077	0.00632	0.04434	1362.08	1332.67	1170.53	1451.05	1772.11	1774.43	Up
Hmgcll1	1849.05	-0.3931	0.10718	-3.6675	0.00024	0.00375	2046.55	2072.49	2179.27	1664.17	1454.78	1677.01	Down
Tinag	1334.37	-0.9841	0.35409	-2.7793	0.00545	0.03977	1800.77	2429.58	1087.68	753.569	569.755	1364.87	Down
Mlip	40.4466	-2.3685	0.74454	-3.1811	0.00147	0.01475	98.9005	12.8236	91.615	11.2169	8.24239	19.8816	Down
Klhl31	125.057	-4.0213	0.3702	-10.863	1.74E-27	2.74E-24	251.658	170.652	284.591	13.2563	10.303	19.8816	Down
Elovl5	17635.1	0.45834	0.07915	5.79068	7.01E-09	5.33E-07	15057.4	14197.7	15316.3	20625.8	19855.9	20757.4	Up
Cilk1	4671.6	0.35528	0.12249	2.90036	0.00373	0.03016	4258.6	3536.35	4502.78	5240.31	5374.04	5117.52	Up
Gsta1	16.2725	-1.9339	0.60416	-3.201	0.00137	0.01401	21.5427	25.6472	30.2134	8.15772	4.12119	7.95263	Down
Gsta2	140.613	-1.4392	0.54484	-2.6416	0.00825	0.05374	109.672	157.829	348.917	104.011	27.8181	95.4315	Down
Dppa5a	13.9867	-2.0569	0.68109	-3.0201	0.00253	0.0226	13.709	27.62	26.3149	6.11829	6.18179	3.97631	Down
Ooep	103.757	-0.9049	0.33946	-2.6658	0.00768	0.05085	98.9005	158.815	148.143	90.7546	47.3937	78.5322	Down
Gm8093	3.8678	2.78844	1.32542	2.10382	0.03539	NA	0.97921	0	1.94925	7.138	6.18179	6.95855	Up
Tmem30a	30578.4	0.49791	0.12518	3.97756	6.96E-05	0.00138	24355	28624.2	23081.1	32925.6	38541.4	35942.9	Up
Htr1b	436.97	-0.8641	0.37585	-2.2992	0.02149	0.10714	793.162	284.092	614.99	300.816	212.241	416.519	Down
D430036J	1225.02	-0.8563	0.29355	-2.9172	0.00353	0.02899	1843.86	1085.07	1805.98	974.847	540.907	1099.45	Down
Irak1bp1	704.184	-0.5184	0.22466	-2.3075	0.02102	0.1059	883.25	623.424	981.45	644.46	453.331	639.192	Down
Hmgn3	1026.49	-0.671	0.19871	-3.3767	0.00073	0.00861	1330.75	976.565	1475.59	931	696.482	748.541	Down
Lca5	1466.73	-0.5352	0.25209	-2.123	0.03375	0.14684	2108.25	1190.62	1908.32	1186.95	921.087	1485.15	Down
Gm36120	4.53174	3.68748	1.62295	2.27209	0.02308	NA	1.95843	0	0	12.2366	2.0606	10.9349	Up
Rwdd2a	96.6152	0.80799	0.27729	2.91388	0.00357	0.02925	89.1084	67.0772	54.5791	120.326	115.393	133.207	Up
A330041J	65.5011	-0.8696	0.34961	-2.4872	0.01287	0.07432	75.3994	101.602	76.9956	63.2223	35.0301	40.7572	Down
Cyb5r4	3290.29	0.42408	0.12029	3.52555	0.00042	0.00564	2561.62	2905.04	2963.84	3381.37	3962.53	3967.37	Up
Gm28231	32.7274	-3.6812	0.66641	-5.5239	3.32E-08	2.08E-06	102.817	33.5386	45.8075	4.07886	5.15149	4.97039	Down
Syncrip	1654.57	0.28967	0.12049	2.40403	0.01622	0.08846	1558.91	1469.78	1438.55	1710.06	1710.3	2039.85	Up
9430037G	30.0577	0.87358	0.43995	1.98565	0.04707	0.18351	22.5219	17.7557	23.3911	27.5323	47.3937	41.7513	Up
Gm10634	25.2751	1.065	0.46309	2.29976	0.02146	0.10711	18.605	18.7422	11.6955	38.7492	35.0301	28.8283	Up
Bcl2a1a	492.066	1.2292	0.25587	4.80405	1.55E-06	5.84E-05	320.203	343.278	219.291	645.48	894.299	529.844	Up
Rasgrf1	161.784	-1.4832	0.44881	-3.3047	0.00095	0.01057	153.736	153.883	407.394	91.7743	53.5755	110.343	Down
Ctsh	34601.1	0.3827	0.10839	3.53069	0.00041	0.00558	32121.1	27553	30441.5	42357.9	36983.6	38149.8	Up
Tbc1d2b	502.051	1.0635	0.174	6.11207	9.83E-10	8.78E-08	351.537	290.01	333.323	564.922	715.027	757.488	Up
Plscr1	7075.15	0.26172	0.11104	2.35709	0.01842	0.09677	6911.28	5678.88	6715.18	7545.89	7877.66	7722	Up
1700065D	2.28353	-4.6133	2.02164	-2.2819	0.02249	NA	1.95843	3.94572	7.79702	0	0	0	Down
Gk5	331.833	0.66901	0.18389	3.63801	0.00027	0.00407	283.972	275.214	209.545	383.413	448.18	390.673	Up
Gm28688	25.8046	-1.7537	0.57247	-3.0634	0.00219	0.0202	47.0022	26.6336	45.8075	16.3154	5.15149	13.9171	Down
Gm8520	60.7923	-1.5201	0.32232	-4.7162	2.40E-06	8.48E-05	76.3786	93.7108	100.387	36.7097	25.7575	31.8105	Down
Pxylp1	913.882	-0.8618	0.37316	-2.3095	0.02092	0.10547	928.294	792.103	1816.71	579.198	399.756	967.238	Down
Spsb4	74.93	-1.3821	0.36221	-3.8157	0.00014	0.00238	109.672	70.0365	145.219	42.828	37.0907	44.7335	Down
Nmnat3	267.629	0.6692	0.23252	2.87806	0.004	0.03169	164.508	187.422	268.023	314.072	303.938	367.809	Up
Mrps22	731.421	-0.2554	0.12925	-1.9757	0.04819	0.18648	831.352	753.632	803.093	677.091	600.664	722.695	Down
Cep70	1229.48	0.28376	0.1383	2.0518	0.04019	0.16536	1038.94	1018	1269.94	1427.6	1245.63	1376.8	Up
Gm37249	117.15	0.51706	0.22487	2.29941	0.02148	0.10712	93.0252	100.616	95.5135	156.016	120.545	137.183	Up
Esyt3	907.89	0.47349	0.18453	2.56589	0.01029	0.06325	776.516	756.592	747.539	1340.93	923.147	902.623	Up
Nme9	4778.42	-1.0061	0.26944	-3.7339	0.00019	0.00305	7637.86	4163.72	7338.94	3693.41	2216.17	3620.43	Down
A4gnt	6.90399	-3.6438	1.41546	-2.5743	0.01005	NA	5.87528	24.6607	7.79702	0	3.0909	0	Down
Dzip1l	2277.63	-0.657	0.20429	-3.216	0.0013	0.01347	2919.03	2125.76	3317.63	1995.58	1427.99	1879.8	Down
Gm34397	31.4633	2.95552	0.51542	5.7342	9.80E-09	7.24E-07	9.79213	5.91858	5.84776	40.7886	61.8179	64.6151	Up
Il20rb	239.311	0.41445	0.17563	2.35982	0.01828	0.09622	190.946	209.123	215.393	304.895	270.968	244.543	Up
Stag1	3370.15	0.20222	0.1005	2.0122	0.0442	0.17589	3008.14	3371.62	3023.29	3430.32	3776.04	3611.49	Up
Pccb	3909.15	-0.2121	0.08195	-2.5879	0.00966	0.06019	4037.29	4256.44	4294.21	3602.65	3550.41	3713.88	Down
Ephb1	24.0381	1.1383	0.52567	2.16545	0.03035	0.13621	21.5427	10.8507	12.6702	37.7295	22.6666	38.7691	Up
Ky	837.821	-1.8065	0.37584	-4.8067	1.53E-06	5.77E-05	1437.48	676.691	1795.26	353.841	231.817	531.832	Down
Cep63	2347.15	-0.2918	0.11155	-2.6158	0.0089	0.05685	2819.15	2482.84	2449.24	2074.1	2004.96	2252.58	Down
Ryk	6098.73	-0.2164	0.08228	-2.6305	0.00853	0.0551	6744.82	6225.36	6695.69	5707.34	5683.13	5536.02	Down
1300017J(	72.2794	-1.5313	0.29861	-5.128	2.93E-07	1.43E-05	108.693	89.7651	123.778	35.69	33.9998	41.7513	Down
Tmem108	174.552	-0.7487	0.21489	-3.4839	0.00049	0.00639	189.967	219.974	246.581	150.918	121.575	118.295	Down
Gm29154	41.5038	-3.128	0.70499	-4.437	9.12E-06	0.00026	72.4617	70.0365	80.8941	12.2366	13.3939	0	Down
Gm28305	2.02159	4.48781	2.04969	2.18951	0.02856	NA	0	0	0	5.09857	2.0606	4.97039	Up
Nek11	811.12	-0.8327	0.30909	-2.6941	0.00706	0.04781	1148.62	688.528	1279.69	657.716	358.544	733.63	Down
4930500F	10.6903	-1.8169	0.74049	-2.4536	0.01414	0.07991	16.6466	15.7829	17.5433	7.138	2.0606	4.97039	Down
Poc1a	441.919	-0.4941	0.16292	-3.0327	0.00242	0.02187	430.854	559.306	560.411	378.314	365.756	356.874	Down
Abhd14b	1581.47	-0.2308	0.10241	-2.2535	0.02423	0.11625	1726.35	1640.43	1756.28	1550.99	1372.36	1442.41	Down
Mapkapk3	3719.29	0.52102	0.09308	5.59742	2.18E-08	1.46E-06	3060.04	3047.08	3057.41	4329.71	4695.07	4126.42	Up
Hemk1	770.981	-0.2631	0.12685	-2.0737	0.03811	0.15971	825.476	823.669	874.241	671.992	651.149	779.358	Down
Zmynd10	503.94	-1.0602	0.27948	-3.7934	0.00015	0.00254	792.183	495.188	756.311	363.019	215.332	401.608	Down
Mst1	310.926	-0.9834	0.4001	-2.4579	0.01397	0.07919	541.505	217.015	480.491	182.529	131.878	312.141	Down
Bsn	123.535	0.71914	0.2756	2.60942	0.00907	0.05756	94.0044	72.0094	114.031	132.563	185.454	143.147	Up
Nicn1	1090.51	-0.2717	0.10022	-2.7109	0.00671	0.04611	1215.2	1221.2	1142.26	1023.79	965.39	975.191	Down
Amt	867.096	-0.3996	0.19022	-2.1007	0.03567	0.15271	1121.2	901.597	936.617	691.367	623.331	928.469	Down
Gpx1	21369.4	0.25827	0.12436	2.07685	0.03782	0.15879	17902.9	21426.2	19055.9	23985.7	25130	20715.6	Up
1700102P	129.236	-0.4891	0.24294	-2.0132	0.0441	0.17562	181.154	140.073	131.575	92.7941	118.484	111.337	Down
Ndufaf3	1352.7	-0.2996	0.10654	-2.8125	0.00492	0.03691	1546.18	1397.77	1534.06	1273.62	1149.81	1214.76	Down
Dalrd3	5666.05	-0.3396	0.14861	-2.285	0.02231	0.10976	7009.2	5313.9	6666.45	5409.59	4421.01	5176.17	Down
P4htm	639.561	-0.5167	0.14931	-3.4605	0.00054	0.00683	728.534	737.849	792.372	565.942	444.059	568.613	Down
Celsr3	68.3919	0.93074	0.30198	3.08211	0.00206	0.01918	38.1893	55.2401	47.7567	82.5969	104.06	82.5085	Up
Pfkfb4	5072.25	0.64221	0.0985	6.5198	7.04E-11	7.98E-09	4003.02	4143.99	3737.7	5976.55	6620.7	5951.55	Up
Shisa5	18688.4	0.3202	0.09967	3.21255	0.00132	0.01359	16227.5	17678.8	15962.4	20810.3	22141.1	19310	Up
Gm42755	9.99203	-2.3013	1.00745	-2.2843	0.02235	0.10989	23.5011	2.95929	23.3911	2.03943	3.0909	4.97039	Down
3000002C	156.583	-0.5288	0.20727	-2.5511	0.01074	0.06513	174.3	202.218	178.357	111.149	144.242	129.23	Down
Camp	40.9964	-1.6673	0.46755	-3.566	0.00036	0.00502	61.6904	51.2943	74.0717	31.6112	13.3939	13.9171	Down
Cdc25a	907.82	0.35505	0.12118	2.93003	0.00339	0.02814	754.973	859.18	775.803	1014.62	1089.03	953.321	Up

Cspg5	39.6848	-0.8441	0.35121	-2.4034	0.01624	0.08855	54.8359	53.2672	44.8329	27.5323	27.8181	29.8224	Down
Ngp	171.421	-1.9367	0.89	-2.1761	0.02955	0.13365	238.928	209.123	367.434	158.056	37.0907	17.8934	Down
Klhl18	1447.24	0.30176	0.09444	3.19524	0.0014	0.0142	1313.12	1329.71	1246.55	1549.97	1594.9	1649.18	Up
Kif9	2422.79	-0.7652	0.2797	-2.7356	0.00623	0.04396	3550.63	1968.91	3632.44	1995.58	1227.09	2162.12	Down
Stac	133.247	2.5624	0.28364	9.03388	1.66E-19	8.25E-17	39.1685	49.3215	27.2896	218.219	261.696	203.786	Up
Arpp21	52.0519	1.34535	0.5597	2.40371	0.01623	0.08851	13.709	33.5386	40.9343	115.228	72.1209	36.7809	Up
Fbxl2	922.909	-0.47	0.17056	-2.7558	0.00585	0.04199	1205.41	878.909	1131.54	812.713	674.845	834.032	Down
Susd5	19.9511	1.2588	0.52087	2.41675	0.01566	0.08617	11.7506	9.8643	13.6448	29.5717	33.9998	20.8756	Up
4930520C	108.79	-0.5974	0.2772	-2.1553	0.03114	0.13833	140.027	126.263	126.702	114.208	67.9997	77.5381	Down
Gpd1l	4041	-0.284	0.09323	-3.0465	0.00232	0.02113	4732.54	4190.35	4389.72	3605.71	3572.04	3755.63	Down
Gm5921	49.5476	-1.2875	0.4225	-3.0473	0.00231	0.0211	47.0022	91.738	72.1224	37.7295	27.8181	20.8756	Down
Tgfbr2	22662.7	0.25966	0.12284	2.11387	0.03453	0.14917	19458.9	23105.1	19322	23934.7	27090.7	23064.6	Up
Rbms3	4243.72	0.22972	0.10594	2.16843	0.03013	0.13544	3747.45	4129.19	3842.96	4418.42	5001.07	4323.25	Up
2610509F:	3.10467	-5.0562	1.75914	-2.8742	0.00405	NA	5.87528	6.90501	5.84776	0	0	0	Down
Platr11	1.96182	-4.3939	2.16897	-2.0258	0.04279	NA	0.97921	5.91858	4.87314	0	0	0	Down
Vill	2344.18	-0.6767	0.2894	-2.3384	0.01936	0.09988	3285.26	1699.62	3667.52	2063.9	1321.87	2026.93	Down
Plcd1	723.009	0.36003	0.14499	2.48319	0.01302	0.07504	645.301	653.016	601.345	794.358	928.299	715.737	Up
Dlec1	1719.06	-0.8684	0.3069	-2.8296	0.00466	0.03555	2761.38	1418.49	2484.32	1351.12	756.239	1542.81	Down
Gm10608	31.6177	-0.8702	0.42188	-2.0627	0.03914	0.16261	38.1893	49.3215	35.0866	26.5126	23.6969	16.8993	Down
Xylb	1010.46	-0.8481	0.20473	-4.1425	3.44E-05	0.00077	1458.05	985.443	1454.14	744.392	598.603	822.103	Down
Scn5a	722.905	-1.7291	0.34581	-5.0001	5.73E-07	2.51E-05	1502.11	560.292	1269.94	301.836	266.847	436.4	Down
Cx3cr1	4035.14	-0.5987	0.18368	-3.2593	0.00112	0.012	5151.64	3794.79	5635.29	3669.95	2762.23	3196.96	Down
Slc25a38	1381.12	-0.2267	0.10401	-2.18	0.02926	0.13281	1521.7	1474.71	1471.69	1294.02	1346.6	1177.98	Down
Eif1b	2389.88	-0.2334	0.09664	-2.4146	0.01575	0.08649	2752.57	2497.64	2497.97	2288.24	2163.63	2139.26	Down
Gm39458	11.0928	2.37169	0.9654	2.4567	0.01402	0.07933	6.85449	1.97286	1.94925	5.09857	27.8181	22.8638	Up
Gm47063	6.0366	4.12499	1.45335	2.83826	0.00454	NA	0	1.97286	0	9.17743	6.18179	18.8875	Up
Ctnnb1	7085.42	0.41265	0.15987	2.58108	0.00985	0.06107	7259.88	5853.47	5123.62	7333.79	7994.09	8947.7	Up
Ulk4	1851.16	-0.7821	0.29282	-2.6707	0.00757	0.05031	2819.15	1487.54	2716.29	1477.57	903.572	1702.86	Down
Cck	8.66521	-3.2142	1.17535	-2.7347	0.00624	0.04405	24.4803	3.94572	18.5179	3.05914	0	1.98816	Down
Lyzl4	165.592	-1.0755	0.45959	-2.3402	0.01927	0.09967	275.159	86.8058	311.881	122.366	71.0906	126.248	Down
Nktr	1876.49	0.38771	0.16318	2.37602	0.0175	0.0936	1699.91	1508.25	1669.54	1930.32	1850.42	2600.51	Up
Hhatl	364.621	-1.197	0.30952	-3.8673	0.00011	0.00201	647.26	319.603	556.512	222.298	160.727	281.324	Down
Ccdc13	565.127	-0.7946	0.29259	-2.7156	0.00662	0.04576	866.603	464.608	819.662	436.438	289.514	513.939	Down
Ackr2	1775.26	0.32589	0.15432	2.1118	0.0347	0.14958	1363.06	1481.62	1882.01	1953.77	2113.14	1857.93	Up
Gask1a	396.301	-0.6566	0.17103	-3.8393	0.00012	0.00221	459.251	547.468	448.329	294.698	277.15	350.91	Down
Abhd5	2748.36	0.33583	0.1275	2.63393	0.00844	0.05469	2461.74	2659.41	2168.55	2981.65	3360.83	2857.98	Up
A730085K	107.414	0.91403	0.31325	2.91791	0.00352	0.02895	81.2747	67.0772	75.0463	107.07	194.726	119.289	Up
Gm47135	18.5585	2.13832	0.7293	2.932	0.00337	0.02803	7.8337	8.87787	3.89851	15.2957	53.5755	21.8697	Up
Clec3b	3050.84	-0.3902	0.18706	-2.0861	0.03697	0.15663	3413.54	3706.02	3263.05	2993.88	2994.05	1934.48	Down
Cdcp1	3535.43	0.615	0.08589	7.16011	8.06E-13	1.36E-10	2880.84	2812.31	2686.07	4179.81	4441.62	4211.91	Up
Lars2	4362.83	-0.2851	0.13877	-2.0547	0.03991	0.16479	4171.45	5447.06	4759.11	4322.57	3797.68	3679.08	Down
Sacm1l	1268.59	0.43598	0.18751	2.32511	0.02007	0.10249	1248.5	925.271	1061.37	1235.89	1363.08	1777.41	Up
Fyco1	1029.52	0.40843	0.11613	3.51695	0.00044	0.00578	928.294	808.872	917.124	1208.36	1110.66	1203.83	Up
Xcr1	338.739	0.43768	0.17899	2.44522	0.01448	0.08146	321.182	249.567	292.388	389.531	435.816	343.951	Up
Ccr1	1098.98	0.73241	0.18197	4.02492	5.70E-05	0.00116	877.375	967.687	632.533	1417.4	1440.36	1258.5	Up
Ccr2	1833.3	0.51736	0.20542	2.51859	0.01178	0.06963	1119.24	1753.87	1651.02	1958.87	2564.41	1952.37	Up
Ccr5	814.212	1.13983	0.19975	5.70633	1.15E-08	8.37E-07	422.041	629.342	473.669	957.512	1103.45	1299.26	Up
2010315B	1441.92	0.48329	0.14563	3.3186	0.0009	0.01012	1080.07	1285.32	1242.65	1522.43	1583.57	1937.46	Up
Ppp1r14c	10451.5	-0.6893	0.18443	-3.7373	0.00019	0.00302	14564.8	14581.4	9559.14	7493.88	7669.54	8840.34	Down
Iyd	678.51	-1.468	0.33127	-4.4315	9.36E-06	0.00026	1127.07	795.062	1068.19	381.373	184.423	514.933	Down
Plekhg1	2008.16	0.70785	0.14316	4.94449	7.63E-07	3.18E-05	1594.16	1713.43	1267.99	2364.72	2547.93	2560.75	Up
Gm21781	453.738	-0.3547	0.15914	-2.2291	0.02581	0.1215	469.043	588.898	469.77	385.452	426.544	382.72	Down
Rgs17	126.985	0.79071	0.27726	2.85191	0.00435	0.03376	87.1499	78.9144	113.057	129.504	152.484	200.804	Up
Cnksr3	1054.71	0.30226	0.14064	2.14915	0.03162	0.13998	901.855	1092.96	839.154	1130.86	1144.66	1218.74	Up
Ulbp1	343.865	0.83948	0.23023	3.64622	0.00027	0.00398	201.718	325.522	212.469	388.511	467.755	467.217	Up
Adgb	2371.91	-0.7757	0.29923	-2.5922	0.00954	0.05968	3757.24	1769.65	3457	1893.61	1214.72	2139.26	Down
Rab32	1604.2	1.08553	0.17095	6.35016	2.15E-10	2.20E-08	1059.51	1055.48	967.805	1917.06	2711.75	1913.6	Up
Plagl1	1337.44	-0.284	0.13554	-2.0953	0.03614	0.15417	1514.84	1346.48	1544.78	1267.51	1047.81	1303.24	Down
Adat2	269.579	-0.4258	0.15701	-2.7119	0.00669	0.04608	290.826	317.63	318.703	244.732	232.847	212.733	Down
Hivep2	851.979	0.29726	0.14399	2.06445	0.03898	0.16224	744.202	715.161	834.281	935.079	830.421	1052.73	Up
Cited2	4894.84	0.74749	0.18853	3.96481	7.35E-05	0.00144	3600.57	4175.56	3187.03	6544.53	7182.21	4679.13	Up
Txlnb	93.2027	-1.5979	0.70574	-2.2641	0.02357	0.11388	203.676	29.5929	187.128	20.3943	31.9393	86.4848	Down
Abracl	1596.12	0.30546	0.12196	2.50467	0.01226	0.07166	1446.3	1442.16	1394.69	1807.95	1932.84	1552.75	Up
Ect2l	1025.94	-0.6697	0.33255	-2.0137	0.04404	0.17551	1571.64	858.194	1349.86	861.659	433.756	1080.56	Down
Nhsl1	764.724	-0.3962	0.14182	-2.7934	0.00522	0.03858	931.231	875.949	800.169	686.268	577.997	716.731	Down
Gm48297	3.04263	4.03536	1.77482	2.27367	0.02299	NA	0.97921	0	0	2.03943	9.27269	5.96447	Up
Arfgef3	4735.88	0.55703	0.20188	2.75922	0.00579	0.04174	3346.95	5028.82	3122.71	4931.34	6189	5796.47	Up
Tnfaip3	1805.47	0.77402	0.11714	6.60775	3.90E-11	4.73E-09	1261.23	1497.4	1238.75	2252.55	2324.35	2258.55	Up
Gm33056	150.395	-1.2468	0.44215	-2.8199	0.0048	0.03622	237.949	106.534	290.439	87.6955	51.5149	128.236	Down
Olig3	262.568	-1.796	0.37714	-4.7623	1.91E-06	6.91E-05	532.692	204.191	486.339	134.602	81.3936	136.189	Down
Ifngr1	9341.79	0.32836	0.11529	2.84819	0.0044	0.03395	9116.47	7876.64	7856.47	11009.9	10704.8	9486.49	Up
Slc35d3	197.828	-2.3331	0.33165	-7.0349	1.99E-12	3.07E-10	397.56	189.394	403.496	62.2026	59.7573	74.5559	Down
Pex7	3611.16	-0.3382	0.13911	-2.4313	0.01504	0.08356	4221.39	3445.6	4430.66	3347.72	2874.53	3347.06	Down
Ahi1	832.935	-0.3945	0.13555	-2.9105	0.00361	0.0295	956.691	878.909	1002.89	670.972	678.967	809.18	Down
Myb	1597.48	-1.0816	0.26258	-4.1192	3.80E-05	0.00083	1920.24	1593.08	2996	1177.77	765.512	1132.26	Down
Sgk1	15018.7	0.68174	0.23325	2.92277	0.00347	0.02857	11175.8	13070.2	10358.3	20987.8	22585.2	11934.9	Up
Gm8540	2.6793	4.89474	2.07782	2.35571	0.01849	NA	0	0	0	1.01971	4.12119	10.9349	Up
Raet1e	3.0323	4.03124	1.69182	2.38278	0.01718	NA	0	0	0.97463	4.07886	6.18179	6.95855	Up
Slc2a12	1722.19	-0.7845	0.21928	-3.5775	0.00035	0.00486	2261.98	1633.53	2642.21	1339.91	993.208	1462.29	Down
Vnn3	1348.04	1.72118	0.59139	2.91041	0.00361	0.0295	776.516	370.898	734.869	2155.68	888.117	3162.16	Up
Vnn1	1611.27	2.01078	0.34294	5.86339	4.54E-09	3.60E-07	594.382	668.799	658.848	2666.55	1195.15	3883.86	Up
Moxd1	30.7729	-0.9494	0.45014	-2.1092	0.03493	0.15033	29.3764	53.2672	38.9851	24.4732	21.6363	16.8993	Down
Med23	1952.78	0.24475	0.11152	2.19463	0.02819	0.1294	1791.96	1927.48	1643.22	1989.46	2192.48	2172.06	Up

Epb41l2	8575.52	0.26936	0.09528	2.82708	0.0047	0.03578	7575.19	7800.69	7955.88	8773.63	10122.7	9225.05	Up
Gm4739	93.1154	-0.6143	0.27749	-2.2139	0.02683	0.12498	124.36	97.6565	115.981	59.1435	71.0906	90.4611	Down
9330159F	170.521	-1.4946	0.39229	-3.81	0.00014	0.00242	219.344	140.073	395.699	112.169	78.3027	77.5381	Down
Trdn	126.014	-2.001	0.66955	-2.9886	0.0028	0.02438	271.242	36.4979	297.261	36.7097	29.8787	84.4967	Down
Rsph4a	3043.96	-1.0532	0.30207	-3.4867	0.00049	0.00635	5292.64	2622.92	4409.21	1995.58	1298.18	2645.24	Down
Gm48018	53.325	-1.6056	0.38655	-4.1536	3.27E-05	0.00074	69.5241	60.1722	111.108	26.5126	26.7878	25.846	Down
Dse	1777.75	0.31469	0.12006	2.6212	0.00876	0.05616	1731.25	1412.57	1610.08	1892.59	1950.35	2069.67	Up
Frk	1530.93	0.84143	0.10809	7.78489	6.98E-15	1.76E-12	1126.09	1007.14	1156.88	1875.26	2012.17	2008.04	Up
Amd2	176.681	0.54534	0.27121	2.01075	0.04435	0.17627	132.194	127.249	171.534	163.154	275.09	190.863	Up
Fam229b	295.288	-1.0291	0.19092	-5.3904	7.03E-08	4.17E-06	422.041	332.427	434.684	177.43	185.454	219.691	Down
Gm16364	301.096	-0.8553	0.18951	-4.5133	6.38E-06	0.00019	464.147	370.898	328.449	208.022	216.363	218.697	Down
Rev3l	2450.86	-0.3327	0.14684	-2.2657	0.02347	0.11357	2706.54	2450.29	3039.86	2283.14	1852.48	2372.87	Down
4930547N	137.061	-1.305	0.2949	-4.425	9.64E-06	0.00027	221.302	135.141	229.037	73.4195	69.03	94.4375	Down
Gm31848	21.2458	-1.1592	0.57699	-2.009	0.04454	0.17658	26.4387	19.7286	41.909	9.17743	11.3333	18.8875	Down
Mfsd4b3-	161.426	-0.919	0.28353	-3.2413	0.00119	0.01264	230.115	149.937	253.403	91.7743	134.969	108.355	Down
Mfsd4b5	141.414	-1.5294	0.36872	-4.1479	3.36E-05	0.00075	196.822	140.073	293.363	70.3603	49.4543	98.4138	Down
Mfsd4b1	404.498	-1.5095	0.30335	-4.9762	6.49E-07	2.78E-05	752.035	353.142	691.011	226.377	164.848	239.573	Down
Amd1	4934.64	0.58643	0.10821	5.41914	5.99E-08	3.59E-06	3684.78	3768.16	4382.9	6119.31	5885.06	5767.64	Up
Cdk19	5876.19	0.92991	0.16539	5.6224	1.88E-08	1.29E-06	3577.06	4546.45	4012.54	9285.52	6673.24	7162.34	Up
Ddo	2930	-1.0773	0.25851	-4.1674	3.08E-05	0.0007	4726.66	2552.88	4648	2076.14	1431.08	2145.22	Down
Mettl24	587.913	-0.557	0.21825	-2.5523	0.0107	0.06509	765.744	493.215	841.103	477.227	448.18	502.01	Down
Cdc40	1943.21	0.30547	0.12161	2.51186	0.01201	0.0706	1818.4	1616.76	1779.67	1924.2	2182.17	2338.07	Up
Ak9	1828.94	-1.147	0.182	-6.3024	2.93E-10	2.90E-08	2677.17	2119.84	2763.07	1229.78	899.451	1284.35	Down
Mical1	1450.65	0.45642	0.16762	2.72289	0.00647	0.04504	1181.91	1384.95	1102.3	1583.62	1998.78	1452.35	Up
Ppil6	3437.19	-0.7022	0.30507	-2.3016	0.02136	0.10682	5172.2	2705.78	4894.58	2943.92	1608.3	3298.35	Down
Cd164	27365.7	0.31959	0.11233	2.84513	0.00444	0.03421	24686	25309.8	23045.1	29993.9	33667.1	27492.2	Up
Armc2	1028.57	-0.9055	0.31212	-2.901	0.00372	0.03015	1547.16	801.967	1674.41	787.22	474.968	885.724	Down
Tdg-ps2	61.0187	-2.446	0.66043	-3.7036	0.00021	0.00335	152.757	136.127	20.4672	20.3943	16.4848	19.8816	Down
Ostm1	3383.44	0.30646	0.08223	3.72702	0.00019	0.00312	2975.83	3030.31	3070.08	3631.2	3886.29	3706.92	Up
Cd24a	52229.9	-0.7075	0.27385	-2.5834	0.00978	0.06074	78281.2	42624.6	73451.8	43584.7	26762	48675.1	Down
Gm40634	23.3412	-1.3583	0.66129	-2.0541	0.03997	0.16496	27.418	23.6743	49.706	6.11829	9.27269	23.8579	Down
4933404K	16.7557	-1.8965	0.64244	-2.9521	0.00316	0.02666	22.5219	16.7693	39.9597	6.11829	7.21209	7.95263	Down
Rtn4ip1	827.56	-0.4198	0.19867	-2.1131	0.03459	0.14932	969.421	794.076	1077.94	798.437	548.119	777.369	Down
Prep	6908.54	0.20843	0.07595	2.74429	0.00606	0.04306	6385.45	6601.19	6244.44	7397.01	7534.57	7288.58	Up
Bves	36.4394	-1.7754	0.65453	-2.7125	0.00668	0.04603	81.2747	21.7015	66.2747	8.15772	13.3939	27.8342	Down
Gpx4-ps2	4630.12	-0.2103	0.107	-1.9652	0.04939	0.18935	4751.14	5360.26	4789.32	4523.46	4392.16	3964.39	Down
Gm48786	16.1911	1.41831	0.6383	2.22199	0.02628	0.12327	9.79213	8.87787	7.79702	13.2563	21.6363	35.7868	Up
Ros1	163.264	-1.2614	0.38421	-3.2832	0.00103	0.01121	115.547	298.888	276.794	74.4392	118.484	95.4315	Down
Dcbld1	833.906	-0.2866	0.1389	-2.0632	0.03909	0.16252	884.229	1043.64	821.611	736.234	728.421	789.298	Down
Nus1	7297.95	0.52179	0.10873	4.79905	1.59E-06	5.90E-05	6218.98	6127.7	5630.42	8272.95	9476.69	8060.98	Up
Man1a	6495.24	0.36528	0.17499	2.08751	0.03684	0.15625	5755.81	6741.26	4534.94	6394.63	8183.66	7361.15	Up
Tbc1d32	1096.45	-0.4437	0.18832	-2.3562	0.01846	0.09694	1324.87	1032.79	1433.68	949.355	762.421	1075.59	Down
Hsf2	1384.51	-0.403	0.12269	-3.2843	0.00102	0.01117	1548.14	1432.3	1749.46	1201.22	1133.33	1242.6	Down
Pkib	477.868	0.45747	0.19503	2.34566	0.01899	0.09875	380.914	468.554	358.663	595.513	619.209	444.353	Up
Gcc2	4318.65	0.27468	0.10469	2.62373	0.0087	0.05587	3580.98	4108.48	4036.91	4438.82	4857.86	4888.88	Up
Ccdc138	961.503	-0.8322	0.2434	-3.4191	0.00063	0.0077	1246.54	930.203	1517.49	715.84	509.998	848.943	Down
Sh3rf3	814.818	-0.5002	0.14485	-3.453	0.00055	0.00697	859.749	1095.92	908.353	629.164	707.815	687.902	Down
Oit3	43.959	-1.2754	0.54254	-2.3508	0.01874	0.09789	63.6488	98.643	24.3657	25.4929	25.7575	25.846	Down
Micu1	5837.91	-0.3286	0.11782	-2.789	0.00529	0.03895	6745.8	5692.69	7061.17	5227.06	4951.61	5349.14	Down
Dnajb12	3215.39	-0.3082	0.11497	-2.6805	0.00735	0.04926	3892.37	3150.66	3629.51	2875.6	2811.68	2932.53	Down
Ascc1	3588.09	-0.3932	0.1501	-2.6192	0.00881	0.05643	4012.81	3740.54	4468.67	3532.29	2591.2	3183.04	Down
Chst3	893.116	0.62628	0.30126	2.07889	0.03763	0.15832	590.465	871.017	645.203	1097.21	1516.6	638.198	Up
Psap	60844.7	0.63819	0.1336	4.77701	1.78E-06	6.50E-05	48605.2	48639.9	45561.9	71536.1	86361.7	64363.6	Up
Slc29a3	979.324	0.41121	0.11443	3.59352	0.00033	0.00466	797.079	898.637	826.484	1075.8	1089.03	1188.92	Up
Pcbd1	951.524	-0.544	0.1568	-3.4698	0.00052	0.00666	1091.82	1023.91	1270.91	773.964	671.755	876.777	Down
Sgpl1	4894.55	0.30054	0.1037	2.89818	0.00375	0.03033	4164.59	4752.62	4242.55	5084.3	5668.7	5454.51	Up
Tbata	317.776	-3.1045	0.41572	-7.4677	8.16E-14	1.66E-11	710.908	337.359	659.823	28.552	85.5148	84.4967	Down
Npffr1	6.56485	-2.1893	0.96056	-2.2792	0.02266	NA	7.8337	9.8643	14.6194	3.05914	1.0303	2.98224	Down
Gm5424	468.452	-0.3511	0.13573	-2.5866	0.00969	0.06036	499.399	501.106	575.03	396.669	410.059	428.448	Down
Fam241b	538.644	-0.2962	0.14563	-2.0339	0.04196	0.17007	584.59	623.424	573.081	488.443	538.846	423.477	Down
Vps26a	5211.46	0.28958	0.08374	3.45794	0.00054	0.00687	4520.05	4808.84	4741.56	5629.85	5976.76	5591.69	Up
Kifbp	3280.47	-0.2651	0.08654	-3.0634	0.00219	0.0202	3654.42	3446.59	3642.18	2982.67	2869.38	3087.61	Down
Stox1	478.923	-0.4579	0.14966	-3.0593	0.00222	0.02046	536.609	547.468	578.929	407.886	351.332	451.312	Down
Pbld2	808.809	1.50681	0.25083	6.00722	1.89E-09	1.59E-07	401.477	496.174	365.485	1072.74	1639.2	877.771	Up
Pbld1	87.239	1.21074	0.33679	3.59498	0.00032	0.00465	47.0022	63.1315	47.7567	124.405	157.636	83.5026	Up
Dnajc12	1282.53	-0.7442	0.13854	-5.3721	7.78E-08	4.54E-06	1552.05	1525.02	1741.66	1003.4	826.299	1046.76	Down
Gm32255	3.03629	5.07442	1.79239	2.83109	0.00464	NA	0	0	0	7.138	4.12119	6.95855	Up
Gm32515	1.86121	4.36781	2.0965	2.08338	0.03722	NA	0	0	0	2.03943	5.15149	3.97631	Up
Cabcoco1	540.335	-1.1891	0.34118	-3.4852	0.00049	0.00636	775.536	543.523	934.668	354.861	175.151	458.27	Down
Rhobtb1	1131.15	-0.644	0.20739	-3.1053	0.0019	0.01801	1451.19	1091.98	1595.46	780.082	771.694	1096.47	Down
Mrln	87.2244	-1.118	0.33889	-3.299	0.00097	0.01074	116.526	87.7922	153.991	63.2223	40.1816	61.6329	Down
Fam13c	318.845	0.57564	0.24531	2.34657	0.01895	0.09856	202.697	344.264	221.24	371.176	432.725	340.969	Up
Phyhipl	74.4518	-1.4511	0.30955	-4.6879	2.76E-06	9.44E-05	108.693	88.7787	129.625	45.8872	32.9696	40.7572	Down
Cisd1	2262.94	-0.3775	0.11465	-3.2928	0.00099	0.01094	2467.62	2523.29	2681.2	2115.91	1759.75	2029.91	Down
Ipmk	3091.82	0.36576	0.09886	3.69988	0.00022	0.00339	2539.1	2749.18	2817.65	3284.5	3652.41	3508.1	Up
Rsph14	726.361	-1.2779	0.29474	-4.3355	1.45E-05	0.00038	1235.77	660.908	1189.05	469.069	287.453	515.927	Down
Rab36	1709	-0.9051	0.22901	-3.9524	7.74E-05	0.00151	2409.84	1634.51	2640.27	1338.89	899.451	1331.07	Down
Ggt5	1056.97	0.7426	0.18553	4.00253	6.27E-05	0.00125	847.998	902.583	621.812	1193.07	1524.84	1251.54	Up
Derl3	138.26	0.59769	0.27458	2.17671	0.0295	0.13353	135.131	78.9144	115.981	193.746	158.666	147.124	Up
Chchd10	2794.25	-0.3282	0.14227	-2.3069	0.02106	0.106	3055.14	2871.5	3405.35	2818.49	2474.78	2140.25	Down
Gm867	3369.63	-0.8191	0.27569	-2.9711	0.00297	0.02549	4347.7	2928.71	5627.5	2806.26	1652.6	2854.99	Down
2610028H	3413.44	-0.5098	0.13335	-3.8231	0.00013	0.00233	4091.15	3862.86	4076.87	2826.65	3212.47	2410.64	Down
Ybey	327.127	-0.3225	0.15466	-2.085	0.03707	0.15681	370.142	334.4	385.952	300.816	307.029	264.425	Down

Lss	393.487	0.36048	0.13948	2.58435	0.00976	0.06062	333.912	333.413	366.46	416.044	438.907	472.187 Up
Col6a2	10803.4	0.45634	0.1183	3.85735	0.00011	0.00208	9283.92	9300.06	8742.41	13558.1	13069.3	10866.3 Up
Col6a1	19543.1	0.43526	0.10014	4.34636	1.38E-05	0.00036	16399.9	17394.7	16057	24383.4	22139.1	20884.6 Up
Col18a1	478.545	0.29452	0.14082	2.09141	0.03649	0.15518	460.23	408.382	421.039	478.246	535.755	567.619 Up
Pofut2	3998.39	-0.2263	0.08881	-2.5481	0.01083	0.06552	4561.17	4211.07	4161.66	3709.72	3773.98	3572.72 Down
Itgb2	8778.3	1.51843	0.2308	6.57896	4.74E-11	5.64E-09	4495.57	5563.46	3569.09	11701.2	17116.3	10224.1 Up
Tspear	10.1873	-2.1626	0.78208	-2.7652	0.00569	0.04112	19.5843	13.81	16.5687	6.11829	2.0606	2.98224 Down
Cfap410	1360.75	-0.4832	0.18548	-2.6054	0.00918	0.05806	1799.79	1212.32	1747.51	1214.48	1001.45	1188.92 Down
Icosl	1225.72	0.55733	0.18931	2.94402	0.00324	0.02713	816.663	901.597	1257.27	1377.63	1613.45	1387.73 Up
Gatd3a	1610.45	-0.2296	0.09905	-2.3182	0.02044	0.10378	1790	1641.42	1783.57	1550.99	1442.42	1454.34 Down
Gm30122	233.468	-0.3993	0.16896	-2.3634	0.01811	0.09593	246.762	274.227	275.82	197.825	186.484	219.691 Down
Cstb	4065.46	0.48474	0.10058	4.81927	1.44E-06	5.48E-05	3509.5	3265.08	3391.7	4778.38	5072.16	4375.93 Up
Gm47015	118.231	1.5908	0.61297	2.59522	0.00945	0.05928	23.5011	105.548	47.7567	139.701	317.332	75.55 Up
Mier2	1081.85	-0.2562	0.11985	-2.138	0.03252	0.14303	1172.12	1169.91	1190.99	1096.19	903.572	958.292 Down
Cfd	533.736	-5.776	1.57963	-3.6565	0.00026	0.00387	2876.93	85.8194	182.255	20.3943	35.0301	1.98816 Down
Abca7	3902.79	0.37824	0.13111	2.88496	0.00391	0.03122	3012.06	3814.52	3355.64	4477.57	4724.95	4031.98 Up
Gpx4	17145.9	-0.2521	0.10091	-2.4983	0.01248	0.07258	17580.8	20188.3	18151.5	16417.4	15762.5	14775 Down
Sbno2	6286.94	0.39695	0.11613	3.41804	0.00063	0.00772	5490.45	5852.49	4939.41	6675.05	7781.84	6982.41 Up
Cbarp	1091.56	-0.911	0.15955	-5.7098	1.13E-08	8.23E-07	1491.34	1192.59	1591.57	676.071	828.36	769.417 Down
Cirbp	3304.74	-0.2699	0.11715	-2.3038	0.02123	0.10646	3280.36	3678.4	3879.99	2943.92	3246.47	2799.33 Down
Fam174c	449.462	-0.338	0.14387	-2.3497	0.01879	0.09806	534.65	510.971	460.024	433.379	375.029	382.72 Down
Ndufs7	2967.35	-0.3099	0.11293	-2.7442	0.00607	0.04306	3471.31	3195.05	3188.01	2857.24	2709.68	2382.81 Down
Pcsk4	407.973	-0.6953	0.2548	-2.7289	0.00635	0.0445	489.606	348.21	675.417	344.664	295.696	294.247 Down
Reep6	2256.69	-0.7079	0.24987	-2.833	0.00461	0.03526	2779.01	2352.63	3266.95	1918.08	1093.15	2130.31 Down
Plk5	419.062	-1.1447	0.34079	-3.359	0.00078	0.00903	714.825	328.481	688.087	301.836	169.999	311.147 Down
Mob3a	2372.43	0.28977	0.09128	3.17465	0.0015	0.015	2142.52	2224.4	2037.95	2639.02	2674.65	2516.01 Up
Izumo4	1240.53	-0.2714	0.11489	-2.3621	0.01817	0.09611	1231.85	1444.13	1394.69	1105.37	1092.12	1175 Down
Lsm7	708.039	-0.3228	0.12346	-2.6144	0.00894	0.05698	852.894	734.89	772.879	609.79	661.452	616.329 Down
Sppl2b	2072.1	-0.3894	0.13124	-2.9674	0.003	0.02572	2459.78	2029.09	2561.32	1909.93	1701.02	1771.45 Down
Tmprss9	34.5732	-1.4815	0.45805	-3.2343	0.00122	0.01289	62.6696	35.5115	54.5791	22.4337	12.3636	19.8816 Down
Thop1	1280.53	-0.3749	0.14286	-2.6245	0.00868	0.05576	1465.88	1295.18	1576.95	1238.95	982.905	1123.31 Down
Zfr2	420.038	-0.6996	0.15673	-4.4638	8.05E-06	0.00024	542.484	484.337	533.121	320.19	280.241	359.856 Down
Tjp3	5295.13	0.66431	0.13222	5.02414	5.06E-07	2.25E-05	4737.43	3629.07	3924.82	6866.76	6215.79	6396.9 Up
Pip5k1c	4810.37	0.2682	0.1057	2.53737	0.01117	0.06704	4323.22	4694.42	4075.89	5117.95	5632.64	5018.11 Up
Mfsd12	511.541	0.80483	0.16004	5.02882	4.94E-07	2.20E-05	401.477	357.088	358.663	657.716	735.633	558.672 Up
Celf5	41.4988	1.282	0.37662	3.404	0.00066	0.00801	20.5635	29.5929	22.4164	47.9266	63.8785	64.6151 Up
Gna15	426.526	0.83744	0.24729	3.38643	0.00071	0.00836	327.057	375.83	215.393	569.001	629.512	442.365 Up
Gm47561	1.96171	-4.3938	2.07676	-2.1157	0.03437	NA	5.87528	3.94572	1.94925	0	0	0 Down
1700028I1	4.74168	-3.6999	1.50892	-2.452	0.01421	NA	7.8337	4.93215	13.6448	2.03943	0	0 Down
Slc41a2	1549.21	0.56352	0.13395	4.2069	2.59E-05	0.00061	1281.79	1367.19	1102.3	1796.74	2023.51	1723.73 Up
Aldh1l2	144.547	-0.8146	0.25426	-3.2039	0.00136	0.01391	210.531	189.394	153.016	127.464	84.4845	102.39 Down
Nuak1	1047.95	0.54605	0.18532	2.94656	0.00321	0.02697	650.197	999.253	906.403	1293	1310.54	1128.28 Up
Gm47962	45.3647	-0.9709	0.39556	-2.4546	0.0141	0.07973	58.7528	59.1858	62.3761	24.4732	22.6666	44.7335 Down
Cry1	1507.61	-0.5159	0.21857	-2.3603	0.01826	0.09622	1643.12	1527.98	2151.98	1151.26	978.784	1592.51 Down
Btbd11	577.834	-0.4717	0.20739	-2.2744	0.02294	0.11172	666.844	544.509	803.093	531.271	389.453	531.832 Down
Bpifc	5.91515	-2.272	0.96138	-2.3633	0.01811	NA	9.79213	9.8643	9.74627	2.03943	2.0606	1.98816 Down
Hsp90b1	11283.2	0.34081	0.15599	2.18491	0.0289	0.13179	11770.1	9204.37	8895.42	11341.3	12150.3	14337.6 Up
Nt5dc3	1532.26	-0.5619	0.14261	-3.94	8.15E-05	0.00157	1987.8	1537.84	1955.1	1153.3	1265.21	1294.29 Down
Gnptab	2563.69	0.84376	0.17118	4.92919	8.26E-07	3.39E-05	2157.21	1790.37	1556.48	2812.37	3302.11	3763.58 Up
Spic	68.6728	1.1287	0.39015	2.89299	0.00382	0.03073	34.2724	44.3893	50.6806	86.6758	61.8179	134.201 Up
Slc5a8	122.031	-3.2151	1.50106	-2.1419	0.0322	0.14205	449.459	5.91858	205.646	17.3352	4.12119	49.7039 Down
Gas2l3	200.632	1.0339	0.21893	4.72257	2.33E-06	8.28E-05	157.653	120.344	116.955	273.284	303.938	231.62 Up
Apaf1	1795.47	0.50582	0.11976	4.22357	2.40E-05	0.00057	1369.92	1539.82	1541.86	2058.8	2314.05	1948.39 Up
Gm4800	16.95	-1.676	0.68777	-2.4368	0.01482	0.08274	13.709	36.4979	27.2896	5.09857	6.18179	12.923 Down
Cfap54	2154.52	-0.8926	0.30559	-2.9211	0.00349	0.02872	3157.96	1824.89	3418.99	1528.55	949.935	2046.81 Down
Gm3571	768.247	0.38966	0.14266	2.73148	0.00631	0.04429	660.969	743.768	590.624	837.186	947.875	829.061 Up
Fgd6	2176.63	0.40008	0.11069	3.61428	0.0003	0.00439	1841.9	2045.86	1742.63	2451.39	2365.57	2612.44 Up
Ndufa12	1795.43	-0.3003	0.10515	-2.8557	0.00429	0.0334	2136.64	1895.92	1912.22	1689.67	1579.45	1558.72 Down
Tmcc3	411.525	-0.4265	0.15403	-2.7689	0.00563	0.04074	522.9	417.26	475.618	356.9	328.665	367.809 Down
Cep83	4521	-0.4978	0.22244	-2.2381	0.02522	0.11948	5907.59	4013.78	5958.87	3917.74	2790.05	4537.97 Down
Plxnc1	1657.35	0.37105	0.14883	2.49303	0.01267	0.07344	1312.15	1688.77	1335.24	1789.6	2066.78	1751.57 Up
Cradd	818.151	-0.4383	0.17288	-2.5352	0.01124	0.06739	1005.65	740.809	1077.94	727.057	665.573	691.879 Down
Gm48882	22.7247	-1.455	0.52874	-2.7518	0.00593	0.04236	33.2932	30.5793	36.0612	16.3154	6.18179	13.9171 Down
5730420D	73.956	-0.9856	0.32739	-3.0104	0.00261	0.0231	88.1291	91.738	115.006	61.1829	33.9998	53.6802 Down
4732465J(	91.1633	-0.7384	0.25218	-2.928	0.00341	0.02826	98.9005	113.439	129.625	72.3998	67.9997	64.6151 Down
Gm47719	15.3264	-1.3991	0.68062	-2.0556	0.03982	0.16461	23.5011	26.6336	16.5687	3.05914	9.27269	12.923 Down
Gm47725	64.1671	-1.3621	0.48997	-2.7801	0.00543	0.0397	90.0876	85.8194	101.361	35.69	13.3939	58.6506 Down
Btg1	9764.76	0.22668	0.10355	2.18908	0.02859	0.13078	8624.91	9359.24	9013.35	11183.2	10870.7	9537.19 Up
Dcn	5182.7	-0.7245	0.18393	-3.9389	8.18E-05	0.00157	7330.39	5531.9	6509.54	4645.82	4006.83	3071.7 Down
Lum	557.753	-0.5533	0.22964	-2.4095	0.01598	0.08743	797.079	497.161	695.884	480.286	518.24	357.868 Down
Atp2b1	8032.65	0.30883	0.10801	2.85932	0.00425	0.03318	6756.57	7053.96	7718.07	8518.7	8475.24	9673.38 Up
B530045E	127.151	-0.6819	0.32368	-2.1068	0.03514	0.15104	170.383	189.394	110.133	83.6166	130.848	78.5322 Down
Nts	12.833	-1.9069	0.95521	-1.9963	0.0459	0.18039	43.0854	13.81	3.89851	6.11829	4.12119	5.96447 Down
Alx1	28.6377	-1.517	0.48063	-3.1563	0.0016	0.01577	47.9814	38.4708	40.9343	16.3154	8.24239	19.8816 Down
Gm15663	342.461	0.6004	0.27969	2.14666	0.03182	0.14062	240.886	387.667	188.103	387.492	471.877	378.744 Up
Gm36283	284.384	-0.4871	0.16635	-2.9283	0.00341	0.02825	306.494	358.074	331.373	219.239	227.696	263.431 Down
Csrp2	2582.13	-1.034	0.19956	-5.1815	2.20E-07	1.10E-05	3641.69	2598.26	4169.46	1887.49	1439.33	1756.54 Down
Osbpl8	3462.2	0.27485	0.09059	3.03421	0.00241	0.02178	3055.14	3195.05	3150	3566.96	3974.89	3831.18 Up
Gm7269	182.712	-0.6058	0.30898	-1.9607	0.04992	0.19068	234.032	135.141	292.388	150.918	126.727	157.064 Down
Kcnc2	158.553	2.40946	0.55519	4.33985	1.43E-05	0.00037	93.0252	12.8236	44.8329	349.762	185.454	265.419 Up
Tbc1d15	3959.89	0.3154	0.08896	3.54562	0.00039	0.00534	3405.7	3710.95	3469.67	4211.42	4496.22	4465.4 Up
Kcnmb4	90.2312	-1.0752	0.31382	-3.4261	0.00061	0.00755	106.734	98.643	161.788	61.1829	48.424	64.6151 Down
4933412E	67.4327	0.8111	0.37577	2.15853	0.03089	0.13769	60.7112	36.4979	49.706	67.3012	71.0906	119.289 Up

Lrrc10	16.7935	-3.9817	1.42524	-2.7937	0.00521	0.03858	49.9399	1.97286	42.8836	0	0	5.96447 Down
Lyz2	698680	0.72745	0.15027	4.84109	1.29E-06	4.99E-05	547723	584014	446780	837233	996183	780150 Up
Gm48903	31.8494	-3.8861	0.74984	-5.1826	2.19E-07	1.09E-05	33.2932	44.3893	101.361	2.03943	2.0606	7.95263 Down
Mdm2	5914.18	-0.2654	0.12048	-2.2032	0.02758	0.12737	6663.54	5830.79	6876	5359.62	4898.04	5857.11 Down
Slc35e3	2486.25	-0.5814	0.20382	-2.8527	0.00434	0.0337	3200.07	2217.49	3524.25	2089.4	1652.6	2233.69 Down
Gm40773	65.3476	-0.7842	0.28109	-2.7899	0.00527	0.03889	80.2954	92.7244	75.0463	48.9463	46.3634	48.7098 Down
Mdm1	1396.54	-0.9756	0.16776	-5.8157	6.04E-09	4.68E-07	1896.74	1650.3	2007.73	1011.56	755.209	1057.7 Down
Gm47461	12.2474	1.55863	0.65853	2.36681	0.01794	0.0953	8.81291	4.93215	4.87314	18.3549	20.606	15.9053 Up
Gm33677	39.1353	-1.0087	0.40178	-2.5105	0.01206	0.07084	53.8567	59.1858	43.8582	18.3549	25.7575	33.7987 Down
Gm47480	14.4258	-2.7559	0.71707	-3.8433	0.00012	0.00218	22.5219	19.7286	33.1373	2.03943	5.15149	3.97631 Down
Gm34045	65.791	-0.7981	0.35542	-2.2454	0.02474	0.11779	84.2123	57.2129	109.158	56.0843	45.3331	42.7454 Down
Helb	219.892	0.68608	0.16344	4.19781	2.70E-05	0.00063	174.3	165.72	165.687	255.948	283.332	274.366 Up
Irak3	614.315	0.46428	0.12836	3.61701	0.0003	0.00435	478.835	568.183	501.933	696.465	736.663	703.808 Up
Wif1	333.756	-0.9172	0.23685	-3.8724	0.00011	0.00198	363.288	367.938	577.954	211.081	258.605	223.668 Down
Gm48410	20.2468	-1.9894	0.61879	-3.215	0.0013	0.0135	36.2309	28.6065	32.1627	13.2563	8.24239	2.98224 Down
4930432C	25.5024	-1.4747	0.65244	-2.2603	0.0238	0.11477	59.732	14.7964	38.0105	8.15772	14.4242	17.8934 Down
Gns	21052.2	0.45015	0.08052	5.59026	2.27E-08	1.49E-06	18439.6	17567.3	17375.7	23395.3	25260.9	24274.4 Up
Gm50454	102.168	-0.6208	0.25034	-2.4798	0.01314	0.07561	143.944	114.426	113.057	91.7743	76.2421	73.5618 Down
Tbk1	4157.87	0.26713	0.09152	2.9187	0.00351	0.02889	3866.91	3603.43	3851.73	4281.78	4694.04	4649.31 Up
D930020E	158.502	-1.6102	0.34247	-4.7018	2.58E-06	8.90E-05	223.261	164.734	328.449	80.5575	54.6058	99.4079 Down
Gm35865	18.0291	-2.5003	0.72915	-3.429	0.00061	0.0075	28.3972	13.81	49.706	4.07886	7.21209	4.97039 Down
Gm48877	12.0363	-2.5967	1.30889	-1.9839	0.04727	0.18417	21.5427	37.4843	2.92388	0	9.27269	0.99408 Down
Slc16a7	2887.14	-0.6573	0.25882	-2.5396	0.0111	0.0667	3182.44	2680.13	4738.64	2152.62	1675.27	2893.76 Down
Marchf9	744.506	-0.4147	0.15455	-2.683	0.0073	0.04902	848.977	779.279	923.947	738.274	609.937	566.625 Down
Cdk4	5790.63	-0.2789	0.09527	-2.9272	0.00342	0.02831	6248.36	5985.65	6811.67	5428.96	5070.1	5199.03 Down
B4galnt1	5147.66	0.42998	0.1042	4.12639	3.69E-05	0.00081	4472.06	4039.43	4647.02	6276.35	5607.91	5843.19 Up
Slc26a10	471.158	0.49468	0.25097	1.97109	0.04871	0.18768	322.161	357.088	494.136	578.178	672.785	402.602 Up
Dtx3	5841.43	-0.3145	0.08575	-3.6674	0.00025	0.00375	6548.97	6278.62	6599.2	5488.11	5006.22	5127.46 Down
Pip4k2c	467.788	0.45507	0.16343	2.78457	0.00536	0.0393	431.833	382.735	369.384	556.764	462.604	603.406 Up
Arhgap9	1156.44	0.47917	0.23122	2.07236	0.03823	0.16001	922.418	1256.71	719.275	1263.43	1621.69	1155.12 Up
Lrp1	14754.2	0.45019	0.14559	3.09214	0.00199	0.01871	11413.7	13370.1	12628.2	16942.6	19774.5	14396.2 Up
Stat6	9805.06	0.21196	0.08538	2.48255	0.01304	0.07515	9405.34	9008.08	8844.74	10121.7	11093.2	10357.3 Up
Sdr9c7	30.6513	-1.6385	0.43647	-3.7539	0.00017	0.00287	40.1477	48.3351	50.6806	18.3549	15.4545	10.9349 Down
Ptges3	9037.37	-0.3471	0.12331	-2.8146	0.00488	0.0367	11119	9083.04	10155.6	8274.99	7195.6	8395.99 Down
Coq10a	2008.53	-0.2254	0.0835	-2.6992	0.00695	0.0473	2122.93	2216.51	2155.88	1838.55	1850.42	1866.88 Down
Ankrd52	548.945	0.3968	0.15401	2.57652	0.00998	0.06182	475.897	457.703	488.288	555.745	590.361	725.677 Up
Ikzf4	232.921	-0.8048	0.23997	-3.354	0.0008	0.00918	286.909	375.83	226.114	166.214	155.575	186.887 Down
Suox	1171.64	-0.3091	0.14227	-2.1725	0.02982	0.13447	1312.15	1465.83	1112.05	1108.43	1032.36	999.049 Down
Cdk2	1459.77	0.36812	0.10189	3.61297	0.0003	0.00441	1206.39	1324.77	1292.36	1677.43	1698.96	1558.72 Up
Dnajc14	3448.68	0.25188	0.08906	2.82816	0.00468	0.03569	3189.3	3157.56	3098.34	3518.02	3870.83	3858.02 Up
Cd63	17678.2	0.36905	0.12373	2.98264	0.00286	0.02477	14071.3	16713.1	15503.4	20305.6	21737.2	17738.3 Up
Itga7	6059.49	0.77757	0.16114	4.82555	1.40E-06	5.33E-05	4281.12	5129.43	3984.28	7430.66	8963.6	6567.88 Up
Ctdsp2-ps	128.774	0.61245	0.25267	2.42395	0.01535	0.08491	93.0252	84.8329	127.676	142.76	149.393	174.958 Up
Vmn2r87	16.2967	1.90586	0.58092	3.28079	0.00104	0.01128	5.87528	8.87787	5.84776	24.4732	28.8484	23.8579 Up
8430429K	289.138	-0.7167	0.18729	-3.8265	0.00013	0.0023	348.6	330.454	399.597	217.199	186.484	252.496 Down
Inpp5j	77.7241	-0.8855	0.35019	-2.5285	0.01146	0.06807	108.693	80.8872	113.057	57.104	35.0301	71.5737 Down
Osbp2	63.6171	-1.7764	0.49703	-3.574	0.00035	0.0049	135.131	39.4572	120.854	25.4929	31.9393	28.8283 Down
4921536K	139.998	-0.7494	0.33229	-2.2551	0.02413	0.11597	197.801	124.29	204.672	101.971	71.0906	140.165 Down
Dusp18	3834.83	-0.7273	0.17815	-4.0825	4.46E-05	0.00095	5284.81	3875.68	5184.04	2833.79	2442.84	3387.82 Down
Slc35e4	1705.35	-0.3006	0.11707	-2.5676	0.01024	0.06303	2002.49	1668.05	1976.54	1569.34	1488.78	1526.9 Down
Tcn2	13030.8	0.24838	0.10331	2.40423	0.01621	0.08846	12741.5	11204.9	11789.1	15035.7	14283	13130.8 Up
4930556J	22.176	-1.8865	0.58775	-3.2097	0.00133	0.0137	25.4595	29.5929	49.706	11.2169	5.15149	11.9289 Down
Ccdc157	3794.63	-0.4364	0.17671	-2.4696	0.01352	0.07723	4709.03	3588.63	4795.17	3326.31	2642.72	3705.92 Down
Osm	934.602	1.49752	0.60742	2.46537	0.01369	0.07797	335.87	745.741	384.978	1408.23	2151.26	581.536 Up
Lif	753.626	1.04239	0.2752	3.78778	0.00015	0.00259	379.935	608.627	489.263	948.335	1387.81	707.784 Up
Mtmr3	5661.29	0.21356	0.07405	2.88409	0.00393	0.03128	5238.79	5240.9	5249.34	5961.25	6237.43	6040.02 Up
Nipsnap1	1363.31	-0.3747	0.1403	-2.6708	0.00757	0.05031	1596.12	1530.94	1491.18	1325.63	999.389	1236.63 Down
Kremen1	3996.95	-0.3842	0.1654	-2.3232	0.02017	0.1028	5017.49	3646.83	4914.07	3354.86	3122.83	3925.62 Down
Dbnl	7241.87	0.52539	0.12718	4.13108	3.61E-05	0.0008	5958.51	6253.96	5600.21	8498.3	9651.84	7488.39 Up
Polm	1290.5	0.41759	0.11161	3.74138	0.00018	0.00298	1049.72	1076.19	1189.05	1507.14	1536.17	1384.75 Up
Gck	511.504	-1.1394	0.32438	-3.5126	0.00044	0.00587	850.936	386.68	873.266	333.447	237.999	386.697 Down
Myo1g	1991.75	0.65828	0.20567	3.2007	0.00137	0.01402	1199.54	2048.81	1386.89	2397.35	2607.69	2310.24 Up
Snhg15	427.774	0.37204	0.17501	2.12581	0.03352	0.14625	363.288	413.314	342.094	535.35	509.998	402.602 Up
Ramp3	320.417	-0.5094	0.25865	-1.9693	0.04892	0.18832	320.203	320.59	488.288	257.988	328.665	206.768 Down
Adcy1	94.3385	-0.9629	0.29842	-3.2266	0.00125	0.01314	122.402	97.6565	153.991	55.0646	77.2724	59.6447 Down
Igfbp3	2798.16	-0.8612	0.31363	-2.746	0.00603	0.04291	5400.36	3181.24	2246.52	2479.95	2053.38	1427.5 Down
Gm11992	849.509	-1.0383	0.32707	-3.1744	0.0015	0.015	1460.01	672.745	1295.28	608.77	355.453	704.802 Down
Abca13	43.3505	-1.0445	0.47557	-2.1963	0.02807	0.12909	62.6696	38.4708	74.0717	16.3154	27.8181	40.7572 Down
Ikzf1	1627.4	0.31164	0.15272	2.04062	0.04129	0.16828	1227.93	1663.12	1465.84	1707	1994.66	1705.84 Up
4930554G	70.6993	1.71814	0.52163	3.2938	0.00099	0.01091	19.5843	30.5793	48.7314	82.5969	60.7876	181.916 Up
Vstm2a	353.359	1.52758	0.39581	3.85939	0.00011	0.00206	106.734	303.82	135.473	396.669	720.179	457.276 Up
Akt2-ps	319.578	0.47182	0.17766	2.65573	0.00791	0.05202	279.076	243.648	280.693	374.235	317.332	422.483 Up
Gm12663	263.114	0.63381	0.17944	3.53209	0.00041	0.00556	199.759	226.879	192.002	335.486	347.211	277.348 Up
Egfr	1323.41	-0.5533	0.19332	-2.862	0.00421	0.03291	2017.18	1436.24	1268.96	1055.4	1004.54	1158.1 Down
Plek	5470.02	1.26372	0.18869	6.69718	2.12E-11	2.75E-09	3067.87	3765.2	2816.67	6588.38	9664.2	6917.79 Up
Ppp3r1	74.0663	0.68385	0.2768	2.47058	0.01349	0.0771	65.6073	50.3079	54.5791	80.5575	98.9087	94.4375 Up
Wdr92	1355.5	-0.4617	0.17291	-2.6702	0.00758	0.05038	1669.56	1250.79	1791.36	1183.89	1009.69	1227.69 Down
Actr2	15534.7	0.29011	0.07944	3.65211	0.00026	0.00393	13724.6	14153.3	14056.1	16408.2	17889.1	16976.9 Up
Slc1a4	3533.75	-0.7815	0.17013	-4.5936	4.36E-06	0.00014	4553.34	4051.27	4800.04	2682.87	2048.23	3066.73 Down
Sertad2	3394.78	0.2599	0.12475	2.0834	0.03721	0.15704	2757.46	3374.58	3137.33	3649.56	4019.19	3430.57 Up
Lgalsl	2431.89	-0.2369	0.11151	-2.1242	0.03365	0.14653	2709.48	2834.01	2349.83	2222.98	2196.6	2278.43 Down
Ugp2	5962.92	0.23109	0.11529	2.00438	0.04503	0.17808	5550.18	5515.13	5393.59	6533.31	7111.12	5674.2 Up
Mdh1	10996.4	-0.228	0.10495	-2.1727	0.0298	0.13446	12387	10727.4	12476.2	10607.1	9650.81	10129.7 Down

Wdpcp	1286.32	-0.6024	0.24439	-2.4649	0.0137	0.07804	1778.25	1126.5	1748.48	1038.07	755.209	1271.43	Down
Otx1	161.592	-2.0952	0.63699	-3.2892	0.001	0.01104	423.999	95.6837	266.073	48.9463	18.5454	116.307	Down
Tmem17	405.598	-0.8024	0.18391	-4.3631	1.28E-05	0.00034	567.943	424.165	554.563	333.447	276.12	277.348	Down
Fam161a	3299.15	-1.015	0.32341	-3.1384	0.0017	0.01655	5263.27	2750.17	5228.88	2590.08	1247.69	2714.83	Down
Pus10	1628.44	0.25643	0.10406	2.46416	0.01373	0.07817	1481.55	1386.92	1583.77	1698.85	1812.29	1807.23	Up
Cfap36	3888.73	-0.3239	0.13544	-2.3918	0.01677	0.09076	4535.71	3700.1	4734.74	3558.8	3154.77	3648.27	Down
Ccdc88a	1389.18	0.49639	0.11956	4.15192	3.30E-05	0.00074	1108.47	1151.16	1197.82	1527.53	1811.26	1538.83	Up
Gm12089	22.0286	1.09732	0.53814	2.03909	0.04144	0.16869	18.605	8.87787	14.6194	34.6703	20.606	34.7927	Up
Clhc1	23.423	-1.1545	0.48396	-2.3856	0.01705	0.09185	37.2101	27.62	32.1627	10.1971	15.4545	17.8934	Down
Acyp2	833.142	-0.321	0.12762	-2.5153	0.01189	0.07008	855.832	925.271	995.094	770.904	774.784	676.967	Down
Stc2	526.371	1.45419	0.2719	5.34834	8.88E-08	5.04E-06	367.205	212.082	265.099	995.242	747.997	570.601	Up
Bod1	2270.34	-0.2316	0.11164	-2.0747	0.03801	0.15939	2526.37	2429.58	2400.51	2281.1	2098.72	1885.77	Down
D630024D	740.196	-0.8001	0.1833	-4.365	1.27E-05	0.00034	1013.49	788.157	1019.46	599.592	439.937	580.542	Down
Nprl3	1528.1	-0.2204	0.09847	-2.2384	0.0252	0.11942	1580.45	1672	1681.23	1433.72	1467.14	1334.05	Down
Sh3pxd2b	2290.1	0.41349	0.14635	2.82541	0.00472	0.03585	2306.05	1776.56	1809.88	2444.26	2545.87	2857.98	Up
Ubtd2	234.13	0.49557	0.20746	2.38872	0.01691	0.09129	192.905	184.462	205.646	264.106	225.635	332.022	Up
Stk10	1455.2	0.57713	0.19073	3.02594	0.00248	0.02227	1046.78	1470.77	986.323	1663.15	1991.57	1572.63	Up
Fgf18	409.39	0.57117	0.26397	2.16376	0.03048	0.13663	275.159	453.758	259.251	500.68	572.846	394.649	Up
Dock2	1883.1	0.66265	0.20767	3.19091	0.00142	0.01433	1243.6	1883.09	1247.52	2123.05	2740.59	2060.72	Up
Ccng1	10854.2	0.26745	0.10882	2.45784	0.01398	0.07919	9125.28	10451.2	9976.28	11364.7	12944.7	11262.9	Up
Ccnjl	110.519	-1.9146	0.65908	-2.9049	0.00367	0.02988	179.196	316.644	28.2642	56.0843	40.1816	42.7454	Down
Il12b	73.3823	0.80019	0.31879	2.51004	0.01207	0.0709	47.0022	46.3622	67.2493	109.109	73.1512	97.4197	Up
Rnf145	7530.27	0.46344	0.11649	3.9785	6.94E-05	0.00137	6221.92	5676.9	7094.31	9020.4	8776.08	8392.01	Up
4930597A	8.12524	2.38252	1.02522	2.32392	0.02013	0.10268	0.97921	2.95929	3.89851	11.2169	24.7272	4.97039	Up
Clint1	8539.35	0.61116	0.09475	6.45018	1.12E-10	1.18E-08	7323.53	6436.45	6511.48	10187	10616.2	10161.5	Up
Gm12165	12.1776	2.37957	0.89785	2.65031	0.00804	0.0527	6.85449	3.94572	0.97463	19.3746	32.9696	8.94671	Up
Adam19	849.045	0.86166	0.17498	4.92443	8.46E-07	3.46E-05	642.364	681.623	484.39	965.67	1188.96	1131.26	Up
Cyfip2	3278.62	-0.848	0.25961	-3.2665	0.00109	0.01178	5004.76	2723.53	4917.97	2491.16	1761.81	2772.49	Down
Havcr2	806.015	1.14008	0.17483	6.52114	6.98E-11	7.98E-09	531.713	495.188	482.441	1001.36	1364.12	961.274	Up
Sgcd	770.951	1.76321	0.2569	6.86343	6.72E-12	9.08E-10	400.498	243.648	408.369	1180.83	1487.75	904.611	Up
Gm12184	263.363	-0.3382	0.164	-2.0624	0.03917	0.1627	268.304	325.522	288.49	241.672	223.575	232.614	Down
Gfpt2	647.512	-0.5681	0.21387	-2.6562	0.0079	0.05198	984.109	708.256	627.66	543.508	583.149	438.389	Down
Mgat4b	6139.56	0.32893	0.11854	2.77482	0.00552	0.04016	5308.31	5328.69	5690.85	6980.97	7525.3	6003.24	Up
Ltc4s	149.389	1.2007	0.37185	3.22902	0.00124	0.01308	112.609	101.602	57.503	198.844	291.574	134.201	Up
Adamts2	1450.18	0.42816	0.17706	2.41819	0.0156	0.08598	1319.98	1202.46	1187.1	1804.9	1913.26	1273.41	Up
Zfp354b	241.684	-0.4966	0.20061	-2.4755	0.0133	0.07618	291.805	280.146	276.794	212.101	157.636	231.62	Down
D930048N	715.355	0.45209	0.1538	2.93947	0.00329	0.02746	629.634	533.658	649.102	814.752	930.359	734.624	Up
Gm39822	362.174	0.66373	0.1957	3.39153	0.0007	0.00829	282.992	270.282	287.515	461.931	523.392	346.933	Up
Cdkl3	641.114	-0.7331	0.21922	-3.3442	0.00083	0.00943	761.828	628.356	1011.66	472.128	410.059	562.648	Down
Fstl4	366.728	-0.8375	0.40892	-2.0481	0.04055	0.16615	676.636	277.187	457.1	298.776	129.818	360.851	Down
Sowaha	125.292	-0.8545	0.2369	-3.6069	0.00031	0.00448	136.111	163.747	184.205	97.8926	79.333	90.4611	Down
Septin8	6355.14	0.20601	0.09254	2.22615	0.026	0.1222	5568.78	6329.92	5807.8	6650.58	6979.24	6794.53	Up
A430108G	231.368	-0.9589	0.26606	-3.604	0.00031	0.00452	292.785	251.54	372.308	203.943	119.515	148.118	Down
Kif3a	1501.09	-0.3238	0.15536	-2.084	0.03716	0.15698	1713.62	1525.02	1767.97	1376.62	1101.39	1521.93	Down
Il4	713.325	-0.4699	0.1709	-2.7496	0.00597	0.04253	904.793	724.039	856.697	658.736	493.513	642.175	Down
Csf2	81.0602	1.16541	0.30161	3.86401	0.00011	0.00203	52.8775	48.3351	48.7314	140.721	109.212	86.4848	Up
Fnip1	3246.07	0.26241	0.08212	3.19555	0.0014	0.0142	2871.05	2990.85	2993.08	3438.48	3640.04	3542.9	Up
Lyrm7	234.85	-0.7208	0.27304	-2.64	0.00829	0.05392	361.33	259.431	256.327	132.563	164.848	234.603	Down
Tnip1	2714.62	0.43076	0.13148	3.27637	0.00105	0.01145	2137.62	2544	2255.29	3315.09	3299.02	2736.7	Up
Gm2a	6900.37	0.41857	0.07749	5.40168	6.60E-08	3.93E-06	5834.15	6051.75	5833.14	8161.8	7715.91	7805.5	Up
Atp5pb-p	330.223	0.29728	0.14601	2.0361	0.04174	0.16953	304.535	290.01	294.337	386.472	372.968	333.016	Up
Fat2	254.344	-0.6148	0.29253	-2.1017	0.03558	0.15239	330.974	308.752	283.617	186.608	129.818	286.295	Down
Gria1	441.933	-0.7391	0.16264	-4.5446	5.50E-06	0.00017	524.858	543.523	589.65	307.954	387.392	298.224	Down
Galnt10	761.138	0.47586	0.17555	2.71066	0.00671	0.04613	676.636	704.311	529.223	981.985	923.147	751.523	Up
Gm12248	28.0914	2.3007	0.77515	2.96808	0.003	0.02568	9.79213	9.8643	8.77165	71.38	60.7876	7.95263	Up
Gm12247	18.8805	1.57749	0.75335	2.09396	0.03626	0.15453	11.7506	7.89144	8.77165	37.7295	40.1816	6.95855	Up
Mrpl22	843.265	-0.2719	0.10556	-2.5757	0.01	0.06193	961.587	882.855	922.972	777.023	768.603	746.553	Down
Lypd8	117.629	1.82214	0.41896	4.34918	1.37E-05	0.00036	50.9191	31.5657	73.097	230.456	103.03	216.709	Up
2210407C	603.06	5.32564	0.88374	6.02626	1.68E-09	1.44E-07	42.1061	8.87787	37.0358	1215.5	1968.9	345.939	Up
Olfr323	10.0619	4.8968	1.48106	3.30628	0.00095	0.01052	1.95843	0	0	16.3154	38.121	3.97631	Up
Olfr317	6.23556	-2.6749	1.04609	-2.5571	0.01056	NA	5.87528	13.81	12.6702	2.03943	1.0303	1.98816	Down
Fam183b	2343.88	-0.7765	0.2698	-2.878	0.004	0.03169	3532.02	1848.57	3498.91	2156.7	1359.99	1667.07	Down
Gm12258	298.737	-0.3415	0.16945	-2.0154	0.04386	0.17506	346.641	340.318	314.805	229.436	298.787	262.437	Down
2810021J1	350.477	0.4362	0.15407	2.83115	0.00464	0.03543	279.076	286.065	328.449	418.083	421.392	369.797	Up
Obscn	258.913	-1.5063	0.5085	-2.9622	0.00305	0.02609	505.274	126.263	517.527	94.8335	96.8481	212.733	Down
Snap47	2405.16	-0.2491	0.09496	-2.6229	0.00872	0.05599	2565.54	2716.63	2554.5	2239.29	2288.29	2066.69	Down
Gm12263	39.5551	0.83485	0.37786	2.20942	0.02715	0.12572	20.5635	31.5657	33.1373	54.0449	45.3331	52.6862	Up
Nlrp3	352.283	0.89023	0.25583	3.47976	0.0005	0.00645	229.136	318.617	192.976	433.379	570.785	368.803	Up
Flcn	4314.93	0.21892	0.08064	2.71499	0.00663	0.04576	4116.61	4009.84	3838.08	4727.4	4594.1	4603.58	Up
Nt5m	941.524	-0.2488	0.10707	-2.324	0.02012	0.10268	1015.44	1021.94	1030.18	917.743	812.905	850.931	Down
Pemt	256.271	-0.6353	0.20531	-3.0944	0.00197	0.0186	279.076	280.146	376.206	176.411	207.09	218.697	Down
Drc3	4453.66	-1.0804	0.29371	-3.6783	0.00023	0.00364	6838.82	4068.04	7235.63	3136.64	1760.78	3682.07	Down
Smcr8	2055.12	0.23999	0.09687	2.47742	0.01323	0.07593	1938.84	1935.37	1779.67	2175.05	2166.72	2335.09	Up
Shmt1	1229.37	0.25463	0.11443	2.22528	0.02606	0.12241	1094.76	1042.66	1226.08	1294.02	1327.02	1391.71	Up
Dhrs7b	1756.6	0.35836	0.13461	2.66226	0.00776	0.05124	1437.48	1701.59	1479.48	1992.52	2174.96	1753.55	Up
Aldh3a1	1896.6	-1.5623	0.6636	-2.3542	0.01856	0.09733	3557.48	1649.31	3294.24	1017.68	337.938	1522.93	Down
Slc47a2	93.869	-1.7395	0.60505	-2.8749	0.00404	0.03191	227.177	55.2401	151.067	30.5914	20.606	78.5322	Down
Slc47a1	366.16	-1.0752	0.39792	-2.702	0.00689	0.047	653.135	207.15	629.609	223.318	187.514	296.235	Down
Gm45605	92.9069	-0.688	0.33826	-2.0341	0.04194	0.17006	128.277	110.48	105.26	43.8477	73.1512	96.4256	Down
Mfap4	20775.6	0.37493	0.16746	2.23897	0.02516	0.11929	15151.4	22443.2	16678.8	25221.6	23464	21694.8	Up
B9d1	1457.62	-0.5046	0.19176	-2.6313	0.00851	0.05504	1962.34	1299.13	1868.36	1379.67	1090.06	1146.17	Down
Specc1	2790.63	0.39511	0.1715	2.30389	0.02123	0.10646	2038.72	2938.57	2255.29	2930.66	3583.38	2997.15	Up
Cenpv	953.404	-0.5559	0.14551	-3.8202	0.00013	0.00235	1169.18	1019.97	1215.36	875.935	694.421	745.559	Down

Trpv2	1401.35	0.89474	0.17515	5.10833	3.25E-07	1.55E-05	992.922	1087.05	860.596	1784.5	2175.99	1507.02	Up
Mmgt2	1204.34	0.24711	0.11925	2.07231	0.03824	0.16001	1102.59	1140.31	1061.37	1435.76	1296.12	1189.91	Up
Zfp287	532.279	0.35677	0.17285	2.06403	0.03901	0.16237	409.311	559.306	431.76	612.849	635.694	544.755	Up
Tekt3	97.2017	-2.0683	0.39735	-5.2051	1.94E-07	9.94E-06	222.281	95.6837	153.016	34.6703	28.8484	48.7098	Down
2810001G	310.232	-0.5739	0.14654	-3.9161	9.00E-05	0.0017	384.831	363.993	364.511	266.146	248.302	233.608	Down
Gm12292	1.96018	-4.3928	2.01383	-2.1813	0.02916	NA	3.91685	3.94572	3.89851	0	0	0	Down
Gm12295	23.2556	-2.3245	0.719	-3.2329	0.00123	0.01294	40.1477	14.7964	61.4015	7.138	4.12119	11.9289	Down
Map2k4	3752.52	0.2042	0.07987	2.55674	0.01057	0.06452	3498.73	3479.14	3484.29	3877.98	4146.95	4028.01	Up
Dnah9	3344.71	-1.024	0.30802	-3.3244	0.00089	0.00997	5421.9	2773.84	5257.14	2161.8	1445.51	3008.08	Down
Shisa6	27.7442	-1.0265	0.44153	-2.3248	0.02008	0.10252	42.1061	27.62	41.909	18.3549	19.5757	16.8993	Down
Tmem220	836.662	-0.3661	0.14152	-2.5866	0.00969	0.06036	997.818	869.044	960.008	715.84	652.179	825.085	Down
Adprm	1749.79	-0.2424	0.11157	-2.1726	0.02981	0.13447	1873.23	1738.09	2077.91	1644.8	1599.02	1565.67	Down
Sco1	825.471	0.34104	0.14992	2.27475	0.02292	0.11169	828.414	734.89	621.812	858.6	946.844	962.268	Up
Myh13	74.9507	0.9705	0.28861	3.36269	0.00077	0.00895	46.023	53.2672	52.6299	90.7546	120.545	86.4848	Up
Dhrs7c	51.3136	-2.7027	1.05455	-2.5629	0.01038	0.06364	158.632	9.8643	98.4374	6.11829	1.0303	33.7987	Down
Cfap52	404.024	-0.8087	0.39239	-2.0611	0.03929	0.16307	713.846	326.508	502.908	313.052	147.333	420.495	Down
Pik3r5	1583.69	1.07976	0.1815	5.94923	2.69E-09	2.20E-07	1013.49	1253.75	784.575	2078.18	2239.87	2132.3	Up
Pik3r6	817.117	0.51888	0.17505	2.96412	0.00304	0.02596	565.985	812.818	636.432	881.034	1057.09	949.345	Up
Mfsd6l	478.701	-0.647	0.26329	-2.4574	0.01399	0.07922	679.574	430.083	643.254	426.241	259.635	433.418	Down
Ccdc42	165.422	0.69365	0.2264	3.06381	0.00219	0.0202	125.339	127.249	126.702	181.509	178.242	253.49	Up
Tmem107	1750.24	-0.8318	0.22953	-3.6239	0.00029	0.00426	2618.41	1659.17	2446.31	1376.62	939.632	1461.3	Down
Rnf227	242.239	-0.6004	0.19784	-3.0349	0.00241	0.02174	276.138	260.417	339.17	218.219	187.514	171.976	Down
Chd3	8482.79	0.41214	0.0988	4.17137	3.03E-05	0.00069	7641.78	7560	6636.24	9407.89	9639.47	10011.4	Up
Dnah2	2167.95	-0.8583	0.27198	-3.1558	0.0016	0.01578	3057.1	1792.34	3534	1710.06	1118.9	1795.31	Down
Efnb3	150.069	-0.984	0.36039	-2.7302	0.00633	0.04437	234.032	126.263	237.809	114.208	62.8482	125.254	Down
Cd68	5939.4	1.92514	0.21239	9.06429	1.25E-19	6.69E-17	2489.16	2944.49	1994.09	8685.93	11989.6	7533.13	Up
Slc35g3	170.736	-1.903	0.40312	-4.7207	2.35E-06	8.34E-05	340.766	145.005	322.602	58.1237	52.5452	105.372	Down
Tmem102	990.991	0.32908	0.12336	2.66767	0.00764	0.05065	836.248	880.882	918.099	1161.46	1161.15	988.114	Up
Nlgn2	2014.15	0.2742	0.13899	1.97279	0.04852	0.18718	1689.14	1964.97	1815.73	2144.46	2523.2	1947.4	Up
Slc2a4	161.621	-1.4077	0.50495	-2.7877	0.00531	0.03904	368.184	69.0501	267.048	90.7546	77.2724	97.4197	Down
Asgr1	85.172	-1.6771	0.60072	-2.7918	0.00524	0.0387	182.134	51.2943	155.94	27.5323	19.5757	74.5559	Down
Mgl2	1193.64	0.60112	0.15205	3.95355	7.70E-05	0.0015	914.585	1086.06	845.002	1327.67	1366.18	1622.34	Up
Clec10a	188.156	-0.5629	0.22872	-2.4611	0.01385	0.07865	252.637	213.069	207.596	167.233	116.424	171.976	Down
Slc16a11	4259.25	-0.6566	0.27618	-2.3774	0.01744	0.09339	5197.66	3367.67	7070.92	4079.88	2464.47	3374.9	Down
Alox12	402.495	-0.8877	0.23275	-3.8138	0.00014	0.00239	424.978	438.961	703.681	274.303	295.696	277.348	Down
Alox15	348.19	-2.5223	1.11493	-2.2623	0.02368	0.11425	1372.86	94.6972	311.881	75.4589	46.3634	187.881	Down
Gm12312	11.8296	1.70677	0.82631	2.06553	0.03887	0.16206	3.91685	5.91858	6.82239	8.15772	12.3636	33.7987	Up
Arrb2	2161.18	0.40496	0.12211	3.31627	0.00091	0.0102	1796.86	2042.9	1739.71	2381.03	2697.32	2309.24	Up
Cxcl16	5633.36	0.47916	0.11388	4.20743	2.58E-05	0.00061	4518.09	5295.15	4305.9	6532.29	6731.97	6416.78	Up
Zmynd15	788.724	0.69274	0.24659	2.80927	0.00497	0.03716	631.592	727.985	449.303	914.684	756.239	1252.54	Up
Pld2	2793.76	0.46362	0.08732	5.3097	1.10E-07	6.04E-06	2360.88	2367.43	2317.66	3380.35	3251.62	3084.63	Up
Mink1	3362.8	0.30083	0.08836	3.40454	0.00066	0.00801	3150.13	2981.98	2908.29	3563.9	3806.95	3765.57	Up
Pfn1	21234.7	0.30266	0.12889	2.34827	0.01886	0.09825	18390.6	19706.9	18948.7	24508.8	26000.6	19852.7	Up
Eno3	1058.98	-0.5179	0.14715	-3.5195	0.00043	0.00574	1346.42	1052.52	1342.06	928.96	874.723	809.18	Down
Kif1c	10707.7	0.361	0.08959	4.02937	5.59E-05	0.00115	9070.45	9815.96	9238.49	12108.1	12626.3	11387.2	Up
Scimp	429.281	1.09944	0.15726	6.99101	2.73E-12	3.94E-10	275.159	291.983	252.428	527.193	657.33	571.595	Up
6330403K	219.541	-1.9285	0.31584	-6.106	1.02E-09	9.07E-08	296.701	235.757	510.705	87.6955	98.9087	87.4789	Down
Nlrp1b	385.796	0.96702	0.2447	3.95194	7.75E-05	0.00151	226.198	309.739	247.555	398.709	461.574	671.003	Up
Aipl1	21.221	-1.4382	0.59342	-2.4237	0.01536	0.08495	41.1269	22.6879	29.2388	10.1971	6.18179	17.8934	Down
Pitpnm3	2864.07	-0.5026	0.12944	-3.883	0.0001	0.00192	3533	3128.95	3412.17	2381.03	2076.05	2653.2	Down
Med31	1335.77	-0.5677	0.15502	-3.662	0.00025	0.0038	1696.98	1400.73	1688.05	1213.46	915.935	1099.45	Down
Tekt1	3318.56	-0.8302	0.23818	-3.4855	0.00049	0.00636	4681.62	3163.48	4898.48	2960.23	1713.39	2494.14	Down
Ggt6	524.204	-0.6025	0.30594	-1.9695	0.0489	0.18827	708.95	437.975	749.488	433.379	262.726	552.708	Down
Spns2	3868.72	0.40883	0.19926	2.05181	0.04019	0.16536	2853.43	4192.33	2926.81	3923.86	5326.64	3989.24	Up
Spns3	88.9694	0.99654	0.33305	2.99212	0.00277	0.02415	50.9191	50.3079	76.9956	145.819	85.5148	124.26	Up
Zzef1	3141.7	0.20324	0.10027	2.02684	0.04268	0.17194	2842.65	3069.77	2849.81	3203.94	3619.44	3264.55	Up
Atp2a3	13410.2	0.22391	0.09737	2.29954	0.02147	0.10712	12573.1	11448.5	13093.1	14085.3	15225.8	14035.4	Up
Itgae	617.745	0.58236	0.15587	3.73609	0.00019	0.00303	560.11	416.273	507.781	780.082	701.633	740.589	Up
P2rx5	722.856	0.44917	0.2127	2.11175	0.03471	0.14958	597.32	779.279	457.1	830.048	914.905	758.482	Up
Tax1bp3	4337.07	0.25257	0.08365	3.01922	0.00253	0.02265	4051	3971.37	3852.7	4793.68	4846.52	4507.15	Up
Ctns	1552.34	0.28819	0.10832	2.66043	0.0078	0.05144	1394.4	1470.77	1328.42	1613.19	1673.2	1834.07	Up
Trpv3	24.8731	-1.2341	0.61156	-2.018	0.04359	0.17438	21.5427	29.5929	53.6045	7.138	16.4848	20.8756	Down
Mnt	1836.03	0.28726	0.11672	2.46114	0.01385	0.07865	1556.95	1635.5	1768.95	1917.06	2215.14	1922.55	Up
Serpinf1	3080.28	0.53394	0.21543	2.47852	0.01319	0.07577	2453.91	1936.36	3159.74	4482.67	3262.96	3186.02	Up
Wdr81	2890.62	0.35277	0.12904	2.73382	0.00626	0.0441	2445.09	2304.3	2867.35	3393.61	3368.05	2965.34	Up
Tlcd2	292.309	0.69793	0.26579	2.62585	0.00864	0.05561	208.572	261.404	198.824	431.339	413.15	240.567	Up
Myo1c	19704.4	0.28944	0.10843	2.6693	0.0076	0.05043	16552.6	18938.5	17711.9	21595.5	23454.7	19973	Up
Doc2b	125.959	-1.8643	0.4038	-4.617	3.89E-06	0.00013	154.716	135.141	303.109	60.1632	35.0301	67.5973	Down
Gm12339	14.5212	-1.3678	0.66765	-2.0487	0.04049	0.16608	13.709	30.5793	18.5179	11.2169	5.15149	7.95263	Down
Rph3al	1742.27	-0.3123	0.12819	-2.4362	0.01484	0.08282	2035.78	1738.09	2016.5	1652.96	1390.9	1619.35	Down
1700016K	2523.63	-0.9432	0.27659	-3.4101	0.00065	0.00789	4038.27	2129.7	3793.25	1971.11	1203.39	2006.05	Down
Vps53	2683.11	0.21901	0.08962	2.44382	0.01453	0.08163	2496.01	2535.12	2408.3	3008.16	2904.41	2746.64	Up
Nxn	1473.3	-0.5534	0.11527	-4.8012	1.58E-06	5.88E-05	1800.77	1589.14	1867.39	1252.21	1193.09	1137.23	Down
Abr	6578.73	0.40059	0.09768	4.10115	4.11E-05	0.00089	5771.48	5589.11	5652.84	7364.38	8129.05	6965.51	Up
Ankrd13b	607.52	-0.3721	0.15572	-2.3896	0.01686	0.09111	706.012	597.776	752.412	585.316	525.452	478.152	Down
Trp53i13	929.173	-0.2676	0.1361	-1.966	0.0493	0.18918	993.901	1025.89	1025.31	887.152	921.087	721.701	Down
Myo18a	6591.25	0.27554	0.09781	2.81719	0.00484	0.03644	5544.3	5976.78	6370.16	7394.97	6878.27	7383.02	Up
Phf12	3392.27	0.2149	0.08017	2.68058	0.00735	0.04926	3099.21	3221.68	3099.31	3605.71	3742.04	3585.64	Up
Flot2	5997.57	0.24811	0.10544	2.35309	0.01862	0.09744	5238.79	5807.11	5403.33	6357.92	7091.54	6086.74	Up
Traf4	1651.58	-0.2576	0.12747	-2.021	0.04328	0.17352	1849.73	1580.26	1965.82	1586.68	1510.42	1416.56	Down
2610507B	9668.55	0.20103	0.0901	2.23123	0.02567	0.12104	9074.36	9220.16	8693.68	9793.34	10214.4	11015.4	Up
Aldoc	1706.95	1.2155	0.15482	7.85089	4.13E-15	1.15E-12	1093.78	869.044	1119.85	2292.32	2706.59	2160.13	Up
Unc119	2109.57	0.49935	0.19594	2.54852	0.01082	0.06552	1648.02	2101.1	1495.08	2525.83	2927.08	1960.32	Up

Vtn	592.723	-0.3778	0.14444	-2.6153	0.00891	0.0569	649.218	625.396	734.869	518.015	564.604	464.235 Down
Ift20	3395.23	-0.2769	0.12534	-2.2088	0.02719	0.12588	3832.64	4010.82	3316.66	3342.63	2816.84	3051.82 Down
Nos2	297.071	0.62326	0.30654	2.0332	0.04203	0.17024	248.72	300.861	152.042	349.762	463.634	267.407 Up
Rab11fip4	869.219	-1.1694	0.28613	-4.0868	4.37E-05	0.00094	1334.67	800.981	1474.61	586.336	361.635	657.086 Down
Adap2	334.736	0.39681	0.17926	2.21362	0.02685	0.12502	284.951	264.363	317.728	383.413	324.544	433.418 Up
Spaca3	14.1238	-1.6771	0.77374	-2.1675	0.03019	0.13571	27.418	10.8507	26.3149	8.15772	2.0606	9.94079 Down
Ccl12	231.577	0.6244	0.23261	2.68436	0.00727	0.04884	140.027	179.53	227.088	312.033	281.271	249.514 Up
Fndc8	13.6413	-2.2508	0.70117	-3.2101	0.00133	0.01369	13.709	26.6336	27.2896	6.11829	4.12119	3.97631 Down
Slfn9	394.459	0.81747	0.36223	2.25676	0.02402	0.11556	138.069	280.146	438.582	549.626	425.513	534.814 Up
Slfn2	171.964	0.70626	0.1859	3.79903	0.00015	0.00251	130.235	140.073	121.828	213.12	199.878	226.65 Up
Gm11427	143.885	0.77511	0.25813	3.0028	0.00268	0.02356	98.9005	97.6565	121.828	137.662	217.393	189.869 Up
Gas2l2	794.2	-0.9616	0.2759	-3.4855	0.00049	0.00636	1225	708.256	1215.36	591.435	375.029	650.127 Down
1700020L	195.169	-0.4226	0.19957	-2.1176	0.03421	0.14823	194.863	258.445	217.342	181.509	162.787	156.07 Down
Ccl5	457.462	-0.7495	0.25441	-2.9459	0.00322	0.027	438.687	796.049	486.339	297.757	375.029	350.91 Down
Ccl9	1516.93	0.75485	0.13432	5.61962	1.91E-08	1.30E-06	1193.66	1234.02	959.033	1907.89	1969.93	1837.06 Up
E230016K	1114.81	2.65646	0.2419	10.9818	4.67E-28	8.04E-25	408.332	290.01	217.342	1625.43	2274.9	1872.84 Up
Wfdc17	930.151	1.65552	0.18619	8.89139	6.03E-19	2.85E-16	439.667	460.663	444.43	1155.34	1305.39	1775.42 Up
Wfdc21	435.536	1.52834	0.24283	6.29381	3.10E-10	2.99E-08	272.221	237.73	162.763	590.415	795.39	554.696 Up
Heatr6	1460.93	0.20498	0.09024	2.27144	0.02312	0.11243	1349.36	1336.61	1385.92	1530.59	1609.33	1553.74 Up
Dusp14	3341.59	-0.6222	0.16626	-3.7423	0.00018	0.00298	4231.18	3227.6	4694.78	2909.25	2529.38	2457.36 Down
Dhrs11	781.168	-0.2326	0.11484	-2.0254	0.04282	0.17231	869.541	789.144	873.266	754.589	701.633	698.837 Down
Car4	19506.4	0.83266	0.17409	4.78287	1.73E-06	6.34E-05	13611.1	15931.8	12542.5	23807.3	30517.4	20628.1 Up
Usp32	3338.61	0.29074	0.1032	2.81731	0.00484	0.03644	2805.44	3195.05	3009.65	3455.81	3698.77	3866.97 Up
Trim37	2485.44	-0.4397	0.14872	-2.9563	0.00311	0.02641	3008.14	2433.52	3142.2	2040.45	1918.42	2369.88 Down
Ppm1e	417.515	-0.3281	0.14854	-2.2086	0.0272	0.12588	428.895	454.744	510.705	339.565	389.453	381.726 Down
Tspoap1	974.962	-1.247	0.29692	-4.1998	2.67E-05	0.00062	1431.61	905.542	1778.69	546.567	407.998	779.358 Down
Mks1	1008.59	-0.5863	0.25929	-2.2611	0.02375	0.11458	1287.66	875.949	1468.76	773.964	594.482	1050.74 Down
Cuedc1	2148.26	-0.2953	0.14487	-2.0381	0.04154	0.16897	2314.86	2008.37	2778.66	1966.01	1906.05	1915.59 Down
Mrps23	1948.51	-0.2465	0.08702	-2.8327	0.00462	0.03529	2073.97	2116.88	2152.95	1764.11	1739.14	1844.02 Down
Msi2	5773.27	-0.3794	0.16067	-2.3615	0.0182	0.09612	7200.15	5683.81	6700.56	4736.58	4354.04	5964.47 Down
Akap1	2309.08	0.27193	0.12033	2.25994	0.02383	0.11482	2046.55	2261.88	1967.77	2620.67	2690.11	2267.49 Up
Scpep1	727.178	0.47209	0.1494	3.16002	0.00158	0.01562	682.511	565.224	579.903	866.758	922.117	746.553 Up
Ankfn1	308.812	-1.3908	0.31115	-4.47	7.82E-06	0.00023	619.842	359.06	362.561	167.233	119.515	224.662 Down
Stxbp4	1022.97	-0.359	0.16836	-2.1325	0.03296	0.14438	1272.98	941.054	1234.85	909.586	790.239	989.108 Down
B230206L	185.392	-1.1153	0.23173	-4.8131	1.49E-06	5.63E-05	256.554	223.92	280.693	121.346	90.6663	139.171 Down
Mycbpap	2525.91	-0.7157	0.28646	-2.4983	0.01248	0.07258	3404.72	2084.33	3930.67	1963.97	1279.63	2492.15 Down
Lrrc59	7244.97	0.34233	0.08825	3.87895	0.0001	0.00194	6816.3	6147.43	6204.48	8236.24	8173.36	7891.99 Up
Sgca	29.5065	-1.8614	0.83309	-2.2343	0.02546	0.12034	71.4825	11.8372	55.5538	11.2169	3.0909	23.8579 Down
Ppp1r9b	6057.05	0.27839	0.13101	2.12498	0.03359	0.14635	5366.09	5946.2	5110.95	6442.56	7543.85	5932.66 Up
Nxph3	378.645	-1.2816	0.19713	-6.501	7.98E-11	8.82E-09	548.359	500.12	561.385	237.594	167.939	256.472 Down
B4galnt2	30.0728	1.01109	0.4513	2.2404	0.02507	0.11897	14.6882	28.6065	16.5687	38.7492	37.0907	44.7335 Up
Calcoco2	90.61	-1.4317	0.48039	-2.9802	0.00288	0.0249	173.321	99.6294	123.778	39.7689	22.6666	84.4967 Down
Ttll6	1598.89	-0.727	0.29532	-2.4619	0.01382	0.07854	1970.18	1358.31	2651.96	1347.04	773.754	1492.11 Down
Skap1	359.072	-0.5259	0.22515	-2.3356	0.01951	0.10044	369.163	553.387	348.917	266.146	296.726	320.093 Down
D030028A	29.5856	0.9421	0.40892	2.3039	0.02123	0.10646	19.5843	19.7286	21.4418	33.6506	45.3331	37.775 Up
Scrn2	702.494	-0.3101	0.13911	-2.229	0.02581	0.1215	814.705	725.026	793.347	706.662	571.816	603.406 Down
Lrrc46	924.345	-1.1916	0.27578	-4.3209	1.55E-05	0.0004	1457.07	875.949	1524.32	709.722	391.513	587.5 Down
Osbpl7	1861.7	0.42255	0.12545	3.36824	0.00076	0.00883	1429.65	1649.31	1693.9	2244.39	2224.41	1928.51 Up
Gm11592	61.1864	-0.9469	0.44302	-2.1374	0.03257	0.14315	94.0044	49.3215	98.4374	49.966	23.6969	51.6921 Down
Srcin1	1515.78	-0.3011	0.14984	-2.0093	0.04451	0.17654	1747.89	1422.43	1849.84	1375.6	1215.75	1483.17 Down
Gm11613	503.525	-0.5514	0.19659	-2.8049	0.00503	0.03756	560.11	571.143	664.696	373.216	341.029	510.956 Down
Cisd3	729.752	-0.3286	0.12091	-2.7181	0.00657	0.04557	874.437	803.94	759.235	660.775	615.088	665.039 Down
1700001P	17.3022	-1.2777	0.61649	-2.0725	0.03821	0.15999	28.3972	25.6472	19.4925	5.09857	9.27269	15.9053 Down
B230217C	86.2392	-0.5641	0.2749	-2.052	0.04017	0.16534	89.1084	106.534	113.057	79.5378	55.6361	73.5618 Down
Stac2	128.463	1.04157	0.30785	3.38335	0.00072	0.00843	114.568	83.8465	53.6045	182.529	176.181	160.047 Up
Fbxl20	2779.91	0.28561	0.1217	2.34676	0.01894	0.09854	2260.02	2742.27	2514.54	3235.56	3120.77	2806.28 Up
Ppp1r1b	94.1947	-1.0533	0.32101	-3.2813	0.00103	0.01127	168.425	121.331	91.615	70.3603	59.7573	53.6802 Down
Pgap3	919.15	-0.3652	0.13989	-2.6106	0.00904	0.05744	1009.57	947.959	1147.14	730.116	795.39	884.73 Down
Igfbp4	7353	-0.3646	0.08191	-4.4513	8.54E-06	0.00025	8331.14	8156.79	8343.78	6135.62	6418.76	6731.9 Down
Tns4	109.422	0.78735	0.3086	2.55137	0.01073	0.06512	80.2954	62.1451	98.4374	116.247	118.484	180.922 Up
A830036E	28.0619	3.08908	1.11848	2.76186	0.00575	0.04144	5.87528	11.8372	0	59.1435	86.5451	4.97039 Up
Krt20	66.9956	1.22545	0.43929	2.78964	0.00528	0.0389	49.9399	24.6607	45.8075	129.504	55.6361	96.4256 Up
Gm11560	409.774	0.42928	0.18417	2.33092	0.01976	0.10135	408.332	326.508	312.855	439.497	543.998	427.454 Up
Krtap17-1	121.244	-1.0054	0.4259	-2.3606	0.01824	0.09622	143.944	198.272	143.27	93.8138	114.363	33.7987 Down
Krt34	12.0551	3.78266	1.22699	3.08288	0.00205	0.01915	4.89606	0	0	26.5126	32.9696	7.95263 Up
Krt17	14.0527	-3.6982	1.62525	-2.2755	0.02288	0.11158	70.5033	0	7.79702	2.03943	0	3.97631 Down
Gm14206	37.2697	-1.2596	0.40793	-3.0878	0.00202	0.0189	68.5449	43.4029	45.8075	19.3746	21.6363	24.852 Down
Eif1	30375.8	-0.2293	0.10787	-2.1258	0.03352	0.14625	35884.2	31961.3	30508.8	29389.2	28447.6	26063.7 Down
Hap1	1110.79	-0.9183	0.25783	-3.5615	0.00037	0.00508	1136.87	1434.27	1787.47	730.116	548.119	1027.88 Down
Ttc25	1378.38	-0.9078	0.25024	-3.6278	0.00029	0.0042	1972.13	1295.18	2127.61	1124.75	678.967	1071.62 Down
Kcnh4	56.4074	1.89997	0.42722	4.44725	8.70E-06	0.00025	26.4387	17.7557	27.2896	101.971	112.303	52.6862 Up
Ghdc	946.79	0.23679	0.12055	1.96423	0.0495	0.18967	838.206	945	824.535	1054.39	1064.3	954.315 Up
Stat5a	1056.84	0.55564	0.12158	4.57008	4.88E-06	0.00015	866.603	894.692	806.017	1271.58	1357.93	1144.18 Up
Atp6v0a1	11069	0.48392	0.17331	2.79227	0.00523	0.0387	8008	10975	8706.35	12320.2	15267	11137.7 Up
Naglu	2171.74	0.26622	0.11958	2.22626	0.026	0.12219	1788.04	1961.02	2166.6	2403.47	2498.47	2212.82 Up
Coasy	1950.82	-0.3018	0.10454	-2.8867	0.00389	0.03112	2108.25	2145.48	2208.51	1893.61	1725.75	1623.33 Down
Psmc3ip	372.011	-0.6028	0.2237	-2.6946	0.00705	0.04777	485.69	376.816	483.415	342.624	219.454	324.07 Down
Ramp2	11651.1	-0.2638	0.11292	-2.3362	0.01948	0.10035	12211.8	12370.8	13557.1	10871.2	11428.1	9467.6 Down
Wnk4	1295.56	0.50309	0.16136	3.11785	0.00182	0.01741	931.231	1133.41	1151.03	1649.9	1642.3	1265.46 Up
Becn1	277.874	0.3958	0.17514	2.25992	0.02383	0.11482	240.886	207.15	271.921	294.698	324.544	328.046 Up
Aoc3	178.701	-1.5925	0.58931	-2.7024	0.00688	0.04697	581.652	111.467	112.082	71.38	107.151	88.473 Down
Ifi35	3215.09	-0.5204	0.13191	-3.945	7.98E-05	0.00154	3714.15	3359.78	4292.26	2755.27	2427.38	2741.67 Down
Rnd2	575.604	-0.463	0.15327	-3.0212	0.00252	0.02252	637.468	629.342	734.869	504.759	419.331	527.856 Down

Tmem106	2507.76	0.59311	0.12056	4.91961	8.67E-07	3.54E-05	1922.19	2038.95	2036.97	2954.11	3383.5	2710.85	Up
Mpp3	275.357	-0.7945	0.29218	-2.7191	0.00655	0.04548	480.793	242.662	324.551	182.529	173.09	248.52	Down
Nags	68.5374	-1.3899	0.32879	-4.2272	2.37E-05	0.00057	99.8797	73.9822	123.778	34.6703	39.1513	39.7631	Down
Hrob	62.4434	-1.3338	0.36629	-3.6413	0.00027	0.00404	70.5033	72.9958	124.752	34.6703	32.9696	38.7691	Down
Asb16	6.73703	-2.2332	1.05552	-2.1157	0.03437	NA	2.93764	15.7829	14.6194	2.03943	2.0606	2.98224	Down
Rundc3a	397.558	0.64195	0.18838	3.40779	0.00065	0.00794	271.242	369.911	290.439	477.227	547.088	429.442	Up
Grn	14058.6	0.54162	0.13932	3.88769	0.0001	0.00188	10322.9	12806.8	11220.9	17085.3	18436.2	14479.7	Up
Itga2b	649.791	-1.0974	0.23454	-4.6789	2.88E-06	9.78E-05	641.384	818.737	1196.84	407.886	420.362	413.537	Down
2810433D	87.9278	-0.8276	0.30899	-2.6784	0.0074	0.04947	119.464	129.222	88.6911	57.104	81.3936	51.6921	Down
Ccdc103	548.656	-0.9741	0.31073	-3.1349	0.00172	0.01668	786.308	450.798	944.414	396.669	254.484	459.264	Down
Gfap	123.17	-0.7903	0.25174	-3.1392	0.00169	0.01651	157.653	138.1	172.509	110.129	73.1512	87.4789	Down
Nmt1	6626.58	0.22073	0.10628	2.0768	0.03782	0.15879	6089.72	6563.7	5708.39	7257.31	7537.66	6602.67	Up
Acbd4	1813.38	-0.2312	0.09633	-2.4002	0.01639	0.08919	1844.84	1972.86	2057.44	1624.41	1693.81	1686.95	Down
Fmnl1	2066.05	0.46027	0.1876	2.45338	0.01415	0.07995	1564.78	2119.84	1533.09	2277.02	2849.81	2051.78	Up
Plekhm1	3415.81	0.39572	0.10642	3.7186	0.0002	0.0032	2857.34	3125.01	2868.33	3756.63	4228.34	3659.2	Up
Gm39397	2.78318	-3.845	1.71773	-2.2384	0.02519	NA	5.87528	3.94572	5.84776	0	1.0303	0	Down
Rprml	127.957	-0.6976	0.32957	-2.1166	0.0343	0.14845	145.903	206.164	122.803	74.4392	133.939	84.4967	Down
Nsf	3725.53	0.48401	0.13071	3.70283	0.00021	0.00336	3032.62	3432.78	2853.71	4115.57	4881.55	4036.95	Up
Arf2	3626.68	0.28676	0.10219	2.80623	0.00501	0.03745	3261.76	3484.07	3056.43	3950.38	4218.04	3789.43	Up
Crhr1	8.19588	-3.4702	1.30615	-2.6568	0.00789	0.05194	5.87528	19.7286	19.4925	4.07886	0	0	Down
Myl4	1634.26	-2.3428	1.05821	-2.214	0.02683	0.12498	4326.16	362.02	3502.81	447.655	194.726	972.209	Down
Marchf10	68.9766	-1.3732	0.40537	-3.3875	0.00071	0.00834	120.443	69.0501	109.158	24.4732	36.0604	54.6743	Down
Map3k3	3024.3	0.3425	0.10028	3.41525	0.00064	0.00777	2817.2	2752.14	2431.7	3402.79	3340.23	3401.74	Up
Limd2	7026.21	0.28457	0.12485	2.27932	0.02265	0.11069	6017.26	6696.87	6292.19	7985.39	8480.39	6685.18	Up
Ccdc47	2651.69	0.26845	0.11207	2.39532	0.01661	0.09007	2441.18	2476.92	2299.15	2701.22	2780.78	3210.87	Up
Prr29	1978.88	-0.9128	0.26922	-3.3904	0.0007	0.0083	2961.14	1803.19	2990.16	1695.79	917.996	1505.03	Down
Ern1	2654.12	0.21481	0.09013	2.3832	0.01716	0.09232	2557.7	2469.03	2343.98	2751.19	2923.99	2878.85	Up
Tex2	2414.17	0.21845	0.10993	1.98719	0.0469	0.18318	2261.98	2417.74	2015.53	2628.83	2649.93	2511.04	Up
Milr1	343.03	1.04129	0.25484	4.086	4.39E-05	0.00094	170.383	304.807	197.849	463.97	527.513	393.655	Up
Cep112	242.236	-0.4996	0.22204	-2.2503	0.02443	0.11684	310.41	252.526	288.49	197.825	157.636	246.531	Down
Gm11668	6.24261	2.42118	1.08544	2.23059	0.02571	NA	2.93764	2.95929	0	12.2366	12.3636	6.95855	Up
Slc16a6	889.862	0.50106	0.17592	2.84829	0.0044	0.03395	706.992	882.855	620.838	910.605	1105.51	1112.37	Up
Arsg	601.897	0.36815	0.15733	2.34002	0.01928	0.09967	449.459	605.668	521.426	647.519	723.27	664.044	Up
Abca9	609.13	0.6403	0.14185	4.51398	6.36E-06	0.00019	471.001	508.998	448.329	783.141	789.209	654.104	Up
Kcnj2	2202.74	-0.6663	0.23765	-2.8037	0.00505	0.03768	2614.5	3596.52	1896.62	1581.58	2014.23	1512.99	Down
Sox9	59.7715	-0.9288	0.34863	-2.6641	0.00772	0.05102	94.9836	68.0636	72.1224	30.5914	40.1816	52.6862	Down
Ttyh2	650.242	1.03922	0.17143	6.06187	1.35E-09	1.16E-07	415.186	421.205	440.532	818.831	1059.15	746.553	Up
Dnaic2	2806.67	-1.0621	0.29828	-3.5607	0.00037	0.00509	4566.07	2292.46	4528.12	1950.71	1230.18	2272.46	Down
Kif19a	2200.19	-0.6312	0.29199	-2.1617	0.03064	0.13706	3288.2	1628.6	3105.16	1977.23	1188.96	2013.01	Down
Gprc5c	1882.42	-0.5912	0.27192	-2.1742	0.02969	0.13412	2617.44	1437.23	2733.83	1618.29	1100.36	1787.35	Down
Cd300a	765.544	0.67449	0.25754	2.61892	0.00882	0.05645	461.209	830.574	477.567	867.777	1125.09	831.05	Up
Cd300lb	412.477	0.83063	0.35333	2.35088	0.01873	0.09789	213.468	481.378	195.9	472.128	667.633	444.353	Up
Cd300ld	378.899	0.92022	0.39526	2.32814	0.0199	0.10194	207.593	445.866	132.549	466.01	606.846	414.531	Up
Cd300c2	1424.67	1.06888	0.24559	4.3523	1.35E-05	0.00035	860.728	1230.08	668.594	1872.2	2340.84	1575.61	Up
Nat9	1720.48	-0.5194	0.15279	-3.3991	0.00068	0.0081	2216.94	1642.41	2221.18	1484.7	1358.96	1398.67	Down
Fdxr	502.517	-0.4185	0.14071	-2.9746	0.00293	0.02526	512.128	581.007	631.558	418.083	438.907	433.418	Down
Fads6	252.713	-0.9816	0.2319	-4.2331	2.30E-05	0.00055	422.041	299.875	284.591	196.805	164.848	148.118	Down
Jpt1	3612.82	0.37356	0.14067	2.65548	0.00792	0.05204	3255.88	3265.08	2921.93	4158.4	4629.13	3446.47	Up
Grb2	5755.91	0.20176	0.09278	2.17462	0.02966	0.13404	5413.09	5394.78	5254.22	6200.89	6548.58	5723.9	Up
Itgb4	1585.17	-0.4568	0.2251	-2.0293	0.04242	0.17118	2037.74	1275.45	2189.01	1401.09	1110.66	1497.08	Down
Galk1	2139.01	-0.5748	0.09617	-5.9767	2.28E-09	1.88E-07	2489.16	2485.8	2703.62	1798.78	1688.66	1668.06	Down
Mrpl38	2371.44	-0.2198	0.08713	-2.5224	0.01166	0.06907	2666.4	2504.54	2484.32	2199.53	2147.14	2226.74	Down
Foxj1	10480.6	-0.7637	0.31225	-2.4457	0.01446	0.08138	15024.1	7716.84	16833.8	9476.21	4958.83	8874.14	Down
St6galnac	702.872	0.59683	0.21649	2.75689	0.00584	0.04191	514.087	477.432	687.112	794.358	696.482	1047.76	Up
Jmjd6	2154.89	-0.2507	0.11653	-2.1509	0.03148	0.13952	2538.12	2251.03	2235.79	2067.98	1788.6	2047.8	Down
Septin9	5969.81	0.38305	0.11075	3.45874	0.00054	0.00686	5316.15	5027.83	5201.59	6550.65	7532.51	6190.13	Up
Gm11734	195.852	-1.5835	0.325	-4.8722	1.10E-06	4.32E-05	336.849	207.15	337.221	82.5969	72.1209	139.171	Down
Tmc8	423.529	-0.5148	0.25086	-2.0521	0.04016	0.16534	391.685	491.242	612.066	271.244	326.605	448.329	Down
Dnah17	65.2775	1.433	0.29195	4.90844	9.18E-07	3.71E-05	36.2309	37.4843	32.1627	98.9123	84.4845	102.39	Up
Cyth1	2736.67	0.20009	0.09272	2.15809	0.03092	0.13777	2676.19	2580.5	2384.91	2972.47	2928.11	2877.86	Up
Tbc1d16	2039.94	-0.4306	0.15571	-2.7653	0.00569	0.04112	2140.56	2537.1	2348.85	1502.04	1645.39	2065.7	Down
Ccdc40	3383.76	-1.0652	0.29498	-3.611	0.00031	0.00442	5366.09	3030.31	5340.96	2469.75	1352.78	2742.66	Down
Card14	82.656	0.7703	0.32606	2.36245	0.01815	0.09607	49.9399	72.0094	61.4015	78.518	97.8784	136.189	Up
Sgsh	571.284	0.42343	0.12232	3.46157	0.00054	0.00681	475.897	494.201	494.136	647.519	616.118	699.831	Up
Slc26a11	658.341	0.47736	0.15567	3.06649	0.00217	0.02005	535.629	617.505	498.035	804.555	826.299	668.021	Up
Nptx1	1435.49	-1.0463	0.25952	-4.0317	5.54E-05	0.00114	1917.3	2047.83	1838.15	701.564	725.33	1382.76	Down
Baiap2	6421.46	-0.4366	0.17978	-2.4283	0.01517	0.08406	8119.63	5855.45	8182	6118.29	4537.43	5715.95	Down
Cep131	772.162	-0.5629	0.22268	-2.5279	0.01147	0.06816	895	686.555	1181.25	652.618	531.634	685.914	Down
Gm11769	12.0008	-1.562	0.76886	-2.0316	0.0422	0.17067	18.605	8.87787	26.3149	8.15772	3.0909	6.95855	Down
2900052L	375.446	0.35144	0.16423	2.13997	0.03236	0.14246	313.348	322.562	353.79	437.458	464.665	360.851	Up
Bahcc1	1086.45	0.37628	0.16129	2.33293	0.01965	0.10092	853.874	921.325	1061.37	1153.3	1439.33	1089.51	Up
Actg1	48322.9	0.30596	0.08465	3.6144	0.0003	0.00439	44901.8	42468.8	42283.2	51853.5	56494.4	51935.6	Up
0610009L	168.434	-0.5109	0.2168	-2.3566	0.01844	0.09687	228.157	182.489	183.23	117.267	148.363	151.1	Down
Oxld1	211.968	-0.3709	0.18121	-2.0467	0.04069	0.16654	260.471	215.042	241.708	195.785	167.939	190.863	Down
Gcgr	87.966	-2.4015	0.36146	-6.6439	3.06E-11	3.88E-09	110.651	154.869	178.357	31.6112	17.5151	34.7927	Down
Mcrip1	3300.47	-0.2731	0.10566	-2.5849	0.00974	0.06054	3532.02	3645.84	3657.78	3198.85	3074.41	2693.95	Down
Anapc11	1751.29	-0.2454	0.0954	-2.5729	0.01009	0.06232	1958.43	1838.7	1902.47	1684.57	1587.69	1535.85	Down
Npb	35.5525	-0.848	0.42576	-1.9918	0.04639	0.18179	44.0646	37.4843	55.5538	18.3549	33.9998	23.8579	Down
Hmga1b	381.845	-1.225	0.42735	-2.8664	0.00415	0.03248	497.44	560.292	546.766	292.658	318.362	75.55	Down
Ccdc57	1126.37	-0.6548	0.20345	-3.2186	0.00129	0.01339	1446.3	1124.53	1562.33	991.163	668.664	965.25	Down
Slc16a3	1250.9	-0.6389	0.17882	-3.5726	0.00035	0.00492	1814.48	1186.67	1569.15	1012.58	904.602	1017.94	Down
Sectm1a	342.682	-0.9103	0.41374	-2.2002	0.02779	0.12812	383.851	343.278	614.99	263.086	100.969	349.916	Down
Cybc1	2656.22	0.20835	0.08813	2.36421	0.01807	0.09578	2531.26	2497.64	2365.42	2744.05	2869.38	2929.55	Up

Narf	1264.77	0.35115	0.15341	2.28901	0.02208	0.109	918.502	1153.14	1263.12	1448	1487.75	1318.15	Up
Fn3k	169.27	-0.8759	0.19555	-4.479	7.50E-06	0.00022	222.281	194.327	240.733	117.267	125.696	115.313	Down
Metrnl	1045.33	0.68026	0.25313	2.68741	0.0072	0.04857	649.218	1112.69	648.127	1384.77	1468.18	1008.99	Up
Dnmt3a	2139.7	0.32695	0.0965	3.38815	0.0007	0.00834	1781.19	2000.48	1913.19	2304.56	2420.17	2418.59	Up
Efr3b	742.526	0.33613	0.16912	1.98746	0.04687	0.18318	623.759	781.252	564.309	741.333	873.693	870.813	Up
Ncoa1	3113.53	0.34088	0.07955	4.28504	1.83E-05	0.00045	2707.52	2835	2699.72	3468.05	3490.65	3480.27	Up
AC241534	51.4169	0.67888	0.33782	2.0096	0.04447	0.17643	37.2101	41.43	39.9597	49.966	78.3027	61.6329	Up
Itsn2	7047.97	0.46865	0.09935	4.717	2.39E-06	8.46E-05	5934.03	6257.91	5547.58	7819.17	8731.78	7997.36	Up
Fkbp1b	391.42	0.80546	0.15513	5.19233	2.08E-07	1.05E-05	312.369	277.187	265.099	524.133	523.392	446.341	Up
Apob	122.215	2.4353	0.54313	4.48381	7.33E-06	0.00022	40.1477	13.81	60.4269	113.188	331.756	173.964	Up
Gm48898	160.337	1.74733	0.29903	5.84342	5.11E-09	4.03E-07	74.4202	96.6701	49.706	259.008	285.393	196.828	Up
Sdc1	26220.2	0.30349	0.0733	4.14014	3.47E-05	0.00077	23084.9	23919.9	23412.5	29788.9	28993.6	28121.5	Up
Wdr35	2222.61	-0.7613	0.20869	-3.6479	0.00026	0.00397	3233.36	2138.58	3015.5	1664.17	1317.75	1966.29	Down
Gm5182	194.977	0.64168	0.27311	2.34954	0.0188	0.09807	200.739	152.897	103.31	236.574	259.635	216.709	Up
7420701I0	5.24016	3.27463	1.26637	2.58584	0.00971	NA	0	1.97286	0.97463	13.2563	9.27269	5.96447	Up
Kcns3	764.739	0.26738	0.13424	1.99181	0.04639	0.18179	694.262	733.904	653.975	796.397	935.511	774.387	Up
Lratd1	1545.49	-1.2724	0.33356	-3.8145	0.00014	0.00239	2461.74	1225.15	2871.25	795.378	645.997	1273.41	Down
Trib2	4740.8	-0.3035	0.1448	-2.0962	0.03606	0.15402	5900.74	4717.11	5095.35	4080.9	3795.62	4855.08	Down
Lpin1	2337.09	0.51473	0.13905	3.70178	0.00021	0.00337	2030.89	1713.43	2029.17	2508.5	3131.08	2609.46	Up
Ntsr2	179.388	-1.353	0.42611	-3.1752	0.0015	0.01499	193.884	136.127	443.455	115.228	106.121	81.5144	Down
E2f6	1844.82	-0.2379	0.08797	-2.7045	0.00684	0.04679	2054.39	1948.2	1987.27	1661.12	1677.33	1740.63	Down
Pqlc3	3932.19	0.29459	0.11645	2.52981	0.01141	0.068	3600.57	3479.14	3516.46	4175.73	4885.68	3935.56	Up
2410004P	3988.13	-0.6368	0.26027	-2.4468	0.01441	0.08117	5471.84	3415.02	5676.23	3556.77	2106.96	3701.95	Down
Kcnf1	208.837	-2.376	0.37736	-6.2963	3.05E-10	2.97E-08	483.731	384.708	182.255	56.0843	59.7573	86.4848	Down
Gm36862	14.2433	-3.2566	0.86209	-3.7775	0.00016	0.00267	45.0438	14.7964	17.5433	2.03943	2.0606	3.97631	Down
5730507C	74.4401	1.18618	0.36888	3.21568	0.0013	0.01348	35.2517	59.1858	41.909	83.6166	142.181	84.4967	Up
Adam17	3447.96	0.33013	0.16218	2.0356	0.04179	0.16966	3134.46	3595.54	2435.59	3586.34	3932.65	4003.15	Up
2410018L	6.57343	2.00624	1.01303	1.98044	0.04765	NA	1.95843	4.93215	0.97463	7.138	16.4848	7.95263	Up
Grhl1	449.207	-0.7746	0.21644	-3.5791	0.00034	0.00485	601.237	443.893	655.924	338.545	269.938	385.702	Down
Id2	5210.96	0.76913	0.19108	4.0251	5.70E-05	0.00116	3487.96	4718.09	3355.64	6840.25	7591.24	5272.59	Up
Gm36723	147.52	0.53812	0.25292	2.12765	0.03337	0.14575	105.755	155.856	99.412	159.076	175.151	189.869	Up
Cmpk2	1514.23	-0.3928	0.18716	-2.0985	0.03586	0.15331	1311.17	2137.59	1708.52	1278.72	1333.21	1316.16	Down
Sh3yl1	881.33	0.45377	0.14295	3.17438	0.0015	0.015	631.592	785.198	814.788	1071.72	975.693	1008.99	Up
Fam110c	428.608	0.35021	0.1663	2.10583	0.03522	0.15124	404.415	404.436	321.627	481.305	525.452	434.412	Up
Gm32899	5.74228	-2.9022	1.08743	-2.6688	0.00761	NA	12.7298	8.87787	8.77165	1.01971	2.0606	0.99408	Down
Slc26a4	6474.24	1.34291	0.30605	4.38792	1.14E-05	0.00031	3405.7	5572.34	2005.78	8652.28	8844.08	10365.3	Up
Bcap29	3250.58	0.2222	0.11238	1.97718	0.04802	0.18621	2861.26	3337.09	2804	3340.59	3540.11	3620.43	Up
Hbp1	632.247	0.3464	0.1173	2.95317	0.00315	0.02659	572.839	551.414	545.791	743.372	715.027	665.039	Up
5430401H	27.085	1.55717	0.57353	2.71505	0.00663	0.04576	11.7506	21.7015	7.79702	43.8477	24.7272	52.6862	Up
Pik3cg	961.64	1.09112	0.14654	7.44583	9.63E-14	1.90E-11	627.675	657.949	557.487	1311.35	1469.21	1146.17	Up
Ccdc71l	1961.56	0.50256	0.1331	3.77587	0.00016	0.00268	1510.93	1742.03	1616.91	2168.93	2622.11	2108.44	Up
Nampt	6074.93	0.36974	0.14862	2.48774	0.01286	0.07427	4533.76	4992.32	6376.01	6645.48	7063.73	6838.27	Up
Efcab10	395.734	-1.1694	0.28749	-4.0676	4.75E-05	0.00101	654.114	354.128	635.457	264.106	180.302	286.295	Down
Agr3	904.322	-1.0445	0.35038	-2.981	0.00287	0.02487	1606.89	714.175	1333.29	631.204	344.12	796.257	Down
Crppa	392.848	-0.3764	0.16628	-2.2636	0.0236	0.11397	392.664	420.219	518.502	327.328	354.423	343.951	Down
Arl4a	2482.47	-0.754	0.26925	-2.8002	0.00511	0.03799	3563.36	1923.54	3863.42	2202.58	1437.27	1904.65	Down
Pnpla8	5793.58	0.246	0.10191	2.41391	0.01578	0.08662	5568.78	5517.1	4816.61	6123.39	6545.49	6190.13	Up
Stxbp6	1955.44	-0.7825	0.15025	-5.2079	1.91E-07	9.83E-06	2841.68	2021.19	2556.45	1441.88	1429.02	1442.41	Down
Strn3	2868.74	0.22534	0.10952	2.05759	0.03963	0.16409	2829.92	2573.59	2532.08	2900.07	3018.77	3358	Up
Heatr5a	3668.52	0.35838	0.11775	3.04343	0.00234	0.02129	3341.07	2973.1	3331.28	3980.97	4571.43	3813.29	Up
Nubpl	600.593	-0.759	0.18894	-4.0171	5.89E-05	0.00119	802.954	585.939	876.19	475.187	407.998	455.288	Down
Gm47431	64.6665	-1.6434	0.45262	-3.6309	0.00028	0.00417	130.235	80.8872	82.8433	42.828	14.4242	36.7809	Down
Akap6	542.165	-1.9191	0.32582	-5.8901	3.86E-09	3.12E-07	1078.11	540.563	954.16	245.751	141.151	293.253	Down
Sptssa	4529.68	-0.2137	0.09892	-2.1606	0.03072	0.13726	4993.99	4541.52	5058.32	4316.45	3931.62	4336.17	Down
Baz1a	2528.87	0.54969	0.11895	4.6214	3.81E-06	0.00012	2182.67	1985.68	1990.19	3325.29	2722.05	2967.32	Up
Nfkbia	6632.2	0.40861	0.13029	3.13622	0.00171	0.01663	5681.39	5484.55	5931.58	8491.17	7750.94	6453.56	Up
Gm36634	35.4734	-2.1384	0.64037	-3.3393	0.00084	0.00957	67.5657	55.2401	50.6806	20.3943	2.0606	16.8993	Down
Gm47682	29.0422	-1.1279	0.41713	-2.7039	0.00685	0.04685	38.1893	41.43	39.9597	19.3746	14.4242	20.8756	Down
Pax9	148.101	-3.2152	1.6343	-1.9673	0.04914	0.189	645.301	1.97286	154.966	20.3943	10.303	55.6684	Down
Gm2568	73.0738	-1.4317	0.67543	-2.1197	0.03403	0.14769	151.778	43.4029	124.752	33.6506	10.303	74.5559	Down
Foxa1	5025.55	-0.4574	0.18078	-2.5303	0.0114	0.06795	6641.02	4758.54	6047.56	4233.86	3500.95	4971.39	Down
Mia2	7744.03	0.23578	0.08546	2.75887	0.0058	0.04176	7197.21	7083.55	7057.28	8389.19	7907.54	8829.41	Up
4930471E	9.81612	-2.8697	1.05437	-2.7217	0.00649	0.04514	26.4387	2.95929	22.4164	2.03943	2.0606	2.98224	Down
Gm31063	272.074	-2.7423	1.22353	-2.2413	0.025	0.11883	883.25	78.9144	458.075	40.7886	12.3636	159.053	Down
Wdr20rt	34.9648	-3.0202	1.09298	-2.7633	0.00572	0.0413	125.339	5.91858	55.5538	3.05914	1.0303	18.8875	Down
Rpl17-ps3	497.161	-0.2458	0.12085	-2.0341	0.04194	0.17006	525.837	555.36	537.02	474.167	449.21	441.371	Down
Gm47518	2.03794	4.49809	2.05826	2.18538	0.02886	NA	0	0	0	6.11829	4.12119	1.98816	Up
Pole2	161.076	0.55016	0.23878	2.30405	0.02122	0.10646	104.776	155.856	131.575	163.154	213.272	197.822	Up
Nemf	4037.19	0.22737	0.09251	2.45766	0.01398	0.07919	3950.14	3644.86	3564.21	4475.53	4171.68	4416.69	Up
Arf6	4003.71	0.21569	0.09965	2.1645	0.03043	0.13644	3931.54	3610.33	3572.98	4589.74	4246.89	4070.75	Up
L2hgdh	769.465	-0.3229	0.12466	-2.5898	0.0096	0.05994	849.957	926.257	789.448	722.978	652.179	675.973	Down
Dmac2l	953.552	-0.4089	0.13657	-2.9941	0.00275	0.02407	1130.01	1055.48	1077.94	921.822	712.967	823.097	Down
Cdkl1	308.456	-1.2655	0.28058	-4.5105	6.47E-06	0.00019	461.209	321.576	524.349	216.18	123.636	203.786	Down
4930512B	6.55857	-2.7615	1.05981	-2.6057	0.00917	NA	11.7506	11.8372	10.7209	0	2.0606	2.98224	Down
Atl1	448.865	-0.5359	0.16428	-3.2624	0.0011	0.0119	505.274	484.337	604.269	404.827	328.665	365.821	Down
Sav1	1971.67	0.37282	0.11942	3.12205	0.0018	0.01724	1821.34	1755.84	1577.92	2088.38	2145.08	2441.46	Up
Nin	3160.18	-0.2392	0.12133	-1.9712	0.0487	0.18768	3615.25	3472.23	3177.28	2664.52	2792.11	3239.7	Down
Abhd12b	643.628	-0.8053	0.36068	-2.2327	0.02557	0.12076	720.701	630.329	1105.23	626.105	227.696	551.714	Down
Gm24474	35.5368	-1.0315	0.46092	-2.2379	0.02522	0.11948	53.8567	52.2808	37.0358	13.2563	31.9393	24.852	Down
Timm9	806.29	-0.2501	0.12056	-2.0747	0.03801	0.15939	827.435	935.135	865.469	686.268	777.875	745.559	Down
Dact1	40.0928	0.73761	0.35391	2.08419	0.03714	0.15696	32.314	28.6065	29.2388	50.9857	56.6664	42.7454	Up
Lrrc9	361.395	-0.9744	0.28571	-3.4105	0.00065	0.00788	442.604	357.088	637.406	255.948	174.12	301.206	Down
Six1	981.09	-1.3012	0.36903	-3.526	0.00042	0.00564	1502.11	835.506	1849.84	570.021	304.968	824.091	Down

Six4	984.408	-1.1287	0.32552	-3.4674	0.00053	0.00671	1370.9	808.872	1873.23	680.15	403.877	769.417	Down
Mnat1	1560.09	-0.3392	0.13221	-2.5658	0.01029	0.06325	1727.33	1535.87	1964.85	1386.81	1302.3	1443.4	Down
Prkch	4218.06	0.23844	0.11442	2.08387	0.03717	0.15698	4352.6	3680.37	3577.86	4449.02	4711.55	4536.97	Up
Dbpht2	11.2943	-2.2049	1.04477	-2.1104	0.03482	0.14998	26.4387	2.95929	26.3149	2.03943	2.0606	7.95263	Down
Rhoj	2367.66	0.82953	0.29344	2.82688	0.0047	0.03578	1234.79	2658.43	1222.18	2940.86	3458.71	2690.97	Up
Sgpp1	4040.14	-0.2672	0.12035	-2.2204	0.02639	0.12361	4618.95	4018.71	4602.19	3687.29	3316.53	3997.19	Down
Hspa2	2376.8	-0.8478	0.27628	-3.0688	0.00215	0.01993	3650.51	2161.27	3355.64	2000.68	1078.72	2014	Down
Ppp1r36	3083.67	-1.1602	0.31742	-3.6552	0.00026	0.00389	4950.9	2663.36	5168.45	2288.24	1117.87	2313.22	Down
Sptb	192.527	-0.6678	0.24506	-2.725	0.00643	0.04484	235.011	181.503	292.388	168.253	131.878	146.13	Down
Mpp5	5405.29	0.23331	0.11468	2.03436	0.04191	0.17006	4441.71	4906.5	5559.27	5815.43	5897.43	5811.38	Up
Arg2	594.448	0.47696	0.18121	2.63205	0.00849	0.05495	595.361	417.26	478.542	621.006	768.603	685.914	Up
Zfyve26	1674.69	0.54952	0.1304	4.21405	2.51E-05	0.00059	1262.21	1526.01	1290.41	1901.77	2177.02	1890.74	Up
Rad51b	778.64	-0.9	0.29131	-3.0893	0.00201	0.01884	944.94	776.32	1320.62	473.148	387.392	769.417	Down
9430078K	119.252	-1.4298	0.38885	-3.6771	0.00024	0.00365	192.905	130.209	198.824	53.0252	39.1513	101.396	Down
Plekhd1os	159.158	-0.5814	0.24287	-2.3939	0.01667	0.09038	196.822	202.218	173.484	105.031	114.363	163.029	Down
Plekhd1	3506.03	0.34629	0.1462	2.36866	0.01785	0.09498	3046.33	3268.04	2947.27	3716.86	4655.92	3401.74	Up
Susd6	12771	0.29658	0.13018	2.27822	0.02271	0.11095	10488.3	13081	10819.3	13121.7	13734.9	15380.4	Up
Smoc1	471.619	-2.2337	0.44811	-4.9847	6.21E-07	2.70E-05	1103.57	247.594	982.424	167.233	137.03	191.857	Down
Rgs6	518.484	-0.442	0.17479	-2.5288	0.01145	0.06807	533.671	654.003	604.269	360.979	471.877	486.104	Down
Acot1	1989.59	-0.6456	0.28173	-2.2914	0.02194	0.10854	3025.77	2883.33	1373.25	1795.72	1541.33	1318.15	Down
Dnal1	2920.23	-0.4993	0.20939	-2.3845	0.0171	0.09211	3736.68	2600.23	3924.82	2570.7	1890.6	2798.33	Down
Pnma1	141.767	-0.592	0.28139	-2.1039	0.03539	0.15178	224.24	134.154	153.016	102.991	102	134.201	Down
Fam161b	1126.37	-0.7012	0.29783	-2.3545	0.01855	0.09728	1520.72	915.407	1748.48	965.67	551.21	1056.71	Down
Entpd5	3913.58	-0.646	0.21841	-2.9575	0.0031	0.02635	4475	3719.83	6131.38	3157.04	2430.47	3567.75	Down
Bbof1	693.985	-0.9496	0.30934	-3.0698	0.00214	0.01988	958.649	596.79	1188.07	560.843	301.877	557.678	Down
Aldh6a1	169.98	-0.8077	0.27071	-2.9835	0.00285	0.02474	235.99	150.924	262.175	125.425	108.181	137.183	Down
Abcd4	1552.82	0.47596	0.18372	2.59062	0.00958	0.05987	1173.1	1520.09	1203.66	1772.26	2164.66	1483.17	Up
Npc2	133932	0.28674	0.14588	1.96554	0.04935	0.18927	114878	144382	102737	144527	153537	143528	Up
Ltbp2	4336.32	0.27245	0.09107	2.99167	0.00277	0.02418	3752.34	4069.02	3962.83	5009.86	4631.19	4592.64	Up
Arel1	1520.95	0.24437	0.12117	2.01672	0.04373	0.17471	1421.82	1418.49	1337.19	1502.04	1603.14	1843.02	Up
Pgf	298.202	0.93243	0.1952	4.7767	1.78E-06	6.50E-05	214.448	167.693	232.936	423.182	407.998	342.957	Up
Eif2b2	2753.05	-0.2622	0.10302	-2.5448	0.01093	0.06598	3072.77	2879.39	3055.46	2658.4	2295.5	2556.77	Down
Acyp1	395.853	-0.2723	0.1355	-2.0097	0.04447	0.17643	453.376	407.395	438.582	358.94	342.059	374.768	Down
Zc2hc1c	894.997	-0.501	0.20999	-2.3858	0.01704	0.09184	1218.14	789.144	1139.34	796.397	606.846	820.115	Down
Flvcr2	929.536	0.93193	0.1795	5.19172	2.08E-07	1.05E-05	613.966	640.193	663.721	1264.45	1446.54	948.351	Up
Ttll5	1039.1	-0.3197	0.1276	-2.5051	0.01224	0.07163	1191.7	1034.76	1234.85	906.527	872.663	994.079	Down
Ift43	3514.83	-0.4905	0.13916	-3.5246	0.00042	0.00565	4374.14	3558.05	4387.77	3124.41	2579.87	3064.74	Down
Vash1	123.823	0.52437	0.25861	2.02762	0.0426	0.17166	126.318	81.8737	96.4881	166.214	131.878	140.165	Up
Gstz1	5670.35	-0.3162	0.14057	-2.2495	0.02448	0.11694	6922.05	5451.01	6494.92	5274.99	4438.53	5440.59	Down
Samd15	53.9873	-1.01	0.44696	-2.2598	0.02383	0.11482	80.2954	57.2129	78.9448	56.0843	19.5757	31.8105	Down
Ahsa1	6476.74	-0.3093	0.13204	-2.3424	0.01916	0.09937	8073.61	6616.97	6814.59	6098.91	5129.86	6126.51	Down
Ism2	21.3943	-1.9572	0.70963	-2.758	0.00581	0.04183	46.023	44.3893	11.6955	9.17743	5.15149	11.9289	Down
Cep128	1008.12	-0.5826	0.235	-2.4793	0.01316	0.07565	1296.48	912.447	1418.08	817.811	606.846	997.061	Down
Tshr	63.3317	-2.6359	0.5066	-5.203	1.96E-07	1.00E-05	184.092	80.8872	62.3761	17.3352	14.4242	20.8756	Down
Ston2	1621.38	-0.2637	0.1044	-2.526	0.01154	0.06846	1896.74	1761.76	1649.07	1440.86	1458.9	1520.94	Down
Sel1l	9434.98	0.2077	0.0815	2.54861	0.01082	0.06552	9218.31	8438.91	8613.76	9988.11	10067	10283.7	Up
Spata7	703.675	-0.5346	0.2095	-2.5518	0.01072	0.06512	892.063	612.573	993.145	564.922	516.18	643.169	Down
Eml5	579.031	-0.468	0.17546	-2.6675	0.00764	0.05065	655.093	646.111	715.376	490.483	392.544	574.577	Down
Efcab11	248.308	-1.3638	0.31942	-4.2698	1.96E-05	0.00048	447.5	228.852	396.673	154.997	95.8178	166.011	Down
Kcnk13	329.45	0.98012	0.29624	3.30854	0.00094	0.01045	183.113	273.241	208.57	380.354	621.27	310.153	Up
4930477G	4.56082	3.04792	1.53061	1.99131	0.04645	NA	0	2.95929	0	3.05914	13.3939	7.95263	Up
Gm10433	48.3703	-1.0996	0.33517	-3.2808	0.00104	0.01128	62.6696	58.1993	76.9956	33.6506	29.8787	28.8283	Down
Ttc7b	1328.42	0.37708	0.14898	2.53116	0.01137	0.06785	1144.7	1312.94	1009.71	1408.23	1678.36	1416.56	Up
Rps6ka5	640.168	0.37067	0.13256	2.79632	0.00517	0.03832	492.544	602.708	579.903	708.702	730.482	726.671	Up
Ppp4r3a	1098.06	0.41142	0.1618	2.54272	0.011	0.06625	946.899	880.882	999.968	1141.06	1106.54	1512.99	Up
Catsperb	290.329	1.15558	0.39615	2.91699	0.00353	0.029	76.3786	275.214	188.103	388.511	469.816	343.951	Up
Tc2n	3916.07	0.60795	0.22124	2.74798	0.006	0.04271	2353.05	4228.82	2727.01	4391.91	5286.46	4509.14	Up
Lgmn	9261.22	0.39511	0.12005	3.2911	0.001	0.01098	7453.77	9020.9	7528.02	11016	10774.9	9773.78	Up
Unc79	606.865	-2.6583	0.33332	-7.9751	1.52E-15	4.57E-13	837.227	658.935	1647.12	132.563	184.423	180.922	Down
Otub2	623.463	-0.4025	0.12684	-3.1734	0.00151	0.01503	682.511	705.297	741.691	573.08	548.119	490.081	Down
Ifi27	9164.47	-0.4059	0.1056	-3.8442	0.00012	0.00217	10016.4	11225.6	10094.2	8542.15	7423.3	7685.22	Down
Gm47267	15.6492	-1.4433	0.66297	-2.177	0.02948	0.1335	19.5843	31.5657	17.5433	8.15772	4.12119	12.923	Down
Serpina16	5.41512	-2.8003	1.18355	-2.366	0.01798	NA	6.85449	7.89144	13.6448	2.03943	2.0606	0	Down
Serpina3n	9082.83	0.50563	0.17425	2.90173	0.00371	0.03011	6137.71	9168.86	7215.17	11386.1	11303.4	9285.69	Up
Gm28875	300.015	-0.352	0.15402	-2.2854	0.02229	0.1097	335.87	314.671	358.663	260.027	283.332	247.526	Down
Snhg10	48.9356	-0.8356	0.37119	-2.251	0.02438	0.11673	84.2123	55.2401	48.7314	36.7097	31.9393	36.7809	Down
D430019H	362.665	0.51381	0.21982	2.33742	0.01942	0.1001	262.429	355.115	278.743	333.447	505.877	440.377	Up
Ak7	5563.55	-0.9754	0.32956	-2.9598	0.00308	0.02621	9419.05	4516.86	8191.74	3736.24	2238.84	5278.56	Down
Gm19554	11.1739	-1.6593	0.79529	-2.0864	0.03694	0.1566	22.5219	14.7964	13.6448	4.07886	2.0606	9.94079	Down
Evl	427.317	0.69759	0.22328	3.12428	0.00178	0.01713	329.995	387.667	260.225	494.562	649.088	442.365	Up
Slc25a47	359.654	-0.4469	0.22228	-2.0106	0.04437	0.17627	425.958	342.291	476.593	326.309	233.878	352.898	Down
Wdr25	353.179	-0.605	0.1861	-3.2509	0.00115	0.01232	405.394	395.558	477.567	326.309	234.908	279.336	Down
Gm34081	2.37357	4.71831	1.94712	2.42323	0.01538	NA	0	0	0	7.138	4.12119	2.98224	Up
Meg3	1087.05	2.13646	0.37799	5.65215	1.58E-08	1.10E-06	337.828	436.002	434.684	1201.22	3112.53	1000.04	Up
Rian	698.281	1.67014	0.34801	4.79916	1.59E-06	5.90E-05	331.953	339.332	330.399	745.412	1785.51	657.086	Up
Mirg	41.1767	1.49655	0.50649	2.95477	0.00313	0.0265	18.605	16.7693	29.2388	43.8477	96.8481	41.7513	Up
Dio3os	24.1967	-1.7364	0.50016	-3.4716	0.00052	0.00663	31.3348	32.5522	47.7567	13.2563	11.3333	8.94671	Down
B930059L	25.282	-0.9485	0.46879	-2.0233	0.04304	0.17289	26.4387	30.5793	42.8836	20.3943	16.4848	14.9112	Down
Hsp90aa1	33664.8	-0.5523	0.22535	-2.4509	0.01425	0.08044	53050.8	32590.6	34452.1	25922.2	22429.6	33543.2	Down
Mok	1800.89	-0.8249	0.25711	-3.2084	0.00133	0.01375	2530.29	1567.44	2808.88	1489.8	941.693	1467.26	Down
Ankrd9	342.285	-0.9872	0.22501	-4.3872	1.15E-05	0.00031	438.687	359.06	567.233	257.988	234.908	195.833	Down
Cdc42bpb	5192.61	0.27614	0.10305	2.6796	0.00737	0.04938	5139.89	4614.52	4337.09	5664.52	5840.76	5558.89	Up
Tnfaip2	612.469	1.65755	0.23292	7.11637	1.11E-12	1.82E-10	339.787	314.671	230.012	749.49	1187.93	852.919	Up

Mark3	3977.67	-0.2096	0.08324	-2.5187	0.01178	0.06963	4343.79	4384.68	4070.04	3752.55	3691.56	3623.42	Down
Ckb	5477.07	-0.4165	0.20407	-2.041	0.04125	0.16818	7095.38	4455.7	7235.63	5348.4	4419.98	4307.34	Down
Klc1	4978.61	0.28354	0.09414	3.01199	0.0026	0.02304	4226.28	4421.18	4825.38	5369.82	5421.43	5607.6	Up
Xrcc3	48.1933	-1.454	0.6369	-2.2829	0.02244	0.11016	133.173	56.2265	22.4164	29.5717	29.8787	17.8934	Down
5033406C	124.082	0.87796	0.34246	2.56368	0.01036	0.06351	58.7528	70.0365	133.524	168.253	163.817	150.106	Up
Aspg	319.496	-2.556	0.49982	-5.1139	3.16E-07	1.52E-05	932.211	454.744	251.454	144.8	44.3028	89.4671	Down
A530016L	55.4433	-9.2147	2.15278	-4.2804	1.87E-05	0.00046	223.261	101.602	7.79702	0	0	0	Down
Tmem179	13.4352	-4.2336	1.20587	-3.5108	0.00045	0.0059	41.1269	26.6336	8.77165	4.07886	0	0	Down
Adssl1	2593.85	-1.4488	0.13444	-10.776	4.45E-27	6.01E-24	3577.06	3415.02	4398.49	1341.94	1370.3	1460.3	Down
Siva1	1163.03	-0.7043	0.10831	-6.5024	7.90E-11	8.80E-09	1349.36	1484.58	1490.21	922.842	891.208	839.996	Down
Ahnak2	2058.73	1.08695	0.24811	4.38092	1.18E-05	0.00032	1051.67	1344.5	1557.45	2951.05	3566.89	1880.8	Up
Clba1	1848.97	-0.9675	0.1742	-5.5539	2.79E-08	1.79E-06	2971.91	2283.58	2084.73	1327.67	1084.9	1341.01	Down
Gpr132	457.273	0.79778	0.29133	2.73845	0.00617	0.04364	210.531	488.283	303.109	603.671	617.149	520.897	Up
Jag2	2750.7	-0.3897	0.16022	-2.4322	0.01501	0.08347	3252.94	2521.31	3585.65	2460.57	2165.69	2518	Down
Nudt14	1573.2	-0.5384	0.14723	-3.6565	0.00026	0.00387	1778.25	1638.46	2173.42	1356.22	1178.66	1314.17	Down
Gm26583	179.477	-3.6467	0.90797	-4.0163	5.91E-05	0.0012	661.948	248.58	86.7418	13.2563	20.606	45.7276	Down
Crip1	3640.1	-0.721	0.30634	-2.3534	0.0186	0.09738	7078.73	3444.61	3070.08	2980.63	3137.26	2129.32	Down
Tmem121	34.2512	-2.5834	0.5563	-4.6439	3.42E-06	0.00011	76.3786	28.6065	71.1478	10.1971	8.24239	10.9349	Down
Ighm	15064.1	1.4018	0.22186	6.3185	2.64E-10	2.66E-08	6752.65	10663.3	7399.37	25377.6	16550.7	23641.2	Up
Ighv2-2	82.1206	2.60636	0.81581	3.1948	0.0014	0.0142	30.3556	11.8372	27.2896	180.49	231.817	10.9349	Up
Ighv2-3	10.2509	3.24346	0.97331	3.33241	0.00086	0.00975	0	1.97286	3.89851	12.2366	18.5454	24.852	Up
Ighv5-9	13.6072	3.23169	1.17929	2.74036	0.00614	0.04344	0	4.93215	2.92388	27.5323	43.2725	2.98224	Up
Ighv5-16	31.6286	5.98553	1.36592	4.38206	1.18E-05	0.00032	0	2.95929	0	4.07886	51.5149	131.218	Up
Ighv4-1	77.2725	3.55494	1.57371	2.25896	0.02389	0.11504	18.605	17.7557	0	28.552	87.5754	311.147	Up
Ighv4-2	4.42941	-4.5499	2.03697	-2.2336	0.02551	NA	13.709	11.8372	0	0	1.0303	0	Down
Ighv3-2	6.86171	-6.2003	2.19066	-2.8303	0.00465	NA	35.2517	5.91858	0	0	0	0	Down
Ighv11-2	28.6333	3.41692	0.77771	4.39356	1.12E-05	0.0003	4.89606	5.91858	3.89851	97.8926	15.4545	43.7395	Up
Ighv7-3	92.5765	2.854	0.81071	3.52039	0.00043	0.00573	29.3764	8.87787	29.2388	70.3603	30.909	386.697	Up
Ighv14-4	36.7376	2.04042	0.57102	3.57326	0.00035	0.00491	17.6258	9.8643	15.594	91.7743	58.727	26.8401	Up
Ighv3-5	6.64588	5.20552	1.59909	3.2553	0.00113	NA	0	0	0.97463	10.1971	24.7272	3.97631	Up
Ighv3-6	61.0077	2.83581	0.60875	4.65841	3.19E-06	0.00011	10.7713	5.91858	28.2642	124.405	52.5452	144.141	Up
Ighv3-8	15.2229	-2.8635	1.30733	-2.1903	0.0285	0.13047	67.5657	7.89144	4.87314	0	2.0606	8.94671	Down
Ighv6-6	190.136	4.60548	0.58588	7.86077	3.82E-15	1.08E-12	11.7506	8.87787	24.3657	666.894	126.727	302.2	Up
Ighv10-1	19.3904	2.22377	0.88534	2.51177	0.01201	0.0706	5.87528	0.98643	13.6448	39.7689	11.3333	44.7335	Up
Ighv1-9	60.0306	1.8253	0.73917	2.4694	0.01353	0.07726	51.8983	4.93215	22.4164	87.6955	152.484	40.7572	Up
Ighv1-12	42.461	2.99674	0.67696	4.42673	9.57E-06	0.00027	1.95843	5.91858	20.4672	56.0843	94.7875	75.55	Up
Ighv1-14	6.56972	-5.1377	1.98805	-2.5843	0.00976	NA	9.79213	28.6065	0	1.01971	0	0	Down
Ighv1-20	2.0397	4.49919	2.04947	2.19529	0.02814	NA	0	0	0	5.09857	5.15149	1.98816	Up
Ighv1-21	2.12567	-4.5096	2.15362	-2.0939	0.03626	NA	7.8337	3.94572	0.97463	0	0	0	Down
Ighv1-26	47.2945	1.90448	0.47103	4.04327	5.27E-05	0.0011	8.81291	28.6065	22.4164	59.1435	78.3027	86.4848	Up
Ighv1-33	5.23871	-3.8589	1.67172	-2.3083	0.02098	NA	23.5011	4.93215	0.97463	0	1.0303	0.99408	Down
Ighv1-39	14.9472	3.38659	0.94426	3.58648	0.00034	0.00476	0	1.97286	5.84776	34.6703	13.3939	33.7987	Up
Ighv1-42	6.22481	2.15283	0.98429	2.1872	0.02873	NA	0.97921	1.97286	3.89851	8.15772	13.3939	8.94671	Up
Ighv1-47	8.66941	4.05728	1.38442	2.93067	0.00338	0.0281	0.97921	1.97286	0	30.5914	16.4848	1.98816	Up
Ighv1-53	41.2617	2.24646	0.83127	2.70244	0.00688	0.04697	30.3556	4.93215	7.79702	142.76	30.909	30.8164	Up
Ighv1-55	101.803	4.52261	0.46122	9.80565	1.06E-22	9.59E-20	8.81291	4.93215	11.6955	252.889	211.211	121.278	Up
Ighv1-58	14.3944	3.3195	1.48778	2.23119	0.02567	0.12104	0	5.91858	1.94925	73.4195	3.0909	1.98816	Up
Ighv1-62-	3.71986	3.37147	1.58828	2.12271	0.03378	NA	0	1.97286	0	4.07886	10.303	5.96447	Up
Ighv8-11	6.04216	-5.015	2.24178	-2.237	0.02528	NA	34.2724	0.98643	0	0	0	0.99408	Down
Ighv1-69	233.829	3.94845	1.23663	3.19292	0.00141	0.01426	31.3348	34.525	19.4925	44.8675	122.606	1150.15	Up
Ighv8-12	33.8032	2.15257	0.59507	3.61735	0.0003	0.00434	16.6466	11.8372	8.77165	24.4732	54.6058	86.4848	Up
Ighv1-82	71.4845	3.19892	0.75502	4.23687	2.27E-05	0.00054	33.2932	4.93215	3.89851	81.5772	85.5148	219.691	Up
Ighv1-83	9.6411	-4.7996	1.96025	-2.4484	0.01435	0.08091	49.9399	5.91858	0	0	0	1.98816	Down
Ighv1-84	9.83939	-3.7529	1.61911	-2.3179	0.02046	0.10384	39.1685	15.7829	0	0	3.0909	0.99408	Down
Gm9260	135.654	-1.1054	0.40253	-2.7463	0.00603	0.04289	231.094	84.8329	239.758	83.6166	75.2118	99.4079	Down
Wdr60	2071.55	-0.6917	0.23112	-2.9929	0.00276	0.0241	3056.12	1863.37	2757.22	1611.15	1220.9	1920.56	Down
Esyt2	9369.27	0.27436	0.12027	2.28121	0.02254	0.11046	7575.19	8315.6	9552.32	9711.76	10581.2	10479.6	Up
Dnah11	1486.68	-0.7849	0.26202	-2.9954	0.00274	0.02398	2104.33	1393.83	2146.13	1113.53	739.754	1422.53	Down
Itgb8	108.638	0.52266	0.2271	2.30149	0.02136	0.10682	78.337	90.7515	98.4374	131.543	120.545	132.212	Up
Ncoa4-ps	227.502	0.92293	0.18635	4.95273	7.32E-07	3.07E-05	175.279	139.087	156.915	296.737	326.605	270.389	Up
Macc1	1278.41	-0.4574	0.16241	-2.816	0.00486	0.03655	1452.17	1372.12	1613.98	1021.75	921.087	1289.32	Down
Gm16505	51.6421	-1.821	0.52141	-3.4925	0.00048	0.00624	65.6073	114.426	61.4015	27.5323	31.9393	8.94671	Down
Calml3	726.523	-2.1839	0.70027	-3.1186	0.00182	0.01737	1816.44	502.093	1254.35	181.509	151.454	453.3	Down
Akr1c21	10.0046	3.20151	1.20671	2.6531	0.00798	0.05232	0	3.94572	1.94925	12.2366	4.12119	37.775	Up
Gm35330	164.561	-0.5989	0.27976	-2.1408	0.03229	0.14235	146.882	201.232	246.581	101.971	153.514	137.183	Down
Gm47507	34.9963	1.71419	0.47747	3.59017	0.00033	0.0047	18.605	21.7015	8.77165	43.8477	49.4543	67.5973	Up
Gm46401	30.4117	1.32908	0.45957	2.89203	0.00383	0.03077	20.5635	18.7422	12.6702	33.6506	40.1816	56.6625	Up
Gm10029	8.21955	-2.8276	0.98381	-2.8741	0.00405	0.03194	10.7713	24.6607	7.79702	2.03943	2.0606	1.98816	Down
Gm5191	10.3633	1.4515	0.71494	2.03026	0.04233	0.17098	7.8337	3.94572	4.87314	14.276	12.3636	18.8875	Up
Adarb2	141.335	0.50294	0.24044	2.09171	0.03646	0.15516	122.402	105.548	122.803	164.174	199.878	133.207	Up
Idi1	2396.4	0.49461	0.12911	3.83092	0.00013	0.00227	2109.22	2127.73	1731.91	2584.98	2863.2	2961.36	Up
Gm36264	54.8099	-2.455	0.46301	-5.3023	1.14E-07	6.22E-06	58.7528	88.7787	130.6	23.4534	12.3636	14.9112	Down
Zmynd11	1297.84	0.34097	0.14403	2.36746	0.01791	0.09518	1204.43	1088.03	1143.24	1341.94	1310.54	1698.88	Up
Chrm3	288.398	-0.6453	0.31629	-2.0402	0.04133	0.16839	363.288	273.241	419.09	240.653	132.908	301.206	Down
Ryr2	633.95	-1.9966	0.39515	-5.0529	4.35E-07	1.99E-05	1245.56	475.459	1320.62	215.16	174.12	372.779	Down
Ero1lb	979.077	1.6913	0.63924	2.6458	0.00815	0.05324	419.103	497.161	472.694	1570.36	466.725	2448.42	Up
Gpr137b-	1123.03	2.02645	0.37924	5.34348	9.12E-08	5.13E-06	404.415	460.663	462.948	1818.15	746.966	2845.05	Up
Gm7046	34.7346	-2.3234	0.7349	-3.1616	0.00157	0.01557	82.2539	64.1179	27.2896	20.3943	12.3636	1.98816	Down
Tbce	3724.06	0.92316	0.25169	3.66788	0.00024	0.00375	2692.84	2514.41	2507.72	5406.53	2935.32	6287.55	Up
Gli3	213.313	-0.3815	0.1705	-2.2377	0.02524	0.1195	242.845	223.92	257.302	173.352	190.605	191.857	Down
Gm5628	71.1975	-1.1746	0.33656	-3.4901	0.00048	0.00628	73.441	108.507	114.031	48.9463	49.4543	32.8046	Down
A930027P	49.4152	-1.2681	0.39172	-3.2372	0.00121	0.01279	77.3578	52.2808	79.9194	28.552	20.606	37.775	Down
Trgc2	104.029	0.50867	0.25225	2.01654	0.04374	0.17474	89.1084	77.9279	90.6403	107.07	112.303	147.124	Up

Sfrp4	67.9292	-0.8272	0.27781	-2.9776	0.00291	0.02507	85.1915	88.7787	86.7418	44.8675	45.3331	56.6625 Down
Aoah	163.858	1.38376	0.38372	3.60617	0.00031	0.00449	69.5241	96.6701	106.234	184.568	149.393	376.756 Up
Gpx6	27.4535	8.25052	3.9084	2.11097	0.03477	0.14983	0	0	0	67.3012	0	97.4197 Up
AK157302	105.081	-0.6798	0.33368	-2.0374	0.04161	0.16916	99.8797	139.087	149.118	78.518	109.212	54.6743 Down
Zfp184	147.205	-0.7371	0.21642	-3.4058	0.00066	0.00798	186.05	198.272	167.636	119.307	119.515	92.4493 Down
H4c9	251.434	-0.8911	0.4492	-1.9837	0.04729	0.18419	381.893	300.861	297.261	144.8	313.211	70.5796 Down
Btn1a1	2.02939	4.49271	1.9953	2.25165	0.02434	NA	0	0	0	4.07886	4.12119	3.97631 Up
Btn2a2	10.0749	2.05411	0.92947	2.20998	0.02711	0.1256	4.89606	0	6.82239	17.3352	16.4848	14.9112 Up
H4c8	65.7524	-1.1912	0.44221	-2.6936	0.00707	0.04784	135.131	64.1179	75.0463	46.9069	49.4543	23.8579 Down
H1f3	84.9738	-0.8898	0.28876	-3.0816	0.00206	0.0192	134.152	86.8058	110.133	60.1632	64.9088	53.6802 Down
H2bc6	599.919	-0.6586	0.1741	-3.783	0.00015	0.00262	753.015	744.754	705.63	479.266	546.058	370.791 Down
H1f4	41.1392	-1.003	0.37105	-2.7031	0.00687	0.04691	66.5865	52.2808	45.8075	23.4534	29.8787	28.8283 Down
H4c3	43.2913	-1.831	0.53998	-3.3909	0.0007	0.0083	108.693	38.4708	55.5538	15.2957	27.8181	13.9171 Down
H1f2	5503.24	-0.4999	0.14602	-3.4235	0.00062	0.00761	7035.64	6382.2	5923.78	4910.95	4979.43	3787.44 Down
Ripor2	1403.65	-0.3889	0.13657	-2.8479	0.0044	0.03396	1368.94	1691.73	1714.37	1238.95	1279.63	1128.28 Down
Acot13	1772.13	-0.447	0.12303	-3.6332	0.00028	0.00414	2135.66	1961.02	2036.97	1558.12	1315.69	1625.32 Down
Dcdc2a	907.576	-1.4654	0.33707	-4.3473	1.38E-05	0.00036	1914.36	953.877	1129.59	461.931	298.787	686.908 Down
Gm11353	159.979	-0.4164	0.20956	-1.9872	0.0469	0.18318	181.154	183.476	184.205	134.602	115.393	161.041 Down
E2f3	563.978	0.4284	0.12583	3.40454	0.00066	0.00801	439.667	490.256	512.654	640.381	640.846	660.068 Up
Gm29675	33.7875	-1.0635	0.50187	-2.1191	0.03408	0.14784	33.2932	48.3351	55.5538	15.2957	15.4545	34.7927 Down
Foxq1	561.471	-0.625	0.21883	-2.8561	0.00429	0.03338	704.054	532.672	806.991	501.7	348.241	475.17 Down
Gmds	920.448	0.41429	0.13778	3.00685	0.00264	0.0233	874.437	723.053	769.956	1037.05	1153.93	964.256 Up
Mylk4	38.1399	-2.1449	1.09361	-1.9613	0.04984	0.19052	106.734	0.98643	78.9448	8.15772	6.18179	27.8342 Down
Serpinb1a	2066.98	0.64685	0.18095	3.57482	0.00035	0.0049	1749.85	1543.76	1539.91	2232.16	3194.96	2141.25 Up
Serpinb1b	118.773	-1.2074	0.3541	-3.4097	0.00065	0.0079	197.801	110.48	189.078	53.0252	62.8482	99.4079 Down
Ripk1	2762.98	0.31888	0.09987	3.1928	0.00141	0.01426	2602.75	2455.22	2318.64	2894.97	3190.83	3115.44 Up
Bphl	5658.14	-0.7263	0.1928	-3.7671	0.00017	0.00275	7406.77	5811.06	7941.26	4885.45	3261.92	4642.35 Down
Gm40909	10.2211	1.53996	0.76215	2.02053	0.04333	0.17364	4.89606	6.90501	3.89851	8.15772	19.5757	17.8934 Up
Pxdc1	9767.71	-0.313	0.09622	-3.253	0.00114	0.01224	11772.1	10443.3	10254.1	8752.21	8486.57	8898 Down
Fam217a	78.5474	-1.0997	0.27565	-3.9895	6.62E-05	0.00132	120.443	96.6701	104.285	47.9266	44.3028	57.6566 Down
Eci2	682.271	0.31458	0.1417	2.21994	0.02642	0.12367	641.384	645.125	537.994	811.693	766.542	690.885 Up
Ly86	777.656	0.56135	0.20652	2.71808	0.00657	0.04557	474.918	782.239	627.66	932.019	823.208	1025.89 Up
Gm47732	20.0606	-1.6981	0.62379	-2.7222	0.00649	0.04512	46.023	15.7829	30.2134	10.1971	7.21209	10.9349 Down
Gm47754	41.696	1.28929	0.48155	2.67737	0.00742	0.04955	24.4803	35.5115	12.6702	41.8083	71.0906	64.6151 Up
Rreb1	3037.17	0.27518	0.14017	1.96312	0.04963	0.19008	2605.69	3028.34	2611.03	3345.68	3754.41	2877.86 Up
Gcnt2	556.241	0.64892	0.13376	4.85154	1.23E-06	4.74E-05	455.334	436.002	408.369	623.046	682.058	732.636 Up
Mak	2171.9	-0.7147	0.28202	-2.5344	0.01126	0.0675	3160.9	1725.27	3211.4	1834.47	1126.12	1973.25 Down
Elovl2	11.3732	-1.4516	0.70979	-2.0451	0.04084	0.16697	9.79213	17.7557	22.4164	5.09857	7.21209	5.96447 Down
Tmem170	2653.92	0.28692	0.10713	2.67815	0.0074	0.04948	2331.51	2630.81	2210.45	2898.03	2872.47	2980.25 Up
Adtrp	138.418	1.97316	0.3134	6.2959	3.06E-10	2.97E-08	50.9191	56.2265	61.4015	245.751	274.059	142.153 Up
Edn1	3003.08	1.07496	0.28984	3.70879	0.00021	0.0033	1265.14	2265.83	2268.93	5619.65	3738.95	2859.96 Up
Cd83	1521.68	0.40717	0.19489	2.08926	0.03668	0.15579	1307.25	1634.51	983.399	1607.07	1760.78	1837.06 Up
1700029N	12.7396	1.6346	0.62555	2.61307	0.00897	0.05716	6.85449	5.91858	5.84776	21.414	17.5151	18.8875 Up
Gm40932	8.41551	2.44162	1.06528	2.29199	0.02191	0.10849	1.95843	3.94572	1.94925	29.5717	4.12119	8.94671 Up
Stmnd1	1886.1	-0.9753	0.34619	-2.8172	0.00484	0.03644	3394.93	1322.8	2783.54	1411.29	802.602	1601.46 Down
Rbm24	1219.44	-1.1867	0.30087	-3.9442	8.01E-05	0.00154	1985.84	1335.63	1762.13	684.229	453.331	1095.47 Down
Cap2	771.853	-0.5243	0.17409	-3.0118	0.0026	0.02304	1015.44	827.614	888.86	634.263	522.361	742.577 Down
Kif13a	4593.24	0.23512	0.09216	2.5513	0.01073	0.06512	4041.21	4361.01	4257.17	4667.24	5169.01	5063.84 Up
Gm48648	1.95974	-4.3925	2.0519	-2.1407	0.0323	NA	5.87528	2.95929	2.92388	0	0	0 Down
Wnk2	90.103	-1.276	0.35981	-3.5462	0.00039	0.00533	129.256	79.9008	173.484	52.0055	45.3331	60.6388 Down
Susd3	289.165	1.41595	0.35028	4.04234	5.29E-05	0.0011	101.838	225.892	145.219	420.123	563.573	278.342 Up
Fgd3	403.882	0.64102	0.24273	2.64094	0.00827	0.05382	248.72	430.083	268.023	460.911	554.301	461.252 Up
Hist1h2al	148.514	-1.1777	0.37336	-3.1542	0.00161	0.01584	283.972	220.96	113.057	81.5772	75.2118	116.307 Down
Diras2	2987.3	-1.2258	0.51592	-2.3758	0.01751	0.0936	5254.46	2125.76	5175.27	1933.38	1058.12	2376.84 Down
Syk	4167.29	0.90009	0.15438	5.83024	5.53E-09	4.33E-07	2691.86	3498.87	2533.06	4966.01	5750.1	5563.86 Up
BB123696	20.8839	-1.6418	0.79578	-2.0632	0.0391	0.16252	28.3972	18.7422	47.7567	20.3943	2.0606	7.95263 Down
Auh	1908.36	-0.2363	0.11277	-2.0954	0.03613	0.15417	2140.56	2089.26	1962.9	1819.17	1858.66	1579.59 Down
Nfil3	1214.78	-0.8687	0.37137	-2.3391	0.01933	0.09984	1991.72	2080.38	637.406	955.473	915.935	707.784 Down
Gm33424	139.051	-1.9026	0.40845	-4.658	3.19E-06	0.00011	195.843	121.331	341.12	59.1435	44.3028	72.5677 Down
Gm48336	41.2621	-2.4016	0.68263	-3.5182	0.00043	0.00576	51.8983	30.5793	125.727	5.09857	13.3939	20.8756 Down
Ror2	119.94	0.64882	0.26997	2.40331	0.01625	0.08855	96.9421	80.8872	102.336	146.839	180.302	112.331 Up
Gm2762	8.89657	3.31006	1.09769	3.01548	0.00257	0.02286	1.95843	0	2.92388	22.4337	6.18179	19.8816 Up
Tspan17	2353.63	0.28158	0.12768	2.2053	0.02743	0.1268	2099.43	2174.09	2100.32	2533.99	2940.47	2273.46 Up
Unc5a	85.0353	3.51374	0.83861	4.18994	2.79E-05	0.00065	17.6258	3.94572	19.4925	252.889	15.4545	200.804 Up
Hk3	417.277	1.25762	0.2348	5.35626	8.50E-08	4.89E-06	211.51	283.105	243.657	562.883	754.178	448.329 Up
Prr7	180.225	-0.8663	0.2543	-3.4065	0.00066	0.00797	256.554	185.449	256.327	151.938	97.8784	133.207 Down
Dok3	1313.91	0.4801	0.20157	2.3818	0.01723	0.09262	868.562	1330.69	1092.56	1432.7	1831.87	1327.09 Up
Caml	2175.29	-0.2232	0.09521	-2.3446	0.01905	0.09891	2414.74	2213.55	2401.48	2082.26	1928.72	2011.02 Down
Pcbd2	350.693	-0.3573	0.14496	-2.4647	0.01371	0.07806	385.81	381.748	414.217	312.033	282.302	328.046 Down
Pitx1	52.3758	-3.0241	0.58331	-5.1844	2.17E-07	1.08E-05	147.861	37.4843	94.5388	11.2169	8.24239	14.9112 Down
Macroh2a	3049.83	0.24608	0.09218	2.66948	0.0076	0.05043	2933.72	2686.05	2751.37	3311.01	3173.32	3443.49 Up
Tifab	637.473	0.8132	0.26979	3.01425	0.00258	0.02295	333.912	632.301	421.039	745.412	1025.15	667.027 Up
Tgfbi	6224.34	0.69417	0.13659	5.08213	3.73E-07	1.76E-05	5093.86	5241.89	3929.7	7597.9	7802.45	7680.25 Up
Spock1	17.263	-1.7121	0.67733	-2.5277	0.01148	0.06818	16.6466	31.5657	31.1881	4.07886	6.18179	13.9171 Down
Klhl3	316.471	-0.8587	0.24103	-3.5624	0.00037	0.00507	477.856	284.092	461.973	210.061	215.332	249.514 Down
Gm26555	655.867	-0.7432	0.30499	-2.4367	0.01482	0.08274	992.922	562.265	908.353	586.336	299.817	585.512 Down
Ntrk2	2371.36	1.63606	0.23334	7.01132	2.36E-12	3.54E-10	911.647	1069.29	1482.41	4619.31	3084.71	3060.77 Up
Golm1	3878.4	1.35876	0.13549	10.0283	1.14E-23	1.20E-20	2367.74	1842.65	2317.66	5170.97	5692.4	5878.98 Up
Tut7	2625.41	0.43264	0.09827	4.40274	1.07E-05	0.00029	2250.23	2267.8	2186.09	2798.1	3038.35	3211.87 Up
Gm5084	37.8587	-0.9874	0.42638	-2.3158	0.02057	0.10432	50.9191	54.2536	45.8075	15.2957	35.0301	25.846 Down
Gm48500	315.542	-0.7987	0.29752	-2.6846	0.00726	0.04881	434.77	281.132	486.339	266.146	152.484	272.378 Down
Gm7240	94.783	0.6265	0.25762	2.43192	0.01502	0.0835	60.7112	88.7787	74.0717	117.267	119.515	108.355 Up
Gm19792	24.5026	1.26547	0.64097	1.97431	0.04835	0.18679	6.85449	22.6879	13.6448	20.3943	54.6058	28.8283 Up

Gm3604	147.357	1.25827	0.24631	5.10858	3.25E-07	1.55E-05	65.6073	91.738	103.31	185.588	212.241	225.656	Up
6720489N	205.014	0.56594	0.19649	2.88024	0.00397	0.03155	140.027	172.625	183.23	260.027	254.484	219.691	Up
Platr25	522.161	0.37765	0.14425	2.61811	0.00884	0.05652	426.937	489.269	446.379	542.488	656.3	571.595	Up
Gm48795	8.58156	-1.6852	0.84719	-1.9892	0.04668	0.18257	13.709	17.7557	7.79702	6.11829	4.12119	1.98816	Down
Fbp1	637.573	-0.93	0.38106	-2.4405	0.01467	0.08216	576.756	1084.09	847.926	197.825	523.392	595.453	Down
Ptch1	3430.34	0.31363	0.11829	2.65136	0.00802	0.05257	2975.83	3284.81	2916.08	3671.99	4224.22	3509.1	Up
1700024I(	10.2349	-1.4728	0.71755	-2.0525	0.04012	0.1653	13.709	19.7286	11.6955	6.11829	6.18179	3.97631	Down
Cdc14b	838.625	0.28167	0.1359	2.07263	0.03821	0.15999	693.283	812.818	765.082	946.295	822.178	992.09	Up
Ctsl	11977.2	0.25034	0.10031	2.49562	0.01257	0.07299	10903.5	10329.9	11588.3	14006.8	12683	12351.4	Up
Cdk20	434.905	-0.4493	0.17937	-2.505	0.01224	0.07163	499.399	413.314	593.548	374.235	347.211	381.726	Down
Ptdss1	1510.6	0.28632	0.09711	2.94849	0.00319	0.02685	1438.46	1296.17	1348.88	1681.51	1675.27	1623.33	Up
4933433G	145.549	0.83413	0.30912	2.69842	0.00697	0.04736	98.9005	129.222	85.7672	212.101	123.636	223.668	Up
Zfp759	309.868	-0.2889	0.14316	-2.0181	0.04358	0.17438	347.621	355.115	319.678	277.362	276.12	283.312	Down
Rsl1	344.219	0.87044	0.26342	3.30437	0.00095	0.01057	233.053	259.431	237.809	581.237	290.544	463.241	Up
Zfp874a	491.403	0.52179	0.20605	2.53231	0.01133	0.06771	427.916	413.314	369.384	562.883	450.24	724.683	Up
Zfp738	348.045	0.33775	0.15452	2.18581	0.02883	0.13156	286.909	328.481	307.008	407.886	410.059	347.927	Up
1700001L	607.615	-0.9401	0.27149	-3.4627	0.00053	0.00679	857.79	564.238	974.627	485.384	288.484	475.17	Down
Gm40999	153.288	1.01944	0.26657	3.82433	0.00013	0.00232	103.797	100.616	99.412	272.264	160.727	182.91	Up
Clptm1l	8934.5	0.22086	0.09786	2.25691	0.02401	0.11556	8460.4	8596.73	7698.58	9773.97	10065	9012.32	Up
E430024I(	562.673	0.33401	0.1272	2.62579	0.00864	0.05561	482.752	499.133	511.679	583.277	620.24	678.956	Up
Cast	5884.88	0.36292	0.09239	3.9281	8.56E-05	0.00163	5060.57	5016.98	5368.25	6294.7	6520.76	7048.02	Up
Fam81b	1149.38	-0.7991	0.30529	-2.6175	0.00886	0.0566	1664.66	1017.01	1697.8	968.729	489.392	1058.69	Down
Mctp1	1265.64	0.52568	0.22847	2.3009	0.0214	0.10689	770.64	1308.99	1033.1	1839.57	1337.33	1304.23	Up
Slf1	1148.56	-0.3752	0.18027	-2.081	0.03743	0.15772	1341.52	1155.11	1394.69	1030.93	787.148	1181.96	Down
2210408I2	1061.68	-0.648	0.25568	-2.5343	0.01127	0.06751	1419.86	982.484	1486.31	821.89	577.997	1081.56	Down
5430425K	248.408	-1.084	0.29413	-3.6855	0.00023	0.00355	256.554	320.59	435.658	184.568	108.181	184.899	Down
Adgrv1	215.606	-1.3855	0.37285	-3.7159	0.0002	0.00322	281.034	212.082	442.481	148.878	67.9997	141.159	Down
Ccnh	1640.51	0.23007	0.11164	2.06085	0.03932	0.1631	1441.4	1618.73	1469.74	1881.37	1788.6	1643.21	Up
Rasa1	1214.05	0.32983	0.16633	1.98299	0.04737	0.1844	1231.85	965.715	1030.18	1219.58	1241.51	1595.5	Up
Edil3	756.085	-0.3643	0.17942	-2.0303	0.04232	0.17098	847.998	1024.9	680.29	624.066	660.421	698.837	Down
Ssbp2	1469.75	-0.2358	0.11287	-2.089	0.0367	0.1558	1624.51	1482.6	1661.74	1370.5	1246.66	1432.47	Down
Ckmt2	229.058	-2.4023	0.9682	-2.4812	0.01309	0.07538	654.114	57.2129	444.43	58.1237	40.1816	120.284	Down
Fam151b	751.293	-0.3963	0.17296	-2.2915	0.02193	0.10854	968.441	705.297	887.885	652.618	571.816	721.701	Down
Cmya5	212.637	-1.2145	0.33856	-3.5873	0.00033	0.00475	363.288	169.666	358.663	158.056	98.9087	127.242	Down
Tent2	3305.5	0.2104	0.09218	2.28244	0.02246	0.11021	2944.49	3253.24	2996.98	3679.13	3495.8	3463.37	Up
Lhfpl2	1163.82	0.97917	0.31371	3.12129	0.0018	0.01727	854.853	1074.22	421.039	1400.07	1922.54	1310.2	Up
Scamp1	8488.16	0.2885	0.11385	2.53393	0.01128	0.06751	7484.12	8382.68	7060.2	8698.17	9268.56	10035.2	Up
Gm32089	2.01556	4.48385	2.15109	2.08446	0.03712	NA	0	0	0	5.09857	1.0303	5.96447	Up
Sv2c	59.6218	-1.3979	0.62384	-2.2408	0.02504	0.11894	143.944	30.5793	84.7926	14.276	46.3634	37.775	Down
Col4a3bp	5757.73	0.26214	0.09083	2.88621	0.0039	0.03114	4976.36	5388.86	5342.91	6631.21	6157.06	6049.96	Up
Hmgcr	5689.44	0.53572	0.1237	4.33077	1.49E-05	0.00038	4647.34	5185.66	4102.21	6593.48	7182.21	6425.72	Up
Gcnt4	67.9465	-1.0812	0.46018	-2.3494	0.0188	0.09807	105.755	42.4165	128.651	33.6506	50.4846	46.7217	Down
Hexb	2874.24	0.68054	0.13514	5.03591	4.76E-07	2.14E-05	2223.79	2346.72	2055.49	3048.95	3607.07	3963.39	Up
Gm5086	46.0536	0.72903	0.37066	1.96683	0.0492	0.18905	30.3556	46.3622	27.2896	49.966	58.727	63.621	Up
Gm34388	8.22344	-1.7773	0.83314	-2.1333	0.0329	0.14419	8.81291	12.8236	16.5687	5.09857	2.0606	3.97631	Down
Arhgef28	4596.49	-0.4281	0.15658	-2.7338	0.00626	0.0441	5910.53	4383.69	5526.14	4349.08	3440.17	3969.36	Down
Gm21976	111.438	-3.0518	0.73187	-4.1698	3.05E-05	0.0007	397.56	24.6607	174.458	28.552	18.5454	24.852	Down
A930014D	15.2455	1.69649	0.62071	2.73313	0.00627	0.04413	3.91685	9.8643	7.79702	24.4732	19.5757	25.846	Up
Map1b	4946.63	-0.4897	0.2459	-1.9917	0.04641	0.18182	6185.69	4189.37	6959.81	4600.95	2886.9	4857.07	Down
BC001981	6.23263	-2.6624	1.24133	-2.1448	0.03197	NA	16.6466	2.95929	12.6702	3.05914	2.0606	0	Down
Smn1	1051.87	-0.2147	0.09854	-2.1787	0.02936	0.13306	1087.91	1135.38	1166.63	985.045	983.935	952.327	Down
Naip2	1640.43	0.883	0.09745	9.06133	1.29E-19	6.69E-17	1217.16	1125.52	1117.9	2045.55	2186.29	2150.19	Up
Naip5	1643.59	0.72673	0.17924	4.05462	5.02E-05	0.00105	1251.43	1075.21	1387.87	2308.63	1618.6	2219.78	Up
Naip6	447.309	0.76526	0.19183	3.98919	6.63E-05	0.00132	255.575	393.585	345.018	586.336	534.725	568.613	Up
Naip1	23.3866	2.16879	0.65243	3.32415	0.00089	0.00997	2.93764	15.7829	6.82239	29.5717	48.424	36.7809	Up
Rnf180	553.265	0.88264	0.15498	5.6953	1.23E-08	8.83E-07	430.854	371.884	364.511	691.367	810.845	650.127	Up
Gm30411	10.9898	-2.3001	0.76408	-3.0102	0.00261	0.0231	20.5635	12.8236	21.4418	4.07886	2.0606	4.97039	Down
Ipo11	471.567	0.26568	0.13409	1.98128	0.04756	0.18495	439.667	421.205	423.963	466.01	536.785	541.773	Up
Zswim6	1751.19	0.20986	0.10612	1.9776	0.04797	0.18606	1696.98	1628.6	1546.73	1806.93	1801.99	2025.93	Up
Ndufaf2	680.313	-0.4661	0.1488	-3.1324	0.00173	0.01679	819.601	696.419	851.824	605.711	506.907	601.418	Down
Elovl7	625.747	0.39888	0.16395	2.43298	0.01498	0.08338	610.05	447.839	561.385	694.426	773.754	667.027	Up
Pde4d	1793.91	-0.5301	0.1064	-4.982	6.29E-07	2.71E-05	2198.33	1999.49	2161.72	1381.71	1448.6	1573.63	Down
Gapt	292.78	3.38899	0.41609	8.14487	3.80E-16	1.24E-13	42.1061	71.0229	39.9597	461.931	861.33	280.33	Up
Plk2	4541.03	0.57369	0.25168	2.27946	0.02264	0.11068	2499.93	5187.63	3262.08	5449.36	6074.64	4772.57	Up
Gm15287	3.20377	4.11446	1.67967	2.44956	0.0143	NA	0.97921	0	0	8.15772	4.12119	5.96447	Up
Il6st	15996	0.27426	0.09841	2.7868	0.00532	0.03912	14449.3	15292.6	13698.4	16234.9	18346.5	17954.1	Up
Gzmk	25.5256	-1.7363	0.7301	-2.3782	0.0174	0.09324	15.6674	67.0772	35.0866	15.2957	4.12119	15.9053	Down
BC067074	831.316	-0.5282	0.20728	-2.5481	0.01083	0.06552	799.038	1252.77	893.733	602.652	658.361	781.346	Down
Ccl28	408.159	1.77682	0.35081	5.06491	4.09E-07	1.88E-05	283.972	102.589	166.661	529.232	555.331	811.168	Up
Dnase1l3	239.728	0.89807	0.36225	2.47913	0.01317	0.07567	243.824	96.6701	161.788	212.101	376.059	347.927	Up
Acox2	2001.17	-1.036	0.34572	-2.9967	0.00273	0.02389	2295.27	2047.83	3727.95	1805.92	670.724	1459.31	Down
Fhit	371.719	-0.9131	0.24223	-3.7695	0.00016	0.00273	506.253	338.345	612.066	278.382	260.666	234.603	Down
Synpr	419.19	-1.5311	0.37703	-4.0608	4.89E-05	0.00103	343.704	857.207	667.62	318.151	181.333	147.124	Down
Sntn	3114.52	-1.009	0.32084	-3.145	0.00166	0.01624	5627.54	2449.3	4407.26	2009.86	1388.84	2804.3	Down
Il3ra	1077.73	0.52151	0.1895	2.75199	0.00592	0.04235	1014.46	840.438	800.169	1014.62	1537.21	1259.5	Up
Slc4a7	1132.64	0.48293	0.19767	2.44309	0.01456	0.08178	1028.17	924.285	882.038	963.631	1515.57	1482.17	Up
Nek10	908.265	-1.3236	0.34332	-3.8554	0.00012	0.00209	1549.11	725.026	1619.83	520.055	331.756	703.808	Down
Rarb	465.629	-0.8201	0.25595	-3.2043	0.00135	0.0139	539.546	618.491	625.711	278.382	254.484	477.158	Down
Ube2e2	1473.94	-0.4226	0.12515	-3.3769	0.00073	0.00861	1518.76	1757.82	1788.44	1277.7	1153.93	1346.98	Down
Gm2237	56.0474	-0.7838	0.38714	-2.0245	0.04292	0.17249	48.9606	86.8058	76.9956	48.9463	28.8484	45.7276	Down
Rtraf	4534.72	-0.2471	0.09222	-2.6792	0.00738	0.04942	4637.55	5005.14	5123.62	4343.99	4023.32	4074.73	Down
Kcnk5	521.753	-0.739	0.16741	-4.4143	1.01E-05	0.00028	657.052	700.365	600.37	404.827	324.544	443.359	Down
Cfap70	1878.3	-0.978	0.28344	-3.4503	0.00056	0.00703	2901.41	1678.9	2894.64	1445.96	817.027	1531.88	Down

Usp54	249.273	0.5175	0.22553	2.29457	0.02176	0.10798	228.157	170.652	216.367	289.599	236.969	353.892	Up
Synpo2l	45.7033	-1.9514	0.81877	-2.3834	0.01715	0.09232	94.0044	11.8372	112.082	16.3154	6.18179	33.7987	Down
Sec24c	420.296	0.6593	0.21387	3.08266	0.00205	0.01916	398.54	267.322	311.881	457.852	470.846	615.335	Up
Vcl	2890.46	0.48024	0.14938	3.21493	0.0013	0.0135	2743.75	2340.8	2156.85	2940.86	3370.11	3790.42	Up
Ap3m1	4220.22	0.25796	0.08403	3.07	0.00214	0.01988	3706.32	4002.93	3822.49	4513.26	4738.34	4537.97	Up
Dusp13	28.7965	0.99137	0.44681	2.21875	0.0265	0.12393	21.5427	15.7829	20.4672	40.7886	46.3634	27.8342	Up
Samd8	2297.37	0.22519	0.10284	2.18972	0.02854	0.1306	2098.45	2248.07	2008.71	2523.79	2585.02	2320.18	Up
Vdac2	14173.8	0.20433	0.09596	2.12939	0.03322	0.14535	13013.7	12890.7	13610.7	15611.8	16007.7	13908.2	Up
A430057N	28.4315	1.31453	0.56103	2.34307	0.01913	0.09924	8.81291	10.8507	29.2388	37.7295	41.2119	42.7454	Up
Zfp503	2600.18	0.51536	0.23264	2.21524	0.02674	0.12472	1687.18	2188.89	2545.73	3003.06	3930.59	2245.62	Up
Kcnma1	52.749	-0.8715	0.39642	-2.1984	0.02792	0.12855	70.5033	46.3622	87.7165	35.69	47.3937	28.8283	Down
Dennd6a	513.395	0.36213	0.1764	2.05293	0.04008	0.16523	455.334	397.531	495.111	532.291	516.18	683.926	Up
Dnah12	2966.59	-0.8344	0.27044	-3.0853	0.00203	0.01902	4474.02	2393.08	4536.89	2184.23	1594.9	2616.41	Down
Asb14	925.12	-0.6437	0.26285	-2.4488	0.01433	0.08084	1173.1	811.832	1399.56	804.555	502.786	858.884	Down
Wnt5a	961.548	-0.4501	0.13892	-3.2401	0.00119	0.01268	1014.46	1048.57	1267.99	757.648	837.633	842.979	Down
Cacna2d3	107.321	-0.8534	0.23092	-3.6958	0.00022	0.00343	145.903	126.263	142.296	71.38	85.5148	72.5677	Down
Prkcd	6748.25	0.50901	0.1168	4.358	1.31E-05	0.00034	5323.98	6136.58	5249.34	8192.39	8372.21	7215.02	Up
Mustn1	1445.07	0.56732	0.17475	3.24637	0.00117	0.01247	1074.2	1420.46	998.993	1766.15	1893.69	1516.96	Up
Smim4	276.784	-0.3963	0.1985	-1.9967	0.04586	0.18031	385.81	279.16	278.743	225.357	242.12	249.514	Down
Dnah1	1722.64	-1.263	0.2633	-4.797	1.61E-06	5.94E-05	2640.94	1650.3	3004.78	1073.76	729.451	1236.63	Down
Capn7	416.24	0.65738	0.19324	3.40184	0.00067	0.00804	363.288	294.942	310.906	474.167	435.816	618.317	Up
Colq	785.491	-1.0447	0.26856	-3.8901	0.0001	0.00187	950.816	1074.22	1149.09	658.736	574.907	305.182	Down
Oxnad1	652.358	-0.3357	0.16538	-2.0296	0.04239	0.17118	752.035	659.921	771.905	554.725	496.604	678.956	Down
Ncoa4	1660.04	0.7328	0.15935	4.59865	4.25E-06	0.00014	1424.75	1165.96	1151.03	1881.37	2430.47	1906.64	Up
1700024G	1085.38	-1.4442	0.29599	-4.8793	1.06E-06	4.20E-05	1799.79	1030.82	1931.71	624.066	384.301	741.583	Down
Tmem273	370.003	1.10514	0.26165	4.22374	2.40E-05	0.00057	207.593	313.685	183.23	441.537	632.603	441.371	Up
Wdfy4	2121.78	0.7551	0.15615	4.83568	1.33E-06	5.10E-05	1493.3	1675.94	1567.2	2454.45	3216.59	2323.16	Up
Frmpd2	1919.48	-0.7183	0.32958	-2.1795	0.0293	0.13288	2823.07	1471.75	2868.33	1718.22	825.269	1810.22	Down
Ptpn20	224.242	-0.9588	0.4029	-2.3796	0.01733	0.09301	327.057	164.734	396.673	121.346	103.03	232.614	Down
Glud1	12686.4	0.21396	0.09654	2.21633	0.02667	0.12452	11228.6	12196.2	11817.4	13520.4	14619.9	12736.1	Up
Sncg	952.344	-1.0229	0.21439	-4.7712	1.83E-06	6.65E-05	1206.39	1008.13	1614.96	725.017	522.361	637.204	Down
Mmrn2	1858.23	0.43266	0.14893	2.90519	0.00367	0.02987	1376.77	1782.48	1585.72	1926.24	2399.57	2078.62	Up
Ldb3	322.546	-1.8255	0.88325	-2.0667	0.03876	0.16172	763.786	84.8329	660.797	127.464	89.636	208.756	Down
Ccser2	6349.51	0.30503	0.08887	3.43231	0.0006	0.00742	5386.65	6038.92	5616.78	6836.17	7104.94	7113.63	Up
Sh2d4b	125.284	0.54372	0.26912	2.02036	0.04335	0.17367	127.298	101.602	76.9956	156.016	127.757	162.035	Up
Prxl2a	3347.49	-0.4391	0.1865	-2.3546	0.01854	0.09728	4497.52	2978.03	4083.69	3065.26	2402.66	3057.79	Down
Dydc2	248.474	-1.0445	0.36142	-2.8899	0.00385	0.03093	384.831	194.327	424.937	200.884	100.969	184.899	Down
Dydc1	158.187	-1.2801	0.4029	-3.1771	0.00149	0.01492	256.554	132.182	283.617	104.011	50.4846	122.272	Down
Sftpd	108973	0.8533	0.14244	5.99044	2.09E-09	1.74E-07	73464.5	92846.7	66650.9	140627	140268	139982	Up
Gm8113	114.04	0.69925	0.25229	2.77155	0.00558	0.04049	67.5657	101.602	91.615	144.8	150.424	128.236	Up
Gm32857	42.7431	1.72121	0.6074	2.83374	0.0046	0.03521	7.8337	14.7964	37.0358	94.8335	44.3028	57.6566	Up
Gm5930	25.1381	1.36816	0.67289	2.03326	0.04203	0.17024	3.91685	14.7964	23.3911	52.0055	29.8787	26.8401	Up
Gm8229	33.1988	1.77671	0.70852	2.50764	0.01215	0.07127	2.93764	11.8372	30.2134	67.3012	40.1816	46.7217	Up
Ero1l	2922.63	0.33006	0.09484	3.48027	0.0005	0.00645	2586.1	2690.98	2492.12	3330.39	3380.41	3055.8	Up
Gm15601	130.177	0.48483	0.21364	2.26943	0.02324	0.11292	99.8797	125.277	100.387	148.878	154.545	152.094	Up
Ddhd1	2827.66	0.85868	0.16001	5.36639	8.03E-08	4.65E-06	2104.33	2314.16	1612.03	3385.45	3929.56	3620.43	Up
Ubb-ps	823.978	0.70137	0.22458	3.12305	0.00179	0.01719	561.089	638.22	683.214	933.039	1356.9	771.405	Up
Bmp4	3378.24	0.48848	0.23279	2.09835	0.03587	0.15334	2192.46	3433.76	2808.88	3893.27	5016.52	2924.58	Up
Gch1	3705.12	0.56306	0.10882	5.17426	2.29E-07	1.13E-05	3052.21	3208.86	2712.39	4221.62	4650.77	4384.88	Up
Lgals3	14063.1	0.37447	0.1541	2.43002	0.0151	0.08377	12824.7	11506.7	12412.9	16301.2	18520.6	12812.7	Up
Dlgap5	112.385	0.47437	0.23595	2.01051	0.04438	0.17627	101.838	91.738	88.6911	113.188	127.757	151.1	Up
Rpl19-ps3	21.6293	-1.3982	0.67129	-2.0828	0.03727	0.15722	50.9191	19.7286	23.3911	10.1971	19.5757	5.96447	Down
Tep1	2491.76	0.45786	0.09071	5.04765	4.47E-07	2.03E-05	2145.46	2081.37	2072.06	2755.27	3040.41	2855.99	Up
Rnase4	46958.7	0.61703	0.14121	4.36953	1.25E-05	0.00033	36338.6	41100.6	33762.1	62050.7	60311.6	48189	Up
Rnase6	551.49	0.42147	0.13115	3.21357	0.00131	0.01355	432.812	508.998	472.694	604.691	662.482	627.264	Up
Ear6	170.065	3.73121	0.4239	8.802	1.34E-18	6.05E-16	23.5011	12.8236	35.0866	304.895	450.24	193.845	Up
Slc39a2	989.793	2.51936	0.27292	9.2311	2.68E-20	1.63E-17	363.288	258.445	260.225	1662.14	2287.26	1107.4	Up
Gm49256	11.8694	-1.748	0.8486	-2.0598	0.03942	0.16343	10.7713	17.7557	26.3149	7.138	8.24239	0.99408	Down
Rpgrip1	407.781	0.62748	0.13825	4.53869	5.66E-06	0.00017	308.452	328.481	324.551	459.891	492.483	532.826	Up
Supt16	1031.63	0.37892	0.1724	2.19788	0.02796	0.12865	900.876	780.266	1009.71	1007.48	1111.69	1379.78	Up
Gm30275	63.673	1.01568	0.44485	2.28319	0.02242	0.11011	30.3556	49.3215	46.7821	46.9069	82.4239	126.248	Up
Gm43305	32.5772	3.25248	1.00812	3.22627	0.00125	0.01314	5.87528	0	12.6702	19.3746	126.727	30.8164	Up
Rem2	166.783	-0.8717	0.23875	-3.651	0.00026	0.00394	214.448	227.865	204.672	147.859	115.393	90.4611	Down
Prmt5	2042.29	-0.2046	0.08776	-2.3318	0.01971	0.10116	2156.23	2128.72	2275.75	1874.24	1881.32	1937.46	Down
Acin1	1168.45	0.40424	0.15605	2.59044	0.00959	0.05987	1128.05	868.058	1021.41	1253.23	1223.99	1515.97	Up
Slc7a8	352.088	0.87449	0.25914	3.37459	0.00074	0.00865	227.177	316.644	201.748	402.787	591.391	372.779	Up
Slc22a17	1275.92	-0.307	0.10584	-2.9005	0.00373	0.03016	1352.29	1472.74	1408.34	1140.04	1207.51	1074.6	Down
Efs	239.022	-0.3548	0.18003	-1.971	0.04872	0.18768	295.722	269.295	239.758	231.475	205.029	192.851	Down
Myh7	67.5339	-0.9667	0.49259	-1.9626	0.0497	0.19018	103.797	48.3351	115.981	33.6506	29.8787	73.5618	Down
Zfhx2os	18.765	-1.4343	0.62745	-2.2859	0.02226	0.10965	31.3348	13.81	37.0358	13.2563	7.21209	9.94079	Down
Ap1g2	1486.29	0.29913	0.11927	2.50807	0.01214	0.07121	1373.84	1353.38	1270.91	1736.57	1724.72	1458.31	Up
Rec8	1065.33	-1.2624	0.2552	-4.9467	7.55E-07	3.15E-05	1335.65	1816.02	1359.61	558.804	870.602	451.312	Down
Ipo4	1851.27	-0.2894	0.11356	-2.548	0.01084	0.06552	2082.79	2101.1	1924.89	1777.36	1716.48	1505.03	Down
Cideb	169.148	1.09594	0.1985	5.52113	3.37E-08	2.11E-06	104.776	110.48	108.184	210.061	261.696	219.691	Up
Ltb4r2	16.6777	1.69972	0.66752	2.54632	0.01089	0.06574	7.8337	9.8643	5.84776	30.5914	33.9998	11.9289	Up
Ripk3	698.69	0.89411	0.14381	6.21711	5.06E-10	4.70E-08	518.004	506.038	442.481	862.679	1018.97	843.973	Up
Nfatc4	638.548	0.35207	0.17923	1.96434	0.04949	0.18965	518.004	590.871	574.055	692.386	866.481	589.489	Up
Khnyn	1713.84	0.32259	0.10673	3.02267	0.00251	0.02247	1536.38	1444.13	1588.64	1842.62	1818.48	2052.77	Up
Mcpt4	16.4227	-2.5658	1.09448	-2.3443	0.01906	0.09898	65.6073	13.81	4.87314	10.1971	2.0606	1.98816	Down
Gm4032	127.415	-0.7057	0.26565	-2.6566	0.00789	0.05196	161.57	125.277	187.128	90.7546	83.4542	116.307	Down
Gm8983	13.6794	2.19274	0.80003	2.74082	0.00613	0.04344	3.91685	7.89144	2.92388	14.276	39.1513	13.9171	Up
Gja3	8.51066	-2.4177	1.1382	-2.1241	0.03366	0.14653	20.5635	4.93215	17.5433	0	2.0606	5.96447	Down
Ift88	3259.47	-0.7965	0.22581	-3.5274	0.00042	0.00563	4711.97	3085.55	4613.89	2542.15	1772.11	2831.14	Down

Mrpl57	1035.11	-0.3452	0.12729	-2.7117	0.00669	0.04609	1220.1	1099.87	1154.93	992.183	932.42	811.168	Down
3110083C	100.69	-0.6037	0.29411	-2.0525	0.04012	0.1653	143.944	100.616	119.879	75.4589	62.8482	101.396	Down
Rcbtb1	2749.33	0.24002	0.09187	2.6126	0.00899	0.05722	2499.93	2385.19	2678.28	2951.05	3002.29	2979.25	Up
Atp8a2	83.0091	-0.5511	0.26041	-2.1162	0.03433	0.14852	112.609	93.7108	89.6657	72.3998	69.03	60.6388	Down
Gm29266	177.986	-1.1651	0.21824	-5.3388	9.36E-08	5.25E-06	235.99	221.947	280.693	120.326	90.6663	118.295	Down
Spata13	1978.5	0.23746	0.11761	2.01905	0.04348	0.1741	1818.4	1644.38	1985.32	2007.82	2217.2	2197.91	Up
Mipep	1727.23	-0.397	0.17863	-2.2222	0.02627	0.12325	2202.25	1538.83	2149.05	1541.81	1297.15	1634.27	Down
Ebpl	737.198	-0.3468	0.17054	-2.0334	0.04201	0.17024	882.271	670.772	922.972	711.761	643.937	591.477	Down
Dleu2	2110.54	-0.3573	0.15509	-2.3038	0.02123	0.10646	2610.58	1988.64	2512.59	1812.03	1659.81	2079.61	Down
Ctsb	46787.8	0.57074	0.09154	6.23455	4.53E-10	4.26E-08	37627.2	39789.6	35538.8	55006.5	58885.7	53879.1	Up
Fdft1	5771.06	0.37521	0.1115	3.36518	0.00076	0.0089	5041.97	5253.72	4778.6	6744.39	6984.39	5823.31	Up
Neil2	144.324	0.45331	0.21339	2.12432	0.03364	0.14653	113.589	125.277	126.702	171.312	142.181	186.887	Up
Sox7	4042.59	0.53373	0.16581	3.21889	0.00129	0.01339	2576.31	3437.71	3895.59	4953.77	4810.46	4581.71	Up
Fzd3	1269.22	-0.3077	0.14958	-2.0571	0.03967	0.16424	1486.44	1376.07	1349.86	1049.29	1008.66	1344.99	Down
Fbxo16	604.72	-0.8368	0.22246	-3.7617	0.00017	0.0028	903.813	593.831	828.433	508.838	343.089	450.318	Down
Scara5	391.811	-1.3212	0.21328	-6.1947	5.84E-10	5.39E-08	685.449	440.934	552.614	236.574	194.726	240.567	Down
Scara3	368.024	-1.2806	0.29065	-4.406	1.05E-05	0.00029	732.451	451.785	380.105	232.495	162.787	248.52	Down
Ptk2b	2006.71	0.82765	0.18456	4.48444	7.31E-06	0.00022	1287.66	1700.6	1350.83	2618.63	3020.84	2061.72	Up
Adra1a	865.282	-0.7225	0.30767	-2.3483	0.01886	0.09825	969.421	1549.68	713.427	743.372	764.481	451.312	Down
Dock5	2091.13	0.41081	0.11034	3.72303	0.0002	0.00315	1853.65	1656.22	1876.16	2415.7	2525.26	2219.78	Up
Adamdec1	1432.68	-0.6367	0.26606	-2.3933	0.0167	0.09047	1761.6	1157.08	2312.79	1290.96	848.966	1224.7	Down
Stc1	162.784	0.39149	0.18547	2.11078	0.03479	0.14987	134.152	148.951	139.372	193.746	187.514	172.97	Up
Sorbs3	11982.4	-0.3468	0.13869	-2.5007	0.01239	0.07223	15148.4	11187.1	13911.8	11056.8	10007.3	10583	Down
Gm49417	323.35	-0.5218	0.16635	-3.1366	0.00171	0.01663	413.228	363.993	366.46	278.382	229.757	288.283	Down
Fndc3a	5321.52	0.37278	0.09549	3.904	9.46E-05	0.00178	4901.94	4579.01	4432.6	5807.28	5826.34	6381.98	Up
Rb1	396.73	0.49027	0.1738	2.82083	0.00479	0.03613	396.581	306.78	286.54	460.911	455.392	474.175	Up
Nudt15	126.661	0.47029	0.20215	2.3264	0.02	0.10219	103.797	113.439	101.361	146.839	149.393	145.135	Up
Lcp1	17510.7	0.98598	0.14103	6.99155	2.72E-12	3.94E-10	11061.2	12958.7	11228.7	20251.5	26873.3	22690.8	Up
Gm4285	134.424	-0.4923	0.24353	-2.0216	0.04322	0.1734	154.716	166.707	150.093	89.7349	106.121	139.171	Down
Tsc22d1	20811.7	-0.3866	0.15623	-2.4746	0.01334	0.07636	23707.7	19295.5	27747.6	19732.5	17237.9	17148.8	Down
Serp2	60.3939	-0.7002	0.32431	-2.1591	0.03085	0.13755	70.5033	63.1315	90.6403	38.7492	54.6058	44.7335	Down
Lacc1	1026.11	0.51704	0.14182	3.64585	0.00027	0.00399	779.453	882.855	870.342	1052.35	1215.75	1355.92	Up
Epsti1	2129.32	0.41666	0.13724	3.03607	0.0024	0.02168	1784.13	1609.85	2077.91	2523.79	2225.44	2554.78	Up
Gm19301	736.444	-0.9578	0.2959	-3.2368	0.00121	0.01279	1121.2	693.46	1102.3	627.125	304.968	569.607	Down
Tnfsf11	29.554	0.93996	0.4108	2.2881	0.02213	0.10921	17.6258	21.7015	21.4418	42.828	32.9696	40.7572	Up
Olfm4	241.69	3.66797	0.4594	7.98425	1.41E-15	4.31E-13	52.8775	20.715	32.1627	701.564	216.363	426.46	Up
Diaph3	536.72	-0.4762	0.23915	-1.9913	0.04644	0.18192	438.687	830.574	604.269	431.339	478.058	437.395	Down
Rps3a2	6698.47	-0.7562	0.22633	-3.3412	0.00083	0.00952	9536.55	7692.18	8016.31	3944.26	4101.62	6899.9	Down
Gm33203	3.19448	4.11063	1.71247	2.40042	0.01638	NA	0.97921	0	0	3.05914	6.18179	8.94671	Up
Pcdh20	40.4978	-1.3983	0.49513	-2.8242	0.00474	0.03591	83.2331	29.5929	63.3508	20.3943	19.5757	26.8401	Down
Pibf1	1262.49	-0.3994	0.14375	-2.7787	0.00546	0.03983	1527.57	1255.72	1525.29	1046.23	999.389	1220.73	Down
Gm41230	90.4795	-2.5297	0.29637	-8.5356	1.39E-17	5.73E-15	169.404	161.774	131.575	31.6112	22.6666	25.846	Down
Gm49225	26.7937	-1.5746	0.56927	-2.766	0.00567	0.04104	45.0438	25.6472	49.706	15.2957	6.18179	18.8875	Down
Lmo7	26301.5	0.30239	0.12557	2.40823	0.01603	0.08767	21697.4	21652.1	27315.9	29820.5	29172.9	28150.3	Up
Kctd12	5845.64	0.26999	0.09478	2.8486	0.00439	0.03395	5002.8	5675.92	5222.05	6149.9	6610.39	6412.8	Up
Mycbp2	4012.22	0.23115	0.08087	2.8582	0.00426	0.03325	3636.8	3766.19	3671.42	4209.38	4493.13	4296.41	Up
Gm34907	350.114	0.78513	0.21299	3.6862	0.00023	0.00354	294.743	243.648	232.936	382.393	554.301	392.661	Up
Slain1	617.751	-0.7399	0.12172	-6.0786	1.21E-09	1.06E-07	726.576	825.642	766.057	459.891	477.028	451.312	Down
Ednrb	3095.05	-0.5456	0.17048	-3.2006	0.00137	0.01402	3466.41	4537.58	3016.47	2333.11	2624.17	2592.56	Down
D130009I1	372.033	-0.4673	0.18264	-2.5584	0.01052	0.06427	386.789	498.147	410.318	359.959	294.665	282.318	Down
Gm6201	43.186	-0.9907	0.46117	-2.1483	0.03169	0.14018	60.7112	53.2672	58.4776	23.4534	16.4848	46.7217	Down
9330188P	76.1229	-2.4614	0.54566	-4.5109	6.46E-06	0.00019	148.84	151.91	85.7672	7.138	41.2119	21.8697	Down
Slitrk6	251.024	-1.2056	0.41734	-2.8889	0.00387	0.03099	550.318	353.142	147.169	124.405	172.06	159.053	Down
Sox21	276.576	-1.7572	0.52543	-3.3444	0.00082	0.00943	682.511	141.059	457.1	153.977	60.7876	164.023	Down
Cldn10	1749.01	-0.8989	0.29889	-3.0073	0.00264	0.02328	2343.26	1900.85	2586.66	1652.96	659.391	1350.95	Down
Dzip1	922.792	-0.9894	0.28231	-3.5048	0.00046	0.00601	1299.42	884.827	1498	643.44	403.877	807.192	Down
Hs6st3	102.476	-0.8524	0.27798	-3.0665	0.00217	0.02005	154.716	100.616	140.346	83.6166	66.9694	68.5914	Down
Gm4681	14.5876	-2.2552	0.75205	-2.9987	0.00271	0.02379	27.418	11.8372	33.1373	3.05914	4.12119	7.95263	Down
Gm49008	1.96018	-4.3928	2.01383	-2.1813	0.02916	NA	3.91685	3.94572	3.89851	0	0	0	Down
Stk24	3687.96	0.26649	0.13027	2.04572	0.04078	0.16679	3401.79	3668.53	2974.56	3831.07	4467.37	3784.46	Up
Slc15a1	216.676	0.4454	0.19927	2.23513	0.02541	0.12018	179.196	155.856	215.393	227.396	263.756	258.46	Up
1810041H	46.0222	-1.1909	0.33946	-3.5083	0.00045	0.00595	57.7736	63.1315	71.1478	26.5126	25.7575	31.8105	Down
Timm8a2	4.57555	-4.5995	1.58423	-2.9033	0.00369	NA	12.7298	4.93215	8.77165	1.01971	0	0	Down
Gm5089	6.69468	-3.1608	1.29857	-2.4341	0.01493	NA	6.85449	3.94572	25.3403	2.03943	0	1.98816	Down
Zic5	62.3413	-1.9073	0.5331	-3.5778	0.00035	0.00486	93.0252	50.3079	152.042	16.3154	20.606	41.7513	Down
Pcca	1547.63	-0.3002	0.09602	-3.1261	0.00177	0.01705	1755.73	1730.2	1638.35	1394.97	1329.08	1437.44	Down
Ggact	813.832	-0.2095	0.10679	-1.962	0.04977	0.19041	850.936	855.234	912.251	781.102	743.875	739.594	Down
C6	12157.3	0.37864	0.1761	2.15019	0.03154	0.13968	9617.83	13166.9	8928.56	12011.2	14520	14699.4	Up
Ptger4	1029.93	0.46251	0.15306	3.02175	0.00251	0.0225	762.807	862.139	973.653	1222.64	1312.6	1045.77	Up
5430437J1	141.054	1.21609	0.2774	4.38393	1.17E-05	0.00031	60.7112	82.8601	111.108	193.746	177.211	220.685	Up
Dab2	6306.67	1.0056	0.1392	7.22394	5.05E-13	8.85E-11	4212.57	4807.86	3560.31	8181.17	9016.14	8061.98	Up
Osmr	5640.6	0.42655	0.1806	2.36183	0.01819	0.09611	5032.17	5389.85	4016.44	5656.36	5816.03	7932.75	Up
Cplane1	1780.27	-0.3039	0.13483	-2.2541	0.02419	0.11619	2037.74	1727.24	2136.38	1501.02	1520.72	1758.52	Down
Spef2	2968.81	-0.7532	0.26477	-2.8448	0.00444	0.03423	4380.02	2534.14	4265.94	2367.78	1537.21	2727.75	Down
4930556M	1440.26	-0.4992	0.13378	-3.7313	0.00019	0.00307	1685.23	1853.5	1522.37	1103.33	1157.03	1320.14	Down
Gm19276	8.89774	-2.2798	1.05578	-2.1593	0.03082	0.13751	6.85449	32.5522	4.87314	2.03943	3.0909	3.97631	Down
Npr3	4108.31	-1.2571	0.28063	-4.4794	7.48E-06	0.00022	8530.9	3761.26	5086.58	1992.52	2828.17	2450.4	Down
Gm34759	5.40315	-2.8126	1.34623	-2.0893	0.03668	NA	19.5843	2.95929	5.84776	2.03943	0	1.98816	Down
4930445E	29.4649	-3.1207	0.66994	-4.6582	3.19E-06	0.00011	86.1707	23.6743	48.7314	4.07886	6.18179	7.95263	Down
Basp1	3704.94	-1.6216	0.35112	-4.6184	3.87E-06	0.00012	8632.74	2832.04	5312.69	1929.3	1297.15	2225.74	Down
Otulinl	2164.96	0.35406	0.12694	2.78919	0.00528	0.03894	1694.04	1960.04	2047.69	2533.99	2554.11	2199.9	Up
Dnah5	5581.39	-1.3834	0.51078	-2.7084	0.00676	0.04638	10651.9	4303.79	9253.11	2879.67	1810.23	4589.66	Down
Ctnnd2	556.463	-0.4188	0.14842	-2.822	0.00477	0.03605	638.447	708.256	563.335	443.576	509.998	475.17	Down

Dap	6410.98	0.45478	0.14085	3.22871	0.00124	0.01308	5017.49	5750.88	5457.91	7523.46	8449.48	6266.67	Up
Ropn1l	292.029	-0.7458	0.24309	-3.0681	0.00215	0.01996	319.223	301.847	476.593	238.613	180.302	235.597	Down
Cmbl	2203.29	-0.7403	0.27989	-2.645	0.00817	0.05331	3311.7	1707.51	3250.38	1891.57	1181.75	1876.82	Down
Gm32618	190.265	-0.4022	0.17909	-2.2456	0.02473	0.11779	222.281	223.92	203.697	149.898	164.848	176.946	Down
Tspyl5	215.808	-0.8293	0.29607	-2.801	0.0051	0.03792	297.681	224.906	306.033	182.529	95.8178	187.881	Down
Erich5	31.0054	-2.0883	0.66992	-3.1173	0.00183	0.01743	77.3578	14.7964	58.4776	11.2169	9.27269	14.9112	Down
Rida	1144.71	-0.3572	0.10716	-3.3331	0.00086	0.00974	1255.35	1326.75	1274.81	1017.68	1061.21	932.446	Down
Nipal2	3437	-0.2208	0.0962	-2.2948	0.02175	0.10795	3778.78	3608.36	3711.38	3204.96	2945.62	3372.91	Down
Vps13b	1275.53	0.30222	0.11852	2.54988	0.01078	0.06532	1208.35	1125.52	1093.53	1365.4	1312.6	1547.78	Up
Rgs22	1596.01	-0.7455	0.33401	-2.232	0.02562	0.12092	2429.43	1129.46	2439.49	1298.1	739.754	1539.83	Down
Spag1	954.52	-0.5254	0.24708	-2.1267	0.03345	0.14604	1271.02	841.424	1267.02	753.569	582.119	1011.97	Down
Pabpc1	20686.8	0.21673	0.10087	2.14871	0.03166	0.1401	18319.1	20607.5	18480.9	22833.5	23195.1	20684.8	Up
Gm49282	4.59826	-3.6508	1.66361	-2.1945	0.0282	NA	1.95843	16.7693	6.82239	2.03943	0	0	Down
Gm10384	45.8473	1.05959	0.38347	2.76316	0.00572	0.0413	39.1685	24.6607	25.3403	75.4589	59.7573	50.698	Up
Gm16136	779.346	-0.4214	0.14962	-2.8163	0.00486	0.03653	961.587	743.768	971.703	670.972	649.088	678.956	Down
Ubr5	6319.55	0.2081	0.09078	2.2923	0.02189	0.10849	5863.53	5841.64	5888.7	6423.18	6618.64	7281.63	Up
2310043C	76.1668	-0.9626	0.30854	-3.12	0.00181	0.0173	92.046	123.304	86.7418	42.828	49.4543	62.6269	Down
1100001I1	51.2551	-1.9857	0.50958	-3.8968	9.75E-05	0.00183	64.628	47.3486	133.524	26.5126	20.606	14.9112	Down
Atp6v1c1	4475.06	0.27089	0.11784	2.29884	0.02151	0.10714	3997.15	3978.27	4192.85	4779.4	5504.88	4397.8	Up
Cthrc1	342.699	-0.8731	0.15935	-5.4795	4.27E-08	2.60E-06	493.523	435.015	401.546	226.377	246.241	253.49	Down
Gm9522	49.7046	-1.4824	0.50074	-2.9605	0.00307	0.02617	62.6696	77.9279	78.9448	27.5323	41.2119	9.94079	Down
Rims2	417.658	-1.1214	0.27212	-4.121	3.77E-05	0.00083	592.424	530.699	593.548	213.12	384.301	191.857	Down
Lrp12	1551.29	1.47529	0.2258	6.53358	6.42E-11	7.45E-09	925.356	872.99	663.721	2170.97	2955.93	1718.76	Up
AU022793	181.387	1.26004	0.29979	4.20313	2.63E-05	0.00062	143.944	96.6701	79.9194	228.416	327.635	211.739	Up
Gm49254	5.40199	2.86195	1.4051	2.03683	0.04167	NA	3.91685	0	0	6.11829	14.4242	7.95263	Up
Gm33251	6.60209	4.26192	1.30966	3.25421	0.00114	NA	0.97921	0.98643	0	14.276	14.4242	8.94671	Up
Gm49271	2.03441	4.49593	2.13984	2.10106	0.03564	NA	0	0	0	8.15772	2.0606	1.98816	Up
A930017M	72.1559	-1.5701	0.4958	-3.1668	0.00154	0.01533	149.82	51.2943	122.803	35.69	21.6363	51.6921	Down
Aard	479.971	-0.7878	0.13107	-6.01	1.86E-09	1.57E-07	617.883	583.966	621.812	350.782	327.635	377.75	Down
Ext1	3277.47	0.3396	0.13831	2.4554	0.01407	0.07957	2752.57	3357.81	2570.09	3639.36	3923.38	3421.62	Up
Samd12	2058.19	0.49649	0.13106	3.78825	0.00015	0.00258	1659.77	1862.38	1600.34	2142.42	2672.59	2411.63	Up
Tnfrsf11b	274.199	0.46022	0.22678	2.02932	0.04243	0.17118	244.803	272.255	175.433	361.999	317.332	273.372	Up
Enpp2	3592.72	-0.4051	0.11402	-3.5529	0.00038	0.00522	3922.73	3926.98	4431.63	2937.8	3390.71	2946.45	Down
Derl1	8845.71	0.22178	0.11156	1.98802	0.04681	0.18301	8137.26	8722.01	7642.05	9593.48	10307.1	8672.34	Up
Atad2	1177.41	0.59054	0.13866	4.25901	2.05E-05	0.0005	895	839.452	1084.76	1419.44	1346.6	1479.19	Up
Wdyhv1	2489.66	-0.5598	0.16887	-3.3148	0.00092	0.01025	3268.61	2453.25	3178.26	2095.51	1690.72	2251.59	Down
Anxa13	46.0001	-1.8596	0.6401	-2.9052	0.00367	0.02987	98.9005	46.3622	71.1478	31.6112	4.12119	23.8579	Down
Fam91a1	6655.07	0.27952	0.09432	2.9636	0.00304	0.02598	5776.38	6271.72	5989.08	6791.3	7518.09	7583.83	Up
Ndufb9	1388.38	-0.2041	0.10112	-2.0182	0.04357	0.17438	1557.93	1444.13	1457.07	1338.89	1310.54	1221.72	Down
Mtss1	5050.71	0.31079	0.09646	3.22206	0.00127	0.01329	4186.13	4840.41	4499.85	5451.4	5710.94	5615.55	Up
Washc5	3290.25	0.23529	0.09764	2.40973	0.01596	0.08739	3071.79	3078.65	2917.06	3489.46	3827.56	3357	Up
Lratd2	11955.7	-0.4653	0.20751	-2.2424	0.02494	0.11857	16359.7	10377.2	14865	9988.11	8178.51	11965.7	Down
Fam49b	4739.78	0.35324	0.13408	2.63459	0.00842	0.05464	4009.88	4724.01	3753.29	4966.01	5878.88	5106.58	Up
Asap1	2668.6	0.36827	0.12647	2.91178	0.00359	0.02941	2258.06	2403.93	2327.41	2799.12	3454.59	2768.51	Up
Oc90	8.7272	2.51085	1.08826	2.30722	0.02104	0.10593	1.95843	0	5.84776	22.4337	7.21209	14.9112	Up
Gm30929	24.7345	-1.2144	0.56154	-2.1626	0.03057	0.13697	30.3556	19.7286	53.6045	17.3352	15.4545	11.9289	Down
Lrrc6	1215.87	-1.0556	0.28799	-3.6654	0.00025	0.00377	1866.38	1131.43	1927.81	866.758	502.786	1000.04	Down
Gm7859	27.0234	-1.2684	0.46752	-2.7132	0.00666	0.04597	31.3348	36.4979	46.7821	13.2563	13.3939	20.8756	Down
Gm2999	99.9063	0.62045	0.26409	2.34936	0.01881	0.09807	97.9213	75.9551	62.3761	116.247	124.666	122.272	Up
Col22a1	10.0944	2.37207	1.01389	2.33958	0.01931	0.09974	7.8337	1.97286	0	20.3943	15.4545	14.9112	Up
Mroh5	18.1631	-1.5636	0.79472	-1.9674	0.04913	0.189	19.5843	34.525	27.2896	2.03943	19.5757	5.96447	Down
Arc	97.9637	-1.4426	0.40255	-3.5836	0.00034	0.00479	196.822	76.9415	155.94	63.2223	40.1816	54.6743	Down
4933427E	5.42914	-2.1186	1.06637	-1.9867	0.04695	NA	10.7713	8.87787	6.82239	4.07886	1.0303	0.99408	Down
Them6	890.778	-0.4544	0.15253	-2.9792	0.00289	0.02496	962.566	1090.99	1036.03	809.654	821.148	624.281	Down
Lypd2	1305.64	-2.6492	0.97334	-2.7218	0.00649	0.04514	3504.6	560.292	2691.92	253.909	113.333	709.772	Down
Slurp2	25.7453	-2.3558	0.59542	-3.9565	7.61E-05	0.00149	53.8567	31.5657	43.8582	9.17743	3.0909	12.923	Down
Lynx1	1752.34	-0.5978	0.15672	-3.8147	0.00014	0.00239	2449.99	1791.36	2089.6	1528.55	1254.9	1399.66	Down
Ly6m	135.266	-1.7063	0.38777	-4.4003	1.08E-05	0.0003	237.949	104.562	278.743	58.1237	56.6664	75.55	Down
Ly6e	71062.8	0.25036	0.09748	2.56818	0.01022	0.06298	64596.7	62399.6	67740.5	82044.2	78641.7	70954.3	Up
Ly6i	3975.58	3.66312	0.5077	7.21508	5.39E-13	9.36E-11	392.664	467.568	884.962	9368.12	3932.65	8807.54	Up
Ly6c2	2561.26	1.43792	0.22206	6.47547	9.45E-11	1.01E-08	1213.24	1877.18	1052.6	3889.19	4044.95	3290.4	Up
Ly6g	11.9066	1.507	0.75834	1.98723	0.0469	0.18318	7.8337	1.97286	8.77165	23.4534	16.4848	12.923	Up
Ly6g2	122.872	3.97367	0.32262	12.317	7.34E-35	1.74E-31	15.6674	15.7829	12.6702	261.047	244.181	187.881	Up
Gm10238	2.36728	3.6488	1.80354	2.02313	0.04306	NA	0	0.98643	0	3.05914	6.18179	3.97631	Up
9030619P	3.15999	5.13264	2.52511	2.03264	0.04209	NA	0	0	0	0	2.0606	16.8993	Up
Gsdmd	2753.81	0.27396	0.11643	2.35292	0.01863	0.09745	2204.21	2641.66	2633.44	3075.46	3100.17	2867.92	Up
Mapk15	5425.2	-0.9759	0.26293	-3.7117	0.00021	0.00327	7571.27	4900.58	9107.89	4534.67	2604.59	3832.17	Down
Iqank1	823.302	-0.5602	0.2146	-2.6104	0.00904	0.05745	1066.36	740.809	1136.42	658.736	550.179	787.31	Down
Nrbp2	3213.25	0.27766	0.09787	2.83703	0.00455	0.03495	2726.13	3003.68	2985.28	3404.83	3427.8	3731.77	Up
Gm41349	152.752	-0.6851	0.24423	-2.8053	0.00503	0.03753	169.404	164.734	230.987	112.169	103.03	136.189	Down
Eppk1	848.589	-0.5928	0.21689	-2.7333	0.00627	0.04413	1064.4	747.714	1249.47	578.178	690.3	761.464	Down
Gm19945	23.9332	-1.0573	0.46439	-2.2768	0.0228	0.11126	33.2932	30.5793	33.1373	11.2169	16.4848	18.8875	Down
Mroh1	5615.38	0.20401	0.09851	2.0709	0.03837	0.16044	4998.88	5247.81	5410.16	6173.35	6326.03	5536.02	Up
Cpsf1	3733.32	0.21833	0.09626	2.2682	0.02332	0.11317	3467.39	3489	3397.55	4144.12	4210.83	3691.01	Up
Tonsl	408.37	0.41393	0.156	2.65333	0.00797	0.0523	315.307	353.142	382.054	514.956	453.331	431.43	Up
Apol9a	296.563	-0.5852	0.28152	-2.0786	0.03765	0.15832	294.743	336.372	436.633	292.658	154.545	264.425	Down
Apol7b	12.0769	1.528	0.74945	2.03883	0.04147	0.16871	6.85449	9.8643	1.94925	22.4337	15.4545	15.9053	Up
Apol7c	67.253	0.94185	0.31633	2.97742	0.00291	0.02507	57.7736	39.4572	40.9343	92.7941	73.1512	99.4079	Up
Apol9b	425.166	-0.4914	0.2352	-2.0894	0.03667	0.15578	364.267	553.387	573.081	379.334	282.302	398.625	Down
Myh9	22814.4	0.36656	0.09972	3.67592	0.00024	0.00366	19869.2	21006	18919.5	23831.8	27468.8	25791.4	Up
A730060N	3.26795	-4.0934	1.65806	-2.4688	0.01356	NA	7.8337	4.93215	5.84776	0	0	0.99408	Down
Ift27	1559.98	-0.5896	0.16256	-3.6266	0.00029	0.00422	1998.57	1541.79	2082.78	1270.56	1098.3	1367.85	Down
Ncf4	946.588	0.72424	0.20689	3.50056	0.00046	0.00608	612.987	928.23	600.37	1136.98	1336.3	1064.66	Up

Csf2rb2	1453.04	1.19442	0.22504	5.30769	1.11E-07	6.07E-06	901.855	1130.45	618.888	1755.95	2057.51	2253.58 Up
Csf2rb	3989.78	1.40506	0.19528	7.19495	6.25E-13	1.06E-10	2252.19	2659.41	1650.04	5260.71	6686.64	5429.66 Up
Mpst	1954.58	-0.2593	0.13049	-1.9869	0.04693	0.18321	2131.75	1978.78	2278.68	2012.92	1616.54	1708.82 Down
Sstr3	18.9937	-2.709	1.02954	-2.6313	0.00851	0.05504	64.628	10.8507	23.3911	0	5.15149	9.94079 Down
Rac2	2995.48	0.54031	0.19608	2.75556	0.00586	0.042	1940.8	3212.8	2169.52	3583.28	3664.77	3401.74 Up
Cyth4	2397.85	0.7759	0.18026	4.30431	1.68E-05	0.00042	1707.75	2149.43	1447.32	2749.15	3483.44	2850.02 Up
Lgals1	4234.91	0.53709	0.1488	3.60945	0.00031	0.00444	3781.72	3453.49	3131.48	5375.94	5497.67	4169.17 Up
Gm10865	20.1591	-1.2644	0.56026	-2.2568	0.02402	0.11556	23.5011	41.43	20.4672	10.1971	14.4242	10.9349 Down
Gcat	13.2479	1.51542	0.66445	2.2807	0.02257	0.11052	8.81291	7.89144	3.89851	14.276	24.7272	19.8816 Up
Galr3	21.7609	1.01819	0.48927	2.08104	0.03743	0.15772	11.7506	17.7557	13.6448	27.5323	35.0301	24.852 Up
Gm3924	85.0864	1.15888	0.29145	3.97622	7.00E-05	0.00138	44.0646	66.0908	47.7567	120.326	131.878	100.402 Up
Ankrd54	1932.57	-0.2792	0.10846	-2.5742	0.01005	0.06215	2040.68	2094.19	2222.15	1883.41	1611.39	1743.61 Down
Micall1	187.987	0.67718	0.20315	3.33341	0.00086	0.00974	132.194	137.114	164.712	217.199	213.272	263.431 Up
1700088E	338.913	-0.6897	0.29869	-2.3092	0.02093	0.10549	488.627	300.861	465.872	265.126	171.03	341.963 Down
Baiap2l2	61.7794	-1.8415	0.41908	-4.3943	1.11E-05	0.0003	103.797	75.9551	110.133	31.6112	13.3939	35.7868 Down
Pla2g6	2148.5	-0.3476	0.11781	-2.9503	0.00317	0.02674	2447.05	2145.48	2625.65	1961.93	1809.2	1901.67 Down
Fam227a	1156.85	-0.7554	0.23091	-3.2713	0.00107	0.01163	1591.22	1045.62	1722.17	888.172	678.967	1014.95 Down
Cby1	1329.33	-0.4213	0.12731	-3.3095	0.00093	0.01042	1551.07	1358.31	1656.87	1181.85	1050.9	1176.99 Down
Dnal4	2076.18	-0.4468	0.14361	-3.111	0.00186	0.01773	2654.65	2088.27	2442.42	1933.38	1561.93	1776.42 Down
AC11359	193.795	0.67037	0.18707	3.58356	0.00034	0.00479	144.923	134.154	169.585	250.85	226.666	236.591 Up
Apobec3	9116.87	0.54864	0.13139	4.17555	2.97E-05	0.00068	7015.08	6538.06	8658.59	10838.5	10742.9	10908 Up
Pdgfb	5787.45	0.44254	0.20819	2.1256	0.03354	0.14629	3988.33	5628.57	5103.15	6196.81	8514.39	5293.47 Up
Slc25a17	5417.96	-0.4227	0.14718	-2.8717	0.00408	0.03211	6570.52	5221.17	6826.29	4683.55	4130.47	5075.76 Down
Chadl	109.345	-0.6404	0.25691	-2.4925	0.01268	0.07351	117.506	125.277	156.915	71.38	87.5754	97.4197 Down
Tob2	2581.4	-0.3417	0.13081	-2.6123	0.00899	0.05725	3018.91	2515.4	3122.71	2481.99	2186.29	2163.11 Down
Csdc2	82.3015	-1.0363	0.40896	-2.534	0.01128	0.06751	147.861	66.0908	117.93	53.0252	72.1209	36.7809 Down
Pmm1	1173.28	-0.3898	0.14073	-2.7701	0.0056	0.04065	1210.31	1262.63	1519.44	1027.87	1088	931.452 Down
Septin3	334.926	0.64907	0.23518	2.75986	0.00578	0.04168	306.494	192.354	283.617	332.427	452.301	442.365 Up
Naga	8100.35	0.20545	0.08705	2.36001	0.01827	0.09622	7875.81	7198.96	7498.78	8915.37	8826.57	8286.64 Up
Cyp2d10	85.8083	2.43469	0.43997	5.5337	3.14E-08	1.99E-06	36.2309	22.6879	21.4418	226.377	94.7875	113.325 Up
Cyp2d34	44.1748	4.56121	0.83182	5.48341	4.17E-08	2.56E-06	8.81291	0	1.94925	145.819	59.7573	48.7098 Up
Nfam1	1846.19	0.91597	0.23357	3.92163	8.80E-05	0.00167	1136.87	1673.97	1026.28	2098.57	3055.87	2085.58 Up
Bik	800.769	-0.4263	0.19626	-2.1722	0.02984	0.13453	1015.44	1016.02	723.173	611.829	811.875	626.269 Down
Efcab6	978.136	-1.0907	0.29906	-3.6472	0.00027	0.00397	1520.72	935.135	1537.96	655.677	384.301	835.026 Down
Sult4a1	289.464	-0.8189	0.25221	-3.247	0.00117	0.01246	324.119	313.685	470.745	190.687	174.12	263.431 Down
Pnpla5	70.9429	0.96569	0.41882	2.30575	0.02112	0.10618	53.8567	48.3351	41.909	95.8532	135.999	49.7039 Up
Gm33432	23.9294	1.83758	0.61356	2.99495	0.00274	0.02401	12.7298	10.8507	7.79702	25.4929	62.8482	23.8579 Up
Parvg	1000.21	1.03429	0.18759	5.51359	3.52E-08	2.20E-06	627.675	735.876	605.244	1242.01	1671.14	1119.33 Up
Rtl6	419.798	-0.338	0.13391	-2.5241	0.0116	0.0688	466.105	460.663	479.517	340.585	382.241	389.679 Down
Prr5	238.935	-0.6397	0.25409	-2.5177	0.01181	0.06974	316.286	357.088	199.799	194.766	193.696	171.976 Down
5031439G	2669.98	0.39826	0.10705	3.72028	0.0002	0.00318	2372.63	2339.81	2198.76	3046.91	3277.38	2784.41 Up
Upk3a	280.921	-1.4337	0.45858	-3.1264	0.00177	0.01704	360.35	289.024	580.878	156.016	57.6967	241.561 Down
Ribc2	248.342	-1.4537	0.31364	-4.6349	3.57E-06	0.00012	493.523	211.096	386.927	138.681	123.636	136.189 Down
Wnt7b	1323.94	-0.7209	0.25675	-2.8079	0.00499	0.03729	1895.76	1114.67	1933.66	1162.47	735.633	1101.44 Down
AU02275	416.057	-0.6967	0.29306	-2.3772	0.01745	0.09342	524.858	365.965	653	414.004	218.423	320.093 Down
Lncppara	365.795	0.73213	0.16882	4.33666	1.45E-05	0.00037	295.722	226.879	302.134	455.813	443.028	471.193 Up
Ppara	172.85	-0.9062	0.30712	-2.9507	0.00317	0.02672	251.658	225.892	198.824	131.543	72.1209	157.064 Down
Cdpf1	1438.96	-0.3921	0.10401	-3.7697	0.00016	0.00273	1741.04	1551.65	1607.16	1301.16	1222.96	1209.79 Down
Tbc1d22a	2042.86	0.36313	0.12381	2.933	0.00336	0.02795	1711.66	2009.36	1640.3	2376.96	2369.69	2149.2 Up
Tafa5	178.504	-0.5368	0.25386	-2.1146	0.03446	0.14899	225.219	191.367	217.342	114.208	192.666	130.224 Down
Ttll8	318.094	-1.1686	0.30683	-3.8085	0.00014	0.00243	408.332	275.214	637.406	181.509	184.423	221.68 Down
Mlc1	7662.8	0.39105	0.15426	2.53494	0.01125	0.06742	6581.29	7906.23	5404.31	8396.33	8906.93	8781.69 Up
Mov10l1	7.68668	-3.3793	1.13277	-2.9833	0.00285	0.02474	21.5427	6.90501	13.6448	2.03943	0	1.98816 Down
Panx2	39.6848	-1.2978	0.48303	-2.6867	0.00722	0.04863	64.628	30.5793	74.0717	17.3352	22.6666	28.8283 Down
Mapk11	1359.26	0.58984	0.1514	3.8958	9.79E-05	0.00183	1012.51	1015.04	1228.03	1404.15	1800.96	1694.9 Up
Plxnb2	22340.1	0.33262	0.09165	3.6294	0.00028	0.00419	19683.2	18529.1	21116.3	24771.9	24196.6	25743.7 Up
Tymp	1817.52	-1.005	0.2351	-4.2747	1.91E-05	0.00047	3080.6	1727.24	2470.68	1279.74	956.117	1390.72 Down
Odf3b	2440.63	-0.9173	0.22381	-4.0986	4.16E-05	0.0009	3387.1	2390.12	3797.15	1958.87	1274.48	1836.06 Down
Rabl2	2307.94	-0.9139	0.23627	-3.8678	0.00011	0.00201	3510.48	2114.91	3420.94	1731.48	1219.87	1849.98 Down
Syt10	93.031	-2.1759	0.39613	-5.493	3.95E-08	2.44E-06	186.05	90.7515	180.306	27.5323	27.8181	45.7276 Down
Cpne8	2357.31	0.31989	0.1259	2.54079	0.01106	0.06649	2017.18	2294.44	1979.47	2361.66	2878.65	2612.44 Up
Kif21a	2584.58	-0.9382	0.27011	-3.4734	0.00051	0.0066	3955.04	2279.64	3955.04	1686.61	1294.05	2337.08 Down
Abcd2	1874.85	1.14243	0.24677	4.62961	3.66E-06	0.00012	1060.49	1488.52	958.059	2236.23	3465.92	2039.85 Up
Lrrk2	19740.6	0.41293	0.18676	2.21096	0.02704	0.12551	17331.1	18453.1	15020	19206.3	19474.7	28958.5 Up
Prickle1	4128.09	0.6398	0.21007	3.04572	0.00232	0.02116	2420.61	3804.66	3457	4630.53	6164.28	4291.44 Up
Irak4	1327.68	0.48247	0.11211	4.30374	1.68E-05	0.00042	1153.51	1148.2	1021.41	1521.41	1644.36	1477.2 Up
Twf1	553.313	0.4591	0.19678	2.33301	0.01965	0.10092	559.13	391.613	447.354	590.415	573.876	757.488 Up
Ano6	1533.25	0.44389	0.13851	3.20477	0.00135	0.01388	1443.36	1160.04	1294.31	1604.01	1747.39	1950.38 Up
D030018L	1073.23	-1.903	0.32953	-5.7747	7.71E-09	5.81E-07	1299.42	1297.15	2484.32	552.685	271.999	533.82 Down
Slc38a4	1237.26	-1.4933	0.37424	-3.9901	6.60E-05	0.00132	2035.78	2102.08	1340.11	617.947	298.787	1028.87 Down
Rpap3	1231.2	-0.284	0.12404	-2.2899	0.02203	0.10888	1379.71	1227.12	1449.27	1144.12	1018.97	1168.04 Down
Tmem106	1607.06	-0.3244	0.12706	-2.5531	0.01068	0.06502	1837.98	1691.73	1831.32	1539.77	1241.51	1500.06 Down
Senp1	1875.63	0.20965	0.09827	2.13334	0.0329	0.14419	1665.64	1839.69	1713.39	1956.83	2106.96	1971.26 Up
Pfkm	1209.61	-0.401	0.10787	-3.7175	0.0002	0.00321	1347.4	1296.17	1486.31	1067.64	1029.27	1030.86 Down
Ccdc184	162.259	-1.226	0.53379	-2.2968	0.02163	0.10754	225.219	377.803	78.9448	118.287	122.606	50.698 Down
Adcy6	1943.82	0.37089	0.15998	2.31829	0.02043	0.10378	1859.53	1426.38	1800.14	2209.72	1898.84	2468.3 Up
Ccdc65	1214.8	-1.0975	0.26617	-4.1234	3.73E-05	0.00082	2074.95	1129.46	1763.1	853.501	552.24	915.546 Down
Tuba1b	8423.48	-0.2639	0.09885	-2.6695	0.0076	0.05043	9272.17	8901.54	9401.25	8041.47	7955.96	6968.49 Down
4930578N	62.6561	-1.2265	0.36497	-3.3607	0.00078	0.00899	111.63	57.2129	94.5388	34.6703	38.121	39.7631 Down
Tuba1c	4570.36	0.33089	0.12139	2.72579	0.00641	0.04475	4025.54	4344.24	3775.71	5252.55	5510.04	4514.11 Up
Prph	76.855	-1.1248	0.50434	-2.2303	0.02572	0.12122	92.046	152.897	71.1478	44.8675	78.3027	21.8697 Down
Kcnh3	705.027	-0.7622	0.23351	-3.264	0.0011	0.01186	1042.86	606.654	1011.66	520.055	457.453	591.477 Down
Prpf40b	2730.27	-0.3667	0.10957	-3.347	0.00082	0.00935	2963.1	2874.46	3388.78	2420.8	2281.08	2453.39 Down

Fmnl3	3401.31	0.41226	0.15403	2.67652	0.00744	0.04966	2558.68	3373.59	2823.5	3885.11	4361.25	3405.71	Up
Faim2	706.922	-0.6166	0.1554	-3.9678	7.25E-05	0.00143	843.102	880.882	843.053	512.917	664.542	497.039	Down
Aqp2	39.7173	-2.2386	0.63271	-3.5381	0.0004	0.00546	47.0022	41.43	108.184	25.4929	8.24239	7.95263	Down
Asic1	323.642	-0.7237	0.20887	-3.465	0.00053	0.00675	374.059	506.038	329.424	229.436	251.393	251.502	Down
Cox14	2333.28	-0.2732	0.10567	-2.585	0.00974	0.06054	2698.71	2515.4	2446.31	2257.65	2119.32	1962.31	Down
Lima1	577.446	0.4623	0.14328	3.22655	0.00125	0.01314	464.147	482.364	510.705	598.573	658.361	750.529	Up
Tmprss12	34.89	-2.5121	0.59936	-4.1914	2.77E-05	0.00064	63.6488	43.4029	71.1478	7.138	4.12119	19.8816	Down
Mettl7a1	28862.6	-0.4123	0.09328	-4.4202	9.86E-06	0.00027	30483.9	34319.9	34073.9	25162.5	23703	25432.5	Down
Csrnp2	410.294	-0.3616	0.15626	-2.3139	0.02067	0.10455	449.459	515.903	419.09	352.821	333.817	390.673	Down
Bin2	1358.97	0.58653	0.23369	2.50984	0.01208	0.07092	928.294	1449.07	882.038	1492.86	2002.9	1398.67	Up
Cela1	169.225	0.62659	0.20817	3.01	0.00261	0.0231	127.298	155.856	115.981	196.805	223.575	195.833	Up
Scn8a	44.6692	1.86531	0.50148	3.71961	0.0002	0.00319	18.605	7.89144	31.1881	77.4983	74.1815	58.6506	Up
Krt7	24590.7	0.34267	0.1276	2.68555	0.00724	0.04872	21373.3	20961.6	22716.6	29656.4	29521.1	23315.1	Up
Krt87	508.448	0.98278	0.29733	3.30536	0.00095	0.01055	365.246	234.77	424.937	943.236	535.755	546.743	Up
Krt79	951.743	2.01261	0.36371	5.53359	3.14E-08	1.99E-06	477.856	436.002	220.266	1119.65	2383.08	1073.6	Up
Krt78	17.0659	1.2471	0.60038	2.0772	0.03778	0.15871	6.85449	7.89144	15.594	18.3549	28.8484	24.852	Up
Igfbp6	5110.52	-0.6407	0.26547	-2.4135	0.0158	0.0867	7548.75	5298.11	5834.12	5273.97	4245.86	2462.33	Down
Csad	2321.95	-0.3163	0.11328	-2.7918	0.00524	0.0387	2503.85	2397.02	2825.44	2160.78	1942.11	2102.48	Down
Gm9918	13.5216	-1.4849	0.68688	-2.1618	0.03063	0.13706	26.4387	14.7964	18.5179	4.07886	11.3333	5.96447	Down
Prr13	6922.72	0.27691	0.14087	1.96569	0.04933	0.18925	6595	6620.92	5565.12	7653.98	8547.36	6553.96	Up
Pcbp2	11207.8	-0.2055	0.0776	-2.6478	0.0081	0.053	12422.3	12028.5	11563	10641.7	10325.7	10265.8	Down
Atp5g2	3822.95	-0.2056	0.09299	-2.2112	0.02702	0.1255	4098.01	3970.38	4216.24	3764.79	3543.2	3345.07	Down
Nfe2	325.349	0.6328	0.32061	1.97375	0.04841	0.18691	176.258	359.06	230.012	352.821	534.725	299.218	Up
Gpr84	4.22298	2.44697	1.23967	1.97389	0.04839	NA	0.97921	1.97286	0.97463	11.2169	7.21209	2.98224	Up
Itga5	2639.96	0.31819	0.12408	2.56433	0.01034	0.06344	2539.1	2472.98	2037.95	2933.72	3051.74	2804.3	Up
Nckap1l	2551.09	0.86973	0.17119	5.08051	3.76E-07	1.77E-05	1604.93	2201.71	1607.16	3121.35	3752.35	3019.02	Up
Ppp1r1a	148.969	-0.9653	0.2674	-3.61	0.00031	0.00443	241.866	195.313	153.991	95.8532	85.5148	121.278	Down
Cluap1	975.512	-0.5722	0.14562	-3.9294	8.51E-05	0.00162	1126.09	1073.24	1300.15	845.344	685.148	823.097	Down
Slx4	858.637	0.22462	0.10969	2.04784	0.04058	0.16623	760.848	822.682	792.372	912.645	887.087	976.185	Up
Dnase1	82.8258	0.51172	0.24807	2.06276	0.03914	0.16261	72.4617	71.0229	61.4015	92.7941	97.8784	101.396	Up
Adcy9	487.053	0.26794	0.13189	2.03149	0.04221	0.17067	454.355	431.07	440.532	501.7	514.119	580.542	Up
Srl	261.52	-1.5447	0.49258	-3.1359	0.00171	0.01663	549.338	112.453	506.806	123.386	107.151	169.987	Down
Glis2	2188.51	0.43825	0.21786	2.01161	0.04426	0.1761	1461.96	2113.92	1999.94	2500.34	3138.29	1916.58	Up
4930562C	983.739	-1.0213	0.27537	-3.7086	0.00021	0.0033	1518.76	894.692	1540.89	647.519	461.574	839.002	Down
Mgrn1	2753.81	0.32555	0.09442	3.44797	0.00056	0.00709	2517.56	2288.52	2527.21	2997.96	3011.56	3180.06	Up
Septin12	2.21142	4.6157	2.01377	2.29206	0.0219	NA	0	0	0	5.09857	6.18179	1.98816	Up
Sec14l5	352.735	-1.1529	0.30569	-3.7716	0.00016	0.00271	428.895	313.685	717.326	224.337	190.605	241.561	Down
Nagpa	1545.59	0.34909	0.16349	2.13528	0.03274	0.14381	1272	1589.14	1217.31	1704.96	1977.14	1512.99	Up
Pmm2	3004.17	0.26864	0.08787	3.05726	0.00223	0.02056	2662.48	2685.06	2828.37	3354.86	3137.26	3357	Up
Atf7ip2	40.0751	2.79054	0.43982	6.34477	2.23E-10	2.27E-08	7.8337	7.89144	14.6194	64.242	77.2724	68.5914	Up
Clec16a	2455.14	-0.2575	0.11766	-2.1888	0.02861	0.13081	2515.6	2998.75	2506.74	2214.82	2338.78	2156.16	Down
Shisa9	65.6698	-1.3017	0.29026	-4.4846	7.31E-06	0.00022	97.9213	89.7651	92.5896	43.8477	37.0907	32.8046	Down
Ntan1	1655.43	0.3334	0.09847	3.38583	0.00071	0.00838	1511.9	1481.62	1401.51	1942.56	1764.9	1830.1	Up
Mpv17l	1484.04	-0.3938	0.17273	-2.2799	0.02261	0.11066	1455.11	1611.83	1989.21	1233.86	1126.12	1488.14	Down
Nde1	2728.65	0.28191	0.08918	3.16126	0.00157	0.01557	2346.19	2499.61	2542.8	2984.71	3090.9	2907.68	Up
Fopnl	2283.17	-0.2082	0.09421	-2.2105	0.02707	0.12554	2342.28	2424.64	2575.94	2177.09	2147.14	2031.9	Down
Snai2	587.706	0.43319	0.211	2.05307	0.04007	0.16523	423.02	599.749	477.567	788.24	707.815	529.844	Up
Spag6l	3444.61	-0.883	0.29646	-2.9785	0.0029	0.02501	5172.2	3041.16	5187.94	2588.04	1476.42	3201.93	Down
Igll1	10.2053	-1.7328	0.82736	-2.0943	0.03623	0.15445	22.5219	16.7693	7.79702	6.11829	2.0606	5.96447	Down
2610318N	26.864	1.36561	0.52487	2.60178	0.00927	0.05849	14.6882	10.8507	19.4925	34.6703	55.6361	25.846	Up
Sdf2l1	1225.17	0.34088	0.13257	2.57138	0.01013	0.06252	1234.79	1041.67	966.83	1414.34	1370.3	1323.12	Up
Ccdc116	120.809	0.99774	0.30348	3.2877	0.00101	0.01108	72.4617	64.1179	105.26	203.943	133.939	145.135	Up
Rimbp3	4.07269	3.51892	1.6492	2.13372	0.03287	NA	1.95843	0	0	11.2169	9.27269	1.98816	Up
Pi4ka	2351.08	0.28462	0.11319	2.51449	0.01192	0.07019	2266.88	2157.32	1935.61	2396.33	2664.35	2686	Up
Snap29	2614.3	0.27169	0.10745	2.52846	0.01146	0.06807	2371.65	2458.18	2276.73	2614.55	3087.8	2876.86	Up
Lrrc74b	2081.75	-0.9375	0.33772	-2.7761	0.0055	0.04006	3383.18	1744.99	3077.87	1612.17	768.603	1903.66	Down
P2rx6	1211.89	-0.6223	0.24869	-2.5024	0.01234	0.07199	1636.26	958.81	1812.81	1021.75	781.997	1059.69	Down
Slc7a4	65.5309	-1.3744	0.45611	-3.0134	0.00258	0.02298	110.651	49.3215	123.778	23.4534	42.2422	43.7395	Down
Ccdc74a	1067.71	-0.9291	0.24839	-3.7405	0.00018	0.00299	1557.93	995.307	1647.12	868.797	538.846	798.245	Down
B830017H	28.7418	-4.1677	0.80321	-5.1888	2.12E-07	1.07E-05	73.441	24.6607	65.3	4.07886	0	4.97039	Down
Dgcr6	3.37828	3.22488	1.49642	2.15506	0.03116	NA	0.97921	0	0.97463	8.15772	6.18179	3.97631	Up
Prodh	1800.33	-0.3992	0.11732	-3.4025	0.00067	0.00803	1833.09	2081.37	2228.97	1505.1	1618.6	1534.86	Down
Rtn4r	51.8155	-1.195	0.50368	-2.3726	0.01766	0.09426	114.568	35.5115	66.2747	25.4929	41.2119	27.8342	Down
Dgcr8	551.657	0.30349	0.12197	2.4882	0.01284	0.07423	491.565	469.54	520.451	587.356	605.815	635.216	Up
Arvcf	1522.17	-0.3179	0.13807	-2.3023	0.02132	0.1067	1623.53	1590.12	1853.74	1434.74	1457.87	1173.01	Down
Tbx1	1368.87	-1.1612	0.29211	-3.9754	7.02E-05	0.00138	2116.08	1239.94	2319.61	960.571	549.149	1027.88	Down
4930588K	46.2386	-1.4648	0.43434	-3.3725	0.00075	0.00871	55.8151	61.1586	86.7418	25.4929	14.4242	33.7987	Down
Cldn5	14472	-0.4429	0.21913	-2.0211	0.04327	0.17352	18612.9	19558.9	11856.3	12812.7	14174.8	9816.53	Down
Gm28539	135.82	-0.9427	0.21632	-4.358	1.31E-05	0.00034	203.676	166.707	165.687	96.8729	86.5451	95.4315	Down
Lamp3	74272.8	0.75895	0.17803	4.26312	2.02E-05	0.00049	58242.6	65066.9	42216	83016	95834.2	101261	Up
Cyp2ab1	178.991	0.80251	0.31495	2.54807	0.01083	0.06552	157.653	145.992	87.7165	167.233	294.665	220.685	Up
Abcc5	8983.81	0.25228	0.09435	2.674	0.0075	0.04995	8756.12	8095.63	7749.26	9302.86	9901.17	10097.8	Up
Vwa5b2	7.24867	-1.9095	0.93048	-2.0522	0.04015	NA	6.85449	16.7693	10.7209	5.09857	2.0606	1.98816	Down
Eif4g1	14480.2	0.20557	0.08087	2.54194	0.01102	0.06636	13717.8	12995.2	13638	14874.6	15560.6	16095.1	Up
Fam131a	1052.79	-0.7725	0.18165	-4.2525	2.11E-05	0.00051	1419.86	1078.17	1486.31	844.324	648.058	839.996	Down
Ehhadh	974.011	0.24343	0.11812	2.06082	0.03932	0.1631	919.481	915.407	841.103	1066.62	1130.24	971.215	Up
Etv5	11384.8	0.49698	0.24029	2.06828	0.03861	0.16127	8726.74	12523.7	7078.72	13512.2	16235.4	10232.1	Up
Kng2	3607.01	4.38141	0.24778	17.6829	5.68E-70	1.07E-65	404.415	300.861	285.566	6899.39	9041.9	4709.94	Up
Kng1	234.578	3.31813	0.42746	7.76251	8.33E-15	2.07E-12	50.9191	25.6472	51.6552	317.131	702.664	259.454	Up
Eif4a2	36354.6	0.32394	0.11463	2.82588	0.00472	0.03585	34295	34173.9	28401.6	39080.6	42725.4	39451	Up
Rtp4	2551.11	-0.4451	0.1674	-2.6587	0.00784	0.0517	2441.18	3153.62	3229.91	2212.78	1826.72	2442.45	Down
Sst	42.589	-2.1523	0.76211	-2.8241	0.00474	0.03591	32.314	150.924	25.3403	14.276	24.7272	7.95263	Down
Lpp	5736.36	0.27947	0.10692	2.61396	0.00895	0.05703	5031.2	5623.64	4892.63	5856.22	6644.39	6370.06	Up

Mb21d2	642.869	-0.5764	0.13675	-4.215	2.50E-05	0.00059	789.245	819.723	699.782	505.779	562.543	480.14	Down
Plaat1	21.26	-1.4918	0.50773	-2.9382	0.0033	0.02755	23.5011	36.4979	34.112	11.2169	10.303	11.9289	Down
Gm1968	166.57	-1.0452	0.25993	-4.0213	5.79E-05	0.00118	170.383	242.662	260.225	89.7349	108.181	128.236	Down
Hes1	5320.51	-0.4433	0.17712	-2.5026	0.01233	0.07196	6037.83	4743.74	7612.81	4439.84	4523.01	4565.8	Down
Cpn2	316.712	1.47168	0.31281	4.70472	2.54E-06	8.86E-05	139.048	168.679	195.9	533.311	267.878	595.453	Up
Gp5	123.896	-1.0091	0.32028	-3.1508	0.00163	0.01597	118.485	158.815	219.291	104.011	74.1815	68.5914	Down
Atp13a3	16952	0.29356	0.13475	2.17846	0.02937	0.13307	16298	13047.5	16354.2	19063.6	16872.2	20076.4	Up
Fam43a	2344.1	-0.4012	0.15375	-2.6094	0.00907	0.05756	2817.2	2175.08	3011.6	2154.66	1876.17	2029.91	Down
Gm49755	4.56148	-5.6116	1.72078	-3.2611	0.00111	NA	6.85449	3.94572	16.5687	0	0	0	Down
Gm49756	85.1965	-9.8346	1.31795	-7.4621	8.52E-14	1.71E-11	155.695	60.1722	295.312	0	0	0	Down
Gm49757	61.4139	-9.3624	1.328	-7.05	1.79E-12	2.80E-10	97.9213	49.3215	221.24	0	0	0	Down
Gm46565	299.447	0.85676	0.41297	2.07462	0.03802	0.15939	368.184	167.693	103.31	422.162	302.908	432.424	Up
Acap2	5272.58	0.23916	0.101	2.36791	0.01789	0.09509	4850.04	4473.46	5185.99	5621.69	5990.16	5514.15	Up
Gm41442	330.859	-0.3659	0.18234	-2.0068	0.04477	0.17733	373.08	425.151	319.678	274.303	267.878	325.064	Down
Gm49767	147.659	0.65417	0.24548	2.66481	0.0077	0.05096	97.9213	143.032	103.31	154.997	197.817	188.875	Up
Apod	414.128	-1.5095	0.20423	-7.3913	1.45E-13	2.81E-11	571.86	584.953	682.239	173.352	202.969	269.395	Down
Bdh1	578.426	-0.7176	0.25651	-2.7978	0.00515	0.03819	755.952	468.554	933.693	460.911	382.241	469.205	Down
Gm20056	22.7163	1.01752	0.4636	2.19485	0.02817	0.12938	14.6882	13.81	16.5687	25.4929	31.9393	33.7987	Up
Tctex1d2	1023.09	-0.7301	0.1798	-4.0606	4.89E-05	0.00103	1456.09	1018.98	1354.73	774.983	674.845	858.884	Down
Tfrc	8770.43	0.44097	0.16388	2.6908	0.00713	0.0482	6938.7	7337.06	8045.55	8670.64	9174.81	12455.8	Up
Tnk2	2467.68	-0.2939	0.0838	-3.5073	0.00045	0.00597	2638.98	2775.81	2739.68	2228.08	2259.44	2164.11	Down
Muc4	4136.13	0.87588	0.28878	3.03304	0.00242	0.02185	3678.9	1591.11	3483.32	5200.55	4570.4	6292.52	Up
Muc20	4135.55	1.05649	0.29481	3.58358	0.00034	0.00479	2419.63	1605.91	4031.06	5864.38	4642.52	6249.77	Up
1700007L	53.2027	-0.7999	0.32855	-2.4345	0.01491	0.0831	67.5657	61.1586	74.0717	41.8083	29.8787	44.7335	Down
Gm15657	362.414	0.44574	0.17703	2.51797	0.0118	0.06972	305.514	341.305	273.87	460.911	356.483	436.4	Up
Muc13	587.17	5.04382	0.76239	6.61582	3.69E-11	4.51E-09	38.1893	12.8236	52.6299	1845.68	454.362	1119.33	Up
Kalrn	1538.61	0.57049	0.15223	3.74767	0.00018	0.00293	1161.35	1131.43	1421.98	1778.38	2086.35	1652.16	Up
Ccdc14	508.446	-0.516	0.18207	-2.834	0.0046	0.0352	536.609	519.848	738.767	410.945	411.089	433.418	Down
Sema5b	45.2604	-5.7134	0.74884	-7.6296	2.35E-14	5.50E-12	98.9005	73.9822	93.5642	0	4.12119	0.99408	Down
Slc49a4	3251.58	0.34811	0.09562	3.64049	0.00027	0.00404	2771.17	2812.31	2999.9	3679.13	3821.38	3425.59	Up
Hspbap1	681.704	0.44701	0.11977	3.73224	0.00019	0.00306	594.382	560.292	576.005	815.772	819.087	724.683	Up
Parp14	5107.9	0.42248	0.14782	2.85811	0.00426	0.03325	3715.13	4895.65	4485.23	5206.66	5860.34	6484.37	Up
Dtx3l	2684.41	0.58214	0.1232	4.72519	2.30E-06	8.19E-05	1965.28	2084.33	2400.51	2967.37	3330.95	3358	Up
Parp9	1545.66	0.31298	0.09853	3.17646	0.00149	0.01494	1313.12	1421.45	1401.51	1653.98	1687.63	1796.3	Up
Fam162a	2217.91	0.31099	0.12171	2.55526	0.01061	0.06467	1951.57	2074.46	1913.19	2337.19	2771.5	2259.54	Up
Csta2	97.0098	6.61447	0.65093	10.1615	2.94E-24	3.28E-21	0	1.97286	3.89851	190.687	220.484	165.017	Up
Stfa1	7.91893	6.4575	1.50923	4.27866	1.88E-05	0.00046	0	0	0	21.414	7.21209	18.8875	Up
Slc15a2	5788.97	-0.9869	0.12634	-7.8115	5.65E-15	1.46E-12	8344.85	7379.48	7361.36	3443.58	4338.59	3865.97	Down
Golgb1	6001.42	0.21188	0.09837	2.15385	0.03125	0.13863	5722.52	5337.57	5624.57	6635.28	5913.91	6774.65	Up
Hcls1	1910.2	0.84209	0.16904	4.9817	6.30E-07	2.71E-05	1220.1	1632.54	1251.42	2235.22	2840.53	2281.41	Up
Fstl1	3681.55	0.49746	0.13771	3.61248	0.0003	0.00441	3227.49	3171.37	2760.14	4350.1	4844.46	3735.75	Up
Gsk3b	5892.43	0.2932	0.08321	3.52357	0.00043	0.00567	5178.08	5276.41	5432.57	6353.84	6809.24	6304.45	Up
Nr1i2	409.85	-0.9442	0.34236	-2.758	0.00582	0.04183	605.153	361.033	652.026	333.447	154.545	352.898	Down
Maats1	1631.33	-0.7645	0.29389	-2.6012	0.00929	0.05851	2525.39	1341.54	2294.27	1333.79	780.966	1511.99	Down
Popdc2	586.296	-0.4674	0.16862	-2.7722	0.00557	0.04044	728.534	560.292	752.412	492.522	533.695	450.318	Down
D930030I(	4.43588	-2.4767	1.22799	-2.0168	0.04371	NA	6.85449	9.8643	5.84776	0	2.0606	1.98816	Down
Poglut1	2266.88	0.22126	0.09774	2.26371	0.02359	0.11397	2199.31	2123.78	1957.05	2375.94	2535.56	2409.65	Up
Arhgap31	11257.3	0.60693	0.12904	4.70332	2.56E-06	8.87E-05	7950.23	9754.8	9065.98	12792.3	15185.6	12794.8	Up
Upk1b	2003.17	0.47996	0.15886	3.02124	0.00252	0.02252	1615.7	1608.87	1794.29	2727.74	2371.75	1900.68	Up
Igsf11	247.017	-0.692	0.27895	-2.4807	0.01311	0.07546	328.036	225.892	361.587	208.022	132.908	225.656	Down
Ccdc191	1273.11	-0.7165	0.22669	-3.1605	0.00157	0.0156	1691.1	1162.01	1895.65	1006.46	754.178	1129.27	Down
Atp6v1a	10353.3	0.27714	0.09461	2.92924	0.0034	0.02817	9411.21	9663.06	9011.4	10469.4	11786.6	11777.8	Up
Naa50	6000.14	0.23959	0.0834	2.87282	0.00407	0.03202	5528.64	5401.69	5578.77	6189.67	6795.85	6506.24	Up
Sidt1	790.981	0.85568	0.11976	7.14471	9.02E-13	1.50E-10	566.964	516.889	605.244	981.985	1050.9	1023.9	Up
Cfap44	3045.16	-1.4485	0.31014	-4.6706	3.00E-06	0.0001	5004.76	2918.85	5448.17	1925.22	953.026	2020.96	Down
Nepro	422.459	0.30062	0.12589	2.38786	0.01695	0.09143	394.623	363.993	377.181	466.01	466.725	466.223	Up
Cd200r1	731.405	1.77608	0.21024	8.44787	2.97E-17	1.15E-14	316.286	413.314	262.175	1132.9	1307.45	956.304	Up
Gm19142	25.91	1.76681	0.51134	3.45525	0.00055	0.00693	11.7506	12.8236	10.7209	34.6703	56.6664	28.8283	Up
Cd200r4	1042.09	1.69373	0.21538	7.8638	3.73E-15	1.07E-12	523.879	512.943	439.557	1560.16	2041.02	1175	Up
Cd200r2	66.3076	4.42802	0.75843	5.83838	5.27E-09	4.14E-07	5.87528	7.89144	3.89851	93.8138	256.544	29.8224	Up
Ccdc80	1559.16	0.68631	0.20248	3.38953	0.0007	0.00831	1557.93	1078.17	949.287	2023.11	2029.69	1716.77	Up
Slc35a5	2325.57	0.48598	0.10858	4.47578	7.61E-06	0.00022	1841.9	1889.01	2081.8	2701.22	2541.75	2897.74	Up
Atg3	4227.51	0.30633	0.09849	3.11015	0.00187	0.01776	3606.44	3817.48	3917.03	4695.79	4973.25	4355.06	Up
Btla	1031.64	0.5363	0.17433	3.07634	0.0021	0.0195	744.202	919.352	862.545	1181.85	1460.96	1020.92	Up
Cd200	8494.71	0.94444	0.10291	9.17696	4.43E-20	2.62E-17	5690.21	6179	5559.27	11947	10332.9	11259.9	Up
Gm609	20.8377	1.83178	0.58853	3.11247	0.00186	0.01765	5.87528	6.90501	14.6194	39.7689	33.9998	23.8579	Up
Tagln3	27.5941	1.64316	0.53846	3.0516	0.00228	0.02088	19.5843	8.87787	11.6955	27.5323	41.2119	56.6625	Up
Abhd10	835.44	0.31129	0.13109	2.37461	0.01757	0.09386	657.052	820.709	759.235	956.493	904.602	914.552	Up
Phldb2	13275.9	0.30295	0.14913	2.03145	0.04221	0.17067	11662.4	13823.8	10175.1	13395	14312.9	16286	Up
Retnlb	8.82526	3.63855	1.26495	2.87645	0.00402	0.0318	0	3.94572	0	15.2957	25.7575	7.95263	Up
Retnla	54157.8	1.8895	0.22768	8.29897	1.05E-16	3.75E-14	22432.8	22107.9	24522.6	84537.4	114090	57255.9	Up
Cip2a	238.067	0.42984	0.16997	2.52893	0.01144	0.06807	193.884	210.11	204.672	255.948	258.605	305.182	Up
Ift57	1534.32	-0.8147	0.22594	-3.6057	0.00031	0.00449	2275.69	1389.88	2203.63	1139.02	899.451	1298.27	Down
Dubr	2375.26	1.15496	0.09215	12.5334	4.90E-36	1.55E-32	1442.38	1529.95	1444.4	3123.39	3320.65	3390.8	Up
Nfkbiz	2881.31	0.29787	0.14473	2.05815	0.03958	0.16394	2461.74	3006.64	2286.48	3452.75	2898.23	3182.05	Up
Nxpe3	836.203	-0.601	0.17024	-3.5302	0.00042	0.00559	870.52	1148.2	1004.84	712.781	721.209	559.666	Down
Gm28037	14.1209	2.13768	0.65044	3.2865	0.00101	0.01111	2.93764	6.90501	5.84776	25.4929	22.6666	20.8756	Up
Pcnp	5903.69	0.4457	0.09588	4.64853	3.34E-06	0.00011	5047.84	5303.05	4646.05	7002.38	6525.91	6896.92	Up
Adgrg7	28.7013	2.59051	0.62865	4.12078	3.78E-05	0.00083	13.709	8.87787	1.94925	41.8083	42.2422	63.621	Up
Tbc1d23	639.39	0.3443	0.13978	2.4632	0.01377	0.07833	642.364	512.943	535.07	700.544	707.815	737.606	Up
Cmss1	616.455	-0.5869	0.11905	-4.9304	8.21E-07	3.38E-05	763.786	695.433	761.184	512.917	485.271	480.14	Down
Col8a1	447.135	-0.4544	0.17965	-2.5294	0.01142	0.06802	434.77	584.953	531.172	428.28	362.665	340.969	Down
Gpr15	44.22	1.63295	0.37006	4.41266	1.02E-05	0.00028	23.5011	22.6879	18.5179	54.0449	69.03	77.5381	Up

Riox2	856.357	0.4006	0.11802	3.39438	0.00069	0.00822	809.809	685.569	719.275	974.847	968.481	980.161	Up
Crybg3	3366.33	0.29552	0.10347	2.85613	0.00429	0.03338	3126.63	2887.28	3054.48	3554.73	3533.92	4040.93	Up
Arl6	1329.75	-0.522	0.21591	-2.4175	0.01563	0.08609	1902.61	1181.74	1618.86	1228.76	869.572	1176.99	Down
4930547E	2.77499	-4.8944	1.90903	-2.5638	0.01035	NA	9.79213	2.95929	3.89851	0	0	0	Down
Pros1	6681.63	0.23739	0.10565	2.24691	0.02465	0.11748	6576.39	6088.24	5734.71	6996.26	7796.27	6897.91	Up
Cggbp1	8385.61	0.2038	0.08977	2.27008	0.0232	0.11279	7996.25	7776.02	7610.86	8682.87	8634.93	9612.74	Up
Cadm2	102	-1.2516	0.34284	-3.6507	0.00026	0.00394	190.946	112.453	127.676	50.9857	48.424	81.5144	Down
Samsn1	744.077	0.70796	0.22911	3.08997	0.002	0.01881	453.376	750.673	491.212	887.152	1081.81	800.233	Up
4930578N	61.3362	-2.5115	0.47564	-5.2801	1.29E-07	6.92E-06	83.2331	147.964	81.8687	10.1971	28.8484	15.9053	Down
Nrip1	1744.27	0.24062	0.10396	2.31457	0.02064	0.10448	1671.52	1656.22	1469.74	1829.37	1941.08	1897.7	Up
Mir99ahg	459.582	-0.2928	0.13391	-2.1866	0.02877	0.13136	464.147	531.686	522.4	396.669	413.15	429.442	Down
Chodl	83.6205	-1.3162	0.60104	-2.19	0.02853	0.13055	68.5449	243.648	45.8075	35.69	47.3937	60.6388	Down
Mrpl39	2421.05	-0.3344	0.13495	-2.4783	0.0132	0.07579	2985.62	2436.48	2679.25	2271.92	1887.51	2265.5	Down
Jam2	3941.54	0.27429	0.11783	2.32785	0.01992	0.10199	3413.54	3500.84	3789.35	4359.28	4733.19	3853.05	Up
Cyyr1	6652.93	0.36543	0.16532	2.21036	0.02708	0.12554	5245.64	6791.57	5407.23	6900.41	8893.54	6679.21	Up
Adamts1	6499.83	0.55355	0.26728	2.07104	0.03835	0.16043	5400.36	6867.52	3535.95	8603.33	9126.38	5465.44	Up
Adamts5	774.96	-0.7923	0.16815	-4.712	2.45E-06	8.59E-05	1016.42	1040.68	890.809	571.04	467.755	663.05	Down
Bach1	3839.05	0.31876	0.08377	3.80508	0.00014	0.00246	3395.91	3463.35	3390.73	4051.33	4354.04	4378.92	Up
Grik1	54.3464	-1.757	0.6501	-2.7026	0.00688	0.04697	52.8775	80.8872	117.93	25.4929	5.15149	43.7395	Down
Cldn8	584.674	-1.3614	0.40455	-3.3653	0.00076	0.0089	695.241	631.315	1198.79	328.348	148.363	505.986	Down
[truncated: 198,044 more chars]
