# Supplementary material for: Overexpression of ELF3 in the PTEN-deficient lung epithelium promotes lung cancer development by inhibiting ferroptosis
Source: Cell Death Dis. 2024 Dec 18;15(12):897. doi: 10.1038/s41419-024-07274-5 (PMC11655876; doi:10.1038/s41419-024-07274-5)
Supplement: Supplementary file 2 — Original WB [file 41419_2024_7274_MOESM2_ESM.pdf]

Blank(loading buffer)	H1650 *PTEN*$^{null/null}$	H1650 *PTEN*$^{null/null}$*ELF3*^{ov}	Marker	Marker	NL20 WT	NL20 *ELF3*^{ov}	NL20 *PTEN*$^{-/-}$	NL20 *PTEN*$^{-/-}$*ELF3*^{ov}	Marker

ELF3

β-actin

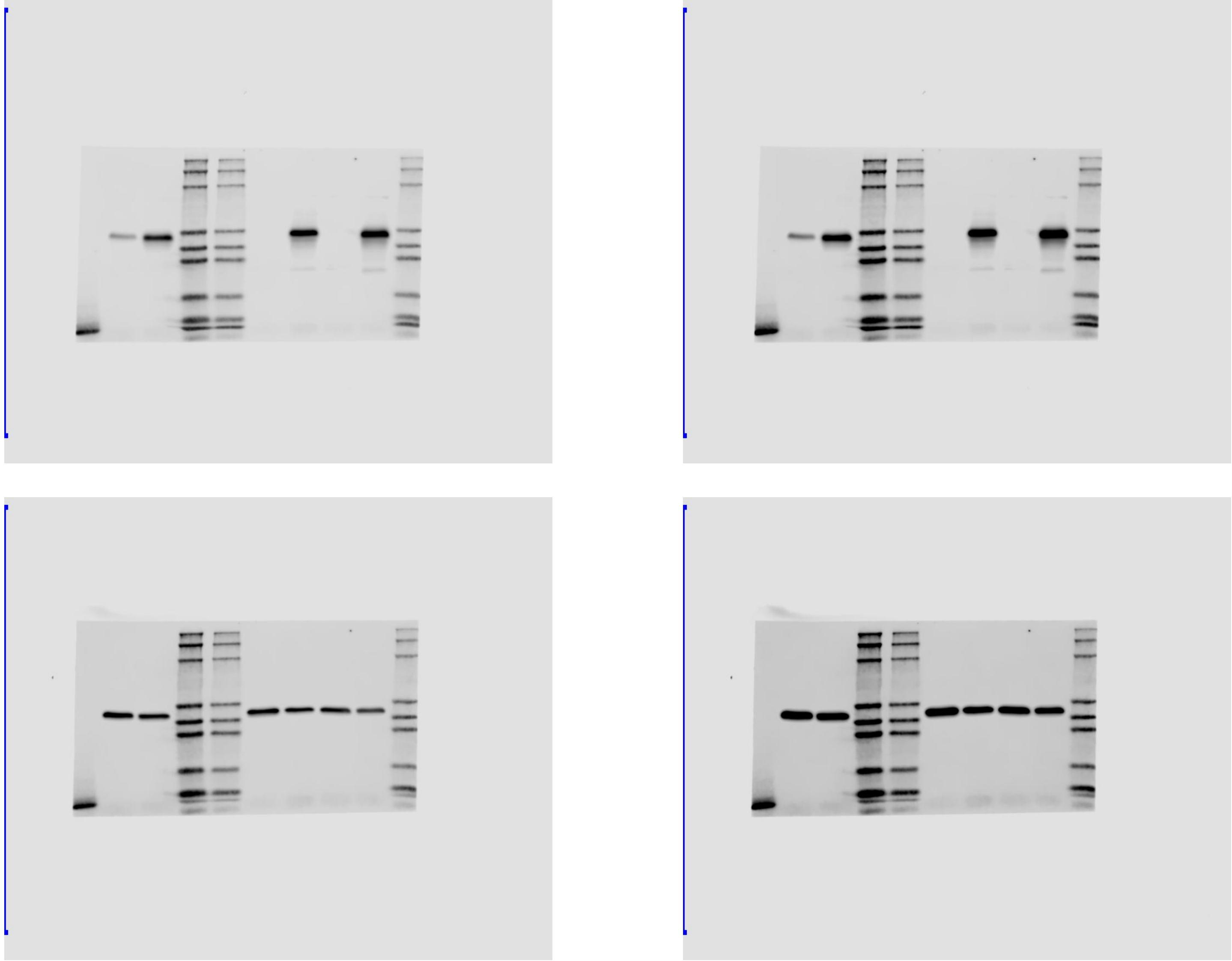

short exposure

long exposure

Beas2B WT	Beas2B *ELF3*^{ov}	Beas2B *PTEN*$^{-/-}$	Beas2B *PTEN*$^{-/-}$*ELF3*^{ov}	Marker		

ELF3

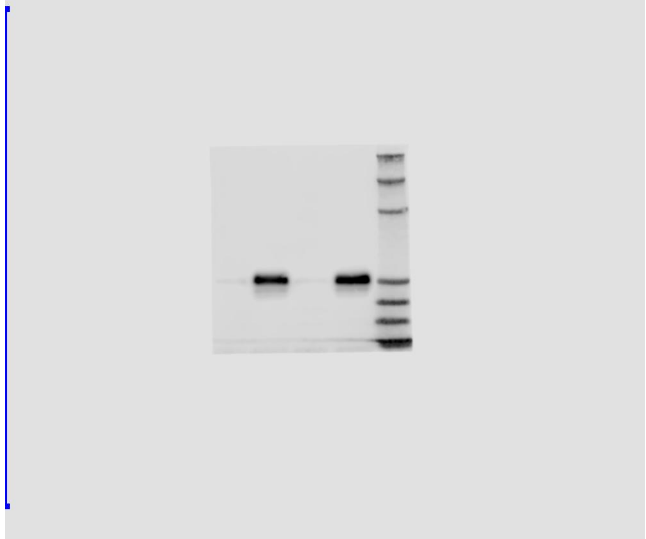

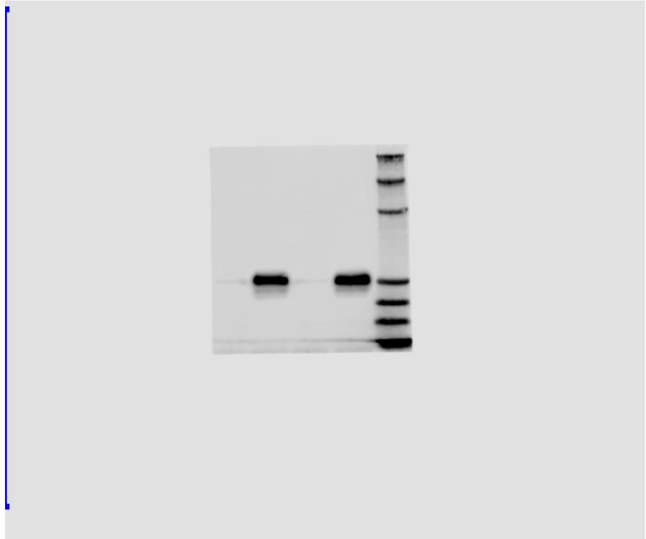

β-actin

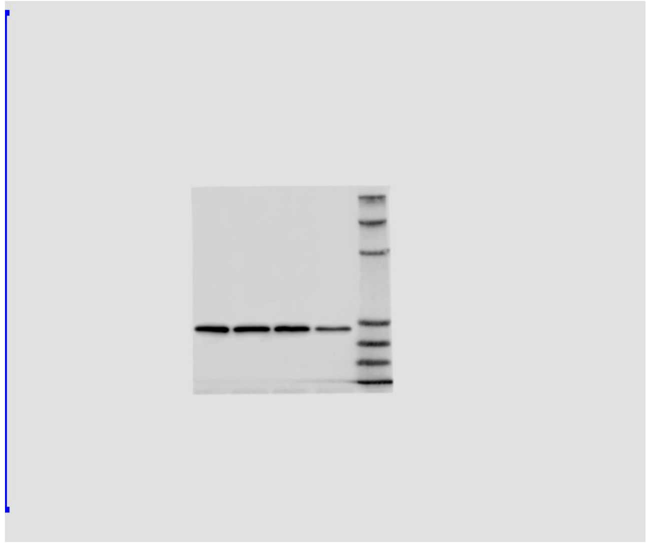

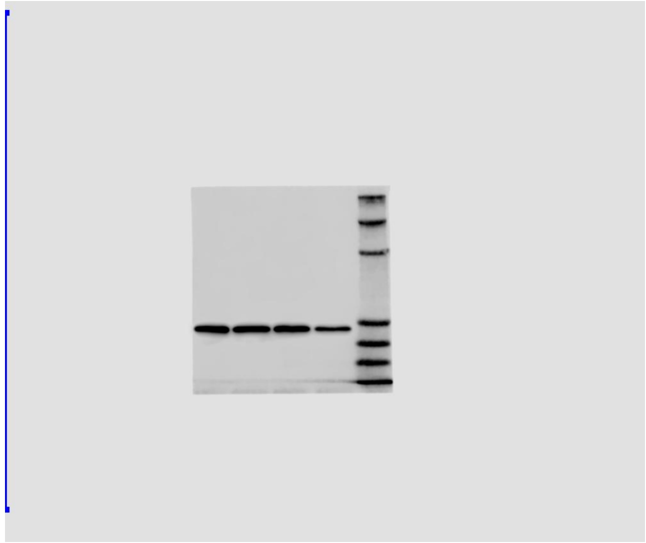

short exposure

long exposure

Marker	NL20 WT	NL20 *ELF3*^{ov}	NL20 *PTEN*$^{-/-}$	NL20 *PTEN*$^{-/-}$*ELF3*^{ov}	H1650 *PTEN*$^{null/null}$	H1650 *PTEN*$^{null/null}$*ELF3*^{ov}	Marker

PTEN

β-actin

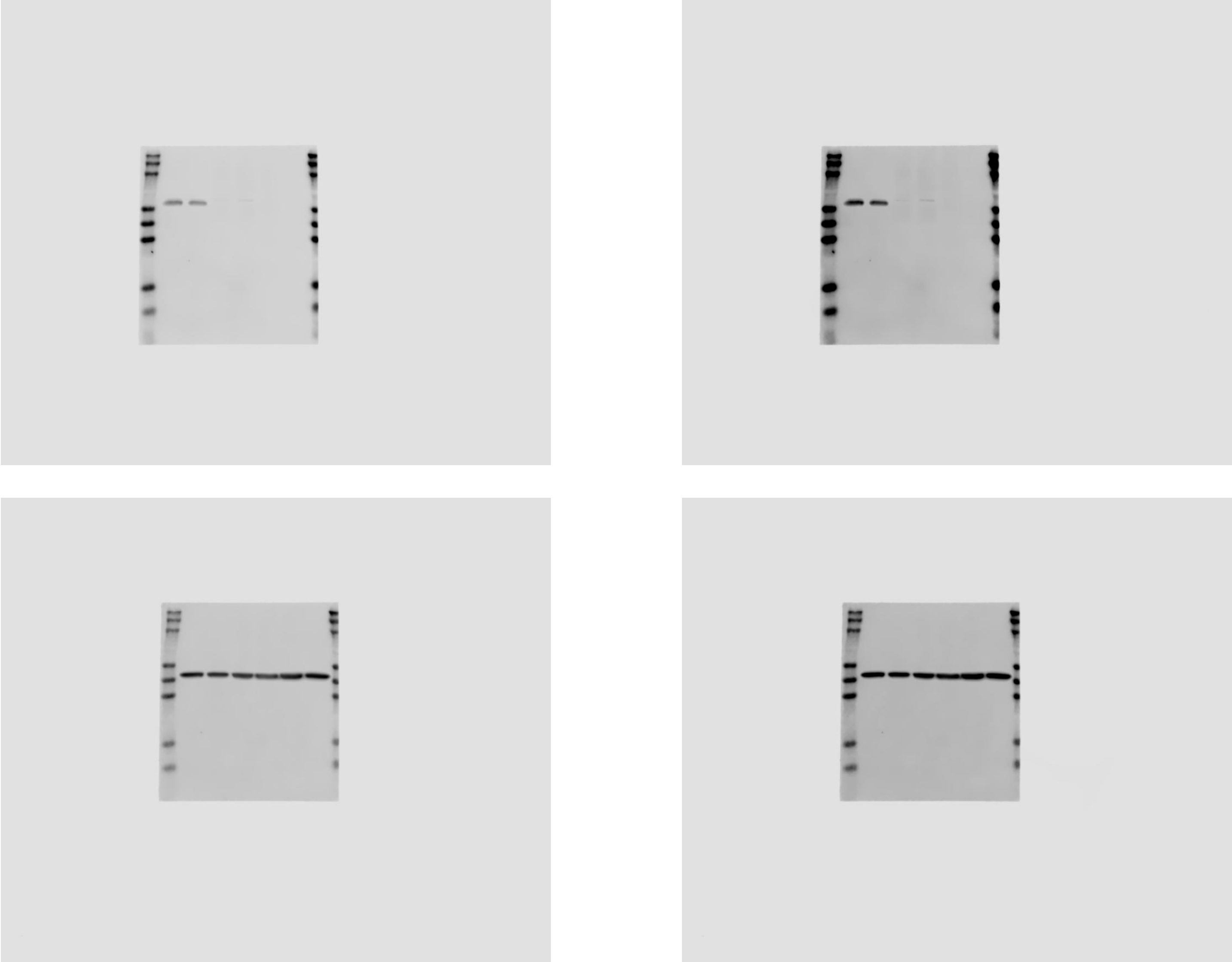

short exposure

long exposure

Marker	Beas2B WT	Beas2B *ELF3*^{ov}	Beas2B *PTEN*$^{-/-}$	Beas2B *PTEN*$^{-/-}$*ELF3*^{ov}			

PTEN

β-actin

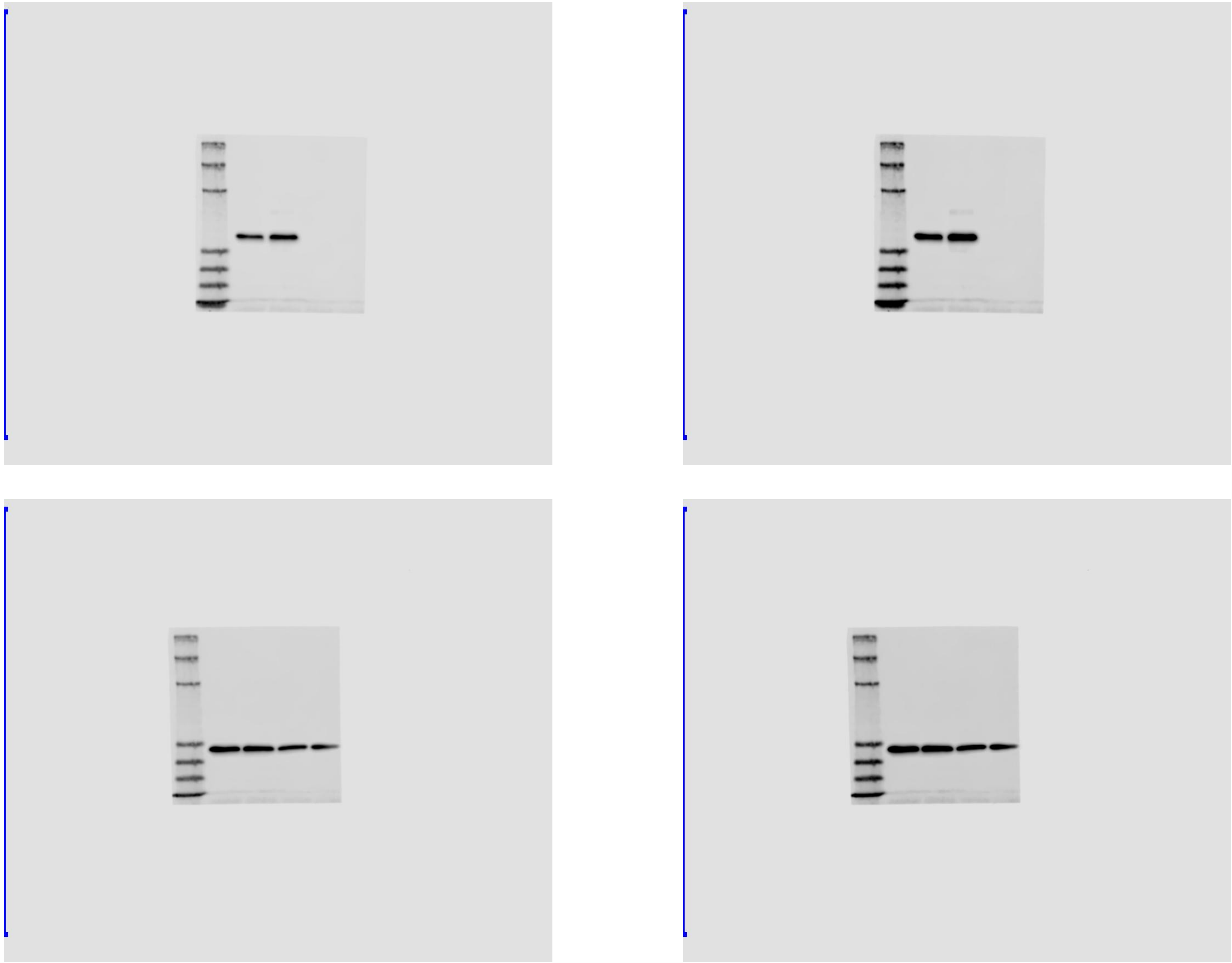

short exposure

long exposure

Marker	NL20 WT	NL20 *ELF3*^{ov}	NL20 *PTEN*$^{-/-}$	NL20 *PTEN*$^{-/-}$*ELF3*^{ov}	H1650 *PTEN*$^{null/null}$	H1650 *PTEN*$^{null/null}$*ELF3*^{ov}	Marker

SLC7A11

β-actin

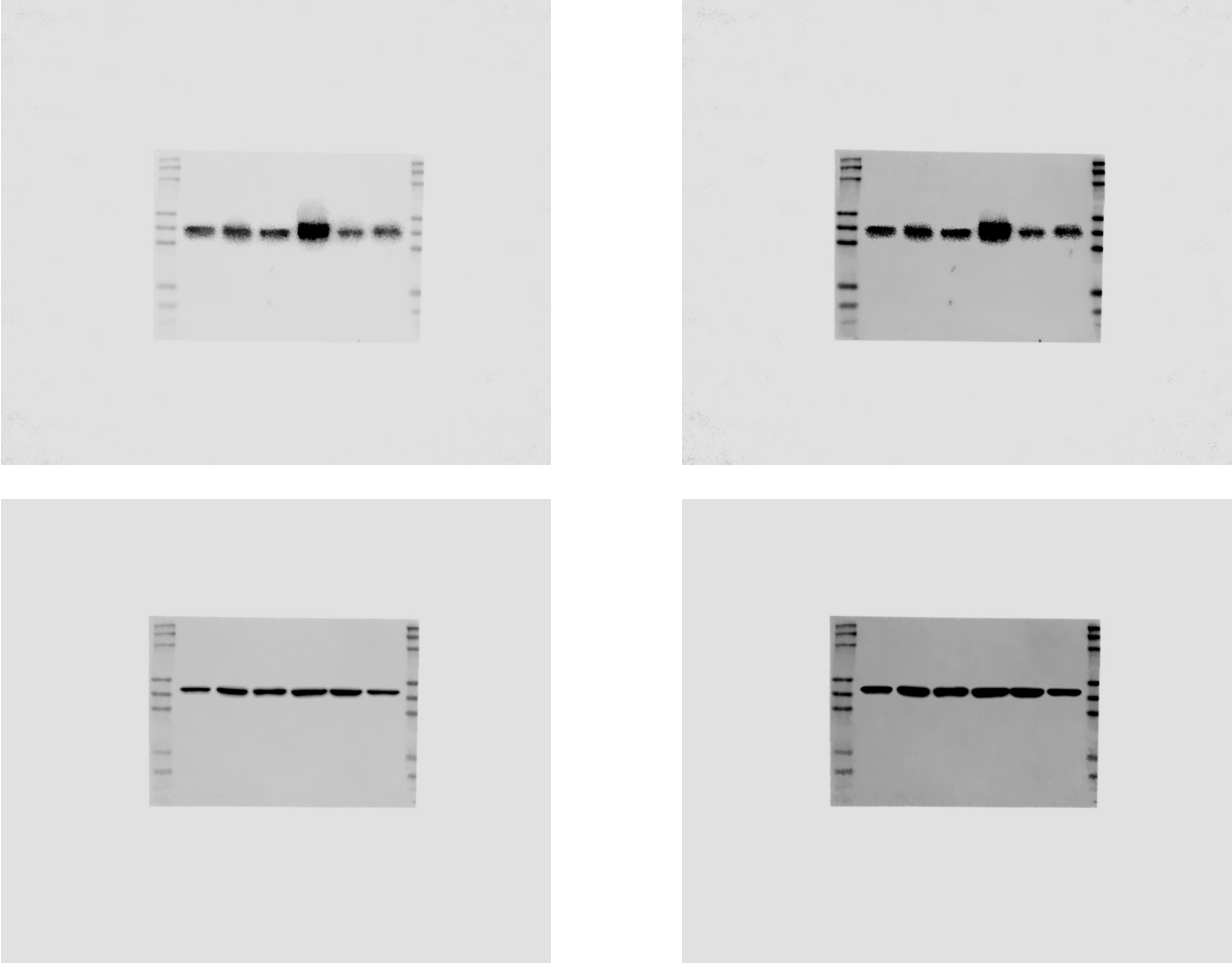

short exposure

long exposure
